# Resolving the graft ischemia-reperfusion injury during liver transplantation at the single cell resolution

**DOI:** 10.1038/s41419-021-03878-3

**Published:** 2021-06-08

**Authors:** Linhe Wang, Jie Li, Shuai He, Yang Liu, Haitian Chen, Shujiao He, Meixian Yin, Dawei Zou, Shirui Chen, Tao Luo, Xinyu Yu, Xuesi Wan, Shunwei Huang, Zhiyong Guo, Xiaoshun He

**Affiliations:** 1 Organ Transplant Center, The First Affiliated Hospital, Sun Yat-Sen University, Guangzhou, 510080 P. R. China; 2 Guangdong Provincial Key Laboratory of Organ Donation and Transplant Immunology, The First Affiliated Hospital, Sun Yat-Sen University, Guangzhou, 510080 P. R. China; 3 Guangdong Provincial International Cooperation Base of Science and Technology (Organ Transplantation), The First Affiliated Hospital, Sun Yat-sen University, Guangzhou, 510080 P. R. China; 4 Sun Yat-sen University Cancer Center, State Key Laboratory of Oncology in South China, Collaborative Innovation Centre for Cancer Medicine, Guangzhou, 510060 P. R. China; 5 Department of Endocrinology and Diabetes Center, The First Affiliated Hospital, Sun Yat-Sen University, Guangzhou, 510080 P. R. China; 6 Surgical Intensive Care Unit, The First Affiliated Hospital, Sun Yat-Sen University, Guangzhou, 510080 P. R. China

**Keywords:** Cell signalling, Mechanisms of disease

## Abstract

Ischemia–reperfusion injury (IRI) remains the major reason for impaired donor graft function and increased mortality post-liver transplantation. The mechanism of IRI involves multiple pathophysiological processes and numerous types of cells. However, a systematic and comprehensive single-cell transcriptional profile of intrahepatic cells during liver transplantation is still unclear. We performed a single-cell transcriptome analysis of 14,313 cells from liver tissues collected from pre-procurement, at the end of preservation and 2 h post-reperfusion. We made detailed annotations of mononuclear phagocyte, endothelial cell, NK/T, B and plasma cell clusters, and we described the dynamic changes of the transcriptome of these clusters during IRI and the interaction between mononuclear phagocyte clusters and other cell clusters. In addition, we found that TNFAIP3 interacting protein 3 (*TNIP3)*, specifically and highly expressed in Kupffer cell clusters post-reperfusion, may have a protective effect on IRI. In summary, our study provides the first dynamic transcriptome map of intrahepatic cell clusters during liver transplantation at single-cell resolution.

## Introduction

Liver transplantation is the standard therapy for end-stage liver disease^1^. At present, almost all transplanted livers suffer ischemia during cold preservation and subsequent reperfusion injury^2^, posing a huge challenge to the functional recovery of donor livers and the prognosis of recipients. The mechanism involved in ischemia–reperfusion injury (IRI) is complex, including changes in a variety of cellular components, inflammatory factors, and mediators^3–5^. For example, hepatocytes and liver sinusoidal endothelial cells (LSEC) are sensitive to ischemia. The lack of oxygen leads to disorders of the respiratory chain, accelerated glycolysis, and electrolyte disturbances, which in turn leads to microcirculation disorders and impaired cell functions^6,7^. After reperfusion, harmful molecules especially lipopolysaccharide (LPS) enters the liver and activates Kupffer cells, produces reactive free radicals (ROS) and pro-inflammatory cytokines, including tumor necrosis factor-α (TNF-α) and interleukin-1β (IL-1β), which triggers the inflammatory cascade and cell apoptosis after IRI^8,9^.

Immune cells and non-immune cells in the liver are heterogeneous and consist of multiple subpopulations of various immunological and physiological functions, including T cells, natural killer (NK) cells, B cells, plasma cells, MP, and endothelial cells^10^. The flow cytometry used in previous studies can only target specific cell subpopulations and marker genes^11–13^. The recent development of unbiased single-cell RNA sequencing (scRNA-seq) technology can well identify different cell populations and explain the heterogeneity and relevance between cells. The scRNA-seq has annotated the cellular transcription profile of adult liver tissues^14^ and revealed the molecular mechanism of myocardial ischemia injury^15,16^. However, the cellular and molecular mechanism of graft IRI during liver transplantation is still unknown at the single-cell resolution level.

Herein, we used scRNA-seq to obtain the first unbiased and comprehensive liver transplant cell atlas by collecting liver tissue samples pre-procurement (PP), at the end of preservation (EP), and 2 h post-reperfusion (PR). This atlas annotated different cell subgroups, revealed their changes in the transcriptome, and illustrated the interactions between different cell subpopulations during liver transplantation. This research will serve as an important resource to further understand the cellular and molecular mechanism of graft IRI during liver transplantation.

## Materials and methods

### Human liver tissue collection and dissociation

All PP, EP, and PR liver tissues were obtained from a 47-year-old male brain death donor. The recipient was a 51-year-old man with chronic hepatitis B virus infection and hepatocellular carcinoma.

Blood or cold preservation solution (University of Wisconsin (UW) solution) in the liver tissues were immediately washed away with 4 °C physiological saline after tissue collection. The tissues were cut into 3 mm pieces, incubated with 1 mM EGTA Sigma-Aldrich, Cat. No. E0396-10G) and rotate at 37 °C, 50 rpm for 10 min. After washing away the EGTA, the tissues were treated in the digestion solution (1 mg/ml Collagenase II + 1 mg/ml Collagenase IV + 50 ug/ml DNase I) at 100 rpm and 37 °C for 30 min. The cell suspension was passed through a 70 mm nylon cell strainer (BD, Cat. No. 352350, USA), and then centrifuged at 50×*g* for 3 min to collect the cell pellet, and then the remaining suspension centrifuged at 300×*g* for 5 min to collect the remaining cell pellet. Each tissue was resuspended to a concentration of 50–500 million cells per milliliter with resuspension buffer. Then used LIVE/DEAD Viability/Cytotoxicity Kit (Invitrogen, Cat. No. L3224, USA) to stained cells, and only AM+ EH− cells were collected by fluorescence-activated cell sorting for each tissue.

### Single-cell RNA-seq data processing

Single-cell cDNA libraries were constructed using the standard procedure of GemCode technology (10X Genomics, USA). For each tissue, the 5′-end cDNA libraries were sequenced using data volumes of 8000 cells. Illumina HiSeq XTen platform was used for sequencing at pared-end 150 bp length. The cDNA library measured 120 G base data volume.

The Illumina software bcl2fastq (version v2.19.0.316) was used to convert the raw data (BCL files) into fastq files. CellRanger (“count” option; version 3.0.1; 10× Genomics) was used to count to align the sequence to the human reference genome (hg38, https://cf.10xgenomics.com/supp/cell-exp/refdata-cellranger-GRCh38-3.0.0.tar.gz)^17^ and calculate the gene expression matrix^18^. The original gene expression matrix in the “filtered_feature_bc_matrix” folder generated by CellRanger software was used for further analysis. The reference gene set used for gene expression estimation in our study was from the Ensembl database^19^.

### Doublets classifying, cell clustering, and DEG analysis

Data filtering, normalizing, dimensionality reduction, and clustering for cells were performed using R software (version 3.6.1; https://www.r-project.org) and Seurat package (version 3.1.2; https://satijalab.org/seurat)^20^. “DoubletFinder” (version 2.0.3; https://github.com/chris-mcginnis-ucsf/DoubletFinder) was used to identify doublets in each sample. A further method of cell filtration is to remove cells with low quality (UMI < 1000, gene number < 500), and high (>0.25) mitochondrial genome transcript ratio. In the analysis of each cell type, a subpopulation of cells expressing multiple markers of different cell types will be defined as a non-single cell population and will be removed in the subsequent analysis. “NormalizedData” function was used to normalize the expression data and then used the “ScaleData” function to perform regression to remove the influence of UMI and mitochondrial content. “FindVariableGenes” was used to identify genes with high variation for principal component analysis (PCA analysis). For each sample and main cell type, we used a different number of PC and resolution for dimensionality reduction analysis. Use the same number of PCs to identify cell types as in UMAP dimensionality reduction (“FindClusters” function). “FindAllMarkers” function was used to analyze the differential expression markers, and use Wilcoxon to test the significance level. Genes with an absolute value of fold-change (FC) natural number logarithm (|lnFC|) greater than 0.25 and a *p* adjust value lower than 0.05 after adjustment by Bonferroni are considered to have significant differences. The marker genes used to define different cell types were selected according to the literature and verified by the online tool CellMaker^21^. Differential genes in bulk RNA-seq data were identified by DeseqR package (v.1.8.3), and differentially expressed genes were identified by FC values greater than 2 and *p* values less than 0.05.

### Analysis of pathway and cellular interaction analysis

Gene Ontology (GO) enrichment analysis was performed using the online tools Metascape (http://metascape.org/gp/index.html)^22^. Genes with LnFC greater than 0.405 and *p*-adjust value less than 0.05 were selected for GO enrichment analysis. GSVA was performed to identify enriched cellular pathways in our dataset, using R package GSVA (version 1.32.0) (https://www.gsea-msigdb.org/gsea/index.jsp) on the C2: Canonical pathways—BIOCARTA subset (c2.cp.biocarta.v7.1.symbols.gmt), PID subset (c2.cp.pid.v7.1.symbols.gmt) and 50 hallmark pathways with default parameters. “AddModuleScore” function in the R Seurat package was used to calculate the antigen presentation scores related to MHC I and MHC II molecules according to the REACTOME database geneset. We used the default parameters of the CellphoneDB software (version 2.0)^23^ to investigate the interaction between cells. Taking into account the biological effects and computational burden, we only showed the interactions between clusters of MP and other cell types, such as endothelial cells, NK and T cells, B cells, and plasma cells after reperfusion (PR sample). The filter condition for effective ligand–receptor pairs was that the *p* value was less than 0.05, and the average expression of the interaction pair was greater than 0.

### Immunofluorescence, Western blot, and RT-PCR

The paraffin-embedded tissue sections were deparaffinized, rehydrated and treated with 3% H_2_O_2_ to block endogenous peroxidase activity and underwent high-temperature antigen retrieval. The repaired tissue was incubated with 3% bovine serum albumin for 30 min at room temperature, and incubated overnight with CD68 antibody (Servicebio, GB11067, China) in a refrigerator at 4 °C. Then the slides were incubated with the secondary antibody (HRP polymer, anti-rabbit IgG) for 50 min at room temperature. Subsequently, the tissue was treated with a solution containing TSA reagent for 10 min. After each treatment with TSA, the slides were subjected to microwave heat treatment, incubated overnight with TNIP3 antibody (Abcam, ab198697, UK) in a refrigerator at 4 °C, and then incubated with the secondary antibody and treated with TSA. For each sample, after all antigen labeling was completed, the nucleus was stained with 4′−6′-di-yl-2-phenylindole (DAPI).

Total protein was extracted using RIPR lysis buffer (KeyGEN, China) and quantified using the BCA protein assay (Thermo Scientific). The proteins of tissue samples were resolved by 10% SDS-PAGE and transferred to the PVDF membrane (0.2 µm pore size, Millipore, Billerica, MA, USA). The membranes were immunoblotted with primary antibodies overnight at 4 °C followed by secondary antibody incubation for 2 h, and subsequent visualization with Chemiluminescent HRP substrate (Millipore). The antibodies were used as follows: TNIP3 (1:2000, Abcam, ab198697); β-tubulin (1:2000, cat. No. 100941-1-AP; Proteintech, USA); Goat anti-Rabbit IgG antibody (1:5000, cat. No. ab205718; Abcam).

Samples were obtained from 16 pairs of CLT cases for quantitative real-time polymerase chain reaction (RT-PCR). Standard procedures were followed to extract RNA, synthesize cDNA and quantitative RT-PCR. TNIP3 primers were: Forward, CGTCTCCTCATCCAAAACGG; Reverse, CCTGGAGTCTTCAGAGAACATAGA. FC = 2^(−ΔΔCT)^, and Student’s *t* test was used to evaluate significant differences (mean + s.d.).

## Results

### ScRNA-seq identifies intrahepatic cell subpopulations

**Fig. 1:**
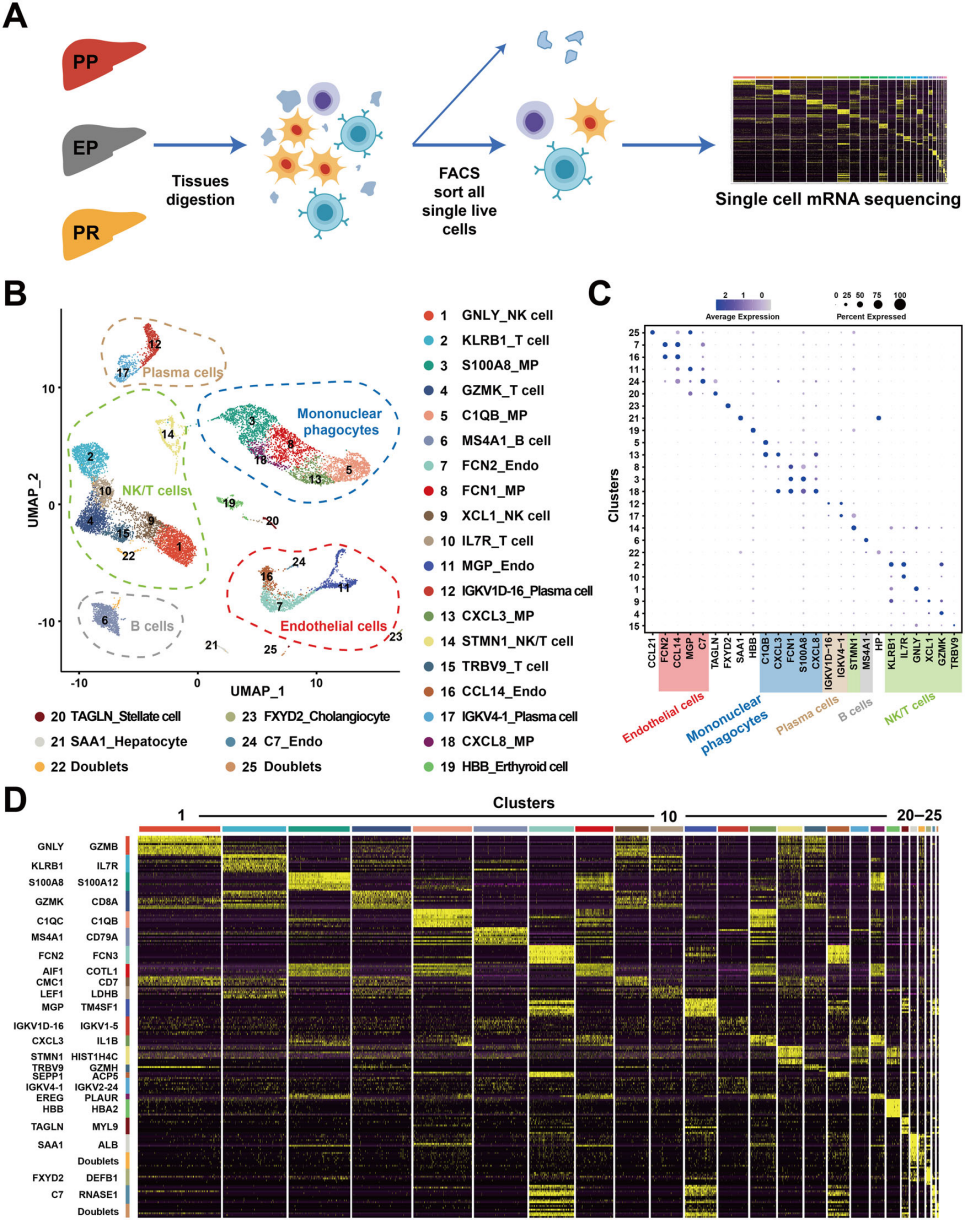
Overview of scRNA-seq from samples in liver transplantation. **A** Workflow of tissue collection, sample processing, and data acquisition. Collect samples from three-time points, PP, EP, and PR, for tissue digestion. Next, sort live cells by FACS and construct cDNA libraries, then perform high-throughput sequencing and downstream analyses. **B** UMAP visualization of all cells (14,313) in 25 clusters. Each dot represents one cell, with colors coded according to the different clusters. Clusters are named by the most specific and highly expressed genes. MP mononuclear phagocytes, Endo endothelial cells. **C** Dot plots showing the most highly expressed marker genes (*x*-axis) of major cell types (*y*-axis) in Fig. 1B. The color of the dots represents the level of gene expression while the size of the dot represents the percentage of cells expressing the gene. **D** Heatmap of top ten differentially expressed genes between different clusters. The line is colored according to clusters in Fig. 1B. Each cluster lists the top two genes shown on the left.

We performed scRNA-seq of intrahepatic cells in PP, EP, and PR samples from an adult donor liver. We obtained an average of more than 400 million sequencing reads per sample, with a median sequencing saturation of 86% (84.2–91%). A total of 18,793 single-cell transcriptomes were obtained using the 10× Genomics platform (Fig. 1A, Supplementary Table 1). We identified 1160 doublets using “DoubletFinder” software (Supplementary Fig. S1A). Cells with low quality (UMI < 1000, gene number < 500) and high (>0.25) mitochondrial genome transcript ratio were removed. Finally, a total of 14,313 single-cell transcriptomes were obtained for further analysis after quality control (QC) filters. The median UMI and genes were 3262–3796 and 1262–1474. The information on sequencing and data processing is shown in Supplementary Table 1. Changes in cell-type-specific gene expression caused by the IRI response can make joint analysis difficult. Therefore, we performed an integrated analysis for cell-type identification and comparison using Seurat R package v3.

Clustering the intrahepatic cells revealed 25 populations (Fig. 1B). We named these clusters with their most specifically and highly expressed marker genes (Fig. 1C). And the differential gene expression (DEG) analysis showed that each cluster had a specific gene signature (Fig. 1D). These clusters across five major cell lineages, including mononuclear phagocytes (MP), endothelial cells, NK/T cells, B cells, and plasma cells, were identified by their canonical signature gene profiles such as *CD68*^24^ (Kupffer cell marker), *CD3D*^25^ (T cell marker), *FCGR3A*^26^ (NK cell marker), *MS4A1*^27^ (B cell marker), *SDC1*^27^ (plasma cell marker), and *PECAM1*^28^ (endothelial marker), respectively (Fig. 1B, C, Supplementary Fig. S1B, Supplementary Table 2). These five populations contained cells from samples collected at all of the three time points (PP, EP, and PR) (Supplementary Fig. S1C).

### Different pro- and anti-inflammatory phenotypes in MP

**Fig. 2:**
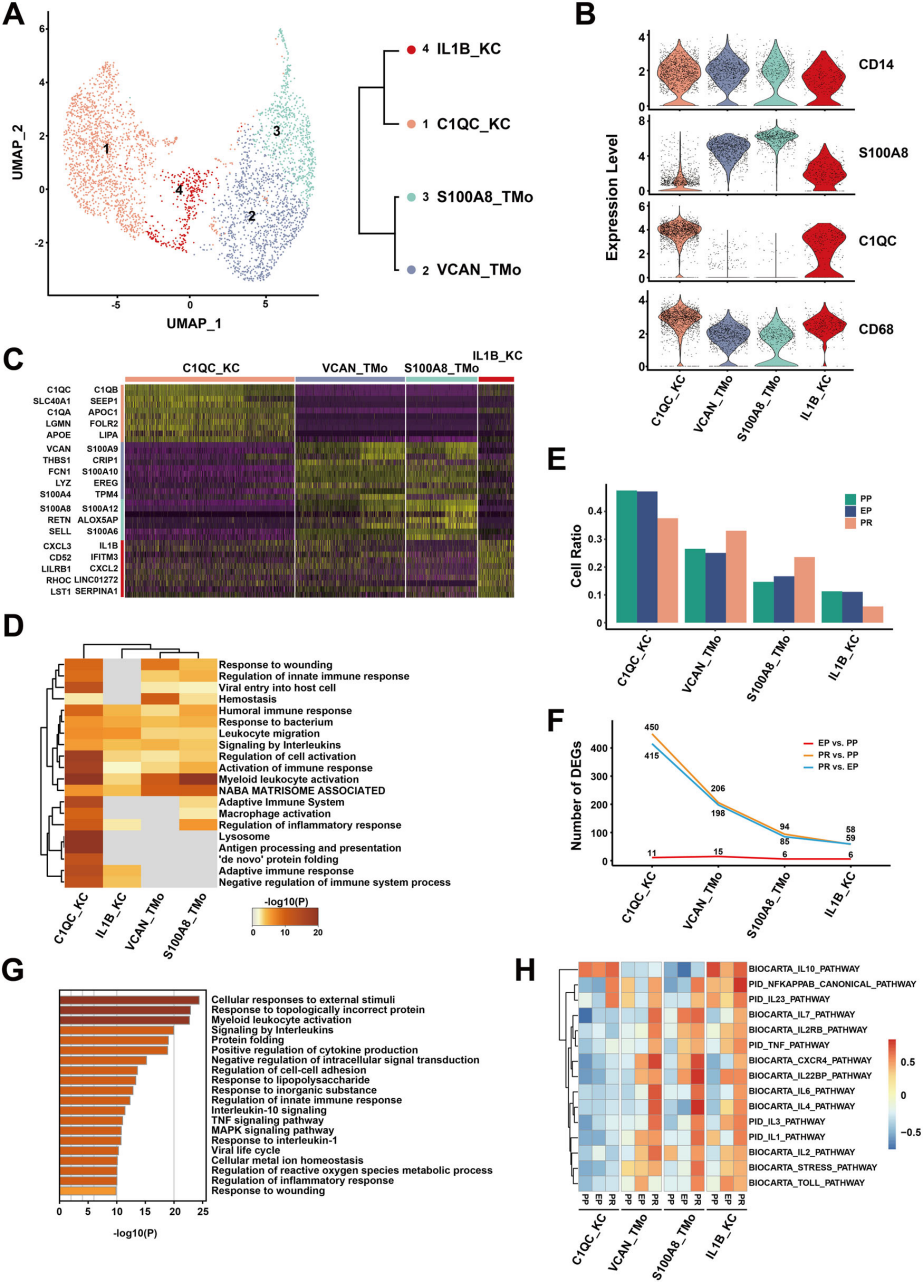
scRNA-seq of mononuclear phagocytes in liver transplantation. **A** UMAP plot showing four mononuclear phagocyte clusters (3622 cells) in liver transplantation, colored according to different clusters (left panel). Dendrogram of four clusters by the hierarchical clustering analysis based on their normalized mean expression values (right panel). **B** Violin plots showing the normalized expression of *CD14*, *S100A8*, *C1QC*, and *CD68* genes (*y*-axis) for TMo and KC clusters (*x*-axis). **C** Heatmap of top ten differentially expressed genes between different mononuclear phagocyte clusters. The line is colored according to clusters in Fig. 2A. **D** Gene Ontology enrichment analysis results of mononuclear phagocyte clusters. Only the top 20 most significant GO terms (*p* value < 0.05) are shown in rows. **E** Cell ratio of different mononuclear phagocyte clusters in PP, EP, and PR samples. **F** Number of upregulated DEGs between different timepoint samples in different mononuclear phagocyte clusters. **G** Gene Ontology enrichment analysis results of upregulated DEGs in C1QC_KC cluster after reperfusion (PR vs. EP). Only the top 20 significant GO terms (*p* value < 0.05) are shown in rows. **H** The gene set variation analysis (GSVA) showing the pathways (PID and BIOCARTA gene sets) with significantly different activation in different samples of mononuclear phagocyte clusters. Different colors represent different activation scores.

We obtained 3622 MP from PP, EP, and PR samples, which were regrouped into four distinct clusters (Fig. 2A left panel, Supplementary Fig. S2A), annotated as two tissue monocytes (with a suffix of TMo, highly expressed *S100A9* gene) and two Kupffer cell clusters (with a suffix of KC, highly expressed *C1QC* gene) (Fig. 2B)^29^, based on the most specific, highly expressed gene in that cluster. The hierarchical clustering analysis revealed that the two KC clusters were closely related as a branch node, as were the two TMo clusters (Fig. 2A right panel).

VCAN_TMo and S100A8_TMo clusters highly expressed *S100A12*, *S100A8*, and other genes similar to those expressed by peripheral monocytes, indicating that cells in these two clusters might just be recruited and differentiated from peripheral blood circulating monocytes. In the VCAN_TMo cluster, the five most highly expressed genes were *VCAN*, *S100A9*, *THBS1*, *CRIP1*, and *FCN1* while in the S100A8_TMo cluster were *S100A8*, *S100A12*, *RETN*, *ALOX5AP*, and *S100A9*. C1QC_KC and IL1B_KC clusters highly expressed *C1QC*, *C1QB,* and other tissue-resident marker genes in macrophages, indicating that they are Kupffer cells, and the top five highly expressed genes were *C1QC*, *C1QB*, *SLC40A1*, and *SEPP1*, and *C1QA* in C1QC_KC and *IL1B*, *CXCL3*, *IFITM3*, *CD52*, and *CXCL2* in IL1B_KC (Fig. 2C).

We investigated the potential biological functions of different TMo and KC clusters using GO analysis with upregulated DEGs, and the analysis results revealed the differences and commonalities between the various clusters (Supplementary Table 3). The biological function commonly enriched by TMo and KC clusters were *Myeloid leukocyte activation*, *Leukocyte migration*, *Signaling by interleukins*, and other pathways of immune response (Fig. 2D). In addition, the C1QC_KC cluster showed strong immune regulatory ability and high enrichment in the *Regulation of protein stability, Regulation of tumor necrosis factor production, Antigen processing and presentation*, and other pathways. Most of the pathways enriched in the IL1B_KC cluster were the same as those in the C1QC_KC cluster, except for pathways such as *Negative regulation of the viral process*. S100A8_TMo cluster was enriched in *Formyl peptide receptors bind formyl peptides* and *Defense response to fungus* pathways except for the common enrichment immune response-related pathways, while VCAN_TMo cluster was additionally enriched in *Dissolution of Fibrin Clot*, *Positive regulation of organelle organization* and other pathways (Supplementary Fig. S2B).

In order to explore the dynamic changes of different TMo and KC clusters during liver transplantation, we performed a comparative analysis between EP and PP samples in the cold preservation stage (EP vs. PP), PR and EP samples in the reperfusion stage (PR vs. EP), as well as PR and PP samples in the overall stage (PR vs. PP). In the PP and EP samples, we found that the proportion of each cluster in the corresponding sample did not change significantly. In the PR sample, the proportion of KC clusters was lower than that of PP and EP samples, while the proportion of TMo clusters was higher than those of PP and EP samples (Fig. 2E).

Next, we performed DEG analysis and found that only a small number of differentially expressed genes were upregulated in each cluster during the cold preservation stage. In contrast, more genes were upregulated in each cluster during the reperfusion stage and overall stage when compared with the cold preservation stage, respectively. The number of upregulated genes between the reperfusion stage and overall stage was very close, but the amount of DEG upregulation in the overall stage was slightly more than that in the reperfusion stage. These two stages shared most of the genes that were upregulated, especially those with high expression levels; a similar trend was observed in the downregulated genes (Fig. 2F, Supplementary Fig. S2C, D, Supplementary Tables 4–6).

The results of GO analysis showed that the C1QC_KC cluster upregulated multiple stress, inflammatory response pathways such as *Cellular responses to external stimuli*, *Response to topologically incorrect protein*, and cell activation pathways such as *Myeloid leukocyte activation* in the reperfusion stage (Fig. 2G). Like the C1QC_KC cluster, the IL1B_KC, VCAN_TMo, and S100A8_TMo clusters also up-regulated stress response pathways and cell activation pathways in the reperfusion stage (Supplementary Fig. S3A left panel). The most obvious down-regulated pathways for C1QC_KC, IL1B_KC, VCAN_TMo, and S100A8_TMo clusters in the reperfusion stage are *G protein-coupled purinergic receptor signaling pathway*, *Influenza A*, *Response to an antibiotic*, and *Response to inorganic substance* respectively (Supplementary Fig. S3A right panel). This phenomenon was similarly found in the overall stage with the reperfusion stage, there was no obvious enrichment pathway in the cold preservation stage because of the small number of DEGs (Supplementary Fig. S3B).

In order to further explore the changes of specific inflammation-related pathways in different stages, we performed gene set variation analysis (GSVA) and selected the pathways related to IRI. The results showed that various pro-inflammatory pathways such as the *IL1 pathway, IL2 pathway, TNF pathway*, and *NFKAPPAB pathway* were activated after reperfusion in VCAN_TMo, S100A8_TMo, and IL1B_KC clusters, while these pathways maintained a low activation level in the C1QC_KC cluster during the cold preservation and reperfusion stage. In contrast, the *IL10 pathway*, which is considered as protective during IRI^30^, were significantly activated in the C1QC_KC and IL1B_KC cluster of samples collected at various time points. This result suggests that the C1QC_KC cluster may have an anti-inflammatory regulatory effect in IRI, while VCAN_TMo and S100A8_TMo clusters may play a pro-inflammatory role in IRI. IL1B_KC cluster tends to be an intermediate cell population, which has both pro-inflammatory and anti-inflammatory effects (Fig. 2H). In addition, we assessed the antigen-presenting abilities for extracellular antigens as reflected by antigen-presenting score (APS), and found that the KC clusters, particularly the C1QC_KC cluster, had higher APS than the TMo clusters in MHC class II genes (*p* < 0.0001) (Supplementary Fig. S2E).

### Specific upregulation of TNIP3 in KC clusters after reperfusion

To further explore the anti-inflammatory mechanism of KC clusters, we studied the DEGs that may be related to the anti-inflammatory effect during the cold preservation stage and the reperfusion stage. In the reperfusion stage, in addition to the heat shock protein family proteins and metal ion binding proteins, the role of which is already well known in IRI, we found that nuclear factor kappa-B (NF-κB) activation inhibitor *TNIP3* was specifically and highly expressed in the KC clusters after reperfusion (FC = 2.2 in PR vs. EP of C1QC_KC cluster; FC = 3 in PR vs. EP of IL1B_KC cluster).

**Fig. 3:**
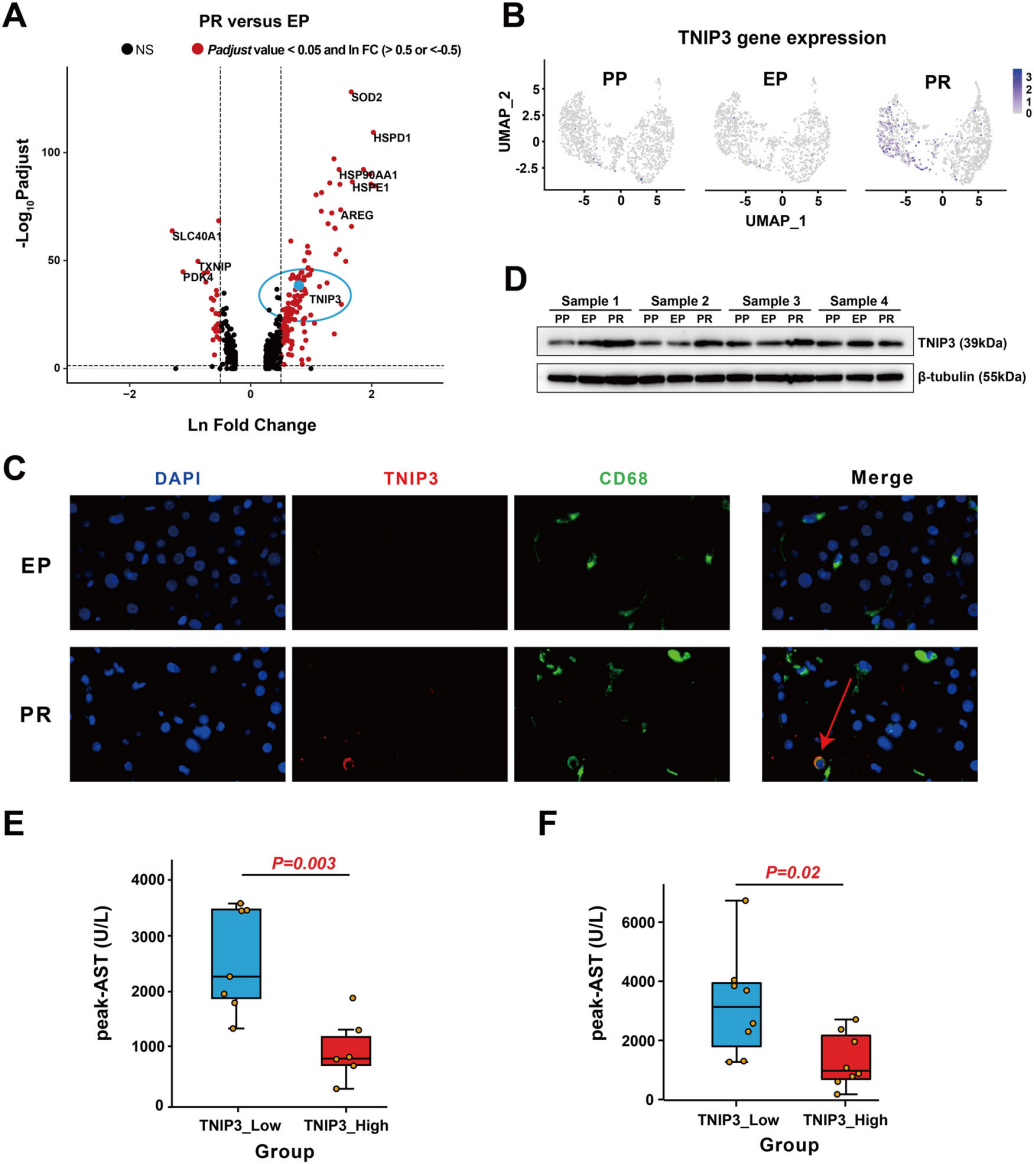
TNIP3 expression is specifically increased in KC clusters after IRI. **A** Volcano plot shows the DEGs of PR vs. EP in the C1QC_KC cluster. The red dots represent *p* adjust value less than 0.05 and lnFC greater than 0.5 or less than −0.5, the blue dot represents *TNIP3*, and the rest are black dots. **B** Feature plots showing the normalized expression of *TNIP3* in mononuclear phagocyte clusters of PP, EP, and PR. Color represents the level of gene expression. **C** Immunofluorescence showed that the expression of TNIP3 (red), CD68 (green), and DAPI (purple) in EP and PR samples. CD68+ TNIP3+ cells only appeared in partly cells in the PR sample. **D** Western Blot results showed that the expression of TNIP3 in PR samples was higher than that in the EP and PP samples. **E** The postoperative peak-AST level in the TNIP3_low group was higher than the TNIP3_high group (*n* = 13, 880 vs. 2506 U/L, *p* = 0.003) according to bulk RNA-seq data analysis results, each point represents a patient. **F** The postoperative peak-AST level in the TNIP3_low group was higher than the TNIP3_high group (*n* = 16, 1316 vs. 3216 U/L, *p* = 0.02) according to the RT-PCR results of 16 pairs of liver samples.

*TNIP3* was almost not expressed in PP and EP samples, while the expression level in the PR sample was significantly increased. Interestingly, it was almost only expressed in the KC clusters especially in the C1QC_KC cluster when compared with other intrahepatic cells in our dataset (Fig. 3A, B). In addition, the high expression of *TNIP3* after reperfusion (log_2_ FC = 3.91) was verified in our bulk RNA sequencing data from 14 EP–PR pairs of donor liver samples (Supplementary Table 7).

Immunofluorescence results showed that in the other four paired samples, CD68+ cells did not express TNIP3 in the EP samples, while a proportion of CD68+ cells clearly expressed TNIP3 protein in the PR samples (Fig. 3C). The Western Blot results also showed that the expression of TNIP3 protein in the PR group was higher than that in the PP and EP group (Fig. 3D).

To further explore the role of *TNIP3* in IRI, we analyzed the results of bulk RNA sequencing of 14 pairs of liver tissue samples before and after reperfusion and divided them into *TNIP3* high expression group and low expression group according to the median value of FC (3.438) before and after reperfusion. We found that the peak aspartate aminotransferase (AST) levels of patients within 7 days post-transplantation in the high expression group were significantly lower than those in the low expression group (880 vs. 2506 U/L, *p* = 0.003) (Fig. 3E, Supplementary Table 8). The RT-PCR results of 16 pairs of patient liver tissues also showed that AST levels of patients within 7 days post-transplantation in the high expression group were significantly lower than those in the low expression group (1316 vs. 3216 U/L, *p* = 0.02, median value of FC = 1.26) (Fig. 3F, Supplementary Table 9), suggesting a protective role of *TNIP3* in graft IRI.

### Endothelial cells in liver transplant IRI

**Fig. 4:**
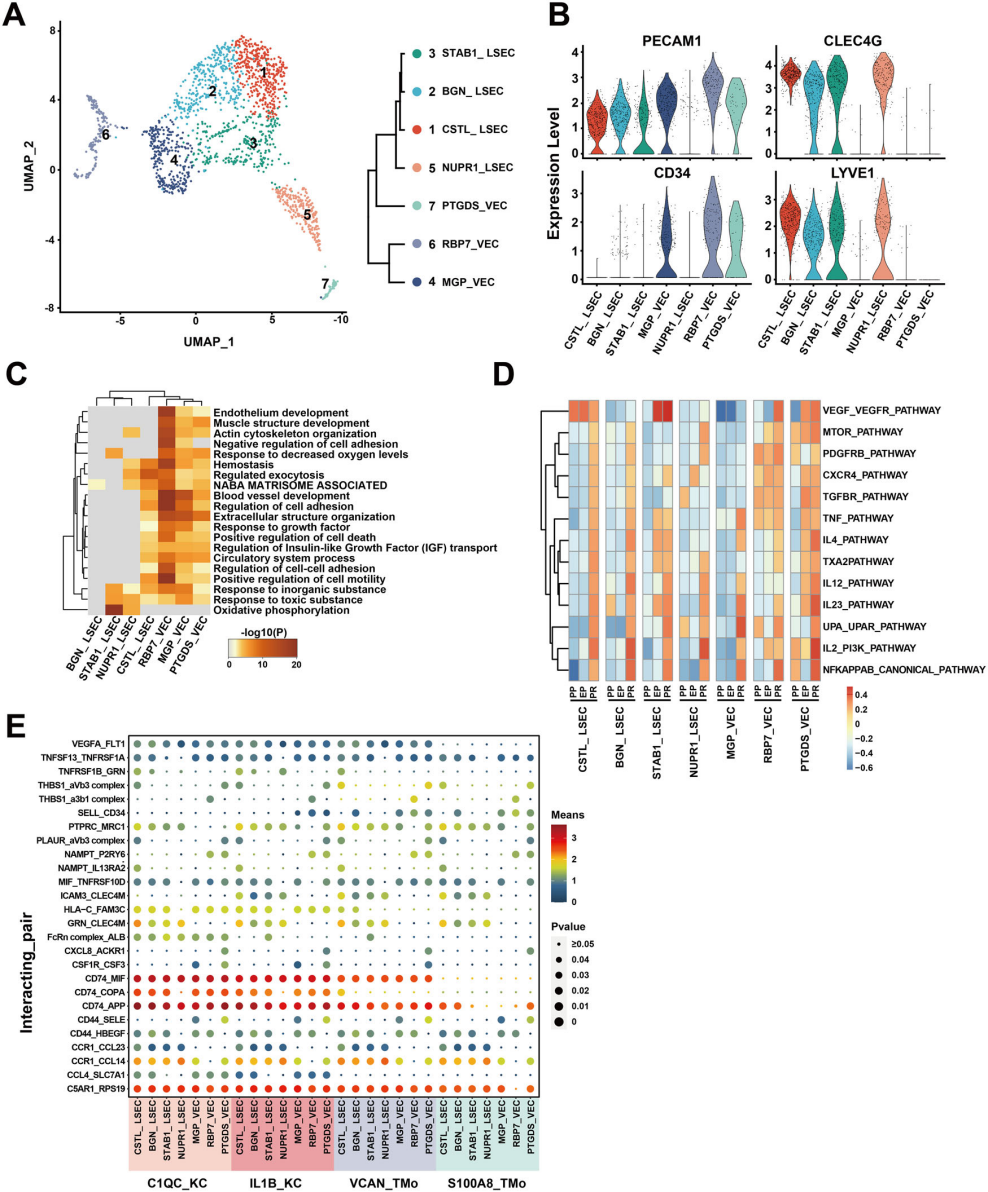
scRNA-seq of endothelial cells in liver transplantation. **A** UMAP plot showing seven endothelial cell clusters in liver transplantation, colored according to different clusters (left panel). Dendrogram of seven clusters by hierarchical clustering analysis based on their normalized mean expression values (right panel). **B** Violin plots showing the normalized expression of *PECAM1*, *CLEC4G*, *CD34*, and *LYVE1* genes (*y*-axis) for endothelial cell clusters (*x*-axis). **C** Gene Ontology enrichment analysis results of endothelial cell clusters. Only the top 20 most significant GO terms (*p* value < 0.05) are shown in rows. **D** GSVA showing the pathways (PID gene sets) with significantly different activation in different samples of endothelial cell clusters. Different colors represent different activation scores. **E** Cell–cell interaction analysis between mononuclear phagocyte clusters and different endothelial cell clusters in PR samples. Ligand–receptor pairs are labeled in *y*-axis. The size of the circle represents the level of *p* value while different colors represent different means value. Ligands come from mononuclear phagocyte clusters, and receptors come from endothelial cell clusters.

We obtained single-cell transcriptomes of 1766 cells from the endothelial cell lineage of three samples and reclustered them into seven clusters (Fig. 4A, Supplementary Fig. S4A). These clusters uncovered two major populations of endothelial cells in the liver with different canonical marker genes, the LSEC and vascular endothelial cells (VEC). LSEC is a group of endothelial cells with different functions and morphologies that exist in the liver sinusoids with filtering and scavenging roles^31,32^. The VEC mainly lined in the hepatic artery and vein^33^. We annotated the four *CLEC4G*^*+*^*PECAM1*^*low*^ clusters as LSEC based on previous studies^14,34–36^. Their highly expressed top five genes were *CTSL, CLEC4M, CCL23, CD14*, and *FCGR2B* in CTSL_LSEC; *BGN*, *CPM*, *IGFBP3*, *CD24*, and *PLPP3* in BGN_LSEC; *STAB1*, *MT1G*, *MEG3*, *SERPINA1*, and *NEAT1* in STAB1_LSEC; *NUPR1*, *GPX4*, *CSTB*, *PVALB*, and *POLR2L* in NUPR1_LSEC.

The other three clusters were *CD34*^*+*^*PECAM1*^*high*^ and annotated as VEC based on published literatures^14,34–36^. Their top five highly expressed genes were *MGP*, *ADIRF*, *CLU*, *MT1M*, and *CPE* in MGP_VEC; *RBP7*, *GSN*, *RGCC*, *CXCL12*, and *PLVAP* in RBP7_VEC; *PTGDS*, *ADGRG6*, *POSTN*, *TAGLN*, and *IL1RL1* in PTGDS_VEC (Fig. 4B, Supplementary Fig. S4B, Supplementary Table 10).

In order to compare the differences in the biological functions of different endothelial cell clusters, we performed GO analysis in the seven endothelial cell clusters. The VEC clusters were enriched in *Extracellular structure organization, Muscle structure development*, *Endothelium development,* and other pathways, while the LSEC clusters were quite different. STAB1_LSEC and NUPR1_LSEC clusters were specifically enriched in the *Oxidative phosphorylation* pathway. Many pathways enriched in CSTL_LSEC were similar to those of VEC clusters. There were few DEG enrichment pathways in the BGN_LSEC cluster (Fig. 4C).

In the study of the dynamic changes of various endothelial cell clusters in liver transplantation, we found that there were few genes differentially expressed in each cluster during the cold preservation stage, while the number of DEGs significantly increased in the reperfusion stage and the overall stage, and most of the high expression level genes are shared between them (Supplementary Fig. S4C, D, Supplementary Tables 11–13). The GO analysis of DEGs showed that almost all endothelial cell clusters had upregulation of *Protein folding* or *Response to topologically incorrect protein* related to stress response and cell adhesion-related inflammation-regulation pathways in the reperfusion stage. In addition, the CSTL_LSEC and BGN_LSEC clusters also upregulated apoptosis-related pathways in the reperfusion stage. The main downregulated pathways in endothelial cell clusters included *Blood vessel development* and other pathways in the reperfusion stage (Supplementary Fig. S5). This phenomenon was similarly found in the overall stage with the reperfusion stage (Supplementary Fig. S6). GSVA results indicated the dynamic changes of specific inflammatory pathways at different stages. In general, all endothelial clusters showed varying degrees of inflammatory activation after reperfusion. Pathways such as *NFKAPPAB_CANONICAL_PATHWAY*, *IL2_PI3K_PATHWAY*, *IL12_PATHWAY*, and *IL23_PATHWAY* were significantly activated after reperfusion in almost all LSEC and VEC clusters (Fig. 4D).

In order to investigate the potential intercellular communication network between mononuclear phagocyte and endothelial cell clusters after reperfusion, we analyzed the ligand-receptor pairs between these clusters (Fig. 4E). Mononuclear phagocyte clusters commonly had *CD74_MIF*, *CD74_APP*, *C5AR1_RPS19*, and *CCR1_CCL14* interactions with different endothelial cell clusters. The interactions between MP and LSEC clusters were highly enriched in *GRN_CLEC4M*, *ICAM3_CLEC4M*, *CCR1_CCL23*, and other inflammatory cytokines^37^ and cell adhesion-related functions^38^. Meanwhile, the interactions between MP and VEC clusters were enriched in nicotinamide phosphoribosyltransferase related, *CD44_SELE*, and other immune regulation related functions^39,40^. Compared to TMo clusters, the interactions between KC and endothelial cell clusters were specifically enriched in *HLA*−*C_FAM3C*, *CCL4_SLC7A1*, and *CD74_COPA*, most of which play an important role in IRI^41–43^.

### NK/T cells in graft IRI of liver transplantation

**Fig. 5:**
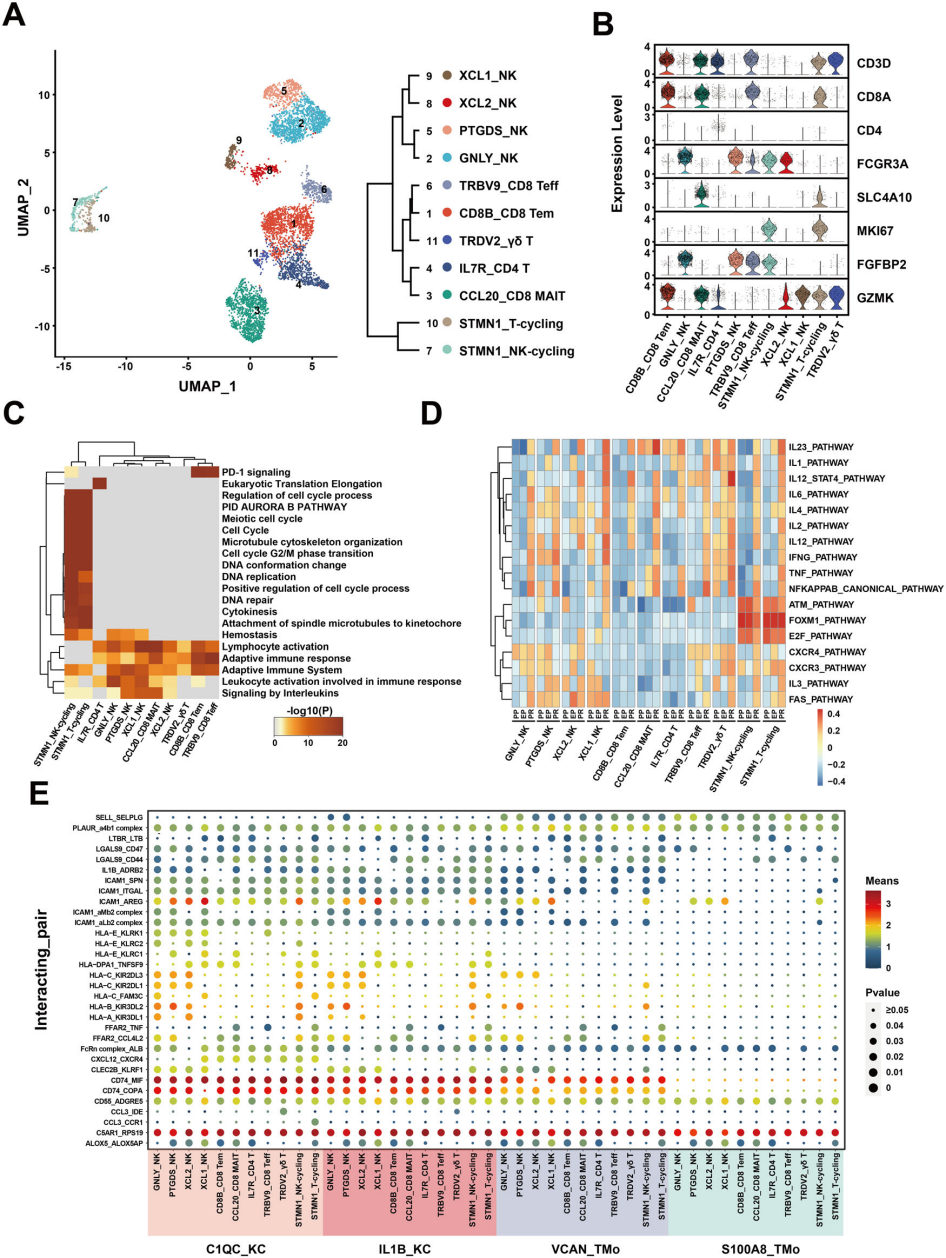
scRNA-seq of NK/T cells in liver transplantation. **A** UMAP plot showing 11 NK/T cell clusters in liver transplantation, colored according to different clusters (left panel). Dendrogram of 11 clusters by the hierarchical clustering analysis based on their normalized mean expression values (right panel). **B** Violin plots showing the normalized expression of marker genes (*y*-axis) for NK/T cell clusters (*x*-axis). **C** Gene Ontology enrichment analysis results of NK/T cell clusters. Only the top 20 most significant GO terms (*p* value < 0.05) are shown in rows. **D** GSVA showing the pathways (PID gene sets) with significantly different activation in different samples of NK/T cell clusters. Different colors represent different activation scores. **E** Cell–cell interaction analysis between mononuclear phagocyte clusters and different NK/T cell clusters in PR samples. Ligand–receptor pairs labeled in *y*-axis. The size of the circle represents the level of *p* value, and different colors represent different means values. Ligands come from mononuclear phagocyte clusters, and receptors come from NK/T cell clusters.

A total of 5844 single-cell transcriptomes were obtained from NK and T cell lineages of three samples and regrouped into 11 clusters (Fig. 5A, Supplementary Fig. S7A). Among them, there were four clusters of NK cells, which expressed high levels of *FCGR3A* and low levels of *CD3D*. The top five highly expressed genes were *GNLY*, *FGFBP2*, *FCGR3A*, *GZMB*, and *TYROBP* in GNLY_NK cluster; *PTGDS*, *FCER1G*, *MYOM2*, *AREG*, and *SPON2* in PTGDS_NK cluster; *XCL2*, *CMC1*, *KLRC3*, *KLRF1*, and *TYROBP* in XCL2_NK cluster; *XCL1*, *FCER1G*, *XCL2*, *AREG*, and *IL2RB* in XCL1_NK cluster. There were five clusters of T cell, including three CD8 T cell clusters, a CD4 T cell cluster and a γδ T cell cluster according to previous studies^44,45^. We annotated them according to their characteristic expression profiles as *GZMK*+ effect memory T cells (Tem), *FGFBP2*+ *NKG7*+ effect T cells (Teff), and *SLC4A10*+ mucosal-associated invariant T cells (MAIT). The top five highly expressed genes were *CD8B*, *GZMK*, *RGS1*, *CD8A*, and *COTL1* in CD8B_CD8 Tem; *CCL20*, *KLRB1*, *TRAV1-2*, *IL7R*, and *SLC4A10* in CCL20_CD8 MAIT; *IL7R*, *GPR183*, *LTB, RGCC*, and *LEF1* in IL7R_CD4 T; *TRBV9*, *TRAV38-2DV8*, *TRBV13*, *RP11-291B21.2*, and *TRGV5* in TRBV9_CD8 Teff; *TRDV2*, *TRGV9*, *KLRC1*, *TRDC*, and *TRAC* in TRDV2_γδ T. For the other two clusters that highly expressed proliferation markers such as *MKI67* in comparison with other clusters, we annotated them as cycling (*CD8*+) T and (*FCGR3A*+ *CD3D−*) NK cells based on previous studies^44,45^ (Fig. 5A, B, Supplementary Fig. S7B, Supplementary Table 14).

The results of GO analysis showed that T and NK cell clusters are commonly enriched in the *Lymphocyte activation* and *Adaptive immune response* pathways, and the DEGs of NK cell clusters were mainly enriched in the *Phagocytosis* and *Regulation of reactive oxygen species metabolic process*. In T cell clusters, different clusters had different patterns. CD4 T cell cluster was specifically enriched in *Eukaryotic Translation Elongation*. CD8B_CD8 Tem and TRBV9_CD8 Teff cell clusters were specifically enriched in *PD-1 signaling*, while γδ T cell cluster rarely had a common enrichment pathway with other clusters. STMN1_T-cycling and STMN1_NK-cycling were clustered with high proliferation ability, and the DEGs were mainly enriched in *DNA replication* and *Positive regulation of the cell cycle process* (Fig. 5C, Supplementary Fig. S7C).

In the study of the dynamic changes of NK and T cell clusters during liver transplantation, we found that IL7R_CD4 T, PTGDS_NK cells, TRBV9_CD8 Teff, and CD8B_CD8 Tem clusters were more widely distributed in the PR sample than EP and PP samples (Supplementary Fig. S7D). Compared with other clusters, the CCL20_CD8 MAIT cluster expressed more DEGs in the reperfusion stage, and the GNLY_NK cluster expressed more DEGs in the cold preservation stage, indicating that the CCL20_CD8 MAIT cluster was more sensitive to reperfusion injury and the GNLY_NK cluster was more sensitive to cold storage injury. TRDV2_γδ T cluster had almost no DEGs during IRI, suggesting that the TRDV2_γδ T cluster was not sensitive to IRI (Supplementary Fig. S7E). The amount of upregulated DEGs in the overall stage was slightly more than that in the reperfusion stage, and they shared most of the genes especially with high expression levels (Supplementary Fig. S7F, Supplementary Tables 15–17).

The GO analysis of DEGs in EP and PR samples showed that most of the cell clusters were activated in *TNF signaling pathway, NF-kappa B signaling pathway, Myeloid leukocyte activation, T cell activation*, and other pathways related to inflammation and cell activation in the reperfusion stage (Supplementary Fig. S8A, B). GSVA was performed to observe the dynamic changes of specific inflammatory pathways. We found that inflammation-related pathways, such as *IL23_PATHWAY*, *IL6_PATHWAY*, *TNF_PATHWAY*, and *NFKAPPAB_CANONICAL_PATHWAY* were obviously activated after reperfusion in most NK and T cell clusters. Cell cycle-related pathways, such as *ATM_PATHWAY*, *FOXM1_PATHWAY*, and *E2F_PATHWAY* were mainly expressed in cycling cell clusters. Pathways such as *FAS_PATHWAY*, *CXCR4_PATHWAY*, and *CXCR3_PATHWAY* were mainly expressed in most NK and T cell clusters except for CD8B_CD8 Tem, CCL20_CD8 MAIT, and IL7R_CD4 T cell clusters (Fig. 5D).

Mononuclear phagocyte clusters had universal interactions with NK/T cell clusters in cell-cell interaction analysis (Fig. 5E), including C5AR1_RPS19, CD55_ADGRE5, PLAUR_a4b1 complex, and other ligand–receptor pairs. Compared with the S100A8_TMo cluster, the VCAN_TMo cluster was enriched in ligand-receptor pairs such as CD74_MIF, CD74_COPA, and ICAM1_ITGAL in the interaction with NK and T cell clusters. The interactions between NK cell and KC cell clusters were specifically enriched in human leukocyte antigen (HLA) related and killer cell immunoglobulin-like receptors (KIR) related receptor-ligand pairs such as HLA−C_KIR2DL3 and HLA−B_KIR3DL2. The interactions between T cell and KC cell clusters (especially in C1QC_KC) were mainly enriched in chemokine ligand-related receptor-ligand pairs such as CXCL12_CXCR4, indicating the role of immune surveillance and T cell recruitment of C1QC_KC cluster^46^.

### B and plasma cells in graft IRI of liver transplantation

**Fig. 6:**
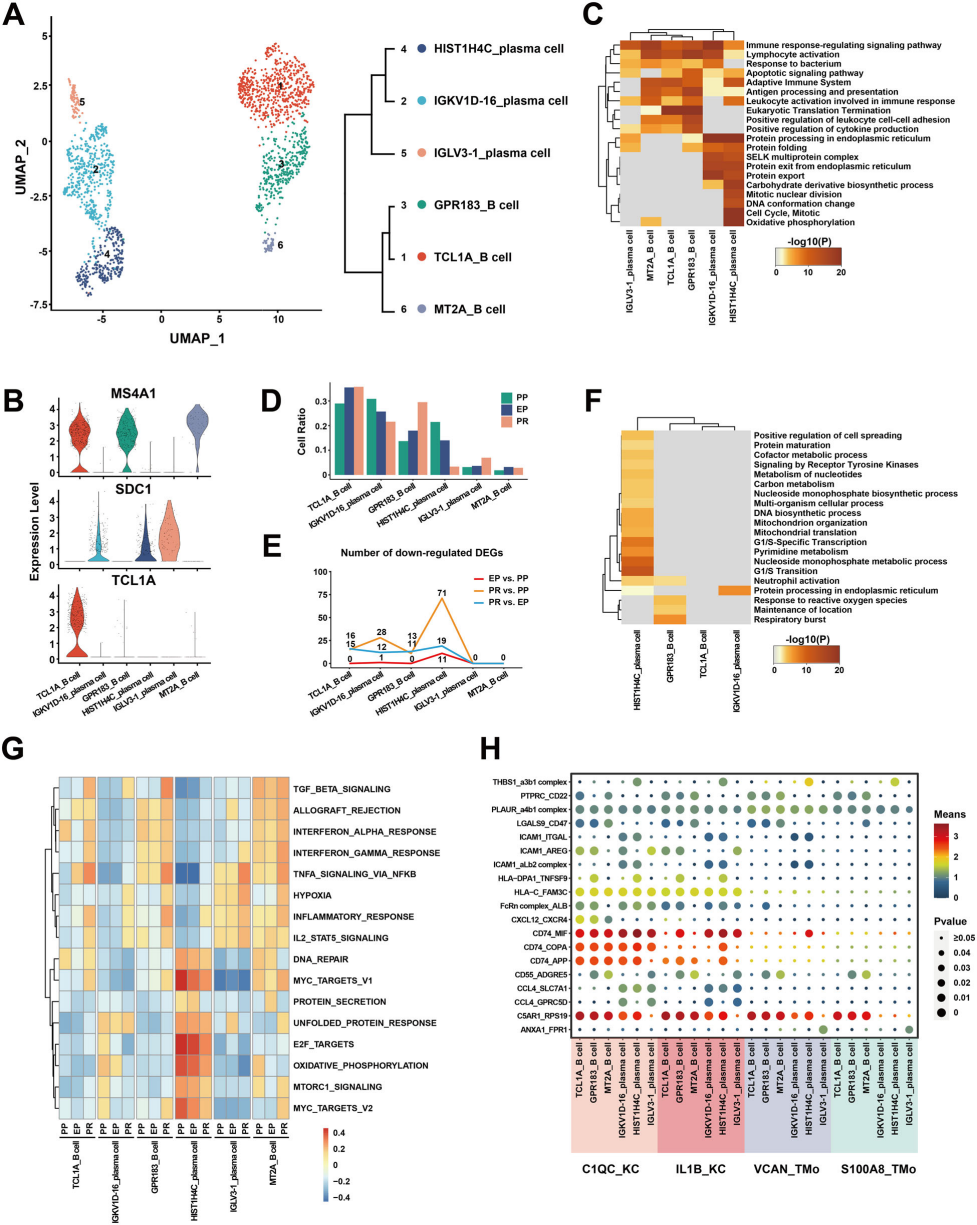
scRNA-seq of B/plasma cells in liver transplantation. **A** UMAP plot showing six B/plasma cell clusters in liver transplantation, colored according to different clusters (left panel). Dendrogram of six clusters by hierarchical clustering analysis based on their normalized mean expression values (right panel). **B** Violin plots showing the normalized expression of *MS4A1*, *SDC1*, and *TCL1A* genes (*y*-axis) for B/plasma cell clusters (*x*-axis). **C** Gene Ontology enrichment analysis results of B/plasma cell clusters. Only the top 20 most significant GO terms (*p* value < 0.05) are shown in rows. **D** Cell ratio of different B/plasma cell clusters in PP, EP, and PR samples. GSVA showing the pathways (PID gene sets) with significantly different activation in different samples of B/plasma cell clusters. Different colors represent different activation scores. **E** Number of down-regulated DEGs between different samples in different B/plasma cell clusters. **F** Gene Ontology enrichment analysis results of downregulated DEGs from PR vs. PP. Only the top 20 most significant GO terms (*p* value < 0.05) are shown in rows. **G** GSVA showing the pathways (HALLMARK gene sets) with significantly different activation in different samples of B/plasma cell clusters. Different colors represent different activation scores. **H** Cell–cell interaction analysis between mononuclear phagocyte clusters and different B/plasma cell clusters in PR samples. Ligand-receptor pairs labeled in *y*-axis. The size of the circle represents the level of *p* value, and different colors represent different means values. Ligands come from mononuclear phagocyte clusters, and receptors come from B/plasma cell clusters.

We obtained the single-cell transcriptomes of 1796 cells from the B and plasma cells lineage of three samples and reclustered them into six clusters (Fig. 6A, Supplementary Fig. S9A). These clusters uncovered three *MS4A1*+ B cell clusters and three *SDC1*+ plasma cell clusters in the liver with different canonical marker genes^27^. The top five highly expressed genes in the B cell clusters were *TCL1A, TXNIP, BTG1, CD37*, and *FCER2* in TCL1A_B cell cluster; *GPR183*, *TNF*, *COTL1*, *AC079767*, and *NR4A2* in GPR183_B cell cluster; *MT2A*, *FGR*, *FCRL3*, *FCRL5*, and *CIB1* in MT2A_B cell cluster. The top five highly expressed genes in the three plasma clusters were *IGKV1D-16*, *IGKV3D-20*, *IGKV1-16*, *IGHV3-72*, and *IGKV2D-28* in the IGKV1D-16_plasma cell cluster; *HIST1H4C*, *RRM2*, *GAPDH*, *HMGB2*, and *IGHV6-1* in the HIST1H4C_plasma cell cluster; *IGLV3-1*, *IGKV1D-39*, *CH17-224D4.2*, *IGKV1D-12*, and *IGLV3-21* in IGLV3-1_plasma cell cluster (Fig. 6B, Supplementary Fig. S9B, Supplementary Table 18).

The GO analysis results showed that different B and plasma cell clusters have different physiological functions. The common enrichment pathways of B cell and plasma cell clusters included *Lymphocyte activation*, *Immune response-regulating signaling pathway*, *Adaptive Immune System,* and so on. In addition, the B cell clusters were specifically enriched in the *Eukaryotic Translation Termination* and *Positive regulation of leukocyte cell–cell adhesion* pathways while the plasma cell clusters were mainly enriched in the *Protein processing in endoplasmic reticulum* and *Protein export* pathways. We observed that the *HIST1H4C*_plasma cell cluster was also specifically enriched in *Cell Cycle*, *DNA conformation change*, and *mitotic nuclear division* pathways, indicating that this cluster may have an active proliferation function (Fig. 6C).

In the study of dynamic changes at different stages of liver transplantation, we found that the number of DEGs in B and plasma cell clusters during the cold preservation stage and reperfusion stage was less than that of other cell lineages, suggesting that the expression profile of B and plasma cell clusters was not obviously altered after IRI (Supplementary Fig. S9C). Interestingly, we found that the number of downregulated genes in the *HIST1H4C*_plasma cell cluster increased during the cold preservation and reperfusion stage (Fig. 6E). These downregulated genes were mainly enriched in cell cycle-related pathways such as *G1/S Transition*, *G1/S-Specific Transcription*, and *mitochondrial translation* (Fig. 6F, Supplementary Fig. S9D–F, Supplementary Tables 19–21). At the same time, the results of GSVA analysis showed that the expression of genes in the HIST1H4C_plasma cell cluster related to *E2F_TARGETS*, *G2M_CHECKPOINT*, and *PROTEIN_SECRETION* pathways gradually decreased during the cold preservation and reperfusion stage (Fig. 6G). Combined with the proportional decrease in the cell number of HIST1H4C_plasma cell clusters throughout the PP, EP, and PR samples, we speculate that IRI can affect the cell cycle and reduce the cell proliferation of HIST1H4C_plasma cell cluster (Fig. 6D). In addition, we found that the APS of plasma cells was lower than that of B cells in MHC class II genes (*p* < 0.0001), and the APS of the HIST1H4C_plasma cell cluster decreased significantly after reperfusion (*p* = 0.0002).

In the study of the cell-cell interaction between mononuclear phagocyte and B plasma cell clusters after reperfusion, we found that the ligand-receptor pairs such as *PLAUR_a4b1 complex* and *C5AR1_RPS19* were commonly enriched between mononuclear phagocyte and B plasma cell clusters. The interactions between MP and B cells were specifically enriched in *LGALS9_CD47* and *PTPRC_CD22* and other cell-cell/cell-matrix interactions related functions^47,48^. In addition, ligand-receptor pairs such as *HLA*−*C_FAM3C*, *CD74_MIF, CD74_COPA*, *CD74_APP* which play an important role in IRI, were enriched in the interaction between KC and B plasma cell clusters^41–43^. Compare to the interactions between KC and B cell clusters, those between KC and plasma cell clusters were specifically enriched in *CCL4_SLC7A1* and *CCL4_GPRC5D* which may relate to possible chemotaxis function in KC clusters^49,50^ (Fig. 6H).

## Discussion

To the best of our knowledge, this study obtained the first unbiased and comprehensive liver transplant cell atlas by using scRNA-seq. We annotated the transcriptome characteristics of intrahepatic mononuclear phagocyte, endothelial, NK, T, B, and plasma cell clusters in detail and revealed the dynamic changes of transcriptome in each cluster during IRI. In addition, we identified the potential interactions between mononuclear phagocyte clusters and other cell clusters after graft reperfusion.

MacParland et al.^14^ had reported a map of the cellular landscape of the human liver using single-cell RNA sequencing and observed the existence of macrophages with different functions in fresh liver tissue. Different from them, in addition to the classification, annotation of cells, and the biological functions of different cell clusters in the liver, our study paid more attention to decoding the dynamic changes of the transcriptome of different cell clusters during IRI and expanded personalized bioinformatics analysis such as intercellular communication and antigen presentation capabilities. Not only that, for the study of macrophages with different anti-inflammatory and pro-inflammatory effects in the liver, MacParland et al.’s study mainly introduced the discovery that MARCO can be used as a marker gene to distinguish the anti-inflammatory and pro-inflammatory phenotypes of macrophages, which were also found in our liver transplantation scRNA-seq dataset (Supplementary Fig. S1D). In contrast, our research mainly introduced the changes of different anti-inflammatory and pro-inflammatory macrophage subpopulations in IRI, and the up-regulation of *TNIP3* by pro-inflammatory macrophage subpopulations after IRI might protect graft IRI via inhibition of the NFκB pathway.

Excessive inflammation caused by KCs is the key mechanism that causes pathological damage in IRI. After reperfusion, the accumulated endogenous damage-associated molecular patterns and pathogen-associated molecular patterns, are released into the liver, which activates KCs and produces ROS, TNF-α, IL-1β, and other pro-inflammatory cytokines, forming a positive feedback loop^8,51^. At the same time, these cytokines lead to the expression of adhesion molecules on the surface of LSEC and the recruitment of monocytes, neutrophils, and T cells^52,53^. On the other hand, some studies have reported that KCs can reduce liver IRI damage by producing nitric oxide to relax blood vessels, upregulate the expression of hemeoxygenase 1 to eliminate pro-inflammatory factors, and produce IL-10^30,54–56^. In this study, we demonstrated a possible protective effect of *TNIP3* expression in KC clusters during IRI. In recent studies, Liu et al.^57^ confirmed that adenovirus-mediated *TNIP3* expression in the liver blocked NASH progression in mice, indicating that *TNIP3* may be a promising therapeutic target for NASH. TNIP3 can bind to zinc finger protein TNFAIP3 and inhibit NF-κB activation induced by TNF, Toll-like receptor 4 (TLR4), interleukin-1, and 12-O-tetradecanoyl phorbol-13-acetate^58^. Consistent with previous studies^59,60^, the NFκB pathway was generally activated after reperfusion in mononuclear phagocyte clusters in our dataset. At the same time, we observed that KC clusters specifically showed increased expression of *TNIP3* after reperfusion and the *TNIP3* high expression group demonstrated a reduced level of postoperative liver injury. These results indicated that the inhibition of NF-κB pathway activation by *TNIP3* may be one of the mechanisms by which the KC cluster plays a protective role against graft IRI during liver transplantation.

We discovered the potential interaction pathways between MP and four LSEC clusters in our study. In the current dataset, four LSEC clusters specifically expressed *CLEC4M* and might receive *GRN* signaling molecules from MP. In previous studies, promoting *GRN* expression can reduce brain IRI-induced brain damage by inhibiting neuronal apoptosis and ROS production^61^; recombinant-GRN treatment can inhibit the recruitment of neutrophils and reduced the activation of NF-κB and MMP-9 in the brain IRI, and GRN has been reported to have a protective effect on inflammation caused by kidney and brain IRI^62,63^. Therefore, MP may express *GRN* and send a signal to the *CLEC4M* receptor on LSEC to reduce the recruitment of neutrophils, thereby reducing liver IRI. In addition, we discovered a special interaction mechanism between the C1QC_KC cluster and specific T and B cell clusters. Previous studies have confirmed that the expression of *CXCL12* increases in damaged liver tissue and contributes to the recruitment of *CXCR4*+ cells^64^. In our study, compared to other mononuclear phagocyte clusters, the C1QC_KC cluster specifically expresses *CXCL12* and sends a signal to the *CXCR4* receptor expressed on the T cell and B cell clusters, and increases the migration ability of the corresponding cell clusters.

We acknowledge that the current research has some limitations. Firstly, we did not obtain a sufficient number of hepatocytes and bile duct epithelial cells, which also play a very important role in IRI. The hepatocyte populations are particularly susceptible to dissociation. Nowadays, the collagenase perfusion method is routinely used to separate hepatocytes, but due to the need to obtain liver tissues from the same donor at different time points and the limited sample size, we cannot use the collagenase perfusion method to separate cells. Secondly, our samples have no biological replicates, a larger sample size will strengthen our conclusions. Notably, each sample had a sufficient number of thousands of cells, which enables us to understand the heterogeneity between cells. The cell clustering and annotation results in this study are consistent with previous studies^14,29^. Furthermore, in terms of important molecular mechanisms, we combined bulk RNA-seq data, immunofluorescence, Western Blot, and RT-PCR experiments to verify our conclusions.

Overall, we have obtained the first unbiased and comprehensive liver transplant cell atlas. We annotated the subpopulations of multiple cell types and described the dynamic changes of their transcriptome in IRI and the interaction of mononuclear phagocyte clusters with other cell clusters after reperfusion, which allows us to investigate the mechanism of IRI from single-cell resolution. At the same time, the *TNIP3* gene which is specifically upregulated in KC clusters after reperfusion may be a potential therapeutic target of IRI.

## Data sharing

The fastq file of the raw sequencing data and the processed data counts file have been deposited in Gene Expression Omnibus (GEO) repository with the primary accession code GSE171539 (SRA: SRP313633). All related codes and data analysis scripts are available at github (https://github.com/Leowangsysu/Guo_lab).

## Supplementary information

Supplementary Figures

Supplementary Tables
