# Supplementary material for: Resolving the graft ischemia-reperfusion injury during liver transplantation at the single cell resolution
Source: Cell Death Dis. 2021 Jun 8;12(6):589. doi: 10.1038/s41419-021-03878-3 (PMC8187624; doi:10.1038/s41419-021-03878-3)
Supplement: Supplementary file 2 — Supplementary Tables [file 41419_2021_3878_MOESM2_ESM.pdf]

Table 1. Basal information of sequencing and data processing.

Sample	PP	EP	PR
Library Type		cDNA	
Number of read pairs	421386589	420211932	425404590
Mean Reads per Cell	32414	32323	32723
Sequencing Saturation	84.20%	86.00%	91.00%
Cell number (original)	7133	6719	4941
Cell number (doublets removed)	443	415	302
Cell number (after QC)	5469	5120	3724
Median UMI (after QC)	3796	3339	3262
Median genes (after QC)	1474	1383	1262

Table 2. List of markers information for each cluster, related to Figure 1.

	p_val	avg_logFC	pct.1	pct.2	p_val_adj	gene	cluster	cell type
1	0	2.946714536	0.927	0.086	0	GNLY	1	GNLY_NK cell
2	0	2.2896698	0.961	0.109	0	GZMB	1	GNLY_NK cell
3	0	2.248770851	0.881	0.058	0	FGFBP2	1	GNLY_NK cell
4	0	2.036317689	0.998	0.387	0	NKG7	1	GNLY_NK cell
5	1.87E-258	2.018026522	0.184	0.017	4.09E-254	PTGDS	1	GNLY_NK cell
6	0	1.997748095	0.968	0.234	0	PRF1	1	GNLY_NK cell
7	0	1.90775879	0.797	0.119	0	SPON2	1	GNLY_NK cell
8	0	1.87810219	0.961	0.171	0	KLRD1	1	GNLY_NK cell
9	0	1.753542083	0.928	0.221	0	CTSW	1	GNLY_NK cell
10	0	1.711648376	0.83	0.084	0	KLRF1	1	GNLY_NK cell
11	0	1.64636499	0.828	0.159	0	GZMH	1	GNLY_NK cell
12	0	1.645136324	0.877	0.192	0	CD247	1	GNLY_NK cell
13	0	1.632724003	0.985	0.333	0	CST7	1	GNLY_NK cell
14	0	1.539902791	0.799	0.155	0	TRBC1	1	GNLY_NK cell
15	0	1.451825527	0.572	0.069	0	CLIC3	1	GNLY_NK cell
16	0	1.397824627	0.867	0.234	0	FCGR3A	1	GNLY_NK cell
17	0	1.370861244	0.724	0.181	0	HOPX	1	GNLY_NK cell
18	0	1.344134679	0.766	0.285	0	CMC1	1	GNLY_NK cell
19	0	1.336806044	0.614	0.101	0	ADGRG1	1	GNLY_NK cell
20	0	1.336247682	0.51	0.034	0	SH2D1B	1	GNLY_NK cell
21	0	1.325040532	0.55	0.071	0	KLRC3	1	GNLY_NK cell
22	0	1.314346597	0.48	0.031	0	KIR3DL1	1	GNLY_NK cell
23	0	1.246868403	0.937	0.358	0	CCL5	1	GNLY_NK cell
24	0	1.205137442	0.84	0.273	0	CD7	1	GNLY_NK cell
25	0	1.16151525	0.904	0.3	0	GZMA	1	GNLY_NK cell
26	0	1.147926811	0.987	0.532	0	IFITM1	1	GNLY_NK cell
27	0	1.109464676	0.946	0.485	0	CCL4	1	GNLY_NK cell
28	0	1.078570064	0.384	0.038	0	CX3CR1	1	GNLY_NK cell
29	0	1.054676459	0.452	0.069	0	S1PR5	1	GNLY_NK cell
30	0	1.022572035	0.304	0.017	0	KIR2DL1	1	GNLY_NK cell
31	0	1.014912726	0.647	0.184	0	GZMM	1	GNLY_NK cell
32	0	0.99182733	0.802	0.368	0	IL2RG	1	GNLY_NK cell
33	0	0.987345894	0.406	0.082	0	TTC38	1	GNLY_NK cell
34	3.20E-292	0.981219527	0.712	0.332	6.97E-288	GNG2	1	GNLY_NK cell
35	0	0.964294551	0.546	0.149	0	ZAP70	1	GNLY_NK cell
36	0	0.962212772	0.537	0.16	0	C12orf75	1	GNLY_NK cell
37	2.92E-265	0.9593142	0.533	0.162	6.36E-261	IFNG	1	GNLY_NK cell
38	0	0.946288552	0.215	0.017	0	LAIR2	1	GNLY_NK cell
39	0	0.934470894	0.388	0.05	0	FCRL6	1	GNLY_NK cell
40	6.04E-156	0.930010627	0.608	0.303	1.32E-151	CCL3	1	GNLY_NK cell
41	2.51E-304	0.926425002	0.523	0.152	5.47E-300	PYHIN1	1	GNLY_NK cell
42	4.14E-198	0.899539635	0.731	0.354	9.03E-194	CCL4L2	1	GNLY_NK cell
43	0	0.897192203	0.369	0.044	0	TRDC	1	GNLY_NK cell
44	0	0.896990433	0.391	0.073	0	TXK	1	GNLY_NK cell
45	1.50E-233	0.895192929	0.585	0.266	3.27E-229	APMAP	1	GNLY_NK cell
46	0	0.891915155	0.9	0.58	0	HCST	1	GNLY_NK cell
47	1.61E-204	0.874005078	0.54	0.245	3.51E-200	MBP	1	GNLY_NK cell
48	7.33E-240	0.87388722	0.56	0.208	1.60E-235	SYTL3	1	GNLY_NK cell
49	0	0.861758503	0.247	0.016	0	KIR2DL3	1	GNLY_NK cell
50	0	0.85724835	0.877	0.444	0	PTPRCAP	1	GNLY_NK cell
51	0	2.228798658	0.962	0.21	0	KLRB1	2	KLRB1_T cell
52	0	2.191354319	0.974	0.122	0	IL7R	2	KLRB1_T cell
53	0	1.83725759	0.308	0.035	0	CCL20	2	KLRB1_T cell
54	0	1.53695444	0.755	0.235	0	TNF	2	KLRB1_T cell
55	0	1.520137453	0.991	0.436	0	CD69	2	KLRB1_T cell
56	0	1.49186976	0.578	0.013	0	TRAV1-2	2	KLRB1_T cell
57	0	1.429001841	0.726	0.121	0	KLRG1	2	KLRB1_T cell
58	0	1.324627489	0.543	0.012	0	SLC4A10	2	KLRB1_T cell
59	0	1.284735627	0.723	0.156	0	LTB	2	KLRB1_T cell
60	0	1.269568865	0.31	0.009	0	TRBV6-1	2	KLRB1_T cell
61	0	1.195004943	0.827	0.146	0	GZMK	2	KLRB1_T cell
62	0	1.194225151	0.983	0.381	0	IL32	2	KLRB1_T cell
63	0	1.19130666	0.935	0.516	0	DUSP2	2	KLRB1_T cell
64	0	1.146937518	0.677	0.128	0	SPOCK2	2	KLRB1_T cell
65	1.24E-168	1.125994062	0.563	0.226	2.70E-164	CCL3L3	2	KLRB1_T cell
66	0	1.106137194	0.818	0.167	0	CD3D	2	KLRB1_T cell
67	0	1.102031082	0.594	0.109	0	NCR3	2	KLRB1_T cell
68	0	1.05346551	0.678	0.139	0	CD8A	2	KLRB1_T cell
69	0	1.046683484	0.336	0.044	0	KLRC1	2	KLRB1_T cell
70	0	1.033578537	0.495	0.071	0	AC092580.4	2	KLRB1_T cell
71	0	1.032892222	0.414	0.023	0	CD40LG	2	KLRB1_T cell

72	0	1.028675801	0.616	0.168	0	RORA	2	KLRB1_T cell
73	2.04E-179	1.026427017	0.777	0.471	4.45E-175	TNFAIP3	2	KLRB1_T cell
74	2.44E-249	1.016130574	0.921	0.5	5.32E-245	CCL4	2	KLRB1_T cell
75	0	1.010890153	0.729	0.227	0	PARP8	2	KLRB1_T cell
76	6.42E-250	0.995316494	0.95	0.786	1.40E-245	NFKBIA	2	KLRB1_T cell
77	3.79E-165	0.986371455	0.73	0.365	8.27E-161	CCL4L2	2	KLRB1_T cell
78	0	0.984482881	0.959	0.321	0	CD3E	2	KLRB1_T cell
79	0	0.980675216	0.425	0.036	0	CXCR6	2	KLRB1_T cell
80	0	0.979254566	0.648	0.159	0	CD96	2	KLRB1_T cell
81	0	0.976991618	0.175	0.006	0	TRAV13-2	2	KLRB1_T cell
82	2.99E-209	0.925196891	0.715	0.346	6.52E-205	GPR65	2	KLRB1_T cell
83	9.91E-289	0.912704321	0.514	0.131	2.16E-284	SATB1	2	KLRB1_T cell
84	0	0.911905252	0.61	0.122	0	CD3G	2	KLRB1_T cell
85	1.84E-255	0.878614464	0.957	0.633	4.02E-251	CXCR4	2	KLRB1_T cell
86	0	0.867495965	0.44	0.053	0	CAMK4	2	KLRB1_T cell
87	0	0.859545907	0.602	0.152	0	TC2N	2	KLRB1_T cell
88	0	0.856287302	0.148	0.004	0	TRBV6-4	2	KLRB1_T cell
89	4.04E-246	0.849429143	0.494	0.14	8.81E-242	AQP3	2	KLRB1_T cell
90	0	0.83923991	0.392	0.042	0	TMIGD2	2	KLRB1_T cell
91	0	0.826568514	0.349	0.025	0	CCR6	2	KLRB1_T cell
92	1.28E-133	0.825727486	0.681	0.411	2.79E-129	BTG2	2	KLRB1_T cell
93	3.26E-244	0.820604279	0.523	0.148	7.12E-240	GBP5	2	KLRB1_T cell
94	3.63E-242	0.790740506	0.983	0.812	7.91E-238	ZFP36L2	2	KLRB1_T cell
95	6.75E-150	0.782983596	0.73	0.441	1.47E-145	NFKBIZ	2	KLRB1_T cell
96	0	0.776422729	0.363	0.055	0	GPR171	2	KLRB1_T cell
97	6.46E-279	0.772707529	0.747	0.244	1.41E-274	CD2	2	KLRB1_T cell
98	1.90E-272	0.758693937	0.881	0.319	4.13E-268	GZMA	2	KLRB1_T cell
99	2.11E-51	0.756258888	0.366	0.188	4.61E-47	IFNG	2	KLRB1_T cell
100	1.01E-151	0.751000787	0.741	0.424	2.20E-147	TAGAP	2	KLRB1_T cell
101	0	3.556750337	0.996	0.44	0	S100A8	3	S100A8_MP
102	0	3.248529577	0.957	0.136	0	S100A12	3	S100A8_MP
103	0	3.148871786	0.98	0.347	0	S100A9	3	S100A8_MP
104	0	2.626882239	0.987	0.235	0	LYZ	3	S100A8_MP
105	0	2.589417168	0.919	0.079	0	VCAN	3	S100A8_MP
106	0	2.022821736	0.887	0.113	0	FCN1	3	S100A8_MP
107	0	1.865658139	0.997	0.725	0	S100A6	3	S100A8_MP
108	0	1.857638277	0.84	0.156	0	MNDA	3	S100A8_MP
109	0	1.836078097	0.853	0.134	0	CSTA	3	S100A8_MP
110	0	1.822055658	0.738	0.157	0	THBS1	3	S100A8_MP
111	0	1.728248465	0.85	0.253	0	GCA	3	S100A8_MP
112	0	1.618864344	0.808	0.277	0	STXBP2	3	S100A8_MP
113	0	1.596737967	0.672	0.087	0	PLBD1	3	S100A8_MP
114	0	1.558880861	0.497	0.059	0	IL1R2	3	S100A8_MP
115	0	1.483694582	0.929	0.455	0	TSPO	3	S100A8_MP
116	0	1.451023805	0.711	0.129	0	FPR1	3	S100A8_MP
117	0	1.434592411	0.978	0.581	0	S100A11	3	S100A8_MP
118	0	1.428504987	0.977	0.28	0	FCER1G	3	S100A8_MP
119	0	1.382718193	0.318	0.018	0	RETN	3	S100A8_MP
120	0	1.379654168	0.986	0.648	0	S100A4	3	S100A8_MP
121	0	1.357054865	0.747	0.225	0	PGD	3	S100A8_MP
122	0	1.336265017	0.669	0.11	0	LILRA5	3	S100A8_MP
123	0	1.332188568	0.926	0.493	0	LGALS1	3	S100A8_MP
124	0	1.316291262	0.963	0.693	0	S100A10	3	S100A8_MP
125	0	1.294347858	0.729	0.196	0	PLAUR	3	S100A8_MP
126	0	1.288163622	0.64	0.134	0	SELL	3	S100A8_MP
127	0	1.271775974	0.677	0.141	0	SLC11A1	3	S100A8_MP
128	0	1.265178389	0.576	0.148	0	CXCL8	3	S100A8_MP
129	0	1.257438285	0.503	0.032	0	MCEMP1	3	S100A8_MP
130	0	1.210960868	0.888	0.483	0	SERPINB1	3	S100A8_MP
131	0	1.171805653	0.424	0.063	0	EREG	3	S100A8_MP
132	0	1.155521034	0.937	0.462	0	COTL1	3	S100A8_MP
133	0	1.138676132	0.909	0.221	0	AIF1	3	S100A8_MP
134	0	1.133932416	0.58	0.105	0	CAPG	3	S100A8_MP
135	0	1.133239786	0.669	0.186	0	IFI30	3	S100A8_MP
136	4.30E-271	1.119100799	0.829	0.412	9.38E-267	NAMPT	3	S100A8_MP
137	0	1.118935569	0.781	0.232	0	C1orf162	3	S100A8_MP
138	0	1.115060661	0.987	0.439	0	TYROBP	3	S100A8_MP
139	0	1.109030458	0.713	0.171	0	C5AR1	3	S100A8_MP
140	0	1.108145959	0.992	0.859	0	SH3BGRL3	3	S100A8_MP
141	0	1.09616965	0.706	0.208	0	SPI1	3	S100A8_MP
142	0	1.091881068	0.471	0.053	0	CDA	3	S100A8_MP
143	0	1.091588867	0.574	0.064	0	MGST1	3	S100A8_MP
144	3.27E-303	1.088426723	0.553	0.146	7.14E-299	ACSL1	3	S100A8_MP

145	2.31E-274	1.077488858	0.759	0.374	5.05E-270	TKT	3	S100A8_MP
146	2.55E-277	1.077171259	0.689	0.287	5.57E-273	APLP2	3	S100A8_MP
147	3.37E-306	1.06177404	0.299	0.036	7.34E-302	TNFAIP6	3	S100A8_MP
148	8.34E-280	1.060855527	0.742	0.37	1.82E-275	TALDO1	3	S100A8_MP
149	0	1.056413704	0.402	0.044	0	RP11-1143G9.4	3	S100A8_MP
150	1.79E-306	1.04946038	0.666	0.231	3.89E-302	TYMP	3	S100A8_MP
151	0	1.753024554	0.815	0.151	0	GZMK	4	GZMK_T cell
152	0	1.487362952	0.718	0.139	0	CD8A	4	GZMK_T cell
153	0	1.44870455	0.532	0.061	0	CD8B	4	GZMK_T cell
154	2.65E-291	1.406053335	0.622	0.203	5.78E-287	RGS1	4	GZMK_T cell
155	4.24E-268	1.401380412	0.474	0.108	9.24E-264	XCL2	4	GZMK_T cell
156	2.83E-178	1.179045981	0.745	0.366	6.17E-174	CCL4L2	4	GZMK_T cell
157	8.62E-172	1.160846947	0.345	0.084	1.88E-167	XCL1	4	GZMK_T cell
158	0	1.147093454	0.712	0.18	0	CD3D	4	GZMK_T cell
159	0	1.140939115	0.931	0.328	0	CD3E	4	GZMK_T cell
160	0	1.133812905	0.973	0.375	0	CCL5	4	GZMK_T cell
161	5.80E-271	1.054415251	0.7	0.251	1.26E-266	CD2	4	GZMK_T cell
162	1.06E-181	0.962514384	0.327	0.076	2.31E-177	CRTAM	4	GZMK_T cell
163	2.36E-279	0.91885868	0.907	0.391	5.14E-275	IL32	4	GZMK_T cell
164	2.46E-150	0.912216522	0.636	0.312	5.36E-146	CMC1	4	GZMK_T cell
165	4.28E-252	0.901306156	0.509	0.134	9.34E-248	CD3G	4	GZMK_T cell
166	5.78E-223	0.865420489	0.968	0.814	1.26E-218	ZFP36L2	4	GZMK_T cell
167	1.22E-205	0.865268031	0.925	0.503	2.66E-201	CCL4	4	GZMK_T cell
168	2.73E-163	0.851413954	0.438	0.147	5.96E-159	SH2D1A	4	GZMK_T cell
169	2.62E-121	0.841215095	0.463	0.18	5.71E-117	IFNG	4	GZMK_T cell
170	3.78E-202	0.837838478	0.874	0.524	8.24E-198	DUSP2	4	GZMK_T cell
171	3.41E-114	0.797964714	0.35	0.126	7.44E-110	CD27	4	GZMK_T cell
172	1.80E-211	0.79422746	0.798	0.33	3.92E-207	GZMA	4	GZMK_T cell
173	1.44E-87	0.791931173	0.443	0.23	3.15E-83	ITM2A	4	GZMK_T cell
174	2.67E-131	0.784592684	0.76	0.577	5.82E-127	SRSF7	4	GZMK_T cell
175	3.05E-119	0.756368361	0.567	0.29	6.66E-115	FYN	4	GZMK_T cell
176	4.46E-165	0.746043499	0.905	0.64	9.72E-161	CXCR4	4	GZMK_T cell
177	6.15E-216	0.735076235	0.918	0.446	1.34E-211	CD69	4	GZMK_T cell
178	1.88E-67	0.734325129	0.516	0.345	4.09E-63	PIK3R1	4	GZMK_T cell
179	1.64E-107	0.727315181	0.524	0.255	3.57E-103	TRBC2	4	GZMK_T cell
180	3.37E-151	0.710260143	0.278	0.064	7.34E-147	THEMIS	4	GZMK_T cell
181	0	0.709748914	0.995	0.986	0	RPS27	4	GZMK_T cell
182	1.45E-217	0.708145324	0.198	0.021	3.16E-213	PDCD1	4	GZMK_T cell
183	1.03E-124	0.701416257	0.529	0.234	2.24E-120	LCK	4	GZMK_T cell
184	1.88E-50	0.691392731	0.35	0.197	4.11E-46	TSPYL2	4	GZMK_T cell
185	1.49E-155	0.688432916	0.819	0.464	3.24E-151	PTPRCAP	4	GZMK_T cell
186	2.24E-76	0.687034581	0.735	0.592	4.89E-72	DNAJB1	4	GZMK_T cell
187	1.65E-91	0.685069958	0.536	0.293	3.60E-87	LBH	4	GZMK_T cell
188	6.58E-55	0.676055855	0.439	0.283	1.44E-50	TUBA4A	4	GZMK_T cell
189	1.46E-162	0.669524732	0.975	0.914	3.19E-158	JUNB	4	GZMK_T cell
190	4.30E-164	0.668626755	0.925	0.704	9.37E-160	PTPRC	4	GZMK_T cell
191	2.15E-107	0.668038871	0.418	0.171	4.69E-103	TC2N	4	GZMK_T cell
192	1.62E-45	0.667051034	0.441	0.312	3.54E-41	KIAA1551	4	GZMK_T cell
193	2.04E-91	0.662917128	0.57	0.339	4.44E-87	STK17A	4	GZMK_T cell
194	6.02E-215	0.657958327	0.968	0.912	1.31E-210	RPS29	4	GZMK_T cell
195	6.04E-107	0.651194809	0.483	0.213	1.32E-102	GZMM	4	GZMK_T cell
196	4.51E-92	0.650444525	0.571	0.34	9.83E-88	RARRES3	4	GZMK_T cell
197	2.63E-109	0.648196415	0.27	0.078	5.75E-105	INPP4B	4	GZMK_T cell
198	3.01E-69	0.646290816	0.431	0.239	6.57E-65	1-Sep	4	GZMK_T cell
199	5.98E-40	0.623494097	0.289	0.166	1.31E-35	SRRT	4	GZMK_T cell
200	1.90E-73	0.61928477	0.255	0.092	4.14E-69	TRAC	4	GZMK_T cell
201	0	3.006177973	0.995	0.086	0	C1QB	5	C1QB_MP
202	0	2.947844656	0.993	0.084	0	C1QC	5	C1QB_MP
203	0	2.870768197	0.993	0.092	0	C1QA	5	C1QB_MP
204	0	2.211068615	0.955	0.232	0	LIPA	5	C1QB_MP
205	0	2.183121462	0.902	0.154	0	SLC40A1	5	C1QB_MP
206	0	2.163503243	0.998	0.385	0	CST3	5	C1QB_MP
207	0	2.078351842	0.985	0.237	0	CD68	5	C1QB_MP
208	0	2.037555305	0.885	0.048	0	FOLR2	5	C1QB_MP
209	0	1.991344996	0.955	0.121	0	MS4A7	5	C1QB_MP
210	0	1.978118609	0.932	0.115	0	MS4A4A	5	C1QB_MP
211	0	1.960133407	0.926	0.154	0	HMOX1	5	C1QB_MP
212	0	1.928451883	0.98	0.181	0	CD163	5	C1QB_MP
213	0	1.915128147	0.993	0.483	0	HLA-DRB5	5	C1QB_MP
214	0	1.880048703	0.678	0.054	0	MARCO	5	C1QB_MP
215	0	1.860042156	1	0.978	0	FTL	5	C1QB_MP
216	0	1.783210596	0.974	0.379	0	CTSB	5	C1QB_MP
217	0	1.762969199	0.983	0.269	0	MS4A6A	5	C1QB_MP

218	0	1.720388279	0.85	0.075	0	VSIG4	5	C1QB_MP
219	0	1.648141007	0.916	0.202	0	CREG1	5	C1QB_MP
220	0	1.607684615	0.982	0.217	0	AIF1	5	C1QB_MP
221	0	1.607332646	0.992	0.473	0	NPC2	5	C1QB_MP
222	0	1.591185773	0.508	0.053	0	APOE	5	C1QB_MP
223	0	1.533384234	0.969	0.579	0	PSAP	5	C1QB_MP
224	0	1.518228712	0.995	0.44	0	HLA-DRA	5	C1QB_MP
225	0	1.506613629	0.975	0.386	0	CTSS	5	C1QB_MP
226	0	1.499928154	0.979	0.527	0	HLA-DPA1	5	C1QB_MP
227	0	1.487909692	0.833	0.085	0	CSF1R	5	C1QB_MP
228	0	1.481915284	1	0.823	0	CD74	5	C1QB_MP
229	0	1.462063145	0.828	0.117	0	IGSF6	5	C1QB_MP
230	0	1.437630153	0.834	0.155	0	CFD	5	C1QB_MP
231	0	1.417266014	0.684	0.033	0	GPNMB	5	C1QB_MP
232	0	1.401409268	0.942	0.327	0	RNASET2	5	C1QB_MP
233	0	1.369128355	0.795	0.126	0	CPM	5	C1QB_MP
234	0	1.367189972	0.904	0.208	0	MAFB	5	C1QB_MP
235	0	1.358546037	0.939	0.319	0	FCGRT	5	C1QB_MP
236	0	1.346971438	0.975	0.495	0	HLA-DPB1	5	C1QB_MP
237	0	1.307116576	0.777	0.122	0	CPVL	5	C1QB_MP
238	0	1.263318861	0.929	0.219	0	LST1	5	C1QB_MP
239	0	1.25094162	0.754	0.107	0	MCOLN1	5	C1QB_MP
240	0	1.244671357	0.984	0.282	0	FCER1G	5	C1QB_MP
241	0	1.237204485	0.76	0.118	0	RBP7	5	C1QB_MP
242	0	1.229866511	0.822	0.133	0	FAM26F	5	C1QB_MP
243	0	1.229801125	0.923	0.407	0	GLUL	5	C1QB_MP
244	0	1.22664113	0.71	0.085	0	APOC1	5	C1QB_MP
245	0	1.225554444	0.925	0.223	0	CD302	5	C1QB_MP
246	0	1.217986513	0.855	0.191	0	FGL2	5	C1QB_MP
247	0	1.217283165	0.925	0.284	0	BLVRB	5	C1QB_MP
248	0	1.21583156	0.514	0.029	0	TMIGD3	5	C1QB_MP
249	0	1.215733987	0.701	0.157	0	PDK4	5	C1QB_MP
250	0	1.214180912	0.905	0.363	0	GRN	5	C1QB_MP
251	0	2.332873622	0.835	0.025	0	MS4A1	6	MS4A1_B cell
252	0	2.322939003	0.908	0.074	0	CD79A	6	MS4A1_B cell
253	0	1.919717129	0.809	0.247	0	CD83	6	MS4A1_B cell
254	0	1.71024699	0.979	0.576	0	CD37	6	MS4A1_B cell
255	0	1.568192176	0.38	0.003	0	TCL1A	6	MS4A1_B cell
256	0	1.550713943	0.999	0.443	0	HLA-DRA	6	MS4A1_B cell
257	0	1.545759318	0.59	0.016	0	BANK1	6	MS4A1_B cell
258	0	1.538800048	0.899	0.279	0	HLA-DQA1	6	MS4A1_B cell
259	0	1.504055472	0.524	0.007	0	LINC00926	6	MS4A1_B cell
260	0	1.470337914	1	0.824	0	CD74	6	MS4A1_B cell
261	0	1.350684909	0.937	0.457	0	HLA-DQB1	6	MS4A1_B cell
262	0	1.332881709	0.686	0.168	0	LTB	6	MS4A1_B cell
263	0	1.2693093	0.532	0.121	0	CD79B	6	MS4A1_B cell
264	0	1.190276148	0.405	0.03	0	ADAM28	6	MS4A1_B cell
265	3.42E-302	1.189012724	0.9	0.499	7.46E-298	CD52	6	MS4A1_B cell
266	4.06E-271	1.18346695	0.571	0.174	8.85E-267	IRF8	6	MS4A1_B cell
267	0	1.180363923	0.3	0.01	0	STAG3	6	MS4A1_B cell
268	0	1.148653415	0.344	0.011	0	FCER2	6	MS4A1_B cell
269	0	1.127407037	0.401	0.016	0	AFF3	6	MS4A1_B cell
270	0	1.119434334	0.361	0.002	0	FAM129C	6	MS4A1_B cell
271	0	1.107777217	0.376	0.012	0	FCRLA	6	MS4A1_B cell
272	1.25E-259	1.107239395	0.627	0.209	2.73E-255	FCMR	6	MS4A1_B cell
273	6.36E-271	1.068497751	0.448	0.102	1.39E-266	HVCN1	6	MS4A1_B cell
274	0	1.059565918	0.343	0.02	0	BACH2	6	MS4A1_B cell
275	0	1.05536879	0.403	0.054	0	RALGPS2	6	MS4A1_B cell
276	2.84E-150	1.046074687	0.69	0.415	6.19E-146	EZR	6	MS4A1_B cell
277	0	1.028821165	0.37	0.036	0	ARHGAP24	6	MS4A1_B cell
278	4.39E-211	1.018764885	0.428	0.109	9.57E-207	LY9	6	MS4A1_B cell
279	2.66E-280	1.005279609	0.952	0.682	5.79E-276	LAPTM5	6	MS4A1_B cell
280	0	0.994677333	0.316	0.003	0	CXCR5	6	MS4A1_B cell
281	0	0.980164143	0.312	0.005	0	RP11-693J15.5	6	MS4A1_B cell
282	5.19E-167	0.977757095	0.531	0.204	1.13E-162	BIRC3	6	MS4A1_B cell
283	0	0.976751743	0.99	0.924	0	RPS5	6	MS4A1_B cell
284	2.19E-272	0.96196454	0.915	0.503	4.78E-268	HLA-DPB1	6	MS4A1_B cell
285	7.05E-142	0.960350248	0.49	0.191	1.54E-137	GPR183	6	MS4A1_B cell
286	3.38E-146	0.950210657	0.577	0.283	7.36E-142	MEF2C	6	MS4A1_B cell
287	4.68E-278	0.946630454	0.992	0.86	1.02E-273	BTG1	6	MS4A1_B cell
288	0	0.943752194	0.329	0.022	0	CD19	6	MS4A1_B cell
289	0	0.941036141	0.278	0.003	0	AC079767.4	6	MS4A1_B cell
290	0	0.933222027	0.329	0.015	0	BLK	6	MS4A1_B cell

291	0	0.926272497	0.292	0.005	0	CD22	6	MS4A1_B cell
292	0	0.916432554	0.998	0.983	0	RPS19	6	MS4A1_B cell
293	0	0.905041406	0.996	0.977	0	RPL18A	6	MS4A1_B cell
294	9.33E-109	0.890517047	0.78	0.621	2.04E-104	HERPUD1	6	MS4A1_B cell
295	0	0.882539584	0.273	0.012	0	CCR7	6	MS4A1_B cell
296	2.92E-121	0.869382284	0.709	0.475	6.36E-117	CD55	6	MS4A1_B cell
297	6.21E-138	0.867788556	0.519	0.227	1.35E-133	NCF1	6	MS4A1_B cell
298	0	0.864209356	0.261	0.005	0	FCRL1	6	MS4A1_B cell
299	7.71E-227	0.863107344	0.956	0.677	1.68E-222	HLA-DRB1	6	MS4A1_B cell
300	0	0.856640055	0.258	0.003	0	VPREB3	6	MS4A1_B cell
301	0	2.969477079	0.982	0.048	0	FCN2	7	FCN2_Endo
302	0	2.962846266	0.989	0.07	0	FCN3	7	FCN2_Endo
303	0	2.955881941	0.998	0.074	0	DNASE1L3	7	FCN2_Endo
304	0	2.775391757	0.967	0.047	0	CLEC4G	7	FCN2_Endo
305	0	2.701604924	0.987	0.057	0	CRHBP	7	FCN2_Endo
306	0	2.671816857	0.999	0.095	0	CCL14	7	FCN2_Endo
307	0	2.526476422	0.981	0.239	0	CTSL	7	FCN2_Endo
308	0	2.488849198	0.902	0.03	0	CLEC4M	7	FCN2_Endo
309	0	2.421825092	0.97	0.034	0	OIT3	7	FCN2_Endo
310	0	2.379842891	0.947	0.135	0	FCGR2B	7	FCN2_Endo
311	0	2.376264391	0.97	0.17	0	ACP5	7	FCN2_Endo
312	0	2.317665494	0.995	0.109	0	TFPI	7	FCN2_Endo
313	0	2.259690376	0.94	0.032	0	CLEC1B	7	FCN2_Endo
314	0	2.202994782	0.957	0.053	0	AKAP12	7	FCN2_Endo
315	0	2.090190361	0.969	0.066	0	RAMP3	7	FCN2_Endo
316	0	2.087795059	0.979	0.098	0	IGFBP4	7	FCN2_Endo
317	0	2.081478676	0.982	0.136	0	ENG	7	FCN2_Endo
318	0	2.073050686	0.948	0.065	0	PLPP3	7	FCN2_Endo
319	0	2.065638363	0.985	0.193	0	SEPP1	7	FCN2_Endo
320	0	2.058992126	0.995	0.165	0	APP	7	FCN2_Endo
321	0	2.051543208	0.975	0.07	0	SPARC	7	FCN2_Endo
322	0	2.038502056	0.975	0.181	0	LGMN	7	FCN2_Endo
323	0	2.021038493	0.978	0.061	0	TSPAN7	7	FCN2_Endo
324	0	1.946405944	0.983	0.092	0	TM4SF1	7	FCN2_Endo
325	0	1.936654824	0.979	0.07	0	IL33	7	FCN2_Endo
326	0	1.921837395	0.768	0.032	0	CCL23	7	FCN2_Endo
327	0	1.898274866	0.955	0.075	0	EGFL7	7	FCN2_Endo
328	0	1.895384788	0.995	0.397	0	TIMP1	7	FCN2_Endo
329	0	1.893284087	0.953	0.075	0	SDPR	7	FCN2_Endo
330	0	1.89153873	0.988	0.243	0	IL6ST	7	FCN2_Endo
331	0	1.876400543	0.92	0.055	0	CLDN5	7	FCN2_Endo
332	0	1.831851369	0.952	0.136	0	MRC1	7	FCN2_Endo
333	0	1.800812112	0.939	0.054	0	LIFR	7	FCN2_Endo
334	0	1.762709761	0.969	0.086	0	GNG11	7	FCN2_Endo
335	0	1.752512278	0.986	0.085	0	TIMP3	7	FCN2_Endo
336	0	1.747001789	0.892	0.076	0	LYVE1	7	FCN2_Endo
337	0	1.742496691	0.924	0.065	0	F8	7	FCN2_Endo
338	0	1.741856264	0.962	0.247	0	PRCP	7	FCN2_Endo
339	0	1.72176379	0.923	0.159	0	CD4	7	FCN2_Endo
340	0	1.690468757	0.849	0.022	0	STAB2	7	FCN2_Endo
341	0	1.686665962	0.911	0.151	0	STAB1	7	FCN2_Endo
342	0	1.668935882	0.94	0.056	0	PPFIBP1	7	FCN2_Endo
343	0	1.643791988	0.947	0.066	0	LDB2	7	FCN2_Endo
344	0	1.628132897	0.911	0.062	0	CLEC3B	7	FCN2_Endo
345	0	1.623678357	0.939	0.063	0	TINAGL1	7	FCN2_Endo
346	0	1.623288826	0.722	0.037	0	IGFBP3	7	FCN2_Endo
347	0	1.604488649	0.922	0.042	0	ADGRF5	7	FCN2_Endo
348	0	1.588999863	0.807	0.029	0	TFPI2	7	FCN2_Endo
349	0	1.583164332	0.929	0.052	0	MYCT1	7	FCN2_Endo
350	0	1.578201728	0.905	0.125	0	CD9	7	FCN2_Endo
351	0	1.451825698	0.88	0.139	0	FCN1	8	FCN1_MP
352	0	1.423137464	0.99	0.24	0	AIF1	8	FCN1_MP
353	0	1.340870647	0.973	0.476	0	COTL1	8	FCN1_MP
354	0	1.319388149	0.994	0.302	0	FCER1G	8	FCN1_MP
355	1.81E-304	1.288134373	1	0.974	3.94E-300	FTH1	8	FCN1_MP
356	0	1.286426604	0.927	0.24	0	LST1	8	FCN1_MP
357	0	1.274984132	0.872	0.181	0	C5AR1	8	FCN1_MP
358	3.21E-297	1.209086312	0.992	0.594	7.00E-293	S100A11	8	FCN1_MP
359	4.51E-182	1.203017391	0.62	0.182	9.84E-178	THBS1	8	FCN1_MP
360	0	1.188826603	0.986	0.26	0	LYZ	8	FCN1_MP
361	0	1.187362903	0.88	0.216	0	SPI1	8	FCN1_MP
362	1.51E-306	1.167685938	0.999	0.403	3.29E-302	CST3	8	FCN1_MP
363	0	1.164571085	0.917	0.244	0	C1orf162	8	FCN1_MP

364	1.38E-288	1.153949928	0.969	0.404	3.01E-284	CTSS	8	FCN1_MP
365	0	1.13535119	0.741	0.15	0	LINC01272	8	FCN1_MP
366	4.27E-292	1.116006082	0.994	0.457	9.32E-288	TYROBP	8	FCN1_MP
367	3.50E-242	1.088416544	0.999	0.792	7.64E-238	SAT1	8	FCN1_MP
368	0	1.079716948	0.831	0.194	0	IFI30	8	FCN1_MP
369	0	1.055574898	0.873	0.23	0	MAFB	8	FCN1_MP
370	0	1.047262331	0.746	0.142	0	LILRB2	8	FCN1_MP
371	3.12E-234	1.041729517	0.972	0.591	6.81E-230	PSAP	8	FCN1_MP
372	5.73E-237	1.020190822	0.992	0.456	1.25E-232	HLA-DRA	8	FCN1_MP
373	2.41E-294	1.007904142	0.542	0.09	5.26E-290	IL1B	8	FCN1_MP
374	4.52E-226	0.999171997	0.989	0.734	9.86E-222	S100A6	8	FCN1_MP
375	0	0.984181171	0.839	0.208	0	PLAUR	8	FCN1_MP
376	0	0.962968721	0.72	0.117	0	VCAN	8	FCN1_MP
377	3.44E-231	0.953919637	1	0.979	7.50E-227	FTL	8	FCN1_MP
378	0	0.950852031	0.913	0.208	0	CD163	8	FCN1_MP
379	1.68E-235	0.941927431	0.846	0.283	3.67E-231	BCL2A1	8	FCN1_MP
380	1.00E-185	0.937230471	0.887	0.395	2.18E-181	SOD2	8	FCN1_MP
381	1.30E-222	0.931309328	0.996	0.863	2.84E-218	SH3BGRL3	8	FCN1_MP
382	4.27E-167	0.928056964	0.885	0.423	9.31E-163	NAMPT	8	FCN1_MP
383	0	0.92363802	0.754	0.155	0	SLC11A1	8	FCN1_MP
384	8.30E-166	0.921402852	0.937	0.542	1.81E-161	HLA-DPA1	8	FCN1_MP
385	3.34E-302	0.90073252	0.782	0.177	7.29E-298	CFD	8	FCN1_MP
386	8.26E-230	0.89839737	0.813	0.272	1.80E-225	LGALS3	8	FCN1_MP
387	2.92E-256	0.883523874	0.748	0.186	6.36E-252	HMOX1	8	FCN1_MP
388	1.90E-143	0.882060579	0.92	0.512	4.14E-139	HLA-DPB1	8	FCN1_MP
389	0	0.877417256	0.944	0.262	0	CD68	8	FCN1_MP
390	0	0.876017308	0.659	0.111	0	CLEC4E	8	FCN1_MP
391	0	0.871333266	0.714	0.14	0	CLEC7A	8	FCN1_MP
392	3.18E-288	0.865902426	0.638	0.13	6.93E-284	LILRA5	8	FCN1_MP
393	0	0.852245849	0.831	0.159	0	CSTA	8	FCN1_MP
394	4.44E-210	0.84965379	0.93	0.47	9.68E-206	TSPO	8	FCN1_MP
395	5.53E-171	0.849596101	0.986	0.66	1.21E-166	S100A4	8	FCN1_MP
396	1.62E-283	0.847555303	0.792	0.198	3.54E-279	CYBB	8	FCN1_MP
397	1.55E-167	0.842924093	0.979	0.682	3.37E-163	HLA-DRB1	8	FCN1_MP
398	0	0.840462003	0.472	0.045	0	CD300E	8	FCN1_MP
399	0	0.836605274	0.663	0.132	0	HCK	8	FCN1_MP
400	2.37E-169	0.83603938	0.914	0.503	5.17E-165	HLA-DRB5	8	FCN1_MP
401	0	2.057529375	0.552	0.084	0	XCL1	9	XCL1_NK cell
402	0	1.860341593	0.626	0.114	0	XCL2	9	XCL1_NK cell
403	7.52E-194	1.483898479	0.767	0.318	1.64E-189	CMC1	9	XCL1_NK cell
404	7.12E-263	1.331357407	0.887	0.31	1.55E-258	CD7	9	XCL1_NK cell
405	0	1.309374266	0.939	0.226	0	KLRD1	9	XCL1_NK cell
406	1.31E-203	1.245193474	0.547	0.131	2.85E-199	IL2RB	9	XCL1_NK cell
407	1.14E-271	1.178554389	0.668	0.143	2.48E-267	KLRF1	9	XCL1_NK cell
408	5.16E-106	1.174387389	0.7	0.32	1.13E-101	CCL3	9	XCL1_NK cell
409	9.28E-135	1.138908169	0.58	0.185	2.02E-130	IFNG	9	XCL1_NK cell
410	1.64E-200	1.087921563	0.931	0.398	3.57E-196	CCL5	9	XCL1_NK cell
411	1.04E-155	1.053426831	0.956	0.516	2.27E-151	CCL4	9	XCL1_NK cell
412	5.09E-186	1.040399107	0.767	0.251	1.11E-181	KLRB1	9	XCL1_NK cell
413	2.08E-220	1.016539272	0.986	0.43	4.54E-216	NKG7	9	XCL1_NK cell
414	4.81E-192	1.005187543	0.984	0.564	1.05E-187	IFITM1	9	XCL1_NK cell
415	1.67E-142	0.997497028	0.316	0.057	3.63E-138	KLRC1	9	XCL1_NK cell
416	5.12E-205	0.984770468	0.398	0.065	1.12E-200	TRDC	9	XCL1_NK cell
417	3.76E-169	0.948937273	0.478	0.108	8.20E-165	CLIC3	9	XCL1_NK cell
418	2.97E-92	0.942418374	0.643	0.345	6.48E-88	PIK3R1	9	XCL1_NK cell
419	8.29E-166	0.939105283	0.325	0.053	1.81E-161	TRGV9	9	XCL1_NK cell
420	2.81E-196	0.927050953	0.769	0.244	6.13E-192	CD247	9	XCL1_NK cell
421	2.60E-185	0.92236437	0.131	0.006	5.67E-181	TRDV2	9	XCL1_NK cell
422	4.32E-53	0.904923268	0.608	0.35	9.42E-49	AREG	9	XCL1_NK cell
423	4.60E-160	0.879167966	0.376	0.073	1.00E-155	CD160	9	XCL1_NK cell
424	2.70E-175	0.875305431	0.898	0.342	5.89E-171	GZMA	9	XCL1_NK cell
425	9.03E-131	0.85741224	0.432	0.109	1.97E-126	KLRC3	9	XCL1_NK cell
426	1.17E-166	0.854530843	0.682	0.204	2.55E-162	TRBC1	9	XCL1_NK cell
427	7.01E-127	0.8520594	0.904	0.535	1.53E-122	DUSP2	9	XCL1_NK cell
428	1.25E-148	0.846277401	0.948	0.461	2.72E-144	CD69	9	XCL1_NK cell
429	3.53E-167	0.820095883	0.796	0.276	7.69E-163	CTSW	9	XCL1_NK cell
430	6.31E-78	0.814140319	0.725	0.38	1.38E-73	CCL4L2	9	XCL1_NK cell
431	4.88E-146	0.790004352	0.414	0.094	1.06E-141	TXK	9	XCL1_NK cell
432	1.67E-113	0.784769391	0.61	0.23	3.63E-109	SYTL3	9	XCL1_NK cell
433	1.00E-128	0.783766408	0.619	0.217	2.18E-124	GZMM	9	XCL1_NK cell
434	5.67E-163	0.76501494	0.929	0.38	1.24E-158	CST7	9	XCL1_NK cell
435	6.98E-116	0.763897414	0.459	0.137	1.52E-111	MATK	9	XCL1_NK cell
436	3.11E-163	0.761056989	0.825	0.291	6.79E-159	PRF1	9	XCL1_NK cell

437	1.91E-119	0.750086573	0.822	0.354	4.17E-115	CD3E	9	XCL1_NK cell
438	5.90E-104	0.738987444	0.668	0.295	1.29E-99	FYN	9	XCL1_NK cell
439	1.62E-84	0.737850496	0.649	0.343	3.53E-80	STK17A	9	XCL1_NK cell
440	9.79E-106	0.73549946	0.53	0.185	2.13E-101	CD96	9	XCL1_NK cell
441	1.37E-124	0.734893405	0.926	0.601	2.99E-120	HCST	9	XCL1_NK cell
442	2.50E-84	0.729882427	0.588	0.288	5.45E-80	APMAP	9	XCL1_NK cell
443	1.28E-99	0.716446036	0.55	0.187	2.79E-95	GZMK	9	XCL1_NK cell
444	2.13E-93	0.713335814	0.498	0.178	4.65E-89	PYHIN1	9	XCL1_NK cell
445	3.97E-56	0.693261536	0.769	0.575	8.65E-52	NR4A2	9	XCL1_NK cell
446	1.05E-68	0.688809896	0.489	0.217	2.29E-64	DENND2D	9	XCL1_NK cell
447	3.88E-111	0.676794843	0.888	0.474	8.45E-107	PTPRCAP	9	XCL1_NK cell
448	1.15E-106	0.676021024	0.976	0.819	2.50E-102	ZFP36L2	9	XCL1_NK cell
449	7.99E-61	0.658675486	0.561	0.285	1.74E-56	PTGER4	9	XCL1_NK cell
450	1.75E-86	0.654226005	0.564	0.226	3.82E-82	HOPX	9	XCL1_NK cell
451	0	1.674727273	0.829	0.166	0	IL7R	10	IL7R_T cell
452	1.41E-105	0.967532843	0.537	0.189	3.07E-101	LTB	10	IL7R_T cell
453	3.05E-144	0.79577016	0.656	0.202	6.65E-140	CD3D	10	IL7R_T cell
454	0	0.787011652	0.227	0.01	0	LEF1	10	IL7R_T cell
455	3.73E-63	0.753048198	0.68	0.504	8.13E-59	LDHB	10	IL7R_T cell
456	2.34E-48	0.746099373	0.436	0.215	5.11E-44	RGCC	10	IL7R_T cell
457	5.46E-141	0.719804454	0.362	0.073	1.19E-136	CAMK4	10	IL7R_T cell
458	3.81E-96	0.713403647	0.907	0.804	8.32E-92	SARAF	10	IL7R_T cell
459	2.45E-124	0.703300992	0.269	0.046	5.34E-120	CD40LG	10	IL7R_T cell
460	6.51E-105	0.690566095	0.341	0.082	1.42E-100	INPP4B	10	IL7R_T cell
461	4.63E-86	0.689627847	0.463	0.162	1.01E-81	SPOCK2	10	IL7R_T cell
462	2.19E-79	0.689282905	0.622	0.271	4.78E-75	CD2	10	IL7R_T cell
463	3.42E-74	0.679571886	0.94	0.821	7.46E-70	ZFP36L2	10	IL7R_T cell
464	4.38E-114	0.678535609	0.843	0.354	9.54E-110	CD3E	10	IL7R_T cell
465	1.92E-90	0.663348241	0.387	0.114	4.18E-86	ITK	10	IL7R_T cell
466	3.45E-114	0.647259657	0.318	0.067	7.52E-110	TRAT1	10	IL7R_T cell
467	4.56E-84	0.646934186	0.834	0.414	9.95E-80	IL32	10	IL7R_T cell
468	7.55E-45	0.629456698	0.584	0.391	1.65E-40	LEPROTL1	10	IL7R_T cell
469	1.27E-127	0.629073426	0.232	0.034	2.78E-123	ICOS	10	IL7R_T cell
470	2.30E-164	0.627467246	0.997	0.991	5.01E-160	RPS12	10	IL7R_T cell
471	5.43E-25	0.604714847	0.354	0.203	1.18E-20	BATF	10	IL7R_T cell
472	1.30E-79	0.594348515	0.354	0.106	2.83E-75	CD6	10	IL7R_T cell
473	4.45E-52	0.591357212	0.755	0.517	9.70E-48	CD52	10	IL7R_T cell
474	1.26E-51	0.591100112	0.842	0.668	2.76E-47	TXNIP	10	IL7R_T cell
475	1.46E-68	0.590651954	0.423	0.152	3.17E-64	CD3G	10	IL7R_T cell
476	2.90E-100	0.586596973	0.959	0.915	6.31E-96	RPS29	10	IL7R_T cell
477	4.71E-33	0.584977327	0.352	0.179	1.03E-28	AIM1	10	IL7R_T cell
478	2.04E-55	0.576241796	0.431	0.179	4.44E-51	TC2N	10	IL7R_T cell
479	2.25E-99	0.575489251	0.954	0.905	4.91E-95	RPSA	10	IL7R_T cell
480	1.65E-122	0.571712645	0.987	0.987	3.59E-118	RPS27	10	IL7R_T cell
481	1.36E-32	0.559849592	0.408	0.229	2.96E-28	ARHGAP15	10	IL7R_T cell
482	8.14E-101	0.558827911	0.194	0.03	1.77E-96	TCF7	10	IL7R_T cell
483	1.37E-87	0.554614494	0.886	0.465	2.99E-83	CD69	10	IL7R_T cell
484	7.23E-140	0.546237212	0.98	0.977	1.58E-135	RPS3	10	IL7R_T cell
485	6.01E-153	0.543519218	0.992	0.988	1.31E-148	RPL30	10	IL7R_T cell
486	9.23E-40	0.54128053	0.486	0.26	2.01E-35	PARP8	10	IL7R_T cell
487	3.64E-93	0.5292825	0.946	0.916	7.95E-89	RPL10A	10	IL7R_T cell
488	2.37E-143	0.523529902	0.989	0.989	5.16E-139	RPS27A	10	IL7R_T cell
489	3.10E-132	0.518653196	0.985	0.988	6.76E-128	RPL34	10	IL7R_T cell
490	1.97E-128	0.518512816	0.977	0.974	4.30E-124	RPS21	10	IL7R_T cell
491	2.94E-73	0.515064753	0.917	0.856	6.40E-69	EEF1B2	10	IL7R_T cell
492	3.26E-90	0.515058489	0.971	0.952	7.11E-86	RPLP0	10	IL7R_T cell
493	2.16E-62	0.507805256	0.144	0.025	4.72E-58	CCR7	10	IL7R_T cell
494	1.43E-131	0.505888267	0.998	0.99	3.11E-127	RPL13	10	IL7R_T cell
495	3.05E-109	0.502757747	0.977	0.969	6.65E-105	RPS25	10	IL7R_T cell
496	6.96E-105	0.492830034	0.979	0.972	1.52E-100	RPS18	10	IL7R_T cell
497	9.11E-121	0.491961563	0.98	0.988	1.99E-116	RPL32	10	IL7R_T cell
498	3.38E-15	0.488896855	0.364	0.264	7.36E-11	UGP2	10	IL7R_T cell
499	9.09E-37	0.487987801	0.356	0.16	1.98E-32	SCML4	10	IL7R_T cell
500	3.88E-29	0.486489608	0.284	0.137	8.45E-25	PBXIP1	10	IL7R_T cell
501	0	3.223552399	0.881	0.027	0	MGP	11	MGP_Endo
502	0	2.648376377	0.968	0.109	0	TM4SF1	11	MGP_Endo
503	0	2.392162271	0.888	0.095	0	PLPP1	11	MGP_Endo
504	0	2.35970956	0.779	0.022	0	SPARCL1	11	MGP_Endo
505	0	2.281764846	0.946	0.193	0	ID1	11	MGP_Endo
506	0	2.241425068	0.896	0.105	0	TIMP3	11	MGP_Endo
507	0	2.104217254	0.667	0.061	0	SLC9A3R2	11	MGP_Endo
508	0	2.097899352	0.921	0.139	0	CD9	11	MGP_Endo
509	0	2.08018493	0.833	0.059	0	RAMP2	11	MGP_Endo

510	0	2.066620314	0.674	0.028	0	PLVAP	11	MGP_Endo
511	0	2.029107539	0.779	0.18	0	GSN	11	MGP_Endo
512	0	2.009050557	0.848	0.096	0	IFI27	11	MGP_Endo
513	0	2.006386122	0.921	0.101	0	CALCRL	11	MGP_Endo
514	0	1.996965286	0.888	0.078	0	CLEC3B	11	MGP_Endo
515	0	1.99004288	0.873	0.081	0	CRIP2	11	MGP_Endo
516	0	1.987829711	0.948	0.145	0	RNASE1	11	MGP_Endo
517	0	1.952273007	0.896	0.084	0	HSPG2	11	MGP_Endo
518	0	1.949816584	0.729	0.074	0	EMP1	11	MGP_Endo
519	0	1.945205884	0.824	0.058	0	CLEC14A	11	MGP_Endo
520	0	1.905399004	0.846	0.103	0	EPAS1	11	MGP_Endo
521	0	1.885649238	0.86	0.237	0	ID3	11	MGP_Endo
522	0	1.870955167	0.637	0.02	0	VWF	11	MGP_Endo
523	0	1.870583049	0.806	0.084	0	CAV1	11	MGP_Endo
524	0	1.865537242	0.672	0.018	0	AQP1	11	MGP_Endo
525	0	1.862793001	0.774	0.034	0	ADIRF	11	MGP_Endo
526	0	1.856026052	0.724	0.066	0	ADAMTS1	11	MGP_Endo
527	0	1.855318563	0.992	0.454	0	IFITM3	11	MGP_Endo
528	0	1.844517195	0.676	0.047	0	GJA4	11	MGP_Endo
529	0	1.837998581	0.819	0.121	0	A2M	11	MGP_Endo
530	0	1.830952783	0.873	0.233	0	PECAM1	11	MGP_Endo
531	0	1.762823779	0.86	0.095	0	EGFL7	11	MGP_Endo
532	0	1.728484704	0.594	0.072	0	C7	11	MGP_Endo
533	0	1.711892764	0.783	0.052	0	EMCN	11	MGP_Endo
534	0	1.708495151	0.88	0.212	0	IGFBP7	11	MGP_Endo
535	0	1.692198354	0.697	0.081	0	CLDN5	11	MGP_Endo
536	0	1.675811661	0.759	0.096	0	IL33	11	MGP_Endo
537	0	1.670164793	0.92	0.104	0	GNG11	11	MGP_Endo
538	0	1.58668753	0.773	0.089	0	LDB2	11	MGP_Endo
539	5.32E-266	1.583882387	0.948	0.401	1.16E-261	HSPB1	11	MGP_Endo
540	0	1.57394587	0.836	0.12	0	IGFBP4	11	MGP_Endo
541	0	1.573028963	0.766	0.089	0	TGM2	11	MGP_Endo
542	3.34E-147	1.558466132	0.903	0.412	7.29E-143	TIMP1	11	MGP_Endo
543	0	1.553802374	0.724	0.097	0	SPARC	11	MGP_Endo
544	0	1.547536643	0.589	0.063	0	SRPX	11	MGP_Endo
545	0	1.517064541	0.721	0.091	0	PLPP3	11	MGP_Endo
546	0	1.516979079	0.696	0.089	0	HYAL2	11	MGP_Endo
547	0	1.498878009	0.212	0.006	0	IGF2	11	MGP_Endo
548	0	1.491469279	0.809	0.085	0	TINAGL1	11	MGP_Endo
549	0	1.483953832	0.431	0.033	0	ANGPTL4	11	MGP_Endo
550	2.57E-134	1.480513213	0.607	0.212	5.59E-130	CXCL2	11	MGP_Endo
551	7.76E-74	4.556588525	0.452	0.151	1.69E-69	IGKV1D-16	12	IGKV1D-16_Plasma cell
552	8.76E-94	4.043320756	0.454	0.131	1.91E-89	IGKV1-5	12	IGKV1D-16_Plasma cell
553	1.28E-127	3.935253471	0.523	0.16	2.80E-123	IGHM	12	IGKV1D-16_Plasma cell
554	1.61E-112	3.891665782	0.512	0.155	3.51E-108	IGHV3-23	12	IGKV1D-16_Plasma cell
555	3.54E-29	3.878962159	0.213	0.076	7.72E-25	IGKV3-15	12	IGKV1D-16_Plasma cell
556	2.47E-28	3.869106566	0.371	0.17	5.39E-24	IGKV3-20	12	IGKV1D-16_Plasma cell
557	3.56E-86	3.843157856	0.429	0.128	7.76E-82	IGHV3-74	12	IGKV1D-16_Plasma cell
558	1.42E-52	3.822010271	0.142	0.027	3.10E-48	IGHV3-48	12	IGKV1D-16_Plasma cell
559	1.82E-15	3.800393283	0.33	0.177	3.97E-11	IGLV2-14	12	IGKV1D-16_Plasma cell
560	1.94E-234	3.790448057	0.491	0.078	4.24E-230	IGKV1-12	12	IGKV1D-16_Plasma cell
561	4.14E-77	3.788228382	0.289	0.069	9.04E-73	IGHV3-7	12	IGKV1D-16_Plasma cell
562	0	3.773839301	0.621	0.084	0	IGHG1	12	IGKV1D-16_Plasma cell
563	0	3.764719512	0.961	0.183	0	JCHAIN	12	IGKV1D-16_Plasma cell
564	3.09E-39	3.69651482	0.227	0.071	6.74E-35	IGKV3-11	12	IGKV1D-16_Plasma cell
565	2.48E-112	3.682874613	0.287	0.052	5.41E-108	IGLV6-57	12	IGKV1D-16_Plasma cell
566	1.32E-27	3.671998209	0.257	0.101	2.88E-23	IGLV2-23	12	IGKV1D-16_Plasma cell
567	2.07E-81	3.656746333	0.199	0.035	4.51E-77	IGHV3-30	12	IGKV1D-16_Plasma cell
568	0	3.633754552	0.801	0.176	0	IGKC	12	IGKV1D-16_Plasma cell
569	3.57E-37	3.62738133	0.191	0.057	7.78E-33	IGLV2-8	12	IGKV1D-16_Plasma cell
570	7.65E-40	3.573884625	0.145	0.035	1.67E-35	IGKV2D-28	12	IGKV1D-16_Plasma cell
571	7.32E-48	3.539089521	0.14	0.029	1.60E-43	IGHV4-39	12	IGKV1D-16_Plasma cell
572	1.07E-22	3.489586891	0.2	0.079	2.34E-18	IGLV1-47	12	IGKV1D-16_Plasma cell
573	2.08E-39	3.479992909	0.328	0.13	4.53E-35	IGKV2-30	12	IGKV1D-16_Plasma cell
574	6.58E-85	3.457416719	0.22	0.041	1.44E-80	IGKV1-16	12	IGKV1D-16_Plasma cell
575	2.31E-85	3.419925445	0.188	0.03	5.03E-81	IGKV1-27	12	IGKV1D-16_Plasma cell
576	4.63E-72	3.392622483	0.131	0.018	1.01E-67	IGHV4-59	12	IGKV1D-16_Plasma cell
577	2.41E-82	3.383921281	0.594	0.23	5.26E-78	IGKV4-1	12	IGKV1D-16_Plasma cell
578	2.78E-29	3.313596923	0.142	0.04	6.05E-25	IGLV1-44	12	IGKV1D-16_Plasma cell
579	1.27E-82	3.279923017	0.119	0.012	2.77E-78	IGHV3-72	12	IGKV1D-16_Plasma cell
580	2.26E-76	3.275216705	0.135	0.018	4.92E-72	IGHV4-61	12	IGKV1D-16_Plasma cell
581	4.44E-213	3.260320843	0.44	0.074	9.68E-209	IGHG2	12	IGKV1D-16_Plasma cell
582	4.78E-35	3.250301165	0.284	0.109	1.04E-30	IGKV2-24	12	IGKV1D-16_Plasma cell

583	0	3.222113991	0.365	0.019	0	IGLV3-1	12	IGKV1D-16_Plasma cell
584	1.14E-21	3.205531959	0.232	0.096	2.48E-17	IGLV2-11	12	IGKV1D-16_Plasma cell
585	3.90E-59	3.166370678	0.19	0.041	8.51E-55	IGHV4-34	12	IGKV1D-16_Plasma cell
586	1.41E-87	3.15384116	0.137	0.016	3.08E-83	IGKV3D-20	12	IGKV1D-16_Plasma cell
587	6.01E-08	3.007969236	0.103	0.049	0.001311617	IGHV1-18	12	IGKV1D-16_Plasma cell
588	4.34E-38	2.98633146	0.112	0.023	9.46E-34	IGHV3-15	12	IGKV1D-16_Plasma cell
589	0	2.90356363	0.511	0.054	0	IGHA1	12	IGKV1D-16_Plasma cell
590	1.25E-16	2.901320498	0.115	0.04	2.73E-12	IGLV1-40	12	IGKV1D-16_Plasma cell
591	0	2.75715481	0.993	0.07	0	MZB1	12	IGKV1D-16_Plasma cell
592	5.32E-150	2.753215227	0.163	0.012	1.16E-145	IGKV1-6	12	IGKV1D-16_Plasma cell
593	9.49E-46	2.678168431	0.131	0.026	2.07E-41	IGKV2-29	12	IGKV1D-16_Plasma cell
594	1.88E-77	2.60206159	0.105	0.01	4.10E-73	IGKV1-17	12	IGKV1D-16_Plasma cell
595	8.28E-64	2.534888053	0.2	0.043	1.81E-59	IGHV6-1	12	IGKV1D-16_Plasma cell
596	5.21E-76	2.445448304	0.209	0.042	1.14E-71	IGLC2	12	IGKV1D-16_Plasma cell
597	3.19E-212	2.391137066	0.2	0.013	6.95E-208	IGHA2	12	IGKV1D-16_Plasma cell
598	5.67E-99	2.328734551	0.199	0.03	1.24E-94	IGLC3	12	IGKV1D-16_Plasma cell
599	4.07E-117	2.303935586	0.145	0.013	8.88E-113	IGKV2D-30	12	IGKV1D-16_Plasma cell
600	1.92E-54	2.301222521	0.122	0.02	4.18E-50	IGHV3-33	12	IGKV1D-16_Plasma cell
601	0	2.210328864	0.809	0.111	0	CXCL3	13	CXCL3_MP
602	0	1.987451787	0.811	0.088	0	IL1B	13	CXCL3_MP
603	0	1.933404869	0.996	0.132	0	C1QA	13	CXCL3_MP
604	0	1.930272281	0.988	0.127	0	C1QB	13	CXCL3_MP
605	0	1.91858288	0.988	0.125	0	C1QC	13	CXCL3_MP
606	0	1.785804721	0.912	0.204	0	CXCL2	13	CXCL3_MP
607	0	1.706999047	0.823	0.16	0	CXCL8	13	CXCL3_MP
608	2.73E-246	1.596902437	0.538	0.085	5.95E-242	G0S2	13	CXCL3_MP
609	0	1.54100153	0.99	0.271	0	CD68	13	CXCL3_MP
610	3.00E-247	1.527316201	0.994	0.399	6.54E-243	SOD2	13	CXCL3_MP
611	2.18E-257	1.5240794	0.996	0.506	4.75E-253	HLA-DRB5	13	CXCL3_MP
612	0	1.503518717	0.945	0.159	0	MS4A7	13	CXCL3_MP
613	0	1.458525799	0.978	0.217	0	CD163	13	CXCL3_MP
614	1.34E-204	1.453888691	0.898	0.317	2.93E-200	CCL3	13	CXCL3_MP
615	8.54E-237	1.445108396	1	0.464	1.86E-232	HLA-DRA	13	CXCL3_MP
616	1.27E-224	1.44153333	1	0.979	2.77E-220	FTL	13	CXCL3_MP
617	5.73E-237	1.434285571	1	0.795	1.25E-232	SAT1	13	CXCL3_MP
618	1.68E-252	1.432515606	1	0.413	3.66E-248	CST3	13	CXCL3_MP
619	4.47E-262	1.380383926	0.992	0.405	9.75E-258	CTSB	13	CXCL3_MP
620	0	1.368461678	0.481	0.05	0	IL1RN	13	CXCL3_MP
621	0	1.355524372	0.927	0.152	0	MS4A4A	13	CXCL3_MP
622	8.40E-225	1.350096468	0.986	0.516	1.83E-220	HLA-DPB1	13	CXCL3_MP
623	1.25E-303	1.349634192	0.951	0.265	2.72E-299	LIPA	13	CXCL3_MP
624	0	1.336114235	0.931	0.19	0	C5AR1	13	CXCL3_MP
625	2.67E-216	1.294662039	0.994	0.546	5.83E-212	HLA-DPA1	13	CXCL3_MP
626	2.37E-192	1.245326915	1	0.831	5.16E-188	CD74	13	CXCL3_MP
627	2.32E-298	1.242488402	0.959	0.237	5.06E-294	MAFB	13	CXCL3_MP
628	0	1.237219959	0.896	0.19	0	HMOX1	13	CXCL3_MP
629	0	1.235880056	0.845	0.11	0	VSIG4	13	CXCL3_MP
630	7.32E-209	1.221582889	0.992	0.596	1.60E-204	PSAP	13	CXCL3_MP
631	1.01E-197	1.209911723	0.998	0.686	2.21E-193	HLA-DRB1	13	CXCL3_MP
632	1.35E-256	1.20516247	0.982	0.301	2.94E-252	MS4A6A	13	CXCL3_MP
633	0	1.196546825	0.815	0.088	0	FOLR2	13	CXCL3_MP
634	1.61E-170	1.179813831	0.762	0.24	3.51E-166	CHMP1B	13	CXCL3_MP
635	1.08E-263	1.177721783	0.882	0.22	2.35E-259	FGL2	13	CXCL3_MP
636	5.16E-225	1.17706745	0.988	0.496	1.13E-220	NPC2	13	CXCL3_MP
637	0	1.165106823	0.862	0.117	0	CSF1R	13	CXCL3_MP
638	1.08E-205	1.16461073	0.839	0.257	2.35E-201	NINJ1	13	CXCL3_MP
639	1.62E-228	1.145726934	0.947	0.301	3.52E-224	HLA-DQA1	13	CXCL3_MP
640	0	1.13630161	0.78	0.112	0	ABCA1	13	CXCL3_MP
641	5.09E-269	1.135244472	0.242	0.013	1.11E-264	CXCL10	13	CXCL3_MP
642	0	1.127738115	0.821	0.155	0	CPM	13	CXCL3_MP
643	1.27E-206	1.126919456	0.978	0.428	2.76E-202	GLUL	13	CXCL3_MP
644	0	1.123439097	0.458	0.036	0	MMP19	13	CXCL3_MP
645	5.15E-277	1.109903469	0.955	0.263	1.12E-272	CD83	13	CXCL3_MP
646	5.70E-201	1.106731024	0.98	0.412	1.24E-196	CTSS	13	CXCL3_MP
647	4.41E-255	1.098728164	0.819	0.186	9.61E-251	CFD	13	CXCL3_MP
648	2.31E-169	1.096739419	0.804	0.259	5.03E-165	ICAM1	13	CXCL3_MP
649	8.73E-271	1.084756763	0.9	0.235	1.90E-266	CREG1	13	CXCL3_MP
650	1.75E-221	1.081668628	0.906	0.27	3.81E-217	KLF4	13	CXCL3_MP
651	0	2.822801639	0.976	0.282	0	STMN1	14	STMN1_NK/T cell
652	4.14E-229	2.183714334	0.841	0.284	9.02E-225	HIST1H4C	14	STMN1_NK/T cell
653	0	2.167310035	0.794	0.033	0	UBE2C	14	STMN1_NK/T cell
654	4.92E-300	2.0710895	0.955	0.376	1.07E-295	HMGB2	14	STMN1_NK/T cell
655	8.17E-269	2.046790654	0.974	0.51	1.78E-264	TUBB	14	STMN1_NK/T cell

656	7.66E-246	2.021849365	0.966	0.584	1.67E-241	TUBA1B	14	STMN1_NK/T cell
657	0	1.933512698	0.751	0.031	0	ASPM	14	STMN1_NK/T cell
658	0	1.912360033	0.805	0.042	0	MKI67	14	STMN1_NK/T cell
659	0	1.838662984	0.732	0.031	0	CENPF	14	STMN1_NK/T cell
660	1.42E-266	1.833362492	0.989	0.603	3.09E-262	HMGN2	14	STMN1_NK/T cell
661	0	1.771218043	0.73	0.034	0	TOP2A	14	STMN1_NK/T cell
662	1.02E-179	1.76464362	0.899	0.511	2.22E-175	ARL6IP1	14	STMN1_NK/T cell
663	0	1.733345497	0.775	0.049	0	NUSAP1	14	STMN1_NK/T cell
664	0	1.732758571	0.682	0.046	0	TYMS	14	STMN1_NK/T cell
665	0	1.561701211	0.8	0.123	0	SMC4	14	STMN1_NK/T cell
666	0	1.482055497	0.764	0.104	0	PTTG1	14	STMN1_NK/T cell
667	0	1.461698789	0.68	0.028	0	TPX2	14	STMN1_NK/T cell
668	0	1.453334302	0.646	0.046	0	RRM2	14	STMN1_NK/T cell
669	4.20E-202	1.385923354	0.974	0.661	9.15E-198	H2AFZ	14	STMN1_NK/T cell
670	1.23E-241	1.325806184	0.882	0.323	2.69E-237	H2AFV	14	STMN1_NK/T cell
671	0	1.322896922	0.715	0.081	0	CKS1B	14	STMN1_NK/T cell
672	0	1.304199325	0.659	0.037	0	BIRC5	14	STMN1_NK/T cell
673	0	1.303530801	0.532	0.032	0	CDC20	14	STMN1_NK/T cell
674	1.58E-207	1.277315064	0.985	0.809	3.45E-203	HMGB1	14	STMN1_NK/T cell
675	0	1.266732366	0.62	0.047	0	CDK1	14	STMN1_NK/T cell
676	0	1.201948004	0.663	0.051	0	MAD2L1	14	STMN1_NK/T cell
677	0	1.198331274	0.552	0.027	0	HMMR	14	STMN1_NK/T cell
678	0	1.191784062	0.648	0.07	0	KIF20B	14	STMN1_NK/T cell
679	1.79E-175	1.16570522	0.607	0.155	3.90E-171	KPNA2	14	STMN1_NK/T cell
680	0	1.146884982	0.588	0.041	0	CDKN3	14	STMN1_NK/T cell
681	2.94E-183	1.142238764	0.854	0.345	6.41E-179	NUCKS1	14	STMN1_NK/T cell
682	2.20E-173	1.140585424	0.841	0.346	4.79E-169	RAD21	14	STMN1_NK/T cell
683	0	1.133913628	0.536	0.032	0	CCNB2	14	STMN1_NK/T cell
684	0	1.111150526	0.571	0.022	0	CDCA8	14	STMN1_NK/T cell
685	0	1.108673863	0.56	0.031	0	CCNA2	14	STMN1_NK/T cell
686	0	1.105813152	0.541	0.027	0	DLGAP5	14	STMN1_NK/T cell
687	0	1.077220876	0.562	0.024	0	GTSE1	14	STMN1_NK/T cell
688	5.43E-149	1.074293814	0.73	0.261	1.18E-144	TUBA1C	14	STMN1_NK/T cell
689	4.50E-191	1.071845976	0.8	0.253	9.81E-187	NUCB2	14	STMN1_NK/T cell
690	0	1.070237081	0.539	0.026	0	CASC5	14	STMN1_NK/T cell
691	0	1.068729569	0.554	0.027	0	KIFC1	14	STMN1_NK/T cell
692	1.31E-167	1.059676413	0.798	0.289	2.85E-163	CKS2	14	STMN1_NK/T cell
693	2.84E-205	1.056117462	0.727	0.191	6.19E-201	TMPO	14	STMN1_NK/T cell
694	4.22E-152	1.055872401	0.899	0.493	9.21E-148	ANP32B	14	STMN1_NK/T cell
695	0	1.049721454	0.442	0.03	0	CCNB1	14	STMN1_NK/T cell
696	0	1.049131623	0.521	0.025	0	TROAP	14	STMN1_NK/T cell
697	0	1.024677518	0.56	0.04	0	PRC1	14	STMN1_NK/T cell
698	0	1.024056897	0.545	0.028	0	NUF2	14	STMN1_NK/T cell
699	5.01E-143	1.022333311	0.83	0.393	1.09E-138	CALM3	14	STMN1_NK/T cell
700	0	1.664602941	0.348	0.011	0	TRBV9	15	TRBV9_T cell
701	3.82E-250	1.495128638	0.873	0.214	8.33E-246	GZMH	15	TRBV9_T cell
702	6.75E-249	1.405782781	0.799	0.166	1.47E-244	CD8A	15	TRBV9_T cell
703	0	1.346247598	0.311	0.006	0	TRAV38-2DV8	15	TRBV9_T cell
704	3.19E-189	1.253153378	0.779	0.205	6.95E-185	CD3D	15	TRBV9_T cell
705	2.21E-222	1.214336927	0.145	0.004	4.81E-218	TRBV13	15	TRBV9_T cell
706	5.11E-162	1.209028068	0.985	0.439	1.11E-157	NKG7	15	TRBV9_T cell
707	4.21E-183	1.143448472	0.779	0.185	9.18E-179	GZMB	15	TRBV9_T cell
708	6.05E-184	1.130413622	0.512	0.086	1.32E-179	CD8B	15	TRBV9_T cell
709	2.35E-156	1.110330797	0.988	0.405	5.12E-152	CCL5	15	TRBV9_T cell
710	7.47E-160	1.09953814	0.627	0.15	1.63E-155	CD3G	15	TRBV9_T cell
711	1.70E-180	1.018317362	0.243	0.019	3.72E-176	RP11-291B21.2	15	TRBV9_T cell
712	2.55E-139	1.017960733	0.917	0.359	5.56E-135	CD3E	15	TRBV9_T cell
713	3.51E-135	0.999342974	0.958	0.417	7.65E-131	IL32	15	TRBV9_T cell
714	4.83E-151	0.970610531	0.833	0.241	1.05E-146	KLRD1	15	TRBV9_T cell
715	2.41E-171	0.938695009	0.287	0.029	5.25E-167	TRGV5	15	TRBV9_T cell
716	3.00E-126	0.922089592	0.939	0.389	6.55E-122	CST7	15	TRBV9_T cell
717	6.97E-108	0.901670253	0.542	0.137	1.52E-103	FGFBP2	15	TRBV9_T cell
718	7.71E-113	0.877156755	0.814	0.3	1.68E-108	PRF1	15	TRBV9_T cell
719	1.18E-113	0.872817941	0.792	0.285	2.57E-109	CTSW	15	TRBV9_T cell
720	1.46E-99	0.820333878	0.963	0.571	3.18E-95	IFITM1	15	TRBV9_T cell
721	3.01E-71	0.796830277	0.637	0.265	6.57E-67	TRBC2	15	TRBV9_T cell
722	6.43E-89	0.791568098	0.713	0.274	1.40E-84	CD2	15	TRBV9_T cell
723	2.99E-88	0.787430687	0.841	0.353	6.52E-84	GZMA	15	TRBV9_T cell
724	6.97E-67	0.782606812	0.586	0.237	1.52E-62	SYTL3	15	TRBV9_T cell
725	1.86E-104	0.774555496	0.287	0.045	4.06E-100	TRGV2	15	TRBV9_T cell
726	5.45E-46	0.749434638	0.752	0.52	1.19E-41	CD52	15	TRBV9_T cell
727	2.36E-62	0.733842272	0.591	0.247	5.14E-58	LCK	15	TRBV9_T cell
728	1.25E-72	0.700558745	0.414	0.115	2.73E-68	KLRC3	15	TRBV9_T cell

729	6.70E-62	0.70051487	0.561	0.225	1.46E-57	GZMM	15	TRBV9_T cell
730	9.47E-56	0.698721332	0.186	0.035	2.07E-51	TRGV3	15	TRBV9_T cell
731	2.84E-68	0.695030243	0.373	0.104	6.19E-64	LAG3	15	TRBV9_T cell
732	4.89E-74	0.682433511	0.882	0.481	1.07E-69	PTPRCAP	15	TRBV9_T cell
733	8.19E-52	0.682316785	0.654	0.349	1.79E-47	RARRES3	15	TRBV9_T cell
734	3.76E-67	0.663902549	0.907	0.607	8.19E-63	HCST	15	TRBV9_T cell
735	1.54E-55	0.646307533	0.743	0.406	3.35E-51	IL2RG	15	TRBV9_T cell
736	1.42E-47	0.64141087	0.35	0.119	3.10E-43	ITK	15	TRBV9_T cell
737	5.93E-41	0.635840121	0.635	0.372	1.29E-36	EVL	15	TRBV9_T cell
738	6.77E-38	0.626800332	0.586	0.349	1.48E-33	C9orf142	15	TRBV9_T cell
739	2.40E-58	0.615358948	0.718	0.324	5.22E-54	CD7	15	TRBV9_T cell
740	5.47E-46	0.612995839	0.385	0.141	1.19E-41	SAMD3	15	TRBV9_T cell
741	8.68E-32	0.612084744	0.569	0.331	1.89E-27	CMC1	15	TRBV9_T cell
742	4.07E-36	0.60981538	0.566	0.304	8.87E-32	FYN	15	TRBV9_T cell
743	4.12E-31	0.603988564	0.38	0.179	8.99E-27	LIME1	15	TRBV9_T cell
744	1.92E-37	0.600613693	0.569	0.322	4.18E-33	SLC9A3R1	15	TRBV9_T cell
745	3.76E-35	0.597288634	0.365	0.152	8.20E-31	ADGRG1	15	TRBV9_T cell
746	3.17E-37	0.589067056	0.431	0.194	6.92E-33	C12orf75	15	TRBV9_T cell
747	5.29E-44	0.579316872	0.326	0.11	1.15E-39	CD6	15	TRBV9_T cell
748	2.17E-27	0.577351936	0.417	0.223	4.73E-23	LYAR	15	TRBV9_T cell
749	4.09E-24	0.572373648	0.387	0.204	8.91E-20	BATF	15	TRBV9_T cell
750	0	2.520826902	0.983	0.124	0	CCL14	16	CCL14_Endo
751	0	2.414809692	0.97	0.099	0	FCN3	16	CCL14_Endo
752	0	2.390843269	0.916	0.078	0	CLEC4G	16	CCL14_Endo
753	0	2.357609654	0.978	0.104	0	DNASE1L3	16	CCL14_Endo
754	0	2.316933447	0.928	0.079	0	FCN2	16	CCL14_Endo
755	0	2.09211243	0.78	0.062	0	CLEC4M	16	CCL14_Endo
756	1.63E-297	1.999356321	0.914	0.22	3.56E-293	SEPP1	16	CCL14_Endo
757	4.55E-278	1.982300484	0.933	0.264	9.92E-274	CTSL	16	CCL14_Endo
758	2.74E-254	1.978650461	0.81	0.2	5.97E-250	ACP5	16	CCL14_Endo
759	0	1.949368265	0.738	0.067	0	CLEC1B	16	CCL14_Endo
760	2.59E-164	1.857991242	0.652	0.183	5.65E-160	STAB1	16	CCL14_Endo
761	5.28E-257	1.8293461	0.765	0.166	1.15E-252	FCGR2B	16	CCL14_Endo
762	0	1.824572828	0.733	0.102	0	RAMP3	16	CCL14_Endo
763	0	1.813332147	0.891	0.14	0	TFPI	16	CCL14_Endo
764	0	1.787960388	0.773	0.12	0	GNG11	16	CCL14_Endo
765	0	1.78465662	0.723	0.071	0	OIT3	16	CCL14_Endo
766	0	1.746650712	0.79	0.092	0	CRHBP	16	CCL14_Endo
767	8.48E-259	1.687266445	0.509	0.063	1.85E-254	CCL23	16	CCL14_Endo
768	0	1.646811842	0.763	0.105	0	IL33	16	CCL14_Endo
769	2.05E-209	1.601384492	0.975	0.417	4.47E-205	TIMP1	16	CCL14_Endo
770	2.99E-201	1.580752834	0.788	0.211	6.52E-197	LGMN	16	CCL14_Endo
771	6.10E-221	1.580575407	0.615	0.114	1.33E-216	CNN3	16	CCL14_Endo
772	5.46E-157	1.563699459	0.677	0.208	1.19E-152	MARCKSL1	16	CCL14_Endo
773	6.00E-238	1.552707854	0.588	0.092	1.31E-233	LIFR	16	CCL14_Endo
774	9.35E-250	1.534326693	0.551	0.081	2.04E-245	NUPR1	16	CCL14_Endo
775	1.66E-271	1.52903751	0.501	0.058	3.63E-267	STAB2	16	CCL14_Endo
776	5.61E-137	1.521091138	0.625	0.192	1.22E-132	CD4	16	CCL14_Endo
777	1.44E-226	1.512743314	0.756	0.169	3.13E-222	ENG	16	CCL14_Endo
778	4.94E-147	1.473178543	0.756	0.273	1.08E-142	IL6ST	16	CCL14_Endo
779	1.62E-189	1.472009235	0.551	0.106	3.54E-185	S100A13	16	CCL14_Endo
780	9.36E-208	1.44344527	0.533	0.09	2.04E-203	S100A16	16	CCL14_Endo
781	9.31E-216	1.393081482	0.622	0.113	2.03E-211	IFI27	16	CCL14_Endo
782	2.67E-227	1.380197926	0.647	0.112	5.82E-223	EGFL7	16	CCL14_Endo
783	3.17E-215	1.375884807	0.605	0.11	6.92E-211	NPDC1	16	CCL14_Endo
784	4.48E-179	1.355651447	0.563	0.111	9.76E-175	LYVE1	16	CCL14_Endo
785	2.76E-156	1.35181879	0.931	0.463	6.01E-152	IFITM3	16	CCL14_Endo
786	2.40E-127	1.340738878	0.738	0.276	5.22E-123	CD14	16	CCL14_Endo
787	1.96E-217	1.337149584	0.412	0.049	4.27E-213	RELN	16	CCL14_Endo
788	5.19E-101	1.33683514	0.637	0.258	1.13E-96	MAF	16	CCL14_Endo
789	4.05E-141	1.333501056	0.617	0.171	8.84E-137	MRC1	16	CCL14_Endo
790	2.36E-217	1.332932738	0.575	0.093	5.14E-213	CLDN5	16	CCL14_Endo
791	4.87E-224	1.330314415	0.701	0.134	1.06E-219	IGFBP4	16	CCL14_Endo
792	1.26E-214	1.327997675	0.625	0.113	2.74E-210	SDPR	16	CCL14_Endo
793	2.59E-166	1.324367426	0.469	0.083	5.66E-162	ADGRL4	16	CCL14_Endo
794	2.57E-206	1.283965146	0.593	0.101	5.61E-202	TSPAN7	16	CCL14_Endo
795	6.50E-162	1.276416886	0.716	0.2	1.42E-157	APP	16	CCL14_Endo
796	7.30E-188	1.263189154	0.437	0.065	1.59E-183	EHD3	16	CCL14_Endo
797	7.05E-75	1.247801723	0.738	0.415	1.54E-70	HSPB1	16	CCL14_Endo
798	6.19E-157	1.233725304	0.486	0.093	1.35E-152	MYCT1	16	CCL14_Endo
799	2.97E-117	1.232015645	0.627	0.206	6.49E-113	PDLIM1	16	CCL14_Endo
800	8.80E-64	3.07195586	0.636	0.235	1.92E-59	IGKV4-1	17	IGKV4-1_Plasma cell
801	2.73E-26	3.001846507	0.302	0.111	5.95E-22	IGKV2-24	17	IGKV4-1_Plasma cell

802	7.22E-42	2.900733859	0.148	0.025	1.57E-37	IGKV2-28	17	IGKV4-1_Plasma cell
803	1.38E-24	2.834990195	0.334	0.133	3.01E-20	IGKV2-30	17	IGKV4-1_Plasma cell
804	3.20E-11	2.817248443	0.183	0.082	6.97E-07	IGLV1-47	17	IGKV4-1_Plasma cell
805	1.20E-24	2.674851493	0.328	0.136	2.61E-20	IGHV3-74	17	IGKV4-1_Plasma cell
806	1.71E-126	2.669563644	0.325	0.043	3.74E-122	IGHV6-1	17	IGKV4-1_Plasma cell
807	3.97E-07	2.666389526	0.192	0.099	0.008663441	IGLV2-11	17	IGKV4-1_Plasma cell
808	2.62E-17	2.572780603	0.109	0.028	5.71E-13	IGKV2-29	17	IGKV4-1_Plasma cell
809	5.82E-172	2.539668465	0.5	0.078	1.27E-167	IGHG2	17	IGKV4-1_Plasma cell
810	2.78E-13	2.501169963	0.13	0.045	6.07E-09	IGHV4-34	17	IGKV4-1_Plasma cell
811	0	2.486238622	0.994	0.195	0	JCHAIN	17	IGKV4-1_Plasma cell
812	2.27E-12	2.485556082	0.343	0.174	4.95E-08	IGKV3-20	17	IGKV4-1_Plasma cell
813	1.76E-48	2.448805513	0.462	0.162	3.83E-44	IGHV3-23	17	IGKV4-1_Plasma cell
814	0	2.414751846	0.997	0.084	0	MZB1	17	IGKV4-1_Plasma cell
815	2.11E-22	2.40486664	0.109	0.024	4.61E-18	IGHV3-15	17	IGKV4-1_Plasma cell
816	2.06E-25	2.353644966	0.142	0.033	4.49E-21	IGKV1-27	17	IGKV4-1_Plasma cell
817	2.64E-23	2.345928615	0.142	0.037	5.76E-19	IGKV2D-28	17	IGKV4-1_Plasma cell
818	1.16E-16	2.344917082	0.112	0.031	2.53E-12	IGHV4-39	17	IGKV4-1_Plasma cell
819	3.97E-225	2.321073787	0.802	0.186	8.65E-221	IGKC	17	IGKV4-1_Plasma cell
820	1.69E-24	2.256112801	0.355	0.138	3.68E-20	IGKV1-5	17	IGKV4-1_Plasma cell
821	4.51E-11	2.11485667	0.186	0.079	9.84E-07	IGKV3-15	17	IGKV4-1_Plasma cell
822	1.32E-17	2.113826231	0.178	0.059	2.88E-13	IGLV2-8	17	IGKV4-1_Plasma cell
823	1.43E-43	2.082566233	0.204	0.045	3.12E-39	IGLC2	17	IGKV4-1_Plasma cell
824	1.42E-258	2.011802777	0.553	0.061	3.10E-254	IGHA1	17	IGKV4-1_Plasma cell
825	2.94E-14	2.01082674	0.192	0.075	6.42E-10	IGHV3-7	17	IGKV4-1_Plasma cell
826	2.22E-110	2.009159058	0.462	0.086	4.84E-106	IGKV1-12	17	IGKV4-1_Plasma cell
827	5.02E-22	1.945742307	0.145	0.039	1.10E-17	IGHV3-30	17	IGKV4-1_Plasma cell
828	3.95E-201	1.928507689	1	0.639	8.61E-197	HSP90B1	17	IGKV4-1_Plasma cell
829	0	1.857491266	0.746	0.09	0	IGHG1	17	IGKV4-1_Plasma cell
830	3.09E-66	1.831278544	0.142	0.015	6.75E-62	IGKV2D-30	17	IGKV4-1_Plasma cell
831	1.89E-302	1.750987352	0.947	0.224	4.12E-298	SEC11C	17	IGKV4-1_Plasma cell
832	0	1.735222579	0.876	0.051	0	TXNDC5	17	IGKV4-1_Plasma cell
833	3.68E-196	1.684570401	0.997	0.728	8.02E-192	PPIB	17	IGKV4-1_Plasma cell
834	0	1.681306404	0.849	0.051	0	IGLL5	17	IGKV4-1_Plasma cell
835	3.26E-95	1.661678953	0.109	0.006	7.11E-91	IGKV2D-24	17	IGKV4-1_Plasma cell
836	0	1.65907214	0.926	0.098	0	ITM2C	17	IGKV4-1_Plasma cell
837	3.86E-193	1.622621086	0.973	0.457	8.41E-189	XBP1	17	IGKV4-1_Plasma cell
838	0	1.60211482	0.858	0.046	0	RRM2	17	IGKV4-1_Plasma cell
839	5.87E-07	1.590562653	0.284	0.16	0.012794954	IGKV1D-16	17	IGKV4-1_Plasma cell
840	6.80E-211	1.560021697	0.938	0.372	1.48E-206	MYDGF	17	IGKV4-1_Plasma cell
841	1.06E-107	1.521861254	0.222	0.022	2.31E-103	IGHG3	17	IGKV4-1_Plasma cell
842	8.30E-232	1.515729086	0.973	0.37	1.81E-227	SSR3	17	IGKV4-1_Plasma cell
843	0	1.511729128	0.899	0.052	0	DERL3	17	IGKV4-1_Plasma cell
844	4.77E-44	1.504157497	0.45	0.168	1.04E-39	IGHM	17	IGKV4-1_Plasma cell
845	0	1.502733061	0.911	0.065	0	POU2AF1	17	IGKV4-1_Plasma cell
846	0	1.496084075	0.891	0.039	0	TNFRSF17	17	IGKV4-1_Plasma cell
847	7.78E-274	2.279740707	0.935	0.169	1.70E-269	CXCL8	18	CXCL8_MP
848	1.25E-283	2.229395239	0.844	0.122	2.72E-279	CXCL3	18	CXCL8_MP
849	0	2.201013819	0.844	0.099	0	IL1B	18	CXCL8_MP
850	1.11E-206	1.900397519	0.901	0.191	2.42E-202	THBS1	18	CXCL8_MP
851	0	1.900064346	0.794	0.08	0	EREG	18	CXCL8_MP
852	6.72E-236	1.86650878	0.981	0.225	1.46E-231	PLAUR	18	CXCL8_MP
853	7.38E-145	1.821994406	0.977	0.388	1.61E-140	S100A9	18	CXCL8_MP
854	1.21E-196	1.818107354	0.92	0.216	2.64E-192	CXCL2	18	CXCL8_MP
855	4.15E-181	1.770825794	0.969	0.299	9.05E-177	BCL2A1	18	CXCL8_MP
856	7.56E-279	1.759445853	0.908	0.133	1.65E-274	VCAN	18	CXCL8_MP
857	8.22E-187	1.709051991	1	0.283	1.79E-182	LYZ	18	CXCL8_MP
858	4.49E-261	1.586513517	0.958	0.161	9.78E-257	FCN1	18	CXCL8_MP
859	3.74E-150	1.536084531	0.989	0.409	8.15E-146	SOD2	18	CXCL8_MP
860	7.04E-211	1.483821595	0.664	0.09	1.54E-206	G0S2	18	CXCL8_MP
861	0	1.459782087	0.622	0.047	0	TNFAIP6	18	CXCL8_MP
862	6.67E-206	1.453285541	0.935	0.202	1.46E-201	C5AR1	18	CXCL8_MP
863	0	1.433745121	0.798	0.088	0	NLRP3	18	CXCL8_MP
864	7.04E-121	1.282428837	1	0.974	1.53E-116	FTH1	18	CXCL8_MP
865	1.71E-122	1.281916424	0.989	0.476	3.73E-118	S100A8	18	CXCL8_MP
866	1.31E-237	1.240618974	0.622	0.072	2.86E-233	PTGS2	18	CXCL8_MP
867	8.82E-184	1.213921101	0.866	0.18	1.92E-179	CSTA	18	CXCL8_MP
868	5.60E-114	1.207196449	0.977	0.436	1.22E-109	NAMPT	18	CXCL8_MP
869	2.20E-99	1.20689918	0.363	0.053	4.79E-95	CCL20	18	CXCL8_MP
870	2.00E-128	1.205271211	0.992	0.325	4.37E-124	FCER1G	18	CXCL8_MP
871	2.30E-210	1.193694551	0.767	0.127	5.03E-206	CLEC4E	18	CXCL8_MP
872	1.42E-54	1.14473903	0.752	0.355	3.10E-50	AREG	18	CXCL8_MP
873	3.06E-104	1.142923325	0.996	0.742	6.68E-100	S100A6	18	CXCL8_MP
874	1.48E-172	1.131767316	0.347	0.029	3.22E-168	CXCL1	18	CXCL8_MP

875	2.58E-112	1.129048606	0.973	0.492	5.63E-108	COTL1	18	CXCL8_MP
876	5.13E-161	1.113297436	0.79	0.165	1.12E-156	FPR1	18	CXCL8_MP
877	1.70E-103	1.098811571	0.992	0.607	3.70E-99	S100A11	18	CXCL8_MP
878	3.77E-140	1.096049905	0.821	0.201	8.22E-136	MNDA	18	CXCL8_MP
879	1.10E-106	1.089865005	0.958	0.485	2.39E-102	TSPO	18	CXCL8_MP
880	8.66E-131	1.085332742	0.855	0.237	1.89E-126	SPI1	18	CXCL8_MP
881	3.06E-153	1.073163827	0.79	0.173	6.66E-149	SLC11A1	18	CXCL8_MP
882	8.46E-86	1.067275014	0.882	0.397	1.85E-81	IER3	18	CXCL8_MP
883	4.94E-109	1.065487883	0.989	0.422	1.08E-104	CTSS	18	CXCL8_MP
884	2.13E-146	1.06100393	0.817	0.192	4.64E-142	S100A12	18	CXCL8_MP
885	9.84E-102	1.056264437	0.92	0.396	2.15E-97	TKT	18	CXCL8_MP
886	1.43E-230	1.054354548	0.363	0.023	3.12E-226	SERPINB2	18	CXCL8_MP
887	3.99E-130	1.050481572	0.966	0.264	8.70E-126	AIF1	18	CXCL8_MP
888	1.46E-87	1.040601963	0.817	0.311	3.19E-83	STXBP2	18	CXCL8_MP
889	1.09E-224	1.022677315	0.592	0.067	2.38E-220	TREM1	18	CXCL8_MP
890	8.09E-102	1.021161618	1	0.474	1.76E-97	TYROBP	18	CXCL8_MP
891	5.94E-140	1.019201265	0.76	0.169	1.30E-135	ACSL1	18	CXCL8_MP
892	1.27E-99	0.995302828	0.931	0.376	2.77E-95	SAMSN1	18	CXCL8_MP
893	1.09E-226	0.994108372	0.58	0.063	2.38E-222	AQP9	18	CXCL8_MP
894	1.22E-99	0.993741576	0.836	0.292	2.65E-95	GCA	18	CXCL8_MP
895	3.18E-185	0.989409388	0.5	0.057	6.93E-181	IL1RN	18	CXCL8_MP
896	1.22E-155	0.984263597	0.767	0.157	2.65E-151	CLEC7A	18	CXCL8_MP
897	3.58E-222	6.873680195	1	0.352	7.81E-218	HBB	19	HBB_Erthyroid cell
898	0	5.8895557	1	0.093	0	HBA2	19	HBB_Erthyroid cell
899	0	5.347421695	0.996	0.039	0	CA1	19	HBB_Erthyroid cell
900	0	4.78532134	0.973	0.019	0	HBM	19	HBB_Erthyroid cell
901	0	4.651736571	0.976	0.019	0	HBD	19	HBB_Erthyroid cell
902	0	4.487890698	0.988	0.017	0	AHSP	19	HBB_Erthyroid cell
903	0	4.482359711	0.988	0.029	0	HBA1	19	HBB_Erthyroid cell
904	0	3.693351535	0.992	0.052	0	CA2	19	HBB_Erthyroid cell
905	5.46E-229	3.082105099	0.996	0.318	1.19E-224	PRDX2	19	HBB_Erthyroid cell
906	0	3.079494765	0.969	0.002	0	GYPA	19	HBB_Erthyroid cell
907	0	2.931983917	0.945	0.004	0	SLC4A1	19	HBB_Erthyroid cell
908	0	2.867375114	0.984	0.182	0	SLC25A37	19	HBB_Erthyroid cell
909	0	2.866597265	0.969	0.003	0	ALAS2	19	HBB_Erthyroid cell
910	0	2.527183104	0.925	0.004	0	HEMGN	19	HBB_Erthyroid cell
911	0	2.422726444	0.929	0.002	0	GYPB	19	HBB_Erthyroid cell
912	0	2.379740579	0.851	0.108	0	H1F0	19	HBB_Erthyroid cell
913	0	2.341872232	0.949	0.052	0	HMBS	19	HBB_Erthyroid cell
914	2.14E-218	2.289513522	0.988	0.322	4.67E-214	BLVRB	19	HBB_Erthyroid cell
915	0	2.283214792	0.945	0.023	0	SNCA	19	HBB_Erthyroid cell
916	0	2.227101344	0.933	0.002	0	RHAG	19	HBB_Erthyroid cell
917	0	2.143365069	0.945	0.134	0	UROD	19	HBB_Erthyroid cell
918	9.63E-288	2.01553864	0.941	0.179	2.10E-283	SLC25A39	19	HBB_Erthyroid cell
919	2.03E-99	1.804105451	0.776	0.294	4.43E-95	HIST1H4C	19	HBB_Erthyroid cell
920	4.89E-122	1.746690586	0.62	0.13	1.07E-117	HIST1H1C	19	HBB_Erthyroid cell
921	5.05E-158	1.742049467	0.976	0.5	1.10E-153	GYPC	19	HBB_Erthyroid cell
922	0	1.739096462	0.922	0.131	0	TFRC	19	HBB_Erthyroid cell
923	0	1.714647902	0.859	0.002	0	EPB42	19	HBB_Erthyroid cell
924	0	1.711761962	0.851	0.003	0	KLF1	19	HBB_Erthyroid cell
925	0	1.67122467	0.875	0.037	0	FECH	19	HBB_Erthyroid cell
926	0	1.658735964	0.902	0.087	0	FAM210B	19	HBB_Erthyroid cell
927	0	1.63989989	0.753	0.047	0	TUBG1	19	HBB_Erthyroid cell
928	1.13E-242	1.629014345	0.922	0.196	2.46E-238	CAT	19	HBB_Erthyroid cell
929	0	1.587892528	0.69	0.054	0	RRM2	19	HBB_Erthyroid cell
930	0	1.587272404	0.851	0.018	0	SELENBP1	19	HBB_Erthyroid cell
931	0	1.574326634	0.773	0.013	0	SMIM1	19	HBB_Erthyroid cell
932	1.34E-83	1.571058159	0.867	0.592	2.91E-79	TUBA1B	19	HBB_Erthyroid cell
933	0	1.559720175	0.761	0.001	0	MYL4	19	HBB_Erthyroid cell
934	0	1.548649528	0.827	0.012	0	RFESD	19	HBB_Erthyroid cell
935	0	1.507072839	0.773	0.001	0	RHD	19	HBB_Erthyroid cell
936	1.60E-87	1.490251075	0.851	0.52	3.49E-83	TUBB	19	HBB_Erthyroid cell
937	0	1.462719431	0.804	0.08	0	UBAC1	19	HBB_Erthyroid cell
938	5.91E-108	1.459082059	0.867	0.387	1.29E-103	HMGB2	19	HBB_Erthyroid cell
939	0	1.433653565	0.765	0.012	0	TMEM56	19	HBB_Erthyroid cell
940	2.08E-146	1.422555859	0.914	0.352	4.54E-142	RAD23A	19	HBB_Erthyroid cell
941	0	1.419604731	0.78	0.001	0	SPTA1	19	HBB_Erthyroid cell
942	7.34E-122	1.413583031	0.875	0.366	1.60E-117	MGST3	19	HBB_Erthyroid cell
943	0	1.398335667	0.8	0.01	0	DMTN	19	HBB_Erthyroid cell
944	0	1.395538618	0.663	0.062	0	NUSAP1	19	HBB_Erthyroid cell
945	0	1.393460333	0.769	0.067	0	MINPP1	19	HBB_Erthyroid cell
946	0	1.35104072	0.749	0.008	0	CR1L	19	HBB_Erthyroid cell
947	0	4.579099362	0.825	0.038	0	TAGLN	20	TAGLN_Stellate cell

948	0	4.020183312	0.929	0.022	0	MYL9	20	TAGLN_Stellate cell
949	4.12E-269	3.878885813	0.802	0.061	8.99E-265	ACTA2	20	TAGLN_Stellate cell
950	0	3.808886202	0.905	0.033	0	TPM2	20	TAGLN_Stellate cell
951	2.67E-12	3.50884363	0.135	0.03	5.83E-08	ITLN1	20	TAGLN_Stellate cell
952	0	3.472012145	0.865	0.058	0	ADIRF	20	TAGLN_Stellate cell
953	0	3.390960243	0.46	0.005	0	RGS5	20	TAGLN_Stellate cell
954	0	3.264398794	0.817	0.047	0	SPARCL1	20	TAGLN_Stellate cell
955	0	3.123554893	0.794	0.005	0	IGFBP5	20	TAGLN_Stellate cell
956	0	3.096453529	0.937	0.061	0	CALD1	20	TAGLN_Stellate cell
957	6.67E-221	3.079258717	0.881	0.095	1.45E-216	TPM1	20	TAGLN_Stellate cell
958	4.94E-104	3.042009988	0.889	0.234	1.08E-99	IGFBP7	20	TAGLN_Stellate cell
959	0	2.881895781	0.69	0.005	0	MYH11	20	TAGLN_Stellate cell
960	0	2.754604744	0.794	0.006	0	SOD3	20	TAGLN_Stellate cell
961	7.00E-77	2.631381337	0.897	0.36	1.53E-72	DSTN	20	TAGLN_Stellate cell
962	1.11E-232	2.598842105	0.825	0.075	2.42E-228	BGN	20	TAGLN_Stellate cell
963	9.00E-216	2.519586359	0.762	0.064	1.96E-211	MT1M	20	TAGLN_Stellate cell
964	0	2.424245096	0.492	0.001	0	PLN	20	TAGLN_Stellate cell
965	3.46E-100	2.269063463	0.389	0.034	7.54E-96	MT1A	20	TAGLN_Stellate cell
966	3.24E-163	2.268400285	0.135	0.002	7.06E-159	PRG4	20	TAGLN_Stellate cell
967	0	2.236481748	0.492	0.001	0	RERGL	20	TAGLN_Stellate cell
968	2.69E-202	2.141477105	0.738	0.069	5.86E-198	MFGE8	20	TAGLN_Stellate cell
969	1.06E-240	2.131240591	0.667	0.044	2.31E-236	PPP1R14A	20	TAGLN_Stellate cell
970	0	2.106229026	0.579	0.018	0	IGFBP6	20	TAGLN_Stellate cell
971	7.59E-162	2.070437653	0.659	0.065	1.65E-157	C11orf96	20	TAGLN_Stellate cell
972	0	2.066149502	0.452	0.002	0	NDUFA4L2	20	TAGLN_Stellate cell
973	0	2.050417045	0.683	0.004	0	CRYAB	20	TAGLN_Stellate cell
974	7.55E-39	1.995990546	0.135	0.011	1.65E-34	SLPI	20	TAGLN_Stellate cell
975	0	1.981824069	0.73	0.013	0	PLAC9	20	TAGLN_Stellate cell
976	0	1.939412452	0.69	0.016	0	CSRP2	20	TAGLN_Stellate cell
977	6.53E-187	1.877318163	0.706	0.065	1.42E-182	BCAM	20	TAGLN_Stellate cell
978	1.06E-110	1.87170925	0.881	0.178	2.31E-106	MT1E	20	TAGLN_Stellate cell
979	3.02E-79	1.859862368	0.675	0.147	6.58E-75	CSRP1	20	TAGLN_Stellate cell
980	0	1.859691429	0.444	0.001	0	DCN	20	TAGLN_Stellate cell
981	1.97E-22	1.85471231	0.659	0.337	4.29E-18	MT1X	20	TAGLN_Stellate cell
982	9.27E-104	1.8478268	0.754	0.139	2.02E-99	SELM	20	TAGLN_Stellate cell
983	0	1.838218858	0.508	0.002	0	MFAP4	20	TAGLN_Stellate cell
984	9.71E-268	1.837690808	0.817	0.056	2.12E-263	MGP	20	TAGLN_Stellate cell
985	5.08E-130	1.83405978	0.794	0.118	1.11E-125	SERPING1	20	TAGLN_Stellate cell
986	3.33E-40	1.821010937	0.841	0.513	7.26E-36	CRIP1	20	TAGLN_Stellate cell
987	0	1.798561864	0.603	0.009	0	MYLK	20	TAGLN_Stellate cell
988	0	1.77925811	0.683	0.016	0	MAP1B	20	TAGLN_Stellate cell
989	1.97E-194	1.747686177	0.667	0.055	4.30E-190	ISYNA1	20	TAGLN_Stellate cell
990	0	1.737070554	0.437	0.008	0	FABP4	20	TAGLN_Stellate cell
991	7.35E-143	1.723227005	0.786	0.108	1.60E-138	CAV1	20	TAGLN_Stellate cell
992	2.24E-154	1.722626861	0.659	0.069	4.89E-150	PALLD	20	TAGLN_Stellate cell
993	2.03E-25	1.71055467	0.286	0.061	4.42E-21	CCL2	20	TAGLN_Stellate cell
994	0	1.700424204	0.714	0.028	0	PRKCDBP	20	TAGLN_Stellate cell
995	3.18E-46	1.68126467	0.286	0.039	6.93E-42	CFH	20	TAGLN_Stellate cell
996	1.03E-106	1.670536401	0.548	0.068	2.24E-102	NET1	20	TAGLN_Stellate cell
997	0	5.132647075	1	0.072	0	SAA1	21	SAA1_Hepatocyte
998	9.69E-185	4.955501969	1	0.164	2.11E-180	ALB	21	SAA1_Hepatocyte
999	0	4.908471192	1	0.048	0	APOC3	21	SAA1_Hepatocyte
1000	1.02E-298	4.855125512	0.992	0.088	2.23E-294	MT1G	21	SAA1_Hepatocyte
1001	0	4.395086513	0.984	0.031	0	SAA2	21	SAA1_Hepatocyte
1002	8.23E-228	4.375837349	1	0.126	1.79E-223	APOC1	21	SAA1_Hepatocyte
1003	9.32E-122	4.307425515	1	0.291	2.03E-117	SERPINA1	21	SAA1_Hepatocyte
1004	0	4.267782532	0.852	0.02	0	CYP2E1	21	SAA1_Hepatocyte
1005	0	4.207786357	0.951	0.026	0	TTR	21	SAA1_Hepatocyte
1006	0	4.170767324	0.943	0.02	0	APOA2	21	SAA1_Hepatocyte
1007	1.28E-294	4.155772892	0.943	0.077	2.78E-290	HP	21	SAA1_Hepatocyte
1008	0	4.152809542	0.959	0.054	0	ORM1	21	SAA1_Hepatocyte
1009	0	4.109291027	0.992	0.037	0	ASS1	21	SAA1_Hepatocyte
1010	0	4.10269421	0.992	0.018	0	ADH1B	21	SAA1_Hepatocyte
1011	0	3.922137094	0.836	0.042	0	APOA1	21	SAA1_Hepatocyte
1012	0	3.911271711	0.967	0.016	0	APOC4-APOC2	21	SAA1_Hepatocyte
1013	0	3.829311263	0.91	0.024	0	RBP4	21	SAA1_Hepatocyte
1014	4.44E-123	3.744710648	1	0.289	9.69E-119	DCXR	21	SAA1_Hepatocyte
1015	2.73E-110	3.612768183	1	0.334	5.95E-106	MT1X	21	SAA1_Hepatocyte
1016	0	3.606831252	0.975	0.011	0	HPD	21	SAA1_Hepatocyte
1017	0	3.597663616	0.967	0.062	0	MT1M	21	SAA1_Hepatocyte
1018	0	3.587194836	0.934	0.012	0	FABP1	21	SAA1_Hepatocyte
1019	0	3.485192369	0.975	0.01	0	AGXT	21	SAA1_Hepatocyte
1020	5.34E-167	3.409252424	0.992	0.177	1.17E-162	MT1E	21	SAA1_Hepatocyte

1021	0	3.392463114	0.811	0.028	0	FGG	21	SAA1_Hepatocyte
1022	0	3.389460076	0.705	0.018	0	HAMP	21	SAA1_Hepatocyte
1023	0	3.356834625	0.828	0.014	0	MT1H	21	SAA1_Hepatocyte
1024	0	3.268562657	0.934	0.022	0	NNMT	21	SAA1_Hepatocyte
1025	2.07E-221	3.267738949	0.975	0.119	4.51E-217	MT1F	21	SAA1_Hepatocyte
1026	0	3.218392093	0.82	0.032	0	FGB	21	SAA1_Hepatocyte
1027	0	3.145660966	0.959	0.008	0	GNMT	21	SAA1_Hepatocyte
1028	0	3.13605049	0.951	0.012	0	ALDOB	21	SAA1_Hepatocyte
1029	2.50E-156	3.061578256	0.992	0.199	5.46E-152	CYB5A	21	SAA1_Hepatocyte
1030	0	3.031263948	0.934	0.054	0	HRSP12	21	SAA1_Hepatocyte
1031	0	3.009319266	0.951	0.006	0	TAT	21	SAA1_Hepatocyte
1032	0	2.966286556	0.918	0.006	0	GSTA1	21	SAA1_Hepatocyte
1033	0	2.915044752	0.918	0.026	0	VTN	21	SAA1_Hepatocyte
1034	0	2.905684131	0.844	0.024	0	AMBP	21	SAA1_Hepatocyte
1035	0	2.902679699	0.91	0.004	0	ADH4	21	SAA1_Hepatocyte
1036	0	2.797854864	0.836	0.004	0	TDO2	21	SAA1_Hepatocyte
1037	0	2.734926993	0.705	0.005	0	SAA4	21	SAA1_Hepatocyte
1038	0	2.71065212	0.902	0.013	0	ARG1	21	SAA1_Hepatocyte
1039	0	2.682378712	0.828	0.021	0	SERPINA3	21	SAA1_Hepatocyte
1040	0	2.677449419	0.877	0.004	0	SULT2A1	21	SAA1_Hepatocyte
1041	0	2.644996158	0.877	0.045	0	GATM	21	SAA1_Hepatocyte
1042	1.92E-235	2.643035913	0.885	0.081	4.20E-231	APOE	21	SAA1_Hepatocyte
1043	0	2.607868258	0.697	0.015	0	APOH	21	SAA1_Hepatocyte
1044	1.09E-177	2.603649386	0.902	0.128	2.37E-173	GSTM1	21	SAA1_Hepatocyte
1045	3.34E-98	2.60155431	0.984	0.378	7.28E-94	PEBP1	21	SAA1_Hepatocyte
1046	1.29E-131	2.540096638	0.975	0.234	2.82E-127	ADI1	21	SAA1_Hepatocyte
1047	1.78E-166	1.643592673	0.78	0.079	3.87E-162	HP	22	Mixed cell
1048	9.17E-117	1.548503587	0.899	0.165	2.00E-112	ALB	22	Mixed cell
1049	2.56E-184	1.457106131	0.706	0.056	5.57E-180	ORM1	22	Mixed cell
1050	9.26E-140	1.396494573	0.495	0.035	2.02E-135	FGB	22	Mixed cell
1051	1.83E-89	1.155698286	0.45	0.045	3.99E-85	APOA1	22	Mixed cell
1052	3.25E-131	1.102201116	0.459	0.032	7.08E-127	FGG	22	Mixed cell
1053	6.15E-130	1.093839445	0.385	0.023	1.34E-125	FGA	22	Mixed cell
1054	1.01E-131	1.024450354	0.321	0.015	2.21E-127	TF	22	Mixed cell
1055	2.65E-84	0.86696482	0.367	0.031	5.78E-80	VTN	22	Mixed cell
1056	1.01E-107	0.848443232	0.312	0.018	2.21E-103	HPX	22	Mixed cell
1057	1.39E-74	0.845868481	0.33	0.028	3.03E-70	AMBP	22	Mixed cell
1058	1.98E-83	0.823347422	0.56	0.076	4.31E-79	SAA1	22	Mixed cell
1059	4.19E-107	0.805190717	0.321	0.019	9.14E-103	APOH	22	Mixed cell
1060	6.04E-07	0.788032669	0.284	0.132	0.013161746	CD79A	22	Mixed cell
1061	1.10E-14	0.740794952	0.284	0.081	2.40E-10	MS4A1	22	Mixed cell
1062	5.56E-10	0.702264096	0.128	0.029	1.21E-05	TCL1A	22	Mixed cell
1063	5.53E-37	0.682900624	0.431	0.086	1.21E-32	APOE	22	Mixed cell
1064	6.65E-64	0.677614783	0.321	0.031	1.45E-59	TTR	22	Mixed cell
1065	1.27E-20	0.649230296	1	0.981	2.77E-16	MT-ND3	22	Mixed cell
1066	9.28E-62	0.641544395	0.413	0.054	2.02E-57	APOC3	22	Mixed cell
1067	5.40E-20	0.640815067	0.651	0.294	1.18E-15	SERPINA1	22	Mixed cell
1068	5.81E-08	0.628359144	0.266	0.117	0.001267873	A1BG	22	Mixed cell
1069	1.11E-12	0.628345078	0.211	0.055	2.42E-08	BANK1	22	Mixed cell
1070	6.43E-85	0.619996175	0.394	0.036	1.40E-80	SAA2	22	Mixed cell
1071	1.68E-08	0.617835602	0.725	0.604	0.000366148	CD37	22	Mixed cell
1072	5.41E-52	0.608240782	0.202	0.015	1.18E-47	AZGP1	22	Mixed cell
1073	2.57E-10	0.607493245	0.853	0.674	5.61E-06	TXNIP	22	Mixed cell
1074	8.06E-19	0.604788414	0.954	0.917	1.76E-14	RPS29	22	Mixed cell
1075	5.63E-41	0.598655192	0.239	0.026	1.23E-36	SERPINA3	22	Mixed cell
1076	2.27E-12	0.58453115	0.963	0.952	4.94E-08	MT-ND4	22	Mixed cell
1077	2.02E-09	0.579264631	0.183	0.055	4.41E-05	ADAM28	22	Mixed cell
1078	2.42E-09	0.572361263	0.587	0.355	5.28E-05	STK17A	22	Mixed cell
1079	5.89E-11	0.569841088	0.945	0.925	1.28E-06	MT-ND2	22	Mixed cell
1080	9.18E-33	0.568616995	0.202	0.024	2.00E-28	C3	22	Mixed cell
1081	8.34E-15	0.55157898	0.872	0.48	1.82E-10	CD69	22	Mixed cell
1082	8.23E-12	0.547437484	0.697	0.413	1.79E-07	IL2RG	22	Mixed cell
1083	3.40E-52	0.545417057	0.119	0.005	7.41E-48	PLG	22	Mixed cell
1084	2.83E-10	0.544596299	0.44	0.202	6.17E-06	LTB	22	Mixed cell
1085	4.12E-09	0.544513475	0.156	0.043	8.98E-05	LINC00926	22	Mixed cell
1086	6.44E-45	0.543541553	0.266	0.03	1.40E-40	RBP4	22	Mixed cell
1087	3.66E-08	0.539145912	0.44	0.237	0.00079853	FCMR	22	Mixed cell
1088	0	4.090608533	1	0.009	0	FXYD2	23	FXYD2_Cholangiocytes
1089	0	3.722191417	0.962	0.007	0	DEFB1	23	FXYD2_Cholangiocytes
1090	0	3.402967943	0.962	0.034	0	KRT18	23	FXYD2_Cholangiocytes
1091	0	3.3333814	0.936	0.034	0	KRT8	23	FXYD2_Cholangiocytes
1092	7.88E-120	3.133011514	0.974	0.154	1.72E-115	ANXA4	23	FXYD2_Cholangiocytes
1093	3.84E-181	3.009714787	1	0.096	8.38E-177	CLU	23	FXYD2_Cholangiocytes

1094	1.59E-104	3.005161694	0.962	0.167	3.47E-100	ALB	23	FXYD2_Cholangiocytes
1095	0	2.753970475	0.923	0.026	0	AMBP	23	FXYD2_Cholangiocytes
1096	0	2.664376115	0.756	0.003	0	ELF3	23	FXYD2_Cholangiocytes
1097	0	2.623406999	0.718	0.001	0	CLDN4	23	FXYD2_Cholangiocytes
1098	0	2.5328405	0.795	0.004	0	KRT7	23	FXYD2_Cholangiocytes
1099	1.01E-74	2.513762067	1	0.293	2.20E-70	SERPINA1	23	FXYD2_Cholangiocytes
1100	0	2.485758072	0.808	0.006	0	SPP1	23	FXYD2_Cholangiocytes
1101	0	2.481782091	0.859	0.007	0	AGT	23	FXYD2_Cholangiocytes
1102	4.13E-39	2.344748179	0.41	0.061	9.01E-35	CCL2	23	FXYD2_Cholangiocytes
1103	0	2.271946598	0.731	0.024	0	SERPINA3	23	FXYD2_Cholangiocytes
1104	0	2.271845849	0.872	0.029	0	VTN	23	FXYD2_Cholangiocytes
1105	0	2.243689804	0.859	0.002	0	TM4SF4	23	FXYD2_Cholangiocytes
1106	0	2.237707002	0.795	0.011	0	PAH	23	FXYD2_Cholangiocytes
1107	0	2.204966169	0.744	0.021	0	C3	23	FXYD2_Cholangiocytes
1108	3.37E-245	2.184814632	0.718	0.031	7.36E-241	FGG	23	FXYD2_Cholangiocytes
1109	1.09E-92	2.055927093	0.526	0.046	2.37E-88	TNFRSF12A	23	FXYD2_Cholangiocytes
1110	0	2.050766644	0.782	0.028	0	RBP4	23	FXYD2_Cholangiocytes
1111	3.32E-103	2.021555151	0.859	0.12	7.24E-99	SERPING1	23	FXYD2_Cholangiocytes
1112	0	2.009209175	0.577	0.002	0	TACSTD2	23	FXYD2_Cholangiocytes
1113	1.71E-79	1.992566207	0.91	0.18	3.74E-75	MT1E	23	FXYD2_Cholangiocytes
1114	0	1.988095152	0.679	0	0	SFRP5	23	FXYD2_Cholangiocytes
1115	0	1.942969308	0.731	0.011	0	GC	23	FXYD2_Cholangiocytes
1116	0	1.935014351	0.667	0.003	0	KRT19	23	FXYD2_Cholangiocytes
1117	0	1.901743443	0.731	0.006	0	SERPINA5	23	FXYD2_Cholangiocytes
1118	3.52E-60	1.890875349	0.295	0.021	7.68E-56	SCGB3A1	23	FXYD2_Cholangiocytes
1119	0	1.832446429	0.654	0.002	0	CLDN3	23	FXYD2_Cholangiocytes
1120	0	1.816628874	0.718	0.002	0	CLDN10	23	FXYD2_Cholangiocytes
1121	5.99E-154	1.814532757	0.731	0.055	1.31E-149	SH3YL1	23	FXYD2_Cholangiocytes
1122	0	1.803137164	0.397	0.001	0	UBD	23	FXYD2_Cholangiocytes
1123	0	1.767006421	0.59	0.003	0	LGALS4	23	FXYD2_Cholangiocytes
1124	0	1.766142107	0.526	0.001	0	CXCL6	23	FXYD2_Cholangiocytes
1125	1.53E-137	1.746881953	0.756	0.064	3.33E-133	CD24	23	FXYD2_Cholangiocytes
1126	0	1.73522367	0.538	0.012	0	SERPINA3.1	23	FXYD2_Cholangiocytes
1127	0	1.732807046	0.577	0.004	0	CYP3A5	23	FXYD2_Cholangiocytes
1128	0	1.706893406	0.564	0.003	0	CLDN1	23	FXYD2_Cholangiocytes
1129	6.95E-66	1.687863082	0.385	0.033	1.52E-61	CXCL1	23	FXYD2_Cholangiocytes
1130	0	1.561778398	0.5	0.001	0	PIGR	23	FXYD2_Cholangiocytes
1131	6.56E-236	1.538711155	0.603	0.022	1.43E-231	FGA	23	FXYD2_Cholangiocytes
1132	1.98E-79	1.528033991	0.436	0.035	4.31E-75	CYR61	23	FXYD2_Cholangiocytes
1133	4.15E-50	1.523724761	0.628	0.118	9.04E-46	ANPEP	23	FXYD2_Cholangiocytes
1134	6.29E-59	1.506242135	0.833	0.202	1.37E-54	CYB5A	23	FXYD2_Cholangiocytes
1135	3.52E-255	1.504252034	0.5	0.013	7.68E-251	APCS	23	FXYD2_Cholangiocytes
1136	1.94E-160	1.487222388	0.679	0.042	4.23E-156	AQP1	23	FXYD2_Cholangiocytes
1137	9.22E-158	1.483045464	0.308	0.008	2.01E-153	CRYAB	23	FXYD2_Cholangiocytes
1138	2.89E-110	2.690017156	0.979	0.091	6.30E-106	C7	24	C7_Endo
1139	5.47E-64	2.528139896	1	0.176	1.19E-59	RNASE1	24	C7_Endo
1140	8.09E-166	2.524143108	0.854	0.043	1.76E-161	VWF	24	C7_Endo
1141	2.06E-69	2.476587179	1	0.169	4.50E-65	PRSS23	24	C7_Endo
1142	6.49E-92	2.353297284	1	0.117	1.41E-87	RAMP3	24	C7_Endo
1143	1.79E-81	2.089524027	0.917	0.103	3.90E-77	LIFR	24	C7_Endo
1144	0	2.086896603	0.458	0.003	0	POSTN	24	C7_Endo
1145	0	2.081967043	0.667	0.01	0	IL1RL1	24	C7_Endo
1146	1.51E-133	2.070473399	0.979	0.075	3.29E-129	IL1R1	24	C7_Endo
1147	1.85E-57	2.045595847	0.979	0.183	4.04E-53	ENG	24	C7_Endo
1148	2.08E-36	1.965544083	0.625	0.1	4.54E-32	EMP1	24	C7_Endo
1149	5.45E-30	1.959055698	1	0.431	1.19E-25	TIMP1	24	C7_Endo
1150	0	1.915369417	0.833	0.019	0	ADGRG6	24	C7_Endo
1151	6.12E-177	1.911269793	0.896	0.045	1.33E-172	LTC4S	24	C7_Endo
1152	3.05E-36	1.910180857	0.896	0.226	6.65E-32	CXCL2	24	C7_Endo
1153	7.62E-77	1.885998123	0.938	0.115	1.66E-72	HSPG2	24	C7_Endo
1154	1.33E-116	1.86741065	0.979	0.082	2.90E-112	SRPX	24	C7_Endo
1155	1.16E-72	1.862428156	1	0.126	2.54E-68	DNASE1L3	24	C7_Endo
1156	4.96E-85	1.85636617	0.917	0.099	1.08E-80	PTPRB	24	C7_Endo
1157	0	1.804522115	0.5	0.006	0	SELE	24	C7_Endo
1158	1.33E-44	1.803585811	0.938	0.212	2.91E-40	APP	24	C7_Endo
1159	3.02E-59	1.776707571	0.625	0.061	6.58E-55	CCL2	24	C7_Endo
1160	5.71E-61	1.771963497	1	0.159	1.24E-56	TFPI	24	C7_Endo
1161	1.62E-57	1.764055833	0.958	0.142	3.54E-53	TM4SF1	24	C7_Endo
1162	2.30E-27	1.750144402	0.438	0.061	5.02E-23	MGP	24	C7_Endo
1163	0	1.730102148	0.75	0.003	0	RSPO3	24	C7_Endo
1164	1.80E-47	1.728035209	1	0.237	3.92E-43	IGFBP7	24	C7_Endo
1165	2.59E-286	1.727282735	0.396	0.004	5.66E-282	CSF3	24	C7_Endo
1166	2.29E-70	1.705669999	1	0.135	4.99E-66	TIMP3	24	C7_Endo

1167	1.19E-127	1.703182048	0.5	0.018	2.59E-123	CTGF	24	C7_Endo
1168	2.44E-32	1.676908434	0.354	0.036	5.33E-28	MT1A	24	C7_Endo
1169	1.41E-48	1.654266359	0.729	0.107	3.08E-44	PMP22	24	C7_Endo
1170	3.62E-22	1.64770444	0.812	0.276	7.90E-18	ICAM1	24	C7_Endo
1171	4.66E-86	1.638483757	0.833	0.078	1.02E-81	MMRN1	24	C7_Endo
1172	8.98E-68	1.637098223	0.875	0.114	1.96E-63	TGM2	24	C7_Endo
1173	7.39E-35	1.625817077	0.354	0.034	1.61E-30	CXCL1	24	C7_Endo
1174	1.10E-16	1.615908667	0.979	0.658	2.39E-12	MT2A	24	C7_Endo
1175	0	1.615009203	0.812	0.005	0	SELP	24	C7_Endo
1176	3.45E-21	1.602796939	0.417	0.074	7.52E-17	TFPI2	24	C7_Endo
1177	3.41E-82	1.589269408	0.917	0.098	7.45E-78	CLU	24	C7_Endo
1178	0	1.577306214	0.771	0.007	0	INMT	24	C7_Endo
1179	8.19E-117	1.565327975	0.771	0.051	1.79E-112	PKHD1L1	24	C7_Endo
1180	1.35E-56	1.558310265	0.5	0.042	2.94E-52	SERPINE1	24	C7_Endo
1181	6.44E-66	1.550565485	0.875	0.104	1.40E-61	AKAP12	24	C7_Endo
1182	0	1.547843681	0.729	0.012	0	FBLN2	24	C7_Endo
1183	1.18E-37	1.546245842	0.938	0.26	2.57E-33	CD59	24	C7_Endo
1184	1.86E-86	1.545549346	0.833	0.079	4.05E-82	LIMS2	24	C7_Endo
1185	7.64E-59	1.543701683	0.875	0.125	1.67E-54	PLPP1	24	C7_Endo
1186	6.95E-66	1.528067387	0.708	0.071	1.52E-61	GJA4	24	C7_Endo
1187	1.17E-61	1.520524134	0.917	0.124	2.54E-57	SDPR	24	C7_Endo
1188	0	5.02097809	0.882	0.007	0	CCL21	25	Mixed cell
1189	3.03E-220	3.869762547	0.971	0.031	6.60E-216	TFF3	25	Mixed cell
1190	0	3.460164474	0.735	0.01	0	FABP4	25	Mixed cell
1191	3.49E-93	2.795798443	0.971	0.078	7.62E-89	MMRN1	25	Mixed cell
1192	2.55E-77	2.721781861	0.824	0.061	5.57E-73	MGP	25	Mixed cell
1193	0	2.495743106	0.647	0.008	0	NTS	25	Mixed cell
1194	2.02E-38	2.467816309	1	0.238	4.41E-34	IGFBP7	25	Mixed cell
1195	6.47E-58	2.460111244	0.882	0.099	1.41E-53	CLU	25	Mixed cell
1196	2.58E-56	2.229092885	1	0.136	5.64E-52	GNG11	25	Mixed cell
1197	1.83E-156	2.217620898	0.853	0.033	3.98E-152	SNCG	25	Mixed cell
1198	1.22E-45	2.205825119	0.971	0.16	2.66E-41	TFPI	25	Mixed cell
1199	9.59E-82	2.175099872	0.853	0.063	2.09E-77	ADIRF	25	Mixed cell
1200	0	2.095924093	0.529	0.004	0	EFEMP1	25	Mixed cell
1201	7.04E-29	2.084039452	0.941	0.267	1.54E-24	FABP5	25	Mixed cell
1202	1.51E-34	2.021199159	0.853	0.148	3.28E-30	IGFBP4	25	Mixed cell
1203	1.34E-33	1.943029866	0.941	0.212	2.91E-29	APP	25	Mixed cell
1204	3.19E-50	1.874432687	0.853	0.107	6.95E-46	PPFIBP1	25	Mixed cell
1205	1.08E-38	1.859555131	0.853	0.136	2.35E-34	TIMP3	25	Mixed cell
1206	4.73E-104	1.857514859	0.853	0.051	1.03E-99	PKHD1L1	25	Mixed cell
1207	4.12E-66	1.84152331	0.912	0.09	8.99E-62	RAMP2	25	Mixed cell
1208	8.77E-50	1.800632466	0.941	0.125	1.91E-45	SDPR	25	Mixed cell
1209	1.14E-45	1.740571344	0.882	0.126	2.48E-41	CNN3	25	Mixed cell
1210	4.11E-143	1.710754178	0.765	0.028	8.97E-139	NNMT	25	Mixed cell
1211	0	1.696589485	0.412	0.002	0	APOD	25	Mixed cell
1212	8.17E-13	1.649477427	0.353	0.062	1.78E-08	CCL2	25	Mixed cell
1213	2.07E-21	1.641877794	0.971	0.492	4.51E-17	ANXA2	25	Mixed cell
1214	5.35E-49	1.632926011	0.824	0.102	1.17E-44	FXYD6	25	Mixed cell
1215	1.09E-42	1.626887565	0.412	0.028	2.37E-38	PLAT	25	Mixed cell
1216	3.21E-61	1.605112487	0.794	0.074	6.99E-57	ECSCR.1	25	Mixed cell
1217	1.06E-72	1.580109002	0.824	0.067	2.31E-68	CALD1	25	Mixed cell
1218	6.72E-40	1.577995457	0.882	0.133	1.47E-35	CALCRL	25	Mixed cell
1219	1.77E-32	1.569294681	0.735	0.128	3.86E-28	SHC1	25	Mixed cell
1220	1.95E-142	1.559235863	0.853	0.036	4.25E-138	PROCR	25	Mixed cell
1221	2.46E-24	1.550055376	0.882	0.223	5.35E-20	ID1	25	Mixed cell
1222	2.91E-77	1.530742681	0.882	0.071	6.35E-73	COX7A1	25	Mixed cell
1223	2.16E-31	1.508415497	0.794	0.137	4.71E-27	THBD	25	Mixed cell
1224	6.89E-23	1.500862492	0.882	0.261	1.50E-18	CD59	25	Mixed cell
1225	1.77E-301	1.500072129	0.618	0.008	3.86E-297	ABI3BP	25	Mixed cell
1226	3.28E-35	1.490124882	0.647	0.083	7.16E-31	NRP2	25	Mixed cell
1227	1.43E-16	1.466404901	1	0.84	3.12E-12	VIM	25	Mixed cell
1228	1.94E-46	1.465657251	0.794	0.093	4.22E-42	NUPR1	25	Mixed cell
1229	2.99E-33	1.465294357	0.912	0.17	6.52E-29	CD9	25	Mixed cell
1230	1.19E-35	1.461434147	0.824	0.126	2.59E-31	PLPP1	25	Mixed cell
1231	1.03E-56	1.419798106	0.706	0.061	2.25E-52	NR2F1	25	Mixed cell
1232	5.45E-24	1.402301526	0.912	0.272	1.19E-19	CD151	25	Mixed cell
1233	6.87E-47	1.392216616	0.647	0.062	1.50E-42	PLSCR4	25	Mixed cell
1234	1.42E-35	1.390466212	0.765	0.112	3.09E-31	CAV1	25	Mixed cell
1235	4.58E-142	1.380405138	0.618	0.018	9.98E-138	RARRES2	25	Mixed cell
1236	7.01E-49	1.378998589	0.853	0.102	1.53E-44	PTRF	25	Mixed cell
1237	3.02E-24	1.378197319	0.912	0.259	6.58E-20	TGFBR2	25	Mixed cell

Table 3. List of markers information for mononuclear phagocyte clusters, related to Figure 2.

	p_val	avg_logFC	pct.1	pct.2	p_val_adj	gene	cluster	cell type
1	0	2.417559111	0.964	0.185	0	C1QC	1	C1QC_KC
2	0	2.318395253	0.965	0.18	0	C1QB	1	C1QC_KC
3	0	2.283970776	0.813	0.107	0	SLC40A1	1	C1QC_KC
4	0	2.198496178	0.766	0.049	0	SEPP1	1	C1QC_KC
5	0	2.19335533	0.967	0.217	0	C1QA	1	C1QC_KC
6	0	1.955031671	0.65	0.066	0	APOC1	1	C1QC_KC
7	0	1.89287979	0.786	0.064	0	LGMN	1	C1QC_KC
8	0	1.81717709	0.807	0.098	0	FOLR2	1	C1QC_KC
9	3.04E-211	1.796815215	0.455	0.03	6.64E-207	APOE	1	C1QC_KC
10	0	1.632536636	0.92	0.464	0	LIPA	1	C1QC_KC
11	1.14E-93	1.42422339	0.426	0.136	2.49E-89	RNASE1	1	C1QC_KC
12	1.87E-159	1.421917259	0.593	0.211	4.08E-155	MARCO	1	C1QC_KC
13	0	1.413525674	0.887	0.253	0	HLA-DQA1	1	C1QC_KC
14	0	1.407972391	0.891	0.346	0	MS4A4A	1	C1QC_KC
15	0	1.353316623	0.985	0.65	0	HLA-DRB5	1	C1QC_KC
16	5.07E-96	1.339987528	0.53	0.214	1.10E-91	CCL4L2	1	C1QC_KC
17	1.27E-193	1.302820953	0.627	0.174	2.76E-189	PDK4	1	C1QC_KC
18	2.21E-283	1.259259798	0.613	0.064	4.81E-279	GPNMB	1	C1QC_KC
19	0	1.241606923	0.965	0.575	0	HLA-DPB1	1	C1QC_KC
20	0	1.230180393	0.684	0.053	0	MRC1	1	C1QC_KC
21	1.97E-294	1.220265063	0.828	0.251	4.29E-290	VSIG4	1	C1QC_KC
22	1.29E-305	1.213841752	0.921	0.481	2.82E-301	MS4A7	1	C1QC_KC
23	1.02E-160	1.179448797	0.869	0.522	2.23E-156	HSPA1A	1	C1QC_KC
24	1.05E-140	1.169028901	0.665	0.258	2.29E-136	HSPB1	1	C1QC_KC
25	0	1.166824563	0.975	0.636	0	HLA-DPA1	1	C1QC_KC
26	7.28E-253	1.122432059	0.773	0.257	1.59E-248	CPM	1	C1QC_KC
27	0	1.120004299	0.998	0.96	0	CD74	1	C1QC_KC
28	8.33E-275	1.063864181	0.89	0.32	1.82E-270	FCGR3A	1	C1QC_KC
29	2.79E-259	1.059173029	0.881	0.482	6.09E-255	CREG1	1	C1QC_KC
30	5.25E-174	1.056364715	0.456	0.06	1.15E-169	RGS1	1	C1QC_KC
31	6.37E-271	1.055207124	0.938	0.541	1.39E-266	HLA-DQB1	1	C1QC_KC
32	0	1.040685759	0.972	0.787	0	MS4A6A	1	C1QC_KC
33	6.15E-209	1.023843895	0.891	0.534	1.34E-204	HMOX1	1	C1QC_KC
34	6.00E-165	1.018235699	0.373	0.024	1.31E-160	LYVE1	1	C1QC_KC
35	1.37E-246	0.996157382	0.811	0.316	2.99E-242	CSF1R	1	C1QC_KC
36	2.36E-101	0.995964627	0.58	0.244	5.15E-97	HSPH1	1	C1QC_KC
37	1.56E-305	0.994104141	0.991	0.872	3.41E-301	HLA-DRA	1	C1QC_KC
38	0	0.972083211	0.97	0.824	0	CD68	1	C1QC_KC
39	1.58E-109	0.969568459	0.821	0.523	3.44E-105	JUN	1	C1QC_KC
40	3.72E-223	0.954356708	0.663	0.158	8.12E-219	MAF	1	C1QC_KC
41	5.35E-98	0.953717426	0.716	0.414	1.17E-93	DNAJB1	1	C1QC_KC
42	1.01E-240	0.943062078	0.93	0.692	2.20E-236	FCGRT	1	C1QC_KC
43	3.01E-155	0.93781981	0.444	0.073	6.57E-151	TMIGD3	1	C1QC_KC
44	6.97E-271	0.931026719	0.982	0.834	1.52E-266	HLA-DRB1	1	C1QC_KC
45	1.24E-74	0.914255102	0.627	0.35	2.70E-70	CCL4	1	C1QC_KC
46	2.25E-209	0.912675509	0.889	0.495	4.90E-205	CTSC	1	C1QC_KC
47	7.86E-252	0.91213726	0.96	0.832	1.71E-247	CTSB	1	C1QC_KC
48	5.95E-228	0.910911167	0.672	0.171	1.30E-223	TSPAN4	1	C1QC_KC
49	6.69E-188	0.89234887	0.706	0.261	1.46E-183	MCOLN1	1	C1QC_KC
50	1.62E-72	0.871113156	0.63	0.352	3.52E-68	CCL3	1	C1QC_KC
51	8.69E-118	0.866556789	0.701	0.32	1.90E-113	HSPA1B	1	C1QC_KC
52	2.28E-52	0.861087938	0.282	0.094	4.97E-48	BAG3	1	C1QC_KC
53	3.01E-204	0.857597072	0.574	0.106	6.57E-200	GPR34	1	C1QC_KC
54	2.47E-250	0.856989905	0.937	0.62	5.38E-246	RNASET2	1	C1QC_KC
55	3.58E-194	0.852027108	0.665	0.186	7.81E-190	ABCA1	1	C1QC_KC
56	2.80E-74	0.843258048	0.671	0.38	6.12E-70	HSPD1	1	C1QC_KC
57	0	0.838387737	0.983	0.85	0	NPC2	1	C1QC_KC
58	2.64E-207	0.833788475	0.797	0.298	5.75E-203	FAM26F	1	C1QC_KC
59	1.60E-102	0.832692108	0.929	0.754	3.49E-98	HSP90AA1	1	C1QC_KC
60	2.63E-127	0.826809644	0.396	0.069	5.74E-123	ID3	1	C1QC_KC
61	1.87E-199	0.815928824	0.595	0.123	4.08E-195	DAB2	1	C1QC_KC
62	1.91E-130	0.813244933	0.902	0.655	4.17E-126	GADD45B	1	C1QC_KC
63	1.41E-185	0.811278826	0.387	0.015	3.07E-181	PLTP	1	C1QC_KC
64	4.02E-197	0.793020227	0.87	0.473	8.76E-193	HLA-DMA	1	C1QC_KC
65	4.96E-153	0.777851914	0.628	0.209	1.08E-148	SMPDL3A	1	C1QC_KC
66	6.83E-153	0.769410195	0.81	0.384	1.49E-148	CTSL	1	C1QC_KC
67	1.83E-37	0.769167555	0.311	0.135	3.99E-33	HSPA6	1	C1QC_KC
68	4.48E-189	0.763366052	0.594	0.136	9.77E-185	MERTK	1	C1QC_KC
69	4.60E-180	0.756083601	0.622	0.167	1.00E-175	MSR1	1	C1QC_KC
70	3.10E-282	0.754144962	0.998	0.988	6.76E-278	CST3	1	C1QC_KC
71	7.50E-172	0.753175132	0.491	0.084	1.64E-167	FUCA1	1	C1QC_KC
72	1.41E-201	0.752910725	0.446	0.031	3.07E-197	SDC3	1	C1QC_KC
73	9.84E-66	0.751602582	0.223	0.038	2.15E-61	SDS	1	C1QC_KC

74	9.99E-224	0.748405563	0.536	0.062	2.18E-219	CLIC2	1	C1QC_KC
75	9.12E-110	0.737922527	0.664	0.309	1.99E-105	FABP5	1	C1QC_KC
76	8.23E-33	0.728460579	0.444	0.262	1.80E-28	CCL3L3	1	C1QC_KC
77	2.48E-180	0.724148516	0.474	0.065	5.41E-176	ATP1B1	1	C1QC_KC
78	1.17E-38	0.720521322	0.135	0.023	2.55E-34	CXCL10	1	C1QC_KC
79	9.60E-230	0.720483206	0.461	0.016	2.09E-225	SLCO2B1	1	C1QC_KC
80	2.21E-147	0.71373069	0.671	0.26	4.82E-143	IL18	1	C1QC_KC
81	5.25E-300	0.706007713	0.999	0.999	1.14E-295	FTL	1	C1QC_KC
82	1.64E-155	0.69849957	0.341	0.016	3.59E-151	SPIC	1	C1QC_KC
83	7.05E-78	0.692463434	0.722	0.412	1.54E-73	HSPE1	1	C1QC_KC
84	3.18E-153	0.679413496	0.591	0.167	6.93E-149	SLC1A3	1	C1QC_KC
85	2.36E-183	0.676894414	0.967	0.848	5.15E-179	CD163	1	C1QC_KC
86	7.47E-33	0.673854569	0.703	0.549	1.63E-28	HSPA5	1	C1QC_KC
87	2.59E-170	0.671002194	0.495	0.081	5.64E-166	EBI3	1	C1QC_KC
88	4.39E-220	0.670440399	0.979	0.893	9.57E-216	ITM2B	1	C1QC_KC
89	1.45E-169	0.668396828	0.468	0.07	3.15E-165	NR1H3	1	C1QC_KC
90	1.44E-68	0.655234184	0.715	0.447	3.14E-64	SGK1	1	C1QC_KC
91	5.41E-156	0.651442655	0.349	0.019	1.18E-151	LILRB5	1	C1QC_KC
92	1.12E-113	0.649660437	0.48	0.14	2.45E-109	GLO1	1	C1QC_KC
93	1.26E-147	0.649356914	0.31	0.008	2.75E-143	CXCL12	1	C1QC_KC
94	1.82E-185	0.64060553	0.392	0.016	3.96E-181	NRP1	1	C1QC_KC
95	1.80E-193	0.633195593	0.416	0.022	3.92E-189	TMEM37	1	C1QC_KC
96	1.93E-57	0.63124381	0.125	0.002	4.20E-53	CD5L	1	C1QC_KC
97	5.00E-171	0.630652674	0.382	0.021	1.09E-166	SIGLEC1	1	C1QC_KC
98	1.33E-140	0.624523208	0.367	0.04	2.90E-136	ARL4C	1	C1QC_KC
99	1.36E-152	0.622479267	0.528	0.122	2.96E-148	FRMD4B	1	C1QC_KC
100	1.45E-138	0.622360115	0.511	0.128	3.16E-134	CD84	1	C1QC_KC
101	1.06E-143	0.619313684	0.783	0.371	2.31E-139	CXCL16	1	C1QC_KC
102	1.98E-181	0.614302772	0.945	0.784	4.32E-177	HLA-E	1	C1QC_KC
103	1.62E-133	0.610904396	0.489	0.115	3.53E-129	CD81	1	C1QC_KC
104	2.08E-104	0.607103497	0.792	0.505	4.54E-100	CECR1	1	C1QC_KC
105	1.76E-130	0.606388139	0.635	0.241	3.83E-126	SCARB2	1	C1QC_KC
106	6.12E-87	0.602753903	0.843	0.584	1.33E-82	HSP90B1	1	C1QC_KC
107	3.06E-39	0.602635604	0.542	0.332	6.66E-35	ICAM1	1	C1QC_KC
108	5.34E-55	0.600568499	0.214	0.045	1.16E-50	DNAJA4	1	C1QC_KC
109	2.54E-151	0.598857805	0.375	0.035	5.53E-147	CD72	1	C1QC_KC
110	1.54E-116	0.597023836	0.637	0.274	3.35E-112	HLA-DMB	1	C1QC_KC
111	1.48E-91	0.593928109	0.794	0.52	3.22E-87	IGSF6	1	C1QC_KC
112	7.95E-82	0.591713536	0.783	0.51	1.73E-77	NINJ1	1	C1QC_KC
113	2.72E-145	0.591004641	0.378	0.04	5.93E-141	CD59	1	C1QC_KC
114	3.18E-123	0.589983153	0.583	0.207	6.94E-119	ADAP2	1	C1QC_KC
115	2.62E-108	0.579987347	0.928	0.753	5.70E-104	GLUL	1	C1QC_KC
116	1.01E-134	0.575775801	0.299	0.012	2.20E-130	A2M	1	C1QC_KC
117	7.66E-147	0.574225659	0.458	0.083	1.67E-142	FMNL2	1	C1QC_KC
118	6.80E-95	0.572525763	0.701	0.351	1.48E-90	CLEC2B	1	C1QC_KC
119	4.64E-50	0.569483896	0.295	0.102	1.01E-45	MMP19	1	C1QC_KC
120	1.51E-126	0.568003565	0.608	0.221	3.29E-122	TECR	1	C1QC_KC
121	2.62E-149	0.567012932	0.357	0.028	5.70E-145	DST	1	C1QC_KC
122	2.21E-92	0.561074478	0.892	0.665	4.83E-88	HSPA8	1	C1QC_KC
123	1.21E-119	0.558798008	0.899	0.692	2.63E-115	BLVRB	1	C1QC_KC
124	3.47E-130	0.557936872	0.319	0.025	7.56E-126	OTOA	1	C1QC_KC
125	2.89E-72	0.556839847	0.878	0.655	6.30E-68	HSP90AB1	1	C1QC_KC
126	7.58E-113	0.556261467	0.656	0.276	1.65E-108	STOM	1	C1QC_KC
127	1.33E-105	0.550180457	0.541	0.2	2.91E-101	HEXA	1	C1QC_KC
128	2.61E-114	0.54892533	0.682	0.307	5.69E-110	SNX5	1	C1QC_KC
129	1.29E-113	0.544963094	0.602	0.229	2.82E-109	CD4	1	C1QC_KC
130	2.25E-87	0.542269209	0.714	0.396	4.92E-83	PLD3	1	C1QC_KC
131	1.74E-111	0.541607646	0.921	0.747	3.80E-107	ASAH1	1	C1QC_KC
132	1.90E-122	0.538166432	0.427	0.09	4.15E-118	AKR1B1	1	C1QC_KC
133	1.96E-70	0.537255727	0.718	0.447	4.28E-66	RBP7	1	C1QC_KC
134	5.39E-125	0.527248299	0.961	0.924	1.17E-120	PSAP	1	C1QC_KC
135	4.97E-35	0.524264891	0.301	0.133	1.08E-30	ZFAND2A	1	C1QC_KC
136	2.44E-96	0.523754244	0.786	0.5	5.32E-92	TPP1	1	C1QC_KC
137	3.68E-80	0.520967936	0.836	0.559	8.03E-76	UBB	1	C1QC_KC
138	6.13E-75	0.519142203	0.745	0.486	1.34E-70	CPVL	1	C1QC_KC
139	4.16E-99	0.516560848	0.981	0.883	9.07E-95	MT-ATP8	1	C1QC_KC
140	5.35E-123	0.516243256	0.351	0.049	1.17E-118	GFRA2	1	C1QC_KC
141	7.48E-88	0.515951767	0.618	0.296	1.63E-83	TCN2	1	C1QC_KC
142	4.24E-116	0.514636467	0.991	0.952	9.24E-112	MT-ND4L	1	C1QC_KC
143	6.21E-97	0.513726654	0.457	0.141	1.35E-92	ACP5	1	C1QC_KC
144	6.14E-75	0.510968247	0.427	0.156	1.34E-70	P2RY13	1	C1QC_KC
145	6.31E-127	0.50921368	0.31	0.025	1.38E-122	ANKH	1	C1QC_KC
146	1.05E-46	0.507443539	0.387	0.178	2.29E-42	PAPSS2	1	C1QC_KC
147	1.09E-64	0.502395291	0.747	0.46	2.37E-60	ID2	1	C1QC_KC
148	8.92E-110	0.501178394	0.536	0.181	1.95E-105	PEBP1	1	C1QC_KC

149	3.56E-85	0.499195982	0.483	0.181	7.76E-81	DNASE2	1	C1QC_KC
150	3.67E-70	0.497527327	0.844	0.606	8.01E-66	FGL2	1	C1QC_KC
151	2.14E-107	0.496505662	0.37	0.072	4.67E-103	WWP1	1	C1QC_KC
152	2.41E-94	0.490430862	0.926	0.788	5.26E-90	CD63	1	C1QC_KC
153	6.03E-109	0.48978306	0.442	0.115	1.31E-104	GPR137B	1	C1QC_KC
154	1.28E-80	0.486772378	0.673	0.357	2.80E-76	PRNP	1	C1QC_KC
155	5.35E-22	0.485692649	0.426	0.274	1.17E-17	EGR1	1	C1QC_KC
156	1.03E-37	0.484589194	0.603	0.396	2.24E-33	CHMP1B	1	C1QC_KC
157	3.00E-64	0.482861803	0.875	0.689	6.53E-60	WSB1	1	C1QC_KC
158	1.39E-79	0.47986495	0.901	0.76	3.03E-75	GRN	1	C1QC_KC
159	7.01E-140	0.478314277	0.283	0.003	1.53E-135	AXL	1	C1QC_KC
160	1.19E-125	0.476576957	0.364	0.05	2.60E-121	CD38	1	C1QC_KC
161	1.26E-116	0.475438853	0.344	0.049	2.75E-112	ABCC5	1	C1QC_KC
162	5.74E-104	0.474468484	0.484	0.152	1.25E-99	CRYL1	1	C1QC_KC
163	7.60E-27	0.474375496	0.188	0.071	1.66E-22	C15orf48	1	C1QC_KC
164	7.38E-73	0.473505369	0.503	0.21	1.61E-68	CACYBP	1	C1QC_KC
165	1.94E-89	0.472715244	0.698	0.361	4.22E-85	DRAM2	1	C1QC_KC
166	1.14E-95	0.471503615	0.9	0.739	2.49E-91	HCLS1	1	C1QC_KC
167	1.18E-111	0.471275751	0.263	0.015	2.57E-107	PMP22	1	C1QC_KC
168	3.42E-95	0.470827632	0.395	0.103	7.47E-91	ACP2	1	C1QC_KC
169	1.15E-122	0.469616881	0.966	0.893	2.50E-118	HLA-A	1	C1QC_KC
170	2.76E-121	0.467156679	0.335	0.04	6.01E-117	EPB41L2	1	C1QC_KC
171	6.95E-90	0.467065961	0.857	0.623	1.52E-85	MFSD1	1	C1QC_KC
172	1.05E-129	0.466976782	0.283	0.01	2.28E-125	FRMD4A	1	C1QC_KC
173	1.12E-84	0.464260354	0.665	0.343	2.44E-80	GAA	1	C1QC_KC
174	4.29E-61	0.46223095	0.892	0.681	9.36E-57	RHOB	1	C1QC_KC
175	1.66E-83	0.461897274	0.594	0.281	3.61E-79	CORO1B	1	C1QC_KC
176	7.18E-84	0.461593709	0.514	0.204	1.57E-79	IDH1	1	C1QC_KC
177	8.59E-78	0.461433519	0.734	0.442	1.87E-73	SNX6	1	C1QC_KC
178	1.10E-96	0.459869202	0.453	0.131	2.39E-92	RGL1	1	C1QC_KC
179	4.14E-79	0.4572614	0.679	0.365	9.02E-75	LAMP1	1	C1QC_KC
180	1.60E-84	0.453137558	0.828	0.541	3.49E-80	LY96	1	C1QC_KC
181	2.08E-76	0.451644993	0.531	0.229	4.54E-72	SIGLEC10	1	C1QC_KC
182	5.25E-77	0.447649736	0.593	0.282	1.14E-72	RASSF4	1	C1QC_KC
183	1.40E-71	0.446772952	0.365	0.113	3.06E-67	ARRDC3	1	C1QC_KC
184	6.34E-94	0.446589557	0.45	0.136	1.38E-89	EVL	1	C1QC_KC
185	6.11E-91	0.4439465	0.22	0.013	1.33E-86	ITLN1	1	C1QC_KC
186	6.07E-53	0.440447115	0.871	0.684	1.32E-48	ZFP36L1	1	C1QC_KC
187	2.30E-109	0.43893345	0.277	0.023	5.02E-105	C2	1	C1QC_KC
188	1.70E-62	0.437100063	0.779	0.518	3.70E-58	RNASE6	1	C1QC_KC
189	1.96E-70	0.434363811	0.583	0.289	4.28E-66	NPL	1	C1QC_KC
190	7.12E-56	0.432137738	0.829	0.613	1.55E-51	HERPUD1	1	C1QC_KC
191	5.52E-84	0.429837834	0.424	0.134	1.20E-79	AP2A2	1	C1QC_KC
192	1.15E-78	0.42768655	0.876	0.677	2.50E-74	SARAF	1	C1QC_KC
193	3.11E-36	0.42757691	0.412	0.218	6.78E-32	GLA	1	C1QC_KC
194	5.99E-80	0.425584981	0.588	0.268	1.31E-75	PRCP	1	C1QC_KC
195	3.38E-113	0.423336023	0.265	0.015	7.37E-109	FPR3	1	C1QC_KC
196	1.53E-34	0.422860387	0.5	0.3	3.33E-30	EIF4A3	1	C1QC_KC
197	3.21E-29	0.422322718	0.673	0.505	7.01E-25	CD83	1	C1QC_KC
198	9.42E-74	0.421874988	0.872	0.637	2.05E-69	NPM1	1	C1QC_KC
199	6.31E-134	0.421843202	0.978	0.945	1.38E-129	HLA-C	1	C1QC_KC
200	1.58E-71	0.416557751	0.662	0.361	3.44E-67	DPP7	1	C1QC_KC
201	2.66E-41	0.41498191	0.791	0.608	5.81E-37	KLF4	1	C1QC_KC
202	1.31E-52	0.414888898	0.33	0.119	2.85E-48	ID1	1	C1QC_KC
203	9.61E-86	0.414723216	0.406	0.12	2.09E-81	FCHO2	1	C1QC_KC
204	3.84E-95	0.414723147	0.339	0.07	8.37E-91	SORBS3	1	C1QC_KC
205	2.56E-50	0.413616821	0.81	0.62	5.58E-46	IFNGR1	1	C1QC_KC
206	4.05E-37	0.409217346	0.903	0.837	8.84E-33	KLF6	1	C1QC_KC
207	6.59E-33	0.409107303	0.269	0.114	1.44E-28	TWISTNB	1	C1QC_KC
208	1.34E-70	0.40439635	0.718	0.418	2.93E-66	TMEM123	1	C1QC_KC
209	5.51E-96	0.403919271	0.288	0.039	1.20E-91	ADORA3	1	C1QC_KC
210	1.01E-46	0.402234664	0.103	0.002	2.19E-42	VCAM1	1	C1QC_KC
211	1.62E-69	0.400757596	0.502	0.218	3.52E-65	CISD2	1	C1QC_KC
212	4.10E-81	0.39969528	0.435	0.146	8.94E-77	SIGLEC7	1	C1QC_KC
213	2.97E-75	0.398516587	0.417	0.145	6.48E-71	DNPH1	1	C1QC_KC
214	2.25E-22	0.398324393	0.588	0.426	4.90E-18	CITED2	1	C1QC_KC
215	1.12E-68	0.393857015	0.476	0.192	2.44E-64	DAPK1	1	C1QC_KC
216	1.11E-48	0.392520293	0.248	0.072	2.42E-44	DNAJB4	1	C1QC_KC
217	3.26E-97	0.391796432	0.246	0.02	7.11E-93	ETV5	1	C1QC_KC
218	1.12E-82	0.391184291	0.996	0.989	2.44E-78	MT-ATP6	1	C1QC_KC
219	4.70E-96	0.391157749	0.999	0.982	1.02E-91	SAT1	1	C1QC_KC
220	1.96E-65	0.390552654	0.573	0.29	4.28E-61	GUSB	1	C1QC_KC
221	5.17E-87	0.38999079	0.274	0.041	1.13E-82	C1orf54	1	C1QC_KC
222	2.13E-54	0.387036078	0.785	0.571	4.64E-50	CD164	1	C1QC_KC
223	1.29E-51	0.385621885	0.775	0.518	2.81E-47	CALR	1	C1QC_KC

224	4.41E-63	0.385168851	0.749	0.461	9.61E-59	CHCHD10	1	C1QC_KC
225	3.86E-63	0.383919349	0.678	0.392	8.42E-59	ST13	1	C1QC_KC
226	4.39E-67	0.381325966	0.629	0.323	9.58E-63	MTSS1	1	C1QC_KC
227	1.30E-31	0.379294551	0.722	0.513	2.83E-27	MT-ND6	1	C1QC_KC
228	2.05E-84	0.379152247	0.325	0.071	4.48E-80	BEX4	1	C1QC_KC
229	6.32E-105	0.376188941	0.283	0.029	1.38E-100	ABCG1	1	C1QC_KC
230	7.76E-91	0.374292126	0.273	0.037	1.69E-86	MMD	1	C1QC_KC
231	2.17E-56	0.372626287	0.541	0.266	4.74E-52	FGD2	1	C1QC_KC
232	8.22E-37	0.370982867	0.531	0.313	1.79E-32	PELI1	1	C1QC_KC
233	2.61E-68	0.370945677	0.549	0.252	5.69E-64	DDAH2	1	C1QC_KC
234	3.41E-65	0.364605748	0.431	0.167	7.44E-61	GLMP	1	C1QC_KC
235	2.17E-76	0.363190019	0.276	0.053	4.73E-72	MS4A4E	1	C1QC_KC
236	6.92E-61	0.362984998	0.557	0.27	1.51E-56	CD300A	1	C1QC_KC
237	5.80E-86	0.362884344	0.247	0.029	1.26E-81	SPATS2L	1	C1QC_KC
238	1.44E-60	0.361756351	0.329	0.105	3.13E-56	P4HA1	1	C1QC_KC
239	1.61E-71	0.361448594	0.359	0.106	3.51E-67	SNX9	1	C1QC_KC
240	1.74E-55	0.359859199	0.616	0.344	3.78E-51	PABPC4	1	C1QC_KC
241	4.26E-56	0.359408697	0.633	0.367	9.30E-52	MAN2B1	1	C1QC_KC
242	1.76E-92	0.35927116	0.238	0.02	3.83E-88	SLC46A1	1	C1QC_KC
243	6.65E-68	0.35873544	0.185	0.017	1.45E-63	CETP	1	C1QC_KC
244	4.22E-67	0.357545836	0.354	0.112	9.21E-63	CREBL2	1	C1QC_KC
245	4.76E-47	0.355824659	0.617	0.372	1.04E-42	PDIA6	1	C1QC_KC
246	1.40E-56	0.354879977	0.715	0.441	3.05E-52	NDFIP1	1	C1QC_KC
247	6.72E-48	0.350812624	0.603	0.341	1.47E-43	SOD1	1	C1QC_KC
248	1.22E-45	0.350388947	0.64	0.383	2.67E-41	NABP1	1	C1QC_KC
249	2.09E-36	0.34988232	0.75	0.562	4.56E-32	TXNIP	1	C1QC_KC
250	5.37E-64	0.349625971	0.223	0.039	1.17E-59	FILIP1L	1	C1QC_KC
251	2.35E-79	0.34765564	0.237	0.03	5.12E-75	TNFRSF21	1	C1QC_KC
252	3.90E-99	0.347077614	0.236	0.013	8.50E-95	GATM	1	C1QC_KC
253	1.13E-100	0.344477008	0.223	0.007	2.47E-96	DHRS3	1	C1QC_KC
254	1.17E-30	0.341483605	0.769	0.579	2.55E-26	SQSTM1	1	C1QC_KC
255	2.43E-76	0.341130539	0.294	0.061	5.30E-72	NMRK1	1	C1QC_KC
256	5.08E-54	0.339928411	0.541	0.278	1.11E-49	RB1	1	C1QC_KC
257	2.50E-71	0.339504591	0.295	0.069	5.45E-67	AIG1	1	C1QC_KC
258	4.07E-51	0.33767136	0.422	0.186	8.88E-47	TCF7L2	1	C1QC_KC
259	7.64E-59	0.337034247	0.352	0.123	1.67E-54	SESN1	1	C1QC_KC
260	3.76E-46	0.336389327	0.909	0.747	8.19E-42	MAFB	1	C1QC_KC
261	2.98E-33	0.33171708	0.412	0.22	6.50E-29	CREM	1	C1QC_KC
262	7.29E-61	0.331708461	0.319	0.097	1.59E-56	PTMS	1	C1QC_KC
263	2.10E-23	0.331045792	0.248	0.123	4.59E-19	FKBP4	1	C1QC_KC
264	5.59E-46	0.33022974	0.791	0.574	1.22E-41	TMEM59	1	C1QC_KC
265	1.70E-49	0.329022296	0.712	0.461	3.71E-45	ARL6IP1	1	C1QC_KC
266	1.47E-44	0.328818722	0.75	0.506	3.21E-40	LCP2	1	C1QC_KC
267	4.27E-62	0.328715905	0.379	0.133	9.32E-58	TMC6	1	C1QC_KC
268	7.46E-79	0.328448291	0.267	0.045	1.63E-74	OLFML2B	1	C1QC_KC
269	4.57E-89	0.32788709	0.215	0.013	9.96E-85	SLC7A8	1	C1QC_KC
270	9.13E-63	0.326086666	0.292	0.078	1.99E-58	GCLC	1	C1QC_KC
271	2.75E-34	0.324882854	0.641	0.424	6.00E-30	CTSZ	1	C1QC_KC
272	1.11E-20	0.324316119	0.502	0.352	2.42E-16	ATF3	1	C1QC_KC
273	6.09E-53	0.323982904	0.215	0.047	1.33E-48	HES1	1	C1QC_KC
274	7.14E-46	0.321255391	0.226	0.064	1.56E-41	KMO	1	C1QC_KC
275	1.23E-58	0.321117223	0.564	0.277	2.69E-54	TMEM14C	1	C1QC_KC
276	3.23E-30	0.32101239	0.938	0.866	7.04E-26	UBC	1	C1QC_KC
277	6.33E-48	0.32099954	0.887	0.692	1.38E-43	VAMP8	1	C1QC_KC
278	1.33E-51	0.32094822	0.397	0.167	2.91E-47	GNPDA1	1	C1QC_KC
279	1.99E-44	0.319949529	0.48	0.243	4.34E-40	BHLHE40	1	C1QC_KC
280	1.45E-69	0.318119625	0.255	0.05	3.17E-65	CMKLR1	1	C1QC_KC
281	4.48E-55	0.316960207	0.997	0.991	9.76E-51	MT-ND3	1	C1QC_KC
282	8.36E-33	0.315871242	0.393	0.209	1.82E-28	DDIT3	1	C1QC_KC
283	6.81E-63	0.315569888	0.305	0.085	1.49E-58	PRKACB	1	C1QC_KC
284	1.16E-53	0.314482642	0.344	0.127	2.52E-49	PHACTR2	1	C1QC_KC
285	2.23E-54	0.314264365	0.476	0.216	4.86E-50	CNDP2	1	C1QC_KC
286	3.78E-105	0.31355882	0.999	1	8.25E-101	B2M	1	C1QC_KC
287	4.59E-81	0.313211808	0.192	0.009	1.00E-76	SUCNR1	1	C1QC_KC
288	2.55E-48	0.312708987	0.558	0.301	5.55E-44	AKR1A1	1	C1QC_KC
289	5.63E-49	0.312187023	0.456	0.216	1.23E-44	ABI3	1	C1QC_KC
290	1.01E-67	0.312138212	0.998	0.999	2.21E-63	MT-CO2	1	C1QC_KC
291	1.48E-51	0.311973386	0.62	0.346	3.22E-47	LAMP2	1	C1QC_KC
292	3.88E-36	0.31029874	0.984	0.938	8.47E-32	MT-ND5	1	C1QC_KC
293	1.70E-21	0.309019382	0.793	0.708	3.71E-17	CFD	1	C1QC_KC
294	7.48E-70	0.308227186	0.199	0.022	1.63E-65	KCNMA1	1	C1QC_KC
295	1.80E-43	0.305188352	0.739	0.495	3.93E-39	LGALS9	1	C1QC_KC
296	7.17E-34	0.304884952	0.93	0.867	1.56E-29	CTSD	1	C1QC_KC
297	2.35E-29	0.304576539	0.166	0.052	5.11E-25	EGR2	1	C1QC_KC
298	5.14E-40	0.302527612	0.767	0.535	1.12E-35	TBXAS1	1	C1QC_KC

299	6.18E-56	0.302212254	0.319	0.105	1.35E-51	TSPAN3	1	C1QC_KC
300	3.86E-43	0.30208809	0.534	0.295	8.41E-39	GYPC	1	C1QC_KC
301	1.09E-58	0.302004626	0.999	0.999	2.39E-54	MT-CO1	1	C1QC_KC
302	3.25E-70	0.300935223	0.226	0.034	7.09E-66	PLA2G15	1	C1QC_KC
303	2.90E-68	0.30025911	0.317	0.085	6.33E-64	CHID1	1	C1QC_KC
304	7.38E-42	0.299956557	0.528	0.297	1.61E-37	AOAH	1	C1QC_KC
305	2.03E-44	0.299804635	0.542	0.297	4.43E-40	SERPINB6	1	C1QC_KC
306	3.74E-38	0.298489315	0.642	0.403	8.16E-34	EIF4A2	1	C1QC_KC
307	5.64E-41	0.297805922	0.514	0.283	1.23E-36	CD86	1	C1QC_KC
308	3.76E-51	0.297191431	0.4	0.167	8.19E-47	RENBP	1	C1QC_KC
309	1.88E-30	0.296896586	0.796	0.642	4.10E-26	PRDX1	1	C1QC_KC
310	4.88E-56	0.296312617	0.277	0.079	1.06E-51	ME1	1	C1QC_KC
311	9.03E-41	0.295165776	0.544	0.302	1.97E-36	IRF8	1	C1QC_KC
312	1.34E-35	0.29513313	0.11	0.014	2.92E-31	TREM2	1	C1QC_KC
313	2.72E-48	0.294968904	0.368	0.151	5.92E-44	DEGS1	1	C1QC_KC
314	6.98E-30	0.294169597	0.425	0.239	1.52E-25	ELL2	1	C1QC_KC
315	6.93E-67	0.294104474	0.245	0.048	1.51E-62	SIGLEC11	1	C1QC_KC
316	8.86E-19	0.292917863	0.83	0.687	1.93E-14	MT2A	1	C1QC_KC
317	2.49E-40	0.292510152	0.537	0.305	5.44E-36	RPN2	1	C1QC_KC
318	1.48E-55	0.292284097	0.283	0.085	3.23E-51	GIMAP5	1	C1QC_KC
319	1.63E-48	0.292134533	0.906	0.716	3.56E-44	CD302	1	C1QC_KC
320	6.35E-37	0.292052374	0.621	0.397	1.39E-32	NUCB1	1	C1QC_KC
321	7.58E-66	0.292050151	0.235	0.043	1.65E-61	SHMT1	1	C1QC_KC
322	8.22E-47	0.291849215	0.305	0.112	1.79E-42	CEPT1	1	C1QC_KC
323	1.85E-67	0.290302219	0.27	0.058	4.03E-63	SLC46A3	1	C1QC_KC
324	1.53E-48	0.290258526	0.388	0.162	3.33E-44	FUOM	1	C1QC_KC
325	1.20E-72	0.289295045	0.986	0.962	2.61E-68	HLA-B	1	C1QC_KC
326	1.32E-41	0.289172315	0.709	0.485	2.89E-37	RNF13	1	C1QC_KC
327	9.23E-32	0.288852077	0.774	0.563	2.01E-27	PTGES3	1	C1QC_KC
328	4.20E-57	0.287879881	0.283	0.079	9.15E-53	SLC37A2	1	C1QC_KC
329	1.55E-46	0.287658464	0.426	0.194	3.38E-42	CYFIP1	1	C1QC_KC
330	8.07E-41	0.287164505	0.549	0.305	1.76E-36	FNBP1	1	C1QC_KC
331	1.44E-66	0.286699048	0.248	0.048	3.14E-62	AMDHD2	1	C1QC_KC
332	2.24E-17	0.285794389	0.251	0.142	4.89E-13	BATF	1	C1QC_KC
333	2.37E-42	0.285400695	0.624	0.376	5.18E-38	SMS	1	C1QC_KC
334	8.59E-39	0.28453766	0.532	0.301	1.87E-34	RNF141	1	C1QC_KC
335	1.86E-25	0.283309809	0.427	0.255	4.05E-21	IER5	1	C1QC_KC
336	1.41E-30	0.282693801	0.71	0.522	3.09E-26	PPT1	1	C1QC_KC
337	7.97E-51	0.282661929	0.952	0.858	1.74E-46	YBX1	1	C1QC_KC
338	1.64E-49	0.281093818	0.334	0.122	3.57E-45	FLVCR2	1	C1QC_KC
339	4.14E-43	0.280627269	0.376	0.165	9.04E-39	APH1B	1	C1QC_KC
340	9.90E-52	0.280463957	0.256	0.071	2.16E-47	SLC38A6	1	C1QC_KC
341	4.86E-42	0.280437635	0.274	0.098	1.06E-37	SLC8B1	1	C1QC_KC
342	3.64E-39	0.280324484	0.673	0.432	7.93E-35	ATP6AP1	1	C1QC_KC
343	1.45E-39	0.279136474	0.527	0.291	3.17E-35	RAB32	1	C1QC_KC
344	2.56E-27	0.278899826	0.991	0.971	5.57E-23	MT-ND2	1	C1QC_KC
345	8.24E-35	0.278661246	0.305	0.133	1.80E-30	MIR181A1HG	1	C1QC_KC
346	3.17E-54	0.278626296	0.349	0.127	6.90E-50	STARD3	1	C1QC_KC
347	1.60E-55	0.278431259	0.278	0.08	3.49E-51	CD180	1	C1QC_KC
348	3.78E-31	0.277745447	0.229	0.089	8.24E-27	CLEC10A	1	C1QC_KC
349	1.60E-43	0.277253732	0.268	0.093	3.49E-39	TPM1	1	C1QC_KC
350	1.11E-39	0.27693186	0.449	0.23	2.41E-35	SCIMP	1	C1QC_KC
351	1.24E-50	0.275820206	0.257	0.073	2.69E-46	CTNND1	1	C1QC_KC
352	2.53E-63	0.275817112	0.239	0.048	5.51E-59	PIK3IP1	1	C1QC_KC
353	2.14E-46	0.274817862	0.319	0.122	4.67E-42	PCBD1	1	C1QC_KC
354	5.36E-69	0.272190717	0.188	0.018	1.17E-64	HLA-DOA	1	C1QC_KC
355	3.33E-41	0.271875108	0.385	0.175	7.25E-37	GOLGA4	1	C1QC_KC
356	1.74E-47	0.271775229	0.376	0.156	3.79E-43	FEZ2	1	C1QC_KC
357	7.07E-37	0.271729977	0.462	0.251	1.54E-32	NANS	1	C1QC_KC
358	6.11E-44	0.271685991	0.279	0.097	1.33E-39	CH17-340M24.3	1	C1QC_KC
359	1.79E-38	0.271673846	0.471	0.253	3.90E-34	ADI1	1	C1QC_KC
360	2.17E-59	0.271574919	0.298	0.086	4.73E-55	ABHD12	1	C1QC_KC
361	2.61E-30	0.270474634	0.33	0.161	5.68E-26	ARID5A	1	C1QC_KC
362	4.15E-41	0.270158842	0.335	0.137	9.05E-37	PVRL2	1	C1QC_KC
363	3.75E-32	0.26959547	0.216	0.079	8.18E-28	ABL2	1	C1QC_KC
364	5.53E-21	0.269064576	0.495	0.324	1.21E-16	STAB1	1	C1QC_KC
365	2.50E-43	0.268647818	0.41	0.189	5.45E-39	EPB41L3	1	C1QC_KC
366	2.66E-33	0.267988912	0.332	0.156	5.80E-29	FAM46A	1	C1QC_KC
367	5.17E-76	0.267723484	0.178	0.007	1.13E-71	SCN1B	1	C1QC_KC
368	8.13E-58	0.267402159	0.221	0.044	1.77E-53	SLC29A1	1	C1QC_KC
369	2.11E-38	0.267359852	0.573	0.334	4.59E-34	LILRB4	1	C1QC_KC
370	1.19E-26	0.266391666	0.522	0.328	2.59E-22	SRSF7	1	C1QC_KC
371	4.96E-50	0.26579086	0.997	1	1.08E-45	MT-CO3	1	C1QC_KC
372	1.56E-48	0.264815524	0.273	0.086	3.40E-44	ENG	1	C1QC_KC
373	2.31E-45	0.264595243	0.263	0.085	5.03E-41	SLC4A7	1	C1QC_KC

374	2.90E-30	0.26458585	0.577	0.365	6.32E-26	TNFAIP2	1	C1QC_KC
375	3.27E-42	0.263950811	0.283	0.104	7.13E-38	HERC1	1	C1QC_KC
376	8.90E-27	0.263912012	0.598	0.389	1.94E-22	GNAS	1	C1QC_KC
377	3.18E-26	0.263067524	0.504	0.321	6.93E-22	ZCCHC6	1	C1QC_KC
378	1.83E-14	0.262889512	0.615	0.479	3.99E-10	DNAJB6	1	C1QC_KC
379	8.13E-43	0.262613368	0.369	0.159	1.77E-38	ATM	1	C1QC_KC
380	2.00E-35	0.262157163	0.544	0.326	4.36E-31	BLVRA	1	C1QC_KC
381	5.91E-75	0.262026458	0.184	0.011	1.29E-70	CNRIP1	1	C1QC_KC
382	1.34E-14	0.261483846	0.297	0.185	2.93E-10	LMNA	1	C1QC_KC
383	3.10E-12	0.260935198	0.699	0.572	6.75E-08	DNAJA1	1	C1QC_KC
384	3.34E-15	0.260601007	0.669	0.51	7.28E-11	PLIN2	1	C1QC_KC
385	1.84E-49	0.260544268	0.213	0.05	4.01E-45	SLC23A2	1	C1QC_KC
386	1.67E-53	0.259760347	0.141	0.011	3.64E-49	RP11-1080G15.1	1	C1QC_KC
387	5.25E-39	0.259322538	0.297	0.119	1.15E-34	SESN3	1	C1QC_KC
388	2.42E-62	0.258537247	0.184	0.022	5.28E-58	PKD2L1	1	C1QC_KC
389	2.89E-42	0.257840021	0.289	0.108	6.30E-38	GSTM1	1	C1QC_KC
390	6.47E-34	0.257564513	0.277	0.116	1.41E-29	LPAR6	1	C1QC_KC
391	8.36E-24	0.256695244	0.283	0.147	1.82E-19	CCRL2	1	C1QC_KC
392	1.30E-65	0.256330554	0.175	0.015	2.83E-61	ITGA9	1	C1QC_KC
393	3.44E-29	0.256234321	0.505	0.31	7.50E-25	SNAP23	1	C1QC_KC
394	1.58E-67	0.256094954	0.168	0.011	3.45E-63	RHOBTB1	1	C1QC_KC
395	5.09E-32	0.255038096	0.687	0.465	1.11E-27	TUBB	1	C1QC_KC
396	1.13E-34	0.254924994	0.719	0.497	2.46E-30	RAB5C	1	C1QC_KC
397	1.65E-51	0.254604153	0.15	0.017	3.60E-47	CCL24	1	C1QC_KC
398	1.33E-44	0.254543154	0.381	0.163	2.89E-40	GNB4	1	C1QC_KC
399	8.83E-41	0.254360609	0.257	0.09	1.93E-36	PDE4DIP	1	C1QC_KC
400	5.29E-43	0.254049574	0.377	0.161	1.15E-38	FNIP2	1	C1QC_KC
401	2.19E-20	0.253409824	0.71	0.526	4.79E-16	DDIT4	1	C1QC_KC
402	3.05E-37	0.250869795	0.614	0.374	6.65E-33	HNMT	1	C1QC_KC
403	2.13E-53	0.250320718	0.197	0.037	4.65E-49	P2RY12	1	C1QC_KC
404	4.72E-33	0.250088042	0.606	0.381	1.03E-28	IL10RA	1	C1QC_KC
405	9.29E-08	-0.250060997	0.456	0.474	0.00202553	RBM8A	1	C1QC_KC
406	3.37E-13	-0.250577153	0.163	0.248	7.35E-09	CARD19	1	C1QC_KC
407	2.59E-29	-0.251022464	0.859	0.849	5.65E-25	PTPRC	1	C1QC_KC
408	1.28E-10	-0.251085178	0.495	0.532	2.80E-06	IRAK3	1	C1QC_KC
409	1.70E-17	-0.251176537	0.791	0.793	3.71E-13	DAZAP2	1	C1QC_KC
410	6.39E-08	-0.252555262	0.212	0.265	0.001394558	QSOX1	1	C1QC_KC
411	1.39E-14	-0.253404551	0.708	0.696	3.02E-10	PSMA7	1	C1QC_KC
412	4.31E-66	-0.253994969	0.975	0.982	9.40E-62	RPL35A	1	C1QC_KC
413	3.24E-24	-0.254085479	0.808	0.806	7.07E-20	YWHAZ	1	C1QC_KC
414	2.77E-08	-0.256015117	0.527	0.549	0.000604395	DDX21	1	C1QC_KC
415	9.59E-60	-0.256214287	0.981	0.986	2.09E-55	SERF2	1	C1QC_KC
416	7.16E-16	-0.25696199	0.146	0.241	1.56E-11	DIAPH1	1	C1QC_KC
417	4.04E-22	-0.257082394	0.757	0.806	8.80E-18	C5AR1	1	C1QC_KC
418	1.40E-74	-0.257557688	0.991	0.993	3.05E-70	RPS3A	1	C1QC_KC
419	1.03E-09	-0.257611212	0.424	0.465	2.24E-05	CYTIP	1	C1QC_KC
420	2.71E-15	-0.258181165	0.748	0.724	5.91E-11	CDC42	1	C1QC_KC
421	6.71E-19	-0.258887397	0.679	0.709	1.46E-14	LRRFIP1	1	C1QC_KC
422	4.67E-85	-0.259083311	0.995	0.998	1.02E-80	RPL11	1	C1QC_KC
423	9.09E-09	-0.259283555	0.445	0.471	0.000198149	PSMA1	1	C1QC_KC
424	3.86E-18	-0.259412996	0.728	0.734	8.42E-14	RHOG	1	C1QC_KC
425	2.05E-10	-0.259689076	0.559	0.564	4.48E-06	C7orf73	1	C1QC_KC
426	7.93E-14	-0.259979725	0.206	0.29	1.73E-09	CCDC109B	1	C1QC_KC
427	1.90E-10	-0.260079868	0.343	0.419	4.14E-06	TMEM176A	1	C1QC_KC
428	2.83E-08	-0.260482441	0.236	0.292	0.000616987	PTK2B	1	C1QC_KC
429	1.40E-16	-0.260703455	0.776	0.766	3.06E-12	H2AFZ	1	C1QC_KC
430	1.31E-10	-0.260784171	0.524	0.548	2.86E-06	PET100	1	C1QC_KC
431	2.02E-10	-0.261010259	0.119	0.186	4.41E-06	TLE3	1	C1QC_KC
432	2.34E-07	-0.261633684	0.277	0.322	0.00510896	SHOC2	1	C1QC_KC
433	1.60E-81	-0.261722545	0.993	0.995	3.49E-77	RPS15A	1	C1QC_KC
434	1.03E-22	-0.26190969	0.789	0.794	2.24E-18	UBL5	1	C1QC_KC
435	2.12E-11	-0.262148685	0.224	0.298	4.63E-07	ACTN4	1	C1QC_KC
436	4.35E-13	-0.262459548	0.103	0.181	9.50E-09	TMEM170B	1	C1QC_KC
437	2.35E-24	-0.263364306	0.832	0.822	5.12E-20	MYL12B	1	C1QC_KC
438	3.09E-65	-0.264188226	0.968	0.97	6.74E-61	RPS4X	1	C1QC_KC
439	3.36E-11	-0.265600601	0.49	0.533	7.32E-07	STK17B	1	C1QC_KC
440	4.21E-07	-0.265602343	0.281	0.323	0.009185303	FAM198B	1	C1QC_KC
441	8.23E-19	-0.266495535	0.742	0.741	1.79E-14	COX7A2	1	C1QC_KC
442	2.63E-13	-0.266718331	0.266	0.346	5.73E-09	SHKBP1	1	C1QC_KC
443	1.02E-113	-0.267606377	0.995	0.998	2.22E-109	RPL28	1	C1QC_KC
444	1.40E-11	-0.267900167	0.51	0.541	3.05E-07	SRSF2	1	C1QC_KC
445	1.63E-25	-0.268888928	0.813	0.783	3.55E-21	HNRNPA1	1	C1QC_KC
446	2.11E-07	-0.269166606	0.481	0.498	0.004590823	COX17	1	C1QC_KC
447	3.22E-78	-0.27044697	0.996	0.996	7.02E-74	RPS13	1	C1QC_KC
448	1.43E-10	-0.271344219	0.24	0.307	3.12E-06	RARA	1	C1QC_KC

449	1.13E-15	-0.272145985	0.11	0.197	2.47E-11	MSRB1	1	C1QC_KC
450	1.46E-13	-0.272362544	0.51	0.552	3.18E-09	RBX1	1	C1QC_KC
451	3.73E-15	-0.273351613	0.132	0.22	8.13E-11	PXN	1	C1QC_KC
452	4.67E-13	-0.274742288	0.612	0.626	1.02E-08	AHNAK	1	C1QC_KC
453	1.18E-07	-0.275517486	0.132	0.184	0.002571355	IL1RAP	1	C1QC_KC
454	1.61E-09	-0.275936432	0.297	0.355	3.50E-05	CLEC4A	1	C1QC_KC
455	9.05E-17	-0.276187061	0.389	0.489	1.97E-12	MXD1	1	C1QC_KC
456	1.37E-71	-0.276292343	0.965	0.979	2.99E-67	RPS3	1	C1QC_KC
457	1.35E-10	-0.276487524	0.309	0.37	2.94E-06	PECAM1	1	C1QC_KC
458	1.43E-23	-0.276560705	0.749	0.755	3.11E-19	PRR13	1	C1QC_KC
459	5.42E-09	-0.277134473	0.207	0.273	0.000118237	ZC3H12A	1	C1QC_KC
460	3.71E-17	-0.28031448	0.69	0.688	8.08E-13	ACTR2	1	C1QC_KC
461	1.38E-11	-0.280482988	0.157	0.231	3.02E-07	PLXNC1	1	C1QC_KC
462	9.03E-15	-0.281531815	0.597	0.612	1.97E-10	C4orf3	1	C1QC_KC
463	3.52E-18	-0.281535171	0.766	0.754	7.68E-14	AP1S2	1	C1QC_KC
464	1.76E-09	-0.281902697	0.331	0.388	3.84E-05	HACD4	1	C1QC_KC
465	6.88E-23	-0.282104899	0.827	0.824	1.50E-18	TKT	1	C1QC_KC
466	6.35E-11	-0.282346332	0.28	0.345	1.39E-06	FLOT1	1	C1QC_KC
467	1.13E-13	-0.282938327	0.26	0.341	2.46E-09	PLIN3	1	C1QC_KC
468	1.94E-45	-0.283230414	0.932	0.925	4.24E-41	BTF3	1	C1QC_KC
469	6.04E-14	-0.283637678	0.27	0.352	1.32E-09	LAT2	1	C1QC_KC
470	1.44E-107	-0.284127907	0.993	0.995	3.15E-103	RPL32	1	C1QC_KC
471	1.13E-20	-0.285031381	0.679	0.735	2.46E-16	RGS2	1	C1QC_KC
472	1.86E-13	-0.2851197	0.436	0.489	4.05E-09	PPP4C	1	C1QC_KC
473	2.22E-14	-0.285126443	0.547	0.571	4.84E-10	COX7A2L	1	C1QC_KC
474	6.89E-15	-0.28615512	0.383	0.465	1.50E-10	LINC00936	1	C1QC_KC
475	9.15E-09	-0.286639246	0.305	0.353	0.000199464	CHIC2	1	C1QC_KC
476	3.73E-73	-0.286793398	0.979	0.979	8.14E-69	RPL6	1	C1QC_KC
477	1.82E-12	-0.287002557	0.412	0.463	3.96E-08	RNF7	1	C1QC_KC
478	1.06E-32	-0.287321362	0.806	0.805	2.32E-28	FKBP1A	1	C1QC_KC
479	7.48E-35	-0.287731808	0.855	0.841	1.63E-30	TMA7	1	C1QC_KC
480	3.24E-18	-0.288084319	0.133	0.241	7.07E-14	CREB5	1	C1QC_KC
481	4.55E-39	-0.288346524	0.855	0.855	9.92E-35	ARHGDIB	1	C1QC_KC
482	8.06E-12	-0.288798328	0.557	0.565	1.76E-07	MSN	1	C1QC_KC
483	8.48E-69	-0.288802279	0.981	0.989	1.85E-64	ATP5E	1	C1QC_KC
484	2.20E-13	-0.290936029	0.785	0.749	4.79E-09	TAGLN2	1	C1QC_KC
485	1.55E-14	-0.293240127	0.151	0.24	3.39E-10	R3HDM4	1	C1QC_KC
486	1.26E-09	-0.294988919	0.307	0.361	2.74E-05	USP3	1	C1QC_KC
487	7.88E-08	-0.296087113	0.407	0.43	0.001718329	NUP214	1	C1QC_KC
488	1.55E-18	-0.296127389	0.122	0.224	3.37E-14	HEBP2	1	C1QC_KC
489	4.50E-17	-0.296363434	0.118	0.212	9.82E-13	ZNF281	1	C1QC_KC
490	4.11E-08	-0.296604031	0.239	0.293	0.000897137	MIR4435-2HG	1	C1QC_KC
491	1.21E-12	-0.297180137	0.383	0.443	2.65E-08	CKLF	1	C1QC_KC
492	5.01E-112	-0.297786082	0.996	0.997	1.09E-107	RPLP1	1	C1QC_KC
493	1.83E-88	-0.29879881	0.974	0.985	4.00E-84	RPL8	1	C1QC_KC
494	8.02E-11	-0.298875356	0.18	0.251	1.75E-06	RAB11FIP1	1	C1QC_KC
495	2.03E-13	-0.300046067	0.718	0.68	4.42E-09	CD99	1	C1QC_KC
496	7.03E-17	-0.300110695	0.249	0.351	1.53E-12	JAML	1	C1QC_KC
497	1.28E-30	-0.300156958	0.815	0.798	2.80E-26	SPI1	1	C1QC_KC
498	4.71E-13	-0.30063495	0.392	0.451	1.03E-08	EFHD2	1	C1QC_KC
499	2.32E-13	-0.300840147	0.202	0.283	5.06E-09	TMPO	1	C1QC_KC
500	4.60E-13	-0.301300413	0.306	0.378	1.00E-08	ARPC4	1	C1QC_KC
501	1.17E-43	-0.301517018	0.907	0.902	2.56E-39	RPS26	1	C1QC_KC
502	3.04E-14	-0.302003147	0.117	0.199	6.64E-10	ANXA6	1	C1QC_KC
503	1.65E-10	-0.302061475	0.483	0.511	3.59E-06	CSF3R	1	C1QC_KC
504	8.17E-14	-0.302712562	0.18	0.262	1.78E-09	SLC36A4	1	C1QC_KC
505	2.68E-88	-0.303105414	0.984	0.991	5.84E-84	RPS7	1	C1QC_KC
506	2.12E-41	-0.30403891	0.862	0.877	4.61E-37	GNG5	1	C1QC_KC
507	9.51E-49	-0.304045387	0.88	0.892	2.07E-44	ARPC2	1	C1QC_KC
508	9.65E-15	-0.304685016	0.409	0.483	2.10E-10	TNFSF13B	1	C1QC_KC
509	1.77E-20	-0.305033467	0.66	0.687	3.86E-16	POLR2L	1	C1QC_KC
510	4.86E-16	-0.305458948	0.618	0.625	1.06E-11	GNAI2	1	C1QC_KC
511	2.14E-60	-0.305732413	0.939	0.942	4.67E-56	RPL7	1	C1QC_KC
512	8.87E-20	-0.306019622	0.58	0.614	1.93E-15	PSMB3	1	C1QC_KC
513	5.88E-10	-0.309600844	0.532	0.591	1.28E-05	CXCL8	1	C1QC_KC
514	4.64E-13	-0.310034259	0.245	0.325	1.01E-08	ZDHHC20	1	C1QC_KC
515	3.51E-11	-0.310286399	0.495	0.518	7.66E-07	SFPQ	1	C1QC_KC
516	1.27E-14	-0.310317511	0.314	0.39	2.77E-10	NUMB	1	C1QC_KC
517	2.39E-15	-0.311014968	0.16	0.255	5.22E-11	NFIL3	1	C1QC_KC
518	3.44E-25	-0.31103716	0.712	0.73	7.50E-21	CAP1	1	C1QC_KC
519	1.76E-13	-0.31211136	0.355	0.42	3.83E-09	MYH9	1	C1QC_KC
520	6.27E-16	-0.31231499	0.627	0.639	1.37E-11	NDUFB1	1	C1QC_KC
521	3.50E-16	-0.31307431	0.256	0.345	7.64E-12	POLE4	1	C1QC_KC
522	1.74E-33	-0.313291679	0.776	0.797	3.79E-29	COMMD6	1	C1QC_KC
523	2.76E-127	-0.316327042	0.992	0.998	6.02E-123	RPL34	1	C1QC_KC

524	8.19E-38	-0.316754198	0.796	0.824	1.79E-33	TPI1	1	C1QC_KC
525	4.28E-25	-0.318706782	0.674	0.692	9.33E-21	PRELID1	1	C1QC_KC
526	7.43E-14	-0.319679214	0.126	0.211	1.62E-09	ANKRD28	1	C1QC_KC
527	5.88E-27	-0.321666358	0.83	0.875	1.28E-22	NAMPT	1	C1QC_KC
528	5.47E-24	-0.323748427	0.181	0.304	1.19E-19	SASH3	1	C1QC_KC
529	4.01E-14	-0.323791619	0.292	0.375	8.74E-10	SLC25A37	1	C1QC_KC
530	2.21E-27	-0.324178352	0.712	0.775	4.82E-23	SAMSN1	1	C1QC_KC
531	4.93E-19	-0.324752781	0.885	0.878	1.08E-14	TIMP1	1	C1QC_KC
532	9.79E-14	-0.325104702	0.167	0.251	2.13E-09	PHC2	1	C1QC_KC
533	4.06E-31	-0.325594587	0.71	0.745	8.86E-27	SEC61B	1	C1QC_KC
534	3.92E-52	-0.327211205	0.874	0.897	8.56E-48	ARPC1B	1	C1QC_KC
535	3.01E-28	-0.329524643	0.671	0.73	6.57E-24	HCST	1	C1QC_KC
536	2.86E-19	-0.330040892	0.468	0.534	6.23E-15	MTPN	1	C1QC_KC
537	3.46E-36	-0.331241906	0.79	0.81	7.54E-32	COX8A	1	C1QC_KC
538	8.83E-32	-0.33423141	0.81	0.818	1.93E-27	ZEB2	1	C1QC_KC
539	1.67E-35	-0.337641969	0.755	0.77	3.64E-31	ZYX	1	C1QC_KC
540	7.49E-81	-0.341511892	0.967	0.977	1.63E-76	NACA	1	C1QC_KC
541	1.22E-37	-0.341640257	0.822	0.839	2.66E-33	BTG1	1	C1QC_KC
542	2.03E-16	-0.341753855	0.336	0.42	4.43E-12	GNAQ	1	C1QC_KC
543	1.88E-135	-0.343533456	0.996	0.996	4.10E-131	RPS8	1	C1QC_KC
544	1.21E-23	-0.344305751	0.437	0.545	2.63E-19	TMEM176B	1	C1QC_KC
545	2.37E-20	-0.349042007	0.524	0.579	5.17E-16	ROMO1	1	C1QC_KC
546	8.40E-23	-0.351508774	0.178	0.296	1.83E-18	GAS7	1	C1QC_KC
547	4.73E-20	-0.35151273	0.525	0.59	1.03E-15	UPP1	1	C1QC_KC
548	9.03E-44	-0.35222474	0.828	0.847	1.97E-39	LCP1	1	C1QC_KC
549	1.92E-119	-0.352873477	0.974	0.978	4.19E-115	OAZ1	1	C1QC_KC
550	1.23E-14	-0.354604944	0.338	0.407	2.69E-10	GYG1	1	C1QC_KC
551	4.30E-133	-0.355724283	0.989	0.994	9.38E-129	FAU	1	C1QC_KC
552	2.80E-19	-0.356652612	0.225	0.337	6.11E-15	PTGER2	1	C1QC_KC
553	3.63E-21	-0.357960282	0.155	0.265	7.92E-17	HRH2	1	C1QC_KC
554	8.42E-20	-0.358925295	0.424	0.5	1.84E-15	PTP4A2	1	C1QC_KC
555	1.68E-18	-0.359754009	0.385	0.461	3.67E-14	FERMT3	1	C1QC_KC
556	3.19E-31	-0.360817746	0.7	0.748	6.95E-27	PGK1	1	C1QC_KC
557	5.74E-26	-0.361420354	0.517	0.597	1.25E-21	TRAPPC1	1	C1QC_KC
558	1.59E-19	-0.361490185	0.246	0.355	3.46E-15	PLEC	1	C1QC_KC
559	1.09E-23	-0.362494113	0.253	0.371	2.37E-19	IL17RA	1	C1QC_KC
560	1.21E-19	-0.362586962	0.492	0.545	2.65E-15	ROCK1	1	C1QC_KC
561	5.40E-27	-0.363327987	0.695	0.741	1.18E-22	IFI30	1	C1QC_KC
562	1.01E-09	-0.364908017	0.17	0.096	2.21E-05	MT1G	1	C1QC_KC
563	9.63E-174	-0.366650143	0.994	0.996	2.10E-169	RPS24	1	C1QC_KC
564	8.72E-24	-0.367865484	0.228	0.347	1.90E-19	G6PD	1	C1QC_KC
565	3.72E-19	-0.367942145	0.283	0.386	8.12E-15	SH3KBP1	1	C1QC_KC
566	1.54E-24	-0.368495753	0.205	0.33	3.35E-20	RASSF2	1	C1QC_KC
567	3.84E-17	-0.37027772	0.458	0.517	8.37E-13	CNIH4	1	C1QC_KC
568	5.62E-71	-0.377059258	0.925	0.949	1.23E-66	EEF1D	1	C1QC_KC
569	7.45E-27	-0.378566378	0.227	0.359	1.63E-22	FMNL1	1	C1QC_KC
570	2.32E-28	-0.381460695	0.167	0.305	5.06E-24	FAM200B	1	C1QC_KC
571	4.41E-39	-0.385944096	0.171	0.345	9.63E-35	CAPN2	1	C1QC_KC
572	1.08E-27	-0.389046265	0.466	0.554	2.36E-23	C20orf24	1	C1QC_KC
573	7.29E-23	-0.393482196	0.326	0.427	1.59E-18	LSM6	1	C1QC_KC
574	4.73E-29	-0.397497998	0.209	0.349	1.03E-24	ASGR1	1	C1QC_KC
575	2.70E-25	-0.401079314	0.228	0.357	5.89E-21	CSGALNACT2	1	C1QC_KC
576	2.45E-36	-0.403011106	0.652	0.74	5.34E-32	CD55	1	C1QC_KC
577	3.12E-59	-0.409636945	0.809	0.846	6.81E-55	C14orf2	1	C1QC_KC
578	2.57E-24	-0.409704273	0.52	0.613	5.59E-20	ACSL1	1	C1QC_KC
579	3.14E-40	-0.409820668	0.599	0.683	6.84E-36	CAST	1	C1QC_KC
580	7.20E-25	-0.411129915	0.181	0.312	1.57E-20	CD36	1	C1QC_KC
581	3.36E-13	-0.411866231	0.397	0.445	7.34E-09	PAG1	1	C1QC_KC
582	1.40E-58	-0.412906271	0.808	0.855	3.04E-54	TCEB2	1	C1QC_KC
583	9.98E-40	-0.413063071	0.698	0.739	2.18E-35	SEC61G	1	C1QC_KC
584	7.88E-29	-0.413225174	0.107	0.238	1.72E-24	CPD	1	C1QC_KC
585	4.11E-23	-0.413322314	0.199	0.317	8.97E-19	UBR4	1	C1QC_KC
586	4.16E-51	-0.41640492	0.752	0.775	9.08E-47	RBM3	1	C1QC_KC
587	4.66E-47	-0.417284751	0.726	0.779	1.02E-42	SUB1	1	C1QC_KC
588	5.11E-32	-0.418155964	0.522	0.626	1.12E-27	RILPL2	1	C1QC_KC
589	2.00E-54	-0.420242562	0.806	0.825	4.37E-50	TPM3	1	C1QC_KC
590	2.07E-102	-0.422301258	0.977	0.994	4.50E-98	VIM	1	C1QC_KC
591	2.16E-24	-0.427788855	0.37	0.472	4.71E-20	IRS2	1	C1QC_KC
592	3.52E-30	-0.432770199	0.348	0.481	7.69E-26	ITGAM	1	C1QC_KC
593	3.25E-27	-0.438745559	0.395	0.493	7.08E-23	CCND3	1	C1QC_KC
594	3.08E-69	-0.442004126	0.838	0.89	6.72E-65	MYL12A	1	C1QC_KC
595	1.93E-67	-0.443671035	0.821	0.858	4.20E-63	FXYD5	1	C1QC_KC
596	1.29E-38	-0.445115751	0.518	0.608	2.80E-34	CD48	1	C1QC_KC
597	1.81E-46	-0.445190907	0.121	0.305	3.94E-42	PSTPIP1	1	C1QC_KC
598	1.92E-32	-0.446815984	0.185	0.336	4.19E-28	LRRK2	1	C1QC_KC

599	4.65E-32	-0.448685346	0.217	0.365	1.01E-27	LYST	1	C1QC_KC
600	1.15E-36	-0.450574863	0.503	0.609	2.50E-32	UBE2D1	1	C1QC_KC
601	1.07E-54	-0.458363904	0.701	0.77	2.33E-50	GMFG	1	C1QC_KC
602	1.05E-39	-0.458497684	0.278	0.433	2.28E-35	PNPLA2	1	C1QC_KC
603	3.83E-181	-0.462774049	0.997	0.997	8.36E-177	RPL39	1	C1QC_KC
604	2.01E-29	-0.463991473	0.231	0.37	4.39E-25	RNASE2	1	C1QC_KC
605	2.45E-51	-0.464132066	0.121	0.317	5.33E-47	CCDC69	1	C1QC_KC
606	2.14E-40	-0.465966138	0.385	0.553	4.66E-36	MYADM	1	C1QC_KC
607	4.94E-44	-0.469419214	0.57	0.663	1.08E-39	NDUFB9	1	C1QC_KC
608	1.43E-52	-0.48408316	0.583	0.713	3.12E-48	TYMP	1	C1QC_KC
609	3.35E-31	-0.484105963	0.158	0.303	7.31E-27	ASPH	1	C1QC_KC
610	4.05E-45	-0.485669323	0.431	0.572	8.83E-41	NCF2	1	C1QC_KC
611	7.75E-43	-0.488718548	0.488	0.595	1.69E-38	PGAM1	1	C1QC_KC
612	2.69E-43	-0.490800372	0.483	0.596	5.88E-39	RAC2	1	C1QC_KC
613	8.78E-56	-0.491610783	0.692	0.746	1.92E-51	ENO1	1	C1QC_KC
614	1.21E-57	-0.493214617	0.124	0.341	2.63E-53	ANPEP	1	C1QC_KC
615	5.30E-30	-0.493490953	0.279	0.426	1.16E-25	NLRP3	1	C1QC_KC
616	5.31E-25	-0.498588386	0.617	0.663	1.16E-20	CD52	1	C1QC_KC
617	5.76E-51	-0.49993809	0.654	0.727	1.26E-46	APLP2	1	C1QC_KC
618	3.54E-40	-0.504337608	0.457	0.579	7.72E-36	TMEM167A	1	C1QC_KC
619	6.59E-43	-0.505719351	0.219	0.398	1.44E-38	CDC42EP3	1	C1QC_KC
620	6.51E-94	-0.507767841	0.798	0.889	1.42E-89	EMP3	1	C1QC_KC
621	2.06E-36	-0.510795445	0.449	0.646	4.49E-32	AREG	1	C1QC_KC
622	2.08E-26	-0.51338803	0.297	0.419	4.53E-22	NCF1	1	C1QC_KC
623	8.68E-29	-0.518508875	0.299	0.422	1.89E-24	AGFG1	1	C1QC_KC
624	8.14E-53	-0.524933178	0.244	0.436	1.78E-48	C19orf38	1	C1QC_KC
625	2.46E-29	-0.530802469	0.137	0.275	5.37E-25	NKG7	1	C1QC_KC
626	8.92E-53	-0.532075187	0.503	0.638	1.95E-48	AGTRAP	1	C1QC_KC
627	6.05E-31	-0.536041417	0.133	0.276	1.32E-26	CYP1B1	1	C1QC_KC
628	1.30E-204	-0.545847261	0.964	0.978	2.84E-200	PFN1	1	C1QC_KC
629	1.25E-48	-0.55090789	0.174	0.374	2.74E-44	TREM1	1	C1QC_KC
630	5.40E-58	-0.551995052	0.405	0.6	1.18E-53	CLEC4E	1	C1QC_KC
631	3.35E-49	-0.552808884	0.362	0.528	7.32E-45	FBXL5	1	C1QC_KC
632	5.57E-127	-0.552819539	0.852	0.914	1.21E-122	CLIC1	1	C1QC_KC
633	3.52E-67	-0.555029933	0.639	0.73	7.67E-63	ANXA2	1	C1QC_KC
634	3.35E-150	-0.55526876	0.983	0.996	7.30E-146	SRGN	1	C1QC_KC
635	7.16E-69	-0.560606983	0.417	0.621	1.56E-64	FLNA	1	C1QC_KC
636	2.82E-189	-0.563688303	0.972	0.987	6.16E-185	S100A11	1	C1QC_KC
637	3.43E-71	-0.566579658	0.225	0.475	7.48E-67	MYO1G	1	C1QC_KC
638	9.64E-217	-0.566867491	0.972	0.991	2.10E-212	H3F3A	1	C1QC_KC
639	2.32E-68	-0.578574448	0.588	0.728	5.06E-64	SLC11A1	1	C1QC_KC
640	4.63E-165	-0.579610374	0.893	0.946	1.01E-160	ALDOA	1	C1QC_KC
641	5.41E-50	-0.589184291	0.383	0.537	1.18E-45	METTL9	1	C1QC_KC
642	5.61E-45	-0.594187177	0.442	0.583	1.22E-40	HMGB2	1	C1QC_KC
643	1.21E-111	-0.596826602	0.614	0.846	2.64E-107	ANXA1	1	C1QC_KC
644	1.67E-196	-0.608739259	0.933	0.961	3.63E-192	CFL1	1	C1QC_KC
645	1.09E-70	-0.62740042	0.476	0.662	2.38E-66	PTPRE	1	C1QC_KC
646	2.17E-49	-0.627663765	0.422	0.574	4.73E-45	TPM4	1	C1QC_KC
647	2.45E-103	-0.635125354	0.759	0.888	5.33E-99	SERPINB1	1	C1QC_KC
648	1.46E-54	-0.646094033	0.148	0.353	3.19E-50	FPR2	1	C1QC_KC
649	7.95E-98	-0.651826389	0.575	0.765	1.73E-93	CD37	1	C1QC_KC
650	1.12E-72	-0.657363779	0.625	0.798	2.45E-68	PLAUR	1	C1QC_KC
651	7.07E-94	-0.659869416	0.618	0.756	1.54E-89	TALDO1	1	C1QC_KC
652	1.37E-199	-0.661060758	0.939	0.982	2.98E-195	ACTG1	1	C1QC_KC
653	3.12E-45	-0.665565132	0.838	0.821	6.80E-41	IFITM3	1	C1QC_KC
654	2.81E-130	-0.679992848	0.764	0.864	6.14E-126	SERPINA1	1	C1QC_KC
655	8.01E-214	-0.682982814	0.898	0.964	1.75E-209	COTL1	1	C1QC_KC
656	1.80E-83	-0.692664631	0.101	0.369	3.93E-79	CD300E	1	C1QC_KC
657	1.48E-105	-0.693476158	0.517	0.71	3.24E-101	FGR	1	C1QC_KC
658	1.28E-82	-0.702186921	0.55	0.726	2.79E-78	FPR1	1	C1QC_KC
659	1.78E-88	-0.707349222	0.595	0.805	3.87E-84	BCL2A1	1	C1QC_KC
660	6.83E-235	-0.710145516	0.943	0.98	1.49E-230	MYL6	1	C1QC_KC
661	3.10E-79	-0.719525897	0.365	0.622	6.75E-75	SLC2A3	1	C1QC_KC
662	3.30E-92	-0.734156247	0.427	0.641	7.20E-88	LTA4H	1	C1QC_KC
663	5.87E-70	-0.741164992	0.137	0.377	1.28E-65	RP11-1143G9.4	1	C1QC_KC
664	5.88E-152	-0.745550883	0.666	0.825	1.28E-147	CORO1A	1	C1QC_KC
665	5.05E-101	-0.747383045	0.561	0.734	1.10E-96	PGD	1	C1QC_KC
666	7.13E-98	-0.754943471	0.448	0.684	1.55E-93	LILRA5	1	C1QC_KC
667	2.80E-109	-0.757204222	0.376	0.655	6.12E-105	LSP1	1	C1QC_KC
668	6.99E-132	-0.776772123	0.625	0.79	1.52E-127	PKM	1	C1QC_KC
669	0	-0.798029378	0.996	1	0	ACTB	1	C1QC_KC
670	3.64E-29	-0.805962477	0.259	0.387	7.94E-25	IL1R2	1	C1QC_KC
671	6.26E-69	-0.825142748	0.202	0.461	1.37E-64	EREG	1	C1QC_KC
672	8.56E-135	-0.844038144	0.214	0.569	1.87E-130	GLIPR2	1	C1QC_KC
673	1.04E-117	-0.860402873	0.215	0.548	2.26E-113	FAM65B	1	C1QC_KC

674	1.54E-143	-0.885327093	0.15	0.543	3.36E-139	CAPG	1	C1QC_KC
675	5.33E-239	-0.927917849	0.798	0.947	1.16E-234	LGALS1	1	C1QC_KC
676	0	-0.932937776	0.967	0.994	0	SH3BGRL3	1	C1QC_KC
677	4.63E-148	-0.938888714	0.118	0.5	1.01E-143	MGST1	1	C1QC_KC
678	4.44E-295	-0.985833546	0.787	0.944	9.68E-291	TSPO	1	C1QC_KC
679	4.83E-198	-0.989490997	0.788	0.92	1.05E-193	IFITM2	1	C1QC_KC
680	2.68E-148	-0.995844042	0.642	0.822	5.85E-144	GCA	1	C1QC_KC
681	1.67E-171	-1.030400857	0.175	0.586	3.64E-167	PLP2	1	C1QC_KC
682	4.78E-143	-1.052275925	0.104	0.499	1.04E-138	ALOX5AP	1	C1QC_KC
683	2.08E-93	-1.05232773	0.46	0.723	4.53E-89	THBS1	1	C1QC_KC
684	0	-1.053310986	0.969	0.995	0	GAPDH	1	C1QC_KC
685	7.32E-119	-1.053481566	0.297	0.59	1.60E-114	PLBD1	1	C1QC_KC
686	3.04E-219	-1.092437467	0.53	0.851	6.62E-215	CSTA	1	C1QC_KC
687	1.46E-187	-1.156403939	0.538	0.809	3.19E-183	MNDA	1	C1QC_KC
688	7.98E-120	-1.1593338	0.423	0.679	1.74E-115	CRIP1	1	C1QC_KC
689	3.73E-151	-1.185359103	0.118	0.505	8.14E-147	SELL	1	C1QC_KC
690	1.04E-217	-1.310341575	0.514	0.823	2.28E-213	STXBP2	1	C1QC_KC
691	0	-1.492075664	0.749	0.975	0	S100A10	1	C1QC_KC
692	0	-1.698825964	0.929	0.994	0	LYZ	1	C1QC_KC
693	0	-1.779998673	0.769	0.991	0	S100A4	1	C1QC_KC
694	0	-1.837453737	0.806	0.995	0	S100A6	1	C1QC_KC
695	0	-1.843919439	0.295	0.913	0	FCN1	1	C1QC_KC
696	0	-2.522344019	0.122	0.853	0	VCAN	1	C1QC_KC
697	0	-2.634180423	0.132	0.792	0	S100A12	1	C1QC_KC
698	0	-2.735410856	0.767	0.977	0	S100A9	1	C1QC_KC
699	0	-3.170438222	0.491	0.974	0	S100A8	1	C1QC_KC
700	1.01E-240	1.073081743	0.96	0.362	2.21E-236	VCAN	2	VCAN_TMo
701	6.77E-190	1.014887234	0.985	0.845	1.48E-185	S100A9	2	VCAN_TMo
702	4.18E-111	0.948979348	0.852	0.511	9.12E-107	THBS1	2	VCAN_TMo
703	1.29E-114	0.885441073	0.788	0.479	2.81E-110	CRIP1	2	VCAN_TMo
704	2.69E-198	0.858755689	0.976	0.509	5.87E-194	FCN1	2	VCAN_TMo
705	3.73E-189	0.770105725	0.991	0.83	8.14E-185	S100A10	2	VCAN_TMo
706	2.56E-183	0.740098907	1	0.951	5.59E-179	LYZ	2	VCAN_TMo
707	5.41E-77	0.737332471	0.568	0.259	1.18E-72	EREG	2	VCAN_TMo
708	8.11E-158	0.679785947	0.998	0.852	1.77E-153	S100A4	2	VCAN_TMo
709	5.57E-100	0.673969833	0.733	0.418	1.21E-95	TPM4	2	VCAN_TMo
710	8.76E-100	0.659439196	0.487	0.158	1.91E-95	CD300E	2	VCAN_TMo
711	1.37E-119	0.614209915	0.698	0.29	2.98E-115	PLP2	2	VCAN_TMo
712	2.46E-148	0.613494396	1	0.877	5.37E-144	S100A6	2	VCAN_TMo
713	1.13E-84	0.591861556	0.405	0.118	2.48E-80	APOBEC3A	2	VCAN_TMo
714	1.77E-181	0.582650451	0.997	0.976	3.87E-177	SH3BGRL3	2	VCAN_TMo
715	2.06E-59	0.575052486	0.247	0.056	4.49E-55	SERPINB2	2	VCAN_TMo
716	6.33E-108	0.564921245	0.661	0.255	1.38E-103	CAPG	2	VCAN_TMo
717	9.27E-68	0.56072105	0.89	0.655	2.02E-63	PLAUR	2	VCAN_TMo
718	7.97E-72	0.549399264	0.489	0.205	1.74E-67	TREM1	2	VCAN_TMo
719	4.54E-127	0.548084783	0.981	0.842	9.89E-123	LGALS1	2	VCAN_TMo
720	2.88E-86	0.54722038	0.837	0.632	6.28E-82	ANXA2	2	VCAN_TMo
721	9.87E-91	0.540769083	0.423	0.127	2.15E-86	LGALS2	2	VCAN_TMo
722	1.49E-159	0.539589064	0.998	0.977	3.25E-155	GAPDH	2	VCAN_TMo
723	2.31E-78	0.539448652	0.475	0.17	5.03E-74	AQP9	2	VCAN_TMo
724	2.28E-72	0.52915434	0.691	0.396	4.97E-68	MYADM	2	VCAN_TMo
725	1.02E-136	0.528131726	0.999	0.987	2.23E-132	SRGN	2	VCAN_TMo
726	5.21E-85	0.511554812	0.854	0.663	1.14E-80	PKM	2	VCAN_TMo
727	4.74E-38	0.511342492	0.725	0.495	1.03E-33	AREG	2	VCAN_TMo
728	2.91E-48	0.496148981	0.964	0.848	6.34E-44	TIMP1	2	VCAN_TMo
729	4.86E-93	0.4949904	0.497	0.15	1.06E-88	MCEMP1	2	VCAN_TMo
730	9.10E-54	0.493188289	0.697	0.434	1.98E-49	SLC2A3	2	VCAN_TMo
731	5.07E-91	0.476363566	0.669	0.311	1.11E-86	GLIPR2	2	VCAN_TMo
732	2.44E-117	0.476068018	0.987	0.953	5.32E-113	ACTG1	2	VCAN_TMo
733	4.79E-136	0.475463268	0.988	0.933	1.04E-131	CFL1	2	VCAN_TMo
734	5.60E-50	0.47173898	0.536	0.292	1.22E-45	NLRP3	2	VCAN_TMo
735	1.60E-83	0.460616551	0.92	0.675	3.49E-79	ANXA1	2	VCAN_TMo
736	4.22E-62	0.460611379	0.702	0.44	9.21E-58	CLEC4E	2	VCAN_TMo
737	1.89E-72	0.459364961	0.798	0.557	4.11E-68	FGR	2	VCAN_TMo
738	1.77E-89	0.457576733	0.589	0.231	3.85E-85	MGST1	2	VCAN_TMo
739	2.54E-112	0.454855306	0.997	0.982	5.54E-108	VIM	2	VCAN_TMo
740	6.24E-107	0.449427752	0.969	0.838	1.36E-102	TSPO	2	VCAN_TMo
741	9.61E-52	0.448648591	0.794	0.591	2.10E-47	FPR1	2	VCAN_TMo
742	3.80E-65	0.445788202	0.762	0.509	8.29E-61	PTPRE	2	VCAN_TMo
743	4.06E-57	0.43768141	0.713	0.481	8.85E-53	LTA4H	2	VCAN_TMo
744	9.38E-63	0.431606231	0.724	0.455	2.05E-58	FLNA	2	VCAN_TMo
745	6.74E-69	0.428196455	0.951	0.808	1.47E-64	EMP3	2	VCAN_TMo
746	2.09E-49	0.421733192	0.399	0.176	4.56E-45	ASPH	2	VCAN_TMo
747	4.30E-75	0.421617396	0.451	0.164	9.38E-71	ANPEP	2	VCAN_TMo
748	2.30E-71	0.421535751	0.453	0.16	5.02E-67	ICAM3	2	VCAN_TMo

749	2.22E-69	0.419853379	0.88	0.705	4.85E-65	CORO1A	2	VCAN_TMo
750	3.66E-51	0.41242406	0.308	0.104	7.99E-47	TRMT6	2	VCAN_TMo
751	8.88E-56	0.40649183	0.818	0.649	1.94E-51	CD99	2	VCAN_TMo
752	1.91E-48	0.405199725	0.732	0.52	4.17E-44	RILPL2	2	VCAN_TMo
753	9.96E-38	0.405171585	0.841	0.734	2.17E-33	TAGLN2	2	VCAN_TMo
754	4.75E-63	0.403664468	0.875	0.721	1.04E-58	RBM3	2	VCAN_TMo
755	2.18E-121	0.39960979	0.995	0.962	4.75E-117	PFN1	2	VCAN_TMo
756	1.49E-81	0.398490605	0.965	0.856	3.24E-77	CLIC1	2	VCAN_TMo
757	1.94E-38	0.3983794	0.43	0.231	4.24E-34	PTGER2	2	VCAN_TMo
758	6.93E-54	0.397486491	0.837	0.677	1.51E-49	ENO1	2	VCAN_TMo
759	4.42E-18	0.393865893	0.66	0.528	9.63E-14	CXCL8	2	VCAN_TMo
760	3.54E-114	0.389661377	1	0.998	7.72E-110	ACTB	2	VCAN_TMo
761	9.09E-67	0.389357398	0.842	0.63	1.98E-62	MNDA	2	VCAN_TMo
762	1.33E-48	0.383870892	0.531	0.3	2.90E-44	PNPLA2	2	VCAN_TMo
763	1.25E-54	0.380127607	0.822	0.645	2.74E-50	APLP2	2	VCAN_TMo
764	1.03E-50	0.375929672	0.71	0.462	2.24E-46	LSP1	2	VCAN_TMo
765	2.90E-50	0.373155016	0.547	0.293	6.31E-46	MYO1G	2	VCAN_TMo
766	8.94E-38	0.371296211	0.747	0.57	1.95E-33	AHNAK	2	VCAN_TMo
767	6.34E-57	0.368341369	0.935	0.775	1.38E-52	SERPINA1	2	VCAN_TMo
768	2.21E-38	0.367188027	0.455	0.251	4.83E-34	RNASE2	2	VCAN_TMo
769	2.70E-48	0.36586365	0.807	0.611	5.88E-44	SLC11A1	2	VCAN_TMo
770	2.30E-34	0.359713707	0.532	0.322	5.02E-30	HBEGF	2	VCAN_TMo
771	6.58E-39	0.357612849	0.647	0.438	1.44E-34	TMEM176B	2	VCAN_TMo
772	1.96E-83	0.356962218	0.987	0.914	4.28E-79	COTL1	2	VCAN_TMo
773	1.24E-41	0.353502017	0.657	0.452	2.70E-37	NCF2	2	VCAN_TMo
774	2.97E-83	0.352023161	0.99	0.953	6.48E-79	MYL6	2	VCAN_TMo
775	2.00E-56	0.351460973	0.838	0.628	4.36E-52	STXBP2	2	VCAN_TMo
776	8.82E-13	0.349102707	0.411	0.299	1.92E-08	IL1R2	2	VCAN_TMo
777	6.31E-81	0.349030504	0.255	0.04	1.38E-76	NRG1	2	VCAN_TMo
778	8.06E-71	0.34815715	0.969	0.904	1.76E-66	ALDOA	2	VCAN_TMo
779	1.53E-30	0.346355741	0.675	0.517	3.35E-26	UPP1	2	VCAN_TMo
780	5.14E-52	0.343535562	0.327	0.116	1.12E-47	BST1	2	VCAN_TMo
781	1.07E-48	0.343433353	0.867	0.722	2.34E-44	ZYX	2	VCAN_TMo
782	1.12E-48	0.342563667	0.937	0.833	2.45E-44	IFITM2	2	VCAN_TMo
783	8.81E-37	0.342173542	0.723	0.513	1.92E-32	ACSL1	2	VCAN_TMo
784	5.49E-51	0.342064739	0.533	0.256	1.20E-46	SELL	2	VCAN_TMo
785	1.10E-48	0.341753102	0.817	0.648	2.40E-44	TALDO1	2	VCAN_TMo
786	4.43E-48	0.340355639	0.431	0.204	9.66E-44	CAPN2	2	VCAN_TMo
787	1.17E-37	0.340174742	0.857	0.655	2.56E-33	BCL2A1	2	VCAN_TMo
788	1.91E-34	0.337422464	0.293	0.126	4.16E-30	ANKRD28	2	VCAN_TMo
789	1.49E-39	0.336734996	0.79	0.603	3.24E-35	TYMP	2	VCAN_TMo
790	1.16E-36	0.330253595	0.913	0.799	2.54E-32	BTG1	2	VCAN_TMo
791	1.57E-34	0.323848858	0.653	0.475	3.42E-30	TMEM167A	2	VCAN_TMo
792	6.25E-32	0.321611454	0.945	0.869	1.36E-27	NFKBIA	2	VCAN_TMo
793	2.25E-38	0.31627378	0.567	0.336	4.90E-34	FAM65B	2	VCAN_TMo
794	1.06E-20	0.315721438	0.892	0.803	2.31E-16	IFITM3	2	VCAN_TMo
795	1.19E-73	0.313954232	0.991	0.976	2.59E-69	S100A11	2	VCAN_TMo
796	4.35E-42	0.313876859	0.902	0.783	9.48E-38	TPM3	2	VCAN_TMo
797	9.02E-28	0.312918432	0.925	0.828	1.97E-23	NAMPT	2	VCAN_TMo
798	4.10E-34	0.311847791	0.903	0.78	8.95E-30	ZEB2	2	VCAN_TMo
799	5.78E-12	0.310940598	0.256	0.158	1.26E-07	RGCC	2	VCAN_TMo
800	1.86E-26	0.310397094	0.486	0.322	4.05E-22	AGFG1	2	VCAN_TMo
801	1.18E-22	0.307799651	0.453	0.304	2.58E-18	CSRNP1	2	VCAN_TMo
802	1.67E-29	0.303197095	0.531	0.356	3.65E-25	MAP2K1	2	VCAN_TMo
803	6.55E-16	0.303181518	0.723	0.611	1.43E-11	CD52	2	VCAN_TMo
804	1.89E-28	0.30207523	0.62	0.464	4.11E-24	SFPQ	2	VCAN_TMo
805	3.00E-38	0.300741916	0.911	0.795	6.55E-34	ITGB2	2	VCAN_TMo
806	3.19E-65	0.300351596	0.234	0.045	6.96E-61	FCAR	2	VCAN_TMo
807	1.10E-59	0.300173471	0.228	0.047	2.39E-55	CCR2	2	VCAN_TMo
808	1.65E-34	0.299565236	0.6	0.417	3.60E-30	METTL9	2	VCAN_TMo
809	1.46E-09	0.298242532	0.243	0.167	3.19E-05	SLC39A8	2	VCAN_TMo
810	2.31E-36	0.297130576	0.612	0.401	5.05E-32	PLBD1	2	VCAN_TMo
811	7.68E-86	0.296764688	0.996	0.977	1.67E-81	H3F3A	2	VCAN_TMo
812	9.33E-33	0.295895046	0.378	0.203	2.03E-28	CCDC109B	2	VCAN_TMo
813	1.37E-21	0.295431135	0.344	0.204	2.99E-17	ZC3H12A	2	VCAN_TMo
814	4.12E-40	0.295173219	0.368	0.168	8.98E-36	PSTPIP1	2	VCAN_TMo
815	4.59E-31	0.294408033	0.554	0.347	1.00E-26	GPR183	2	VCAN_TMo
816	7.00E-45	0.291787688	0.251	0.077	1.53E-40	ASGR2	2	VCAN_TMo
817	2.06E-68	0.291430516	0.884	0.641	4.48E-64	CSTA	2	VCAN_TMo
818	4.34E-43	0.289381482	0.38	0.171	9.45E-39	CCDC69	2	VCAN_TMo
819	2.61E-26	0.28935781	0.538	0.384	5.70E-22	FERMT3	2	VCAN_TMo
820	9.74E-30	0.289329197	0.481	0.3	2.12E-25	C19orf38	2	VCAN_TMo
821	6.41E-43	0.286444854	0.916	0.812	1.40E-38	FXYD5	2	VCAN_TMo
822	2.75E-90	0.286079424	0.998	0.99	6.01E-86	TPT1	2	VCAN_TMo
823	1.67E-39	0.286016229	0.778	0.61	3.63E-35	PGD	2	VCAN_TMo

824	3.46E-26	0.285577999	0.637	0.484	7.55E-22	SRSF2	2	VCAN_TMo
825	2.16E-29	0.285064277	0.689	0.535	4.71E-25	AGTRAP	2	VCAN_TMo
826	6.10E-34	0.284577372	0.818	0.661	1.33E-29	CD44	2	VCAN_TMo
827	7.76E-35	0.28382713	0.871	0.767	1.69E-30	HNRNPA1	2	VCAN_TMo
828	8.44E-32	0.282873305	0.418	0.236	1.84E-27	ASGR1	2	VCAN_TMo
829	2.88E-22	0.2823638	0.713	0.548	6.27E-18	IER3	2	VCAN_TMo
830	5.91E-23	0.280283702	0.467	0.317	1.29E-18	ODF3B	2	VCAN_TMo
831	1.36E-25	0.278447305	0.586	0.396	2.96E-21	GK	2	VCAN_TMo
832	1.51E-49	0.278298991	0.998	0.97	3.29E-45	NEAT1	2	VCAN_TMo
833	3.23E-26	0.277973548	0.419	0.26	7.05E-22	PLIN3	2	VCAN_TMo
834	3.75E-67	0.276956086	0.994	0.953	8.19E-63	RPS9	2	VCAN_TMo
835	2.03E-27	0.276127968	0.546	0.377	4.43E-23	EFHD2	2	VCAN_TMo
836	5.63E-26	0.276109798	0.513	0.335	1.23E-21	TMEM176A	2	VCAN_TMo
837	4.65E-21	0.276106407	0.445	0.297	1.01E-16	PNP	2	VCAN_TMo
838	2.49E-27	0.275975023	0.472	0.311	5.42E-23	NUMB	2	VCAN_TMo
839	2.15E-26	0.273861068	0.612	0.454	4.68E-22	CSF3R	2	VCAN_TMo
840	4.34E-44	0.273383924	0.438	0.205	9.47E-40	RP11-1143G9.4	2	VCAN_TMo
841	5.67E-34	0.272488983	0.281	0.116	1.24E-29	F13A1	2	VCAN_TMo
842	1.33E-20	0.272049467	0.446	0.307	2.89E-16	KLF10	2	VCAN_TMo
843	1.26E-31	0.271705621	0.75	0.605	2.75E-27	CAST	2	VCAN_TMo
844	4.32E-18	0.271461459	0.557	0.425	9.43E-14	FCGR1A	2	VCAN_TMo
845	4.19E-26	0.26990994	0.63	0.478	9.14E-22	ROCK1	2	VCAN_TMo
846	3.87E-22	0.269584583	0.531	0.368	8.43E-18	EZR	2	VCAN_TMo
847	1.19E-27	0.267185382	0.627	0.471	2.60E-23	C20orf24	2	VCAN_TMo
848	5.42E-16	0.264979796	0.386	0.271	1.18E-11	FBP1	2	VCAN_TMo
849	1.25E-21	0.264289957	0.472	0.33	2.72E-17	AHR	2	VCAN_TMo
850	2.40E-24	0.261339865	0.541	0.382	5.24E-20	IRS2	2	VCAN_TMo
851	3.25E-22	0.261240669	0.608	0.466	7.10E-18	VASP	2	VCAN_TMo
852	1.60E-34	0.260643865	0.947	0.857	3.48E-30	RPL10A	2	VCAN_TMo
853	2.16E-37	0.260567065	0.344	0.148	4.70E-33	CKAP4	2	VCAN_TMo
854	8.69E-21	0.260523278	0.368	0.221	1.90E-16	FPR2	2	VCAN_TMo
855	1.59E-28	0.259076651	0.874	0.778	3.46E-24	SPI1	2	VCAN_TMo
856	1.55E-26	0.258877316	0.361	0.206	3.39E-22	SASH3	2	VCAN_TMo
857	1.65E-26	0.258145897	0.491	0.308	3.61E-22	RP11-670E13.6	2	VCAN_TMo
858	8.75E-25	0.258088266	0.566	0.404	1.91E-20	GPCPD1	2	VCAN_TMo
859	2.78E-29	0.256166556	0.384	0.203	6.06E-25	CD36	2	VCAN_TMo
860	4.36E-28	0.255242184	0.578	0.406	9.51E-24	FBXL5	2	VCAN_TMo
861	9.57E-12	0.254900777	0.281	0.177	2.09E-07	TNFAIP6	2	VCAN_TMo
862	1.34E-24	0.253777648	0.894	0.798	2.93E-20	TKT	2	VCAN_TMo
863	1.13E-27	0.253606231	0.42	0.254	2.46E-23	FMNL1	2	VCAN_TMo
864	1.09E-28	0.253556653	0.912	0.808	2.37E-24	RPL4	2	VCAN_TMo
865	4.42E-11	0.252477115	0.139	0.069	9.65E-07	CCL20	2	VCAN_TMo
866	6.89E-27	0.251516911	0.688	0.521	1.50E-22	CD48	2	VCAN_TMo
867	4.33E-31	0.251157018	0.853	0.717	9.44E-27	SUB1	2	VCAN_TMo
868	1.23E-14	0.250212675	0.458	0.347	2.67E-10	BACH1	2	VCAN_TMo
869	1.31E-24	-0.252126478	0.442	0.622	2.86E-20	CTSL	2	VCAN_TMo
870	8.99E-13	-0.254169377	0.352	0.471	1.96E-08	GIMAP4	2	VCAN_TMo
871	5.67E-09	-0.254989833	0.618	0.655	0.000123686	TXNIP	2	VCAN_TMo
872	4.75E-23	-0.255736609	0.131	0.291	1.04E-18	FCHO2	2	VCAN_TMo
873	2.48E-18	-0.25609319	0.882	0.882	5.42E-14	LST1	2	VCAN_TMo
874	5.16E-15	-0.256903943	0.612	0.689	1.12E-10	LY96	2	VCAN_TMo
875	1.14E-10	-0.257102406	0.379	0.487	2.49E-06	SOD1	2	VCAN_TMo
876	4.99E-16	-0.260352301	0.943	0.884	1.09E-11	CD163	2	VCAN_TMo
877	3.48E-25	-0.261992285	0.122	0.289	7.59E-21	TMC6	2	VCAN_TMo
878	3.46E-29	-0.2653376	0.998	0.989	7.55E-25	MT-ATP6	2	VCAN_TMo
879	2.79E-20	-0.266320196	0.105	0.242	6.09E-16	P4HA1	2	VCAN_TMo
880	8.85E-11	-0.267844825	0.687	0.716	1.93E-06	HERPUD1	2	VCAN_TMo
881	8.02E-19	-0.269028274	0.449	0.589	1.75E-14	TMEM123	2	VCAN_TMo
882	1.12E-18	-0.269351585	0.283	0.441	2.44E-14	CD300A	2	VCAN_TMo
883	3.27E-25	-0.270449704	0.148	0.322	7.13E-21	SIGLEC7	2	VCAN_TMo
884	4.78E-17	-0.272375797	0.362	0.508	1.04E-12	LAMP2	2	VCAN_TMo
885	1.35E-20	-0.273289317	0.264	0.43	2.95E-16	DDAH2	2	VCAN_TMo
886	2.48E-14	-0.275690455	0.416	0.538	5.41E-10	LAMP1	2	VCAN_TMo
887	1.51E-17	-0.276209045	0.291	0.443	3.28E-13	GYPC	2	VCAN_TMo
888	1.36E-25	-0.278323061	0.143	0.313	2.96E-21	DNPH1	2	VCAN_TMo
889	1.73E-20	-0.278862869	0.824	0.804	3.77E-16	HCLS1	2	VCAN_TMo
890	2.46E-18	-0.281610748	0.786	0.782	5.37E-14	BLVRB	2	VCAN_TMo
891	9.75E-21	-0.28207863	0.282	0.451	2.13E-16	TMEM14C	2	VCAN_TMo
892	5.11E-24	-0.282094044	0.161	0.332	1.11E-19	GLMP	2	VCAN_TMo
893	4.42E-21	-0.285909411	0.153	0.305	9.64E-17	AP2A2	2	VCAN_TMo
894	2.44E-19	-0.286696386	0.946	0.918	5.31E-15	MT-ATP8	2	VCAN_TMo
895	1.16E-20	-0.286859188	0.225	0.389	2.53E-16	CISD2	2	VCAN_TMo
896	5.10E-24	-0.288842259	0.757	0.816	1.11E-19	CD302	2	VCAN_TMo
897	8.38E-23	-0.289899914	0.761	0.766	1.83E-18	SARAF	2	VCAN_TMo
898	3.18E-12	-0.29060177	0.889	0.857	6.93E-08	KLF6	2	VCAN_TMo

899	7.38E-11	-0.292490421	0.329	0.44	1.61E-06	PELI1	2	VCAN_TMo
900	2.62E-20	-0.298714179	0.228	0.384	5.72E-16	IDH1	2	VCAN_TMo
901	1.60E-20	-0.300871743	0.121	0.264	3.48E-16	ARRDC3	2	VCAN_TMo
902	3.34E-16	-0.301856277	0.323	0.456	7.29E-12	NPL	2	VCAN_TMo
903	3.45E-20	-0.303780336	0.292	0.455	7.53E-16	PRCP	2	VCAN_TMo
904	2.43E-10	-0.304246722	0.119	0.207	5.30E-06	TWISTNB	2	VCAN_TMo
905	6.09E-20	-0.311273655	0.417	0.557	1.33E-15	ST13	2	VCAN_TMo
906	5.64E-18	-0.312082937	0.341	0.476	1.23E-13	TCN2	2	VCAN_TMo
907	7.09E-17	-0.31264921	0.85	0.822	1.55E-12	GLUL	2	VCAN_TMo
908	3.18E-26	-0.313031028	0.185	0.369	6.93E-22	DAPK1	2	VCAN_TMo
909	3.22E-17	-0.316444344	0.4	0.534	7.03E-13	PRNP	2	VCAN_TMo
910	1.67E-27	-0.316845131	0.202	0.39	3.65E-23	PEBP1	2	VCAN_TMo
911	1.44E-20	-0.319411669	0.564	0.65	3.14E-16	TPP1	2	VCAN_TMo
912	4.53E-26	-0.327315033	0.227	0.415	9.88E-22	SIGLEC10	2	VCAN_TMo
913	2.82E-78	-0.328671688	1	0.998	6.14E-74	MT-CO2	2	VCAN_TMo
914	8.75E-23	-0.329121451	0.297	0.466	1.91E-18	RASSF4	2	VCAN_TMo
915	1.20E-12	-0.333537744	0.649	0.717	2.62E-08	HSP90B1	2	VCAN_TMo
916	1.94E-20	-0.336112847	0.224	0.384	4.22E-16	CACYBP	2	VCAN_TMo
917	2.82E-26	-0.337099942	0.384	0.559	6.14E-22	DRAM2	2	VCAN_TMo
918	2.90E-31	-0.342095609	0.161	0.352	6.32E-27	CRYL1	2	VCAN_TMo
919	2.38E-23	-0.345929707	0.32	0.479	5.20E-19	HLA-DMB	2	VCAN_TMo
920	1.08E-29	-0.352516219	0.662	0.751	2.35E-25	MFSD1	2	VCAN_TMo
921	1.41E-41	-0.35360838	0.987	0.962	3.08E-37	MT-ND4L	2	VCAN_TMo
922	1.36E-28	-0.354695918	0.333	0.527	2.96E-24	SNX5	2	VCAN_TMo
923	9.44E-23	-0.359702192	0.479	0.606	2.06E-18	SNX6	2	VCAN_TMo
924	1.82E-27	-0.360040759	0.22	0.402	3.97E-23	HEXA	2	VCAN_TMo
925	1.10E-12	-0.362448476	0.744	0.757	2.40E-08	HSP90AB1	2	VCAN_TMo
926	6.17E-27	-0.363250972	0.186	0.365	1.35E-22	DNASE2	2	VCAN_TMo
927	2.05E-29	-0.371385104	0.236	0.426	4.48E-25	ADAP2	2	VCAN_TMo
928	1.01E-20	-0.37282799	0.396	0.548	2.21E-16	CLEC2B	2	VCAN_TMo
929	5.73E-26	-0.377613695	0.153	0.323	1.25E-21	P2RY13	2	VCAN_TMo
930	3.57E-32	-0.379427238	0.204	0.412	7.78E-28	SLC1A3	2	VCAN_TMo
931	9.85E-25	-0.379783559	0.741	0.783	2.15E-20	WSB1	2	VCAN_TMo
932	1.41E-36	-0.380860358	0.129	0.34	3.07E-32	ACP5	2	VCAN_TMo
933	4.97E-08	-0.381799665	0.151	0.229	0.001084857	ZFAND2A	2	VCAN_TMo
934	2.79E-40	-0.391780816	0.978	0.926	6.09E-36	PSAP	2	VCAN_TMo
935	8.90E-37	-0.392217025	0.139	0.35	1.94E-32	TCF7L2	2	VCAN_TMo
936	9.80E-38	-0.393560188	0.23	0.458	2.14E-33	CD4	2	VCAN_TMo
937	6.09E-21	-0.396727898	0.584	0.663	1.33E-16	IGSF6	2	VCAN_TMo
938	6.52E-25	-0.40715504	0.618	0.706	1.42E-20	UBB	2	VCAN_TMo
939	7.39E-42	-0.414287677	0.817	0.826	1.61E-37	ASAH1	2	VCAN_TMo
940	1.18E-45	-0.419906409	0.128	0.368	2.58E-41	FRMD4B	2	VCAN_TMo
941	5.71E-38	-0.421249801	0.125	0.34	1.24E-33	CD81	2	VCAN_TMo
942	5.83E-41	-0.425114325	0.226	0.457	1.27E-36	TECR	2	VCAN_TMo
943	3.90E-57	-0.427088842	0.876	0.846	8.51E-53	HLA-E	2	VCAN_TMo
944	2.50E-38	-0.429233159	0.253	0.478	5.44E-34	SCARB2	2	VCAN_TMo
945	7.44E-40	-0.429851223	0.458	0.638	1.62E-35	CHCHD10	2	VCAN_TMo
946	6.51E-26	-0.430329647	0.728	0.792	1.42E-21	RHOB	2	VCAN_TMo
947	5.96E-33	-0.430490677	0.575	0.676	1.30E-28	HLA-DMA	2	VCAN_TMo
948	2.18E-29	-0.437464281	0.64	0.739	4.75E-25	FGL2	2	VCAN_TMo
949	4.03E-42	-0.439190767	0.408	0.609	8.79E-38	CXCL16	2	VCAN_TMo
950	1.22E-16	-0.441201567	0.18	0.306	2.66E-12	PAPSS2	2	VCAN_TMo
951	1.59E-30	-0.453424214	0.739	0.775	3.48E-26	HSPA8	2	VCAN_TMo
952	4.48E-22	-0.459392013	0.566	0.655	9.78E-18	NINJ1	2	VCAN_TMo
953	3.02E-46	-0.463000095	0.124	0.364	6.58E-42	CD84	2	VCAN_TMo
954	1.32E-38	-0.471649783	0.135	0.35	2.88E-34	GLO1	2	VCAN_TMo
955	1.72E-37	-0.49028254	0.288	0.504	3.76E-33	STOM	2	VCAN_TMo
956	1.93E-21	-0.497010616	0.757	0.766	4.22E-17	GADD45B	2	VCAN_TMo
957	2.15E-51	-0.499467906	0.179	0.441	4.68E-47	MSR1	2	VCAN_TMo
958	7.47E-37	-0.502940505	0.545	0.665	1.63E-32	CECR1	2	VCAN_TMo
959	9.98E-94	-0.503502541	1	0.999	2.18E-89	FTL	2	VCAN_TMo
960	3.69E-42	-0.510485531	0.279	0.505	8.05E-38	IL18	2	VCAN_TMo
961	6.17E-50	-0.522607896	0.149	0.402	1.35E-45	DAB2	2	VCAN_TMo
962	2.00E-53	-0.52622381	0.151	0.411	4.37E-49	MERTK	2	VCAN_TMo
963	2.65E-50	-0.533092867	0.92	0.875	5.79E-46	CTSB	2	VCAN_TMo
964	3.76E-10	-0.541276664	0.146	0.239	8.21E-06	HSPA6	2	VCAN_TMo
965	1.33E-96	-0.55638206	0.943	0.926	2.91E-92	ITM2B	2	VCAN_TMo
966	9.10E-57	-0.576780677	0.941	0.883	1.98E-52	HLA-DRB1	2	VCAN_TMo
967	3.42E-44	-0.580721324	0.613	0.722	7.45E-40	HMOX1	2	VCAN_TMo
968	1.91E-116	-0.586614441	0.999	0.99	4.17E-112	CST3	2	VCAN_TMo
969	7.42E-51	-0.590245568	0.777	0.804	1.62E-46	FCGRT	2	VCAN_TMo
970	1.40E-100	-0.602305419	0.931	0.9	3.05E-96	NPC2	2	VCAN_TMo
971	1.04E-21	-0.606093788	0.441	0.591	2.26E-17	HSPE1	2	VCAN_TMo
972	1.22E-53	-0.632356564	0.278	0.527	2.66E-49	MCOLN1	2	VCAN_TMo
973	2.19E-28	-0.632604472	0.826	0.833	4.77E-24	HSP90AA1	2	VCAN_TMo

974	1.16E-67	-0.634932387	0.101	0.395	2.52E-63	GPR34	2	VCAN_TMo
975	1.20E-47	-0.638383477	0.418	0.625	2.63E-43	RBP7	2	VCAN_TMo
976	5.19E-68	-0.668226288	0.177	0.483	1.13E-63	ABCA1	2	VCAN_TMo
977	6.84E-39	-0.669761707	0.332	0.549	1.49E-34	HSPA1B	2	VCAN_TMo
978	9.09E-77	-0.684572845	0.165	0.48	1.98E-72	TSPAN4	2	VCAN_TMo
979	7.66E-96	-0.696069504	0.683	0.79	1.67E-91	RNASET2	2	VCAN_TMo
980	2.31E-78	-0.70450325	0.963	0.909	5.03E-74	HLA-DRA	2	VCAN_TMo
981	2.00E-18	-0.715625851	0.416	0.544	4.37E-14	HSPD1	2	VCAN_TMo
982	2.19E-14	-0.718297171	0.404	0.502	4.77E-10	CCL3	2	VCAN_TMo
983	1.19E-23	-0.728212181	0.463	0.579	2.58E-19	DNAJB1	2	VCAN_TMo
984	1.50E-76	-0.733359141	0.335	0.613	3.27E-72	CSF1R	2	VCAN_TMo
985	2.35E-79	-0.743985961	0.145	0.473	5.12E-75	MAF	2	VCAN_TMo
986	1.73E-62	-0.748072444	0.554	0.697	3.77E-58	CREG1	2	VCAN_TMo
987	1.20E-38	-0.748190303	0.532	0.702	2.61E-34	JUN	2	VCAN_TMo
988	2.17E-107	-0.756433061	0.904	0.882	4.73E-103	CD68	2	VCAN_TMo
989	1.57E-88	-0.788289486	0.145	0.491	3.43E-84	SMPDL3A	2	VCAN_TMo
990	6.74E-103	-0.790049659	0.846	0.877	1.47E-98	MS4A6A	2	VCAN_TMo
991	1.38E-104	-0.806134222	0.991	0.971	3.00E-100	CD74	2	VCAN_TMo
992	2.49E-71	-0.818613262	0.301	0.586	5.44E-67	VSIG4	2	VCAN_TMo
993	8.74E-74	-0.820908502	0.28	0.564	1.91E-69	CPM	2	VCAN_TMo
994	1.19E-08	-0.82317508	0.206	0.286	0.000259657	RNASE1	2	VCAN_TMo
995	9.04E-84	-0.829520989	0.615	0.756	1.97E-79	HLA-DQB1	2	VCAN_TMo
996	4.70E-34	-0.83018853	0.248	0.448	1.02E-29	HSPH1	2	VCAN_TMo
997	1.38E-112	-0.876884209	0.248	0.624	3.01E-108	FAM26F	2	VCAN_TMo
998	3.57E-90	-0.883825209	0.531	0.722	7.79E-86	CTSC	2	VCAN_TMo
999	4.88E-53	-0.926804487	0.193	0.444	1.06E-48	PDK4	2	VCAN_TMo
1000	2.43E-24	-0.953391417	0.368	0.513	5.29E-20	CCL4	2	VCAN_TMo
1001	4.45E-53	-0.973199855	0.559	0.72	9.70E-49	HSPA1A	2	VCAN_TMo
1002	7.57E-38	-1.045853288	0.247	0.431	1.65E-33	MARCO	2	VCAN_TMo
1003	4.57E-54	-1.068032477	0.249	0.512	9.98E-50	HSPB1	2	VCAN_TMo
1004	1.97E-117	-1.076862665	0.729	0.807	4.30E-113	HLA-DPA1	2	VCAN_TMo
1005	1.52E-111	-1.088124009	0.664	0.778	3.30E-107	HLA-DPB1	2	VCAN_TMo
1006	3.35E-127	-1.094379501	0.722	0.827	7.32E-123	HLA-DRB5	2	VCAN_TMo
1007	1.93E-124	-1.104316006	0.497	0.745	4.20E-120	MS4A7	2	VCAN_TMo
1008	1.86E-148	-1.23978754	0.282	0.684	4.05E-144	FCGR3A	2	VCAN_TMo
1009	2.80E-145	-1.300041344	0.311	0.694	6.10E-141	MS4A4A	2	VCAN_TMo
1010	7.86E-34	-1.351067461	0.216	0.407	1.71E-29	CCL4L2	2	VCAN_TMo
1011	1.03E-114	-1.352710924	0.507	0.726	2.24E-110	LIPA	2	VCAN_TMo
1012	3.84E-164	-1.464895493	0.197	0.664	8.37E-160	HLA-DQA1	2	VCAN_TMo
1013	2.11E-224	-2.691256563	0.147	0.705	4.60E-220	C1QA	2	VCAN_TMo
1014	2.57E-228	-2.914268973	0.11	0.692	5.60E-224	C1QC	2	VCAN_TMo
1015	9.94E-231	-3.038857252	0.103	0.692	2.17E-226	C1QB	2	VCAN_TMo
1016	5.65E-285	2.059592112	0.998	0.709	1.23E-280	S100A8	3	S100A8_TMo
1017	6.01E-260	2.051188953	0.95	0.401	1.31E-255	S100A12	3	S100A8_TMo
1018	5.88E-74	1.365784058	0.347	0.094	1.28E-69	RETN	3	S100A8_TMo
1019	8.90E-175	1.304977041	0.727	0.235	1.94E-170	ALOX5AP	3	S100A8_TMo
1020	1.13E-156	1.131735217	0.986	0.862	2.47E-152	S100A9	3	S100A8_TMo
1021	1.44E-97	1.095102324	0.611	0.272	3.15E-93	SELL	3	S100A8_TMo
1022	3.24E-222	1.075605633	1	0.892	7.06E-218	S100A6	3	S100A8_TMo
1023	1.41E-116	1.014716251	0.847	0.679	3.07E-112	CSTA	3	S100A8_TMo
1024	9.70E-193	1.014701498	0.989	0.96	2.12E-188	LYZ	3	S100A8_TMo
1025	1.47E-136	1.014293568	0.89	0.451	3.21E-132	VCAN	3	S100A8_TMo
1026	2.65E-99	1.007964435	0.824	0.725	5.77E-95	GCA	3	S100A8_TMo
1027	3.89E-75	1.003803605	0.646	0.419	8.48E-71	PLBD1	3	S100A8_TMo
1028	1.04E-80	0.99197916	0.79	0.664	2.27E-76	STXBP2	3	S100A8_TMo
1029	3.90E-104	0.991025894	0.824	0.66	8.50E-100	MNDA	3	S100A8_TMo
1030	2.48E-62	0.923464062	0.467	0.213	5.41E-58	CDA	3	S100A8_TMo
1031	5.36E-161	0.892124448	0.986	0.872	1.17E-156	S100A4	3	S100A8_TMo
1032	7.42E-35	0.823425713	0.477	0.298	1.62E-30	IL1R2	3	S100A8_TMo
1033	3.46E-54	0.812533854	0.45	0.203	7.54E-50	MCEMP1	3	S100A8_TMo
1034	2.97E-92	0.768372686	0.869	0.822	6.47E-88	SERPINB1	3	S100A8_TMo
1035	4.98E-121	0.766665857	0.961	0.856	1.08E-116	S100A10	3	S100A8_TMo
1036	3.24E-23	0.76066732	0.378	0.247	7.06E-19	RP11-1143G9.4	3	S100A8_TMo
1037	1.23E-49	0.758087162	0.515	0.291	2.68E-45	MGST1	3	S100A8_TMo
1038	3.76E-36	0.755953383	0.601	0.504	8.21E-32	HMGB2	3	S100A8_TMo
1039	8.61E-162	0.744947925	0.992	0.981	1.88E-157	GAPDH	3	S100A8_TMo
1040	1.52E-49	0.714904548	0.701	0.648	3.31E-45	PGD	3	S100A8_TMo
1041	3.20E-32	0.675332882	0.341	0.173	6.98E-28	CKAP4	3	S100A8_TMo
1042	2.49E-99	0.669358089	0.91	0.867	5.43E-95	TSPO	3	S100A8_TMo
1043	4.35E-23	0.66651734	0.327	0.179	9.49E-19	TNFAIP6	3	S100A8_TMo
1044	1.69E-43	0.659335997	0.266	0.086	3.69E-39	CES1	3	S100A8_TMo
1045	3.12E-29	0.65612925	0.323	0.166	6.81E-25	EMB	3	S100A8_TMo
1046	2.34E-175	0.641714114	0.998	0.997	5.11E-171	RPL39	3	S100A8_TMo
1047	1.93E-34	0.64155963	0.153	0.036	4.21E-30	CST7	3	S100A8_TMo
1048	5.26E-34	0.636831765	0.533	0.377	1.15E-29	PLP2	3	S100A8_TMo

1049	1.52E-21	0.632034651	0.482	0.383	3.32E-17	FAM65B	3	S100A8_TMo
1050	9.83E-64	0.631833151	0.833	0.598	2.14E-59	FCN1	3	S100A8_TMo
1051	4.13E-14	0.595256755	0.293	0.195	9.01E-10	CYP1B1	3	S100A8_TMo
1052	1.15E-28	0.595170344	0.149	0.04	2.52E-24	S100P	3	S100A8_TMo
1053	1.57E-23	0.58799584	0.495	0.394	3.43E-19	GLIPR2	3	S100A8_TMo
1054	1.01E-21	0.586729109	0.303	0.172	2.21E-17	MEGF9	3	S100A8_TMo
1055	5.42E-27	0.575874	0.631	0.569	1.18E-22	LILRA5	3	S100A8_TMo
1056	9.65E-17	0.573251395	0.495	0.463	2.11E-12	METTL9	3	S100A8_TMo
1057	1.36E-34	0.570196126	0.682	0.699	2.97E-30	TALDO1	3	S100A8_TMo
1058	2.72E-27	0.566591594	0.492	0.343	5.92E-23	CAPG	3	S100A8_TMo
1059	3.74E-63	0.564612324	0.896	0.878	8.15E-59	LGALS1	3	S100A8_TMo
1060	9.53E-14	0.561175983	0.423	0.352	2.08E-09	NCF1	3	S100A8_TMo
1061	2.38E-25	0.558910785	0.366	0.215	5.18E-21	ICAM3	3	S100A8_TMo
1062	2.06E-22	0.557881805	0.255	0.124	4.49E-18	RP6-159A1.4	3	S100A8_TMo
1063	1.02E-13	0.537478081	0.476	0.45	2.23E-09	FBXL5	3	S100A8_TMo
1064	1.29E-133	0.531078072	1	0.998	2.82E-129	ACTB	3	S100A8_TMo
1065	8.14E-19	0.527560348	0.225	0.112	1.78E-14	CLU	3	S100A8_TMo
1066	1.42E-12	0.523307943	0.333	0.247	3.09E-08	FPR2	3	S100A8_TMo
1067	9.65E-78	0.517230963	0.962	0.964	2.10E-73	MYL6	3	S100A8_TMo
1068	4.45E-32	0.499153835	0.198	0.063	9.70E-28	NFE2	3	S100A8_TMo
1069	2.06E-24	0.489425609	0.173	0.06	4.49E-20	1-Mar	3	S100A8_TMo
1070	2.36E-15	0.481446261	0.559	0.526	5.14E-11	LSP1	3	S100A8_TMo
1071	7.61E-14	0.478656956	0.583	0.631	1.66E-09	NDUFB9	3	S100A8_TMo
1072	9.40E-39	0.475375757	0.875	0.859	2.05E-34	IFITM2	3	S100A8_TMo
1073	2.95E-19	0.473602688	0.581	0.493	6.43E-15	SLC2A3	3	S100A8_TMo
1074	1.05E-07	0.47010164	0.305	0.256	0.002300362	UBR4	3	S100A8_TMo
1075	3.58E-26	0.46895967	0.219	0.087	7.80E-22	QPCT	3	S100A8_TMo
1076	5.09E-22	0.467730805	0.171	0.064	1.11E-17	CLEC4D	3	S100A8_TMo
1077	6.16E-11	0.466353925	0.571	0.648	1.34E-06	NDUFB1	3	S100A8_TMo
1078	1.05E-17	0.465520759	0.638	0.651	2.29E-13	FPR1	3	S100A8_TMo
1079	1.12E-13	0.463949467	0.159	0.075	2.45E-09	CTB-61M7.2	3	S100A8_TMo
1080	3.02E-12	0.462176323	0.254	0.164	6.59E-08	CPD	3	S100A8_TMo
1081	2.90E-16	0.458479301	0.197	0.091	6.32E-12	SERPINB2	3	S100A8_TMo
1082	6.26E-49	0.453704235	0.904	0.946	1.37E-44	EEF1D	3	S100A8_TMo
1083	1.44E-85	0.451846199	0.991	0.98	3.14E-81	SH3BGRL3	3	S100A8_TMo
1084	4.67E-21	0.443517634	0.683	0.725	1.02E-16	PKM	3	S100A8_TMo
1085	3.26E-26	0.428788682	0.803	0.882	7.11E-22	MYL12A	3	S100A8_TMo
1086	1.02E-32	0.424973914	0.805	0.731	2.23E-28	ANXA1	3	S100A8_TMo
1087	3.00E-13	0.424264426	0.644	0.738	6.53E-09	SEC61G	3	S100A8_TMo
1088	1.03E-06	0.422209615	0.282	0.236	0.022464484	FAM200B	3	S100A8_TMo
1089	2.55E-09	0.418524487	0.631	0.693	5.56E-05	CD37	3	S100A8_TMo
1090	1.15E-22	0.414257187	0.751	0.854	2.51E-18	TCEB2	3	S100A8_TMo
1091	1.93E-06	0.411733071	0.347	0.314	0.042142886	CDC42EP3	3	S100A8_TMo
1092	3.80E-07	0.404796694	0.512	0.555	0.008296101	LTA4H	3	S100A8_TMo
1093	2.43E-07	0.403019858	0.529	0.59	0.005299818	AGTRAP	3	S100A8_TMo
1094	2.07E-07	0.402044435	0.497	0.56	0.004516899	PGAM1	3	S100A8_TMo
1095	3.60E-57	0.399972924	0.982	0.982	7.84E-53	H3F3A	3	S100A8_TMo
1096	7.13E-18	0.399716677	0.12	0.04	1.55E-13	FAM101B	3	S100A8_TMo
1097	3.75E-13	0.394732539	0.191	0.101	8.17E-09	TESC	3	S100A8_TMo
1098	7.82E-24	0.394717422	0.73	0.76	1.71E-19	CORO1A	3	S100A8_TMo
1099	1.53E-112	0.39406405	0.994	0.997	3.34E-108	RPLP1	3	S100A8_TMo
1100	2.19E-71	0.393842011	0.989	0.992	4.78E-67	FAU	3	S100A8_TMo
1101	2.39E-09	0.38880646	0.611	0.714	5.22E-05	APLP2	3	S100A8_TMo
1102	2.58E-12	0.377814422	0.65	0.76	5.64E-08	GMFG	3	S100A8_TMo
1103	4.37E-60	0.377271423	0.992	0.997	9.53E-56	RPS13	3	S100A8_TMo
1104	2.69E-21	0.376433844	0.743	0.849	5.87E-17	C14orf2	3	S100A8_TMo
1105	3.24E-18	0.374820119	0.131	0.045	7.07E-14	FOLR3	3	S100A8_TMo
1106	5.50E-18	0.373282961	0.123	0.041	1.20E-13	PADI4	3	S100A8_TMo
1107	1.79E-06	0.369356632	0.173	0.118	0.039043514	DYSF	3	S100A8_TMo
1108	1.64E-43	0.367511289	0.901	0.927	3.57E-39	ALDOA	3	S100A8_TMo
1109	2.26E-51	0.364332142	0.983	0.986	4.93E-47	ATP5E	3	S100A8_TMo
1110	3.07E-91	0.358119765	0.994	0.996	6.69E-87	RPS24	3	S100A8_TMo
1111	6.30E-21	0.353296529	0.787	0.889	1.37E-16	GNG5	3	S100A8_TMo
1112	1.92E-10	0.350838508	0.113	0.05	4.18E-06	PROK2	3	S100A8_TMo
1113	7.44E-07	0.349811318	0.173	0.113	0.01621709	VNN2	3	S100A8_TMo
1114	1.34E-44	0.348730423	0.976	0.96	2.91E-40	ACTG1	3	S100A8_TMo
1115	2.00E-08	0.346166699	0.206	0.137	0.000436806	RAB27A	3	S100A8_TMo
1116	6.34E-42	0.341429072	0.974	0.982	1.38E-37	S100A11	3	S100A8_TMo
1117	2.48E-09	0.341320783	0.641	0.782	5.41E-05	SUB1	3	S100A8_TMo
1118	6.14E-11	0.335240668	0.695	0.809	1.34E-06	COMMD6	3	S100A8_TMo
1119	3.47E-20	0.32868039	0.102	0.026	7.56E-16	SERPINB10	3	S100A8_TMo
1120	1.44E-09	0.326193719	0.637	0.751	3.15E-05	SEC61B	3	S100A8_TMo
1121	7.10E-19	0.325694193	0.131	0.044	1.55E-14	F5	3	S100A8_TMo
1122	4.71E-18	0.316683797	0.817	0.924	1.03E-13	RPS26	3	S100A8_TMo
1123	6.86E-24	0.314675603	0.833	0.899	1.50E-19	CLIC1	3	S100A8_TMo

1124	3.09E-07	0.31118844	0.628	0.767	0.00673081	COX7A2	3	S100A8_TMo
1125	2.36E-11	0.30787537	0.674	0.593	5.15E-07	THBS1	3	S100A8_TMo
1126	1.86E-55	0.307680762	0.994	0.994	4.05E-51	RPS15A	3	S100A8_TMo
1127	9.36E-09	0.294203606	0.683	0.817	0.000204063	UBL5	3	S100A8_TMo
1128	2.25E-36	0.286932878	0.976	0.985	4.91E-32	SERF2	3	S100A8_TMo
1129	1.55E-37	0.286014827	0.98	0.989	3.39E-33	RPS7	3	S100A8_TMo
1130	1.04E-08	0.284224179	0.691	0.832	0.000227079	UQCR11	3	S100A8_TMo
1131	1.17E-10	0.27745888	0.74	0.871	2.55E-06	TMA7	3	S100A8_TMo
1132	9.91E-07	0.276479046	0.644	0.776	0.021619543	PRR13	3	S100A8_TMo
1133	4.24E-41	0.276029596	0.995	0.996	9.24E-37	RPS8	3	S100A8_TMo
1134	4.38E-07	0.275259469	0.725	0.868	0.009541714	FXYD5	3	S100A8_TMo
1135	4.67E-65	0.274908072	0.998	0.996	1.02E-60	RPL28	3	S100A8_TMo
1136	9.36E-16	0.270700074	0.904	0.964	2.04E-11	ATP5G2	3	S100A8_TMo
1137	4.43E-48	0.266222359	0.997	0.995	9.65E-44	RPL34	3	S100A8_TMo
1138	9.53E-08	0.262588385	0.101	0.05	0.002077485	CRISPLD2	3	S100A8_TMo
1139	8.89E-35	0.254436255	1	0.996	1.94E-30	RPL30	3	S100A8_TMo
1140	2.75E-24	-0.25230878	0.443	0.768	6.00E-20	EIF3F	3	S100A8_TMo
1141	1.17E-25	-0.253353765	0.53	0.85	2.56E-21	GPX4	3	S100A8_TMo
1142	4.47E-19	-0.255899944	0.856	0.981	9.75E-15	MT-ND5	3	S100A8_TMo
1143	2.22E-22	-0.257730099	0.656	0.908	4.84E-18	RPSA	3	S100A8_TMo
1144	2.15E-35	-0.258593533	0.164	0.478	4.69E-31	CD47	3	S100A8_TMo
1145	3.03E-33	-0.259300848	0.159	0.453	6.61E-29	SLC31A2	3	S100A8_TMo
1146	2.31E-36	-0.259460501	0.497	0.807	5.04E-32	MT2A	3	S100A8_TMo
1147	9.78E-33	-0.259697479	0.174	0.477	2.13E-28	AUP1	3	S100A8_TMo
1148	2.85E-32	-0.262095874	0.168	0.458	6.20E-28	SERPINB6	3	S100A8_TMo
1149	1.50E-16	-0.262554745	0.326	0.559	3.27E-12	SOCS3	3	S100A8_TMo
1150	1.91E-30	-0.263238855	0.56	0.891	4.16E-26	ANXA5	3	S100A8_TMo
1151	2.73E-31	-0.263769588	0.339	0.704	5.96E-27	NAP1L1	3	S100A8_TMo
1152	3.19E-31	-0.264344166	0.116	0.374	6.96E-27	SCIMP	3	S100A8_TMo
1153	1.45E-12	-0.264942408	0.712	0.901	3.17E-08	KLF6	3	S100A8_TMo
1154	1.04E-25	-0.265308541	0.479	0.798	2.26E-21	EIF3E	3	S100A8_TMo
1155	7.06E-31	-0.265466524	0.374	0.736	1.54E-26	TMEM59	3	S100A8_TMo
1156	1.58E-31	-0.265720283	0.267	0.602	3.44E-27	NFE2L2	3	S100A8_TMo
1157	1.01E-36	-0.26861803	0.994	0.996	2.20E-32	EEF1A1	3	S100A8_TMo
1158	1.35E-19	-0.270616033	0.89	0.976	2.95E-15	DUSP1	3	S100A8_TMo
1159	5.91E-34	-0.271658263	0.186	0.499	1.29E-29	OXA1L	3	S100A8_TMo
1160	4.70E-31	-0.271975937	0.243	0.567	1.03E-26	ATP1B3	3	S100A8_TMo
1161	1.70E-27	-0.272311998	0.243	0.547	3.70E-23	RNF144B	3	S100A8_TMo
1162	3.79E-34	-0.272444301	0.188	0.515	8.27E-30	SLC3A2	3	S100A8_TMo
1163	2.81E-37	-0.273413223	0.23	0.587	6.14E-33	SNHG8	3	S100A8_TMo
1164	1.47E-28	-0.275138982	0.131	0.387	3.22E-24	IDH1	3	S100A8_TMo
1165	2.70E-21	-0.276233541	0.366	0.642	5.90E-17	NFKBIZ	3	S100A8_TMo
1166	9.59E-34	-0.278448631	0.155	0.45	2.09E-29	SNAP23	3	S100A8_TMo
1167	1.94E-11	-0.27866217	0.21	0.37	4.23E-07	EGR1	3	S100A8_TMo
1168	5.68E-25	-0.279968112	0.104	0.321	1.24E-20	SDF2L1	3	S100A8_TMo
1169	1.49E-36	-0.280160096	0.231	0.584	3.26E-32	FAM96A	3	S100A8_TMo
1170	1.18E-26	-0.280181327	0.547	0.847	2.57E-22	SLC25A5	3	S100A8_TMo
1171	4.70E-37	-0.280648751	0.2	0.543	1.03E-32	HNMT	3	S100A8_TMo
1172	4.36E-29	-0.282030514	0.392	0.747	9.51E-25	GSTK1	3	S100A8_TMo
1173	6.42E-47	-0.282406485	0.965	0.993	1.40E-42	RPS27	3	S100A8_TMo
1174	4.53E-31	-0.283105518	0.218	0.527	9.89E-27	MYDGF	3	S100A8_TMo
1175	1.52E-35	-0.283353152	0.255	0.604	3.31E-31	TOMM20	3	S100A8_TMo
1176	1.16E-38	-0.284030585	0.281	0.652	2.52E-34	RNF13	3	S100A8_TMo
1177	1.98E-27	-0.284732017	0.407	0.757	4.31E-23	RPS20	3	S100A8_TMo
1178	8.51E-31	-0.287200632	0.704	0.937	1.86E-26	RPL13A	3	S100A8_TMo
1179	3.34E-33	-0.290959882	0.85	0.976	7.29E-29	CTSS	3	S100A8_TMo
1180	8.94E-27	-0.293008565	0.111	0.344	1.95E-22	CKS2	3	S100A8_TMo
1181	1.13E-28	-0.293668862	0.462	0.786	2.47E-24	MFSD1	3	S100A8_TMo
1182	1.96E-34	-0.294822437	0.12	0.399	4.26E-30	CHD2	3	S100A8_TMo
1183	1.12E-29	-0.296795818	0.263	0.577	2.44E-25	CTSZ	3	S100A8_TMo
1184	3.11E-37	-0.297896151	0.117	0.414	6.79E-33	HLA-F	3	S100A8_TMo
1185	2.97E-34	-0.298369033	0.128	0.409	6.48E-30	NAGK	3	S100A8_TMo
1186	1.00E-35	-0.299207909	0.339	0.716	2.19E-31	MGAT1	3	S100A8_TMo
1187	4.71E-32	-0.299569728	0.102	0.356	1.03E-27	DDX3Y	3	S100A8_TMo
1188	7.84E-34	-0.30033702	0.107	0.381	1.71E-29	IER5	3	S100A8_TMo
1189	7.56E-40	-0.302350626	0.186	0.527	1.65E-35	MPP1	3	S100A8_TMo
1190	4.47E-29	-0.302879265	0.218	0.515	9.74E-25	INSIG1	3	S100A8_TMo
1191	2.29E-36	-0.303086368	0.161	0.476	4.99E-32	RASSF4	3	S100A8_TMo
1192	6.48E-40	-0.304006813	0.204	0.561	1.41E-35	NUCB1	3	S100A8_TMo
1193	1.74E-33	-0.304471351	0.772	0.937	3.80E-29	RPS5	3	S100A8_TMo
1194	3.76E-51	-0.304614177	0.943	0.993	8.20E-47	EIF1	3	S100A8_TMo
1195	9.35E-41	-0.305237928	0.152	0.483	2.04E-36	BLVRA	3	S100A8_TMo
1196	7.39E-41	-0.308200233	0.212	0.567	1.61E-36	LRRC25	3	S100A8_TMo
1197	1.38E-37	-0.308343248	0.276	0.64	3.02E-33	UBXN1	3	S100A8_TMo
1198	1.92E-35	-0.308512541	0.284	0.639	4.19E-31	FUS	3	S100A8_TMo

1199	1.06E-37	-0.308815953	0.156	0.472	2.31E-33	AKR1A1	3	S100A8_TMo
1200	1.42E-38	-0.309586002	0.104	0.397	3.09E-34	NANS	3	S100A8_TMo
1201	1.92E-36	-0.31048272	0.155	0.466	4.18E-32	RAB20	3	S100A8_TMo
1202	1.22E-43	-0.310889492	0.252	0.643	2.67E-39	ARL6IP1	3	S100A8_TMo
1203	1.07E-28	-0.310992401	0.682	0.928	2.32E-24	RPL10A	3	S100A8_TMo
1204	9.15E-32	-0.312261819	0.368	0.721	2.00E-27	PTGES3	3	S100A8_TMo
1205	1.10E-30	-0.314051048	0.652	0.927	2.40E-26	RPL38	3	S100A8_TMo
1206	5.82E-29	-0.315216871	0.182	0.46	1.27E-24	PELI1	3	S100A8_TMo
1207	3.82E-37	-0.315870608	0.153	0.466	8.32E-33	IRF8	3	S100A8_TMo
1208	6.48E-41	-0.316760945	0.146	0.47	1.41E-36	EIF3D	3	S100A8_TMo
1209	1.52E-63	-0.31682822	1	0.999	3.31E-59	B2M	3	S100A8_TMo
1210	3.63E-41	-0.316885546	0.141	0.469	7.91E-37	PRCP	3	S100A8_TMo
1211	2.63E-34	-0.317080202	0.101	0.37	5.75E-30	ELL2	3	S100A8_TMo
1212	2.25E-43	-0.317321169	0.186	0.546	4.90E-39	IL10RA	3	S100A8_TMo
1213	7.96E-39	-0.317356972	0.269	0.638	1.74E-34	SNX6	3	S100A8_TMo
1214	2.12E-35	-0.3174914	0.272	0.626	4.62E-31	TUBA1B	3	S100A8_TMo
1215	1.11E-38	-0.318163205	0.128	0.438	2.43E-34	ITGA4	3	S100A8_TMo
1216	2.19E-39	-0.318283919	0.275	0.649	4.78E-35	BST2	3	S100A8_TMo
1217	2.97E-38	-0.321559026	0.156	0.477	6.48E-34	NPL	3	S100A8_TMo
1218	2.33E-34	-0.323242994	0.71	0.941	5.07E-30	RPL3	3	S100A8_TMo
1219	7.52E-16	-0.325292876	0.446	0.687	1.64E-11	CD52	3	S100A8_TMo
1220	7.84E-37	-0.326680302	0.296	0.661	1.71E-32	MAP3K8	3	S100A8_TMo
1221	4.36E-36	-0.327874077	0.192	0.518	9.51E-32	TNFAIP2	3	S100A8_TMo
1222	2.40E-35	-0.329181843	0.143	0.442	5.24E-31	FCGR2A	3	S100A8_TMo
1223	1.97E-42	-0.329651294	0.204	0.576	4.29E-38	EIF4A2	3	S100A8_TMo
1224	2.51E-46	-0.329702775	0.2	0.588	5.48E-42	MARCKS	3	S100A8_TMo
1225	1.43E-37	-0.33124803	0.97	0.997	3.13E-33	MT-ATP6	3	S100A8_TMo
1226	7.71E-39	-0.332403092	0.356	0.737	1.68E-34	LY96	3	S100A8_TMo
1227	1.59E-11	-0.332500021	0.683	0.856	3.47E-07	SOD2	3	S100A8_TMo
1228	1.81E-33	-0.333450169	0.132	0.411	3.95E-29	LY6E	3	S100A8_TMo
1229	1.76E-41	-0.336633522	0.215	0.568	3.83E-37	LAMP1	3	S100A8_TMo
1230	7.89E-32	-0.336935076	0.428	0.773	1.72E-27	PRDX1	3	S100A8_TMo
1231	1.56E-38	-0.337099052	0.144	0.456	3.40E-34	AOAH	3	S100A8_TMo
1232	5.60E-45	-0.337238708	0.251	0.631	1.22E-40	NDFIP1	3	S100A8_TMo
1233	2.56E-41	-0.33768792	0.261	0.631	5.59E-37	TUBB	3	S100A8_TMo
1234	1.39E-44	-0.338136802	0.126	0.461	3.03E-40	UTRN	3	S100A8_TMo
1235	1.08E-36	-0.338387393	0.137	0.438	2.35E-32	ADGRE2	3	S100A8_TMo
1236	2.01E-31	-0.338435549	0.498	0.823	4.39E-27	EEF1G	3	S100A8_TMo
1237	5.52E-44	-0.34126038	0.179	0.529	1.20E-39	MCTP1	3	S100A8_TMo
1238	1.11E-33	-0.341307217	0.335	0.694	2.43E-29	DNAJA1	3	S100A8_TMo
1239	1.35E-39	-0.34210304	0.182	0.513	2.94E-35	WASF2	3	S100A8_TMo
1240	1.65E-41	-0.345140658	0.144	0.474	3.59E-37	NAAA	3	S100A8_TMo
1241	1.54E-18	-0.345224487	0.773	0.916	3.35E-14	NFKBIA	3	S100A8_TMo
1242	4.10E-52	-0.349661158	0.862	0.976	8.94E-48	RPS16	3	S100A8_TMo
1243	6.28E-39	-0.350803493	0.282	0.654	1.37E-34	PDIA3	3	S100A8_TMo
1244	1.80E-39	-0.350993039	0.104	0.402	3.93E-35	BHLHE40	3	S100A8_TMo
1245	3.65E-38	-0.35138301	0.67	0.921	7.96E-34	SLC25A6	3	S100A8_TMo
1246	1.95E-42	-0.351547687	0.347	0.748	4.25E-38	LAPTM4A	3	S100A8_TMo
1247	5.84E-33	-0.353250149	0.605	0.883	1.27E-28	EEF2	3	S100A8_TMo
1248	9.78E-42	-0.354311598	0.207	0.558	2.13E-37	DPP7	3	S100A8_TMo
1249	2.45E-39	-0.355188263	0.134	0.441	5.34E-35	CD86	3	S100A8_TMo
1250	1.13E-35	-0.358267737	0.119	0.397	2.47E-31	CSRNP1	3	S100A8_TMo
1251	5.06E-26	-0.35900766	0.52	0.796	1.10E-21	CFD	3	S100A8_TMo
1252	1.06E-40	-0.360377632	0.179	0.519	2.31E-36	SOD1	3	S100A8_TMo
1253	7.94E-36	-0.361718542	0.344	0.698	1.73E-31	RNASE6	3	S100A8_TMo
1254	5.43E-40	-0.362713343	0.21	0.558	1.18E-35	DOK2	3	S100A8_TMo
1255	2.59E-37	-0.364650711	0.584	0.895	5.66E-33	RPL4	3	S100A8_TMo
1256	1.70E-48	-0.365091781	0.264	0.669	3.70E-44	RAB5C	3	S100A8_TMo
1257	1.10E-38	-0.366652654	0.479	0.847	2.39E-34	RPL27A	3	S100A8_TMo
1258	9.80E-27	-0.368591	0.366	0.649	2.14E-22	CXCR4	3	S100A8_TMo
1259	2.44E-43	-0.36971076	0.246	0.618	5.32E-39	TMEM123	3	S100A8_TMo
1260	1.05E-42	-0.370493629	0.135	0.461	2.29E-38	ZCCHC6	3	S100A8_TMo
1261	2.44E-47	-0.37094068	0.206	0.588	5.32E-43	ST13	3	S100A8_TMo
1262	2.93E-39	-0.371911379	0.221	0.57	6.39E-35	CYCS	3	S100A8_TMo
1263	5.13E-35	-0.374288132	0.425	0.767	1.12E-30	IFNGR1	3	S100A8_TMo
1264	2.19E-28	-0.375565282	0.701	0.922	4.78E-24	TIMP1	3	S100A8_TMo
1265	1.94E-47	-0.377898639	0.225	0.612	4.22E-43	GLTSCR2	3	S100A8_TMo
1266	1.49E-45	-0.379937698	0.117	0.447	3.25E-41	FAM49A	3	S100A8_TMo
1267	8.88E-43	-0.380121995	0.206	0.562	1.94E-38	PRNP	3	S100A8_TMo
1268	2.97E-34	-0.380634891	0.228	0.558	6.47E-30	SELK	3	S100A8_TMo
1269	2.82E-33	-0.382052963	0.104	0.365	6.15E-29	ZNF331	3	S100A8_TMo
1270	2.25E-42	-0.39031886	0.48	0.845	4.92E-38	VAMP8	3	S100A8_TMo
1271	5.25E-46	-0.391093807	0.198	0.568	1.15E-41	LILRB1	3	S100A8_TMo
1272	9.37E-31	-0.392079598	0.508	0.83	2.04E-26	WSB1	3	S100A8_TMo
1273	7.80E-45	-0.399268517	0.272	0.658	1.70E-40	CHCHD10	3	S100A8_TMo

1274	1.87E-36	-0.402717914	0.575	0.878	4.08E-32	GRN	3	S100A8_TMo
1275	2.40E-28	-0.403392632	0.264	0.55	5.23E-24	CITED2	3	S100A8_TMo
1276	9.85E-46	-0.407900223	0.27	0.657	2.15E-41	REL	3	S100A8_TMo
1277	6.24E-29	-0.408587948	0.509	0.794	1.36E-24	PPP1R15A	3	S100A8_TMo
1278	1.46E-38	-0.409114519	0.707	0.94	3.19E-34	UBC	3	S100A8_TMo
1279	6.83E-54	-0.411770248	0.26	0.664	1.49E-49	SLC7A7	3	S100A8_TMo
1280	1.50E-52	-0.413555645	0.167	0.555	3.27E-48	MAN2B1	3	S100A8_TMo
1281	2.44E-42	-0.417630447	0.411	0.778	5.31E-38	FGL2	3	S100A8_TMo
1282	6.89E-46	-0.417695399	0.119	0.447	1.50E-41	FGD2	3	S100A8_TMo
1283	8.82E-52	-0.422415196	0.189	0.582	1.92E-47	DRAM2	3	S100A8_TMo
1284	8.07E-51	-0.424534589	0.275	0.69	1.76E-46	LCP2	3	S100A8_TMo
1285	3.92E-76	-0.431208238	0.916	0.987	8.55E-72	RPS19	3	S100A8_TMo
1286	1.10E-50	-0.431217269	0.179	0.553	2.39E-46	GAA	3	S100A8_TMo
1287	3.11E-53	-0.432958699	0.12	0.48	6.77E-49	GUSB	3	S100A8_TMo
1288	1.18E-54	-0.435181637	0.284	0.717	2.57E-50	TBXAS1	3	S100A8_TMo
1289	1.48E-43	-0.441785344	0.299	0.673	3.23E-39	MT-ND6	3	S100A8_TMo
1290	2.37E-42	-0.444853009	0.383	0.757	5.17E-38	KLF4	3	S100A8_TMo
1291	6.86E-36	-0.44994081	0.144	0.434	1.50E-31	DUSP2	3	S100A8_TMo
1292	2.61E-41	-0.449995246	0.132	0.445	5.69E-37	EIF4A3	3	S100A8_TMo
1293	4.09E-49	-0.453070588	0.308	0.697	8.93E-45	TPP1	3	S100A8_TMo
1294	1.29E-51	-0.453789448	0.176	0.548	2.80E-47	PDIA6	3	S100A8_TMo
1295	3.37E-52	-0.45784365	0.116	0.467	7.35E-48	RNF141	3	S100A8_TMo
1296	9.56E-53	-0.460826571	0.524	0.874	2.09E-48	HCLS1	3	S100A8_TMo
1297	5.39E-53	-0.461167364	0.138	0.506	1.17E-48	LILRB4	3	S100A8_TMo
1298	7.23E-61	-0.462381055	0.245	0.683	1.58E-56	LGALS9	3	S100A8_TMo
1299	1.02E-47	-0.465662975	0.335	0.736	2.22E-43	SQSTM1	3	S100A8_TMo
1300	5.39E-38	-0.469384143	0.152	0.447	1.18E-33	SMPDL3A	3	S100A8_TMo
1301	8.67E-49	-0.477789508	0.607	0.903	1.89E-44	CD63	3	S100A8_TMo
1302	7.52E-62	-0.481390187	0.131	0.538	1.64E-57	PABPC4	3	S100A8_TMo
1303	6.56E-53	-0.48279869	0.48	0.851	1.43E-48	BLVRB	3	S100A8_TMo
1304	1.12E-52	-0.483801726	0.551	0.885	2.44E-48	ASAH1	3	S100A8_TMo
1305	1.52E-50	-0.484143641	0.787	0.952	3.31E-46	JUNB	3	S100A8_TMo
1306	7.43E-21	-0.485776722	0.101	0.284	1.62E-16	TNF	3	S100A8_TMo
1307	5.21E-49	-0.488308565	0.132	0.482	1.14E-44	ATF3	3	S100A8_TMo
1308	4.52E-40	-0.495020249	0.372	0.706	9.86E-36	TXNIP	3	S100A8_TMo
1309	6.88E-51	-0.500940183	0.535	0.883	1.50E-46	MAFB	3	S100A8_TMo
1310	3.43E-16	-0.502452312	0.116	0.269	7.47E-12	IL1RN	3	S100A8_TMo
1311	2.37E-41	-0.50314477	0.279	0.647	5.17E-37	PLIN2	3	S100A8_TMo
1312	8.00E-40	-0.508694384	0.395	0.716	1.74E-35	NR4A2	3	S100A8_TMo
1313	2.37E-27	-0.512194385	0.369	0.645	5.16E-23	IER3	3	S100A8_TMo
1314	1.04E-08	-0.521034075	0.227	0.341	0.000227609	G0S2	3	S100A8_TMo
1315	1.59E-55	-0.522731639	0.369	0.784	3.46E-51	HERPUD1	3	S100A8_TMo
1316	4.12E-56	-0.527256304	0.3	0.705	8.98E-52	CECR1	3	S100A8_TMo
1317	2.49E-97	-0.531996875	0.857	0.982	5.43E-93	HLA-C	3	S100A8_TMo
1318	7.79E-63	-0.53245868	0.429	0.84	1.70E-58	SARAF	3	S100A8_TMo
1319	2.54E-68	-0.539356645	0.401	0.816	5.53E-64	NPM1	3	S100A8_TMo
1320	9.53E-58	-0.539935871	0.131	0.511	2.08E-53	IL18	3	S100A8_TMo
1321	6.08E-52	-0.549523247	0.38	0.77	1.33E-47	HSP90B1	3	S100A8_TMo
1322	7.98E-56	-0.554886542	0.315	0.714	1.74E-51	IGSF6	3	S100A8_TMo
1323	8.40E-58	-0.558376988	0.206	0.61	1.83E-53	PLD3	3	S100A8_TMo
1324	2.63E-77	-0.563550923	0.88	0.989	5.73E-73	MT-ND4L	3	S100A8_TMo
1325	3.21E-48	-0.56957954	0.309	0.675	7.00E-44	CDKN1A	3	S100A8_TMo
1326	2.14E-73	-0.574153104	0.119	0.551	4.67E-69	SNX5	3	S100A8_TMo
1327	1.30E-68	-0.581855398	0.101	0.509	2.84E-64	HLA-DMB	3	S100A8_TMo
1328	4.46E-64	-0.592288329	0.321	0.762	9.72E-60	UBB	3	S100A8_TMo
1329	2.06E-53	-0.592858721	0.284	0.679	4.48E-49	DDIT4	3	S100A8_TMo
1330	3.35E-125	-0.596178983	0.895	0.99	7.31E-121	HLA-B	3	S100A8_TMo
1331	1.31E-69	-0.601194793	0.102	0.513	2.87E-65	TCN2	3	S100A8_TMo
1332	2.18E-56	-0.601748859	0.297	0.704	4.76E-52	NINJ1	3	S100A8_TMo
1333	2.89E-49	-0.617187088	0.131	0.468	6.31E-45	GPR183	3	S100A8_TMo
1334	2.08E-102	-0.62895909	0.665	0.952	4.54E-98	YBX1	3	S100A8_TMo
1335	4.93E-70	-0.636908437	0.267	0.713	1.07E-65	CALR	3	S100A8_TMo
1336	7.62E-72	-0.636912954	0.79	0.956	1.66E-67	ZFP36	3	S100A8_TMo
1337	1.53E-61	-0.639598539	0.254	0.661	3.34E-57	ID2	3	S100A8_TMo
1338	1.04E-67	-0.643874168	0.395	0.834	2.28E-63	HSP90AB1	3	S100A8_TMo
1339	4.06E-85	-0.646478813	0.739	0.968	8.85E-81	MT-ATP8	3	S100A8_TMo
1340	6.27E-56	-0.646756919	0.482	0.84	1.37E-51	RHOB	3	S100A8_TMo
1341	3.69E-97	-0.648327901	0.763	0.969	8.04E-93	ITM2B	3	S100A8_TMo
1342	1.63E-77	-0.65024085	0.52	0.9	3.56E-73	GLUL	3	S100A8_TMo
1343	1.84E-103	-0.659694485	0.965	0.999	4.01E-99	CST3	3	S100A8_TMo
1344	3.24E-60	-0.685186437	0.212	0.624	7.07E-56	HSPE1	3	S100A8_TMo
1345	6.37E-79	-0.686423482	0.224	0.685	1.39E-74	CPVL	3	S100A8_TMo
1346	1.74E-38	-0.695970344	0.351	0.677	3.79E-34	HSPA5	3	S100A8_TMo
1347	8.92E-82	-0.6972018	0.126	0.59	1.95E-77	CLEC2B	3	S100A8_TMo
1348	7.89E-150	-0.699176662	0.997	1	1.72E-145	FTL	3	S100A8_TMo

1349	1.03E-50	-0.699489213	0.195	0.553	2.25E-46	CHMP1B	3	S100A8_TMo
1350	3.28E-132	-0.709560297	0.949	0.999	7.15E-128	SAT1	3	S100A8_TMo
1351	1.63E-91	-0.711776415	0.685	0.949	3.56E-87	CD163	3	S100A8_TMo
1352	3.96E-84	-0.724140246	0.401	0.848	8.63E-80	ZFP36L1	3	S100A8_TMo
1353	8.10E-83	-0.731455917	0.14	0.602	1.77E-78	FAM26F	3	S100A8_TMo
1354	2.84E-103	-0.731658951	0.551	0.923	6.20E-99	HLA-E	3	S100A8_TMo
1355	4.92E-77	-0.737155809	0.105	0.536	1.07E-72	MCOLN1	3	S100A8_TMo
1356	3.47E-56	-0.753640018	0.123	0.492	7.58E-52	ICAM1	3	S100A8_TMo
1357	7.11E-61	-0.756511862	0.237	0.638	1.55E-56	SGK1	3	S100A8_TMo
1358	5.37E-133	-0.75921114	0.734	0.968	1.17E-128	HLA-A	3	S100A8_TMo
1359	2.10E-73	-0.78257565	0.173	0.608	4.59E-69	NR4A1	3	S100A8_TMo
1360	2.78E-100	-0.786253949	0.128	0.647	6.07E-96	CXCL16	3	S100A8_TMo
1361	1.90E-92	-0.792274189	0.404	0.846	4.15E-88	HSPA8	3	S100A8_TMo
1362	8.71E-82	-0.794018012	0.299	0.737	1.90E-77	CREG1	3	S100A8_TMo
1363	3.98E-76	-0.806839888	0.212	0.661	8.69E-72	CD83	3	S100A8_TMo
1364	4.02E-85	-0.820652183	0.646	0.918	8.77E-81	MS4A6A	3	S100A8_TMo
1365	2.82E-90	-0.822105718	0.461	0.872	6.14E-86	FCGRT	3	S100A8_TMo
1366	7.29E-59	-0.865298079	0.186	0.581	1.59E-54	HSPD1	3	S100A8_TMo
1367	3.85E-141	-0.871698875	0.808	0.97	8.39E-137	PSAP	3	S100A8_TMo
1368	2.20E-49	-0.875767354	0.38	0.716	4.80E-45	JUN	3	S100A8_TMo
1369	3.63E-115	-0.895859723	0.366	0.848	7.93E-111	RNASET2	3	S100A8_TMo
1370	4.80E-79	-0.915314772	0.11	0.545	1.05E-74	FABP5	3	S100A8_TMo
1371	1.92E-145	-0.918639063	0.662	0.964	4.18E-141	NPC2	3	S100A8_TMo
1372	7.68E-40	-0.933263545	0.116	0.393	1.68E-35	CCL3L3	3	S100A8_TMo
1373	3.57E-50	-0.970129397	0.272	0.614	7.79E-46	CXCL2	3	S100A8_TMo
1374	1.24E-64	-0.988762157	0.556	0.893	2.70E-60	HSP90AA1	3	S100A8_TMo
1375	1.67E-101	-1.000331275	0.39	0.848	3.63E-97	GADD45B	3	S100A8_TMo
1376	4.42E-131	-1.009886358	0.628	0.947	9.64E-127	CTSB	3	S100A8_TMo
1377	7.89E-63	-1.012018583	0.168	0.559	1.72E-58	HSPA1B	3	S100A8_TMo
1378	8.39E-139	-1.029648743	0.62	0.949	1.83E-134	CD68	3	S100A8_TMo
1379	5.63E-115	-1.034692079	0.237	0.765	1.23E-110	CTSC	3	S100A8_TMo
1380	4.40E-45	-1.075148539	0.164	0.47	9.59E-41	IL1B	3	S100A8_TMo
1381	9.31E-143	-1.082682304	0.162	0.757	2.03E-138	HLA-DMA	3	S100A8_TMo
1382	4.26E-52	-1.140235463	0.149	0.49	9.30E-48	CXCL3	3	S100A8_TMo
1383	5.64E-78	-1.192277175	0.189	0.627	1.23E-73	DNAJB1	3	S100A8_TMo
1384	2.39E-122	-1.225877716	0.267	0.787	5.22E-118	HMOX1	3	S100A8_TMo
1385	3.24E-108	-1.233495146	0.186	0.675	7.06E-104	MS4A4A	3	S100A8_TMo
1386	5.16E-118	-1.235341495	0.119	0.673	1.13E-113	CTSL	3	S100A8_TMo
1387	1.60E-134	-1.240699939	0.231	0.774	3.48E-130	MS4A7	3	S100A8_TMo
1388	1.83E-59	-1.280303116	0.177	0.541	3.98E-55	CCL3	3	S100A8_TMo
1389	5.04E-62	-1.311913201	0.128	0.507	1.10E-57	HSPB1	3	S100A8_TMo
1390	2.90E-170	-1.440009498	0.227	0.826	6.32E-166	HLA-DQB1	3	S100A8_TMo
1391	6.90E-207	-1.444609725	0.596	0.968	1.50E-202	HLA-DRB1	3	S100A8_TMo
1392	9.45E-56	-1.478677436	0.194	0.535	2.06E-51	CCL4	3	S100A8_TMo
1393	7.64E-96	-1.504531726	0.3	0.758	1.67E-91	HSPA1A	3	S100A8_TMo
1394	2.01E-234	-1.539491666	0.892	0.996	4.37E-230	CD74	3	S100A8_TMo
1395	4.32E-227	-1.575130411	0.667	0.982	9.41E-223	HLA-DRA	3	S100A8_TMo
1396	3.16E-156	-1.724554752	0.191	0.771	6.90E-152	LIPA	3	S100A8_TMo
1397	2.92E-202	-1.768795053	0.314	0.891	6.38E-198	HLA-DPA1	3	S100A8_TMo
1398	9.95E-208	-1.954010283	0.38	0.891	2.17E-203	HLA-DRB5	3	S100A8_TMo
1399	3.30E-211	-1.954278921	0.228	0.863	7.19E-207	HLA-DPB1	3	S100A8_TMo
1400	1.30E-30	0.964506021	0.668	0.388	2.85E-26	IL1B	4	IL1B_KC
1401	1.40E-23	0.961308905	0.638	0.406	3.06E-19	CXCL3	4	IL1B_KC
1402	3.18E-64	0.874168552	0.967	0.814	6.93E-60	IFITM3	4	IL1B_KC
1403	1.28E-56	0.816597079	0.908	0.616	2.79E-52	CD52	4	IL1B_KC
1404	8.59E-18	0.706978322	0.739	0.532	1.87E-13	CXCL2	4	IL1B_KC
1405	2.18E-54	0.675841215	0.828	0.467	4.75E-50	LILRB1	4	IL1B_KC
1406	2.33E-50	0.669374141	0.908	0.633	5.07E-46	LINC01272	4	IL1B_KC
1407	1.10E-43	0.65118782	0.585	0.251	2.41E-39	RHOC	4	IL1B_KC
1408	1.71E-62	0.641296207	0.97	0.805	3.73E-58	SERPINA1	4	IL1B_KC
1409	4.85E-62	0.630611874	0.985	0.872	1.06E-57	LST1	4	IL1B_KC
1410	2.86E-56	0.625500647	0.95	0.531	6.24E-52	FCGR3A	4	IL1B_KC
1411	2.87E-08	0.597249262	0.605	0.458	0.000626351	CCL4	4	IL1B_KC
1412	1.05E-16	0.59711681	0.436	0.231	2.29E-12	TNF	4	IL1B_KC
1413	4.99E-37	0.595292024	0.95	0.688	1.09E-32	BCL2A1	4	IL1B_KC
1414	1.47E-35	0.571912067	0.819	0.498	3.20E-31	NR4A1	4	IL1B_KC
1415	3.15E-45	0.553976597	0.902	0.616	6.87E-41	LILRB2	4	IL1B_KC
1416	5.41E-29	0.544034513	0.958	0.852	1.18E-24	IFITM2	4	IL1B_KC
1417	5.44E-57	0.539691482	0.991	0.929	1.19E-52	COTL1	4	IL1B_KC
1418	4.87E-39	0.534522668	0.825	0.542	1.06E-34	CD48	4	IL1B_KC
1419	1.09E-28	0.532918885	0.813	0.569	2.38E-24	NFKBIZ	4	IL1B_KC
1420	1.14E-38	0.515829464	0.588	0.259	2.49E-34	WARS	4	IL1B_KC
1421	1.39E-38	0.503018871	0.662	0.329	3.04E-34	LY6E	4	IL1B_KC
1422	1.59E-07	0.480956884	0.136	0.063	0.003472895	MIR155HG	4	IL1B_KC
1423	6.00E-66	0.449951227	0.997	0.972	1.31E-61	RPS19	4	IL1B_KC

1424	6.48E-31	0.437489027	0.772	0.463	1.41E-26	CNIH4	4	IL1B_KC
1425	6.43E-26	0.430224642	0.697	0.422	1.40E-21	CYTIP	4	IL1B_KC
1426	4.52E-20	0.421698305	0.935	0.757	9.86E-16	RHOB	4	IL1B_KC
1427	5.42E-26	0.410519632	0.739	0.471	1.18E-21	PSME2	4	IL1B_KC
1428	2.33E-63	0.406776365	1	0.995	5.08E-59	EEF1A1	4	IL1B_KC
1429	5.49E-32	0.40212008	0.57	0.261	1.20E-27	TCF7L2	4	IL1B_KC
1430	7.53E-43	0.396481734	0.991	0.935	1.64E-38	PSAP	4	IL1B_KC
1431	5.74E-25	0.395858093	0.875	0.612	1.25E-20	NAP1L1	4	IL1B_KC
1432	4.32E-23	0.382590831	0.442	0.203	9.42E-19	BIRC3	4	IL1B_KC
1433	6.49E-34	0.379500642	0.588	0.266	1.42E-29	ICAM2	4	IL1B_KC
1434	1.31E-19	0.376859739	0.935	0.77	2.86E-15	C5AR1	4	IL1B_KC
1435	3.90E-22	0.376802652	0.718	0.464	8.49E-18	PSMB9	4	IL1B_KC
1436	2.44E-27	0.370035887	0.647	0.354	5.32E-23	ITGA4	4	IL1B_KC
1437	2.68E-26	0.367968081	0.837	0.564	5.85E-22	SLC7A7	4	IL1B_KC
1438	7.24E-25	0.367703159	0.772	0.524	1.58E-20	ZNF706	4	IL1B_KC
1439	3.02E-26	0.363030571	0.843	0.561	6.58E-22	CHCHD10	4	IL1B_KC
1440	1.41E-29	0.362475056	0.991	0.764	3.07E-25	HLA-DPA1	4	IL1B_KC
1441	1.20E-16	0.35845787	0.697	0.484	2.62E-12	VAMP5	4	IL1B_KC
1442	3.92E-08	0.355509577	0.516	0.367	0.000855161	DUSP2	4	IL1B_KC
1443	2.21E-24	0.355454362	0.955	0.745	4.82E-20	HSPA8	4	IL1B_KC
1444	2.29E-30	0.354823003	0.546	0.256	4.99E-26	CAMK1	4	IL1B_KC
1445	2.54E-11	0.352260607	0.404	0.235	5.55E-07	PTGS2	4	IL1B_KC
1446	3.28E-25	0.352133391	0.76	0.471	7.15E-21	PILRA	4	IL1B_KC
1447	1.67E-21	0.350598088	0.445	0.22	3.64E-17	PTGER4	4	IL1B_KC
1448	1.74E-35	0.345946381	0.988	0.89	3.80E-31	YBX1	4	IL1B_KC
1449	8.85E-18	0.341092467	0.626	0.392	1.93E-13	NAAA	4	IL1B_KC
1450	1.63E-15	0.340266031	0.78	0.558	3.55E-11	CD83	4	IL1B_KC
1451	8.21E-29	0.337200879	0.442	0.183	1.79E-24	SIDT2	4	IL1B_KC
1452	1.10E-23	0.337121087	0.585	0.309	2.41E-19	9-Sep	4	IL1B_KC
1453	3.40E-20	0.331972705	0.733	0.469	7.41E-16	DOK2	4	IL1B_KC
1454	1.63E-25	0.326663331	0.579	0.295	3.54E-21	ABI3	4	IL1B_KC
1455	1.13E-26	0.324134602	0.386	0.155	2.45E-22	GPBAR1	4	IL1B_KC
1456	1.54E-18	0.322874803	0.347	0.158	3.37E-14	FAM110A	4	IL1B_KC
1457	6.25E-19	0.321925214	0.893	0.645	1.36E-14	CTSC	4	IL1B_KC
1458	3.83E-26	0.320580351	0.493	0.225	8.36E-22	GCH1	4	IL1B_KC
1459	2.67E-20	0.319073956	0.703	0.432	5.82E-16	MTSS1	4	IL1B_KC
1460	2.50E-22	0.317653882	0.947	0.776	5.44E-18	SLC25A5	4	IL1B_KC
1461	4.54E-14	0.312018196	0.78	0.578	9.91E-10	CXCR4	4	IL1B_KC
1462	1.04E-20	0.311351572	0.573	0.325	2.28E-16	RAB24	4	IL1B_KC
1463	1.58E-25	0.3112194	0.896	0.659	3.45E-21	CD37	4	IL1B_KC
1464	8.38E-24	0.311148949	0.51	0.25	1.83E-19	EVL	4	IL1B_KC
1465	1.46E-16	0.310963515	0.611	0.36	3.18E-12	ADGRE2	4	IL1B_KC
1466	9.98E-20	0.306937585	0.662	0.4	2.18E-15	PAG1	4	IL1B_KC
1467	2.15E-28	0.306429937	0.267	0.078	4.70E-24	RP11-362F19.1	4	IL1B_KC
1468	6.01E-18	0.305522147	0.724	0.479	1.31E-13	LRRC25	4	IL1B_KC
1469	3.98E-20	0.305239093	0.688	0.425	8.67E-16	STX11	4	IL1B_KC
1470	3.02E-32	0.303688204	0.997	0.957	6.60E-28	AIF1	4	IL1B_KC
1471	1.88E-23	0.303638054	0.958	0.866	4.10E-19	SLC25A6	4	IL1B_KC
1472	5.12E-12	0.302224482	0.43	0.254	1.12E-07	PAPSS2	4	IL1B_KC
1473	1.22E-27	0.297870422	0.294	0.093	2.66E-23	SLC2A6	4	IL1B_KC
1474	2.81E-15	0.295107739	0.534	0.324	6.13E-11	PECAM1	4	IL1B_KC
1475	3.41E-24	0.291625571	0.985	0.721	7.44E-20	HLA-DPB1	4	IL1B_KC
1476	6.67E-20	0.29150631	0.389	0.186	1.46E-15	ADK	4	IL1B_KC
1477	1.46E-18	0.287504203	0.923	0.732	3.19E-14	MT2A	4	IL1B_KC
1478	2.81E-19	0.285126632	0.638	0.375	6.12E-15	BID	4	IL1B_KC
1479	6.43E-15	0.284307053	0.54	0.327	1.40E-10	BIN2	4	IL1B_KC
1480	9.87E-16	0.282577423	0.504	0.283	2.15E-11	FBP1	4	IL1B_KC
1481	2.79E-15	0.279664362	0.62	0.4	6.08E-11	HN1	4	IL1B_KC
1482	5.93E-13	0.279567999	0.172	0.064	1.29E-08	IFITM1	4	IL1B_KC
1483	3.83E-16	0.275066665	0.941	0.82	8.35E-12	BTG1	4	IL1B_KC
1484	1.22E-18	0.274522767	0.953	0.843	2.67E-14	PTPRC	4	IL1B_KC
1485	1.83E-06	0.273641739	0.944	0.885	0.039874317	NFKBIA	4	IL1B_KC
1486	3.65E-23	0.272303004	0.454	0.21	7.96E-19	LILRA1	4	IL1B_KC
1487	6.07E-16	0.270670482	0.864	0.666	1.32E-11	PRELID1	4	IL1B_KC
1488	5.67E-11	0.270286596	0.914	0.724	1.24E-06	PPP1R15A	4	IL1B_KC
1489	7.22E-21	0.269632838	0.24	0.082	1.57E-16	SPN	4	IL1B_KC
1490	2.14E-17	0.268832714	0.843	0.603	4.67E-13	CARD16	4	IL1B_KC
1491	1.15E-15	0.267082789	0.715	0.468	2.52E-11	RNF144B	4	IL1B_KC
1492	7.50E-17	0.265751519	0.335	0.151	1.63E-12	ISG20	4	IL1B_KC
1493	2.18E-07	0.264514027	0.769	0.632	0.004749854	TXNIP	4	IL1B_KC
1494	7.57E-21	0.264108621	0.371	0.159	1.65E-16	CDC42EP2	4	IL1B_KC
1495	5.63E-16	0.263901999	0.448	0.243	1.23E-11	OAZ2	4	IL1B_KC
1496	6.57E-14	0.263471274	0.804	0.58	1.43E-09	RAP1B	4	IL1B_KC
1497	3.22E-18	0.259375173	0.362	0.169	7.03E-14	TBC1D8	4	IL1B_KC
1498	2.17E-16	0.259340866	0.763	0.492	4.73E-12	FAM26F	4	IL1B_KC

1499	1.34E-13	0.258582176	0.677	0.457	2.93E-09	LILRA2	4	IL1B_KC
1500	4.60E-22	0.256520365	1	0.988	1.00E-17	SAT1	4	IL1B_KC
1501	2.27E-15	0.255860906	0.789	0.575	4.94E-11	TNFRSF1B	4	IL1B_KC
1502	5.65E-13	0.253918364	0.688	0.484	1.23E-08	CASP1	4	IL1B_KC
1503	2.06E-17	0.253514834	0.573	0.333	4.48E-13	POU2F2	4	IL1B_KC
1504	3.48E-10	0.252016421	0.641	0.447	7.60E-06	MCTP1	4	IL1B_KC
1505	8.26E-09	-0.250400028	0.608	0.679	0.000180114	TXN	4	IL1B_KC
1506	4.08E-14	-0.26499366	0.994	0.994	8.89E-10	MT-ND4	4	IL1B_KC
1507	1.58E-07	-0.286282871	0.864	0.818	0.003446946	GRN	4	IL1B_KC
1508	2.63E-08	-0.305328338	0.151	0.285	0.000574085	RGL1	4	IL1B_KC
1509	8.45E-09	-0.313707853	0.591	0.653	0.000184316	NAIP	4	IL1B_KC
1510	8.30E-09	-0.313792919	0.825	0.779	0.000181088	BLVRB	4	IL1B_KC
1511	2.75E-07	-0.338950272	0.884	0.867	0.006003971	MS4A6A	4	IL1B_KC
1512	6.47E-08	-0.356730517	0.217	0.342	0.001411565	DAB2	4	IL1B_KC
1513	7.70E-07	-0.373308037	0.439	0.505	0.016788235	CSF3R	4	IL1B_KC
1514	1.30E-11	-0.379590753	0.777	0.8	2.83E-07	CD14	4	IL1B_KC
1515	1.45E-06	-0.383253805	0.884	0.826	0.031587505	SERPINB1	4	IL1B_KC
1516	8.43E-08	-0.394113292	0.356	0.452	0.00183788	STOM	4	IL1B_KC
1517	1.81E-07	-0.395495975	0.136	0.25	0.00395752	ASPH	4	IL1B_KC
1518	1.05E-10	-0.441637713	0.887	0.787	2.30E-06	FCGRT	4	IL1B_KC
1519	1.03E-10	-0.447961867	0.668	0.698	2.25E-06	APLP2	4	IL1B_KC
1520	9.30E-25	-0.448963385	0.899	0.894	2.03E-20	CTSD	4	IL1B_KC
1521	3.40E-07	-0.461376843	0.282	0.379	0.007415307	CAPG	4	IL1B_KC
1522	2.26E-09	-0.461935012	0.11	0.249	4.92E-05	TMIGD3	4	IL1B_KC
1523	1.15E-12	-0.467952433	0.243	0.415	2.52E-08	STAB1	4	IL1B_KC
1524	3.34E-22	-0.475830912	0.881	0.902	7.29E-18	CD163	4	IL1B_KC
1525	1.69E-12	-0.481479015	0.439	0.546	3.69E-08	PLD3	4	IL1B_KC
1526	4.90E-14	-0.494272156	0.988	0.983	1.07E-09	GAPDH	4	IL1B_KC
1527	1.15E-09	-0.521182994	0.43	0.531	2.51E-05	HMGB2	4	IL1B_KC
1528	3.52E-14	-0.536928237	0.703	0.749	7.69E-10	ANXA1	4	IL1B_KC
1529	2.33E-13	-0.539113308	0.62	0.661	5.08E-09	CREG1	4	IL1B_KC
1530	8.04E-41	-0.550503823	0.861	0.901	1.75E-36	GPX1	4	IL1B_KC
1531	3.91E-11	-0.553420589	0.202	0.346	8.53E-07	MGST1	4	IL1B_KC
1532	7.22E-09	-0.557267583	0.68	0.691	0.000157467	MNDA	4	IL1B_KC
1533	3.71E-23	-0.559211402	0.543	0.713	8.09E-19	CD99	4	IL1B_KC
1534	4.71E-15	-0.575099698	0.154	0.348	1.03E-10	MRC1	4	IL1B_KC
1535	6.81E-11	-0.749564173	0.208	0.348	1.48E-06	SELL	4	IL1B_KC
1536	5.38E-18	-0.783139421	0.427	0.626	1.17E-13	THBS1	4	IL1B_KC
1537	5.98E-17	-0.85360512	0.136	0.351	1.30E-12	IL1R2	4	IL1B_KC
1538	1.18E-11	-0.890878568	0.261	0.394	2.58E-07	LGMN	4	IL1B_KC
1539	4.93E-23	-0.964605784	0.982	0.963	1.08E-18	LYZ	4	IL1B_KC
1540	8.63E-12	-1.128986346	0.187	0.337	1.88E-07	APOC1	4	IL1B_KC
1541	8.92E-22	-1.228692567	0.142	0.386	1.94E-17	SEPP1	4	IL1B_KC
1542	1.63E-16	-1.31784614	0.264	0.433	3.56E-12	SLC40A1	4	IL1B_KC
1543	1.21E-19	-1.572939721	0.451	0.54	2.63E-15	VCAN	4	IL1B_KC
1544	1.32E-09	-1.720712173	0.932	0.88	2.88E-05	S100A9	4	IL1B_KC
1545	7.06E-09	-2.296292833	0.864	0.752	0.000153929	S100A8	4	IL1B_KC
1546	2.25E-39	-2.609308863	0.214	0.532	4.91E-35	S100A12	4	IL1B_KC

Table 4: List of DEGs of mononuclear phagocyte clusters in reperfusion stage (PR versus EP)

List of differentially expressed genes of C1QC_KC in reperfusion stage (PR versus EP)

gene	p_val	avg_logFC	pct.1	pct.2	p_val_adj
SOD2	2.77E-133	1.663902524	0.985	0.77	6.03E-129
HSPD1	1.61E-114	2.02824182	0.937	0.602	3.51E-110
SOCS3	2.70E-102	1.376352647	0.895	0.342	5.88E-98
HSP90AA1	2.48E-97	1.461144222	0.983	0.943	5.42E-93
HSPH1	2.69E-97	1.865338871	0.899	0.547	5.87E-93
BAG3	3.97E-95	1.97348345	0.727	0.117	8.66E-91
HSPE1	1.39E-91	1.679770254	0.918	0.693	3.02E-87
CDKN1A	4.51E-91	1.307995537	0.908	0.5	9.85E-87
HSPB1	1.65E-90	1.988087889	0.91	0.607	3.60E-86
HSPA5	2.04E-90	1.475764387	0.924	0.627	4.45E-86
G0S2	1.16E-89	2.052734071	0.739	0.127	2.53E-85
BATF	1.37E-86	1.171877521	0.668	0.084	3.00E-82
NAMPT	1.38E-85	1.080593087	0.96	0.785	3.02E-81
AREG	1.19E-78	1.488394161	0.859	0.295	2.59E-74
LITAF	6.09E-78	1.167239133	0.891	0.602	1.33E-73
HSPA1A	3.88E-77	1.341964887	0.979	0.908	8.46E-73
TMSB4X	1.32E-73	-0.52927664	0.996	0.998	2.88E-69
CTSL	3.95E-72	1.278804275	0.916	0.734	8.60E-68
THBS1	5.83E-71	1.666782478	0.788	0.346	1.27E-66
ICAM1	3.51E-70	1.390190688	0.83	0.445	7.65E-66
DNAJA4	5.40E-70	1.398185803	0.59	0.076	1.18E-65
SLC40A1	6.35E-69	-1.296704709	0.62	0.9	1.38E-64
H3F3B	3.60E-64	0.663794327	0.989	0.953	7.86E-60
PIM1	1.07E-61	0.939824244	0.616	0.133	2.33E-57
IL1RN	3.64E-60	1.465410564	0.58	0.104	7.94E-56
SERPINB1	6.28E-59	0.940944904	0.889	0.732	1.37E-54
ACSL1	8.01E-59	0.964477786	0.798	0.41	1.75E-54
C15orf48	4.13E-58	1.410413553	0.504	0.055	9.01E-54
SNHG15	4.63E-55	0.889744246	0.685	0.234	1.01E-50
MT2A	8.38E-55	1.571875297	0.939	0.818	1.83E-50
TXNIP	8.54E-55	-0.871119844	0.504	0.863	1.86E-50
GK	8.93E-52	0.950314842	0.712	0.297	1.95E-47
PLAUR	1.55E-51	0.944651039	0.847	0.521	3.39E-47
LMNA	1.09E-50	1.006340238	0.616	0.172	2.37E-46
PDK4	6.32E-50	-1.118541187	0.305	0.732	1.38E-45
HSP90AB1	9.67E-50	0.847867885	0.954	0.859	2.11E-45
GIMAP7	1.14E-49	-0.712306486	0.061	0.484	2.48E-45
LRG1	1.67E-49	0.830455226	0.418	0.031	3.63E-45
IFNGR1	2.48E-49	-0.77303641	0.622	0.891	5.40E-45
SELK	1.29E-48	0.978108387	0.788	0.479	2.82E-44
SLC7A5	2.13E-48	0.692983365	0.433	0.043	4.65E-44
RP11-670E13.6	2.62E-48	0.952515889	0.607	0.188	5.72E-44
MIR3945HG	7.88E-48	0.79327	0.393	0.023	1.72E-43
RELB	1.21E-47	0.709255244	0.508	0.102	2.63E-43
SAMSN1	1.75E-47	0.883626886	0.868	0.648	3.82E-43
SLCO4A1	2.67E-47	0.724323727	0.46	0.066	5.82E-43
MAP3K8	7.31E-47	0.754142294	0.866	0.627	1.59E-42
PNRC1	1.02E-46	0.663766904	0.947	0.867	2.22E-42
PAPSS2	4.12E-46	0.883443462	0.674	0.256	8.97E-42
PIM3	8.98E-46	0.821071701	0.588	0.201	1.96E-41
P2RY13	3.24E-45	-0.738747992	0.124	0.547	7.06E-41
PLSCR1	3.86E-45	0.798811653	0.775	0.459	8.41E-41
ZFAND2A	8.96E-45	1.262070746	0.592	0.215	1.95E-40
ABL2	1.20E-44	0.76777828	0.498	0.107	2.62E-40
TNIP3	1.36E-43	0.796746531	0.328	0.004	2.96E-39
DNAJB1	5.14E-43	1.137459899	0.884	0.699	1.12E-38
CREM	1.80E-42	0.801157543	0.664	0.273	3.93E-38
THBD	5.99E-42	0.884918659	0.546	0.164	1.31E-37
FTH1	9.17E-42	0.432990869	1	1	2.00E-37
PTPN1	1.50E-41	0.73896582	0.666	0.303	3.27E-37
DNAJB6	1.81E-41	0.837782599	0.828	0.57	3.94E-37
CALM2	2.64E-41	-0.566978758	0.834	0.941	5.75E-37
CACYBP	4.61E-41	0.899336052	0.714	0.408	1.00E-36
C1QC	5.15E-40	-0.440556564	0.956	0.971	1.12E-35
FKBP4	6.61E-40	0.911001932	0.517	0.152	1.44E-35
B4GALT5	3.02E-39	0.631880724	0.445	0.09	6.59E-35
BCL3	3.48E-39	0.643141203	0.492	0.123	7.58E-35

ADORA3	3.55E-39	-0.558068286	0.057	0.416	7.75E-35
DNAJA1	5.50E-39	0.773224579	0.847	0.654	1.20E-34
PELI1	9.70E-39	0.849718373	0.739	0.469	2.12E-34
ATP13A3	1.78E-38	0.724700285	0.521	0.158	3.88E-34
NINJ1	2.38E-38	0.818048152	0.87	0.771	5.20E-34
SAT1	4.99E-38	0.442326077	1	0.998	1.09E-33
RPS29	7.97E-38	0.469013796	0.952	0.904	1.74E-33
GIMAP4	1.03E-37	-0.65166274	0.303	0.666	2.26E-33
NFKB1	9.06E-37	0.811781171	0.569	0.23	1.98E-32
SAMHD1	9.84E-37	-0.611592292	0.519	0.807	2.15E-32
BCL2A1	1.44E-36	0.741953013	0.815	0.51	3.13E-32
UPP1	1.09E-35	0.698510379	0.735	0.434	2.38E-31
PNP	1.55E-35	0.772175265	0.534	0.199	3.38E-31
NFKB2	2.13E-35	0.621591853	0.466	0.123	4.65E-31
FPR1	2.70E-35	0.696669612	0.733	0.459	5.89E-31
SLC2A3	6.31E-35	0.767155325	0.628	0.25	1.38E-30
CXCL10	6.97E-35	1.499526913	0.349	0.045	1.52E-30
IRF1	9.94E-35	0.744942255	0.653	0.338	2.17E-30
INSIG1	2.25E-33	0.812904807	0.704	0.381	4.91E-29
CD44	3.84E-33	0.630456957	0.836	0.633	8.38E-29
C1QB	4.60E-33	-0.401959916	0.952	0.977	1.00E-28
MAP2K3	8.30E-33	0.588099889	0.588	0.244	1.81E-28
PDE4B	1.13E-32	0.651074244	0.584	0.242	2.47E-28
FOSL2	1.18E-32	0.637617624	0.565	0.221	2.58E-28
ARPC1B	1.53E-32	-0.449263605	0.815	0.924	3.34E-28
CCRL2	2.20E-32	0.693410933	0.508	0.188	4.81E-28
CPVL	2.31E-32	-0.636149172	0.571	0.807	5.04E-28
TNFRSF10D	2.68E-32	0.535598226	0.334	0.043	5.84E-28
EIF1	3.70E-32	0.305708777	0.998	0.994	8.06E-28
SDC4	7.04E-32	0.627277266	0.336	0.045	1.53E-27
HIF1A	9.13E-32	0.708566583	0.752	0.5	1.99E-27
RP11-295G20.2	9.96E-32	0.586631257	0.351	0.057	2.17E-27
EMP1	2.70E-31	0.762952425	0.387	0.084	5.89E-27
MS4A7	1.13E-30	-0.505931984	0.887	0.939	2.47E-26
RNASE6	1.38E-30	-0.573959894	0.634	0.834	3.01E-26
GIMAP1	1.45E-30	-0.513054693	0.174	0.508	3.17E-26
CD55	1.45E-30	0.675177778	0.788	0.574	3.17E-26
C1orf162	1.56E-30	-0.458774784	0.807	0.93	3.40E-26
EMP3	1.91E-30	0.622149483	0.872	0.77	4.17E-26
DRAM1	2.62E-30	0.587909767	0.553	0.234	5.71E-26
MS4A6A	2.77E-30	-0.432990069	0.96	0.982	6.05E-26
DEFA3	3.14E-30	-0.526149679	0.036	0.316	6.84E-26
ANXA1	7.41E-30	0.993482253	0.769	0.559	1.62E-25
DDIT3	1.67E-29	0.619139645	0.605	0.281	3.64E-25
SBNO2	3.40E-29	0.47922422	0.315	0.049	7.40E-25
C5AR1	4.52E-29	0.607465332	0.882	0.732	9.85E-25
GADD45B	7.71E-29	0.594494284	0.964	0.857	1.68E-24
P4HA1	1.25E-28	0.609540284	0.532	0.23	2.73E-24
B3GNT5	1.56E-28	0.597219553	0.395	0.1	3.39E-24
TWISTNB	2.14E-28	0.895895037	0.481	0.184	4.66E-24
ARID5A	2.21E-28	0.635543221	0.54	0.252	4.83E-24
TRIM25	3.10E-28	0.559978441	0.393	0.104	6.76E-24
WTAP	4.24E-28	0.680469759	0.632	0.375	9.24E-24
PDE4DIP	8.36E-28	0.499999037	0.464	0.162	1.82E-23
TXN	9.12E-28	0.869950333	0.805	0.648	1.99E-23
ARL5B	9.87E-28	0.72842779	0.532	0.234	2.15E-23
SEMA6B	1.06E-27	0.434184178	0.244	0.014	2.32E-23
PVRL2	1.24E-27	0.564747359	0.542	0.242	2.70E-23
SDF2L1	2.67E-27	0.559888651	0.569	0.264	5.82E-23
BIRC3	4.07E-27	0.631737044	0.475	0.172	8.87E-23
PPIF	6.03E-27	0.56562916	0.517	0.217	1.32E-22
VAMP8	6.64E-27	-0.408387699	0.828	0.922	1.45E-22
DNAJB9	7.49E-27	0.563348371	0.557	0.26	1.63E-22
ARHGDIB	1.73E-26	-0.431041797	0.792	0.9	3.77E-22
SDS	4.09E-26	1.055656422	0.433	0.146	8.92E-22
CSF1R	5.72E-26	-0.491091175	0.725	0.857	1.25E-21
SPHK1	6.56E-26	0.417788394	0.286	0.045	1.43E-21
ZFP36L1	6.76E-26	-0.457491706	0.752	0.92	1.47E-21
ZFP36L2	6.88E-26	-0.53686472	0.601	0.852	1.50E-21
TIMP1	8.10E-26	0.869098208	0.918	0.863	1.77E-21

LDLR	1.61E-25	0.459664036	0.286	0.045	3.50E-21
PFKFB3	2.00E-25	0.544814569	0.622	0.324	4.35E-21
TM9SF2	2.29E-25	-0.515388971	0.351	0.654	4.99E-21
CTSS	8.63E-25	-0.383096787	0.941	0.975	1.88E-20
GPR34	9.07E-25	-0.568956465	0.376	0.648	1.98E-20
FGD2	9.99E-25	-0.484948356	0.34	0.631	2.18E-20
NUDC	1.08E-24	0.583509387	0.569	0.301	2.37E-20
HES4	1.28E-24	0.472095297	0.214	0.012	2.79E-20
MTHFD2	2.14E-24	0.595705472	0.628	0.395	4.67E-20
KDM6B	2.40E-24	0.538132615	0.58	0.303	5.24E-20
TUBA1C	6.84E-24	0.519510689	0.513	0.232	1.49E-19
ETS2	8.25E-24	0.537127371	0.813	0.691	1.80E-19
DDX21	9.39E-24	0.564784229	0.691	0.504	2.05E-19
CXCR4	1.63E-23	-0.607260365	0.498	0.754	3.54E-19
PTPN2	1.80E-23	0.525353335	0.618	0.357	3.94E-19
ABI3	1.81E-23	-0.470468547	0.275	0.561	3.96E-19
GABARAP	1.99E-23	-0.323433045	0.956	0.977	4.33E-19
TFRC	2.65E-23	0.679022266	0.437	0.17	5.78E-19
CD163	2.73E-23	-0.350404101	0.956	0.971	5.95E-19
MYADM	5.58E-23	0.581586409	0.576	0.303	1.22E-18
SERPINB9	6.57E-23	0.551877528	0.55	0.281	1.43E-18
WASF2	8.98E-23	-0.475461539	0.353	0.635	1.96E-18
LDHA	9.10E-23	0.519579558	0.796	0.654	1.98E-18
SLC25A37	1.06E-22	0.6315513	0.483	0.223	2.32E-18
MAF	1.13E-22	-0.521429434	0.529	0.789	2.47E-18
SPIC	1.17E-22	-0.541214643	0.17	0.451	2.54E-18
LIPA	1.82E-22	-0.53237123	0.889	0.945	3.97E-18
FUCA1	2.58E-22	-0.57278358	0.288	0.559	5.64E-18
GPCPD1	3.73E-22	0.570068223	0.578	0.328	8.14E-18
PPP1R15A	3.90E-22	0.482392052	0.891	0.695	8.50E-18
MMP19	4.42E-22	0.892097229	0.45	0.189	9.64E-18
EAF1	5.46E-22	0.435105103	0.336	0.092	1.19E-17
EZR	6.22E-22	0.60344131	0.576	0.328	1.36E-17
MAFF	2.02E-21	0.490598945	0.378	0.125	4.40E-17
FRMD4A	2.49E-21	-0.386814696	0.126	0.393	5.43E-17
RNF144B	2.67E-21	0.556155399	0.723	0.5	5.82E-17
HSPA6	4.08E-21	1.382501599	0.464	0.229	8.89E-17
MIDN	4.32E-21	0.461588699	0.632	0.359	9.42E-17
XBP1	5.29E-21	0.488673072	0.748	0.531	1.15E-16
STAT4	5.82E-21	0.324487759	0.183	0.01	1.27E-16
DSE	6.43E-21	0.492692783	0.586	0.336	1.40E-16
MXD1	8.01E-21	0.554783349	0.58	0.332	1.75E-16
ARPC5	8.85E-21	-0.397932464	0.782	0.879	1.93E-16
TNFAIP8L2	9.65E-21	-0.274694985	0.034	0.242	2.11E-16
HHEX	1.17E-20	-0.367648891	0.057	0.279	2.54E-16
OLR1	1.36E-20	0.602820078	0.258	0.049	2.97E-16
STIP1	1.39E-20	0.424754451	0.416	0.17	3.03E-16
UGCG	1.49E-20	0.51077761	0.403	0.162	3.24E-16
SESN1	2.83E-20	-0.37434857	0.179	0.459	6.18E-16
RAB27A	3.14E-20	0.371018176	0.239	0.041	6.86E-16
LIMK2	3.86E-20	0.369782972	0.256	0.051	8.42E-16
LRRFIP1	3.87E-20	0.49904115	0.811	0.662	8.44E-16
DUSP1	4.40E-20	-0.364592806	0.979	0.965	9.58E-16
IRF2	4.41E-20	-0.387571385	0.216	0.492	9.63E-16
MRPL18	4.97E-20	0.597138728	0.489	0.266	1.08E-15
MCTP2	5.20E-20	0.352115317	0.216	0.029	1.13E-15
RBX1	6.04E-20	-0.374730415	0.33	0.598	1.32E-15
SLAMF1	7.10E-20	0.394764654	0.151	0	1.55E-15
RGS1	8.17E-20	-0.556470417	0.332	0.602	1.78E-15
KLHL6	8.35E-20	0.380055103	0.324	0.094	1.82E-15
ZFP36	8.67E-20	0.421662342	0.966	0.936	1.89E-15
ODF3B	8.76E-20	0.469836575	0.523	0.277	1.91E-15
TNFAIP3	1.48E-19	0.531080511	0.79	0.582	3.23E-15
CMKLR1	1.99E-19	-0.327520798	0.097	0.342	4.34E-15
GBP2	2.07E-19	0.509533781	0.494	0.256	4.52E-15
TAOK3	3.09E-19	0.538552395	0.666	0.477	6.73E-15
AHSA1	4.46E-19	0.484150166	0.443	0.219	9.72E-15
DNTTIP2	7.07E-19	0.481648887	0.422	0.199	1.54E-14
REL	7.09E-19	0.498625206	0.79	0.652	1.55E-14
FCGR3A	8.30E-19	-0.351101773	0.847	0.93	1.81E-14

CPM	8.68E-19	-0.512219825	0.689	0.814	1.89E-14
UBC	9.38E-19	0.428594563	0.975	0.924	2.05E-14
ATF3	1.16E-18	0.443494579	0.695	0.441	2.54E-14
FFAR3	1.38E-18	0.448086469	0.183	0.018	3.00E-14
RGCC	1.54E-18	0.55371573	0.357	0.121	3.36E-14
HBB	2.03E-18	-0.622880943	0.12	0.354	4.43E-14
AQP9	2.18E-18	0.419804248	0.197	0.025	4.74E-14
DNAJB11	2.24E-18	0.440812784	0.506	0.268	4.89E-14
LPAR6	2.74E-18	-0.35084417	0.111	0.348	5.97E-14
TMEM2	2.77E-18	0.444718331	0.412	0.172	6.04E-14
CORO1A	3.01E-18	-0.440602498	0.534	0.752	6.57E-14
HAMP	3.48E-18	0.912556752	0.179	0.018	7.59E-14
GCNT1	3.72E-18	-0.351569623	0.084	0.305	8.12E-14
EREG	3.89E-18	0.481314604	0.389	0.139	8.49E-14
PHLDA2	4.36E-18	0.43795249	0.38	0.143	9.51E-14
MIR155HG	4.63E-18	0.630885769	0.252	0.059	1.01E-13
ADM	4.73E-18	0.584823573	0.599	0.35	1.03E-13
RTEL1-TNFRSF6B	4.75E-18	0.352111116	0.147	0.004	1.04E-13
CD274	4.84E-18	0.280898365	0.147	0.004	1.05E-13
STX11	4.97E-18	0.395513706	0.603	0.369	1.08E-13
C10orf54	5.58E-18	-0.360698724	0.672	0.844	1.22E-13
TOM1	5.86E-18	0.429122837	0.439	0.209	1.28E-13
IL4R	6.12E-18	0.416484922	0.313	0.104	1.34E-13
RGS18	6.48E-18	-0.298560177	0.181	0.441	1.41E-13
PLIN2	7.79E-18	0.633290731	0.767	0.645	1.70E-13
TRAPPC1	7.94E-18	-0.405694866	0.357	0.609	1.73E-13
DUSP2	8.37E-18	0.534957378	0.616	0.352	1.83E-13
YBX3	9.10E-18	0.465460986	0.666	0.461	1.99E-13
TRIM14	9.41E-18	-0.289069142	0.084	0.307	2.05E-13
ATP1B3	9.64E-18	0.64237124	0.687	0.537	2.10E-13
GRN	1.21E-17	-0.340481458	0.874	0.93	2.64E-13
MRPS6	1.28E-17	0.537049209	0.511	0.287	2.79E-13
MT1A	1.35E-17	0.681513378	0.139	0.002	2.95E-13
NUCB1	1.37E-17	0.451654066	0.721	0.545	2.99E-13
FOS	1.47E-17	-0.343224659	0.943	0.977	3.20E-13
NFIL3	1.64E-17	0.380864031	0.319	0.104	3.57E-13
ARRDC3	2.08E-17	0.471552568	0.546	0.318	4.54E-13
FPR2	2.17E-17	0.412871349	0.286	0.086	4.74E-13
SLC16A10	2.92E-17	0.327134411	0.174	0.018	6.36E-13
MCL1	3.86E-17	0.31556967	0.929	0.832	8.41E-13
FRMD4B	3.89E-17	-0.354993491	0.357	0.621	8.49E-13
YPEL2	4.34E-17	-0.320512578	0.111	0.34	9.47E-13
GIMAP8	4.92E-17	-0.313357404	0.099	0.318	1.07E-12
STAT3	5.38E-17	0.429396291	0.611	0.396	1.17E-12
NAIP	6.04E-17	-0.336968223	0.658	0.805	1.32E-12
CHMP1B	6.19E-17	0.540709959	0.754	0.584	1.35E-12
NAPA	7.06E-17	0.497995402	0.666	0.49	1.54E-12
RGS19	7.20E-17	-0.363918585	0.271	0.523	1.57E-12
ELL2	8.28E-17	0.502998113	0.597	0.385	1.81E-12
EIF2AK3	9.08E-17	0.365582919	0.286	0.088	1.98E-12
KLF4	9.44E-17	0.426478163	0.866	0.773	2.06E-12
CSRNP1	9.71E-17	0.44610092	0.504	0.279	2.12E-12
MAP2K1	1.03E-16	0.469795099	0.544	0.33	2.26E-12
MERTK	1.13E-16	-0.381418153	0.441	0.676	2.46E-12
IRF5	1.24E-16	-0.323282739	0.118	0.342	2.71E-12
LMNB1	1.25E-16	0.286101238	0.214	0.043	2.73E-12
GPBP1	1.27E-16	0.4341636	0.618	0.424	2.77E-12
WBP5	1.30E-16	0.465114929	0.269	0.078	2.83E-12
UQCR10	1.58E-16	-0.343977047	0.641	0.809	3.44E-12
MS4A4A	1.74E-16	-0.357463845	0.84	0.9	3.79E-12
FAM105A	2.85E-16	-0.330501066	0.13	0.357	6.21E-12
SCIMP	2.93E-16	-0.392277796	0.275	0.51	6.38E-12
APOBEC3A	3.04E-16	0.451478829	0.164	0.018	6.62E-12
HSP90B1	3.09E-16	0.406350124	0.905	0.848	6.74E-12
CH25H	3.29E-16	0.683699755	0.181	0.025	7.17E-12
ZC3H12A	3.78E-16	0.408310718	0.357	0.146	8.25E-12
HVCN1	3.95E-16	-0.321672141	0.181	0.416	8.61E-12
HMGN3	3.99E-16	-0.372742371	0.353	0.604	8.70E-12
ELOVL5	4.00E-16	0.412449575	0.454	0.242	8.72E-12
APBB1IP	4.25E-16	-0.383390598	0.468	0.672	9.26E-12

FEM1C	5.36E-16	0.359145876	0.292	0.098	1.17E-11
MALAT1	5.81E-16	0.357154561	0.985	0.99	1.27E-11
TNFAIP6	5.98E-16	0.507255373	0.206	0.041	1.30E-11
OPTN	6.24E-16	0.266035248	0.145	0.01	1.36E-11
PPT1	6.40E-16	-0.383173678	0.59	0.789	1.40E-11
MPEG1	6.76E-16	-0.398479886	0.59	0.762	1.47E-11
FLT1	6.87E-16	0.286291677	0.145	0.01	1.50E-11
PHLDA1	6.89E-16	0.472269062	0.223	0.051	1.50E-11
SAV1	7.44E-16	0.33868114	0.235	0.057	1.62E-11
CD83	7.64E-16	0.446770632	0.836	0.633	1.67E-11
GPR84	7.72E-16	0.268822875	0.168	0.021	1.68E-11
KIAA1551	7.80E-16	-0.30029911	0.09	0.301	1.70E-11
ADGRE2	8.83E-16	0.494171198	0.601	0.4	1.93E-11
AIF1	8.98E-16	-0.265711367	0.966	0.977	1.96E-11
ELMSAN1	1.02E-15	0.43378807	0.353	0.146	2.21E-11
NDRG1	1.04E-15	0.360811085	0.336	0.133	2.26E-11
HELZ	1.07E-15	-0.327321342	0.21	0.449	2.33E-11
LRP1	1.18E-15	-0.365815465	0.336	0.578	2.58E-11
EHD1	1.26E-15	0.381347165	0.353	0.152	2.75E-11
FYB	1.38E-15	-0.329485946	0.744	0.867	3.00E-11
TUBB4B	1.43E-15	0.434450863	0.563	0.342	3.12E-11
HLA-DPA1	1.48E-15	-0.305281185	0.96	0.984	3.23E-11
CLIC4	1.57E-15	0.328371667	0.351	0.145	3.42E-11
DNMBP	2.02E-15	0.328949846	0.212	0.047	4.40E-11
PPARD	2.31E-15	0.257325152	0.227	0.053	5.04E-11
TCP1	2.47E-15	0.439119758	0.586	0.398	5.38E-11
RALGDS	3.40E-15	0.362037065	0.269	0.084	7.41E-11
SERPINH1	3.46E-15	0.400333781	0.151	0.016	7.55E-11
MAP3K1	3.59E-15	-0.300420307	0.128	0.344	7.82E-11
CREB5	4.86E-15	0.374117326	0.273	0.088	1.06E-10
GMFG	6.65E-15	-0.355121893	0.59	0.748	1.45E-10
ARID1A	8.56E-15	-0.326655894	0.13	0.34	1.87E-10
KCTD12	9.84E-15	-0.384185053	0.433	0.676	2.15E-10
GNA13	1.04E-14	0.38720939	0.529	0.312	2.27E-10
RASGEF1B	1.20E-14	0.392487494	0.584	0.387	2.61E-10
PTPN6	1.26E-14	-0.353337756	0.471	0.678	2.74E-10
CCL18	1.27E-14	0.849474372	0.139	0.012	2.78E-10
AZIN1	1.36E-14	0.40573207	0.571	0.377	2.97E-10
LUCAT1	1.45E-14	0.265743903	0.172	0.027	3.15E-10
DOCK8	1.56E-14	-0.326232927	0.265	0.498	3.40E-10
ISG20	1.59E-14	0.412814499	0.313	0.123	3.48E-10
IL1B	2.58E-14	0.659868913	0.576	0.35	5.62E-10
VIM	2.60E-14	0.287006402	0.983	0.986	5.67E-10
PIGA	3.08E-14	0.370624619	0.237	0.07	6.71E-10
CHSY1	3.09E-14	0.272026496	0.271	0.088	6.75E-10
NEDD8	3.35E-14	-0.363635416	0.454	0.668	7.31E-10
LAP3	4.15E-14	0.365433687	0.637	0.477	9.04E-10
GPR65	4.31E-14	-0.325651968	0.382	0.623	9.40E-10
SULT1A1	4.49E-14	-0.375260777	0.41	0.625	9.79E-10
CD84	5.35E-14	-0.411457688	0.376	0.588	1.17E-09
BAZ1A	5.75E-14	0.471625485	0.605	0.443	1.25E-09
AKR1A1	6.02E-14	-0.351350469	0.414	0.627	1.31E-09
MAPK6	6.57E-14	0.337530823	0.332	0.143	1.43E-09
ZNF106	6.74E-14	-0.355995655	0.239	0.463	1.47E-09
KMO	8.23E-14	0.455800347	0.351	0.158	1.80E-09
GLIPR1	9.01E-14	-0.360390982	0.55	0.725	1.97E-09
RABGEF1	9.23E-14	0.252197149	0.202	0.049	2.01E-09
RBPJ	9.29E-14	-0.34706586	0.46	0.682	2.03E-09
BTG3	1.14E-13	0.270740998	0.267	0.09	2.48E-09
SLC3A2	1.14E-13	0.367490192	0.674	0.502	2.48E-09
PHACTR1	1.15E-13	0.396126862	0.481	0.277	2.51E-09
IRAK2	1.26E-13	0.258360884	0.185	0.039	2.74E-09
PTP4A1	1.28E-13	0.433301845	0.59	0.4	2.78E-09
SNX6	1.29E-13	-0.347633229	0.645	0.795	2.81E-09
SOCS1	1.39E-13	0.264234086	0.244	0.074	3.02E-09
PLK3	1.43E-13	0.34731622	0.37	0.178	3.11E-09
CSK	1.45E-13	-0.312599574	0.254	0.479	3.16E-09
MIS18BP1	1.45E-13	-0.302175153	0.204	0.424	3.16E-09
FAM107B	1.49E-13	0.379141259	0.33	0.145	3.25E-09
METRNL	1.56E-13	0.3301511	0.317	0.131	3.40E-09

TMEM37	1.58E-13	-0.33040519	0.244	0.465	3.45E-09
FAM49A	1.61E-13	0.355571886	0.603	0.412	3.52E-09
DNAAF1	1.73E-13	0.308992656	0.134	0.014	3.77E-09
HIVEP1	1.78E-13	0.289352984	0.233	0.068	3.89E-09
LAMTOR4	1.89E-13	-0.294225849	0.695	0.859	4.12E-09
GABARAPL2	1.97E-13	-0.284884011	0.401	0.633	4.30E-09
ABRACL	2.03E-13	-0.346895295	0.58	0.766	4.42E-09
SLC2A6	2.16E-13	0.281315916	0.2	0.051	4.71E-09
GIMAP2	2.69E-13	-0.320804756	0.195	0.408	5.87E-09
UBAP1	2.74E-13	0.320458419	0.334	0.15	5.98E-09
VEGFA	3.42E-13	0.346209036	0.288	0.109	7.45E-09
NANS	3.58E-13	0.394475488	0.563	0.398	7.81E-09
ID2	3.59E-13	-0.377240969	0.672	0.799	7.83E-09
NSL1	3.65E-13	-0.308386188	0.193	0.41	7.96E-09
ADGRE5	3.78E-13	0.461901322	0.632	0.492	8.25E-09
ADAP2	3.88E-13	-0.363473374	0.456	0.648	8.46E-09
ATP2A2	4.03E-13	0.367318392	0.487	0.293	8.79E-09
OTUD1	4.21E-13	0.439875392	0.393	0.205	9.18E-09
PDE4A	4.54E-13	0.324534634	0.261	0.096	9.90E-09
CALR	4.56E-13	0.329443707	0.84	0.768	9.94E-09
DDX17	4.59E-13	-0.324898776	0.422	0.643	1.00E-08
ATP1A1	5.23E-13	0.406576526	0.565	0.375	1.14E-08
ST8SIA4	5.39E-13	-0.306681507	0.191	0.41	1.18E-08
HCLS1	5.40E-13	-0.272392643	0.874	0.912	1.18E-08
RAPGEF1	5.74E-13	0.30770165	0.271	0.104	1.25E-08
SNN	6.09E-13	0.311646748	0.315	0.133	1.33E-08
HLA-DMB	6.91E-13	-0.333356883	0.496	0.719	1.51E-08
PYCARD	7.08E-13	-0.286798536	0.721	0.859	1.54E-08
NCKAP1L	7.09E-13	-0.364012622	0.332	0.531	1.55E-08
SFT2D2	7.53E-13	-0.311830242	0.212	0.43	1.64E-08
POLD4	7.55E-13	-0.34160001	0.359	0.566	1.65E-08
MAT2B	8.08E-13	-0.34060409	0.246	0.455	1.76E-08
LSM6	9.40E-13	-0.332340486	0.191	0.396	2.05E-08
METTL7A	1.00E-12	-0.341265142	0.372	0.59	2.19E-08
SPOP	1.12E-12	-0.258834695	0.103	0.283	2.43E-08
TNFSF10	1.18E-12	-0.254231339	0.116	0.303	2.57E-08
HERPUD1	1.23E-12	0.328525858	0.882	0.836	2.68E-08
ADCY7	1.30E-12	-0.296600592	0.164	0.375	2.83E-08
MANF	1.32E-12	0.352181964	0.408	0.211	2.88E-08
MIR22HG	1.33E-12	0.33880048	0.319	0.139	2.91E-08
XRCC5	1.34E-12	-0.332808075	0.319	0.525	2.91E-08
MIR4435-2HG	1.43E-12	0.400429756	0.38	0.195	3.12E-08
SCP2	1.45E-12	-0.307848466	0.401	0.625	3.15E-08
PTX3	1.45E-12	0.444414755	0.16	0.029	3.16E-08
RHOBTB1	1.49E-12	-0.266320792	0.069	0.232	3.25E-08
STAB1	1.50E-12	-0.397484054	0.391	0.594	3.26E-08
PTGES3	1.68E-12	0.451265501	0.824	0.779	3.65E-08
PHKB	1.70E-12	-0.279163717	0.105	0.283	3.70E-08
MRPS18B	1.74E-12	-0.260911052	0.132	0.326	3.80E-08
GCLC	1.87E-12	-0.289246303	0.164	0.363	4.08E-08
ATM	1.96E-12	-0.293060445	0.195	0.406	4.28E-08
SLCO2B1	2.44E-12	-0.336993469	0.311	0.525	5.33E-08
RNF135	2.49E-12	-0.278137632	0.197	0.402	5.43E-08
CTSZ	2.55E-12	0.423433185	0.739	0.627	5.56E-08
RCSD1	2.66E-12	-0.308029784	0.317	0.531	5.79E-08
PNPLA8	3.12E-12	0.388900033	0.479	0.291	6.80E-08
WARS	3.12E-12	0.420219524	0.412	0.236	6.80E-08
IGSF6	3.24E-12	-0.378066817	0.693	0.832	7.06E-08
CDKN1B	3.28E-12	-0.316813569	0.279	0.486	7.15E-08
TCF7L2	3.44E-12	-0.380445029	0.284	0.484	7.49E-08
NEAT1	3.47E-12	0.264577708	0.979	0.967	7.58E-08
KRCC1	3.54E-12	-0.250947886	0.149	0.348	7.72E-08
FLI1	3.63E-12	-0.320353314	0.256	0.463	7.92E-08
FCGR1A	3.73E-12	0.402722161	0.567	0.391	8.14E-08
RSF1	3.92E-12	-0.258053218	0.147	0.346	8.55E-08
KCNK6	4.03E-12	-0.285825954	0.09	0.26	8.78E-08
SCAMP2	4.13E-12	-0.303213928	0.313	0.529	9.01E-08
HMGN2	4.29E-12	-0.352033293	0.708	0.83	9.36E-08
MT-ATP8	4.41E-12	0.287188035	0.985	0.967	9.61E-08
FAM26F	4.43E-12	-0.293667598	0.714	0.818	9.66E-08

GLUL	4.43E-12	0.381636096	0.945	0.943	9.67E-08
NPEPPS	4.96E-12	-0.291185788	0.151	0.338	1.08E-07
CCNL1	4.98E-12	0.377168391	0.779	0.643	1.09E-07
SAA1	5.05E-12	0.485254598	0.244	0.09	1.10E-07
FAM96A	5.62E-12	-0.313513248	0.5	0.693	1.23E-07
MBOAT7	5.91E-12	0.305219897	0.286	0.119	1.29E-07
GNPDA1	6.26E-12	-0.341028199	0.248	0.455	1.36E-07
SLC38A10	6.71E-12	-0.264797041	0.13	0.312	1.46E-07
UTRN	7.02E-12	-0.35704813	0.426	0.609	1.53E-07
TRIB1	7.74E-12	0.376828193	0.479	0.287	1.69E-07
WWP1	7.81E-12	-0.302189695	0.244	0.453	1.70E-07
SYAP1	7.84E-12	0.401836924	0.502	0.32	1.71E-07
DUSP6	9.07E-12	-0.295044102	0.42	0.637	1.98E-07
NRP2	9.66E-12	0.299296121	0.261	0.098	2.11E-07
ANKRD28	1.00E-11	0.272490551	0.231	0.078	2.19E-07
LACTB	1.06E-11	0.328663577	0.418	0.24	2.32E-07
EPN1	1.15E-11	-0.32374619	0.361	0.559	2.51E-07
ABCC5	1.16E-11	-0.317445678	0.229	0.424	2.53E-07
STX10	1.28E-11	-0.266775602	0.185	0.383	2.80E-07
KPNB1	1.32E-11	-0.287514952	0.307	0.516	2.88E-07
IL10	1.37E-11	0.626453509	0.328	0.154	2.98E-07
TCEB1	1.50E-11	0.386579465	0.607	0.459	3.27E-07
LBR	1.65E-11	-0.330844011	0.204	0.389	3.60E-07
TREM1	1.76E-11	0.295175833	0.275	0.111	3.85E-07
ZC3HAV1	1.78E-11	0.408393364	0.477	0.297	3.88E-07
TMEM70	1.96E-11	0.356695298	0.441	0.277	4.28E-07
FCGR2A	2.03E-11	0.392800708	0.59	0.424	4.43E-07
LEPROT	2.07E-11	-0.321188851	0.328	0.525	4.51E-07
CCL3L3	2.08E-11	-0.598627452	0.34	0.521	4.55E-07
SNX17	2.29E-11	-0.296054363	0.267	0.455	5.00E-07
ZBTB43	2.59E-11	0.292229381	0.254	0.1	5.64E-07
PSMB10	2.59E-11	-0.269108658	0.292	0.5	5.65E-07
MT1E	2.86E-11	0.675723438	0.296	0.127	6.23E-07
HSPA4	2.91E-11	0.428059655	0.441	0.285	6.35E-07
MT1F	2.96E-11	0.322408093	0.187	0.053	6.45E-07
MPHOSPH6	3.08E-11	0.32188055	0.279	0.125	6.72E-07
RIPK2	3.08E-11	0.381291597	0.41	0.248	6.72E-07
GSTP1	3.09E-11	-0.307066072	0.824	0.906	6.75E-07
LYZ	3.30E-11	-0.285429889	0.897	0.949	7.20E-07
MYO1G	3.32E-11	0.316619349	0.351	0.178	7.24E-07
IL10RA	3.37E-11	-0.273727245	0.473	0.658	7.34E-07
EIF5	3.44E-11	0.316775164	0.758	0.668	7.51E-07
FAM120A	3.56E-11	-0.262133651	0.29	0.494	7.77E-07
PRKCD	3.61E-11	-0.254465794	0.149	0.332	7.88E-07
USP36	3.61E-11	0.337225925	0.282	0.123	7.88E-07
PAPD5	4.05E-11	0.326450856	0.29	0.129	8.83E-07
MLKL	4.18E-11	0.347347156	0.246	0.096	9.11E-07
SRGN	4.20E-11	0.267080594	0.985	0.986	9.16E-07
LCP2	4.23E-11	0.381705132	0.815	0.762	9.22E-07
PPP1CB	4.37E-11	0.359988943	0.639	0.516	9.53E-07
SLC8B1	4.78E-11	0.324568087	0.414	0.23	1.04E-06
PLA2G15	4.80E-11	-0.255543206	0.118	0.287	1.05E-06
UBE2D3	4.98E-11	0.257289038	0.863	0.828	1.09E-06
MTHFR	5.41E-11	0.337892676	0.431	0.262	1.18E-06
JUND	5.50E-11	0.430022854	0.704	0.559	1.20E-06
FABP5	7.38E-11	0.507374372	0.758	0.646	1.61E-06
NARS	8.08E-11	-0.253003079	0.181	0.369	1.76E-06
ZNF267	8.31E-11	0.330650045	0.401	0.229	1.81E-06
RREB1	8.60E-11	-0.257299235	0.103	0.268	1.88E-06
LST1	9.16E-11	-0.270866689	0.889	0.93	2.00E-06
MRPS34	9.40E-11	-0.264744406	0.235	0.436	2.05E-06
NFE2L2	1.01E-10	0.383748352	0.721	0.623	2.19E-06
TMBIM4	1.02E-10	-0.277481252	0.567	0.738	2.22E-06
TNFRSF21	1.04E-10	0.330966086	0.353	0.184	2.26E-06
CD164	1.04E-10	-0.318660265	0.718	0.83	2.27E-06
FAM177A1	1.07E-10	0.359707513	0.435	0.285	2.32E-06
DYNLL1	1.09E-10	-0.286068656	0.653	0.791	2.37E-06
TALDO1	1.10E-10	-0.290399193	0.513	0.686	2.40E-06
PARP14	1.17E-10	-0.273561258	0.225	0.414	2.54E-06
AGO2	1.24E-10	0.253681492	0.256	0.104	2.71E-06

RAB5A	1.39E-10	0.306639796	0.471	0.311	3.02E-06
GLIPR2	1.40E-10	0.299945666	0.319	0.158	3.06E-06
GSTK1	1.41E-10	-0.284469833	0.674	0.797	3.07E-06
ARFGAP3	1.51E-10	0.269195326	0.34	0.178	3.30E-06
BTF3L4	1.70E-10	-0.277422717	0.357	0.549	3.70E-06
RILPL2	1.76E-10	0.347766969	0.605	0.475	3.84E-06
LIMD2	1.77E-10	-0.307340313	0.504	0.66	3.86E-06
IGLV2-14	2.13E-10	-0.323272468	0.137	0.307	4.63E-06
TGOLN2	2.13E-10	-0.307000387	0.319	0.508	4.63E-06
MRPL57	2.25E-10	-0.251259983	0.298	0.494	4.90E-06
FPR3	2.29E-10	-0.312943522	0.164	0.336	5.00E-06
BCL2L11	2.38E-10	0.308258195	0.271	0.121	5.19E-06
HSPA8	2.41E-10	0.43449029	0.903	0.92	5.26E-06
NAGK	2.52E-10	-0.278141528	0.334	0.527	5.49E-06
PTPRC	2.57E-10	-0.257250539	0.798	0.877	5.60E-06
ZCCHC6	2.57E-10	-0.347800787	0.393	0.566	5.61E-06
MAP1LC3B	2.61E-10	0.301963876	0.739	0.621	5.69E-06
SPAG9	2.64E-10	0.338250333	0.435	0.275	5.77E-06
RUNX1	2.68E-10	0.344317758	0.485	0.324	5.85E-06
ATP5F1	3.13E-10	-0.266541251	0.546	0.719	6.82E-06
SQSTM1	3.41E-10	0.327666219	0.826	0.773	7.43E-06
RNF145	3.64E-10	0.288563056	0.422	0.252	7.93E-06
IL1R2	3.70E-10	0.417373732	0.363	0.203	8.06E-06
JKAMP	3.73E-10	-0.264277071	0.172	0.346	8.13E-06
RYBP	4.16E-10	0.362509083	0.324	0.164	9.07E-06
CRIP1	4.32E-10	0.49892318	0.525	0.371	9.42E-06
FKBP1A	4.68E-10	-0.298715723	0.769	0.842	1.02E-05
AC058791.1	5.28E-10	0.250709765	0.174	0.055	1.15E-05
BCAS2	5.68E-10	0.435558601	0.382	0.23	1.24E-05
ID1	6.35E-10	0.360264823	0.433	0.26	1.38E-05
IER2	8.27E-10	-0.380238589	0.836	0.855	1.80E-05
NDFIP1	8.32E-10	-0.269533272	0.616	0.766	1.81E-05
SH3BP5	9.39E-10	0.326089436	0.542	0.393	2.05E-05
GBP1	9.89E-10	0.26625837	0.269	0.125	2.16E-05
TUBA1A	1.06E-09	0.392372953	0.422	0.26	2.30E-05
TIPARP	1.09E-09	0.325394148	0.345	0.186	2.37E-05
PLCB2	1.13E-09	-0.26079382	0.162	0.33	2.47E-05
NUDT16	1.16E-09	-0.285629322	0.46	0.631	2.53E-05
PRELID1	1.17E-09	0.29827048	0.748	0.637	2.56E-05
ITGB2	1.33E-09	-0.251147906	0.765	0.854	2.90E-05
COTL1	1.38E-09	-0.272271976	0.855	0.943	3.00E-05
RAB1A	1.41E-09	0.318779241	0.576	0.459	3.08E-05
NOP58	1.51E-09	0.32326767	0.338	0.188	3.29E-05
ABCA1	1.51E-09	0.269108323	0.769	0.646	3.29E-05
FAM102B	1.59E-09	0.303395258	0.273	0.129	3.47E-05
SESTD1	1.60E-09	0.322463499	0.317	0.172	3.48E-05
NDUFB2	1.62E-09	-0.25812355	0.639	0.746	3.53E-05
EMD	1.66E-09	0.356764645	0.464	0.318	3.61E-05
RGL1	1.85E-09	-0.342481871	0.342	0.5	4.03E-05
CCL20	1.93E-09	0.95660427	0.124	0.025	4.21E-05
IVNS1ABP	1.99E-09	0.359703955	0.626	0.52	4.34E-05
NPL	2.10E-09	-0.361186133	0.487	0.627	4.59E-05
EIF4A1	2.14E-09	0.271312774	0.742	0.635	4.66E-05
NOTCH1	2.17E-09	0.255021148	0.17	0.055	4.72E-05
HSD17B7	2.25E-09	0.293562434	0.246	0.111	4.92E-05
TICAM1	2.26E-09	0.256944823	0.244	0.105	4.92E-05
RRAGC	2.26E-09	0.284330159	0.313	0.164	4.93E-05
UBB	2.35E-09	0.405216711	0.868	0.834	5.13E-05
AP2S1	2.68E-09	-0.281793615	0.695	0.809	5.84E-05
BACH1	2.74E-09	0.303795463	0.489	0.322	5.98E-05
SRP9	2.84E-09	-0.296515617	0.34	0.506	6.20E-05
STK17B	3.18E-09	-0.257381359	0.357	0.549	6.93E-05
WSB1	3.25E-09	-0.276706809	0.832	0.881	7.10E-05
LSM7	3.59E-09	-0.284159619	0.342	0.51	7.84E-05
ATF4	4.06E-09	0.291590039	0.599	0.459	8.86E-05
CAP1	4.15E-09	-0.256814042	0.683	0.781	9.05E-05
NDEL1	4.30E-09	0.25903949	0.225	0.092	9.37E-05
N4BP2L2	4.56E-09	-0.260385718	0.357	0.545	9.95E-05
MT1M	4.57E-09	0.84422676	0.143	0.039	9.97E-05
ATP1B1	4.58E-09	-0.343284082	0.357	0.514	1.00E-04

GFRA2	4.62E-09	-0.29504071	0.235	0.396	0.000100793
MAT2A	4.62E-09	0.359452545	0.634	0.5	0.000100819
UBE2B	4.86E-09	0.330588306	0.708	0.613	0.000105984
VSIG4	4.87E-09	-0.274553527	0.761	0.859	0.000106272
TYMP	4.92E-09	0.345414442	0.67	0.566	0.000107264
NRP1	4.98E-09	-0.280357348	0.275	0.455	0.000108594
NABP1	4.99E-09	0.313981948	0.708	0.607	0.00010888
SGK1	5.02E-09	0.347322595	0.794	0.686	0.000109536
ZYX	5.08E-09	-0.265208776	0.693	0.787	0.000110856
GABARAPL1	5.44E-09	0.300678558	0.414	0.264	0.000118626
APPL1	5.49E-09	-0.271354632	0.261	0.424	0.000119645
CD33	5.75E-09	-0.25701887	0.338	0.508	0.000125364
PSMA4	5.99E-09	-0.283305338	0.315	0.498	0.000130595
PTGER2	6.23E-09	0.299189475	0.296	0.154	0.000135893
TMC8	7.30E-09	-0.254606381	0.143	0.291	0.000159119
ALB	7.96E-09	0.352036524	0.42	0.266	0.000173609
HLX	8.03E-09	0.274858197	0.242	0.111	0.000175091
CNPY3	8.41E-09	-0.254376212	0.605	0.746	0.0001835
SLC9A8	9.02E-09	0.280470061	0.2	0.08	0.000196762
GPR183	9.50E-09	0.271205399	0.544	0.369	0.000207076
BRK1	1.05E-08	-0.254506703	0.544	0.713	0.000229141
CDC37	1.24E-08	0.372989752	0.63	0.529	0.000270598
DDX3Y	1.31E-08	0.326240973	0.502	0.357	0.000285465
VIMP	1.32E-08	0.336142498	0.534	0.406	0.0002874
S100A4	1.33E-08	-0.380320082	0.733	0.801	0.000289629
CYBB	1.67E-08	-0.266988522	0.706	0.818	0.000363499
HMGB1	1.74E-08	-0.250962193	0.821	0.885	0.000380009
PLEKHG2	1.80E-08	0.2505749	0.21	0.086	0.000391524
RP11-1080G15.1	1.88E-08	0.286258492	0.202	0.082	0.000411075
CECR1	1.90E-08	-0.260009305	0.744	0.824	0.00041327
NR1H3	2.02E-08	-0.309206366	0.37	0.523	0.000439555
RPL36A	2.10E-08	0.250484815	0.914	0.869	0.000458989
PRDX1	2.58E-08	-0.257162573	0.723	0.814	0.000561587
SLC5A3	2.59E-08	0.291333122	0.231	0.104	0.000565112
UBE2J1	2.75E-08	0.283065345	0.622	0.508	0.000599172
SDC3	2.80E-08	-0.316203408	0.328	0.484	0.000609968
IL6	2.88E-08	0.284455963	0.113	0.025	0.000627448
AHNAK	3.02E-08	-0.312270235	0.553	0.678	0.000659246
NDUFA4	3.06E-08	-0.253714023	0.71	0.818	0.000668248
CD300E	3.16E-08	0.27658343	0.185	0.072	0.000689785
MNDA	3.40E-08	-0.307587626	0.431	0.572	0.000740965
CYCS	3.43E-08	0.487954866	0.626	0.543	0.000747449
CLTA	3.52E-08	-0.259175212	0.674	0.783	0.000768351
SAR1A	3.59E-08	0.281943808	0.328	0.193	0.000783151
NR4A1	3.97E-08	0.297678513	0.737	0.564	0.000864857
TSPAN4	4.01E-08	-0.266661697	0.571	0.697	0.000874
TNFRSF1B	4.04E-08	0.317828406	0.708	0.623	0.000881521
MORF4L2	4.32E-08	0.265615836	0.454	0.299	0.000941412
GADD45A	4.39E-08	0.290868291	0.216	0.096	0.00095689
TPM4	4.51E-08	0.300750409	0.525	0.406	0.000982978
IRF7	4.57E-08	0.300459379	0.42	0.287	0.000996539
SMIM3	4.60E-08	0.273462605	0.296	0.168	0.001003276
ARL4A	4.73E-08	0.273922545	0.712	0.615	0.001031612
C19orf70	4.96E-08	-0.251547987	0.372	0.555	0.00108258
FAM20A	5.31E-08	0.277750596	0.424	0.287	0.001158733
MRC1	5.75E-08	-0.327093666	0.616	0.738	0.001254272
ANKRD37	5.93E-08	0.270997348	0.261	0.131	0.001294079
RANGAP1	6.68E-08	0.252296181	0.197	0.084	0.001456225
CITED2	7.12E-08	-0.389181074	0.462	0.605	0.001552321
BHLHE40	7.62E-08	0.268551845	0.613	0.467	0.001662035
SEPHS2	8.64E-08	0.332931707	0.368	0.238	0.001883708
GYPC	9.51E-08	0.253288049	0.63	0.498	0.002074536
MT-ND6	9.73E-08	-0.282346975	0.664	0.766	0.002121194
SOD1	1.13E-07	0.33895134	0.662	0.574	0.002472101
HCAR3	1.29E-07	0.333075146	0.168	0.064	0.002805214
NAAA	1.29E-07	-0.268567531	0.418	0.561	0.002809984
SLC16A6	1.39E-07	0.307408397	0.204	0.094	0.003041043
CHD2	1.42E-07	0.255552933	0.536	0.393	0.003094887
EVI2B	1.54E-07	-0.256817133	0.683	0.77	0.003357589
TSPYL2	1.67E-07	0.276498887	0.229	0.107	0.003631907

IL6ST	1.78E-07	0.270566888	0.416	0.277	0.003888615
AP1S2	2.11E-07	-0.252124248	0.716	0.811	0.004603395
ATF7IP2	2.88E-07	0.450010466	0.204	0.096	0.006283519
SYK	2.90E-07	-0.260141863	0.239	0.391	0.00633488
CHORDC1	3.07E-07	0.312759322	0.368	0.254	0.006691261
MT1X	4.30E-07	0.854174673	0.513	0.373	0.009370464
CYTIP	4.32E-07	-0.263845435	0.332	0.477	0.00942337
TANK	4.33E-07	0.276561062	0.473	0.336	0.009439566
IFITM3	4.43E-07	0.263331663	0.87	0.838	0.009651376
EGR1	4.49E-07	-0.463528214	0.342	0.484	0.009782741
GLA	5.10E-07	0.554186924	0.473	0.365	0.011113655
S100A10	5.17E-07	0.388639782	0.803	0.719	0.011275139
FNIP2	6.04E-07	0.323270437	0.473	0.377	0.013172841
STK38L	6.51E-07	0.255834992	0.305	0.184	0.014194298
VPS13C	6.85E-07	-0.252309774	0.342	0.484	0.014939248
CFLAR	8.12E-07	0.279267387	0.634	0.533	0.017714922
GUK1	8.13E-07	0.273697596	0.689	0.635	0.017725149
PRNP	8.34E-07	0.317690414	0.723	0.67	0.018190386
ZFAND5	8.70E-07	0.328540194	0.811	0.752	0.018971234
IRAK3	8.82E-07	0.298336678	0.569	0.465	0.019225777
NUDT3	9.50E-07	-0.251392753	0.166	0.293	0.020719183
CASP4	1.02E-06	0.27315351	0.534	0.422	0.022167374
EML4	1.02E-06	0.329007034	0.374	0.256	0.022326475
TNFSF13B	1.14E-06	0.366109254	0.485	0.387	0.024853693
KYNU	1.25E-06	0.267842592	0.481	0.363	0.027159563
GNA15	1.42E-06	0.263834839	0.321	0.205	0.030871481
C11orf31	1.51E-06	-0.256186431	0.424	0.551	0.032950659

List of differentially expressed genes of VCAN_Tmo in reperfusion stage (PR versus EP)

gene	p_val	avg_logFC	pct.1	pct.2	p_val_adj
HLA-DRB5	4.50E-61	-1.267756909	0.401	0.938	9.82E-57
THBS1	2.03E-56	1.290499442	0.962	0.765	4.42E-52
NAMPT	1.29E-50	0.884883095	0.993	0.893	2.81E-46
ACSL1	8.83E-42	0.909493474	0.89	0.581	1.93E-37
SRGN	3.26E-39	0.496140506	1	1	7.10E-35
H3F3B	6.98E-39	0.565344955	0.995	0.985	1.52E-34
ACTB	2.83E-38	0.375341711	1	1	6.16E-34
VCAN	4.01E-37	0.73001184	0.983	0.945	8.74E-33
FPR1	4.39E-37	0.814800651	0.9	0.695	9.57E-33
UPP1	2.13E-36	0.835369896	0.847	0.496	4.64E-32
CST3	1.22E-35	-0.504831227	0.998	1	2.65E-31
ASPH	7.18E-35	0.934731608	0.649	0.224	1.57E-30
HLA-DQB1	8.51E-34	-0.80214729	0.332	0.783	1.86E-29
S100A8	1.03E-33	0.793008849	0.998	0.993	2.24E-29
FCER1G	2.15E-33	0.524695182	1	0.993	4.70E-29
DEFA3	3.76E-33	-0.658645176	0.017	0.349	8.20E-29
AQP9	3.98E-33	0.860790581	0.702	0.268	8.67E-29
S100A9	6.26E-33	0.86407844	0.993	0.978	1.37E-28
TPM4	1.61E-32	0.755438163	0.864	0.625	3.51E-28
TMSB4X	2.16E-32	-0.369724126	1	0.993	4.70E-28
TRMT6	3.10E-32	0.957515433	0.558	0.143	6.77E-28
LITAF	4.67E-32	0.859978981	0.842	0.57	1.02E-27
EEF1A1	1.16E-31	-0.321467062	0.998	0.996	2.53E-27
S100A12	1.50E-31	0.985267306	0.938	0.831	3.27E-27
GIMAP1	8.91E-31	-0.618966708	0.076	0.452	1.94E-26
FPR2	3.22E-30	0.908560235	0.582	0.169	7.03E-26
CTSL	4.75E-29	1.63787354	0.642	0.301	1.04E-24
EVI2B	1.90E-28	-0.683801141	0.556	0.86	4.14E-24
TIMP1	2.47E-28	0.90586156	0.979	0.952	5.38E-24
FGL2	4.76E-28	-0.62217022	0.425	0.846	1.04E-23
PABPC1	7.41E-27	-0.429926052	0.931	0.982	1.62E-22
RPL10	2.61E-26	-0.265092433	1	0.993	5.68E-22
GIMAP2	3.62E-26	-0.52771133	0.053	0.371	7.90E-22
METTL7A	1.13E-25	-0.631720814	0.153	0.529	2.46E-21
RPL5	1.47E-25	-0.3799378	0.964	0.978	3.19E-21
SAT1	5.95E-25	0.423159569	0.998	1	1.30E-20
CD63	5.96E-25	0.610076221	0.916	0.779	1.30E-20
MCEMP1	6.37E-25	0.821649823	0.661	0.316	1.39E-20

PLSCR1	1.00E-24	0.680921229	0.754	0.478	2.18E-20
IL1R2	3.76E-24	1.184803314	0.599	0.276	8.21E-20
HSPA1B	8.64E-24	-0.81464928	0.229	0.566	1.88E-19
TXNIP	1.86E-23	-0.773666757	0.453	0.783	4.06E-19
SLC39A8	3.67E-23	0.955811609	0.437	0.099	8.00E-19
AREG	6.28E-23	0.922225552	0.871	0.629	1.37E-18
BLVRB	8.11E-23	-0.547513872	0.63	0.871	1.77E-18
RNASE6	1.13E-22	-0.594078698	0.368	0.717	2.47E-18
RPS14	1.25E-22	-0.253030897	0.993	0.996	2.73E-18
CSF1R	2.66E-22	-0.536185201	0.126	0.46	5.79E-18
HSPA8	3.93E-22	-0.462567032	0.589	0.868	8.58E-18
SELL	4.04E-22	0.834999381	0.69	0.404	8.80E-18
SLC2A3	6.60E-22	0.716590442	0.833	0.625	1.44E-17
IFITM2	9.42E-22	0.646052758	0.936	0.926	2.05E-17
NACA	1.19E-21	-0.308374134	0.993	0.982	2.60E-17
VAMP8	1.21E-21	-0.523123606	0.613	0.842	2.63E-17
S100A4	1.69E-21	-0.299082087	1	0.993	3.69E-17
HBB	2.14E-21	-0.560973265	0.086	0.39	4.68E-17
SULT1A1	2.15E-21	-0.556921492	0.208	0.555	4.70E-17
VSIG4	2.42E-21	-0.683785558	0.1	0.401	5.28E-17
NUP214	3.10E-21	-0.542728034	0.265	0.621	6.75E-17
BTG1	3.51E-21	-0.510322258	0.842	0.967	7.66E-17
PIM1	9.02E-21	0.656789262	0.396	0.081	1.97E-16
RHOB	1.00E-20	-0.636775721	0.649	0.857	2.18E-16
AGFG1	1.33E-20	0.714316159	0.663	0.39	2.90E-16
RPS29	1.38E-20	0.391741942	0.952	0.956	3.01E-16
CXCR4	2.02E-20	-0.633296138	0.432	0.757	4.40E-16
GIMAP7	2.87E-20	-0.461253325	0.048	0.301	6.26E-16
LIMD2	3.80E-20	-0.566724903	0.372	0.695	8.30E-16
GPX1	4.43E-20	0.424928459	0.957	0.919	9.65E-16
NFKBIZ	4.56E-20	-0.605705677	0.42	0.746	9.94E-16
GNB2L1	6.73E-20	-0.314158186	0.974	0.982	1.47E-15
SERPINB9	7.78E-20	0.606272752	0.589	0.261	1.70E-15
RPL29	9.33E-20	-0.250854779	0.988	0.985	2.03E-15
SLC25A6	1.30E-19	-0.375119731	0.881	0.941	2.83E-15
SERPINB1	1.39E-19	0.529784594	0.926	0.86	3.02E-15
MAP3K8	1.49E-19	0.697977486	0.733	0.496	3.24E-15
SEC11A	1.62E-19	-0.427516539	0.644	0.904	3.52E-15
ETS2	2.78E-19	0.610096771	0.759	0.562	6.07E-15
SLC25A37	3.37E-19	0.608837657	0.609	0.305	7.36E-15
RPL7A	4.83E-19	-0.25720786	0.986	0.989	1.05E-14
FCGR3A	5.02E-19	-0.670257867	0.107	0.386	1.09E-14
MYL6	5.21E-19	0.351783915	0.99	0.978	1.14E-14
PAG1	7.36E-19	0.672932235	0.601	0.301	1.61E-14
PNP	7.68E-19	0.69080632	0.566	0.283	1.67E-14
GCA	7.77E-19	0.572246044	0.869	0.842	1.70E-14
PIM3	9.27E-19	0.63839631	0.535	0.232	2.02E-14
MIS18BP1	9.31E-19	-0.428469512	0.084	0.357	2.03E-14
RUNX1	1.19E-18	0.599430001	0.578	0.276	2.60E-14
PLD3	1.38E-18	-0.555974013	0.301	0.607	3.00E-14
TPM3	1.61E-18	0.406580238	0.921	0.882	3.51E-14
GIMAP4	3.93E-18	-0.484887474	0.191	0.511	8.56E-14
CFL1	6.37E-18	0.295198715	0.99	0.985	1.39E-13
HIF1A	8.25E-18	0.615427326	0.723	0.529	1.80E-13
RCSD1	1.22E-17	-0.450483714	0.136	0.43	2.67E-13
RPL18	1.25E-17	-0.250282415	0.995	0.989	2.72E-13
CRTAP	1.72E-17	-0.509917803	0.482	0.761	3.75E-13
DUSP1	1.76E-17	-0.394039184	0.969	0.985	3.83E-13
MARCKS	1.87E-17	0.705637764	0.632	0.353	4.09E-13
SAMSN1	2.06E-17	0.518855191	0.878	0.765	4.49E-13
CLIC1	2.12E-17	0.355597022	0.974	0.938	4.62E-13
JUN	2.62E-17	-0.762555758	0.434	0.688	5.72E-13
IFI30	4.37E-17	0.517702711	0.854	0.768	9.53E-13
IL1RAP	4.88E-17	0.648850832	0.372	0.096	1.06E-12
RPL14	5.95E-17	-0.262279899	0.971	0.989	1.30E-12
FAM65B	6.27E-17	0.622156919	0.685	0.493	1.37E-12
CD93	6.71E-17	0.580643735	0.64	0.364	1.46E-12
LRG1	7.25E-17	0.618787955	0.315	0.055	1.58E-12
C20orf27	1.14E-16	-0.411703409	0.181	0.489	2.49E-12
TKT	1.19E-16	-0.454140747	0.823	0.941	2.60E-12

ADAMTS2	1.21E-16	-0.435549922	0.103	0.368	2.64E-12
B4GALT5	1.26E-16	0.588804352	0.372	0.103	2.74E-12
FCGR1A	2.11E-16	0.661229315	0.666	0.449	4.60E-12
TNFSF10	2.80E-16	-0.444161657	0.045	0.265	6.12E-12
RGCC	3.39E-16	0.929872992	0.411	0.129	7.39E-12
RASSF4	4.43E-16	-0.44084031	0.141	0.419	9.65E-12
PYCARD	4.44E-16	-0.438706309	0.659	0.871	9.68E-12
CD300E	4.78E-16	0.686412617	0.628	0.404	1.04E-11
SLC25A5	4.84E-16	-0.414606599	0.656	0.875	1.06E-11
MCTP2	5.50E-16	0.524464675	0.308	0.059	1.20E-11
CKAP4	6.61E-16	0.604374798	0.492	0.221	1.44E-11
IFITM3	7.38E-16	0.711393944	0.89	0.875	1.61E-11
POMP	8.48E-16	0.463309099	0.845	0.728	1.85E-11
ANXA1	8.69E-16	0.417595896	0.936	0.893	1.89E-11
MS4A6A	2.17E-15	-0.471518886	0.745	0.89	4.73E-11
RAB31	2.28E-15	0.549933478	0.783	0.654	4.98E-11
TYMP	3.50E-15	0.496500552	0.854	0.739	7.63E-11
BIN2	3.84E-15	-0.387325603	0.165	0.449	8.37E-11
CD82	4.58E-15	0.493605508	0.341	0.088	9.98E-11
CD74	7.22E-15	-0.302088795	0.988	0.985	1.57E-10
CXCL8	8.86E-15	-0.791915431	0.537	0.787	1.93E-10
FLT3	9.07E-15	-0.338354956	0.079	0.312	1.98E-10
PSTPIP1	9.20E-15	-0.396987058	0.184	0.463	2.01E-10
RPL6	9.39E-15	-0.255065834	0.986	0.993	2.05E-10
GDI2	9.93E-15	-0.429639023	0.566	0.82	2.17E-10
GRINA	1.20E-14	0.464830128	0.828	0.706	2.62E-10
HVCN1	1.39E-14	-0.358501974	0.043	0.239	3.04E-10
P2RY13	1.41E-14	-0.351821636	0.036	0.224	3.07E-10
TOP1	2.20E-14	0.587674134	0.592	0.375	4.79E-10
PAK1	2.73E-14	-0.396303042	0.107	0.353	5.94E-10
EIF4EBP1	3.47E-14	-0.383933099	0.186	0.471	7.57E-10
LIPA	3.49E-14	-0.559967429	0.334	0.625	7.61E-10
CLNS1A	3.65E-14	-0.360402793	0.141	0.404	7.96E-10
SERPINB2	4.13E-14	0.772853251	0.37	0.107	9.01E-10
RPL24	4.15E-14	-0.263458257	0.955	0.982	9.05E-10
MT-CYB	4.19E-14	0.289895161	1	1	9.14E-10
SEMA6B	4.72E-14	0.47843202	0.234	0.026	1.03E-09
DSE	5.36E-14	0.561321406	0.523	0.29	1.17E-09
RPLP0	5.47E-14	-0.27931726	0.959	0.963	1.19E-09
POU2F2	6.10E-14	-0.367081615	0.236	0.533	1.33E-09
ABI3	6.78E-14	-0.355602839	0.076	0.29	1.48E-09
HNMT	7.45E-14	-0.464190792	0.239	0.522	1.62E-09
ATP1A1	8.67E-14	0.516120809	0.609	0.393	1.89E-09
AP1S2	1.17E-13	-0.390955365	0.706	0.846	2.54E-09
ATP5G2	1.29E-13	-0.260082672	0.94	0.982	2.82E-09
SH3BP1	1.43E-13	-0.346307034	0.1	0.335	3.11E-09
NR4A2	1.87E-13	-0.498282498	0.618	0.835	4.08E-09
KCNE3	2.04E-13	-0.366843777	0.107	0.338	4.45E-09
CYBRD1	2.13E-13	-0.282411906	0.026	0.191	4.65E-09
GNG10	2.42E-13	0.540796102	0.621	0.43	5.27E-09
RPS5	2.83E-13	-0.275330847	0.933	0.949	6.18E-09
BCL2A1	3.52E-13	0.302411432	0.926	0.787	7.67E-09
CALHM2	3.55E-13	-0.336508645	0.045	0.228	7.75E-09
NKG7	4.23E-13	0.719037437	0.418	0.191	9.23E-09
CIR1	4.37E-13	-0.325073463	0.067	0.272	9.53E-09
HLA-DPA1	4.40E-13	-0.469427247	0.575	0.801	9.59E-09
NR4A1	5.48E-13	-0.638904651	0.406	0.625	1.20E-08
DNASE2	6.34E-13	-0.315376701	0.06	0.254	1.38E-08
HLA-DMB	6.53E-13	-0.382599265	0.167	0.419	1.42E-08
SULT1B1	7.88E-13	-0.370423469	0.098	0.324	1.72E-08
EEF2	7.95E-13	-0.372304154	0.847	0.919	1.73E-08
GK	8.05E-13	0.545407066	0.68	0.489	1.75E-08
AMPH	8.84E-13	-0.271659938	0.031	0.199	1.93E-08
MEF2C	8.88E-13	-0.468191931	0.208	0.474	1.94E-08
ZFAS1	9.48E-13	-0.361574514	0.764	0.915	2.07E-08
GLMP	9.59E-13	-0.318680901	0.057	0.246	2.09E-08
CNPY3	9.63E-13	-0.366777043	0.496	0.776	2.10E-08
OGFRL1	1.12E-12	-0.326307438	0.091	0.309	2.43E-08
CNIH4	1.16E-12	0.59774683	0.592	0.43	2.53E-08
STXBP2	1.28E-12	0.568089291	0.862	0.798	2.80E-08

LDHB	1.33E-12	-0.414349576	0.332	0.61	2.89E-08
FGD2	1.57E-12	-0.440021511	0.148	0.386	3.43E-08
PFKFB3	1.67E-12	0.47306474	0.461	0.217	3.63E-08
STX10	1.85E-12	-0.390787858	0.232	0.504	4.03E-08
NR4A3	2.10E-12	-0.480011916	0.191	0.43	4.57E-08
MYL12A	2.13E-12	0.33841369	0.952	0.89	4.65E-08
PPIF	2.24E-12	0.573669908	0.484	0.257	4.88E-08
GK5	2.30E-12	0.44193724	0.246	0.048	5.02E-08
EIF3E	2.37E-12	-0.360875699	0.656	0.864	5.17E-08
MAFB	2.91E-12	0.511742908	0.857	0.798	6.35E-08
LST1	2.96E-12	-0.342450196	0.804	0.919	6.47E-08
NME3	3.47E-12	-0.347632435	0.091	0.301	7.57E-08
MS4A7	3.71E-12	-0.459521355	0.305	0.57	8.09E-08
INPP5D	3.73E-12	-0.288549154	0.06	0.25	8.13E-08
SDC4	4.30E-12	0.500120209	0.291	0.081	9.37E-08
MID1IP1	4.31E-12	-0.423421957	0.167	0.408	9.41E-08
IL18	5.50E-12	-0.392531256	0.126	0.346	1.20E-07
RBKS	6.05E-12	-0.518259088	0.181	0.426	1.32E-07
NDE1	7.74E-12	-0.262939122	0.053	0.232	1.69E-07
UBL7	7.77E-12	-0.305009037	0.086	0.287	1.69E-07
CPM	7.91E-12	-0.359559083	0.167	0.412	1.73E-07
RGS2	8.58E-12	-0.418070929	0.692	0.879	1.87E-07
LILRA5	9.04E-12	0.460382597	0.766	0.614	1.97E-07
CXCL2	1.17E-11	-0.613253174	0.511	0.721	2.56E-07
SLCO4A1	1.30E-11	0.400512993	0.205	0.026	2.83E-07
RBMS1	1.30E-11	0.494459711	0.606	0.419	2.84E-07
HLA-DPB1	1.62E-11	-0.509435279	0.516	0.724	3.52E-07
GIMAP8	1.67E-11	-0.281774526	0.031	0.184	3.63E-07
CD1D	1.80E-11	-0.348394841	0.129	0.349	3.93E-07
TMEM69	1.99E-11	-0.294334855	0.086	0.279	4.35E-07
CALML4	2.04E-11	-0.25383756	0.041	0.202	4.45E-07
HLA-A	2.24E-11	-0.284376888	0.947	0.982	4.87E-07
FAM129B	2.25E-11	0.431083342	0.26	0.062	4.90E-07
LMNB1	2.28E-11	0.453731378	0.248	0.059	4.98E-07
HEBP1	2.39E-11	-0.315999984	0.131	0.353	5.20E-07
MT1E	2.57E-11	-0.875613952	0.091	0.276	5.60E-07
ZFP36L2	2.61E-11	-0.405374719	0.704	0.875	5.68E-07
PHB2	2.88E-11	-0.344951886	0.308	0.555	6.27E-07
RNF141	4.21E-11	-0.429634244	0.193	0.419	9.18E-07
GIMAP6	5.10E-11	-0.251695171	0.05	0.217	1.11E-06
MCL1	5.68E-11	0.293799841	0.931	0.893	1.24E-06
AC004556.1	7.18E-11	0.313721587	0.143	0	1.57E-06
PLAUR	7.60E-11	0.425756338	0.928	0.846	1.66E-06
ANP32B	8.33E-11	-0.329907427	0.425	0.68	1.82E-06
ANKRD44	8.56E-11	-0.271709263	0.112	0.32	1.87E-06
SDC2	8.63E-11	0.447001123	0.21	0.037	1.88E-06
CMTM7	8.75E-11	-0.37009401	0.289	0.555	1.91E-06
ATP13A3	1.22E-10	0.481562719	0.425	0.213	2.66E-06
RBM47	1.57E-10	0.465656634	0.666	0.504	3.43E-06
ADM	1.59E-10	0.487430103	0.547	0.324	3.47E-06
TAGAP	1.61E-10	-0.484630993	0.236	0.46	3.51E-06
VAPA	1.64E-10	0.421573687	0.785	0.739	3.58E-06
SRSF2	1.80E-10	0.421217711	0.704	0.607	3.92E-06
HP	1.97E-10	0.420773689	0.241	0.055	4.30E-06
DOK2	2.13E-10	-0.315234019	0.368	0.64	4.64E-06
SSR3	2.25E-10	-0.33784409	0.351	0.61	4.90E-06
CHSY1	2.42E-10	0.396091036	0.289	0.096	5.28E-06
NFIA	2.66E-10	-0.280918012	0.038	0.184	5.81E-06
DCK	2.88E-10	-0.280651799	0.072	0.243	6.28E-06
EVI2A	2.99E-10	-0.273851157	0.16	0.39	6.53E-06
LYSMD2	3.05E-10	-0.28142095	0.105	0.305	6.64E-06
FLOT1	3.14E-10	0.482224329	0.518	0.346	6.85E-06
WIPF1	3.33E-10	-0.290965907	0.27	0.518	7.25E-06
HRH2	3.36E-10	0.450124892	0.42	0.221	7.33E-06
AHNAK	3.41E-10	-0.410281685	0.623	0.835	7.43E-06
ZEB2	3.48E-10	0.317362704	0.897	0.912	7.59E-06
CCL3L3	3.99E-10	-0.561054738	0.193	0.419	8.69E-06
YWHAZ	4.32E-10	0.301578135	0.878	0.857	9.43E-06
ZFAND5	4.57E-10	-0.426720767	0.718	0.849	9.98E-06
SUB1	4.83E-10	0.338952309	0.864	0.835	1.05E-05

FAM96A	5.83E-10	-0.350034497	0.32	0.577	1.27E-05
YPEL3	5.98E-10	-0.259584456	0.064	0.232	1.30E-05
ADCY7	6.42E-10	-0.309971276	0.086	0.265	1.40E-05
PTPN1	6.74E-10	0.424576239	0.415	0.217	1.47E-05
SERPINA1	6.86E-10	0.342375463	0.943	0.912	1.50E-05
SARAF	7.31E-10	-0.283574499	0.642	0.853	1.59E-05
PRELID1	7.37E-10	0.382805687	0.778	0.684	1.61E-05
SLC7A7	7.42E-10	-0.374318768	0.403	0.618	1.62E-05
BTG2	7.48E-10	-0.361217704	0.224	0.452	1.63E-05
DDX60L	7.60E-10	0.454018414	0.36	0.169	1.66E-05
ENO1	7.86E-10	0.326109713	0.859	0.809	1.71E-05
ERP29	8.31E-10	-0.385448308	0.501	0.735	1.81E-05
CHCHD10	1.00E-09	-0.36962908	0.308	0.54	2.18E-05
PRAM1	1.00E-09	-0.282121466	0.158	0.371	2.18E-05
RSL24D1	1.01E-09	-0.274679108	0.413	0.676	2.21E-05
BAZ2B	1.07E-09	-0.286379856	0.146	0.36	2.34E-05
ATF3	1.13E-09	-0.358639995	0.315	0.559	2.47E-05
BTK	1.26E-09	-0.261928852	0.095	0.279	2.75E-05
JARID2	1.27E-09	0.464171196	0.461	0.276	2.76E-05
SMIM14	1.28E-09	-0.286262558	0.115	0.309	2.79E-05
OXA1L	1.29E-09	-0.346915032	0.296	0.526	2.82E-05
IGBP1	1.43E-09	-0.302094232	0.189	0.412	3.11E-05
SLA	1.56E-09	0.435334645	0.628	0.478	3.41E-05
KYNU	1.67E-09	0.438153199	0.544	0.353	3.65E-05
PET100	1.75E-09	0.404538695	0.673	0.522	3.81E-05
NCF1	1.84E-09	-0.473965696	0.267	0.485	4.00E-05
CCNI	2.22E-09	-0.267979079	0.811	0.93	4.84E-05
ZFAND1	2.29E-09	-0.257689245	0.074	0.239	5.00E-05
ARHGDIA	2.71E-09	0.377794376	0.692	0.581	5.91E-05
RNASE2	2.76E-09	-0.377202341	0.282	0.515	6.01E-05
JKAMP	3.22E-09	-0.252087869	0.086	0.257	7.03E-05
MNDA	3.25E-09	-0.360305689	0.742	0.879	7.09E-05
FOS	3.28E-09	-0.266707234	0.99	0.985	7.16E-05
LY86	3.29E-09	-0.329986274	0.437	0.669	7.18E-05
CD302	3.40E-09	-0.320444961	0.621	0.842	7.42E-05
PHF3	3.51E-09	-0.271757713	0.112	0.298	7.65E-05
ANKRD28	3.62E-09	0.495390538	0.406	0.21	7.90E-05
VASP	3.72E-09	0.402117138	0.649	0.526	8.11E-05
MRPL34	3.95E-09	-0.253452198	0.081	0.25	8.62E-05
PSMB9	4.05E-09	-0.293923631	0.317	0.562	8.84E-05
S100P	4.19E-09	0.427199275	0.15	0.015	9.14E-05
EIF3M	4.25E-09	-0.28095712	0.52	0.724	9.27E-05
METTL7B	4.34E-09	0.383732384	0.155	0.018	9.46E-05
MRPS34	4.34E-09	-0.252322184	0.217	0.449	9.47E-05
RNASET2	4.40E-09	-0.362890231	0.542	0.732	9.59E-05
TFRC	4.86E-09	0.52234632	0.401	0.217	0.000105903
CD37	4.99E-09	-0.286436339	0.692	0.853	0.000108754
G0S2	5.14E-09	0.540962745	0.525	0.312	0.000112168
PPP1CC	5.23E-09	-0.325665841	0.317	0.551	0.000114119
MIR4435-2HG	5.33E-09	0.503956175	0.456	0.283	0.000116336
EMP3	5.37E-09	0.324954731	0.959	0.926	0.000117055
SLC11A1	5.51E-09	0.390722891	0.819	0.783	0.000120193
JUNB	5.62E-09	-0.261388118	0.962	0.971	0.000122468
PSMG2	5.89E-09	-0.321355788	0.265	0.485	0.000128463
MBNL1	6.10E-09	-0.294406204	0.47	0.706	0.00013309
CAMK1	6.76E-09	-0.293289754	0.1	0.272	0.000147371
GPX4	6.90E-09	-0.298617092	0.716	0.846	0.000150372
C1orf162	8.30E-09	-0.275151403	0.816	0.952	0.000180964
MAT2B	8.48E-09	-0.328472742	0.174	0.371	0.000184913
PTPN18	8.60E-09	-0.280090479	0.16	0.357	0.000187499
CD44	8.76E-09	0.337917364	0.85	0.779	0.000191139
RILPL2	9.54E-09	0.316419456	0.764	0.662	0.000208149
ASGR1	9.70E-09	-0.293144264	0.263	0.493	0.00021162
BCL3	1.00E-08	0.381831754	0.47	0.279	0.000219116
CD14	1.14E-08	0.34748336	0.869	0.86	0.000248322
SLC8A1	1.16E-08	-0.31694963	0.153	0.342	0.000252815
IFI6	1.16E-08	0.479440428	0.329	0.151	0.000253348
BAZ1A	1.16E-08	0.464823938	0.609	0.467	0.000253563
DAZAP2	1.23E-08	0.309265919	0.869	0.842	0.000267505
TXN	1.27E-08	0.357104838	0.74	0.665	0.000276777

RELB	1.30E-08	0.419753106	0.346	0.169	0.000283466
MRPS21	1.31E-08	-0.316544455	0.308	0.559	0.000284856
CSK	1.36E-08	-0.277614979	0.303	0.551	0.000295555
TLR2	1.41E-08	0.428108514	0.57	0.426	0.00030815
UTRN	1.41E-08	-0.278296569	0.198	0.419	0.0003084
BZW1	1.52E-08	0.391216216	0.573	0.415	0.00033139
DDX21	1.59E-08	0.433343631	0.666	0.522	0.000346869
C14orf166	1.60E-08	-0.30692729	0.432	0.643	0.000348045
GNA15	1.61E-08	0.435084125	0.391	0.232	0.000351843
IFRD1	1.61E-08	-0.346465663	0.198	0.397	0.000352012
IER2	1.62E-08	-0.352088004	0.85	0.926	0.000353021
SNHG7	1.64E-08	-0.319313938	0.148	0.338	0.000358624
CLEC5A	1.72E-08	0.319301025	0.141	0.015	0.000374786
FLI1	1.73E-08	-0.268141658	0.184	0.386	0.000377619
ABHD5	1.76E-08	-0.416100954	0.21	0.412	0.000382799
GNPDA1	1.80E-08	-0.253114039	0.091	0.254	0.000393434
POLR1D	2.02E-08	-0.276897075	0.279	0.515	0.000440293
NSL1	2.20E-08	-0.256288895	0.122	0.301	0.000480422
HMGN3	2.35E-08	-0.264197482	0.267	0.489	0.000512983
PKM	2.42E-08	0.289742258	0.866	0.842	0.000526835
BNIP3L	2.47E-08	-0.251140769	0.461	0.695	0.00053819
LINC00936	2.56E-08	-0.378184314	0.399	0.614	0.000558614
CD33	2.81E-08	-0.282138327	0.229	0.445	0.000611814
RETN	3.27E-08	0.650299307	0.298	0.132	0.000712337
NPM1	3.48E-08	-0.282928066	0.587	0.82	0.00075887
RSL1D1	3.53E-08	-0.3127124	0.365	0.577	0.000770079
CECR1	3.54E-08	-0.287910961	0.437	0.676	0.000771065
IL13RA1	3.62E-08	-0.291871693	0.22	0.43	0.000788774
IGLV2-14	3.79E-08	-0.310982723	0.105	0.268	0.000825556
NDUFB9	3.79E-08	0.320662308	0.74	0.669	0.000826424
CD163	3.79E-08	0.286486774	0.945	0.938	0.000827424
RGL1	3.86E-08	0.432977929	0.282	0.118	0.000841058
JAK3	3.96E-08	0.321698545	0.212	0.062	0.000864477
ATP5A1	4.17E-08	-0.323560257	0.401	0.614	0.000909682
NDFIP1	4.22E-08	-0.270368046	0.346	0.585	0.00092112
TNFAIP2	4.29E-08	-0.301985894	0.26	0.482	0.000934686
LCP1	4.43E-08	0.27354392	0.909	0.879	0.000965149
ATM	5.02E-08	-0.255061085	0.072	0.217	0.001094569
STEAP4	5.10E-08	0.274362199	0.103	0	0.001112771
TNRC6B	5.11E-08	-0.285853224	0.103	0.268	0.001113929
GSN	5.13E-08	-0.324463632	0.222	0.419	0.001119072
PHLDA1	5.17E-08	0.400755488	0.2	0.055	0.001127077
PTX3	5.20E-08	0.46692242	0.251	0.092	0.001133225
ARL6IP4	5.38E-08	-0.258787429	0.489	0.706	0.001173318
CRIP1	5.43E-08	-0.326337746	0.687	0.846	0.001184573
CD52	5.43E-08	-0.368957293	0.599	0.783	0.001184595
FCGR1B	5.49E-08	0.446935394	0.356	0.188	0.001197875
NOTCH2	5.67E-08	-0.2957992	0.177	0.371	0.001237185
RASA2	5.92E-08	0.404566639	0.289	0.132	0.0012909
CTSB	6.06E-08	0.355425098	0.936	0.919	0.00132075
CD83	6.95E-08	-0.338812254	0.468	0.699	0.001515561
IFNGR2	7.48E-08	0.415235795	0.68	0.574	0.00163069
SH3GLB1	7.55E-08	0.446975458	0.566	0.452	0.00164599
UQCR10	8.13E-08	-0.303784698	0.616	0.82	0.001772829
LILRA2	8.20E-08	-0.259880487	0.327	0.562	0.0017883
CKS2	8.30E-08	-0.339571839	0.224	0.419	0.001809854
RNF44	8.41E-08	-0.261134971	0.072	0.213	0.001833322
TMEM167A	8.71E-08	0.371998573	0.702	0.614	0.001899718
CLEC4E	8.89E-08	-0.314435799	0.566	0.754	0.001937916
ID2	9.14E-08	-0.410208923	0.406	0.603	0.001993535
SQLE	9.19E-08	0.375676309	0.241	0.088	0.00200339
DPYSL2	9.43E-08	-0.307632873	0.169	0.36	0.002056442
HSD17B11	9.91E-08	-0.312376022	0.518	0.71	0.002161742
ALDH2	9.98E-08	-0.303467793	0.344	0.559	0.002175385
THBD	1.13E-07	0.466951907	0.401	0.224	0.00245855
GLRX	1.17E-07	-0.279354202	0.449	0.673	0.002558901
RGS18	1.20E-07	-0.267194757	0.217	0.423	0.002606109
PLA2G7	1.27E-07	0.304401689	0.162	0.033	0.002768358
GLTSCR2	1.30E-07	-0.285644423	0.344	0.566	0.002834601
CTB-61M7.2	1.31E-07	0.387203035	0.284	0.121	0.002853902

HLA-DMA	1.42E-07	-0.252669627	0.442	0.647	0.003086354
PTGER2	1.44E-07	0.420779807	0.496	0.346	0.003146866
MRPS24	1.45E-07	-0.277408149	0.255	0.467	0.003154659
EIF4B	1.56E-07	-0.262113027	0.442	0.654	0.003403061
SNHG8	1.75E-07	-0.292583042	0.339	0.57	0.003813915
BACH1	1.87E-07	0.416857266	0.544	0.401	0.004068766
LAP3	1.89E-07	0.360581335	0.449	0.294	0.004111827
TREM1	1.90E-07	0.396883711	0.544	0.397	0.004150474
NDUFS7	1.93E-07	-0.282270726	0.406	0.618	0.004200939
FRMD4B	2.01E-07	-0.305668358	0.045	0.162	0.004384256
UBE2J1	2.16E-07	0.36636228	0.656	0.577	0.004712598
RPAIN	2.22E-07	-0.25016835	0.093	0.239	0.00483616
EMP1	2.43E-07	0.442740964	0.208	0.07	0.005297923
UBE2D1	2.45E-07	0.310339117	0.695	0.614	0.005342582
TNFAIP6	2.50E-07	0.694685223	0.363	0.213	0.005444679
APLP2	2.67E-07	0.33313803	0.845	0.798	0.005818892
CES1	2.83E-07	0.386962051	0.301	0.143	0.00616381
CORO1B	2.86E-07	-0.277451278	0.203	0.39	0.006243508
IL10RA	2.91E-07	-0.306737577	0.303	0.511	0.006353134
STK17A	2.96E-07	0.358179881	0.296	0.14	0.00645339
MESDC1	3.20E-07	0.323801684	0.217	0.077	0.006980568
CDKN2D	3.21E-07	0.353829564	0.351	0.18	0.007000825
FTH1	3.35E-07	0.264104464	1	1	0.007308109
CSRNP1	3.36E-07	0.416466265	0.482	0.327	0.007333036
MAP2K1	3.51E-07	0.395690354	0.582	0.489	0.007659114
JAZF1	3.61E-07	-0.252801148	0.129	0.29	0.007861965
MX1	3.63E-07	0.349849248	0.134	0.022	0.007911986
SBNO2	3.69E-07	0.320263738	0.184	0.055	0.008053512
FOSB	4.46E-07	-0.273273984	0.74	0.864	0.009736066
HIPK2	4.48E-07	0.396246911	0.346	0.195	0.009768451
HPGD	5.04E-07	0.445791645	0.248	0.107	0.010999061
TMBIM4	5.44E-07	-0.269375099	0.451	0.662	0.011859896
HIST2H2AA4	6.13E-07	0.314610131	0.229	0.088	0.01336864
RBP7	6.41E-07	-0.295810777	0.294	0.496	0.013978252
SNHG12	6.55E-07	-0.265737053	0.11	0.257	0.014291395
ITGA4	7.16E-07	-0.299218852	0.241	0.426	0.015622815
IL1R1	7.21E-07	0.31375186	0.131	0.022	0.01572682
USB1	7.62E-07	0.303996719	0.232	0.092	0.016622792
ARL6IP5	7.62E-07	-0.279131784	0.499	0.717	0.016623389
FCAR	8.31E-07	0.307879985	0.298	0.143	0.018121032
APEX1	8.35E-07	-0.280528037	0.198	0.375	0.018213072
SERPINB10	8.63E-07	0.268929542	0.117	0.015	0.018819613
PTPRE	8.75E-07	0.318725038	0.795	0.743	0.019072957
GSTO1	8.79E-07	0.335852912	0.671	0.57	0.019165037
MPHOSPH6	9.15E-07	0.365027912	0.339	0.184	0.019964633
DYSF	9.21E-07	0.273517167	0.234	0.088	0.020084094
PLBD1	9.79E-07	0.351061493	0.649	0.548	0.021344999
ATG16L2	9.80E-07	-0.257705514	0.153	0.32	0.021382712
EIF4A2	1.02E-06	-0.250371698	0.339	0.548	0.022174759
SMIM3	1.07E-06	0.41555917	0.291	0.151	0.023292578
TMEM120A	1.35E-06	0.36054354	0.387	0.254	0.029369323
POR	1.37E-06	0.312503952	0.289	0.14	0.029955243
HSPA6	1.46E-06	-0.311857072	0.079	0.206	0.031786179
EIF3L	1.62E-06	-0.256158343	0.556	0.746	0.035278298
TTC3	1.80E-06	-0.265382009	0.131	0.272	0.039197922
ZC3H12A	1.82E-06	0.388116194	0.401	0.25	0.03958482
TRMT1	1.89E-06	-0.275733943	0.232	0.408	0.041152755
PDE4A	2.19E-06	0.292464028	0.184	0.062	0.047782332
CDC42	2.29E-06	0.319368393	0.776	0.768	0.049928887

List of differentially expressed genes of S100A8_TMo in reperfusion stage (PR versus EP)

gene	p_val	avg_logFC	pct.1	pct.2	p_val_adj
VCAN	2.24E-30	0.754061723	0.963	0.845	4.88E-26
THBS1	2.48E-28	1.22191915	0.873	0.53	5.42E-24
SRGN	1.23E-24	0.475324039	0.997	0.989	2.68E-20
SAT1	6.69E-24	0.608034365	0.973	0.928	1.46E-19
S100A9	2.40E-22	0.745420421	0.99	0.978	5.24E-18
ACSL1	6.74E-22	1.092354744	0.679	0.282	1.47E-17

HLA-DRB5	6.51E-21	-0.978050161	0.157	0.547	1.42E-16
SAMSN1	1.60E-19	0.798672552	0.833	0.497	3.50E-15
FPR2	3.73E-18	0.968634026	0.538	0.155	8.14E-14
RPS13	5.96E-18	-0.380958587	0.993	0.983	1.30E-13
RPS29	8.22E-18	0.711295451	0.816	0.575	1.79E-13
S100A12	9.72E-18	0.529752137	0.973	0.939	2.12E-13
SLC39A8	4.27E-17	1.084179474	0.348	0.017	9.30E-13
TNFAIP6	7.55E-17	0.971843616	0.545	0.144	1.65E-12
PAG1	9.73E-17	0.913574631	0.565	0.193	2.12E-12
SELL	1.16E-16	0.806635335	0.739	0.464	2.53E-12
NAMPT	1.52E-16	0.654943894	0.916	0.696	3.32E-12
TRMT6	9.37E-16	0.963823361	0.395	0.061	2.04E-11
ACTB	4.44E-15	0.252749088	1	1	9.67E-11
CKAP4	7.14E-15	0.850118165	0.518	0.171	1.56E-10
RPL10	8.02E-15	-0.334246438	0.99	0.989	1.75E-10
RPS3A	1.04E-14	-0.311440071	0.987	0.994	2.27E-10
CST3	4.46E-14	-0.408681832	0.97	0.945	9.72E-10
PLAC8	4.79E-14	0.92722535	0.552	0.238	1.04E-09
BCL2A1	8.34E-14	0.652375926	0.803	0.497	1.82E-09
H3F3B	1.08E-13	0.380075874	0.946	0.945	2.35E-09
RPL11	1.29E-13	-0.272874479	0.993	0.994	2.81E-09
MALAT1	1.36E-13	-0.312962876	1	1	2.96E-09
AREG	1.40E-13	0.870305487	0.742	0.425	3.05E-09
DEFA3	3.46E-13	-1.718662953	0.03	0.243	7.54E-09
VIM	5.68E-13	-0.327715559	0.983	0.994	1.24E-08
PABPC1	6.72E-13	-0.511963615	0.779	0.89	1.47E-08
RPL30	8.29E-13	-0.295825835	1	1	1.81E-08
GPX4	8.93E-13	-0.742767041	0.385	0.657	1.95E-08
RETN	1.88E-12	1.076883768	0.498	0.199	4.10E-08
FCER1G	2.18E-12	0.438661455	0.99	0.961	4.75E-08
HLA-DQB1	2.53E-12	-0.761925466	0.094	0.348	5.51E-08
NKG7	2.73E-12	0.747450036	0.408	0.105	5.95E-08
MT-CYB	3.38E-12	0.441254453	0.993	0.972	7.37E-08
CRIP1	3.40E-12	-0.940450568	0.341	0.597	7.43E-08
S100A8	3.62E-12	0.290724157	0.997	1	7.90E-08
JUN	3.93E-12	-0.811068105	0.294	0.575	8.58E-08
ASPH	6.24E-12	0.818193826	0.388	0.099	1.36E-07
FPR1	6.47E-12	0.575470492	0.749	0.519	1.41E-07
RPL14	1.48E-11	-0.375955959	0.923	0.961	3.23E-07
AGFG1	5.79E-11	0.729568448	0.532	0.254	1.26E-06
RPL18	7.08E-11	-0.321127677	0.957	0.972	1.55E-06
RPL5	7.49E-11	-0.368466347	0.839	0.923	1.63E-06
CFL1	8.36E-11	0.369263129	0.94	0.856	1.82E-06
CD63	1.24E-10	0.621247309	0.699	0.481	2.70E-06
MCEMP1	1.42E-10	0.755487274	0.555	0.304	3.10E-06
CD74	1.65E-10	-0.472996047	0.856	0.906	3.61E-06
NACA	1.88E-10	-0.358115933	0.923	0.956	4.10E-06
TYMP	2.02E-10	0.565201878	0.709	0.47	4.41E-06
CD36	3.97E-10	0.745040548	0.405	0.144	8.67E-06
SEC11A	4.51E-10	-0.579790445	0.455	0.702	9.84E-06
TPM4	4.71E-10	0.700104251	0.512	0.232	1.03E-05
AP1S2	4.86E-10	-0.569220883	0.522	0.718	1.06E-05
FOS	5.05E-10	-0.33379616	0.963	0.983	1.10E-05
CYBA	5.77E-10	0.413937124	0.849	0.79	1.26E-05
STXBP2	7.47E-10	0.471655469	0.876	0.746	1.63E-05
BTG1	7.69E-10	-0.533557195	0.602	0.757	1.68E-05
SERP1	8.60E-10	-0.447025766	0.732	0.845	1.88E-05
IL1R2	1.01E-09	0.730305337	0.602	0.354	2.21E-05
FGL2	1.03E-09	-0.605601603	0.274	0.536	2.24E-05
CLU	1.23E-09	0.723291384	0.338	0.094	2.68E-05
PFKFB3	1.34E-09	0.647284679	0.301	0.061	2.91E-05
LCP1	1.60E-09	0.437489357	0.833	0.669	3.50E-05
S100P	1.92E-09	0.724070212	0.268	0.044	4.19E-05
ADM	2.79E-09	0.704522289	0.361	0.116	6.09E-05
G0S2	3.20E-09	0.665311232	0.348	0.105	6.98E-05
LGALS2	3.55E-09	-0.607226171	0.054	0.232	7.74E-05
BAZ1A	3.74E-09	0.646296069	0.515	0.282	8.16E-05
RUNX1	3.94E-09	0.662655592	0.395	0.16	8.60E-05
NUP214	4.04E-09	-0.520805705	0.211	0.47	8.82E-05
PGAM1	4.79E-09	0.503233129	0.609	0.365	0.000104516

GK5	6.00E-09	0.658479005	0.181	0.006	0.000130944
HVCN1	6.43E-09	-0.394808861	0.01	0.138	0.000140189
SERPINB1	6.87E-09	0.342960441	0.91	0.823	0.000149723
SLC25A6	7.28E-09	-0.48477251	0.565	0.729	0.000158813
EVI2B	9.19E-09	-0.641593065	0.411	0.619	0.000200329
HP	9.40E-09	0.61143513	0.284	0.061	0.00020506
UPP1	9.97E-09	0.623045552	0.565	0.348	0.000217452
CTSS	1.06E-08	-0.337108689	0.809	0.878	0.000231258
TPM3	1.19E-08	0.453345649	0.776	0.597	0.000259606
PKM	1.30E-08	0.498308991	0.742	0.591	0.000283847
CST7	1.36E-08	0.71776288	0.274	0.061	0.000297487
ARHGDIA	1.61E-08	0.634182779	0.505	0.282	0.000350895
TIMP1	1.74E-08	0.783033592	0.766	0.608	0.000378915
MYL6	1.74E-08	0.276210755	0.98	0.939	0.000379952
HBB	1.80E-08	-1.005077632	0.054	0.221	0.000392932
RAB31	2.08E-08	0.559805571	0.635	0.448	0.000453648
BZW1	2.15E-08	0.550359536	0.435	0.188	0.000467859
MS4A7	2.30E-08	-0.714243111	0.114	0.32	0.000500572
TXNIP	3.42E-08	-0.687020816	0.278	0.492	0.000745713
RGS10	3.44E-08	-0.574286933	0.144	0.354	0.000751164
CD37	3.99E-08	-0.473673363	0.518	0.74	0.000869772
EEF1D	4.05E-08	-0.35024627	0.866	0.939	0.000883516
RGS2	4.48E-08	-0.483007408	0.538	0.746	0.000976682
GRINA	4.86E-08	0.516733469	0.652	0.448	0.00105883
SLC11A1	5.28E-08	0.514992677	0.682	0.481	0.001152226
AQP9	5.40E-08	0.523911399	0.418	0.177	0.001177841
HSPA8	6.53E-08	-0.458327898	0.288	0.525	0.001423857
MNDA	6.61E-08	-0.427684837	0.739	0.873	0.00144079
RPS4X	8.90E-08	-0.280894714	0.933	0.945	0.001941663
DOK2	1.13E-07	-0.466429233	0.12	0.32	0.002458226
CD14	1.17E-07	0.56384562	0.672	0.503	0.002549171
SLC25A5	1.26E-07	-0.528792835	0.418	0.624	0.002742797
RNASE6	1.27E-07	-0.576342904	0.214	0.42	0.002767434
PLSCR1	1.37E-07	0.582507766	0.535	0.315	0.00298538
CSTA	1.57E-07	-0.415150784	0.783	0.878	0.00341496
GCA	1.79E-07	0.384941047	0.843	0.823	0.003896096
ARPC3	2.42E-07	-0.286738904	0.819	0.923	0.005270576
RBMS1	2.48E-07	0.64600808	0.441	0.227	0.005418598
STOM	2.71E-07	0.450860681	0.348	0.127	0.005909926
SOD2	2.72E-07	0.390249758	0.789	0.586	0.005924617
MS4A4A	3.16E-07	0.462090154	0.284	0.088	0.00688161
EVI2A	3.18E-07	-0.512861657	0.084	0.254	0.00693268
PICK1	3.34E-07	-0.343793057	0.027	0.155	0.007279009
FAM26F	3.34E-07	-0.56629116	0.047	0.193	0.007286254
MCTP2	3.41E-07	0.549797158	0.214	0.044	0.007431408
TKT	3.73E-07	-0.43526956	0.609	0.746	0.008144234
C12orf57	4.68E-07	-0.55565438	0.104	0.282	0.010196593
RHOB	5.54E-07	-0.511803487	0.405	0.608	0.012078048
TNFAIP3	5.92E-07	0.58696074	0.559	0.32	0.0129026
CPM	6.96E-07	-0.393005878	0.033	0.16	0.015172856
FXYD5	7.77E-07	-0.429553646	0.622	0.762	0.016939426
METTL7B	7.86E-07	0.432334519	0.137	0.006	0.01714899
GIMAP2	8.26E-07	-0.377232826	0.05	0.193	0.01800569
LRG1	8.94E-07	0.441833178	0.204	0.039	0.019499261
RBKS	9.38E-07	-0.469960232	0.06	0.21	0.020465676
JUNB	9.67E-07	-0.410462194	0.739	0.829	0.021086753
SULT1A1	1.02E-06	-0.432172787	0.127	0.309	0.022335044
AMPH	1.05E-06	-0.309589616	0.013	0.116	0.022816157
PTCH2	1.05E-06	-0.467758144	0.023	0.138	0.022917726
MCL1	1.08E-06	0.345408787	0.816	0.68	0.023575284
IFI30	1.28E-06	0.41462762	0.709	0.547	0.02794868
HSD17B11	1.46E-06	-0.472350209	0.331	0.541	0.031787473
COMMD6	1.58E-06	-0.319051434	0.562	0.751	0.034505658
CLEC4E	1.61E-06	-0.56042719	0.311	0.508	0.035072043
DDX21	1.62E-06	0.53828902	0.508	0.315	0.035320066
CD82	1.62E-06	0.48963867	0.201	0.044	0.035392094
BTF3	1.63E-06	-0.339133791	0.819	0.867	0.035490506
F5	1.85E-06	0.454297816	0.217	0.055	0.040291082
SLC2A3	1.88E-06	0.451074724	0.699	0.525	0.04089271
APLP2	1.99E-06	0.431344115	0.706	0.569	0.043502331

SERPINB2	2.07E-06	0.511804177	0.291	0.105	0.04520706
MAFB	2.11E-06	0.52667567	0.632	0.453	0.04602892
SQRDL	2.21E-06	0.512484488	0.351	0.166	0.048102448
B4GALT5	2.27E-06	0.544715858	0.271	0.094	0.049466391

List of differentially expressed genes of IL1B_KC in reperfusion stage (PR versus EP)

gene	p_val	avg_logFC	pct.1	pct.2	p_val_adj
PIM1	3.46E-17	1.092706203	0.676	0.117	7.54E-13
NAMPT	1.30E-16	0.857958464	0.973	0.933	2.84E-12
SOD2	3.76E-15	0.953435768	0.986	0.917	8.20E-11
CTSL	3.98E-15	1.307436789	0.892	0.6	8.68E-11
SOCS3	1.53E-14	1.015779864	0.851	0.558	3.33E-10
ACSL1	4.03E-14	1.019626862	0.824	0.533	8.78E-10
THBS1	5.82E-14	1.728442141	0.77	0.308	1.27E-09
RHOB	6.93E-13	-0.985495477	0.797	0.967	1.51E-08
LITAF	9.52E-13	1.079806068	0.878	0.65	2.08E-08
TNIP3	2.06E-12	1.11640451	0.378	0.008	4.50E-08
DSE	2.12E-12	0.795549714	0.703	0.25	4.63E-08
H3F3B	2.27E-12	0.635668098	1	0.983	4.95E-08
NFKBIZ	2.87E-12	-0.85562584	0.541	0.917	6.25E-08
PIM3	3.19E-12	0.924817423	0.662	0.233	6.96E-08
BATF	2.18E-11	0.911415094	0.473	0.083	4.75E-07
ZFP36L2	2.87E-11	-0.797996142	0.554	0.917	6.26E-07
TIMP1	3.41E-11	0.905331466	0.959	0.933	7.43E-07
CDKN1A	4.91E-11	0.88470146	0.878	0.633	1.07E-06
HSP90AA1	5.40E-11	1.404129638	0.946	0.942	1.18E-06
TMSB4X	5.82E-11	-0.432367356	0.986	1	1.27E-06
FPR1	6.34E-11	0.77890929	0.838	0.558	1.38E-06
MIR3945HG	1.28E-10	0.964583886	0.459	0.075	2.79E-06
GIMAP2	1.56E-10	-0.595905115	0.068	0.533	3.41E-06
CXCR4	1.63E-10	-0.816275807	0.5	0.842	3.56E-06
HSPE1	1.86E-10	1.579556638	0.824	0.75	4.06E-06
B4GALT5	2.18E-10	0.694410159	0.595	0.183	4.74E-06
AREG	2.22E-10	0.9401154	0.878	0.458	4.85E-06
MAP3K8	2.31E-10	0.707170328	0.892	0.658	5.04E-06
SLC25A37	3.97E-10	0.861299781	0.581	0.183	8.66E-06
NINJ1	4.66E-10	0.930249873	0.878	0.625	1.02E-05
HSPD1	8.00E-10	1.838673195	0.757	0.65	1.74E-05
DUSP1	9.40E-10	-0.440354896	1	1	2.05E-05
NFKB2	2.32E-09	0.71228654	0.514	0.15	5.05E-05
C1orf162	2.38E-09	-0.548370758	0.757	0.933	5.18E-05
LDHA	2.47E-09	0.575303056	0.878	0.75	5.40E-05
ATP13A3	3.10E-09	0.675090561	0.527	0.142	6.77E-05
PABPC1	3.83E-09	-0.41586248	0.986	0.992	8.35E-05
BAG3	6.10E-09	1.367763597	0.486	0.142	0.00013305
G0S2	6.77E-09	1.218861006	0.676	0.342	0.000147608
HSPH1	6.91E-09	1.738798788	0.73	0.575	0.000150715
DNAJA4	8.56E-09	1.126301691	0.338	0.042	0.000186634
LRG1	1.57E-08	0.668819738	0.392	0.075	0.000342603
LIMK2	1.74E-08	0.542888722	0.338	0.042	0.000380115
HIF1A	1.96E-08	0.73042813	0.743	0.475	0.000427678
TXNIP	2.05E-08	-0.909363189	0.514	0.817	0.000447413
RELB	2.15E-08	0.577804512	0.527	0.167	0.000468904
IL1RN	2.46E-08	1.504303561	0.527	0.192	0.000537552
HSPA5	2.83E-08	1.190958151	0.811	0.592	0.000616936
PTPN2	3.36E-08	0.647134974	0.676	0.408	0.000732566
PNP	4.87E-08	0.716538857	0.568	0.25	0.001061267
KLF2	5.68E-08	-0.628581537	0.351	0.792	0.00123818
HSPB1	6.89E-08	1.937651851	0.743	0.558	0.001501785
THBD	7.93E-08	0.784826987	0.473	0.142	0.001730002
BCL3	8.18E-08	0.624751386	0.554	0.208	0.00178394
FPR2	9.13E-08	0.949854316	0.541	0.233	0.001990646
GIMAP4	1.33E-07	-0.654287875	0.257	0.642	0.002904561
FTH1	1.33E-07	0.32861885	1	1	0.002907881
HLA-DRB5	1.98E-07	-0.456482394	0.851	1	0.004323799
AQP9	2.04E-07	0.676004758	0.581	0.258	0.004440209
PVRL2	2.12E-07	0.702959522	0.419	0.133	0.004616867

CALM2	3.77E-07	-0.468693668	0.851	0.942	0.008226876
IFNGR1	4.34E-07	-0.482919429	0.514	0.858	0.009459625
BIRC3	4.35E-07	0.602238683	0.662	0.35	0.009489338
ARHGDIB	4.78E-07	-0.47164867	0.757	0.942	0.010434266
FCGR1B	5.36E-07	0.753777736	0.446	0.142	0.011687137
SERPINB1	6.44E-07	0.647941388	0.851	0.858	0.014037904
TRIP10	6.96E-07	0.389891496	0.216	0.008	0.015184364
C15orf48	7.71E-07	1.201955196	0.338	0.075	0.01682024
UPP1	8.63E-07	0.653365123	0.77	0.592	0.018820566
SAMSN1	8.95E-07	0.585572368	0.932	0.792	0.019519101
FOS	9.07E-07	-0.333921065	0.973	1	0.019769643
LMNA	9.89E-07	0.724950599	0.365	0.092	0.021577586
SLC2A3	1.06E-06	0.722880271	0.649	0.392	0.023037051
CACYBP	1.53E-06	0.689450514	0.635	0.425	0.033333956
MEF2C	1.54E-06	-0.494021114	0.203	0.575	0.033526309
OPTN	1.62E-06	0.312302807	0.203	0.008	0.035418546
FLI1	1.96E-06	-0.396131545	0.189	0.542	0.042765564
GK	2.12E-06	0.757715954	0.676	0.4	0.046268396
C10orf54	2.13E-06	-0.414519092	0.73	0.917	0.046453855
HMGN3	2.26E-06	-0.499299625	0.311	0.683	0.049193088

Table 5: List of DEGs of mononuclear phagocyte clusters in overall stage (PR versus PP)

List of differentially expressed genes of C1QC_KC in overall stage (PR versus PP)

gene	p_val	avg_logFC	pct.1	pct.2	p_val_adj
SOD2	3.44E-135	1.601678008	0.985	0.69	7.49E-131
HSP90AA1	9.61E-134	1.944290828	0.983	0.874	2.10E-129
HSPD1	1.19E-131	2.175357118	0.937	0.521	2.59E-127
HSPH1	3.88E-130	2.318295652	0.899	0.355	8.47E-126
SOCS3	2.39E-126	1.54513476	0.895	0.267	5.21E-122
BAG3	1.04E-115	2.03100127	0.727	0.071	2.26E-111
HSPE1	4.00E-111	1.872644533	0.918	0.592	8.73E-107
HSPB1	2.04E-110	2.17386554	0.91	0.521	4.45E-106
HSPA1A	1.22E-108	1.697704987	0.979	0.748	2.65E-104
G0S2	1.50E-106	2.122737602	0.739	0.098	3.27E-102
HSPA5	4.81E-102	1.537160465	0.924	0.594	1.05E-97
BATF	4.17E-101	1.183950729	0.668	0.063	9.09E-97
LITAF	1.46E-98	1.346640383	0.891	0.517	3.18E-94
CDKN1A	5.25E-93	1.246162565	0.908	0.481	1.15E-88
DNAJA4	5.73E-92	1.491110766	0.59	0.035	1.25E-87
AREG	1.92E-91	1.533646326	0.859	0.257	4.18E-87
NAMPT	1.12E-88	1.048233644	0.96	0.765	2.45E-84
MT2A	5.95E-85	2.109339325	0.939	0.755	1.30E-80
TMSB4X	2.01E-84	-0.602941624	0.996	0.993	4.38E-80
ICAM1	3.59E-80	1.414685496	0.83	0.396	7.83E-76
THBS1	2.51E-76	1.525133145	0.788	0.299	5.47E-72
C1QC	1.16E-72	-0.696276149	0.956	0.964	2.54E-68
ACSL1	6.29E-72	1.055528915	0.798	0.393	1.37E-67
PDK4	3.02E-71	-1.365991126	0.305	0.793	6.59E-67
HSP90AB1	3.07E-70	1.012410793	0.954	0.834	6.69E-66
C15orf48	1.30E-67	1.452260289	0.504	0.051	2.83E-63
SERPINB1	2.90E-67	0.993735201	0.889	0.678	6.33E-63
PIM1	2.76E-66	0.935058565	0.616	0.143	6.03E-62
DNAJB6	4.12E-66	1.075911902	0.828	0.486	8.98E-62
DNAJB1	2.66E-64	1.344808503	0.884	0.597	5.80E-60
LMNA	4.80E-64	1.107090844	0.616	0.151	1.05E-59
ZFAND2A	6.65E-64	1.38592985	0.592	0.144	1.45E-59
PIM3	2.38E-63	0.903368097	0.588	0.136	5.19E-59
SLC40A1	1.16E-62	-1.224090597	0.62	0.892	2.54E-58
SNHG15	2.36E-59	0.861827593	0.685	0.239	5.15E-55
CTSL	4.47E-59	1.047123	0.916	0.789	9.75E-55
RELB	9.81E-59	0.746435627	0.508	0.086	2.14E-54
IFNGR1	1.06E-58	-0.83930506	0.622	0.891	2.31E-54
MS4A6A	3.72E-58	-0.557448596	0.96	0.973	8.12E-54
SLC7A5	4.30E-58	0.723714593	0.433	0.033	9.39E-54
LRG1	1.54E-57	0.853073282	0.418	0.03	3.36E-53
ABL2	3.48E-55	0.787780233	0.498	0.086	7.59E-51
SLCO4A1	1.34E-54	0.731323833	0.46	0.061	2.93E-50
NFKB1	1.03E-53	0.887358429	0.569	0.151	2.25E-49
IL1RN	2.35E-53	1.323930493	0.58	0.161	5.12E-49
ATP13A3	2.80E-53	0.800225294	0.521	0.109	6.10E-49
MIR3945HG	5.73E-53	0.788456757	0.393	0.028	1.25E-48
RPS29	1.04E-52	0.551654644	0.952	0.902	2.26E-48
FKBP4	1.06E-52	0.986397506	0.517	0.118	2.31E-48
TXNIP	1.34E-52	-0.864835758	0.504	0.847	2.93E-48
GIMAP7	1.56E-52	-0.757908422	0.061	0.488	3.40E-48
CPVL	4.28E-52	-0.778497639	0.571	0.831	9.34E-48
DEFA3	9.20E-52	-0.948157883	0.036	0.449	2.01E-47
MAP3K8	4.08E-51	0.752913347	0.866	0.587	8.91E-47
P2RY13	9.09E-51	-0.812060073	0.124	0.564	1.98E-46
GK	1.25E-50	0.902056183	0.712	0.335	2.72E-46
DNAJA1	2.07E-50	0.879610537	0.847	0.62	4.51E-46
NFKB2	3.00E-49	0.68966694	0.466	0.088	6.54E-45
B4GALT5	3.05E-49	0.650992239	0.445	0.068	6.65E-45
SELK	3.17E-49	0.937478827	0.788	0.476	6.91E-45
H3F3B	1.31E-48	0.500953391	0.989	0.965	2.87E-44
RP11-670E13.6	4.45E-48	0.90469449	0.607	0.206	9.71E-44
PNRC1	6.05E-48	0.637599977	0.947	0.831	1.32E-43
TNIP3	6.29E-48	0.787699515	0.328	0.01	1.37E-43
PELI1	7.37E-48	0.898658609	0.739	0.42	1.61E-43
PAPSS2	4.10E-46	0.802037502	0.674	0.272	8.94E-42
THBD	6.14E-46	0.901071747	0.546	0.167	1.34E-41

C1QB	7.40E-46	-0.503992835	0.952	0.967	1.61E-41
BCL3	1.90E-45	0.641044386	0.492	0.114	4.15E-41
SAMSN1	4.50E-44	0.768935614	0.868	0.642	9.82E-40
CACYBP	5.45E-44	0.89438807	0.714	0.416	1.19E-39
PLSCR1	1.20E-43	0.742369324	0.775	0.471	2.61E-39
PLAUR	3.56E-42	0.728065738	0.847	0.537	7.77E-38
FTH1	8.97E-42	0.429783578	1	1	1.96E-37
HIF1A	1.09E-41	0.796390983	0.752	0.441	2.38E-37
SLC2A3	2.55E-41	0.810965322	0.628	0.255	5.56E-37
INSIG1	7.46E-41	0.939060683	0.704	0.385	1.63E-36
CXCL10	1.47E-40	1.482197271	0.349	0.041	3.21E-36
B3GNT5	5.40E-40	0.680584732	0.395	0.07	1.18E-35
IRF1	7.57E-40	0.745028306	0.653	0.307	1.65E-35
LRRFIP1	1.08E-39	0.694925859	0.811	0.589	2.36E-35
PTPN1	2.61E-39	0.651471212	0.666	0.33	5.68E-35
MS4A4A	3.30E-39	-0.572597235	0.84	0.922	7.21E-35
WTAP	4.07E-39	0.770392704	0.632	0.325	8.87E-35
FOSL2	1.58E-38	0.660851952	0.565	0.212	3.45E-34
NINJ1	2.56E-38	0.779361979	0.87	0.723	5.59E-34
UPP1	4.57E-38	0.687401469	0.735	0.436	9.97E-34
RNASE6	5.41E-38	-0.633954005	0.634	0.846	1.18E-33
CREM	7.13E-37	0.709585752	0.664	0.332	1.55E-32
EMP1	4.59E-36	0.752715879	0.387	0.078	1.00E-31
TRIM25	4.69E-36	0.55187487	0.393	0.081	1.02E-31
CTSS	4.80E-36	-0.431521525	0.941	0.973	1.05E-31
CALM2	3.17E-35	-0.510604406	0.834	0.925	6.92E-31
CD44	1.11E-34	0.641199521	0.836	0.632	2.43E-30
HBB	1.11E-34	-0.783255589	0.12	0.459	2.43E-30
ARL5B	1.46E-34	0.744479582	0.532	0.204	3.18E-30
DDX21	1.83E-34	0.649113206	0.691	0.416	3.99E-30
TWISTNB	2.08E-34	0.963063057	0.481	0.174	4.54E-30
PFKFB3	2.60E-34	0.613919707	0.622	0.297	5.68E-30
SDS	2.75E-34	1.140943978	0.433	0.121	6.00E-30
SPHK1	4.23E-34	0.4354706	0.286	0.028	9.22E-30
B2M	6.81E-34	-0.302267411	1	0.997	1.48E-29
MAP2K3	8.73E-34	0.55667401	0.588	0.259	1.90E-29
ARID5A	1.44E-33	0.645731578	0.54	0.231	3.14E-29
CCRL2	1.69E-33	0.630197012	0.508	0.187	3.69E-29
SDF2L1	2.56E-33	0.650535444	0.569	0.255	5.58E-29
GIMAP4	3.15E-33	-0.606563479	0.303	0.635	6.87E-29
MS4A7	3.90E-33	-0.511145348	0.887	0.934	8.51E-29
LDLR	5.59E-33	0.477847177	0.286	0.032	1.22E-28
SBNO2	1.18E-32	0.45235291	0.315	0.046	2.58E-28
BCL2A1	1.28E-32	0.471745037	0.815	0.493	2.79E-28
SEMA6B	2.01E-32	0.454039181	0.244	0.013	4.39E-28
ADORA3	2.11E-32	-0.499288124	0.057	0.362	4.60E-28
C1orf162	2.77E-32	-0.508608688	0.807	0.909	6.04E-28
FPR1	3.10E-32	0.626119112	0.733	0.483	6.75E-28
TNFAIP3	3.35E-32	0.648324832	0.79	0.458	7.30E-28
PDE4B	9.67E-32	0.57521712	0.584	0.254	2.11E-27
GPR34	1.03E-31	-0.661676267	0.376	0.667	2.25E-27
AZIN1	2.20E-31	0.588113676	0.571	0.27	4.79E-27
ETS2	3.19E-31	0.601281287	0.813	0.645	6.96E-27
SAT1	4.63E-31	0.378861414	1	1	1.01E-26
BIRC3	8.86E-31	0.632888605	0.475	0.167	1.93E-26
REL	1.40E-30	0.632699101	0.79	0.592	3.05E-26
RPS27	1.94E-30	0.313597344	0.994	0.99	4.24E-26
KDM6B	2.03E-30	0.542490009	0.58	0.269	4.43E-26
LDHA	2.05E-30	0.612135368	0.796	0.622	4.47E-26
SERPINB9	2.22E-30	0.611493712	0.55	0.245	4.83E-26
PPP1CB	2.23E-30	0.609795466	0.639	0.363	4.86E-26
MTHFD2	2.63E-30	0.614755737	0.628	0.348	5.74E-26
C1QA	2.70E-30	-0.381274821	0.958	0.968	5.88E-26
MRPS6	3.15E-30	0.644847244	0.511	0.212	6.88E-26
HES4	3.43E-30	0.480182631	0.214	0.007	7.48E-26
DNAJB9	4.45E-30	0.60119184	0.557	0.269	9.71E-26
DRAM1	4.95E-30	0.563939214	0.553	0.26	1.08E-25
PVRL2	5.37E-30	0.588622471	0.542	0.25	1.17E-25
TCP1	5.82E-30	0.58682655	0.586	0.302	1.27E-25
RP11-295G20.2	6.22E-30	0.546294353	0.351	0.08	1.36E-25

NEAT1	6.42E-30	0.367265496	0.979	0.967	1.40E-25
FOS	6.72E-30	-0.578821217	0.943	0.975	1.46E-25
GIMAP1	6.83E-30	-0.503506978	0.174	0.489	1.49E-25
PTP4A1	7.38E-30	0.613033692	0.59	0.299	1.61E-25
YBX3	7.90E-30	0.565996088	0.666	0.386	1.72E-25
RGCC	1.99E-29	0.643017876	0.357	0.08	4.34E-25
CD163	2.59E-29	-0.393966754	0.956	0.973	5.65E-25
TFRC	2.60E-29	0.718848659	0.437	0.151	5.66E-25
SDC4	2.70E-29	0.586807921	0.336	0.071	5.88E-25
FUCA1	3.67E-29	-0.606968746	0.288	0.594	8.00E-25
MXD1	4.04E-29	0.628240391	0.58	0.287	8.81E-25
AIF1	6.67E-29	-0.366947335	0.966	0.978	1.46E-24
SLC25A37	1.71E-28	0.655806374	0.483	0.199	3.74E-24
PDE4DIP	1.76E-28	0.464472543	0.464	0.174	3.83E-24
HSPA1B	1.77E-28	0.929478928	0.79	0.534	3.86E-24
P4HA1	2.11E-28	0.572541758	0.532	0.252	4.60E-24
LIMK2	2.76E-28	0.422175255	0.256	0.032	6.01E-24
PPIF	3.05E-28	0.540485574	0.517	0.217	6.66E-24
ZFP36L1	3.16E-28	-0.496248239	0.752	0.922	6.90E-24
TAOK3	3.39E-28	0.620431633	0.666	0.41	7.40E-24
PHLDA2	6.99E-28	0.58659101	0.38	0.108	1.52E-23
CHMP1B	2.05E-27	0.678503877	0.754	0.499	4.47E-23
DDIT3	2.09E-27	0.560049004	0.605	0.322	4.56E-23
VAMP8	3.30E-27	-0.419531541	0.828	0.905	7.21E-23
KLHL6	3.41E-27	0.418409569	0.324	0.071	7.43E-23
WBP5	3.86E-27	0.54019574	0.269	0.043	8.42E-23
PNP	5.85E-27	0.630471693	0.534	0.255	1.28E-22
DSE	8.27E-27	0.573574407	0.586	0.325	1.80E-22
XBP1	1.12E-26	0.564433305	0.748	0.534	2.45E-22
MIDN	1.17E-26	0.495123602	0.632	0.338	2.54E-22
ELL2	1.33E-26	0.597151837	0.597	0.323	2.90E-22
TNFRSF10D	1.54E-26	0.465283707	0.334	0.078	3.36E-22
VSIG4	2.44E-26	-0.530232277	0.761	0.856	5.31E-22
APBB1IP	2.80E-26	-0.482902328	0.468	0.715	6.10E-22
CSF1R	3.04E-26	-0.477558906	0.725	0.841	6.63E-22
CST3	3.23E-26	-0.333202153	0.996	0.998	7.05E-22
ARRDC3	6.20E-26	0.507050092	0.546	0.26	1.35E-21
EREG	8.44E-26	0.525222972	0.389	0.108	1.84E-21
EMP3	9.50E-26	0.5368102	0.872	0.765	2.07E-21
TXN	9.65E-26	0.809030271	0.805	0.67	2.10E-21
ARHGDIB	1.01E-25	-0.411157939	0.792	0.867	2.21E-21
C5AR1	1.01E-25	0.512681489	0.882	0.68	2.21E-21
CD55	1.23E-25	0.557704703	0.788	0.612	2.68E-21
RPS21	1.43E-25	0.265409622	0.981	0.973	3.12E-21
HSP90B1	1.63E-25	0.532103528	0.905	0.789	3.55E-21
WASF2	1.71E-25	-0.481237533	0.353	0.642	3.72E-21
CD74	2.34E-25	-0.280287698	0.996	0.998	5.11E-21
EZR	2.79E-25	0.617621526	0.576	0.323	6.07E-21
EAF1	3.06E-25	0.439647443	0.336	0.09	6.67E-21
STIP1	8.04E-25	0.463439359	0.416	0.163	1.75E-20
UGCG	8.41E-25	0.509530104	0.403	0.144	1.83E-20
MCTP2	8.61E-25	0.34744357	0.216	0.022	1.88E-20
NPC2	1.45E-24	-0.278039418	0.975	0.982	3.16E-20
RAB27A	1.53E-24	0.384954881	0.239	0.035	3.33E-20
ODF3B	2.09E-24	0.481413333	0.523	0.252	4.56E-20
MYADM	2.27E-24	0.537626027	0.576	0.305	4.96E-20
HSPA8	2.50E-24	0.632721329	0.903	0.859	5.45E-20
FGD2	2.56E-24	-0.512783956	0.34	0.622	5.58E-20
RNF144B	3.58E-24	0.538113506	0.723	0.479	7.81E-20
NFIL3	4.24E-24	0.428465944	0.319	0.081	9.25E-20
GLIPR1	5.08E-24	-0.439809843	0.55	0.76	1.11E-19
PTPN2	5.97E-24	0.505099663	0.618	0.367	1.30E-19
PPP1R15A	6.56E-24	0.388756609	0.891	0.655	1.43E-19
TOM1	7.09E-24	0.459234733	0.439	0.181	1.55E-19
DNAJB11	1.03E-23	0.494661782	0.506	0.25	2.24E-19
TNFAIP8L2	1.79E-23	-0.300664062	0.034	0.26	3.91E-19
RPL39	2.02E-23	0.256487809	0.998	0.995	4.41E-19
HLA-DPA1	2.36E-23	-0.338440199	0.96	0.978	5.15E-19
HAMP	2.39E-23	0.931652289	0.179	0.01	5.21E-19
AHSA1	2.79E-23	0.516862082	0.443	0.201	6.09E-19

STAT3	3.18E-23	0.485265864	0.611	0.365	6.93E-19
ZFP36L2	3.66E-23	-0.502479041	0.601	0.829	7.97E-19
NUDC	3.85E-23	0.518361163	0.569	0.332	8.40E-19
SLAMF1	5.44E-23	0.394764654	0.151	0	1.19E-18
VEGFA	5.95E-23	0.400018339	0.288	0.065	1.30E-18
NAPA	6.40E-23	0.563428122	0.666	0.439	1.40E-18
OTUD1	9.84E-23	0.468606987	0.393	0.144	2.15E-18
SAMHD1	1.17E-22	-0.457790438	0.519	0.751	2.56E-18
ATF3	1.35E-22	0.402812791	0.695	0.4	2.93E-18
ANXA1	1.38E-22	0.715005614	0.769	0.539	3.02E-18
SLC16A10	1.44E-22	0.351517627	0.174	0.01	3.15E-18
CHSY1	2.51E-22	0.340491916	0.271	0.06	5.47E-18
FFAR3	2.61E-22	0.418139016	0.183	0.013	5.68E-18
MRPL18	2.61E-22	0.590313972	0.489	0.254	5.70E-18
MAFF	3.55E-22	0.48319715	0.378	0.136	7.73E-18
OLR1	5.28E-22	0.627858209	0.258	0.056	1.15E-17
ATP2A2	5.82E-22	0.478661722	0.487	0.237	1.27E-17
FCGR3A	6.82E-22	-0.390964971	0.847	0.891	1.49E-17
STAT4	9.76E-22	0.317054027	0.183	0.017	2.13E-17
CREB5	9.98E-22	0.407941682	0.273	0.061	2.18E-17
MCL1	1.26E-21	0.328270863	0.929	0.766	2.74E-17
DUSP2	1.30E-21	0.483751046	0.616	0.32	2.84E-17
HERPUD1	1.34E-21	0.451996952	0.882	0.781	2.93E-17
GNA13	1.48E-21	0.433819305	0.529	0.28	3.23E-17
FAM107B	1.51E-21	0.39701824	0.33	0.103	3.29E-17
EIF4A1	1.61E-21	0.436489546	0.742	0.559	3.51E-17
CALR	1.88E-21	0.434289844	0.84	0.73	4.09E-17
ATM	1.99E-21	-0.417024908	0.195	0.474	4.35E-17
METRNL	2.98E-21	0.410122506	0.317	0.095	6.51E-17
RNASET2	3.42E-21	-0.315377825	0.916	0.952	7.47E-17
VIM	3.63E-21	0.35084104	0.983	0.965	7.92E-17
CH25H	3.79E-21	0.717780029	0.181	0.017	8.26E-17
ATP1B3	5.20E-21	0.687803459	0.687	0.512	1.13E-16
HMGN3	6.66E-21	-0.445735364	0.353	0.619	1.45E-16
MTHFR	6.84E-21	0.441873136	0.431	0.191	1.49E-16
MIR155HG	6.85E-21	0.658461337	0.252	0.055	1.49E-16
EIF2AK3	9.42E-21	0.391999102	0.286	0.08	2.06E-16
NDRG1	1.04E-20	0.418829946	0.336	0.114	2.26E-16
EHD1	1.04E-20	0.408694335	0.353	0.126	2.27E-16
TMSB10	1.12E-20	-0.250716556	0.998	0.995	2.45E-16
LACTB	1.15E-20	0.409758446	0.418	0.182	2.51E-16
GPR65	1.62E-20	-0.426704406	0.382	0.643	3.54E-16
GABARAP	2.02E-20	-0.273300483	0.956	0.964	4.41E-16
MAP2K1	2.27E-20	0.496015709	0.544	0.305	4.94E-16
RTEL1-TNFRSF6	2.40E-20	0.35048627	0.147	0.005	5.23E-16
ITM2B	2.88E-20	-0.270758904	0.981	0.972	6.28E-16
UQCR10	3.41E-20	-0.430225733	0.641	0.801	7.44E-16
ARPC1B	5.18E-20	-0.343240585	0.815	0.877	1.13E-15
DNMBP	5.31E-20	0.341255996	0.212	0.036	1.16E-15
TMEM37	5.48E-20	-0.404021263	0.244	0.511	1.20E-15
ADGRE2	5.51E-20	0.520576703	0.601	0.393	1.20E-15
HNRNPC	5.83E-20	0.3769826	0.8	0.607	1.27E-15
CCNL1	6.18E-20	0.463593394	0.779	0.59	1.35E-15
SCIMP	6.46E-20	-0.423324453	0.275	0.536	1.41E-15
TM9SF2	6.59E-20	-0.447305	0.351	0.61	1.44E-15
BHLHE40	7.60E-20	0.437023729	0.613	0.386	1.66E-15
RYBP	9.16E-20	0.444252096	0.324	0.108	2.00E-15
PLIN2	1.09E-19	0.641762327	0.767	0.612	2.38E-15
LPAR6	1.32E-19	-0.386809781	0.111	0.348	2.88E-15
LCP2	1.74E-19	0.480523883	0.815	0.69	3.80E-15
GLUL	1.92E-19	0.455241692	0.945	0.902	4.18E-15
CTSZ	2.24E-19	0.495757211	0.739	0.575	4.88E-15
RPL36A	2.30E-19	0.365020253	0.914	0.854	5.01E-15
CD83	2.80E-19	0.434292066	0.836	0.577	6.11E-15
FOLR2	2.92E-19	-0.490169891	0.739	0.862	6.37E-15
HLA-DRB5	3.03E-19	-0.279356166	0.979	0.985	6.62E-15
FAM26F	4.09E-19	-0.370621537	0.714	0.844	8.92E-15
PLK3	4.16E-19	0.372243094	0.37	0.143	9.07E-15
RPL37A	4.40E-19	0.271091898	0.971	0.967	9.59E-15
MT1A	4.54E-19	0.672104054	0.139	0.005	9.90E-15

HSPA6	5.85E-19	1.225774648	0.464	0.26	1.27E-14
RBX1	6.11E-19	-0.358557382	0.33	0.579	1.33E-14
HCLS1	6.95E-19	-0.327300977	0.874	0.91	1.52E-14
LIPA	8.09E-19	-0.474686865	0.889	0.922	1.76E-14
RPS2	9.15E-19	0.300319585	0.96	0.937	2.00E-14
TNFSF10	9.87E-19	-0.354246058	0.116	0.352	2.15E-14
GPR84	1.10E-18	0.265322152	0.168	0.018	2.40E-14
GRN	1.23E-18	-0.376815641	0.874	0.899	2.68E-14
JUND	1.36E-18	0.462637073	0.704	0.469	2.96E-14
TIMP1	1.47E-18	0.675835437	0.918	0.877	3.21E-14
IGSF6	1.67E-18	-0.440926041	0.693	0.842	3.63E-14
PDE4A	1.74E-18	0.340163062	0.261	0.071	3.79E-14
NFE2L2	1.97E-18	0.440525441	0.721	0.559	4.30E-14
CXCR4	2.15E-18	-0.553234956	0.498	0.705	4.68E-14
N4BP2L2	2.32E-18	-0.430710742	0.357	0.619	5.07E-14
SULT1A1	2.81E-18	-0.423212799	0.41	0.643	6.12E-14
PAPD5	3.00E-18	0.387398713	0.29	0.093	6.54E-14
HHEX	3.01E-18	-0.312631622	0.057	0.257	6.56E-14
ZC3HAV1	3.22E-18	0.483067637	0.477	0.254	7.02E-14
DNTTIP2	4.22E-18	0.428466987	0.422	0.206	9.20E-14
ZC3H12A	4.29E-18	0.388923296	0.357	0.139	9.36E-14
LMNB1	4.40E-18	0.26552971	0.214	0.043	9.59E-14
RASGEF1B	4.46E-18	0.397412171	0.584	0.358	9.72E-14
FPR2	5.43E-18	0.386839604	0.286	0.091	1.19E-13
CLIC4	5.82E-18	0.357161952	0.351	0.139	1.27E-13
CTSC	6.30E-18	-0.38297198	0.838	0.919	1.37E-13
ELMSAN1	8.04E-18	0.451258108	0.353	0.148	1.75E-13
CITED2	8.47E-18	-0.602767613	0.462	0.673	1.85E-13
MALAT1	9.16E-18	0.273188824	0.985	0.988	2.00E-13
PHACTR1	1.03E-17	0.401680781	0.481	0.242	2.25E-13
DNAAF1	1.06E-17	0.319153493	0.134	0.007	2.31E-13
SH3BP5	1.11E-17	0.441152398	0.542	0.325	2.43E-13
TNFAIP6	1.16E-17	0.396036401	0.206	0.04	2.53E-13
PPARD	1.23E-17	0.271925934	0.227	0.053	2.68E-13
LIMD2	1.32E-17	-0.39375396	0.504	0.701	2.88E-13
ITGB2	1.32E-17	-0.353221849	0.765	0.867	2.89E-13
RALGDS	1.42E-17	0.364689093	0.269	0.083	3.09E-13
MT1X	1.98E-17	0.950202265	0.513	0.295	4.32E-13
APOBEC3A	2.01E-17	0.400076551	0.164	0.02	4.39E-13
PTPRC	2.23E-17	-0.342553263	0.798	0.891	4.87E-13
FEM1C	2.76E-17	0.339364177	0.292	0.098	6.03E-13
CD274	2.93E-17	0.258898809	0.147	0.013	6.39E-13
MIR4435-2HG	2.99E-17	0.424281053	0.38	0.166	6.53E-13
CCL18	3.31E-17	0.824805046	0.139	0.01	7.22E-13
RNF146	3.31E-17	-0.275519	0.145	0.386	7.22E-13
SQSTM1	3.37E-17	0.406717535	0.826	0.72	7.35E-13
MANF	3.85E-17	0.399325984	0.408	0.192	8.39E-13
OPTN	4.04E-17	0.264611851	0.145	0.013	8.82E-13
MORF4L2	4.38E-17	0.408825336	0.454	0.232	9.55E-13
IRAK2	5.09E-17	0.275968153	0.185	0.033	1.11E-12
ELOVL5	5.17E-17	0.402626403	0.454	0.244	1.13E-12
ABI3	6.04E-17	-0.397850329	0.275	0.509	1.32E-12
BTG3	6.37E-17	0.286639231	0.267	0.081	1.39E-12
NOTCH1	7.17E-17	0.280480829	0.17	0.025	1.56E-12
UBE2B	7.20E-17	0.45457524	0.708	0.566	1.57E-12
KIAA1551	8.31E-17	-0.30441701	0.09	0.3	1.81E-12
FCGR2A	8.54E-17	0.493253323	0.59	0.401	1.86E-12
SLC8B1	9.84E-17	0.378800472	0.414	0.201	2.14E-12
CLEC12A	9.88E-17	-0.384298021	0.273	0.514	2.15E-12
SLC2A6	1.17E-16	0.273233244	0.2	0.041	2.55E-12
MT-ND5	1.30E-16	-0.358905569	0.975	0.987	2.84E-12
SOCS1	1.35E-16	0.29025969	0.244	0.068	2.94E-12
SAV1	1.49E-16	0.343687974	0.235	0.065	3.24E-12
FABP5	1.52E-16	0.595891876	0.758	0.604	3.32E-12
SCAMP2	2.08E-16	-0.349739281	0.313	0.549	4.53E-12
NRP2	2.36E-16	0.360602284	0.261	0.083	5.15E-12
ABCA1	2.56E-16	0.381615367	0.769	0.599	5.59E-12
REEP5	2.78E-16	-0.352685809	0.45	0.673	6.07E-12
FAM49A	2.95E-16	0.409184936	0.603	0.41	6.44E-12
C10orf54	2.96E-16	-0.361237968	0.672	0.823	6.45E-12

ABRACL	3.03E-16	-0.377675921	0.58	0.761	6.61E-12
CMKLR1	3.10E-16	-0.29936917	0.097	0.307	6.77E-12
PHLDA1	4.06E-16	0.450115212	0.223	0.058	8.86E-12
BLVRB	4.18E-16	-0.309242979	0.855	0.924	9.11E-12
HIVEP1	4.81E-16	0.326677598	0.233	0.066	1.05E-11
RRAGC	5.25E-16	0.336980899	0.313	0.121	1.15E-11
TNFSF13	6.04E-16	-0.367011475	0.277	0.506	1.32E-11
IER2	6.47E-16	-0.49075415	0.836	0.892	1.41E-11
RGS19	6.64E-16	-0.339218337	0.271	0.504	1.45E-11
LRP1	7.48E-16	-0.358885953	0.336	0.567	1.63E-11
CD33	7.66E-16	-0.367758878	0.338	0.572	1.67E-11
PTGES3	8.37E-16	0.496999114	0.824	0.73	1.83E-11
FAM102B	9.83E-16	0.347388539	0.273	0.095	2.14E-11
HVCN1	1.09E-15	-0.342313407	0.181	0.405	2.38E-11
ARPC5	1.15E-15	-0.345925893	0.782	0.857	2.50E-11
GMFG	1.20E-15	-0.352760138	0.59	0.748	2.63E-11
DNAJC25	1.24E-15	0.25203004	0.189	0.04	2.70E-11
TUBA1C	1.29E-15	0.42825593	0.513	0.318	2.81E-11
WARS	1.98E-15	0.423641675	0.412	0.217	4.32E-11
CPM	2.18E-15	-0.410906909	0.689	0.804	4.76E-11
FRMD4B	2.36E-15	-0.382146588	0.357	0.584	5.14E-11
IRF2	2.40E-15	-0.352364223	0.216	0.436	5.23E-11
SERPINH1	2.50E-15	0.383244736	0.151	0.022	5.45E-11
TBXAS1	2.55E-15	-0.317721534	0.662	0.828	5.55E-11
GPCPD1	2.58E-15	0.450634566	0.578	0.413	5.62E-11
AQP9	3.06E-15	0.349229659	0.197	0.045	6.68E-11
SLC38A10	3.26E-15	-0.319996976	0.13	0.34	7.11E-11
MERTK	3.36E-15	-0.378354638	0.441	0.645	7.33E-11
SHFM1	3.43E-15	-0.367944132	0.468	0.687	7.49E-11
CSRNP1	3.89E-15	0.376444884	0.504	0.29	8.48E-11
BAZ1A	4.19E-15	0.458669997	0.605	0.441	9.14E-11
MBOAT7	5.26E-15	0.338644235	0.286	0.111	1.15E-10
SPIC	5.34E-15	-0.504702629	0.17	0.381	1.17E-10
GPX1	5.58E-15	0.299416983	0.952	0.925	1.22E-10
IL4R	6.02E-15	0.34032531	0.313	0.126	1.31E-10
ARL4C	8.49E-15	0.47710173	0.471	0.272	1.85E-10
RAB5A	9.50E-15	0.330375247	0.471	0.267	2.07E-10
EIF5	1.03E-14	0.356275988	0.758	0.63	2.25E-10
STX10	1.18E-14	-0.299202136	0.185	0.408	2.58E-10
SESN1	1.22E-14	-0.327017897	0.179	0.398	2.67E-10
RNF13	1.23E-14	-0.33390033	0.62	0.769	2.67E-10
CD68	1.60E-14	-0.250183924	0.975	0.96	3.50E-10
GSTK1	1.73E-14	-0.330907572	0.674	0.813	3.77E-10
ADGRE5	2.02E-14	0.480767852	0.632	0.484	4.40E-10
GPBP1	2.20E-14	0.382255499	0.618	0.453	4.79E-10
SYAP1	2.42E-14	0.418413034	0.502	0.305	5.27E-10
MS4A4E	2.50E-14	-0.36967788	0.162	0.363	5.45E-10
FLT1	2.55E-14	0.271238345	0.145	0.022	5.57E-10
MT1E	3.22E-14	0.893322603	0.296	0.124	7.03E-10
DUSP1	3.41E-14	-0.341852766	0.979	0.965	7.44E-10
TRAPPC1	4.14E-14	-0.374023237	0.357	0.566	9.03E-10
TMEM2	4.42E-14	0.340384616	0.412	0.209	9.63E-10
IRF5	4.97E-14	-0.282668887	0.118	0.317	1.08E-09
SNN	5.17E-14	0.313359127	0.315	0.138	1.13E-09
ZBTB43	5.58E-14	0.312560327	0.254	0.093	1.22E-09
GCNT1	5.94E-14	-0.253492164	0.084	0.265	1.29E-09
TRIP10	6.68E-14	0.261971548	0.166	0.035	1.46E-09
P2RY12	7.42E-14	-0.25321016	0.082	0.259	1.62E-09
GBP2	7.52E-14	0.404312902	0.494	0.303	1.64E-09
MYO1G	8.77E-14	0.323898159	0.351	0.166	1.91E-09
HYOU1	9.00E-14	0.275818938	0.229	0.075	1.96E-09
FAM105A	9.02E-14	-0.262005338	0.13	0.33	1.97E-09
SCP2	9.32E-14	-0.343984866	0.401	0.605	2.03E-09
ADRB2	9.93E-14	-0.257863426	0.082	0.257	2.17E-09
MAPK6	9.98E-14	0.324715981	0.332	0.154	2.18E-09
ORMDL1	1.04E-13	-0.341802946	0.351	0.559	2.26E-09
MMP19	1.06E-13	0.715832327	0.45	0.262	2.32E-09
C16orf72	1.18E-13	0.307113053	0.355	0.171	2.58E-09
RCSD1	1.21E-13	-0.323019016	0.317	0.544	2.65E-09
WSB1	1.28E-13	-0.378108368	0.832	0.904	2.79E-09

LAP3	1.33E-13	0.3461194	0.637	0.494	2.91E-09
MIS18BP1	1.36E-13	-0.320465369	0.204	0.415	2.97E-09
IFI30	1.47E-13	0.361883643	0.756	0.645	3.20E-09
ARPC3	1.50E-13	-0.267472802	0.943	0.959	3.27E-09
YPEL2	1.59E-13	-0.267328997	0.111	0.302	3.48E-09
PSMB10	1.60E-13	-0.28270593	0.292	0.511	3.49E-09
FAM177A1	1.61E-13	0.35090699	0.435	0.25	3.52E-09
FNIP2	1.62E-13	0.42553048	0.473	0.302	3.52E-09
ACP2	1.65E-13	-0.279159411	0.252	0.481	3.60E-09
AKR1A1	1.72E-13	-0.334537841	0.414	0.612	3.74E-09
BACH1	1.82E-13	0.356816582	0.489	0.3	3.96E-09
KLF4	1.82E-13	0.316916426	0.866	0.746	3.97E-09
FRMD4A	2.17E-13	-0.320700831	0.126	0.313	4.73E-09
GADD45B	2.19E-13	0.339025725	0.964	0.891	4.78E-09
POLD4	2.28E-13	-0.32578794	0.359	0.561	4.98E-09
ISG20	2.33E-13	0.373836476	0.313	0.141	5.09E-09
SLCO2B1	2.52E-13	-0.333643143	0.311	0.526	5.50E-09
TMEM70	2.55E-13	0.376226287	0.441	0.27	5.56E-09
PYCARD	2.79E-13	-0.306584957	0.721	0.816	6.08E-09
STX11	2.90E-13	0.328852838	0.603	0.423	6.33E-09
PPT1	3.04E-13	-0.366346196	0.59	0.738	6.62E-09
GBP1	3.31E-13	0.326321761	0.269	0.108	7.21E-09
TSPAN4	3.58E-13	-0.322889426	0.571	0.73	7.81E-09
METTL7A	3.83E-13	-0.345688159	0.372	0.58	8.35E-09
PPTC7	3.87E-13	0.262119549	0.216	0.07	8.45E-09
NDUFA12	4.71E-13	-0.271195237	0.307	0.526	1.03E-08
PTX3	4.97E-13	0.467679187	0.16	0.035	1.08E-08
MAP1LC3B	5.61E-13	0.341596137	0.739	0.597	1.22E-08
IL10RA	5.68E-13	-0.315222567	0.473	0.667	1.24E-08
PTPN6	5.80E-13	-0.311754467	0.471	0.66	1.26E-08
C3AR1	5.84E-13	-0.322318195	0.368	0.577	1.27E-08
SLC3A2	6.10E-13	0.36798881	0.674	0.531	1.33E-08
ACBD3	6.87E-13	0.255559547	0.223	0.075	1.50E-08
TPM4	7.25E-13	0.377438911	0.525	0.355	1.58E-08
EVI2B	7.46E-13	-0.336608162	0.683	0.809	1.63E-08
RANGAP1	7.86E-13	0.275017315	0.197	0.058	1.71E-08
AGO2	8.22E-13	0.302776925	0.256	0.1	1.79E-08
ATG16L2	8.46E-13	-0.381737657	0.33	0.536	1.85E-08
VIMP	9.23E-13	0.378256668	0.534	0.367	2.01E-08
GIMAP2	1.02E-12	-0.287525712	0.195	0.395	2.22E-08
DUSP6	1.02E-12	-0.411856893	0.42	0.622	2.22E-08
GNPDA1	1.03E-12	-0.301299809	0.248	0.466	2.25E-08
ZFP36	1.12E-12	0.29834875	0.966	0.932	2.44E-08
RIPK2	1.12E-12	0.35115741	0.41	0.234	2.44E-08
IFT57	1.24E-12	0.252375184	0.17	0.043	2.70E-08
EPN1	1.24E-12	-0.320479536	0.361	0.551	2.71E-08
SRP9	1.53E-12	-0.322975159	0.34	0.529	3.34E-08
RAPGEF2	1.56E-12	0.286820084	0.284	0.124	3.39E-08
HSPA4	1.65E-12	0.426836266	0.441	0.282	3.59E-08
COX6C	1.76E-12	-0.266155548	0.794	0.887	3.83E-08
BTF3L4	1.85E-12	-0.28906821	0.357	0.57	4.04E-08
CORO1A	1.93E-12	-0.349462286	0.534	0.697	4.21E-08
GIMAP5	1.98E-12	-0.260482536	0.16	0.357	4.32E-08
LSM7	2.91E-12	-0.294113366	0.342	0.539	6.35E-08
ARFGAP3	2.92E-12	0.278238086	0.34	0.171	6.37E-08
TCF7L2	3.01E-12	-0.382972007	0.284	0.479	6.56E-08
NAA20	3.04E-12	-0.325352251	0.206	0.396	6.63E-08
NDUFA2	3.93E-12	-0.334134467	0.412	0.612	8.56E-08
HLA-DMB	4.09E-12	-0.301844453	0.496	0.68	8.91E-08
HSD17B7	4.12E-12	0.307332831	0.246	0.098	8.99E-08
ANKRD28	5.18E-12	0.311342306	0.231	0.085	1.13E-07
USP36	5.84E-12	0.329717727	0.282	0.126	1.27E-07
NAIP	5.86E-12	-0.26607199	0.658	0.791	1.28E-07
PER1	6.12E-12	0.285242399	0.529	0.342	1.33E-07
COX5A	6.90E-12	-0.261562662	0.653	0.768	1.50E-07
LBR	7.21E-12	-0.323455462	0.204	0.391	1.57E-07
SLC5A3	7.38E-12	0.309119146	0.231	0.083	1.61E-07
TICAM1	8.63E-12	0.281982469	0.244	0.1	1.88E-07
IER5	9.33E-12	0.465463782	0.511	0.355	2.03E-07
FCHO2	1.15E-11	-0.305080638	0.286	0.473	2.52E-07

KMO	1.21E-11	0.381976473	0.351	0.184	2.64E-07
NSL1	1.23E-11	-0.28628943	0.193	0.388	2.68E-07
CD84	1.44E-11	-0.392965528	0.376	0.552	3.15E-07
RAPGEF1	1.46E-11	0.3039044	0.271	0.119	3.18E-07
RNF145	1.65E-11	0.296637054	0.422	0.25	3.60E-07
SPAG9	1.76E-11	0.327827562	0.435	0.265	3.83E-07
SLC46A1	1.92E-11	-0.278711446	0.132	0.303	4.20E-07
MT-ND4L	2.10E-11	-0.267129953	0.992	0.993	4.57E-07
LST1	2.20E-11	-0.280896476	0.889	0.942	4.80E-07
SAR1A	2.24E-11	0.283757034	0.328	0.163	4.89E-07
TNFRSF1B	2.26E-11	0.356760735	0.708	0.61	4.93E-07
MNDA	2.28E-11	-0.379799333	0.431	0.594	4.98E-07
JKAMP	2.45E-11	-0.287822208	0.172	0.353	5.35E-07
PNPLA8	2.48E-11	0.350747431	0.479	0.31	5.41E-07
NDEL1	2.63E-11	0.266164642	0.225	0.086	5.74E-07
GNA15	2.71E-11	0.341554952	0.321	0.171	5.91E-07
GUK1	2.75E-11	0.374468953	0.689	0.587	5.99E-07
CD300E	2.75E-11	0.274257652	0.185	0.058	6.00E-07
SESTD1	2.85E-11	0.318411623	0.317	0.161	6.22E-07
TYMP	2.86E-11	0.353267314	0.67	0.527	6.24E-07
TRIB1	2.89E-11	0.317362248	0.479	0.303	6.31E-07
PSAP	2.97E-11	-0.259618453	0.964	0.944	6.48E-07
DDIT4	3.03E-11	-0.381508422	0.613	0.766	6.60E-07
GFRA2	3.30E-11	-0.356916777	0.235	0.405	7.20E-07
TNFRSF21	3.87E-11	0.344215723	0.353	0.191	8.43E-07
AC058791.1	4.12E-11	0.251082048	0.174	0.055	8.98E-07
GRASP	4.22E-11	0.28774986	0.3	0.141	9.19E-07
GABARAPL1	4.45E-11	0.299204737	0.414	0.24	9.71E-07
NUDT16	4.70E-11	-0.284270766	0.46	0.633	1.02E-06
CD69	4.87E-11	0.320763814	0.143	0.033	1.06E-06
ZNF106	5.32E-11	-0.329607431	0.239	0.425	1.16E-06
CCL20	5.43E-11	0.903706601	0.124	0.023	1.18E-06
SDCBP	5.46E-11	0.270005651	0.914	0.877	1.19E-06
CTD-3252C9.4	5.78E-11	0.329846559	0.275	0.128	1.26E-06
PPP1R21	6.21E-11	-0.264643672	0.095	0.247	1.35E-06
EMD	6.57E-11	0.345802852	0.464	0.302	1.43E-06
NEDD8	6.91E-11	-0.284699457	0.454	0.635	1.51E-06
GADD45A	7.35E-11	0.311157909	0.216	0.083	1.60E-06
SPTLC2	7.45E-11	-0.264110847	0.168	0.348	1.63E-06
CD302	8.09E-11	-0.250536787	0.853	0.924	1.76E-06
ELF1	1.02E-10	0.380382032	0.637	0.504	2.23E-06
NAGK	1.04E-10	-0.279608493	0.334	0.529	2.27E-06
PRDX1	1.08E-10	-0.277881735	0.723	0.837	2.36E-06
ATP1B1	1.13E-10	-0.360874584	0.357	0.532	2.46E-06
MEF2C	1.13E-10	-0.303008004	0.382	0.582	2.47E-06
MPHOSPH6	1.17E-10	0.305023837	0.279	0.134	2.54E-06
SLC16A6	1.27E-10	0.326850777	0.204	0.076	2.77E-06
ADAP2	1.30E-10	-0.339506713	0.456	0.629	2.83E-06
LAMTOR4	1.32E-10	-0.262288956	0.695	0.791	2.88E-06
UBAP1	1.40E-10	0.252984418	0.334	0.177	3.06E-06
GLRX	1.44E-10	-0.294720395	0.487	0.662	3.13E-06
LIMS1	1.53E-10	0.321523395	0.708	0.587	3.33E-06
FAM96A	1.59E-10	-0.270243009	0.5	0.677	3.47E-06
C4orf3	1.69E-10	-0.268431799	0.489	0.653	3.68E-06
UBB	1.74E-10	0.456623391	0.868	0.813	3.80E-06
CHORDC1	1.85E-10	0.317186281	0.368	0.214	4.03E-06
CSK	1.94E-10	-0.288670097	0.254	0.439	4.23E-06
NANS	2.14E-10	0.322414802	0.563	0.436	4.66E-06
DOCK8	2.16E-10	-0.296999756	0.265	0.434	4.72E-06
ST3GAL1	2.46E-10	0.265242702	0.382	0.221	5.36E-06
ZNF267	2.46E-10	0.319516052	0.401	0.252	5.37E-06
RAB21	2.47E-10	0.252291887	0.309	0.154	5.39E-06
GCLC	2.62E-10	-0.259707977	0.164	0.333	5.70E-06
FCGRT	2.66E-10	-0.275613811	0.914	0.929	5.81E-06
TCEB1	2.69E-10	0.354943253	0.607	0.483	5.86E-06
STK38L	3.01E-10	0.31592583	0.305	0.161	6.56E-06
MRPS34	3.22E-10	-0.289892433	0.235	0.418	7.02E-06
OAS1	3.27E-10	-0.253383508	0.162	0.328	7.14E-06
EGR2	3.32E-10	-0.435683629	0.097	0.24	7.25E-06
GLMP	3.33E-10	-0.286855774	0.324	0.502	7.27E-06

EAF2	3.35E-10	-0.274175207	0.202	0.376	7.30E-06
7-Sep	3.62E-10	-0.274138306	0.361	0.546	7.90E-06
PRELID1	3.76E-10	0.291274943	0.748	0.648	8.19E-06
SMIM3	4.30E-10	0.272072784	0.296	0.154	9.37E-06
SGK1	4.67E-10	0.355170121	0.794	0.677	1.02E-05
BCL2L11	4.80E-10	0.262970261	0.271	0.126	1.05E-05
STK17B	4.93E-10	-0.272907384	0.357	0.546	1.08E-05
NUCB1	5.14E-10	0.31974969	0.721	0.607	1.12E-05
FLOT1	5.15E-10	0.273432969	0.374	0.222	1.12E-05
TMEM59	5.16E-10	-0.258303251	0.744	0.804	1.12E-05
RSRP1	5.20E-10	-0.332578358	0.454	0.629	1.13E-05
ATPIF1	5.36E-10	-0.282887389	0.34	0.526	1.17E-05
PIGA	5.94E-10	0.28829246	0.237	0.104	1.29E-05
PDCD6	7.47E-10	-0.263441644	0.237	0.421	1.63E-05
TIPARP	7.48E-10	0.293603225	0.345	0.189	1.63E-05
SPI1	7.66E-10	-0.258364687	0.773	0.834	1.67E-05
RAB1A	8.31E-10	0.317328634	0.576	0.473	1.81E-05
UBC	9.01E-10	0.301982578	0.975	0.922	1.96E-05
ATP5J2	9.04E-10	-0.26358005	0.651	0.791	1.97E-05
NAAA	1.01E-09	-0.296376484	0.418	0.584	2.19E-05
MLKL	1.31E-09	0.290975161	0.246	0.113	2.86E-05
SFT2D2	1.33E-09	-0.267165566	0.212	0.388	2.91E-05
MT-CYB	1.35E-09	-0.251094904	0.992	0.992	2.93E-05
SLC9A8	1.49E-09	0.266870577	0.2	0.078	3.25E-05
DYNLL1	1.53E-09	-0.288470972	0.653	0.774	3.35E-05
SDC3	1.55E-09	-0.314362734	0.328	0.507	3.37E-05
PDXK	1.58E-09	0.253596319	0.252	0.118	3.44E-05
NDUFA4	1.70E-09	-0.269577778	0.71	0.791	3.71E-05
SNAP23	1.90E-09	0.332880223	0.578	0.451	4.14E-05
BTG1	1.91E-09	0.26824645	0.859	0.786	4.16E-05
GLIPR2	1.91E-09	0.271663824	0.319	0.177	4.17E-05
SRSF3	1.96E-09	0.252266581	0.712	0.587	4.27E-05
LINC01272	2.03E-09	-0.357186683	0.649	0.746	4.43E-05
ARL6IP5	2.16E-09	-0.271954849	0.66	0.788	4.71E-05
CRIP1	2.33E-09	0.396659148	0.525	0.386	5.08E-05
AOAH	2.39E-09	-0.307181259	0.429	0.579	5.22E-05
TANK	2.49E-09	0.271374447	0.473	0.318	5.44E-05
ZFAND5	2.56E-09	0.330690095	0.811	0.71	5.58E-05
FAM20A	2.56E-09	0.27353889	0.424	0.277	5.58E-05
PIAS1	2.57E-09	-0.254859356	0.277	0.458	5.61E-05
NCKAP1L	2.63E-09	-0.313601044	0.332	0.498	5.74E-05
ITGAX	2.65E-09	0.303231279	0.389	0.237	5.78E-05
IL6ST	2.65E-09	0.316546877	0.416	0.265	5.78E-05
DEGS1	2.67E-09	-0.267813567	0.254	0.433	5.82E-05
YTHDF3	2.77E-09	0.258542501	0.431	0.277	6.03E-05
PSTPIP2	3.00E-09	0.297505865	0.353	0.206	6.54E-05
NABP1	3.00E-09	0.288592145	0.708	0.615	6.54E-05
IRF7	3.37E-09	0.304824397	0.42	0.277	7.35E-05
ADRBK1	3.51E-09	-0.277797121	0.242	0.42	7.66E-05
ATP6V0D1	3.69E-09	0.280240983	0.672	0.575	8.04E-05
IL1R2	4.22E-09	0.433264081	0.363	0.224	9.20E-05
DPYD	4.26E-09	-0.255380143	0.433	0.604	9.30E-05
CFLAR	4.55E-09	0.265454442	0.634	0.489	9.92E-05
SNX3	4.79E-09	0.258579694	0.851	0.776	0.000104364
NDUFA1	5.80E-09	-0.265100534	0.666	0.784	0.000126441
GYPC	5.80E-09	0.255232188	0.63	0.488	0.000126589
DDX3Y	5.99E-09	0.263360535	0.502	0.352	0.000130564
SLC9A9	6.29E-09	-0.281280524	0.137	0.279	0.00013707
SEPP1	7.31E-09	-0.412805319	0.718	0.808	0.000159395
AFF4	7.36E-09	0.257763117	0.477	0.335	0.000160522
RBP7	7.47E-09	-0.319223153	0.67	0.774	0.000162841
FCGR1A	7.71E-09	0.335793851	0.567	0.444	0.000168078
ERGIC1	7.84E-09	0.282015375	0.328	0.184	0.000170978
CD14	7.88E-09	-0.251253736	0.811	0.879	0.000171935
CD72	8.50E-09	0.286533776	0.475	0.33	0.000185427
IVNS1ABP	8.54E-09	0.360537835	0.626	0.532	0.000186251
NPEPPS	9.30E-09	-0.254873972	0.151	0.297	0.000202747
RGL1	9.56E-09	-0.298193839	0.342	0.499	0.000208552
EVL	9.71E-09	-0.250018399	0.334	0.501	0.000211795
UBE2J1	9.78E-09	0.285814724	0.622	0.522	0.000213307

TUBB4B	1.03E-08	0.297300908	0.563	0.433	0.000224431
DAD1	1.15E-08	-0.267586864	0.542	0.668	0.000251781
RILPL2	1.23E-08	0.289985385	0.605	0.498	0.000267677
GCH1	1.24E-08	0.279563899	0.321	0.187	0.000270259
UBE2F	1.28E-08	0.27353974	0.355	0.221	0.000279476
ERN1	1.31E-08	0.258376188	0.233	0.113	0.000285387
LILRA2	1.33E-08	-0.282338067	0.416	0.557	0.000289008
OCIAD1	1.43E-08	-0.253762298	0.37	0.526	0.000312331
EIF2AK4	1.49E-08	0.261641896	0.357	0.216	0.000325217
COX7B	1.56E-08	-0.282755706	0.674	0.773	0.000339551
MPEG1	1.64E-08	-0.282209413	0.59	0.718	0.000358477
RASSF4	1.65E-08	-0.304381983	0.504	0.663	0.000360491
C11orf31	1.78E-08	-0.268460735	0.424	0.574	0.000388994
ATP1A1	1.85E-08	0.283525453	0.565	0.433	0.000403615
WWP1	1.86E-08	-0.280939751	0.244	0.4	0.000404793
NPL	2.05E-08	-0.256043321	0.487	0.622	0.000447975
LAMTOR1	2.18E-08	-0.269868077	0.498	0.629	0.000474624
IL10	2.48E-08	0.589301379	0.328	0.196	0.000541004
LINC00152	2.78E-08	0.326976684	0.334	0.199	0.000606977
PTPRE	2.89E-08	0.309769877	0.54	0.423	0.000630619
CD164	3.04E-08	-0.252313917	0.718	0.799	0.000662615
ID2	3.11E-08	-0.316981421	0.672	0.763	0.000678041
BASP1	3.19E-08	0.285087218	0.475	0.343	0.000694695
TNFSF13B	3.19E-08	0.365567167	0.485	0.368	0.000695631
C20orf27	3.49E-08	-0.256932566	0.248	0.403	0.00076145
TNF	3.55E-08	-0.543300595	0.221	0.368	0.000773745
DPEP2	3.58E-08	-0.255037114	0.252	0.405	0.000781733
MT-ND1	3.68E-08	-0.254867279	0.983	0.985	0.000802383
SOD1	3.99E-08	0.364263131	0.662	0.582	0.000870684
ANXA2	4.02E-08	0.267408476	0.704	0.607	0.000876918
SNX6	4.04E-08	-0.250353154	0.645	0.753	0.000881848
CFD	4.07E-08	-0.28738182	0.75	0.829	0.000888416
TREM1	4.26E-08	0.250195414	0.275	0.148	0.000929925
ALB	4.38E-08	0.258312238	0.42	0.265	0.000954305
ZCCHC6	4.50E-08	-0.295965734	0.393	0.539	0.000981501
GBP5	4.58E-08	0.250081411	0.164	0.063	0.00099809
TGOLN2	4.73E-08	-0.252600934	0.319	0.479	0.001031944
TCIRG1	4.93E-08	-0.272832964	0.534	0.657	0.001075366
ELOVL1	6.16E-08	-0.250364835	0.29	0.439	0.001342967
HNMT	6.41E-08	-0.260733379	0.521	0.653	0.001397568
CEBPB	6.67E-08	0.257552267	0.75	0.662	0.001455339
CSF2RB	7.14E-08	0.264076296	0.233	0.119	0.001556008
GOLPH3	7.28E-08	0.268444002	0.437	0.32	0.001587527
MBNL1	7.46E-08	-0.269339676	0.538	0.677	0.001627963
DNASE2	7.75E-08	-0.266424575	0.395	0.542	0.001690799
AKIRIN2	7.93E-08	-0.262200779	0.515	0.645	0.001729198
NRP1	8.24E-08	-0.262023026	0.275	0.431	0.001796612
CYCS	9.09E-08	0.453676051	0.626	0.541	0.0019818
LILRA5	1.03E-07	0.319979054	0.523	0.403	0.002241733
MAT2A	1.13E-07	0.300172654	0.634	0.524	0.00246623
AP000769.1	1.14E-07	0.333866293	0.311	0.192	0.002490787
JUNB	1.20E-07	-0.273026641	0.931	0.937	0.002626078
TUBA1A	1.39E-07	0.33674038	0.422	0.294	0.003030241
MYO1F	1.45E-07	-0.263206363	0.443	0.589	0.003171697
APP	1.84E-07	0.294230992	0.29	0.171	0.004002756
TMIGD3	2.11E-07	-0.442788357	0.382	0.488	0.004608676
SAA1	2.29E-07	0.382474073	0.244	0.129	0.004999353
CCL3L3	2.66E-07	-0.63320467	0.34	0.461	0.005805131
PRDM1	2.75E-07	0.251731971	0.233	0.123	0.005987764
ACSL3	2.88E-07	0.328454438	0.399	0.289	0.006290737
PRNP	3.07E-07	0.304248739	0.723	0.637	0.006691054
MARCKS	3.11E-07	0.29002367	0.691	0.62	0.006790211
LGALS1	3.28E-07	0.253204682	0.84	0.765	0.007145008
ILF3-AS1	3.45E-07	-0.255227828	0.084	0.196	0.007533817
ABCC5	4.00E-07	-0.264822738	0.229	0.368	0.008712862
S100A10	4.04E-07	0.351210217	0.803	0.731	0.008821007
MAF	5.17E-07	-0.260770782	0.529	0.662	0.011274186
CDC37	5.23E-07	0.302799065	0.63	0.547	0.011395912
EML4	5.83E-07	0.284890827	0.374	0.247	0.012717345
SEC62	5.85E-07	0.250989824	0.697	0.602	0.012766575

SEPHS2	6.64E-07	0.292457567	0.368	0.264	0.014483942
CTNNB1	6.67E-07	0.285782164	0.433	0.327	0.01455341
ETF1	7.04E-07	0.253925033	0.433	0.322	0.015360219
MGST2	7.22E-07	-0.25900879	0.33	0.471	0.015746418
ATP6V1C1	7.99E-07	0.268324177	0.38	0.267	0.017432419
ARL8B	8.61E-07	0.253581072	0.563	0.446	0.018769444
WDR45B	1.15E-06	0.31617893	0.298	0.192	0.024983677
CTSA	1.48E-06	-0.260421896	0.641	0.725	0.032178358
CYTIP	1.54E-06	-0.263923578	0.332	0.453	0.033678974
MT1M	1.84E-06	0.640547592	0.143	0.058	0.04020151
ATF7IP2	1.89E-06	0.406975724	0.204	0.108	0.041158925
DNAJB4	2.22E-06	0.287236896	0.321	0.207	0.048467573

List of differentially expressed genes of VCAN_TMo in overall stage (PR versus PP)

gene	p_val	avg_logFC	pct.1	pct.2	p_val_adj
HLA-DRB5	1.22E-74	-1.341245149	0.401	0.947	2.66E-70
THBS1	5.71E-69	1.404183804	0.962	0.786	1.25E-64
NAMPT	9.17E-68	1.014836809	0.993	0.866	2.00E-63
TMSB4X	2.48E-58	-0.438032705	1	1	5.41E-54
ACSL1	1.29E-53	0.989413486	0.89	0.629	2.81E-49
DEFA3	5.89E-52	-0.805940579	0.017	0.487	1.28E-47
EEF1A1	4.11E-51	-0.400257477	0.998	1	8.97E-47
VCAN	4.13E-50	0.82932295	0.983	0.944	9.00E-46
CST3	7.83E-50	-0.575722624	0.998	1	1.71E-45
GPX1	9.86E-48	0.692557525	0.957	0.923	2.15E-43
HLA-DQB1	1.46E-45	-0.888432165	0.332	0.831	3.18E-41
H3F3B	1.48E-40	0.532472299	0.995	0.979	3.24E-36
ASPH	2.97E-40	0.948265473	0.649	0.228	6.49E-36
TRMT6	1.57E-39	0.995298514	0.558	0.131	3.43E-35
NACA	1.89E-39	-0.396664373	0.993	0.997	4.12E-35
S100A4	1.10E-38	-0.437069698	1	1	2.40E-34
SAT1	1.47E-38	0.528563099	0.998	0.994	3.20E-34
LITAF	1.49E-38	0.895367817	0.842	0.576	3.25E-34
FPR1	7.94E-38	0.755792067	0.9	0.742	1.73E-33
EVI2B	1.05E-37	-0.672751859	0.556	0.905	2.29E-33
TPM4	6.21E-37	0.754954295	0.864	0.659	1.35E-32
RPL11	9.68E-37	-0.309780997	1	0.997	2.11E-32
RPL5	2.36E-36	-0.413935873	0.964	0.994	5.15E-32
SLC2A3	2.60E-36	0.919358291	0.833	0.588	5.67E-32
RPL29	5.18E-35	-0.326038368	0.988	0.994	1.13E-30
RPL10	8.51E-35	-0.296923172	1	1	1.86E-30
RPS29	3.64E-34	0.514029067	0.952	0.92	7.95E-30
TNFSF10	4.19E-34	-0.602884575	0.045	0.415	9.13E-30
CTSL	5.85E-34	1.642462181	0.642	0.306	1.28E-29
PABPC1	7.23E-34	-0.445269196	0.931	0.985	1.58E-29
UPP1	1.95E-33	0.730670685	0.847	0.605	4.26E-29
SLC25A6	4.03E-33	-0.491063578	0.881	0.97	8.79E-29
RPL18	4.57E-32	-0.320428326	0.995	0.991	9.97E-28
AGFG1	9.45E-32	0.873945364	0.663	0.344	2.06E-27
VSIG4	2.73E-31	-0.822848692	0.1	0.469	5.95E-27
S100A9	5.05E-31	0.74926727	0.993	0.982	1.10E-26
S100A12	8.86E-31	0.876640734	0.938	0.843	1.93E-26
AQP9	1.55E-30	0.779241334	0.702	0.359	3.37E-26
VAMP8	1.63E-30	-0.576037427	0.613	0.908	3.55E-26
PLSCR1	2.38E-30	0.739896614	0.754	0.496	5.19E-26
RNASE6	4.81E-30	-0.622591279	0.368	0.783	1.05E-25
RPL7A	5.97E-30	-0.30241318	0.986	0.994	1.30E-25
RPL30	6.11E-30	-0.26929012	0.998	0.997	1.33E-25
BLVRB	6.56E-30	-0.584915588	0.63	0.911	1.43E-25
RPS3A	2.12E-29	-0.272885954	0.995	0.997	4.63E-25
S100A8	3.02E-29	0.654277195	0.998	0.994	6.59E-25
SRGN	6.51E-29	0.385107163	1	0.997	1.42E-24
TKT	6.83E-29	-0.544048562	0.823	0.944	1.49E-24
RPS14	8.01E-29	-0.267087239	0.993	1	1.75E-24
RPL6	9.03E-29	-0.32319182	0.986	0.994	1.97E-24
SULT1A1	2.35E-28	-0.589277829	0.208	0.608	5.13E-24
FPR2	6.21E-28	0.861722553	0.582	0.261	1.35E-23

ACTB	6.77E-28	0.267754367	1	1	1.48E-23
GNB2L1	9.38E-28	-0.345188962	0.974	0.985	2.04E-23
HBB	1.51E-27	-1.755314906	0.086	0.43	3.29E-23
SELL	1.75E-27	0.935285802	0.69	0.442	3.82E-23
CSF1R	1.91E-27	-0.566643388	0.126	0.493	4.17E-23
HIF1A	1.94E-27	0.781032434	0.723	0.478	4.22E-23
LIMD2	3.17E-27	-0.560098539	0.372	0.757	6.91E-23
IFI30	3.62E-27	0.643987986	0.854	0.757	7.90E-23
RPL14	4.84E-27	-0.324304399	0.971	1	1.06E-22
RPS7	8.64E-27	-0.279139646	0.998	0.991	1.88E-22
PLD3	1.34E-26	-0.57273893	0.301	0.68	2.93E-22
RBKS	1.74E-26	-0.654625604	0.181	0.552	3.79E-22
IL1R2	2.19E-26	1.1487653	0.599	0.285	4.77E-22
SEC11A	2.60E-26	-0.495118226	0.644	0.935	5.66E-22
CD74	3.15E-26	-0.438018491	0.988	1	6.88E-22
C20orf27	3.44E-26	-0.525481592	0.181	0.567	7.50E-22
GIMAP1	2.78E-25	-0.478100783	0.076	0.395	6.05E-21
TIMP1	4.64E-25	0.769397064	0.979	0.955	1.01E-20
PAG1	1.37E-24	0.746280079	0.601	0.306	2.99E-20
RPLP0	1.61E-24	-0.348192373	0.959	0.997	3.52E-20
SLC39A8	3.38E-24	0.908768023	0.437	0.119	7.36E-20
FCER1G	5.01E-24	0.379518704	1	0.994	1.09E-19
TPM3	5.35E-24	0.458054877	0.921	0.893	1.17E-19
CD63	8.26E-24	0.530217808	0.916	0.858	1.80E-19
PIM3	1.33E-23	0.664739748	0.535	0.217	2.90E-19
AREG	1.69E-23	0.845916132	0.871	0.62	3.69E-19
CD93	2.62E-23	0.728416961	0.64	0.38	5.72E-19
MIS18BP1	5.71E-23	-0.435809102	0.084	0.392	1.24E-18
FCGR3A	7.76E-23	-0.650427976	0.107	0.415	1.69E-18
PIM1	1.15E-22	0.704933089	0.396	0.107	2.50E-18
HNMT	4.46E-22	-0.4679704	0.239	0.611	9.72E-18
METTL7A	5.59E-22	-0.463350283	0.153	0.496	1.22E-17
TYMP	6.10E-22	0.573492934	0.854	0.751	1.33E-17
MS4A7	6.85E-22	-0.565557254	0.305	0.677	1.49E-17
CD300E	1.03E-21	0.791875715	0.628	0.38	2.24E-17
MARCKS	1.03E-21	0.80351017	0.632	0.368	2.25E-17
GCA	1.26E-21	0.593105549	0.869	0.81	2.75E-17
MCTP2	1.46E-21	0.561991429	0.308	0.042	3.18E-17
GIMAP7	1.52E-21	-0.528451712	0.048	0.306	3.32E-17
MS4A6A	1.62E-21	-0.486497771	0.745	0.938	3.53E-17
IFITM2	2.44E-21	0.607287044	0.936	0.947	5.32E-17
RAB31	2.52E-21	0.631109544	0.783	0.608	5.48E-17
GIMAP2	2.72E-21	-0.416903908	0.053	0.318	5.93E-17
MNDA	3.19E-21	-0.496526382	0.742	0.938	6.95E-17
RPS4X	4.82E-21	-0.26479748	0.99	0.997	1.05E-16
HVCN1	4.85E-21	-0.414996347	0.043	0.294	1.06E-16
PSTPIP1	4.97E-21	-0.464462887	0.184	0.519	1.08E-16
BIN2	7.36E-21	-0.435503656	0.165	0.504	1.61E-16
PYCARD	7.54E-21	-0.445354974	0.659	0.902	1.65E-16
HSD17B11	8.89E-21	-0.482883772	0.518	0.81	1.94E-16
RUNX1	9.19E-21	0.606287952	0.578	0.303	2.00E-16
MAP3K8	9.37E-21	0.683638245	0.733	0.525	2.04E-16
ETS2	1.03E-20	0.593161447	0.759	0.632	2.25E-16
ADAMTS2	1.47E-20	-0.473438765	0.103	0.398	3.21E-16
MCEMP1	1.73E-20	0.714910859	0.661	0.439	3.76E-16
RCSD1	2.29E-20	-0.432419291	0.136	0.454	5.00E-16
LRG1	2.52E-20	0.635080921	0.315	0.053	5.51E-16
ATP5G2	2.97E-20	-0.330929784	0.94	0.985	6.48E-16
PFKFB3	3.30E-20	0.663527286	0.461	0.184	7.20E-16
STX10	3.84E-20	-0.395813608	0.232	0.596	8.37E-16
NUP214	4.54E-20	-0.498242459	0.265	0.608	9.90E-16
HLA-DPA1	8.43E-20	-0.601378329	0.575	0.861	1.84E-15
FXYD5	1.13E-19	-0.374461475	0.866	0.97	2.45E-15
FLT3	1.37E-19	-0.353315018	0.079	0.356	2.98E-15
JUNB	1.51E-19	-0.376573371	0.962	0.979	3.29E-15
SAMSN1	1.78E-19	0.533983725	0.878	0.813	3.89E-15
SLC25A37	1.87E-19	0.586656471	0.609	0.362	4.07E-15
RNASE2	2.29E-19	-0.51865364	0.282	0.623	5.00E-15
LDHB	6.84E-19	-0.476129093	0.332	0.665	1.49E-14
FAM65B	9.64E-19	0.617948065	0.685	0.481	2.10E-14

SERPINB1	1.22E-18	0.475967061	0.926	0.902	2.66E-14
HLA-DPB1	1.45E-18	-0.53409505	0.516	0.801	3.17E-14
RBM47	1.55E-18	0.589164308	0.666	0.436	3.38E-14
DOK2	1.60E-18	-0.377060393	0.368	0.718	3.49E-14
EEF2	2.15E-18	-0.383158941	0.847	0.95	4.70E-14
MAFB	2.27E-18	0.607548346	0.857	0.804	4.96E-14
JARID2	3.08E-18	0.65509889	0.461	0.214	6.71E-14
IL18	5.72E-18	-0.384748368	0.126	0.415	1.25E-13
MT-ND3	6.34E-18	0.262101737	1	0.997	1.38E-13
CFL1	8.26E-18	0.268113825	0.99	0.988	1.80E-13
MYL6	8.88E-18	0.298708831	0.99	1	1.94E-13
B4GALT5	1.13E-17	0.587174869	0.372	0.122	2.45E-13
RBMS1	1.42E-17	0.587570045	0.606	0.395	3.09E-13
CD37	2.22E-17	-0.382636444	0.692	0.917	4.85E-13
HP	2.42E-17	0.535153807	0.241	0.024	5.28E-13
GNA15	4.80E-17	0.572533599	0.391	0.145	1.05E-12
FGL2	6.99E-17	-0.433595444	0.425	0.742	1.53E-12
P2RY13	9.52E-17	-0.356626701	0.036	0.24	2.08E-12
LILRA5	1.27E-16	0.561691765	0.766	0.653	2.77E-12
NR4A1	1.34E-16	-0.572029129	0.406	0.677	2.93E-12
TOP1	1.55E-16	0.616957399	0.592	0.389	3.37E-12
DNASE2	1.55E-16	-0.330159309	0.06	0.288	3.38E-12
CD1D	1.67E-16	-0.335602186	0.129	0.407	3.64E-12
GLRX	1.68E-16	-0.438602278	0.449	0.76	3.67E-12
SLC25A5	2.20E-16	-0.403134777	0.656	0.861	4.81E-12
CRTAP	2.47E-16	-0.41957517	0.482	0.757	5.38E-12
EIF3E	2.70E-16	-0.359245305	0.656	0.887	5.89E-12
BTG1	2.77E-16	-0.374286647	0.842	0.958	6.05E-12
ABI3	3.01E-16	-0.380580745	0.076	0.309	6.57E-12
C1orf162	3.08E-16	-0.364585367	0.816	0.973	6.72E-12
LY86	3.53E-16	-0.383835148	0.437	0.745	7.69E-12
GPR65	3.55E-16	-0.417059395	0.284	0.596	7.75E-12
PAK1	4.81E-16	-0.365869057	0.107	0.365	1.05E-11
SLC7A7	5.52E-16	-0.392917364	0.403	0.703	1.20E-11
MT-CYB	6.20E-16	0.296251057	1	1	1.35E-11
GSTK1	6.88E-16	-0.381209179	0.563	0.849	1.50E-11
POMP	8.10E-16	0.431824242	0.845	0.804	1.77E-11
CKAP4	9.11E-16	0.595100445	0.492	0.261	1.99E-11
SERPINB9	1.26E-15	0.566170805	0.589	0.377	2.74E-11
SH3BP1	1.36E-15	-0.341432958	0.1	0.35	2.96E-11
GLTSCR2	1.55E-15	-0.41357995	0.344	0.65	3.38E-11
ZFP36L2	1.63E-15	-0.423080566	0.704	0.911	3.55E-11
CMTM7	1.63E-15	-0.392983938	0.289	0.608	3.56E-11
FCGR1A	1.82E-15	0.627832275	0.666	0.51	3.97E-11
ZEB2	1.97E-15	0.406980822	0.897	0.902	4.31E-11
RGCC	2.18E-15	0.887714837	0.411	0.166	4.75E-11
NFKBIZ	2.61E-15	-0.560079273	0.42	0.685	5.70E-11
CXCL3	2.64E-15	-1.21227825	0.372	0.605	5.75E-11
CALHM2	2.66E-15	-0.284324531	0.045	0.246	5.79E-11
OXA1L	3.22E-15	-0.394737495	0.296	0.596	7.02E-11
CD52	3.58E-15	-0.531549	0.599	0.828	7.81E-11
STXBP2	4.39E-15	0.598695781	0.862	0.84	9.57E-11
NR4A2	4.86E-15	-0.459784227	0.618	0.834	1.06E-10
RNASET2	5.39E-15	-0.420330882	0.542	0.819	1.17E-10
EEF1G	5.96E-15	-0.31401012	0.718	0.884	1.30E-10
FAM96A	6.50E-15	-0.392926401	0.32	0.62	1.42E-10
GK5	6.75E-15	0.426431923	0.246	0.042	1.47E-10
CD82	6.78E-15	0.494889427	0.341	0.116	1.48E-10
AP1S2	8.43E-15	-0.359357172	0.706	0.893	1.84E-10
OGFRL1	9.69E-15	-0.320648262	0.091	0.326	2.11E-10
ATP1A1	9.86E-15	0.528642481	0.609	0.442	2.15E-10
HSPA8	1.02E-14	-0.308291799	0.589	0.822	2.22E-10
LIPA	1.17E-14	-0.500057641	0.334	0.626	2.55E-10
MCL1	1.17E-14	0.347691396	0.931	0.902	2.56E-10
APEX1	1.25E-14	-0.373236718	0.198	0.478	2.72E-10
LPAR6	1.33E-14	-0.329022521	0.033	0.214	2.91E-10
ALDH2	1.37E-14	-0.354958719	0.344	0.656	2.99E-10
SEMA6B	1.39E-14	0.461189773	0.234	0.039	3.02E-10
RGS14	1.69E-14	-0.28192467	0.055	0.258	3.69E-10
RASSF4	1.72E-14	-0.391258518	0.141	0.392	3.75E-10

PHB2	2.14E-14	-0.357829176	0.308	0.596	4.66E-10
NME3	2.25E-14	-0.282722121	0.091	0.323	4.90E-10
JKAMP	2.36E-14	-0.268912131	0.086	0.318	5.14E-10
EIF4EBP1	2.44E-14	-0.342035037	0.186	0.466	5.32E-10
SRSF2	2.53E-14	0.481968712	0.704	0.579	5.52E-10
LST1	2.82E-14	-0.353027314	0.804	0.95	6.15E-10
RSL24D1	3.20E-14	-0.34497125	0.413	0.709	6.97E-10
CLNS1A	3.31E-14	-0.308933704	0.141	0.398	7.22E-10
SLA	3.33E-14	0.522983732	0.628	0.448	7.26E-10
RELB	3.47E-14	0.544729368	0.346	0.134	7.57E-10
CXCL2	3.75E-14	-0.887324338	0.511	0.721	8.17E-10
CLEC2B	4.64E-14	-0.346246205	0.243	0.546	1.01E-09
TMEM134	4.79E-14	-0.261086847	0.036	0.214	1.05E-09
AMPH	4.89E-14	-0.305142647	0.031	0.205	1.07E-09
RPL3	5.59E-14	-0.268557831	0.933	0.979	1.22E-09
POU2F2	5.86E-14	-0.264323062	0.236	0.543	1.28E-09
HLA-DMB	5.97E-14	-0.321297743	0.167	0.43	1.30E-09
GRINA	1.10E-13	0.418699076	0.828	0.786	2.39E-09
CNPY3	1.12E-13	-0.354768827	0.496	0.772	2.45E-09
SDC2	1.24E-13	0.4546952	0.21	0.03	2.71E-09
SLCO4A1	1.30E-13	0.417504339	0.205	0.03	2.84E-09
EIF4B	1.36E-13	-0.331705973	0.442	0.721	2.97E-09
APLP2	1.56E-13	0.436471339	0.845	0.813	3.40E-09
HMGN3	1.74E-13	-0.337348565	0.267	0.546	3.80E-09
MT2A	1.89E-13	1.636275718	0.749	0.677	4.13E-09
RPL36A	1.91E-13	0.344107061	0.9	0.905	4.16E-09
BCL3	1.99E-13	0.452271233	0.47	0.243	4.34E-09
TECR	2.07E-13	-0.310945642	0.107	0.335	4.52E-09
EIF3M	2.14E-13	-0.353577386	0.52	0.789	4.67E-09
ASGR1	2.18E-13	-0.360739887	0.263	0.552	4.76E-09
RSL1D1	2.50E-13	-0.391940722	0.365	0.659	5.45E-09
ZNF581	2.53E-13	-0.28757085	0.064	0.264	5.52E-09
TNF	2.90E-13	-0.892699849	0.141	0.362	6.32E-09
CXCL8	3.64E-13	-0.806642735	0.537	0.709	7.95E-09
POLR1D	4.21E-13	-0.316428213	0.279	0.582	9.18E-09
FLI1	4.57E-13	-0.341245376	0.184	0.439	9.97E-09
G0S2	4.72E-13	0.619564905	0.525	0.279	1.03E-08
GSN	5.83E-13	-0.345574912	0.222	0.499	1.27E-08
MID1IP1	6.27E-13	-0.443701271	0.167	0.409	1.37E-08
UQCRB	6.89E-13	-0.289111334	0.811	0.961	1.50E-08
UQCRH	7.05E-13	-0.305793287	0.785	0.92	1.54E-08
PPIF	8.31E-13	0.55505819	0.484	0.282	1.81E-08
CTSB	9.50E-13	0.442945072	0.936	0.902	2.07E-08
IL1RAP	1.01E-12	0.544057788	0.372	0.163	2.20E-08
DBNDD2	1.02E-12	-0.274297258	0.06	0.246	2.23E-08
IGBP1	1.09E-12	-0.301065899	0.189	0.448	2.38E-08
DDX3X	1.17E-12	0.417834135	0.73	0.638	2.56E-08
MRPL34	1.24E-12	-0.26336245	0.081	0.288	2.72E-08
ERP29	1.35E-12	-0.36900606	0.501	0.763	2.95E-08
MBNL1	1.38E-12	-0.392486064	0.47	0.745	3.01E-08
KCNE3	1.46E-12	-0.292850357	0.107	0.326	3.19E-08
PRDX1	1.50E-12	-0.298274605	0.597	0.855	3.28E-08
TWF2	1.71E-12	-0.314594664	0.337	0.629	3.72E-08
CRIP1	1.73E-12	-0.432392712	0.687	0.866	3.77E-08
NCF1	1.79E-12	-0.478909772	0.267	0.531	3.91E-08
GPR34	1.91E-12	-0.268730943	0.021	0.166	4.17E-08
SULT1B1	1.98E-12	-0.344482577	0.098	0.309	4.32E-08
NPM1	2.01E-12	-0.368678168	0.587	0.825	4.37E-08
SH3BGRL	2.11E-12	-0.271486356	0.671	0.896	4.60E-08
AC004556.1	2.13E-12	0.307680327	0.143	0.003	4.65E-08
NPC2	2.30E-12	-0.259153834	0.876	0.976	5.02E-08
TMEM173	2.49E-12	-0.27821512	0.124	0.353	5.42E-08
TLR2	2.57E-12	0.53456317	0.57	0.407	5.60E-08
TAF12	2.57E-12	-0.253300176	0.076	0.276	5.61E-08
LCP1	2.68E-12	0.332223678	0.909	0.905	5.85E-08
SDC4	2.69E-12	0.493936497	0.291	0.098	5.86E-08
CORO1A	2.88E-12	-0.282971985	0.814	0.938	6.29E-08
LETMD1	3.27E-12	-0.25796423	0.067	0.255	7.14E-08
FLOT1	3.33E-12	0.512771122	0.518	0.335	7.27E-08
CXCR4	3.40E-12	-0.478538474	0.432	0.653	7.41E-08

PSMG2	3.63E-12	-0.289837632	0.265	0.549	7.92E-08
LGALS2	3.66E-12	-0.344781449	0.274	0.543	7.97E-08
IER3	3.85E-12	-0.454862496	0.616	0.822	8.39E-08
CAMK1	4.81E-12	-0.364676252	0.1	0.303	1.05E-07
RNF141	4.97E-12	-0.357810311	0.193	0.436	1.08E-07
HBA2	5.53E-12	-0.856653524	0.017	0.151	1.21E-07
ARL2BP	6.38E-12	-0.263888005	0.098	0.303	1.39E-07
RGL1	7.57E-12	0.494394678	0.282	0.095	1.65E-07
ATPIF1	8.06E-12	-0.283251481	0.258	0.528	1.76E-07
ATG16L2	1.09E-11	-0.286509939	0.153	0.386	2.37E-07
TXNIP	1.10E-11	-0.495504456	0.453	0.688	2.40E-07
EIF3G	1.12E-11	-0.305085222	0.508	0.766	2.44E-07
GDI2	1.45E-11	-0.336286827	0.566	0.813	3.16E-07
LMNA	1.49E-11	-0.318953994	0.16	0.386	3.25E-07
GPX4	1.53E-11	-0.270626767	0.716	0.914	3.35E-07
MAT2B	1.58E-11	-0.295539477	0.174	0.415	3.44E-07
SDCBP	1.59E-11	0.325495486	0.933	0.914	3.46E-07
TFRC	1.68E-11	0.564708456	0.401	0.208	3.66E-07
GIMAP4	1.69E-11	-0.349653219	0.191	0.424	3.68E-07
ZFAS1	1.73E-11	-0.30786593	0.764	0.908	3.76E-07
MEF2C	2.67E-11	-0.285581258	0.208	0.457	5.83E-07
ADRB2	2.78E-11	-0.250200274	0.05	0.214	6.06E-07
EIF3F	3.05E-11	-0.292212128	0.647	0.858	6.66E-07
C14orf166	3.09E-11	-0.339502056	0.432	0.682	6.73E-07
CHCHD10	3.17E-11	-0.341851873	0.308	0.579	6.92E-07
CLEC4E	3.19E-11	-0.313722654	0.566	0.831	6.95E-07
DDX21	3.64E-11	0.455901834	0.666	0.564	7.94E-07
SSR3	3.78E-11	-0.311453188	0.351	0.626	8.25E-07
PTGER4	3.81E-11	-0.300363415	0.062	0.228	8.31E-07
PDK4	3.86E-11	-0.415143187	0.093	0.285	8.41E-07
ARF6	4.14E-11	0.43956975	0.513	0.335	9.03E-07
LYSMD2	4.31E-11	-0.280284618	0.105	0.306	9.41E-07
CD163	4.49E-11	0.335670281	0.945	0.944	9.80E-07
PRAM1	4.87E-11	-0.270978732	0.158	0.383	1.06E-06
RPS4Y1	4.91E-11	-0.2936635	0.711	0.884	1.07E-06
SNHG7	5.42E-11	-0.274512407	0.148	0.365	1.18E-06
NKG7	5.79E-11	0.654292884	0.418	0.246	1.26E-06
CORO1B	5.88E-11	-0.322752006	0.203	0.433	1.28E-06
IGKV2-24	5.93E-11	-0.317168435	0.043	0.193	1.29E-06
CD302	6.03E-11	-0.302744213	0.621	0.858	1.31E-06
HEBP1	6.47E-11	-0.258577376	0.131	0.344	1.41E-06
PTPN1	7.25E-11	0.445629231	0.415	0.237	1.58E-06
MRPL51	8.47E-11	-0.285977941	0.236	0.49	1.85E-06
UBL7	9.16E-11	-0.259222664	0.086	0.267	2.00E-06
SUB1	9.74E-11	0.334478441	0.864	0.855	2.13E-06
BACH1	9.85E-11	0.520452479	0.544	0.398	2.15E-06
ADM	9.96E-11	0.537477185	0.547	0.383	2.17E-06
IFITM3	1.01E-10	0.588441274	0.89	0.908	2.21E-06
GK	1.03E-10	0.479288769	0.68	0.546	2.24E-06
SNRPC	1.07E-10	-0.260124031	0.165	0.389	2.34E-06
FAM26F	1.15E-10	-0.30659195	0.138	0.344	2.51E-06
CAST	1.18E-10	-0.304740006	0.64	0.837	2.58E-06
SFPQ	1.33E-10	0.443056398	0.668	0.567	2.90E-06
FGD2	1.48E-10	-0.339541714	0.148	0.353	3.23E-06
CUTA	1.51E-10	-0.265666589	0.32	0.588	3.29E-06
HPGD	1.73E-10	0.500861551	0.248	0.083	3.77E-06
CEBPB	1.83E-10	0.438883201	0.737	0.635	3.99E-06
BTG2	1.86E-10	-0.32912434	0.224	0.457	4.05E-06
LSM4	2.19E-10	-0.296319433	0.181	0.407	4.78E-06
ATP5A1	2.52E-10	-0.328191065	0.401	0.656	5.51E-06
ADSS	2.53E-10	-0.25274206	0.179	0.407	5.52E-06
NAGK	2.66E-10	-0.271651572	0.179	0.398	5.81E-06
SNX2	2.93E-10	-0.279047202	0.339	0.602	6.40E-06
MYL12A	3.00E-10	0.266065754	0.952	0.944	6.53E-06
TNRC6B	3.05E-10	-0.257861756	0.103	0.297	6.65E-06
MGST3	3.26E-10	-0.289035351	0.346	0.608	7.11E-06
ITGA4	3.44E-10	-0.307412853	0.241	0.475	7.51E-06
ABHD5	3.46E-10	-0.291063708	0.21	0.442	7.55E-06
ADK	4.54E-10	-0.256194507	0.072	0.234	9.91E-06
FAM129B	5.63E-10	0.408033384	0.26	0.095	1.23E-05

ANKRD28	5.76E-10	0.459384081	0.406	0.22	1.26E-05
PNP	5.82E-10	0.474277948	0.566	0.424	1.27E-05
UBXN1	6.34E-10	-0.302144923	0.439	0.682	1.38E-05
MCTP1	6.82E-10	-0.334273636	0.286	0.54	1.49E-05
TNFAIP2	6.90E-10	-0.290332671	0.26	0.513	1.50E-05
RGS19	7.54E-10	-0.257958382	0.351	0.623	1.64E-05
ATP5F1	7.68E-10	-0.259617576	0.551	0.789	1.67E-05
GNG10	8.05E-10	0.466234132	0.621	0.528	1.76E-05
BAZ1A	8.95E-10	0.498719906	0.609	0.481	1.95E-05
F13A1	9.04E-10	-0.290601402	0.181	0.386	1.97E-05
LILRA2	9.43E-10	-0.250624827	0.327	0.596	2.06E-05
EVI2A	1.01E-09	-0.250943959	0.16	0.374	2.20E-05
ENO1	1.04E-09	0.315111665	0.859	0.831	2.27E-05
IFNGR2	1.05E-09	0.447591141	0.68	0.596	2.28E-05
RNF130	1.05E-09	-0.281075661	0.64	0.837	2.28E-05
EIF3H	1.13E-09	-0.265755234	0.625	0.804	2.46E-05
PEBP1	1.15E-09	-0.263960125	0.11	0.288	2.50E-05
SH3BP5	1.33E-09	0.405848703	0.413	0.246	2.90E-05
NPL	1.39E-09	-0.273944046	0.208	0.445	3.03E-05
YWHAZ	1.41E-09	0.272083541	0.878	0.887	3.08E-05
CLEC5A	1.49E-09	0.31881133	0.141	0.018	3.24E-05
PPP1CC	1.52E-09	-0.284984783	0.317	0.564	3.32E-05
AHNAK	1.59E-09	-0.325153389	0.623	0.831	3.47E-05
FCGR1B	1.61E-09	0.478945422	0.356	0.193	3.51E-05
PSMB9	1.61E-09	-0.26104544	0.317	0.579	3.51E-05
RGS2	1.65E-09	-0.419229125	0.692	0.816	3.60E-05
TUFM	2.01E-09	-0.292951813	0.358	0.611	4.39E-05
METTL7B	2.11E-09	0.369203635	0.155	0.027	4.61E-05
DAD1	2.13E-09	-0.281723683	0.401	0.656	4.63E-05
RNASE1	2.22E-09	-0.718411688	0.126	0.294	4.83E-05
DRAM2	2.24E-09	-0.260833945	0.258	0.499	4.89E-05
ARL6IP5	2.38E-09	-0.256048952	0.499	0.76	5.18E-05
CPPED1	2.63E-09	-0.280054631	0.286	0.522	5.75E-05
SH3GLB1	2.68E-09	0.486955034	0.566	0.466	5.85E-05
CNIH4	2.76E-09	0.444229925	0.592	0.478	6.02E-05
MIR4435-2HG	2.98E-09	0.508364491	0.456	0.312	6.50E-05
MAN2B1	3.18E-09	-0.251033592	0.31	0.552	6.93E-05
CD83	3.87E-09	-0.299008222	0.468	0.697	8.44E-05
HRH2	3.94E-09	0.410230163	0.42	0.255	8.59E-05
LINC00936	3.97E-09	-0.313319561	0.399	0.635	8.65E-05
NDFIP1	4.23E-09	-0.281149854	0.346	0.576	9.23E-05
NDUFB5	4.61E-09	-0.265516943	0.317	0.564	0.000100615
TMEM167A	5.31E-09	0.392429283	0.702	0.623	0.000115833
S100P	5.66E-09	0.440689543	0.15	0.027	0.000123509
UQCR10	6.00E-09	-0.281215685	0.616	0.828	0.000130879
SLC11A1	6.33E-09	0.382897843	0.819	0.813	0.000138108
SNHG8	6.71E-09	-0.256304857	0.339	0.588	0.000146422
ZFAND5	6.78E-09	-0.362742585	0.718	0.819	0.000147895
METRNL	7.01E-09	0.372752673	0.315	0.151	0.000152881
TBXAS1	7.13E-09	-0.267441695	0.48	0.742	0.000155433
TMEM256	7.25E-09	-0.275914442	0.37	0.605	0.000158011
MRPS24	8.87E-09	-0.270571514	0.255	0.478	0.000193464
PTGS2	1.04E-08	-0.420114959	0.186	0.374	0.000226432
CCL3L3	1.12E-08	-0.556048562	0.193	0.377	0.000243203
LINC00493	1.13E-08	-0.254332846	0.298	0.537	0.000245548
TAGAP	1.13E-08	-0.313842379	0.236	0.448	0.000246002
VAPA	1.18E-08	0.367585826	0.785	0.763	0.000257155
EIF3L	1.31E-08	-0.250660757	0.556	0.772	0.000286233
PKM	1.36E-08	0.289739859	0.866	0.849	0.000297635
SYF2	1.44E-08	-0.268000174	0.382	0.62	0.000313749
FOS	1.48E-08	-0.327885371	0.99	0.988	0.000322504
LIMS1	1.50E-08	0.379019526	0.683	0.585	0.000326533
DYSF	1.54E-08	0.327264406	0.234	0.086	0.000335368
MBOAT7	1.64E-08	0.403775298	0.36	0.205	0.000357332
CCT8	1.75E-08	-0.257676365	0.294	0.534	0.000380835
INSIG1	1.82E-08	0.48080318	0.604	0.51	0.000397368
ARHGDIA	1.91E-08	0.333785898	0.692	0.62	0.00041596
DAZAP2	2.10E-08	0.292916599	0.869	0.89	0.000457993
CHSY1	2.60E-08	0.34398371	0.289	0.136	0.000567634
ZNHIT1	2.75E-08	-0.253342498	0.332	0.564	0.000598921

RAB1A	2.80E-08	0.361630849	0.513	0.392	0.000611197
TMEM219	3.06E-08	-0.25970211	0.375	0.608	0.000666524
PLA2G7	3.13E-08	0.286980053	0.162	0.039	0.0006828
RHOC	3.26E-08	-0.28234546	0.131	0.297	0.000710464
IFI6	3.39E-08	0.530415212	0.329	0.184	0.000738762
PGLS	3.83E-08	-0.252740221	0.57	0.789	0.000836088
VNN1	3.98E-08	0.361549006	0.177	0.05	0.000867767
CTB-61M7.2	4.14E-08	0.390656299	0.284	0.134	0.000903565
LIMK2	4.35E-08	0.311821588	0.186	0.056	0.000949394
UBE2J1	5.15E-08	0.346030071	0.656	0.576	0.001122324
CD44	5.52E-08	0.302176643	0.85	0.81	0.001203018
RGS10	5.63E-08	-0.262042823	0.372	0.599	0.001228268
PPARG	5.97E-08	0.385706932	0.203	0.071	0.001302751
BIN1	7.38E-08	0.300337628	0.21	0.074	0.00160992
BZW1	7.58E-08	0.351528525	0.573	0.484	0.001653831
UBE2D1	8.20E-08	0.320875776	0.695	0.668	0.001788472
STEAP4	8.28E-08	0.253008927	0.103	0.009	0.001806044
PSMA7	8.28E-08	0.309157493	0.771	0.748	0.00180664
IER2	8.54E-08	-0.289938208	0.85	0.941	0.001861332
NR3C1	8.79E-08	0.37299521	0.437	0.288	0.001917172
NDUFS7	9.49E-08	-0.260909508	0.406	0.632	0.002070659
RBP7	1.01E-07	-0.26783169	0.294	0.51	0.002212124
CDC42	1.07E-07	0.339503278	0.776	0.783	0.002333199
PLAUR	1.11E-07	0.325859323	0.928	0.878	0.002421283
HLA-DRA	1.13E-07	-0.251931328	0.94	0.976	0.00247254
NFKB2	1.16E-07	0.342124326	0.236	0.098	0.00253098
REEP5	1.17E-07	-0.252092538	0.43	0.647	0.002548598
NCOA4	1.30E-07	-0.269069433	0.365	0.579	0.002828605
PTPN12	1.55E-07	0.440875011	0.387	0.258	0.003385393
SQLE	1.55E-07	0.375247498	0.241	0.107	0.003386553
PLIN2	1.58E-07	0.449759378	0.656	0.567	0.003448407
C12orf57	1.74E-07	-0.273172999	0.205	0.392	0.003802833
BATF	1.76E-07	0.378132328	0.243	0.11	0.003838339
ADGRE2	2.00E-07	0.382655355	0.432	0.291	0.004353664
SERPINB2	2.01E-07	0.512283826	0.37	0.208	0.004393776
RASA2	2.08E-07	0.349192859	0.289	0.148	0.004545437
EIF4A1	2.30E-07	0.328999966	0.654	0.599	0.005012619
NDUFS8	2.33E-07	-0.260215527	0.296	0.501	0.005083856
TNFAIP3	2.33E-07	0.367661459	0.723	0.626	0.005090934
PTK2B	2.37E-07	0.361258649	0.399	0.261	0.005167173
CCL4L2	2.40E-07	-0.590381887	0.143	0.291	0.005229013
PLIN3	2.41E-07	0.381521346	0.48	0.377	0.005250736
CFP	2.42E-07	-0.264421706	0.695	0.852	0.00527462
TMEM120A	2.76E-07	0.378305964	0.387	0.252	0.006024787
SBNO2	3.14E-07	0.307043271	0.184	0.065	0.006847766
MAP2K1	3.21E-07	0.386588523	0.582	0.501	0.006998741
DSE	3.29E-07	0.373711964	0.523	0.418	0.007170198
PPP1CB	3.46E-07	0.36999974	0.547	0.469	0.007553649
CAPZA1	3.55E-07	0.337246823	0.683	0.623	0.007741256
JAK3	3.92E-07	0.282662801	0.212	0.083	0.008550705
CES1	4.41E-07	0.374116045	0.301	0.16	0.009619586
PTPRE	4.53E-07	0.332730471	0.795	0.736	0.009879917
BASP1	4.72E-07	0.442874749	0.391	0.249	0.01029172
BLOC1S1	4.73E-07	0.335655036	0.604	0.528	0.010315654
ATP13A3	5.28E-07	0.390249737	0.425	0.288	0.011514613
NDUFA11	5.33E-07	0.367313561	0.554	0.466	0.011627487
MPHOSPH6	5.88E-07	0.38940687	0.339	0.208	0.012827793
TXN	5.97E-07	0.291077458	0.74	0.724	0.013011306
STAT3	7.04E-07	0.360148557	0.451	0.335	0.015349114
PET100	8.48E-07	0.323595114	0.673	0.614	0.018494894
CXCL1	9.42E-07	-0.587348778	0.079	0.196	0.020544791
RILPL2	9.75E-07	0.277832522	0.764	0.748	0.021262278
IL1R1	1.01E-06	0.309989949	0.131	0.033	0.021945441
KLHL2	1.10E-06	0.303928931	0.227	0.101	0.023951048
FAM20A	1.20E-06	0.373316942	0.305	0.178	0.026171289
EMP1	1.20E-06	0.407414476	0.208	0.089	0.026250301
PLAC8	1.24E-06	0.443500391	0.468	0.35	0.026982265
LDLR	1.41E-06	0.370518245	0.325	0.196	0.030773836
FTH1	1.47E-06	0.2601452	1	1	0.032134873
CCR1	1.66E-06	0.427957845	0.377	0.258	0.036288536

FAM49B	1.84E-06	0.287480896	0.721	0.677	0.040234892
EFHD2	1.94E-06	0.343554159	0.58	0.528	0.042208516
SGK1	1.96E-06	0.325154879	0.609	0.484	0.042647933
PHC2	2.16E-06	0.392140615	0.356	0.231	0.04709934

List of differentially expressed genes of S100A8_TMo in overall stage (PR versus PP)

gene	p_val	avg_logFC	pct.1	pct.2	p_val_adj
VCAN	6.66E-37	0.905344837	0.963	0.817	1.45E-32
THBS1	1.31E-35	1.50134048	0.873	0.495	2.85E-31
S100A4	1.26E-30	-0.467337362	0.997	0.984	2.74E-26
SAT1	9.00E-29	0.716416111	0.973	0.93	1.96E-24
NAMPT	7.41E-26	0.918853724	0.916	0.624	1.62E-21
SAMSN1	1.31E-24	0.933459179	0.833	0.543	2.85E-20
DEFA3	2.60E-23	-1.895513427	0.03	0.371	5.68E-19
HLA-DRB5	4.12E-23	-1.018954267	0.157	0.575	8.98E-19
CST3	1.93E-22	-0.54317497	0.97	0.978	4.21E-18
ACSL1	1.09E-21	1.049575805	0.679	0.269	2.37E-17
RPS13	1.35E-21	-0.395662889	0.993	1	2.94E-17
RPL11	2.28E-21	-0.35315759	0.993	1	4.97E-17
MT-CYB	2.55E-21	0.642858081	0.993	0.962	5.56E-17
NACA	1.15E-20	-0.494023832	0.923	0.978	2.51E-16
SRGN	1.30E-20	0.447465844	0.997	0.984	2.84E-16
RPL30	2.29E-19	-0.353615744	1	1	5.00E-15
S100A6	2.03E-18	-0.311356653	1	1	4.42E-14
TRMT6	2.67E-18	1.086263019	0.395	0.043	5.82E-14
TNFAIP6	6.00E-18	1.140725663	0.545	0.156	1.31E-13
MT-ND3	1.07E-17	0.499433249	0.993	0.946	2.33E-13
FPR2	1.38E-17	0.974584601	0.538	0.177	3.00E-13
RPS29	8.35E-17	0.66721325	0.816	0.661	1.82E-12
RPL5	1.52E-16	-0.463006443	0.839	0.946	3.31E-12
RPL18	2.39E-16	-0.398603394	0.957	0.989	5.22E-12
SLC39A8	2.59E-16	1.037103272	0.348	0.027	5.65E-12
RPL14	2.60E-16	-0.419264259	0.923	0.978	5.68E-12
PAG1	5.67E-16	0.913791646	0.565	0.215	1.24E-11
PABPC1	8.42E-16	-0.509702952	0.779	0.941	1.84E-11
FXYD5	5.47E-15	-0.642546234	0.622	0.855	1.19E-10
MNDA	5.93E-15	-0.627926375	0.739	0.914	1.29E-10
HBB	1.18E-14	-0.751081605	0.054	0.312	2.57E-10
SELL	1.44E-14	0.705574847	0.739	0.548	3.15E-10
RPL10	3.21E-14	-0.317031545	0.99	0.995	6.99E-10
CD74	3.51E-14	-0.517392309	0.856	0.935	7.66E-10
S100A9	3.99E-14	0.510944502	0.99	0.989	8.70E-10
GPX1	1.17E-13	0.670503523	0.793	0.645	2.56E-09
AGFG1	1.26E-13	0.84456784	0.532	0.22	2.75E-09
RPS7	1.29E-13	-0.300925305	0.977	0.995	2.81E-09
RPL32	1.59E-13	-0.251856117	0.99	0.995	3.47E-09
SLC2A3	1.69E-13	0.774446604	0.699	0.446	3.69E-09
ETS2	1.76E-13	0.826194205	0.528	0.215	3.84E-09
RPL29	2.96E-13	-0.354289827	0.943	0.984	6.44E-09
CRIP1	3.13E-13	-0.920474271	0.341	0.64	6.82E-09
SEC11A	4.14E-13	-0.612423593	0.455	0.742	9.03E-09
H3F3B	4.37E-13	0.425185601	0.946	0.941	9.54E-09
TMSB4X	8.70E-13	-0.293829944	0.987	1	1.90E-08
CKAP4	2.93E-12	0.735201415	0.518	0.22	6.39E-08
LGALS2	3.58E-12	-0.629588863	0.054	0.28	7.81E-08
RPL19	3.83E-12	-0.277565141	0.987	0.989	8.35E-08
S100A12	5.94E-12	0.370482141	0.973	0.925	1.30E-07
EVI2B	6.37E-12	-0.630920511	0.411	0.677	1.39E-07
MT-CO1	8.41E-12	0.31941512	1	0.989	1.83E-07
GAPDH	8.45E-12	-0.270170595	0.993	1	1.84E-07
APLP2	9.03E-12	0.652131743	0.706	0.5	1.97E-07
SLC25A6	9.55E-12	-0.56179545	0.565	0.78	2.08E-07
STXBP2	1.18E-11	0.491737255	0.876	0.694	2.57E-07
HP	1.31E-11	0.761359151	0.284	0.032	2.85E-07
COMMD6	1.56E-11	-0.40392673	0.562	0.855	3.41E-07
RPL35A	2.19E-11	-0.325126255	0.94	0.989	4.77E-07
RAB31	3.26E-11	0.665049234	0.635	0.403	7.12E-07

ATP5G2	3.94E-11	-0.382820789	0.883	0.935	8.60E-07
UPP1	4.55E-11	0.725304258	0.565	0.296	9.92E-07
CSTA	4.73E-11	-0.475179959	0.783	0.919	1.03E-06
AQP9	4.99E-11	0.735873205	0.418	0.145	1.09E-06
PLAC8	5.01E-11	0.79055861	0.552	0.306	1.09E-06
RPL8	5.10E-11	-0.29486275	0.953	0.995	1.11E-06
AREG	5.46E-11	0.713266958	0.742	0.495	1.19E-06
VIM	6.77E-11	-0.307040059	0.983	0.989	1.48E-06
NKG7	7.08E-11	0.801427295	0.408	0.134	1.54E-06
MT-ND4	7.69E-11	0.313671287	0.997	0.973	1.68E-06
STOM	8.02E-11	0.759819579	0.348	0.091	1.75E-06
HLA-DQB1	9.76E-11	-0.646749063	0.094	0.323	2.13E-06
RPS3A	9.98E-11	-0.254593439	0.987	0.984	2.18E-06
RPS14	1.18E-10	-0.300542029	0.943	0.984	2.56E-06
RETN	1.21E-10	1.13206837	0.498	0.247	2.63E-06
CTSS	1.61E-10	-0.387394998	0.809	0.887	3.51E-06
ASPH	2.13E-10	0.798116144	0.388	0.134	4.64E-06
EEF1A1	2.54E-10	-0.266110021	0.993	1	5.55E-06
NCF1	2.56E-10	-0.625046114	0.311	0.575	5.59E-06
RPL6	3.23E-10	-0.313518252	0.94	0.984	7.05E-06
SLC25A5	3.25E-10	-0.574249487	0.418	0.677	7.09E-06
AP1S2	3.79E-10	-0.521776959	0.522	0.731	8.27E-06
ADAMTS2	3.96E-10	-0.474431614	0.05	0.242	8.64E-06
GPX4	4.43E-10	-0.552865067	0.385	0.64	9.66E-06
LCP1	4.57E-10	0.434977639	0.833	0.656	9.96E-06
CST7	5.59E-10	0.826327261	0.274	0.048	1.22E-05
P2RY13	6.45E-10	-0.471438651	0.013	0.161	1.41E-05
ARPC3	8.03E-10	-0.359097857	0.819	0.914	1.75E-05
FAM26F	9.05E-10	-0.454926693	0.047	0.237	1.97E-05
CD52	1.35E-09	-0.587181695	0.328	0.586	2.94E-05
FPR1	1.60E-09	0.518298772	0.749	0.575	3.48E-05
ERP29	1.64E-09	-0.540382921	0.278	0.538	3.57E-05
IFI30	1.78E-09	0.520722721	0.709	0.527	3.88E-05
CD63	1.89E-09	0.563436734	0.699	0.581	4.12E-05
IL1R2	2.40E-09	0.770512375	0.602	0.398	5.23E-05
TKT	2.78E-09	-0.495358534	0.609	0.753	6.07E-05
C20orf27	3.16E-09	-0.497212468	0.14	0.371	6.89E-05
FAM65B	3.21E-09	0.624145809	0.562	0.333	7.00E-05
GK5	3.23E-09	0.669625325	0.181	0.005	7.04E-05
HSD17B11	3.32E-09	-0.519570561	0.331	0.597	7.23E-05
RPL7A	4.16E-09	-0.269361663	0.946	0.984	9.07E-05
RSL1D1	4.31E-09	-0.498677984	0.191	0.452	9.39E-05
HVCN1	4.70E-09	-0.351506437	0.01	0.14	0.000102473
MT-ATP6	5.13E-09	0.379874996	0.987	0.952	0.000111817
BCL2A1	5.28E-09	0.399548619	0.803	0.554	0.000115196
HIF1A	5.74E-09	0.732265944	0.465	0.22	0.000125095
BTF3	6.55E-09	-0.378282588	0.819	0.909	0.000142852
BAZ1A	6.59E-09	0.625321149	0.515	0.29	0.000143664
LILRA5	7.24E-09	0.558241177	0.706	0.532	0.000157932
RNASE6	7.81E-09	-0.443922895	0.214	0.478	0.000170266
GCA	7.84E-09	0.431096722	0.843	0.796	0.000170976
RPL24	8.98E-09	-0.31487601	0.903	0.952	0.000195742
PFKFB3	1.00E-08	0.685581793	0.301	0.086	0.000218348
TFRC	1.25E-08	0.683251708	0.298	0.081	0.000272593
VAMP8	1.37E-08	-0.511533116	0.365	0.602	0.000297967
MS4A7	1.41E-08	-0.449356973	0.114	0.333	0.000307513
NOP10	1.67E-08	-0.426094528	0.672	0.833	0.000365004
BCL3	1.70E-08	0.590832343	0.321	0.091	0.000370562
GLRX	1.76E-08	-0.503914842	0.341	0.581	0.000384019
SLC11A1	1.90E-08	0.579942486	0.682	0.516	0.000414863
S100P	1.98E-08	0.707525641	0.268	0.059	0.000431024
RPL37A	2.47E-08	0.399076927	0.893	0.796	0.000539421
SLA	2.63E-08	0.629699193	0.441	0.22	0.000572525
TPM3	2.85E-08	0.45170458	0.776	0.629	0.000621888
BLVRB	2.90E-08	-0.533380841	0.368	0.602	0.000633372
ATP1A1	2.94E-08	0.615799406	0.334	0.118	0.000641823
MARCKS	3.77E-08	0.822751146	0.308	0.102	0.000823173
TYMP	4.68E-08	0.444280659	0.709	0.473	0.001021148
TNFAIP3	6.15E-08	0.625521896	0.559	0.328	0.001341176
RUNX1	6.37E-08	0.55111689	0.395	0.167	0.001390175

HBA2	7.39E-08	-0.424282523	0.01	0.124	0.001610909
RPS27	7.63E-08	0.266247165	0.983	0.952	0.001664812
LITAF	7.85E-08	0.633684748	0.548	0.323	0.001711286
CD36	9.13E-08	0.672463979	0.405	0.194	0.001990826
MAFB	9.25E-08	0.581838621	0.632	0.457	0.002016713
AIF1	1.00E-07	-0.392543901	0.866	0.93	0.002181358
SH3BP5	1.06E-07	0.670722905	0.278	0.086	0.00232086
SLC40A1	1.14E-07	-0.378055528	0.013	0.129	0.00249594
RILPL2	1.17E-07	0.565203448	0.559	0.36	0.002554046
CD14	1.21E-07	0.573249726	0.672	0.522	0.002630554
C12orf57	1.26E-07	-0.625038603	0.104	0.29	0.002739274
LRG1	1.46E-07	0.471024544	0.204	0.032	0.003175835
SULT1A1	1.61E-07	-0.378806047	0.127	0.333	0.003503492
PHB2	2.14E-07	-0.379324834	0.151	0.36	0.004661027
CD99	2.19E-07	-0.497344296	0.431	0.656	0.004766687
HLA-DPA1	2.24E-07	-0.554302398	0.211	0.425	0.00488694
GABARAP	2.36E-07	-0.277131281	0.903	0.968	0.005143816
PLD3	2.60E-07	-0.43039801	0.117	0.306	0.005672329
CFL1	2.88E-07	0.29093303	0.94	0.903	0.006273487
CD44	2.88E-07	0.449577374	0.639	0.435	0.006278139
FGL2	2.99E-07	-0.465310475	0.274	0.511	0.006512401
B4GALT5	3.17E-07	0.612157061	0.271	0.086	0.006915158
PGAM1	3.27E-07	0.462822355	0.609	0.446	0.00712726
TPM4	3.35E-07	0.638725952	0.512	0.333	0.0073079
CCPG1	3.35E-07	-0.38547978	0.134	0.333	0.007311601
SOD2	3.52E-07	0.44598494	0.789	0.608	0.007666513
RBMS1	4.50E-07	0.630253055	0.441	0.237	0.009824208
ACTR2	4.85E-07	0.502336089	0.619	0.489	0.010585751
ABTB1	5.34E-07	-0.368248927	0.064	0.22	0.011648305
ADM	5.43E-07	0.622856921	0.361	0.161	0.011833129
SNHG8	5.84E-07	-0.469824679	0.137	0.328	0.012744591
FAM96A	6.29E-07	-0.397972927	0.14	0.333	0.013712659
LIMD2	6.33E-07	-0.522682979	0.258	0.462	0.013804454
KLF6	6.52E-07	0.398743566	0.756	0.624	0.014210189
CNIH4	6.67E-07	0.550918737	0.505	0.306	0.014537097
CUTA	7.64E-07	-0.458441236	0.207	0.414	0.01666395
GNAQ	7.83E-07	0.560742824	0.418	0.22	0.017073583
RNASET2	8.48E-07	-0.466852038	0.271	0.484	0.018491144
NUP214	8.72E-07	-0.452846832	0.211	0.419	0.019015449
RPL17	1.00E-06	-0.282065195	0.87	0.946	0.021884117
FCER1G	1.13E-06	0.292068447	0.99	0.973	0.024611613
CYP1B1	1.13E-06	0.669642315	0.391	0.204	0.024699785
GNB2L1	1.15E-06	-0.26914497	0.863	0.903	0.02506724
SH3BGRL	1.18E-06	-0.42194136	0.405	0.645	0.025706337
SPCS1	1.60E-06	-0.390783101	0.361	0.581	0.034920945
OAS1	1.66E-06	-0.291201542	0.03	0.151	0.036187433
IGSF6	1.74E-06	-0.484364422	0.241	0.435	0.037893708
GRINA	1.82E-06	0.446267005	0.652	0.478	0.039734469
AC004556.1	1.94E-06	0.361738786	0.114	0	0.042362855
CLEC5A	1.97E-06	0.383987112	0.127	0.005	0.043004898
MXD1	1.99E-06	0.548229508	0.438	0.253	0.043315483
IGKV2-24	2.00E-06	-0.431314619	0.027	0.14	0.043644857
HBEGF	2.12E-06	0.542085853	0.308	0.124	0.04617982
NEAT1	2.17E-06	0.295300535	0.963	0.946	0.047372298
G0S2	2.27E-06	0.610831497	0.348	0.151	0.049562523
IL18	2.40E-06	-0.318770715	0.057	0.199	0.052314443
CD37	2.99E-06	-0.394535743	0.518	0.704	0.065308491
PTPN12	3.11E-06	0.451981094	0.298	0.113	0.067784923
DDX21	3.43E-06	0.570481646	0.508	0.349	0.074800351
HSP90AA1	3.46E-06	0.754277921	0.625	0.425	0.075433304
POMP	3.50E-06	0.452895236	0.699	0.532	0.076311996
TMEM141	3.54E-06	-0.34387588	0.047	0.177	0.077100019
F5	4.17E-06	0.471231913	0.217	0.065	0.090837455
PRCP	4.38E-06	-0.32075117	0.07	0.215	0.095495769
STX10	4.78E-06	-0.423229881	0.167	0.349	0.104192523
ELOVL1	4.83E-06	-0.272411572	0.043	0.167	0.105402846
SERP1	5.03E-06	-0.346752432	0.732	0.833	0.109597547
RGS2	5.35E-06	-0.465538762	0.538	0.688	0.116571127
PSTPIP2	5.41E-06	0.521945002	0.221	0.065	0.118046319
RCSD1	5.92E-06	-0.368860722	0.117	0.28	0.129147115

AGTRAP	6.15E-06	-0.457806108	0.435	0.624	0.134076063
RBM47	6.42E-06	0.595459717	0.371	0.204	0.140001899
FAM200B	6.69E-06	-0.417149276	0.197	0.387	0.145926044
LSM4	6.71E-06	-0.355085644	0.074	0.215	0.146240888
ANG	6.92E-06	-0.318008015	0.02	0.118	0.150959732
LINC00116	7.00E-06	-0.346159891	0.08	0.226	0.152618736
METTL7B	7.02E-06	0.379841183	0.137	0.016	0.153155397
MGST3	7.18E-06	-0.360820275	0.244	0.446	0.156637705
SARAF	7.57E-06	-0.39602371	0.318	0.516	0.16515817
FKBP5	7.96E-06	0.474084239	0.532	0.36	0.173561238
ZEB2	8.15E-06	0.402956278	0.712	0.586	0.177746222
NPC2	8.24E-06	-0.261181028	0.565	0.769	0.179800846
TSC22D3	8.27E-06	0.434009728	0.726	0.608	0.180366974
EIF3M	8.55E-06	-0.419823572	0.331	0.527	0.186478938
VNN1	8.70E-06	0.470033122	0.164	0.032	0.18963555
SLC25A37	8.72E-06	0.515221237	0.408	0.226	0.190185889
SERPINB1	8.93E-06	0.280917988	0.91	0.849	0.194753859
IL1RAP	9.23E-06	0.582300115	0.217	0.07	0.201210819
FERMT3	9.23E-06	0.513269752	0.435	0.263	0.201391184
GIMAP7	9.52E-06	-0.340805796	0.087	0.231	0.207557279
CDC42	9.65E-06	0.372808774	0.652	0.538	0.210498034
CDA	9.73E-06	-0.364283022	0.371	0.575	0.212271922
SERPINB9	9.82E-06	0.539281528	0.278	0.113	0.214249435
LDHB	9.94E-06	-0.340084209	0.161	0.339	0.216721283
PHC2	1.03E-05	0.562436207	0.304	0.14	0.224779405
FCGR1A	1.04E-05	0.603181576	0.408	0.237	0.226440002
IFITM2	1.08E-05	0.353667233	0.913	0.866	0.235805703
EEF1D	1.10E-05	-0.254148011	0.866	0.93	0.239109998
MTHFD2	1.14E-05	0.455809126	0.291	0.124	0.249227938
MPST	1.14E-05	-0.370759733	0.067	0.199	0.24940018
ROMO1	1.16E-05	0.444329707	0.559	0.398	0.253117906
PYCARD	1.22E-05	-0.366758473	0.579	0.71	0.266842651
IMPDH1	1.23E-05	-0.36617137	0.12	0.28	0.267360865
ATG16L2	1.33E-05	-0.319282879	0.064	0.194	0.289776009
CCND3	1.37E-05	0.480241754	0.488	0.349	0.299513497
MS4A4A	1.44E-05	0.516232534	0.284	0.124	0.313058989
GIMAP2	1.45E-05	-0.27631644	0.05	0.172	0.315774699
HIGD2A	1.52E-05	-0.367462904	0.482	0.651	0.332202584
UBE2H	1.53E-05	0.389966624	0.187	0.048	0.333126077
CD82	1.62E-05	0.440239752	0.201	0.059	0.352547795
ADGRE2	1.62E-05	0.504407114	0.187	0.054	0.353604137
SLC27A3	1.65E-05	-0.328383531	0.04	0.151	0.358871523
CBWD2	1.66E-05	-0.266744867	0.04	0.151	0.361144133
CLU	1.68E-05	0.519091318	0.338	0.172	0.366794163
GLMP	1.74E-05	-0.277010574	0.037	0.145	0.378552281
WDR26	1.74E-05	0.505892826	0.311	0.145	0.380325816
SDC4	1.75E-05	0.411838138	0.117	0.011	0.381877701
GIMAP1	1.84E-05	-0.330550131	0.097	0.242	0.401426333
EEF1B2	1.86E-05	-0.301059013	0.666	0.812	0.406636177
LIMK2	1.92E-05	0.413332122	0.137	0.022	0.418543381
MEAF6	1.93E-05	-0.359967877	0.1	0.247	0.420205664
ANKRD28	1.94E-05	0.412828831	0.227	0.075	0.422600063
MAP3K8	2.00E-05	0.553963835	0.385	0.21	0.436778807
SFPQ	2.04E-05	0.578645661	0.438	0.29	0.444772359
ARL6IP5	2.05E-05	-0.377114954	0.278	0.484	0.44756098
KLF2	2.18E-05	0.540800859	0.328	0.167	0.475609181
PTPN1	2.24E-05	0.435650844	0.241	0.091	0.488495776
JAK3	2.28E-05	0.456266037	0.174	0.043	0.497537296

List of differentially expressed genes of IL1B_KC in overall stage (PR versus PP)

gene	p_val	avg_logFC	pct.1	pct.2	p_val_adj
NAMPT	2.84E-20	0.956645755	0.973	0.888	6.19E-16
PIM1	3.69E-20	1.137578099	0.676	0.112	8.04E-16
HSP90AA1	5.37E-19	2.030209759	0.946	0.902	1.17E-14
SOD2	1.68E-17	1.048642265	0.986	0.916	3.67E-13
ACSL1	9.65E-16	1.058050011	0.824	0.517	2.10E-11
MT2A	2.34E-15	1.691094484	0.959	0.881	5.10E-11

SOCS3	2.06E-14	0.964145864	0.851	0.58	4.49E-10
HSPE1	3.11E-14	1.834113751	0.824	0.636	6.77E-10
TMSB4X	4.34E-14	-0.522091876	0.986	1	9.46E-10
HSPH1	4.86E-14	2.170570366	0.73	0.399	1.06E-09
BAG3	5.44E-14	1.534485882	0.486	0.07	1.19E-09
LITAF	8.51E-14	1.112342287	0.878	0.706	1.86E-09
THBS1	9.39E-14	1.645777437	0.77	0.35	2.05E-09
ZFP36L2	1.33E-13	-0.877388154	0.554	0.965	2.91E-09
BATF	2.34E-13	0.892932815	0.473	0.063	5.11E-09
H3F3B	3.48E-13	0.631492377	1	0.993	7.58E-09
MIR3945HG	6.28E-13	0.9863389	0.459	0.063	1.37E-08
CTSL	8.28E-13	1.140879585	0.892	0.755	1.81E-08
B4GALT5	9.90E-13	0.772659575	0.595	0.168	2.16E-08
AREG	1.05E-12	1.0629402	0.878	0.399	2.29E-08
CXCR4	1.06E-12	-0.904725104	0.5	0.874	2.31E-08
DSE	1.30E-12	0.774566675	0.703	0.28	2.84E-08
HSPD1	1.62E-12	2.122186914	0.757	0.601	3.54E-08
PIM3	3.85E-12	0.884021104	0.662	0.266	8.39E-08
HSPB1	7.34E-12	2.171156542	0.743	0.434	1.60E-07
C1orf162	1.32E-11	-0.599876773	0.757	0.972	2.89E-07
DNAJA4	2.06E-11	1.14396563	0.338	0.021	4.50E-07
HIF1A	2.09E-11	0.873006858	0.743	0.427	4.55E-07
ITGB2	4.47E-11	-0.561834337	0.784	0.986	9.75E-07
TNIP3	4.79E-11	1.041100406	0.378	0.042	1.04E-06
GIMAP2	6.72E-11	-0.566015148	0.068	0.538	1.47E-06
EEF1A1	7.05E-11	-0.315868574	1	1	1.54E-06
BCL3	9.48E-11	0.709557401	0.554	0.168	2.07E-06
MAP3K8	1.36E-10	0.68334633	0.892	0.72	2.98E-06
HSPA5	1.46E-10	1.309226711	0.811	0.545	3.19E-06
ATP13A3	2.00E-10	0.725913286	0.527	0.147	4.36E-06
CALM2	2.45E-10	-0.558187216	0.851	0.972	5.34E-06
GPR65	2.87E-10	-0.63185002	0.284	0.755	6.25E-06
NFKBIZ	3.09E-10	-0.809774365	0.541	0.867	6.74E-06
SLC25A37	4.30E-10	0.888690395	0.581	0.238	9.37E-06
NFKB2	4.38E-10	0.723027017	0.514	0.154	9.54E-06
FPR1	6.00E-10	0.684821804	0.838	0.727	1.31E-05
ABI3	6.62E-10	-0.542068519	0.284	0.741	1.44E-05
NINJ1	9.25E-10	0.89660126	0.878	0.804	2.02E-05
G0S2	9.72E-10	1.028771138	0.676	0.287	2.12E-05
GIMAP4	1.17E-09	-0.649171047	0.257	0.72	2.54E-05
GK	1.42E-09	0.987059874	0.676	0.378	3.10E-05
GLRX	1.60E-09	-0.588070003	0.351	0.811	3.48E-05
NEAT1	1.67E-09	0.525707998	0.986	0.986	3.64E-05
HSPA1A	2.09E-09	1.290332716	0.851	0.783	4.56E-05
CORO1A	2.49E-09	-0.513581593	0.662	0.923	5.44E-05
HMGN3	2.56E-09	-0.538258004	0.311	0.762	5.58E-05
TXNIP	2.64E-09	-0.804447392	0.514	0.86	5.75E-05
DEFA3	4.67E-09	-0.626136872	0.068	0.476	0.0001019
RPS29	5.26E-09	0.445752405	0.946	0.979	0.000114643
GIMAP1	6.47E-09	-0.603396634	0.149	0.58	0.000141066
ARHGDIB	8.30E-09	-0.519653483	0.757	0.965	0.000180919
RELB	8.52E-09	0.599405537	0.527	0.196	0.000185752
CACYBP	1.07E-08	0.819199214	0.635	0.35	0.00023259
JUNB	1.15E-08	-0.476336493	0.946	1	0.00024987
CTSS	1.73E-08	-0.354326063	0.973	0.993	0.000377601
MIS18BP1	1.75E-08	-0.511867252	0.149	0.594	0.000380799
HLA-DRB5	1.77E-08	-0.514624421	0.851	1	0.000386922
THBD	2.02E-08	0.809055288	0.473	0.154	0.000440845
DUSP1	2.07E-08	-0.410213695	1	1	0.000451298
BIN2	2.28E-08	-0.571911907	0.27	0.699	0.00049818
FCGR3A	2.34E-08	-0.404735939	0.878	0.986	0.000510277
HBB	2.50E-08	-0.818816136	0.095	0.483	0.000546258
APBB1IP	3.05E-08	-0.5658268	0.324	0.748	0.000666013
SCIMP	5.15E-08	-0.485007431	0.162	0.573	0.001123069
METTL7A	5.28E-08	-0.454285661	0.149	0.545	0.00115174
IFNGR1	5.38E-08	-0.542562736	0.514	0.874	0.001172934
LILRA2	6.01E-08	-0.568994966	0.459	0.811	0.00131121
CDKN1A	6.84E-08	0.651410114	0.878	0.769	0.00149167
LRG1	7.35E-08	0.632726507	0.392	0.105	0.00160265
PELI1	7.65E-08	0.723314847	0.689	0.448	0.001669173

TKT	8.12E-08	-0.504105307	0.716	0.965	0.001771402
COTL1	8.71E-08	-0.324882365	0.973	1	0.001899192
RHOB	9.26E-08	-0.634973538	0.797	0.979	0.002018439
MEF2C	9.88E-08	-0.523866326	0.203	0.615	0.002153748
PPP1CB	1.01E-07	0.731078336	0.622	0.371	0.002211785
RPL36A	1.28E-07	0.410813614	0.919	0.951	0.002791967
PDLIM2	1.39E-07	-0.4711727	0.068	0.427	0.003022271
RGS19	1.81E-07	-0.451288941	0.351	0.755	0.003940081
RPL39	1.99E-07	0.302614264	1	0.993	0.004343249
C15orf48	2.01E-07	1.242955545	0.338	0.077	0.00439136
CSK	2.29E-07	-0.501399049	0.284	0.699	0.005004917
SAMSN1	2.31E-07	0.562368681	0.932	0.839	0.005034503
LIMD2	2.49E-07	-0.437633176	0.5	0.853	0.005432786
UPP1	2.92E-07	0.674567401	0.77	0.629	0.006372608
HSPB11	3.01E-07	-0.408152638	0.041	0.371	0.006574414
VAMP8	3.51E-07	-0.425639215	0.703	0.944	0.007647516
OTUB1	3.65E-07	-0.449445392	0.203	0.608	0.007958412
IGBP1	3.78E-07	-0.418115294	0.162	0.545	0.008244637
PRAM1	4.13E-07	-0.391385519	0.095	0.455	0.008998288
PVRL2	4.46E-07	0.639844137	0.419	0.14	0.009725816
GPBAR1	4.56E-07	-0.453843022	0.122	0.49	0.00995084
STX10	4.97E-07	-0.457102517	0.243	0.615	0.01083859
LIMK2	5.08E-07	0.497438744	0.338	0.084	0.011081382
FCGR1A	5.96E-07	0.608765989	0.649	0.413	0.012988628
ADRB2	6.05E-07	-0.372445023	0	0.28	0.013184631
CDKN1B	8.01E-07	-0.432725608	0.284	0.678	0.017473445
SULT1B1	8.10E-07	-0.433008578	0.095	0.441	0.017660729
RPL7A	8.18E-07	-0.250527742	1	1	0.017841516
HSP90AB1	8.38E-07	0.864837351	0.919	0.874	0.018272037
C10orf54	8.42E-07	-0.412698654	0.73	0.937	0.018353378
GIMAP7	8.55E-07	-0.503785276	0.081	0.413	0.018636801
ATPIF1	1.20E-06	-0.366046504	0.216	0.629	0.026131923
PABPC1	1.35E-06	-0.309379858	0.986	0.986	0.029509558
MRPS35	1.35E-06	-0.440036318	0.108	0.448	0.029535549
PTPRC	1.50E-06	-0.386109699	0.838	1	0.03264317
MAP1LC3A	1.53E-06	0.388828907	0.176	0.007	0.033410413
FLI1	1.55E-06	-0.384545151	0.189	0.545	0.033858943
MALAT1	1.70E-06	0.430294484	0.986	1	0.03707363
LDHA	1.86E-06	0.412069203	0.878	0.839	0.040661705
IFI30	2.11E-06	0.661911401	0.797	0.804	0.045908238

Table 6: List of DEGs of mononuclear phagocyte clusters in cold preservation stage (EP versus PP)

List of differentially expressed genes of C1QC_KC in cold preservation stage (EP versus PP)

gene	p_val	avg_logFC	pct.1	pct.2	p_val_adj
HSP90AA1	2.03E-28	0.483146606	0.943	0.874	4.43E-24
HSPA1B	1.62E-26	0.466592419	0.816	0.534	3.53E-22
HSPA1A	2.75E-23	0.3557401	0.908	0.748	5.99E-19
MT-ATP8	1.42E-19	-0.409727338	0.967	0.988	3.09E-15
IGLV2-14	7.25E-19	0.387113879	0.307	0.096	1.58E-14
IGLV2-23	1.91E-18	0.295491767	0.162	0.017	4.16E-14
C1QC	5.60E-17	-0.255719585	0.971	0.964	1.22E-12
HSPH1	9.65E-15	0.452956781	0.547	0.355	2.10E-10
GPX1	3.19E-14	0.257773726	0.969	0.925	6.96E-10
RGS1	4.43E-10	0.362141987	0.602	0.43	9.65E-06
MT2A	9.04E-09	0.537464027	0.818	0.755	0.000197196
IGKV2-24	4.02E-08	0.546126276	0.062	0.171	0.000876957
TNF	4.95E-08	-0.530089829	0.225	0.368	0.00108016
MAF	6.33E-08	0.260658652	0.789	0.662	0.001379688
ADM	6.98E-08	-0.379747894	0.35	0.491	0.001523083
VSIG4	7.05E-08	-0.25567875	0.859	0.856	0.001537931
RSRP1	4.77E-07	-0.306944573	0.531	0.629	0.010391656
FOLR2	1.61E-06	-0.251706387	0.805	0.862	0.035135719
DEFA3	1.75E-06	-0.422008204	0.316	0.449	0.038143692

List of differentially expressed genes of VCAN_TMo in cold preservation stage (EP versus PP)

gene	p_val	avg_logFC	pct.1	pct.2	p_val_adj
HSP90AA1	1.77E-17	0.626540073	0.886	0.807	3.86E-13
MT1E	4.36E-15	1.059251622	0.276	0.053	9.52E-11
HSPA1B	5.75E-15	0.537083216	0.566	0.27	1.25E-10
NEAT1	3.71E-14	0.33534573	1	0.997	8.09E-10
MT1X	1.10E-11	1.365030606	0.518	0.306	2.41E-07
MT2A	1.17E-10	1.26720749	0.779	0.677	2.55E-06
KLF6	2.52E-10	0.39943313	0.93	0.875	5.49E-06
RHOB	2.52E-10	0.416761513	0.857	0.721	5.49E-06
IGKV2-24	1.54E-08	-0.32431382	0.04	0.193	0.000336168
MT1G	1.90E-08	1.284259824	0.162	0.033	0.000414592
JUN	1.92E-08	0.44269514	0.688	0.528	0.000419077
IGLV2-23	4.94E-08	0.30667244	0.14	0.024	0.001077159
GPX1	8.27E-07	0.267629066	0.919	0.923	0.018032005
HSPA1A	9.21E-07	0.419407285	0.669	0.579	0.020086312
IGLV2-14	1.49E-06	0.326433667	0.268	0.122	0.032475784
HSPH1	1.61E-06	0.388925484	0.379	0.214	0.035115497

List of differentially expressed genes of S100A8_TMo in cold preservation stage (EP versus PP)

gene	p_val	avg_logFC	pct.1	pct.2	p_val_adj
MT1X	1.91E-08	0.837855545	0.453	0.226	0.000415617
JUN	6.61E-08	0.685201364	0.575	0.328	0.001440706
MT-ND3	7.97E-08	0.289453663	0.972	0.946	0.001737612
MT2A	5.39E-07	0.831379914	0.597	0.414	0.011745676
MT-ATP6	6.24E-07	0.337488221	0.961	0.952	0.013613524
NEAT1	8.18E-07	0.305036912	0.956	0.946	0.017841712

List of differentially expressed genes of IL1B_KC in cold preservation stage (EP versus PP)

gene	p_val	avg_logFC	pct.1	pct.2	p_val_adj
NEAT1	3.12E-10	0.439661833	1	0.986	6.80E-06
HSP90AA1	3.80E-10	0.62608012	0.942	0.902	8.29E-06
HSPA1B	1.85E-09	0.48504073	0.783	0.434	4.03E-05
IGLV2-14	2.18E-08	0.647393245	0.342	0.077	0.000475729
HSPA1A	1.15E-06	0.282257263	0.917	0.783	0.025068005
IGLV2-23	1.58E-06	0.284329332	0.183	0.014	0.034477496

able 7. List of markers information for bulk RNA sequencing data from 14 EP-PR pairs of donor liver sample

ID	log2FoldChange	pvalue	padj	gene_name
ENSG00000227300	9.732648439	6.24E-18	4.05E-15	KRT16P2
ENSG00000108342	9.575885426	2.14E-23	3.47E-20	CSF3
ENSG00000136244	6.933343394	1.06E-18	8.07E-16	IL6
ENSG00000267607	6.901879081	2.06E-18	1.53E-15	AC011511.5
ENSG00000126583	6.741541442	6.71E-13	1.50E-10	PRKCG
ENSG00000204614	6.598034068	6.39E-08	4.22E-06	TRIM40
ENSG00000196611	6.535225305	1.04E-08	9.00E-07	MMP1
ENSG00000135625	6.370841279	1.05E-14	3.77E-12	EGR4
ENSG00000168334	6.304738312	5.41E-14	1.56E-11	XIRP1
ENSG00000175592	6.08766392	6.95E-27	3.61E-23	FOSL1
ENSG00000197632	6.021238854	3.00E-11	4.66E-09	SERPINB2
ENSG00000119508	5.94494471	7.40E-26	2.13E-22	NR4A3
ENSG00000173110	5.838250319	4.60E-26	1.71E-22	HSPA6
ENSG00000128342	5.810316714	2.15E-17	1.27E-14	LIF
ENSG00000121742	5.77201028	6.22E-11	8.87E-09	GJB6
ENSG00000198576	5.770874933	5.49E-20	5.48E-17	ARC
ENSG00000240457	5.763222465	3.56E-09	3.40E-07	RN7SL472P
ENSG00000115009	5.645891491	4.71E-15	1.82E-12	CCL20
ENSG00000100341	5.583686824	5.20E-07	2.63E-05	PNPLA5
ENSG00000204388	5.536284961	2.07E-32	2.69E-28	HSPA1B
ENSG00000170961	5.37593242	4.24E-15	1.69E-12	HAS2
ENSG00000169429	5.354942353	8.01E-18	4.95E-15	CXCL8
ENSG00000188886	5.298981141	4.74E-12	8.99E-10	ASTL
ENSG00000179148	5.233843526	5.84E-10	6.61E-08	ALOXE3
ENSG00000161270	5.135854858	8.62E-07	4.12E-05	NPHS1
ENSG00000108688	5.124242909	1.23E-07	7.38E-06	CCL7
ENSG00000125740	5.107727442	2.49E-12	4.93E-10	FOSB
ENSG00000105509	5.072848935	2.43E-19	2.10E-16	HAS1
ENSG00000187658	5.052136358	4.69E-07	2.41E-05	C5orf52
ENSG00000267195	5.049603396	3.30E-14	1.02E-11	MIR212
ENSG00000204389	5.028372576	1.00E-32	2.61E-28	HSPA1A
ENSG00000123610	5.011379554	3.38E-15	1.37E-12	TNFAIP6
ENSG00000073756	4.96058298	1.04E-23	1.79E-20	PTGS2
ENSG00000207980	4.936636559	5.18E-08	3.58E-06	MIR23A
ENSG00000223086	4.838930764	1.63E-07	9.46E-06	RNA5SP155
ENSG00000268812	4.824736249	2.81E-08	2.16E-06	AC004264.1
ENSG00000115008	4.766861284	2.64E-09	2.60E-07	IL1A
ENSG00000166670	4.747542003	1.38E-06	6.17E-05	MMP10
ENSG00000259285	4.710169109	1.37E-09	1.44E-07	AC025431.1
ENSG00000158859	4.701448052	7.43E-17	4.01E-14	ADAMTS4
ENSG00000173391	4.698885047	1.47E-12	3.09E-10	OLR1
ENSG00000179388	4.659931863	8.47E-20	7.85E-17	EGR3
ENSG00000185897	4.637074771	2.61E-14	8.47E-12	FFAR3
ENSG00000124882	4.621378475	5.11E-11	7.49E-09	EREG
ENSG00000224959	4.609414837	1.22E-08	1.04E-06	AC017002.1
ENSG00000069482	4.606064334	3.01E-06	0.000123077	GAL
ENSG00000218809	4.591291265	7.85E-06	0.000281475	AL391903.1
ENSG00000148677	4.543175008	8.80E-08	5.55E-06	ANKRD1
ENSG00000268734	4.530656994	6.35E-14	1.73E-11	AC245128.3
ENSG00000109321	4.517174592	7.42E-09	6.54E-07	AREG
ENSG00000122877	4.458699826	8.39E-22	1.04E-18	EGR2
ENSG00000102794	4.42757525	0.000342595	0.006198724	ACOD1
ENSG00000179674	4.394772889	4.72E-06	0.000181941	ARL14
ENSG00000182782	4.387370113	5.64E-15	2.15E-12	HCAR2
ENSG00000049249	4.378555452	1.61E-15	6.97E-13	TNFRSF9
ENSG00000255434	4.36033708	3.65E-06	0.000145882	AP001922.6
ENSG00000149968	4.350079128	0.002192651	0.026001157	MMP3
ENSG00000099985	4.290880608	7.25E-13	1.57E-10	OSM
ENSG00000189410	4.274937649	3.87E-06	0.00015312	SH2D5
ENSG00000140403	4.263114819	3.42E-28	2.22E-24	DNAJA4
ENSG00000230928	4.262409941	8.68E-07	4.13E-05	AL139241.1
ENSG00000182950	4.258722983	3.20E-14	9.99E-12	ODF3L1
ENSG00000125538	4.246658318	4.33E-14	1.26E-11	IL1B
ENSG00000176887	4.236633224	0.0001242	0.002782815	SOX11
ENSG00000013588	4.231454379	3.45E-14	1.04E-11	GPRC5A
ENSG00000261257	4.216430933	0.001011376	0.014529984	AP000821.1
ENSG00000175793	4.215707396	3.20E-08	2.38E-06	SFN
ENSG00000162772	4.199053375	1.45E-26	6.29E-23	ATF3

ENSG00000198535	4.187588477	3.07E-08	2.31E-06	C2CD4A
ENSG00000205177	4.186655619	1.25E-11	2.19E-09	C11orf91
ENSG00000166527	4.18232652	1.91E-09	1.93E-07	CLEC4D
ENSG00000260467	4.167954377	1.98E-06	8.48E-05	AC018552.2
ENSG00000124731	4.162855928	8.46E-16	3.85E-13	TREM1
ENSG00000196361	4.146283205	5.16E-06	0.000196482	ELAVL3
ENSG00000173237	4.143997587	8.49E-05	0.002027562	C11orf86
ENSG00000204421	4.126492756	1.39E-07	8.26E-06	LY6G6C
ENSG00000186431	4.124692194	5.75E-14	1.62E-11	FCAR
ENSG00000102802	4.107394396	1.13E-10	1.53E-08	MEDAG
ENSG00000110680	4.088190691	3.20E-11	4.91E-09	CALCA
ENSG00000275215	4.084897459	7.29E-08	4.68E-06	RNA5-8SN4
ENSG00000011422	4.080504003	3.89E-24	7.34E-21	PLAUR
ENSG00000226380	4.071901689	2.53E-20	2.62E-17	AC016831.1
ENSG00000151929	4.058579252	9.27E-25	2.41E-21	BAG3
ENSG00000261026	4.048620267	9.53E-08	5.96E-06	AC105046.1
ENSG00000079393	4.045137473	2.69E-06	0.000111242	DUSP13
ENSG00000185022	4.021935963	2.18E-18	1.57E-15	MAFF
ENSG00000232618	4.016969315	4.48E-07	2.33E-05	AL355304.1
ENSG00000253295	3.993650567	2.31E-05	0.000708782	AC022217.2
ENSG00000100985	3.983678538	1.98E-14	6.58E-12	MMP9
ENSG00000267200	3.981253257	0.000712793	0.010975743	MIR132
ENSG00000108691	3.972642582	1.84E-16	9.36E-14	CCL2
ENSG00000218565	3.969820176	2.52E-07	1.40E-05	AL592429.1
ENSG00000229672	3.961270804	0.000576617	0.009326501	AL450322.2
ENSG00000126251	3.946659684	3.26E-06	0.00013206	GPR42
ENSG00000279430	3.935333908	2.94E-05	0.000847461	AL590560.3
ENSG00000232530	3.920618432	0.000176306	0.003704006	LIF-AS1
ENSG00000255398	3.912581497	9.79E-11	1.34E-08	HCAR3
ENSG00000132002	3.912239499	7.07E-30	6.11E-26	DNAJB1
ENSG00000261618	3.911293247	7.61E-11	1.07E-08	AC083837.1
ENSG00000050730	3.906504156	6.69E-09	5.98E-07	TNIP3
ENSG00000131459	3.885836211	5.44E-11	7.84E-09	GFPT2
ENSG00000115844	3.88380021	6.45E-05	0.001617268	DLX2
ENSG00000188581	3.868034868	0.00062925	0.009991751	KRTAP1-1
ENSG00000120694	3.862909141	7.11E-22	9.22E-19	HSPH1
ENSG00000159339	3.833110584	3.06E-12	5.90E-10	PADI4
ENSG00000248323	3.826616714	2.13E-12	4.32E-10	LUCAT1
ENSG00000223414	3.816973629	4.36E-07	2.28E-05	LINC00473
ENSG00000139572	3.816281197	2.27E-10	2.82E-08	GPR84
ENSG00000204020	3.808525887	1.48E-06	6.57E-05	LIPN
ENSG00000170827	3.805438531	1.85E-06	7.99E-05	CELP
ENSG00000140379	3.798129501	2.89E-13	7.09E-11	BCL2A1
ENSG00000123358	3.793383448	3.29E-15	1.35E-12	NR4A1
ENSG00000188056	3.783796484	3.78E-05	0.001042744	TREML4
ENSG00000196979	3.774303852	9.66E-06	0.000334051	AL360004.1
ENSG00000241158	3.756774518	5.51E-10	6.33E-08	ADAMTS9-AS1
ENSG00000112149	3.750483456	2.39E-24	5.16E-21	CD83
ENSG00000239948	3.749696775	3.70E-05	0.001023665	RN7SL368P
ENSG00000137801	3.73915792	7.23E-15	2.64E-12	THBS1
ENSG00000134531	3.710289504	8.98E-16	4.02E-13	EMP1
ENSG00000213386	3.708737185	8.74E-05	0.002078157	AC022217.1
ENSG00000172548	3.707171563	7.46E-07	3.65E-05	NIPAL4
ENSG00000138135	3.674706344	7.58E-11	1.07E-08	CH25H
ENSG00000169194	3.667652821	0.000210011	0.004277029	IL13
ENSG00000207736	3.660897659	0.000228353	0.004571646	MIR657
ENSG00000282855	3.656857731	1.95E-06	8.40E-05	AC093591.3
ENSG00000237330	3.656237382	8.14E-06	0.000288379	RNF223
ENSG00000251230	3.647071781	4.94E-11	7.28E-09	MIR3945HG
ENSG00000128383	3.645429296	1.04E-12	2.22E-10	APOBEC3A
ENSG00000231412	3.634383862	1.16E-05	0.000391962	AC005392.2
ENSG00000240163	3.628317098	0.00050921	0.008420632	AC087385.1
ENSG00000135824	3.61876429	2.87E-05	0.000834787	RGS8
ENSG00000233791	3.617842931	1.23E-06	5.62E-05	LINC01136
ENSG00000261429	3.602434616	5.30E-05	0.001379089	DPPA2P4
ENSG00000217527	3.585586813	6.20E-05	0.001572939	RPS16P5
ENSG00000276176	3.585450352	0.00026566	0.005102592	MIR6090
ENSG00000111537	3.582613196	1.28E-07	7.68E-06	IFNG
ENSG00000176597	3.572688393	6.50E-18	4.11E-15	B3GNT5
ENSG00000261488	3.571692929	0.000102973	0.002389747	AC128688.2

ENSG00000260859	3.569920762	3.63E-05	0.001011526	AC025280.1
ENSG00000162631	3.557667378	0.001163305	0.016192655	NTNG1
ENSG00000182885	3.552164764	8.50E-12	1.54E-09	ADGRG3
ENSG00000255326	3.542444805	5.52E-06	0.000207889	AP001922.5
ENSG00000163421	3.537016834	2.22E-10	2.77E-08	PROK2
ENSG00000006128	3.525526607	2.41E-05	0.00073371	TAC1
ENSG00000126262	3.510421947	1.40E-10	1.83E-08	FFAR2
ENSG00000226652	3.505573757	2.34E-05	0.000715854	PSMD10P2
ENSG00000184106	3.503799651	4.64E-07	2.38E-05	TREML3P
ENSG00000163734	3.50195495	1.49E-14	5.09E-12	CXCL3
ENSG00000167210	3.493568784	5.89E-09	5.32E-07	LOXHD1
ENSG00000284387	3.471608863	0.000191614	0.003960925	MIR24-2
ENSG00000276462	3.469207112	0.00142244	0.01880955	BX255923.2
ENSG00000207808	3.465532294	4.21E-05	0.001133514	MIR27A
ENSG00000248690	3.457674285	0.000283837	0.005355949	HAS2-AS1
ENSG00000279770	3.443389689	0.000209615	0.004272324	LINC00552
ENSG00000234436	3.402793651	0.000487625	0.008136284	AC245884.2
ENSG00000238279	3.387973605	5.13E-06	0.000195491	BX470102.1
ENSG00000070985	3.387769278	8.12E-08	5.15E-06	TRPM5
ENSG00000172602	3.383309177	7.32E-14	1.95E-11	RND1
ENSG00000255443	3.37962482	4.40E-06	0.000170714	CD44-AS1
ENSG00000225173	3.378922527	0.003098425	0.03328088	AL662890.2
ENSG00000186354	3.375117315	2.86E-10	3.45E-08	C9orf47
ENSG00000105605	3.371089002	0.000885199	0.01308683	CACNG7
ENSG00000279691	3.366307526	9.64E-06	0.000334014	AC113410.3
ENSG00000005001	3.366113534	2.33E-08	1.86E-06	PRSS22
ENSG00000177535	3.365345488	0.000653703	0.010291858	OR2B11
ENSG00000169908	3.362629615	6.95E-13	1.53E-10	TM4SF1
ENSG00000270069	3.358635463	3.75E-11	5.69E-09	MIR222HG
ENSG00000241155	3.354662495	1.26E-07	7.56E-06	ARHGAP31-AS1
ENSG00000261227	3.349090473	0.000210281	0.004279172	AC140912.1
ENSG00000163497	3.34602742	0.001097127	0.015504392	FEV
ENSG00000115602	3.333231967	3.79E-09	3.59E-07	IL1RL1
ENSG00000204241	3.333122542	3.75E-06	0.000149088	AP000911.1
ENSG00000198454	3.331238027	0.004161222	0.041161671	PRR31
ENSG00000116741	3.328763904	9.88E-19	7.77E-16	RGS2
ENSG00000182759	3.323670323	5.91E-08	3.97E-06	MAFA
ENSG00000104941	3.321294119	0.002455039	0.028197629	RSPH6A
ENSG00000277452	3.319503969	1.08E-07	6.64E-06	RN7SL473P
ENSG00000272235	3.316882091	0.000508023	0.008406357	AL590438.1
ENSG00000123342	3.308103578	7.15E-13	1.56E-10	MMP19
ENSG00000184545	3.297557182	4.56E-12	8.70E-10	DUSP8
ENSG00000275166	3.295944196	3.51E-05	0.000982216	MIR6814
ENSG00000112195	3.286163784	3.51E-09	3.36E-07	TREML2
ENSG00000136997	3.282953872	2.25E-19	2.01E-16	MYC
ENSG00000207870	3.272571681	0.001511558	0.019580077	MIR221
ENSG00000164400	3.266585265	0.000274886	0.005232725	CSF2
ENSG00000104970	3.266404278	0.000203937	0.00418619	KIR3DX1
ENSG00000214856	3.263775615	0.003481931	0.036252879	KRT16P1
ENSG00000276070	3.259312258	7.42E-20	7.13E-17	CCL4L2
ENSG00000113070	3.257698138	3.71E-16	1.85E-13	HBEGF
ENSG00000231302	3.253951625	0.000300003	0.005595881	RPL36P2
ENSG00000239218	3.252045094	6.34E-05	0.001595997	RPS20P22
ENSG00000235448	3.251092077	0.000306046	0.005696325	LURAP1L-AS1
ENSG00000143217	3.250924423	1.70E-05	0.000541748	NECTIN4
ENSG00000163221	3.246448984	7.55E-10	8.34E-08	S100A12
ENSG00000227560	3.244647451	0.000212998	0.004331074	RPS15AP30
ENSG00000203804	3.243674075	6.30E-15	2.34E-12	ADAMTSL4-AS1
ENSG00000175505	3.235177584	6.78E-13	1.50E-10	CLCF1
ENSG00000125657	3.233826013	2.83E-11	4.45E-09	TNFSF9
ENSG00000260166	3.223927731	0.000447001	0.007645271	AC134312.3
ENSG00000236278	3.21464322	0.000290943	0.005450401	PEBP1P3
ENSG00000163464	3.206734895	7.03E-10	7.79E-08	CXCR1
ENSG00000120738	3.205658067	9.81E-14	2.50E-11	EGR1
ENSG00000278493	3.205129055	1.01E-05	0.000346715	AC039056.2
ENSG00000105376	3.20439183	3.93E-09	3.70E-07	ICAM5
ENSG00000118160	3.202159989	2.27E-08	1.82E-06	SLC8A2
ENSG00000106366	3.200282307	2.06E-06	8.77E-05	SERPINE1
ENSG00000233559	3.200098926	8.29E-11	1.16E-08	LINC00513
ENSG00000132510	3.199059514	9.27E-23	1.34E-19	KDM6B

ENSG00000135477	3.198253826	3.45E-06	0.000139205	KRT87P
ENSG00000279806	3.192502287	5.51E-05	0.001429018	AC018629.1
ENSG00000118503	3.191985274	8.69E-21	9.80E-18	TNFAIP3
ENSG00000124762	3.190238237	6.48E-23	9.89E-20	CDKN1A
ENSG00000269902	3.189100382	0.003068137	0.033086406	AC234772.3
ENSG00000122861	3.18734295	4.29E-11	6.40E-09	PLAU
ENSG00000260078	3.182986731	9.39E-06	0.000328032	AC007342.3
ENSG00000267943	3.181403734	0.001316592	0.017764061	AC010328.1
ENSG00000223336	3.178173908	0.004962334	0.046598884	RNU2-6P
ENSG00000128564	3.176193753	4.72E-05	0.001244856	VGF
ENSG00000236525	3.164402674	0.000120935	0.002719563	AC007278.2
ENSG00000264741	3.162368598	0.000225457	0.004534648	MIR4505
ENSG00000213144	3.159326466	8.09E-08	5.14E-06	AC084880.1
ENSG00000137270	3.158611035	0.002062529	0.024855729	GCM1
ENSG00000201581	3.15290403	0.002613222	0.02941547	RN7SKP78
ENSG00000124610	3.148847883	0.005466702	0.049890625	HIST1H1A
ENSG00000181652	3.148658705	2.54E-10	3.10E-08	ATG9B
ENSG00000087074	3.144542256	2.33E-17	1.35E-14	PPP1R15A
ENSG00000188089	3.143705788	0.000462422	0.007846953	PLA2G4E
ENSG00000143816	3.140786409	1.58E-08	1.30E-06	WNT9A
ENSG00000164023	3.140361529	1.84E-12	3.82E-10	SGMS2
ENSG00000244124	3.137120367	1.52E-05	0.000490839	ATP1B3-AS1
ENSG00000274213	3.131092832	6.66E-12	1.23E-09	AC015912.3
ENSG00000176170	3.126925347	1.40E-10	1.83E-08	SPHK1
ENSG00000102554	3.126312744	2.39E-10	2.94E-08	KLF5
ENSG00000131355	3.124183075	7.33E-07	3.60E-05	ADGRE3
ENSG00000279637	3.121668052	0.0002913	0.005453154	AC018445.3
ENSG00000224821	3.121528522	7.06E-05	0.001735617	COL4A2-AS2
ENSG00000120217	3.113899429	9.73E-10	1.04E-07	CD274
ENSG00000170893	3.106926389	0.002670426	0.029916613	TRH
ENSG00000270210	3.105228069	7.58E-06	0.000272624	AC104695.2
ENSG00000114529	3.103509755	1.14E-07	6.93E-06	C3orf52
ENSG00000258881	3.103475668	1.21E-05	0.000404097	AC007040.2
ENSG00000059804	3.102917033	1.10E-15	4.82E-13	SLC2A3
ENSG00000152049	3.09912915	3.20E-09	3.10E-07	KCNE4
ENSG00000140932	3.097568424	1.41E-08	1.18E-06	CMTM2
ENSG00000142627	3.094005551	6.49E-14	1.75E-11	EPHA2
ENSG00000130720	3.092828143	0.000980278	0.014156996	FIBCD1
ENSG00000134668	3.09206107	1.32E-08	1.10E-06	SPOCD1
ENSG00000239503	3.069915283	3.37E-05	0.000948162	MARK2P8
ENSG00000145040	3.069139685	0.001330655	0.017922037	UCN2
ENSG00000222047	3.065405277	6.79E-05	0.001681803	C10orf55
ENSG00000090339	3.058959382	3.04E-13	7.37E-11	ICAM1
ENSG00000230495	3.052650117	0.001112488	0.01566176	AL132765.1
ENSG00000228719	3.038614903	0.001791207	0.02226864	AL022313.2
ENSG00000146592	3.032989029	5.56E-16	2.67E-13	CREB5
ENSG00000274963	3.031503275	9.52E-06	0.000331092	RN7SL600P
ENSG00000168398	3.030446822	5.57E-08	3.79E-06	BDKRB2
ENSG00000261592	3.02900485	8.87E-08	5.57E-06	AC010531.3
ENSG00000257446	3.019397743	3.62E-06	0.000145078	ZNF878
ENSG00000183019	3.007095526	9.38E-11	1.30E-08	MCEMP1
ENSG00000162040	3.005414825	0.000226678	0.004552167	HS3ST6
ENSG00000273972	2.995336646	0.000321793	0.005913055	AC068722.2
ENSG00000273980	2.981822207	0.001214723	0.016728876	AL592071.1
ENSG00000257453	2.973544824	0.000139547	0.003086685	AC011611.3
ENSG00000254281	2.970995139	0.000998678	0.014395395	AP003354.2
ENSG00000164283	2.969272356	5.88E-05	0.0015008	ESM1
ENSG00000270607	2.965319002	4.33E-06	0.000168597	AC009549.1
ENSG00000258820	2.962345373	0.001772286	0.022128839	AF111167.1
ENSG00000173846	2.954883488	4.47E-18	2.97E-15	PLK3
ENSG00000255801	2.951394923	0.005230065	0.0483218	AC092746.1
ENSG00000136689	2.949954907	2.99E-15	1.25E-12	IL1RN
ENSG00000262477	2.948357537	0.001767684	0.022092649	AC021224.1
ENSG00000183625	2.943076341	0.001193702	0.016535933	CCR3
ENSG00000270640	2.941065486	2.61E-05	0.000775284	AC104695.3
ENSG00000163273	2.937321737	0.000207781	0.004244943	NPPC
ENSG00000131016	2.932335379	6.30E-15	2.34E-12	AKAP12
ENSG00000257335	2.925425132	5.44E-08	3.71E-06	MGAM
ENSG00000237453	2.925347874	6.60E-05	0.00164909	AC096536.2
ENSG00000231956	2.924236636	0.001737437	0.021788084	HNRNPA1P9

ENSG00000259630	2.922883868	1.31E-06	5.90E-05	AC104046.1
ENSG00000273199	2.922540578	2.13E-08	1.72E-06	AP000692.2
ENSG00000214650	2.91330812	0.001406131	0.018657311	AC073592.1
ENSG00000104722	2.911457838	0.000875095	0.012989254	NEFM
ENSG00000233930	2.909774256	1.58E-09	1.63E-07	KRTAP5-AS1
ENSG00000275238	2.903174269	0.000938642	0.013674348	MIR4734
ENSG00000139289	2.902103799	1.49E-16	7.73E-14	PHLDA1
ENSG00000173334	2.901284299	6.16E-26	2.00E-22	TRIB1
ENSG00000243870	2.899917028	0.000534545	0.008778048	RN7SL236P
ENSG00000267275	2.899590658	0.002271032	0.026686679	AC020911.2
ENSG00000280890	2.897815549	0.000429961	0.007397726	AC073324.2
ENSG00000250659	2.887607122	4.46E-05	0.001185895	AP001363.1
ENSG00000277437	2.885668823	0.000188267	0.00391723	MIR3687-1
ENSG00000274552	2.88335507	0.000244255	0.004793821	MIR6889
ENSG00000006327	2.881406391	9.28E-10	1.00E-07	TNFRSF12A
ENSG00000273172	2.879564041	0.00336731	0.03535744	LINC02091
ENSG00000054356	2.878954339	0.001841889	0.02281129	PTPRN
ENSG00000138166	2.868644213	2.11E-20	2.29E-17	DUSP5
ENSG00000204044	2.866650125	0.000564045	0.009163872	SLC12A5-AS1
ENSG00000197106	2.865751973	0.000452865	0.007720127	SLC6A17
ENSG00000067082	2.864638839	3.96E-24	7.34E-21	KLF6
ENSG00000205502	2.862452302	5.17E-12	9.66E-10	C2CD4B
ENSG00000181634	2.86051663	7.60E-07	3.70E-05	TNFSF15
ENSG00000226012	2.855260009	0.004550063	0.043821805	AP001434.1
ENSG00000177822	2.852547565	0.004304247	0.042174464	AC098864.1
ENSG00000133055	2.852083398	0.003582227	0.036882728	MYBPH
ENSG00000160271	2.850624235	7.37E-14	1.95E-11	RALGDS
ENSG00000162616	2.848651258	1.49E-14	5.09E-12	DNAJB4
ENSG00000175352	2.847492136	2.94E-07	1.61E-05	NRIP3
ENSG00000248767	2.844402532	0.002031641	0.024597737	AC187653.1
ENSG00000206172	2.843840428	0.00027457	0.005232725	HBA1
ENSG00000184557	2.840364253	1.96E-12	4.00E-10	SOCS3
ENSG00000180316	2.837226143	4.63E-05	0.001225091	PNPLA1
ENSG00000170956	2.834587311	1.31E-10	1.75E-08	CEACAM3
ENSG00000279463	2.829822284	0.002826979	0.031198982	AL159972.1
ENSG00000178053	2.825149928	2.59E-12	5.10E-10	MLF1
ENSG00000138892	2.820653059	0.003008719	0.032690214	TTLL8
ENSG00000229951	2.819020652	2.80E-08	2.16E-06	FLJ31356
ENSG00000259883	2.814800905	1.30E-06	5.84E-05	EHD4-AS1
ENSG00000166592	2.81295893	2.19E-11	3.55E-09	RRAD
ENSG00000162975	2.812914906	3.31E-05	0.000933366	KCNF1
ENSG00000196358	2.811422671	2.09E-10	2.62E-08	NTNG2
ENSG00000181649	2.811152359	1.61E-07	9.37E-06	PHLDA2
ENSG00000163739	2.804437843	9.40E-09	8.21E-07	CXCL1
ENSG00000269959	2.803942761	0.004666851	0.044577365	SPACA6P-AS
ENSG00000275302	2.80003657	2.98E-18	2.09E-15	CCL4
ENSG00000258947	2.793928937	0.000207538	0.004243328	TUBB3
ENSG00000204936	2.78752264	2.97E-08	2.26E-06	CD177
ENSG00000228323	2.780383001	5.74E-07	2.86E-05	AC008440.1
ENSG00000267519	2.779456502	3.63E-14	1.08E-11	AC020916.1
ENSG00000099812	2.778183753	4.13E-05	0.00111805	MISP
ENSG00000250027	2.777640308	0.00048089	0.008060195	AC022272.1
ENSG00000151014	2.776768479	1.08E-16	5.74E-14	NOCT
ENSG00000163638	2.776051992	1.17E-07	7.08E-06	ADAMTS9
ENSG00000165997	2.773361587	1.94E-21	2.29E-18	ARL5B
ENSG00000177606	2.765354385	5.09E-13	1.17E-10	JUN
ENSG00000260910	2.762185327	8.96E-05	0.002116617	LINC00565
ENSG00000279035	2.758947951	0.003639896	0.037357888	AC022211.4
ENSG00000222041	2.756044758	1.89E-08	1.54E-06	CYTOR
ENSG00000103196	2.755572102	1.51E-08	1.25E-06	CRISPLD2
ENSG00000171049	2.75482806	5.38E-11	7.80E-09	FPR2
ENSG00000261888	2.750622363	5.73E-10	6.55E-08	AC144831.1
ENSG00000232456	2.749544318	0.003857001	0.038915711	AL355994.2
ENSG00000156030	2.747693995	8.26E-18	4.98E-15	ELMSAN1
ENSG00000250274	2.746923233	0.001788021	0.022261029	AC034199.1
ENSG00000059728	2.742645295	1.80E-24	4.24E-21	MXD1
ENSG00000181418	2.740976047	7.68E-05	0.001864013	DDN
ENSG00000075426	2.738407098	1.75E-13	4.37E-11	FOSL2
ENSG00000234604	2.736271258	3.73E-05	0.001029514	AL021068.2
ENSG00000163874	2.735332621	2.70E-15	1.15E-12	ZC3H12A

ENSG00000250796	2.731082978	0.001610908	0.020553971	AC112484.3
ENSG00000237136	2.723620702	0.005161106	0.047979239	C4orf51
ENSG00000188536	2.723151517	5.77E-05	0.00148198	HBA2
ENSG00000167612	2.71644743	0.001753071	0.021952203	ANKRD33
ENSG00000264635	2.714735968	1.07E-05	0.000365571	AP001020.2
ENSG00000142319	2.712871864	0.00236413	0.027605633	SLC6A3
ENSG00000229647	2.711354197	0.001013289	0.01454137	MYOSLID
ENSG00000167767	2.707232607	5.27E-06	0.000199443	KRT80
ENSG00000245293	2.700624334	5.30E-07	2.67E-05	AC096564.1
ENSG00000250415	2.698087503	0.004044312	0.040302437	AC022113.2
ENSG00000231274	2.695837886	1.13E-07	6.85E-06	SBK3
ENSG00000233214	2.694507595	0.000315107	0.005806657	AC002511.2
ENSG00000244953	2.692800809	1.66E-07	9.57E-06	AC087521.1
ENSG00000141682	2.690699575	4.71E-09	4.32E-07	PMAIP1
ENSG00000081181	2.688176646	7.07E-12	1.29E-09	ARG2
ENSG00000184389	2.685023484	0.000308301	0.005725976	A3GALT2
ENSG00000143333	2.68327786	1.65E-07	9.56E-06	RGS16
ENSG00000272256	2.683195282	0.000151311	0.003299081	AC044849.2
ENSG00000250068	2.679932385	0.002000595	0.024324006	AL445187.1
ENSG00000159871	2.678251319	1.30E-08	1.10E-06	LYPD5
ENSG00000165046	2.675600554	2.66E-12	5.18E-10	LETM2
ENSG00000232680	2.670379647	0.001479773	0.019303266	AC002511.1
ENSG00000228113	2.66986345	1.57E-05	0.000506478	AC003991.1
ENSG00000171621	2.669614011	5.89E-10	6.65E-08	SPSB1
ENSG00000170345	2.667867344	1.93E-07	1.09E-05	FOS
ENSG00000237803	2.659084563	0.003817473	0.038670516	LINC00211
ENSG00000261068	2.656730574	0.001561319	0.020104204	AL512274.1
ENSG00000180871	2.656221582	6.25E-08	4.16E-06	CXCR2
ENSG00000163661	2.654612879	1.10E-07	6.76E-06	PTX3
ENSG00000162711	2.653215965	4.51E-13	1.06E-10	NLRP3
ENSG00000188396	2.65119265	1.67E-12	3.50E-10	TCTEX1D4
ENSG00000100604	2.64168359	0.000812403	0.012198265	CHGA
ENSG00000204610	2.635485501	9.58E-10	1.03E-07	TRIM15
ENSG00000008438	2.63499388	1.22E-06	5.55E-05	PGLYRP1
ENSG00000276085	2.63300052	2.45E-11	3.89E-09	CCL3L3
ENSG00000236776	2.632600876	0.000207133	0.004239778	RPL21P23
ENSG00000275339	2.628917992	0.001124145	0.015774514	Z99129.1
ENSG00000172738	2.624329926	5.00E-09	4.54E-07	TMEM217
ENSG00000237989	2.623530953	1.82E-10	2.30E-08	LINC01679
ENSG00000167434	2.615442341	2.13E-05	0.000658082	CA4
ENSG00000235481	2.614273422	0.000752552	0.011458751	UBE2R2-AS1
ENSG00000087494	2.608598787	5.51E-05	0.001429018	PTHLH
ENSG00000251009	2.605301663	0.001787331	0.022261029	AC133961.1
ENSG00000144136	2.604236308	1.06E-14	3.77E-12	SLC20A1
ENSG00000275894	2.602403288	3.85E-14	1.13E-11	AL021578.1
ENSG00000229191	2.594242301	0.001784723	0.022241313	AL358473.1
ENSG00000218416	2.593177813	5.29E-07	2.66E-05	PP14571
ENSG00000229331	2.591464012	3.94E-05	0.001077156	GK-IT1
ENSG00000271824	2.590650401	0.00023948	0.004717957	AC009014.1
ENSG00000246130	2.58913558	1.07E-05	0.000365571	AC107959.2
ENSG00000160161	2.586036597	5.12E-08	3.55E-06	CILP2
ENSG00000266709	2.58505516	1.29E-07	7.69E-06	AC005224.4
ENSG00000213438	2.583996811	0.000526713	0.008687914	YBX2P1
ENSG00000224621	2.583036868	0.00026569	0.005102592	AL451042.1
ENSG00000260454	2.57950444	0.002242183	0.026416126	AL355607.2
ENSG00000164949	2.578332264	6.58E-09	5.91E-07	GEM
ENSG00000233538	2.568540716	0.004402105	0.042828936	AC017104.3
ENSG00000205716	2.556188693	4.63E-05	0.001225091	FAM183DP
ENSG00000130164	2.555705316	1.79E-14	6.05E-12	LDLR
ENSG00000130522	2.550121505	2.43E-12	4.85E-10	JUND
ENSG00000128965	2.548175076	5.94E-08	3.98E-06	CHAC1
ENSG00000169508	2.54368894	1.54E-10	1.98E-08	GPR183
ENSG00000269952	2.541804504	3.70E-05	0.001023665	AL117336.2
ENSG00000143546	2.537927724	2.56E-08	2.00E-06	S100A8
ENSG00000154734	2.535776628	2.99E-09	2.92E-07	ADAMTS1
ENSG00000164935	2.535764888	1.96E-05	0.000615105	DCSTAMP
ENSG00000129451	2.535308407	0.000114108	0.002594188	KLK10
ENSG00000163659	2.532625859	6.30E-22	8.60E-19	TIPARP
ENSG00000243415	2.526116968	3.79E-05	0.001044668	AC107021.1
ENSG00000178878	2.525490157	1.56E-07	9.12E-06	APOLD1

ENSG00000197019	2.522116417	7.01E-16	3.25E-13	SERTAD1
ENSG00000126259	2.519567549	0.001205687	0.016659064	KIRREL2
ENSG00000086967	2.516787136	0.000153037	0.003331119	MYBPC2
ENSG00000261253	2.513524185	1.21E-05	0.000404097	AC137932.2
ENSG00000274008	2.513427896	0.000371101	0.006567929	Metazoa_SRP
ENSG00000231507	2.512466168	0.000172396	0.003630678	LINC01353
ENSG00000204420	2.511484371	6.51E-08	4.28E-06	MPIG6B
ENSG00000258512	2.508982855	0.001923808	0.023533774	LINC00239
ENSG00000183421	2.504021546	7.64E-13	1.64E-10	RIPK4
ENSG00000234193	2.503643121	5.08E-05	0.001327761	AC013476.1
ENSG00000069849	2.5014324	1.25E-09	1.32E-07	ATP1B3
ENSG00000144550	2.498703599	3.63E-05	0.001011526	CPNE9
ENSG00000136327	2.497544485	0.00365362	0.03743055	NKX2-8
ENSG00000167874	2.495994724	4.55E-09	4.19E-07	TMEM88
ENSG00000279762	2.493191216	1.18E-05	0.000396142	AC005899.8
ENSG00000142871	2.490124962	1.37E-11	2.34E-09	CYR61
ENSG00000102760	2.485313246	6.19E-09	5.58E-07	RGCC
ENSG00000086544	2.482738587	9.70E-14	2.49E-11	ITPKC
ENSG00000236056	2.48095262	0.000232146	0.004601483	GAPDHP14
ENSG00000237399	2.478202994	1.01E-07	6.25E-06	PITRM1-AS1
ENSG00000187479	2.474714001	4.58E-07	2.36E-05	C11orf96
ENSG00000172965	2.471111602	3.95E-09	3.70E-07	MIR4435-2HG
ENSG00000115607	2.470114941	7.43E-07	3.65E-05	IL18RAP
ENSG00000183638	2.466142743	0.000184676	0.003845581	RP1L1
ENSG00000039987	2.464789246	0.002416092	0.027923351	BEST2
ENSG00000149798	2.464399102	3.45E-10	4.07E-08	CDC42EP2
ENSG00000159388	2.460015703	2.36E-11	3.79E-09	BTG2
ENSG00000232034	2.458771057	0.000876971	0.013009653	AC092168.2
ENSG00000166483	2.455743938	3.73E-17	2.10E-14	WEE1
ENSG00000210049	2.455683207	0.004425747	0.042991554	MT-TF
ENSG00000237513	2.45486038	6.58E-05	0.00164517	AC007384.1
ENSG00000267655	2.453126472	0.00115806	0.016145637	AC125437.1
ENSG00000125845	2.448122514	5.55E-12	1.03E-09	BMP2
ENSG00000237781	2.443879729	1.72E-10	2.20E-08	AL356356.1
ENSG00000178726	2.442372366	6.49E-10	7.26E-08	THBD
ENSG00000155307	2.441565029	2.05E-11	3.36E-09	SAMSN1
ENSG00000181773	2.440380123	1.28E-06	5.79E-05	GPR3
ENSG00000112137	2.439796833	6.26E-14	1.73E-11	PHACTR1
ENSG00000173535	2.435552242	1.42E-07	8.35E-06	TNFRSF10C
ENSG00000128254	2.434003434	0.000168311	0.003556181	C22orf24
ENSG00000120833	2.430780646	1.44E-10	1.87E-08	SOCS2
ENSG00000105825	2.429270912	2.85E-08	2.19E-06	TFPI2
ENSG00000227218	2.428100383	3.06E-07	1.67E-05	AL157935.1
ENSG00000140519	2.426724762	4.99E-09	4.54E-07	RHCG
ENSG00000153234	2.424764703	1.40E-11	2.36E-09	NR4A2
ENSG00000283525	2.424135748	0.005358569	0.049197955	AC005622.1
ENSG00000260727	2.421540875	1.37E-06	6.10E-05	SLC7A5P1
ENSG00000152661	2.418830968	2.33E-10	2.88E-08	GJA1
ENSG00000255092	2.415588801	0.002013248	0.024420637	AC010768.2
ENSG00000227959	2.413395903	3.85E-07	2.04E-05	AL451042.2
ENSG00000136826	2.412559101	8.67E-10	9.45E-08	KLF4
ENSG00000282508	2.41185328	2.55E-06	0.000106338	LINC01002
ENSG00000196754	2.41147566	0.002209401	0.026128135	S100A2
ENSG00000281491	2.408864856	0.000739548	0.011320531	DNAJB5-AS1
ENSG00000238160	2.405888034	0.001033868	0.014752095	AC116366.2
ENSG00000267909	2.403167753	0.002947552	0.032214489	CCDC177
ENSG00000240023	2.400505397	0.004218205	0.041551077	AL133163.1
ENSG00000279622	2.400252348	0.00376958	0.038316216	AC025280.2
ENSG00000207730	2.399798159	0.000418053	0.007240851	MIR200B
ENSG00000129990	2.398949222	0.001741918	0.021833726	SYT5
ENSG00000267149	2.39817989	0.000318106	0.005853597	AC011476.2
ENSG00000121797	2.396921966	1.51E-11	2.52E-09	CCRL2
ENSG00000196428	2.393725064	6.35E-17	3.51E-14	TSC22D2
ENSG00000272986	2.393531436	0.002443143	0.028123241	AC009570.1
ENSG00000280035	2.393056691	0.000323243	0.005931306	AC011676.5
ENSG00000188011	2.392688918	0.001200639	0.016623012	RTP5
ENSG00000139890	2.392212213	1.70E-07	9.77E-06	REM2
ENSG00000107984	2.39114102	0.000309479	0.005726819	DKK1
ENSG00000144655	2.389972356	7.76E-14	2.01E-11	CSRNP1
ENSG00000102445	2.389187296	2.37E-08	1.88E-06	RUBCNL

ENSG00000170525	2.387680799	5.78E-08	3.89E-06	PFKFB3
ENSG00000229337	2.387394604	0.003894353	0.039179091	AC079305.3
ENSG00000131435	2.384684112	7.00E-06	0.000254327	PDLIM4
ENSG00000124145	2.38021985	6.76E-16	3.19E-13	SDC4
ENSG00000158050	2.378569712	4.80E-06	0.000184884	DUSP2
ENSG00000200879	2.374996236	0.000391881	0.006870106	SNORD14E
ENSG00000162571	2.369547857	0.0004552	0.007744666	TTLL10
ENSG00000261051	2.368123597	3.25E-05	0.000920492	AC107021.2
ENSG00000139797	2.367605735	0.004344809	0.042427708	RNF113B
ENSG00000197279	2.36585204	4.08E-08	2.94E-06	ZNF165
ENSG00000187556	2.362280505	7.82E-05	0.001887974	NANOS3
ENSG00000267122	2.362233081	0.000273748	0.005222556	AC004490.1
ENSG00000260823	2.359537621	0.001905582	0.023390868	AC026461.3
ENSG00000223573	2.35599408	6.81E-05	0.001682743	TINCR
ENSG00000128422	2.354401141	0.000637436	0.010097022	KRT17
ENSG00000070404	2.352679075	8.84E-08	5.57E-06	FSTL3
ENSG00000248713	2.347755799	0.001947844	0.023794144	AC083902.2
ENSG00000160886	2.346750763	0.000160415	0.00343977	LY6K
ENSG00000132965	2.345422568	9.30E-07	4.37E-05	ALOX5AP
ENSG00000230673	2.344896518	0.000476831	0.008012854	PABPC1P3
ENSG00000218073	2.341854924	0.00262287	0.029460163	AL021407.3
ENSG00000224411	2.335367621	3.66E-08	2.68E-06	HSP90AA2P
ENSG00000170542	2.330339409	1.45E-13	3.65E-11	SERPINB9
ENSG00000254166	2.326962673	0.000200042	0.004116007	CASC19
ENSG00000218991	2.321887549	0.002797437	0.030965142	CCNG1P1
ENSG00000234883	2.31850814	7.57E-06	0.000272624	MIR155HG
ENSG00000188766	2.314477998	1.23E-05	0.000411297	SPRED3
ENSG00000228863	2.311301561	0.001605713	0.020504675	AL121985.1
ENSG00000089169	2.310505164	0.001498506	0.019488835	RPH3A
ENSG00000258986	2.306561663	0.000131395	0.002923818	TMEM179
ENSG00000124216	2.303465243	1.59E-09	1.63E-07	SNAI1
ENSG00000267270	2.303366783	4.90E-11	7.27E-09	PARD6G-AS1
ENSG00000224969	2.300255599	0.005256164	0.04851527	AL645608.2
ENSG00000179820	2.298486547	2.90E-13	7.09E-11	MYADM
ENSG00000236095	2.296475325	0.001395958	0.018564596	AL807757.2
ENSG00000137331	2.295132002	8.69E-07	4.13E-05	IER3
ENSG00000236345	2.288036956	2.08E-05	0.000649221	AL354719.2
ENSG00000131746	2.287929074	5.37E-06	0.000202861	TNS4
ENSG00000124134	2.281077248	0.00340583	0.035617758	KCNS1
ENSG00000008516	2.280891172	3.08E-07	1.68E-05	MMP25
ENSG00000163600	2.280824101	0.000954672	0.013861171	ICOS
ENSG00000258521	2.279065216	1.55E-05	0.000500506	AL157871.2
ENSG00000197405	2.278486417	6.65E-13	1.50E-10	C5AR1
ENSG00000231233	2.277505732	0.001116826	0.015688768	CFAP58-AS1
ENSG00000188112	2.276487532	5.77E-05	0.001481766	C6orf132
ENSG00000133874	2.273439408	8.29E-10	9.11E-08	RNF122
ENSG00000274370	2.271681099	2.48E-05	0.000748089	AC130371.2
ENSG00000184292	2.269617955	0.000113274	0.002578088	TACSTD2
ENSG00000235947	2.267547184	0.001012779	0.01454137	EGOT
ENSG00000165071	2.267291449	2.10E-07	1.18E-05	TMEM71
ENSG00000226644	2.266951543	1.54E-07	9.04E-06	AL121899.1
ENSG00000230911	2.260409224	0.000476057	0.008010799	AP000907.1
ENSG00000116299	2.251749803	3.56E-06	0.000143008	KIAA1324
ENSG00000253276	2.250552096	1.16E-07	7.03E-06	CCDC71L
ENSG00000113303	2.244222441	6.37E-06	0.000235546	BTNL8
ENSG00000283849	2.240252064	5.83E-10	6.61E-08	AC092053.2
ENSG00000108576	2.239398366	0.000577076	0.009326501	SLC6A4
ENSG00000007908	2.236947311	2.84E-06	0.000116656	SELE
ENSG00000152413	2.236143169	3.71E-11	5.66E-09	HOMER1
ENSG00000167281	2.234975487	4.85E-07	2.47E-05	RBFOX3
ENSG00000188996	2.234808565	0.000602671	0.009640514	HUS1B
ENSG00000223461	2.228371131	4.85E-05	0.001277753	AC004471.1
ENSG00000117115	2.225484495	1.55E-06	6.82E-05	PADI2
ENSG00000268355	2.224606602	1.50E-05	0.00048683	AC243960.3
ENSG00000183484	2.216763295	2.75E-08	2.14E-06	GPR132
ENSG00000201512	2.213360522	1.83E-05	0.000575873	SNORA71C
ENSG00000198517	2.209204116	1.39E-11	2.36E-09	MAFK
ENSG00000198976	2.209112307	0.000191679	0.003960925	MIR429
ENSG00000228817	2.208492497	7.81E-05	0.001887974	BACH1-IT2
ENSG00000135604	2.208395285	2.09E-11	3.41E-09	STX11

ENSG00000138131	2.204660144	4.59E-05	0.001218503	LOXL4
ENSG00000269951	2.20098242	0.000522439	0.008622896	AC090181.2
ENSG00000106328	2.199881455	8.42E-05	0.002012108	FSCN3
ENSG00000228140	2.199620857	0.004100895	0.040720181	AL031283.1
ENSG00000222009	2.198410619	4.16E-11	6.28E-09	BTBD19
ENSG00000183696	2.197476233	3.39E-13	8.14E-11	UPP1
ENSG00000225217	2.196147593	8.08E-07	3.90E-05	HSPA7
ENSG00000226261	2.193980902	0.002064163	0.024863869	AC064836.1
ENSG00000120875	2.192895882	2.36E-06	9.95E-05	DUSP4
ENSG00000187037	2.190768026	7.43E-05	0.001808967	GPR141
ENSG00000099625	2.189216019	1.81E-06	7.86E-05	CBARP
ENSG00000159167	2.189168534	0.000196966	0.004055945	STC1
ENSG00000080824	2.185407059	3.96E-16	1.94E-13	HSP90AA1
ENSG00000273237	2.180145847	5.86E-07	2.91E-05	AC004520.1
ENSG00000269425	2.179875904	0.00521995	0.048301289	AC104521.1
ENSG00000260604	2.177859412	3.49E-05	0.000977643	AL590004.4
ENSG00000095752	2.176918279	1.44E-05	0.000471922	IL11
ENSG00000274536	2.176185011	1.69E-07	9.72E-06	AL034397.3
ENSG00000166523	2.173615281	0.000567494	0.009208387	CLEC4E
ENSG00000279386	2.171878311	0.003976081	0.039893037	AC021106.3
ENSG00000174010	2.171043604	2.99E-14	9.45E-12	KLHL15
ENSG00000173702	2.168522404	0.000373965	0.006614107	MUC13
ENSG00000128016	2.164634218	1.33E-10	1.77E-08	ZFP36
ENSG00000140678	2.161945937	2.35E-08	1.87E-06	ITGAX
ENSG00000104808	2.156781414	9.61E-08	5.99E-06	DHDH
ENSG00000180611	2.156005766	9.70E-09	8.45E-07	MB21D2
ENSG00000230638	2.155559549	0.001734182	0.021768307	AL445933.1
ENSG00000251194	2.154933615	7.15E-05	0.001753345	AL133330.1
ENSG00000205869	2.151641743	0.002257908	0.026556514	KRTAP5-1
ENSG00000125347	2.148350201	4.42E-15	1.74E-12	IRF1
ENSG00000135750	2.143580793	1.95E-06	8.40E-05	KCNK1
ENSG00000276107	2.142922076	4.38E-06	0.000170333	AC037198.2
ENSG00000229656	2.142027936	0.00029192	0.005460824	AL365203.1
ENSG00000138650	2.139932659	0.000352202	0.006315295	PCDH10
ENSG00000263050	2.137847417	5.71E-07	2.85E-05	AC090617.6
ENSG00000184588	2.132968791	1.08E-11	1.93E-09	PDE4B
ENSG00000223356	2.126385981	0.000807714	0.012156005	AL590666.1
ENSG00000148841	2.122185249	7.39E-09	6.54E-07	ITPRIP
ENSG00000260633	2.120101223	0.002577728	0.029104321	AC010207.1
ENSG00000213085	2.117821772	0.000333303	0.00606018	CFAP45
ENSG00000241978	2.117272624	2.78E-05	0.000816759	AKAP2
ENSG00000125462	2.110832624	0.000856725	0.012760381	C1orf61
ENSG00000179168	2.11029331	4.19E-05	0.001130071	GGN
ENSG00000140511	2.108735283	1.98E-07	1.12E-05	HAPLN3
ENSG00000250899	2.10815297	1.31E-06	5.90E-05	AC125807.2
ENSG00000236269	2.103975819	0.002039884	0.024674517	ENO1-IT1
ENSG00000177238	2.102817265	0.001122272	0.015756747	TRIM72
ENSG00000117228	2.101114401	2.23E-11	3.60E-09	GBP1
ENSG00000264553	2.097752461	0.000754357	0.011479505	MIR4257
ENSG00000186407	2.097574685	1.10E-05	0.000372718	CD300E
ENSG00000006555	2.097249954	3.39E-08	2.50E-06	TTC22
ENSG00000233013	2.094080219	2.94E-05	0.000847654	FAM157B
ENSG00000236782	2.092387396	7.33E-05	0.001787975	AL391650.1
ENSG00000163735	2.090687969	0.000706768	0.010908866	CXCL5
ENSG00000132326	2.087052995	3.15E-11	4.87E-09	PER2
ENSG00000259083	2.083717631	2.41E-05	0.00073371	AL132639.3
ENSG00000276853	2.0804794	0.000218871	0.004426206	AC026124.2
ENSG00000130513	2.079692472	0.000123869	0.002777801	GDF15
ENSG00000205277	2.076587056	5.87E-06	0.000219206	MUC12
ENSG00000236567	2.075471427	4.97E-05	0.001304273	TCF3P1
ENSG00000155090	2.073906083	2.49E-14	8.17E-12	KLF10
ENSG00000087903	2.073407847	8.01E-09	7.02E-07	RFX2
ENSG00000270988	2.07243307	0.004504065	0.04354041	AC019257.2
ENSG00000188404	2.072105365	1.15E-07	7.00E-06	SELL
ENSG00000229598	2.071982895	1.98E-06	8.48E-05	PRDX3P1
ENSG00000196136	2.071181039	8.14E-07	3.91E-05	SERPINA3
ENSG00000198805	2.070797658	2.25E-09	2.24E-07	PNP
ENSG00000148344	2.070035463	5.20E-05	0.001356082	PTGES
ENSG00000282572	2.06802197	6.71E-06	0.000245723	AC215522.2
ENSG00000163220	2.067768086	2.77E-06	0.000114237	S100A9

ENSG00000108244	2.064822737	0.000419925	0.007268431	KRT23
ENSG00000152784	2.063830321	0.000128124	0.002863316	PRDM8
ENSG00000260660	2.060518729	0.005170653	0.047999197	AC113208.3
ENSG00000106211	2.060516385	1.48E-10	1.91E-08	HSPB1
ENSG00000137193	2.059623922	1.23E-10	1.65E-08	PIM1
ENSG00000111837	2.05934943	2.53E-07	1.40E-05	MAK
ENSG00000275993	2.054579888	2.72E-06	0.000112329	CU639417.2
ENSG00000139514	2.052559639	3.60E-06	0.000144456	SLC7A1
ENSG00000122641	2.050378112	7.37E-08	4.72E-06	INHBA
ENSG00000269906	2.048235939	1.18E-08	1.01E-06	AL606834.2
ENSG00000187288	2.047997781	0.002352905	0.027511705	CIDEC
ENSG00000259687	2.044596067	0.000442699	0.007576698	LINC01220
ENSG00000263718	2.043853503	2.58E-05	0.000768978	AC068594.1
ENSG00000178752	2.043338389	0.000156843	0.00337993	ERFE
ENSG00000143514	2.042765085	2.95E-11	4.61E-09	TP53BP2
ENSG00000235631	2.042466232	0.001508551	0.019550877	RNF148
ENSG00000277639	2.042154642	3.11E-06	0.000126464	AC007906.2
ENSG00000171223	2.039489885	4.80E-09	4.38E-07	JUNB
ENSG00000054598	2.038547934	0.000223554	0.004503373	FOXC1
ENSG00000263393	2.037337726	0.00346067	0.036074942	AC011825.2
ENSG00000150347	2.032079505	6.70E-11	9.49E-09	ARID5B
ENSG00000274021	2.031780935	1.20E-06	5.51E-05	AC024909.3
ENSG00000005513	2.028079772	4.45E-05	0.00118551	SOX8
ENSG00000255202	2.01656639	0.001370717	0.018313406	AL049629.1
ENSG00000164236	2.016552893	2.56E-06	0.000106449	ANKRD33B
ENSG00000113739	2.012548673	6.72E-05	0.001671606	STC2
ENSG00000179431	2.012317053	2.17E-05	0.000669643	FJX1
ENSG00000123689	2.009265389	5.86E-06	0.000219206	G0S2
ENSG00000180549	2.007605	0.000165465	0.003513218	FUT7
ENSG00000137309	2.005773008	2.20E-09	2.19E-07	HMGA1
ENSG00000115963	2.003751788	3.08E-18	2.10E-15	RND3
ENSG00000143384	2.000510147	4.43E-19	3.59E-16	MCL1
ENSG00000249138	1.999894125	2.87E-05	0.000834787	SLED1
ENSG00000129654	1.998441156	0.003287213	0.034758935	FOXJ1
ENSG00000104783	1.994832481	0.000123679	0.002775941	KCNN4
ENSG00000179111	1.991998608	0.000249029	0.00484544	HES7
ENSG00000227036	1.991916564	0.003287537	0.034758935	LINC00511
ENSG00000256304	1.991048682	0.000333957	0.00606559	CCDC150P1
ENSG00000176845	1.989331356	1.29E-08	1.09E-06	METRNL
ENSG00000179292	1.987593421	6.74E-05	0.001675231	TMEM151A
ENSG00000130844	1.98524182	4.42E-19	3.59E-16	ZNF331
ENSG00000106236	1.983208446	1.74E-05	0.000553537	NPTX2
ENSG00000139318	1.981917901	4.69E-10	5.43E-08	DUSP6
ENSG00000228384	1.979596154	0.000157818	0.003398135	AC007040.1
ENSG00000140563	1.97702334	2.85E-05	0.000831337	MCTP2
ENSG00000144476	1.970116004	4.87E-06	0.000187048	ACKR3
ENSG00000232118	1.964911831	0.001405065	0.018657022	BACH1-AS1
ENSG00000263624	1.96455633	9.15E-05	0.002155276	AC055811.1
ENSG00000006747	1.964483314	0.003063165	0.03307854	SCIN
ENSG00000131196	1.963484261	3.21E-07	1.73E-05	NFATC1
ENSG00000277632	1.961259061	6.49E-10	7.26E-08	CCL3
ENSG00000228261	1.960208178	0.004754048	0.045149538	AL162742.1
ENSG00000099860	1.958107704	8.35E-10	9.14E-08	GADD45B
ENSG00000104856	1.957935687	1.12E-07	6.84E-06	RELB
ENSG00000121871	1.954671194	0.000689206	0.010733571	SLITRK3
ENSG00000160888	1.952265797	3.33E-14	1.02E-11	IER2
ENSG00000165105	1.950746065	8.60E-05	0.002049004	RASEF
ENSG00000235677	1.948424286	0.000622677	0.009917731	NPM1P26
ENSG00000162438	1.945080495	0.002856742	0.031411262	CTRC
ENSG00000188290	1.944800453	6.28E-05	0.001586425	HES4
ENSG00000100453	1.938661875	1.78E-06	7.76E-05	GZMB
ENSG00000235523	1.93523154	0.000997564	0.01438732	AL135924.2
ENSG00000018280	1.93519479	2.91E-05	0.000841562	SLC11A1
ENSG00000273319	1.933602445	6.15E-06	0.000228125	AC058791.1
ENSG00000123685	1.932142196	2.08E-06	8.85E-05	BATF3
ENSG00000010310	1.927394861	0.000112057	0.002554861	GIPR
ENSG00000021355	1.927117262	3.80E-09	3.59E-07	SERPINB1
ENSG00000162747	1.92404619	0.000180131	0.003766054	FCGR3B
ENSG00000148840	1.923589779	3.08E-10	3.67E-08	PPRC1
ENSG00000249884	1.922391319	0.000325693	0.005959406	RNF103-CHMP3

ENSG00000265096	1.917657544	0.000163166	0.003490247	C1QTNF1-AS1
ENSG00000215784	1.915221434	0.000355977	0.006374184	FAM72D
ENSG00000279541	1.913873895	2.45E-08	1.93E-06	AC005261.6
ENSG00000283413	1.910651672	0.001478502	0.019296384	AC008703.1
ENSG00000164736	1.910160489	0.000102309	0.002376456	SOX17
ENSG00000249741	1.907973358	0.001579875	0.020273728	AC093890.1
ENSG00000167470	1.907051088	2.53E-11	4.01E-09	MIDN
ENSG00000119535	1.902701519	8.12E-06	0.00028812	CSF3R
ENSG00000273812	1.898621968	0.001614493	0.020574474	BX640514.2
ENSG00000062582	1.898220797	0.000142804	0.003145335	MRPS24
ENSG00000137571	1.895142882	0.001370508	0.018313406	SLCO5A1
ENSG00000213694	1.894560876	4.81E-07	2.46E-05	S1PR3
ENSG00000230914	1.892981254	0.005339405	0.049056726	AC004840.1
ENSG00000272669	1.892893404	1.08E-08	9.30E-07	AL021707.6
ENSG00000188211	1.892111324	9.19E-06	0.000322273	NCR3LG1
ENSG00000280721	1.892007506	0.001240529	0.017021034	LINC01943
ENSG00000152137	1.887790917	2.69E-07	1.48E-05	HSPB8
ENSG00000159496	1.886766243	5.29E-08	3.63E-06	RGL4
ENSG00000238273	1.886584152	0.001111379	0.015654643	AC108058.1
ENSG00000232803	1.885503795	0.000299767	0.005595881	SLCO4A1-AS1
ENSG00000273368	1.882256241	0.004957598	0.046588132	AC006566.1
ENSG00000124159	1.879661144	0.000696107	0.010802154	MATN4
ENSG00000007350	1.878845214	0.001459865	0.019120472	TKTL1
ENSG00000224251	1.878249368	4.46E-06	0.000172865	AL391427.1
ENSG00000071282	1.877154998	2.00E-06	8.54E-05	LMCD1
ENSG00000162924	1.876319276	4.29E-11	6.40E-09	REL
ENSG00000275708	1.876311442	0.004971712	0.046670058	MIR3648-1
ENSG00000096006	1.874957863	0.002406489	0.027862009	CRISP3
ENSG00000202290	1.872657097	0.001523601	0.019716383	RNA5SP37
ENSG00000258096	1.87157508	9.75E-07	4.53E-05	AC025031.2
ENSG00000132334	1.86982492	5.16E-08	3.57E-06	PTPRE
ENSG00000111912	1.869799959	7.22E-08	4.65E-06	NCOA7
ENSG00000197714	1.867889406	5.17E-12	9.66E-10	ZNF460
ENSG00000280138	1.867615367	2.86E-08	2.19E-06	AC027290.2
ENSG00000173338	1.86725123	1.89E-07	1.08E-05	KCNK7
ENSG00000116717	1.862542641	3.07E-12	5.90E-10	GADD45A
ENSG00000274322	1.86120666	0.000397402	0.006948115	AL136531.2
ENSG00000165030	1.859869874	2.66E-09	2.61E-07	NFIL3
ENSG00000226091	1.858679251	0.002207869	0.026121914	LINC00937
ENSG00000010818	1.858592175	3.98E-09	3.72E-07	HIVEP2
ENSG00000259581	1.856966979	0.002327216	0.027260467	TYRO3P
ENSG00000250889	1.856954084	0.000369629	0.006546346	LINC01336
ENSG00000151651	1.853989047	1.49E-05	0.000483891	ADAM8
ENSG00000212493	1.850099645	0.00063042	0.010004208	SNORD19
ENSG00000180846	1.84949892	3.70E-05	0.001023665	CSNK1G2-AS1
ENSG00000010030	1.84547271	0.000149301	0.003260745	ETV7
ENSG00000100906	1.844921379	2.20E-12	4.42E-10	NFKBIA
ENSG00000170837	1.839335882	0.000166555	0.003527711	GPR27
ENSG00000205037	1.83883153	0.000549378	0.008947991	AC134312.1
ENSG00000138678	1.836019813	1.58E-08	1.30E-06	GPAT3
ENSG00000254317	1.835716612	0.005167598	0.047988007	AC022973.4
ENSG00000165507	1.834971184	3.19E-07	1.73E-05	C10orf10
ENSG00000255874	1.834946211	1.12E-10	1.52E-08	LINC00346
ENSG00000213145	1.834237595	0.000101836	0.002369714	CRIP1
ENSG00000279838	1.832034384	0.000206863	0.00423955	AL356273.3
ENSG00000262001	1.831332258	7.37E-06	0.000266571	DLGAP1-AS2
ENSG00000173926	1.830734557	6.25E-05	0.001583497	3-Mar
ENSG00000124225	1.830721453	0.000149203	0.003260745	PMEPA1
ENSG00000119699	1.8291655	3.42E-07	1.83E-05	TGFB3
ENSG00000242616	1.828343356	0.000486923	0.008129789	GNG10
ENSG00000104312	1.828129075	2.89E-14	9.26E-12	RIPK2
ENSG00000171051	1.826567821	7.42E-06	0.000268221	FPR1
ENSG00000176092	1.825253073	0.001611915	0.020553971	CRYBG2
ENSG00000142405	1.823764639	3.84E-05	0.001055892	NLRP12
ENSG00000197921	1.823535067	0.000715306	0.011001781	HES5
ENSG00000236438	1.817262067	3.99E-05	0.001085266	FAM157A
ENSG00000168309	1.815874573	0.000702902	0.010862114	FAM107A
ENSG00000052749	1.81355686	1.08E-09	1.15E-07	RRP12
ENSG00000234459	1.806493662	0.005328154	0.04898805	AC002064.2
ENSG00000243404	1.805892966	0.002554588	0.028943817	AC011276.1

ENSG00000178789	1.805700302	2.82E-05	0.000826716	CD300LB
ENSG00000186352	1.804711544	2.16E-07	1.21E-05	ANKRD37
ENSG00000257671	1.804030011	0.001448836	0.019043319	KRT7-AS
ENSG00000183114	1.802991639	9.52E-05	0.002228063	FAM43B
ENSG00000273076	1.796535537	0.00040386	0.007056264	AL021707.7
ENSG00000176014	1.795578128	1.80E-05	0.000568759	TUBB6
ENSG00000145777	1.792535689	1.98E-07	1.12E-05	TSLP
ENSG00000162783	1.789382584	7.43E-09	6.54E-07	IER5
ENSG00000113448	1.788782568	1.68E-09	1.72E-07	PDE4D
ENSG00000272367	1.785325869	0.000957274	0.013891179	AC018754.1
ENSG00000258086	1.782302157	3.12E-05	0.000886876	AC079313.1
ENSG00000069812	1.78206306	0.004458475	0.043244706	HES2
ENSG00000164171	1.778496789	0.000540001	0.008834093	ITGA2
ENSG00000115828	1.776974906	1.99E-05	0.000620448	QPCT
ENSG00000123975	1.776644872	2.46E-09	2.44E-07	CKS2
ENSG00000112773	1.776273681	7.63E-14	2.00E-11	FAM46A
ENSG00000183742	1.77444337	0.000213963	0.004343887	MACC1
ENSG00000197329	1.774202461	1.65E-11	2.74E-09	PELI1
ENSG00000116514	1.77240373	1.96E-10	2.47E-08	RNF19B
ENSG00000258469	1.772227048	0.003481518	0.036252879	CHMP4BP1
ENSG00000228340	1.768680352	0.003124195	0.033482187	MIR646HG
ENSG00000258959	1.767594731	0.002599554	0.029299756	AL118558.1
ENSG00000235713	1.766992271	0.000262607	0.005062105	AC004522.1
ENSG00000154099	1.762942869	0.000170627	0.003596328	DNAAF1
ENSG00000250548	1.76280712	0.004238828	0.04165804	AL355916.1
ENSG00000186891	1.762089699	0.00028442	0.005363057	TNFRSF18
ENSG00000280046	1.761768355	1.38E-05	0.000454526	AC104581.5
ENSG00000162413	1.759717907	4.52E-09	4.17E-07	KLHL21
ENSG00000135046	1.758600822	4.80E-08	3.37E-06	ANXA1
ENSG00000262652	1.757474971	0.001268946	0.017299455	AC124283.3
ENSG00000184205	1.756892883	7.89E-07	3.81E-05	TSPYL2
ENSG00000179564	1.756475821	0.000497602	0.008265547	LSMEM2
ENSG00000105205	1.756393134	0.001213644	0.016722892	CLC
ENSG00000115507	1.755047808	0.004554577	0.043832739	OTX1
ENSG00000280042	1.754677665	0.000104685	0.002424818	AC022336.3
ENSG00000158517	1.754443614	2.10E-05	0.000652057	NCF1
ENSG00000237892	1.754417884	0.000214367	0.004348674	KLF7-IT1
ENSG00000134571	1.753615037	1.14E-05	0.000385696	MYBPC3
ENSG00000119121	1.752191422	6.26E-05	0.001583497	TRPM6
ENSG00000280594	1.749938904	2.61E-05	0.000775284	BTG3-AS1
ENSG00000196550	1.747031764	0.000231761	0.004600823	FAM72A
ENSG00000183486	1.746923598	3.83E-08	2.79E-06	MX2
ENSG00000165732	1.742671093	7.29E-09	6.50E-07	DDX21
ENSG00000250116	1.737690272	0.000179192	0.003752481	AC018682.1
ENSG00000140941	1.734607324	1.09E-14	3.81E-12	MAP1LC3B
ENSG00000164691	1.734562081	1.24E-06	5.65E-05	TAGAP
ENSG00000027869	1.733049666	1.34E-05	0.000442816	SH2D2A
ENSG00000254810	1.731554901	3.98E-05	0.001084112	AP001189.3
ENSG00000236581	1.730250861	0.000426483	0.007352517	STARD13-AS
ENSG00000173530	1.729874028	2.24E-07	1.25E-05	TNFRSF10D
ENSG00000103313	1.729353067	1.54E-05	0.000496906	MEFV
ENSG00000279821	1.72873658	0.000207201	0.004239778	AC145098.2
ENSG00000261644	1.728673096	2.50E-05	0.000752452	AC007728.2
ENSG00000204618	1.726385183	0.000189211	0.003927047	RNF39
ENSG00000182687	1.721001675	0.000324788	0.005947031	GALR2
ENSG00000163132	1.720134838	7.62E-06	0.000273448	MSX1
ENSG00000224080	1.718968986	3.53E-06	0.000142052	UBE2FP1
ENSG00000171056	1.715115407	6.09E-07	3.02E-05	SOX7
ENSG00000140332	1.710126373	6.40E-08	4.22E-06	TLE3
ENSG00000145632	1.710087236	1.75E-11	2.89E-09	PLK2
ENSG00000076662	1.705067569	0.001307234	0.017665356	ICAM3
ENSG00000115758	1.704760472	2.39E-08	1.89E-06	ODC1
ENSG00000232934	1.703854218	2.61E-05	0.000775284	AL157786.1
ENSG00000109943	1.703497111	0.0004078	0.007096423	CRTAM
ENSG00000053524	1.697663008	0.00096338	0.013964624	MCF2L2
ENSG00000077150	1.694900538	3.17E-09	3.08E-07	NFKB2
ENSG00000255176	1.693672295	0.00223582	0.026356472	AP000941.1
ENSG00000173451	1.693671617	6.75E-06	0.000246668	THAP2
ENSG00000236304	1.69252213	0.000170167	0.00358955	AP001189.1
ENSG00000096384	1.691143079	5.72E-14	1.62E-11	HSP90AB1

ENSG00000277595	1.690446794	0.000255928	0.004959148	AC007546.1
ENSG00000233030	1.688804431	0.000407489	0.007096423	AC243772.2
ENSG00000131873	1.688598159	6.21E-08	4.14E-06	CHSY1
ENSG00000167604	1.685920423	4.61E-08	3.24E-06	NFKBID
ENSG00000023330	1.682240323	7.01E-08	4.52E-06	ALAS1
ENSG00000104892	1.682064027	0.001355355	0.018164271	KLC3
ENSG00000164070	1.68085433	7.89E-06	0.000282485	HSPA4L
ENSG00000258227	1.679568308	0.000706124	0.010905418	CLEC5A
ENSG00000110172	1.677746102	2.18E-08	1.76E-06	CHORDC1
ENSG00000114541	1.676924889	1.09E-10	1.49E-08	FRMD4B
ENSG00000233578	1.67652246	0.003286175	0.034758935	EIF4EP1
ENSG00000284669	1.676517798	6.29E-05	0.001586819	AC092053.3
ENSG00000131188	1.67600038	6.75E-05	0.001675893	PRR7
ENSG00000132003	1.675931661	4.00E-06	0.000157567	ZSWIM4
ENSG00000188910	1.675604763	2.16E-06	9.15E-05	GJB3
ENSG00000181409	1.675003204	6.96E-05	0.001715259	AATK
ENSG00000235852	1.674789604	0.00106637	0.01516006	AC005540.1
ENSG00000117479	1.674632788	1.00E-07	6.22E-06	SLC19A2
ENSG00000167207	1.67336607	9.97E-07	4.62E-05	NOD2
ENSG00000070729	1.670734792	0.003739628	0.038080217	CNGB1
ENSG00000262147	1.667948281	0.000349949	0.0062923	AC124283.2
ENSG00000142513	1.667824309	0.003875116	0.039030966	ACP4
ENSG00000122035	1.66492957	3.32E-09	3.20E-07	RASL11A
ENSG00000065911	1.662527365	3.12E-05	0.000886876	MTHFD2
ENSG00000272512	1.662310796	0.003378578	0.035393364	AL645608.8
ENSG00000127528	1.659979209	0.000421676	0.007289009	KLF2
ENSG00000213089	1.658853159	0.000602443	0.009640514	PDCL3P5
ENSG00000222937	1.658220986	5.41E-05	0.001405079	SNORD63B
ENSG00000110218	1.657630524	6.61E-08	4.31E-06	PANX1
ENSG00000143367	1.654646122	6.90E-08	4.47E-06	TUFT1
ENSG00000279863	1.653831602	1.00E-05	0.000346034	AC069547.1
ENSG00000124466	1.653804548	1.49E-06	6.60E-05	LYPD3
ENSG00000130751	1.653578612	0.003527342	0.036545592	NPAS1
ENSG00000274911	1.651963916	0.004266422	0.04185126	AL627230.2
ENSG00000134070	1.651088604	1.27E-08	1.08E-06	IRAK2
ENSG00000237499	1.650536396	2.09E-06	8.86E-05	AL357060.2
ENSG00000198754	1.649261919	0.002004172	0.024333294	OXCT2
ENSG00000115590	1.646970534	0.000568066	0.009211906	IL1R2
ENSG00000122862	1.646737917	2.48E-07	1.38E-05	SRGN
ENSG00000135114	1.6458306	8.62E-08	5.45E-06	OASL
ENSG00000134107	1.645798467	4.40E-08	3.13E-06	BHLHE40
ENSG00000154710	1.644714479	3.10E-07	1.68E-05	RABGEF1
ENSG00000103966	1.642571762	3.97E-08	2.86E-06	EHD4
ENSG00000160789	1.642183024	1.53E-09	1.58E-07	LMNA
ENSG00000125848	1.641549093	2.05E-06	8.72E-05	FLRT3
ENSG00000253406	1.635903293	0.000179158	0.003752481	AC012613.2
ENSG00000225614	1.635400218	0.000263493	0.005071651	ZNF469
ENSG00000178860	1.63208293	0.0001464	0.00320819	MSC
ENSG00000139722	1.630398752	6.71E-08	4.37E-06	VPS37B
ENSG00000164251	1.626645406	8.04E-06	0.000286442	F2RL1
ENSG00000157557	1.625387084	8.85E-10	9.61E-08	ETS2
ENSG00000161960	1.620846156	1.40E-06	6.21E-05	EIF4A1
ENSG00000197261	1.618949296	0.002194974	0.026016815	C6orf141
ENSG00000249661	1.618541731	1.49E-05	0.000483891	TNRC18P1
ENSG00000088826	1.616825574	0.000261394	0.005046232	SMOX
ENSG00000115194	1.614293009	0.00088082	0.013044387	SLC30A3
ENSG00000280385	1.610501182	7.84E-07	3.80E-05	AP000648.3
ENSG00000175040	1.610252142	2.57E-05	0.000767195	CHST2
ENSG00000131015	1.607996228	0.000116703	0.002642222	ULBP2
ENSG00000267325	1.607849811	0.000617388	0.009851625	LINC01415
ENSG00000263606	1.607051134	6.79E-05	0.001681803	AP000919.1
ENSG00000178381	1.606794333	7.64E-08	4.88E-06	ZFAND2A
ENSG00000260528	1.605322266	0.000166375	0.003526776	FAM157C
ENSG00000188215	1.603907626	5.29E-11	7.72E-09	DCUN1D3
ENSG00000112303	1.601110238	0.000105802	0.00244012	VNN2
ENSG00000223776	1.598659748	0.000473775	0.007982183	LGALS8-AS1
ENSG00000154479	1.595801378	0.000905509	0.013273635	CCDC173
ENSG00000206989	1.594798797	2.66E-05	0.000786472	SNORD63
ENSG00000258441	1.593121526	1.26E-06	5.69E-05	LINC00641
ENSG00000034152	1.593023667	2.01E-09	2.01E-07	MAP2K3

ENSG00000183199	1.591567964	3.74E-06	0.000149088	AC093768.1
ENSG00000254027	1.587898039	0.001911858	0.02343177	AC009902.2
ENSG00000163563	1.587239011	0.000327035	0.005971328	MNDA
ENSG00000235529	1.587110578	0.004353076	0.042464738	AGAP1-IT1
ENSG00000149257	1.585563028	3.18E-08	2.38E-06	SERPINH1
ENSG00000060138	1.584607207	1.95E-06	8.40E-05	YBX3
ENSG00000275385	1.583619016	0.000245907	0.004818961	CCL18
ENSG00000248099	1.582534316	0.001313163	0.017727021	INSL3
ENSG00000108700	1.582217603	0.003493369	0.036294208	CCL8
ENSG00000279447	1.580042042	0.004766086	0.045247302	AL118508.4
ENSG00000165807	1.57820892	0.004237326	0.04165804	PPP1R36
ENSG00000137265	1.577855912	0.00199469	0.024286361	IRF4
ENSG00000167106	1.57777063	3.28E-07	1.76E-05	FAM102A
ENSG00000105656	1.576479384	3.38E-09	3.25E-07	ELL
ENSG00000264947	1.575980551	0.001205801	0.016659064	MIR3181
ENSG00000100368	1.574625188	0.000119852	0.00270173	CSF2RB
ENSG00000125772	1.574585284	1.31E-08	1.10E-06	GPCPD1
ENSG00000237437	1.573851657	0.000310908	0.005741512	ASS1P12
ENSG00000233203	1.569740429	0.005109212	0.047602752	AC096536.1
ENSG00000254873	1.569419669	0.004001634	0.0400256	AP001267.1
ENSG00000127124	1.567304739	2.59E-05	0.000770462	HIVEP3
ENSG00000188064	1.566918674	0.001758697	0.022001514	WNT7B
ENSG00000154553	1.566160243	3.62E-05	0.001008879	PDLIM3
ENSG00000023445	1.564061597	0.001864498	0.023015023	BIRC3
ENSG00000196396	1.563737811	6.08E-11	8.71E-09	PTPN1
ENSG00000248476	1.561955329	0.003086823	0.033191344	BACH1-IT1
ENSG00000103257	1.561432431	1.35E-07	8.01E-06	SLC7A5
ENSG00000162892	1.560466248	0.000789443	0.01193641	IL24
ENSG00000117090	1.559457483	0.000463916	0.007862023	SLAMF1
ENSG00000212452	1.559307794	0.001437077	0.018936714	SNORD69
ENSG00000270120	1.558866206	0.002682142	0.029970223	AC007728.3
ENSG00000115956	1.557599886	5.62E-06	0.00021142	PLEK
ENSG00000189143	1.55694032	0.000856581	0.012760381	CLDN4
ENSG00000204165	1.556668568	0.002369288	0.027633641	CXorf65
ENSG00000117036	1.554594343	1.26E-11	2.20E-09	ETV3
ENSG00000205336	1.554490273	5.88E-05	0.0015008	ADGRG1
ENSG00000249992	1.551602885	6.40E-05	0.00160803	TMEM158
ENSG00000121101	1.551035517	1.64E-05	0.000524539	TEX14
ENSG00000101307	1.549091756	0.000532455	0.008752318	SIRPB1
ENSG00000167103	1.5486488	0.000145107	0.003187924	PIP5KL1
ENSG00000259863	1.546604711	0.000374304	0.0066156	SH3RF3-AS1
ENSG00000198142	1.544751424	1.03E-07	6.40E-06	SOWAHC
ENSG00000145780	1.542990294	5.11E-13	1.17E-10	FEM1C
ENSG00000232043	1.542354658	2.81E-05	0.000825037	AL133230.1
ENSG00000140406	1.540127749	1.73E-05	0.000552936	TLNRD1
ENSG00000181826	1.536667321	4.83E-08	3.38E-06	RELL1
ENSG00000165195	1.534812337	7.00E-10	7.79E-08	PIGA
ENSG00000168298	1.534719554	0.001388793	0.018507258	HIST1H1E
ENSG00000118194	1.531686138	0.00187689	0.023123355	TNNT2
ENSG00000184012	1.529146585	4.58E-08	3.23E-06	TMPRSS2
ENSG00000259583	1.529076624	0.003831328	0.038755601	AC015712.2
ENSG00000265136	1.528037822	4.61E-05	0.001222979	AC124283.4
ENSG00000163492	1.527195947	0.00451547	0.043625392	CCDC141
ENSG00000077984	1.526859147	3.79E-06	0.000150279	CST7
ENSG00000154537	1.526779411	0.003490322	0.036294208	FAM27C
ENSG00000123405	1.525085875	0.000828495	0.012389707	NFE2
ENSG00000156273	1.524776997	2.91E-10	3.48E-08	BACH1
ENSG00000105835	1.524203009	9.19E-08	5.76E-06	NAMPT
ENSG00000283199	1.523374527	0.000159374	0.003425953	FP565324.1
ENSG00000129226	1.522245716	0.000273345	0.005222546	CD68
ENSG00000143322	1.51930863	6.43E-08	4.24E-06	ABL2
ENSG00000272610	1.517053284	0.003654414	0.03743055	MAGI1-IT1
ENSG00000235750	1.516882024	7.66E-07	3.71E-05	KIAA0040
ENSG00000227200	1.516066697	0.005025265	0.047007821	AL162724.1
ENSG00000120063	1.515542862	1.31E-11	2.26E-09	GNA13
ENSG00000144031	1.512164877	4.50E-08	3.19E-06	ANKRD53
ENSG00000229644	1.511818313	5.52E-06	0.000207889	NAMPTP1
ENSG00000182752	1.511719906	0.001735228	0.021770899	PAPPA
ENSG00000278434	1.511649003	0.002411726	0.027897749	AC023830.3
ENSG00000223403	1.510158791	4.35E-05	0.001166794	MEG9

ENSG00000197905	1.506179309	0.000167655	0.003548098	TEAD4
ENSG00000095794	1.50389926	1.39E-10	1.83E-08	CREM
ENSG00000241163	1.503496631	5.23E-05	0.00136354	LINC00877
ENSG00000265907	1.502769394	0.001179759	0.016369004	AP000919.2
ENSG00000104804	1.502018866	0.00464502	0.044439415	TULP2
ENSG00000236060	1.501572149	0.000154751	0.003348766	HSPB1P1
ENSG00000273604	1.5007037	1.02E-05	0.000349367	EPOP
ENSG00000163602	1.50060452	6.59E-13	1.50E-10	RYBP
ENSG00000204390	1.500109794	1.25E-06	5.67E-05	HSPA1L
ENSG00000150991	1.500053329	5.06E-13	1.17E-10	UBC
ENSG00000184378	1.499686481	4.80E-07	2.45E-05	ACTRT3
ENSG00000232956	1.498284905	8.22E-06	0.000291071	SNHG15
ENSG00000126368	1.496222514	1.58E-06	6.94E-05	NR1D1
ENSG00000158406	1.495156101	0.000882725	0.013065129	HIST1H4H
ENSG00000169155	1.494722503	4.32E-08	3.09E-06	ZBTB43
ENSG00000144802	1.494423107	3.24E-08	2.40E-06	NFKBIZ
ENSG00000223956	1.493176719	0.00065593	0.010320656	LINC01767
ENSG00000101096	1.491855552	3.54E-07	1.88E-05	NFATC2
ENSG00000036672	1.490453807	2.95E-05	0.000849535	USP2
ENSG00000130477	1.489397419	0.004489666	0.043456439	UNC13A
ENSG00000237232	1.488055699	0.000377578	0.006659851	ZNF295-AS1
ENSG00000274026	1.487305249	0.000813511	0.01220078	FAM27E3
ENSG00000164778	1.486808112	0.001905816	0.023390868	EN2
ENSG00000180530	1.486087848	1.75E-05	0.000555108	NRIP1
ENSG00000185947	1.485823923	1.48E-08	1.23E-06	ZNF267
ENSG00000145491	1.485153468	0.001605854	0.020504675	ROPN1L
ENSG00000260196	1.484683166	0.000647458	0.010212119	AC124798.1
ENSG00000188389	1.484226865	0.004220828	0.041558771	PDCD1
ENSG00000166197	1.480189369	4.22E-09	3.93E-07	NOLC1
ENSG00000196352	1.474386789	4.37E-07	2.28E-05	CD55
ENSG00000136158	1.473109561	1.70E-08	1.39E-06	SPRY2
ENSG00000117152	1.472935757	0.00031241	0.005761037	RGS4
ENSG00000100036	1.471081157	7.25E-05	0.001772284	SLC35E4
ENSG00000144381	1.470749212	1.45E-09	1.51E-07	HSPD1
ENSG00000110047	1.467585523	8.02E-08	5.11E-06	EHD1
ENSG00000055483	1.466729441	1.91E-12	3.93E-10	USP36
ENSG00000156127	1.462385304	6.34E-05	0.001595997	BATF
ENSG00000166106	1.46020066	0.000147621	0.003232216	ADAMTS15
ENSG00000006062	1.459440254	9.61E-11	1.33E-08	MAP3K14
ENSG00000258667	1.457274969	0.005378381	0.049292647	HIF1A-AS2
ENSG00000244161	1.455765497	0.00020452	0.004194843	FLNB-AS1
ENSG00000120129	1.455758772	0.000963412	0.013964624	DUSP1
ENSG00000140297	1.455742355	0.002815864	0.031099518	GCNT3
ENSG00000233029	1.455656904	0.003652353	0.03743055	AC244453.2
ENSG00000241886	1.454980841	0.003414726	0.035667667	AC112496.1
ENSG00000109846	1.454596132	2.85E-05	0.000831337	CRYAB
ENSG00000117877	1.454353953	8.08E-05	0.001939432	CD3EAP
ENSG00000230724	1.452435541	0.00057729	0.009326501	LINC01001
ENSG00000177875	1.450568344	0.001862788	0.023015023	CCDC184
ENSG00000118985	1.450441757	2.50E-07	1.39E-05	ELL2
ENSG00000253250	1.449864686	0.002793455	0.030934266	C8orf88
ENSG00000185467	1.449086966	0.002438449	0.028118318	KPNA7
ENSG00000141526	1.448820922	0.000238009	0.004699682	SLC16A3
ENSG00000006652	1.447380941	4.47E-13	1.06E-10	IFRD1
ENSG00000267216	1.446895467	0.000428545	0.007378262	AC020915.2
ENSG00000022567	1.446045877	0.000102159	0.002375115	SLC45A4
ENSG00000230701	1.444941034	0.000111537	0.00254524	FBXW4P1
ENSG00000100226	1.443090678	1.18E-11	2.09E-09	GTPBP1
ENSG00000107968	1.442195375	2.85E-07	1.57E-05	MAP3K8
ENSG00000111981	1.441344062	0.001893275	0.023280997	ULBP1
ENSG00000230149	1.439487527	0.000254483	0.004934831	AL021707.3
ENSG00000110848	1.436226734	0.002196135	0.026018688	CD69
ENSG00000143226	1.436016456	3.22E-05	0.000912325	FCGR2A
ENSG00000100401	1.436005593	5.92E-06	0.000220617	RANGAP1
ENSG00000181026	1.436004584	9.47E-07	4.44E-05	AEN
ENSG00000253930	1.43591452	0.000209267	0.004268582	TNFRSF10A-AS1
ENSG00000102393	1.435205822	3.91E-08	2.84E-06	GLA
ENSG00000185046	1.435067215	0.001467343	0.019189357	ANKS1B
ENSG00000109320	1.433376091	3.96E-08	2.86E-06	NFKB1
ENSG00000168386	1.432632621	3.19E-06	0.000129329	FILIP1L

ENSG00000157551	1.432443566	0.002385429	0.027741971	KCNJ15
ENSG00000122733	1.431703979	0.001436971	0.018936714	PHF24
ENSG00000184274	1.430168797	0.003156096	0.03374045	LINC00315
ENSG00000132475	1.428733494	6.30E-14	1.73E-11	H3F3B
ENSG00000196189	1.426771455	6.29E-05	0.001586819	SEMA4A
ENSG00000175556	1.426116618	3.32E-10	3.93E-08	LONRF3
ENSG00000215910	1.426071396	0.002678163	0.029964475	C1orf167
ENSG00000154548	1.425584176	0.000953058	0.013845494	SRSF12
ENSG00000130449	1.422671005	8.73E-07	4.14E-05	ZSWIM6
ENSG00000148154	1.420263137	1.79E-09	1.82E-07	UGCG
ENSG00000139438	1.419173994	1.48E-05	0.000480823	FAM222A
ENSG00000107130	1.41896172	1.45E-05	0.00047244	NCS1
ENSG00000246334	1.417923357	0.000329385	0.006010007	PRR7-AS1
ENSG00000266970	1.417512914	0.002404374	0.027862009	AC061992.1
ENSG00000008056	1.41724394	0.001320487	0.017807352	SYN1
ENSG00000238018	1.415829953	0.001839217	0.022789082	AC093110.1
ENSG00000090376	1.414600069	5.62E-05	0.001451166	IRAK3
ENSG00000271303	1.4142326	1.47E-06	6.53E-05	SRXN1
ENSG00000089558	1.413877559	0.001507792	0.019550813	KCNH4
ENSG00000177426	1.413736472	1.31E-10	1.75E-08	TGIF1
ENSG00000135074	1.41136101	1.02E-05	0.000349483	ADAM19
ENSG00000278330	1.409668425	3.88E-05	0.001063531	AC018529.2
ENSG00000141384	1.408738794	0.003591983	0.036953847	TAF4B
ENSG00000054967	1.407211255	8.02E-06	0.000286393	RELT
ENSG00000008405	1.40625217	3.03E-07	1.65E-05	CRY1
ENSG00000180616	1.406119507	8.87E-07	4.20E-05	SSTR2
ENSG00000138685	1.403336258	0.000158169	0.003402865	FGF2
ENSG00000104885	1.402929633	2.02E-06	8.62E-05	DOT1L
ENSG00000143479	1.398791646	1.12E-07	6.84E-06	DYRK3
ENSG00000164741	1.397259309	4.64E-10	5.40E-08	DLC1
ENSG00000163545	1.396128798	1.26E-06	5.69E-05	NUAK2
ENSG00000176928	1.393416636	0.000164671	0.003506527	GCNT4
ENSG00000196878	1.393401182	7.06E-05	0.001735617	LAMB3
ENSG00000111424	1.390360938	0.000339055	0.006147532	VDR
ENSG00000231964	1.390308049	0.005047508	0.047159759	AL731567.1
ENSG00000258317	1.389366431	0.000966764	0.014005397	AC034102.6
ENSG00000137507	1.386952365	5.74E-05	0.001477071	LRRC32
ENSG00000086061	1.385081616	4.98E-08	3.48E-06	DNAJA1
ENSG00000105321	1.384808026	3.69E-10	4.31E-08	CCDC9
ENSG00000237624	1.384190532	0.003563162	0.03671742	OXCT2P1
ENSG00000187775	1.383115688	9.92E-07	4.61E-05	DNAH17
ENSG00000104951	1.379907355	0.002982735	0.032448654	IL4I1
ENSG00000276600	1.379220555	0.001593059	0.020391472	RAB7B
ENSG00000163660	1.3766799	1.28E-11	2.21E-09	CCNL1
ENSG00000143878	1.376462065	4.15E-07	2.18E-05	RHOB
ENSG00000130821	1.376454656	0.005306229	0.048855723	SLC6A8
ENSG00000169252	1.374156882	3.42E-06	0.000138221	ADRB2
ENSG00000164867	1.369102767	0.000242753	0.004767946	NOS3
ENSG00000078804	1.368907001	1.50E-09	1.56E-07	TP53INP2
ENSG00000211574	1.36695467	0.003126624	0.033494375	MIR770
ENSG00000100678	1.366489657	0.002120633	0.025379121	SLC8A3
ENSG00000197208	1.364715288	2.83E-05	0.000828182	SLC22A4
ENSG00000136383	1.364677086	0.000547779	0.008933163	ALPK3
ENSG00000237181	1.36219748	8.79E-05	0.002085993	AC147651.3
ENSG00000158615	1.359513817	1.23E-09	1.30E-07	PPP1R15B
ENSG00000263675	1.35936765	0.004389023	0.042730804	MIR5581
ENSG00000214193	1.359365019	4.39E-05	0.001171984	SH3D21
ENSG00000158792	1.356539411	1.13E-05	0.000381902	SPATA2L
ENSG00000160223	1.355851354	0.001227663	0.016878063	ICOSLG
ENSG00000256694	1.355357603	0.001209334	0.0166901	AC026369.2
ENSG00000176788	1.354015437	3.67E-05	0.001019615	BASP1
ENSG00000200913	1.351942761	0.00374372	0.038106924	SNORD46
ENSG00000123146	1.35054575	1.02E-05	0.000348894	ADGRE5
ENSG00000167995	1.34984223	7.87E-05	0.001899039	BEST1
ENSG00000235151	1.349263077	0.002442229	0.028123241	AC131097.4
ENSG00000154153	1.348975834	0.000246682	0.004819583	RETREG1
ENSG00000280303	1.348890577	0.00415488	0.041135528	AC067931.2
ENSG00000112033	1.347548715	9.15E-06	0.000321733	PPARD
ENSG00000259207	1.346995506	0.000257776	0.00498379	ITGB3
ENSG00000073737	1.345938785	0.000880715	0.013044387	DHRS9

ENSG00000067334	1.345052851	1.12E-11	2.00E-09	DNTTIP2
ENSG00000229808	1.344888639	0.000263021	0.005066326	AL391825.1
ENSG00000116991	1.344596697	0.001090508	0.015444498	SIPA1L2
ENSG00000163121	1.343283254	0.002131404	0.025469147	NEURL3
ENSG00000141448	1.342902727	5.59E-08	3.80E-06	GATA6
ENSG00000166401	1.342656459	2.56E-06	0.000106449	SERPINB8
ENSG00000154640	1.342556151	3.35E-08	2.48E-06	BTG3
ENSG00000255150	1.340459993	1.33E-05	0.000440463	EID3
ENSG00000143867	1.340372251	0.00489863	0.046201325	OSR1
ENSG00000275183	1.336363986	9.52E-07	4.44E-05	LENG9
ENSG00000123908	1.330891105	4.10E-07	2.16E-05	AGO2
ENSG00000174946	1.32965155	0.004730737	0.044977539	GPR171
ENSG00000100079	1.329263141	0.004420582	0.042957457	LGALS2
ENSG00000166900	1.327004873	4.25E-08	3.05E-06	STX3
ENSG00000171786	1.325762214	0.003685794	0.03767792	NHLH1
ENSG00000164674	1.325145729	3.87E-05	0.001060902	SYTL3
ENSG00000196843	1.32512521	3.84E-06	0.00015192	ARID5A
ENSG00000101187	1.322052842	0.000765054	0.011635459	SLCO4A1
ENSG00000267632	1.321064519	0.002528954	0.028744545	AC067852.6
ENSG00000268903	1.319413372	0.001113783	0.015662986	AL627309.7
ENSG00000109819	1.319044426	0.000105256	0.002431856	PPARGC1A
ENSG00000269981	1.318764158	0.001456382	0.019107953	AL627309.8
ENSG00000148926	1.318159175	0.000366014	0.006506898	ADM
ENSG00000148339	1.316987142	4.43E-06	0.000171755	SLC25A25
ENSG00000187116	1.316902285	0.001024676	0.014664233	LILRA5
ENSG00000109971	1.316634913	3.05E-05	0.000871171	HSPA8
ENSG00000133065	1.315455022	4.15E-05	0.001119523	SLC41A1
ENSG00000056972	1.315328533	0.00018871	0.00392329	TRAF3IP2
ENSG00000028137	1.314124023	3.13E-08	2.35E-06	TNFRSF1B
ENSG00000137494	1.311493955	5.24E-08	3.60E-06	ANKRD42
ENSG00000177464	1.311204527	7.06E-05	0.001735617	GPR4
ENSG00000124253	1.311158063	0.000191741	0.003960925	PCK1
ENSG00000237094	1.310678909	0.001904466	0.023390868	AL732372.2
ENSG00000092820	1.310026292	2.44E-05	0.000739178	EZR
ENSG00000229915	1.30897978	0.002669534	0.029916613	AC016999.1
ENSG00000279133	1.304862496	0.000177533	0.003726597	AC018628.1
ENSG00000090924	1.304649629	8.10E-06	0.00028812	PLEKHG2
ENSG00000154252	1.298141108	0.003213876	0.034217163	GAL3ST2
ENSG00000111057	1.298096508	5.77E-06	0.00021627	KRT18
ENSG00000254285	1.294446414	0.000588068	0.009453534	KRT8P3
ENSG00000232713	1.293637319	0.001566013	0.020154649	AC010733.1
ENSG00000104689	1.290889365	6.45E-06	0.000237188	TNFRSF10A
ENSG00000152952	1.28924048	0.002563177	0.02900314	PLOD2
ENSG00000163435	1.288417159	0.000477752	0.008023142	ELF3
ENSG00000057704	1.288340098	7.20E-05	0.001762256	TMCC3
ENSG00000143067	1.285252967	3.06E-06	0.000124972	ZNF697
ENSG00000085265	1.279834907	0.001977984	0.024116906	FCN1
ENSG00000180525	1.278963564	1.94E-06	8.37E-05	PRR26
ENSG00000130592	1.277267549	0.001443481	0.018992171	LSP1
ENSG00000163083	1.274495775	0.00030669	0.005704213	INHBB
ENSG00000172183	1.274274288	0.000742616	0.011360806	ISG20
ENSG00000175768	1.273948332	0.000154628	0.003348766	TOMM5
ENSG00000266171	1.273797952	0.004721279	0.044904072	AP001020.3
ENSG00000101162	1.272000076	0.000208708	0.004260539	TUBB1
ENSG00000087085	1.271678843	0.000230993	0.004599563	ACHE
ENSG00000196182	1.27135602	4.54E-08	3.21E-06	STK40
ENSG00000198355	1.271326421	6.55E-08	4.29E-06	PIM3
ENSG00000172059	1.271265279	4.20E-05	0.001130311	KLF11
ENSG00000124251	1.271135709	0.003064665	0.03307854	TP53TG5
ENSG00000230844	1.269953387	9.49E-05	0.002221149	ZNF674-AS1
ENSG00000186594	1.268618612	2.31E-05	0.000708782	MIR22HG
ENSG00000187134	1.267102943	0.00022226	0.004480783	AKR1C1
ENSG00000167034	1.266614272	6.38E-08	4.22E-06	NKX3-1
ENSG00000188483	1.266375746	0.00379316	0.038489378	IER5L
ENSG00000171368	1.265812966	1.29E-05	0.000427267	TPPP
ENSG00000184254	1.265244519	0.000227731	0.004567085	ALDH1A3
ENSG00000189320	1.264280681	0.000141295	0.003117386	FAM180A
ENSG00000070495	1.261375521	8.56E-12	1.54E-09	JMJD6
ENSG00000107372	1.260844554	2.88E-10	3.45E-08	ZFAND5
ENSG00000164142	1.258455835	0.000648666	0.010224959	FAM160A1

ENSG00000159216	1.257369429	0.000337657	0.006126476	RUNX1
ENSG00000101236	1.255515372	5.94E-06	0.000221237	RNF24
ENSG00000273472	1.255259934	0.00044976	0.007677287	AC096733.2
ENSG00000131019	1.254294654	0.003112883	0.033402345	ULBP3
ENSG00000158470	1.251606833	2.54E-05	0.000758816	B4GALT5
ENSG00000277443	1.25111743	5.66E-05	0.001458466	MARCKS
ENSG00000176907	1.250739905	8.90E-05	0.002108042	C8orf4
ENSG00000145362	1.245928158	0.000248138	0.004837107	ANK2
ENSG00000106952	1.244770467	0.000830547	0.012413231	TNFSF8
ENSG00000049759	1.244058903	3.71E-07	1.97E-05	NEDD4L
ENSG00000188897	1.238668557	3.65E-05	0.001014332	AC099489.1
ENSG00000235908	1.236931614	0.005115311	0.047621767	RHOA-IT1
ENSG00000116701	1.236922495	0.000164474	0.003506526	NCF2
ENSG00000278709	1.23536375	0.003787311	0.03844506	NKILA
ENSG00000205890	1.234249066	0.004182243	0.041290895	AC108134.1
ENSG00000279539	1.232349144	0.005287872	0.048721281	AC006486.2
ENSG00000237719	1.231735275	0.002803709	0.030994902	Z95152.1
ENSG00000166016	1.231301296	2.84E-06	0.000116696	ABTB2
ENSG00000184922	1.2311042	0.001290189	0.017507975	FMNL1
ENSG00000111913	1.230070931	0.002562423	0.02900314	RIPOR2
ENSG00000116954	1.229300032	2.12E-07	1.19E-05	RRAGC
ENSG00000152484	1.229273235	2.70E-07	1.48E-05	USP12
ENSG00000256678	1.228688746	0.002178484	0.025880469	AC025423.3
ENSG00000232810	1.228114508	0.003075172	0.033107222	TNF
ENSG00000224888	1.226903891	0.001439616	0.018960552	AC138028.2
ENSG00000020577	1.226035915	9.41E-06	0.00032822	SAMD4A
ENSG00000219891	1.225505026	0.003226292	0.034293065	ZSCAN12P1
ENSG00000272078	1.222611292	1.10E-05	0.000372718	AL139423.1
ENSG00000279198	1.221239238	0.000640888	0.010126965	AC008894.3
ENSG00000151967	1.220390911	0.000332974	0.006058442	SCHIP1
ENSG00000275880	1.21692473	2.31E-05	0.000708782	AL139385.1
ENSG00000004478	1.216085664	6.64E-06	0.000243445	FKBP4
ENSG00000122254	1.215686128	0.001951321	0.023825394	HS3ST2
ENSG00000136040	1.213618596	0.000772379	0.011719383	PLXNC1
ENSG00000145779	1.212627746	0.000190006	0.003937031	TNFAIP8
ENSG00000116852	1.210976984	0.000350567	0.006296952	KIF21B
ENSG00000130066	1.209776602	2.51E-06	0.000104675	SAT1
ENSG00000167173	1.208741352	9.11E-06	0.000320784	C15orf39
ENSG00000164164	1.20816916	2.42E-05	0.000733818	OTUD4
ENSG00000064932	1.207211821	1.87E-05	0.000588599	SBNO2
ENSG00000122694	1.206554866	4.98E-05	0.001304273	GLIPR2
ENSG00000105993	1.205633084	1.14E-08	9.77E-07	DNAJB6
ENSG00000100644	1.205585114	7.77E-05	0.001881663	HIF1A
ENSG00000278864	1.205550886	0.001680157	0.021244323	AC055811.4
ENSG00000000938	1.203653718	0.000349345	0.006286017	FGR
ENSG00000184602	1.203185062	1.83E-08	1.50E-06	SNN
ENSG00000130222	1.202147945	0.004677644	0.044636318	GADD45G
ENSG00000173559	1.19975767	0.000153656	0.003338998	NABP1
ENSG00000107338	1.198879174	2.45E-05	0.000739896	SHB
ENSG00000151151	1.193747435	1.04E-07	6.41E-06	IPMK
ENSG00000228436	1.192899796	0.000536712	0.008799112	AL139260.1
ENSG00000116574	1.191900321	4.45E-09	4.12E-07	RHOU
ENSG00000171988	1.189088429	4.63E-07	2.38E-05	JMJD1C
ENSG00000280407	1.188136569	0.000148631	0.003251594	AC132872.4
ENSG00000279765	1.184169895	0.000969035	0.014030461	AC013394.1
ENSG00000175868	1.182698867	0.002734843	0.030401985	CALCB
ENSG00000171777	1.182559488	0.001425832	0.018817214	RASGRP4
ENSG00000268279	1.182167329	0.002511626	0.028632093	AC090004.1
ENSG00000143507	1.181862794	0.000177669	0.003726597	DUSP10
ENSG00000243444	1.181617374	0.003066133	0.03307854	PALM2
ENSG00000115598	1.181574362	0.000109617	0.002512468	IL1RL2
ENSG00000216490	1.178256553	0.000279916	0.005305118	IFI30
ENSG00000280434	1.176099764	0.004354173	0.042464738	AL031595.3
ENSG00000159713	1.175429515	0.003407544	0.035621325	TPPP3
ENSG00000161681	1.173996781	0.003391364	0.035480778	SHANK1
ENSG00000227741	1.173011062	0.001265984	0.017278912	AL121987.2
ENSG00000127507	1.17281831	0.000639484	0.010117102	ADGRE2
ENSG00000077782	1.170903242	0.000473506	0.007982183	FGFR1
ENSG00000277782	1.165153788	0.004156183	0.041135528	AC068870.2
ENSG00000170421	1.164551509	2.44E-05	0.000739115	KRT8

ENSG00000100221	1.163120925	1.76E-10	2.24E-08	JOSD1
ENSG00000172803	1.162819346	0.000366275	0.006506898	SNX32
ENSG00000189067	1.162713535	2.80E-08	2.16E-06	LITAF
ENSG00000115641	1.162458263	0.004114444	0.040829245	FHL2
ENSG00000150510	1.160850249	0.000288584	0.005417671	FAM124A
ENSG00000147872	1.159611969	0.000906694	0.013283497	PLIN2
ENSG00000121966	1.159002934	0.004883757	0.04611134	CXCR4
ENSG00000214264	1.158242058	0.005187351	0.048085394	KCTD9P4
ENSG00000026508	1.158064304	0.000674055	0.010548272	CD44
ENSG00000141441	1.153416503	0.000601458	0.009638189	GAREM1
ENSG00000081320	1.152932713	0.000608239	0.00971161	STK17B
ENSG00000168404	1.152630232	1.31E-05	0.000433742	MLKL
ENSG00000185112	1.151201593	0.003035731	0.032901037	FAM43A
ENSG00000076604	1.149578819	1.89E-06	8.17E-05	TRAF4
ENSG00000133134	1.148378769	0.004529006	0.043683864	BEX2
ENSG00000142227	1.147989079	0.000972839	0.014077682	EMP3
ENSG00000179094	1.147700632	0.000273596	0.005222556	PER1
ENSG00000166579	1.147546333	3.61E-09	3.43E-07	NDEL1
ENSG00000273796	1.14626148	0.002486039	0.028440374	BX322562.1
ENSG00000250295	1.14325382	0.001708762	0.021543028	RDH10-AS1
ENSG00000109220	1.142527027	2.46E-06	0.000103167	CHIC2
ENSG00000213430	1.142511705	1.62E-05	0.000519792	HSPD1P1
ENSG00000034677	1.142384293	7.61E-07	3.70E-05	RNF19A
ENSG00000146278	1.142249323	1.42E-06	6.29E-05	PNRC1
ENSG00000115520	1.139738783	9.82E-09	8.52E-07	COQ10B
ENSG00000244482	1.138804481	0.000486005	0.008127847	LILRA6
ENSG00000171522	1.138713765	0.001435436	0.018934327	PTGER4
ENSG00000233058	1.138321834	0.000347611	0.006267739	LINC00884
ENSG00000178607	1.137815885	1.19E-07	7.19E-06	ERN1
ENSG00000225313	1.137345212	9.15E-05	0.002155276	AL513327.1
ENSG00000147883	1.136546	0.004115026	0.040829245	CDKN2B
ENSG00000275888	1.135823985	0.000721895	0.011083005	AC132872.3
ENSG00000205129	1.135391271	0.004342642	0.04242534	C4orf47
ENSG00000224383	1.134044626	0.001932776	0.023632325	PRR29
ENSG00000120889	1.133322031	9.69E-07	4.51E-05	TNFRSF10B
ENSG00000134013	1.131113454	0.002972259	0.032375407	LOXL2
ENSG00000147650	1.129902073	1.29E-05	0.000427267	LRP12
ENSG00000159399	1.127159665	0.000114182	0.002594188	HK2
ENSG00000169896	1.125335916	0.000662282	0.01039538	ITGAM
ENSG00000171867	1.123369362	1.79E-07	1.02E-05	PRNP
ENSG00000138772	1.119115187	0.001731808	0.021749026	ANXA3
ENSG00000106089	1.117054538	0.001379673	0.018414091	STX1A
ENSG00000106993	1.117009404	1.25E-06	5.67E-05	CDC37L1
ENSG00000170385	1.116323083	2.64E-05	0.000781904	SLC30A1
ENSG00000114861	1.113967939	4.40E-08	3.13E-06	FOXP1
ENSG00000162645	1.113384633	2.51E-05	0.000752615	GBP2
ENSG00000258102	1.111732414	3.75E-06	0.000149143	MAP1LC3B2
ENSG00000174791	1.111603749	0.004578731	0.044016212	RIN1
ENSG00000120949	1.110300416	0.003346816	0.035209751	TNFRSF8
ENSG00000252759	1.10996446	0.002605994	0.02934684	Y_RNA
ENSG00000146232	1.109400352	8.11E-06	0.00028812	NFKBIE
ENSG00000183779	1.109110496	0.001720327	0.021636258	ZNF703
ENSG00000047365	1.109032193	0.000239898	0.004722613	ARAP2
ENSG00000119138	1.108918049	0.00102773	0.014691723	KLF9
ENSG00000178409	1.108447674	0.000134446	0.002984028	BEND3
ENSG00000026652	1.10392876	0.000269674	0.005167617	AGPAT4
ENSG00000112658	1.102185475	5.77E-08	3.89E-06	SRF
ENSG00000181472	1.101702993	1.20E-09	1.28E-07	ZBTB2
ENSG00000142867	1.099372451	3.83E-08	2.79E-06	BCL10
ENSG00000197885	1.098839017	6.39E-06	0.000235546	NKIRAS1
ENSG00000168439	1.097899297	1.65E-05	0.000526857	STIP1
ENSG00000115271	1.097697898	7.09E-05	0.001741172	GCA
ENSG00000184014	1.097530802	1.02E-06	4.70E-05	DENND5A
ENSG00000101367	1.095825764	1.63E-07	9.46E-06	MAPRE1
ENSG00000198431	1.095413713	8.06E-05	0.00193745	TXNRD1
ENSG00000131263	1.094388253	1.38E-07	8.19E-06	RLIM
ENSG00000126561	1.092455276	3.39E-05	0.000954329	STAT5A
ENSG00000146112	1.092065761	1.09E-05	0.000370445	PPP1R18
ENSG00000130589	1.091444821	2.36E-05	0.000721418	HELZ2
ENSG00000146072	1.089931762	0.00055704	0.009055736	TNFRSF21

ENSG00000113763	1.089861307	0.001254965	0.017173693	UNC5A
ENSG00000231721	1.089521587	9.43E-07	4.42E-05	LINC-PINT
ENSG00000115137	1.088619639	6.90E-06	0.000251452	DNAJC27
ENSG00000242265	1.088353496	0.00353285	0.036548373	PEG10
ENSG00000204758	1.087073112	0.004587137	0.044080689	AC008429.1
ENSG00000185950	1.086386783	0.000553958	0.00901128	IRS2
ENSG00000035403	1.086102912	3.42E-05	0.00095902	VCL
ENSG00000023909	1.084360124	8.00E-05	0.001924811	GCLM
ENSG00000062716	1.083843812	9.26E-06	0.000324299	VMP1
ENSG00000173918	1.083176137	0.000519968	0.008587586	C1QTNF1
ENSG00000254612	1.082222819	0.000945262	0.013747633	AP001000.1
ENSG00000092871	1.081393138	0.00035823	0.006401261	RFFL
ENSG00000249700	1.081288918	0.004093315	0.040676049	SRD5A3-AS1
ENSG00000135842	1.076105578	0.003258701	0.034552621	FAM129A
ENSG00000180801	1.075095254	0.000189043	0.003927047	ARSJ
ENSG00000163565	1.074981909	0.000224473	0.004518369	IFI16
ENSG00000153922	1.074622851	1.04E-06	4.78E-05	CHD1
ENSG00000134317	1.074141807	0.000309202	0.005726819	GRHL1
ENSG00000187678	1.073731997	4.00E-06	0.000157567	SPRY4
ENSG00000172578	1.073011244	0.000324082	0.005939747	KLHL6
ENSG00000169299	1.071527412	6.17E-07	3.05E-05	PGM2
ENSG00000226479	1.069341909	5.26E-06	0.000199108	TMEM185B
ENSG00000153443	1.06842606	0.000161196	0.003453662	UBALD1
ENSG00000163378	1.06810139	0.000410542	0.007134568	EOGT
ENSG00000173575	1.067965386	2.55E-07	1.41E-05	CHD2
ENSG00000255864	1.065183732	0.001027177	0.014691723	AC069208.1
ENSG00000120709	1.064409306	3.58E-10	4.21E-08	FAM53C
ENSG00000134294	1.06355511	0.000168002	0.003552557	SLC38A2
ENSG00000116161	1.062308905	2.70E-05	0.000797341	CACYBP
ENSG00000120705	1.060830904	4.80E-10	5.53E-08	ETF1
ENSG00000043462	1.05921221	0.000725986	0.011132646	LCP2
ENSG00000122547	1.058669723	2.28E-08	1.82E-06	EEPD1
ENSG00000139146	1.056192551	2.44E-05	0.000739896	FAM60A
ENSG00000279520	1.05529793	2.13E-05	0.000657394	AC093525.8
ENSG00000154359	1.050723942	4.47E-05	0.001189589	LONRF1
ENSG00000105281	1.050311018	0.001586028	0.020341609	SLC1A5
ENSG00000197147	1.049236561	0.000260082	0.005024633	LRRC8B
ENSG00000127666	1.048084141	6.60E-07	3.25E-05	TICAM1
ENSG00000150457	1.04727029	4.03E-06	0.000158538	LATS2
ENSG00000143751	1.046434011	2.54E-08	1.99E-06	SDE2
ENSG00000117000	1.046172709	8.11E-07	3.90E-05	RLF
ENSG00000113368	1.046063984	0.000356527	0.006379618	LMNB1
ENSG00000188042	1.045615613	0.000807395	0.012156005	ARL4C
ENSG00000100647	1.04530303	7.60E-07	3.70E-05	SUSD6
ENSG00000280173	1.044407455	0.001628253	0.020739636	AC104447.1
ENSG00000141458	1.043822391	0.000274687	0.005232725	NPC1
ENSG00000141540	1.043614715	0.000672499	0.010530272	TTYH2
ENSG00000084112	1.042348978	3.32E-07	1.78E-05	SSH1
ENSG00000147894	1.04105323	7.52E-06	0.000271224	C9orf72
ENSG00000140450	1.040834618	4.15E-06	0.000162129	ARRDC4
ENSG00000111266	1.040182318	1.36E-06	6.07E-05	DUSP16
ENSG00000167657	1.039189467	2.91E-05	0.000841562	DAPK3
ENSG00000159200	1.03736602	3.94E-06	0.000155424	RCAN1
ENSG00000174951	1.036807097	0.004187843	0.041314743	FUT1
ENSG00000253669	1.03555013	0.00045421	0.007737965	AP003356.1
ENSG00000222043	1.034332084	0.005109599	0.047602752	AC079305.1
ENSG00000196756	1.030365603	0.000344774	0.006233802	SNHG17
ENSG00000156875	1.027274396	0.000164799	0.003506527	MFSD14A
ENSG00000135763	1.02668063	0.000581424	0.009375771	URB2
ENSG00000162889	1.025748443	5.80E-07	2.88E-05	MAPKAPK2
ENSG00000072274	1.022983627	8.39E-07	4.02E-05	TFRC
ENSG00000163877	1.021618206	3.27E-07	1.76E-05	SNIP1
ENSG00000125148	1.020161204	0.004355148	0.042464738	MT2A
ENSG00000134775	1.019920185	0.003070051	0.033093286	FHOD3
ENSG00000120438	1.019773258	1.55E-07	9.10E-06	TCP1
ENSG00000125430	1.018231942	9.91E-06	0.000341874	HS3ST3B1
ENSG00000277462	1.01807351	0.000531403	0.008745491	ZNF670
ENSG00000130940	1.016587588	1.86E-07	1.06E-05	CASZ1
ENSG00000175155	1.015769273	0.000163172	0.003490247	YPEL2
ENSG00000112096	1.013444852	0.003374669	0.035383183	SOD2

ENSG00000134954	1.011640677	4.99E-05	0.0013056	ETS1
ENSG00000134242	1.009161285	0.002838389	0.031284978	PTPN22
ENSG00000265206	1.008781146	0.003625786	0.037227792	AC004687.1
ENSG00000118689	1.007428091	0.000276658	0.005258734	FOXO3
ENSG00000268205	1.007290213	7.26E-05	0.001772946	AC005261.2
ENSG00000142549	1.006933252	0.000322853	0.005928341	IGLON5
ENSG00000116679	1.006281819	5.65E-06	0.000212039	IVNS1ABP
ENSG00000159840	1.006087462	2.91E-05	0.000842769	ZYX
ENSG00000146707	1.004971824	0.001875917	0.02312235	POMZP3
ENSG00000160588	1.004844369	1.98E-08	1.60E-06	MPZL3
ENSG00000145685	1.004700629	2.13E-05	0.000657394	LHFPL2
ENSG00000184731	1.004451503	1.34E-06	6.03E-05	FAM110C
ENSG00000154319	1.003410172	0.002360034	0.027570215	FAM167A
ENSG00000066697	1.003088431	0.001331063	0.017922037	MSANTD3
ENSG00000163811	1.002134297	3.04E-05	0.000871171	WDR43
ENSG00000107249	1.000367192	2.31E-05	0.000708782	GLIS3
ENSG00000185499	0.998257287	0.004041512	0.040300185	MUC1
ENSG00000055044	0.996880571	3.02E-08	2.29E-06	NOP58
ENSG00000279095	0.995346742	0.000137859	0.003057171	AC243964.3
ENSG00000115523	0.993175633	0.001237405	0.016996146	GNLY
ENSG00000178127	0.992133146	0.000460183	0.007814081	NDUFV2
ENSG00000132661	0.991468419	0.00014231	0.003137115	NXT1
ENSG00000225422	0.990075364	0.002518277	0.028665107	RBMS1P1
ENSG00000167553	0.989746902	0.000536846	0.008799112	TUBA1C
ENSG00000162407	0.988385475	1.60E-05	0.00051465	PLPP3
ENSG00000164543	0.988150486	0.000279438	0.005299919	STK17A
ENSG00000111012	0.98667017	0.003700992	0.037790611	CYP27B1
ENSG00000244274	0.986485817	0.003144125	0.033654073	DBNDD2
ENSG00000118707	0.983453897	5.28E-07	2.66E-05	TGIF2
ENSG00000115594	0.982669287	0.000231356	0.004599563	IL1R1
ENSG00000158480	0.979413257	3.60E-07	1.91E-05	SPATA2
ENSG00000110713	0.979314336	2.91E-08	2.22E-06	NUP98
ENSG00000225746	0.978819743	0.003073655	0.033104626	MEG8
ENSG00000050820	0.977070327	8.06E-05	0.00193745	BCAR1
ENSG00000146457	0.973857618	1.09E-07	6.71E-06	WTAP
ENSG00000161940	0.972885205	0.000926409	0.013518909	BCL6B
ENSG00000139926	0.972793105	0.005046257	0.047159759	FRMD6
ENSG00000176915	0.972446216	4.24E-05	0.001140536	ANKLE2
ENSG00000283154	0.97198347	8.31E-05	0.001989266	IQCJ-SCHIP1
ENSG00000276517	0.971881778	0.001537743	0.019849889	AL133243.3
ENSG00000109743	0.971629557	0.000109857	0.002513542	BST1
ENSG00000173276	0.970990419	0.00304976	0.033020851	ZBTB21
ENSG00000175182	0.96606618	2.68E-05	0.00079066	FAM131A
ENSG00000174574	0.966029025	3.07E-05	0.000876244	AKIRIN1
ENSG00000107937	0.963708518	1.24E-05	0.000414802	GTPBP4
ENSG00000180747	0.963704084	0.001697626	0.021428347	SMG1P3
ENSG00000154127	0.963379825	0.001499935	0.019497656	UBASH3B
ENSG00000198604	0.963211963	1.03E-06	4.73E-05	BAZ1A
ENSG00000077238	0.963105974	8.89E-05	0.00210758	IL4R
ENSG00000185015	0.960509073	0.000139861	0.003090143	CA13
ENSG00000133121	0.960173347	0.000156123	0.003371972	STARD13
ENSG00000148143	0.959072801	0.003275778	0.034691154	ZNF462
ENSG00000042493	0.957968919	0.005022995	0.047007821	CAPG
ENSG00000261150	0.957714783	0.002574566	0.029084871	EPPK1
ENSG00000109099	0.957671496	0.00455433	0.043832739	PMP22
ENSG00000135048	0.957466276	0.000623982	0.009926319	TMEM2
ENSG00000105639	0.956264925	0.00536601	0.04923143	JAK3
ENSG00000033327	0.95419499	3.50E-05	0.000979079	GAB2
ENSG00000119139	0.953990266	0.000910761	0.013328034	TJP2
ENSG00000160570	0.953232507	0.000288524	0.005417671	DEDD2
ENSG00000135269	0.953165035	0.000369624	0.006546346	TES
ENSG00000137094	0.950447411	1.81E-05	0.000571726	DNAJB5
ENSG00000121316	0.950091258	0.001488604	0.019398951	PLBD1
ENSG00000115762	0.948513364	8.70E-07	4.13E-05	PLEKHB2
ENSG00000100292	0.946243239	0.000507236	0.00839869	HMOX1
ENSG00000170776	0.945941318	2.47E-06	0.000103167	AKAP13
ENSG00000204160	0.943037735	2.91E-06	0.000119286	ZDHHC18
ENSG00000130147	0.941032911	0.00048195	0.00807274	SH3BP4
ENSG00000155367	0.940094326	0.002497683	0.028520562	PPM1J
ENSG00000144597	0.937955024	2.81E-06	0.00011584	EAF1

ENSG00000150907	0.937021217	6.99E-06	0.000254327	FOXO1
ENSG00000141543	0.935566171	3.14E-08	2.35E-06	EIF4A3
ENSG00000266094	0.934595094	1.36E-05	0.000446275	RASSF5
ENSG00000167703	0.933044719	0.001405033	0.018657022	SLC43A2
ENSG00000275464	0.932335691	0.000349358	0.006286017	FP565260.1
ENSG00000182378	0.931646672	1.79E-05	0.000567622	PLCXD1
ENSG00000138162	0.925344622	5.64E-05	0.001453769	TACC2
ENSG00000101265	0.923552583	0.003253199	0.034522495	RASSF2
ENSG00000168906	0.923463192	0.002222483	0.026246944	MAT2A
ENSG00000215301	0.922525174	4.58E-07	2.36E-05	DDX3X
ENSG00000102804	0.919916846	6.01E-06	0.000223513	TSC22D1
ENSG00000073792	0.919604135	0.004878209	0.046109292	IGF2BP2
ENSG00000185222	0.917436433	5.09E-05	0.001328351	TCEAL9
ENSG00000113369	0.917379402	0.002115519	0.025329601	ARRDC3
ENSG00000182718	0.914780087	0.000858907	0.012785546	ANXA2
ENSG00000272768	0.914282183	0.004744806	0.045084616	AC004854.2
ENSG00000177337	0.914231769	4.40E-05	0.001175001	DLGAP1-AS1
ENSG00000125810	0.912334618	0.004035156	0.040252267	CD93
ENSG00000169641	0.911824028	0.000109454	0.002510969	LUZP1
ENSG00000153187	0.91030495	1.93E-09	1.94E-07	HNRNPU
ENSG00000205189	0.909719571	3.66E-08	2.68E-06	ZBTB10
ENSG00000267107	0.908490575	0.000220704	0.004456339	PCAT19
ENSG00000164430	0.908241855	0.001746873	0.021885253	MB21D1
ENSG00000180667	0.906527955	0.000169964	0.003588183	YOD1
ENSG00000241839	0.905194413	0.000324161	0.005939747	PLEKHO2
ENSG00000176641	0.903263139	0.001385995	0.018479456	RNF152
ENSG00000125812	0.90265935	1.30E-08	1.10E-06	GZF1
ENSG00000104228	0.902338795	0.002295015	0.026931912	TRIM35
ENSG00000141582	0.901813385	0.003725242	0.037963524	CBX4
ENSG00000128918	0.900072318	0.000855772	0.012760381	ALDH1A2
ENSG00000006210	0.899479195	1.29E-05	0.000427267	CX3CL1
ENSG00000164687	0.899424106	0.001823451	0.022636964	FABP5
ENSG00000153714	0.899421102	0.000901867	0.013242698	LURAP1L
ENSG00000072401	0.898738824	2.08E-05	0.000649221	UBE2D1
ENSG00000134480	0.896783485	5.13E-06	0.000195491	CCNH
ENSG00000119669	0.896770512	0.000330951	0.006030099	IRF2BPL
ENSG00000103855	0.896366059	2.76E-05	0.000813114	CD276
ENSG00000165259	0.895536911	0.001201911	0.016623012	HDX
ENSG00000197852	0.894663089	0.000405157	0.007069411	FAM212B
ENSG00000122257	0.894591149	1.59E-07	9.30E-06	RBBP6
ENSG00000102755	0.894540326	0.004634691	0.044356955	FLT1
ENSG00000165527	0.892462825	5.18E-07	2.62E-05	ARF6
ENSG00000101544	0.891041587	2.78E-08	2.16E-06	ADNP2
ENSG00000095739	0.889956895	0.000539465	0.008830895	BAMBI
ENSG00000113742	0.889379198	2.55E-10	3.10E-08	CPEB4
ENSG00000129355	0.887295044	0.001299611	0.017607745	CDKN2D
ENSG00000127954	0.886008314	0.004342024	0.04242534	STEAP4
ENSG00000158122	0.885250969	5.86E-05	0.001498681	AAED1
ENSG00000134686	0.884126587	0.000645326	0.01018469	PHC2
ENSG00000136603	0.883514432	1.60E-06	7.02E-05	SKIL
ENSG00000145414	0.883033762	1.61E-05	0.000516309	NAF1
ENSG00000157111	0.881401033	4.82E-05	0.00127043	TMEM171
ENSG00000129315	0.878509131	4.30E-07	2.25E-05	CCNT1
ENSG00000171729	0.877929766	0.002061988	0.024855729	TMEM51
ENSG00000060558	0.877861629	0.00219777	0.026026173	GNA15
ENSG00000110880	0.877434975	5.38E-08	3.68E-06	CORO1C
ENSG00000196338	0.873980734	0.003093197	0.033246103	NLGN3
ENSG00000268061	0.871151513	0.002841479	0.031292451	NAPA-AS1
ENSG00000204524	0.869081204	0.000190129	0.003937031	ZNF805
ENSG00000166949	0.868752884	0.000587981	0.009453534	SMAD3
ENSG00000132819	0.868732792	0.000130991	0.002917325	RBM38
ENSG00000204396	0.868466875	0.001253645	0.017173693	VWA7
ENSG00000152409	0.865703177	1.33E-05	0.00044041	JMY
ENSG00000011198	0.864819834	6.78E-05	0.001680661	ABHD5
ENSG00000089159	0.864451584	0.000165317	0.003512957	PXN
ENSG00000203497	0.864372484	0.003857674	0.038915711	PDCD4-AS1
ENSG00000196954	0.863545832	0.001167774	0.016233801	CASP4
ENSG00000198042	0.861844143	0.000543236	0.008869193	MAK16
ENSG00000102034	0.861665346	0.001157022	0.016139833	ELF4
ENSG00000182827	0.861145247	9.76E-05	0.002279015	ACBD3

ENSG00000112031	0.860880177	3.35E-05	0.000944077	MTRF1L
ENSG00000162702	0.860208618	3.22E-08	2.40E-06	ZNF281
ENSG00000184730	0.859113618	0.004274653	0.041916151	APOBR
ENSG00000163694	0.858152847	2.96E-05	0.000851387	RBM47
ENSG00000260971	0.856803101	0.001459383	0.019120472	AC119674.1
ENSG00000156675	0.855572276	0.001572292	0.020215401	RAB11FIP1
ENSG00000113916	0.855344364	2.86E-05	0.000834287	BCL6
ENSG00000170027	0.854866917	1.07E-06	4.92E-05	YWHAG
ENSG00000169946	0.854452393	0.001395474	0.018564596	ZFPM2
ENSG00000163162	0.85397609	0.000107982	0.002483779	RNF149
ENSG00000033867	0.852847017	0.00275375	0.030533674	SLC4A7
ENSG00000122644	0.85231899	0.001875178	0.02312235	ARL4A
ENSG00000171612	0.851161567	0.000527184	0.008690161	SLC25A33
ENSG00000198342	0.84977445	0.000309892	0.005726819	ZNF442
ENSG00000127311	0.849726404	0.001503796	0.019518508	HELB
ENSG00000267493	0.849276687	0.002154498	0.025640233	CIRBP-AS1
ENSG00000183386	0.849232205	0.002385137	0.027741971	FHL3
ENSG00000165868	0.848140182	0.0045179	0.043625392	HSPA12A
ENSG00000012660	0.847557177	0.002127967	0.025447717	ELOVL5
ENSG00000258738	0.847218292	0.001590021	0.020369981	AL121603.2
ENSG00000165175	0.846713693	4.97E-05	0.001304273	MID1IP1
ENSG00000159459	0.846544063	3.39E-05	0.000954329	UBR1
ENSG00000129911	0.843888631	0.00055023	0.008956261	KLF16
ENSG00000232533	0.843627098	0.000268298	0.005145057	AC093673.1
ENSG00000145365	0.842885736	0.002878416	0.031605322	TIFA
ENSG00000139946	0.840256809	0.002665499	0.029887224	PELI2
ENSG00000141664	0.839238807	1.71E-07	9.77E-06	ZCCHC2
ENSG00000119986	0.839006171	0.000215607	0.004367016	AVPI1
ENSG00000164938	0.837305282	0.001282713	0.017424745	TP53INP1
ENSG00000101596	0.835198414	0.000287879	0.005416466	SMCHD1
ENSG00000101421	0.834624962	4.28E-05	0.001148378	CHMP4B
ENSG00000095951	0.83422769	0.000747885	0.0114212	HIVEP1
ENSG00000198858	0.833717448	4.36E-05	0.001167459	R3HDM4
ENSG00000111817	0.833281415	6.89E-05	0.001700142	DSE
ENSG00000158019	0.833197957	4.37E-05	0.001168044	BABAM2
ENSG00000080371	0.83258769	1.51E-07	8.87E-06	RAB21
ENSG00000139372	0.830800537	0.000802545	0.012099271	TDG
ENSG00000106665	0.830570438	0.001209136	0.0166901	CLIP2
ENSG00000112245	0.829424666	0.000315673	0.005812949	PTP4A1
ENSG00000030110	0.828039288	0.000164408	0.003506526	BAK1
ENSG00000184371	0.827304449	0.000126454	0.002830863	CSF1
ENSG00000027697	0.826977508	0.000805645	0.012138944	IFNGR1
ENSG00000179604	0.826789655	0.000145898	0.003199895	CDC42EP4
ENSG00000204577	0.825960865	0.004342932	0.04242534	LILRB3
ENSG00000170989	0.824980979	0.000438957	0.00752754	S1PR1
ENSG00000080561	0.82467565	0.000639277	0.010117102	MID2
ENSG00000065989	0.824001928	0.002599491	0.029299756	PDE4A
ENSG00000136527	0.823858967	2.72E-07	1.50E-05	TRA2B
ENSG00000140968	0.823522909	2.39E-05	0.000728346	IRF8
ENSG00000065802	0.820518814	5.58E-05	0.001443142	ASB1
ENSG00000188313	0.820393615	0.002136573	0.025487593	PLSCR1
ENSG00000112578	0.819494524	0.000903614	0.013253343	BYSL
ENSG00000184009	0.817809369	0.000362826	0.00647893	ACTG1
ENSG00000163376	0.817290996	0.00315111	0.03370103	KBTBD8
ENSG00000205710	0.815259056	0.001018413	0.014590697	C17orf107
ENSG00000240891	0.813471271	0.000543501	0.008869193	PLCXD2
ENSG00000272009	0.81333191	0.00383284	0.038755601	AL121944.1
ENSG00000115306	0.812655652	0.000262591	0.005062105	SPTBN1
ENSG00000115541	0.8122345	0.000365328	0.006506898	HSPE1
ENSG00000101773	0.811812227	0.001408914	0.018669909	RBBP8
ENSG00000198742	0.810838658	2.17E-06	9.17E-05	SMURF1
ENSG00000152503	0.810140914	0.002201322	0.026056345	TRIM36
ENSG00000063660	0.809552529	9.81E-05	0.002288481	GPC1
ENSG00000137393	0.809190634	0.000280897	0.005319827	RNF144B
ENSG00000188636	0.808835742	0.000232149	0.004601483	RTL6
ENSG00000141551	0.806572656	5.20E-06	0.000197199	CSNK1D
ENSG00000073008	0.806140158	0.000238693	0.004706029	PVR
ENSG00000161011	0.804753915	5.33E-07	2.67E-05	SQSTM1
ENSG00000276023	0.804204337	4.93E-05	0.0012964	DUSP14
ENSG00000050327	0.803381296	0.000819993	0.012269635	ARHGEF5

ENSG00000135241	0.802968637	1.97E-05	0.000616257	PNPLA8
ENSG00000197461	0.802408055	0.001453547	0.019095559	PDGFA
ENSG00000109814	0.802030068	0.000886029	0.013091632	UGDH
ENSG00000078269	0.800167754	0.001796662	0.022325764	SYNJ2
ENSG00000165006	0.797495683	5.13E-07	2.61E-05	UBAP1
ENSG00000169991	0.796762129	0.002862917	0.031461776	IFFO2
ENSG00000125753	0.796410739	0.001343644	0.018035273	VASP
ENSG00000171552	0.796122542	0.001399509	0.018602287	BCL2L1
ENSG00000131941	0.795539781	7.18E-05	0.001759474	RHPN2
ENSG00000152684	0.79475666	5.18E-06	0.000196601	PELO
ENSG00000115604	0.791997896	0.001442963	0.018992171	IL18R1
ENSG00000147439	0.787572046	4.15E-06	0.000162129	BIN3
ENSG00000007944	0.786691304	0.000893364	0.013140151	MYLIP
ENSG00000168175	0.784459902	6.81E-08	4.42E-06	MAPK1IP1L
ENSG00000198369	0.783008535	0.000818046	0.012254633	SPRED2
ENSG00000157800	0.780418266	0.000413559	0.007182202	SLC37A3
ENSG00000070961	0.779414027	4.55E-05	0.001208816	ATP2B1
ENSG00000137462	0.778780009	0.003866246	0.038983195	TLR2
ENSG00000064666	0.778735883	0.000878253	0.013021226	CNN2
ENSG00000076641	0.777429112	0.000305067	0.005682176	PAG1
ENSG00000135605	0.773947526	0.00294556	0.032206282	TEC
ENSG00000171174	0.771383308	3.21E-07	1.73E-05	RBKS
ENSG00000198223	0.770531677	0.005364504	0.04923143	CSF2RA
ENSG00000153721	0.76864521	4.32E-05	0.001158385	CNKSR3
ENSG00000182831	0.768475094	3.07E-08	2.31E-06	C16orf72
ENSG00000147144	0.766670001	0.004349112	0.042453745	CCDC120
ENSG00000160908	0.766504931	6.45E-06	0.000237188	ZNF394
ENSG00000090020	0.765945886	0.004335603	0.042417626	SLC9A1
ENSG00000142634	0.765279404	0.001691599	0.021368178	EFHD2
ENSG00000049130	0.76521079	0.00446166	0.043250965	KITLG
ENSG00000162775	0.76455146	1.39E-09	1.45E-07	RBM15
ENSG00000249673	0.763887049	0.000192506	0.003973559	NOP14-AS1
ENSG00000154124	0.763340703	6.63E-05	0.001651695	OTULIN
ENSG00000154380	0.760803247	0.000577889	0.009330372	ENAH
ENSG00000163349	0.760494776	2.72E-05	0.000801169	HIPK1
ENSG00000124201	0.755872718	6.45E-05	0.001617268	ZNFX1
ENSG00000133703	0.755203191	9.15E-05	0.002155276	KRAS
ENSG00000134470	0.755178277	0.004228387	0.041603991	IL15RA
ENSG00000168884	0.754204317	0.000100134	0.002332204	TNIP2
ENSG00000272886	0.752693273	3.30E-05	0.000933366	DCP1A
ENSG00000164924	0.750949712	4.14E-05	0.001119523	YWHAZ
ENSG00000196935	0.75092714	0.003375217	0.035383183	SRGAP1
ENSG00000132603	0.750787095	0.0006631	0.010401929	NIP7
ENSG00000153250	0.750290446	0.000345889	0.006249616	RBMS1
ENSG00000037897	0.749968976	0.002016155	0.024444471	METTL1
ENSG00000166025	0.748832509	0.003302548	0.03488921	AMOTL1
ENSG00000179119	0.748653471	7.28E-06	0.000263669	SPTY2D1
ENSG00000008083	0.748631951	4.90E-05	0.001288297	JARID2
ENSG00000121039	0.748423377	0.000816518	0.012238815	RDH10
ENSG00000124171	0.748225269	0.000110588	0.002528034	PARD6B
ENSG00000171763	0.744463453	0.000588804	0.009456077	SPATA5L1
ENSG00000124789	0.744420879	1.40E-05	0.000459843	NUP153
ENSG00000141568	0.744107476	2.15E-06	9.09E-05	FOXK2
ENSG00000131669	0.743239072	0.000213396	0.004335767	NINJ1
ENSG00000175895	0.742364592	5.34E-05	0.001389304	PLEKHF2
ENSG00000141574	0.742045329	0.003530794	0.036545592	SECTM1
ENSG00000023287	0.740190394	3.04E-05	0.000870869	RB1CC1
ENSG00000205534	0.739833589	0.000748498	0.011423797	SMG1P2
ENSG00000173545	0.738412379	1.61E-05	0.000517389	ZNF622
ENSG00000136160	0.738080762	0.002005241	0.024334883	EDNRB
ENSG00000115738	0.738022109	0.002619904	0.029460163	ID2
ENSG00000102572	0.73755523	0.000231343	0.004599563	STK24
ENSG00000025772	0.737252513	0.000671723	0.010524475	TOMM34
ENSG00000255112	0.736415667	1.74E-07	9.97E-06	CHMP1B
ENSG00000158186	0.736312502	0.00060435	0.009661408	MRAS
ENSG00000142528	0.735835035	9.19E-06	0.000322273	ZNF473
ENSG00000147454	0.735422842	0.001302033	0.017616053	SLC25A37
ENSG00000173598	0.734134588	2.11E-05	0.00065275	NUDT4
ENSG00000119326	0.734121737	0.000122914	0.002761142	CTNNAL1
ENSG00000198824	0.733580522	0.000627343	0.009967563	CHAMP1

ENSG00000185989	0.733487899	0.003446344	0.035965574	RASA3
ENSG00000147324	0.732963572	0.00405232	0.040345929	MFHAS1
ENSG00000115183	0.731316129	0.001965192	0.023983477	TANC1
ENSG00000173039	0.729642255	1.40E-07	8.27E-06	RELA
ENSG00000135334	0.728996851	6.61E-08	4.31E-06	AKIRIN2
ENSG00000163938	0.728709248	0.002955312	0.032285694	GNL3
ENSG00000164916	0.72753979	0.001590595	0.020369981	FOXK1
ENSG00000090061	0.727512126	0.000397113	0.006947746	CCNK
ENSG00000204152	0.725767172	2.92E-05	0.000842806	TIMM23B
ENSG00000095397	0.725749392	0.005226663	0.048317303	WHRN
ENSG00000167491	0.723940857	0.000357593	0.006394288	GATAD2A
ENSG00000204592	0.723776636	6.39E-05	0.001606311	HLA-E
ENSG00000118939	0.723636455	0.005218196	0.048301289	UCHL3
ENSG00000125733	0.723344325	0.003054852	0.033023414	TRIP10
ENSG00000119280	0.722896663	0.002741685	0.030451949	C1orf198
ENSG00000241878	0.722076297	0.000549353	0.008947991	PISD
ENSG00000170606	0.721283354	3.98E-05	0.001084112	HSPA4
ENSG00000251022	0.720310513	0.002499096	0.028520562	THAP9-AS1
ENSG00000163754	0.72014823	0.00071041	0.010953678	GYG1
ENSG00000116273	0.719436123	5.38E-07	2.69E-05	PHF13
ENSG00000139793	0.719371464	0.001308483	0.017673036	MBNL2
ENSG00000171940	0.718870565	0.000385967	0.006784755	ZNF217
ENSG00000114648	0.718733427	4.30E-06	0.000167643	KLHL18
ENSG00000137502	0.717703224	0.000154125	0.003343575	RAB30
ENSG00000104164	0.71742035	0.000311724	0.005752483	BLOC1S6
ENSG00000146374	0.71720777	0.000886546	0.013091816	RSPO3
ENSG00000139154	0.716801673	4.14E-06	0.000162129	AEBP2
ENSG00000260805	0.716780814	0.004183866	0.041291211	AC092803.2
ENSG00000101361	0.716419518	0.001715603	0.021597787	NOP56
ENSG00000123395	0.71595639	0.000246389	0.004819583	ATG101
ENSG00000059758	0.715724096	0.000138369	0.003065854	CDK17
ENSG00000130779	0.715645018	0.001527272	0.019754031	CLIP1
ENSG00000109332	0.715610602	3.43E-07	1.83E-05	UBE2D3
ENSG00000168575	0.715581241	0.003996883	0.039993491	SLC20A2
ENSG00000127080	0.715291591	0.004606697	0.044150368	IPPK
ENSG00000170185	0.713955164	0.000279376	0.005299919	USP38
ENSG00000110104	0.713450298	0.002572583	0.029084871	CCDC86
ENSG00000244513	0.712412891	0.003990765	0.039993491	AC109587.1
ENSG00000151748	0.712399538	3.74E-06	0.000149088	SAV1
ENSG00000165355	0.711659328	7.94E-05	0.001912481	FBXO33
ENSG00000104450	0.711262445	0.00102391	0.014661348	SPAG1
ENSG00000121274	0.710322665	0.000665513	0.010433472	PAPD5
ENSG00000124831	0.709794907	1.86E-05	0.000584727	LRRFIP1
ENSG00000174485	0.708050364	0.001769568	0.022105548	DENND4A
ENSG00000168994	0.707325473	0.001274576	0.017341458	PXDC1
ENSG00000008294	0.70717773	7.71E-05	0.001869013	SPAG9
ENSG00000166333	0.702803139	0.003351991	0.035239366	ILK
ENSG00000179833	0.699796451	0.002451283	0.028179437	SERTAD2
ENSG00000160785	0.698354774	0.000309294	0.005726819	SLC25A44
ENSG00000188295	0.698161582	0.005163535	0.047979659	ZNF669
ENSG00000112242	0.696895059	0.000916719	0.013400114	E2F3
ENSG00000225190	0.696488435	7.61E-06	0.000273396	PLEKHM1
ENSG00000150977	0.696317456	5.86E-05	0.001498681	RILPL2
ENSG00000251562	0.695923321	0.000689712	0.010735008	MALAT1
ENSG00000105849	0.694586864	0.000235192	0.004654694	TWISTNB
ENSG00000118496	0.694117237	1.83E-05	0.000575873	FBXO30
ENSG00000134697	0.693697932	0.000276567	0.005258734	GNL2
ENSG00000104825	0.692556394	0.000486514	0.008128198	NFKBIB
ENSG00000162636	0.691835746	0.001587385	0.020348954	FAM102B
ENSG00000107554	0.691396041	0.00050708	0.00839869	DNMBP
ENSG00000138670	0.690633879	0.001148179	0.016033717	RASGEF1B
ENSG00000136950	0.690323156	0.000779471	0.011820081	ARPC5L
ENSG00000168394	0.68956481	0.002994374	0.032550559	TAP1
ENSG00000137575	0.688086023	3.59E-06	0.000144336	SDCBP
ENSG00000153179	0.687103936	0.000302739	0.005642869	RASSF3
ENSG00000072201	0.686730195	0.002399286	0.027862009	LNX1
ENSG00000166233	0.686673703	9.58E-06	0.000332509	ARIH1
ENSG00000237296	0.686520515	0.000236453	0.004676073	SMG1P1
ENSG00000198900	0.685306612	7.67E-05	0.001864013	TOP1
ENSG00000075624	0.684959123	5.02E-06	0.000191913	ACTB

ENSG00000135766	0.684892509	0.00045592	0.007751836	EGLN1
ENSG00000106546	0.684174707	5.57E-05	0.001441497	AHR
ENSG00000096717	0.683420517	4.57E-07	2.36E-05	SIRT1
ENSG00000120690	0.683383127	0.000152026	0.003311896	ELF1
ENSG00000086062	0.682051543	0.001674409	0.021210864	B4GALT1
ENSG00000071054	0.681014481	0.000809352	0.012166533	MAP4K4
ENSG00000111641	0.680660235	0.001612092	0.020553971	NOP2
ENSG00000221914	0.679955909	2.58E-06	0.000106892	PPP2R2A
ENSG00000140598	0.679216228	0.002444854	0.028130461	EFL1
ENSG00000101216	0.677868843	0.000112607	0.002565136	GMEB2
ENSG00000185650	0.677334573	0.000588955	0.009456077	ZFP36L1
ENSG00000068028	0.674663182	0.003072601	0.033104626	RASSF1
ENSG00000116016	0.673023524	0.000251401	0.004882372	EPAS1
ENSG00000080845	0.671644171	1.49E-05	0.000483891	DLGAP4
ENSG00000151694	0.670184283	0.004835206	0.045752825	ADAM17
ENSG00000236753	0.667824594	0.002471229	0.028299169	MKLN1-AS
ENSG00000083799	0.667260145	0.001094211	0.015480036	CYLD
ENSG00000171466	0.667129707	0.002528799	0.028744545	ZNF562
ENSG00000187079	0.664863852	0.001945179	0.02377278	TEAD1
ENSG00000135913	0.664698898	0.004600015	0.044150368	USP37
ENSG00000115946	0.664556706	0.002959982	0.032323103	PNO1
ENSG00000166833	0.662776527	0.001553477	0.020013168	NAV2
ENSG00000142961	0.662682583	0.000145307	0.003189632	MOB3C
ENSG00000233230	0.662159355	0.001350915	0.018114123	AC079807.2
ENSG00000174749	0.661901139	0.000426124	0.007351201	C4orf32
ENSG00000110046	0.6608442	0.002906544	0.031873706	ATG2A
ENSG00000170677	0.660724258	0.000106214	0.002445283	SOCS6
ENSG00000119729	0.660145956	0.002748637	0.030503049	RHOQ
ENSG00000116044	0.659879658	2.40E-06	0.00010087	NFE2L2
ENSG00000110852	0.659430018	0.001221028	0.01680679	CLEC2B
ENSG00000115816	0.65922673	0.003347817	0.035209751	CEBPZ
ENSG00000161638	0.658634062	0.002065553	0.024867801	ITGA5
ENSG00000254087	0.658509438	0.001258099	0.017189382	LYN
ENSG00000170315	0.658291319	0.003702923	0.037795451	UBB
ENSG00000114019	0.657470534	0.002275424	0.026726193	AMOTL2
ENSG00000125505	0.656285579	0.000656452	0.010322607	MBOAT7
ENSG00000065613	0.654478895	2.86E-05	0.000834287	SLK
ENSG00000170852	0.65309168	2.10E-05	0.000652057	KBTBD2
ENSG00000062194	0.652084285	1.84E-06	7.99E-05	GPBP1
ENSG00000188785	0.650695917	3.72E-05	0.001028726	ZNF548
ENSG00000132467	0.649728218	0.001423077	0.01880955	UTP3
ENSG00000076356	0.648518454	0.000575542	0.009321476	PLXNA2
ENSG00000127314	0.64817067	0.000105534	0.002436113	RAP1B
ENSG00000186432	0.645543938	5.89E-05	0.001501246	KPNA4
ENSG00000196850	0.645125858	0.001211527	0.016702587	PPTC7
ENSG00000130826	0.642402109	0.001528584	0.01976116	DKC1
ENSG00000100591	0.642277309	0.000448442	0.007659819	AHSA1
ENSG00000130766	0.641502744	0.003320133	0.035056605	SESN2
ENSG00000141867	0.640985433	9.51E-07	4.44E-05	BRD4
ENSG00000104142	0.640914402	0.003123979	0.033482187	VPS18
ENSG00000135503	0.640123512	0.001968365	0.024010905	ACVR1B
ENSG00000225648	0.639827038	0.001549138	0.019967186	SBDSP1
ENSG00000154582	0.639468438	0.002662801	0.029869877	ELOC
ENSG00000130830	0.639406547	0.000166143	0.003524728	MPP1
ENSG00000179134	0.638663221	6.78E-06	0.00024737	SAMD4B
ENSG00000213719	0.638131942	0.00151357	0.019596355	CLIC1
ENSG00000096063	0.637949024	9.30E-05	0.002180862	SRPK1
ENSG00000088727	0.637449527	0.002846125	0.031330318	KIF9
ENSG00000162734	0.637441641	0.002457914	0.028205678	PEA15
ENSG00000065491	0.637397026	2.90E-05	0.000841562	TBC1D22B
ENSG00000112406	0.636151758	6.26E-05	0.001583497	HECA
ENSG00000118263	0.635401199	0.00069571	0.010802154	KLF7
ENSG00000146966	0.634593996	0.003563342	0.03671742	DENND2A
ENSG00000112029	0.634317634	0.00203073	0.024597737	FBXO5
ENSG00000159128	0.633797453	0.001413688	0.018714055	IFNGR2
ENSG00000264522	0.633645821	9.16E-05	0.00215571	OTUD7B
ENSG00000126391	0.632352851	0.004342113	0.04242534	FRMD8
ENSG00000105447	0.632319134	0.00352143	0.036517595	GRWD1
ENSG00000160584	0.631762172	1.73E-05	0.000552721	SIK3
ENSG00000182253	0.63150027	0.004031429	0.040245336	SYNM

ENSG00000177105	0.631444702	0.00317613	0.03392666	RHOG
ENSG00000175606	0.631098122	0.001603714	0.020504675	TMEM70
ENSG00000101493	0.630989732	0.000120958	0.002719563	ZNF516
ENSG00000120278	0.63096875	0.000286048	0.005386346	PLEKHG1
ENSG00000144566	0.630952792	0.000394245	0.006902214	RAB5A
ENSG00000100664	0.630949344	0.000657444	0.010331943	EIF5
ENSG00000173744	0.63036595	0.000492845	0.008202281	AGFG1
ENSG00000172071	0.628554334	0.004880847	0.04611134	EIF2AK3
ENSG00000164933	0.628416241	0.000229617	0.004586321	SLC25A32
ENSG00000097007	0.628084174	0.003381645	0.035393364	ABL1
ENSG00000118200	0.627276083	0.001032935	0.014749884	CAMSAP2
ENSG00000163961	0.626929296	0.000685756	0.010686259	RNF168
ENSG00000162419	0.626354542	6.60E-05	0.00164909	GMEB1
ENSG00000141076	0.62250059	0.002098582	0.025173279	UTP4
ENSG00000092330	0.621104391	0.000111342	0.00254302	TINF2
ENSG00000113575	0.619765223	2.41E-06	0.00010131	PPP2CA
ENSG00000204590	0.618668598	0.000299824	0.005595881	GNL1
ENSG00000145819	0.618558995	0.004044845	0.040302437	ARHGAP26
ENSG00000100345	0.618499524	0.000164879	0.003506527	MYH9
ENSG00000136451	0.617614461	4.83E-06	0.000185499	VEZF1
ENSG00000117523	0.616927841	0.001036118	0.014770947	PRRC2C
ENSG00000197063	0.616587228	0.000439675	0.00753488	MAFG
ENSG00000072364	0.615995934	1.02E-05	0.000349592	AFF4
ENSG00000205808	0.615710543	0.001115667	0.015680988	PLPP6
ENSG00000272391	0.615658474	2.52E-05	0.000756921	POM121C
ENSG00000168495	0.614917281	0.001494207	0.019442964	POLR3D
ENSG00000147526	0.61474537	0.003148175	0.033683522	TACC1
ENSG00000197321	0.61410634	0.003996856	0.039993491	SVIL
ENSG00000087157	0.614051566	0.002450297	0.028179437	PGS1
ENSG00000107362	0.613519841	0.001034227	0.014752095	ABHD17B
ENSG00000065809	0.613447898	5.79E-05	0.001484262	FAM107B
ENSG00000006712	0.611384246	0.002046121	0.024715391	PAF1
ENSG00000169756	0.610740807	8.55E-05	0.002039484	LIMS1
ENSG00000173812	0.609951302	3.16E-05	0.000896412	EIF1
ENSG00000070444	0.609667925	0.000365857	0.006506898	MNT
ENSG00000144824	0.609545265	0.005291052	0.048733271	PHLDB2
ENSG00000106608	0.609136904	0.004499522	0.043515498	URGCP
ENSG00000155366	0.609056853	0.004250708	0.04172867	RHOC
ENSG00000069020	0.606844967	0.004241116	0.04165804	MAST4
ENSG00000145901	0.606557797	0.002574835	0.029084871	TNIP1
ENSG00000148737	0.606161824	0.004701679	0.044816225	TCF7L2
ENSG00000117143	0.605372991	0.00238201	0.027739506	UAP1
ENSG00000196470	0.605292692	0.000232589	0.004606689	SIAH1
ENSG00000107438	0.605259029	0.000229095	0.004579424	PDLIM1
ENSG00000092978	0.605150677	0.000163324	0.00349061	GPATCH2
ENSG00000102226	0.604573904	0.000844426	0.012598888	USP11
ENSG00000104765	0.603522392	0.001032267	0.014748464	BNIP3L
ENSG00000101558	0.603469615	4.31E-07	2.25E-05	VAPA
ENSG00000005483	0.602739197	0.00248327	0.028421222	KMT2E
ENSG00000196313	0.602590935	0.000182132	0.003798708	POM121
ENSG00000151327	0.599983746	6.23E-05	0.001579545	FAM177A1
ENSG00000067182	0.59923657	0.000200715	0.004126585	TNFRSF1A
ENSG00000128245	0.59880592	3.46E-05	0.000969309	YWHAH
ENSG00000116604	0.598383978	9.23E-05	0.002164372	MEF2D
ENSG00000089195	0.59635754	0.002358681	0.027566817	TRMT6
ENSG00000169635	0.593520929	3.01E-05	0.000864726	HIC2
ENSG00000153317	0.592765739	0.002533636	0.028756655	ASAP1
ENSG00000168769	0.592391563	0.002471515	0.028299169	TET2
ENSG00000136802	0.591362163	0.000228828	0.004577625	LRRC8A
ENSG00000185291	0.591103248	0.00449981	0.043515498	IL3RA
ENSG00000087263	0.589539028	0.000999726	0.014402491	OGFOD1
ENSG00000174840	0.588661953	0.001271378	0.017316096	PDE12
ENSG00000083168	0.588609395	0.000868391	0.012911909	KAT6A
ENSG00000197622	0.588165519	0.000434617	0.007467928	CDC42SE1
ENSG00000083896	0.586162449	5.06E-08	3.52E-06	YTHDC1
ENSG00000148175	0.586048925	0.000785109	0.011884741	STOM
ENSG00000141522	0.585996672	0.002405516	0.027862009	ARHGDIA
ENSG00000172939	0.585130608	0.000118136	0.002669998	OXSR1
ENSG00000164134	0.585005575	0.003980308	0.039920008	NAA15
ENSG00000166226	0.584288845	8.12E-05	0.001947373	CCT2

ENSG00000123094	0.581494882	0.003912202	0.039328166	RASSF8
ENSG00000196449	0.580568158	0.004127154	0.040933919	YRDC
ENSG00000204574	0.579630845	1.60E-06	7.02E-05	ABCF1
ENSG00000196839	0.577442035	0.002398681	0.027862009	ADA
ENSG00000121579	0.575463729	0.000733126	0.011228866	NAA50
ENSG00000217128	0.575091555	0.000589356	0.009456674	FNIP1
ENSG00000105355	0.574585235	0.005077201	0.047368952	PLIN3
ENSG00000132669	0.57427094	0.002922642	0.031982654	RIN2
ENSG00000211455	0.574047981	0.000365101	0.006506898	STK38L
ENSG00000148572	0.573987581	0.002695098	0.030076132	NRBF2
ENSG00000244462	0.573423961	0.001068539	0.015174778	RBM12
ENSG00000171456	0.572072724	0.001192468	0.016527656	ASXL1
ENSG00000157764	0.571279684	6.04E-05	0.001535646	BRAF
ENSG00000144580	0.569928939	0.000583567	0.009398651	CNOT9
ENSG00000139112	0.569449594	0.000181722	0.003793199	GABARAPL1
ENSG00000136891	0.569208299	0.001269489	0.017299455	TEX10
ENSG00000039523	0.569199525	0.000940537	0.013694267	RIPOR1
ENSG00000205339	0.568847381	0.002679948	0.029967533	IPO7
ENSG00000134758	0.568289373	1.16E-06	5.33E-05	RNF138
ENSG00000093167	0.567635253	0.00023152	0.004599563	LRRFIP2
ENSG00000069956	0.567311594	0.002080534	0.024996733	MAPK6
ENSG00000136807	0.566393542	0.001141397	0.01595619	CDK9
ENSG00000083937	0.565875424	0.002835459	0.031265968	CHMP2B
ENSG00000089737	0.565074713	4.09E-05	0.001108798	DDX24
ENSG00000140750	0.564834613	0.00147154	0.019224867	ARHGAP17
ENSG00000132823	0.564400901	5.87E-06	0.000219206	OSER1
ENSG00000273559	0.56415942	0.000684365	0.010670996	CWC25
ENSG00000135049	0.563141173	0.002128326	0.025447717	AGTPBP1
ENSG00000163347	0.561595043	0.001817406	0.022572718	CLDN1
ENSG00000178209	0.560069149	0.004068429	0.040490774	PLEC
ENSG00000130202	0.55977682	0.004883578	0.04611134	NECTIN2
ENSG00000145860	0.559313898	0.003892096	0.039171576	RNF145
ENSG00000137055	0.558441229	0.000365983	0.006506898	PLAA
ENSG00000188786	0.557265727	0.003197976	0.034103854	MTF1
ENSG00000107643	0.555322806	0.002871818	0.031546227	MAPK8
ENSG00000234518	0.554263928	0.003840446	0.038817382	PTGES3P1
ENSG00000131979	0.554034855	0.004774104	0.045290271	GCH1
ENSG00000146909	0.552494165	0.000819954	0.012269635	NOM1
ENSG00000117758	0.552183173	0.000153637	0.003338998	STX12
ENSG00000091527	0.552084856	0.000193796	0.003997002	CDV3
ENSG00000117868	0.551518052	0.000892742	0.013140151	ESYT2
ENSG00000071127	0.5507976	0.000247557	0.004833046	WDR1
ENSG00000169180	0.550659626	0.002538319	0.028788443	XPO6
ENSG00000068878	0.550507479	0.000139532	0.003086685	PSME4
ENSG00000186591	0.54968695	3.09E-06	0.00012583	UBE2H
ENSG00000186416	0.548484294	0.000382847	0.006739036	NKRF
ENSG00000108256	0.54648304	0.001849376	0.02289309	NUFIP2
ENSG00000116752	0.545552165	2.78E-05	0.000817796	BCAS2
ENSG00000137947	0.545288879	0.000887147	0.013093239	GTF2B
ENSG00000171316	0.543232215	0.001192184	0.016527656	CHD7
ENSG00000183624	0.542814387	0.005267382	0.048593542	HMCES
ENSG00000163558	0.541986112	0.004241908	0.04165804	PRKCI
ENSG00000116560	0.539721177	0.004462454	0.043250965	SFPQ
ENSG00000173933	0.539240146	0.00021474	0.004352841	RBM4
ENSG00000146676	0.539189518	7.88E-05	0.001899039	PURB
ENSG00000109111	0.538499451	9.35E-06	0.000327095	SUPT6H
ENSG00000197971	0.538097827	0.004647471	0.044446475	MBP
ENSG00000113712	0.53785088	0.00178983	0.022263618	CSNK1A1
ENSG00000124228	0.537173129	0.000237045	0.004684212	DDX27
ENSG00000154642	0.534650769	0.003332409	0.035147437	C21orf91
ENSG00000113649	0.53426336	0.005214254	0.048300261	TCERG1
ENSG00000105821	0.534163523	0.002805544	0.031001975	DNAJC2
ENSG00000178764	0.533871288	0.001444641	0.018997799	ZHX2
ENSG00000154447	0.532902735	0.00253199	0.028750556	SH3RF1
ENSG00000112941	0.532875114	0.000376459	0.006644627	PAPD7
ENSG00000107959	0.532519859	0.00501897	0.047007821	PITRM1
ENSG00000143153	0.53232599	0.000229929	0.004589032	ATP1B1
ENSG00000155096	0.531187365	0.000143411	0.003156006	AZIN1
ENSG00000133773	0.528137666	0.000495256	0.008231842	CCDC59
ENSG00000140743	0.527787982	0.000909429	0.013316055	CDR2

ENSG00000169446	0.52718104	0.001753909	0.021952203	MMGT1
ENSG00000163788	0.526923713	6.63E-05	0.001651695	SNRK
ENSG00000166974	0.524450517	2.10E-05	0.000652057	MAPRE2
ENSG00000130402	0.52419087	0.000334068	0.00606559	ACTN4
ENSG00000054267	0.523207929	0.004523024	0.043658625	ARID4B
ENSG00000073712	0.521282271	0.002162718	0.025704943	FERMT2
ENSG00000109756	0.521043461	0.003696162	0.037756151	RAPGEF2
ENSG00000076053	0.519767789	0.002136063	0.025487593	RBM7
ENSG00000165650	0.519580147	0.003025469	0.032817234	PDZD8
ENSG00000155256	0.518747048	0.005439134	0.049691467	ZFYVE27
ENSG00000104695	0.517016574	0.000701233	0.010850029	PPP2CB
ENSG00000169504	0.516314316	0.003531172	0.036545592	CLIC4
ENSG00000139496	0.51607872	0.00037622	0.006644627	NUP58
ENSG00000122696	0.516013112	0.00067995	0.010614904	SLC25A51
ENSG00000143398	0.515898884	0.000202482	0.00415961	PIP5K1A
ENSG00000110422	0.515848683	0.003602058	0.03702813	HIPK3
ENSG00000175073	0.515796805	0.000860176	0.012797086	VCPIP1
ENSG00000198841	0.514274323	0.003459554	0.036074942	KTI12
ENSG00000167996	0.513339138	7.59E-05	0.00184459	FTH1
ENSG00000170836	0.512764876	1.30E-05	0.000429251	PPM1D
ENSG00000086712	0.512349472	0.000676109	0.010573745	TXLNG
ENSG00000172115	0.511675639	0.005224644	0.048317303	CYCS
ENSG00000111737	0.509940642	4.00E-05	0.001087363	RAB35
ENSG00000100483	0.509034338	0.002371854	0.027633641	VCPKMT
ENSG00000117139	0.508543414	0.00210323	0.025205728	KDM5B
ENSG00000112378	0.508233596	0.002977862	0.032409227	PERP
ENSG00000168066	0.508200501	1.11E-05	0.000374332	SF1
ENSG00000077044	0.508181569	0.001334682	0.017961436	DGKD
ENSG00000159176	0.508139317	0.001161884	0.016181551	CSRP1
ENSG00000177733	0.506326129	0.003261864	0.03457203	HNRNPA0
ENSG00000131013	0.505660398	5.55E-05	0.001438309	PPIL4
ENSG00000102390	0.504870839	0.003018712	0.032757633	PBDC1
ENSG00000123374	0.504855788	0.005248271	0.048459654	CDK2
ENSG00000060069	0.502521883	0.003687052	0.03767792	CTDP1
ENSG00000213516	0.501010784	0.001731428	0.021749026	RBMXL1
ENSG00000087269	0.499921836	0.002976669	0.032409227	NOP14
ENSG00000163374	0.496116373	0.000144127	0.003169082	YY1AP1
ENSG00000163875	0.495295099	0.002686838	0.030009774	MEAF6
ENSG00000143621	0.495009891	0.000318351	0.005853967	ILF2
ENSG00000169895	0.493851099	1.23E-05	0.000411297	SYAP1
ENSG00000106635	0.492069422	0.000228103	0.004570168	BCL7B
ENSG00000178974	0.491961044	0.005186011	0.048085394	FBXO34
ENSG00000153815	0.491871328	0.00177817	0.022170302	CMIP
ENSG00000102543	0.491300415	0.00403291	0.040245336	CDADC1
ENSG00000010404	0.489986145	0.000238344	0.004702717	IDS
ENSG00000111011	0.48914535	0.002433294	0.028072144	RSRC2
ENSG00000114933	0.486277859	0.002489472	0.028454552	INO80D
ENSG00000029363	0.483716269	6.85E-05	0.0016899	BCLAF1
ENSG00000119048	0.483115162	1.39E-05	0.000456352	UBE2B
ENSG00000181222	0.483031041	0.001376003	0.01837456	POLR2A
ENSG00000065526	0.482588109	0.00049329	0.008204422	SPEN
ENSG00000198964	0.482422633	0.005312425	0.048878077	SGMS1
ENSG00000103496	0.481657668	0.002514511	0.02865239	STX4
ENSG00000103342	0.48049091	0.002677531	0.029964475	GSPT1
ENSG00000140299	0.479801193	0.0003937	0.006897334	BNIP2
ENSG00000161526	0.478481985	0.001996028	0.024291249	SAP30BP
ENSG00000025293	0.477473715	0.002692916	0.030064716	PHF20
ENSG00000113504	0.477125284	0.001660152	0.021063235	SLC12A7
ENSG00000101150	0.476448708	0.002043152	0.024702528	TPD52L2
ENSG00000198815	0.476175745	0.001248152	0.017116566	FOXJ3
ENSG00000102921	0.472313683	0.001459143	0.019120472	N4BP1
ENSG00000197183	0.471508321	0.001886931	0.023213998	NOL4L
ENSG00000171490	0.471366368	0.000413898	0.007183272	RSL1D1
ENSG00000171161	0.467856768	0.001363782	0.018248938	ZNF672
ENSG00000221823	0.467703143	1.14E-05	0.000383752	PPP3R1
ENSG00000143549	0.467116699	2.85E-05	0.000831337	TPM3
ENSG00000102119	0.466128112	0.00240156	0.027862009	EMD
ENSG00000068323	0.465715897	0.0003555	0.006370028	TFE3
ENSG00000136141	0.465649863	0.004147046	0.041115501	LRCH1
ENSG00000109606	0.465304827	0.002741439	0.030451949	DHX15

ENSG00000186141	0.464708649	0.004415815	0.042927214	POLR3C
ENSG00000107263	0.464490424	0.003209852	0.03418835	RAPGEF1
ENSG00000136875	0.463034838	3.65E-05	0.001014805	PRPF4
ENSG00000168264	0.462604992	0.003854283	0.038915711	IRF2BP2
ENSG00000110925	0.459854138	0.004992959	0.046818693	CSRNP2
ENSG00000121749	0.458631978	0.000658154	0.010336846	TBC1D15
ENSG00000088205	0.457681056	0.000156189	0.003371972	DDX18
ENSG00000123130	0.457274867	0.00387296	0.039024401	ACOT9
ENSG00000144747	0.455679061	0.000731886	0.011216495	TMF1
ENSG00000083520	0.453070225	0.005073553	0.047368952	DIS3
ENSG00000178951	0.452742407	0.000649598	0.010233439	ZBTB7A
ENSG00000085449	0.452673806	0.002464323	0.028256528	WDFY1
ENSG00000156671	0.452611606	0.002994611	0.032550559	SAMD8
ENSG00000185728	0.451557344	0.002491066	0.028460241	YTHDF3
ENSG00000143319	0.451191268	0.001257803	0.017189382	ISG20L2
ENSG00000213740	0.449442997	0.002518941	0.028665107	SERBP1P1
ENSG00000166225	0.448452285	0.002967083	0.032346187	FRS2
ENSG00000119953	0.447994224	0.000350694	0.006296952	SMNDC1
ENSG00000170832	0.446532855	0.003974018	0.039887769	USP32
ENSG00000134352	0.446325518	0.001990341	0.024244786	IL6ST
ENSG00000108963	0.445293277	0.001909799	0.023417607	DPH1
ENSG00000180957	0.445020263	0.001535662	0.019832897	PITPNB
ENSG00000004961	0.443968884	0.004771886	0.045285787	HCCS
ENSG00000119801	0.441349365	5.90E-05	0.001502222	YPEL5
ENSG00000131504	0.441033884	0.003264133	0.034581945	DIAPH1
ENSG00000173120	0.437736152	0.001066919	0.01516006	KDM2A
ENSG00000067560	0.43464957	2.56E-05	0.000766126	RHOA
ENSG00000153113	0.43459746	0.001356472	0.018169861	CAST
ENSG00000147224	0.433696048	0.002911772	0.031917551	PRPS1
ENSG00000138814	0.433475485	0.001168918	0.016235939	PPP3CA
ENSG00000090432	0.432969674	0.004151857	0.041135528	MUL1
ENSG00000089902	0.431655545	0.001392245	0.018543735	RCOR1
ENSG00000135018	0.430079366	0.004177861	0.041279045	UBQLN1
ENSG00000112081	0.429977649	0.00022576	0.00453723	SRSF3
ENSG00000160877	0.427791046	0.004919846	0.046334053	NACC1
ENSG00000085721	0.427774109	0.002235305	0.026356472	RRN3
ENSG00000011304	0.42605049	0.001714114	0.021589514	PTBP1
ENSG00000131149	0.425877493	0.002003361	0.024333294	GSE1
ENSG00000134108	0.423154733	0.002404159	0.027862009	ARL8B
ENSG00000166716	0.422327202	0.000625439	0.009943402	ZNF592
ENSG00000065135	0.421616226	0.000410175	0.007132977	GNAI3
ENSG00000006634	0.421614367	0.004490353	0.043456439	DBF4
ENSG00000204469	0.419811907	0.001675518	0.021210864	PRRC2A
ENSG00000198887	0.419544374	0.00379886	0.038532138	SMC5
ENSG00000121774	0.417495557	0.001210119	0.016692053	KHDRBS1
ENSG00000196792	0.417147396	0.001672233	0.021195784	STRN3
ENSG00000107581	0.414144778	0.003614602	0.037127662	EIF3A
ENSG00000137814	0.413768106	0.003051619	0.033020851	HAUS2
ENSG00000170242	0.412894833	0.004468792	0.043280058	USP47
ENSG00000184007	0.412408776	0.00041679	0.007228633	PTP4A2
ENSG00000158796	0.411818911	0.001227824	0.016878063	DEDD
ENSG00000135801	0.408890444	0.004236765	0.04165804	TAF5L
ENSG00000109670	0.407388533	0.003238194	0.034405476	FBXW7
ENSG00000198060	0.406008222	0.00024789	0.004835896	5-Mar
ENSG00000122482	0.405459753	0.003988726	0.039988979	ZNF644
ENSG00000182606	0.402865523	0.002727266	0.030349537	TRAK1
ENSG00000138660	0.401885546	0.005112453	0.047612242	AP1AR
ENSG00000004897	0.401831614	0.005335984	0.049042668	CDC27
ENSG00000169375	0.399490065	0.002095177	0.025144056	SIN3A
ENSG00000082153	0.398697612	0.005083374	0.047392458	BZW1
ENSG00000205937	0.398658569	0.002180266	0.025889789	RNPS1
ENSG00000116095	0.396576968	0.000542205	0.008864555	PLEKHA3
ENSG00000148335	0.395100509	0.005080595	0.047383579	NTMT1
ENSG00000131408	0.395072633	0.003904011	0.039261033	NR1H2
ENSG00000148516	0.394423464	0.000942037	0.013708402	ZEB1
ENSG00000179562	0.394028141	0.004533146	0.043707546	GCC1
ENSG00000073921	0.391176326	0.000424298	0.007324576	PICALM
ENSG00000069345	0.384847994	0.000586688	0.009443054	DNAJA2
ENSG00000108061	0.384070539	0.002087384	0.025062134	SHOC2
ENSG00000126653	0.382410918	0.000712237	0.010973696	NSRP1

ENSG00000175104	0.382402461	0.000404891	0.007069411	TRAF6
ENSG00000126524	0.381368999	0.004989428	0.046802497	SBDS
ENSG00000099783	0.381002282	0.003526376	0.036545592	HNRNPM
ENSG00000095787	0.379256199	0.000640145	0.010121398	WAC
ENSG00000091436	0.378150927	0.003542659	0.036591493	MAP3K20
ENSG00000092203	0.377228732	0.000241728	0.004751411	TOX4
ENSG00000175376	0.376929697	0.000388997	0.006824155	EIF1AD
ENSG00000109381	0.376877758	0.001872104	0.02309729	ELF2
ENSG00000110075	0.372375002	0.000698112	0.010826785	PPP6R3
ENSG00000075785	0.371195873	3.54E-05	0.00098776	RAB7A
ENSG00000197694	0.368868265	0.004792705	0.045450122	SPTAN1
ENSG00000149658	0.364503344	0.002242917	0.026416126	YTHDF1
ENSG00000171475	0.363434786	0.004074057	0.040531239	WIPF2
ENSG00000177425	0.362067302	0.00125736	0.017189382	PAWR
ENSG00000107771	0.36076726	0.001505797	0.019534705	CCSER2
ENSG00000070756	0.356341286	0.002215222	0.026180347	PABPC1
ENSG00000169564	0.356341127	0.005273798	0.048610595	PCBP1
ENSG00000101911	0.355215363	0.001718754	0.021626957	PRPS2
ENSG00000152242	0.354712051	0.00047977	0.008051813	C18orf25
ENSG00000213281	0.350631112	0.004716701	0.044904072	NRAS
ENSG00000135932	0.349972254	0.005008857	0.046936486	CAB39
ENSG00000128989	0.349012184	0.003718276	0.037907426	ARPP19
ENSG00000084463	0.348403771	0.000914233	0.013371302	WBP11
ENSG00000134371	0.347475797	0.003492869	0.036294208	CDC73
ENSG00000163510	0.345225984	0.005029654	0.04702681	CWC22
ENSG00000126261	0.344689325	0.004604993	0.044150368	UBA2
ENSG00000122692	0.343606491	0.000767019	0.011658514	SMU1
ENSG00000197879	0.337537953	0.002917589	0.031954312	MYO1C
ENSG00000140320	0.33648829	0.00544468	0.049724628	BAHD1
ENSG00000164808	0.335030735	0.002799459	0.030974315	SPIDR
ENSG00000167182	0.33415143	0.000808691	0.012163645	SP2
ENSG00000157540	0.331528351	0.003650155	0.03743055	DYRK1A
ENSG00000068354	0.328108104	0.004320172	0.042298557	TBC1D25
ENSG00000099995	0.327813499	0.002538656	0.028788443	SF3A1
ENSG00000128908	0.32758562	0.005009139	0.046936486	INO80
ENSG00000140455	0.325918936	0.003381223	0.035393364	USP3
ENSG00000197555	0.322696431	2.45E-05	0.00074136	SIPA1L1
ENSG00000062650	0.321368937	0.005421136	0.049579412	WAPL
ENSG00000140829	0.3149025	0.000980503	0.014156996	DHX38
ENSG00000154945	0.312950284	0.002076012	0.02496024	ANKRD40
ENSG00000158545	0.31214953	0.000469686	0.007933894	ZC3H18
ENSG00000114416	0.311741661	0.00455782	0.043847678	FXR1
ENSG00000120616	0.30316425	0.003202663	0.034125788	EPC1
ENSG00000180228	0.302585384	0.001654822	0.021025741	PRKRA
ENSG00000131626	0.302182491	0.004009252	0.040070902	PPFIA1
ENSG00000257923	0.29799868	0.00408607	0.040635172	CUX1
ENSG00000130703	0.297289541	0.000366399	0.006506898	OSBPL2
ENSG00000162923	0.290114793	0.003355899	0.035266162	WDR26
ENSG00000204256	0.276291177	0.001644832	0.020920002	BRD2
ENSG00000156304	0.266156741	0.00123973	0.017019073	SCAF4
ENSG00000106052	0.259790946	0.004801921	0.045503923	TAX1BP1
ENSG00000086589	0.25133757	0.003714136	0.037893164	RBM22
ENSG00000075413	0.231090799	0.002605188	0.02934684	MARK3
ENSG00000107341	0.22680899	0.003751522	0.038171372	UBE2R2
ENSG00000160695	-0.285465302	4.44E-05	0.001182946	VPS11
ENSG00000164465	-0.293267693	0.003494293	0.036294208	DCBLD1
ENSG00000204977	-0.320014883	0.002367675	0.027633641	TRIM13
ENSG00000178988	-0.320624715	0.001503651	0.019518508	MRFAP1L1
ENSG00000124608	-0.329664698	0.001341537	0.018021573	AARS2
ENSG00000055147	-0.330329221	0.00438032	0.042664199	FAM114A2
ENSG00000009830	-0.331197423	0.001339337	0.018005406	POMT2
ENSG00000185379	-0.334670171	0.001494229	0.019442964	RAD51D
ENSG00000159445	-0.33511809	0.003600723	0.03702813	THEM4
ENSG00000164576	-0.347443956	0.000715332	0.011001781	SAP30L
ENSG00000053900	-0.350018378	0.001577324	0.02026002	ANAPC4
ENSG00000047932	-0.350977479	0.004664629	0.044577365	GOPC
ENSG00000111731	-0.361560692	0.003371114	0.035368753	C2CD5
ENSG00000196312	-0.363835121	0.003611756	0.037113122	MFSD14C
ENSG00000159596	-0.364751043	0.001302227	0.017616053	TMEM69
ENSG00000065029	-0.368209877	0.002938207	0.032139431	ZNF76

ENSG00000146802	-0.372054015	0.002143949	0.025540364	TMEM168
ENSG00000215041	-0.376242797	0.001923115	0.023533774	NEURL4
ENSG00000105829	-0.377168994	0.004721065	0.044904072	BET1
ENSG00000151445	-0.377426156	0.005227267	0.048317303	VIPAS39
ENSG00000105393	-0.378315941	0.0054119	0.049519474	BABAM1
ENSG00000159199	-0.38167204	0.003553761	0.036667051	ATP5G1
ENSG00000108666	-0.384662578	0.005327025	0.04898805	C17orf75
ENSG00000136444	-0.389865631	0.002542354	0.02881779	RSAD1
ENSG00000151148	-0.390241463	0.003339622	0.035180606	UBE3B
ENSG00000173409	-0.392154659	0.00111366	0.015662986	ARV1
ENSG00000143643	-0.394865525	0.005433719	0.04965948	TTC13
ENSG00000114026	-0.396214669	0.000386345	0.006786802	OGG1
ENSG00000131495	-0.402330074	0.00536873	0.049238977	NDUFA2
ENSG00000155729	-0.403850612	0.00285711	0.031411262	KCTD18
ENSG00000119906	-0.406321237	0.000273028	0.005222546	SLF2
ENSG00000227372	-0.408607816	0.001669228	0.021168036	TP73-AS1
ENSG00000214022	-0.410354927	0.001860318	0.023006584	REPIN1
ENSG00000189339	-0.410896684	0.004961455	0.046598884	SLC35E2B
ENSG00000151474	-0.413907556	0.003653346	0.03743055	FRMD4A
ENSG00000235106	-0.417751248	0.002747801	0.030503049	LINC00094
ENSG00000238045	-0.418733514	0.00466803	0.044577365	AC009133.1
ENSG00000155229	-0.41914021	2.55E-06	0.000106224	MMS19
ENSG00000196704	-0.420913143	0.000606757	0.009693921	AMZ2
ENSG00000165792	-0.421429092	0.00383071	0.038755601	METTL17
ENSG00000276234	-0.423220071	0.004906712	0.046236158	TADA2A
ENSG00000043514	-0.426498437	0.001364886	0.018254291	TRIT1
ENSG00000107672	-0.426611211	0.001632108	0.020778541	NSMCE4A
ENSG00000185917	-0.428465992	0.001085551	0.015387011	SETD4
ENSG00000099795	-0.429666444	0.00493448	0.046438164	NDUFB7
ENSG00000186104	-0.429783755	6.81E-05	0.001682743	CYP2R1
ENSG00000118518	-0.429925109	0.003254988	0.034527358	RNF146
ENSG00000116213	-0.43060095	3.09E-06	0.00012583	WRAP73
ENSG00000120458	-0.430886362	0.002312917	0.027117467	MSANTD2
ENSG00000163867	-0.43157755	0.002249241	0.026466576	ZMYM6
ENSG00000091732	-0.431919926	0.002408813	0.027876481	ZC3HC1
ENSG00000014123	-0.438267852	0.002080974	0.024996733	UFL1
ENSG00000147118	-0.445285582	0.00182724	0.022662314	ZNF182
ENSG00000001461	-0.445372329	0.003535823	0.036564553	NIPAL3
ENSG00000133895	-0.446357488	0.00148261	0.019330554	MEN1
ENSG00000136631	-0.45102088	0.003042474	0.032960351	VPS45
ENSG00000006530	-0.451090869	0.001134614	0.015869914	AGK
ENSG00000068097	-0.452527412	0.000618856	0.009865399	HEATR6
ENSG00000214194	-0.454408781	0.002529447	0.028744545	SMIM30
ENSG00000237765	-0.457314631	0.002370234	0.027633641	FAM200B
ENSG00000124574	-0.457665637	8.70E-05	0.00207135	ABCC10
ENSG00000183309	-0.459543694	0.003218017	0.034247193	ZNF623
ENSG00000075336	-0.460919571	0.001501604	0.01950957	TIMM21
ENSG00000148399	-0.465346082	0.003506119	0.036387909	DPH7
ENSG00000221838	-0.466787952	0.001425328	0.018817214	AP4M1
ENSG00000155636	-0.467012713	0.004947621	0.046511218	RBM45
ENSG00000215012	-0.468315016	0.001357326	0.018171915	RTL10
ENSG00000010322	-0.468593711	0.003103422	0.033314598	NISCH
ENSG00000273015	-0.468962126	0.001272529	0.017322684	AC008124.1
ENSG00000135828	-0.46986036	0.004602599	0.044150368	RNASEL
ENSG00000189319	-0.470340586	0.002720987	0.030312897	FAM53B
ENSG00000124181	-0.471728845	0.004415023	0.042927214	PLCG1
ENSG00000188092	-0.473695238	0.00167903	0.021240426	GPR89B
ENSG00000167785	-0.474294958	0.0037367	0.03806534	ZNF558
ENSG00000132646	-0.474782519	0.003323295	0.035065555	PCNA
ENSG00000135315	-0.475684945	0.003857662	0.038915711	CEP162
ENSG00000150433	-0.479563768	0.003655631	0.03743055	TMEM218
ENSG00000167380	-0.480877243	0.001878327	0.023130071	ZNF226
ENSG00000162222	-0.481333762	1.39E-05	0.000456821	TTC9C
ENSG00000120699	-0.48179434	0.001278098	0.017371148	EXOSC8
ENSG00000239779	-0.482557535	0.003802101	0.038534884	WBP1
ENSG00000151366	-0.483970177	0.001789945	0.022263618	NDUFC2
ENSG00000112367	-0.484519926	0.002267247	0.026654281	FIG4
ENSG00000014138	-0.485945943	0.005351912	0.049154232	POLA2
ENSG00000118418	-0.487615654	0.004449258	0.043187596	HMGN3
ENSG00000143971	-0.491208198	0.000635622	0.010078687	ETAA1

ENSG00000169740	-0.493262174	0.004289872	0.042049499	ZNF32
ENSG00000186017	-0.494699313	0.003489239	0.036294208	ZNF566
ENSG00000050426	-0.49479696	0.000491071	0.008178007	LETMD1
ENSG00000076650	-0.498549092	8.75E-05	0.002078157	GPATCH1
ENSG00000103248	-0.498907803	0.001129289	0.015821026	MTHFSD
ENSG00000136098	-0.500423025	0.004676296	0.044636318	NEK3
ENSG00000148835	-0.50227455	0.002465612	0.028256528	TAF5
ENSG00000134897	-0.503347628	0.003854439	0.038915711	BIVM
ENSG00000181896	-0.506218667	0.000619011	0.009865399	ZNF101
ENSG00000125247	-0.507537924	0.001134017	0.015869914	TMTC4
ENSG00000110717	-0.507594045	0.002224822	0.026262612	NDUFS8
ENSG00000159792	-0.507809435	0.002044547	0.024707874	PSKH1
ENSG00000111850	-0.510953564	0.001856999	0.022976493	SMIM8
ENSG00000183513	-0.512268158	0.002840062	0.031290124	COA5
ENSG00000264538	-0.51314899	0.00367654	0.037600116	SUZ12P1
ENSG00000198551	-0.51325101	0.000893271	0.013140151	ZNF627
ENSG00000111653	-0.513724877	0.002404955	0.027862009	ING4
ENSG00000143224	-0.513799938	0.000421242	0.007286362	PPOX
ENSG00000111224	-0.514440621	0.002138437	0.025488883	PARP11
ENSG00000163463	-0.517246817	0.004453368	0.043211324	KRTCAP2
ENSG00000143258	-0.519384751	0.001324625	0.017853882	USP21
ENSG00000131788	-0.522612432	0.005021887	0.047007821	PIAS3
ENSG00000236287	-0.523035676	4.14E-05	0.001119523	ZBED5
ENSG00000108799	-0.523320833	0.003995095	0.039993491	EZH1
ENSG00000101452	-0.524487723	0.002922019	0.031982654	DHX35
ENSG00000145416	-0.52647485	0.003201868	0.034125788	1-Mar
ENSG00000197162	-0.527818008	0.002371802	0.027633641	ZNF785
ENSG00000064933	-0.529310503	0.002487996	0.028450222	PMS1
ENSG00000198912	-0.530935869	0.001533742	0.019817964	C1orf174
ENSG00000196290	-0.533868818	0.002073667	0.024943611	NIF3L1
ENSG00000139651	-0.534234526	0.000977638	0.014131361	ZNF740
ENSG00000198040	-0.538133573	0.0003845	0.006763549	ZNF84
ENSG00000187815	-0.539065311	0.001986855	0.02421369	ZFP69
ENSG00000169592	-0.539921391	0.001579954	0.020273728	INO80E
ENSG00000148950	-0.54060843	0.004099792	0.040720181	IMMP1L
ENSG00000197362	-0.542226527	0.00140689	0.018657311	ZNF786
ENSG00000112983	-0.544519086	0.001605756	0.020504675	BRD8
ENSG00000164414	-0.551706993	0.00025096	0.004877463	SLC35A1
ENSG00000186812	-0.552651946	0.003239848	0.034408961	ZNF397
ENSG00000135722	-0.552816179	0.001690226	0.021361233	FBXL8
ENSG00000179523	-0.555492813	0.0011256	0.015779917	EIF3J-AS1
ENSG00000011143	-0.558623986	0.001999753	0.024324006	MKS1
ENSG00000130772	-0.560734815	0.000565321	0.009178854	MED18
ENSG00000167291	-0.560809468	0.002155297	0.025640233	TBC1D16
ENSG00000174365	-0.561573988	0.002673972	0.029943407	SNHG11
ENSG00000133460	-0.562680519	0.005231478	0.0483218	SLC2A11
ENSG00000146556	-0.563019121	0.00518136	0.048064202	WASH2P
ENSG00000166965	-0.563419291	0.000985006	0.014214113	RCCD1
ENSG00000245680	-0.563512822	0.004718038	0.044904072	ZNF585B
ENSG00000165819	-0.563560021	0.00063589	0.010078687	METTL3
ENSG00000178188	-0.563913373	0.001164096	0.016194982	SH2B1
ENSG00000074582	-0.566261459	0.002853876	0.031402315	BCS1L
ENSG00000272419	-0.568958302	0.002706568	0.030191146	AC241585.2
ENSG00000103199	-0.569004735	0.000253404	0.004917584	ZNF500
ENSG00000188177	-0.571466233	0.004222189	0.041558771	ZC3H6
ENSG00000105967	-0.571857407	0.004803497	0.045503923	TFEC
ENSG00000197629	-0.572953048	0.003054164	0.033023414	MPEG1
ENSG00000115392	-0.574570089	0.002035864	0.024637376	FANCL
ENSG00000188542	-0.577490906	0.000903342	0.013253343	DUSP28
ENSG00000186376	-0.578340133	0.003182815	0.033970105	ZNF75D
ENSG00000084774	-0.581873609	0.002619294	0.029460163	CAD
ENSG00000010292	-0.58449643	0.000796578	0.01201629	NCAPD2
ENSG00000139719	-0.587440299	0.000267527	0.005134068	VPS33A
ENSG00000130731	-0.587920337	0.004517452	0.043625392	METTL26
ENSG00000007202	-0.590593968	0.003554214	0.036667051	KIAA0100
ENSG00000173369	-0.592467689	0.001675876	0.021210864	C1QB
ENSG00000183723	-0.596785489	0.003190373	0.034036771	CMTM4
ENSG00000204271	-0.598230014	0.000576976	0.009326501	SPIN3
ENSG00000176444	-0.598242702	7.08E-06	0.000256776	CLK2
ENSG00000180376	-0.598514717	0.002244308	0.026420511	CCDC66

ENSG00000183718	-0.598630446	0.000781989	0.01185133	TRIM52
ENSG00000172349	-0.600047088	0.003341916	0.035190482	IL16
ENSG00000175701	-0.600081854	0.002966585	0.032346187	LINC00116
ENSG00000249042	-0.600469605	0.002583532	0.029155488	AC008771.1
ENSG00000277476	-0.60403815	0.002148152	0.025578688	AC005332.8
ENSG00000148444	-0.604197384	0.000832163	0.012430229	COMMD3
ENSG00000188234	-0.605364738	0.001461479	0.019131956	AGAP4
ENSG00000136514	-0.606523349	0.004907669	0.046236158	RTP4
ENSG00000114735	-0.607983116	0.003540684	0.036585659	HEMK1
ENSG00000115459	-0.609189237	0.000919704	0.013436169	ELMOD3
ENSG00000164002	-0.609949553	1.13E-05	0.000381902	EXO5
ENSG00000198169	-0.613669252	0.00024566	0.004817764	ZNF251
ENSG00000010295	-0.616550799	0.003359716	0.035291979	IFFO1
ENSG00000132801	-0.618649712	0.003055932	0.033023414	ZSWIM3
ENSG00000108848	-0.619129928	0.000156213	0.003371972	LUC7L3
ENSG00000184939	-0.620684947	0.000898501	0.013200742	ZFP90
ENSG00000262814	-0.622651728	0.002102053	0.02520327	MRPL12
ENSG00000254726	-0.62266613	0.003473255	0.036191593	MEX3A
ENSG00000178175	-0.625330795	0.003754132	0.038182951	ZNF366
ENSG00000178096	-0.625390812	0.004712465	0.044902543	BOLA1
ENSG00000165275	-0.626287273	4.69E-06	0.000180913	TRMT10B
ENSG00000274895	-0.626681714	0.004168799	0.041205204	AC011700.1
ENSG00000171806	-0.628164979	0.000308871	0.005726819	METTL18
ENSG00000173275	-0.628322159	0.003675358	0.037600116	ZNF449
ENSG00000128699	-0.62885324	8.89E-07	4.20E-05	ORMDL1
ENSG00000198081	-0.629699627	0.000791747	0.011959293	ZBTB14
ENSG00000103494	-0.6333844	0.004719591	0.044904072	RPGRIP1L
ENSG00000274828	-0.633788585	0.004602491	0.044150368	AC068473.5
ENSG00000182742	-0.635039776	0.003119096	0.03345517	HOXB4
ENSG00000169964	-0.635333337	0.002905191	0.031872335	TMEM42
ENSG00000175548	-0.638835615	3.81E-05	0.001048972	ALG10B
ENSG00000100802	-0.639499257	0.0004896	0.008163987	C14orf93
ENSG00000100359	-0.642929486	0.003580119	0.036875654	SGSM3
ENSG00000213918	-0.64348454	0.000462838	0.007848877	DNASE1
ENSG00000204282	-0.64385561	0.00542461	0.049593703	TNRC6C-AS1
ENSG00000251369	-0.643857497	0.00078283	0.011857158	ZNF550
ENSG00000134297	-0.644103953	0.002785883	0.030863585	PLEKHA8P1
ENSG00000135637	-0.647229576	0.001455754	0.019107953	CCDC142
ENSG00000173611	-0.64826244	0.000340889	0.006176466	SCAI
ENSG00000174151	-0.651506088	0.001414823	0.018719526	CYB561D1
ENSG00000214135	-0.653594353	0.00087366	0.012975377	AC132008.2
ENSG00000103343	-0.655009256	0.000257449	0.004981188	ZNF174
ENSG00000197128	-0.656761749	0.000467244	0.00790295	ZNF772
ENSG00000280798	-0.658337885	0.003336965	0.035167921	LINC00294
ENSG00000113971	-0.658388041	0.001249277	0.017122944	NPHP3
ENSG00000154743	-0.659244013	0.000132342	0.002939853	TSEN2
ENSG00000175611	-0.662251838	0.004926993	0.046384529	LINC00476
ENSG00000116001	-0.662532937	0.001264735	0.017270955	TIA1
ENSG00000140326	-0.663058258	7.45E-05	0.001813024	CDAN1
ENSG00000178977	-0.665096051	0.000767777	0.011663205	LINC00324
ENSG00000101751	-0.6655598	3.12E-06	0.000126512	POLI
ENSG00000106066	-0.666091798	0.00405072	0.040345487	CPVL
ENSG00000197044	-0.667295847	0.002418968	0.027944139	ZNF441
ENSG00000174804	-0.667364015	0.001277641	0.017371148	FZD4
ENSG00000218510	-0.667832782	0.000750463	0.011440366	LINC00339
ENSG00000221909	-0.669871942	1.19E-05	0.000398076	FAM200A
ENSG00000261799	-0.670328916	0.002335048	0.027339872	AC007406.5
ENSG00000139679	-0.670653427	0.000189345	0.003927047	LPAR6
ENSG00000183111	-0.671745789	0.003224254	0.034285454	ARHGEF37
ENSG00000164011	-0.674305337	0.001424946	0.018817214	ZNF691
ENSG00000169413	-0.674412144	0.00421091	0.041494979	RNASE6
ENSG00000166927	-0.676365449	0.003286598	0.034758935	MS4A7
ENSG00000215271	-0.679601647	0.003135759	0.03357838	HOMEZ
ENSG00000100271	-0.679901514	0.001601418	0.020488361	TTLL1
ENSG00000196981	-0.681493685	0.000459688	0.00781078	WDR5B
ENSG00000125846	-0.682807213	1.94E-05	0.000607342	ZNF133
ENSG00000105497	-0.683480214	2.11E-05	0.000654524	ZNF175
ENSG00000105609	-0.683694694	0.002066406	0.024867801	LILRB5
ENSG00000186666	-0.684792364	0.00047631	0.008010799	BCDIN3D
ENSG00000141219	-0.684968989	0.000927202	0.013522866	C17orf80

ENSG00000263072	-0.685219491	0.00097697	0.014129579	ZNF213-AS1
ENSG00000007312	-0.687770376	0.001885555	0.023208064	CD79B
ENSG00000128694	-0.68848128	0.00125456	0.017173693	OSGEPL1
ENSG00000099326	-0.689666528	0.00049968	0.008294756	MZF1
ENSG00000176896	-0.69057026	0.004818574	0.045612079	TCEANC
ENSG00000236144	-0.690996522	0.001540368	0.019873886	TMEM147-AS1
ENSG00000281162	-0.691970161	0.003165004	0.033821743	AC005035.1
ENSG00000204176	-0.693079385	0.002894893	0.031772794	SYT15
ENSG00000122678	-0.695362553	0.001824906	0.022644194	POLM
ENSG00000169683	-0.696659662	0.00318017	0.033955843	LRRC45
ENSG00000154016	-0.696685242	0.002559563	0.028987529	GRAP
ENSG00000173209	-0.697082665	0.003512533	0.036439896	AHSA2
ENSG00000089335	-0.698391314	0.000491018	0.008178007	ZNF302
ENSG00000089091	-0.698981245	0.005273985	0.048610595	DZANK1
ENSG00000233184	-0.702180711	0.000543514	0.008869193	AC093157.1
ENSG00000163472	-0.703492239	0.000347881	0.00626813	TMEM79
ENSG00000197372	-0.704123898	0.003292193	0.034793993	ZNF675
ENSG00000162227	-0.704442902	0.001655577	0.021025741	TAF6L
ENSG00000155659	-0.70793314	0.000747877	0.0114212	VSIG4
ENSG00000163516	-0.709916676	1.02E-05	0.000348894	ANKZF1
ENSG00000220785	-0.711943044	0.004903623	0.046231614	MTMR9LP
ENSG00000122481	-0.713574154	0.00024021	0.004725158	RWDD3
ENSG00000151065	-0.716541516	2.50E-05	0.000752147	DCP1B
ENSG00000259943	-0.718575668	0.000427888	0.007371841	AL050341.2
ENSG00000272760	-0.719213462	0.000154403	0.003346812	AC093726.1
ENSG00000243335	-0.720044193	0.000231105	0.004599563	KCTD7
ENSG00000110077	-0.7205389	0.000440973	0.007552127	MS4A6A
ENSG00000159189	-0.721199902	0.000590817	0.009474257	C1QC
ENSG00000233901	-0.721334523	0.001381925	0.018434667	LINC01503
ENSG00000197782	-0.722494836	0.004591433	0.044105636	ZNF780A
ENSG00000263002	-0.722617178	0.001010148	0.014520386	ZNF234
ENSG00000120253	-0.723450571	3.94E-05	0.001077156	NUP43
ENSG00000234444	-0.725472066	0.000115108	0.002610667	ZNF736
ENSG00000178573	-0.72728348	0.0002832	0.005347825	MAF
ENSG00000161929	-0.72813709	1.76E-05	0.000559326	SCIMP
ENSG00000204650	-0.728709924	0.00015398	0.003343239	LINC02210
ENSG00000175970	-0.729436646	0.000282913	0.005346291	UNC119B
ENSG00000164241	-0.731300462	0.002632285	0.029553117	C5orf63
ENSG00000138380	-0.734674603	0.001709636	0.021543577	CARF
ENSG00000239911	-0.736276325	0.002750278	0.030508213	PRKAG2-AS1
ENSG00000117010	-0.737159731	1.79E-05	0.000566494	ZNF684
ENSG00000083814	-0.739484401	0.000351472	0.006306557	ZNF671
ENSG00000162639	-0.740847165	0.003818464	0.038670516	HENMT1
ENSG00000173914	-0.743647926	1.83E-05	0.000575873	RBM4B
ENSG00000255182	-0.745181668	0.003380643	0.035393364	AC084125.2
ENSG00000235314	-0.74772965	0.000332605	0.006055982	LINC00957
ENSG00000198221	-0.748292642	0.000378725	0.006675539	AFDN-AS1
ENSG00000234420	-0.74934645	0.000288441	0.005417671	ZNF37BP
ENSG00000263272	-0.749977621	0.003562442	0.03671742	AC004148.2
ENSG00000144026	-0.751660355	1.74E-06	7.57E-05	ZNF514
ENSG00000168517	-0.751948269	0.001573501	0.020220933	HEXIM2
ENSG00000185220	-0.752007216	0.003668119	0.037543596	PGBD2
ENSG00000227946	-0.752377621	0.005025811	0.047007821	AC007383.2
ENSG00000198298	-0.754585073	0.005412674	0.049519474	ZNF485
ENSG00000204611	-0.756186289	8.21E-05	0.001966982	ZNF616
ENSG00000237190	-0.756527512	0.004606989	0.044150368	CDKN2AIPNL
ENSG00000197062	-0.758055493	0.000282913	0.005346291	ZSCAN26
ENSG00000197363	-0.758349532	0.001777641	0.022170302	ZNF517
ENSG00000238105	-0.75973097	0.00539368	0.049397965	GOLGA2P5
ENSG00000213380	-0.761599518	0.000810306	0.01217383	COG8
ENSG00000169169	-0.762032663	0.001201309	0.016623012	CPT1C
ENSG00000179941	-0.762327813	0.000436209	0.007490326	BBS10
ENSG00000115282	-0.76270095	0.001305124	0.017646039	TTC31
ENSG00000234616	-0.763871985	0.000131856	0.002931572	JRK
ENSG00000144134	-0.766761651	9.19E-05	0.002159019	RABL2A
ENSG00000167840	-0.768020428	8.70E-06	0.000307442	ZNF232
ENSG00000173928	-0.768687627	0.002133063	0.025469147	SWSAP1
ENSG00000213865	-0.769775523	0.001294835	0.017561828	C8orf44
ENSG00000188227	-0.770482386	0.002584507	0.029155488	ZNF793
ENSG00000250132	-0.771013411	0.000701906	0.010853192	AC004803.1

ENSG00000125434	-0.771795039	0.001544262	0.019914221	SLC25A35
ENSG00000140451	-0.772849614	0.005210793	0.048285441	PIF1
ENSG00000167578	-0.782248287	0.000246584	0.004819583	RAB4B
ENSG00000196345	-0.785668484	0.000698704	0.010829489	ZKSCAN7
ENSG00000080293	-0.785700678	0.004255324	0.041758181	SCTR
ENSG00000232434	-0.790135078	0.004527538	0.043683864	C9orf172
ENSG00000230733	-0.792620356	0.002341347	0.027388907	AC092171.2
ENSG00000264247	-0.793487212	3.92E-08	2.84E-06	LINC00909
ENSG00000215883	-0.794920454	0.000644694	0.010180915	CYB5RL
ENSG00000173917	-0.796767002	0.000347617	0.006267739	HOXB2
ENSG00000257433	-0.797377397	0.002055607	0.024795338	AC004241.1
ENSG00000076351	-0.79910368	0.002299114	0.026967821	SLC46A1
ENSG00000269834	-0.80217022	0.004024437	0.040191703	ZNF528-AS1
ENSG00000237945	-0.803139565	0.003321095	0.035056605	LINC00649
ENSG00000242294	-0.804986618	0.003537998	0.036572473	STAG3L5P
ENSG00000250571	-0.805698032	0.000579584	0.009351916	GLI4
ENSG00000198182	-0.806404929	0.001765096	0.022070935	ZNF607
ENSG00000247240	-0.810571655	0.000288778	0.005417671	UBL7-AS1
ENSG00000272853	-0.813540785	0.000752534	0.011458751	AC069544.1
ENSG00000120093	-0.815290146	0.001286238	0.017463486	HOXB3
ENSG00000162063	-0.816889609	0.001394299	0.018561563	CCNF
ENSG00000133561	-0.817413506	6.11E-08	4.08E-06	GIMAP6
ENSG00000163154	-0.818574653	0.000330291	0.006022303	TNFAIP8L2
ENSG00000247828	-0.821354497	0.001017374	0.014583856	TMEM161B-AS1
ENSG00000162999	-0.821859422	0.000163536	0.003492271	DUSP19
ENSG00000237840	-0.821940616	0.002530353	0.028744545	FAM21FP
ENSG00000105383	-0.822038197	0.000480643	0.008060195	CD33
ENSG00000131634	-0.822393662	0.002138646	0.025488883	TMEM204
ENSG00000180257	-0.825644523	0.000725081	0.011125346	ZNF816
ENSG00000175538	-0.827259807	0.001464266	0.019158777	KCNE3
ENSG00000213139	-0.829728319	0.000884127	0.013078428	CRYGS
ENSG00000216895	-0.830846944	8.91E-07	4.20E-05	AC009403.1
ENSG00000258301	-0.83297167	0.002226466	0.026270073	VASH1-AS1
ENSG00000145649	-0.833774498	0.002726839	0.030349537	GZMA
ENSG00000196689	-0.834049121	0.001015678	0.0145676	TRPV1
ENSG00000183734	-0.835617054	0.002440009	0.028118318	ASCL2
ENSG00000261526	-0.839380888	0.004380537	0.042664199	AC012615.1
ENSG00000187626	-0.84119673	0.000257268	0.004981188	ZKSCAN4
ENSG00000196366	-0.842074642	0.000231026	0.004599563	C9orf163
ENSG00000196458	-0.843153968	0.001410982	0.018687766	ZNF605
ENSG00000167984	-0.845648745	0.005069947	0.047352353	NLRC3
ENSG00000188171	-0.847818419	0.0014567	0.019107953	ZNF626
ENSG00000099869	-0.847883821	0.005076372	0.047368952	IGF2-AS
ENSG00000169598	-0.848326065	1.10E-05	0.000372718	DFFB
ENSG00000185513	-0.848736147	3.24E-05	0.000917631	L3MBTL1
ENSG00000235194	-0.852537151	0.000271827	0.005205045	PPP1R3E
ENSG00000232593	-0.853613548	0.004181321	0.041290895	KANTR
ENSG00000106560	-0.858828198	0.000623968	0.009926319	GIMAP2
ENSG00000198185	-0.859443905	0.001473143	0.019236118	ZNF334
ENSG00000234432	-0.862846303	0.002283124	0.0268045	AC092171.3
ENSG00000117616	-0.863514755	0.002384556	0.027741971	RSRP1
ENSG00000269486	-0.866416875	0.003098998	0.03328088	AC011455.2
ENSG00000269044	-0.866848828	0.000484345	0.008107627	AC024075.2
ENSG00000128482	-0.866863519	0.00480364	0.045503923	RNF112
ENSG00000128000	-0.867709896	0.0004764	0.008010799	ZNF780B
ENSG00000196670	-0.868899783	0.000114847	0.002607009	ZFP62
ENSG00000260285	-0.871455373	0.001053327	0.014999794	AL133367.1
ENSG00000133574	-0.873740186	1.50E-06	6.60E-05	GIMAP4
ENSG00000004139	-0.875585328	1.43E-05	0.000466795	SARM1
ENSG00000198133	-0.878426131	0.000129833	0.002896514	TMEM229B
ENSG00000223705	-0.8797792	0.000117904	0.002667079	NSUN5P1
ENSG00000258534	-0.883370569	0.002621491	0.029460163	AL132712.1
ENSG00000133624	-0.884735233	1.97E-05	0.000616257	ZNF767P
ENSG00000226752	-0.887231963	0.000701283	0.010850029	PSMD5-AS1
ENSG00000198934	-0.88846397	0.001125747	0.015779917	MAGEE1
ENSG00000283175	-0.889188088	0.002716245	0.030286076	AC007920.2
ENSG00000261269	-0.889932218	8.71E-06	0.000307442	AC093278.2
ENSG00000037757	-0.890725089	3.01E-05	0.00086516	MRI1
ENSG00000177599	-0.892308117	0.002802281	0.030992316	ZNF491
ENSG00000172123	-0.893107793	0.000787164	0.01190889	SLFN12

ENSG00000198105	-0.893230602	0.000246411	0.004819583	ZNF248
ENSG00000262580	-0.893790068	0.002727785	0.030349537	AC087741.1
ENSG00000258297	-0.89435428	0.000118757	0.0026817	AP001157.1
ENSG00000181690	-0.894379876	0.004156994	0.041135528	PLAG1
ENSG00000242600	-0.894770424	0.000455132	0.007744666	MBL1P
ENSG00000245571	-0.895110328	6.57E-05	0.001644461	AP001258.1
ENSG00000197124	-0.897864302	0.000601783	0.009638189	ZNF682
ENSG00000137834	-0.898276579	0.00084402	0.012598888	SMAD6
ENSG00000080947	-0.898340622	0.000173989	0.003658281	CROCCP3
ENSG00000120784	-0.89897992	0.000513973	0.008493981	ZFP30
ENSG00000166928	-0.899493105	0.00400671	0.040060923	MS4A14
ENSG00000116205	-0.901143084	0.000407768	0.007096423	TCEANC2
ENSG00000231711	-0.901602633	0.000891416	0.013133832	LINC00899
ENSG00000230487	-0.902533288	0.000109732	0.002512896	PSMG3-AS1
ENSG00000106479	-0.903618103	0.001049137	0.014948331	ZNF862
ENSG00000240449	-0.904619643	0.001468124	0.019189892	AC005586.1
ENSG00000120215	-0.909863022	0.004165641	0.041189676	MLANA
ENSG00000104863	-0.92041992	0.001168138	0.016233801	LIN7B
ENSG00000245849	-0.920549162	0.000889389	0.013111417	RAD51-AS1
ENSG00000181631	-0.921152405	0.003867369	0.038983195	P2RY13
ENSG00000261067	-0.921156116	0.000692881	0.01076871	AC109460.3
ENSG00000115657	-0.926057957	1.61E-06	7.03E-05	ABCB6
ENSG00000259877	-0.926311667	0.001300256	0.017607745	AC009113.1
ENSG00000234618	-0.927929864	0.002215834	0.026180347	RPSAP9
ENSG00000180539	-0.929547929	0.002053747	0.024784423	C9orf139
ENSG00000204851	-0.931337358	0.000432284	0.007432764	PNMA8B
ENSG00000153896	-0.932471484	6.18E-07	3.05E-05	ZNF599
ENSG00000256771	-0.933188921	0.000265373	0.005102592	ZNF253
ENSG00000224307	-0.937568646	0.001341929	0.018021573	AL161785.1
ENSG00000171596	-0.938233298	0.000451379	0.007699849	NMUR1
ENSG00000185670	-0.940867678	0.000710516	0.010953678	ZBTB3
ENSG00000272145	-0.943903266	0.004024209	0.040191703	NFYC-AS1
ENSG00000124587	-0.945522329	0.004541332	0.043753951	PEX6
ENSG00000182685	-0.946415585	0.005150817	0.047935117	BRICD5
ENSG00000186715	-0.947166362	0.001335378	0.017961493	MST1L
ENSG00000230590	-0.948294565	3.94E-05	0.001077156	FTX
ENSG00000234028	-0.949899765	0.001297879	0.017593924	AC062029.1
ENSG00000100122	-0.951873159	0.000791878	0.011959293	CRYBB1
ENSG00000160298	-0.957102893	0.001061138	0.015102733	C21orf58
ENSG00000180884	-0.958070218	9.02E-07	4.25E-05	ZNF792
ENSG00000175787	-0.959798302	4.09E-06	0.000160429	ZNF169
ENSG00000121933	-0.962429426	0.001349663	0.018106704	TMIGD3
ENSG00000246089	-0.96293971	0.00038834	0.00681724	AC016065.1
ENSG00000251247	-0.963026035	0.000278256	0.005285235	ZNF345
ENSG00000146192	-0.964256334	3.95E-05	0.001078639	FGD2
ENSG00000268403	-0.965419332	0.00156749	0.020163657	AC132192.2
ENSG00000229754	-0.969098387	0.003943369	0.039626122	CXCR2P1
ENSG00000278267	-0.972365306	0.003495934	0.036296718	MIR6859-1
ENSG00000244041	-0.976553862	0.000104858	0.002424818	LINC01011
ENSG00000215068	-0.978972302	0.002500743	0.028520562	AC025171.2
ENSG00000131400	-0.986852918	0.002816768	0.031099518	NAPSA
ENSG00000102886	-0.987561818	0.003051877	0.033020851	GDPD3
ENSG00000280120	-0.995787653	0.004335476	0.042417626	AC073857.1
ENSG00000274925	-0.999506684	0.000159874	0.003431	AC008741.2
ENSG00000139725	-1.000377746	0.00436088	0.042504654	RHOF
ENSG00000266777	-1.002402024	0.00358683	0.036915471	AC090616.6
ENSG00000237651	-1.003443649	0.00089653	0.013179251	C2orf74
ENSG00000244701	-1.005167956	0.000367449	0.006516631	AC004918.1
ENSG00000272356	-1.005715028	0.000700388	0.01084911	AL080317.3
ENSG00000251359	-1.007554536	0.000105945	0.002441244	WWC2-AS2
ENSG00000273899	-1.009123941	6.62E-06	0.000243123	NOL12
ENSG00000279833	-1.012535657	0.004576344	0.044009566	AL031846.2
ENSG00000108511	-1.013307376	0.000691306	0.010753377	HOXB6
ENSG00000260778	-1.019422069	3.11E-05	0.000886427	AC009065.4
ENSG00000214787	-1.020033053	0.004308722	0.042202379	MS4A4E
ENSG00000280149	-1.026019435	0.004402404	0.042828936	AC004877.2
ENSG00000235706	-1.026132219	0.001146162	0.01601418	DICER1-AS1
ENSG00000204623	-1.031480378	0.000532641	0.008752318	ZNRD1ASP
ENSG00000205866	-1.032823541	0.000770382	0.011695926	FAM99A
ENSG00000170835	-1.037186454	0.004210863	0.041494979	CEL

ENSG00000174600	-1.039504367	8.97E-05	0.002117125	CMKLR1
ENSG00000082196	-1.041519671	9.59E-06	0.000332509	C1QTNF3
ENSG00000269837	-1.042678889	0.000109261	0.002508739	IPO5P1
ENSG00000267309	-1.045127951	0.000364387	0.006502331	AC092295.2
ENSG00000182700	-1.047395224	0.000156412	0.003373462	IGIP
ENSG00000259772	-1.051917274	0.000718228	0.011039783	AC012236.1
ENSG00000164100	-1.052627559	0.004898593	0.046201325	NDST3
ENSG00000139187	-1.057103991	1.27E-05	0.00042437	KLRG1
ENSG00000205865	-1.057109029	5.08E-05	0.001327807	FAM99B
ENSG00000186026	-1.057518807	9.99E-05	0.002328841	ZNF284
ENSG00000149634	-1.059636228	0.003065262	0.03307854	SPATA25
ENSG00000204822	-1.060138841	5.70E-05	0.001466206	MRPL53
ENSG00000133256	-1.061251132	0.000812994	0.012200076	PDE6B
ENSG00000181036	-1.063682962	0.004947003	0.046511218	FCRL6
ENSG00000213967	-1.067110645	0.005308149	0.048856056	ZNF726
ENSG00000214189	-1.067554757	0.000448358	0.007659819	ZNF788
ENSG00000180061	-1.072944833	9.66E-05	0.002257368	TMEM150B
ENSG00000175544	-1.085123526	9.69E-06	0.000334646	CABP4
ENSG00000184601	-1.08592422	0.000407705	0.007096423	C14orf180
ENSG00000249201	-1.092781956	0.003956707	0.039744766	CTD-3080P12.3
ENSG00000232229	-1.099447341	0.000796046	0.012015253	LINC00865
ENSG00000261556	-1.10025531	0.00027321	0.005222546	SMG1P7
ENSG00000267152	-1.101071269	0.000684248	0.010670996	AC093227.1
ENSG00000161640	-1.101093728	0.000249126	0.00484544	SIGLEC11
ENSG00000121807	-1.10266029	0.004745475	0.045084616	CCR2
ENSG00000280145	-1.103604361	0.002399967	0.027862009	CU638689.4
ENSG00000187808	-1.108288853	0.000248521	0.004840941	SOWAHD
ENSG00000110025	-1.108564607	0.000437315	0.007504354	SNX15
ENSG00000227051	-1.110626809	5.45E-07	2.72E-05	C14orf132
ENSG00000253686	-1.110899881	0.000676498	0.010573745	LINC01484
ENSG00000236778	-1.11095899	0.002574884	0.029084871	INTS6-AS1
ENSG00000260103	-1.115241973	0.00250027	0.028520562	AC012435.1
ENSG00000173894	-1.117755256	0.003418083	0.035688365	CBX2
ENSG00000213203	-1.125757744	2.54E-05	0.000758816	GIMAP1
ENSG00000274292	-1.125807093	0.00046648	0.007897085	AC084018.2
ENSG00000196172	-1.132210078	0.001657123	0.02103508	ZNF681
ENSG00000205864	-1.137982646	0.002427192	0.028014202	KRTAP5-6
ENSG00000161664	-1.140255564	0.001913293	0.023438292	ASB16
ENSG00000232442	-1.14241053	0.002326239	0.027260467	MHENCR
ENSG00000272692	-1.149802813	0.003529148	0.036545592	AC010997.4
ENSG00000273373	-1.152658459	0.000218541	0.004422988	AL355488.2
ENSG00000231345	-1.157907013	0.002915573	0.031945717	BEND3P1
ENSG00000261474	-1.162766318	0.000326313	0.00596233	AC026471.4
ENSG00000270012	-1.163072868	0.000720383	0.01106635	AC232271.1
ENSG00000279452	-1.163139937	0.000471032	0.007951455	AC006277.1
ENSG00000163794	-1.164019184	0.002717831	0.030290745	UCN
ENSG00000273084	-1.164345879	0.001100545	0.015535764	AC092171.5
ENSG00000162739	-1.182021031	0.00048618	0.008127847	SLAMF6
ENSG00000226659	-1.183051363	0.004689862	0.044720018	AC021028.1
ENSG00000267508	-1.183763436	0.00489159	0.046168495	ZNF285
ENSG00000197935	-1.186149456	0.001407244	0.018657311	ZNF311
ENSG00000240972	-1.188920414	0.0026385	0.029610086	MIF
ENSG00000142065	-1.192325688	0.0011514	0.016070048	ZFP14
ENSG00000146215	-1.19257098	0.000921898	0.013460639	CRIP3
ENSG00000126882	-1.214241385	5.14E-07	2.61E-05	FAM78A
ENSG00000276744	-1.215761298	0.001134481	0.015869914	AC105137.3
ENSG00000166845	-1.216286829	0.000471914	0.007961168	C18orf54
ENSG00000180626	-1.216913363	2.29E-05	0.000704891	ZNF594
ENSG00000167968	-1.218098695	0.002440548	0.028118318	DNASE1L2
ENSG00000179914	-1.219713577	0.002680746	0.029967533	ITLN1
ENSG00000264176	-1.220550755	0.003552733	0.036667051	MAGOH2P
ENSG00000124613	-1.225000592	0.001228158	0.016878063	ZNF391
ENSG00000273253	-1.22786913	0.000326113	0.00596233	AL022328.4
ENSG00000204872	-1.228470852	0.002132264	0.025469147	NAT8B
ENSG00000245888	-1.230807583	0.003028891	0.032840619	FLJ21408
ENSG00000271654	-1.234119664	0.000466593	0.007897085	AC025062.2
ENSG00000212694	-1.23647405	4.93E-06	0.000188775	LINC01089
ENSG00000260329	-1.240248701	0.000115673	0.002621176	AC007541.1
ENSG00000185614	-1.241681568	0.000366975	0.006512678	FAM212A
ENSG00000243238	-1.245317388	0.001654339	0.021025741	IGKV2-30

ENSG00000149050	-1.249882762	0.001001641	0.014414079	ZNF214
ENSG00000180938	-1.252506767	0.000120065	0.002704162	ZNF572
ENSG00000279518	-1.255763497	0.000309814	0.005726819	AC083843.3
ENSG00000235997	-1.265630386	0.004685857	0.044698253	LINC01936
ENSG00000274471	-1.267989011	4.05E-07	2.14E-05	AC242376.2
ENSG00000123999	-1.273831648	0.00218741	0.025962734	INHA
ENSG00000261326	-1.27979712	8.97E-06	0.000316366	LINC01355
ENSG00000261707	-1.280106805	0.003221339	0.034268497	AC092134.1
ENSG00000261879	-1.281225485	0.000159872	0.003431	AC087500.1
ENSG00000271551	-1.281571658	0.001774272	0.022142985	AL355297.4
ENSG00000163406	-1.283898723	7.33E-05	0.001787975	SLC15A2
ENSG00000171115	-1.285297743	1.93E-08	1.57E-06	GIMAP8
ENSG00000163126	-1.289485973	0.00034192	0.006190833	ANKRD23
ENSG00000229719	-1.291018734	3.12E-05	0.000886876	MIR194-2HG
ENSG00000260572	-1.292263787	0.000503349	0.008344976	AC069224.1
ENSG00000171657	-1.292913568	0.003337062	0.035167921	GPR82
ENSG00000260293	-1.29472233	0.002051551	0.024769448	AC106820.4
ENSG00000258168	-1.305722487	0.000290873	0.005450401	AC025569.1
ENSG00000199753	-1.306096431	0.000104819	0.002424818	SNORD104
ENSG00000228653	-1.312325736	0.001008179	0.01450012	HNRNPCP7
ENSG00000277067	-1.313203011	0.003801273	0.038534884	CU634019.1
ENSG00000284237	-1.323109865	6.05E-05	0.001536753	AL356275.1
ENSG00000276256	-1.324965357	0.00109188	0.015455499	AC011043.1
ENSG00000267102	-1.3268238	0.003080441	0.033150174	AC060766.1
ENSG00000259108	-1.327363086	0.005409905	0.049519474	LINC01595
ENSG00000248554	-1.328095132	0.00251726	0.028665107	AC114956.2
ENSG00000139193	-1.33275537	0.000952166	0.013840283	CD27
ENSG00000272849	-1.33379329	0.000219956	0.004444687	AC084018.1
ENSG00000147138	-1.335852697	0.005384357	0.049329986	GPR174
ENSG00000283341	-1.337106699	8.95E-05	0.002116562	AC068205.2
ENSG00000130173	-1.337313439	0.002424715	0.027998061	ANGPTL8
ENSG00000248429	-1.338017878	0.000308731	0.005726819	AC098679.1
ENSG00000211666	-1.338920739	0.005268389	0.048593542	IGLV2-14
ENSG00000271971	-1.345505583	0.000281103	0.005319829	AC120053.1
ENSG00000161643	-1.349562601	6.33E-06	0.000234686	SIGLEC16
ENSG00000273148	-1.352202058	0.000183936	0.003833263	AL035563.1
ENSG00000239382	-1.356042322	2.74E-09	2.69E-07	ALKBH6
ENSG00000166343	-1.367404959	4.51E-07	2.34E-05	MSS51
ENSG00000274422	-1.369317893	0.000130497	0.002908818	AC245060.5
ENSG00000253347	-1.371612814	0.000888001	0.013098398	AC040934.1
ENSG00000203896	-1.375880726	0.003969097	0.039853785	LIME1
ENSG00000226491	-1.381627407	0.000575339	0.009321476	FTOP1
ENSG00000264075	-1.383047045	0.001064745	0.015145762	MIR4783
ENSG00000257958	-1.385019087	0.00334772	0.035209751	AC008125.1
ENSG00000102468	-1.386593414	0.004442814	0.043141188	HTR2A
ENSG00000257808	-1.38684869	0.003832837	0.038755601	AC073573.1
ENSG00000163141	-1.395137008	0.002968331	0.032346201	BNIPL
ENSG00000273576	-1.39924146	2.42E-08	1.91E-06	AC009283.1
ENSG00000221886	-1.402424088	0.000126697	0.002833861	ZBED8
ENSG00000156509	-1.404862592	0.004664025	0.044577365	FBXO43
ENSG00000213999	-1.404948867	0.000346966	0.006264696	MEF2B
ENSG00000089012	-1.406250678	0.000423636	0.007318007	SIRPG
ENSG00000132465	-1.407255296	4.89E-06	0.000187345	JCHAIN
ENSG00000171659	-1.40946853	5.69E-08	3.85E-06	GPR34
ENSG00000275371	-1.41455868	0.005449651	0.049752513	AC012645.4
ENSG00000268947	-1.414623543	0.000528254	0.008702279	AC002128.1
ENSG00000250365	-1.414916863	0.000748935	0.011423797	AL139353.2
ENSG00000274220	-1.424124929	4.13E-05	0.00111805	AC009163.7
ENSG00000165449	-1.429822905	0.000747821	0.0114212	SLC16A9
ENSG00000266217	-1.43032453	0.00030695	0.005704964	CTSLP2
ENSG00000264290	-1.438791471	0.000531552	0.008745491	AC104564.4
ENSG00000275449	-1.44039071	0.002162296	0.025704943	MIR6859-3
ENSG00000258451	-1.442458305	8.37E-05	0.002000624	AL163636.1
ENSG00000263412	-1.445255637	9.19E-05	0.002158659	AC004477.1
ENSG00000262877	-1.458731417	0.000181037	0.003781963	AC110285.2
ENSG00000247950	-1.460485389	0.002106757	0.025236342	SEC24B-AS1
ENSG00000275549	-1.467995975	0.002730991	0.030372177	STPG3-AS1
ENSG00000267530	-1.478124923	0.003015585	0.032737394	LINC01836
ENSG00000179144	-1.478889465	2.69E-10	3.26E-08	GIMAP7
ENSG00000260773	-1.483914042	0.002026949	0.02456386	AC055855.2

ENSG00000237886	-1.513646603	6.39E-06	0.000235546	NALT1
ENSG00000259895	-1.517427762	0.000934748	0.013625261	AC106820.2
ENSG00000125787	-1.526927719	0.00484836	0.045860571	GNRH2
ENSG00000226751	-1.529695307	0.003447405	0.035965574	AF127936.1
ENSG00000271784	-1.534637418	8.90E-05	0.002108042	AL031055.1
ENSG00000261192	-1.536285113	0.002413123	0.027901462	RNF126P1
ENSG00000280399	-1.544519182	0.001267553	0.017291235	AC022497.1
ENSG00000184619	-1.551294814	0.003770188	0.038316216	KRBA2
ENSG00000211653	-1.554152345	0.00053761	0.008806082	IGLV1-40
ENSG00000211943	-1.560357149	0.001178863	0.016365313	IGHV3-15
ENSG00000224713	-1.561821501	0.001909531	0.023417607	AC025165.1
ENSG00000236754	-1.566496407	0.000150624	0.003286875	AC007666.1
ENSG00000199038	-1.574098312	0.003715417	0.037893164	MIR210
ENSG00000205929	-1.582084004	0.004608813	0.044150368	C21orf62
ENSG00000137078	-1.593593183	6.63E-05	0.001651695	SIT1
ENSG00000271576	-1.601947342	1.58E-06	6.95E-05	AL359504.2
ENSG00000268297	-1.604974236	2.57E-05	0.000767195	CLEC4GP1
ENSG00000224689	-1.615514636	0.004987507	0.046801392	ZNF812P
ENSG00000236617	-1.6162157	0.004468041	0.043280058	AC127070.2
ENSG00000269086	-1.618164176	0.00169897	0.021429989	AC008555.2
ENSG00000249669	-1.618295332	0.000194606	0.00401053	CARMN
ENSG00000223749	-1.625130709	4.51E-06	0.000174338	MIR503HG
ENSG00000180139	-1.62681846	0.004090015	0.04065882	ACTA2-AS1
ENSG00000271662	-1.627130654	0.003483719	0.036256946	AC233280.2
ENSG00000278367	-1.635459225	0.004609703	0.044150368	AL356652.1
ENSG00000240382	-1.639847706	0.003413886	0.035667667	IGKV1-17
ENSG00000272040	-1.642426806	0.000292774	0.005472849	AC010245.2
ENSG00000230910	-1.643384374	0.001099273	0.015526254	AL391807.1
ENSG00000262692	-1.654191761	0.000108816	0.002500742	AC116914.2
ENSG00000230006	-1.654542745	0.002457727	0.028205678	ANKRD36BP2
ENSG00000272983	-1.657769524	0.004852559	0.045883561	AL117339.4
ENSG00000274929	-1.659997805	0.002962782	0.032340068	AL157813.1
ENSG00000278690	-1.663543495	0.003369499	0.035366108	AC244100.4
ENSG00000236417	-1.676315682	0.00028607	0.005386346	CTSLP1
ENSG00000263884	-1.690686542	0.000119588	0.002698103	AP000845.1
ENSG00000211660	-1.692939822	0.000502642	0.008338581	IGLV2-23
ENSG00000168229	-1.694739417	0.000379856	0.006690931	PTGDR
ENSG00000266973	-1.701893043	0.001101895	0.015546359	AC092296.1
ENSG00000267543	-1.703784414	0.002621979	0.029460163	AC015802.3
ENSG00000188681	-1.706339954	0.002830334	0.03122272	TEKT4P2
ENSG00000236305	-1.708599001	0.001862497	0.023015023	SLC12A9-AS1
ENSG00000279278	-1.716620983	9.45E-06	0.000329234	AC245060.6
ENSG00000141028	-1.724150723	0.001698014	0.021428347	CDRT15P1
ENSG00000264177	-1.726938718	0.005377064	0.049292647	AL353997.2
ENSG00000237494	-1.729805833	0.001000733	0.014408994	AL360007.1
ENSG00000171217	-1.734779808	0.004538637	0.043744232	CLDN20
ENSG00000259349	-1.738682467	0.002339747	0.027382531	AC011921.1
ENSG00000224358	-1.74979625	0.003775965	0.038359899	AL451074.2
ENSG00000139445	-1.75871618	0.001725844	0.021695126	FOXN4
ENSG00000267391	-1.759109574	6.37E-06	0.000235546	AC105105.2
ENSG00000273473	-1.765130604	0.003081717	0.033150174	BX649601.1
ENSG00000226777	-1.765849917	2.39E-05	0.000728346	FAM30A
ENSG00000272525	-1.774668465	0.000139941	0.003090143	AC099522.2
ENSG00000276409	-1.780463768	3.13E-07	1.70E-05	CCL14
ENSG00000272970	-1.782230519	0.005154235	0.047949727	AC107294.2
ENSG00000283991	-1.785911343	0.003015395	0.032737394	AC017000.1
ENSG00000256124	-1.788699955	0.000871686	0.012953479	LINC01152
ENSG00000235241	-1.806391327	5.84E-05	0.001495064	BX284668.4
ENSG00000257989	-1.812004466	0.004196942	0.041388769	AC078864.1
ENSG00000280129	-1.831691309	0.001085856	0.015387011	AL132780.5
ENSG00000258273	-1.832885069	0.000679859	0.010614904	AC024257.2
ENSG00000261366	-1.840342747	0.001160318	0.016168428	MANEA-AS1
ENSG00000260362	-1.843471468	0.003884745	0.039112769	AC007218.1
ENSG00000232283	-1.850962235	0.001105661	0.015582548	HSD17B3-AS1
ENSG00000239839	-1.872651147	0.00107469	0.01525378	DEFA3
ENSG00000282608	-1.87738986	0.000104216	0.002416441	ADORA3
ENSG00000270550	-1.879444334	0.004805535	0.04550526	IGHV3-30
ENSG00000252412	-1.902179047	0.001896971	0.0233154	Y_RNA
ENSG00000272170	-1.91298389	0.004155705	0.041135528	AL355385.1
ENSG00000230778	-1.925502869	0.000221882	0.00447664	ANKRD63

ENSG00000260296	-1.93201531	3.85E-05	0.001055892	AC095057.3
ENSG00000005381	-1.957156504	2.51E-05	0.000752615	MPO
ENSG00000236763	-1.972921385	0.000417431	0.007234918	TRMT112P4
ENSG00000239571	-1.986114449	0.004614147	0.044176627	IGKV2D-30
ENSG00000225891	-2.028024453	0.002001979	0.024329434	AL513365.2
ENSG00000228639	-2.054038299	0.005216966	0.048301289	LINC02095
ENSG00000211598	-2.069829893	1.13E-06	5.20E-05	IGKV4-1
ENSG00000237484	-2.084646717	0.005173287	0.048006477	LINC01684
ENSG00000048462	-2.086103708	0.002465421	0.028256528	TNFRSF17
ENSG00000225698	-2.089945719	0.005156501	0.04795361	IGHV3-72
ENSG00000270605	-2.101889767	1.45E-05	0.000472207	AL353622.1
ENSG00000272719	-2.121299339	0.000534935	0.008778892	AC006483.2
ENSG00000269681	-2.136998784	0.001864549	0.023015023	AC063977.7
ENSG00000266274	-2.137382678	0.000172975	0.003639902	RN7SL138P
ENSG00000269974	-2.150174024	0.003778555	0.038371191	AC091057.3
ENSG00000272953	-2.152108301	7.32E-09	6.50E-07	AC092171.4
ENSG00000197705	-2.269318706	0.001834664	0.022743528	KLHL14
ENSG00000283312	-2.302719473	0.004945877	0.046511218	AC017104.4
ENSG00000144407	-2.318417966	0.001490931	0.019419531	PTH2R
ENSG00000271993	-2.324649102	0.002964803	0.032346187	AC126118.1
ENSG00000279561	-2.350444129	0.002454966	0.028197629	AL845472.1
ENSG00000267370	-2.353839557	7.91E-06	0.000282853	AC008752.3
ENSG00000272128	-2.367617028	0.002406428	0.027862009	AP006545.2
ENSG00000196415	-2.381220584	7.71E-05	0.001869013	PRTN3
ENSG00000218631	-2.425309575	0.003996003	0.039993491	AL117344.1
ENSG00000267895	-2.461418912	0.001102611	0.01554801	AC063977.1
ENSG00000275580	-2.478661098	0.000227773	0.004567085	AC022306.2
ENSG00000258654	-2.490335301	0.000468284	0.007915372	AC026495.1
ENSG00000265046	-2.560231226	0.001916927	0.023471729	AC004253.2
ENSG00000206047	-2.61106198	0.003244213	0.03444122	DEFA1
ENSG00000270933	-2.616050966	1.85E-09	1.87E-07	AC010719.1
ENSG00000225972	-2.618351414	0.001084419	0.015383452	MTND1P23
ENSG00000260476	-2.623507215	0.00276057	0.030596224	AC104794.3
ENSG00000213876	-2.687251575	0.001640048	0.020869387	AC120057.1
ENSG00000278212	-2.713364015	0.000693122	0.01076871	AC134878.2
ENSG00000227210	-2.748098162	0.000179354	0.003752845	AC079145.1
ENSG00000262319	-2.886119256	0.00219241	0.026001157	AC007952.6
ENSG00000279926	-2.919556184	7.48E-07	3.65E-05	AL138831.3
ENSG00000274444	-2.936275148	0.001095432	0.015488868	AC078909.2
ENSG00000169627	-2.991644402	4.67E-05	0.001233865	BOLA2B
ENSG00000271259	-3.00695573	0.000582694	0.009390428	AC010201.1
ENSG00000280057	-3.069361634	3.11E-05	0.000886427	AL022069.2
ENSG00000260949	-3.122174262	0.000129649	0.002894906	AP006545.1
ENSG00000211942	-3.250873246	0.00516485	0.047979659	IGHV3-13
ENSG00000164821	-3.658229011	8.32E-07	3.99E-05	DEFA4

Table 8. *TNIP3* and the AST levels of patients in 14 pairs of bulk RNA-seq samples.

Samples	EP TNIP3 level	PR TNIP3 level	Fold change	peak AST (U/L)	Group
1	0.038302044	0.131689084	3.438173808	3428	Low
2	0.113352733	0.12748214	1.124649901	1900	Low
3	0.069499956	0.041258365	0.593645924	2218	Low
4	0.069954428	0.47702207	6.819040383	765	High
5	0.028241698	0.222543886	7.879975356	1779	High
6	0.016308596	1.400345129	85.86546134	1133	High
7	0.025045605	0.029457	1.176134505	3531	Low
8	0.020632536	0.902089562	43.72170102	723	High
9	0.01	3.221400473	322.1400473	249	High
10	0.023946643	0.08191076	3.420552903	1762	Low
11	0.079317862	0.077060498	0.971540289	1285	Low
12	0.01	1.338119331	133.8119331	629	High
13	0.056009758	0.011532451	0.205900741	3420	Low
14	0.01	0.048186575	4.818657524	25083	NA

*The 14th case was ruled out because of abnormally elevated liver enzymes due to surgical factors.

Table 9. *TNIP3* and the AST levels of patients in 16 pairs of RT-PCR samples.

Samples	Fold change	peak AST (U/L)	Group
1	11.44640844	175	High
2	0.325454491	6728	Low
3	0.628155114	3688	Low
4	0.829332649	2297	Low
5	1.548178658	2706	High
6	1.100558702	1268	Low
7	0.485854499	4036	Low
8	0.007269502	2573	Low
9	4.776011905	2371	High
10	8.542385488	1956	High
11	3.008172551	1067	High
12	8.45064661	876	High
13	0.325454491	3843	Low
14	2.972042519	776	High
15	0.238625327	1298	Low
16	1.430437287	603	High

Table 10. List of markers information for endothelial cell clusters, related to Figure 4.

	p_val	avg_logFC	pct.1	pct.2	p_val_adj	gene	cluster	cell type
1	1.50E-102	1.063009685	0.997	0.767	3.26E-98	CTSL	1	CTSL_LSEC
2	1.37E-105	0.996884506	0.992	0.469	2.98E-101	CLEC4M	1	CTSL_LSEC
3	1.97E-80	0.961381669	0.911	0.386	4.29E-76	CCL23	1	CTSL_LSEC
4	9.69E-85	0.94009916	0.962	0.437	2.11E-80	CD14	1	CTSL_LSEC
5	6.41E-100	0.924854064	0.997	0.535	1.40E-95	FCGR2B	1	CTSL_LSEC
6	2.93E-93	0.887400435	1	0.533	6.39E-89	MS4A6A	1	CTSL_LSEC
7	2.40E-97	0.869592606	0.98	0.434	5.24E-93	LYVE1	1	CTSL_LSEC
8	9.10E-92	0.857127552	1	0.603	1.99E-87	FCN2	1	CTSL_LSEC
9	1.06E-90	0.847530387	1	0.553	2.31E-86	CLEC4G	1	CTSL_LSEC
10	5.76E-91	0.83368593	1	0.55	1.26E-86	OIT3	1	CTSL_LSEC
11	3.49E-87	0.81669904	0.997	0.639	7.61E-83	ACP5	1	CTSL_LSEC
12	1.62E-80	0.813461424	0.992	0.697	3.54E-76	LGMN	1	CTSL_LSEC
13	1.54E-91	0.794357372	1	0.848	3.36E-87	SEPP1	1	CTSL_LSEC
14	2.45E-88	0.78315668	1	0.771	5.33E-84	CTSD	1	CTSL_LSEC
15	8.34E-90	0.765665331	0.995	0.531	1.82E-85	CD4	1	CTSL_LSEC
16	1.97E-79	0.760095263	0.997	0.504	4.29E-75	CLEC1B	1	CTSL_LSEC
17	1.34E-84	0.724142775	1	0.776	2.92E-80	DNASE1L3	1	CTSL_LSEC
18	6.27E-91	0.721654124	0.906	0.288	1.37E-86	RELN	1	CTSL_LSEC
19	8.05E-67	0.704324009	1	0.764	1.76E-62	FCN3	1	CTSL_LSEC
20	5.80E-73	0.679093202	0.987	0.623	1.26E-68	MRC1	1	CTSL_LSEC
21	9.22E-79	0.670275752	0.791	0.24	2.01E-74	PASK	1	CTSL_LSEC
22	2.71E-71	0.665617208	0.98	0.615	5.91E-67	P4HB	1	CTSL_LSEC
23	1.38E-75	0.664393909	0.98	0.5	3.01E-71	CXCL16	1	CTSL_LSEC
24	4.25E-68	0.607247454	0.867	0.345	9.26E-64	CFP	1	CTSL_LSEC
25	4.22E-67	0.606028923	0.901	0.41	9.19E-63	NID1	1	CTSL_LSEC
26	1.46E-59	0.5941154	1	0.79	3.18E-55	CALR	1	CTSL_LSEC
27	7.40E-61	0.587132965	0.865	0.35	1.61E-56	EHD3	1	CTSL_LSEC
28	1.66E-47	0.57997727	0.941	0.568	3.62E-43	ADM	1	CTSL_LSEC
29	5.16E-57	0.577270497	0.987	0.661	1.12E-52	PDIA6	1	CTSL_LSEC
30	3.90E-49	0.577248153	0.995	0.856	8.50E-45	HSP90B1	1	CTSL_LSEC
31	3.05E-57	0.550442355	0.972	0.597	6.65E-53	ASAH1	1	CTSL_LSEC
32	1.51E-55	0.537461716	0.946	0.504	3.30E-51	COTL1	1	CTSL_LSEC
33	2.42E-67	0.537172278	0.801	0.284	5.28E-63	PXDN	1	CTSL_LSEC
34	1.72E-38	0.524547719	0.992	0.732	3.75E-34	CRHBP	1	CTSL_LSEC
35	2.18E-63	0.520455597	0.936	0.401	4.76E-59	STAB2	1	CTSL_LSEC
36	1.58E-52	0.519384199	0.872	0.415	3.45E-48	SEMA6A	1	CTSL_LSEC
37	2.41E-50	0.514372706	0.941	0.55	5.25E-46	JAK1	1	CTSL_LSEC
38	2.77E-21	0.513135856	0.931	0.666	6.04E-17	HSPA5	1	CTSL_LSEC
39	3.05E-76	0.508755482	0.594	0.125	6.66E-72	DHCR24	1	CTSL_LSEC
40	6.24E-46	0.503960023	0.987	0.696	1.36E-41	PRCP	1	CTSL_LSEC
41	2.02E-60	0.501237504	0.773	0.277	4.41E-56	TMEM37	1	CTSL_LSEC
42	2.31E-48	0.497466205	0.931	0.515	5.03E-44	SLC40A1	1	CTSL_LSEC
43	5.65E-50	0.49260805	0.941	0.535	1.23E-45	CTSA	1	CTSL_LSEC
44	1.90E-45	0.492139475	0.908	0.475	4.15E-41	KDR	1	CTSL_LSEC
45	8.32E-52	0.489249927	0.997	0.755	1.81E-47	PSAP	1	CTSL_LSEC
46	4.81E-51	0.486068253	0.926	0.494	1.05E-46	SNX5	1	CTSL_LSEC
47	5.73E-75	0.484115076	0.724	0.191	1.25E-70	NPL	1	CTSL_LSEC
48	4.45E-44	0.476712086	0.982	0.694	9.71E-40	PDIA3	1	CTSL_LSEC
49	3.87E-49	0.473659925	1	0.812	8.44E-45	RAMP3	1	CTSL_LSEC
50	5.51E-44	0.469431856	0.954	0.528	1.20E-39	F2R	1	CTSL_LSEC
51	5.25E-41	0.468943866	0.888	0.468	1.15E-36	MMRN1	1	CTSL_LSEC
52	3.75E-45	0.451102281	0.875	0.447	8.17E-41	CALU	1	CTSL_LSEC
53	2.04E-71	0.449842328	0.531	0.101	4.45E-67	ECM1	1	CTSL_LSEC
54	4.30E-32	0.443391258	0.849	0.48	9.38E-28	PLIN2	1	CTSL_LSEC
55	1.83E-42	0.439599492	0.969	0.656	3.99E-38	CTSB	1	CTSL_LSEC
56	2.10E-49	0.438998377	0.844	0.404	4.58E-45	SNX2	1	CTSL_LSEC
57	3.03E-33	0.428918752	0.987	0.766	6.61E-29	SDPR	1	CTSL_LSEC
58	3.63E-48	0.427339113	0.416	0.092	7.92E-44	MS4A4A	1	CTSL_LSEC
59	2.57E-47	0.425761666	0.709	0.267	5.61E-43	ANPEP	1	CTSL_LSEC
60	3.71E-44	0.422995641	0.61	0.215	8.09E-40	SLC27A3	1	CTSL_LSEC
61	5.52E-43	0.419311013	0.99	0.825	1.20E-38	FCGRT	1	CTSL_LSEC
62	1.19E-38	0.418240374	0.834	0.418	2.60E-34	YES1	1	CTSL_LSEC
63	1.56E-48	0.415606566	0.523	0.154	3.41E-44	MAP7D3	1	CTSL_LSEC
64	3.72E-35	0.409876244	0.895	0.482	8.10E-31	CD36	1	CTSL_LSEC
65	1.46E-39	0.408155333	0.982	0.677	3.18E-35	GRN	1	CTSL_LSEC
66	9.31E-40	0.407045245	0.651	0.26	2.03E-35	CBLB	1	CTSL_LSEC
67	1.51E-42	0.406886768	0.837	0.389	3.30E-38	DAB2	1	CTSL_LSEC
68	4.98E-40	0.403176108	0.64	0.243	1.09E-35	PHLDA1	1	CTSL_LSEC

69	7.63E-64	0.398958427	0.388	0.055	1.66E-59	LILRB5	1	CTSL_LSEC
70	3.02E-36	0.398934218	0.931	0.577	6.60E-32	LAMP1	1	CTSL_LSEC
71	1.02E-39	0.39819838	0.804	0.397	2.23E-35	NRP2	1	CTSL_LSEC
72	6.22E-54	0.398110425	1	0.944	1.36E-49	HLA-E	1	CTSL_LSEC
73	1.04E-74	0.397984153	0.454	0.064	2.27E-70	SLC18A2	1	CTSL_LSEC
74	3.10E-31	0.394298016	0.934	0.622	6.76E-27	ADGRL4	1	CTSL_LSEC
75	3.03E-41	0.393604241	0.566	0.203	6.61E-37	SH3TC1	1	CTSL_LSEC
76	5.27E-34	0.388442758	0.918	0.571	1.15E-29	CANX	1	CTSL_LSEC
77	4.05E-40	0.386783071	0.658	0.263	8.84E-36	TSN	1	CTSL_LSEC
78	1.14E-37	0.386192996	0.732	0.326	2.49E-33	GIMAP5	1	CTSL_LSEC
79	1.13E-55	0.384532055	0.987	0.576	2.46E-51	STAB1	1	CTSL_LSEC
80	3.21E-47	0.384330752	0.622	0.205	7.01E-43	ITGA1	1	CTSL_LSEC
81	2.52E-55	0.379290707	0.388	0.066	5.50E-51	MRO	1	CTSL_LSEC
82	1.39E-25	0.3791075	0.967	0.726	3.04E-21	SGK1	1	CTSL_LSEC
83	4.44E-44	0.378660277	0.702	0.277	9.69E-40	CREG1	1	CTSL_LSEC
84	1.24E-40	0.378505864	0.824	0.382	2.70E-36	TPP1	1	CTSL_LSEC
85	1.73E-53	0.377049396	0.561	0.153	3.77E-49	P3H2	1	CTSL_LSEC
86	3.13E-52	0.376511599	0.571	0.162	6.84E-48	RBM47	1	CTSL_LSEC
87	2.44E-32	0.376059722	0.959	0.645	5.31E-28	TMEM123	1	CTSL_LSEC
88	9.41E-28	0.374682949	0.814	0.448	2.05E-23	UGCG	1	CTSL_LSEC
89	2.02E-28	0.368669602	0.913	0.619	4.41E-24	LPAR6	1	CTSL_LSEC
90	1.63E-32	0.367612805	0.827	0.418	3.55E-28	DENND4C	1	CTSL_LSEC
91	1.08E-35	0.366938216	0.997	0.878	2.35E-31	APP	1	CTSL_LSEC
92	1.80E-39	0.365705028	0.709	0.29	3.92E-35	MYO10	1	CTSL_LSEC
93	9.77E-34	0.364732771	0.834	0.418	2.13E-29	TM4SF18	1	CTSL_LSEC
94	8.95E-31	0.362383674	0.702	0.349	1.95E-26	PDIA4	1	CTSL_LSEC
95	4.95E-39	0.36140825	0.992	0.769	1.08E-34	SPARC	1	CTSL_LSEC
96	8.31E-33	0.361287862	0.709	0.329	1.81E-28	EPOR	1	CTSL_LSEC
97	3.60E-47	0.36069139	0.592	0.191	7.86E-43	CD82	1	CTSL_LSEC
98	5.45E-34	0.358774165	0.77	0.385	1.19E-29	TMEM2	1	CTSL_LSEC
99	1.43E-32	0.358635808	0.967	0.646	3.11E-28	PPFIBP1	1	CTSL_LSEC
100	2.08E-38	0.356883106	0.712	0.32	4.53E-34	FHL1	1	CTSL_LSEC
101	1.52E-25	0.356543656	0.98	0.663	3.32E-21	AKAP12	1	CTSL_LSEC
102	3.26E-32	0.356505357	0.929	0.545	7.10E-28	NTN4	1	CTSL_LSEC
103	2.48E-33	0.355571091	0.663	0.293	5.40E-29	SLC35A1	1	CTSL_LSEC
104	2.23E-39	0.35435107	0.531	0.18	4.87E-35	CHID1	1	CTSL_LSEC
105	1.46E-24	0.353676933	0.977	0.724	3.18E-20	SARAF	1	CTSL_LSEC
106	2.95E-44	0.347372438	0.668	0.245	6.43E-40	SNX8	1	CTSL_LSEC
107	3.99E-43	0.34244263	0.564	0.186	8.69E-39	ACSM3	1	CTSL_LSEC
108	3.21E-33	0.342262434	0.663	0.278	6.99E-29	LRG1	1	CTSL_LSEC
109	1.11E-33	0.341476331	1	0.855	2.43E-29	ENG	1	CTSL_LSEC
110	1.37E-37	0.3394846	0.638	0.258	2.99E-33	DAAM1	1	CTSL_LSEC
111	1.10E-59	0.338470638	0.607	0.156	2.40E-55	FAM102A	1	CTSL_LSEC
112	4.09E-27	0.338342923	0.997	0.864	8.91E-23	IL6ST	1	CTSL_LSEC
113	2.03E-39	0.337571405	0.694	0.291	4.42E-35	DRAM2	1	CTSL_LSEC
114	6.90E-36	0.332736782	0.855	0.443	1.51E-31	LRRFIP2	1	CTSL_LSEC
115	3.15E-35	0.330585614	0.992	0.813	6.87E-31	NPC2	1	CTSL_LSEC
116	3.45E-34	0.326971018	0.584	0.237	7.52E-30	SCPEP1	1	CTSL_LSEC
117	1.82E-32	0.326056265	0.946	0.567	3.98E-28	LY96	1	CTSL_LSEC
118	3.48E-19	0.324431935	0.663	0.357	7.58E-15	OLFM1	1	CTSL_LSEC
119	1.19E-27	0.323369863	0.969	0.721	2.59E-23	CCNI	1	CTSL_LSEC
120	8.73E-30	0.323349692	0.834	0.452	1.90E-25	GRINA	1	CTSL_LSEC
121	7.71E-39	0.322119853	0.633	0.239	1.68E-34	ART4	1	CTSL_LSEC
122	3.11E-27	0.317056527	0.727	0.377	6.78E-23	TSPAN6	1	CTSL_LSEC
123	3.13E-38	0.316099263	0.582	0.207	6.84E-34	NR5A2	1	CTSL_LSEC
124	1.42E-32	0.31479651	0.597	0.245	3.10E-28	TMEM106C	1	CTSL_LSEC
125	4.79E-33	0.314715887	0.681	0.29	1.05E-28	ALDH6A1	1	CTSL_LSEC
126	9.23E-45	0.313711689	0.357	0.073	2.01E-40	CD200R1	1	CTSL_LSEC
127	3.20E-33	0.310868661	0.972	0.715	6.98E-29	GYPC	1	CTSL_LSEC
128	1.06E-28	0.30934059	0.602	0.269	2.31E-24	NUAK1	1	CTSL_LSEC
129	3.46E-23	0.30791959	0.913	0.564	7.55E-19	DLC1	1	CTSL_LSEC
130	3.49E-34	0.306544594	0.38	0.108	7.61E-30	ME2	1	CTSL_LSEC
131	4.34E-49	0.306218008	0.36	0.065	9.46E-45	SLC8A1	1	CTSL_LSEC
132	2.12E-42	0.305363654	0.408	0.102	4.62E-38	SLC31A2	1	CTSL_LSEC
133	4.55E-27	0.304797925	0.865	0.511	9.93E-23	PDE2A	1	CTSL_LSEC
134	6.40E-32	0.303999638	0.365	0.106	1.40E-27	HTR2B	1	CTSL_LSEC
135	3.24E-32	0.300338375	0.773	0.375	7.07E-28	HEXB	1	CTSL_LSEC
136	1.80E-31	0.299350412	0.645	0.284	3.93E-27	RAB20	1	CTSL_LSEC
137	3.22E-25	0.299191142	0.908	0.583	7.02E-21	NUCB1	1	CTSL_LSEC
138	1.56E-25	0.299143514	0.949	0.613	3.39E-21	CLEC2B	1	CTSL_LSEC

139	1.90E-38	0.298053945	0.418	0.116	4.14E-34	USP13	1	CTSL_LSEC
140	1.94E-35	0.297940312	0.5	0.173	4.22E-31	PDIA5	1	CTSL_LSEC
141	2.62E-46	0.297594993	0.268	0.034	5.70E-42	COL21A1	1	CTSL_LSEC
142	2.00E-23	0.296879167	0.997	0.852	4.36E-19	IGFBP4	1	CTSL_LSEC
143	8.22E-28	0.295713713	0.974	0.709	1.79E-23	MARCKSL1	1	CTSL_LSEC
144	2.85E-30	0.295692335	0.556	0.23	6.21E-26	CHST12	1	CTSL_LSEC
145	1.71E-34	0.295265618	0.51	0.183	3.73E-30	DERA	1	CTSL_LSEC
146	1.24E-28	0.295174124	0.849	0.456	2.70E-24	TMEM50B	1	CTSL_LSEC
147	1.46E-30	0.293758514	0.454	0.162	3.19E-26	RP11-228B15.4	1	CTSL_LSEC
148	1.33E-24	0.293609121	0.839	0.49	2.89E-20	CD164	1	CTSL_LSEC
149	1.55E-22	0.292055185	0.992	0.787	3.39E-18	TSPAN7	1	CTSL_LSEC
150	7.83E-19	0.291640474	0.546	0.272	1.71E-14	DNAJB11	1	CTSL_LSEC
151	7.73E-31	0.29146233	0.827	0.432	1.69E-26	SMTN	1	CTSL_LSEC
152	1.91E-38	0.291389048	0.617	0.234	4.16E-34	CMTM3	1	CTSL_LSEC
153	1.62E-25	0.289942039	0.885	0.516	3.52E-21	MYDGF	1	CTSL_LSEC
154	2.50E-31	0.289734066	0.403	0.126	5.44E-27	MANEA	1	CTSL_LSEC
155	5.93E-24	0.289261828	0.628	0.309	1.29E-19	ERP44	1	CTSL_LSEC
156	6.10E-27	0.288395847	0.997	0.881	1.33E-22	PPIB	1	CTSL_LSEC
157	5.94E-45	0.288043717	0.324	0.059	1.30E-40	RCSD1	1	CTSL_LSEC
158	6.90E-39	0.286598849	0.462	0.138	1.51E-34	TBC1D4	1	CTSL_LSEC
159	4.66E-24	0.28632592	0.753	0.409	1.02E-19	SLC44A2	1	CTSL_LSEC
160	6.98E-25	0.284266305	0.694	0.349	1.52E-20	CSF1	1	CTSL_LSEC
161	9.89E-24	0.283876229	0.587	0.278	2.16E-19	ARHGEF7	1	CTSL_LSEC
162	1.03E-34	0.283020287	0.352	0.092	2.24E-30	CXADR	1	CTSL_LSEC
163	3.88E-42	0.282993855	0.441	0.122	8.45E-38	SMPDL3A	1	CTSL_LSEC
164	4.16E-27	0.282853644	0.52	0.219	9.08E-23	PELO	1	CTSL_LSEC
165	4.91E-20	0.282759036	0.212	0.057	1.07E-15	GDF15	1	CTSL_LSEC
166	2.09E-31	0.282463567	0.599	0.25	4.56E-27	TMED3	1	CTSL_LSEC
167	9.42E-25	0.282444552	0.895	0.567	2.06E-20	AP2M1	1	CTSL_LSEC
168	7.63E-26	0.281571454	1	0.902	1.66E-21	TFPI	1	CTSL_LSEC
169	6.14E-36	0.28044319	0.474	0.151	1.34E-31	ZNF765	1	CTSL_LSEC
170	3.12E-39	0.278829096	0.27	0.047	6.80E-35	GATA3	1	CTSL_LSEC
171	3.53E-23	0.278392046	0.862	0.464	7.70E-19	TFPI2	1	CTSL_LSEC
172	1.67E-29	0.277393553	0.309	0.084	3.64E-25	RGS16	1	CTSL_LSEC
173	2.49E-21	0.276963803	0.913	0.596	5.42E-17	KDELR1	1	CTSL_LSEC
174	3.44E-24	0.276506626	0.745	0.383	7.49E-20	PHACTR2	1	CTSL_LSEC
175	7.19E-29	0.276303356	0.821	0.44	1.57E-24	ACTN1	1	CTSL_LSEC
176	1.83E-23	0.274611607	0.992	0.892	4.00E-19	ACTG1	1	CTSL_LSEC
177	9.56E-23	0.273500111	0.819	0.483	2.09E-18	BNIP3L	1	CTSL_LSEC
178	5.16E-21	0.273473844	0.872	0.545	1.13E-16	LEPROT	1	CTSL_LSEC
179	3.04E-19	0.272207808	0.952	0.707	6.63E-15	CD151	1	CTSL_LSEC
180	3.52E-30	0.271712727	0.62	0.265	7.68E-26	CNST	1	CTSL_LSEC
181	9.64E-26	0.271653118	0.801	0.419	2.10E-21	NPY1R	1	CTSL_LSEC
182	1.19E-33	0.271441328	0.615	0.237	2.60E-29	VMO1	1	CTSL_LSEC
183	1.56E-33	0.270389551	0.651	0.278	3.41E-29	GLMP	1	CTSL_LSEC
184	1.85E-26	0.269616661	0.543	0.226	4.04E-22	SORBS1	1	CTSL_LSEC
185	1.40E-29	0.269120256	0.462	0.17	3.05E-25	TRIM35	1	CTSL_LSEC
186	4.64E-39	0.268538748	0.406	0.106	1.01E-34	SLC7A8	1	CTSL_LSEC
187	1.22E-37	0.267907339	0.431	0.124	2.66E-33	PHLDB2	1	CTSL_LSEC
188	1.71E-34	0.267615652	0.393	0.112	3.72E-30	ANKRD44	1	CTSL_LSEC
189	3.42E-36	0.267458368	0.485	0.156	7.46E-32	HTATIP2	1	CTSL_LSEC
190	1.42E-34	0.266798759	0.472	0.152	3.09E-30	ATP1B1	1	CTSL_LSEC
191	1.40E-24	0.266788083	0.74	0.38	3.04E-20	LIMCH1	1	CTSL_LSEC
192	1.53E-29	0.266625695	0.773	0.385	3.34E-25	RRBP1	1	CTSL_LSEC
193	3.16E-23	0.26656301	0.793	0.443	6.90E-19	RPN1	1	CTSL_LSEC
194	2.97E-25	0.265741259	0.523	0.225	6.47E-21	ZEB2	1	CTSL_LSEC
195	5.15E-24	0.265365166	0.691	0.354	1.12E-19	DDOST	1	CTSL_LSEC
196	5.96E-40	0.262939366	0.39	0.099	1.30E-35	PIEZO2	1	CTSL_LSEC
197	1.27E-33	0.262541217	0.431	0.135	2.77E-29	TBXA2R	1	CTSL_LSEC
198	6.74E-21	0.262107034	0.969	0.691	1.47E-16	ITGB1	1	CTSL_LSEC
199	1.86E-28	0.261758303	0.426	0.154	4.05E-24	TMTC4	1	CTSL_LSEC
200	4.84E-26	0.260846738	0.804	0.411	1.06E-21	NRP1	1	CTSL_LSEC
201	2.75E-25	0.260644336	0.679	0.34	6.00E-21	DEGS1	1	CTSL_LSEC
202	2.28E-25	0.260603125	0.796	0.438	4.98E-21	CCNG1	1	CTSL_LSEC
203	3.12E-31	0.257351521	0.763	0.353	6.81E-27	NR2F1	1	CTSL_LSEC
204	1.99E-25	0.254871656	0.724	0.367	4.33E-21	CTSF	1	CTSL_LSEC
205	4.18E-28	0.253877979	0.599	0.266	9.11E-24	EPS15	1	CTSL_LSEC
206	5.96E-29	0.252789035	0.934	0.564	1.30E-24	MAF	1	CTSL_LSEC
207	5.36E-20	0.251449902	0.977	0.707	1.17E-15	LIFR	1	CTSL_LSEC
208	7.00E-29	0.251303398	0.571	0.245	1.53E-24	BFAR	1	CTSL_LSEC

209	1.25E-31	0.251042801	0.357	0.103	2.72E-27	HS3ST3B1	1	CTSL_LSEC
210	1.93E-27	0.250990625	0.635	0.29	4.21E-23	BLCAP	1	CTSL_LSEC
211	7.73E-08	-0.260245385	0.964	0.858	0.001685292	RPS26	1	CTSL_LSEC
212	9.43E-11	-0.262616615	1	0.985	2.06E-06	TMSB4X	1	CTSL_LSEC
213	2.27E-08	-0.262707869	0.954	0.839	0.000494926	TMA7	1	CTSL_LSEC
214	8.34E-12	-0.269758812	0.997	0.945	1.82E-07	PTMA	1	CTSL_LSEC
215	2.67E-31	-0.28292106	0.995	0.988	5.83E-27	RPL34	1	CTSL_LSEC
216	4.62E-25	-0.336068874	1	0.977	1.01E-20	IFITM3	1	CTSL_LSEC
217	3.03E-17	-0.346803818	0.997	0.935	6.61E-13	IFITM2	1	CTSL_LSEC
218	2.70E-07	-0.370398959	0.916	0.789	0.005895728	SRGN	1	CTSL_LSEC
219	3.37E-13	-0.448162095	0.908	0.786	7.35E-09	CLEC3B	1	CTSL_LSEC
220	8.52E-10	-0.454140524	0.921	0.798	1.86E-05	HSPG2	1	CTSL_LSEC
221	4.07E-07	-0.457249401	0.291	0.367	0.008886204	JAM2	1	CTSL_LSEC
222	2.07E-20	-0.461920922	0.926	0.824	4.51E-16	FKBP1A	1	CTSL_LSEC
223	2.86E-08	-0.462720364	0.122	0.239	0.000622696	LTC4S	1	CTSL_LSEC
224	1.16E-10	-0.471629079	0.117	0.257	2.52E-06	LGALS3	1	CTSL_LSEC
225	2.18E-07	-0.474899017	0.839	0.694	0.004755075	EPAS1	1	CTSL_LSEC
226	6.96E-13	-0.490467276	0.898	0.774	1.52E-08	CALCRL	1	CTSL_LSEC
227	2.63E-09	-0.503622706	0.253	0.36	5.73E-05	EMP2	1	CTSL_LSEC
228	4.93E-07	-0.503713387	0.196	0.29	0.010761571	FAM107A	1	CTSL_LSEC
229	3.42E-11	-0.510661001	0.768	0.694	7.46E-07	S100A11	1	CTSL_LSEC
230	4.56E-09	-0.550102422	0.375	0.442	9.95E-05	BCAM	1	CTSL_LSEC
231	3.45E-07	-0.57245724	0.434	0.468	0.007525383	ATP1A1	1	CTSL_LSEC
232	1.35E-11	-0.574439462	0.133	0.282	2.94E-07	ALPL	1	CTSL_LSEC
233	1.01E-10	-0.591254167	0.153	0.287	2.20E-06	LTBP4	1	CTSL_LSEC
234	1.41E-07	-0.626994215	0.347	0.416	0.003079875	MATN2	1	CTSL_LSEC
235	4.80E-08	-0.628499408	0.258	0.355	0.001046287	HLA-DRA	1	CTSL_LSEC
236	4.67E-08	-0.635690334	0.452	0.506	0.001019249	GJA4	1	CTSL_LSEC
237	2.13E-54	-0.63851078	1	0.988	4.66E-50	TMSB10	1	CTSL_LSEC
238	6.09E-07	-0.646057597	0.872	0.731	0.013284626	PLPP1	1	CTSL_LSEC
239	2.28E-12	-0.657645538	0.74	0.653	4.97E-08	CLEC14A	1	CTSL_LSEC
240	1.88E-13	-0.675259046	0.707	0.67	4.10E-09	CRIP2	1	CTSL_LSEC
241	7.03E-18	-0.675525349	0.929	0.879	1.53E-13	HSPB1	1	CTSL_LSEC
242	9.90E-25	-0.701411123	0.885	0.801	2.16E-20	IFITM1	1	CTSL_LSEC
243	7.21E-07	-0.723340566	0.446	0.514	0.015732541	EMP1	1	CTSL_LSEC
244	7.77E-13	-0.735725001	0.594	0.596	1.70E-08	TAGLN2	1	CTSL_LSEC
245	6.33E-15	-0.787653731	0.651	0.638	1.38E-10	RAMP2	1	CTSL_LSEC
246	1.05E-06	-0.895946684	0.633	0.57	0.022911557	GSN	1	CTSL_LSEC
247	2.87E-34	-0.942472168	0.75	0.8	6.26E-30	RNASE1	1	CTSL_LSEC
248	1.61E-10	-0.979119381	0.934	0.868	3.52E-06	MT2A	1	CTSL_LSEC
249	2.39E-24	-1.067606787	0.694	0.72	5.22E-20	CD74	1	CTSL_LSEC
250	5.00E-08	-1.079107656	0.207	0.307	0.001091348	CLU	1	CTSL_LSEC
251	1.64E-13	-1.104195981	0.265	0.403	3.57E-09	PLVAP	1	CTSL_LSEC
252	5.36E-09	-1.110855656	0.222	0.341	0.000116936	MT1E	1	CTSL_LSEC
253	1.82E-14	-1.316763756	0.589	0.606	3.97E-10	S100A6	1	CTSL_LSEC
254	2.78E-28	-1.349930765	0.128	0.394	6.06E-24	VWF	1	CTSL_LSEC
255	1.21E-32	-1.400353097	0.161	0.449	2.64E-28	ADIRF	1	CTSL_LSEC
256	1.35E-41	-1.494399233	0.824	0.795	2.95E-37	VIM	1	CTSL_LSEC
257	1.16E-65	0.868074669	0.894	0.443	2.54E-61	BGN	2	BGN_LSEC
258	2.84E-67	0.76295241	0.728	0.24	6.20E-63	CPM	2	BGN_LSEC
259	1.35E-44	0.744659386	0.813	0.421	2.93E-40	IGFBP3	2	BGN_LSEC
260	2.27E-27	0.731913781	0.477	0.192	4.96E-23	CD24	2	BGN_LSEC
261	1.84E-46	0.655637287	0.988	0.744	4.02E-42	PLPP3	2	BGN_LSEC
262	3.67E-40	0.578479916	0.737	0.323	8.00E-36	MATN2	2	BGN_LSEC
263	2.55E-35	0.565521591	0.994	0.743	5.55E-31	CRHBP	2	BGN_LSEC
264	2.73E-38	0.537827341	0.61	0.245	5.95E-34	FAM84A	2	BGN_LSEC
265	3.72E-31	0.502656935	0.776	0.427	8.12E-27	BMPER	2	BGN_LSEC
266	5.55E-35	0.471153942	0.529	0.197	1.21E-30	NUDT10	2	BGN_LSEC
267	1.77E-25	0.469760633	0.692	0.371	3.85E-21	EDNRB	2	BGN_LSEC
268	3.18E-28	0.441213299	0.931	0.613	6.93E-24	CLEC14A	2	BGN_LSEC
269	1.97E-18	0.439552559	0.202	0.056	4.29E-14	FGF23	2	BGN_LSEC
270	8.04E-26	0.435522474	0.761	0.44	1.75E-21	SC5D	2	BGN_LSEC
271	2.81E-27	0.403891348	0.97	0.712	6.14E-23	PRCP	2	BGN_LSEC
272	4.14E-18	0.388351673	0.656	0.372	9.03E-14	OLFM1	2	BGN_LSEC
273	5.77E-21	0.386091937	0.577	0.298	1.26E-16	HBEGF	2	BGN_LSEC
274	2.19E-24	0.368981875	0.894	0.56	4.77E-20	F2R	2	BGN_LSEC
275	4.30E-17	0.359832742	0.943	0.732	9.37E-13	SOCS3	2	BGN_LSEC
276	1.09E-24	0.358942669	0.985	0.774	2.38E-20	CLEC3B	2	BGN_LSEC
277	6.18E-22	0.357053396	0.625	0.321	1.35E-17	HECW2	2	BGN_LSEC
278	1.02E-15	0.356147817	0.861	0.647	2.23E-11	TSC22D3	2	BGN_LSEC

279	2.05E-29	0.354629524	0.48	0.178	4.47E-25	GPRC5C	2	BGN_LSEC
280	5.94E-17	0.351076423	0.991	0.838	1.30E-12	FOS	2	BGN_LSEC
281	3.91E-18	0.343011664	0.767	0.488	8.53E-14	EFNB1	2	BGN_LSEC
282	1.69E-20	0.341300064	0.776	0.459	3.68E-16	ADD3	2	BGN_LSEC
283	1.09E-21	0.341278834	0.619	0.317	2.39E-17	LHFP	2	BGN_LSEC
284	1.03E-16	0.334232782	0.903	0.638	2.25E-12	DUSP6	2	BGN_LSEC
285	4.54E-15	0.325861693	0.967	0.783	9.90E-11	SRGN	2	BGN_LSEC
286	6.79E-12	0.322001928	0.909	0.707	1.48E-07	DDIT4	2	BGN_LSEC
287	1.02E-24	0.320094856	0.42	0.157	2.24E-20	RP11-767I20.1	2	BGN_LSEC
288	3.95E-15	0.318789219	0.562	0.318	8.61E-11	LRG1	2	BGN_LSEC
289	4.34E-21	0.316736316	0.97	0.765	9.46E-17	LDB2	2	BGN_LSEC
290	1.44E-17	0.313227211	0.589	0.321	3.15E-13	PCDH17	2	BGN_LSEC
291	2.74E-16	0.312419663	0.903	0.631	5.97E-12	FOSB	2	BGN_LSEC
292	1.39E-22	0.312197928	0.459	0.187	3.03E-18	FAM198B	2	BGN_LSEC
293	1.25E-17	0.30965744	0.837	0.534	2.72E-13	ST6GALNAC3	2	BGN_LSEC
294	4.45E-16	0.308748279	0.931	0.727	9.71E-12	F8	2	BGN_LSEC
295	5.92E-15	0.308133756	0.994	0.803	1.29E-10	ZFP36	2	BGN_LSEC
296	2.81E-16	0.305237666	0.918	0.661	6.12E-12	CDKN1A	2	BGN_LSEC
297	8.28E-18	0.300062613	0.982	0.799	1.80E-13	TSPAN7	2	BGN_LSEC
298	6.08E-20	0.299186309	0.514	0.243	1.33E-15	DSE	2	BGN_LSEC
299	8.16E-18	0.298330261	0.915	0.658	1.78E-13	ETS2	2	BGN_LSEC
300	1.83E-18	0.297104461	0.991	0.857	3.98E-14	DUSP1	2	BGN_LSEC
301	1.41E-11	0.293903836	0.909	0.737	3.07E-07	RHOB	2	BGN_LSEC
302	2.76E-18	0.291712076	0.991	0.818	6.02E-14	JUNB	2	BGN_LSEC
303	4.45E-17	0.291262642	0.985	0.816	9.72E-13	JUN	2	BGN_LSEC
304	6.54E-16	0.289243887	0.976	0.748	1.43E-11	TINAGL1	2	BGN_LSEC
305	5.22E-26	0.289108326	0.526	0.222	1.14E-21	CLEC11A	2	BGN_LSEC
306	3.13E-15	0.288692099	0.387	0.183	6.83E-11	RBKS	2	BGN_LSEC
307	1.14E-18	0.288328684	0.864	0.562	2.49E-14	SRPX	2	BGN_LSEC
308	3.62E-14	0.286153354	0.949	0.684	7.90E-10	AKAP12	2	BGN_LSEC
309	8.61E-11	0.285951669	0.695	0.473	1.88E-06	HES1	2	BGN_LSEC
310	1.57E-17	0.283206382	0.961	0.725	3.42E-13	A2M	2	BGN_LSEC
311	7.21E-15	0.281522193	0.927	0.666	1.57E-10	KLF4	2	BGN_LSEC
312	7.94E-14	0.27977184	0.689	0.429	1.73E-09	MDK	2	BGN_LSEC
313	4.25E-19	0.2796894	0.918	0.637	9.27E-15	RDX	2	BGN_LSEC
314	4.79E-16	0.278970549	0.921	0.633	1.04E-11	ADGRF5	2	BGN_LSEC
315	1.13E-10	0.27887863	0.713	0.492	2.47E-06	GPX3	2	BGN_LSEC
316	8.37E-24	0.278247219	0.311	0.098	1.83E-19	SNAI2	2	BGN_LSEC
317	1.11E-12	0.278059232	0.934	0.72	2.42E-08	CLDN5	2	BGN_LSEC
318	5.51E-17	0.274154666	0.994	0.839	1.20E-12	IL33	2	BGN_LSEC
319	1.41E-15	0.274135769	0.779	0.5	3.07E-11	TFPI2	2	BGN_LSEC
320	7.07E-12	0.272977678	0.779	0.535	1.54E-07	MYC	2	BGN_LSEC
321	1.78E-14	0.270637671	0.704	0.437	3.87E-10	PDCD4	2	BGN_LSEC
322	6.60E-17	0.270611623	0.532	0.271	1.44E-12	SPRY4	2	BGN_LSEC
323	3.09E-13	0.270169271	0.671	0.417	6.73E-09	SOX17	2	BGN_LSEC
324	3.08E-14	0.265585024	0.538	0.295	6.72E-10	ERVK3-1	2	BGN_LSEC
325	5.38E-13	0.262386297	0.447	0.238	1.17E-08	PAPSS2	2	BGN_LSEC
326	4.87E-17	0.260569911	0.813	0.505	1.06E-12	NR4A1	2	BGN_LSEC
327	2.35E-17	0.260256649	0.55	0.281	5.13E-13	APOLD1	2	BGN_LSEC
328	7.02E-11	0.259683319	0.541	0.335	1.53E-06	CITED2	2	BGN_LSEC
329	1.02E-12	0.255368934	0.958	0.711	2.23E-08	PDIA3	2	BGN_LSEC
330	8.51E-18	0.254094485	0.746	0.441	1.86E-13	NRP1	2	BGN_LSEC
331	1.71E-14	0.252259602	0.819	0.517	3.73E-10	CD36	2	BGN_LSEC
332	1.53E-10	0.250641437	0.888	0.652	3.33E-06	PPP1R15A	2	BGN_LSEC
333	6.88E-09	-0.379080544	1	0.962	0.000150114	MT-CO1	2	BGN_LSEC
334	7.74E-10	-0.405609443	0.997	0.954	1.69E-05	MT-CO3	2	BGN_LSEC
335	4.23E-08	-0.442762022	0.952	0.872	0.000921935	MT-ND2	2	BGN_LSEC
336	7.01E-11	-0.472692503	0.994	0.933	1.53E-06	MT-ND3	2	BGN_LSEC
337	2.48E-23	-0.494841738	0.967	0.928	5.41E-19	S100A10	2	BGN_LSEC
338	1.56E-09	-0.775769966	0.112	0.254	3.40E-05	AQP1	2	BGN_LSEC
339	1.29E-13	-1.191506278	0.523	0.621	2.82E-09	S100A6	2	BGN_LSEC
340	4.73E-24	1.052984986	0.779	0.645	1.03E-19	STAB1	3	STAB1_LSEC
341	1.32E-25	0.956496694	0.432	0.197	2.87E-21	MT1G	3	STAB1_LSEC
342	1.43E-25	0.938978236	0.656	0.445	3.12E-21	MEG3	3	STAB1_LSEC
343	1.38E-13	0.924362105	0.405	0.249	3.01E-09	SERPINA1	3	STAB1_LSEC
344	4.79E-29	0.901852515	0.956	0.824	1.04E-24	NEAT1	3	STAB1_LSEC
345	1.25E-65	0.820607471	1	0.974	2.72E-61	MT-CO2	3	STAB1_LSEC
346	7.91E-09	0.805365331	0.429	0.32	0.000172516	ALB	3	STAB1_LSEC
347	7.12E-66	0.802903196	0.997	0.964	1.55E-61	MT-CO1	3	STAB1_LSEC
348	7.96E-45	0.787162804	0.98	0.885	1.74E-40	MT-ATP8	3	STAB1_LSEC

349	8.77E-45	0.763277721	0.98	0.937	1.91E-40	MT-ATP6	3	STAB1_LSEC
350	3.38E-45	0.738611406	0.966	0.872	7.38E-41	MT-ND2	3	STAB1_LSEC
351	1.35E-49	0.730124027	0.997	0.955	2.93E-45	MT-CO3	3	STAB1_LSEC
352	2.76E-46	0.71457163	0.983	0.929	6.01E-42	MT-ND4L	3	STAB1_LSEC
353	1.32E-13	0.694557122	0.306	0.151	2.88E-09	APOC1	3	STAB1_LSEC
354	2.59E-44	0.685776783	0.99	0.935	5.65E-40	MT-ND3	3	STAB1_LSEC
355	1.08E-06	0.682671972	0.102	0.037	0.023523531	HAMP	3	STAB1_LSEC
356	1.44E-44	0.682122986	0.986	0.922	3.13E-40	MT-CYB	3	STAB1_LSEC
357	3.80E-34	0.679097758	0.969	0.872	8.28E-30	MT-ND5	3	STAB1_LSEC
358	1.62E-35	0.648982343	0.98	0.901	3.53E-31	MT-ND4	3	STAB1_LSEC
359	4.08E-13	0.646501053	0.276	0.127	8.90E-09	APOC3	3	STAB1_LSEC
360	2.53E-11	0.640578572	0.636	0.497	5.51E-07	STAB2	3	STAB1_LSEC
361	1.67E-31	0.638233798	0.966	0.885	3.65E-27	MT-ND1	3	STAB1_LSEC
362	4.39E-27	0.636859253	0.99	0.893	9.58E-23	MALAT1	3	STAB1_LSEC
363	4.71E-07	0.585536917	0.534	0.512	0.010274506	TNFRSF10D	3	STAB1_LSEC
364	7.42E-19	0.565307564	0.799	0.701	1.62E-14	PPFIBP1	3	STAB1_LSEC
365	1.05E-10	0.552192479	0.82	0.772	2.30E-06	SLC2A3	3	STAB1_LSEC
366	1.69E-19	0.528656311	0.153	0.03	3.68E-15	FABP1	3	STAB1_LSEC
367	1.08E-10	0.497879279	0.718	0.558	2.35E-06	CLEC4M	3	STAB1_LSEC
368	1.52E-06	0.495680606	0.694	0.67	0.033077892	PDK4	3	STAB1_LSEC
369	1.38E-08	0.47222887	0.599	0.561	0.000301572	N4BP2L2	3	STAB1_LSEC
370	1.67E-07	0.44262323	0.687	0.644	0.003647117	ADM	3	STAB1_LSEC
371	6.99E-07	0.42648453	0.677	0.656	0.015242688	FUS	3	STAB1_LSEC
372	4.61E-11	0.381367959	0.16	0.055	1.01E-06	SAA2	3	STAB1_LSEC
373	3.17E-09	0.379773861	0.711	0.683	6.91E-05	CLEC2B	3	STAB1_LSEC
374	3.48E-14	0.353395633	0.173	0.052	7.58E-10	APOA2	3	STAB1_LSEC
375	2.41E-14	0.346680814	0.976	0.924	5.26E-10	CCL14	3	STAB1_LSEC
376	4.11E-10	0.339179833	0.827	0.664	8.96E-06	FCN2	3	STAB1_LSEC
377	9.20E-09	0.332891644	0.129	0.045	0.000200545	TTR	3	STAB1_LSEC
378	9.15E-11	0.308360197	0.942	0.802	1.99E-06	DNASE1L3	3	STAB1_LSEC
379	2.56E-09	0.307423338	0.105	0.031	5.58E-05	ALDOB	3	STAB1_LSEC
380	8.82E-10	0.291761346	0.119	0.036	1.92E-05	MT1H	3	STAB1_LSEC
381	1.33E-06	0.286321565	0.102	0.038	0.029002536	ADH1B	3	STAB1_LSEC
382	2.85E-15	0.281037509	0.105	0.018	6.21E-11	AGXT	3	STAB1_LSEC
383	2.59E-09	-0.250066121	0.184	0.392	5.66E-05	SMAGP	3	STAB1_LSEC
384	2.35E-07	-0.250097814	0.558	0.76	0.005114015	CTSB	3	STAB1_LSEC
385	1.42E-09	-0.250273452	0.163	0.376	3.10E-05	ERVK3-1	3	STAB1_LSEC
386	1.53E-07	-0.250322637	0.102	0.251	0.003334213	LGALS3	3	STAB1_LSEC
387	6.88E-09	-0.251208753	0.452	0.687	0.000150015	DAZAP2	3	STAB1_LSEC
388	1.98E-08	-0.25158843	0.442	0.647	0.000431635	EGR1	3	STAB1_LSEC
389	3.00E-07	-0.251760896	0.31	0.523	0.006539924	MRPL33	3	STAB1_LSEC
390	1.28E-11	-0.251925176	0.293	0.584	2.79E-07	CDC42	3	STAB1_LSEC
391	9.66E-09	-0.252169359	0.381	0.641	0.000210769	SMDT1	3	STAB1_LSEC
392	5.93E-11	-0.25268407	0.466	0.749	1.29E-06	ANXA5	3	STAB1_LSEC
393	5.60E-10	-0.252761497	0.418	0.694	1.22E-05	C12orf57	3	STAB1_LSEC
394	1.35E-09	-0.253751855	0.463	0.702	2.93E-05	LDHA	3	STAB1_LSEC
395	8.17E-09	-0.254119938	0.486	0.732	0.00017828	PTRF	3	STAB1_LSEC
396	4.44E-14	-0.254399554	0.881	0.97	9.68E-10	RPS9	3	STAB1_LSEC
397	6.05E-09	-0.254617164	0.197	0.406	0.000131845	PEA15	3	STAB1_LSEC
398	1.35E-08	-0.256237083	0.435	0.681	0.000295332	AP2M1	3	STAB1_LSEC
399	1.86E-06	-0.256368877	0.133	0.275	0.040556095	SLCO2A1	3	STAB1_LSEC
400	3.67E-07	-0.256503374	0.493	0.693	0.007995817	HEBP1	3	STAB1_LSEC
401	4.60E-12	-0.256832492	0.238	0.507	1.00E-07	VAMP3	3	STAB1_LSEC
402	5.35E-16	-0.256853218	0.949	0.998	1.17E-11	RPS27	3	STAB1_LSEC
403	2.23E-09	-0.257148796	0.19	0.408	4.86E-05	PSMB5	3	STAB1_LSEC
404	3.04E-08	-0.257475819	0.551	0.771	0.00066301	EIF3F	3	STAB1_LSEC
405	2.94E-08	-0.257998246	0.187	0.382	0.000642054	JAM2	3	STAB1_LSEC
406	2.48E-10	-0.258261266	0.418	0.673	5.41E-06	RAP1B	3	STAB1_LSEC
407	9.46E-10	-0.259336826	0.259	0.5	2.06E-05	LTA4H	3	STAB1_LSEC
408	4.78E-11	-0.259715182	0.211	0.456	1.04E-06	PPP2R1A	3	STAB1_LSEC
409	1.55E-08	-0.260659716	0.259	0.478	0.000337607	IGBP1	3	STAB1_LSEC
410	3.44E-10	-0.261001498	0.224	0.46	7.51E-06	PGAM1	3	STAB1_LSEC
411	6.51E-14	-0.261689969	0.901	0.984	1.42E-09	RPL7A	3	STAB1_LSEC
412	7.65E-08	-0.261765914	0.167	0.347	0.001667629	ADAM15	3	STAB1_LSEC
413	4.22E-11	-0.261843482	0.231	0.474	9.20E-07	LAP3	3	STAB1_LSEC
414	2.01E-10	-0.262534831	0.136	0.337	4.38E-06	ADIPOR1	3	STAB1_LSEC
415	3.13E-12	-0.263221375	0.833	0.949	6.83E-08	RPS2	3	STAB1_LSEC
416	2.31E-09	-0.26335753	0.293	0.531	5.03E-05	EIF3M	3	STAB1_LSEC
417	1.41E-08	-0.264098179	0.139	0.317	0.000308006	RFK	3	STAB1_LSEC
418	1.26E-11	-0.267516029	0.347	0.627	2.74E-07	TMED2	3	STAB1_LSEC

419	2.43E-10	-0.269371191	0.395	0.658	5.31E-06	GNB2	3	STAB1_LSEC
420	3.78E-11	-0.269547758	0.279	0.557	8.24E-07	RNH1	3	STAB1_LSEC
421	1.33E-10	-0.269883379	0.435	0.716	2.90E-06	TPM3	3	STAB1_LSEC
422	1.40E-11	-0.270233164	0.177	0.425	3.06E-07	PSMD8	3	STAB1_LSEC
423	3.96E-10	-0.270421008	0.354	0.615	8.63E-06	NRN1	3	STAB1_LSEC
424	1.10E-10	-0.270429205	0.293	0.56	2.39E-06	CAP1	3	STAB1_LSEC
425	1.49E-09	-0.27092875	0.568	0.803	3.25E-05	YBX1	3	STAB1_LSEC
426	1.04E-09	-0.271018471	0.408	0.679	2.27E-05	GABARAPL2	3	STAB1_LSEC
427	4.21E-11	-0.271363041	0.177	0.413	9.18E-07	LHFP	3	STAB1_LSEC
428	4.27E-12	-0.271468478	0.173	0.418	9.32E-08	RNF11	3	STAB1_LSEC
429	5.75E-11	-0.272148756	0.367	0.65	1.25E-06	SLC25A5	3	STAB1_LSEC
430	1.40E-11	-0.273015069	0.146	0.376	3.05E-07	RRAGA	3	STAB1_LSEC
431	6.46E-11	-0.2734266	0.459	0.737	1.41E-06	WDR83OS	3	STAB1_LSEC
432	2.44E-11	-0.274048303	0.197	0.438	5.33E-07	GHITM	3	STAB1_LSEC
433	8.10E-08	-0.274470124	0.656	0.844	0.001765943	RHOC	3	STAB1_LSEC
434	4.07E-10	-0.274508842	0.31	0.559	8.88E-06	PGK1	3	STAB1_LSEC
435	9.05E-11	-0.275042184	0.32	0.585	1.97E-06	WARS	3	STAB1_LSEC
436	2.24E-08	-0.275774042	0.507	0.737	0.000488238	HYAL2	3	STAB1_LSEC
437	3.88E-10	-0.277801502	0.35	0.615	8.47E-06	HERPUD1	3	STAB1_LSEC
438	6.31E-11	-0.277832101	0.245	0.505	1.38E-06	CHMP5	3	STAB1_LSEC
439	1.07E-10	-0.278133251	0.449	0.717	2.32E-06	ARPC2	3	STAB1_LSEC
440	1.39E-15	-0.279152198	0.874	0.984	3.04E-11	RPS4X	3	STAB1_LSEC
441	1.17E-21	-0.281357356	0.966	0.997	2.55E-17	AC090498.1	3	STAB1_LSEC
442	7.80E-08	-0.281530731	0.527	0.763	0.001700845	TPI1	3	STAB1_LSEC
443	2.25E-10	-0.28182226	0.126	0.33	4.90E-06	PSMD4	3	STAB1_LSEC
444	3.67E-09	-0.282238134	0.286	0.51	8.01E-05	SOX7	3	STAB1_LSEC
445	9.86E-12	-0.283648375	0.194	0.446	2.15E-07	RAB1B	3	STAB1_LSEC
446	6.21E-11	-0.283843084	0.224	0.471	1.35E-06	EIF2S3	3	STAB1_LSEC
447	2.78E-13	-0.28548803	0.116	0.355	6.06E-09	MAPK1IP1L	3	STAB1_LSEC
448	1.77E-08	-0.285955216	0.531	0.738	0.000386305	S100A16	3	STAB1_LSEC
449	5.61E-12	-0.287278976	0.197	0.448	1.22E-07	RAP1A	3	STAB1_LSEC
450	4.44E-07	-0.288532029	0.646	0.797	0.009688477	GYPC	3	STAB1_LSEC
451	9.69E-11	-0.289338511	0.517	0.752	2.11E-06	SLC25A3	3	STAB1_LSEC
452	5.54E-11	-0.289602967	0.303	0.566	1.21E-06	CYB5R3	3	STAB1_LSEC
453	3.19E-12	-0.293471835	0.578	0.826	6.96E-08	SLC25A6	3	STAB1_LSEC
454	2.26E-14	-0.293731949	0.18	0.461	4.94E-10	ANXA11	3	STAB1_LSEC
455	2.59E-13	-0.294123959	0.269	0.56	5.64E-09	SERINC3	3	STAB1_LSEC
456	7.24E-13	-0.29553109	0.327	0.616	1.58E-08	ERGIC3	3	STAB1_LSEC
457	9.21E-08	-0.295637373	0.51	0.741	0.002007826	DNAJB1	3	STAB1_LSEC
458	3.35E-11	-0.297712526	0.187	0.419	7.31E-07	TMEM109	3	STAB1_LSEC
459	6.85E-21	-0.298954597	0.901	0.986	1.49E-16	RPL15	3	STAB1_LSEC
460	1.64E-13	-0.299192956	0.35	0.645	3.57E-09	CSDE1	3	STAB1_LSEC
461	2.13E-10	-0.299754636	0.286	0.533	4.65E-06	UBE2J1	3	STAB1_LSEC
462	1.17E-11	-0.301515113	0.156	0.389	2.56E-07	PSMB2	3	STAB1_LSEC
463	4.89E-10	-0.301533845	0.714	0.889	1.07E-05	HSP90AB1	3	STAB1_LSEC
464	2.37E-11	-0.30324508	0.293	0.552	5.17E-07	ARPC1B	3	STAB1_LSEC
465	3.44E-09	-0.30792635	0.265	0.499	7.49E-05	ATP1A1	3	STAB1_LSEC
466	7.56E-14	-0.308094678	0.762	0.925	1.65E-09	RPL10A	3	STAB1_LSEC
467	4.42E-09	-0.309671283	0.66	0.815	9.64E-05	PLPP3	3	STAB1_LSEC
468	9.27E-17	-0.310788889	0.833	0.959	2.02E-12	RPLP0	3	STAB1_LSEC
469	7.36E-18	-0.311265715	0.85	0.973	1.61E-13	RPL17	3	STAB1_LSEC
470	2.32E-08	-0.311609357	0.235	0.433	0.000506359	MATN2	3	STAB1_LSEC
471	3.44E-13	-0.316972904	0.272	0.546	7.50E-09	BZW1	3	STAB1_LSEC
472	1.57E-08	-0.317125742	0.116	0.285	0.000341496	LTBP4	3	STAB1_LSEC
473	4.78E-13	-0.31755422	0.541	0.791	1.04E-08	LAPTM4A	3	STAB1_LSEC
474	1.29E-14	-0.317932445	0.344	0.648	2.81E-10	ATP5B	3	STAB1_LSEC
475	8.20E-12	-0.318271854	0.697	0.883	1.79E-07	BST2	3	STAB1_LSEC
476	5.18E-16	-0.319324127	0.714	0.921	1.13E-11	EEF1D	3	STAB1_LSEC
477	3.82E-13	-0.323473568	0.422	0.705	8.34E-09	VDAC2	3	STAB1_LSEC
478	3.87E-28	-0.327618775	0.983	0.999	8.44E-24	RPL10	3	STAB1_LSEC
479	8.97E-15	-0.331225988	0.429	0.719	1.96E-10	YWHAZ	3	STAB1_LSEC
480	2.08E-14	-0.33193103	0.745	0.912	4.53E-10	IGFBP4	3	STAB1_LSEC
481	1.76E-09	-0.332740068	0.551	0.785	3.84E-05	SNHG7	3	STAB1_LSEC
482	1.44E-22	-0.333229793	0.881	0.984	3.13E-18	RPS3	3	STAB1_LSEC
483	2.47E-08	-0.334705462	0.129	0.298	0.000537909	FAM107A	3	STAB1_LSEC
484	1.16E-08	-0.335700411	0.697	0.842	0.000253145	SPARC	3	STAB1_LSEC
485	8.92E-12	-0.33702413	0.605	0.829	1.95E-07	CNN3	3	STAB1_LSEC
486	2.54E-14	-0.337174818	0.49	0.759	5.54E-10	TPM4	3	STAB1_LSEC
487	2.22E-22	-0.337462511	0.847	0.979	4.85E-18	GNB2L1	3	STAB1_LSEC
488	1.87E-13	-0.338194835	0.687	0.91	4.09E-09	MYL12B	3	STAB1_LSEC

489	1.63E-12	-0.341098608	0.371	0.647	3.56E-08	EMCN	3	STAB1_LSEC
490	1.05E-19	-0.343148849	0.844	0.967	2.28E-15	RPL5	3	STAB1_LSEC
491	3.68E-14	-0.344829135	0.231	0.52	8.03E-10	VAT1	3	STAB1_LSEC
492	4.06E-08	-0.345249015	0.279	0.484	0.000886088	PNP	3	STAB1_LSEC
493	6.65E-13	-0.3480834	0.269	0.545	1.45E-08	GINM1	3	STAB1_LSEC
494	9.03E-19	-0.350133809	0.813	0.957	1.97E-14	RPL21	3	STAB1_LSEC
495	1.57E-12	-0.3510566	0.595	0.809	3.42E-08	ANXA2	3	STAB1_LSEC
496	2.39E-16	-0.352832624	0.653	0.883	5.21E-12	SDCBP	3	STAB1_LSEC
497	4.19E-15	-0.353991266	0.493	0.78	9.13E-11	PTGES3	3	STAB1_LSEC
498	4.56E-11	-0.354204296	0.395	0.658	9.95E-07	ESAM	3	STAB1_LSEC
499	6.67E-25	-0.354939858	0.925	0.988	1.46E-20	GAPDH	3	STAB1_LSEC
500	2.54E-11	-0.356712928	0.197	0.432	5.53E-07	LRRC32	3	STAB1_LSEC
501	6.89E-14	-0.357222493	0.459	0.739	1.50E-09	GUK1	3	STAB1_LSEC
502	1.95E-10	-0.357612314	0.463	0.714	4.25E-06	CLEC14A	3	STAB1_LSEC
503	4.72E-23	-0.359774017	0.881	0.971	1.03E-18	HLA-E	3	STAB1_LSEC
504	1.55E-16	-0.359887475	0.388	0.717	3.38E-12	ARF1	3	STAB1_LSEC
505	3.16E-11	-0.361264918	0.456	0.723	6.90E-07	CRIP2	3	STAB1_LSEC
506	2.25E-13	-0.363287237	0.507	0.787	4.90E-09	EIF3K	3	STAB1_LSEC
507	7.15E-14	-0.365738955	0.306	0.594	1.56E-09	TUBB	3	STAB1_LSEC
508	2.03E-14	-0.370205171	0.327	0.619	4.43E-10	EIF3L	3	STAB1_LSEC
509	3.14E-13	-0.370545522	0.463	0.743	6.85E-09	DSTN	3	STAB1_LSEC
510	1.89E-22	-0.371589758	0.816	0.927	4.13E-18	PCBP2	3	STAB1_LSEC
511	4.19E-14	-0.376215685	0.503	0.775	9.14E-10	MORF4L1	3	STAB1_LSEC
512	5.84E-16	-0.376477879	0.306	0.608	1.27E-11	BNIP3L	3	STAB1_LSEC
513	2.75E-16	-0.379012841	0.201	0.493	5.99E-12	ARPC1A	3	STAB1_LSEC
514	4.42E-15	-0.37980162	0.272	0.577	9.63E-11	CLIC4	3	STAB1_LSEC
515	8.87E-16	-0.381606862	0.364	0.668	1.93E-11	CNBP	3	STAB1_LSEC
516	3.75E-19	-0.384588595	0.68	0.879	8.17E-15	PABPC1	3	STAB1_LSEC
517	3.58E-18	-0.384859461	0.85	0.972	7.81E-14	ACTB	3	STAB1_LSEC
518	1.92E-30	-0.386438706	0.901	0.984	4.19E-26	RPL8	3	STAB1_LSEC
519	7.62E-27	-0.386895574	0.864	0.98	1.66E-22	RPL3	3	STAB1_LSEC
520	7.19E-12	-0.392855025	0.299	0.573	1.57E-07	LMNA	3	STAB1_LSEC
521	6.16E-18	-0.394069775	0.357	0.671	1.34E-13	EIF4B	3	STAB1_LSEC
522	3.48E-16	-0.397816809	0.469	0.771	7.59E-12	PCBP1	3	STAB1_LSEC
523	1.01E-07	-0.39938034	0.241	0.424	0.002194731	HSPH1	3	STAB1_LSEC
524	9.74E-19	-0.40295415	0.796	0.938	2.12E-14	ACTG1	3	STAB1_LSEC
525	4.64E-18	-0.404062552	0.327	0.645	1.01E-13	GDI2	3	STAB1_LSEC
526	4.51E-10	-0.404767454	0.551	0.762	9.83E-06	EPAS1	3	STAB1_LSEC
527	1.11E-16	-0.406515311	0.561	0.827	2.41E-12	EIF3E	3	STAB1_LSEC
528	3.15E-17	-0.407331158	0.299	0.61	6.87E-13	CRTAP	3	STAB1_LSEC
529	2.27E-15	-0.408868409	0.677	0.87	4.94E-11	ALDOA	3	STAB1_LSEC
530	8.43E-09	-0.408973527	0.446	0.68	0.000183794	RAMP2	3	STAB1_LSEC
531	1.54E-28	-0.409906945	0.837	0.977	3.36E-24	RPS10	3	STAB1_LSEC
532	1.00E-17	-0.413468622	0.636	0.889	2.19E-13	FKBP1A	3	STAB1_LSEC
533	2.01E-06	-0.41380416	0.255	0.411	0.043816053	ADIRF	3	STAB1_LSEC
534	9.37E-19	-0.419940825	0.442	0.77	2.04E-14	CAPZA2	3	STAB1_LSEC
535	5.45E-18	-0.424675354	0.347	0.67	1.19E-13	HNRNPF	3	STAB1_LSEC
536	8.72E-09	-0.427225577	0.323	0.528	0.00019024	GJA4	3	STAB1_LSEC
537	7.78E-11	-0.434363387	0.296	0.55	1.70E-06	GLUL	3	STAB1_LSEC
538	1.32E-22	-0.443064995	0.673	0.888	2.89E-18	RPSA	3	STAB1_LSEC
539	1.80E-06	-0.469494528	0.565	0.744	0.039278921	CD74	3	STAB1_LSEC
540	4.59E-24	-0.471024117	0.65	0.889	1.00E-19	RHOA	3	STAB1_LSEC
541	2.49E-18	-0.476667571	0.361	0.681	5.43E-14	RAB5C	3	STAB1_LSEC
542	6.30E-24	-0.478350343	0.537	0.83	1.37E-19	EEF1G	3	STAB1_LSEC
543	1.60E-30	-0.480417241	0.759	0.95	3.49E-26	RPS5	3	STAB1_LSEC
544	3.57E-12	-0.48210454	0.306	0.551	7.78E-08	LITAF	3	STAB1_LSEC
545	9.04E-21	-0.495220002	0.367	0.697	1.97E-16	CAV1	3	STAB1_LSEC
546	1.29E-11	-0.496917932	0.252	0.501	2.82E-07	BAG3	3	STAB1_LSEC
547	6.52E-58	-0.510113832	0.98	0.999	1.42E-53	EEF1A1	3	STAB1_LSEC
548	2.25E-16	-0.518457306	0.517	0.747	4.90E-12	CDKN1A	3	STAB1_LSEC
549	6.83E-07	-0.52621794	0.221	0.385	0.014900479	MGP	3	STAB1_LSEC
550	2.83E-11	-0.536213294	0.565	0.802	6.18E-07	PLPP1	3	STAB1_LSEC
551	4.22E-31	-0.538669713	0.69	0.908	9.19E-27	EEF2	3	STAB1_LSEC
552	4.67E-29	-0.561138369	0.486	0.834	1.02E-24	CCNI	3	STAB1_LSEC
553	4.92E-19	-0.596280645	0.483	0.755	1.07E-14	PECAM1	3	STAB1_LSEC
554	2.32E-30	-0.6473466	0.588	0.877	5.05E-26	HSPA8	3	STAB1_LSEC
555	5.07E-20	-0.65776792	0.33	0.649	1.11E-15	TAGLN2	3	STAB1_LSEC
556	3.02E-10	-0.676570019	0.17	0.368	6.59E-06	VWF	3	STAB1_LSEC
557	2.67E-07	-0.687680912	0.459	0.631	0.005832914	S100A6	3	STAB1_LSEC
558	8.37E-14	-0.691620684	0.35	0.63	1.82E-09	GSN	3	STAB1_LSEC

559	1.46E-15	-0.80425011	0.701	0.821	3.19E-11	VIM	3	STAB1_LSEC
560	1.41E-14	-0.825817351	0.17	0.413	3.07E-10	PLVAP	3	STAB1_LSEC
561	6.93E-08	-0.896558431	0.18	0.324	0.001512183	SPARCL1	3	STAB1_LSEC
562	2.42E-166	2.257600283	0.982	0.239	5.28E-162	MGP	4	MGP_VEC
563	3.20E-134	1.763118842	0.915	0.284	6.98E-130	ADIRF	4	MGP_VEC
564	8.30E-84	1.701193889	0.695	0.207	1.81E-79	CLU	4	MGP_VEC
565	4.13E-18	1.28534918	0.433	0.214	9.01E-14	MT1M	4	MGP_VEC
566	3.83E-131	1.264592761	0.539	0.036	8.36E-127	CPE	4	MGP_VEC
567	9.32E-30	1.255133095	0.574	0.265	2.03E-25	MT1E	4	MGP_VEC
568	2.70E-96	1.195320382	0.433	0.034	5.89E-92	FN1	4	MGP_VEC
569	1.49E-98	1.192746269	0.996	0.764	3.24E-94	VIM	4	MGP_VEC
570	2.18E-100	1.170373424	0.833	0.199	4.75E-96	SPARCL1	4	MGP_VEC
571	1.46E-105	1.147321415	0.734	0.131	3.18E-101	AQP1	4	MGP_VEC
572	2.09E-39	1.133747024	0.457	0.147	4.56E-35	CD320	4	MGP_VEC
573	6.49E-38	1.09210042	0.798	0.505	1.42E-33	CXCL2	4	MGP_VEC
574	2.86E-38	1.063832721	0.362	0.09	6.24E-34	HES4	4	MGP_VEC
575	9.16E-79	1.056009979	0.78	0.25	2.00E-74	VWF	4	MGP_VEC
576	2.49E-23	1.036665978	0.188	0.036	5.43E-19	IL6	4	MGP_VEC
577	1.22E-63	1.028448548	0.624	0.187	2.67E-59	LTBP4	4	MGP_VEC
578	4.07E-32	0.951842932	0.372	0.106	8.88E-28	ANXA1	4	MGP_VEC
579	5.39E-19	0.942125392	0.521	0.303	1.18E-14	ANGPTL4	4	MGP_VEC
580	1.11E-48	0.876505913	0.734	0.337	2.42E-44	MATN2	4	MGP_VEC
581	1.34E-48	0.871673524	0.883	0.549	2.91E-44	S100A6	4	MGP_VEC
582	3.96E-21	0.86909956	0.706	0.493	8.64E-17	LMNA	4	MGP_VEC
583	2.07E-37	0.844652395	0.794	0.586	4.52E-33	SRPX	4	MGP_VEC
584	9.30E-27	0.803858273	0.745	0.452	2.03E-22	EMP1	4	MGP_VEC
585	1.43E-09	0.790104105	0.929	0.873	3.12E-05	MT2A	4	MGP_VEC
586	1.23E-63	0.759933364	0.323	0.03	2.69E-59	FBLN2	4	MGP_VEC
587	1.04E-10	0.742720671	0.262	0.122	2.28E-06	MT1A	4	MGP_VEC
588	5.32E-07	0.723409454	0.55	0.435	0.011592992	MT1X	4	MGP_VEC
589	3.60E-14	0.718349429	0.408	0.218	7.85E-10	VCAM1	4	MGP_VEC
590	1.43E-45	0.715285118	0.255	0.028	3.12E-41	NTS	4	MGP_VEC
591	2.49E-22	0.711710354	0.319	0.106	5.43E-18	STC1	4	MGP_VEC
592	2.75E-33	0.704796796	0.543	0.239	5.99E-29	RFK	4	MGP_VEC
593	1.73E-48	0.677725898	0.596	0.186	3.78E-44	SLCO2A1	4	MGP_VEC
594	7.63E-50	0.677004069	0.968	0.791	1.66E-45	IFITM1	4	MGP_VEC
595	8.58E-34	0.676162676	0.872	0.644	1.87E-29	C7	4	MGP_VEC
596	3.25E-32	0.671046716	0.298	0.065	7.08E-28	FABP5	4	MGP_VEC
597	2.75E-50	0.670462594	0.33	0.051	6.01E-46	BMX	4	MGP_VEC
598	1.84E-16	0.667362724	0.174	0.043	4.01E-12	MCTP1	4	MGP_VEC
599	6.90E-33	0.661635304	0.844	0.639	1.50E-28	CLEC14A	4	MGP_VEC
600	3.16E-57	0.657021783	0.305	0.032	6.89E-53	PLAC9	4	MGP_VEC
601	2.88E-66	0.651122055	0.301	0.022	6.28E-62	MPZL2	4	MGP_VEC
602	5.42E-17	0.650014868	0.447	0.231	1.18E-12	ATP13A3	4	MGP_VEC
603	1.42E-46	0.649798552	0.716	0.307	3.10E-42	PLVAP	4	MGP_VEC
604	2.86E-11	0.648538105	0.418	0.251	6.23E-07	ADAMTS9	4	MGP_VEC
605	6.38E-31	0.646182385	0.351	0.103	1.39E-26	SCGB3A1	4	MGP_VEC
606	1.24E-21	0.633159179	0.433	0.194	2.71E-17	COL8A1	4	MGP_VEC
607	1.06E-18	0.632110778	0.66	0.439	2.32E-14	SOX4	4	MGP_VEC
608	3.52E-42	0.627846094	0.277	0.042	7.67E-38	CDH11	4	MGP_VEC
609	4.35E-47	0.621021356	0.947	0.827	9.48E-43	FKBP1A	4	MGP_VEC
610	3.05E-20	0.620070245	0.741	0.509	6.65E-16	ADAMTS1	4	MGP_VEC
611	1.25E-30	0.59987093	0.954	0.757	2.73E-26	RNASE1	4	MGP_VEC
612	5.06E-34	0.598322816	0.181	0.019	1.10E-29	CYP1B1	4	MGP_VEC
613	7.60E-24	0.593518889	0.628	0.391	1.66E-19	GAS6	4	MGP_VEC
614	1.35E-26	0.591971895	0.727	0.476	2.95E-22	MMRN2	4	MGP_VEC
615	2.76E-45	0.590500164	0.535	0.15	6.03E-41	CD34	4	MGP_VEC
616	3.13E-25	0.590297786	0.706	0.452	6.83E-21	CDC42EP3	4	MGP_VEC
617	2.81E-27	0.589515933	0.989	0.903	6.13E-23	TM4SF1	4	MGP_VEC
618	6.08E-37	0.585039577	0.968	0.898	1.33E-32	IGFBP7	4	MGP_VEC
619	9.17E-08	0.583541507	0.557	0.439	0.00199879	C11orf96	4	MGP_VEC
620	8.24E-24	0.576297486	0.277	0.081	1.80E-19	LEPR	4	MGP_VEC
621	1.28E-17	0.574872961	0.709	0.542	2.80E-13	ZFP36L2	4	MGP_VEC
622	6.54E-36	0.564126078	0.28	0.051	1.43E-31	PTGS1	4	MGP_VEC
623	6.72E-16	0.563635469	0.443	0.236	1.46E-11	PLEC	4	MGP_VEC
624	3.12E-54	0.558043232	0.23	0.014	6.80E-50	CGNL1	4	MGP_VEC
625	9.99E-28	0.556905897	0.45	0.179	2.18E-23	SNCG	4	MGP_VEC
626	1.59E-19	0.554813292	0.656	0.423	3.46E-15	ATP1A1	4	MGP_VEC
627	2.65E-32	0.554638253	0.95	0.786	5.79E-28	CD9	4	MGP_VEC
628	5.75E-25	0.552823354	0.851	0.704	1.25E-20	TGM2	4	MGP_VEC

629	5.92E-38	0.547992281	0.27	0.045	1.29E-33	MYOF	4	MGP_VEC
630	3.22E-11	0.547103809	0.589	0.423	7.02E-07	PNP	4	MGP_VEC
631	1.79E-11	0.542104105	0.227	0.091	3.91E-07	CTGF	4	MGP_VEC
632	1.95E-31	0.531282761	0.355	0.102	4.25E-27	FBLIM1	4	MGP_VEC
633	3.30E-29	0.529659069	0.337	0.094	7.19E-25	ASRGL1	4	MGP_VEC
634	3.88E-13	0.52598969	0.823	0.73	8.46E-09	DDIT4	4	MGP_VEC
635	1.33E-26	0.525181551	0.592	0.304	2.91E-22	JAM2	4	MGP_VEC
636	4.98E-11	0.522376857	0.613	0.455	1.09E-06	MYADM	4	MGP_VEC
637	2.81E-44	0.514147688	0.145	0.003	6.13E-40	OTC	4	MGP_VEC
638	1.21E-12	0.513801983	0.397	0.227	2.64E-08	GADD45A	4	MGP_VEC
639	2.60E-36	0.51081479	0.184	0.018	5.66E-32	SGMS2	4	MGP_VEC
640	1.17E-20	0.510781754	0.539	0.298	2.56E-16	EMP2	4	MGP_VEC
641	4.36E-17	0.507726418	0.521	0.293	9.50E-13	NDRG1	4	MGP_VEC
642	1.06E-09	0.499431994	0.184	0.074	2.32E-05	BIRC3	4	MGP_VEC
643	4.37E-07	0.498810251	0.404	0.281	0.009519231	PKIG	4	MGP_VEC
644	1.62E-17	0.496864048	0.532	0.316	3.52E-13	CTNNAL1	4	MGP_VEC
645	6.38E-40	0.491461056	0.16	0.008	1.39E-35	ALDH1A2	4	MGP_VEC
646	2.67E-26	0.491142097	0.294	0.081	5.82E-22	DPYSL3	4	MGP_VEC
647	2.44E-08	0.490095736	0.472	0.344	0.000532835	MAT2A	4	MGP_VEC
648	4.82E-25	0.489130066	0.174	0.028	1.05E-20	PDLIM4	4	MGP_VEC
649	2.81E-26	0.486941521	0.801	0.557	6.12E-22	TAGLN2	4	MGP_VEC
650	1.46E-14	0.486134347	0.603	0.429	3.19E-10	YBX3	4	MGP_VEC
651	8.84E-31	0.485712675	0.248	0.048	1.93E-26	PLCXD3	4	MGP_VEC
652	1.17E-21	0.483413898	0.376	0.15	2.54E-17	BCR	4	MGP_VEC
653	7.76E-15	0.483196546	0.887	0.783	1.69E-10	ID3	4	MGP_VEC
654	3.45E-22	0.480576779	0.826	0.639	7.52E-18	HNRNPA1	4	MGP_VEC
655	1.66E-19	0.480221204	0.801	0.611	3.63E-15	RAMP2	4	MGP_VEC
656	1.01E-14	0.475888671	0.94	0.844	2.20E-10	ID1	4	MGP_VEC
657	8.71E-11	0.47443666	0.553	0.401	1.90E-06	ITGA5	4	MGP_VEC
658	7.54E-12	0.471222678	0.603	0.477	1.64E-07	TUBB4B	4	MGP_VEC
659	4.61E-19	0.470628793	0.865	0.741	1.01E-14	ARGLU1	4	MGP_VEC
660	1.33E-15	0.466942104	0.574	0.358	2.89E-11	LRRC32	4	MGP_VEC
661	7.07E-17	0.466026316	0.691	0.456	1.54E-12	GJA4	4	MGP_VEC
662	6.06E-15	0.465366204	0.348	0.16	1.32E-10	FLRT2	4	MGP_VEC
663	2.97E-34	0.461586772	0.301	0.065	6.47E-30	DKK3	4	MGP_VEC
664	1.24E-27	0.458010167	0.337	0.094	2.71E-23	IGFBP2	4	MGP_VEC
665	4.62E-15	0.4513295	0.429	0.23	1.01E-10	MECOM	4	MGP_VEC
666	1.52E-08	0.449470715	0.652	0.545	0.000332071	NR4A1	4	MGP_VEC
667	5.73E-07	0.446877914	0.45	0.333	0.012488869	SERPINB9	4	MGP_VEC
668	4.47E-09	0.44547162	0.461	0.334	9.74E-05	EVA1C	4	MGP_VEC
669	1.40E-26	0.441166394	0.316	0.091	3.05E-22	ABCG2	4	MGP_VEC
670	8.34E-15	0.43603251	0.312	0.137	1.82E-10	FOXC1	4	MGP_VEC
671	4.08E-07	0.434356845	0.511	0.378	0.008895864	ADAMTS4	4	MGP_VEC
672	4.42E-13	0.429387777	0.688	0.521	9.63E-09	CALM2	4	MGP_VEC
673	4.85E-10	0.421423508	0.709	0.626	1.06E-05	SRSF7	4	MGP_VEC
674	1.15E-27	0.414649549	0.309	0.083	2.51E-23	ABLIM1	4	MGP_VEC
675	1.35E-15	0.4124475	0.33	0.148	2.95E-11	RP11-382A20.3	4	MGP_VEC
676	1.99E-09	0.411277941	0.422	0.278	4.34E-05	TUBA1A	4	MGP_VEC
677	1.44E-12	0.408416151	0.248	0.104	3.14E-08	NFATC1	4	MGP_VEC
678	2.79E-45	0.407772415	0.156	0.004	6.09E-41	ABI3BP	4	MGP_VEC
679	4.07E-10	0.407671418	0.379	0.229	8.88E-06	ITPR1	4	MGP_VEC
680	1.46E-20	0.407486601	0.918	0.798	3.19E-16	SRGN	4	MGP_VEC
681	1.55E-18	0.407482212	0.472	0.231	3.38E-14	FAM107A	4	MGP_VEC
682	8.64E-22	0.405128546	0.309	0.098	1.88E-17	SYNPO	4	MGP_VEC
683	5.32E-19	0.404629909	0.578	0.339	1.16E-14	STMN1	4	MGP_VEC
684	2.70E-19	0.402491617	0.801	0.612	5.89E-15	CAV1	4	MGP_VEC
685	3.14E-11	0.399702297	0.493	0.338	6.84E-07	TPRKB	4	MGP_VEC
686	7.85E-26	0.396902908	0.355	0.106	1.71E-21	PALMD	4	MGP_VEC
687	2.63E-11	0.392951994	0.628	0.497	5.74E-07	CYYR1	4	MGP_VEC
688	2.55E-16	0.391368462	0.394	0.196	5.56E-12	F11R	4	MGP_VEC
689	5.35E-09	0.386013867	0.55	0.419	0.000116776	MYH9	4	MGP_VEC
690	1.73E-10	0.383903678	0.319	0.168	3.76E-06	BTG2	4	MGP_VEC
691	8.94E-15	0.383690615	0.777	0.631	1.95E-10	PTMS	4	MGP_VEC
692	6.94E-08	0.383663171	0.787	0.687	0.001514002	HSPA1B	4	MGP_VEC
693	5.56E-11	0.38039623	0.876	0.803	1.21E-06	IER2	4	MGP_VEC
694	3.75E-22	0.378789297	0.245	0.063	8.18E-18	MMP28	4	MGP_VEC
695	1.22E-06	0.378703894	0.429	0.32	0.026648255	ADGRG1	4	MGP_VEC
696	1.02E-16	0.378039469	0.216	0.066	2.22E-12	NFATC2	4	MGP_VEC
697	1.69E-12	0.376968128	0.493	0.314	3.69E-08	ALDH2	4	MGP_VEC
698	1.87E-08	0.374030602	0.897	0.838	0.000407137	JUN	4	MGP_VEC

699	3.59E-20	0.373449407	0.22	0.057	7.83E-16	SLC39A14	4	MGP_VEC
700	9.39E-27	0.371740895	0.209	0.039	2.05E-22	ATP1B2	4	MGP_VEC
701	2.13E-08	0.370869103	0.649	0.536	0.000464355	JUND	4	MGP_VEC
702	4.21E-44	0.365181552	0.188	0.011	9.19E-40	AR	4	MGP_VEC
703	1.64E-16	0.363980634	0.273	0.1	3.58E-12	HSPA2	4	MGP_VEC
704	1.98E-10	0.363652936	0.908	0.84	4.33E-06	JUNB	4	MGP_VEC
705	5.32E-11	0.362606575	0.291	0.138	1.16E-06	FLNA	4	MGP_VEC
706	1.33E-13	0.36186268	0.858	0.729	2.90E-09	LAPTM4A	4	MGP_VEC
707	1.18E-11	0.357920359	0.738	0.631	2.56E-07	C12orf57	4	MGP_VEC
708	1.77E-08	0.357063158	0.323	0.191	0.000385197	NR4A2	4	MGP_VEC
709	1.15E-15	0.355234083	0.138	0.03	2.50E-11	CLCF1	4	MGP_VEC
710	7.24E-09	0.354363824	0.348	0.208	0.000157792	GATA2	4	MGP_VEC
711	7.14E-07	0.352168346	0.67	0.58	0.015568919	JMJD1C	4	MGP_VEC
712	9.43E-16	0.350019097	0.216	0.068	2.06E-11	IGFBP6	4	MGP_VEC
713	9.45E-33	0.348975589	0.174	0.018	2.06E-28	GJA5	4	MGP_VEC
714	4.60E-11	0.347829545	0.589	0.413	1.00E-06	EMP3	4	MGP_VEC
715	5.48E-07	0.34768617	0.291	0.181	0.011945461	GMDS	4	MGP_VEC
716	2.72E-07	0.347143884	0.578	0.459	0.005929962	MDK	4	MGP_VEC
717	2.22E-07	0.347048797	0.351	0.223	0.004847921	MAFB	4	MGP_VEC
718	3.56E-12	0.346677696	0.872	0.771	7.76E-08	GNG5	4	MGP_VEC
719	2.51E-15	0.34558811	0.191	0.057	5.47E-11	SMIM3	4	MGP_VEC
720	1.06E-18	0.344113265	0.826	0.688	2.31E-14	PECAM1	4	MGP_VEC
721	3.68E-15	0.343244269	0.333	0.148	8.02E-11	JAG1	4	MGP_VEC
722	4.61E-11	0.342265994	0.266	0.123	1.01E-06	KLRG1	4	MGP_VEC
723	1.28E-23	0.34114668	0.858	0.644	2.80E-19	CRIP2	4	MGP_VEC
724	1.45E-07	0.341063854	0.33	0.211	0.003171828	PLP2	4	MGP_VEC
725	2.00E-13	0.340490944	0.816	0.691	4.35E-09	S100A11	4	MGP_VEC
726	1.88E-08	0.339704525	0.585	0.452	0.000409818	AHNAK	4	MGP_VEC
727	1.28E-11	0.336876761	0.777	0.657	2.78E-07	HMGN3	4	MGP_VEC
728	1.04E-16	0.336675335	0.287	0.108	2.26E-12	OCIAD2	4	MGP_VEC
729	6.24E-08	0.33436661	0.897	0.827	0.001359982	NFKBIA	4	MGP_VEC
730	4.62E-10	0.333659355	0.319	0.166	1.01E-05	MALL	4	MGP_VEC
731	1.65E-13	0.33176366	0.869	0.755	3.60E-09	ANXA2	4	MGP_VEC
732	2.91E-16	0.330745124	0.947	0.894	6.35E-12	UBC	4	MGP_VEC
733	4.56E-08	0.330437562	0.404	0.265	0.000995185	NAA10	4	MGP_VEC
734	1.44E-11	0.330381457	0.833	0.713	3.15E-07	PRSS23	4	MGP_VEC
735	7.21E-08	0.329627438	0.333	0.197	0.001572619	HEG1	4	MGP_VEC
736	9.15E-23	0.327889372	0.206	0.044	2.00E-18	PTGIS	4	MGP_VEC
737	4.99E-09	0.325438213	0.277	0.148	0.00010876	SGCE	4	MGP_VEC
738	1.51E-08	0.320237546	0.716	0.609	0.00032981	TUBA1B	4	MGP_VEC
739	2.07E-08	0.320098748	0.149	0.057	0.000450433	VEGFA	4	MGP_VEC
740	1.23E-06	0.320036487	0.227	0.127	0.026786158	FNIP2	4	MGP_VEC
741	6.88E-20	0.319919869	0.975	0.944	1.50E-15	IFITM2	4	MGP_VEC
742	3.03E-10	0.319207183	0.255	0.117	6.61E-06	SULF2	4	MGP_VEC
743	5.04E-12	0.318799371	0.734	0.576	1.10E-07	EMCN	4	MGP_VEC
744	3.47E-09	0.318632221	0.989	0.936	7.56E-05	MT-ND3	4	MGP_VEC
745	5.70E-12	0.317326538	0.894	0.798	1.24E-07	CLEC3B	4	MGP_VEC
746	2.95E-09	0.316307856	0.688	0.586	6.44E-05	HNRNPDL	4	MGP_VEC
747	8.26E-10	0.313262314	0.291	0.152	1.80E-05	ANKRD37	4	MGP_VEC
748	2.01E-09	0.311989494	0.355	0.21	4.38E-05	TMEM14A	4	MGP_VEC
749	3.01E-11	0.31190987	0.22	0.092	6.56E-07	RNASE4	4	MGP_VEC
750	6.55E-12	0.310514412	0.312	0.152	1.43E-07	GSTM3	4	MGP_VEC
751	2.64E-14	0.309206709	0.897	0.784	5.75E-10	CALCRL	4	MGP_VEC
752	3.00E-35	0.309104858	0.142	0.007	6.53E-31	GATA5	4	MGP_VEC
753	1.63E-16	0.308997423	0.188	0.051	3.55E-12	ITGA10	4	MGP_VEC
754	6.47E-16	0.308757853	0.223	0.072	1.41E-11	HACD1	4	MGP_VEC
755	2.67E-32	0.30773014	0.17	0.017	5.81E-28	VIPR1	4	MGP_VEC
756	9.16E-08	0.307335705	0.723	0.647	0.001998593	ARL6IP1	4	MGP_VEC
757	2.21E-13	0.306954767	0.95	0.879	4.83E-09	HSPB1	4	MGP_VEC
758	2.09E-10	0.306136763	0.975	0.884	4.56E-06	MT-ND1	4	MGP_VEC
759	2.03E-09	0.305703751	0.362	0.21	4.44E-05	GPRC5C	4	MGP_VEC
760	1.58E-08	0.304881632	0.316	0.173	0.000344339	THBS1	4	MGP_VEC
761	1.19E-06	0.303125595	0.472	0.354	0.026017211	FOXP1	4	MGP_VEC
762	2.93E-07	0.301677162	0.124	0.046	0.00638967	SELE	4	MGP_VEC
763	1.69E-06	0.30139701	0.238	0.136	0.036760901	BRIX1	4	MGP_VEC
764	1.79E-12	0.300966586	0.904	0.811	3.91E-08	HSPG2	4	MGP_VEC
765	3.53E-07	0.300919795	0.202	0.102	0.007698072	INHBB	4	MGP_VEC
766	9.90E-10	0.300106395	0.993	0.928	2.16E-05	MT-ND4L	4	MGP_VEC
767	7.93E-10	0.299051516	0.986	0.923	1.73E-05	MT-CYB	4	MGP_VEC
768	1.59E-08	0.298970741	0.652	0.56	0.000346654	TSPO	4	MGP_VEC

769	6.58E-08	0.296802694	0.309	0.181	0.001434114	MKNK2	4	MGP_VEC
770	3.87E-12	0.295759521	0.184	0.063	8.44E-08	SVIL	4	MGP_VEC
771	2.07E-28	0.29548043	0.149	0.015	4.52E-24	DOK5	4	MGP_VEC
772	2.67E-13	0.295187683	0.255	0.103	5.81E-09	TUBA4A	4	MGP_VEC
773	9.28E-08	0.294617231	0.241	0.127	0.002024856	UGDH	4	MGP_VEC
774	4.50E-10	0.294406051	0.213	0.092	9.82E-06	SPSB1	4	MGP_VEC
775	2.97E-07	0.292529251	0.188	0.092	0.006471425	AHI1	4	MGP_VEC
776	3.01E-07	0.291780349	0.362	0.224	0.006557785	CD24	4	MGP_VEC
777	5.90E-08	0.290562674	0.17	0.072	0.00128759	NNMT	4	MGP_VEC
778	2.03E-06	0.289331454	0.152	0.071	0.044170824	WNK3	4	MGP_VEC
779	3.14E-09	0.286603975	0.507	0.34	6.85E-05	LRRFIP1	4	MGP_VEC
780	3.99E-30	0.285136523	0.993	0.989	8.71E-26	RPL34	4	MGP_VEC
781	3.77E-10	0.283115602	0.163	0.059	8.21E-06	CDA	4	MGP_VEC
782	6.27E-07	0.282120942	0.709	0.617	0.013669973	EFNA1	4	MGP_VEC
783	2.10E-16	0.280430513	0.16	0.037	4.58E-12	TMOD1	4	MGP_VEC
784	8.87E-11	0.280290663	0.574	0.399	1.93E-06	BCAM	4	MGP_VEC
785	2.32E-16	0.279949999	0.982	0.965	5.07E-12	RPS6	4	MGP_VEC
786	1.12E-08	0.279573824	0.426	0.278	0.000244743	METTL7A	4	MGP_VEC
787	6.48E-08	0.277001321	0.691	0.581	0.001412266	SNHG8	4	MGP_VEC
788	1.90E-11	0.274234455	0.113	0.028	4.14E-07	BMP4	4	MGP_VEC
789	7.02E-23	0.27417988	0.11	0.009	1.53E-18	KCNIP4	4	MGP_VEC
790	3.99E-12	0.273467929	0.252	0.101	8.70E-08	HS3ST1	4	MGP_VEC
791	9.20E-07	0.273283234	0.699	0.6	0.020063149	TXN	4	MGP_VEC
792	1.71E-08	0.272580502	0.943	0.871	0.000371881	DUSP1	4	MGP_VEC
793	4.67E-10	0.271813953	0.947	0.843	1.02E-05	HSP90AB1	4	MGP_VEC
794	1.65E-11	0.271005156	0.128	0.034	3.59E-07	LYST	4	MGP_VEC
795	5.01E-14	0.270817181	0.996	0.893	1.09E-09	MALAT1	4	MGP_VEC
796	2.64E-07	0.270691735	0.241	0.132	0.005759427	RIMKLB	4	MGP_VEC
797	3.27E-19	0.270104766	0.106	0.012	7.13E-15	MEDAG	4	MGP_VEC
798	4.12E-07	0.269970266	0.266	0.154	0.008983497	FRY	4	MGP_VEC
799	4.93E-08	0.269765402	0.195	0.089	0.001074803	DUSP4	4	MGP_VEC
800	1.59E-06	0.268884862	0.493	0.375	0.034772332	CAV2	4	MGP_VEC
801	4.44E-29	0.267796369	0.131	0.009	9.68E-25	SSTR1	4	MGP_VEC
802	9.52E-09	0.267080164	0.954	0.875	0.000207512	MT-ND5	4	MGP_VEC
803	4.56E-09	0.264969378	0.667	0.524	9.95E-05	ECSCR.1	4	MGP_VEC
804	1.52E-07	0.264952672	0.415	0.276	0.003325604	COX17	4	MGP_VEC
805	1.15E-12	0.264062384	0.191	0.065	2.51E-08	C16orf45	4	MGP_VEC
806	1.96E-07	0.262508096	0.206	0.102	0.004277504	ARC	4	MGP_VEC
807	1.77E-17	0.261280744	0.848	0.689	3.85E-13	CD74	4	MGP_VEC
808	1.45E-06	0.261070602	0.486	0.377	0.03159772	CD40	4	MGP_VEC
809	2.36E-26	0.260829334	0.996	0.991	5.15E-22	AC090498.1	4	MGP_VEC
810	8.82E-14	0.260544319	0.142	0.034	1.92E-09	SELP	4	MGP_VEC
811	2.53E-07	0.258988914	0.39	0.255	0.005509474	PAPSS2	4	MGP_VEC
812	1.02E-19	0.258337638	0.128	0.018	2.23E-15	SYBU	4	MGP_VEC
813	6.28E-08	0.257794206	0.755	0.653	0.001368962	TPM3	4	MGP_VEC
814	4.16E-07	0.255762168	0.262	0.152	0.009075047	MIR99AHG	4	MGP_VEC
815	3.74E-07	0.254851701	0.174	0.082	0.008158692	ENPP2	4	MGP_VEC
816	7.74E-08	0.253353463	0.131	0.049	0.001688359	GPC1	4	MGP_VEC
817	2.01E-22	0.251168773	0.106	0.009	4.38E-18	ANKRD29	4	MGP_VEC
818	3.81E-07	-0.250168122	0.181	0.344	0.008303364	PLOD1	4	MGP_VEC
819	1.32E-08	-0.253010528	0.855	0.889	0.000287472	IGFBP4	4	MGP_VEC
820	8.34E-07	-0.255407363	0.167	0.319	0.018195176	CNDP2	4	MGP_VEC
821	1.37E-07	-0.256993174	0.103	0.251	0.002981749	RASAL2	4	MGP_VEC
822	1.36E-06	-0.260780168	0.163	0.311	0.029693818	ZNF160	4	MGP_VEC
823	1.35E-10	-0.263472867	0.947	0.962	2.94E-06	MYL6	4	MGP_VEC
824	1.57E-07	-0.26884374	0.11	0.259	0.003433486	CD68	4	MGP_VEC
825	2.27E-06	-0.270332	0.337	0.483	0.049581311	LIMCH1	4	MGP_VEC
826	7.92E-07	-0.270578454	0.23	0.398	0.017273835	PCDH17	4	MGP_VEC
827	1.53E-08	-0.274154957	0.475	0.656	0.00033447	SERPINH1	4	MGP_VEC
828	6.11E-07	-0.275045713	0.121	0.261	0.013330169	ST3GAL4	4	MGP_VEC
829	1.54E-07	-0.277868915	0.124	0.278	0.003364586	WWP1	4	MGP_VEC
830	1.63E-07	-0.278416084	0.738	0.784	0.003552446	CCNI	4	MGP_VEC
831	2.82E-07	-0.278563511	0.117	0.265	0.006155629	TRIB2	4	MGP_VEC
832	6.75E-07	-0.278979513	0.138	0.287	0.014714337	MXD4	4	MGP_VEC
833	1.07E-06	-0.279981308	0.209	0.369	0.023303689	TMEM140	4	MGP_VEC
834	5.81E-08	-0.280221021	0.181	0.36	0.0012663	ABCG1	4	MGP_VEC
835	7.71E-07	-0.280840893	0.149	0.299	0.01680821 7	RCBTB2	4	MGP_VEC
836	3.93E-07	-0.282281733	0.351	0.52	0.008570944	APH1A	4	MGP_VEC
837	5.16E-07	-0.285317081	0.291	0.458	0.011262831	ICA1	4	MGP_VEC
838	5.57E-07	-0.285488411	0.216	0.38	0.012149958	TLR4	4	MGP_VEC

839	4.32E-07	-0.2870781	0.27	0.453	0.009417056	CD302	4	MGP_VEC
840	1.31E-07	-0.287695135	0.145	0.307	0.0028517	CHCHD10	4	MGP_VEC
841	3.89E-07	-0.287706992	0.23	0.393	0.008481245	BLCAP	4	MGP_VEC
842	3.69E-12	-0.287938423	0.989	0.989	8.04E-08	TMSB4X	4	MGP_VEC
843	1.93E-08	-0.288855576	0.621	0.776	0.000421826	SEC14L1	4	MGP_VEC
844	4.34E-08	-0.290944368	0.227	0.411	0.000946655	DPP7	4	MGP_VEC
845	2.47E-08	-0.291920807	0.103	0.258	0.000538739	SNX18	4	MGP_VEC
846	1.03E-06	-0.293336463	0.323	0.474	0.022358768	ROBO4	4	MGP_VEC
847	1.72E-09	-0.295825891	0.383	0.584	3.75E-05	TFPI2	4	MGP_VEC
848	1.35E-09	-0.296296031	0.145	0.329	2.95E-05	HEXA	4	MGP_VEC
849	3.71E-07	-0.297845585	0.167	0.319	0.008101181	CMTM8	4	MGP_VEC
850	1.63E-06	-0.300075518	0.433	0.563	0.035589803	LAMTOR1	4	MGP_VEC
851	9.57E-07	-0.301247472	0.66	0.725	0.020876476	TMEM123	4	MGP_VEC
852	1.10E-08	-0.302274689	0.234	0.429	0.000240792	GTF2I	4	MGP_VEC
853	5.43E-07	-0.302483426	0.291	0.451	0.011839483	CSF1	4	MGP_VEC
854	1.75E-08	-0.302761702	0.408	0.569	0.000381487	TMEM50B	4	MGP_VEC
855	8.02E-07	-0.304352138	0.344	0.497	0.017491819	VAT1	4	MGP_VEC
856	2.79E-11	-0.307804058	0.879	0.912	6.07E-07	MYL12A	4	MGP_VEC
857	5.42E-07	-0.308921151	0.454	0.597	0.011814257	BSG	4	MGP_VEC
858	9.81E-08	-0.315938024	0.638	0.727	0.002138746	RAB11A	4	MGP_VEC
859	9.39E-09	-0.316828212	0.202	0.386	0.000204788	TBC1D15	4	MGP_VEC
860	9.85E-08	-0.316982216	0.443	0.593	0.00214823	CUTA	4	MGP_VEC
861	8.19E-10	-0.318856586	0.106	0.284	1.79E-05	DERA	4	MGP_VEC
862	1.60E-09	-0.31899195	0.209	0.408	3.49E-05	KIAA1033	4	MGP_VEC
863	3.08E-07	-0.319559137	0.16	0.31	0.006722139	PELO	4	MGP_VEC
864	1.86E-08	-0.319764144	0.113	0.274	0.00040651	ERRFI1	4	MGP_VEC
865	1.08E-09	-0.320258439	0.411	0.597	2.35E-05	CD164	4	MGP_VEC
866	8.49E-10	-0.322949525	0.156	0.344	1.85E-05	SCPEP1	4	MGP_VEC
867	3.64E-10	-0.32354389	0.372	0.57	7.94E-06	FKBP5	4	MGP_VEC
868	6.11E-07	-0.324953094	0.199	0.352	0.013335302	GIMAP2	4	MGP_VEC
869	6.46E-07	-0.325050215	0.383	0.527	0.014094141	NPY1R	4	MGP_VEC
870	1.13E-08	-0.325312061	0.316	0.508	0.000247495	LAMP2	4	MGP_VEC
871	4.27E-09	-0.325712341	0.131	0.309	9.31E-05	NISCH	4	MGP_VEC
872	3.60E-08	-0.329518814	0.28	0.455	0.00078592	PDIA4	4	MGP_VEC
873	9.51E-10	-0.330771542	0.539	0.691	2.07E-05	KDELR1	4	MGP_VEC
874	2.48E-09	-0.330818855	0.216	0.407	5.40E-05	ALDH6A1	4	MGP_VEC
875	1.57E-06	-0.332002347	0.457	0.617	0.034143223	ATOX1	4	MGP_VEC
876	3.15E-09	-0.333319723	0.188	0.373	6.88E-05	CNST	4	MGP_VEC
877	8.25E-08	-0.334406122	0.472	0.619	0.001800173	TCN2	4	MGP_VEC
878	2.93E-09	-0.335961207	0.702	0.809	6.39E-05	CNN3	4	MGP_VEC
879	2.75E-08	-0.337001499	0.358	0.522	0.000598799	NPR1	4	MGP_VEC
880	1.17E-07	-0.33738137	0.234	0.405	0.002541527	HECW2	4	MGP_VEC
881	1.18E-08	-0.339318689	0.27	0.46	0.000257853	C10orf11	4	MGP_VEC
882	1.10E-07	-0.339412357	0.199	0.361	0.002395315	TMEM88	4	MGP_VEC
883	6.03E-10	-0.343358749	0.17	0.367	1.31E-05	GUSB	4	MGP_VEC
884	1.67E-06	-0.344945827	0.156	0.301	0.036507355	IL32	4	MGP_VEC
885	9.93E-08	-0.345330522	0.177	0.338	0.002166251	GNG12	4	MGP_VEC
886	8.62E-10	-0.345824269	0.326	0.537	1.88E-05	FZD4	4	MGP_VEC
887	7.96E-09	-0.346183073	0.404	0.573	0.000173565	SAT2	4	MGP_VEC
888	1.91E-10	-0.346373034	0.152	0.356	4.16E-06	TMEM256-PLSCR3	4	MGP_VEC
889	4.47E-11	-0.346765504	0.195	0.412	9.74E-07	YPEL5	4	MGP_VEC
890	4.95E-10	-0.347860238	0.11	0.286	1.08E-05	CHID1	4	MGP_VEC
891	1.05E-09	-0.34837184	0.443	0.627	2.29E-05	MYDGF	4	MGP_VEC
892	1.29E-09	-0.349404088	0.181	0.37	2.80E-05	EPS15	4	MGP_VEC
893	1.09E-08	-0.352906081	0.557	0.701	0.000237978	CYBA	4	MGP_VEC
894	2.10E-09	-0.354095672	0.17	0.356	4.58E-05	SETX	4	MGP_VEC
895	1.03E-09	-0.356747737	0.422	0.611	2.25E-05	SNX6	4	MGP_VEC
896	3.68E-09	-0.356899546	0.316	0.501	8.02E-05	RRBP1	4	MGP_VEC
897	1.94E-09	-0.359539847	0.301	0.493	4.23E-05	TRAPPC5	4	MGP_VEC
898	8.43E-08	-0.359631397	0.238	0.406	0.001838498	PTPN1	4	MGP_VEC
899	1.08E-09	-0.364216116	0.585	0.748	2.35E-05	VAMP5	4	MGP_VEC
900	1.38E-06	-0.36474402	0.372	0.509	0.030040808	PDCD4	4	MGP_VEC
901	5.36E-11	-0.365402212	0.287	0.497	1.17E-06	HEXB	4	MGP_VEC
902	1.86E-09	-0.365588719	0.181	0.362	4.05E-05	GNS	4	MGP_VEC
903	3.23E-13	-0.368107447	0.837	0.879	7.04E-09	GABARAP	4	MGP_VEC
904	8.02E-10	-0.369538252	0.191	0.376	1.75E-05	ARHGEF7	4	MGP_VEC
905	1.69E-09	-0.370504888	0.723	0.801	3.69E-05	POLR2L	4	MGP_VEC
906	4.66E-12	-0.371600963	0.16	0.382	1.02E-07	CBLB	4	MGP_VEC
907	5.20E-10	-0.371847041	0.277	0.483	1.13E-05	FCHSD2	4	MGP_VEC
908	1.65E-15	-0.374396882	0.83	0.907	3.60E-11	ATP5G2	4	MGP_VEC

909	1.16E-08	-0.376831482	0.259	0.43	0.000253057	AKR1B1	4	MGP_VEC
910	1.85E-09	-0.380342922	0.223	0.416	4.03E-05	NUDT16	4	MGP_VEC
911	1.05E-10	-0.380508064	0.603	0.723	2.29E-06	S100A16	4	MGP_VEC
912	1.21E-12	-0.381465742	0.348	0.582	2.65E-08	HPCAL1	4	MGP_VEC
913	8.15E-12	-0.382282274	0.56	0.738	1.78E-07	ARHGDIB	4	MGP_VEC
914	1.12E-11	-0.383254083	0.188	0.41	2.45E-07	SLC35A1	4	MGP_VEC
915	1.43E-11	-0.383607944	0.145	0.352	3.12E-07	CMTM3	4	MGP_VEC
916	6.99E-09	-0.384635367	0.358	0.539	0.000152389	YES1	4	MGP_VEC
917	8.39E-09	-0.387988052	0.177	0.35	0.000183014	TMEM106C	4	MGP_VEC
918	6.46E-12	-0.388140554	0.177	0.396	1.41E-07	GLMP	4	MGP_VEC
919	5.50E-13	-0.389996823	0.73	0.79	1.20E-08	SARAF	4	MGP_VEC
920	1.08E-12	-0.393676974	0.117	0.331	2.35E-08	SORBS1	4	MGP_VEC
921	6.60E-12	-0.394397795	0.266	0.501	1.44E-07	PHACTR2	4	MGP_VEC
922	4.25E-13	-0.394988708	0.681	0.772	9.26E-09	PDIA3	4	MGP_VEC
923	4.02E-15	-0.394991792	0.872	0.889	8.76E-11	HSP90B1	4	MGP_VEC
924	1.31E-13	-0.397718805	0.745	0.829	2.85E-09	SDPR	4	MGP_VEC
925	9.30E-12	-0.401160808	0.195	0.41	2.03E-07	WASL	4	MGP_VEC
926	3.33E-10	-0.401416971	0.355	0.562	7.26E-06	UGCG	4	MGP_VEC
927	8.35E-10	-0.402018179	0.277	0.46	1.82E-05	CARHSP1	4	MGP_VEC
928	6.71E-08	-0.402292223	0.681	0.775	0.001463836	CLDN5	4	MGP_VEC
929	3.36E-11	-0.406873551	0.369	0.565	7.32E-07	LRRFIP2	4	MGP_VEC
930	1.28E-10	-0.408119319	0.567	0.713	2.80E-06	RDX	4	MGP_VEC
931	3.58E-09	-0.410211774	0.493	0.629	7.81E-05	SLC40A1	4	MGP_VEC
932	1.37E-10	-0.413455732	0.394	0.564	2.99E-06	GRINA	4	MGP_VEC
933	6.10E-14	-0.414796247	0.606	0.761	1.33E-09	GSTO1	4	MGP_VEC
934	1.19E-10	-0.415220341	0.241	0.438	2.59E-06	FHL1	4	MGP_VEC
935	7.78E-10	-0.41657058	0.362	0.551	1.70E-05	LEPROTL1	4	MGP_VEC
936	1.14E-11	-0.4169614	0.206	0.421	2.48E-07	TSPAN4	4	MGP_VEC
937	1.31E-10	-0.41789429	0.287	0.487	2.86E-06	OSTF1	4	MGP_VEC
938	6.13E-12	-0.417895049	0.128	0.336	1.34E-07	SLC27A3	4	MGP_VEC
939	1.10E-13	-0.419649804	0.22	0.458	2.40E-09	TNFRSF14	4	MGP_VEC
940	1.68E-12	-0.419799143	0.11	0.317	3.66E-08	SH3TC1	4	MGP_VEC
941	4.22E-13	-0.421449138	0.188	0.42	9.20E-09	CLTC	4	MGP_VEC
942	5.00E-13	-0.423005588	0.305	0.547	1.09E-08	DENND4C	4	MGP_VEC
943	9.81E-13	-0.429651869	0.326	0.555	2.14E-08	ADD3	4	MGP_VEC
944	4.39E-11	-0.435103881	0.316	0.535	9.57E-07	SC5D	4	MGP_VEC
945	2.71E-12	-0.437954995	0.17	0.384	5.91E-08	CRYL1	4	MGP_VEC
946	2.22E-06	-0.441737413	0.309	0.447	0.048364985	OLFM1	4	MGP_VEC
947	3.48E-12	-0.443283008	0.17	0.385	7.60E-08	TSN	4	MGP_VEC
948	3.61E-08	-0.449131421	0.191	0.358	0.000787214	PHLDA1	4	MGP_VEC
949	2.35E-21	-0.452169456	0.826	0.869	5.12E-17	FCGRT	4	MGP_VEC
950	4.65E-10	-0.456021616	0.684	0.782	1.02E-05	LIFR	4	MGP_VEC
951	1.83E-17	-0.45617669	0.752	0.82	3.98E-13	PSAP	4	MGP_VEC
952	3.23E-13	-0.458493656	0.376	0.573	7.05E-09	CALU	4	MGP_VEC
953	3.39E-13	-0.459374077	0.124	0.341	7.38E-09	EIF4EBP1	4	MGP_VEC
954	2.13E-12	-0.461586317	0.28	0.507	4.64E-08	TMEM2	4	MGP_VEC
955	4.47E-15	-0.465800211	0.397	0.626	9.74E-11	PDE2A	4	MGP_VEC
956	1.09E-15	-0.466634104	0.199	0.462	2.38E-11	NECAP2	4	MGP_VEC
957	2.91E-13	-0.46805739	0.103	0.324	6.34E-09	RENBP	4	MGP_VEC
958	1.56E-25	-0.473228476	0.879	0.912	3.41E-21	PPIB	4	MGP_VEC
959	3.08E-12	-0.477613899	0.277	0.489	6.71E-08	TSPAN6	4	MGP_VEC
960	2.51E-29	-0.480619221	0.865	0.912	5.48E-25	APP	4	MGP_VEC
961	1.37E-14	-0.482888837	0.209	0.452	2.98E-10	EPOR	4	MGP_VEC
962	2.48E-17	-0.484210436	0.5	0.685	5.42E-13	LAMP1	4	MGP_VEC
963	3.97E-15	-0.492098229	0.486	0.669	8.65E-11	AP2M1	4	MGP_VEC
964	2.85E-12	-0.500379269	0.28	0.518	6.21E-08	MEG3	4	MGP_VEC
965	1.73E-12	-0.501773658	0.394	0.611	3.77E-08	NUPR1	4	MGP_VEC
966	4.75E-16	-0.50178421	0.152	0.404	1.04E-11	RAB20	4	MGP_VEC
967	8.27E-18	-0.502204826	0.156	0.423	1.80E-13	DRAM2	4	MGP_VEC
968	1.68E-20	-0.503200186	0.738	0.843	3.66E-16	NDUFA4	4	MGP_VEC
969	4.71E-26	-0.50362508	0.805	0.862	1.03E-21	NPC2	4	MGP_VEC
970	1.13E-13	-0.509816518	0.163	0.402	2.46E-09	LRG1	4	MGP_VEC
971	1.49E-15	-0.510181114	0.443	0.666	3.26E-11	NTN4	4	MGP_VEC
972	1.14E-17	-0.510265297	0.379	0.635	2.50E-13	ARHGAP29	4	MGP_VEC
973	2.45E-15	-0.512894059	0.195	0.454	5.34E-11	HIPK2	4	MGP_VEC
974	1.88E-15	-0.513929788	0.213	0.479	4.10E-11	COL4A1	4	MGP_VEC
975	4.56E-18	-0.514809392	0.149	0.428	9.95E-14	MYO10	4	MGP_VEC
976	6.06E-14	-0.517028197	0.124	0.349	1.32E-09	FAM84A	4	MGP_VEC
977	7.21E-16	-0.535914186	0.319	0.555	1.57E-11	CCNG1	4	MGP_VEC
978	3.12E-19	-0.539609778	0.546	0.741	6.80E-15	TGFBR2	4	MGP_VEC

979	1.14E-15	-0.539930502	0.206	0.463	2.48E-11	MZT2A	4	MGP_VEC
980	2.96E-19	-0.550820413	0.613	0.756	6.45E-15	PDIA6	4	MGP_VEC
981	3.97E-20	-0.555353147	0.209	0.507	8.66E-16	COL4A2	4	MGP_VEC
982	1.88E-21	-0.556167044	0.582	0.753	4.10E-17	CTSB	4	MGP_VEC
983	2.38E-22	-0.559458302	0.613	0.795	5.19E-18	CLTA	4	MGP_VEC
984	4.91E-23	-0.575251164	0.631	0.827	1.07E-18	GPX4	4	MGP_VEC
985	2.90E-20	-0.580354863	0.358	0.638	6.31E-16	GPX1	4	MGP_VEC
986	1.32E-20	-0.585719576	0.45	0.673	2.87E-16	JAK1	4	MGP_VEC
987	6.13E-19	-0.588548056	0.191	0.492	1.34E-14	NR2F1	4	MGP_VEC
988	3.69E-19	-0.58855762	0.404	0.664	8.06E-15	ICAM2	4	MGP_VEC
989	6.05E-22	-0.589006186	0.557	0.748	1.32E-17	PPFIBP1	4	MGP_VEC
990	9.85E-20	-0.592221995	0.514	0.716	2.15E-15	LPAR6	4	MGP_VEC
991	2.83E-27	-0.592570456	0.84	0.904	6.16E-23	IL6ST	4	MGP_VEC
992	9.69E-18	-0.592761466	0.28	0.54	2.11E-13	NRP1	4	MGP_VEC
993	6.76E-21	-0.593361569	0.429	0.693	1.47E-16	GMFG	4	MGP_VEC
994	2.93E-19	-0.613578334	0.103	0.375	6.38E-15	CPM	4	MGP_VEC
995	1.84E-21	-0.625923726	0.241	0.526	4.00E-17	TPP1	4	MGP_VEC
996	2.89E-24	-0.63182597	0.465	0.73	6.29E-20	ADGRF5	4	MGP_VEC
997	4.59E-29	-0.6330423	0.582	0.796	1.00E-24	AP2S1	4	MGP_VEC
998	9.02E-26	-0.63411672	0.638	0.784	1.97E-21	PRCP	4	MGP_VEC
999	4.36E-27	-0.642945949	0.582	0.776	9.50E-23	GRN	4	MGP_VEC
1000	6.96E-21	-0.652149657	0.525	0.722	1.52E-16	ADGRL4	4	MGP_VEC
1001	4.27E-22	-0.655450833	0.234	0.535	9.32E-18	NRP2	4	MGP_VEC
1002	1.07E-51	-0.656290781	0.975	0.993	2.34E-47	FTL	4	MGP_VEC
1003	2.03E-20	-0.657679761	0.468	0.713	4.42E-16	PDK4	4	MGP_VEC
1004	1.07E-29	-0.667989399	0.745	0.854	2.34E-25	CALR	4	MGP_VEC
1005	8.41E-25	-0.668554519	0.422	0.693	1.83E-20	GLO1	4	MGP_VEC
1006	5.78E-25	-0.683796846	0.248	0.55	1.26E-20	SNX2	4	MGP_VEC
1007	3.18E-24	-0.685600427	0.535	0.726	6.93E-20	P4HB	4	MGP_VEC
1008	1.75E-23	-0.685923641	0.227	0.538	3.81E-19	DAB2	4	MGP_VEC
1009	1.36E-18	-0.686621637	0.355	0.629	2.96E-14	C8orf4	4	MGP_VEC
1010	4.87E-26	-0.687156655	0.113	0.453	1.06E-21	PXDN	4	MGP_VEC
1011	7.18E-27	-0.687904075	0.121	0.472	1.56E-22	GIMAP5	4	MGP_VEC
1012	1.49E-43	-0.690303666	0.929	0.933	3.24E-39	CCL14	4	MGP_VEC
1013	1.32E-26	-0.690674596	0.457	0.731	2.88E-22	CLEC2B	4	MGP_VEC
1014	3.00E-28	-0.704476998	0.848	0.904	6.53E-24	TIMP3	4	MGP_VEC
1015	1.03E-27	-0.70650084	0.613	0.797	2.26E-23	MARCKSL1	4	MGP_VEC
1016	4.14E-18	-0.710754576	0.358	0.6	9.02E-14	PLIN2	4	MGP_VEC
1017	4.90E-31	-0.717132295	0.557	0.799	1.07E-26	CSTB	4	MGP_VEC
1018	1.54E-22	-0.718137737	0.33	0.62	3.35E-18	CD36	4	MGP_VEC
1019	1.24E-26	-0.730676332	0.422	0.695	2.71E-22	LY96	4	MGP_VEC
1020	7.10E-28	-0.747108468	0.227	0.571	1.55E-23	SEMA6A	4	MGP_VEC
1021	5.28E-27	-0.782559495	0.348	0.636	1.15E-22	SNX5	4	MGP_VEC
1022	1.07E-32	-0.81350133	0.355	0.677	2.33E-28	CTSA	4	MGP_VEC
1023	1.92E-30	-0.817540001	0.216	0.577	4.19E-26	SMTN	4	MGP_VEC
1024	5.66E-32	-0.828231644	0.195	0.571	1.24E-27	TM4SF18	4	MGP_VEC
1025	5.23E-25	-0.856993745	0.436	0.692	1.14E-20	ADM	4	MGP_VEC
1026	1.02E-37	-0.866400112	0.323	0.677	2.23E-33	AP1S2	4	MGP_VEC
1027	7.72E-43	-0.88236029	0.72	0.879	1.68E-38	RAMP3	4	MGP_VEC
1028	4.68E-51	-0.884036168	0.752	0.913	1.02E-46	ENG	4	MGP_VEC
1029	1.21E-31	-0.887220525	0.472	0.72	2.65E-27	ASAH1	4	MGP_VEC
1030	9.99E-32	-0.900764546	0.223	0.575	2.18E-27	NID1	4	MGP_VEC
1031	3.00E-46	-0.922217711	0.642	0.852	6.53E-42	SPARC	4	MGP_VEC
1032	3.25E-40	-0.922220686	0.216	0.638	7.09E-36	KDR	4	MGP_VEC
1033	8.99E-49	-0.933154386	0.504	0.823	1.96E-44	GYPC	4	MGP_VEC
1034	1.11E-40	-0.99592244	0.557	0.822	2.42E-36	SGK1	4	MGP_VEC
1035	8.31E-46	-1.138605331	0.465	0.749	1.81E-41	MRC1	4	MGP_VEC
1036	1.74E-49	-1.186425795	0.255	0.673	3.79E-45	CXCL16	4	MGP_VEC
1037	6.88E-57	-1.257811219	0.191	0.681	1.50E-52	COTL1	4	MGP_VEC
1038	3.65E-60	-1.30577286	0.223	0.726	7.95E-56	MAF	4	MGP_VEC
1039	7.61E-41	-1.31125175	0.387	0.72	1.66E-36	STAB1	4	MGP_VEC
1040	7.25E-58	-1.348055099	0.528	0.84	1.58E-53	CRHBP	4	MGP_VEC
1041	4.40E-68	-1.494776411	0.574	0.869	9.59E-64	CTSD	4	MGP_VEC
1042	7.15E-41	-1.517084454	0.138	0.572	1.56E-36	CCL23	4	MGP_VEC
1043	1.04E-61	-1.571959123	0.227	0.711	2.26E-57	CD4	4	MGP_VEC
1044	3.20E-74	-1.626123109	0.723	0.912	6.99E-70	SEPP1	4	MGP_VEC
1045	2.43E-71	-1.74550079	0.45	0.822	5.31E-67	LGMN	4	MGP_VEC
1046	4.36E-69	-1.854348305	0.365	0.786	9.50E-65	ACP5	4	MGP_VEC
1047	1.99E-80	-2.188607076	0.113	0.736	4.34E-76	MS4A6A	4	MGP_VEC
1048	6.90E-85	-2.227164586	0.113	0.752	1.50E-80	OIT3	4	MGP_VEC

1049	8.21E-109	-2.481374546	0.309	0.913	1.79E-104	FCN3	4	MGP_VEC
1050	1.07E-113	-2.498267849	0.351	0.916	2.34E-109	DNASE1L3	4	MGP_VEC
1051	2.61E-100	-2.651590026	0.422	0.894	5.68E-96	CTSL	4	MGP_VEC
1052	3.06E-15	0.899295166	0.59	0.574	6.67E-11	NUPR1	5	NUPR1_LSEC
1053	5.01E-26	0.789573309	0.791	0.796	1.09E-21	GPX4	5	NUPR1_LSEC
1054	5.42E-22	0.763515934	0.736	0.764	1.18E-17	CSTB	5	NUPR1_LSEC
1055	9.31E-07	0.762795768	0.272	0.18	0.020312316	PVALB	5	NUPR1_LSEC
1056	4.46E-20	0.758885066	0.728	0.798	9.72E-16	POLR2L	5	NUPR1_LSEC
1057	2.37E-31	0.757900993	0.828	0.763	5.16E-27	GYPC	5	NUPR1_LSEC
1058	8.88E-23	0.734910128	0.778	0.766	1.94E-18	MARCKSL1	5	NUPR1_LSEC
1059	6.62E-77	0.72594599	1	0.989	1.44E-72	FTL	5	NUPR1_LSEC
1060	7.28E-10	0.725882563	0.556	0.599	1.59E-05	GPX1	5	NUPR1_LSEC
1061	1.34E-41	0.721088246	0.983	0.866	2.92E-37	SEPP1	5	NUPR1_LSEC
1062	2.65E-30	0.684145336	0.828	0.88	5.79E-26	GABARAP	5	NUPR1_LSEC
1063	7.53E-10	0.683621189	0.586	0.661	1.64E-05	GMFG	5	NUPR1_LSEC
1064	1.35E-10	0.681548478	0.628	0.649	2.95E-06	MAF	5	NUPR1_LSEC
1065	3.52E-19	0.674315834	0.724	0.768	7.69E-15	AP2S1	5	NUPR1_LSEC
1066	1.40E-15	0.669351966	0.674	0.73	3.06E-11	VAMP5	5	NUPR1_LSEC
1067	2.01E-30	0.658333639	0.946	0.606	4.39E-26	CLEC4G	5	NUPR1_LSEC
1068	1.66E-70	0.636068924	1	0.992	3.62E-66	RPL39	5	NUPR1_LSEC
1069	2.85E-13	0.629836188	0.632	0.598	6.22E-09	COTL1	5	NUPR1_LSEC
1070	2.71E-15	0.627636265	0.699	0.777	5.91E-11	CLTA	5	NUPR1_LSEC
1071	3.35E-28	0.610461398	0.874	0.898	7.31E-24	ATP5G2	5	NUPR1_LSEC
1072	2.88E-10	0.600272518	0.628	0.716	6.28E-06	S100A16	5	NUPR1_LSEC
1073	1.38E-38	0.600249408	0.983	0.976	3.01E-34	SERF2	5	NUPR1_LSEC
1074	8.42E-10	0.585615456	0.611	0.656	1.84E-05	GLO1	5	NUPR1_LSEC
1075	4.47E-09	0.579624408	0.628	0.756	9.75E-05	UQCR11	5	NUPR1_LSEC
1076	8.74E-17	0.574188925	0.854	0.697	1.91E-12	ACP5	5	NUPR1_LSEC
1077	9.55E-08	0.573139569	0.502	0.458	0.002082389	EHD3	5	NUPR1_LSEC
1078	4.54E-19	0.566138804	0.757	0.522	9.89E-15	CD14	5	NUPR1_LSEC
1079	4.54E-08	0.554889223	0.636	0.687	0.000989506	ASAH1	5	NUPR1_LSEC
1080	2.18E-12	0.554230696	0.757	0.851	4.75E-08	ALDOA	5	NUPR1_LSEC
1081	8.16E-11	0.540144527	0.711	0.838	1.78E-06	COX5B	5	NUPR1_LSEC
1082	4.30E-23	0.532623721	0.983	0.79	9.38E-19	FCN3	5	NUPR1_LSEC
1083	2.34E-15	0.52618022	0.791	0.585	5.10E-11	CLEC1B	5	NUPR1_LSEC
1084	2.11E-30	0.524614718	0.975	0.953	4.60E-26	UBA52	5	NUPR1_LSEC
1085	5.57E-07	0.506103987	0.686	0.809	0.012149463	CNN3	5	NUPR1_LSEC
1086	1.67E-06	0.505031569	0.586	0.61	0.036452441	CXCL16	5	NUPR1_LSEC
1087	3.27E-19	0.504397565	0.854	0.931	7.13E-15	ATP5E	5	NUPR1_LSEC
1088	2.08E-13	0.500721847	0.841	0.75	4.53E-09	LGMN	5	NUPR1_LSEC
1089	2.54E-09	0.49301316	0.711	0.844	5.54E-05	NDUFA4	5	NUPR1_LSEC
1090	3.24E-07	0.490713123	0.628	0.789	0.00707219	CHCHD2	5	NUPR1_LSEC
1091	6.51E-17	0.487945194	0.833	0.918	1.42E-12	MYL12A	5	NUPR1_LSEC
1092	3.91E-07	0.482357847	0.657	0.63	0.008522935	CD4	5	NUPR1_LSEC
1093	5.61E-07	0.475172271	0.623	0.754	0.012244272	GSTO1	5	NUPR1_LSEC
1094	1.07E-31	0.463519181	1	0.922	2.34E-27	CCL14	5	NUPR1_LSEC
1095	1.01E-10	0.460684532	0.72	0.843	2.19E-06	PRDX1	5	NUPR1_LSEC
1096	7.87E-13	0.452125863	0.82	0.911	1.72E-08	PFN1	5	NUPR1_LSEC
1097	8.35E-23	0.440732229	0.967	0.795	1.82E-18	CTSL	5	NUPR1_LSEC
1098	1.78E-11	0.433842687	0.883	0.813	3.88E-07	CTSD	5	NUPR1_LSEC
1099	9.08E-39	0.432500767	1	0.988	1.98E-34	RPS27	5	NUPR1_LSEC
1100	5.84E-14	0.431250967	0.816	0.608	1.27E-09	MS4A6A	5	NUPR1_LSEC
1101	7.05E-44	0.428880082	1	0.993	1.54E-39	RPL28	5	NUPR1_LSEC
1102	5.89E-54	0.428602637	1	0.993	1.29E-49	RPS28	5	NUPR1_LSEC
1103	5.90E-07	0.423388644	0.682	0.823	0.01285596	PPDPF	5	NUPR1_LSEC
1104	1.20E-11	0.415026023	0.753	0.859	2.61E-07	SDCBP	5	NUPR1_LSEC
1105	3.84E-11	0.413860365	0.866	0.915	8.38E-07	RPS29	5	NUPR1_LSEC
1106	1.23E-08	0.413743527	0.799	0.862	0.000268246	RAMP3	5	NUPR1_LSEC
1107	2.01E-32	0.412555089	1	0.984	4.38E-28	RPS13	5	NUPR1_LSEC
1108	1.63E-12	0.408753583	0.937	0.954	3.57E-08	ACTB	5	NUPR1_LSEC
1109	6.94E-07	0.408748336	0.707	0.852	0.015132827	OST4	5	NUPR1_LSEC
1110	7.01E-16	0.404144861	0.912	0.952	1.53E-11	RPL35	5	NUPR1_LSEC
1111	5.67E-37	0.396610704	0.996	0.991	1.24E-32	RPS12	5	NUPR1_LSEC
1112	1.73E-18	0.38910829	0.929	0.964	3.77E-14	MYL6	5	NUPR1_LSEC
1113	1.55E-06	0.388624883	0.686	0.846	0.033833127	MIF	5	NUPR1_LSEC
1114	6.01E-21	0.387260439	0.996	0.982	1.31E-16	RPL12	5	NUPR1_LSEC
1115	4.61E-25	0.384141928	0.992	0.988	1.00E-20	RPLP2	5	NUPR1_LSEC
1116	6.70E-09	0.383247784	0.774	0.889	0.000146066	MYL12B	5	NUPR1_LSEC
1117	7.44E-38	0.382767069	1	0.997	1.62E-33	RPLP1	5	NUPR1_LSEC
1118	3.64E-13	0.381971522	0.908	0.919	7.94E-09	RPS5	5	NUPR1_LSEC

1119	3.32E-21	0.378250502	0.983	0.974	7.24E-17	RPL18A	5	NUPR1_LSEC
1120	2.04E-12	0.373514723	0.9	0.944	4.45E-08	RPL9	5	NUPR1_LSEC
1121	4.90E-37	0.372923001	1	0.995	1.07E-32	EEF1A1	5	NUPR1_LSEC
1122	6.40E-11	0.369904987	0.808	0.909	1.40E-06	RPL7	5	NUPR1_LSEC
1123	1.89E-09	0.36710417	0.82	0.891	4.11E-05	RPS4Y1	5	NUPR1_LSEC
1124	3.09E-24	0.360303257	1	0.987	6.74E-20	RPL36	5	NUPR1_LSEC
1125	3.70E-15	0.358585895	0.933	0.963	8.08E-11	PPIA	5	NUPR1_LSEC
1126	6.84E-09	0.352367331	0.812	0.61	0.000149142	FCGR2B	5	NUPR1_LSEC
1127	1.03E-28	0.341774293	0.992	0.99	2.25E-24	RPL19	5	NUPR1_LSEC
1128	3.47E-12	0.341239955	0.958	0.649	7.57E-08	FCN2	5	NUPR1_LSEC
1129	4.05E-11	0.339180317	0.971	0.803	8.83E-07	DNASE1L3	5	NUPR1_LSEC
1130	5.07E-28	0.338913429	0.996	0.989	1.11E-23	RPL32	5	NUPR1_LSEC
1131	1.05E-10	0.335112453	0.904	0.937	2.29E-06	RPL21	5	NUPR1_LSEC
1132	3.36E-30	0.334384137	1	0.991	7.33E-26	RPL11	5	NUPR1_LSEC
1133	1.30E-23	0.333604531	0.996	0.986	2.83E-19	RPS15	5	NUPR1_LSEC
1134	2.05E-20	0.321719216	0.987	0.984	4.46E-16	RPL29	5	NUPR1_LSEC
1135	8.83E-14	0.321423705	0.937	0.963	1.93E-09	RPL24	5	NUPR1_LSEC
1136	3.70E-23	0.320763125	0.979	0.98	8.08E-19	RPL18	5	NUPR1_LSEC
1137	5.01E-09	0.320390957	0.891	0.942	0.00010922	S100A10	5	NUPR1_LSEC
1138	1.11E-21	0.319551027	0.992	0.988	2.42E-17	TMSB4X	5	NUPR1_LSEC
1139	3.09E-19	0.316906091	0.987	0.973	6.74E-15	RPS3A	5	NUPR1_LSEC
1140	4.25E-15	0.316039869	0.975	0.983	9.28E-11	RPS7	5	NUPR1_LSEC
1141	3.31E-23	0.311987993	1	0.991	7.23E-19	RPL13	5	NUPR1_LSEC
1142	3.71E-26	0.310377603	1	0.995	8.08E-22	RPL10	5	NUPR1_LSEC
1143	1.50E-18	0.308127838	0.996	0.983	3.27E-14	RPL35A	5	NUPR1_LSEC
1144	1.03E-22	0.306226912	0.996	0.989	2.25E-18	RPL37	5	NUPR1_LSEC
1145	1.17E-11	0.305507208	0.967	0.971	2.56E-07	RPL7A	5	NUPR1_LSEC
1146	5.79E-14	0.304387136	0.967	0.971	1.26E-09	RPL8	5	NUPR1_LSEC
1147	9.29E-15	0.304008267	0.979	0.982	2.03E-10	FAU	5	NUPR1_LSEC
1148	4.35E-13	0.302560236	0.937	0.976	9.49E-09	RPL22	5	NUPR1_LSEC
1149	6.46E-08	0.30045197	0.757	0.558	0.00140845	CLEC4M	5	NUPR1_LSEC
1150	1.28E-06	0.291603234	0.849	0.943	0.027999152	RPL38	5	NUPR1_LSEC
1151	8.83E-11	0.29105081	0.967	0.979	1.93E-06	GAPDH	5	NUPR1_LSEC
1152	5.47E-10	0.287566253	0.937	0.956	1.19E-05	RPS10	5	NUPR1_LSEC
1153	1.24E-07	0.285206669	0.745	0.887	0.00270943	SUMO2	5	NUPR1_LSEC
1154	1.11E-06	0.280691685	0.87	0.921	0.024302138	ACTG1	5	NUPR1_LSEC
1155	1.50E-13	0.278623817	0.971	0.988	3.27E-09	EIF1	5	NUPR1_LSEC
1156	3.62E-07	0.276962045	0.904	0.938	0.007891965	BTF3	5	NUPR1_LSEC
1157	6.08E-08	0.265523541	0.962	0.951	0.001325587	RPL17	5	NUPR1_LSEC
1158	1.59E-12	0.264866282	0.979	0.971	3.47E-08	RPL15	5	NUPR1_LSEC
1159	1.67E-09	0.263983171	0.941	0.963	3.64E-05	RPL3	5	NUPR1_LSEC
1160	5.84E-16	0.263097621	0.992	0.986	1.27E-11	RPS14	5	NUPR1_LSEC
1161	2.44E-16	0.262771206	1	0.987	5.33E-12	RPS15A	5	NUPR1_LSEC
1162	6.67E-13	0.261142267	0.992	0.986	1.45E-08	RPS24	5	NUPR1_LSEC
1163	1.51E-20	0.260941613	1	0.99	3.30E-16	AC090498.1	5	NUPR1_LSEC
1164	5.78E-11	0.260590072	0.967	0.977	1.26E-06	RPL37A	5	NUPR1_LSEC
1165	1.57E-17	0.254139008	0.996	0.989	3.43E-13	RPL34	5	NUPR1_LSEC
1166	1.96E-12	-0.250118802	0.167	0.479	4.28E-08	COL4A1	5	NUPR1_LSEC
1167	1.47E-09	-0.250577725	0.686	0.939	3.22E-05	APP	5	NUPR1_LSEC
1168	5.00E-12	-0.250787643	0.389	0.808	1.09E-07	SEC14L1	5	NUPR1_LSEC
1169	2.46E-17	-0.251047011	0.322	0.769	5.35E-13	CDKN1A	5	NUPR1_LSEC
1170	3.63E-16	-0.252813303	0.109	0.436	7.92E-12	KLF10	5	NUPR1_LSEC
1171	2.65E-12	-0.253535395	0.213	0.545	5.79E-08	SC5D	5	NUPR1_LSEC
1172	1.08E-15	-0.253843615	0.28	0.728	2.36E-11	PLSCR1	5	NUPR1_LSEC
1173	2.68E-17	-0.253888958	0.268	0.732	5.84E-13	TPM3	5	NUPR1_LSEC
1174	6.52E-18	-0.255556228	0.126	0.491	1.42E-13	TAF1D	5	NUPR1_LSEC
1175	3.31E-11	-0.25658273	0.406	0.801	7.22E-07	HLA-DRB5	5	NUPR1_LSEC
1176	8.10E-12	-0.25774301	0.113	0.373	1.77E-07	SLCO4A1	5	NUPR1_LSEC
1177	3.78E-11	-0.258727888	0.113	0.365	8.24E-07	CPM	5	NUPR1_LSEC
1178	1.16E-20	-0.260220139	0.146	0.559	2.54E-16	SRRM1	5	NUPR1_LSEC
1179	1.84E-19	-0.262729013	0.126	0.509	4.02E-15	SRRM2	5	NUPR1_LSEC
1180	1.07E-15	-0.263284684	0.159	0.521	2.34E-11	SOX7	5	NUPR1_LSEC
1181	6.79E-17	-0.263508616	0.146	0.509	1.48E-12	IRF1	5	NUPR1_LSEC
1182	6.29E-09	-0.263646412	0.289	0.593	0.000137211	TFPI2	5	NUPR1_LSEC
1183	5.46E-13	-0.264725486	0.188	0.527	1.19E-08	IL1R1	5	NUPR1_LSEC
1184	2.01E-19	-0.269256905	0.163	0.578	4.39E-15	CYB5R3	5	NUPR1_LSEC
1185	2.31E-14	-0.272927917	0.1	0.394	5.05E-10	TLR4	5	NUPR1_LSEC
1186	1.07E-16	-0.275927346	0.159	0.528	2.34E-12	MDK	5	NUPR1_LSEC
1187	4.44E-19	-0.276249159	0.1	0.464	9.67E-15	NDUFS2	5	NUPR1_LSEC
1188	1.85E-18	-0.277663394	0.126	0.5	4.03E-14	SHISA5	5	NUPR1_LSEC

1189	1.10E-19	-0.280013247	0.105	0.475	2.40E-15	HLA-F	5	NUPR1_LSEC
1190	4.33E-17	-0.28230804	0.134	0.494	9.43E-13	GNAI2	5	NUPR1_LSEC
1191	7.62E-16	-0.28385987	0.364	0.814	1.66E-11	ITGB1	5	NUPR1_LSEC
1192	3.05E-16	-0.284211806	0.109	0.435	6.65E-12	LINC00657	5	NUPR1_LSEC
1193	7.91E-21	-0.285806552	0.13	0.534	1.72E-16	RBMS1	5	NUPR1_LSEC
1194	1.20E-12	-0.287810199	0.397	0.785	2.62E-08	PRSS23	5	NUPR1_LSEC
1195	2.77E-13	-0.289664586	0.36	0.76	6.04E-09	ETS2	5	NUPR1_LSEC
1196	2.13E-18	-0.290267391	0.109	0.467	4.64E-14	SCAF11	5	NUPR1_LSEC
1197	1.55E-16	-0.290352284	0.201	0.597	3.38E-12	TMEM50B	5	NUPR1_LSEC
1198	6.76E-17	-0.291532767	0.255	0.701	1.47E-12	TNFRSF1A	5	NUPR1_LSEC
1199	3.40E-18	-0.293349512	0.197	0.62	7.41E-14	TMEM50A	5	NUPR1_LSEC
1200	8.90E-12	-0.293812565	0.536	0.91	1.94E-07	HSP90AB1	5	NUPR1_LSEC
1201	8.56E-18	-0.294261316	0.134	0.505	1.87E-13	ARL6IP5	5	NUPR1_LSEC
1202	6.13E-22	-0.295396074	0.163	0.614	1.34E-17	SERBP1	5	NUPR1_LSEC
1203	2.31E-18	-0.2986169	0.1	0.449	5.03E-14	TMEM30A	5	NUPR1_LSEC
1204	2.08E-20	-0.303185163	0.155	0.57	4.53E-16	MTDH	5	NUPR1_LSEC
1205	1.35E-14	-0.304808497	0.113	0.415	2.95E-10	CITED2	5	NUPR1_LSEC
1206	1.60E-20	-0.306238019	0.188	0.63	3.50E-16	TPST2	5	NUPR1_LSEC
1207	3.74E-17	-0.306593939	0.247	0.686	8.17E-13	SERPINH1	5	NUPR1_LSEC
1208	5.49E-22	-0.309252159	0.13	0.544	1.20E-17	TEK	5	NUPR1_LSEC
1209	1.55E-20	-0.309487663	0.126	0.525	3.37E-16	ASAP1	5	NUPR1_LSEC
1210	1.69E-16	-0.310444127	0.109	0.437	3.69E-12	ACTN4	5	NUPR1_LSEC
1211	2.07E-11	-0.315600446	0.368	0.737	4.51E-07	TSC22D3	5	NUPR1_LSEC
1212	4.07E-20	-0.316385926	0.138	0.538	8.89E-16	CEBPD	5	NUPR1_LSEC
1213	1.28E-19	-0.318479294	0.243	0.724	2.80E-15	ARL6IP1	5	NUPR1_LSEC
1214	3.17E-12	-0.322315359	0.172	0.465	6.92E-08	OLFM1	5	NUPR1_LSEC
1215	7.77E-17	-0.324827345	0.887	0.967	1.69E-12	HLA-E	5	NUPR1_LSEC
1216	3.33E-18	-0.326180686	0.326	0.816	7.27E-14	LAPTM4A	5	NUPR1_LSEC
1217	3.08E-21	-0.329281481	0.222	0.706	6.72E-17	EIF4A2	5	NUPR1_LSEC
1218	3.24E-20	-0.329707049	0.276	0.76	7.07E-16	MEF2C	5	NUPR1_LSEC
1219	2.11E-16	-0.333051985	0.234	0.651	4.61E-12	ARHGAP29	5	NUPR1_LSEC
1220	2.53E-15	-0.33590588	0.167	0.511	5.51E-11	SOX17	5	NUPR1_LSEC
1221	3.84E-19	-0.3360494	0.197	0.627	8.38E-15	HNRNPU	5	NUPR1_LSEC
1222	1.59E-11	-0.337755397	0.427	0.773	3.46E-07	ZFP36L1	5	NUPR1_LSEC
1223	7.90E-17	-0.338337703	0.28	0.726	1.72E-12	TACC1	5	NUPR1_LSEC
1224	5.44E-21	-0.339731133	0.925	0.99	1.19E-16	HLA-B	5	NUPR1_LSEC
1225	7.49E-10	-0.343901839	0.13	0.365	1.63E-05	HLA-DRA	5	NUPR1_LSEC
1226	9.88E-19	-0.346862941	0.243	0.686	2.15E-14	TUBA1B	5	NUPR1_LSEC
1227	5.31E-21	-0.347336883	0.109	0.491	1.16E-16	PIK3C2A	5	NUPR1_LSEC
1228	3.27E-14	-0.356033605	0.481	0.889	7.13E-10	STOM	5	NUPR1_LSEC
1229	1.23E-10	-0.362418353	0.544	0.84	2.69E-06	ID3	5	NUPR1_LSEC
1230	1.14E-17	-0.363184973	0.201	0.599	2.48E-13	PMP22	5	NUPR1_LSEC
1231	1.94E-19	-0.364334374	0.155	0.552	4.22E-15	NRP1	5	NUPR1_LSEC
1232	2.13E-18	-0.36866736	0.167	0.564	4.65E-14	YES1	5	NUPR1_LSEC
1233	5.14E-21	-0.371246778	0.142	0.547	1.12E-16	CDC42EP3	5	NUPR1_LSEC
1234	1.43E-27	-0.375999655	0.134	0.614	3.11E-23	SF3B1	5	NUPR1_LSEC
1235	3.33E-13	-0.377193054	0.234	0.572	7.27E-09	ICAM1	5	NUPR1_LSEC
1236	8.64E-22	-0.380635299	0.109	0.504	1.88E-17	TNFSF10	5	NUPR1_LSEC
1237	1.38E-21	-0.382255002	0.121	0.525	3.00E-17	CTNNB1	5	NUPR1_LSEC
1238	1.09E-20	-0.383211348	0.18	0.618	2.38E-16	EIF5	5	NUPR1_LSEC
1239	1.23E-18	-0.387415614	0.276	0.722	2.69E-14	LDHA	5	NUPR1_LSEC
1240	3.28E-17	-0.388217233	0.276	0.711	7.16E-13	S1PR1	5	NUPR1_LSEC
1241	4.94E-21	-0.394460255	0.151	0.568	1.08E-16	ITM2A	5	NUPR1_LSEC
1242	6.90E-20	-0.397503138	0.109	0.479	1.50E-15	GAS6	5	NUPR1_LSEC
1243	4.61E-22	-0.399372877	0.268	0.761	1.00E-17	MCL1	5	NUPR1_LSEC
1244	2.03E-25	-0.401634866	0.121	0.572	4.43E-21	SERINC3	5	NUPR1_LSEC
1245	8.73E-21	-0.4017393	0.226	0.688	1.90E-16	TGFBR3	5	NUPR1_LSEC
1246	1.30E-21	-0.402805716	0.105	0.491	2.83E-17	SNRK	5	NUPR1_LSEC
1247	7.74E-23	-0.403014867	0.243	0.739	1.69E-18	CDH5	5	NUPR1_LSEC
1248	2.59E-12	-0.404688162	0.192	0.49	5.65E-08	PNP	5	NUPR1_LSEC
1249	4.69E-23	-0.405472849	0.222	0.713	1.02E-18	NCL	5	NUPR1_LSEC
1250	1.33E-20	-0.405485881	0.109	0.478	2.89E-16	DDX21	5	NUPR1_LSEC
1251	4.42E-14	-0.407623203	0.331	0.733	9.64E-10	CRIP2	5	NUPR1_LSEC
1252	3.74E-18	-0.407796156	0.31	0.747	8.15E-14	ADGRF5	5	NUPR1_LSEC
1253	3.96E-19	-0.40854793	0.155	0.546	8.63E-15	BMPER	5	NUPR1_LSEC
1254	9.28E-21	-0.408763233	0.247	0.703	2.02E-16	DLC1	5	NUPR1_LSEC
1255	1.01E-08	-0.409254416	0.686	0.94	0.000220261	SAT1	5	NUPR1_LSEC
1256	4.99E-26	-0.412628908	0.176	0.669	1.09E-21	UBXN4	5	NUPR1_LSEC
1257	1.37E-24	-0.413214052	0.13	0.568	2.99E-20	DENND4C	5	NUPR1_LSEC
1258	7.20E-18	-0.415690497	0.142	0.506	1.57E-13	CD93	5	NUPR1_LSEC

1259	5.12E-27	-0.418407988	0.155	0.646	1.12E-22	SF1	5	NUPR1_LSEC
1260	6.34E-17	-0.419247987	0.335	0.756	1.38E-12	HYAL2	5	NUPR1_LSEC
1261	2.83E-26	-0.423092302	0.222	0.741	6.18E-22	RAB13	5	NUPR1_LSEC
1262	1.30E-20	-0.429106813	0.335	0.826	2.83E-16	CDC37	5	NUPR1_LSEC
1263	2.18E-13	-0.429799561	0.502	0.834	4.76E-09	RNASE1	5	NUPR1_LSEC
1264	1.62E-12	-0.430604084	0.523	0.887	3.53E-08	NFKBIA	5	NUPR1_LSEC
1265	2.54E-24	-0.430633064	0.155	0.609	5.54E-20	CALM2	5	NUPR1_LSEC
1266	2.92E-24	-0.431266881	0.163	0.627	6.38E-20	CD46	5	NUPR1_LSEC
1267	4.78E-27	-0.431515675	0.142	0.625	1.04E-22	SERINC1	5	NUPR1_LSEC
1268	1.36E-19	-0.436769443	0.322	0.79	2.97E-15	EPAS1	5	NUPR1_LSEC
1269	5.98E-14	-0.439168059	0.351	0.698	1.30E-09	ADM	5	NUPR1_LSEC
1270	1.74E-23	-0.439653322	0.146	0.583	3.80E-19	ACTN1	5	NUPR1_LSEC
1271	2.56E-25	-0.44335946	0.121	0.561	5.58E-21	EIF4A1	5	NUPR1_LSEC
1272	1.76E-20	-0.443660804	0.18	0.597	3.84E-16	EFNB1	5	NUPR1_LSEC
1273	5.34E-25	-0.44501691	0.117	0.551	1.16E-20	ZFAND5	5	NUPR1_LSEC
1274	4.77E-19	-0.44994155	0.577	0.908	1.04E-14	EGFL7	5	NUPR1_LSEC
1275	2.82E-27	-0.453179911	0.213	0.741	6.14E-23	SRSF5	5	NUPR1_LSEC
1276	3.41E-22	-0.453437718	0.36	0.838	7.43E-18	ANXA2	5	NUPR1_LSEC
1277	5.58E-28	-0.455258023	0.782	0.984	1.22E-23	PTMA	5	NUPR1_LSEC
1278	3.61E-13	-0.456645615	0.464	0.817	7.86E-09	A2M	5	NUPR1_LSEC
1279	2.38E-27	-0.457333201	0.142	0.621	5.20E-23	TIE1	5	NUPR1_LSEC
1280	4.36E-20	-0.458942455	0.406	0.851	9.52E-16	TINAGL1	5	NUPR1_LSEC
1281	6.19E-21	-0.460105976	0.113	0.496	1.35E-16	NEDD9	5	NUPR1_LSEC
1282	3.27E-23	-0.462006149	0.665	0.957	7.12E-19	H3F3B	5	NUPR1_LSEC
1283	5.77E-20	-0.463607421	0.628	0.946	1.26E-15	UBC	5	NUPR1_LSEC
1284	3.08E-28	-0.463760704	0.159	0.66	6.72E-24	ANP32B	5	NUPR1_LSEC
1285	2.38E-26	-0.46716384	0.105	0.547	5.18E-22	VIMP	5	NUPR1_LSEC
1286	2.21E-16	-0.475296477	0.464	0.882	4.81E-12	HSPG2	5	NUPR1_LSEC
1287	8.61E-11	-0.476148463	0.427	0.72	1.88E-06	C7	5	NUPR1_LSEC
1288	3.64E-26	-0.479313633	0.184	0.675	7.94E-22	RTN4	5	NUPR1_LSEC
1289	1.32E-28	-0.48080417	0.1	0.559	2.88E-24	TUBB4B	5	NUPR1_LSEC
1290	8.06E-23	-0.481525233	0.117	0.526	1.76E-18	TMEM2	5	NUPR1_LSEC
1291	6.24E-26	-0.484355596	0.1	0.531	1.36E-21	ERG	5	NUPR1_LSEC
1292	2.04E-14	-0.49302236	0.381	0.766	4.44E-10	CD74	5	NUPR1_LSEC
1293	8.24E-29	-0.505152118	0.117	0.593	1.80E-24	SFPQ	5	NUPR1_LSEC
1294	3.71E-30	-0.516297398	0.121	0.61	8.09E-26	PTTG1IP	5	NUPR1_LSEC
1295	9.96E-27	-0.516786584	0.653	0.99	2.17E-22	MT-ND3	5	NUPR1_LSEC
1296	8.55E-30	-0.518625483	0.159	0.658	1.87E-25	DDX3X	5	NUPR1_LSEC
1297	1.34E-19	-0.522523778	0.134	0.508	2.92E-15	C11orf96	5	NUPR1_LSEC
1298	3.29E-13	-0.53154317	0.678	0.929	7.18E-09	TIMP3	5	NUPR1_LSEC
1299	1.23E-29	-0.534333037	0.126	0.617	2.68E-25	STAT3	5	NUPR1_LSEC
1300	1.44E-32	-0.545071151	0.163	0.702	3.14E-28	KTN1	5	NUPR1_LSEC
1301	2.21E-25	-0.54839954	0.314	0.813	4.83E-21	PTPRB	5	NUPR1_LSEC
1302	1.57E-11	-0.548736487	0.155	0.415	3.42E-07	CCL2	5	NUPR1_LSEC
1303	1.65E-30	-0.553210719	0.218	0.765	3.60E-26	RBM39	5	NUPR1_LSEC
1304	6.72E-32	-0.563912389	0.201	0.743	1.47E-27	HNRNPA1	5	NUPR1_LSEC
1305	9.45E-23	-0.564407454	0.435	0.872	2.06E-18	CLEC3B	5	NUPR1_LSEC
1306	1.90E-35	-0.572397284	0.745	0.996	4.15E-31	MT-CO3	5	NUPR1_LSEC
1307	1.18E-26	-0.576399815	0.126	0.574	2.57E-22	EFNB2	5	NUPR1_LSEC
1308	1.38E-24	-0.580724326	0.226	0.685	3.01E-20	ID2	5	NUPR1_LSEC
1309	7.71E-31	-0.580946863	0.167	0.684	1.68E-26	ESAM	5	NUPR1_LSEC
1310	6.62E-28	-0.581118023	0.126	0.59	1.44E-23	PLK2	5	NUPR1_LSEC
1311	6.29E-20	-0.582860609	0.268	0.699	1.37E-15	RAMP2	5	NUPR1_LSEC
1312	1.78E-28	-0.590982385	0.519	0.976	3.88E-24	MT-ND4	5	NUPR1_LSEC
1313	2.20E-26	-0.592918557	0.126	0.571	4.79E-22	MT-ND6	5	NUPR1_LSEC
1314	1.28E-30	-0.593941029	0.151	0.66	2.79E-26	ST6GALNAC3	5	NUPR1_LSEC
1315	7.37E-25	-0.599074062	0.159	0.603	1.61E-20	KLF2	5	NUPR1_LSEC
1316	8.71E-28	-0.600972757	0.1	0.546	1.90E-23	ATF3	5	NUPR1_LSEC
1317	1.25E-31	-0.602391356	0.1	0.583	2.72E-27	CYYR1	5	NUPR1_LSEC
1318	1.66E-34	-0.607612563	0.351	0.906	3.62E-30	DDX5	5	NUPR1_LSEC
1319	9.32E-25	-0.609289889	0.117	0.53	2.03E-20	SOX4	5	NUPR1_LSEC
1320	5.70E-35	-0.620061201	0.113	0.639	1.24E-30	N4BP2L2	5	NUPR1_LSEC
1321	3.50E-29	-0.631963294	0.172	0.669	7.64E-25	EMCN	5	NUPR1_LSEC
1322	9.06E-34	-0.633706032	0.423	0.961	1.98E-29	MT-ND5	5	NUPR1_LSEC
1323	2.34E-17	-0.636955748	0.188	0.542	5.11E-13	GJA4	5	NUPR1_LSEC
1324	1.70E-20	-0.640036506	0.117	0.48	3.72E-16	EDNRB	5	NUPR1_LSEC
1325	7.01E-35	-0.661085604	0.1	0.614	1.53E-30	SRSF3	5	NUPR1_LSEC
1326	2.36E-28	-0.670970277	0.209	0.71	5.15E-24	CAV1	5	NUPR1_LSEC
1327	1.05E-37	-0.685764943	0.134	0.688	2.29E-33	SRSF11	5	NUPR1_LSEC
1328	1.18E-24	-0.68779186	0.51	0.914	2.57E-20	ID1	5	NUPR1_LSEC

1329	2.45E-31	-0.68942716	0.251	0.756	5.34E-27	DUSP6	5	NUPR1_LSEC
1330	4.01E-36	-0.690692916	0.126	0.662	8.75E-32	BHLHE40	5	NUPR1_LSEC
1331	8.93E-21	-0.700820135	0.184	0.581	1.95E-16	LMNA	5	NUPR1_LSEC
1332	9.94E-33	-0.701888938	0.18	0.703	2.17E-28	EFNA1	5	NUPR1_LSEC
1333	2.43E-36	-0.704272871	0.201	0.766	5.31E-32	RDX	5	NUPR1_LSEC
1334	1.08E-24	-0.7078232	0.205	0.645	2.35E-20	C8orf4	5	NUPR1_LSEC
1335	2.52E-27	-0.709707889	0.105	0.539	5.49E-23	MYADM	5	NUPR1_LSEC
1336	3.42E-38	-0.713236489	0.64	0.991	7.45E-34	MT-ATP6	5	NUPR1_LSEC
1337	4.15E-34	-0.71488619	0.226	0.771	9.04E-30	FLT1	5	NUPR1_LSEC
1338	5.00E-26	-0.715335943	0.444	0.843	1.09E-21	PLPP3	5	NUPR1_LSEC
1339	6.39E-27	-0.716690648	0.192	0.642	1.39E-22	MYC	5	NUPR1_LSEC
1340	1.02E-41	-0.718775003	0.594	0.992	2.23E-37	MT-ND4L	5	NUPR1_LSEC
1341	3.13E-46	-0.720173905	0.841	0.999	6.82E-42	MT-CO2	5	NUPR1_LSEC
1342	2.22E-34	-0.724659559	0.109	0.615	4.83E-30	NOTCH4	5	NUPR1_LSEC
1343	4.98E-40	-0.725003026	0.197	0.775	1.09E-35	PPP1R15A	5	NUPR1_LSEC
1344	5.45E-28	-0.740529075	0.159	0.625	1.19E-23	NR4A1	5	NUPR1_LSEC
1345	1.76E-41	-0.761788205	0.197	0.799	3.85E-37	PPFIBP1	5	NUPR1_LSEC
1346	1.02E-25	-0.76288291	0.159	0.585	2.22E-21	BGN	5	NUPR1_LSEC
1347	1.70E-20	-0.780946718	0.176	0.544	3.70E-16	IGFBP3	5	NUPR1_LSEC
1348	2.63E-18	-0.781853936	0.226	0.593	5.75E-14	SLC9A3R2	5	NUPR1_LSEC
1349	5.10E-32	-0.784888337	0.184	0.687	1.11E-27	SRPX	5	NUPR1_LSEC
1350	1.89E-39	-0.795561542	0.146	0.717	4.12E-35	TCF4	5	NUPR1_LSEC
1351	5.95E-35	-0.79863425	0.402	0.885	1.30E-30	IFITM1	5	NUPR1_LSEC
1352	3.16E-26	-0.802772695	0.36	0.805	6.88E-22	DDIT4	5	NUPR1_LSEC
1353	1.25E-42	-0.807298759	0.452	0.971	2.73E-38	MT-ATP8	5	NUPR1_LSEC
1354	8.40E-35	-0.809191976	0.126	0.639	1.83E-30	ZFP36L2	5	NUPR1_LSEC
1355	4.76E-35	-0.824484009	0.397	0.883	1.04E-30	SRGN	5	NUPR1_LSEC
1356	2.57E-48	-0.828523515	0.1	0.717	5.60E-44	SPTBN1	5	NUPR1_LSEC
1357	1.12E-45	-0.844573695	0.414	0.974	2.43E-41	MT-ND1	5	NUPR1_LSEC
1358	2.18E-43	-0.847384337	0.1	0.672	4.74E-39	JMJD1C	5	NUPR1_LSEC
1359	2.05E-54	-0.847518809	0.791	0.997	4.46E-50	MT-CO1	5	NUPR1_LSEC
1360	2.14E-51	-0.870259091	0.556	0.991	4.66E-47	MT-CYB	5	NUPR1_LSEC
1361	2.80E-31	-0.880693562	0.385	0.829	6.10E-27	RHOB	5	NUPR1_LSEC
1362	9.38E-45	-0.886009595	0.389	0.965	2.05E-40	MT-ND2	5	NUPR1_LSEC
1363	2.65E-38	-0.88980564	0.251	0.797	5.77E-34	KLF6	5	NUPR1_LSEC
1364	1.34E-51	-0.907946976	0.222	0.871	2.93E-47	HNRNPA2B1	5	NUPR1_LSEC
1365	2.08E-40	-0.914588114	0.322	0.877	4.53E-36	CALCRL	5	NUPR1_LSEC
1366	2.62E-47	-0.925130928	0.205	0.832	5.71E-43	SLC38A2	5	NUPR1_LSEC
1367	1.28E-43	-0.931527677	0.201	0.79	2.79E-39	PECAM1	5	NUPR1_LSEC
1368	3.36E-39	-0.950822227	0.197	0.747	7.32E-35	CLEC14A	5	NUPR1_LSEC
1369	3.47E-35	-1.003649418	0.301	0.834	7.56E-31	PLPP1	5	NUPR1_LSEC
1370	7.80E-59	-1.066255656	0.113	0.795	1.70E-54	WSB1	5	NUPR1_LSEC
1371	8.63E-47	-1.070187779	0.623	0.963	1.88E-42	TM4SF1	5	NUPR1_LSEC
1372	5.95E-50	-1.085414455	0.326	0.888	1.30E-45	CD9	5	NUPR1_LSEC
1373	5.25E-20	-1.102723292	0.268	0.655	1.14E-15	S100A6	5	NUPR1_LSEC
1374	3.88E-62	-1.109862591	0.146	0.857	8.47E-58	ARGLU1	5	NUPR1_LSEC
1375	1.60E-39	-1.132057245	0.31	0.8	3.50E-35	AKAP12	5	NUPR1_LSEC
1376	8.20E-42	-1.14686785	0.142	0.686	1.79E-37	EGR1	5	NUPR1_LSEC
1377	1.47E-37	-1.17021313	0.13	0.655	3.20E-33	GSN	5	NUPR1_LSEC
1378	2.90E-63	-1.182848643	0.46	0.948	6.33E-59	DUSP1	5	NUPR1_LSEC
1379	2.69E-57	-1.203234776	0.243	0.855	5.86E-53	SOCS3	5	NUPR1_LSEC
1380	1.19E-63	-1.271207848	0.117	0.808	2.60E-59	KLF4	5	NUPR1_LSEC
1381	6.02E-57	-1.33848013	0.28	0.862	1.31E-52	GADD45B	5	NUPR1_LSEC
1382	2.04E-61	-1.411671884	0.109	0.771	4.44E-57	FOSB	5	NUPR1_LSEC
1383	7.72E-70	-1.422202057	0.176	0.874	1.68E-65	SLC2A3	5	NUPR1_LSEC
1384	7.42E-76	-1.53853717	0.218	0.908	1.62E-71	IER2	5	NUPR1_LSEC
1385	1.55E-40	-1.674141113	0.113	0.621	3.38E-36	CXCL2	5	NUPR1_LSEC
1386	6.39E-81	-1.710127985	0.326	0.929	1.39E-76	JUN	5	NUPR1_LSEC
1387	2.02E-83	-1.719424853	0.255	0.93	4.40E-79	ZFP36	5	NUPR1_LSEC
1388	1.05E-97	-2.038927512	0.272	0.941	2.29E-93	JUNB	5	NUPR1_LSEC
1389	6.74E-76	-2.207319691	0.251	0.887	1.47E-71	VIM	5	NUPR1_LSEC
1390	1.13E-99	-2.235611285	0.31	0.954	2.46E-95	FOS	5	NUPR1_LSEC
1391	1.39E-118	-2.388915011	0.347	0.997	3.04E-114	MALAT1	5	NUPR1_LSEC
1392	4.84E-161	2.532401693	0.704	0.048	1.06E-156	RBP7	6	RBP7_VEC
1393	6.94E-91	2.019384033	0.961	0.541	1.51E-86	GSN	6	RBP7_VEC
1394	5.49E-69	1.920529025	0.453	0.058	1.20E-64	RGCC	6	RBP7_VEC
1395	3.03E-75	1.8699271	0.508	0.071	6.62E-71	CXCL12	6	RBP7_VEC
1396	1.15E-58	1.7510969	0.793	0.325	2.52E-54	PLVAP	6	RBP7_VEC
1397	6.02E-44	1.665842727	0.804	0.514	1.31E-39	SLC9A3R2	6	RBP7_VEC
1398	9.60E-27	1.636333268	0.268	0.052	2.09E-22	IGF2	6	RBP7_VEC

1399	2.76E-67	1.63320792	0.804	0.243	6.02E-63	SPARCL1	6	RBP7_VEC
1400	7.41E-59	1.626066747	0.939	0.565	1.62E-54	S100A6	6	RBP7_VEC
1401	2.57E-43	1.510984127	0.939	0.742	5.61E-39	PLPP1	6	RBP7_VEC
1402	9.20E-43	1.431276337	0.922	0.691	2.01E-38	CD74	6	RBP7_VEC
1403	1.42E-110	1.417633244	0.514	0.035	3.09E-106	LMCD1	6	RBP7_VEC
1404	7.50E-41	1.406363969	0.62	0.229	1.64E-36	SPRY1	6	RBP7_VEC
1405	1.45E-23	1.402204507	0.469	0.193	3.15E-19	C10orf10	6	RBP7_VEC
1406	1.03E-74	1.394871635	0.749	0.169	2.25E-70	AQP1	6	RBP7_VEC
1407	5.74E-34	1.387467318	0.156	0.009	1.25E-29	FABP4	6	RBP7_VEC
1408	3.24E-46	1.361175845	0.425	0.081	7.07E-42	CRIP1	6	RBP7_VEC
1409	4.23E-33	1.292020843	0.966	0.887	9.23E-29	TIMP3	6	RBP7_VEC
1410	4.06E-64	1.267365527	0.665	0.16	8.86E-60	CD34	6	RBP7_VEC
1411	2.25E-60	1.251184709	0.939	0.649	4.90E-56	CRIP2	6	RBP7_VEC
1412	1.16E-30	1.247134069	0.933	0.87	2.53E-26	SRP14	6	RBP7_VEC
1413	3.16E-63	1.214908213	0.95	0.683	6.89E-59	PECAM1	6	RBP7_VEC
1414	2.36E-51	1.203906828	0.67	0.22	5.15E-47	PODXL	6	RBP7_VEC
1415	2.73E-117	1.198418764	0.341	0.002	5.96E-113	SSUH2	6	RBP7_VEC
1416	2.58E-74	1.187412118	0.57	0.096	5.62E-70	PRKCDBP	6	RBP7_VEC
1417	7.50E-83	1.187220632	0.609	0.093	1.64E-78	PALMD	6	RBP7_VEC
1418	7.44E-12	1.164888162	0.631	0.494	1.62E-07	GLUL	6	RBP7_VEC
1419	6.34E-54	1.156929048	0.207	0.008	1.38E-49	IGFBP5	6	RBP7_VEC
1420	4.60E-42	1.148839462	0.872	0.615	1.00E-37	RAMP2	6	RBP7_VEC
1421	2.86E-80	1.134951224	0.425	0.037	6.25E-76	SLC6A6	6	RBP7_VEC
1422	5.33E-56	1.124346431	0.642	0.179	1.16E-51	LGALS3	6	RBP7_VEC
1423	2.94E-29	1.102702481	0.631	0.3	6.42E-25	HLA-DRA	6	RBP7_VEC
1424	3.70E-52	1.087535832	0.939	0.703	8.07E-48	EPAS1	6	RBP7_VEC
1425	1.14E-40	1.055126612	0.849	0.567	2.50E-36	TAGLN2	6	RBP7_VEC
1426	3.22E-45	1.054783951	0.777	0.388	7.03E-41	BCAM	6	RBP7_VEC
1427	1.63E-50	1.048199106	0.531	0.125	3.55E-46	ASS1	6	RBP7_VEC
1428	3.01E-36	1.038509666	0.855	0.618	6.57E-32	CAV1	6	RBP7_VEC
1429	1.64E-51	1.01463319	1	0.779	3.58E-47	VIM	6	RBP7_VEC
1430	4.36E-34	1.004198521	0.587	0.234	9.51E-30	FAM107A	6	RBP7_VEC
1431	1.26E-28	0.997415677	0.447	0.147	2.74E-24	JAG1	6	RBP7_VEC
1432	1.24E-49	0.993787871	0.547	0.136	2.69E-45	MTUS1	6	RBP7_VEC
1433	1.09E-07	0.983383033	0.112	0.032	0.002385205	ACKR1	6	RBP7_VEC
1434	1.11E-28	0.962169035	0.598	0.285	2.42E-24	ADAM15	6	RBP7_VEC
1435	1.10E-35	0.956911898	0.698	0.319	2.39E-31	PIK3R3	6	RBP7_VEC
1436	3.75E-59	0.951864155	0.201	0.004	8.18E-55	FMO2	6	RBP7_VEC
1437	1.82E-33	0.945744028	0.587	0.214	3.96E-29	SLCO2A1	6	RBP7_VEC
1438	1.23E-92	0.933676117	0.436	0.029	2.68E-88	SORBS2	6	RBP7_VEC
1439	2.14E-118	0.92435585	0.408	0.01	4.67E-114	CHRM3	6	RBP7_VEC
1440	2.18E-49	0.917129548	0.475	0.098	4.76E-45	TMEM47	6	RBP7_VEC
1441	1.25E-32	0.911983218	0.453	0.134	2.73E-28	PPA1	6	RBP7_VEC
1442	1.16E-15	0.910814331	0.38	0.165	2.54E-11	PLAT	6	RBP7_VEC
1443	1.32E-13	0.905629338	0.547	0.311	2.88E-09	VWF	6	RBP7_VEC
1444	7.90E-24	0.903821394	0.587	0.302	1.72E-19	UTRN	6	RBP7_VEC
1445	1.73E-11	0.896637667	0.615	0.48	3.77E-07	GJA4	6	RBP7_VEC
1446	4.75E-29	0.893080733	0.559	0.214	1.04E-24	ALPL	6	RBP7_VEC
1447	8.00E-12	0.887025417	0.24	0.087	1.75E-07	FABP5	6	RBP7_VEC
1448	1.22E-51	0.884112823	1	0.989	2.67E-47	TMSB10	6	RBP7_VEC
1449	1.57E-30	0.867781968	0.838	0.575	3.42E-26	EMCN	6	RBP7_VEC
1450	1.56E-27	0.865310713	0.927	0.82	3.41E-23	IFI27	6	RBP7_VEC
1451	5.77E-21	0.865078187	0.721	0.471	1.26E-16	TSC22D1	6	RBP7_VEC
1452	4.84E-51	0.863877026	0.486	0.099	1.06E-46	PREX1	6	RBP7_VEC
1453	5.87E-105	0.853877022	0.296	0.001	1.28E-100	SEMA3G	6	RBP7_VEC
1454	4.89E-59	0.852349291	0.441	0.064	1.07E-54	AIF1L	6	RBP7_VEC
1455	7.56E-31	0.849702303	0.603	0.258	1.65E-26	DOCK9	6	RBP7_VEC
1456	6.71E-54	0.844520058	0.536	0.105	1.46E-49	ANXA1	6	RBP7_VEC
1457	2.08E-21	0.836215063	0.447	0.18	4.54E-17	KCTD12	6	RBP7_VEC
1458	2.05E-24	0.815473973	0.709	0.432	4.47E-20	ATP1A1	6	RBP7_VEC
1459	3.41E-26	0.807367805	0.425	0.144	7.44E-22	ARL15	6	RBP7_VEC
1460	2.96E-13	0.80001653	0.458	0.267	6.47E-09	PPP1R14A	6	RBP7_VEC
1461	1.77E-34	0.795561279	0.436	0.117	3.86E-30	CDH13	6	RBP7_VEC
1462	3.67E-42	0.79349031	0.497	0.125	8.00E-38	RAPGEF3	6	RBP7_VEC
1463	1.07E-23	0.78628774	0.676	0.361	2.33E-19	LRRC32	6	RBP7_VEC
1464	3.66E-54	0.783972714	0.307	0.028	7.98E-50	JAG2	6	RBP7_VEC
1465	1.40E-14	0.769339928	0.525	0.301	3.05E-10	SOX18	6	RBP7_VEC
1466	1.83E-16	0.763127078	0.631	0.415	3.98E-12	COL4A1	6	RBP7_VEC
1467	3.41E-31	0.762976556	0.687	0.331	7.43E-27	LRRFIP1	6	RBP7_VEC
1468	4.30E-34	0.762119296	0.313	0.057	9.37E-30	FUT8	6	RBP7_VEC

1469	1.38E-28	0.761661319	0.916	0.753	3.02E-24	A2M	6	RBP7_VEC
1470	8.51E-31	0.758410342	0.872	0.607	1.85E-26	SPTBN1	6	RBP7_VEC
1471	3.65E-37	0.757785648	0.43	0.1	7.97E-33	IGFBP2	6	RBP7_VEC
1472	1.11E-48	0.756168088	0.391	0.062	2.43E-44	ITGA6	6	RBP7_VEC
1473	1.92E-24	0.751444968	0.48	0.19	4.19E-20	HEG1	6	RBP7_VEC
1474	3.54E-38	0.742142877	0.933	0.756	7.72E-34	ANXA2	6	RBP7_VEC
1475	2.33E-15	0.740186851	0.642	0.47	5.08E-11	MRPL33	6	RBP7_VEC
1476	1.10E-21	0.738247587	0.832	0.618	2.40E-17	TCF4	6	RBP7_VEC
1477	1.23E-18	0.73600591	0.827	0.674	2.68E-14	RDX	6	RBP7_VEC
1478	1.15E-21	0.734601856	0.771	0.596	2.51E-17	ESAM	6	RBP7_VEC
1479	3.50E-30	0.734393988	0.436	0.127	7.63E-26	HLA-DMA	6	RBP7_VEC
1480	2.64E-26	0.726775027	0.57	0.26	5.76E-22	SWAP70	6	RBP7_VEC
1481	6.76E-67	0.720597969	0.257	0.009	1.47E-62	MMP2	6	RBP7_VEC
1482	7.89E-35	0.716332641	0.413	0.1	1.72E-30	SYNPO	6	RBP7_VEC
1483	3.42E-25	0.713893096	0.391	0.121	7.46E-21	THSD7A	6	RBP7_VEC
1484	8.38E-49	0.70888134	0.134	0	1.83E-44	PI16	6	RBP7_VEC
1485	8.15E-13	0.705552226	0.927	0.806	1.78E-08	SPARC	6	RBP7_VEC
1486	7.92E-35	0.704169441	0.313	0.055	1.73E-30	NES	6	RBP7_VEC
1487	8.17E-10	0.697896547	0.553	0.398	1.78E-05	TXNIP	6	RBP7_VEC
1488	3.12E-21	0.69652607	0.737	0.522	6.80E-17	PTTG1IP	6	RBP7_VEC
1489	2.80E-10	0.692144228	0.486	0.314	6.10E-06	APOLD1	6	RBP7_VEC
1490	7.46E-94	0.682632877	0.313	0.006	1.63E-89	EBF1	6	RBP7_VEC
1491	9.00E-11	0.680974771	0.402	0.222	1.96E-06	HEY1	6	RBP7_VEC
1492	4.61E-16	0.680551774	0.654	0.434	1.01E-11	CD93	6	RBP7_VEC
1493	2.60E-18	0.678975724	0.603	0.347	5.67E-14	SYNE2	6	RBP7_VEC
1494	1.02E-62	0.67547236	0.307	0.021	2.22E-58	LCN6	6	RBP7_VEC
1495	1.64E-44	0.671869986	0.246	0.021	3.57E-40	ANXA3	6	RBP7_VEC
1496	8.64E-42	0.667910098	0.363	0.06	1.88E-37	ENTPD1	6	RBP7_VEC
1497	9.03E-21	0.666762187	0.877	0.733	1.97E-16	HLA-DRB5	6	RBP7_VEC
1498	3.56E-10	0.665956149	0.553	0.403	7.75E-06	HLA-DPA1	6	RBP7_VEC
1499	5.13E-29	0.663416864	0.363	0.095	1.12E-24	C9orf3	6	RBP7_VEC
1500	5.65E-18	0.662301262	0.709	0.505	1.23E-13	CLIC4	6	RBP7_VEC
1501	1.40E-44	0.661725334	0.168	0.006	3.05E-40	NOV	6	RBP7_VEC
1502	1.38E-22	0.658273087	0.48	0.201	3.02E-18	SSFA2	6	RBP7_VEC
1503	6.95E-26	0.658254995	0.246	0.043	1.52E-21	MCTP1	6	RBP7_VEC
1504	2.56E-31	0.656402141	0.397	0.102	5.59E-27	ARHGEF3	6	RBP7_VEC
1505	3.39E-12	0.655357225	0.469	0.285	7.38E-08	UACA	6	RBP7_VEC
1506	8.16E-17	0.653803182	0.642	0.423	1.78E-12	GNAI2	6	RBP7_VEC
1507	2.06E-22	0.649257992	0.358	0.108	4.48E-18	ASRGL1	6	RBP7_VEC
1508	5.81E-09	0.648615399	0.654	0.531	0.000126656	KLF2	6	RBP7_VEC
1509	7.50E-14	0.646865347	0.626	0.423	1.63E-09	NEDD9	6	RBP7_VEC
1510	8.41E-71	0.638646689	0.257	0.008	1.83E-66	MGLL	6	RBP7_VEC
1511	3.93E-49	0.636392783	0.358	0.047	8.56E-45	TMTC1	6	RBP7_VEC
1512	4.55E-65	0.630396772	0.263	0.011	9.93E-61	CX3CL1	6	RBP7_VEC
1513	4.24E-38	0.623405365	0.994	0.953	9.25E-34	PTMA	6	RBP7_VEC
1514	3.02E-26	0.622030752	0.352	0.095	6.58E-22	SOS1	6	RBP7_VEC
1515	9.11E-09	0.612405147	0.469	0.319	0.000198745	HLA-DPB1	6	RBP7_VEC
1516	7.49E-23	0.607209277	0.369	0.118	1.63E-18	TSPAN13	6	RBP7_VEC
1517	9.49E-21	0.604104501	0.832	0.647	2.07E-16	TACC1	6	RBP7_VEC
1518	4.31E-26	0.598162362	0.274	0.056	9.39E-22	C10orf54	6	RBP7_VEC
1519	1.33E-18	0.597650111	0.67	0.414	2.89E-14	MYH9	6	RBP7_VEC
1520	6.83E-10	0.59728384	0.592	0.426	1.49E-05	LGALS1	6	RBP7_VEC
1521	2.94E-08	0.596187918	0.346	0.18	0.000642105	CD320	6	RBP7_VEC
1522	1.08E-22	0.593038019	0.374	0.112	2.36E-18	GJA1	6	RBP7_VEC
1523	3.77E-16	0.583152003	0.687	0.449	8.22E-12	AHNAK	6	RBP7_VEC
1524	2.85E-07	0.582925799	0.352	0.202	0.00622616	CYR61	6	RBP7_VEC
1525	1.40E-12	0.58258582	0.536	0.329	3.06E-08	JAM2	6	RBP7_VEC
1526	4.31E-14	0.578185881	0.966	0.882	9.39E-10	HSPB1	6	RBP7_VEC
1527	1.02E-06	0.577587156	0.525	0.42	0.022256631	EDNRB	6	RBP7_VEC
1528	4.80E-16	0.577120894	0.425	0.195	1.05E-11	RPS6KA2	6	RBP7_VEC
1529	3.21E-16	0.575802269	0.832	0.681	6.99E-12	DSTN	6	RBP7_VEC
1530	8.71E-20	0.575587064	0.531	0.26	1.90E-15	NAA10	6	RBP7_VEC
1531	1.05E-15	0.573377748	0.486	0.24	2.29E-11	ARID5B	6	RBP7_VEC
1532	3.75E-07	0.57217325	0.587	0.48	0.008171159	CD81	6	RBP7_VEC
1533	1.46E-11	0.572019587	0.631	0.459	3.19E-07	YWHAH	6	RBP7_VEC
1534	6.04E-32	0.562916222	0.212	0.025	1.32E-27	SLC14A1	6	RBP7_VEC
1535	2.00E-28	0.558921971	0.251	0.042	4.37E-24	ADAMTS6	6	RBP7_VEC
1536	8.11E-16	0.558556184	0.385	0.164	1.77E-11	ACVRL1	6	RBP7_VEC
1537	5.06E-16	0.558298644	0.559	0.311	1.10E-11	EMP2	6	RBP7_VEC
1538	9.86E-17	0.555566057	0.43	0.192	2.15E-12	PTPRM	6	RBP7_VEC

1539	2.73E-74	0.554899072	0.218	0.001	5.95E-70	CD200	6	RBP7_VEC
1540	3.11E-08	0.554080308	0.101	0.024	0.00067911	POSTN	6	RBP7_VEC
1541	6.49E-13	0.552396787	0.447	0.241	1.42E-08	MECOM	6	RBP7_VEC
1542	8.94E-14	0.551937225	0.564	0.353	1.95E-09	AES	6	RBP7_VEC
1543	1.18E-10	0.549077747	0.464	0.257	2.58E-06	IL32	6	RBP7_VEC
1544	1.76E-47	0.545840134	0.33	0.042	3.83E-43	GPRC5B	6	RBP7_VEC
1545	3.69E-09	0.542126363	0.687	0.514	8.06E-05	THBD	6	RBP7_VEC
1546	1.67E-24	0.537306666	0.994	0.988	3.64E-20	TMSB4X	6	RBP7_VEC
1547	9.39E-63	0.531969893	0.173	0	2.05E-58	COL15A1	6	RBP7_VEC
1548	8.58E-15	0.531228052	0.475	0.248	1.87E-10	LMO2	6	RBP7_VEC
1549	4.97E-12	0.530524883	0.654	0.492	1.08E-07	NFIB	6	RBP7_VEC
1550	7.57E-15	0.52974412	0.659	0.449	1.65E-10	CTNNB1	6	RBP7_VEC
1551	9.47E-09	0.529090367	0.849	0.76	0.000206542	RHOB	6	RBP7_VEC
1552	9.25E-17	0.528842194	0.922	0.788	2.02E-12	CALCRL	6	RBP7_VEC
1553	1.50E-44	0.528682132	0.14	0.002	3.27E-40	CLIC3	6	RBP7_VEC
1554	3.61E-10	0.527446048	0.43	0.255	7.87E-06	MEF2A	6	RBP7_VEC
1555	2.69E-07	0.52626414	0.307	0.172	0.005865645	GPIHBP1	6	RBP7_VEC
1556	4.83E-13	0.521893695	0.832	0.737	1.05E-08	SNHG7	6	RBP7_VEC
1557	2.85E-09	0.520858662	0.291	0.136	6.22E-05	AZIN1	6	RBP7_VEC
1558	1.28E-10	0.520366082	0.358	0.171	2.80E-06	MALL	6	RBP7_VEC
1559	6.84E-15	0.515927381	0.821	0.698	1.49E-10	S100A11	6	RBP7_VEC
1560	3.38E-15	0.515719894	0.944	0.812	7.38E-11	HSPG2	6	RBP7_VEC
1561	1.17E-27	0.515023877	0.313	0.067	2.55E-23	MMP28	6	RBP7_VEC
1562	1.60E-14	0.514726176	0.693	0.471	3.50E-10	NAA38	6	RBP7_VEC
1563	6.16E-19	0.512049761	0.413	0.164	1.34E-14	EFCAB14	6	RBP7_VEC
1564	2.26E-29	0.510754661	0.229	0.033	4.94E-25	PDGFB	6	RBP7_VEC
1565	1.13E-06	0.509232328	0.67	0.532	0.024612953	ADAMTS1	6	RBP7_VEC
1566	2.12E-12	0.504640055	0.782	0.681	4.62E-08	PTRF	6	RBP7_VEC
1567	4.76E-15	0.496968378	0.559	0.304	1.04E-10	NDRG1	6	RBP7_VEC
1568	1.40E-08	0.496446574	0.955	0.912	0.000304646	TM4SF1	6	RBP7_VEC
1569	2.62E-20	0.496000013	0.352	0.115	5.72E-16	TPM1	6	RBP7_VEC
1570	2.35E-07	0.495031467	0.458	0.321	0.005123574	TMEM88	6	RBP7_VEC
1571	1.08E-11	0.492181373	0.659	0.484	2.36E-07	NDUFA12	6	RBP7_VEC
1572	5.49E-13	0.489648451	0.212	0.064	1.20E-08	CTSC	6	RBP7_VEC
1573	4.57E-09	0.4893478	0.749	0.693	9.98E-05	HYAL2	6	RBP7_VEC
1574	4.43E-13	0.487302094	0.922	0.808	9.66E-09	IFITM1	6	RBP7_VEC
1575	3.11E-07	0.484690225	0.369	0.226	0.006784959	HEXIM1	6	RBP7_VEC
1576	2.32E-15	0.48304452	0.771	0.468	5.06E-11	EMP1	6	RBP7_VEC
1577	1.86E-13	0.481638181	0.497	0.276	4.05E-09	COX17	6	RBP7_VEC
1578	4.53E-12	0.48092185	0.67	0.505	9.89E-08	CYB5R3	6	RBP7_VEC
1579	2.76E-13	0.478890703	0.48	0.266	6.03E-09	MAP4	6	RBP7_VEC
1580	2.64E-12	0.477329891	0.642	0.494	5.76E-08	ARPC1B	6	RBP7_VEC
1581	3.34E-20	0.475583099	0.972	0.946	7.28E-16	IFITM2	6	RBP7_VEC
1582	1.50E-11	0.475499008	0.413	0.218	3.27E-07	WNK1	6	RBP7_VEC
1583	1.35E-11	0.475092474	0.944	0.85	2.95E-07	ID1	6	RBP7_VEC
1584	2.95E-10	0.474850506	0.363	0.195	6.43E-06	CD58	6	RBP7_VEC
1585	4.96E-11	0.474469411	0.559	0.374	1.08E-06	ACTN4	6	RBP7_VEC
1586	1.13E-11	0.472642802	0.453	0.251	2.46E-07	GABARAPL1	6	RBP7_VEC
1587	4.52E-14	0.468699698	0.721	0.505	9.86E-10	LMNA	6	RBP7_VEC
1588	2.61E-19	0.46689374	0.268	0.071	5.69E-15	TES	6	RBP7_VEC
1589	3.06E-24	0.466790947	0.285	0.064	6.67E-20	NQO1	6	RBP7_VEC
1590	1.01E-11	0.463599025	0.531	0.32	2.20E-07	ADCY4	6	RBP7_VEC
1591	2.66E-24	0.46163776	0.229	0.042	5.81E-20	SLC12A2	6	RBP7_VEC
1592	7.21E-10	0.460881259	0.922	0.862	1.57E-05	HLA-DRB1	6	RBP7_VEC
1593	1.72E-12	0.459312401	0.927	0.767	3.75E-08	GADD45B	6	RBP7_VEC
1594	3.17E-40	0.45908516	0.173	0.009	6.92E-36	PLLP	6	RBP7_VEC
1595	2.14E-22	0.45747358	0.961	0.87	4.66E-18	H3F3A	6	RBP7_VEC
1596	1.83E-52	0.457218562	0.168	0.003	3.98E-48	SCNN1B	6	RBP7_VEC
1597	4.11E-09	0.456697898	0.469	0.307	8.97E-05	HLA-DQB1	6	RBP7_VEC
1598	9.50E-10	0.456293757	0.587	0.424	2.07E-05	NUCKS1	6	RBP7_VEC
1599	2.15E-15	0.455932377	0.966	0.798	4.69E-11	IER2	6	RBP7_VEC
1600	8.09E-37	0.45553526	0.235	0.025	1.76E-32	TM6SF1	6	RBP7_VEC
1601	2.39E-52	0.453011249	0.207	0.008	5.20E-48	FAM13C	6	RBP7_VEC
1602	1.87E-13	0.452892049	0.804	0.68	4.07E-09	GUK1	6	RBP7_VEC
1603	2.39E-10	0.452459992	0.883	0.804	5.22E-06	CD9	6	RBP7_VEC
1604	1.12E-10	0.449867085	0.587	0.417	2.45E-06	LAP3	6	RBP7_VEC
1605	5.44E-14	0.4498175	0.235	0.073	1.19E-09	CXorf36	6	RBP7_VEC
1606	6.80E-37	0.447735257	0.212	0.02	1.48E-32	KCNN3	6	RBP7_VEC
1607	8.08E-20	0.445383028	0.983	0.91	1.76E-15	H3F3B	6	RBP7_VEC
1608	1.60E-10	0.444943218	0.665	0.466	3.49E-06	GIMAP7	6	RBP7_VEC

1609	1.56E-11	0.44325056	0.391	0.196	3.40E-07	RUNX1T1	6	RBP7_VEC
1610	1.75E-08	0.443054551	0.486	0.34	0.000380985	ADD1	6	RBP7_VEC
1611	1.66E-10	0.440164846	0.665	0.502	3.61E-06	CYYR1	6	RBP7_VEC
1612	4.37E-13	0.43995146	0.235	0.076	9.52E-09	RAPGEF1	6	RBP7_VEC
1613	3.70E-11	0.438630753	0.469	0.268	8.06E-07	SKIL	6	RBP7_VEC
1614	4.89E-09	0.438358754	0.425	0.26	0.000106683	CAPN2	6	RBP7_VEC
1615	1.13E-36	0.435647277	0.106	0.001	2.47E-32	FBLN5	6	RBP7_VEC
1616	2.14E-12	0.435496983	0.475	0.246	4.67E-08	PLEC	6	RBP7_VEC
1617	7.14E-08	0.433027375	0.363	0.222	0.001557034	ZBTB38	6	RBP7_VEC
1618	3.71E-09	0.432031977	0.855	0.712	8.09E-05	ZFP36L1	6	RBP7_VEC
1619	3.81E-11	0.43179852	0.425	0.241	8.31E-07	FKBP9	6	RBP7_VEC
1620	9.53E-25	0.430790444	0.983	0.982	2.08E-20	IFITM3	6	RBP7_VEC
1621	9.66E-10	0.427840483	0.43	0.255	2.11E-05	GNAQ	6	RBP7_VEC
1622	8.64E-38	0.426929638	0.179	0.011	1.88E-33	VEGFC	6	RBP7_VEC
1623	1.22E-11	0.422735308	0.296	0.126	2.65E-07	CTSH	6	RBP7_VEC
1624	5.07E-10	0.421819488	0.263	0.11	1.11E-05	HS3ST1	6	RBP7_VEC
1625	4.44E-14	0.420867441	0.246	0.078	9.69E-10	HSPA12B	6	RBP7_VEC
1626	1.16E-08	0.41632416	0.587	0.44	0.000253343	ARL6IP5	6	RBP7_VEC
1627	1.77E-09	0.415809962	0.866	0.807	3.85E-05	RHOC	6	RBP7_VEC
1628	4.57E-09	0.412876817	0.816	0.656	9.97E-05	CLEC14A	6	RBP7_VEC
1629	5.89E-17	0.411649069	0.95	0.838	1.28E-12	RHOA	6	RBP7_VEC
1630	9.21E-31	0.410552254	0.179	0.017	2.01E-26	VWA1	6	RBP7_VEC
1631	1.15E-08	0.409107852	0.14	0.042	0.000250429	HLA-DQA1	6	RBP7_VEC
1632	5.31E-26	0.408618592	0.168	0.019	1.16E-21	PDGFD	6	RBP7_VEC
1633	1.63E-13	0.407823215	0.955	0.933	3.55E-09	S100A10	6	RBP7_VEC
1634	1.32E-14	0.407103063	0.268	0.09	2.88E-10	CD109	6	RBP7_VEC
1635	8.26E-09	0.404407442	0.313	0.161	0.00018023	SMARCA2	6	RBP7_VEC
1636	1.11E-12	0.403674287	0.302	0.118	2.42E-08	SH3BGRL2	6	RBP7_VEC
1637	3.42E-07	0.402939871	0.514	0.398	0.007447817	SP100	6	RBP7_VEC
1638	1.09E-07	0.402179152	0.458	0.309	0.002374535	MAST4	6	RBP7_VEC
1639	4.21E-08	0.401767203	0.223	0.097	0.000918999	XPR1	6	RBP7_VEC
1640	1.36E-07	0.401395486	0.693	0.555	0.002961123	ZFP36L2	6	RBP7_VEC
1641	5.20E-13	0.401239202	0.302	0.117	1.13E-08	GALNT15	6	RBP7_VEC
1642	5.45E-09	0.400318101	0.771	0.638	0.000118749	S1PR1	6	RBP7_VEC
1643	3.51E-07	0.399680896	0.626	0.503	0.007643998	MMRN2	6	RBP7_VEC
1644	1.53E-08	0.397994959	0.899	0.803	0.000334454	CLEC3B	6	RBP7_VEC
1645	4.56E-09	0.397578159	0.866	0.836	9.95E-05	CD59	6	RBP7_VEC
1646	3.95E-09	0.39353795	0.385	0.212	8.60E-05	GATA2	6	RBP7_VEC
1647	6.97E-11	0.3931332	0.626	0.422	1.52E-06	IL3RA	6	RBP7_VEC
1648	4.04E-09	0.39190242	0.335	0.173	8.80E-05	FES	6	RBP7_VEC
1649	6.27E-10	0.391728717	0.307	0.145	1.37E-05	RFTN1	6	RBP7_VEC
1650	1.84E-08	0.391573873	0.777	0.649	0.000401815	LDHA	6	RBP7_VEC
1651	4.71E-07	0.391031128	0.302	0.166	0.010278848	NUDT4	6	RBP7_VEC
1652	2.95E-10	0.389069931	0.872	0.773	6.43E-06	HNRNPA2B1	6	RBP7_VEC
1653	1.49E-15	0.388934625	0.939	0.772	3.25E-11	RNASE1	6	RBP7_VEC
1654	2.07E-07	0.385380198	0.654	0.527	0.004521003	ETS1	6	RBP7_VEC
1655	2.86E-07	0.383438682	0.408	0.258	0.006234657	BCL2L1	6	RBP7_VEC
1656	6.39E-32	0.381729502	0.173	0.014	1.39E-27	APOL4	6	RBP7_VEC
1657	1.00E-07	0.380766169	0.419	0.259	0.002180924	TMEM173	6	RBP7_VEC
1658	2.22E-10	0.380624729	0.24	0.092	4.84E-06	MYL9	6	RBP7_VEC
1659	3.68E-11	0.380403021	0.251	0.095	8.02E-07	SUN2	6	RBP7_VEC
1660	4.60E-08	0.379968041	0.592	0.464	0.001002681	MSN	6	RBP7_VEC
1661	1.11E-26	0.379189537	0.123	0.008	2.42E-22	IMPG2	6	RBP7_VEC
1662	1.75E-29	0.378455225	0.173	0.016	3.81E-25	ITGB4	6	RBP7_VEC
1663	1.28E-17	0.37774626	0.151	0.024	2.79E-13	CFH	6	RBP7_VEC
1664	2.77E-23	0.374868312	0.983	0.951	6.05E-19	HLA-C	6	RBP7_VEC
1665	2.66E-10	0.371392032	0.313	0.144	5.79E-06	PPIC	6	RBP7_VEC
1666	1.05E-11	0.37137878	0.229	0.08	2.30E-07	NAV1	6	RBP7_VEC
1667	4.47E-08	0.371334057	0.196	0.079	0.000974749	TNFRSF4	6	RBP7_VEC
1668	3.16E-25	0.371312695	1	0.979	6.88E-21	HLA-B	6	RBP7_VEC
1669	6.27E-33	0.366044041	0.14	0.007	1.37E-28	UNC5B	6	RBP7_VEC
1670	1.36E-08	0.364895652	0.341	0.183	0.00029721	SP110	6	RBP7_VEC
1671	7.14E-10	0.362744617	0.955	0.897	1.56E-05	UBC	6	RBP7_VEC
1672	1.37E-08	0.361910674	0.296	0.146	0.000297775	HIP1	6	RBP7_VEC
1673	3.68E-07	0.360212637	0.503	0.358	0.008024975	FOXP1	6	RBP7_VEC
1674	9.34E-19	0.360201783	0.212	0.045	2.04E-14	FILIP1L	6	RBP7_VEC
1675	5.86E-16	0.358773913	0.128	0.02	1.28E-11	CLDN15	6	RBP7_VEC
1676	5.61E-14	0.358515375	0.246	0.078	1.22E-09	C8orf33	6	RBP7_VEC
1677	3.82E-09	0.357627626	0.274	0.124	8.33E-05	SULF2	6	RBP7_VEC
1678	1.06E-11	0.357322026	0.916	0.839	2.32E-07	FKBP1A	6	RBP7_VEC

1679	3.92E-09	0.357278657	0.274	0.124	8.54E-05	ARHGEF15	6	RBP7_VEC
1680	7.04E-10	0.355974281	0.212	0.078	1.54E-05	FAM110D	6	RBP7_VEC
1681	1.08E-06	0.351486883	0.43	0.286	0.023610511	PKIG	6	RBP7_VEC
1682	5.73E-12	0.351028956	0.251	0.092	1.25E-07	CCNY	6	RBP7_VEC
1683	2.07E-10	0.35086245	0.268	0.11	4.51E-06	MCC	6	RBP7_VEC
1684	1.38E-07	0.346882841	0.358	0.206	0.003009718	CASKIN2	6	RBP7_VEC
1685	1.23E-06	0.346736947	0.547	0.423	0.026746216	WASF2	6	RBP7_VEC
1686	2.15E-08	0.344721256	0.682	0.549	0.000468869	TMEM50A	6	RBP7_VEC
1687	1.97E-09	0.343960422	0.693	0.531	4.29E-05	CALM2	6	RBP7_VEC
1688	1.47E-06	0.343425676	0.654	0.529	0.031952103	LIMS2	6	RBP7_VEC
1689	1.53E-07	0.342991324	0.475	0.325	0.003335937	POLR2K	6	RBP7_VEC
1690	7.63E-18	0.342107537	0.184	0.036	1.66E-13	LPCAT2	6	RBP7_VEC
1691	5.17E-12	0.340948228	0.922	0.821	1.13E-07	DDX5	6	RBP7_VEC
1692	4.94E-10	0.34084873	0.838	0.671	1.08E-05	BTG1	6	RBP7_VEC
1693	2.66E-18	0.33961802	0.145	0.021	5.80E-14	HLA-DMB	6	RBP7_VEC
1694	7.01E-08	0.339572563	0.525	0.393	0.001529073	RAP1A	6	RBP7_VEC
1695	2.31E-12	0.339017338	0.218	0.069	5.04E-08	IQCK	6	RBP7_VEC
1696	6.04E-07	0.338484008	0.196	0.085	0.013180355	MCF2L	6	RBP7_VEC
1697	1.18E-08	0.335063551	0.212	0.085	0.000256764	MAP1B	6	RBP7_VEC
1698	2.05E-06	0.334748344	0.408	0.277	0.044787526	PKM	6	RBP7_VEC
1699	1.04E-06	0.333788477	0.626	0.502	0.022771591	MTDH	6	RBP7_VEC
1700	2.92E-10	0.331470497	0.251	0.1	6.37E-06	CRYBG3	6	RBP7_VEC
1701	3.84E-07	0.33028607	0.335	0.19	0.008367879	SLFN5	6	RBP7_VEC
1702	3.45E-09	0.32982517	0.24	0.098	7.51E-05	EPHA4	6	RBP7_VEC
1703	7.99E-11	0.32861845	0.916	0.84	1.74E-06	RAC1	6	RBP7_VEC
1704	1.07E-08	0.328238092	0.235	0.098	0.000232978	FGR	6	RBP7_VEC
1705	5.15E-07	0.327936605	0.268	0.137	0.011240052	ABL2	6	RBP7_VEC
1706	4.09E-10	0.325890288	0.212	0.078	8.91E-06	NHSL2	6	RBP7_VEC
1707	3.50E-07	0.325812323	0.268	0.137	0.00762423	APOL3	6	RBP7_VEC
1708	2.78E-11	0.325498213	0.927	0.829	6.07E-07	HMGB1	6	RBP7_VEC
1709	2.99E-08	0.324191931	0.654	0.508	0.0006518	MAP1LC3B	6	RBP7_VEC
1710	1.31E-06	0.322898714	0.475	0.335	0.028628475	ZEB1	6	RBP7_VEC
1711	3.71E-08	0.322761634	0.201	0.08	0.00080943	RNF144B	6	RBP7_VEC
1712	6.09E-08	0.320431114	0.475	0.304	0.001328364	CTTNBP2NL	6	RBP7_VEC
1713	1.39E-06	0.320267381	0.676	0.534	0.030305496	GIMAP1	6	RBP7_VEC
1714	2.05E-10	0.319294151	0.257	0.104	4.47E-06	KIAA1462	6	RBP7_VEC
1715	6.79E-17	0.317825471	0.156	0.028	1.48E-12	RASGRF2	6	RBP7_VEC
1716	9.71E-11	0.317334356	0.106	0.021	2.12E-06	TPD52L1	6	RBP7_VEC
1717	5.67E-07	0.316159238	0.291	0.156	0.0123722	CYFIP1	6	RBP7_VEC
1718	2.07E-08	0.314693985	0.196	0.074	0.000451563	CSRP2	6	RBP7_VEC
1719	1.27E-06	0.312162096	0.978	0.854	0.027709486	FOS	6	RBP7_VEC
1720	2.69E-08	0.312005492	0.201	0.081	0.000585961	HIP1R	6	RBP7_VEC
1721	4.95E-07	0.311132385	0.698	0.593	0.010796809	EID1	6	RBP7_VEC
1722	1.68E-07	0.310662859	0.296	0.158	0.003654494	MVP	6	RBP7_VEC
1723	1.02E-25	0.310155969	0.106	0.005	2.23E-21	TMEM178A	6	RBP7_VEC
1724	4.83E-07	0.308928336	0.257	0.127	0.010526937	TBC1D8	6	RBP7_VEC
1725	1.29E-09	0.308169544	0.296	0.134	2.81E-05	SHROOM4	6	RBP7_VEC
1726	7.40E-12	0.3078576	0.173	0.047	1.61E-07	CCDC88C	6	RBP7_VEC
1727	1.98E-07	0.307433988	0.117	0.034	0.00430955	PTGDS	6	RBP7_VEC
1728	3.02E-07	0.307181943	0.944	0.827	0.006586413	ZFP36	6	RBP7_VEC
1729	9.91E-10	0.305883765	0.14	0.037	2.16E-05	TNXB	6	RBP7_VEC
1730	1.66E-11	0.305454325	0.922	0.837	3.61E-07	CFL1	6	RBP7_VEC
1731	4.08E-07	0.304985303	0.335	0.191	0.008888606	BCL6B	6	RBP7_VEC
1732	4.29E-07	0.304759489	0.285	0.149	0.00936558	FURIN	6	RBP7_VEC
1733	2.13E-06	0.304464025	0.402	0.264	0.046467267	LUZP1	6	RBP7_VEC
1734	1.16E-07	0.3004333	0.246	0.115	0.002538918	TMEM184B	6	RBP7_VEC
1735	2.52E-16	0.300299166	0.179	0.038	5.49E-12	C1orf115	6	RBP7_VEC
1736	9.55E-08	0.296446013	0.922	0.868	0.002081646	MYL12B	6	RBP7_VEC
1737	1.90E-06	0.296123719	0.251	0.13	0.041472699	RABGAP1	6	RBP7_VEC
1738	8.40E-07	0.295677093	0.709	0.595	0.018318127	EIF4G2	6	RBP7_VEC
1739	8.40E-07	0.294911501	0.765	0.662	0.018309098	ARPC2	6	RBP7_VEC
1740	1.07E-06	0.294501664	0.307	0.175	0.02343041	LRCH1	6	RBP7_VEC
1741	8.53E-07	0.293493612	0.631	0.507	0.018594641	ZNHIT1	6	RBP7_VEC
1742	6.06E-24	0.292468106	0.123	0.009	1.32E-19	CMKLR1	6	RBP7_VEC
1743	7.07E-08	0.292447379	0.302	0.158	0.001541424	FDFT1	6	RBP7_VEC
1744	1.62E-38	0.291706884	0.117	0.001	3.53E-34	RP11-175K6.1	6	RBP7_VEC
1745	3.89E-10	0.290103526	0.156	0.045	8.49E-06	WDR86	6	RBP7_VEC
1746	1.48E-46	0.289506611	0.151	0.003	3.22E-42	AQP7	6	RBP7_VEC
1747	1.31E-22	0.287153442	0.173	0.024	2.86E-18	MSRB3	6	RBP7_VEC
1748	3.56E-08	0.286504163	0.19	0.072	0.000776251	NEURL1B	6	RBP7_VEC

1749	1.88E-06	0.286060599	0.223	0.107	0.040905919	ABLIM1	6	RBP7_VEC
1750	9.38E-07	0.285702478	0.229	0.112	0.02046619	ATP8B1	6	RBP7_VEC
1751	7.50E-21	0.284758071	0.117	0.011	1.64E-16	SYNE3	6	RBP7_VEC
1752	2.06E-06	0.284307452	0.754	0.66	0.044828591	TPM3	6	RBP7_VEC
1753	2.96E-09	0.281913468	0.844	0.737	6.46E-05	SKP1	6	RBP7_VEC
1754	1.21E-07	0.28154734	0.257	0.121	0.002646211	DHRS3	6	RBP7_VEC
1755	6.51E-08	0.280534804	0.235	0.103	0.001420386	FZD6	6	RBP7_VEC
1756	1.93E-08	0.280040497	0.207	0.083	0.00041999	PCNX	6	RBP7_VEC
1757	2.24E-06	0.278358273	0.223	0.109	0.048955011	STK10	6	RBP7_VEC
1758	4.91E-10	0.277481598	0.162	0.049	1.07E-05	SOX13	6	RBP7_VEC
1759	3.13E-08	0.27620615	0.279	0.13	0.000681884	LAMA5	6	RBP7_VEC
1760	2.33E-09	0.275663264	0.603	0.36	5.07E-05	ADIRF	6	RBP7_VEC
1761	1.46E-08	0.274769018	0.179	0.064	0.000317758	KIAA1671	6	RBP7_VEC
1762	2.19E-12	0.27388214	0.101	0.016	4.78E-08	SIPA1L2	6	RBP7_VEC
1763	5.96E-11	0.273183144	0.184	0.055	1.30E-06	GATA2-AS1	6	RBP7_VEC
1764	1.19E-06	0.272318865	0.464	0.323	0.025974394	RRAGA	6	RBP7_VEC
1765	2.25E-07	0.271842645	0.162	0.06	0.004902768	CDK6	6	RBP7_VEC
1766	9.15E-08	0.271075816	0.184	0.071	0.001994712	MAP2	6	RBP7_VEC
1767	4.71E-07	0.270425638	0.972	0.837	0.01027355	JUNB	6	RBP7_VEC
1768	5.35E-08	0.269417461	0.19	0.073	0.001166541	CTSZ	6	RBP7_VEC
1769	4.79E-21	0.268952721	0.134	0.014	1.05E-16	PCDH1	6	RBP7_VEC
1770	4.32E-08	0.267112276	0.196	0.076	0.000942881	PLCG2	6	RBP7_VEC
1771	1.76E-10	0.266289656	0.19	0.061	3.84E-06	SLC48A1	6	RBP7_VEC
1772	9.17E-09	0.265081793	0.201	0.077	0.000199876	FBN1	6	RBP7_VEC
1773	4.90E-31	0.264153735	0.123	0.005	1.07E-26	COBLL1	6	RBP7_VEC
1774	6.03E-16	0.262847218	0.168	0.033	1.32E-11	CDC14B	6	RBP7_VEC
1775	1.01E-13	0.261925485	0.14	0.027	2.20E-09	BEX5	6	RBP7_VEC
1776	2.03E-11	0.259743312	1	0.899	4.43E-07	MALAT1	6	RBP7_VEC
1777	5.24E-08	0.257779295	0.145	0.048	0.001142745	PARP12	6	RBP7_VEC
1778	4.33E-11	0.256117517	0.994	0.96	9.44E-07	HLA-A	6	RBP7_VEC
1779	1.81E-09	0.255563752	0.101	0.021	3.95E-05	XPO6	6	RBP7_VEC
1780	1.18E-14	0.253583668	0.14	0.025	2.57E-10	FGF2	6	RBP7_VEC
1781	4.52E-31	0.252254961	0.106	0.003	9.87E-27	ADAMTSL1	6	RBP7_VEC
1782	3.86E-15	0.251833127	0.117	0.017	8.43E-11	MN1	6	RBP7_VEC
1783	1.20E-08	0.251529982	0.196	0.072	0.000261649	MSI2	6	RBP7_VEC
1784	1.91E-06	0.250986588	0.358	0.212	0.041730719	PDLIM7	6	RBP7_VEC
1785	1.47E-11	-0.25061119	0.978	0.987	3.21E-07	RPL26	6	RBP7_VEC
1786	6.31E-15	-0.263792246	0.983	0.991	1.38E-10	RPL32	6	RBP7_VEC
1787	6.35E-09	-0.267974656	0.95	0.957	0.000138536	UBA52	6	RBP7_VEC
1788	2.89E-08	-0.276118073	0.676	0.831	0.000631087	SDPR	6	RBP7_VEC
1789	2.45E-08	-0.27767784	0.916	0.904	0.000534705	APP	6	RBP7_VEC
1790	4.05E-18	-0.281726124	0.994	0.992	8.83E-14	RPL11	6	RBP7_VEC
1791	1.94E-13	-0.282428302	0.972	0.975	4.24E-09	RPL18A	6	RBP7_VEC
1792	7.83E-08	-0.283352853	0.101	0.301	0.001708264	CD82	6	RBP7_VEC
1793	5.30E-07	-0.287675752	0.14	0.334	0.011558434	SCPEP1	6	RBP7_VEC
1794	2.41E-15	-0.28874998	0.978	0.98	5.25E-11	RPL18	6	RBP7_VEC
1795	9.80E-07	-0.291783412	0.821	0.859	0.021373988	CD63	6	RBP7_VEC
1796	4.54E-15	-0.295924604	0.966	0.987	9.90E-11	RPL29	6	RBP7_VEC
1797	3.21E-13	-0.302286557	0.95	0.953	6.99E-09	RPL23A	6	RBP7_VEC
1798	2.11E-08	-0.310103536	0.67	0.819	0.000460139	LDB2	6	RBP7_VEC
1799	9.87E-08	-0.311218683	0.201	0.411	0.002152968	PRDX4	6	RBP7_VEC
1800	4.46E-19	-0.312641594	0.994	0.992	9.74E-15	RPS12	6	RBP7_VEC
1801	7.94E-20	-0.313682981	0.983	0.991	1.73E-15	RPL19	6	RBP7_VEC
1802	2.00E-16	-0.313966762	0.966	0.977	4.37E-12	RPL37A	6	RBP7_VEC
1803	2.23E-06	-0.316660383	0.229	0.417	0.048536462	FAM213A	6	RBP7_VEC
1804	1.94E-08	-0.316868737	0.777	0.861	0.000424029	BST2	6	RBP7_VEC
1805	6.71E-14	-0.32717996	0.933	0.955	1.46E-09	RPL17	6	RBP7_VEC
1806	8.61E-08	-0.3272423	0.754	0.848	0.001877562	ALDOA	6	RBP7_VEC
1807	5.54E-07	-0.330242821	0.469	0.629	0.012077765	ERP29	6	RBP7_VEC
1808	3.91E-24	-0.330590119	1	0.997	8.53E-20	RPLP1	6	RBP7_VEC
1809	1.23E-08	-0.332142664	0.201	0.438	0.000269201	PLXDC2	6	RBP7_VEC
1810	1.48E-18	-0.333066216	0.978	0.982	3.22E-14	RPS7	6	RBP7_VEC
1811	6.42E-12	-0.333897565	0.894	0.926	1.40E-07	RPL31	6	RBP7_VEC
1812	2.24E-08	-0.336249184	0.849	0.899	0.000489065	IL6ST	6	RBP7_VEC
1813	7.44E-08	-0.337144856	0.128	0.332	0.001622061	MYRIP	6	RBP7_VEC
1814	1.72E-07	-0.339391626	0.285	0.502	0.003743909	TPP1	6	RBP7_VEC
1815	3.05E-08	-0.343299138	0.626	0.764	0.000664574	ZFAS1	6	RBP7_VEC
1816	1.67E-20	-0.345997823	0.983	0.991	3.64E-16	RPS19	6	RBP7_VEC
1817	8.14E-25	-0.346957051	0.994	0.992	1.78E-20	RPL13	6	RBP7_VEC
1818	2.80E-23	-0.348570296	0.989	0.987	6.11E-19	RPS15	6	RBP7_VEC

1819	1.29E-07	-0.350439318	0.173	0.377	0.002803998	TBC1D15	6	RBP7_VEC
1820	1.07E-13	-0.351210206	0.877	0.922	2.33E-09	RPS5	6	RBP7_VEC
1821	3.47E-07	-0.351981217	0.196	0.397	0.00756152	ALDH6A1	6	RBP7_VEC
1822	1.29E-07	-0.35212354	0.318	0.548	0.002821437	OSTC	6	RBP7_VEC
1823	1.00E-08	-0.353086157	0.134	0.353	0.000218676	CPM	6	RBP7_VEC
1824	2.09E-14	-0.355702208	0.894	0.934	4.55E-10	RPS2	6	RBP7_VEC
1825	3.12E-10	-0.356345712	0.274	0.553	6.80E-06	ACTN1	6	RBP7_VEC
1826	6.16E-08	-0.3597966	0.229	0.436	0.001342338	DEGS1	6	RBP7_VEC
1827	1.63E-08	-0.364667317	0.436	0.658	0.00035555	SERPING1	6	RBP7_VEC
1828	2.60E-22	-0.366081697	0.983	0.989	5.68E-18	RPLP2	6	RBP7_VEC
1829	1.38E-08	-0.366806383	0.631	0.732	0.000300824	ST13	6	RBP7_VEC
1830	4.88E-08	-0.368261001	0.173	0.378	0.001063393	HIBCH	6	RBP7_VEC
1831	6.58E-22	-0.374314511	0.955	0.979	1.43E-17	RPS21	6	RBP7_VEC
1832	6.76E-13	-0.3784613	0.905	0.933	1.47E-08	RPL38	6	RBP7_VEC
1833	7.64E-08	-0.380510732	0.631	0.806	0.00166626	POLR2L	6	RBP7_VEC
1834	8.87E-09	-0.385528829	0.101	0.311	0.000193439	NR5A2	6	RBP7_VEC
1835	5.23E-07	-0.386772307	0.123	0.304	0.011409627	PELO	6	RBP7_VEC
1836	4.16E-17	-0.388242747	0.899	0.951	9.06E-13	RPL35	6	RBP7_VEC
1837	9.31E-11	-0.388281033	0.626	0.778	2.03E-06	MYCT1	6	RBP7_VEC
1838	1.10E-06	-0.388370627	0.358	0.543	0.024060524	RPL22L1	6	RBP7_VEC
1839	1.72E-08	-0.390336683	0.218	0.442	0.000376163	MPST	6	RBP7_VEC
1840	3.16E-11	-0.391040835	0.229	0.51	6.89E-07	IL1R1	6	RBP7_VEC
1841	2.62E-08	-0.393316324	0.52	0.671	0.000570477	LAMP1	6	RBP7_VEC
1842	1.36E-10	-0.394649648	0.207	0.482	2.96E-06	GPM6A	6	RBP7_VEC
1843	2.24E-08	-0.395397624	0.246	0.469	0.000488634	CTSF	6	RBP7_VEC
1844	4.02E-08	-0.396941243	0.307	0.533	0.000876276	LITAF	6	RBP7_VEC
1845	2.86E-07	-0.398140599	0.184	0.381	0.006244866	GLMP	6	RBP7_VEC
1846	1.38E-09	-0.398806005	0.223	0.484	3.01E-05	C11orf96	6	RBP7_VEC
1847	4.52E-07	-0.399078102	0.184	0.36	0.009862204	DAAM1	6	RBP7_VEC
1848	1.33E-06	-0.401816596	0.307	0.484	0.029000778	PROS1	6	RBP7_VEC
1849	5.15E-24	-0.403788412	0.983	0.986	1.12E-19	RPS18	6	RBP7_VEC
1850	1.56E-06	-0.404640279	0.33	0.515	0.033916727	RAPGEF5	6	RBP7_VEC
1851	8.77E-09	-0.406163835	0.603	0.766	0.000191239	S100A13	6	RBP7_VEC
1852	1.90E-08	-0.407257251	0.369	0.592	0.000413857	CUTA	6	RBP7_VEC
1853	2.42E-31	-0.408072114	0.989	0.996	5.27E-27	TPT1	6	RBP7_VEC
1854	1.20E-15	-0.409348377	0.821	0.918	2.62E-11	RPS29	6	RBP7_VEC
1855	4.56E-08	-0.412173872	0.274	0.483	0.00099337	TRAPPC5	6	RBP7_VEC
1856	8.14E-10	-0.412320756	0.117	0.352	1.78E-05	MID1	6	RBP7_VEC
1857	1.28E-09	-0.413801112	0.168	0.413	2.79E-05	C1orf21	6	RBP7_VEC
1858	6.54E-12	-0.414527564	0.682	0.86	1.43E-07	PDLIM1	6	RBP7_VEC
1859	3.62E-09	-0.418140558	0.659	0.769	7.90E-05	PDIA3	6	RBP7_VEC
1860	3.40E-22	-0.4255927	0.955	0.955	7.41E-18	RPS9	6	RBP7_VEC
1861	7.81E-11	-0.426904308	0.19	0.452	1.70E-06	CSF1	6	RBP7_VEC
1862	1.79E-10	-0.428945704	0.76	0.834	3.90E-06	NDUFA4	6	RBP7_VEC
1863	1.21E-08	-0.430156217	0.318	0.543	0.000264847	SMTN	6	RBP7_VEC
1864	4.20E-14	-0.431083134	0.81	0.904	9.16E-10	ATP5G2	6	RBP7_VEC
1865	2.96E-28	-0.432879094	0.966	0.991	6.46E-24	RPL36	6	RBP7_VEC
1866	7.36E-38	-0.433159786	0.994	0.994	1.60E-33	RPS28	6	RBP7_VEC
1867	4.06E-13	-0.438553973	0.788	0.882	8.86E-09	GABARAP	6	RBP7_VEC
1868	7.45E-10	-0.439845373	0.151	0.391	1.63E-05	BLCAP	6	RBP7_VEC
1869	2.99E-10	-0.440897427	0.408	0.678	6.51E-06	GMFG	6	RBP7_VEC
1870	1.05E-07	-0.441268422	0.201	0.394	0.002285715	SLC35A1	6	RBP7_VEC
1871	3.30E-09	-0.441416921	0.38	0.589	7.19E-05	CD164	6	RBP7_VEC
1872	1.55E-11	-0.442713486	0.749	0.816	3.39E-07	PSAP	6	RBP7_VEC
1873	1.59E-29	-0.447830871	0.978	0.991	3.48E-25	RPL37	6	RBP7_VEC
1874	1.62E-12	-0.449164565	0.553	0.745	3.53E-08	CTSB	6	RBP7_VEC
1875	1.19E-10	-0.451681472	0.581	0.728	2.59E-06	PNPLA2	6	RBP7_VEC
1876	9.03E-14	-0.452931736	0.352	0.657	1.97E-09	TGFBR3	6	RBP7_VEC
1877	1.78E-10	-0.453103193	0.52	0.725	3.88E-06	S100A16	6	RBP7_VEC
1878	3.82E-08	-0.457308412	0.341	0.55	0.00083332	UGCG	6	RBP7_VEC
1879	5.68E-10	-0.45877007	0.313	0.562	1.24E-05	GRINA	6	RBP7_VEC
1880	2.06E-07	-0.460198444	0.559	0.698	0.004488928	LPAR6	6	RBP7_VEC
1881	5.95E-09	-0.461436461	0.117	0.328	0.000129826	EIF4EBP1	6	RBP7_VEC
1882	7.00E-10	-0.464424105	0.19	0.427	1.53E-05	AKR1B1	6	RBP7_VEC
1883	1.17E-08	-0.467512402	0.581	0.749	0.000254752	PRSS23	6	RBP7_VEC
1884	2.58E-08	-0.469768681	0.207	0.424	0.000563681	FAM167B	6	RBP7_VEC
1885	1.23E-10	-0.471208059	0.486	0.699	2.68E-06	CYBA	6	RBP7_VEC
1886	1.33E-13	-0.471998853	0.609	0.79	2.90E-09	GYPC	6	RBP7_VEC
1887	1.57E-11	-0.47485097	0.436	0.685	3.42E-07	HEBP1	6	RBP7_VEC
1888	1.20E-08	-0.47547503	0.341	0.536	0.000261106	SEMA6A	6	RBP7_VEC

1889	7.06E-09	-0.480697307	0.296	0.51	0.000154026	DAB2	6	RBP7_VEC
1890	4.57E-11	-0.481517227	0.184	0.451	9.96E-07	CD302	6	RBP7_VEC
1891	2.65E-08	-0.484397522	0.341	0.558	0.00057817	RBP1	6	RBP7_VEC
1892	1.32E-09	-0.486390648	0.201	0.421	2.88E-05	PXDN	6	RBP7_VEC
1893	1.13E-11	-0.487572687	0.62	0.759	2.47E-07	GRN	6	RBP7_VEC
1894	4.14E-08	-0.487825458	0.296	0.509	0.000903352	NRP2	6	RBP7_VEC
1895	6.67E-20	-0.496938715	0.749	0.888	1.45E-15	FXYD5	6	RBP7_VEC
1896	3.46E-09	-0.498641655	0.156	0.373	7.55E-05	TSN	6	RBP7_VEC
1897	1.14E-09	-0.501515418	0.106	0.336	2.48E-05	FAM84A	6	RBP7_VEC
1898	3.00E-41	-0.502027584	0.989	0.995	6.54E-37	RPL28	6	RBP7_VEC
1899	6.37E-08	-0.504650354	0.408	0.601	0.001389846	MYC	6	RBP7_VEC
1900	2.09E-13	-0.517924939	0.279	0.577	4.57E-09	SAT2	6	RBP7_VEC
1901	2.04E-14	-0.51988851	0.57	0.766	4.45E-10	SERP1	6	RBP7_VEC
1902	1.57E-11	-0.520212934	0.285	0.526	3.43E-07	SNX2	6	RBP7_VEC
1903	3.40E-32	-0.520813247	0.961	0.986	7.42E-28	RPL12	6	RBP7_VEC
1904	9.02E-12	-0.522465835	0.263	0.536	1.97E-07	DENND4C	6	RBP7_VEC
1905	6.80E-13	-0.52506624	0.128	0.412	1.48E-08	MYO10	6	RBP7_VEC
1906	1.04E-14	-0.542110448	0.575	0.751	2.26E-10	PDIA6	6	RBP7_VEC
1907	3.37E-19	-0.544645325	0.112	0.481	7.35E-15	NR2F1	6	RBP7_VEC
1908	1.12E-12	-0.555131625	0.117	0.392	2.44E-08	RAB20	6	RBP7_VEC
1909	7.49E-13	-0.557367313	0.196	0.484	1.63E-08	TSPAN6	6	RBP7_VEC
1910	4.86E-13	-0.557756159	0.514	0.707	1.06E-08	CLEC2B	6	RBP7_VEC
1911	4.91E-16	-0.560497908	0.52	0.761	1.07E-11	GSTO1	6	RBP7_VEC
1912	7.74E-13	-0.565592586	0.223	0.498	1.69E-08	TMEM2	6	RBP7_VEC
1913	9.23E-15	-0.567050747	0.743	0.866	2.01E-10	RAMP3	6	RBP7_VEC
1914	1.61E-10	-0.567725855	0.346	0.586	3.51E-06	PLIN2	6	RBP7_VEC
1915	6.09E-24	-0.596367416	0.821	0.917	1.33E-19	PPIB	6	RBP7_VEC
1916	9.33E-18	-0.597556158	0.559	0.783	2.04E-13	PRCP	6	RBP7_VEC
1917	8.48E-15	-0.599405878	0.542	0.737	1.85E-10	PPFIBP1	6	RBP7_VEC
1918	1.50E-14	-0.604245228	0.603	0.778	3.28E-10	CSTB	6	RBP7_VEC
1919	3.21E-18	-0.611618769	0.603	0.784	6.99E-14	CLTA	6	RBP7_VEC
1920	6.04E-13	-0.612569942	0.425	0.648	1.32E-08	CTSA	6	RBP7_VEC
1921	5.92E-14	-0.616875019	0.24	0.534	1.29E-09	NPY1R	6	RBP7_VEC
1922	4.89E-13	-0.617204879	0.112	0.392	1.07E-08	LRG1	6	RBP7_VEC
1923	2.45E-18	-0.618622173	0.464	0.764	5.34E-14	AKAP12	6	RBP7_VEC
1924	2.60E-17	-0.623463067	0.715	0.851	5.67E-13	CALR	6	RBP7_VEC
1925	6.51E-16	-0.635068195	0.402	0.677	1.42E-11	GLO1	6	RBP7_VEC
1926	6.26E-20	-0.641901351	0.754	0.902	1.37E-15	HSP90B1	6	RBP7_VEC
1927	5.30E-19	-0.641902092	0.43	0.75	1.16E-14	HIF1A	6	RBP7_VEC
1928	1.65E-19	-0.657710325	0.564	0.784	3.60E-15	AP2S1	6	RBP7_VEC
1929	6.49E-16	-0.664225338	0.235	0.549	1.41E-11	CCNG1	6	RBP7_VEC
1930	2.22E-18	-0.673126023	0.162	0.515	4.84E-14	ST6GAL1	6	RBP7_VEC
1931	2.81E-16	-0.683798726	0.134	0.454	6.13E-12	MZT2A	6	RBP7_VEC
1932	5.65E-17	-0.691504058	0.251	0.566	1.23E-12	LRRFIP2	6	RBP7_VEC
1933	2.84E-13	-0.691547921	0.156	0.456	6.19E-09	OLFM1	6	RBP7_VEC
1934	1.02E-18	-0.692060107	0.469	0.759	2.23E-14	NAMPT	6	RBP7_VEC
1935	6.49E-14	-0.695713904	0.458	0.698	1.42E-09	PDK4	6	RBP7_VEC
1936	2.62E-20	-0.695791341	0.497	0.739	5.72E-16	TMEM123	6	RBP7_VEC
1937	2.95E-14	-0.701049769	0.128	0.431	6.44E-10	MATN2	6	RBP7_VEC
1938	2.47E-27	-0.703017652	0.665	0.852	5.39E-23	TSPAN7	6	RBP7_VEC
1939	2.02E-19	-0.721561238	0.425	0.717	4.40E-15	SERPINB1	6	RBP7_VEC
1940	1.51E-53	-0.723708748	0.978	0.995	3.29E-49	RPL39	6	RBP7_VEC
1941	9.62E-21	-0.7302821	0.564	0.815	2.10E-16	PLPP3	6	RBP7_VEC
1942	1.58E-20	-0.732852723	0.318	0.665	3.44E-16	NTN4	6	RBP7_VEC
1943	3.03E-31	-0.746606888	0.659	0.875	6.61E-27	NPC2	6	RBP7_VEC
1944	2.31E-25	-0.759386388	0.151	0.607	5.03E-21	MMRN1	6	RBP7_VEC
1945	8.35E-23	-0.791794835	0.123	0.515	1.82E-18	C1QTNF1	6	RBP7_VEC
1946	3.93E-34	-0.802432549	0.497	0.91	8.56E-30	IL33	6	RBP7_VEC
1947	3.33E-22	-0.811207956	0.263	0.659	7.27E-18	SRPX	6	RBP7_VEC
1948	1.07E-19	-0.81831511	0.196	0.565	2.33E-15	BGN	6	RBP7_VEC
1949	2.27E-18	-0.825017169	0.229	0.551	4.96E-14	NID1	6	RBP7_VEC
1950	2.78E-23	-0.838992562	0.218	0.617	6.07E-19	NUPR1	6	RBP7_VEC
1951	1.63E-24	-0.856514542	0.43	0.726	3.55E-20	P4HB	6	RBP7_VEC
1952	5.25E-24	-0.857650092	0.246	0.629	1.14E-19	SNX5	6	RBP7_VEC
1953	3.65E-18	-0.85867055	0.38	0.682	7.96E-14	ADM	6	RBP7_VEC
1954	1.36E-52	-0.861009613	0.972	0.992	2.96E-48	FTL	6	RBP7_VEC
1955	4.42E-25	-0.863981115	0.43	0.805	9.64E-21	LIFR	6	RBP7_VEC
1956	3.06E-28	-0.884408774	0.246	0.665	6.68E-24	F2R	6	RBP7_VEC
1957	3.14E-29	-0.889764925	0.453	0.803	6.84E-25	MARCKSL1	6	RBP7_VEC
1958	6.19E-25	-0.904785426	0.263	0.646	1.35E-20	SLC40A1	6	RBP7_VEC

1959	6.71E-24	-0.920646475	0.268	0.64	1.46E-19	COTL1	6	RBP7_VEC
1960	1.36E-28	-0.928171276	0.279	0.693	2.97E-24	LY96	6	RBP7_VEC
1961	2.88E-42	-0.934437795	0.648	0.886	6.28E-38	FCGRT	6	RBP7_VEC
1962	1.62E-34	-0.947973355	0.179	0.737	3.53E-30	C7	6	RBP7_VEC
1963	3.35E-24	-0.957733282	0.168	0.595	7.30E-20	TFPI2	6	RBP7_VEC
1964	3.75E-09	-0.960720907	0.173	0.403	8.17E-05	CCL2	6	RBP7_VEC
1965	5.75E-16	-1.033940411	0.263	0.585	1.25E-11	CXCL2	6	RBP7_VEC
1966	9.20E-29	-1.053560577	0.268	0.689	2.01E-24	MAF	6	RBP7_VEC
1967	3.43E-56	-1.059618917	0.564	0.965	7.49E-52	TFPI	6	RBP7_VEC
1968	9.47E-34	-1.147146424	0.296	0.723	2.07E-29	ASAH1	6	RBP7_VEC
1969	5.65E-54	-1.211642985	0.508	0.98	1.23E-49	CCL14	6	RBP7_VEC
1970	1.88E-38	-1.279699142	0.447	0.829	4.11E-34	CRHBP	6	RBP7_VEC
1971	2.84E-34	-1.282792695	0.564	0.845	6.19E-30	FCN3	6	RBP7_VEC
1972	9.16E-59	-1.321621588	0.81	0.984	2.00E-54	TIMP1	6	RBP7_VEC
1973	4.68E-41	-1.39481315	0.128	0.66	1.02E-36	CXCL16	6	RBP7_VEC
1974	1.13E-38	-1.487620962	0.201	0.72	2.46E-34	STAB1	6	RBP7_VEC
1975	9.62E-40	-1.538416681	0.179	0.685	2.10E-35	CD4	6	RBP7_VEC
1976	2.48E-48	-1.588404253	0.642	0.909	5.40E-44	SEPP1	6	RBP7_VEC
1977	2.56E-54	-1.711925986	0.151	0.766	5.58E-50	MRC1	6	RBP7_VEC
1978	7.53E-53	-1.724787207	0.447	0.868	1.64E-48	DNASE1L3	6	RBP7_VEC
1979	6.15E-50	-1.82645604	0.33	0.812	1.34E-45	LGMN	6	RBP7_VEC
1980	3.34E-56	-1.868624136	0.486	0.86	7.29E-52	CTSD	6	RBP7_VEC
1981	1.50E-46	-2.223183533	0.564	0.847	3.28E-42	CTSL	6	RBP7_VEC
1982	2.67E-85	1.995264483	0.592	0.027	5.83E-81	PTGDS	7	PTGDS_VEC
1983	6.61E-112	1.905926259	0.837	0.044	1.44E-107	ADGRG6	7	PTGDS_VEC
1984	7.56E-59	1.875905242	0.429	0.02	1.65E-54	POSTN	7	PTGDS_VEC
1985	2.35E-88	1.795976522	0.776	0.051	5.12E-84	TAGLN	7	PTGDS_VEC
1986	1.13E-53	1.75720721	0.633	0.059	2.47E-49	IL1RL1	7	PTGDS_VEC
1987	6.13E-158	1.70372494	0.755	0.017	1.34E-153	RSPO3	7	PTGDS_VEC
1988	6.25E-43	1.655958205	0.898	0.193	1.36E-38	LTC4S	7	PTGDS_VEC
1989	8.10E-40	1.582440949	0.49	0.046	1.77E-35	SELE	7	PTGDS_VEC
1990	6.94E-08	1.555823722	0.388	0.139	0.001513056	CXCL3	7	PTGDS_VEC
1991	5.14E-19	1.517542621	0.816	0.321	1.12E-14	VWF	7	PTGDS_VEC
1992	1.34E-123	1.502188431	0.776	0.03	2.92E-119	SELP	7	PTGDS_VEC
1993	4.29E-08	1.481846708	0.408	0.153	0.000934634	CXCL8	7	PTGDS_VEC
1994	6.73E-25	1.441533538	0.327	0.033	1.47E-20	CXCL1	7	PTGDS_VEC
1995	1.63E-35	1.416567505	0.367	0.028	3.55E-31	CSF3	7	PTGDS_VEC
1996	6.55E-89	1.411746371	0.755	0.045	1.43E-84	INMT	7	PTGDS_VEC
1997	3.79E-65	1.359953026	0.714	0.059	8.27E-61	FBLN2	7	PTGDS_VEC
1998	6.22E-17	1.344368441	0.469	0.103	1.36E-12	CTGF	7	PTGDS_VEC
1999	4.28E-23	1.314360076	1	0.725	9.33E-19	PRSS23	7	PTGDS_VEC
2000	6.06E-39	1.314011783	0.776	0.137	1.32E-34	PKHD1L1	7	PTGDS_VEC
2001	7.74E-25	1.289140957	0.959	0.468	1.69E-20	IL1R1	7	PTGDS_VEC
2002	1.55E-17	1.271617849	0.98	0.672	3.38E-13	C7	7	PTGDS_VEC
2003	1.67E-18	1.245393577	1	0.783	3.65E-14	RNASE1	7	PTGDS_VEC
2004	1.04E-06	1.240966792	0.694	0.479	0.022753243	SOD2	7	PTGDS_VEC
2005	5.14E-58	1.217341097	0.714	0.07	1.12E-53	PTGS1	7	PTGDS_VEC
2006	1.20E-16	1.212341881	0.837	0.383	2.62E-12	PLAC8	7	PTGDS_VEC
2007	5.65E-96	1.133294833	0.694	0.033	1.23E-91	TSHZ2	7	PTGDS_VEC
2008	1.94E-69	1.10754227	0.531	0.026	4.23E-65	ACKR1	7	PTGDS_VEC
2009	6.01E-39	1.087615072	0.714	0.099	1.31E-34	CPE	7	PTGDS_VEC
2010	6.24E-16	1.079080362	0.327	0.052	1.36E-11	IL6	7	PTGDS_VEC
2011	2.75E-08	1.061794603	0.98	0.879	0.000599714	MT2A	7	PTGDS_VEC
2012	2.86E-19	1.052779658	0.408	0.072	6.24E-15	GPRC5A	7	PTGDS_VEC
2013	2.58E-22	1.036944827	0.694	0.178	5.63E-18	DUSP23	7	PTGDS_VEC
2014	7.37E-25	1.016184675	0.898	0.267	1.61E-20	CLU	7	PTGDS_VEC
2015	5.06E-97	0.997975325	0.347	0.003	1.10E-92	LYPD2	7	PTGDS_VEC
2016	6.46E-08	0.993269418	0.796	0.519	0.001409436	ICAM1	7	PTGDS_VEC
2017	1.88E-11	0.983027078	0.286	0.056	4.10E-07	FSTL3	7	PTGDS_VEC
2018	8.91E-11	0.974138625	0.857	0.543	1.94E-06	CXCL2	7	PTGDS_VEC
2019	3.80E-11	0.902929782	0.388	0.101	8.28E-07	ARAP2	7	PTGDS_VEC
2020	9.03E-09	0.893329793	0.673	0.32	0.000196914	NDRG1	7	PTGDS_VEC
2021	1.33E-15	0.892648109	0.531	0.138	2.89E-11	FGL2	7	PTGDS_VEC
2022	1.81E-11	0.860837388	0.571	0.212	3.95E-07	PROCR	7	PTGDS_VEC
2023	2.54E-16	0.860334483	0.49	0.117	5.54E-12	PELI2	7	PTGDS_VEC
2024	1.47E-30	0.853514591	0.531	0.075	3.21E-26	RARRES1	7	PTGDS_VEC
2025	2.27E-40	0.847989956	0.388	0.027	4.94E-36	CFH	7	PTGDS_VEC
2026	1.75E-57	0.843188785	0.469	0.026	3.82E-53	RAB3C	7	PTGDS_VEC
2027	3.57E-38	0.839034744	0.531	0.056	7.79E-34	PTGIS	7	PTGDS_VEC
2028	1.46E-17	0.828323541	0.694	0.215	3.19E-13	FGFR1	7	PTGDS_VEC

2029	3.71E-13	0.793475805	0.571	0.176	8.10E-09	PLAT	7	PTGDS_VEC
2030	4.19E-17	0.787761823	0.429	0.083	9.14E-13	PIR	7	PTGDS_VEC
2031	2.16E-14	0.774339533	1	0.85	4.71E-10	RAMP3	7	PTGDS_VEC
2032	1.13E-15	0.762824151	0.408	0.085	2.47E-11	MAOA	7	PTGDS_VEC
2033	3.87E-33	0.755231224	0.429	0.041	8.43E-29	HLA-DQA1	7	PTGDS_VEC
2034	2.01E-08	0.75023372	0.939	0.762	0.000439409	LIFR	7	PTGDS_VEC
2035	6.72E-09	0.748572734	0.408	0.131	0.000146605	GJA1	7	PTGDS_VEC
2036	2.78E-12	0.737071566	0.857	0.432	6.07E-08	NR2F1	7	PTGDS_VEC
2037	1.66E-12	0.720133967	0.98	0.609	3.63E-08	SRPX	7	PTGDS_VEC
2038	5.16E-08	0.716349975	0.551	0.24	0.001125789	ALPL	7	PTGDS_VEC
2039	1.86E-10	0.711093791	0.837	0.579	4.06E-06	LY6E	7	PTGDS_VEC
2040	1.33E-10	0.710806949	0.571	0.222	2.89E-06	TBPL1	7	PTGDS_VEC
2041	5.34E-12	0.708939841	0.571	0.189	1.17E-07	ACKR3	7	PTGDS_VEC
2042	2.09E-99	0.706048973	0.388	0.005	4.55E-95	LHX6	7	PTGDS_VEC
2043	1.66E-08	0.69949832	0.796	0.475	0.000361548	CEBPD	7	PTGDS_VEC
2044	1.87E-11	0.681571839	0.429	0.116	4.08E-07	HS3ST1	7	PTGDS_VEC
2045	9.01E-09	0.681395112	0.816	0.534	0.000196574	LIMS2	7	PTGDS_VEC
2046	3.21E-09	0.67398844	0.755	0.432	7.00E-05	EMP3	7	PTGDS_VEC
2047	2.81E-10	0.665533153	0.469	0.156	6.12E-06	FOXC1	7	PTGDS_VEC
2048	2.83E-09	0.663528095	0.939	0.74	6.18E-05	PTPRB	7	PTGDS_VEC
2049	4.73E-07	0.659881893	0.633	0.352	0.010323828	CRIM1	7	PTGDS_VEC
2050	1.98E-18	0.656208875	0.367	0.056	4.32E-14	NTS	7	PTGDS_VEC
2051	3.73E-18	0.652221979	0.306	0.04	8.14E-14	TNXB	7	PTGDS_VEC
2052	6.26E-09	0.651275579	0.265	0.061	0.00013657	MPZL2	7	PTGDS_VEC
2053	1.28E-08	0.648705601	0.755	0.446	0.000278238	GPM6A	7	PTGDS_VEC
2054	1.48E-07	0.638174557	0.837	0.553	0.003217148	MMRN1	7	PTGDS_VEC
2055	1.35E-10	0.634988681	0.49	0.164	2.95E-06	IPO5	7	PTGDS_VEC
2056	1.31E-17	0.629161037	0.327	0.048	2.86E-13	EPB41L3	7	PTGDS_VEC
2057	1.02E-36	0.621950515	0.469	0.045	2.22E-32	TMOD1	7	PTGDS_VEC
2058	6.40E-08	0.619763094	0.857	0.571	0.001396246	SH3BP5	7	PTGDS_VEC
2059	1.25E-08	0.613231716	1	0.864	0.000273526	HLA-DRB1	7	PTGDS_VEC
2060	2.04E-08	0.602184056	0.592	0.262	0.000444787	PDLIM3	7	PTGDS_VEC
2061	3.77E-08	0.59423993	0.408	0.142	0.000821572	NRIP1	7	PTGDS_VEC
2062	4.60E-11	0.592605765	0.735	0.337	1.00E-06	SNTB2	7	PTGDS_VEC
2063	1.32E-14	0.590008396	0.469	0.114	2.89E-10	RAB27A	7	PTGDS_VEC
2064	2.62E-42	0.586499925	0.265	0.01	5.72E-38	PTHLH	7	PTGDS_VEC
2065	2.95E-07	0.585515807	0.857	0.588	0.006432455	TAGLN2	7	PTGDS_VEC
2066	9.04E-31	0.583679165	0.306	0.022	1.97E-26	TLL1	7	PTGDS_VEC
2067	1.14E-06	0.582655572	0.306	0.097	0.024898784	LDLRAD3	7	PTGDS_VEC
2068	1.36E-85	0.568914604	0.327	0.004	2.97E-81	F5	7	PTGDS_VEC
2069	9.09E-07	0.562602709	0.408	0.164	0.019813318	UAP1	7	PTGDS_VEC
2070	1.31E-07	0.559981643	0.714	0.404	0.002864342	HIPK2	7	PTGDS_VEC
2071	2.14E-08	0.551091471	0.531	0.202	0.000465821	CD34	7	PTGDS_VEC
2072	1.24E-07	0.54848494	0.694	0.362	0.002696524	CALD1	7	PTGDS_VEC
2073	1.23E-06	0.542178233	0.633	0.351	0.026849694	KRAS	7	PTGDS_VEC
2074	3.22E-17	0.540233641	0.449	0.089	7.02E-13	PGM5	7	PTGDS_VEC
2075	4.72E-08	0.538003823	0.531	0.221	0.001028335	PLCB1	7	PTGDS_VEC
2076	1.85E-06	0.536046741	0.551	0.281	0.040317469	RENBP	7	PTGDS_VEC
2077	6.17E-53	0.535600135	0.286	0.008	1.34E-48	CDH23	7	PTGDS_VEC
2078	9.69E-08	0.52472839	0.898	0.594	0.002112574	S100A6	7	PTGDS_VEC
2079	1.42E-17	0.524288003	0.408	0.07	3.10E-13	CTSC	7	PTGDS_VEC
2080	7.83E-08	0.515672875	0.939	0.742	0.001706679	HLA-DRB5	7	PTGDS_VEC
2081	2.93E-15	0.514528548	0.429	0.094	6.39E-11	RARB	7	PTGDS_VEC
2082	2.39E-08	0.512386015	0.306	0.083	0.000521912	MED24	7	PTGDS_VEC
2083	3.80E-20	0.509783514	0.163	0.009	8.28E-16	CYP26B1	7	PTGDS_VEC
2084	2.62E-15	0.502567296	0.388	0.071	5.71E-11	PLCXD3	7	PTGDS_VEC
2085	8.84E-07	0.495658189	0.796	0.537	0.019285117	CFLAR	7	PTGDS_VEC
2086	1.76E-07	0.494935921	0.327	0.1	0.003847276	SLC29A1	7	PTGDS_VEC
2087	2.17E-06	0.493753507	1	0.911	0.047349227	MT-ND4	7	PTGDS_VEC
2088	2.27E-07	0.493166111	0.612	0.311	0.004950933	PICALM	7	PTGDS_VEC
2089	2.27E-16	0.478290193	0.347	0.056	4.94E-12	WNT2	7	PTGDS_VEC
2090	8.99E-09	0.474558649	0.429	0.145	0.00019601	ST3GAL1	7	PTGDS_VEC
2091	1.79E-06	0.470705938	0.531	0.263	0.039002067	MRPL32	7	PTGDS_VEC
2092	7.17E-22	0.467402203	0.286	0.029	1.56E-17	RDH10	7	PTGDS_VEC
2093	7.28E-07	0.455907014	0.878	0.723	0.015869965	TGM2	7	PTGDS_VEC
2094	7.13E-27	0.455824263	0.265	0.019	1.56E-22	KCNIP4	7	PTGDS_VEC
2095	7.50E-07	0.454188399	1	0.842	0.01634776	NEAT1	7	PTGDS_VEC
2096	4.04E-07	0.454072054	0.98	0.885	0.008803749	ENG	7	PTGDS_VEC
2097	9.70E-102	0.451348974	0.265	0	2.11E-97	RP11-384F7.2	7	PTGDS_VEC
2098	1.50E-16	0.450538267	0.163	0.012	3.28E-12	VCAN	7	PTGDS_VEC

2099	1.44E-06	0.433865146	0.98	0.815	0.031333474	IFITM1	7	PTGDS_VEC
2100	8.36E-08	0.43383422	0.449	0.165	0.00182208	AEBP1	7	PTGDS_VEC
2101	2.44E-08	0.433319372	0.245	0.056	0.000532974	PPP1R13L	7	PTGDS_VEC
2102	1.57E-06	0.432980678	0.245	0.068	0.034265041	ITGA10	7	PTGDS_VEC
2103	1.40E-06	0.43197429	0.531	0.24	0.030604137	ERRFI1	7	PTGDS_VEC
2104	5.47E-17	0.430984909	0.286	0.037	1.19E-12	RASA4	7	PTGDS_VEC
2105	4.21E-27	0.424405785	0.143	0.004	9.18E-23	EFEMP1	7	PTGDS_VEC
2106	2.41E-15	0.413982642	0.265	0.036	5.26E-11	RND3	7	PTGDS_VEC
2107	9.27E-07	0.413446015	0.245	0.066	0.020223827	ST8SIA6	7	PTGDS_VEC
2108	1.43E-07	0.411916195	1	0.906	0.003111725	IGFBP7	7	PTGDS_VEC
2109	9.98E-08	0.410980401	0.673	0.331	0.002176218	C2orf88	7	PTGDS_VEC
2110	9.28E-45	0.402194294	0.184	0.003	2.02E-40	OMD	7	PTGDS_VEC
2111	9.85E-07	0.401888178	0.857	0.515	0.021478432	ACTN1	7	PTGDS_VEC
2112	5.02E-11	0.397939342	0.224	0.036	1.09E-06	CLDN11	7	PTGDS_VEC
2113	1.27E-07	0.396487891	0.327	0.099	0.002759376	AMOTL2	7	PTGDS_VEC
2114	4.07E-12	0.395788289	0.204	0.027	8.88E-08	RP11-138A9.1	7	PTGDS_VEC
2115	3.32E-07	0.391245098	0.347	0.111	0.007240327	DLL1	7	PTGDS_VEC
2116	1.18E-06	0.383071347	1	0.896	0.025729838	MT-ND1	7	PTGDS_VEC
2117	3.59E-15	0.380730896	0.184	0.017	7.83E-11	PLA1A	7	PTGDS_VEC
2118	6.18E-16	0.379290433	0.347	0.056	1.35E-11	CCDC69	7	PTGDS_VEC
2119	5.53E-07	0.376079928	0.224	0.055	0.012050861	TLE1	7	PTGDS_VEC
2120	4.09E-08	0.375193146	0.429	0.147	0.000893009	YAF2	7	PTGDS_VEC
2121	2.41E-07	0.366024704	0.327	0.098	0.005250618	HTRA1	7	PTGDS_VEC
2122	1.65E-07	0.364161204	0.204	0.044	0.003605083	KLHL21	7	PTGDS_VEC
2123	4.53E-08	0.36394072	0.204	0.04	0.000988456	ANXA3	7	PTGDS_VEC
2124	8.52E-13	0.362086921	0.184	0.021	1.86E-08	SYT15	7	PTGDS_VEC
2125	2.15E-07	0.361348958	0.204	0.044	0.004688616	NAT8	7	PTGDS_VEC
2126	1.39E-07	0.358521605	0.388	0.128	0.003023599	SIK2	7	PTGDS_VEC
2127	8.12E-09	0.344254666	0.184	0.031	0.000177126	TPBG	7	PTGDS_VEC
2128	1.22E-14	0.323600287	0.143	0.01	2.65E-10	COL3A1	7	PTGDS_VEC
2129	1.94E-06	0.322899804	0.98	0.956	0.042384142	HLA-E	7	PTGDS_VEC
2130	2.78E-13	0.303459629	0.224	0.03	6.06E-09	NEDD4L	7	PTGDS_VEC
2131	1.52E-06	0.299448709	0.184	0.04	0.033189564	SGMS2	7	PTGDS_VEC
2132	1.62E-06	0.294486053	0.306	0.1	0.035229162	NAA15	7	PTGDS_VEC
2133	4.16E-13	0.290154961	0.224	0.03	9.06E-09	IRX3	7	PTGDS_VEC
2134	1.86E-06	0.28781613	0.143	0.027	0.040522479	PEX11A	7	PTGDS_VEC
2135	3.99E-08	0.278920411	0.204	0.04	0.000869132	EXOC3	7	PTGDS_VEC
2136	2.22E-16	0.278637221	0.163	0.012	4.84E-12	SSPN	7	PTGDS_VEC
2137	3.36E-08	0.273061605	0.163	0.026	0.000733392	ARHGEF10	7	PTGDS_VEC
2138	3.25E-15	0.270500727	0.102	0.005	7.09E-11	SULT1C2	7	PTGDS_VEC
2139	1.60E-06	0.267909525	0.245	0.066	0.034900785	CCDC151	7	PTGDS_VEC
2140	4.82E-13	0.266163833	0.122	0.009	1.05E-08	NTRK2	7	PTGDS_VEC
2141	9.19E-48	0.264298413	0.122	0	2.00E-43	LSAMP	7	PTGDS_VEC
2142	6.72E-13	0.257140635	0.224	0.03	1.47E-08	CHST2	7	PTGDS_VEC
2143	1.92E-06	-0.269037047	1	0.994	0.041773532	RPS28	7	PTGDS_VEC
2144	2.40E-07	-0.317872673	1	0.994	0.005223346	RPL28	7	PTGDS_VEC
2145	2.44E-07	-0.355083787	0.98	0.982	0.005323023	RPS7	7	PTGDS_VEC
2146	1.04E-06	-0.532114418	0.816	0.87	0.022578057	IL33	7	PTGDS_VEC
2147	5.44E-07	-0.654202102	0.735	0.805	0.011868245	LDB2	7	PTGDS_VEC
2148	4.53E-09	-0.657914908	0.878	0.908	9.88E-05	PPIB	7	PTGDS_VEC
2149	1.13E-08	-0.695177643	0.918	0.914	0.000246356	ACTG1	7	PTGDS_VEC
2150	1.36E-07	-0.753119727	0.755	0.839	0.002976384	CALR	7	PTGDS_VEC
2151	2.08E-07	-0.760785619	0.49	0.721	0.00453262	TMEM123	7	PTGDS_VEC
2152	2.19E-11	-0.781051156	0.837	0.919	4.78E-07	GNG11	7	PTGDS_VEC
2153	5.96E-08	-0.81654324	0.49	0.733	0.001299997	CTSB	7	PTGDS_VEC
2154	3.77E-08	-0.822728848	0.347	0.704	0.000821972	MEF2C	7	PTGDS_VEC
2155	4.45E-09	-0.847639223	0.347	0.697	9.71E-05	ADGRF5	7	PTGDS_VEC
2156	2.88E-07	-0.852347694	0.163	0.523	0.006275166	EFNB2	7	PTGDS_VEC
2157	7.65E-07	-0.873982086	0.551	0.695	0.016691881	ADGRL4	7	PTGDS_VEC
2158	7.57E-12	-0.912292581	0.959	0.932	1.65E-07	CCL14	7	PTGDS_VEC
2159	1.16E-08	-0.980030377	0.143	0.552	0.000252931	EFNB1	7	PTGDS_VEC
2160	3.11E-08	-1.108452631	0.102	0.491	0.000677316	MEG3	7	PTGDS_VEC
2161	1.14E-07	-1.112868468	0.816	0.884	0.002483159	SEPP1	7	PTGDS_VEC
2162	4.70E-07	-1.128294569	0.286	0.623	0.010257782	CLEC1B	7	PTGDS_VEC
2163	1.63E-12	-1.20105067	0.327	0.782	3.56E-08	A2M	7	PTGDS_VEC
2164	1.45E-09	-1.259592318	0.224	0.662	3.17E-05	OIT3	7	PTGDS_VEC
2165	2.37E-13	-1.293151302	0.633	0.824	5.17E-09	SPARC	7	PTGDS_VEC
2166	2.00E-10	-1.31366294	0.592	0.823	4.36E-06	FCN3	7	PTGDS_VEC
2167	3.85E-08	-1.326971677	0.449	0.698	0.000840367	FCN2	7	PTGDS_VEC
2168	2.05E-06	-1.384546029	0.265	0.562	0.044613885	CD14	7	PTGDS_VEC

2169	1.86E-09	-1.472753718	0.633	0.766	4.07E-05	LGMN	7	PTGDS_VEC
2170	2.69E-19	-2.061114377	0.102	0.779	5.87E-15	CLDN5	7	PTGDS_VEC
2171	4.02E-14	-2.491502777	0.102	0.668	8.77E-10	CLEC4G	7	PTGDS_VEC

Table 11: List of DEGs of endothelial cell clusters in reperfusion stage (PR versus EP)

List of differentially expressed genes of CSTL_LSEC in reperfusion stage (PR versus EP)					
gene	p_val	avg_logFC	pct.1	pct.2	p_val_adj
HSPD1	2.74E-36	1.962726822	0.983	0.538	5.97E-32
HSPE1	3.43E-35	1.775331272	0.974	0.515	7.49E-31
EMP1	2.70E-34	1.693351743	0.906	0.22	5.89E-30
TM4SF1	1.54E-33	1.133219786	1	0.992	3.35E-29
LITAF	1.05E-31	1.37629882	0.983	0.47	2.29E-27
HSP90AA1	1.27E-31	1.262562393	1	0.932	2.78E-27
PNP	1.41E-29	1.34006839	0.889	0.273	3.08E-25
HES1	1.97E-29	-1.337649092	0.239	0.909	4.30E-25
ID3	7.27E-28	-1.328064276	0.573	0.962	1.59E-23
PLAUR	4.02E-27	1.268989891	0.795	0.152	8.76E-23
BAG3	2.13E-26	1.370266721	0.915	0.364	4.64E-22
HSPB1	2.14E-26	1.435604091	0.991	0.886	4.66E-22
AKAP12	1.67E-25	0.961758866	1	0.985	3.64E-21
MIR4435-2HG	2.83E-25	1.193280862	0.829	0.242	6.17E-21
FOSL1	5.97E-25	0.919575064	0.641	0.023	1.30E-20
HSP90AB1	1.31E-24	0.85308268	1	0.939	2.85E-20
PHLDA1	1.62E-24	1.057124249	0.932	0.515	3.52E-20
PMP22	2.88E-24	1.165833499	0.957	0.606	6.28E-20
ADAMTS9	5.22E-24	1.06199865	0.684	0.061	1.14E-19
DDX21	1.84E-23	1.054318536	0.863	0.439	4.01E-19
ZFP36L1	4.84E-23	-0.932942692	0.701	0.955	1.06E-18
TIMP1	5.06E-23	0.703872436	1	1	1.10E-18
ADAMTS4	5.75E-23	1.157588121	0.872	0.311	1.25E-18
LMNA	7.57E-23	1.045126745	0.889	0.402	1.65E-18
S1PR1	1.25E-22	0.879108252	0.966	0.689	2.72E-18
HSPA5	3.79E-22	1.431255166	0.991	0.879	8.27E-18
LINC00152	9.04E-22	1.060410644	0.761	0.182	1.97E-17
HSPH1	1.01E-21	1.381338819	0.855	0.439	2.20E-17
SAT1	1.40E-21	0.839762364	1	0.977	3.06E-17
MESDC1	8.21E-21	0.933478537	0.718	0.167	1.79E-16
MYADM	1.27E-20	0.963686991	0.906	0.462	2.78E-16
PDLIM3	1.16E-19	0.922175329	0.726	0.189	2.52E-15
SELK	1.46E-19	1.044609572	0.923	0.576	3.17E-15
PIM3	2.50E-19	-0.853919725	0.205	0.75	5.45E-15
CNKSR3	2.72E-19	0.845941476	0.778	0.242	5.93E-15
HSPA1A	4.86E-19	0.884574989	1	0.886	1.06E-14
LDLR	5.91E-19	0.89846384	0.735	0.22	1.29E-14
B4GALT5	8.33E-19	0.815841904	0.752	0.227	1.82E-14
HSPA1B	1.02E-18	1.0166313	0.966	0.78	2.22E-14
PLIN2	1.33E-18	0.853575233	0.974	0.773	2.91E-14
THBD	1.68E-18	0.993813772	0.915	0.523	3.66E-14
NUAK1	1.69E-18	-0.780555274	0.214	0.75	3.69E-14
MYC	2.14E-18	0.938838678	0.949	0.667	4.66E-14
SNHG15	2.23E-18	0.814515589	0.624	0.106	4.87E-14
EVA1C	8.49E-18	0.854651785	0.778	0.273	1.85E-13
DUSP6	1.53E-17	0.885673874	0.974	0.72	3.34E-13
CLIC1	1.81E-17	0.55253955	0.983	0.955	3.94E-13
PLPP3	1.98E-17	0.605466685	1	0.977	4.32E-13
ARID5A	4.29E-17	0.71658987	0.556	0.076	9.35E-13
PDLIM1	4.44E-17	0.601283738	0.983	0.97	9.69E-13
LPAR6	7.73E-17	-0.717598203	0.769	0.962	1.69E-12
ATP13A3	1.09E-16	0.752291566	0.598	0.106	2.39E-12
TSC22D4	1.15E-16	-0.767382857	0.111	0.591	2.51E-12
CYCS	1.44E-16	0.750386377	0.769	0.326	3.14E-12
MYL6	1.89E-16	0.473675583	1	0.992	4.13E-12
TSC22D3	1.91E-16	-0.80720399	0.65	0.886	4.15E-12
TRIB1	1.96E-16	0.757448283	0.803	0.28	4.27E-12
ICAM1	2.80E-16	1.135841798	0.94	0.742	6.11E-12
MCAM	3.13E-16	0.807694232	0.692	0.227	6.83E-12
SDF2L1	8.13E-16	0.911333778	0.786	0.348	1.77E-11
KLF6	9.53E-16	0.76558372	0.966	0.826	2.08E-11
PTPN1	1.25E-15	0.768029789	0.846	0.439	2.73E-11
GPR4	1.28E-15	0.709382841	0.59	0.121	2.79E-11
CDC37	2.95E-15	0.606488091	1	0.848	6.43E-11
CD9	3.32E-15	0.893949205	0.974	0.833	7.24E-11
UGCG	3.94E-15	0.780717135	0.932	0.682	8.60E-11
MCL1	6.01E-15	0.639908546	0.974	0.826	1.31E-10

DDIT4	6.24E-15	-0.903266506	0.632	0.924	1.36E-10
CD59	6.67E-15	0.531264342	0.991	0.962	1.46E-10
ZNF593	8.08E-15	0.711588064	0.547	0.098	1.76E-10
IL32	8.20E-15	1.095380061	0.65	0.212	1.79E-10
NOP16	1.02E-14	0.675060205	0.487	0.061	2.22E-10
PVRL2	1.15E-14	0.596964843	0.94	0.795	2.52E-10
HSPA8	1.18E-14	0.598463082	0.991	0.977	2.57E-10
RIN2	1.20E-14	0.819708939	0.718	0.303	2.62E-10
TNFRSF12A	1.36E-14	0.684425228	0.376	0	2.98E-10
SLC20A1	1.50E-14	0.808087577	0.624	0.182	3.28E-10
SOX17	1.59E-14	0.952209445	0.769	0.379	3.48E-10
HNRNPAB	2.68E-14	0.6487935	0.615	0.159	5.84E-10
SPHK1	2.89E-14	0.661179063	0.41	0.023	6.31E-10
MARCKS	3.17E-14	0.579646057	0.949	0.682	6.91E-10
ACER3	3.53E-14	0.773411275	0.709	0.288	7.70E-10
HLA-DRB5	4.04E-14	0.728985127	0.94	0.712	8.80E-10
ZYX	4.25E-14	0.651822306	0.855	0.439	9.27E-10
EFNA1	4.57E-14	-0.709199307	0.598	0.909	9.96E-10
SH3BGRL3	1.11E-13	0.578350047	0.949	0.902	2.42E-09
SBNO2	1.48E-13	0.560899979	0.59	0.152	3.23E-09
RELN	1.85E-13	-0.650073313	0.778	0.985	4.03E-09
EIF4A1	3.16E-13	0.601145441	0.88	0.53	6.89E-09
RHOC	3.35E-13	0.516599602	0.966	0.879	7.31E-09
STIP1	4.03E-13	0.54270188	0.564	0.129	8.78E-09
GIMAP8	4.33E-13	-0.641671185	0.239	0.674	9.45E-09
BAZ1A	4.60E-13	0.686257455	0.667	0.227	1.00E-08
EIF5A	5.54E-13	0.696102399	0.778	0.485	1.21E-08
NME1	5.93E-13	0.494737333	0.41	0.038	1.29E-08
BZW1	6.08E-13	0.61691669	0.829	0.545	1.33E-08
CDC42EP2	6.73E-13	0.712957861	0.607	0.22	1.47E-08
OAF	7.37E-13	0.474798341	0.333	0	1.61E-08
NAMPT	7.73E-13	0.525398991	0.966	0.871	1.69E-08
POMP	7.77E-13	0.551960506	0.966	0.848	1.69E-08
SERPINB1	8.63E-13	0.79180883	0.957	0.78	1.88E-08
DNASE1L3	1.47E-12	-0.329746847	1	1	3.20E-08
EHD4	1.48E-12	0.603827974	0.684	0.265	3.22E-08
PDK4	2.19E-12	-0.61775627	0.761	0.932	4.77E-08
PODXL	2.21E-12	0.611557023	0.47	0.076	4.83E-08
STARD4	2.23E-12	0.582198505	0.453	0.076	4.87E-08
TNFRSF1A	2.74E-12	0.560759866	0.966	0.795	5.97E-08
IGFBP4	2.76E-12	-0.422063936	1	0.992	6.03E-08
PLK3	2.83E-12	0.684941231	0.581	0.189	6.18E-08
LRRC32	3.09E-12	0.708058532	0.632	0.258	6.74E-08
SEPP1	3.13E-12	-0.425593103	1	1	6.83E-08
ENG	3.34E-12	-0.482112733	1	1	7.28E-08
RBM3	3.49E-12	0.540320828	0.932	0.833	7.61E-08
HSPG2	3.50E-12	-0.57197435	0.855	0.947	7.62E-08
SOCS2	3.61E-12	0.626942262	0.88	0.598	7.88E-08
TOMM5	3.80E-12	0.613309651	0.821	0.477	8.28E-08
MT2A	4.35E-12	1.115533517	0.974	0.924	9.48E-08
ADAMTS1	4.91E-12	0.819801175	0.838	0.477	1.07E-07
VIMP	5.14E-12	0.673048227	0.829	0.568	1.12E-07
INPP1	5.20E-12	0.609153065	0.803	0.515	1.13E-07
JUNB	5.26E-12	-0.610317691	0.957	0.992	1.15E-07
PEA15	5.69E-12	0.623088996	0.735	0.348	1.24E-07
CD4	5.70E-12	-0.439234616	0.983	1	1.24E-07
SPRY4	5.71E-12	0.647478056	0.726	0.318	1.24E-07
KDM6B	5.77E-12	0.709475343	0.624	0.242	1.26E-07
NOTCH4	7.29E-12	-0.557008613	0.487	0.856	1.59E-07
UPP1	7.84E-12	0.63153563	0.923	0.735	1.71E-07
SDPR	8.15E-12	-0.488036876	0.974	0.992	1.78E-07
MAT2A	8.66E-12	0.636648168	0.675	0.288	1.89E-07
CLEC4G	8.88E-12	-0.287972513	1	1	1.94E-07
F8	1.05E-11	-0.513758892	0.88	0.992	2.29E-07
STAB1	1.30E-11	-0.561500556	0.966	1	2.83E-07
UBE2D3	1.41E-11	0.477750227	0.983	0.909	3.07E-07
TOP1	1.78E-11	0.610301503	0.803	0.515	3.87E-07
RHOB	1.89E-11	-0.710447343	0.803	0.955	4.12E-07
OIT3	2.24E-11	-0.391269821	1	1	4.88E-07
DNAJB6	2.63E-11	0.720478291	0.821	0.545	5.73E-07

CTSD	2.81E-11	-0.382317978	1	1	6.14E-07
INSIG1	2.87E-11	0.773104537	0.667	0.333	6.27E-07
GIMAP1	3.56E-11	-0.589587143	0.444	0.78	7.75E-07
TFPI2	4.63E-11	0.877028502	0.906	0.811	1.01E-06
SLC44A2	5.28E-11	-0.523070984	0.547	0.871	1.15E-06
HYAL2	5.75E-11	0.630044557	0.957	0.848	1.25E-06
NOP58	6.81E-11	0.592147157	0.675	0.318	1.49E-06
IFITM3	7.53E-11	0.3427865	1	1	1.64E-06
ARL4D	8.08E-11	-0.525753449	0.085	0.447	1.76E-06
ACKR3	1.10E-10	0.644667123	0.496	0.121	2.39E-06
RAP1B	1.11E-10	0.532366336	0.897	0.788	2.41E-06
ANGPTL4	1.15E-10	0.874962303	0.607	0.235	2.51E-06
NR2F2	1.15E-10	-0.615418235	0.573	0.841	2.51E-06
NOLC1	1.16E-10	0.583056387	0.675	0.318	2.52E-06
PDLIM5	1.19E-10	0.742503784	0.701	0.409	2.59E-06
RTEL1-TNFRSF6B	1.24E-10	0.72874358	0.325	0.023	2.71E-06
ARPC5L	1.29E-10	0.528935755	0.778	0.424	2.82E-06
RPS29	1.36E-10	0.369133877	0.991	1	2.98E-06
XBP1	1.63E-10	0.511693348	0.889	0.591	3.56E-06
FOSL2	1.73E-10	0.572966051	0.641	0.273	3.77E-06
BCL2L1	1.81E-10	0.588796244	0.59	0.22	3.94E-06
ID2	2.07E-10	-0.623576659	0.581	0.818	4.52E-06
TMSB10	2.14E-10	0.371678245	1	1	4.66E-06
PHLDB1	2.42E-10	0.436593821	0.41	0.068	5.29E-06
UAP1	2.87E-10	0.478251485	0.436	0.106	6.25E-06
YWHAG	3.54E-10	0.51236489	0.564	0.205	7.72E-06
HLA-DRB1	4.58E-10	0.472817683	1	0.97	9.98E-06
TEAD4	4.97E-10	0.596133249	0.632	0.295	1.08E-05
SRSF5	5.12E-10	0.557277883	0.94	0.795	1.12E-05
SRGN	5.14E-10	0.585956698	0.983	0.871	1.12E-05
PELO	5.31E-10	0.568459923	0.744	0.409	1.16E-05
SOD2	5.43E-10	0.85299783	0.821	0.553	1.18E-05
RAN	6.00E-10	0.630466236	0.761	0.598	1.31E-05
MT1M	6.08E-10	0.696171747	0.35	0.045	1.33E-05
BCL6B	6.09E-10	0.578451395	0.521	0.167	1.33E-05
TMEM37	7.04E-10	-0.569157747	0.598	0.826	1.54E-05
RASSF1	7.06E-10	0.519330987	0.607	0.258	1.54E-05
ZFP36	7.24E-10	-0.564070237	0.957	1	1.58E-05
CCT6A	8.04E-10	0.4745195	0.726	0.371	1.75E-05
NCL	9.62E-10	0.559215814	0.889	0.75	2.10E-05
IL1RL1	9.63E-10	0.989346274	0.299	0.023	2.10E-05
H3F3B	1.03E-09	0.409440505	1	0.977	2.24E-05
PRCP	1.19E-09	-0.398929205	0.966	0.992	2.60E-05
DHCR24	1.25E-09	-0.49675896	0.393	0.742	2.73E-05
TIMM17A	1.39E-09	0.52656621	0.547	0.197	3.04E-05
WDR43	1.41E-09	0.50152974	0.376	0.068	3.07E-05
TUBA1C	1.83E-09	0.592590213	0.675	0.333	4.00E-05
EIF3I	1.86E-09	0.443357327	0.872	0.636	4.06E-05
YBX1	1.97E-09	0.411530281	0.957	0.886	4.30E-05
STAB2	2.12E-09	-0.519251563	0.829	0.977	4.62E-05
DNAJB11	2.14E-09	0.856375666	0.692	0.455	4.66E-05
AHSA1	2.17E-09	0.537991816	0.504	0.167	4.74E-05
MT-ATP6	2.32E-09	-0.390936373	1	1	5.06E-05
EGR1	2.50E-09	-0.646176378	0.718	0.879	5.46E-05
STX12	2.56E-09	0.508591795	0.675	0.303	5.59E-05
PLSCR1	2.67E-09	0.420228843	0.915	0.773	5.82E-05
ILF2	2.71E-09	0.513521232	0.675	0.371	5.91E-05
MALL	3.04E-09	0.597963804	0.35	0.061	6.64E-05
STX11	3.05E-09	0.439944069	0.342	0.053	6.65E-05
CDKN1B	3.11E-09	-0.531004745	0.35	0.652	6.77E-05
GIMAP7	3.30E-09	-0.563241233	0.359	0.697	7.21E-05
MT1X	3.86E-09	0.803629421	0.684	0.333	8.42E-05
SH3TC1	3.95E-09	-0.58965162	0.368	0.682	8.62E-05
ARHGAP23	4.08E-09	0.468331873	0.419	0.106	8.89E-05
MAF	4.24E-09	-0.457732937	0.855	0.962	9.25E-05
SNX5	4.39E-09	-0.447134842	0.846	0.955	9.57E-05
INSR	4.60E-09	0.544040619	0.65	0.326	0.000100241
SFPQ	4.62E-09	0.49540753	0.88	0.606	0.000100811
SLC12A7	5.31E-09	0.605842466	0.59	0.258	0.000115843
SNRPB	5.68E-09	0.452531595	0.838	0.545	0.000123798

CHKA	5.80E-09	-0.404725046	0.145	0.5	0.000126504
SOX18	5.92E-09	-0.503148572	0.154	0.47	0.000129128
SLC9A3R2	6.02E-09	0.793567795	0.744	0.47	0.000131278
ID1	6.19E-09	-0.501293555	0.872	0.955	0.000134984
FAM102A	6.66E-09	-0.541344436	0.402	0.705	0.000145313
BLCAP	6.94E-09	-0.480102042	0.41	0.712	0.000151454
ENO1	6.95E-09	0.46688251	0.821	0.576	0.000151621
DDIT3	7.53E-09	-0.604492881	0.376	0.644	0.000164127
RAMP3	8.48E-09	-0.359245353	1	1	0.000184869
IQCJ-SCHIP1	8.67E-09	0.371668671	0.282	0.023	0.000188968
PHC2	8.74E-09	0.405280854	0.496	0.152	0.000190663
PSAP	8.75E-09	-0.31796334	0.991	1	0.000190748
INHBB	1.01E-08	0.455979337	0.333	0.053	0.000220547
DDX5	1.09E-08	0.38246914	0.991	0.955	0.00023846
AKAP2	1.11E-08	0.463310199	0.59	0.242	0.000241608
SNRPD1	1.15E-08	0.481424564	0.624	0.326	0.000250326
GRN	1.20E-08	-0.333411861	0.974	0.977	0.000261789
MT-CYB	1.22E-08	-0.339366978	1	1	0.000265941
RAB20	1.32E-08	-0.535658086	0.444	0.727	0.000287573
VCAM1	1.33E-08	0.724319215	0.444	0.136	0.000288965
AP2M1	1.36E-08	-0.401035546	0.786	0.932	0.000296075
GIMAP5	1.40E-08	-0.47692217	0.538	0.826	0.000304308
MTSS1	1.43E-08	0.571532806	0.684	0.386	0.000312253
CD151	1.51E-08	-0.398467545	0.906	0.939	0.000329472
GIMAP4	1.55E-08	-0.484483874	0.641	0.871	0.000339113
TNIP2	1.57E-08	0.511689222	0.504	0.189	0.000341955
NOS3	1.60E-08	0.561846736	0.59	0.265	0.000348072
CIRBP	1.61E-08	-0.365585215	0.897	0.955	0.000350786
STOM	1.68E-08	0.392908607	0.983	0.947	0.000365579
WARS	1.72E-08	0.528803476	0.812	0.561	0.000375461
SLCO4A1	1.80E-08	0.516917531	0.761	0.47	0.00039357
SSH1	2.00E-08	0.523147518	0.479	0.167	0.000436246
BRIX1	2.13E-08	0.353960269	0.308	0.045	0.000463619
CREM	2.14E-08	0.599515251	0.487	0.174	0.000467597
PPRC1	2.18E-08	0.42246923	0.359	0.076	0.000476454
IVNS1ABP	2.48E-08	0.481222589	0.718	0.424	0.000541402
PNO1	2.71E-08	0.354137125	0.333	0.053	0.000589938
PGK1	2.91E-08	0.489517103	0.855	0.583	0.000634827
SERPINB9	2.95E-08	0.512069168	0.658	0.364	0.000642306
DLC1	3.21E-08	0.456224524	0.983	0.886	0.000700359
GRWD1	3.38E-08	0.268793519	0.231	0.008	0.000737636
GRPEL1	3.38E-08	0.453127945	0.47	0.152	0.00073803
LIMK2	3.76E-08	0.535202715	0.504	0.197	0.000820372
DUSP1	3.77E-08	-0.394192686	0.991	0.985	0.000822169
EIF4A2	3.77E-08	-0.474585861	0.624	0.879	0.000823009
SPARC	3.85E-08	-0.33756764	0.974	1	0.000838769
PNPLA8	4.11E-08	0.488566414	0.641	0.341	0.000895556
TUBB6	4.14E-08	0.425733495	0.615	0.273	0.000903331
GABARAP	4.25E-08	-0.316800225	0.974	0.985	0.000926704
DEFA3	4.29E-08	-0.401905974	0.068	0.356	0.000936331
KPNA4	4.50E-08	0.445795407	0.47	0.167	0.000981178
SNRPG	4.53E-08	0.430093982	0.786	0.462	0.000986875
BACH1	4.64E-08	0.515975102	0.564	0.265	0.001012905
MIR222HG	4.82E-08	0.430996856	0.359	0.076	0.001051659
MTHFD2	4.90E-08	0.438881006	0.376	0.098	0.001068492
GJA1	4.98E-08	0.375389323	0.205	0	0.001086205
DOT1L	5.15E-08	0.332202507	0.239	0.015	0.001123903
AC017002.2	5.37E-08	0.460088072	0.436	0.121	0.001170285
GJA4	5.37E-08	0.885451404	0.667	0.379	0.001171788
NUDC	6.59E-08	0.443438015	0.692	0.417	0.001436909
HSPA9	7.04E-08	0.497118828	0.53	0.212	0.001534779
SLC40A1	7.17E-08	-0.40458209	0.863	0.955	0.001564191
PISD	7.49E-08	0.33078947	0.291	0.045	0.001632927
CXCL8	7.58E-08	0.801656893	0.444	0.159	0.001652895
LUCAT1	7.97E-08	0.409705059	0.248	0.023	0.001737614
C7	7.98E-08	-0.536095536	0.701	0.879	0.001741122
DNMBP	8.66E-08	0.446137783	0.479	0.174	0.001889294
SMAD6	8.77E-08	-0.448431484	0.06	0.326	0.001912529
TCEB1	9.31E-08	0.464232697	0.88	0.712	0.002030701
TMEM51	1.21E-07	0.362856011	0.325	0.061	0.002649334

NDRG1	1.27E-07	0.495801508	0.496	0.182	0.002761718
PML	1.30E-07	-0.412264931	0.197	0.508	0.00282478
MEIS2	1.30E-07	-0.432655251	0.419	0.75	0.002840351
PTGES3	1.42E-07	0.461917576	0.906	0.856	0.003097305
SF1	1.45E-07	0.458769408	0.863	0.705	0.003172424
GNL3	1.56E-07	0.400748407	0.359	0.098	0.00340432
TSSC4	1.58E-07	0.4547504	0.462	0.167	0.003446415
RASD1	1.59E-07	1.022117776	0.778	0.568	0.003475746
DNAJA4	1.71E-07	0.379917607	0.308	0.061	0.003735124
CEBPZ	1.81E-07	0.411995631	0.624	0.311	0.003954014
PFDN2	1.99E-07	0.527831768	0.735	0.508	0.004339293
TMEM140	2.07E-07	-0.487086104	0.333	0.598	0.00452359
FLT1	2.16E-07	0.426432325	0.966	0.879	0.004703187
SLC4A7	2.30E-07	0.340244099	0.222	0.015	0.005016819
ANKRD28	2.31E-07	0.399213218	0.359	0.083	0.005036116
NFKB2	2.39E-07	0.441542415	0.35	0.091	0.005204808
ACTB	2.42E-07	0.336543973	1	1	0.005280334
RELB	2.45E-07	0.428645607	0.402	0.129	0.005350639
RBM17	2.50E-07	0.469273294	0.65	0.402	0.005453317
CLEC4M	2.62E-07	-0.336713864	1	0.992	0.00572004
RCN1	2.69E-07	0.407872897	0.718	0.447	0.005866993
ARID5B	2.81E-07	0.434463206	0.487	0.189	0.006121348
RHEB	2.94E-07	0.396167347	0.726	0.455	0.006404291
PIK3C2A	3.19E-07	0.437784061	0.752	0.485	0.006963128
AQP3	3.38E-07	0.574199794	0.496	0.242	0.007361539
C10orf10	3.43E-07	-0.444452846	0.077	0.348	0.007474145
NIP7	3.59E-07	0.380850633	0.368	0.106	0.007825965
PIK3IP1	3.70E-07	-0.423921742	0.145	0.417	0.00806063
NRROS	3.77E-07	-0.355408052	0.179	0.485	0.008217101
NFKB1	3.77E-07	0.415237101	0.316	0.068	0.008219182
RUNX1T1	3.79E-07	-0.353663543	0.043	0.288	0.008264641
ETF1	3.93E-07	0.450250805	0.581	0.265	0.008559695
LIPG	4.29E-07	0.40375795	0.256	0.038	0.009353891
RSL1D1	4.83E-07	0.419082297	0.632	0.341	0.010524973
PNPLA2	5.15E-07	-0.372941886	0.846	0.947	0.011222563
VMP1	5.17E-07	0.402289546	0.684	0.379	0.011280113
GIMAP2	5.24E-07	-0.452512252	0.274	0.553	0.011424772
DNAJA1	5.37E-07	0.426229002	0.923	0.795	0.011710915
HMOX2	5.40E-07	0.433870457	0.607	0.348	0.011766095
PA2G4	5.85E-07	0.463527782	0.692	0.447	0.012755791
SMTN	6.15E-07	-0.485092649	0.709	0.864	0.013418938
SLC25A37	6.30E-07	0.447949327	0.718	0.5	0.013749943
ZFAND2A	6.50E-07	0.702910252	0.521	0.25	0.014182518
KRBOX4	7.28E-07	-0.338691222	0.085	0.341	0.015870636
ITPR1	7.40E-07	0.421882321	0.521	0.242	0.016142014
MIR22HG	7.45E-07	0.411437916	0.573	0.273	0.01624725
NPR1	7.53E-07	-0.388406758	0.564	0.841	0.016430501
CHIC2	7.76E-07	0.499289093	0.778	0.545	0.016926105
HBB	7.87E-07	-0.509075816	0.128	0.402	0.017155493
G0S2	7.99E-07	0.383429038	0.171	0	0.017418785
HES4	7.99E-07	0.353046643	0.171	0	0.017418785
GSPT1	8.45E-07	0.433886952	0.419	0.152	0.018431797
PDIA6	9.84E-07	0.30245949	0.991	0.985	0.021455671
EMD	1.00E-06	0.421109338	0.632	0.348	0.021822026
CTNNB1	1.01E-06	0.441140311	0.744	0.477	0.021925882
R3HDM4	1.07E-06	0.396017214	0.402	0.152	0.02328753
MAP2K3	1.12E-06	0.448070754	0.479	0.205	0.024418914
TACC1	1.16E-06	-0.441904484	0.684	0.879	0.025352904
ZNF765	1.17E-06	-0.432686654	0.256	0.538	0.025414542
SERPINB8	1.20E-06	0.359097767	0.359	0.114	0.026121368
BCL3	1.26E-06	0.394874187	0.462	0.182	0.027396353
TNFRSF10B	1.26E-06	0.44346804	0.547	0.295	0.027519205
LUZP1	1.27E-06	0.440934549	0.53	0.25	0.027683646
PAPOLA	1.31E-06	0.369644996	0.675	0.439	0.028580822
RHPN2	1.32E-06	-0.28195292	0.026	0.235	0.028799963
GRAP	1.34E-06	-0.395831059	0.179	0.455	0.02915204
NXT1	1.52E-06	0.315027023	0.598	0.295	0.033256383
FOXN2	1.54E-06	-0.335998809	0.068	0.311	0.033692162
FAM110B	1.57E-06	-0.360635925	0.068	0.303	0.034169417
LPP	1.61E-06	-0.415889822	0.308	0.598	0.035122919

MSN	1.62E-06	0.402934858	0.718	0.508	0.035368084
RP11-7F17.3	1.63E-06	-0.449083628	0.145	0.409	0.035616744
CLK1	1.64E-06	-0.381080388	0.479	0.735	0.035731132
PCGF2	1.71E-06	0.382086584	0.65	0.402	0.037219
MAT2B	1.73E-06	-0.397215459	0.231	0.515	0.037673099
CCT2	1.98E-06	0.492447907	0.624	0.364	0.04319996
HNRNPU	1.99E-06	0.498670361	0.829	0.598	0.043463537
FGL2	2.05E-06	0.38196518	0.299	0.068	0.044778632
MT-ND4L	2.15E-06	-0.27187037	1	0.992	0.046880006
IL16	2.15E-06	-0.27886817	0.034	0.25	0.046977254
MANF	2.18E-06	0.577750162	0.701	0.508	0.04751416
ETS2	2.26E-06	0.361726997	0.923	0.811	0.049212012

List of differentially expressed genes of BGN_LSEC in reperfusion stage (PR versus EP)

gene	p_val	avg_logFC	pct.1	pct.2	p_val_adj
HSPD1	2.34E-31	2.126263209	0.99	0.412	5.11E-27
HSPB1	1.45E-28	1.746214273	1	0.941	3.15E-24
EMP1	1.71E-28	1.677738856	0.99	0.5	3.72E-24
HSPE1	4.92E-27	2.017667681	0.96	0.461	1.07E-22
LITAF	8.06E-27	1.472150682	0.96	0.5	1.76E-22
PLAUR	2.51E-26	1.590017362	0.85	0.118	5.47E-22
BAG3	2.88E-26	1.620542621	0.97	0.392	6.28E-22
HSP90AA1	6.40E-26	1.407999724	0.98	0.912	1.40E-21
TM4SF1	1.86E-25	1.020621005	1	0.99	4.05E-21
HSPH1	3.47E-25	1.796354172	0.89	0.284	7.57E-21
ADAMTS9	4.90E-25	1.363198481	0.88	0.157	1.07E-20
PNP	8.23E-25	1.433512386	0.93	0.324	1.80E-20
HSPA1A	2.56E-24	1.281501118	1	0.912	5.58E-20
THBD	9.16E-23	1.334623325	0.97	0.51	2.00E-18
CD59	4.67E-22	0.81916446	1	0.912	1.02E-17
LMNA	1.16E-21	1.300328258	0.93	0.529	2.53E-17
MIR4435-2HG	1.92E-21	1.47579377	0.83	0.225	4.19E-17
ID3	3.35E-21	-1.19744959	0.63	0.961	7.31E-17
AKAP12	4.30E-21	1.028837459	0.99	0.951	9.38E-17
FOSL1	5.23E-21	1.23025233	0.7	0.049	1.14E-16
TNFRSF12A	1.77E-20	1.092258714	0.63	0.01	3.85E-16
HSPA1B	1.83E-20	1.250403615	0.96	0.735	3.99E-16
HSP90AB1	1.96E-20	0.960425115	1	0.98	4.26E-16
DDX21	3.84E-20	1.117194523	0.87	0.353	8.38E-16
SOX17	1.20E-19	1.220801606	0.92	0.461	2.63E-15
CDC42EP2	1.46E-19	0.995180281	0.75	0.098	3.18E-15
HES1	9.37E-19	-1.320994	0.35	0.863	2.04E-14
SLC40A1	2.69E-18	-0.925844811	0.48	0.912	5.87E-14
LINC00152	2.90E-18	1.243842385	0.78	0.245	6.33E-14
SH3BGRL3	6.44E-18	0.878202754	0.97	0.824	1.40E-13
MAT2A	7.23E-18	1.033164302	0.83	0.304	1.58E-13
INSIG1	1.04E-16	1.218077987	0.78	0.265	2.26E-12
TSC22D3	1.06E-16	-0.923775377	0.64	0.951	2.31E-12
MYADM	1.11E-16	1.150439698	0.89	0.598	2.43E-12
UPP1	2.40E-16	0.955846991	0.9	0.52	5.24E-12
ZFP36L1	3.42E-16	-0.787489576	0.75	0.961	7.47E-12
SAT1	4.61E-16	0.885945213	1	0.98	1.01E-11
SELK	4.70E-16	0.987662169	0.89	0.549	1.02E-11
POMP	6.79E-16	0.714861644	0.97	0.804	1.48E-11
PMP22	1.08E-15	1.131426062	0.85	0.441	2.36E-11
SRSF5	3.63E-15	0.678772299	0.98	0.794	7.92E-11
CD9	1.94E-14	0.915788936	0.99	0.873	4.24E-10
ACKR3	2.00E-14	0.947991119	0.57	0.088	4.37E-10
DDIT4	2.11E-14	-0.998626707	0.75	0.971	4.59E-10
RHOB	2.14E-14	-1.064252663	0.8	0.931	4.68E-10
PTPN1	2.30E-14	1.040757561	0.74	0.314	5.02E-10
RBM3	2.49E-14	0.805144822	0.94	0.696	5.43E-10
HES4	3.05E-14	1.05226502	0.5	0.029	6.64E-10
KLF6	4.33E-14	0.87261561	0.97	0.892	9.44E-10
HSPA5	5.15E-14	1.141695845	0.97	0.853	1.12E-09
HYAL2	5.54E-14	0.97254108	0.95	0.775	1.21E-09
LDLR	6.49E-14	0.828778607	0.75	0.275	1.41E-09

MYC	7.58E-14	0.879072142	0.97	0.667	1.65E-09
RHOC	9.09E-14	0.641102735	0.97	0.853	1.98E-09
TMSB10	1.35E-13	0.481349725	1	0.99	2.94E-09
TRIB1	1.48E-13	0.902329238	0.74	0.225	3.24E-09
NOP16	1.56E-13	0.664143531	0.5	0.039	3.40E-09
DUSP6	1.58E-13	0.741024865	0.97	0.873	3.44E-09
HSPG2	1.66E-13	-0.731923075	0.76	0.951	3.62E-09
TIMP1	1.67E-13	0.949417662	1	1	3.65E-09
HSPA8	1.83E-13	0.766088698	0.98	0.971	3.99E-09
ATP13A3	3.07E-13	0.849897089	0.64	0.137	6.69E-09
CHIC2	3.09E-13	0.802764107	0.85	0.471	6.73E-09
ADAMTS4	3.22E-13	0.980332181	0.88	0.49	7.03E-09
EFNA1	3.47E-13	-0.739666699	0.57	0.902	7.56E-09
EIF4A1	5.29E-13	0.755534296	0.82	0.471	1.15E-08
MESDC1	5.87E-13	0.988398526	0.62	0.157	1.28E-08
NUAK1	9.20E-13	-0.70959941	0.14	0.618	2.01E-08
ANGPTL4	1.13E-12	1.043039892	0.71	0.235	2.47E-08
MYL6	1.25E-12	0.559835053	0.98	0.99	2.73E-08
ARID5A	1.61E-12	0.719343257	0.54	0.088	3.52E-08
GIMAP1	2.17E-12	-0.777342158	0.39	0.794	4.74E-08
MIR22HG	2.25E-12	0.805026462	0.67	0.235	4.90E-08
EHD4	2.94E-12	0.733250934	0.69	0.255	6.42E-08
DNAJB6	3.18E-12	0.859922914	0.89	0.657	6.93E-08
STAB2	4.15E-12	-0.882932945	0.47	0.833	9.04E-08
DLC1	5.59E-12	0.65730289	0.93	0.833	1.22E-07
ATP1A1	6.17E-12	0.778049497	0.83	0.441	1.35E-07
PLIN2	6.76E-12	0.696404746	0.87	0.657	1.47E-07
PDK4	7.68E-12	-0.877689782	0.6	0.902	1.67E-07
TPM3	7.85E-12	0.594280965	0.97	0.755	1.71E-07
SPHK1	7.86E-12	0.565424757	0.38	0	1.71E-07
MALL	7.98E-12	0.864818772	0.55	0.108	1.74E-07
CIRBP	8.60E-12	-0.545279724	0.87	0.961	1.88E-07
WARS	1.11E-11	0.706807975	0.87	0.539	2.42E-07
RPS29	1.22E-11	0.442880297	0.98	0.951	2.66E-07
CYCS	1.25E-11	0.706675476	0.77	0.402	2.72E-07
ID1	1.25E-11	-0.723298309	0.88	0.99	2.73E-07
RIN2	1.28E-11	0.747918873	0.74	0.324	2.78E-07
PHLDB1	1.30E-11	0.627917225	0.5	0.078	2.82E-07
ADAMTS1	1.46E-11	0.875385501	0.9	0.588	3.19E-07
NPY1R	1.61E-11	-0.775026975	0.4	0.784	3.51E-07
RASD1	1.84E-11	1.351552303	0.78	0.441	4.01E-07
MARCKS	1.85E-11	0.76729977	0.84	0.569	4.04E-07
FLT1	1.91E-11	0.682584334	0.94	0.892	4.17E-07
CYR61	2.06E-11	0.957248677	0.62	0.167	4.49E-07
ACTB	2.11E-11	0.553145997	1	1	4.59E-07
TFPI2	2.46E-11	0.919180439	0.91	0.627	5.37E-07
KLF2	3.20E-11	-0.841487747	0.46	0.833	6.97E-07
MCAM	4.98E-11	0.764122024	0.58	0.147	1.09E-06
TOMM5	5.27E-11	0.741176002	0.77	0.451	1.15E-06
JUNB	5.51E-11	-0.652188487	0.98	0.99	1.20E-06
SRGN	8.70E-11	0.681777746	0.99	0.931	1.90E-06
GIMAP8	9.17E-11	-0.740995245	0.22	0.627	2.00E-06
EIF5A	9.74E-11	0.741874245	0.79	0.451	2.12E-06
SPRY4	1.16E-10	0.793524306	0.77	0.412	2.53E-06
ZYX	1.21E-10	0.82762081	0.74	0.431	2.63E-06
MT2A	1.35E-10	1.419591558	0.96	0.853	2.93E-06
BZW1	1.38E-10	0.737917124	0.79	0.5	3.01E-06
MAFF	1.65E-10	0.747395152	0.68	0.265	3.60E-06
SERPINB9	2.06E-10	0.682964462	0.74	0.343	4.49E-06
B3GNT5	2.09E-10	0.702309504	0.49	0.088	4.56E-06
SPSB3	2.10E-10	-0.58481012	0.13	0.539	4.58E-06
UGCG	2.13E-10	0.709081459	0.86	0.549	4.65E-06
WDR43	2.18E-10	0.52956758	0.42	0.049	4.75E-06
GPR4	2.30E-10	0.734210263	0.55	0.147	5.03E-06
MCL1	2.53E-10	0.548902418	0.98	0.843	5.52E-06
SNRPB	2.59E-10	0.605487276	0.84	0.5	5.65E-06
CLIC1	2.71E-10	0.512147552	0.97	0.951	5.90E-06
PTPRB	2.72E-10	-0.653806117	0.71	0.941	5.93E-06
S1PR1	3.32E-10	0.622888797	0.91	0.843	7.23E-06
DEFA3	3.83E-10	-0.550142305	0.03	0.392	8.35E-06

NFKBIZ	4.16E-10	-0.698945857	0.47	0.863	9.08E-06
RUNX1T1	4.22E-10	-0.576939416	0.06	0.431	9.20E-06
NME1	5.57E-10	0.593430315	0.42	0.049	1.22E-05
PGK1	5.88E-10	0.667057282	0.84	0.578	1.28E-05
TCEB1	6.17E-10	0.612380531	0.89	0.696	1.35E-05
HLA-E	6.37E-10	-0.357090343	1	1	1.39E-05
RTEL1-TNFRSF6B	6.42E-10	0.906350066	0.32	0	1.40E-05
SNHG15	6.49E-10	0.630774317	0.64	0.225	1.42E-05
ENG	8.25E-10	-0.62600452	0.96	0.98	1.80E-05
IL32	8.90E-10	0.956817267	0.53	0.147	1.94E-05
FGL2	1.10E-09	0.574378047	0.43	0.059	2.40E-05
EVA1C	1.12E-09	0.682142154	0.69	0.333	2.45E-05
RELB	1.15E-09	0.534516418	0.46	0.078	2.51E-05
GIMAP4	1.23E-09	-0.641257422	0.57	0.843	2.68E-05
PIM3	1.24E-09	-0.662254169	0.17	0.588	2.70E-05
ICAM1	1.25E-09	1.084592077	0.79	0.588	2.72E-05
GPC1	1.31E-09	0.472725166	0.31	0	2.85E-05
CREM	1.41E-09	0.624423809	0.55	0.167	3.07E-05
ST6GAL1	1.62E-09	-0.594370854	0.46	0.794	3.54E-05
RAN	1.72E-09	0.630520949	0.81	0.529	3.75E-05
KDM6B	1.98E-09	0.720042103	0.68	0.353	4.32E-05
DLL4	2.11E-09	-0.654331235	0.27	0.637	4.61E-05
HNRNPAB	2.41E-09	0.68523611	0.55	0.167	5.25E-05
PVRL2	2.62E-09	0.588908027	0.87	0.755	5.71E-05
CXCL8	3.01E-09	1.48262731	0.45	0.098	6.56E-05
CDC37	3.20E-09	0.516411234	0.93	0.882	6.98E-05
PPRC1	3.27E-09	0.545083588	0.39	0.049	7.14E-05
TMEM140	3.45E-09	-0.539970555	0.26	0.637	7.53E-05
TSC22D4	3.49E-09	-0.61212472	0.08	0.441	7.60E-05
MEIS2	4.06E-09	-0.737266484	0.33	0.647	8.86E-05
RHEB	4.11E-09	0.546441496	0.77	0.402	8.95E-05
SDPR	4.41E-09	-0.551757605	0.87	0.941	9.62E-05
SEC61G	5.13E-09	0.531603453	0.91	0.716	0.00011188
PDLIM3	5.56E-09	0.721523276	0.63	0.275	0.000121303
GJA1	5.64E-09	0.670484869	0.38	0.049	0.000123087
ID2	6.04E-09	-0.698091771	0.59	0.833	0.000131746
ZFP36	6.52E-09	-0.650442871	0.98	1	0.000142086
RAP1B	6.55E-09	0.605323447	0.91	0.706	0.000142736
DNAJA4	7.35E-09	0.662569887	0.45	0.088	0.000160339
RTN4	9.04E-09	0.561746512	0.86	0.657	0.000197144
DOT1L	1.06E-08	0.556690811	0.3	0.01	0.000230825
MAF	1.14E-08	-0.591120793	0.74	0.922	0.000248933
ARID5B	1.22E-08	0.607666449	0.67	0.265	0.000266584
AHSA1	1.25E-08	0.595716039	0.47	0.118	0.00027259
PLK3	1.29E-08	0.561303534	0.59	0.206	0.000281768
GADD45A	1.30E-08	0.586704056	0.63	0.255	0.000282529
YES1	1.31E-08	0.603844254	0.82	0.539	0.000286646
GIMAP2	1.45E-08	-0.562586657	0.19	0.529	0.000316456
EIF3I	1.48E-08	0.612940164	0.82	0.52	0.000322324
PTRH2	1.50E-08	0.514305033	0.42	0.078	0.000328169
FOS	1.57E-08	-0.633381843	0.98	0.99	0.000342427
LATS2	1.63E-08	0.463613857	0.52	0.137	0.000354853
PHLDA1	1.79E-08	1.019035804	0.6	0.265	0.000389436
GRAP	1.91E-08	-0.50506392	0.08	0.422	0.000415816
HLA-DRB5	2.06E-08	0.668628134	0.94	0.804	0.000449689
ISG20	2.07E-08	0.555630114	0.38	0.059	0.000452474
PDLIM1	2.09E-08	0.428512514	0.99	0.971	0.000455803
RPS16	2.13E-08	0.302059634	0.99	0.99	0.00046544
TSSC4	2.25E-08	0.633627555	0.51	0.147	0.000491589
GCC2	2.41E-08	-0.61311382	0.15	0.49	0.000525805
SLC20A1	2.47E-08	0.656348634	0.59	0.225	0.000538184
RSRP1	2.54E-08	-0.543301065	0.58	0.873	0.000553728
C11orf96	2.59E-08	0.671044657	0.82	0.471	0.000565399
ETS2	2.61E-08	0.45839718	0.96	0.882	0.000568402
CLDN5	2.62E-08	-0.53321084	0.84	0.961	0.000571968
ART4	2.63E-08	-0.556098522	0.26	0.608	0.000572805
H3F3B	2.66E-08	0.356723877	1	0.98	0.000580866
LIFR	2.75E-08	-0.543885188	0.86	0.971	0.000599714
SEPP1	2.81E-08	-0.463348697	0.98	1	0.000613801
NRP1	2.86E-08	-0.505364662	0.57	0.843	0.000623215

SNU13	2.91E-08	0.548683277	0.85	0.647	0.00063435
VCAM1	2.94E-08	1.023115588	0.56	0.216	0.000640632
ARHGAP23	3.41E-08	0.516959104	0.51	0.147	0.000743226
INPP1	3.51E-08	0.60931916	0.73	0.451	0.000766083
CLK1	3.71E-08	-0.552387274	0.4	0.716	0.000808114
YWHAH	3.75E-08	0.542587426	0.79	0.529	0.000818721
YBX3	3.86E-08	0.623122924	0.8	0.539	0.000840905
EIF1	4.13E-08	0.276440517	1	1	0.000900733
COL4A3BP	4.32E-08	-0.57876312	0.26	0.588	0.00094187
CDKN1B	4.71E-08	-0.548627458	0.25	0.588	0.00102747
TEAD4	5.63E-08	0.601539514	0.64	0.275	0.001228173
KLF10	5.73E-08	-0.577422889	0.29	0.637	0.001248583
PICALM	6.63E-08	0.497409482	0.59	0.206	0.001446432
NOLC1	7.12E-08	0.597175945	0.62	0.275	0.001553498
MANF	8.18E-08	0.463027136	0.72	0.382	0.001784406
IRF2BPL	8.96E-08	-0.551418576	0.24	0.569	0.001953283
RELN	9.43E-08	-0.732236715	0.25	0.569	0.002057241
PEA15	9.46E-08	0.55342909	0.69	0.353	0.002064118
FAM212A	9.73E-08	-0.354732824	0.01	0.275	0.002121601
FSTL3	9.83E-08	0.853244612	0.27	0.01	0.002142952
CLK4	9.86E-08	-0.511828849	0.06	0.363	0.002151267
SLCO4A1	1.12E-07	0.560624885	0.66	0.294	0.002448091
NRROS	1.21E-07	-0.401117708	0.06	0.373	0.002634539
NOP58	1.29E-07	0.50542584	0.64	0.284	0.002822037
ITPR1	1.40E-07	0.574419964	0.56	0.225	0.003052472
TINAGL1	1.42E-07	0.405702391	0.97	1	0.00309885
PNPLA8	1.45E-07	0.546795721	0.63	0.314	0.003167063
SOCS2	1.45E-07	0.595258515	0.84	0.569	0.003168618
NUPR1	1.50E-07	-0.568743828	0.51	0.775	0.003266933
CRHBP	1.51E-07	-0.477823436	0.99	1	0.003286939
AP1S2	1.51E-07	-0.47638842	0.69	0.873	0.003295778
TUBA1C	1.52E-07	0.623983431	0.6	0.265	0.003310548
MT-ND4L	1.55E-07	-0.273637401	0.98	1	0.003373973
TMEM59	1.57E-07	-0.326475517	0.89	0.971	0.003424575
STIP1	1.64E-07	0.44291595	0.4	0.088	0.003581888
SERPINE1	1.65E-07	0.636881874	0.51	0.167	0.003590429
SLC44A2	1.70E-07	-0.576244514	0.52	0.765	0.003708816
SMAD6	1.71E-07	-0.5402142	0.11	0.412	0.003724795
DDIT3	1.92E-07	-0.630246496	0.26	0.569	0.004179629
AKAP2	1.97E-07	0.572804442	0.58	0.255	0.004302498
PHF3	2.02E-07	-0.505383165	0.19	0.52	0.004405931
TMEM204	2.06E-07	-0.446892228	0.37	0.706	0.004493257
ANXA2	2.14E-07	0.498186562	0.93	0.863	0.004656165
ZFAND2A	2.15E-07	0.789019135	0.53	0.186	0.004680208
PDLIM5	2.33E-07	0.618214243	0.68	0.363	0.005084047
PNO1	2.35E-07	0.411707637	0.32	0.039	0.005121675
MTHFD2	2.38E-07	0.451893889	0.37	0.069	0.005180571
UBE2N	2.39E-07	0.523737746	0.71	0.392	0.005213753
ILF2	2.45E-07	0.462695845	0.74	0.402	0.005351366
TES	2.53E-07	0.412197479	0.3	0.029	0.005527327
SSH1	2.69E-07	0.649670133	0.49	0.167	0.005877124
SC5D	2.89E-07	-0.594523134	0.57	0.843	0.006298145
IQCJ-SCHIP1	3.19E-07	0.409678821	0.38	0.078	0.00696175
OIT3	3.46E-07	-0.564898606	0.88	0.951	0.00753705
SNX6	3.76E-07	-0.472414603	0.63	0.863	0.008193863
FLNA	3.82E-07	0.519759703	0.39	0.088	0.008335923
VIMP	3.89E-07	0.624739808	0.76	0.549	0.00848223
ANKRD55	4.11E-07	-0.513568602	0.11	0.412	0.008967462
MTFP1	4.67E-07	0.41909776	0.25	0.01	0.010194421
MT1X	4.72E-07	1.526040097	0.59	0.265	0.010288576
LRRC32	4.73E-07	0.713399985	0.71	0.382	0.010319488
LRRC59	4.88E-07	0.475356696	0.55	0.216	0.010644686
TNFRSF10A	5.28E-07	0.31275748	0.27	0.02	0.011512381
TNFRSF10B	5.53E-07	0.585911657	0.57	0.265	0.012067832
PCMTD2	6.11E-07	-0.512567252	0.21	0.52	0.013319744
ACTG1	6.25E-07	0.467596088	0.98	0.98	0.013633911
NOS3	6.30E-07	0.726231787	0.63	0.343	0.013742011
MIF	6.73E-07	0.444666111	0.95	0.824	0.014687135
F2R	6.78E-07	-0.412905039	0.82	0.912	0.014789424
DNAJB11	6.79E-07	0.6202179	0.62	0.333	0.014804266

BCL3	6.80E-07	0.527854621	0.51	0.196	0.01483562
SDF2L1	7.06E-07	0.668943594	0.59	0.275	0.015390432
F8	7.98E-07	-0.467803671	0.83	0.98	0.017394428
EIF2S1	8.08E-07	0.41977495	0.54	0.206	0.01762387
HMOX2	8.15E-07	0.479871422	0.75	0.461	0.017765367
SEMA6A	8.68E-07	-0.503440883	0.52	0.784	0.018934763
HBB	8.72E-07	-2.793701344	0.14	0.451	0.019025441
NCL	8.76E-07	0.470074673	0.88	0.706	0.019113975
ARHGDIA	8.92E-07	0.536566416	0.79	0.51	0.019443873
PTGES3	8.93E-07	0.476445195	0.97	0.853	0.019463727
NAMPT	9.01E-07	0.461116031	0.96	0.843	0.019639712
FAM84B	9.49E-07	-0.380274149	0.08	0.373	0.020693967
MORF4L2	1.03E-06	0.539991677	0.77	0.539	0.022422123
SNRPD1	1.09E-06	0.489724993	0.63	0.304	0.023692685
NOP10	1.09E-06	0.458221931	0.89	0.745	0.023817775
ADAM15	1.21E-06	0.498244897	0.58	0.255	0.026278789
FHL3	1.27E-06	0.416718892	0.33	0.059	0.027593013
CREB5	1.29E-06	0.371695188	0.23	0.01	0.02817124
ADGRG1	1.30E-06	0.516367751	0.69	0.392	0.028455115
SH3BP5	1.31E-06	0.46079926	0.87	0.755	0.028590324
NR4A2	1.38E-06	-0.430393936	0.19	0.5	0.030022331
ABCE1	1.41E-06	0.433927747	0.41	0.118	0.030666189
FCHO2	1.46E-06	-0.504614312	0.23	0.51	0.03182015
MTHFR	1.48E-06	-0.44471114	0.15	0.451	0.032238652
SAT2	1.54E-06	-0.398827873	0.49	0.784	0.033662043
MT-CO3	1.57E-06	-0.270817363	0.99	1	0.034344277
DUSP1	1.59E-06	-0.370440172	0.98	0.99	0.03475842
STARD4	1.60E-06	0.4884306	0.38	0.098	0.034852592
HNRNPU	1.65E-06	0.379707062	0.91	0.647	0.036022477
B4GALT5	1.65E-06	0.493715244	0.63	0.304	0.036081727
PPARD	1.68E-06	0.337753123	0.29	0.039	0.03661838
GRN	1.69E-06	-0.414212704	0.87	0.951	0.036820796
SHE	1.78E-06	-0.517580941	0.2	0.48	0.038750175
VAPA	1.82E-06	0.443448019	0.93	0.794	0.039593476
LPAR6	1.95E-06	-0.477720851	0.84	0.892	0.042422853
CHKA	1.96E-06	-0.367773555	0.06	0.324	0.042687679
FOXN2	2.01E-06	-0.408362433	0.11	0.392	0.043774396
ALPL	2.01E-06	0.644695989	0.53	0.225	0.043804228
MRTO4	2.10E-06	0.356926539	0.29	0.039	0.045748772
NR2F2	2.19E-06	-0.516522365	0.51	0.755	0.04772945
DNASE1L3	2.20E-06	-0.373250881	0.99	1	0.047986446
CRACR2B	2.22E-06	-0.544610005	0.21	0.48	0.048493596

List of differentially expressed genes of STAB1_LSEC in reperfusion stage (PR versus EP)

gene	p_val	avg_logFC	pct.1	pct.2	p_val_adj
HSPE1	2.68E-17	1.975838608	0.848	0.213	5.84E-13
HSPB1	2.96E-15	1.555256335	0.967	0.689	6.45E-11
HSPD1	3.46E-15	1.744803814	0.837	0.311	7.55E-11
TM4SF1	7.17E-15	1.343069499	0.989	0.852	1.56E-10
AKAP12	1.26E-12	0.954823504	0.978	0.836	2.75E-08
HSP90AA1	2.04E-12	1.251691561	0.924	0.689	4.44E-08
HSP90AB1	4.69E-12	0.976259732	0.967	0.672	1.02E-07
ID3	6.85E-12	-1.21577975	0.446	0.902	1.49E-07
ADAMTS9	8.32E-12	1.773775433	0.63	0.082	1.82E-07
EMP1	2.32E-11	1.72992772	0.685	0.148	5.05E-07
ENG	2.63E-11	-0.993320186	0.739	0.934	5.72E-07
SAT1	2.95E-11	1.135566308	0.978	0.787	6.44E-07
CD9	4.01E-11	0.998904223	0.935	0.607	8.75E-07
PNP	1.04E-10	1.40992749	0.652	0.164	2.27E-06
TIMP1	8.02E-10	1.014711916	0.989	0.951	1.75E-05
TACC1	8.93E-10	-0.91105336	0.293	0.738	1.95E-05
MT2A	9.58E-10	1.600772306	0.967	0.852	2.09E-05
EFNA1	2.19E-09	-0.922418124	0.25	0.738	4.78E-05
IGFBP4	5.98E-09	-0.765474124	0.663	0.918	0.000130348
PLAUR	7.62E-09	1.135221788	0.522	0.049	0.000166105
MIR4435-2HG	8.13E-09	1.353520566	0.554	0.115	0.00017727
RELN	1.08E-08	-1.069137507	0.293	0.721	0.000234962

HLA-E	1.11E-08	-0.532142976	0.891	0.967	0.000241061
HSPA1A	1.17E-08	0.927217319	0.935	0.656	0.000255527
AGFG2	1.20E-08	-0.918089475	0.076	0.492	0.000261285
CIRBP	1.87E-08	-0.801610919	0.457	0.787	0.000408417
THBD	2.16E-08	1.19473522	0.663	0.246	0.000470916
MIR222HG	2.29E-08	1.220999556	0.467	0.049	0.000500057
TOMM5	2.42E-08	0.969120338	0.652	0.262	0.000528208
CD4	4.31E-08	-0.817322286	0.467	0.82	0.00094021
HES1	5.16E-08	-1.034133509	0.174	0.59	0.001124509
ADAMTS1	5.84E-08	1.258448895	0.652	0.213	0.001273254
DEFA3	6.02E-08	-0.839655531	0.043	0.393	0.001312143
CYR61	6.51E-08	1.160600331	0.38	0	0.0014197
MT1X	7.03E-08	1.812331123	0.728	0.443	0.001533957
ADD3	8.36E-08	-0.804293202	0.207	0.623	0.001824099
TSC22D4	8.93E-08	-0.892627665	0.043	0.377	0.001947511
OIT3	9.22E-08	-0.767916796	0.62	0.869	0.002009741
HSPG2	1.12E-07	-0.93225696	0.62	0.885	0.002451525
SH3BGRL3	1.31E-07	0.910499583	0.793	0.607	0.002856135
PDE2A	1.74E-07	-0.623877349	0.293	0.705	0.003792535
NOTCH4	2.00E-07	-0.978030294	0.359	0.721	0.004365093
CDC42EP2	2.17E-07	1.090543212	0.38	0.016	0.004724046
BZW1	2.55E-07	0.95507687	0.478	0.082	0.005568846
ADAMTS4	2.64E-07	1.093197541	0.609	0.197	0.005747994
TMSB10	3.48E-07	0.595229774	1	0.984	0.007579173
ACP5	3.54E-07	-0.818525364	0.685	0.918	0.007728312
HLA-DRB1	3.63E-07	0.701583791	0.913	0.705	0.007906438
HSPA1B	3.67E-07	0.745783058	0.859	0.508	0.008004333
PIM3	6.67E-07	-0.719921483	0.098	0.459	0.014544545
LMNA	7.07E-07	1.196170122	0.576	0.23	0.015419117
MYADM	7.09E-07	1.076860265	0.565	0.213	0.015471823
NRP1	7.32E-07	-0.800988311	0.272	0.656	0.015974113
PDCD4	7.92E-07	-0.785783598	0.163	0.525	0.017281921
SLC44A2	8.21E-07	-0.82436072	0.174	0.525	0.017908571
GIMAP2	8.50E-07	-0.82779189	0.109	0.443	0.018527168
MCL1	8.88E-07	0.810910262	0.783	0.541	0.01936357
MFNG	1.18E-06	-0.691123667	0.043	0.344	0.025732554
POMP	1.19E-06	0.716113273	0.793	0.574	0.025877856
AMHR2	1.34E-06	-0.689875041	0.033	0.311	0.029259666
TGFBR3	1.42E-06	-0.665452273	0.37	0.77	0.030865597
BAG3	1.47E-06	1.216084234	0.489	0.148	0.032004742
STAB2	1.52E-06	-0.95750428	0.478	0.787	0.033090995
SNRPD1	1.54E-06	0.905819084	0.435	0.082	0.03356552
SRGN	1.56E-06	0.85755152	0.848	0.623	0.033973655
FOSL1	1.68E-06	0.972488049	0.337	0.016	0.036701524
MESDC1	1.75E-06	0.915599685	0.457	0.098	0.038078102
WWP1	1.82E-06	-0.699857981	0.054	0.361	0.039695297
MRC1	1.83E-06	-0.706971092	0.576	0.885	0.039877236
DDIT4	1.97E-06	-0.986872948	0.359	0.689	0.042895272
GPR4	2.13E-06	0.815368432	0.337	0.016	0.046375015
HLA-DRB5	2.18E-06	0.839851661	0.739	0.443	0.047436286
MYL6	2.25E-06	0.52665482	0.967	0.885	0.049141387

List of differentially expressed genes of MGP_VEC in reperfusion stage (PR versus EP)

gene	p_val	avg_logFC	pct.1	pct.2	p_val_adj
EMP1	1.30E-24	1.659096934	0.968	0.63	2.84E-20
AKAP12	1.95E-22	2.036046952	0.912	0.384	4.25E-18
TIMP1	5.27E-22	2.321936891	0.976	0.945	1.15E-17
PNP	1.31E-21	1.642983119	0.904	0.356	2.87E-17
HES1	2.64E-20	-1.375308072	0.232	0.849	5.75E-16
LMNA	3.91E-20	1.582799683	0.912	0.63	8.52E-16
HSPB1	5.85E-20	1.372243031	0.984	0.89	1.28E-15
RHOB	3.74E-19	-1.194784567	0.664	0.986	8.16E-15
TM4SF1	4.44E-19	1.080822155	1	0.986	9.69E-15
JUNB	6.03E-19	-0.973324052	0.864	0.986	1.32E-14
ADAMTS9	1.25E-18	1.79141437	0.784	0.151	2.72E-14
SAT1	9.93E-18	1.381547364	0.992	0.904	2.17E-13
HES4	1.25E-17	1.769151604	0.72	0.082	2.74E-13

TSC22D3	7.44E-17	-1.114037839	0.448	0.877	1.62E-12
HSP90AA1	1.72E-16	1.271495596	0.928	0.904	3.76E-12
PLAUR	1.81E-16	1.659453562	0.656	0.041	3.96E-12
DDIT4	5.39E-16	-1.100380394	0.656	0.959	1.18E-11
TXNIP	7.66E-16	-1.042803804	0.136	0.671	1.67E-11
RUNX1T1	8.69E-16	-0.919526963	0.04	0.534	1.89E-11
ANGPTL4	8.77E-16	1.507852643	0.824	0.288	1.91E-11
UPP1	1.84E-15	1.334448747	0.8	0.329	4.01E-11
FOS	8.22E-15	-0.957457471	0.872	1	1.79E-10
HSPA1A	1.04E-14	1.076330115	0.968	0.836	2.26E-10
MT2A	1.21E-14	1.835896434	0.976	0.918	2.64E-10
C11orf96	4.48E-14	1.278326176	0.816	0.315	9.77E-10
DDX21	6.58E-14	1.222591984	0.752	0.301	1.44E-09
HSPA1B	1.47E-13	1.063490713	0.936	0.795	3.20E-09
ADAMTS4	1.64E-13	1.396815202	0.776	0.356	3.57E-09
CD59	1.95E-13	0.776192173	0.944	0.795	4.26E-09
MYC	2.34E-13	1.207138775	0.848	0.534	5.11E-09
SH3BGRL3	2.92E-13	0.916666976	0.888	0.781	6.37E-09
FOSL1	3.00E-13	1.351541665	0.6	0.082	6.53E-09
HSP90AB1	3.23E-13	0.759846934	0.976	0.932	7.05E-09
TCEB1	7.04E-13	1.203728162	0.792	0.479	1.53E-08
THBD	7.44E-13	1.151594969	0.832	0.466	1.62E-08
IER2	9.21E-13	-0.848850173	0.824	0.986	2.01E-08
EGR1	1.58E-12	-0.96276365	0.496	0.89	3.45E-08
VCAM1	2.11E-12	1.571820915	0.68	0.192	4.60E-08
BAG3	2.26E-12	1.458085408	0.744	0.37	4.93E-08
HSPE1	3.13E-12	1.485950018	0.76	0.466	6.83E-08
KLF2	4.20E-12	-0.873420337	0.44	0.849	9.16E-08
MT1X	4.31E-12	2.305854004	0.752	0.37	9.41E-08
HSPD1	6.89E-12	1.426412629	0.752	0.397	1.50E-07
LITAF	1.71E-11	1.210983967	0.72	0.397	3.73E-07
TNFRSF12A	1.81E-11	1.186259755	0.48	0.014	3.94E-07
MYADM	3.19E-11	1.041073821	0.832	0.575	6.96E-07
MIR4435-2HG	4.91E-11	1.248563977	0.648	0.247	1.07E-06
EFNA1	6.60E-11	-0.745634493	0.512	0.904	1.44E-06
HSPH1	7.68E-11	1.216373987	0.704	0.301	1.67E-06
KIAA1551	8.11E-11	-0.639362906	0.016	0.342	1.77E-06
KDM6B	9.22E-11	1.171590017	0.616	0.192	2.01E-06
ZFP36	2.37E-10	-0.688056793	0.904	0.986	5.17E-06
SLC44A2	3.20E-10	-0.73145967	0.192	0.603	6.97E-06
ID3	3.48E-10	-0.713416677	0.776	0.973	7.59E-06
JUN	6.46E-10	-0.790403914	0.88	1	1.41E-05
MAT2A	6.80E-10	1.101019084	0.696	0.342	1.48E-05
ID2	7.12E-10	-0.870629853	0.496	0.808	1.55E-05
ADAMTS1	7.90E-10	0.910220311	0.88	0.671	1.72E-05
SGMS2	7.95E-10	0.962375942	0.4	0	1.73E-05
MT1A	1.31E-09	1.958462083	0.488	0.082	2.85E-05
IL6	1.38E-09	1.881758241	0.408	0.014	3.01E-05
NR2F2-AS1	1.43E-09	-0.681957964	0.04	0.356	3.12E-05
S100A6	1.61E-09	0.969220488	0.92	0.822	3.51E-05
GIMAP1	1.74E-09	-0.818724935	0.24	0.616	3.80E-05
CIRBP	2.13E-09	-0.566333737	0.648	0.932	4.64E-05
ICAM1	2.13E-09	1.510355422	0.64	0.233	4.65E-05
ATP1A1	2.24E-09	0.889679964	0.784	0.548	4.88E-05
LDLR	2.31E-09	0.874166912	0.528	0.11	5.04E-05
PTPRB	2.45E-09	-0.655522984	0.6	0.89	5.34E-05
NUAK1	2.71E-09	-0.770460627	0.104	0.466	5.92E-05
TUBB6	2.88E-09	1.002902242	0.608	0.219	6.27E-05
CYCS	3.13E-09	0.862875649	0.648	0.274	6.82E-05
INSIG1	3.17E-09	1.293900899	0.592	0.205	6.91E-05
DUSP1	3.28E-09	-0.57686737	0.928	0.986	7.16E-05
GIMAP7	3.47E-09	-0.58938832	0.168	0.589	7.57E-05
KLF6	4.72E-09	0.831295644	0.824	0.644	0.000102951
CLEC14A	5.18E-09	-0.620623576	0.776	0.932	0.000112987
TMEM70	5.69E-09	0.977043252	0.528	0.123	0.000124037
MECOM	6.28E-09	-0.804087177	0.224	0.575	0.000137037
EIF1	6.97E-09	0.387607194	1	0.986	0.000151917
HIF3A	7.16E-09	-0.82019545	0.112	0.466	0.000156088
TOMM5	7.65E-09	0.929873421	0.68	0.342	0.000166813
NOP16	8.09E-09	0.920543024	0.432	0.041	0.000176421

RNF144B	8.23E-09	-0.475399816	0.04	0.356	0.000179583
NOS3	8.31E-09	0.994517884	0.608	0.205	0.000181227
ACKR3	9.11E-09	0.893184628	0.52	0.096	0.000198564
CCL2	9.11E-09	1.758144622	0.576	0.178	0.000198628
DLL4	9.26E-09	-0.682093841	0.248	0.644	0.000202001
POMP	9.43E-09	0.777425089	0.848	0.644	0.000205638
TRIB1	1.13E-08	0.942450866	0.456	0.068	0.000247251
HSPA5	1.14E-08	1.414265792	0.8	0.534	0.000248767
CLDN5	1.29E-08	-0.638704731	0.504	0.904	0.000281727
TMEM204	1.34E-08	-0.596386429	0.16	0.534	0.000293187
RSRP1	1.75E-08	-0.54710231	0.32	0.726	0.000381726
HLA-E	1.82E-08	-0.40853776	0.96	0.986	0.000396261
ZFP36L2	2.09E-08	-0.632219804	0.568	0.863	0.000455772
EVA1C	2.36E-08	0.830472722	0.68	0.329	0.000514301
CDC42EP2	2.58E-08	0.844810893	0.456	0.082	0.000562953
WARS	2.79E-08	0.916341118	0.72	0.438	0.000609348
SDPR	2.95E-08	-0.664732936	0.592	0.877	0.000643961
NR4A2	3.24E-08	-0.739971723	0.184	0.534	0.000706171
STC1	3.58E-08	1.367284431	0.512	0.151	0.000781517
MESDC1	4.30E-08	0.903223714	0.464	0.096	0.000937584
GIMAP4	4.32E-08	-0.648503987	0.344	0.712	0.000942365
GADD45A	4.36E-08	0.869465726	0.592	0.219	0.000951681
SERPINB1	4.41E-08	1.095660785	0.784	0.63	0.000962315
EDN1	5.50E-08	-0.880278374	0.216	0.562	0.001199357
SLCO4A1	5.70E-08	0.818519947	0.416	0.055	0.001242231
BIRC3	6.39E-08	1.101601228	0.368	0.027	0.001392626
SLC40A1	6.78E-08	-0.637834451	0.312	0.671	0.001477944
HEY1	7.03E-08	-0.592272942	0.136	0.479	0.001532975
LINC00152	7.29E-08	1.026615441	0.544	0.205	0.001589968
RNF187	7.43E-08	-0.556815427	0.144	0.479	0.001621041
NPY1R	8.40E-08	-0.59639982	0.192	0.548	0.001831311
SORBS3	8.50E-08	-0.502706804	0.224	0.616	0.001853387
HBB	1.01E-07	-0.638371966	0.096	0.425	0.002207199
ZFP36L1	1.04E-07	-0.684536326	0.512	0.863	0.002270436
EIF5A	1.10E-07	0.80238914	0.664	0.397	0.0023969
CALHM2	1.10E-07	-0.529904244	0.072	0.37	0.002399891
HOXB2	1.16E-07	-0.442909976	0.024	0.274	0.002533692
PLPP3	1.18E-07	0.649630338	0.912	0.877	0.00256674
CHIC2	1.22E-07	0.829545632	0.68	0.37	0.002656589
BCL3	1.28E-07	0.957610382	0.464	0.11	0.002793561
MMRN1	1.39E-07	-0.620938658	0.336	0.671	0.003036825
PNKD	1.60E-07	-0.527107524	0.2	0.521	0.003481183
LDB2	1.62E-07	-0.534281094	0.728	0.89	0.003532732
HMGA1	1.65E-07	0.840782959	0.392	0.055	0.003593643
MT1M	1.72E-07	1.91295102	0.64	0.329	0.003755236
KIAA1033	1.93E-07	-0.62348295	0.08	0.37	0.004213809
SNRPB	2.07E-07	0.740583631	0.664	0.342	0.004506359
SPHK1	2.15E-07	0.769000055	0.328	0.014	0.004685671
EIF3I	2.61E-07	0.655943711	0.68	0.411	0.005694909
H3F3B	2.69E-07	0.447784047	0.976	0.945	0.005864076
TMSB10	3.08E-07	0.439151028	1	0.986	0.006726247
GIMAP2	3.17E-07	-0.490815	0.064	0.342	0.00691453
NKTR	3.26E-07	-0.436538353	0.168	0.534	0.00710418
R3HDM4	3.39E-07	0.669244785	0.32	0.014	0.007391519
JUND	3.57E-07	0.673255374	0.848	0.616	0.007781213
IMP3	4.04E-07	-0.390300417	0.144	0.479	0.008814121
SBNO2	4.41E-07	0.924539115	0.4	0.068	0.009622206
ARID5A	4.41E-07	0.795598238	0.512	0.164	0.009622595
RALGDS	4.61E-07	0.821553093	0.52	0.164	0.010057987
BTG2	4.70E-07	-0.634293624	0.168	0.479	0.010249943
S100A11	4.98E-07	0.621291769	0.896	0.726	0.010863195
SMAD7	5.07E-07	-0.616795853	0.088	0.384	0.01105011
STOM	5.12E-07	0.525625536	0.896	0.863	0.011157994
ZFYVE21	5.17E-07	-0.524332216	0.096	0.384	0.011283459
EGFL7	5.42E-07	-0.461773536	0.672	0.959	0.011819912
CFL1	5.95E-07	0.560169676	0.864	0.849	0.01297209
EPHA4	6.36E-07	-0.407200044	0.064	0.329	0.013871586
CCDC28A	6.65E-07	-0.385404697	0.04	0.288	0.014510582
SHE	7.36E-07	-0.634921475	0.152	0.466	0.01605814
JMJD1C	7.92E-07	0.703115044	0.816	0.644	0.017278425

RTEL1-TNFRSF6B	7.92E-07	1.051837057	0.28	0	0.017282199
SQRDL	8.12E-07	-0.420851532	0.048	0.301	0.017713009
UBL7	8.15E-07	-0.431782803	0.064	0.329	0.017768699
ACTB	8.31E-07	0.619561892	0.976	1	0.018117411
RFTN2	8.42E-07	-0.378961775	0.024	0.247	0.018357599
SOX17	8.53E-07	0.974695351	0.664	0.384	0.018596273
FAM212A	8.54E-07	-0.446410667	0.008	0.205	0.018631542
HSPG2	8.61E-07	-0.453016626	0.824	1	0.018769884
KLF10	9.32E-07	-0.616857764	0.304	0.63	0.020329599
PIK3IP1	1.07E-06	-0.382792876	0.008	0.205	0.023251445
MALL	1.09E-06	0.848321851	0.496	0.192	0.02384662
CCDC174	1.18E-06	-0.342600349	0.056	0.315	0.025800658
MAT2B	1.23E-06	-0.446431391	0.12	0.411	0.026743451
SYPL1	1.23E-06	-0.552428829	0.544	0.822	0.026929931
PKIG	1.33E-06	1.052767635	0.56	0.26	0.028950259
BNIP3L	1.40E-06	-0.481658774	0.344	0.671	0.030515244
ETS2	1.50E-06	0.668340776	0.784	0.658	0.032736885
SERPINB9	1.53E-06	0.895555981	0.632	0.397	0.033400088
TNFSF10	1.55E-06	-0.566545868	0.248	0.575	0.033750854
SPSB3	1.71E-06	-0.449826018	0.112	0.411	0.03726517
ZBTB16	1.72E-06	-0.423793613	0.08	0.356	0.03752683
PLEC	1.74E-06	0.848026115	0.6	0.288	0.038054471
TFPI2	1.93E-06	1.205211116	0.576	0.301	0.042073594
NRROS	2.19E-06	-0.345030907	0.008	0.192	0.047860947
EHD4	2.25E-06	0.691244432	0.528	0.205	0.049113886

List of differentially expressed genes of NUPR1_LSEC in reperfusion stage (PR versus EP)

gene	p_val	avg_logFC	pct.1	pct.2	p_val_adj
HSPA1A	5.54E-37	3.328646743	0.968	0.088	1.21E-32
HSPA1B	1.41E-33	2.647444129	0.914	0.053	3.08E-29
HSPE1	7.46E-30	2.581051628	0.871	0.115	1.63E-25
HSPD1	1.30E-27	2.593983836	0.774	0.035	2.84E-23
HSPB1	4.64E-26	1.899736167	0.957	0.655	1.01E-21
DNAJB1	6.61E-25	2.152486843	0.828	0.133	1.44E-20
BAG3	1.38E-22	2.357046659	0.667	0.027	3.00E-18
CDKN1A	7.67E-20	2.021844206	0.688	0.088	1.67E-15
HSPH1	2.41E-19	1.95893929	0.602	0.027	5.26E-15
HSP90AA1	3.36E-19	1.470934066	0.871	0.398	7.32E-15
MT2A	1.46E-17	2.007325785	0.882	0.54	3.19E-13
ZFAND2A	5.36E-15	2.131903437	0.484	0.027	1.17E-10
ID3	9.68E-14	-1.360774888	0.258	0.77	2.11E-09
HSP90AB1	5.73E-13	1.199067671	0.763	0.363	1.25E-08
LITAF	1.03E-12	1.348783482	0.591	0.15	2.25E-08
MCL1	2.74E-12	1.368357921	0.548	0.106	5.98E-08
HSPA8	5.87E-12	1.285743264	0.785	0.442	1.28E-07
MIR4435-2HG	6.25E-12	1.445543045	0.419	0.035	1.36E-07
SAT1	1.32E-11	0.979016696	0.86	0.602	2.88E-07
NR4A1	1.82E-11	1.343516166	0.387	0.018	3.96E-07
CCL2	2.30E-11	1.979126246	0.376	0.018	5.01E-07
PNP	3.01E-11	1.371084503	0.419	0.044	6.57E-07
PDK4	1.36E-10	-0.994287461	0.215	0.664	2.97E-06
TM4SF1	1.92E-10	1.091587928	0.785	0.531	4.20E-06
PHLDA1	2.30E-10	1.28033002	0.43	0.062	5.01E-06
LINC00152	2.39E-10	1.436370948	0.43	0.062	5.22E-06
HBB	2.53E-10	-1.50295826	0	0.354	5.51E-06
MYC	3.25E-10	1.333333242	0.419	0.053	7.08E-06
DDIT4	3.45E-10	-1.247681686	0.108	0.531	7.53E-06
HSPA6	4.51E-10	1.898328346	0.301	0	9.83E-06
DNAJB4	9.19E-10	1.320654822	0.366	0.035	2.00E-05
ZFP36L1	1.58E-09	-0.999958057	0.194	0.628	3.45E-05
DNAJA1	1.93E-09	1.267466721	0.559	0.204	4.22E-05
ID1	2.06E-09	-0.862104972	0.28	0.717	4.48E-05
DEFA3	3.01E-09	-1.285609606	0.065	0.434	6.57E-05
HSPA5	3.41E-09	1.203085465	0.677	0.336	7.44E-05
SOX17	6.71E-09	1.326484443	0.366	0.053	0.000146325
ICAM1	9.36E-09	1.336179498	0.441	0.115	0.000204044
SDF2L1	9.47E-09	1.122898657	0.473	0.133	0.000206443

IGLV2-14	1.44E-08	-1.323993923	0.011	0.319	0.000315094
PLIN2	1.99E-08	1.007230864	0.57	0.239	0.000434914
DDX21	2.08E-08	1.266432288	0.269	0.009	0.000452542
TCEB1	6.74E-08	1.010294111	0.505	0.177	0.001469002
C11orf96	7.18E-08	1.054562353	0.29	0.027	0.001565747
ATF3	1.25E-07	1.162782068	0.247	0.009	0.00271774
DNAJB6	1.26E-07	1.014433135	0.495	0.177	0.00275761
SOD2	2.33E-07	1.073186607	0.441	0.133	0.005084693
CDC42EP2	2.66E-07	1.141395174	0.301	0.044	0.005804599
NXT1	3.84E-07	1.093371905	0.247	0.018	0.008365824
NAMPT	3.96E-07	0.811924596	0.613	0.336	0.008632464
KLF6	4.53E-07	1.062087522	0.43	0.133	0.009868339
ARID5A	4.84E-07	1.039498423	0.28	0.035	0.010558388
ARPC5L	4.86E-07	0.903155315	0.419	0.124	0.010599942
IL32	8.30E-07	1.135625221	0.419	0.133	0.018096783
ADM	9.18E-07	-0.886543508	0.183	0.504	0.020024385
ZNF593	9.28E-07	0.990001656	0.323	0.062	0.020228489
DLC1	9.93E-07	0.8848044	0.43	0.133	0.021648717
MAFF	1.09E-06	0.859429341	0.194	0	0.023808722
GIMAP7	1.51E-06	-1.027095492	0.075	0.363	0.032996181
CXCL2	1.59E-06	1.365547997	0.247	0.027	0.03467403
CTSD	1.72E-06	-0.535143158	0.806	0.929	0.0374038
H3F3B	1.74E-06	0.722794289	0.796	0.611	0.037943131
KDM6B	2.17E-06	0.985221283	0.204	0.009	0.047407919

List of differentially expressed genes of RBP7_VEC in reperfusion stage (PR versus EP)

gene	p_val	avg_logFC	pct.1	pct.2	p_val_adj
HSPA1A	9.37E-18	1.938733675	1	0.755	2.04E-13
HSPA1B	3.54E-17	1.78428606	1	0.653	7.71E-13
HSPB1	1.22E-15	1.531340971	1	0.959	2.67E-11
HSPE1	1.17E-14	2.247955593	0.871	0.204	2.54E-10
ID3	4.22E-12	-1.220134323	0.643	0.939	9.20E-08
HSPH1	4.37E-12	1.948343875	0.743	0.143	9.52E-08
HSP90AA1	8.67E-12	1.399525975	0.957	0.857	1.89E-07
BAG3	1.31E-11	1.614794524	0.857	0.327	2.85E-07
PNP	5.25E-11	1.591809523	0.829	0.408	1.15E-06
HSP90AB1	7.41E-11	0.911700238	0.986	0.898	1.62E-06
DEFA3	2.92E-10	-0.990122562	0	0.469	6.38E-06
ARID5A	8.93E-10	1.219290721	0.657	0.102	1.95E-05
ADAMTS4	5.17E-09	1.598389912	0.557	0.041	0.000112739
AKAP12	9.08E-09	1.942078816	0.729	0.327	0.000198116
HSPD1	1.26E-08	1.718164924	0.757	0.367	0.000273893
HBB	2.69E-08	-1.078002859	0.043	0.469	0.000587604
MT2A	7.83E-08	2.052225974	0.957	0.878	0.001708043
FOSL1	9.63E-08	0.982443594	0.471	0.02	0.002100706
UPP1	1.11E-07	1.123205079	0.686	0.224	0.002409797
TXNIP	1.47E-07	-0.736905246	0.271	0.776	0.003208749
ADAMTS9	1.65E-07	1.047128062	0.729	0.265	0.00359182
POMP	1.73E-07	0.792609859	0.914	0.653	0.003763668
RBM3	3.32E-07	0.961254477	0.7	0.306	0.007232482
HSPA5	3.71E-07	1.363352615	0.814	0.49	0.008088288
THBD	3.72E-07	1.001180928	0.9	0.551	0.008114964
ENG	4.33E-07	-0.808451785	0.843	0.98	0.00944386
SLCO4A1	5.21E-07	1.103158839	0.6	0.143	0.011352435
IGFBP4	5.29E-07	-0.916966222	0.814	0.918	0.011545722
TSC22D1	6.69E-07	1.038578008	0.886	0.592	0.014580336
ANGPTL4	9.06E-07	0.924067111	0.557	0.102	0.019765757
DNAJB1	9.45E-07	1.22499983	0.9	0.714	0.020614624
TM4SF1	1.10E-06	0.716312971	0.971	0.939	0.023943336
EMP1	1.44E-06	0.877351315	0.9	0.714	0.031498075
PLAUR	1.54E-06	1.117384486	0.471	0.061	0.033580512
MAFF	1.84E-06	0.900023937	0.571	0.143	0.040151065
MRPL33	1.98E-06	0.846398564	0.8	0.551	0.043125624

List of differentially expressed genes of PTGDS_VEC in reperfusion stage (PR versus EP)

gene	p_val	avg_logFC	pct.1	pct.2	p_val_adj
KLF6	5.95E-07	2.42549057	1	0.357	0.012973573
ENG	8.09E-07	-0.871572935	0.96	1	0.017643471
MCL1	1.96E-06	1.884311308	0.96	0.429	0.042805393

Table 12: List of DEGs of endothelial cell clusters in overall stage (PR versus PP)

List of differentially expressed genes of CSTL_LSEC in overall stage (PR versus PP)					
gene	p_val	avg_logFC	pct.1	pct.2	p_val_adj
HSPD1	2.23E-40	2.185527129	0.983	0.49	4.85E-36
HSP90AA1	3.91E-40	1.685568544	1	0.916	8.53E-36
HSPE1	5.49E-39	1.929709606	0.974	0.455	1.20E-34
HSPA1A	1.03E-34	1.59465481	1	0.867	2.25E-30
ID3	2.03E-34	-1.52133684	0.573	0.979	4.44E-30
HSP90AB1	2.52E-33	1.052024613	1	0.972	5.50E-29
LITAF	3.81E-33	1.335206805	0.983	0.657	8.32E-29
TM4SF1	1.17E-32	1.026595615	1	0.979	2.56E-28
PLAUR	1.83E-32	1.3745449	0.795	0.098	3.99E-28
PNP	4.73E-31	1.33352835	0.889	0.35	1.03E-26
EMP1	9.65E-31	1.46514152	0.906	0.28	2.11E-26
HSPH1	1.66E-30	1.74780313	0.855	0.273	3.62E-26
HSPA1B	3.45E-30	1.518793081	0.966	0.685	7.52E-26
HSPB1	1.07E-28	1.503511164	0.991	0.916	2.34E-24
ADAMTS4	8.40E-28	1.305991282	0.872	0.301	1.83E-23
BAG3	1.01E-27	1.376685673	0.915	0.427	2.21E-23
AKAP12	1.42E-27	1.016979489	1	0.958	3.09E-23
DDX21	2.79E-26	1.159462904	0.863	0.455	6.09E-22
FOSL1	4.49E-26	0.916021447	0.641	0.035	9.79E-22
LMNA	8.14E-26	1.119978695	0.889	0.399	1.78E-21
ZFP36L1	4.31E-25	-0.928188178	0.701	0.965	9.39E-21
ADAMTS9	9.13E-25	1.073453882	0.684	0.091	1.99E-20
PHLDA1	3.17E-24	1.012735886	0.932	0.517	6.91E-20
HES1	1.15E-23	-1.174028391	0.239	0.839	2.50E-19
TSC22D3	4.33E-23	-0.805860258	0.65	0.958	9.45E-19
HSPA5	8.73E-23	1.399817077	0.991	0.93	1.90E-18
TSC22D4	1.30E-22	-0.815271154	0.111	0.706	2.83E-18
EVA1C	1.54E-22	0.95843567	0.778	0.266	3.35E-18
PMP22	5.76E-22	1.031012566	0.957	0.692	1.26E-17
MIR4435-2HG	7.04E-22	1.043445439	0.829	0.308	1.54E-17
PLPP3	1.84E-21	0.713891014	1	0.944	4.01E-17
PIM3	3.54E-21	-0.863784895	0.205	0.797	7.72E-17
MYADM	4.65E-21	0.937500118	0.906	0.552	1.01E-16
THBD	4.86E-21	1.080241377	0.915	0.601	1.06E-16
TIMP1	9.67E-21	0.634415361	1	1	2.11E-16
HSPA8	1.34E-20	0.743228911	0.991	0.972	2.93E-16
S1PR1	1.37E-20	0.764575857	0.966	0.825	2.98E-16
SELK	1.92E-20	1.035097763	0.923	0.622	4.20E-16
LDLR	3.20E-20	0.923452838	0.735	0.245	6.97E-16
MARCKS	4.06E-20	0.700227776	0.949	0.748	8.85E-16
NUAK1	6.62E-20	-0.834007986	0.214	0.783	1.44E-15
SDF2L1	1.36E-19	1.001302483	0.786	0.329	2.97E-15
B4GALT5	2.09E-19	0.811518919	0.752	0.266	4.57E-15
IGFBP4	7.29E-19	-0.565194841	1	1	1.59E-14
ATP13A3	1.03E-18	0.803091733	0.598	0.112	2.24E-14
TNFRSF1A	1.28E-18	0.712204696	0.966	0.776	2.79E-14
LINC00152	1.43E-18	0.963206737	0.761	0.336	3.11E-14
LPAR6	2.09E-18	-0.756736115	0.769	0.986	4.56E-14
MESDC1	2.15E-18	0.827137903	0.718	0.252	4.68E-14
RIN2	6.54E-18	0.898886107	0.718	0.273	1.43E-13
ICAM1	1.31E-17	1.157515984	0.94	0.804	2.86E-13
HYAL2	1.32E-17	0.812960047	0.957	0.881	2.87E-13
GIMAP1	1.49E-17	-0.71711802	0.444	0.909	3.25E-13
MCL1	3.14E-17	0.696731141	0.974	0.839	6.85E-13
STIP1	3.90E-17	0.621870142	0.564	0.105	8.50E-13
MT2A	4.60E-17	1.441466125	0.974	0.909	1.00E-12
PDLIM3	5.17E-17	0.832229491	0.726	0.287	1.13E-12
SNHG15	5.26E-17	0.759757631	0.624	0.154	1.15E-12
MCAM	6.34E-17	0.795375789	0.692	0.224	1.38E-12
MYL6	9.56E-17	0.461888817	1	1	2.09E-12
DNASE1L3	2.00E-16	-0.355637827	1	1	4.36E-12
EIF4A1	2.33E-16	0.657972267	0.88	0.643	5.08E-12
SH3BGRL3	3.27E-16	0.643506322	0.949	0.895	7.12E-12
CYCS	3.87E-16	0.699884718	0.769	0.413	8.44E-12
SPHK1	8.38E-16	0.668071946	0.41	0.014	1.83E-11
HNRNPAB	1.18E-15	0.687927422	0.615	0.182	2.57E-11
TNFRSF12A	1.43E-15	0.684425228	0.376	0	3.11E-11

CD59	1.66E-15	0.52920652	0.991	0.993	3.62E-11
NOP16	2.03E-15	0.670963119	0.487	0.063	4.42E-11
TOMM5	3.56E-15	0.693812402	0.821	0.524	7.77E-11
CNKSR3	3.81E-15	0.685602458	0.778	0.427	8.31E-11
TRIB1	5.52E-15	0.748833362	0.803	0.399	1.20E-10
JUNB	6.81E-15	-0.613311168	0.957	0.986	1.48E-10
DDIT4	7.85E-15	-0.826668649	0.632	0.951	1.71E-10
NOLC1	8.08E-15	0.668397276	0.675	0.245	1.76E-10
ARID5A	9.08E-15	0.672026849	0.556	0.147	1.98E-10
SEPP1	9.51E-15	-0.42714606	1	1	2.07E-10
PTPN1	1.33E-14	0.721033401	0.846	0.531	2.89E-10
SAT1	1.41E-14	0.596477642	1	1	3.08E-10
RPS29	1.57E-14	0.42347994	0.991	0.986	3.42E-10
CDC42EP2	1.81E-14	0.73993546	0.607	0.189	3.96E-10
ZYX	2.00E-14	0.631214874	0.855	0.566	4.36E-10
CDC37	2.00E-14	0.549342114	1	0.937	4.37E-10
IL32	2.16E-14	1.094273607	0.65	0.28	4.71E-10
PLK3	2.36E-14	0.741638947	0.581	0.182	5.14E-10
SBNO2	2.41E-14	0.571249753	0.59	0.175	5.25E-10
BZW1	3.10E-14	0.633817503	0.829	0.629	6.77E-10
CLIC1	4.05E-14	0.464038511	0.983	0.965	8.84E-10
GPR4	7.23E-14	0.667156758	0.59	0.196	1.58E-09
PLIN2	8.03E-14	0.676811844	0.974	0.818	1.75E-09
HLA-DRB5	8.38E-14	0.693476884	0.94	0.825	1.83E-09
PNPLA8	9.98E-14	0.663973975	0.641	0.266	2.18E-09
INSIG1	1.00E-13	0.873668543	0.667	0.308	2.18E-09
ENG	1.03E-13	-0.454961468	1	1	2.25E-09
IVNS1ABP	1.12E-13	0.686053699	0.718	0.364	2.45E-09
DUSP6	1.22E-13	0.703945925	0.974	0.839	2.66E-09
MYC	1.42E-13	0.687086609	0.949	0.734	3.09E-09
ARPC5L	1.52E-13	0.595313003	0.778	0.434	3.32E-09
EFNA1	1.84E-13	-0.681891953	0.598	0.909	4.01E-09
NME1	2.12E-13	0.489078164	0.41	0.042	4.63E-09
STARD4	2.22E-13	0.58022429	0.453	0.07	4.83E-09
EHD4	3.09E-13	0.615042195	0.684	0.329	6.74E-09
NOP58	3.41E-13	0.659453442	0.675	0.364	7.44E-09
ZNF593	4.23E-13	0.682249366	0.547	0.168	9.22E-09
CLEC4G	6.05E-13	-0.317741245	1	1	1.32E-08
STX11	6.55E-13	0.488956207	0.342	0.014	1.43E-08
SLC20A1	7.50E-13	0.742843661	0.624	0.266	1.64E-08
MAT2A	9.03E-13	0.653368045	0.675	0.329	1.97E-08
LRRC32	9.19E-13	0.707945107	0.632	0.287	2.00E-08
PVRL2	9.22E-13	0.534420839	0.94	0.853	2.01E-08
PDK4	9.69E-13	-0.692296265	0.761	0.993	2.11E-08
PSAP	1.23E-12	-0.381242165	0.991	1	2.69E-08
SRSF5	1.27E-12	0.631885467	0.94	0.762	2.77E-08
DEFA3	1.38E-12	-0.527524042	0.068	0.476	3.01E-08
RASD1	1.49E-12	1.363213614	0.778	0.503	3.25E-08
GIMAP8	1.68E-12	-0.580084548	0.239	0.692	3.66E-08
XBP1	1.86E-12	0.561408711	0.889	0.622	4.06E-08
CD151	2.06E-12	-0.433541522	0.906	1	4.48E-08
PELO	2.37E-12	0.67387594	0.744	0.441	5.16E-08
TUBA1C	2.59E-12	0.687097169	0.675	0.336	5.65E-08
ACKR3	2.99E-12	0.683044751	0.496	0.112	6.52E-08
DNAJA1	3.15E-12	0.619568761	0.923	0.79	6.87E-08
RHOB	3.39E-12	-0.702418014	0.803	0.958	7.39E-08
PDLIM1	3.65E-12	0.482578309	0.983	0.979	7.97E-08
RAP1B	3.97E-12	0.559728275	0.897	0.818	8.65E-08
YWHAG	4.02E-12	0.558479185	0.564	0.21	8.76E-08
RBM3	5.17E-12	0.511674833	0.932	0.853	1.13E-07
UPP1	6.40E-12	0.631007015	0.923	0.727	1.40E-07
PODXL	7.48E-12	0.611383937	0.47	0.112	1.63E-07
UGCG	8.76E-12	0.625741945	0.932	0.839	1.91E-07
OIT3	1.12E-11	-0.393933005	1	1	2.45E-07
DNAJB6	1.15E-11	0.712226599	0.821	0.629	2.50E-07
RTEL1-TNFRSF6B	1.21E-11	0.743263737	0.325	0.021	2.64E-07
SPRY4	1.89E-11	0.634976763	0.726	0.455	4.12E-07
SNU13	1.90E-11	0.606965036	0.838	0.678	4.15E-07
CD9	2.19E-11	0.729364949	0.974	0.944	4.79E-07
POMP	2.22E-11	0.489930255	0.966	0.881	4.85E-07

SDPR	2.34E-11	-0.464333067	0.974	0.993	5.10E-07
OAF	2.48E-11	0.44449401	0.333	0.028	5.42E-07
RHOC	3.85E-11	0.435292289	0.966	0.93	8.39E-07
GOLT1B	4.40E-11	0.52720921	0.504	0.168	9.59E-07
EIF5A	5.17E-11	0.606178357	0.778	0.636	1.13E-06
INPP1	5.35E-11	0.550243213	0.803	0.552	1.17E-06
SLC12A7	5.53E-11	0.66714136	0.59	0.252	1.21E-06
NCL	5.80E-11	0.578704355	0.889	0.769	1.27E-06
ARL4D	5.85E-11	-0.441768192	0.085	0.462	1.28E-06
CCL14	5.90E-11	-0.280681496	1	1	1.29E-06
KDM6B	5.97E-11	0.69964841	0.624	0.315	1.30E-06
PEA15	5.98E-11	0.584817769	0.735	0.441	1.30E-06
TOP1	6.41E-11	0.560396177	0.803	0.573	1.40E-06
DNMBP	6.53E-11	0.546544461	0.479	0.14	1.42E-06
PDLIM5	7.00E-11	0.729100554	0.701	0.427	1.53E-06
SNRPB	7.74E-11	0.485779055	0.838	0.629	1.69E-06
RSRP1	7.78E-11	-0.492851882	0.53	0.867	1.70E-06
ID1	9.69E-11	-0.558107059	0.872	0.986	2.11E-06
BCL6B	1.01E-10	0.604537022	0.521	0.182	2.20E-06
CLK4	1.14E-10	-0.491321259	0.094	0.462	2.48E-06
TFPI2	1.35E-10	0.885789459	0.906	0.874	2.94E-06
MTFP1	1.43E-10	0.473603029	0.325	0.035	3.11E-06
GIMAP4	1.46E-10	-0.494138146	0.641	0.944	3.17E-06
FLT1	1.58E-10	0.490996751	0.966	0.93	3.44E-06
SMAD6	1.61E-10	-0.50963093	0.06	0.406	3.51E-06
TMSB10	1.65E-10	0.362215109	1	1	3.61E-06
MALL	2.02E-10	0.598984858	0.35	0.049	4.41E-06
UBE2D3	2.03E-10	0.429235045	0.983	0.937	4.42E-06
ZFP36	2.43E-10	-0.617488975	0.957	0.993	5.29E-06
TIMM17A	2.60E-10	0.531309745	0.547	0.217	5.66E-06
FOSL2	2.69E-10	0.547722365	0.641	0.308	5.86E-06
HSPA6	2.75E-10	1.002345327	0.265	0.007	5.99E-06
SSH1	2.78E-10	0.53204347	0.479	0.133	6.05E-06
CEBPZ	3.05E-10	0.471127718	0.624	0.266	6.66E-06
ACER3	3.20E-10	0.620494965	0.709	0.413	6.98E-06
INSR	3.22E-10	0.634009316	0.65	0.385	7.01E-06
YBX3	3.25E-10	0.535185702	0.709	0.427	7.08E-06
YBX1	3.49E-10	0.430648377	0.957	0.916	7.61E-06
INHBB	3.81E-10	0.476521216	0.333	0.042	8.31E-06
TMEM140	4.11E-10	-0.496144993	0.333	0.699	8.97E-06
SOX17	5.08E-10	0.775445013	0.769	0.517	1.11E-05
GJA4	5.38E-10	0.919426054	0.667	0.343	1.17E-05
SRGN	5.39E-10	0.583340171	0.983	0.902	1.18E-05
TEAD4	6.44E-10	0.586805174	0.632	0.329	1.40E-05
DLC1	7.37E-10	0.527483708	0.983	0.881	1.61E-05
NR2F2	8.49E-10	-0.485656143	0.573	0.839	1.85E-05
STAB2	8.49E-10	-0.451592097	0.829	0.986	1.85E-05
SPSB3	8.96E-10	-0.415849582	0.205	0.58	1.95E-05
GIMAP7	9.21E-10	-0.569707833	0.359	0.72	2.01E-05
CCT6A	9.80E-10	0.469353021	0.726	0.455	2.14E-05
SOCS2	1.03E-09	0.512538038	0.88	0.79	2.24E-05
DNAJB11	1.17E-09	0.864002315	0.692	0.51	2.55E-05
HLA-DRB1	1.17E-09	0.45028536	1	0.972	2.56E-05
SLCO4A1	1.32E-09	0.549604369	0.761	0.462	2.88E-05
CD4	1.52E-09	-0.370306109	0.983	1	3.31E-05
ETF1	1.55E-09	0.537006869	0.581	0.252	3.39E-05
MTSS1	1.62E-09	0.583951132	0.684	0.392	3.54E-05
GATA4	1.74E-09	-0.482584572	0.188	0.566	3.80E-05
PRCP	2.31E-09	-0.358957922	0.966	1	5.04E-05
KRBOX4	2.35E-09	-0.375678521	0.085	0.427	5.13E-05
HSP90B1	2.38E-09	0.532359326	1	1	5.19E-05
SLC40A1	2.66E-09	-0.424498257	0.863	0.965	5.81E-05
STOM	2.72E-09	0.38985722	0.983	0.986	5.92E-05
NUDC	3.02E-09	0.471146176	0.692	0.441	6.58E-05
R3HDM4	3.11E-09	0.46439423	0.402	0.105	6.79E-05
DNAJA4	3.26E-09	0.409161521	0.308	0.042	7.10E-05
TCEB1	3.34E-09	0.517544062	0.88	0.79	7.28E-05
FOXN2	3.41E-09	-0.373144001	0.068	0.399	7.44E-05
ANGPTL4	3.79E-09	0.790896336	0.607	0.301	8.26E-05
ID2	3.88E-09	-0.569293282	0.581	0.881	8.46E-05

RHEB	3.88E-09	0.455915792	0.726	0.497	8.47E-05
ITM2B	4.27E-09	-0.289382527	1	1	9.32E-05
ENO1	4.43E-09	0.438504101	0.821	0.678	9.66E-05
GNL3	4.69E-09	0.424484665	0.359	0.077	0.000102346
NAMPT	4.85E-09	0.414380654	0.966	0.93	0.000105805
AHSA1	4.95E-09	0.525562206	0.504	0.203	0.00010795
RAN	5.21E-09	0.578621054	0.761	0.657	0.000113699
TSPAN7	5.65E-09	-0.32793334	0.983	0.993	0.000123166
MANF	5.71E-09	0.714603866	0.701	0.483	0.000124493
ABCE1	5.85E-09	0.41459755	0.427	0.133	0.00012762
NOTCH4	5.90E-09	-0.39861917	0.487	0.839	0.000128707
TMEM37	6.54E-09	-0.524197056	0.598	0.867	0.00014257
SNX5	6.54E-09	-0.407994775	0.846	0.965	0.000142612
PGK1	6.68E-09	0.492085185	0.855	0.699	0.000145681
GABARAP	6.72E-09	-0.297772774	0.974	0.986	0.000146465
PFDN2	6.77E-09	0.59702919	0.735	0.58	0.000147536
SYDE1	7.02E-09	-0.36073069	0.051	0.35	0.00015316
FAM110B	7.17E-09	-0.391894482	0.068	0.378	0.000156381
PHC2	7.32E-09	0.416040895	0.496	0.196	0.00015965
KLF6	7.39E-09	0.498809644	0.966	0.923	0.000161146
UAP1	8.19E-09	0.423641842	0.436	0.14	0.000178593
CCT2	8.54E-09	0.585736388	0.624	0.357	0.000186248
CHKA	8.83E-09	-0.391773972	0.145	0.503	0.000192492
VIMP	9.38E-09	0.517532914	0.829	0.706	0.000204548
MIS18BP1	1.02E-08	-0.356053874	0.179	0.545	0.000222587
PDCD4	1.02E-08	-0.520168656	0.521	0.832	0.000223089
PPRC1	1.03E-08	0.445747344	0.359	0.091	0.000225394
PTGES3	1.05E-08	0.49163997	0.906	0.909	0.000229335
AP2M1	1.15E-08	-0.390393731	0.786	0.951	0.000250725
BCL2L1	1.16E-08	0.529965292	0.59	0.329	0.00025254
STX12	1.20E-08	0.496138075	0.675	0.413	0.000261324
ADAMTS1	1.28E-08	0.644087292	0.838	0.566	0.00028022
KLF9	1.30E-08	0.48147782	0.786	0.622	0.000282445
NXT1	1.31E-08	0.410870517	0.598	0.329	0.000285819
NDRG1	1.32E-08	0.573166981	0.496	0.217	0.000288182
FOS	1.38E-08	-0.517382083	0.991	0.986	0.000300665
ZFAND2A	1.42E-08	0.821723553	0.521	0.245	0.00030877
RASIP1	1.46E-08	0.455045991	0.641	0.336	0.000318993
ETS1	1.50E-08	0.421551536	0.812	0.552	0.00032688
LRRC8C	1.50E-08	0.408227053	0.453	0.161	0.000327505
ZMYM6NB	1.56E-08	-0.413351826	0.393	0.741	0.000340938
CIRBP	1.62E-08	-0.326874003	0.897	0.965	0.000352731
KIAA1551	1.63E-08	-0.455632262	0.231	0.573	0.000354897
CHIC2	1.74E-08	0.584012562	0.778	0.622	0.000379503
ABL2	1.89E-08	0.517621115	0.368	0.098	0.000412112
DDX5	2.11E-08	0.378554741	0.991	0.972	0.000459197
DNAJB1	2.15E-08	0.686117196	0.915	0.888	0.00046952
LUCAT1	2.25E-08	0.411388395	0.248	0.021	0.000489719
CTSD	2.32E-08	-0.347227536	1	1	0.000505288
HBB	2.42E-08	-0.436282984	0.128	0.455	0.000527784
RENBP	2.76E-08	-0.425206451	0.325	0.678	0.00060141
ACP5	2.82E-08	-0.351753889	1	1	0.0006141
PLSCR1	2.90E-08	0.386192973	0.915	0.804	0.000633479
DNAJC19	3.10E-08	-0.38984338	0.12	0.441	0.000676103
CLDN5	3.22E-08	-0.497661993	0.915	0.958	0.000701885
APC	3.28E-08	-0.323449369	0.094	0.413	0.000714755
ARHGAP12	3.41E-08	-0.361138339	0.094	0.399	0.00074397
CDKN1B	3.47E-08	-0.46707349	0.35	0.657	0.000757511
WDR43	3.82E-08	0.461934015	0.376	0.105	0.000833794
SLC25A37	3.98E-08	0.529432411	0.718	0.559	0.000867033
TOB1	4.01E-08	-0.320207433	0.051	0.329	0.000875174
RELB	4.03E-08	0.498350199	0.402	0.133	0.000878033
CTSA	4.10E-08	-0.38668896	0.906	0.979	0.000893416
SERPINB9	4.50E-08	0.525138819	0.658	0.434	0.000981658
FAM60A	4.65E-08	0.440271952	0.368	0.105	0.001014819
UBE2N	4.83E-08	0.458857101	0.65	0.392	0.001053581
MEIS2	4.89E-08	-0.424432089	0.419	0.762	0.001065912
BAZ1A	5.81E-08	0.49335591	0.667	0.392	0.001266807
RAMP3	5.88E-08	-0.333364465	1	1	0.001282542
RHPN2	6.08E-08	-0.291359445	0.026	0.28	0.001326989

EPHX1	6.15E-08	-0.41533546	0.444	0.769	0.001341571
SERPINB1	6.46E-08	0.566303456	0.957	0.888	0.001408882
TSSC4	7.48E-08	0.47582652	0.462	0.182	0.001631902
CREM	7.77E-08	0.580409111	0.487	0.21	0.001693598
LATS2	8.06E-08	0.391617219	0.444	0.147	0.00175765
PNO1	8.43E-08	0.342697303	0.333	0.077	0.00183847
RASSF1	8.51E-08	0.442730991	0.607	0.322	0.001855002
IMP3	9.34E-08	-0.373589684	0.231	0.559	0.002035797
GSPT1	9.67E-08	0.478007041	0.419	0.161	0.002109566
ADRM1	1.09E-07	0.44452932	0.812	0.573	0.002383643
ZNF765	1.10E-07	-0.422796872	0.256	0.594	0.002395326
NUPR1	1.12E-07	-0.436460112	0.615	0.853	0.002444021
SNRPD1	1.13E-07	0.441139892	0.624	0.371	0.002459436
MT1X	1.19E-07	0.996335043	0.684	0.455	0.002591671
IL1RL1	1.25E-07	0.926355946	0.299	0.063	0.002717727
IQCJ-SCHIP1	1.34E-07	0.360062917	0.282	0.049	0.002929982
PHLDB1	1.35E-07	0.37405311	0.41	0.133	0.002937587
KCNH7	1.37E-07	-0.35821061	0.179	0.51	0.002983883
LUZP1	1.37E-07	0.490695127	0.53	0.294	0.002986844
TPM3	1.46E-07	0.340332764	0.88	0.825	0.003189863
MAFF	1.55E-07	0.492436499	0.564	0.259	0.003375486
TUBB6	1.59E-07	0.46334636	0.615	0.413	0.003457414
PAG1	1.63E-07	0.335709199	0.291	0.056	0.003544983
DOT1L	1.69E-07	0.316152276	0.239	0.028	0.003691279
GATA3	1.70E-07	-0.320174155	0.094	0.399	0.003710172
ELF1	1.72E-07	0.436623336	0.769	0.622	0.003760872
CXCL8	1.74E-07	0.734076533	0.444	0.168	0.003797984
AQP3	1.77E-07	0.599049222	0.496	0.252	0.003855039
IDI1	1.77E-07	0.4553994	0.675	0.455	0.003860072
FCGRT	1.79E-07	-0.281594997	0.991	0.986	0.00389895
MIR22HG	1.80E-07	0.42792833	0.573	0.294	0.003929816
GRN	1.88E-07	-0.34140386	0.974	0.993	0.004095421
PSMB8	1.89E-07	-0.370204685	0.436	0.783	0.004125913
SFPQ	2.11E-07	0.391755424	0.88	0.755	0.00459951
TNFSF10	2.31E-07	-0.411166559	0.444	0.762	0.005033504
PIK3C2A	2.60E-07	0.409081929	0.752	0.545	0.005675665
AKAP2	2.62E-07	0.430878005	0.59	0.336	0.005708921
ZCCHC24	2.69E-07	-0.326918837	0.094	0.371	0.005869676
GJA1	2.82E-07	0.356868292	0.205	0.014	0.006159955
G0S2	2.92E-07	0.383429038	0.171	0	0.00635736
MAP2K3	3.06E-07	0.454766111	0.479	0.203	0.006663375
SNRPG	3.08E-07	0.407921144	0.786	0.594	0.006719553
TNFRSF10B	3.10E-07	0.446042149	0.547	0.273	0.006760964
FOSB	3.13E-07	-0.646519723	0.778	0.881	0.006817569
ANKRD26	3.15E-07	-0.280200976	0.017	0.238	0.006862276
EDN1	3.17E-07	-0.70866552	0.291	0.608	0.006912872
GIMAP5	3.36E-07	-0.441791216	0.538	0.804	0.007334549
SLC30A7	3.40E-07	0.439952346	0.556	0.28	0.007425015
B3GNT5	3.42E-07	0.33920567	0.231	0.028	0.007460979
OLFM1	3.50E-07	-0.651462953	0.521	0.79	0.007631956
ITPR1	3.52E-07	0.420287235	0.521	0.245	0.007685517
SOD2	3.62E-07	0.712569577	0.821	0.678	0.007885827
CSF1	3.76E-07	-0.358316313	0.504	0.797	0.008195637
RAB20	3.97E-07	-0.388129735	0.444	0.734	0.008662937
MAF	4.00E-07	-0.351625407	0.855	0.972	0.008722998
MRPL45	4.02E-07	-0.377695978	0.188	0.49	0.008767015
ERP29	4.19E-07	-0.328796901	0.769	0.937	0.009130901
ARID5B	4.23E-07	0.43651607	0.487	0.217	0.009227558
HSPA9	4.26E-07	0.471094045	0.53	0.28	0.009295457
SERPINB8	4.34E-07	0.361348778	0.359	0.112	0.009461615
HERC2	4.57E-07	-0.356418041	0.12	0.406	0.009956225
GADD45A	4.83E-07	0.395249693	0.393	0.126	0.010523045
FGR	5.08E-07	0.330738771	0.256	0.042	0.011074468
HSPG2	5.10E-07	-0.38314901	0.855	0.951	0.011131076
SERPINE1	5.14E-07	0.533889932	0.453	0.203	0.011217991
C8orf4	5.26E-07	0.521174703	0.897	0.741	0.01147719
GRPEL1	5.36E-07	0.449989414	0.47	0.231	0.011685582
VCAM1	5.44E-07	0.778026712	0.444	0.196	0.011865251
CTSB	5.47E-07	-0.277764256	0.94	0.986	0.011939066
SDE2	5.81E-07	0.309184407	0.325	0.084	0.012675062

HNRNPDL	6.09E-07	0.367106424	0.872	0.762	0.013290826
NFE2L2	6.19E-07	0.42453991	0.735	0.524	0.013509921
PTPN12	6.33E-07	0.435306808	0.65	0.455	0.013804463
IP6K2	6.56E-07	-0.309241004	0.085	0.336	0.014313947
GCC2	6.64E-07	-0.367115357	0.282	0.608	0.014486594
MERTK	6.69E-07	-0.330810986	0.231	0.566	0.014589986
EIF2S1	6.86E-07	0.376078818	0.496	0.238	0.014967841
MAT2B	6.94E-07	-0.369712217	0.231	0.538	0.015123898
SOX18	7.15E-07	-0.369220033	0.154	0.434	0.015584119
CDK11B	7.40E-07	0.394651558	0.402	0.147	0.01614399
PML	7.82E-07	-0.349766203	0.197	0.497	0.017047588
CHD9	7.93E-07	-0.441252704	0.308	0.622	0.017285407
CNTNAP3B	8.06E-07	-0.370465735	0.188	0.51	0.017573672
MRC1	8.36E-07	-0.33028082	0.957	1	0.018234233
SHE	8.63E-07	-0.308666708	0.205	0.517	0.018814378
GTPBP4	8.99E-07	0.340791439	0.368	0.126	0.019607799
PNPLA2	9.11E-07	-0.321057118	0.846	0.972	0.019864621
NRROS	9.15E-07	-0.318081383	0.179	0.476	0.019946404
MT-CO3	9.26E-07	-0.262095599	1	1	0.020185389
LYPLAL1	9.35E-07	-0.313945652	0.188	0.476	0.020381971
TUBB	9.72E-07	-0.362378313	0.624	0.811	0.021193078
GIMAP2	9.94E-07	-0.383848222	0.274	0.573	0.021682271
MT-CYB	1.01E-06	-0.349436129	1	0.993	0.022054503
PISD	1.01E-06	0.302604522	0.291	0.07	0.02205816
GCH1	1.02E-06	0.256912463	0.256	0.042	0.022208649
BLCAP	1.04E-06	-0.386845195	0.41	0.748	0.022583322
PCYOX1	1.04E-06	-0.36989512	0.171	0.462	0.022665636
SMAD7	1.15E-06	-0.311196166	0.051	0.287	0.02499442
SLC44A2	1.19E-06	-0.390698803	0.547	0.811	0.025870199
HNRNPU	1.20E-06	0.486043625	0.829	0.72	0.026184649
NFKB2	1.21E-06	0.420390726	0.35	0.112	0.026297109
PCMTD1	1.22E-06	-0.349300236	0.41	0.72	0.026663102
RBM17	1.25E-06	0.380734272	0.65	0.49	0.027208378
ACACB	1.25E-06	-0.294392732	0.068	0.315	0.027297561
RAB8A	1.26E-06	-0.285346083	0.137	0.427	0.027539763
IGLV2-23	1.27E-06	0.252836025	0.205	0.021	0.027786488
HES4	1.31E-06	0.345604045	0.171	0.007	0.028606769
FAM102A	1.32E-06	-0.397293445	0.402	0.685	0.028691544
SH3BP5	1.34E-06	0.396615044	0.872	0.804	0.029296399
LIMD2	1.35E-06	-0.302978784	0.197	0.503	0.029459741
PLXNA2	1.36E-06	0.31557767	0.333	0.098	0.029658359
VAT1	1.39E-06	-0.360633563	0.632	0.804	0.030238993
RPL36A	1.42E-06	0.322763023	0.974	0.972	0.030942947
ETS2	1.46E-06	0.380538225	0.923	0.895	0.031826057
KDM7A	1.46E-06	-0.318385424	0.094	0.35	0.031852976
MIDN	1.48E-06	0.433535174	0.744	0.58	0.032302076
CHMP4B	1.51E-06	0.388514566	0.419	0.175	0.032970216
SLC9A3R2	1.56E-06	0.710794704	0.744	0.622	0.03411566
BIRC3	1.57E-06	0.308616822	0.214	0.028	0.034328528
MTHFD2	1.60E-06	0.39297266	0.376	0.14	0.034857961
PVR	1.65E-06	0.33278918	0.376	0.147	0.035995822
BCL3	1.66E-06	0.402868726	0.462	0.224	0.03620188
BCAR1	1.76E-06	0.344663986	0.419	0.182	0.038371899
KLF2	1.98E-06	-0.381434113	0.402	0.671	0.043129751
SSSCA1	1.99E-06	0.347346044	0.385	0.147	0.043340716
C10orf10	2.03E-06	-0.450486689	0.077	0.322	0.044350613
EGLN2	2.03E-06	-0.335399013	0.282	0.601	0.044353086
KPNA4	2.13E-06	0.406750758	0.47	0.259	0.046348733
CRHBP	2.14E-06	-0.414277835	0.983	1	0.046745164
VMP1	2.15E-06	0.356946332	0.684	0.469	0.046801489
HNRNPK	2.17E-06	0.281849123	0.949	0.937	0.047410017
TTC3	2.20E-06	-0.347913378	0.462	0.755	0.047900442
CLCN3	2.21E-06	-0.322433556	0.171	0.476	0.048300686
IFI44	2.22E-06	-0.279455055	0.145	0.427	0.048369804
JUND	2.29E-06	0.468667693	0.726	0.573	0.049904919

List of differentially expressed genes of BGN_LSEC in overall stage (PR versus PP)

gene	p_val	avg_logFC	pct.1	pct.2	p_val_adj
HSPD1	8.30E-35	2.088918941	0.99	0.535	1.81E-30
ADAMTS9	4.00E-33	1.529633205	0.88	0.101	8.73E-29
HSPA1A	5.44E-33	1.701666716	1	0.884	1.19E-28
HSP90AA1	3.77E-32	1.659353872	0.98	0.922	8.22E-28
HSPE1	2.69E-31	2.105965238	0.96	0.496	5.86E-27
HSPH1	2.99E-31	1.952183816	0.89	0.225	6.52E-27
HSPB1	3.96E-31	1.709702836	1	0.868	8.64E-27
EMP1	4.75E-31	1.623407625	0.99	0.55	1.04E-26
PNP	1.03E-29	1.52550922	0.93	0.395	2.25E-25
BAG3	1.50E-29	1.636761548	0.97	0.465	3.28E-25
HSPA1B	6.85E-29	1.650980732	0.96	0.729	1.49E-24
PLAUR	8.71E-29	1.520651388	0.85	0.147	1.90E-24
LITAF	2.04E-28	1.374793337	0.96	0.628	4.45E-24
ID3	5.21E-27	-1.417857435	0.63	0.992	1.14E-22
HSP90AB1	5.46E-26	1.058778403	1	0.961	1.19E-21
FOSL1	1.07E-25	1.282197406	0.7	0.054	2.32E-21
AKAP12	3.06E-25	1.090210945	0.99	0.915	6.67E-21
TNFRSF12A	3.03E-24	1.09075813	0.63	0.016	6.60E-20
TM4SF1	3.03E-23	0.821864192	1	1	6.60E-19
LMNA	9.86E-23	1.199990045	0.93	0.651	2.15E-18
DDX21	5.60E-22	1.089382441	0.87	0.45	1.22E-17
MYADM	2.09E-20	1.242340957	0.89	0.55	4.55E-16
TSC22D3	4.28E-20	-0.94468095	0.64	0.961	9.33E-16
CDC42EP2	5.31E-20	0.947536964	0.75	0.194	1.16E-15
SRSF5	5.42E-20	0.776035377	0.98	0.822	1.18E-15
THBD	5.77E-20	1.065172435	0.97	0.566	1.26E-15
CD59	7.08E-20	0.668518593	1	0.953	1.54E-15
SELK	9.46E-20	1.050419888	0.89	0.612	2.06E-15
POMP	1.04E-19	0.739871385	0.97	0.868	2.26E-15
MIR4435-2HG	2.40E-19	1.252639044	0.83	0.333	5.24E-15
ADAMTS4	3.48E-19	1.170986798	0.88	0.388	7.60E-15
DDIT4	3.75E-18	-1.091530192	0.75	0.984	8.19E-14
ZFP36L1	5.60E-18	-0.858954967	0.75	0.969	1.22E-13
RHOB	6.41E-18	-1.020748978	0.8	0.977	1.40E-13
HES1	3.26E-17	-1.068574934	0.35	0.829	7.11E-13
LINC00152	3.29E-17	1.0969507	0.78	0.31	7.18E-13
LDLR	3.36E-17	0.896498267	0.75	0.264	7.33E-13
SH3BGRL3	5.10E-17	0.77162606	0.97	0.884	1.11E-12
RIN2	8.82E-17	0.896453626	0.74	0.287	1.92E-12
CYR61	1.15E-16	1.132462739	0.62	0.116	2.52E-12
EFNA1	1.16E-16	-0.872920581	0.57	0.93	2.53E-12
UPP1	1.27E-16	0.872297287	0.9	0.659	2.76E-12
CD9	2.01E-16	0.906980196	0.99	0.953	4.38E-12
INSIG1	2.91E-16	1.112151068	0.78	0.326	6.34E-12
PMP22	4.93E-16	1.078382242	0.85	0.558	1.08E-11
GIMAP8	4.98E-16	-0.775493751	0.22	0.76	1.08E-11
GIMAP4	6.29E-16	-0.776901899	0.57	0.899	1.37E-11
GIMAP1	7.31E-16	-0.852014027	0.39	0.814	1.59E-11
CHIC2	7.74E-16	0.846549735	0.85	0.527	1.69E-11
DEFA3	9.36E-16	-0.692473095	0.03	0.543	2.04E-11
RBM3	1.06E-15	0.784950239	0.94	0.721	2.31E-11
MAT2A	1.13E-15	0.850041712	0.83	0.434	2.47E-11
NOP16	1.32E-15	0.659586168	0.5	0.047	2.88E-11
HES4	2.54E-15	1.015926429	0.5	0.054	5.53E-11
DNAJA4	2.95E-15	0.791480647	0.45	0.023	6.43E-11
RASD1	3.01E-15	1.567852607	0.78	0.419	6.56E-11
CYCS	4.72E-15	0.781044451	0.77	0.364	1.03E-10
DLC1	5.46E-15	0.69947979	0.93	0.853	1.19E-10
TIMP1	6.00E-15	0.946778324	1	0.992	1.31E-10
SLC40A1	6.52E-15	-0.796013838	0.48	0.86	1.42E-10
JUNB	7.03E-15	-0.599912059	0.98	1	1.53E-10
S1PR1	8.52E-15	0.763081041	0.91	0.798	1.86E-10
HSPA8	1.28E-14	0.772402705	0.98	0.977	2.80E-10
MARCKS	1.54E-14	0.854679203	0.84	0.597	3.36E-10
MT2A	1.95E-14	1.724632629	0.96	0.922	4.25E-10
MALL	2.02E-14	0.880472266	0.55	0.101	4.40E-10
MESDC1	2.45E-14	1.00613659	0.62	0.194	5.35E-10
GIMAP7	3.31E-14	-0.757845415	0.32	0.814	7.22E-10
MCL1	4.97E-14	0.669640057	0.98	0.868	1.08E-09

DNAJB6	5.29E-14	0.874792048	0.89	0.698	1.15E-09
ANGPTL4	5.32E-14	0.99815807	0.71	0.256	1.16E-09
PLK3	6.00E-14	0.679312678	0.59	0.132	1.31E-09
KDM6B	7.51E-14	0.861806978	0.68	0.295	1.64E-09
HSPA5	7.68E-14	1.083484726	0.97	0.891	1.67E-09
NUAK1	8.18E-14	-0.707884329	0.14	0.62	1.78E-09
FLT1	1.22E-13	0.731196377	0.94	0.86	2.67E-09
ID1	1.92E-13	-0.73206501	0.88	1	4.19E-09
DUSP6	2.31E-13	0.693310048	0.97	0.876	5.04E-09
TSC22D4	3.10E-13	-0.636514686	0.08	0.543	6.76E-09
PDK4	3.61E-13	-0.808515682	0.6	0.938	7.87E-09
PIM3	4.29E-13	-0.733276204	0.17	0.659	9.34E-09
ACKR3	5.35E-13	0.850583758	0.57	0.163	1.17E-08
SLC20A1	6.11E-13	0.791398095	0.59	0.178	1.33E-08
EIF4A1	6.37E-13	0.695848193	0.82	0.566	1.39E-08
ICAM1	7.67E-13	1.281853415	0.79	0.574	1.67E-08
ENG	9.57E-13	-0.659634853	0.96	0.969	2.09E-08
MCAM	1.22E-12	0.795211198	0.58	0.171	2.66E-08
ATP1A1	1.75E-12	0.740677117	0.83	0.558	3.81E-08
RAP1B	1.84E-12	0.701069174	0.91	0.729	4.02E-08
SPHK1	2.06E-12	0.539744464	0.38	0.023	4.49E-08
TRIB1	2.55E-12	0.765852897	0.74	0.295	5.56E-08
HYAL2	2.59E-12	0.840067674	0.95	0.853	5.64E-08
B3GNT5	2.96E-12	0.721592843	0.49	0.101	6.45E-08
SNRPB	3.40E-12	0.633110842	0.84	0.535	7.42E-08
SLCO4A1	3.41E-12	0.752861554	0.66	0.248	7.43E-08
PTPN1	3.59E-12	0.885074734	0.74	0.434	7.82E-08
PNPLA8	3.63E-12	0.681544419	0.63	0.233	7.92E-08
ZYX	4.60E-12	0.852242981	0.74	0.473	1.00E-07
RTEL1-TNFRSF6B	5.80E-12	0.906350066	0.32	0	1.27E-07
CIRBP	5.92E-12	-0.564935915	0.87	0.938	1.29E-07
MAFF	6.11E-12	0.762203281	0.68	0.295	1.33E-07
MYC	6.34E-12	0.71297518	0.97	0.721	1.38E-07
ACTB	7.34E-12	0.537239748	1	0.992	1.60E-07
ARID5A	7.86E-12	0.65632936	0.54	0.155	1.71E-07
EIF5A	8.95E-12	0.749592413	0.79	0.535	1.95E-07
SAT1	9.19E-12	0.607975548	1	0.984	2.00E-07
PHLDB1	9.68E-12	0.577910687	0.5	0.109	2.11E-07
CDC37	9.80E-12	0.587502714	0.93	0.829	2.14E-07
SOX17	9.82E-12	0.722243266	0.92	0.643	2.14E-07
KLF6	1.02E-11	0.701771875	0.97	0.837	2.23E-07
ISG20	1.22E-11	0.590803887	0.38	0.031	2.66E-07
SPRY4	2.01E-11	0.763784811	0.77	0.442	4.38E-07
EIF1	2.43E-11	0.281232505	1	1	5.29E-07
ARHGAP23	2.46E-11	0.531485551	0.51	0.124	5.36E-07
GIMAP2	3.06E-11	-0.636793973	0.19	0.589	6.68E-07
PLIN2	3.64E-11	0.696099015	0.87	0.76	7.95E-07
VCAM1	4.24E-11	1.318530433	0.56	0.202	9.26E-07
PVRL2	4.48E-11	0.617195493	0.87	0.783	9.78E-07
DLL4	5.51E-11	-0.638532531	0.27	0.682	1.20E-06
SSH1	5.72E-11	0.724475731	0.49	0.109	1.25E-06
CLIC1	5.79E-11	0.528279364	0.97	0.907	1.26E-06
RPS29	5.94E-11	0.400197676	0.98	0.961	1.30E-06
ADAMTS1	6.05E-11	0.788307567	0.9	0.721	1.32E-06
PHLDA1	6.39E-11	1.056587825	0.6	0.209	1.39E-06
HSPG2	7.77E-11	-0.628232637	0.76	0.946	1.70E-06
BZW1	9.09E-11	0.686871564	0.79	0.589	1.98E-06
RELB	9.55E-11	0.543456138	0.46	0.101	2.08E-06
EHD4	9.64E-11	0.612580871	0.69	0.341	2.10E-06
CDKN1B	1.00E-10	-0.557476297	0.25	0.674	2.18E-06
TCEB1	1.21E-10	0.605789892	0.89	0.69	2.64E-06
RHOC	1.24E-10	0.481868508	0.97	0.907	2.70E-06
FGL2	1.34E-10	0.553903936	0.43	0.078	2.91E-06
SDPR	1.46E-10	-0.552084136	0.87	0.969	3.18E-06
PDLIM3	1.68E-10	0.72544009	0.63	0.264	3.67E-06
ID2	1.86E-10	-0.793581505	0.59	0.86	4.05E-06
HBB	2.23E-10	-1.373857725	0.14	0.566	4.87E-06
CXCL8	2.37E-10	1.378875894	0.45	0.101	5.18E-06
RHEB	2.68E-10	0.556668467	0.77	0.442	5.85E-06
GPC1	2.69E-10	0.455527408	0.31	0.016	5.87E-06

TOMM5	2.82E-10	0.659221249	0.77	0.55	6.16E-06
TMSB10	2.90E-10	0.347477404	1	1	6.34E-06
PPRC1	3.13E-10	0.547649089	0.39	0.062	6.82E-06
FAM212A	3.30E-10	-0.407448257	0.01	0.349	7.20E-06
GADD45A	3.42E-10	0.596499366	0.63	0.279	7.45E-06
RSRP1	3.85E-10	-0.605926674	0.58	0.868	8.40E-06
SH3BP5	4.07E-10	0.59966081	0.87	0.682	8.87E-06
IGFBP4	4.39E-10	-0.411182124	0.97	0.992	9.58E-06
ITM2B	5.29E-10	-0.34303133	1	1	1.15E-05
WDR43	5.48E-10	0.478287405	0.42	0.078	1.20E-05
JUND	5.70E-10	0.572096706	0.84	0.597	1.24E-05
IL32	5.74E-10	0.997204841	0.53	0.202	1.25E-05
TNFAIP3	5.90E-10	1.088049975	0.72	0.496	1.29E-05
SMAD6	6.21E-10	-0.540313581	0.11	0.488	1.35E-05
SNU13	6.32E-10	0.561946323	0.85	0.729	1.38E-05
ANKRD55	6.76E-10	-0.498859283	0.11	0.496	1.47E-05
NPY1R	7.75E-10	-0.620759493	0.4	0.76	1.69E-05
DOT1L	8.06E-10	0.541101351	0.3	0.016	1.76E-05
MEIS2	8.33E-10	-0.565094089	0.33	0.69	1.82E-05
LRRC59	8.93E-10	0.535972221	0.55	0.194	1.95E-05
MSN	9.03E-10	0.59435114	0.78	0.574	1.97E-05
SC5D	1.15E-09	-0.666436724	0.57	0.845	2.51E-05
YWHAH	1.28E-09	0.580609785	0.79	0.543	2.80E-05
STARD4	1.33E-09	0.564288113	0.38	0.062	2.90E-05
SNHG15	1.33E-09	0.560924787	0.64	0.279	2.90E-05
DNAJB1	1.40E-09	0.643934018	0.96	0.891	3.05E-05
EVA1C	1.50E-09	0.636098816	0.69	0.388	3.27E-05
SOCS2	1.64E-09	0.639548736	0.84	0.605	3.58E-05
MYL6	1.76E-09	0.411656688	0.98	0.992	3.85E-05
STOM	1.90E-09	0.463091869	0.97	0.984	4.14E-05
NME1	2.06E-09	0.548760872	0.42	0.093	4.49E-05
SBNO2	2.12E-09	0.465461225	0.45	0.109	4.62E-05
ARID5B	2.65E-09	0.626429692	0.67	0.318	5.78E-05
LATS2	2.94E-09	0.455395305	0.52	0.155	6.41E-05
CLDN5	2.97E-09	-0.629634913	0.84	0.984	6.48E-05
RUNX1T1	3.44E-09	-0.505433084	0.06	0.395	7.51E-05
STX11	3.46E-09	0.436271455	0.28	0.016	7.55E-05
TSSC4	3.59E-09	0.650609238	0.51	0.171	7.82E-05
PHF3	4.54E-09	-0.503783255	0.19	0.566	9.91E-05
C11orf96	4.56E-09	0.660624499	0.82	0.558	9.93E-05
MIR22HG	5.17E-09	0.600402217	0.67	0.364	0.000112819
KCNH7	5.85E-09	-0.470146665	0.1	0.457	0.000127668
RFX2	6.18E-09	0.335351263	0.26	0.008	0.000134666
GPR4	6.62E-09	0.627433846	0.55	0.209	0.000144322
SLC12A7	7.12E-09	0.629010725	0.52	0.186	0.00015528
GOLT1B	7.57E-09	0.498668287	0.46	0.132	0.000165139
DNMBP	9.94E-09	0.444366224	0.41	0.093	0.000216869
SPSB3	1.02E-08	-0.425967461	0.13	0.496	0.000222673
NOP58	1.04E-08	0.540981005	0.64	0.333	0.000227564
CDK17	1.07E-08	0.599814659	0.73	0.419	0.00023422
IQCJ-SCHIP1	1.09E-08	0.428365362	0.38	0.078	0.000236847
SLC25A37	1.12E-08	0.552884397	0.74	0.434	0.000245305
GJA1	1.14E-08	0.640488636	0.38	0.085	0.000249649
VMP1	1.34E-08	0.483559294	0.65	0.318	0.000293061
SYNJ2	1.45E-08	0.378242087	0.31	0.039	0.000315258
RAN	1.50E-08	0.532301725	0.81	0.574	0.000328187
AP2M1	1.62E-08	-0.44327247	0.65	0.922	0.000352977
AP1B1	1.63E-08	-0.402450552	0.16	0.527	0.000355965
RTN4	1.66E-08	0.492072825	0.86	0.837	0.000362006
PNO1	1.72E-08	0.432539876	0.32	0.047	0.000374345
SOX18	1.73E-08	-0.591136198	0.16	0.519	0.000377816
TEAD4	1.81E-08	0.600675562	0.64	0.341	0.000394305
UAP1	1.88E-08	0.494666223	0.41	0.101	0.000409757
UGCG	2.16E-08	0.573132471	0.86	0.69	0.000471555
TUBB6	2.17E-08	0.627726797	0.59	0.302	0.000473145
RPL36A	2.27E-08	0.345364587	0.96	0.953	0.00049502
SLC4A7	2.47E-08	0.343889145	0.26	0.016	0.00053893
YBX3	2.56E-08	0.576744458	0.8	0.558	0.000558762
HNRNPAB	2.61E-08	0.597095224	0.55	0.233	0.000568666
MAF	2.63E-08	-0.460992606	0.74	0.891	0.000573518

CREM	2.65E-08	0.506969481	0.55	0.225	0.000577527
FLNA	2.73E-08	0.494994945	0.39	0.085	0.000595507
B4GALT5	2.77E-08	0.504602961	0.63	0.271	0.000603614
PGK1	2.79E-08	0.546811197	0.84	0.667	0.000608699
NR2F2	2.83E-08	-0.510838313	0.51	0.829	0.000617674
SRGN	2.86E-08	0.547345249	0.99	0.977	0.000624335
NOLC1	2.93E-08	0.572304916	0.62	0.287	0.00063927
FSTL3	3.06E-08	0.839043782	0.27	0.023	0.000668311
WARS	3.38E-08	0.508513682	0.87	0.69	0.000736879
HSPA6	4.52E-08	1.007059067	0.27	0.023	0.000986377
PLXNA2	4.63E-08	0.420262579	0.35	0.07	0.001008635
TINAGL1	4.65E-08	0.395915062	0.97	0.961	0.001013781
BAZ1A	4.90E-08	0.555564045	0.65	0.326	0.001067947
TMEM140	5.30E-08	-0.523293713	0.26	0.589	0.001155245
INPP1	5.34E-08	0.581070766	0.73	0.574	0.001164353
CYP20A1	5.61E-08	-0.522745868	0.16	0.473	0.001223952
TMEM204	5.71E-08	-0.45487911	0.37	0.744	0.001245959
LRRC32	5.82E-08	0.652643392	0.71	0.403	0.001268239
NEK3	5.91E-08	-0.365168018	0.07	0.395	0.001288522
MANF	5.97E-08	0.468293391	0.72	0.488	0.001302638
SRM	5.99E-08	0.338379531	0.23	0.008	0.001306047
PDLIM1	6.09E-08	0.397853393	0.99	0.938	0.001328876
MORF4L2	6.43E-08	0.547512849	0.77	0.535	0.001402643
SEC61G	6.46E-08	0.467895434	0.91	0.806	0.001408518
C10orf10	6.52E-08	-0.750724525	0.13	0.45	0.001422104
ACTG1	6.79E-08	0.478447918	0.98	1	0.001479737
ZFP36	6.90E-08	-0.584271371	0.98	1	0.001504797
DNAJB11	7.13E-08	0.651072731	0.62	0.372	0.00155512
ARPC5L	8.34E-08	0.55729156	0.7	0.403	0.001817743
KLF2	8.72E-08	-0.615550321	0.46	0.791	0.001901303
FOS	8.77E-08	-0.567730626	0.98	1	0.001912702
CRHBP	8.92E-08	-0.501158201	0.99	0.992	0.001944832
OIT3	9.09E-08	-0.520961689	0.88	0.946	0.001982873
HADHB	9.33E-08	-0.411609197	0.24	0.597	0.002034933
ATP13A3	9.38E-08	0.528318798	0.64	0.279	0.00204657
NRP1	9.64E-08	-0.4396989	0.57	0.806	0.002103267
CHKA	1.03E-07	-0.376066097	0.06	0.364	0.002237308
NIP7	1.03E-07	0.405728004	0.39	0.101	0.0022378
SAT2	1.03E-07	-0.423634986	0.49	0.822	0.002245735
ERP29	1.08E-07	-0.436227075	0.56	0.837	0.002350954
C8orf4	1.10E-07	0.561298414	0.91	0.736	0.0023935
HLA-E	1.12E-07	-0.279241051	1	1	0.002440188
REEP3	1.13E-07	0.520493435	0.72	0.465	0.00246169
PPARD	1.19E-07	0.331749547	0.29	0.039	0.002594912
TMEM37	1.25E-07	-0.478227136	0.4	0.705	0.002736765
MTRF1L	1.28E-07	0.513478421	0.63	0.318	0.002783075
TXNIP	1.28E-07	-0.543736766	0.24	0.574	0.002799667
CLK4	1.30E-07	-0.379105522	0.06	0.357	0.00284545
PDCD4	1.42E-07	-0.553445841	0.57	0.829	0.003104329
ETS2	1.48E-07	0.385944772	0.96	0.907	0.003230407
ENO1	1.49E-07	0.488281862	0.75	0.535	0.003258207
SEMA6A	1.55E-07	-0.496359517	0.52	0.829	0.003371742
INHBB	1.59E-07	0.559194028	0.32	0.062	0.003475441
ZFAND2A	1.75E-07	0.906511911	0.53	0.256	0.003809863
DDIT3	1.75E-07	-0.488481594	0.26	0.597	0.00381454
TNFRSF1A	1.80E-07	0.424255315	0.9	0.806	0.003921959
CTNNB1	1.91E-07	0.522901873	0.74	0.543	0.00416574
NAMPT	1.93E-07	0.461019696	0.96	0.822	0.004209072
BCL2L1	1.94E-07	0.539139597	0.5	0.225	0.004232856
STC1	1.99E-07	0.801893176	0.29	0.047	0.004338798
NUPR1	2.06E-07	-0.481441916	0.51	0.752	0.004483159
SERPINB9	2.07E-07	0.5299533	0.74	0.488	0.004516161
SNN	2.12E-07	0.456284715	0.5	0.178	0.004624941
SDF2L1	2.14E-07	0.674864422	0.59	0.326	0.004671893
F8	2.31E-07	-0.41268898	0.83	0.969	0.005037047
UBE2N	2.34E-07	0.478392022	0.71	0.442	0.005105182
EDN1	2.38E-07	-0.837322632	0.24	0.581	0.005180504
STAB2	2.39E-07	-0.708370253	0.47	0.713	0.005219807
ST6GAL1	2.45E-07	-0.424702129	0.46	0.767	0.005335907
NRROS	2.47E-07	-0.401652796	0.06	0.349	0.005391512

ART4	2.67E-07	-0.405042612	0.26	0.581	0.005815624
NOS3	2.73E-07	0.732245791	0.63	0.349	0.005943117
CREB5	2.89E-07	0.351512382	0.23	0.016	0.006307168
LRRC8C	2.91E-07	0.553956245	0.43	0.147	0.006347153
SLFN5	2.98E-07	-0.446501243	0.08	0.372	0.006492106
INSR	3.02E-07	0.52646315	0.68	0.38	0.006575845
TFPI2	3.02E-07	0.646761151	0.91	0.798	0.006580709
MAP1S	3.04E-07	0.553366472	0.51	0.209	0.006637178
OAF	3.18E-07	0.348109676	0.26	0.031	0.006937237
LPAR6	3.18E-07	-0.400924935	0.84	0.899	0.006938591
YWHAG	3.49E-07	0.488459658	0.57	0.279	0.007610119
ETF1	3.56E-07	0.545891235	0.5	0.225	0.007765188
STRAP	3.58E-07	0.439945951	0.75	0.535	0.007800772
TMEM245	3.63E-07	-0.403588656	0.09	0.38	0.007923668
PLPP3	3.69E-07	0.371567307	0.99	0.984	0.008057589
MLLT10	3.97E-07	-0.397636946	0.07	0.357	0.00866071
FAM177A1	4.07E-07	0.438745116	0.64	0.372	0.008864952
SEPP1	4.09E-07	-0.40234412	0.98	0.984	0.008908881
TNFSF10	4.39E-07	-0.444976321	0.5	0.783	0.009579856
SMAD7	4.66E-07	-0.396732283	0.07	0.349	0.010168644
LIMS2	4.68E-07	-0.503097317	0.48	0.767	0.010196327
ACACB	4.86E-07	-0.422109039	0.08	0.364	0.010606839
GCC2	4.93E-07	-0.437787931	0.15	0.45	0.010744433
AQP3	4.93E-07	0.490519357	0.46	0.155	0.010757719
YES1	4.99E-07	0.47952932	0.82	0.612	0.010888462
TSPAN7	5.26E-07	-0.352771942	0.97	0.984	0.011464054
NFKB1	5.39E-07	0.413546467	0.38	0.116	0.01175885
TNFRSF10A	5.65E-07	0.299456317	0.27	0.039	0.012327753
PNPLA2	5.73E-07	-0.435655022	0.74	0.868	0.012498913
PTGES3	5.92E-07	0.473672828	0.97	0.876	0.012900158
FCHO2	6.17E-07	-0.449884265	0.23	0.519	0.013445838
ARHGDIB	6.42E-07	-0.365863576	0.87	0.946	0.014003119
3-Mar	6.48E-07	0.517746565	0.62	0.333	0.014137849
FCGRT	6.54E-07	-0.273909117	0.97	0.984	0.01426884
BCAR1	6.57E-07	0.462761554	0.49	0.202	0.014338164
MT1X	6.79E-07	1.535102862	0.59	0.326	0.014798221
VAPA	7.00E-07	0.429643153	0.93	0.845	0.015262421
HLA-A	7.41E-07	-0.266784662	0.99	1	0.016159385
ZFYVE21	8.22E-07	-0.371753129	0.18	0.512	0.017936135
AHSA1	8.39E-07	0.506877155	0.47	0.194	0.018304954
ADGRG1	8.71E-07	0.486627267	0.69	0.426	0.018983949
COL4A3BP	8.86E-07	-0.438365834	0.26	0.574	0.019311964
CTSD	8.96E-07	-0.387011821	0.99	0.992	0.019550533
SOD2	9.63E-07	0.652668943	0.78	0.636	0.020995086
NFKBIZ	9.67E-07	-0.469113811	0.47	0.806	0.021090594
HSPA9	9.89E-07	0.423702907	0.53	0.256	0.021572448
TPM3	1.07E-06	0.379220681	0.97	0.853	0.023364437
SHE	1.10E-06	-0.344523866	0.2	0.527	0.023971047
PSMB8	1.16E-06	-0.412377432	0.44	0.713	0.025270983
SFPQ	1.18E-06	0.397817844	0.84	0.643	0.025732053
AP1S2	1.18E-06	-0.388667294	0.69	0.876	0.025765176
HIF1A	1.20E-06	0.429127373	0.89	0.907	0.026092043
MAP3K13	1.25E-06	0.460205332	0.64	0.395	0.027359078
PTPRB	1.27E-06	-0.395005247	0.71	0.938	0.027767809
RASIP1	1.31E-06	0.447428215	0.61	0.326	0.028661301
MIS18BP1	1.33E-06	-0.369405015	0.09	0.364	0.028950381
ZSWIM6	1.34E-06	0.441784825	0.43	0.163	0.029257715
KPNA4	1.39E-06	0.420136066	0.41	0.155	0.030291946
EHBP1	1.46E-06	-0.351374221	0.14	0.426	0.031901103
USP12	1.53E-06	0.390343073	0.39	0.124	0.033332801
CALCOCO2	1.58E-06	-0.403930335	0.46	0.752	0.034495731
VIMP	1.63E-06	0.541997568	0.76	0.643	0.035652481
PSAP	1.69E-06	-0.343262406	0.9	0.984	0.03678186
MTHFD2	1.69E-06	0.399930481	0.37	0.116	0.036827537
MLST8	1.77E-06	-0.322025467	0.07	0.326	0.038573156
SRP19	1.82E-06	0.326844942	0.33	0.085	0.039787917
VPS13C	1.82E-06	-0.402837183	0.22	0.543	0.039793063
CACYBP	1.87E-06	0.530753003	0.66	0.442	0.04088691
ZNF680	1.91E-06	-0.285317672	0.03	0.271	0.041583351
C2orf88	1.96E-06	-0.480435568	0.23	0.519	0.042699233

ATP5G2	1.96E-06	-0.261277826	0.94	0.992	0.042786145
KIAA1551	2.09E-06	-0.474792522	0.18	0.465	0.045581568
F2R	2.14E-06	-0.350503801	0.82	0.938	0.046702892
NMRK1	2.15E-06	-0.35225336	0.48	0.752	0.046862637
PSMB8-AS1	2.19E-06	-0.346848641	0.08	0.341	0.047673336
CHD9	2.20E-06	-0.392615313	0.33	0.643	0.048014898
KLF10	2.21E-06	-0.43450061	0.29	0.597	0.048216989
TES	2.22E-06	0.350230879	0.3	0.062	0.048478435
UNG	2.28E-06	-0.334251322	0.08	0.341	0.049644078

List of differentially expressed genes of STAB1_LSEC in overall stage (PR versus PP)

gene	p_val	avg_logFC	pct.1	pct.2	p_val_adj
HSPD1	1.55E-25	1.856792714	0.837	0.255	3.39E-21
ADAMTS9	6.84E-25	1.847931712	0.63	0.014	1.49E-20
HSP90AB1	1.87E-23	1.215460421	0.967	0.567	4.08E-19
HSPE1	5.36E-23	1.668444	0.848	0.305	1.17E-18
PNP	2.40E-21	1.559310784	0.652	0.085	5.23E-17
AKAP12	2.70E-21	1.126916888	0.978	0.688	5.88E-17
ID3	1.07E-20	-1.536195193	0.446	0.901	2.34E-16
PLAUR	1.13E-20	1.407189247	0.522	0.007	2.46E-16
HSPB1	3.51E-19	1.314288206	0.967	0.688	7.66E-15
HSP90AA1	3.77E-19	1.327018006	0.924	0.667	8.22E-15
TM4SF1	1.74E-18	1.192017113	0.989	0.801	3.80E-14
EMP1	3.49E-18	1.622302037	0.685	0.156	7.62E-14
MIR4435-2HG	1.40E-15	1.369610257	0.554	0.092	3.04E-11
TIMP1	1.90E-15	1.074071284	0.989	0.936	4.15E-11
MT2A	6.33E-15	1.779501713	0.967	0.83	1.38E-10
CD9	3.80E-14	0.869911758	0.935	0.702	8.28E-10
ADAMTS4	5.60E-14	1.401031678	0.609	0.163	1.22E-09
LMNA	2.30E-13	1.319408128	0.576	0.149	5.02E-09
DEFA3	2.94E-13	-1.414195205	0.043	0.511	6.42E-09
UPP1	3.16E-13	1.149289669	0.685	0.277	6.90E-09
DDX21	7.10E-13	1.110903172	0.587	0.163	1.55E-08
DDIT4	1.01E-12	-1.218658701	0.359	0.766	2.20E-08
THBD	1.33E-12	1.060685423	0.663	0.213	2.90E-08
MIR222HG	2.31E-12	1.040272098	0.467	0.071	5.03E-08
TOMM5	2.78E-12	0.89904945	0.652	0.241	6.06E-08
MYADM	5.02E-12	1.044030198	0.565	0.156	1.09E-07
INSIG1	6.78E-12	0.989122845	0.467	0.092	1.48E-07
TNFRSF12A	9.92E-12	0.961406385	0.293	0	2.16E-07
MESDC1	2.55E-11	1.114667138	0.457	0.092	5.55E-07
HSPA1A	3.01E-11	0.789685996	0.935	0.652	6.57E-07
JUND	3.12E-11	0.913322468	0.717	0.34	6.81E-07
MCL1	3.19E-11	0.884715257	0.783	0.426	6.95E-07
ACKR3	4.06E-11	0.907155115	0.37	0.043	8.86E-07
PMP22	6.10E-11	0.965434591	0.652	0.277	1.33E-06
HES1	6.27E-11	-1.261392093	0.174	0.603	1.37E-06
HSPA1B	1.23E-10	0.752895443	0.859	0.475	2.68E-06
CD59	1.24E-10	0.698415816	0.88	0.638	2.70E-06
FOSL1	1.31E-10	0.891564931	0.337	0.028	2.85E-06
BAG3	2.63E-10	1.111579006	0.489	0.142	5.72E-06
SBNO2	3.00E-10	0.926187798	0.37	0.05	6.54E-06
LITAF	3.17E-10	0.924639341	0.533	0.177	6.92E-06
MYC	3.18E-10	0.90727645	0.685	0.312	6.94E-06
ANGPTL4	3.38E-10	1.078776985	0.543	0.17	7.36E-06
PVRL2	3.51E-10	0.963350263	0.63	0.277	7.66E-06
LPAR6	4.59E-10	-0.995679375	0.446	0.745	1.00E-05
ENG	5.00E-10	-0.753554195	0.739	0.887	1.09E-05
RSRP1	5.08E-10	-0.9235573	0.239	0.667	1.11E-05
GIMAP7	7.61E-10	-0.930143886	0.163	0.56	1.66E-05
TFPI2	9.04E-10	1.034502591	0.717	0.397	1.97E-05
PDLIM3	1.01E-09	0.862952336	0.38	0.064	2.21E-05
SNHG15	1.05E-09	0.895859422	0.413	0.078	2.30E-05
NOS3	1.20E-09	0.987931543	0.511	0.149	2.61E-05
CIRBP	1.51E-09	-0.829020498	0.457	0.73	3.29E-05
CDC42EP2	1.69E-09	0.918180523	0.38	0.071	3.68E-05
SPHK1	2.24E-09	0.689755965	0.25	0.007	4.88E-05

NOP56	2.24E-09	0.886259968	0.326	0.043	4.88E-05
WDR43	3.82E-09	0.561105049	0.272	0.014	8.33E-05
HSPH1	4.12E-09	1.065840564	0.467	0.135	8.98E-05
VMP1	5.17E-09	1.006608831	0.522	0.177	0.000112675
HBB	6.52E-09	-1.005931272	0.185	0.56	0.000142284
CDC37	6.77E-09	0.785807763	0.826	0.638	0.0001476
SH3BGRL3	7.23E-09	0.784929647	0.793	0.574	0.000157715
NOP16	7.76E-09	0.717613156	0.304	0.035	0.000169161
EIF5A	1.02E-08	0.71489791	0.522	0.199	0.00022186
HES4	1.12E-08	0.881488167	0.25	0.014	0.000245074
ADAMTS1	1.29E-08	0.912785315	0.652	0.305	0.000280272
HLA-DRB1	1.30E-08	0.604225578	0.913	0.667	0.000283219
MT1A	1.33E-08	1.289067049	0.261	0.021	0.000289308
MARCKS	1.47E-08	0.966964293	0.609	0.305	0.000320362
CHIC2	1.59E-08	0.831709535	0.489	0.17	0.000346348
ICAM1	2.03E-08	1.183798483	0.543	0.213	0.000443764
TSSC4	2.11E-08	0.776607509	0.272	0.028	0.000459376
ID1	2.48E-08	-0.824578568	0.641	0.879	0.000540043
RHOB	2.81E-08	-1.040660907	0.489	0.716	0.000613781
LINC00152	2.84E-08	1.014521678	0.467	0.149	0.000619456
MT1X	3.19E-08	1.396316937	0.728	0.461	0.000695241
GADD45A	3.48E-08	0.691853015	0.348	0.064	0.000758679
ARHGAP23	3.76E-08	0.743498683	0.37	0.078	0.000819932
ARID5A	3.93E-08	0.752613401	0.315	0.05	0.000855985
GPR4	4.06E-08	0.686513246	0.337	0.064	0.000886078
MCAM	4.35E-08	0.86160313	0.391	0.099	0.000947947
VCAM1	4.48E-08	1.195472273	0.402	0.106	0.000976251
NAMPT	4.79E-08	0.52182627	0.826	0.546	0.00104356
PHLDA1	6.34E-08	0.974754196	0.457	0.149	0.001382546
ANKRD28	6.50E-08	0.904240134	0.304	0.05	0.00141782
MAT2A	6.60E-08	0.708430246	0.467	0.149	0.001438622
DLL4	8.87E-08	-0.976626643	0.12	0.44	0.001934516
TCEB1	8.99E-08	0.929363321	0.554	0.262	0.001959514
EIF3I	1.07E-07	0.783588628	0.489	0.206	0.002324377
ANXA2	1.12E-07	0.737851865	0.739	0.489	0.00243321
CYCS	1.15E-07	0.876117391	0.413	0.135	0.002498133
B4GALT5	1.34E-07	0.590919075	0.348	0.071	0.002920627
C11orf96	1.40E-07	0.776510777	0.62	0.305	0.003042988
CLIC1	1.69E-07	0.660844716	0.783	0.582	0.003682
S1PR1	2.01E-07	0.821672141	0.609	0.34	0.004376514
DUSP6	2.88E-07	0.724556944	0.761	0.511	0.006290039
TSC22D3	2.93E-07	-0.699018434	0.25	0.596	0.00637961
SAT1	2.95E-07	0.66052038	0.978	0.872	0.006439592
RTEL1-TNFRSF6B	3.16E-07	0.717985829	0.174	0	0.006890308
TEAD4	3.18E-07	0.819501456	0.326	0.078	0.006933439
CYR61	3.24E-07	0.744046613	0.38	0.099	0.007070251
CXCL8	3.73E-07	1.463280254	0.25	0.035	0.008138169
ZFP36L1	4.19E-07	-0.77826407	0.37	0.681	0.009143898
LDLR	4.90E-07	0.853477746	0.337	0.078	0.010689574
RAN	5.36E-07	0.692567981	0.5	0.206	0.011696075
GJA4	5.66E-07	0.884709307	0.489	0.191	0.0123498
RIN2	7.32E-07	0.880436066	0.467	0.206	0.015971221
ZNF593	7.72E-07	0.636406602	0.293	0.057	0.016845687
PLIN2	7.74E-07	0.767733069	0.63	0.355	0.016887927
MYL6	8.07E-07	0.399085573	0.967	0.872	0.017591046
IL32	8.54E-07	0.952242754	0.38	0.121	0.01862278
TOP1	9.13E-07	0.724480474	0.489	0.206	0.019911586
ARPC5L	9.25E-07	0.760459213	0.402	0.135	0.020174291
EFNA1	1.02E-06	-0.631009061	0.25	0.574	0.022241772
PDK4	1.10E-06	-0.800307387	0.533	0.766	0.023960022
PHLDB1	1.13E-06	0.610838259	0.228	0.028	0.024704961
TINAGL1	1.14E-06	0.550335495	0.804	0.61	0.024812234
YBX3	1.14E-06	0.429960224	0.489	0.191	0.024943357
ID2	1.24E-06	-0.914264472	0.359	0.638	0.027036677
SEMA6B	1.32E-06	0.674189644	0.413	0.142	0.02887235
PVR	1.41E-06	0.548160461	0.239	0.035	0.030679323
POMP	1.50E-06	0.564923907	0.793	0.61	0.032618821
PLXNA2	1.53E-06	0.577564217	0.228	0.028	0.033276063
SRGN	1.54E-06	0.674303701	0.848	0.702	0.033688104
PFDN2	1.58E-06	0.744175127	0.489	0.227	0.034453466

SELK	1.71E-06	0.890291522	0.543	0.326	0.037336388
IGLV6-57	1.92E-06	0.471038718	0.152	0	0.041766705
IMP4	1.95E-06	0.578364612	0.217	0.028	0.042533474

List of differentially expressed genes of MGP_VEC in overall stage (PR versus PP)

gene	p_val	avg_logFC	pct.1	pct.2	p_val_adj
EMP1	3.62E-29	1.914544324	0.968	0.512	7.89E-25
AKAP12	2.04E-26	2.261026963	0.912	0.214	4.45E-22
HSPA1A	2.88E-25	1.758810577	0.968	0.631	6.28E-21
LMNA	1.22E-24	1.845296503	0.912	0.464	2.67E-20
PNP	5.18E-24	1.684540987	0.904	0.321	1.13E-19
HSPA1B	1.03E-23	1.746683038	0.936	0.56	2.26E-19
HSP90AA1	2.85E-23	1.720553993	0.928	0.631	6.22E-19
TIMP1	4.92E-23	2.223479316	0.976	0.976	1.07E-18
ADAMTS9	3.65E-22	1.919498989	0.784	0.107	7.97E-18
HSPB1	3.22E-20	1.254329149	0.984	0.952	7.02E-16
ANGPTL4	4.45E-20	1.806999476	0.824	0.274	9.70E-16
HES4	6.04E-20	1.797257778	0.72	0.071	1.32E-15
MYADM	8.85E-20	1.575149714	0.832	0.321	1.93E-15
DDIT4	1.69E-19	-1.274691998	0.656	0.952	3.68E-15
MT2A	1.73E-19	2.249516482	0.976	0.869	3.76E-15
UPP1	5.70E-19	1.495276548	0.8	0.25	1.24E-14
TXNIP	3.98E-18	-1.213454834	0.136	0.702	8.68E-14
HSP90AB1	4.39E-18	0.899210702	0.976	0.917	9.58E-14
TM4SF1	5.87E-18	0.966274572	1	0.976	1.28E-13
DDX21	1.16E-17	1.371157716	0.752	0.19	2.54E-13
MYC	3.35E-17	1.397961172	0.848	0.405	7.31E-13
FOSL1	6.87E-17	1.45954826	0.6	0.024	1.50E-12
SAT1	8.57E-17	1.221982364	0.992	0.821	1.87E-12
THBD	1.08E-16	1.317311397	0.832	0.405	2.36E-12
BAG3	1.37E-16	1.708520776	0.744	0.25	2.98E-12
TSC22D3	1.52E-16	-1.106924921	0.448	0.81	3.31E-12
HSPH1	4.41E-16	1.549751731	0.704	0.214	9.62E-12
ADAMTS4	4.52E-16	1.424992606	0.776	0.25	9.86E-12
ID3	9.85E-16	-0.983341188	0.776	0.976	2.15E-11
PLAUR	1.28E-15	1.468682407	0.656	0.095	2.78E-11
C11orf96	5.73E-15	1.385393289	0.816	0.381	1.25E-10
VCAM1	1.69E-14	1.723387535	0.68	0.19	3.68E-10
JUND	3.55E-14	1.052326916	0.848	0.381	7.74E-10
ID1	3.98E-14	-0.871516119	0.864	1	8.68E-10
ID2	5.75E-14	-0.927926288	0.496	0.857	1.25E-09
MIR4435-2HG	8.62E-14	1.316460441	0.648	0.155	1.88E-09
TNFRSF12A	1.83E-13	1.223342514	0.48	0	4.00E-09
HSPE1	1.88E-13	1.460311716	0.76	0.357	4.11E-09
HSPD1	3.39E-13	1.471960843	0.752	0.417	7.40E-09
MAT2A	5.35E-13	1.243240482	0.696	0.25	1.17E-08
MCL1	6.00E-13	0.99571369	0.816	0.464	1.31E-08
GIMAP4	8.49E-13	-0.941033833	0.344	0.762	1.85E-08
LDLR	9.88E-13	0.993971316	0.528	0.06	2.15E-08
HES1	9.97E-13	-1.003969291	0.232	0.679	2.17E-08
SH3BGRL3	1.04E-12	0.83814979	0.888	0.702	2.26E-08
KDM6B	1.36E-12	1.22657878	0.616	0.167	2.96E-08
ADAMTS1	2.46E-12	1.013967812	0.88	0.595	5.37E-08
ACKR3	3.99E-12	1.101531381	0.52	0.06	8.69E-08
GIMAP7	4.53E-12	-0.886263079	0.168	0.643	9.87E-08
RHOB	4.80E-12	-1.012622324	0.664	0.857	1.05E-07
CYCS	6.05E-12	0.932998018	0.648	0.179	1.32E-07
TCEB1	6.37E-12	1.051512637	0.792	0.476	1.39E-07
MT1X	8.18E-12	2.042205303	0.752	0.405	1.78E-07
LITAF	9.30E-12	1.126552661	0.72	0.369	2.03E-07
PIM3	1.40E-11	-0.811599767	0.112	0.536	3.05E-07
EVA1C	4.18E-11	0.944071531	0.68	0.25	9.11E-07
RNASE1	4.20E-11	-0.624245441	0.896	1	9.16E-07
MT1M	4.27E-11	2.117678159	0.64	0.214	9.30E-07
RALGDS	4.64E-11	1.052394948	0.52	0.095	1.01E-06
CD59	6.14E-11	0.650925628	0.944	0.762	1.34E-06
IL6	1.03E-10	1.883235978	0.408	0.012	2.25E-06

MT1A	1.32E-10	1.953248173	0.488	0.083	2.88E-06
TFPI2	1.39E-10	1.462079162	0.576	0.167	3.03E-06
SERPINB9	1.69E-10	1.087368269	0.632	0.226	3.69E-06
GIMAP1	2.54E-10	-0.97236143	0.24	0.607	5.53E-06
EFNA1	3.02E-10	-0.69895265	0.512	0.833	6.59E-06
WARS	3.69E-10	1.01067293	0.72	0.393	8.05E-06
NOS3	4.89E-10	1.064291914	0.608	0.238	1.07E-05
INSIG1	5.28E-10	1.317962942	0.592	0.214	1.15E-05
JMJD1C	5.40E-10	0.892179056	0.816	0.476	1.18E-05
MECOM	5.45E-10	-0.906608687	0.224	0.607	1.19E-05
CYR61	5.98E-10	1.346888573	0.504	0.095	1.30E-05
DEFA3	6.76E-10	-0.877511258	0.064	0.429	1.47E-05
TRIB1	7.86E-10	0.97576262	0.456	0.071	1.71E-05
KIAA1551	9.04E-10	-0.613203841	0.016	0.31	1.97E-05
ICAM1	9.35E-10	1.623437865	0.64	0.286	2.04E-05
SGMS2	9.86E-10	0.909742807	0.4	0.024	2.15E-05
TUBB6	1.01E-09	0.978852611	0.608	0.238	2.20E-05
HSPA8	1.10E-09	0.800909199	0.888	0.786	2.40E-05
HSPA5	2.01E-09	1.484857708	0.8	0.56	4.38E-05
SBNO2	2.04E-09	1.025162644	0.4	0.036	4.45E-05
CDC42EP2	2.18E-09	0.872792158	0.456	0.083	4.75E-05
RSRP1	2.53E-09	-0.77058974	0.32	0.702	5.52E-05
NEAT1	2.93E-09	0.712599434	0.952	0.857	6.39E-05
HMGA1	3.26E-09	0.894219005	0.392	0.036	7.11E-05
LDB2	3.33E-09	-0.602308653	0.728	0.881	7.26E-05
PLPP3	3.40E-09	0.690932212	0.912	0.786	7.41E-05
FOSL2	4.03E-09	0.826452109	0.48	0.107	8.79E-05
7-Sep	5.13E-09	-0.630549081	0.344	0.714	0.000111957
FLT1	5.30E-09	0.846286032	0.744	0.357	0.000115683
SPHK1	6.92E-09	0.795619034	0.328	0	0.000150925
CHIC2	1.12E-08	0.827293745	0.68	0.321	0.00024494
MIDN	1.28E-08	0.885003512	0.696	0.405	0.000280172
GJA1	1.33E-08	0.915377438	0.36	0.024	0.000289626
EIF1	1.53E-08	0.359818988	1	1	0.000333348
ATP1A1	1.81E-08	0.814813183	0.784	0.56	0.000395514
CCL2	1.98E-08	1.474310084	0.576	0.19	0.000430756
GCC2	2.12E-08	-0.616313916	0.064	0.381	0.000462536
NOP16	2.36E-08	0.869169277	0.432	0.083	0.000514715
FAM212A	2.38E-08	-0.537725756	0.008	0.25	0.000518825
SNRPB	3.00E-08	0.738702924	0.664	0.31	0.000654572
EIF3I	3.07E-08	0.68711924	0.68	0.393	0.000670428
SOX17	3.22E-08	0.962953236	0.664	0.333	0.000703157
NOLC1	3.47E-08	0.753911512	0.512	0.155	0.000757276
SOX4	3.85E-08	0.915639363	0.736	0.536	0.000838877
HBB	4.59E-08	-2.04883427	0.096	0.429	0.001001548
RELB	4.66E-08	0.743696581	0.352	0.036	0.001016373
B2M	4.76E-08	-0.279967437	1	1	0.001038183
TMEM70	4.79E-08	0.834791348	0.528	0.167	0.001044803
PCMTD1	5.81E-08	-0.665111848	0.28	0.631	0.001267672
ARID5A	6.29E-08	0.847703815	0.512	0.179	0.001371318
DUSP6	6.61E-08	0.764911351	0.84	0.631	0.001441838
PDLIM4	6.99E-08	0.892184432	0.328	0.024	0.001523699
TSC22D1	7.12E-08	1.053245574	0.584	0.25	0.001553597
TMEM204	7.45E-08	-0.672470329	0.16	0.488	0.001624956
HNRNPU	7.49E-08	0.823062577	0.704	0.417	0.001633477
NPY1R	7.72E-08	-0.768444131	0.192	0.524	0.00168258
CALHM2	8.45E-08	-0.542549596	0.072	0.369	0.001843526
LINC00152	8.62E-08	0.979422705	0.544	0.226	0.001880851
MALL	8.84E-08	0.866224941	0.496	0.167	0.001928893
SLCO4A1	9.50E-08	0.786196714	0.416	0.083	0.002071385
SNHG7	9.90E-08	-0.561832328	0.68	0.869	0.002158734
BIRC3	1.01E-07	0.983082716	0.368	0.048	0.002209254
STX11	1.11E-07	0.610013834	0.304	0.012	0.002430984
3-Mar	1.16E-07	0.68463299	0.328	0.024	0.002538806
NR2F2-AS1	1.18E-07	-0.597994672	0.04	0.298	0.002565469
GADD45A	1.18E-07	0.761374664	0.592	0.262	0.00256643
S100A6	1.32E-07	0.83170852	0.92	0.881	0.002883991
TCEAL8	1.43E-07	-0.598516395	0.184	0.536	0.003114926
FLRT2	1.43E-07	-0.756132603	0.176	0.5	0.003125826
RUNX1T1	1.54E-07	-0.658769643	0.04	0.298	0.003364324

NAMPT	1.66E-07	0.789552389	0.816	0.631	0.003625971
SDPR	1.73E-07	-0.581524619	0.592	0.857	0.003767679
STC1	1.76E-07	1.212831494	0.512	0.179	0.003839012
SOD2	2.07E-07	1.161873211	0.576	0.286	0.004517174
PLK3	2.40E-07	0.756053373	0.4	0.083	0.005223481
CLEC14A	2.51E-07	-0.555928301	0.776	0.869	0.005466594
NXT1	3.03E-07	0.760771054	0.416	0.107	0.006613906
POMP	3.22E-07	0.60620126	0.848	0.619	0.007024755
PELI1	3.47E-07	0.748414554	0.52	0.179	0.007560034
CXCL8	3.53E-07	1.183620182	0.264	0	0.007706946
EHD4	3.63E-07	0.736041858	0.528	0.214	0.007914361
CLDN5	3.67E-07	-0.713985294	0.504	0.75	0.008005321
ZFP36L1	3.89E-07	-0.713379991	0.512	0.75	0.008476685
CLCF1	3.90E-07	0.71875436	0.288	0.012	0.008504043
HMGB2	4.13E-07	-0.631197458	0.168	0.488	0.009004628
ITGA5	4.15E-07	0.776140465	0.704	0.417	0.009040273
BMP4	4.21E-07	-0.62646022	0.008	0.214	0.00917997
NEDD9	4.30E-07	0.739916631	0.52	0.202	0.009369568
C6orf136	4.31E-07	-0.42475684	0.008	0.214	0.009407844
GPC1	4.56E-07	0.677398635	0.28	0.012	0.009946593
CIRBP	4.60E-07	-0.462860638	0.648	0.857	0.010037431
NR2F2	4.62E-07	-0.550825542	0.552	0.786	0.010084789
CYYR1	4.81E-07	-0.535537519	0.464	0.762	0.010489415
RTEL1-TNFRSF6B	5.19E-07	1.017029628	0.28	0.012	0.011321677
GABARAP	5.32E-07	-0.40046332	0.768	0.917	0.011598877
TINAGL1	5.58E-07	0.549319674	0.864	0.738	0.01217781
ARHGAP23	5.82E-07	0.789043718	0.408	0.107	0.012695735
DLC1	5.99E-07	0.776304166	0.656	0.429	0.013058819
ARHGDIB	6.06E-07	-0.60326666	0.408	0.714	0.013212325
MIR222HG	6.27E-07	0.85960328	0.376	0.083	0.013680922
EGFL7	6.74E-07	-0.449755917	0.672	0.917	0.014690736
RAPGEF5	7.19E-07	0.735116332	0.696	0.417	0.015672947
HBEGF	7.70E-07	0.901357755	0.496	0.19	0.016797235
STOM	7.79E-07	0.520369706	0.896	0.774	0.016992871
LRRC32	8.16E-07	0.74504927	0.68	0.429	0.017799843
RPS4X	8.60E-07	-0.302132103	0.984	0.988	0.018763019
MESDC1	1.06E-06	0.714915123	0.464	0.143	0.023121151
MAFF	1.19E-06	0.843341169	0.424	0.131	0.025874268
SERPINE1	1.21E-06	1.156023107	0.472	0.19	0.026424769
ETS2	1.23E-06	0.628370517	0.784	0.631	0.026819993
BTG1	1.32E-06	-0.559549028	0.528	0.774	0.028687383
ZNHIT1	1.32E-06	-0.539751268	0.312	0.643	0.028888671
EIF3J	1.34E-06	0.611817907	0.392	0.095	0.029164828
INHBB	1.42E-06	0.706102431	0.352	0.071	0.030943008
MTIF3	1.43E-06	-0.557289227	0.072	0.321	0.031224047
CUTC	1.45E-06	-0.442663991	0.032	0.25	0.031617375
NEIL1	1.48E-06	-0.366966959	0.04	0.274	0.032364631
NPDC1	1.54E-06	-0.487208734	0.632	0.869	0.033637517
MIS18BP1	1.54E-06	-0.380448333	0.04	0.274	0.033666061
NMRK1	1.58E-06	-0.532260535	0.152	0.452	0.034358774
AP2A2	1.62E-06	-0.570570787	0.08	0.333	0.035377261
ANXA1	1.64E-06	1.207034239	0.528	0.238	0.035722332
TNFRSF10B	1.66E-06	0.718935961	0.504	0.226	0.036289736
SLC40A1	1.70E-06	-0.533598973	0.312	0.607	0.03714284
KDELR1	1.71E-06	-0.615222354	0.384	0.667	0.037381242
IL33	1.81E-06	-0.386397641	0.848	0.929	0.039466701
HEY1	1.97E-06	-0.628678059	0.136	0.429	0.042863933
ACTB	2.07E-06	0.585559008	0.976	0.929	0.045165389
TNFSF10	2.09E-06	-0.650638625	0.248	0.524	0.045475922
CD93	2.11E-06	0.718565507	0.616	0.345	0.045974908
CYP1B1	2.23E-06	0.879105093	0.304	0.036	0.048678576

List of differentially expressed genes of NUPR1_LSEC in overall stage (PR versus PP)

gene	p_val	avg_logFC	pct.1	pct.2	p_val_adj
HSPA1A	2.01E-16	3.315134076	0.968	0.121	4.39E-12
HBB	5.14E-16	-2.202700453	0	0.606	1.12E-11
HSPA1B	3.88E-14	2.539966557	0.914	0.061	8.47E-10

HSPE1	2.85E-13	2.565153353	0.871	0.121	6.22E-09
HSPB1	1.92E-12	1.774279957	0.957	0.636	4.19E-08
DNAJB1	6.51E-11	2.17147638	0.828	0.121	1.42E-06
DEFA3	1.04E-10	-1.311013185	0.065	0.606	2.26E-06
HSP90AA1	1.77E-10	1.657276449	0.871	0.333	3.86E-06
BAG3	1.16E-09	2.521902717	0.667	0	2.52E-05
HSPD1	1.16E-09	1.952143225	0.774	0.212	2.53E-05
MT2A	7.87E-09	1.980138351	0.882	0.606	0.000171692
CDKN1A	1.29E-08	2.007963575	0.688	0.091	0.000280992
HSPH1	1.09E-07	1.882457249	0.602	0.03	0.002382171
SAT1	2.01E-07	1.183230858	0.86	0.485	0.004387762
IGKV2-24	3.02E-07	-1.194478822	0.011	0.303	0.006575704
MCL1	5.50E-07	1.804918026	0.548	0.03	0.011987457
SDF2L1	2.25E-06	1.742949054	0.473	0	0.049013651

List of differentially expressed genes of RBP7_VEC in overall stage (PR versus PP)

gene	p_val	avg_logFC	pct.1	pct.2	p_val_adj
HSPA1A	3.90E-20	1.995712448	1	0.75	8.50E-16
HSPA1B	5.21E-20	1.966408592	1	0.533	1.14E-15
HSPB1	3.01E-17	1.496569712	1	0.933	6.57E-13
HSP90AA1	2.23E-15	1.674360591	0.957	0.783	4.86E-11
HSP90AB1	2.02E-14	1.084708336	0.986	0.833	4.39E-10
HSPE1	1.08E-13	1.962034451	0.871	0.433	2.36E-09
BAG3	2.14E-13	1.647272269	0.857	0.317	4.66E-09
ID3	2.25E-13	-1.485550627	0.643	0.933	4.90E-09
ADAMTS9	1.38E-12	1.480488468	0.729	0.1	3.00E-08
HSPH1	2.65E-11	1.686477716	0.743	0.25	5.77E-07
HBB	3.61E-11	-1.388883469	0.043	0.567	7.86E-07
PNP	4.64E-11	1.455823458	0.829	0.4	1.01E-06
DEFA3	7.95E-11	-1.007446675	0	0.483	1.73E-06
DDIT4	1.18E-10	-0.983764682	0.7	0.933	2.57E-06
HSPD1	1.42E-10	1.810096127	0.757	0.317	3.09E-06
MCL1	1.66E-10	1.217904034	0.857	0.433	3.62E-06
ARID5A	1.80E-10	1.143167228	0.657	0.117	3.92E-06
EMP1	4.14E-10	1.226969593	0.9	0.667	9.03E-06
AKAP12	8.04E-10	1.815511764	0.729	0.267	1.75E-05
DNAJB1	1.32E-09	1.450443169	0.9	0.633	2.87E-05
SPRY1	4.67E-09	1.399544754	0.8	0.433	0.000101821
DDX5	6.31E-09	0.570200306	0.986	0.867	0.00013765
ADAMTS4	7.53E-09	1.359579421	0.557	0.083	0.000164182
B2M	1.07E-08	-0.366285386	1	1	0.00023424
THBD	1.18E-08	1.099065336	0.9	0.55	0.000258125
INSIG1	1.45E-08	1.224843797	0.629	0.167	0.000316831
FOSL1	1.62E-08	0.95386754	0.471	0.033	0.00035256
MT2A	1.65E-08	2.0250407	0.957	0.817	0.000360121
JUND	4.08E-08	0.895863875	0.843	0.383	0.000890787
ITM2B	6.63E-08	-0.504800607	0.971	1	0.001444987
DDX21	8.26E-08	0.993251432	0.6	0.167	0.001801486
HSPA5	1.21E-07	1.385141655	0.814	0.517	0.002631283
MAFF	2.32E-07	0.867225243	0.571	0.133	0.005048809
TXNIP	2.50E-07	-0.965196219	0.271	0.7	0.005452367
SLCO4A1	2.68E-07	1.108919137	0.6	0.183	0.005845723
PDLIM1	3.02E-07	0.881746464	0.843	0.55	0.006589515
PLAUR	5.27E-07	1.119776483	0.471	0.083	0.011501592
GJA1	5.95E-07	0.860099206	0.614	0.217	0.012973131
PTPRE	7.29E-07	0.735611448	0.471	0.067	0.015890463
ID1	8.63E-07	-0.71230613	0.914	0.967	0.018819461
NDRG1	9.47E-07	0.755915304	0.743	0.35	0.020659984
MIDN	9.84E-07	0.717905191	0.729	0.3	0.021459747
ITPRIP	1.20E-06	0.782370502	0.557	0.15	0.026068294
CDKN1A	1.22E-06	1.036480619	0.8	0.533	0.026514833
RNASE1	1.45E-06	-0.613702273	0.914	0.967	0.031653171
RHOB	1.88E-06	-0.65434824	0.729	0.95	0.04103292
TCEB1	1.89E-06	0.783034315	0.743	0.433	0.041312436
FOSL2	1.96E-06	0.846379115	0.643	0.267	0.042716821
PGS1	2.03E-06	0.839567502	0.486	0.1	0.044255549

List of differentially expressed genes of PTGDS_VEC in overall stage (PR versus PP)

gene	p_val	avg_logFC	pct.1	pct.2	p_val_adj
NA					

Table 13: List of DEGs of endothelial cell clusters in cold preservation stage (EP versus PP)

List of differentially expressed genes of CSTL_LSEC in cold preservation stage (EP versus PP)

gene	p_val	avg_logFC	pct.1	pct.2	p_val_adj
HSPA1A	1.62E-08	0.710079822	0.886	0.867	0.000353832
NR4A1	5.60E-08	0.644171033	0.727	0.517	0.001221756
HSP90AA1	1.90E-07	0.423006152	0.932	0.916	0.004138503
IGLV2-14	1.92E-06	0.391312504	0.333	0.119	0.041843967

List of differentially expressed genes of STAB1_LSEC in cold preservation stage (EP versus PP)

gene	p_val	avg_logFC	pct.1	pct.2	p_val_adj
HLA-E	3.22E-09	0.525579789	0.967	0.837	7.02E-05
IGLV2-23	7.59E-09	0.809476818	0.246	0.007	0.000165527

List of differentially expressed genes of MGP_VEC in cold preservation stage (EP versus PP)

gene	p_val	avg_logFC	pct.1	pct.2	p_val_adj
NR4A1	1.63E-09	1.042934813	0.836	0.44	3.56E-05
MCL1	2.78E-08	0.818922923	0.822	0.464	0.000606995
ZFP36	1.23E-06	0.615940204	0.986	0.81	0.026794727

List of differentially expressed genes of NUPR1_LSEC in cold preservation stage (EP versus PP)

gene	p_val	avg_logFC	pct.1	pct.2	p_val_adj
IGKV2-24	1.78E-07	-1.122856794	0.018	0.303	0.003881966

Table 14. List of markers information for NK/T cell clusters, related to Figure 5.

	p_val	avg_logFC	pct.1	pct.2	p_val_adj	gene	cluster	cell type
1	1.83E-200	1.13183676	0.523	0.148	3.98E-196	CD8B	1	CD8B_CD8 Tem
2	9.84E-214	1.0841216	0.794	0.377	2.15E-209	GZMK	1	CD8B_CD8 Tem
3	2.88E-120	0.9700973	0.602	0.298	6.28E-116	RGS1	1	CD8B_CD8 Tem
4	1.02E-149	0.85348292	0.71	0.349	2.23E-145	CD8A	1	CD8B_CD8 Tem
5	2.15E-79	0.72331749	0.448	0.213	4.69E-75	COTL1	1	CD8B_CD8 Tem
6	2.73E-73	0.71519443	0.347	0.142	5.96E-69	CD27	1	CD8B_CD8 Tem
7	3.47E-49	0.67694018	0.323	0.155	7.57E-45	CRTAM	1	CD8B_CD8 Tem
8	7.99E-38	0.59918757	0.454	0.274	1.74E-33	XCL2	1	CD8B_CD8 Tem
9	6.77E-63	0.58963336	0.192	0.05	1.48E-58	PDCD1	1	CD8B_CD8 Tem
10	4.60E-35	0.53778914	0.294	0.163	1.00E-30	SRRT	1	CD8B_CD8 Tem
11	2.04E-33	0.53454179	0.445	0.316	4.44E-29	ITM2A	1	CD8B_CD8 Tem
12	8.27E-79	0.53013441	0.718	0.45	1.80E-74	CD3D	1	CD8B_CD8 Tem
13	2.10E-33	0.52754216	0.304	0.166	4.57E-29	HLA-DQA1	1	CD8B_CD8 Tem
14	3.07E-23	0.51070613	0.293	0.181	6.70E-19	HLA-DRA	1	CD8B_CD8 Tem
15	2.64E-21	0.5056277	0.275	0.176	5.75E-17	FABP5	1	CD8B_CD8 Tem
16	1.70E-27	0.49388854	0.44	0.323	3.70E-23	SAMSN1	1	CD8B_CD8 Tem
17	3.54E-25	0.48844868	0.423	0.32	7.73E-21	HLA-DQB1	1	CD8B_CD8 Tem
18	1.68E-35	0.46846607	0.829	0.789	3.67E-31	CD74	1	CD8B_CD8 Tem
19	6.82E-38	0.4620393	0.637	0.506	1.49E-33	HLA-DRB1	1	CD8B_CD8 Tem
20	7.79E-45	0.46026188	0.749	0.671	1.70E-40	SRSF7	1	CD8B_CD8 Tem
21	3.97E-36	0.43574007	0.554	0.419	8.66E-32	HLA-DPB1	1	CD8B_CD8 Tem
22	1.41E-31	0.43571543	0.533	0.42	3.07E-27	HLA-DPA1	1	CD8B_CD8 Tem
23	1.30E-26	0.43394198	0.729	0.661	2.84E-22	DNAJB1	1	CD8B_CD8 Tem
24	3.78E-204	0.42408226	0.995	0.997	8.24E-200	RPS27	1	CD8B_CD8 Tem
25	4.52E-39	0.41638751	0.505	0.335	9.85E-35	CD3G	1	CD8B_CD8 Tem
26	8.66E-15	0.40765658	0.734	0.654	1.89E-10	CCL4L2	1	CD8B_CD8 Tem
27	4.48E-16	0.4031708	0.344	0.258	9.76E-12	TSPYL2	1	CD8B_CD8 Tem
28	4.41E-16	0.40156333	0.256	0.172	9.61E-12	TUBB2A	1	CD8B_CD8 Tem
29	1.97E-18	0.40103159	0.385	0.299	4.30E-14	LINC00152	1	CD8B_CD8 Tem
30	3.17E-22	0.39472684	0.279	0.174	6.92E-18	INPP4B	1	CD8B_CD8 Tem
31	1.39E-90	0.39190532	0.969	0.96	3.04E-86	RPS29	1	CD8B_CD8 Tem
32	6.67E-22	0.39065275	0.266	0.161	1.46E-17	THEMIS	1	CD8B_CD8 Tem
33	1.43E-19	0.38825332	0.33	0.214	3.11E-15	XCL1	1	CD8B_CD8 Tem
34	8.86E-18	0.37629238	0.443	0.376	1.93E-13	SH2D1A	1	CD8B_CD8 Tem
35	2.91E-13	0.37200931	0.433	0.379	6.34E-09	KIAA1551	1	CD8B_CD8 Tem
36	5.68E-174	0.35967774	0.998	0.998	1.24E-169	AC090498.1	1	CD8B_CD8 Tem
37	9.96E-11	0.34725909	0.166	0.107	2.17E-06	ID3	1	CD8B_CD8 Tem
38	5.24E-24	0.33307052	0.692	0.632	1.14E-19	CD2	1	CD8B_CD8 Tem
39	1.79E-15	0.33047513	0.341	0.249	3.91E-11	HLA-DRB5	1	CD8B_CD8 Tem
40	3.98E-09	0.31990409	0.179	0.126	8.69E-05	CD5	1	CD8B_CD8 Tem
41	1.15E-50	0.315998	0.921	0.829	2.52E-46	CD3E	1	CD8B_CD8 Tem
42	3.96E-07	0.31314208	0.156	0.107	0.008638803	TRGV2	1	CD8B_CD8 Tem
43	5.01E-11	0.31068727	0.219	0.153	1.09E-06	SIT1	1	CD8B_CD8 Tem
44	5.44E-12	0.31039635	0.523	0.48	1.19E-07	PIK3R1	1	CD8B_CD8 Tem
45	9.88E-13	0.30337384	0.119	0.062	2.16E-08	TNFRSF9	1	CD8B_CD8 Tem
46	2.68E-09	0.3014498	0.151	0.099	5.85E-05	CMTM7	1	CD8B_CD8 Tem
47	2.17E-09	0.30141156	0.136	0.085	4.73E-05	ICOS	1	CD8B_CD8 Tem
48	9.41E-10	0.30100608	0.196	0.136	2.05E-05	ODC1	1	CD8B_CD8 Tem
49	2.61E-08	0.29715128	0.486	0.479	0.00056976	SOD1	1	CD8B_CD8 Tem
50	1.88E-18	0.29549705	0.127	0.057	4.10E-14	SELM	1	CD8B_CD8 Tem
51	1.81E-12	0.29323736	0.576	0.563	3.95E-08	FYB	1	CD8B_CD8 Tem
52	4.39E-10	0.29315348	0.313	0.25	9.58E-06	LYST	1	CD8B_CD8 Tem
53	5.06E-82	0.29000207	0.99	0.985	1.10E-77	RPS25	1	CD8B_CD8 Tem
54	4.06E-31	0.28115149	0.97	0.961	8.86E-27	JUNB	1	CD8B_CD8 Tem
55	1.20E-09	0.28057649	0.165	0.11	2.62E-05	HLA-DMA	1	CD8B_CD8 Tem
56	4.02E-106	0.27760809	0.993	0.997	8.76E-102	RPL30	1	CD8B_CD8 Tem
57	1.03E-16	0.27672189	0.107	0.046	2.26E-12	CXCR3	1	CD8B_CD8 Tem
58	6.97E-104	0.27572755	0.992	0.996	1.52E-99	RPS28	1	CD8B_CD8 Tem
59	1.44E-08	0.27510307	0.441	0.414	0.000313385	RHOH	1	CD8B_CD8 Tem
60	7.08E-07	0.27486952	0.182	0.137	0.015446879	HNRNPLL	1	CD8B_CD8 Tem
61	1.26E-80	0.27317167	0.993	0.988	2.75E-76	RPS18	1	CD8B_CD8 Tem
62	1.30E-105	0.26773151	0.997	0.997	2.84E-101	RPL39	1	CD8B_CD8 Tem
63	2.53E-104	0.26628803	0.999	0.998	5.53E-100	RPL28	1	CD8B_CD8 Tem
64	3.21E-11	0.26261705	0.131	0.075	7.00E-07	PECAM1	1	CD8B_CD8 Tem
65	6.78E-18	-0.25010519	0.297	0.457	1.48E-13	NME2	1	CD8B_CD8 Tem
66	8.11E-18	-0.25145604	0.493	0.652	1.77E-13	LITAF	1	CD8B_CD8 Tem
67	7.40E-12	-0.25294716	0.112	0.202	1.61E-07	ATF3	1	CD8B_CD8 Tem
68	5.92E-14	-0.25399291	0.255	0.384	1.29E-09	C12orf75	1	CD8B_CD8 Tem

69	7.05E-25	-0.25440669	0.696	0.842	1.54E-20	ARPC2	1	CD8B_CD8 Tem
70	1.38E-18	-0.25497641	0.184	0.33	3.01E-14	BANF1	1	CD8B_CD8 Tem
71	1.30E-31	-0.25617367	0.991	0.999	2.84E-27	ACTB	1	CD8B_CD8 Tem
72	2.83E-14	-0.25664238	0.208	0.333	6.17E-10	H2AFV	1	CD8B_CD8 Tem
73	6.25E-21	-0.25686354	0.244	0.422	1.36E-16	P4HB	1	CD8B_CD8 Tem
74	1.08E-19	-0.2593949	0.204	0.359	2.35E-15	KLF2	1	CD8B_CD8 Tem
75	5.07E-28	-0.25993759	0.685	0.848	1.11E-23	RAC2	1	CD8B_CD8 Tem
76	4.06E-16	-0.26233176	0.491	0.646	8.84E-12	SQSTM1	1	CD8B_CD8 Tem
77	7.42E-23	-0.26256045	0.227	0.409	1.62E-18	SCP2	1	CD8B_CD8 Tem
78	4.03E-27	-0.26266945	0.682	0.832	8.78E-23	CLIC1	1	CD8B_CD8 Tem
79	1.25E-52	-0.26303741	0.982	0.996	2.72E-48	TMSB10	1	CD8B_CD8 Tem
80	2.71E-18	-0.27176077	0.182	0.321	5.90E-14	TPST2	1	CD8B_CD8 Tem
81	3.72E-39	-0.2726667	0.956	0.985	8.11E-35	PFN1	1	CD8B_CD8 Tem
82	6.56E-24	-0.2728041	0.209	0.385	1.43E-19	FKBP11	1	CD8B_CD8 Tem
83	5.06E-18	-0.27359072	0.103	0.22	1.10E-13	YES1	1	CD8B_CD8 Tem
84	1.22E-17	-0.28277313	0.151	0.281	2.67E-13	STARD3NL	1	CD8B_CD8 Tem
85	4.87E-10	-0.28374617	0.78	0.834	1.06E-05	VIM	1	CD8B_CD8 Tem
86	6.47E-25	-0.28449457	0.302	0.511	1.41E-20	BIN2	1	CD8B_CD8 Tem
87	7.47E-20	-0.28626016	0.118	0.251	1.63E-15	ADRB2	1	CD8B_CD8 Tem
88	9.11E-23	-0.28757564	0.534	0.717	1.99E-18	NEAT1	1	CD8B_CD8 Tem
89	8.96E-25	-0.28877862	0.299	0.497	1.95E-20	ARL4C	1	CD8B_CD8 Tem
90	3.71E-24	-0.28953073	0.28	0.47	8.08E-20	AES	1	CD8B_CD8 Tem
91	8.54E-24	-0.29510544	0.14	0.297	1.86E-19	UPP1	1	CD8B_CD8 Tem
92	1.08E-23	-0.29528118	0.255	0.444	2.35E-19	PIM1	1	CD8B_CD8 Tem
93	3.87E-21	-0.29676732	0.107	0.238	8.43E-17	ADAM8	1	CD8B_CD8 Tem
94	3.45E-22	-0.2987168	0.318	0.502	7.53E-18	CTSD	1	CD8B_CD8 Tem
95	1.60E-21	-0.30234759	0.238	0.399	3.50E-17	SPOCK2	1	CD8B_CD8 Tem
96	3.42E-21	-0.30257714	0.26	0.43	7.47E-17	PLEK	1	CD8B_CD8 Tem
97	3.05E-25	-0.30629494	0.12	0.276	6.66E-21	ICAM2	1	CD8B_CD8 Tem
98	7.50E-24	-0.30755481	0.142	0.297	1.64E-19	MAPK1	1	CD8B_CD8 Tem
99	4.71E-44	-0.30819191	0.926	0.969	1.03E-39	ACTG1	1	CD8B_CD8 Tem
100	8.18E-23	-0.31341291	0.256	0.431	1.78E-18	CD63	1	CD8B_CD8 Tem
101	1.03E-23	-0.31483526	0.235	0.411	2.25E-19	SYNE2	1	CD8B_CD8 Tem
102	5.07E-29	-0.31757063	0.28	0.489	1.11E-24	YWHAQ	1	CD8B_CD8 Tem
103	3.15E-23	-0.32103688	0.535	0.695	6.86E-19	CRIP1	1	CD8B_CD8 Tem
104	3.43E-46	-0.32166543	0.888	0.963	7.48E-42	IFITM1	1	CD8B_CD8 Tem
105	3.79E-22	-0.32760947	0.273	0.443	8.26E-18	MAP3K8	1	CD8B_CD8 Tem
106	9.77E-28	-0.3330107	0.187	0.372	2.13E-23	ADD3	1	CD8B_CD8 Tem
107	3.19E-18	-0.33392335	0.251	0.396	6.96E-14	ZEB2	1	CD8B_CD8 Tem
108	4.97E-33	-0.33408875	0.235	0.456	1.08E-28	PTPN6	1	CD8B_CD8 Tem
109	8.26E-30	-0.3381806	0.442	0.647	1.80E-25	EMP3	1	CD8B_CD8 Tem
110	6.07E-37	-0.33843691	0.297	0.547	1.32E-32	OSTF1	1	CD8B_CD8 Tem
111	4.00E-30	-0.3402942	0.232	0.435	8.72E-26	RORA	1	CD8B_CD8 Tem
112	8.98E-21	-0.34487439	0.513	0.679	1.96E-16	PPP1R15A	1	CD8B_CD8 Tem
113	6.95E-29	-0.34572271	0.179	0.365	1.52E-24	TPM4	1	CD8B_CD8 Tem
114	6.89E-31	-0.35280037	0.159	0.344	1.50E-26	DSTN	1	CD8B_CD8 Tem
115	1.95E-29	-0.35337631	0.141	0.313	4.26E-25	ITGB1	1	CD8B_CD8 Tem
116	1.28E-26	-0.35435017	0.218	0.4	2.79E-22	FLNA	1	CD8B_CD8 Tem
117	1.53E-18	-0.35456075	0.77	0.847	3.34E-14	FOS	1	CD8B_CD8 Tem
118	1.23E-38	-0.35811406	0.474	0.715	2.69E-34	RAP1B	1	CD8B_CD8 Tem
119	3.17E-29	-0.35918182	0.232	0.429	6.92E-25	SERPINB1	1	CD8B_CD8 Tem
120	4.06E-34	-0.36066695	0.255	0.479	8.85E-30	PRMT2	1	CD8B_CD8 Tem
121	1.15E-33	-0.36375418	0.442	0.669	2.51E-29	GNG2	1	CD8B_CD8 Tem
122	8.59E-34	-0.36411033	0.179	0.384	1.87E-29	MGAT4A	1	CD8B_CD8 Tem
123	2.08E-06	-0.36677537	0.473	0.571	0.045357322	HMGN2	1	CD8B_CD8 Tem
124	1.35E-37	-0.37086138	0.31	0.551	2.94E-33	S100A11	1	CD8B_CD8 Tem
125	2.13E-24	-0.37120619	0.286	0.459	4.64E-20	TNF	1	CD8B_CD8 Tem
126	7.99E-40	-0.37524832	0.146	0.364	1.74E-35	PTGER2	1	CD8B_CD8 Tem
127	2.74E-34	-0.3851069	0.12	0.305	5.97E-30	GLRX	1	CD8B_CD8 Tem
128	2.00E-34	-0.38535851	0.149	0.345	4.36E-30	GLUL	1	CD8B_CD8 Tem
129	8.99E-22	-0.38597576	0.421	0.57	1.96E-17	GSTP1	1	CD8B_CD8 Tem
130	2.66E-28	-0.38797603	0.112	0.269	5.80E-24	GNPTAB	1	CD8B_CD8 Tem
131	4.04E-22	-0.38840005	0.485	0.622	8.81E-18	CD52	1	CD8B_CD8 Tem
132	1.08E-47	-0.39395837	0.434	0.709	2.35E-43	ANXA1	1	CD8B_CD8 Tem
133	2.63E-41	-0.40523546	0.214	0.449	5.73E-37	CAST	1	CD8B_CD8 Tem
134	2.17E-35	-0.41046709	0.19	0.402	4.73E-31	CD55	1	CD8B_CD8 Tem
135	1.46E-39	-0.41579072	0.259	0.497	3.18E-35	XBP1	1	CD8B_CD8 Tem
136	8.90E-37	-0.41665652	0.136	0.338	1.94E-32	SPN	1	CD8B_CD8 Tem
137	3.52E-31	-0.42959019	0.361	0.551	7.69E-27	TRBC1	1	CD8B_CD8 Tem
138	2.14E-24	-0.43297022	0.183	0.343	4.66E-20	IER3	1	CD8B_CD8 Tem

139	1.92E-23	-0.43842044	0.161	0.309	4.18E-19	KLRC3	1	CD8B_CD8 Tem
140	2.97E-37	-0.4478691	0.39	0.615	6.47E-33	TNFAIP3	1	CD8B_CD8 Tem
141	4.50E-53	-0.457939	0.555	0.792	9.82E-49	CD7	1	CD8B_CD8 Tem
142	1.00E-50	-0.48696337	0.185	0.441	2.19E-46	HMGN3	1	CD8B_CD8 Tem
143	6.18E-61	-0.48744384	0.665	0.827	1.35E-56	S100A6	1	CD8B_CD8 Tem
144	7.00E-48	-0.49928714	0.141	0.373	1.53E-43	SELPLG	1	CD8B_CD8 Tem
145	1.14E-33	-0.50614833	0.268	0.469	2.49E-29	NFKBIZ	1	CD8B_CD8 Tem
146	3.92E-21	-0.53400839	0.184	0.321	8.55E-17	LTB	1	CD8B_CD8 Tem
147	1.64E-53	-0.54912775	0.179	0.431	3.58E-49	UCP2	1	CD8B_CD8 Tem
148	4.99E-14	-0.55881565	0.307	0.42	1.09E-09	AREG	1	CD8B_CD8 Tem
149	6.83E-15	-0.56602927	0.367	0.506	1.49E-10	TUBB	1	CD8B_CD8 Tem
150	7.76E-61	-0.59413708	0.127	0.391	1.69E-56	BHLHE40	1	CD8B_CD8 Tem
151	1.76E-57	-0.62628795	0.888	0.876	3.85E-53	NKG7	1	CD8B_CD8 Tem
152	4.76E-67	-0.66516142	0.469	0.72	1.04E-62	CTSW	1	CD8B_CD8 Tem
153	1.42E-76	-0.66653378	0.294	0.606	3.09E-72	GPR65	1	CD8B_CD8 Tem
154	2.49E-35	-0.66915314	0.273	0.447	5.42E-31	IL7R	1	CD8B_CD8 Tem
155	4.04E-132	-0.69940204	0.711	0.933	8.81E-128	IFITM2	1	CD8B_CD8 Tem
156	1.59E-137	-0.71562725	0.714	0.907	3.47E-133	S100A4	1	CD8B_CD8 Tem
157	6.10E-85	-0.71748879	0.261	0.586	1.33E-80	HOPX	1	CD8B_CD8 Tem
158	1.11E-80	-0.75814668	0.636	0.825	2.41E-76	NFKBIA	1	CD8B_CD8 Tem
159	6.78E-44	-0.78203616	0.2	0.411	1.48E-39	LGALS1	1	CD8B_CD8 Tem
160	8.66E-108	-0.84572786	0.334	0.677	1.89E-103	CD247	1	CD8B_CD8 Tem
161	6.83E-96	-0.88594973	0.329	0.638	1.49E-91	KLRD1	1	CD8B_CD8 Tem
162	3.45E-69	-0.92117489	0.198	0.469	7.51E-65	SPON2	1	CD8B_CD8 Tem
163	6.06E-142	-1.01055959	0.414	0.779	1.32E-137	PRF1	1	CD8B_CD8 Tem
164	1.40E-112	-1.30936804	0.165	0.513	3.05E-108	GZMB	1	CD8B_CD8 Tem
165	1.32E-192	-1.45309419	0.222	0.711	2.87E-188	KLRB1	1	CD8B_CD8 Tem
166	0	2.06667156	0.988	0.231	0	GNLY	2	GNLY_NK
167	0	1.57464118	0.912	0.187	0	FGFBP2	2	GNLY_NK
168	0	1.41406633	0.879	0.208	0	FCGR3A	2	GNLY_NK
169	0	1.23136858	0.959	0.312	0	GZMB	2	GNLY_NK
170	0	1.20196503	0.963	0.345	0	TYROBP	2	GNLY_NK
171	1.64E-291	1.10325067	0.65	0.152	3.58E-287	ADGRG1	2	GNLY_NK
172	3.88E-273	1.07940635	0.536	0.094	8.47E-269	KIR3DL1	2	GNLY_NK
173	1.36E-290	1.06106999	0.91	0.429	2.97E-286	GZMH	2	GNLY_NK
174	2.31E-289	0.98414041	0.835	0.264	5.03E-285	KLRF1	2	GNLY_NK
175	1.97E-233	0.97446592	0.47	0.082	4.29E-229	IGFBP7	2	GNLY_NK
176	1.13E-161	0.97419879	0.667	0.293	2.47E-157	LGALS1	2	GNLY_NK
177	8.36E-197	0.96937785	0.621	0.193	1.82E-192	KLRC3	2	GNLY_NK
178	3.29E-282	0.96443013	0.965	0.638	7.17E-278	PRF1	2	GNLY_NK
179	0	0.96356373	1	0.848	0	NKG7	2	GNLY_NK
180	9.27E-198	0.95678398	0.749	0.329	2.02E-193	SPON2	2	GNLY_NK
181	6.52E-244	0.91015323	0.936	0.601	1.42E-239	CTSW	2	GNLY_NK
182	2.60E-212	0.90376197	0.848	0.428	5.67E-208	TRBC1	2	GNLY_NK
183	1.13E-248	0.88713392	0.952	0.48	2.47E-244	KLRD1	2	GNLY_NK
184	3.13E-167	0.85402535	0.476	0.125	6.82E-163	SH2D1B	2	GNLY_NK
185	2.05E-182	0.8486114	0.398	0.076	4.47E-178	PRSS23	2	GNLY_NK
186	2.02E-170	0.8466858	0.69	0.285	4.41E-166	ZEB2	2	GNLY_NK
187	3.07E-173	0.83625433	0.398	0.08	6.69E-169	CX3CR1	2	GNLY_NK
188	9.13E-212	0.82778305	0.878	0.538	1.99E-207	CD247	2	GNLY_NK
189	4.63E-156	0.81336666	0.778	0.454	1.01E-151	HOPX	2	GNLY_NK
190	3.33E-134	0.78683633	0.427	0.129	7.26E-130	TTC38	2	GNLY_NK
191	3.99E-133	0.77437487	0.308	0.06	8.70E-129	KIR2DL1	2	GNLY_NK
192	1.21E-129	0.76711727	0.546	0.204	2.64E-125	CLIC3	2	GNLY_NK
193	2.56E-122	0.70546407	0.434	0.136	5.57E-118	FCRL6	2	GNLY_NK
194	1.94E-104	0.67657857	0.603	0.296	4.23E-100	C12orf75	2	GNLY_NK
195	6.54E-211	0.67101852	0.988	0.838	1.43E-206	CST7	2	GNLY_NK
196	3.29E-114	0.66642961	0.408	0.124	7.17E-110	TRDC	2	GNLY_NK
197	1.57E-198	0.65799822	0.989	0.861	3.43E-194	IFITM2	2	GNLY_NK
198	5.28E-81	0.63583823	0.448	0.201	1.15E-76	S1PR5	2	GNLY_NK
199	8.69E-91	0.63411463	0.617	0.34	1.90E-86	PLEK	2	GNLY_NK
200	2.86E-121	0.63126182	0.833	0.566	6.24E-117	LITAF	2	GNLY_NK
201	2.32E-145	0.60592858	0.918	0.768	5.07E-141	CYBA	2	GNLY_NK
202	1.23E-89	0.60123452	0.286	0.078	2.68E-85	MTSS1	2	GNLY_NK
203	3.80E-95	0.59767785	0.801	0.527	8.28E-91	CMC1	2	GNLY_NK
204	2.57E-82	0.59586573	0.333	0.114	5.59E-78	LYN	2	GNLY_NK
205	1.67E-109	0.59280574	0.84	0.624	3.64E-105	ITGB2	2	GNLY_NK
206	2.54E-81	0.58498297	0.353	0.125	5.53E-77	FGR	2	GNLY_NK
207	1.95E-76	0.57422672	0.431	0.193	4.24E-72	EFHD2	2	GNLY_NK
208	9.96E-67	0.56893438	0.415	0.192	2.17E-62	GNPTAB	2	GNLY_NK

209	1.66E-66	0.55450055	0.539	0.31	3.62E-62	PLAC8	2	GNLY_NK
210	1.08E-70	0.54898403	0.583	0.348	2.35E-66	CD63	2	GNLY_NK
211	4.65E-63	0.54746814	0.48	0.263	1.01E-58	DSTN	2	GNLY_NK
212	1.29E-72	0.53666148	0.221	0.056	2.82E-68	AKR1C3	2	GNLY_NK
213	2.37E-56	0.53439059	0.526	0.321	5.17E-52	FLNA	2	GNLY_NK
214	1.05E-83	0.53369561	0.743	0.489	2.29E-79	GSTP1	2	GNLY_NK
215	3.27E-55	0.53192582	0.306	0.128	7.14E-51	TGFBR3	2	GNLY_NK
216	1.80E-59	0.52623172	0.508	0.282	3.93E-55	KLF2	2	GNLY_NK
217	8.65E-84	0.52211566	0.197	0.038	1.89E-79	LILRB1	2	GNLY_NK
218	2.18E-78	0.51970052	0.786	0.559	4.76E-74	EMP3	2	GNLY_NK
219	4.55E-56	0.51945405	0.352	0.16	9.93E-52	TBX21	2	GNLY_NK
220	1.08E-61	0.51295035	0.346	0.14	2.36E-57	TIGIT	2	GNLY_NK
221	1.94E-61	0.51046229	0.568	0.339	4.22E-57	DOK2	2	GNLY_NK
222	3.34E-133	0.50673675	0.969	0.893	7.29E-129	SH3BGRL3	2	GNLY_NK
223	5.76E-58	0.50486047	0.299	0.118	1.26E-53	CD300A	2	GNLY_NK
224	7.68E-59	0.50098665	0.22	0.066	1.67E-54	ENC1	2	GNLY_NK
225	4.33E-61	0.50068466	0.28	0.1	9.44E-57	CEP78	2	GNLY_NK
226	5.15E-49	0.49679244	0.536	0.351	1.12E-44	SERPINB1	2	GNLY_NK
227	7.85E-39	0.49371154	0.383	0.222	1.71E-34	STARD3NL	2	GNLY_NK
228	1.33E-42	0.49147252	0.31	0.149	2.91E-38	TRG-AS1	2	GNLY_NK
229	4.72E-59	0.4900423	0.298	0.114	1.03E-54	C1orf21	2	GNLY_NK
230	2.58E-49	0.48641526	0.301	0.133	5.62E-45	HSH2D	2	GNLY_NK
231	1.63E-74	0.47856338	0.178	0.035	3.54E-70	ASCL2	2	GNLY_NK
232	1.87E-48	0.47826045	0.539	0.355	4.08E-44	MBP	2	GNLY_NK
233	9.53E-60	0.47169912	0.259	0.089	2.08E-55	LAT2	2	GNLY_NK
234	2.82E-41	0.46579833	0.655	0.52	6.15E-37	CALR	2	GNLY_NK
235	6.79E-65	0.46544791	0.152	0.028	1.48E-60	FGL2	2	GNLY_NK
236	4.71E-73	0.46273877	0.183	0.038	1.03E-68	PLOD1	2	GNLY_NK
237	1.66E-38	0.45884197	0.365	0.206	3.61E-34	ABI3	2	GNLY_NK
238	4.16E-48	0.45843224	0.396	0.208	9.07E-44	PXN	2	GNLY_NK
239	1.64E-55	0.45743432	0.627	0.423	3.57E-51	CTSD	2	GNLY_NK
240	2.67E-73	0.45701684	0.811	0.628	5.83E-69	RAP1B	2	GNLY_NK
241	4.33E-43	0.45679535	0.468	0.289	9.44E-39	SELPLG	2	GNLY_NK
242	1.49E-44	0.45494463	0.294	0.133	3.26E-40	HDDC2	2	GNLY_NK
243	1.06E-42	0.45090704	0.435	0.257	2.32E-38	TPST2	2	GNLY_NK
244	3.17E-26	0.44439885	0.361	0.228	6.91E-22	RHOB	2	GNLY_NK
245	1.14E-44	0.43932165	0.221	0.082	2.49E-40	LGALS3	2	GNLY_NK
246	2.01E-50	0.43857018	0.195	0.059	4.38E-46	ITGAM	2	GNLY_NK
247	3.02E-58	0.43778408	0.184	0.047	6.59E-54	RP11-81H14.2	2	GNLY_NK
248	8.66E-52	0.43574331	0.21	0.065	1.89E-47	KIR2DL3	2	GNLY_NK
249	1.24E-123	0.4355659	0.992	0.885	2.70E-119	CCL5	2	GNLY_NK
250	1.43E-40	0.43441435	0.518	0.344	3.12E-36	UCP2	2	GNLY_NK
251	2.02E-31	0.43339739	0.413	0.268	4.40E-27	SPN	2	GNLY_NK
252	3.19E-41	0.4250629	0.205	0.075	6.95E-37	HAVCR2	2	GNLY_NK
253	1.24E-127	0.42458566	0.997	0.975	2.71E-123	PFN1	2	GNLY_NK
254	4.09E-37	0.42181616	0.335	0.18	8.93E-33	ADAM8	2	GNLY_NK
255	7.52E-71	0.41880219	0.162	0.029	1.64E-66	GPR141	2	GNLY_NK
256	1.95E-38	0.41865173	0.56	0.406	4.25E-34	ZAP70	2	GNLY_NK
257	3.47E-45	0.41775742	0.244	0.097	7.56E-41	ANXA4	2	GNLY_NK
258	4.89E-78	0.41406476	0.909	0.788	1.07E-73	ARPC2	2	GNLY_NK
259	4.06E-40	0.41260194	0.2	0.074	8.86E-36	SYNGR1	2	GNLY_NK
260	1.43E-34	0.40827157	0.473	0.32	3.13E-30	PRELID1	2	GNLY_NK
261	5.09E-30	0.40575372	0.451	0.306	1.11E-25	RHOC	2	GNLY_NK
262	5.34E-53	0.40412553	0.184	0.051	1.16E-48	KLRC2	2	GNLY_NK
263	4.17E-56	0.40402919	0.77	0.614	9.09E-52	LSP1	2	GNLY_NK
264	5.16E-38	0.39924041	0.456	0.281	1.13E-33	PPP3CC	2	GNLY_NK
265	1.13E-35	0.39772775	0.238	0.106	2.46E-31	GSAP	2	GNLY_NK
266	2.06E-31	0.39364922	0.361	0.211	4.50E-27	TXK	2	GNLY_NK
267	5.22E-38	0.39169811	0.637	0.486	1.14E-33	SLC9A3R1	2	GNLY_NK
268	5.77E-29	0.38967768	0.443	0.306	1.26E-24	ADD3	2	GNLY_NK
269	1.96E-26	0.38891371	0.311	0.183	4.28E-22	RASGRP2	2	GNLY_NK
270	2.59E-30	0.38422691	0.318	0.177	5.66E-26	RASA3	2	GNLY_NK
271	1.73E-38	0.38384405	0.187	0.068	3.78E-34	SLC15A4	2	GNLY_NK
272	1.53E-35	0.38149377	0.188	0.072	3.34E-31	GK5	2	GNLY_NK
273	4.94E-32	0.38085721	0.59	0.441	1.08E-27	LIMD2	2	GNLY_NK
274	3.68E-31	0.37756917	0.599	0.474	8.02E-27	FAM49B	2	GNLY_NK
275	6.45E-24	0.37680317	0.294	0.178	1.41E-19	PRKCB	2	GNLY_NK
276	2.38E-33	0.37474893	0.216	0.093	5.19E-29	UBE2F	2	GNLY_NK
277	1.03E-30	0.37262879	0.214	0.095	2.24E-26	LINC00944	2	GNLY_NK
278	8.77E-27	0.37205492	0.373	0.237	1.91E-22	UPP1	2	GNLY_NK

279	2.06E-36	0.37120007	0.578	0.416	4.50E-32	APMAP	2	GNLY_NK
280	6.64E-24	0.37019248	0.353	0.227	1.45E-19	SYNE1	2	GNLY_NK
281	1.71E-43	0.36813986	0.148	0.04	3.72E-39	CTBP2	2	GNLY_NK
282	2.53E-50	0.36578385	0.145	0.034	5.51E-46	RP11-277P12.6	2	GNLY_NK
283	1.49E-31	0.36465168	0.196	0.082	3.25E-27	RCBTB2	2	GNLY_NK
284	9.86E-43	0.36416571	0.166	0.051	2.15E-38	PLEKHG3	2	GNLY_NK
285	3.67E-26	0.36313594	0.445	0.31	8.01E-22	BHLHE40	2	GNLY_NK
286	2.04E-36	0.36272467	0.167	0.058	4.44E-32	VCL	2	GNLY_NK
287	2.52E-56	0.36162337	0.127	0.022	5.50E-52	PALLD	2	GNLY_NK
288	4.53E-25	0.3611046	0.466	0.348	9.88E-21	SCP2	2	GNLY_NK
289	5.78E-53	0.36028413	0.118	0.021	1.26E-48	ITGAX	2	GNLY_NK
290	3.66E-24	0.36005063	0.291	0.172	7.98E-20	ITGB7	2	GNLY_NK
291	6.32E-26	0.35686874	0.194	0.09	1.38E-21	PTPN12	2	GNLY_NK
292	3.48E-23	0.35478661	0.384	0.267	7.58E-19	NDUFB7	2	GNLY_NK
293	5.70E-30	0.35471175	0.514	0.373	1.24E-25	CAST	2	GNLY_NK
294	1.48E-27	0.35412676	0.523	0.397	3.23E-23	VAMP8	2	GNLY_NK
295	1.60E-44	0.3534247	0.152	0.041	3.50E-40	RASSF4	2	GNLY_NK
296	6.49E-26	0.35132466	0.293	0.165	1.42E-21	PTMS	2	GNLY_NK
297	1.47E-59	0.3505286	0.964	0.843	3.20E-55	S100A4	2	GNLY_NK
298	3.43E-46	0.35014963	0.15	0.039	7.49E-42	RAP1GAP2	2	GNLY_NK
299	1.64E-22	0.34973541	0.214	0.113	3.58E-18	TFDP2	2	GNLY_NK
300	2.20E-20	0.34672072	0.469	0.358	4.79E-16	TCF25	2	GNLY_NK
301	3.64E-25	0.34667125	0.388	0.25	7.93E-21	ITGB1	2	GNLY_NK
302	1.00E-24	0.3461243	0.552	0.432	2.18E-20	ARL4C	2	GNLY_NK
303	3.90E-50	0.34423585	0.12	0.023	8.51E-46	CXCR2	2	GNLY_NK
304	4.15E-27	0.34406785	0.166	0.067	9.04E-23	LAIR2	2	GNLY_NK
305	5.71E-28	0.34396529	0.336	0.193	1.25E-23	AOAH	2	GNLY_NK
306	4.44E-69	0.34282791	0.977	0.928	9.69E-65	CFL1	2	GNLY_NK
307	5.86E-23	0.34121729	0.271	0.158	1.28E-18	TMBIM1	2	GNLY_NK
308	2.00E-20	0.34016171	0.302	0.191	4.36E-16	PTGDR	2	GNLY_NK
309	3.13E-27	0.33969088	0.57	0.442	6.82E-23	BIN2	2	GNLY_NK
310	1.63E-29	0.338623	0.225	0.102	3.56E-25	FCRL3	2	GNLY_NK
311	2.27E-20	0.33755806	0.352	0.239	4.95E-16	TES	2	GNLY_NK
312	1.57E-27	0.33702255	0.54	0.414	3.43E-23	DHRS7	2	GNLY_NK
313	7.55E-25	0.33259564	0.567	0.457	1.65E-20	NDUFB2	2	GNLY_NK
314	5.87E-18	0.33188916	0.262	0.165	1.28E-13	G6PD	2	GNLY_NK
315	1.01E-24	0.33145242	0.526	0.404	2.21E-20	WDR1	2	GNLY_NK
316	1.22E-77	0.33046546	0.989	0.937	2.66E-73	IFITM1	2	GNLY_NK
317	3.30E-23	0.33034885	0.368	0.24	7.20E-19	MAPK1	2	GNLY_NK
318	6.71E-42	0.32855492	0.793	0.629	1.46E-37	CRIP1	2	GNLY_NK
319	1.47E-43	0.32758146	0.123	0.028	3.21E-39	TSPAN2	2	GNLY_NK
320	2.71E-31	0.32748501	0.15	0.054	5.91E-27	RGS9	2	GNLY_NK
321	4.27E-25	0.32704849	0.184	0.084	9.32E-21	S1PR1	2	GNLY_NK
322	1.58E-22	0.32635315	0.432	0.308	3.44E-18	TECR	2	GNLY_NK
323	6.92E-39	0.32160427	0.118	0.029	1.51E-34	MIR181A2HG	2	GNLY_NK
324	6.42E-28	0.32103822	0.166	0.066	1.40E-23	KIR3DL2	2	GNLY_NK
325	1.10E-18	0.31956462	0.28	0.177	2.39E-14	MED15	2	GNLY_NK
326	9.06E-23	0.31868582	0.523	0.395	1.98E-18	PYHIN1	2	GNLY_NK
327	4.71E-23	0.31844962	0.514	0.385	1.03E-18	PTPN6	2	GNLY_NK
328	1.33E-25	0.31822872	0.638	0.52	2.90E-21	PDIA3	2	GNLY_NK
329	2.98E-37	0.31739964	0.13	0.036	6.49E-33	KLRC4	2	GNLY_NK
330	4.32E-16	0.31724033	0.392	0.296	9.41E-12	RASSF1	2	GNLY_NK
331	1.50E-24	0.317053	0.172	0.077	3.27E-20	JAZF1	2	GNLY_NK
332	1.49E-18	0.3151368	0.311	0.204	3.26E-14	IFITM3	2	GNLY_NK
333	1.16E-18	0.31370289	0.419	0.316	2.54E-14	PRDX5	2	GNLY_NK
334	5.60E-31	0.31243844	0.587	0.412	1.22E-26	HLA-DPB1	2	GNLY_NK
335	3.20E-44	0.3121872	0.895	0.768	6.98E-40	S100A6	2	GNLY_NK
336	8.55E-16	0.3121432	0.298	0.205	1.86E-11	ADRB2	2	GNLY_NK
337	1.04E-16	0.31148912	0.376	0.278	2.26E-12	APOBEC3G	2	GNLY_NK
338	5.44E-16	0.30973402	0.34	0.245	1.19E-11	CAPN2	2	GNLY_NK
339	1.91E-26	0.30848479	0.599	0.467	4.16E-22	PPP1R18	2	GNLY_NK
340	5.21E-19	0.30766829	0.401	0.291	1.14E-14	MYO1G	2	GNLY_NK
341	1.37E-20	0.30715923	0.257	0.147	2.98E-16	DTHD1	2	GNLY_NK
342	4.17E-44	0.30636133	0.104	0.019	9.09E-40	AK5	2	GNLY_NK
343	1.76E-40	0.30519352	0.925	0.803	3.83E-36	GZMA	2	GNLY_NK
344	1.25E-18	0.3025158	0.513	0.412	2.73E-14	PRMT2	2	GNLY_NK
345	1.29E-26	0.30197582	0.167	0.07	2.80E-22	OSBPL5	2	GNLY_NK
346	3.53E-93	0.29746416	0.997	0.988	7.69E-89	HLA-C	2	GNLY_NK
347	5.05E-17	0.29728875	0.269	0.171	1.10E-12	HRASLS2	2	GNLY_NK
348	1.41E-68	0.29726444	0.999	0.997	3.07E-64	ACTB	2	GNLY_NK

349	4.08E-20	0.29700625	0.53	0.42	8.90E-16	ANXA6	2	GNLY_NK
350	2.60E-24	0.29679909	0.163	0.07	5.67E-20	NCR1	2	GNLY_NK
351	1.22E-19	0.29676683	0.172	0.086	2.66E-15	HIPK2	2	GNLY_NK
352	1.93E-24	0.29557209	0.638	0.509	4.20E-20	GZMM	2	GNLY_NK
353	2.34E-14	0.29452586	0.288	0.2	5.10E-10	MIEN1	2	GNLY_NK
354	4.05E-20	0.29128706	0.482	0.365	8.83E-16	HMGN3	2	GNLY_NK
355	6.47E-23	0.29061283	0.663	0.558	1.41E-18	CAP1	2	GNLY_NK
356	1.17E-19	0.29042225	0.59	0.479	2.55E-15	S100A11	2	GNLY_NK
357	6.29E-15	0.28994359	0.497	0.407	1.37E-10	MAPRE2	2	GNLY_NK
358	9.63E-18	0.28938419	0.264	0.161	2.10E-13	ARID5B	2	GNLY_NK
359	2.50E-25	0.28896764	0.717	0.599	5.46E-21	GNG2	2	GNLY_NK
360	5.30E-17	0.28877901	0.338	0.235	1.16E-12	FAM65B	2	GNLY_NK
361	5.41E-35	0.28858107	0.681	0.496	1.18E-30	HLA-DRB1	2	GNLY_NK
362	1.38E-43	0.2882041	0.105	0.02	3.01E-39	SLCO4C1	2	GNLY_NK
363	1.01E-18	0.28781569	0.154	0.075	2.20E-14	RRBP1	2	GNLY_NK
364	5.46E-20	0.28670659	0.591	0.486	1.19E-15	PPP1CA	2	GNLY_NK
365	1.88E-36	0.2864126	0.105	0.024	4.11E-32	PPM1L	2	GNLY_NK
366	8.21E-16	0.28639581	0.268	0.174	1.79E-11	STK38	2	GNLY_NK
367	2.23E-15	0.28605463	0.263	0.17	4.87E-11	F2R	2	GNLY_NK
368	2.44E-15	0.28534896	0.407	0.309	5.32E-11	CARD16	2	GNLY_NK
369	1.83E-18	0.2851645	0.42	0.302	4.00E-14	NCR3	2	GNLY_NK
370	2.85E-30	0.28446054	0.774	0.622	6.21E-26	ANXA1	2	GNLY_NK
371	4.50E-14	0.28420382	0.286	0.198	9.82E-10	MRPS6	2	GNLY_NK
372	1.18E-16	0.28332311	0.362	0.26	2.58E-12	CHST12	2	GNLY_NK
373	3.50E-16	0.2833097	0.372	0.272	7.64E-12	VASP	2	GNLY_NK
374	1.36E-21	0.28291432	0.578	0.465	2.96E-17	DBI	2	GNLY_NK
375	1.78E-32	0.28287885	0.115	0.032	3.89E-28	MLC1	2	GNLY_NK
376	1.44E-18	0.28115082	0.184	0.096	3.15E-14	NPRL2	2	GNLY_NK
377	2.71E-16	0.28101735	0.407	0.308	5.91E-12	SUN2	2	GNLY_NK
378	2.67E-14	0.28044886	0.343	0.252	5.82E-10	C5orf56	2	GNLY_NK
379	7.74E-43	0.28034106	0.92	0.843	1.69E-38	CD99	2	GNLY_NK
380	1.82E-18	0.27897788	0.239	0.138	3.96E-14	MCTP2	2	GNLY_NK
381	1.69E-13	0.27752247	0.262	0.178	3.68E-09	LPCAT1	2	GNLY_NK
382	2.94E-26	0.27673017	0.123	0.043	6.40E-22	NMUR1	2	GNLY_NK
383	1.08E-15	0.27614039	0.421	0.323	2.36E-11	PSMB10	2	GNLY_NK
384	5.27E-18	0.27581571	0.661	0.569	1.15E-13	CTSC	2	GNLY_NK
385	4.82E-93	0.274709	1	0.991	1.05E-88	HLA-A	2	GNLY_NK
386	5.48E-16	0.27270118	0.395	0.298	1.19E-11	NDUFA12	2	GNLY_NK
387	1.10E-42	0.27156381	0.954	0.912	2.41E-38	SERF2	2	GNLY_NK
388	3.51E-16	0.2713596	0.271	0.176	7.65E-12	PTPRE	2	GNLY_NK
389	4.35E-11	0.26983048	0.225	0.153	9.49E-07	GLIPR2	2	GNLY_NK
390	1.26E-16	0.26915494	0.507	0.413	2.76E-12	RAC1	2	GNLY_NK
391	2.36E-20	0.26911982	0.196	0.099	5.15E-16	APBA2	2	GNLY_NK
392	1.93E-14	0.26895537	0.32	0.226	4.21E-10	MYO1F	2	GNLY_NK
393	5.17E-23	0.26804093	0.299	0.168	1.13E-18	HLA-DQA1	2	GNLY_NK
394	1.16E-18	0.26638704	0.137	0.062	2.53E-14	FUT11	2	GNLY_NK
395	5.64E-79	0.2646572	1	0.991	1.23E-74	TMSB10	2	GNLY_NK
396	7.61E-31	0.26430668	0.865	0.785	1.66E-26	CLIC1	2	GNLY_NK
397	2.03E-24	0.26422656	0.141	0.055	4.42E-20	BPGM	2	GNLY_NK
398	3.06E-17	0.26187342	0.157	0.078	6.68E-13	FNDC3B	2	GNLY_NK
399	7.76E-12	0.25963724	0.253	0.177	1.69E-07	SRPK2	2	GNLY_NK
400	4.39E-15	0.25826317	0.266	0.174	9.57E-11	SH3BP5	2	GNLY_NK
401	6.57E-15	0.2575389	0.443	0.35	1.43E-10	BSG	2	GNLY_NK
402	3.38E-12	0.25749211	0.281	0.2	7.37E-08	LMO4	2	GNLY_NK
403	2.48E-17	0.25686349	0.62	0.535	5.40E-13	ARPC1B	2	GNLY_NK
404	1.17E-36	0.25679236	0.947	0.908	2.55E-32	CALM1	2	GNLY_NK
405	1.19E-24	0.25614935	0.797	0.715	2.59E-20	IL2RG	2	GNLY_NK
406	4.51E-21	0.25597106	0.727	0.656	9.82E-17	CCND3	2	GNLY_NK
407	2.05E-19	0.25563823	0.69	0.611	4.47E-15	ARL6IP5	2	GNLY_NK
408	3.05E-144	0.25537475	1	1	6.66E-140	B2M	2	GNLY_NK
409	1.21E-14	0.25460754	0.525	0.448	2.65E-10	CD47	2	GNLY_NK
410	2.45E-17	0.25410417	0.181	0.096	5.34E-13	PLCG2	2	GNLY_NK
411	5.28E-15	0.25402816	0.357	0.246	1.15E-10	HLA-DRB5	2	GNLY_NK
412	1.69E-18	0.25360373	0.148	0.069	3.68E-14	SYT11	2	GNLY_NK
413	3.41E-19	0.25355639	0.654	0.568	7.44E-15	RNASEK	2	GNLY_NK
414	5.05E-12	0.25334094	0.407	0.326	1.10E-07	PRKACB	2	GNLY_NK
415	8.92E-18	0.2532222	0.647	0.561	1.94E-13	TPI1	2	GNLY_NK
416	1.31E-16	0.25205012	0.632	0.552	2.86E-12	ACTR3	2	GNLY_NK
417	1.75E-15	0.25160837	0.533	0.448	3.81E-11	CD164	2	GNLY_NK
418	5.30E-14	0.25150794	0.35	0.257	1.16E-09	SYTL1	2	GNLY_NK

419	2.85E-13	0.25138626	0.223	0.143	6.23E-09	UCHL5	2	GNLY_NK
420	1.47E-39	-0.25255869	0.939	0.963	3.20E-35	RPL10A	2	GNLY_NK
421	5.08E-14	-0.25258616	0.225	0.347	1.11E-09	MYADM	2	GNLY_NK
422	2.34E-29	-0.25316734	0.914	0.938	5.10E-25	RPL9	2	GNLY_NK
423	3.75E-46	-0.25685733	0.978	0.988	8.17E-42	RPS25	2	GNLY_NK
424	1.06E-11	-0.25977143	0.205	0.308	2.31E-07	FAM46C	2	GNLY_NK
425	6.50E-96	-0.26026123	1	1	1.42E-91	EEF1A1	2	GNLY_NK
426	4.42E-54	-0.26124992	0.986	0.987	9.63E-50	RPL12	2	GNLY_NK
427	7.25E-23	-0.26209883	0.946	0.967	1.58E-18	JUNB	2	GNLY_NK
428	6.64E-43	-0.26248148	0.945	0.968	1.45E-38	RPSA	2	GNLY_NK
429	5.53E-17	-0.26477853	0.205	0.342	1.21E-12	PEBP1	2	GNLY_NK
430	8.65E-86	-0.26493618	0.999	0.998	1.89E-81	RPS15A	2	GNLY_NK
431	4.49E-71	-0.26609166	0.995	0.991	9.79E-67	RPL37	2	GNLY_NK
432	2.32E-15	-0.26624769	0.262	0.395	5.06E-11	NAP1L1	2	GNLY_NK
433	4.91E-17	-0.26627464	0.1	0.21	1.07E-12	ODF2L	2	GNLY_NK
434	5.87E-09	-0.26694675	0.262	0.353	0.000127974	NR4A1	2	GNLY_NK
435	1.09E-14	-0.27067542	0.41	0.53	2.37E-10	LDHB	2	GNLY_NK
436	1.64E-15	-0.27248186	0.128	0.238	3.59E-11	ERN1	2	GNLY_NK
437	5.32E-64	-0.27278379	0.987	0.993	1.16E-59	RPLP2	2	GNLY_NK
438	1.83E-32	-0.27497985	0.945	0.966	3.98E-28	RPS29	2	GNLY_NK
439	8.65E-17	-0.27522459	0.31	0.451	1.89E-12	TC2N	2	GNLY_NK
440	5.74E-12	-0.27882901	0.322	0.436	1.25E-07	EZR	2	GNLY_NK
441	6.66E-75	-0.27896252	0.997	0.995	1.45E-70	RPS28	2	GNLY_NK
442	4.26E-11	-0.27926659	0.535	0.627	9.29E-07	CITED2	2	GNLY_NK
443	1.87E-49	-0.27984647	0.968	0.979	4.07E-45	RPL36	2	GNLY_NK
444	3.53E-11	-0.28040203	0.159	0.25	7.69E-07	TUBA1C	2	GNLY_NK
445	1.32E-19	-0.28188348	0.36	0.519	2.87E-15	HMGN1	2	GNLY_NK
446	1.49E-18	-0.28742458	0.107	0.227	3.26E-14	BCL11B	2	GNLY_NK
447	4.78E-60	-0.28947686	0.985	0.987	1.04E-55	RPS21	2	GNLY_NK
448	1.40E-17	-0.29163122	0.105	0.218	3.06E-13	PBXIP1	2	GNLY_NK
449	1.22E-76	-0.29236774	0.998	0.997	2.65E-72	RPL34	2	GNLY_NK
450	1.71E-18	-0.29329074	0.298	0.45	3.73E-14	RHOH	2	GNLY_NK
451	2.75E-44	-0.29351376	0.919	0.965	5.99E-40	RPS2	2	GNLY_NK
452	1.18E-28	-0.29356551	0.65	0.772	2.58E-24	EEF1G	2	GNLY_NK
453	4.95E-14	-0.29530123	0.289	0.415	1.08E-09	KIAA1551	2	GNLY_NK
454	5.22E-20	-0.29846665	0.323	0.477	1.14E-15	CNN2	2	GNLY_NK
455	1.37E-107	-0.30133937	0.997	0.999	2.98E-103	RPL28	2	GNLY_NK
456	6.03E-27	-0.30225505	0.648	0.758	1.32E-22	RPS20	2	GNLY_NK
457	9.07E-17	-0.30238096	0.286	0.42	1.98E-12	RORA	2	GNLY_NK
458	2.41E-81	-0.30335402	0.991	0.991	5.27E-77	RPL18A	2	GNLY_NK
459	6.93E-22	-0.30413246	0.364	0.53	1.51E-17	GYPC	2	GNLY_NK
460	2.65E-70	-0.30489087	0.984	0.991	5.78E-66	RPS13	2	GNLY_NK
461	2.53E-14	-0.30561087	0.321	0.442	5.52E-10	ANP32B	2	GNLY_NK
462	1.09E-20	-0.30731593	0.199	0.349	2.38E-16	ELOVL5	2	GNLY_NK
463	3.24E-36	-0.30732305	0.804	0.883	7.05E-32	EEF1B2	2	GNLY_NK
464	3.67E-89	-0.3082126	1	0.999	8.01E-85	RPLP1	2	GNLY_NK
465	1.00E-09	-0.30931357	0.22	0.311	2.19E-05	FAM177A1	2	GNLY_NK
466	4.87E-34	-0.30952523	0.657	0.788	1.06E-29	HINT1	2	GNLY_NK
467	7.93E-91	-0.3100262	0.997	0.994	1.73E-86	RPL32	2	GNLY_NK
468	3.70E-19	-0.31090497	0.269	0.42	8.06E-15	SH2D1A	2	GNLY_NK
469	1.77E-35	-0.31310807	0.811	0.873	3.85E-31	PABPC1	2	GNLY_NK
470	4.67E-22	-0.31590742	0.19	0.346	1.02E-17	TNFAIP8	2	GNLY_NK
471	1.70E-77	-0.31840833	0.988	0.994	3.70E-73	TPT1	2	GNLY_NK
472	5.43E-19	-0.31843305	0.183	0.317	1.18E-14	PIK3IP1	2	GNLY_NK
473	1.10E-11	-0.32263486	0.547	0.605	2.40E-07	CD52	2	GNLY_NK
474	2.77E-68	-0.32285065	0.951	0.977	6.03E-64	RPL8	2	GNLY_NK
475	2.94E-57	-0.32301368	0.927	0.956	6.41E-53	RPS5	2	GNLY_NK
476	1.15E-19	-0.32641605	0.296	0.444	2.51E-15	SNHG8	2	GNLY_NK
477	1.07E-08	-0.3275887	0.819	0.824	0.00023441	VIM	2	GNLY_NK
478	3.61E-20	-0.33111136	0.541	0.67	7.87E-16	PPP1R15A	2	GNLY_NK
479	5.14E-15	-0.33180934	0.181	0.299	1.12E-10	TSPYL2	2	GNLY_NK
480	2.42E-93	-0.33311073	0.998	0.997	5.28E-89	RPS12	2	GNLY_NK
481	3.72E-23	-0.33757677	0.146	0.292	8.11E-19	CD6	2	GNLY_NK
482	6.56E-26	-0.33774554	0.13	0.286	1.43E-21	TMEM123	2	GNLY_NK
483	1.16E-22	-0.34368741	0.564	0.702	2.52E-18	DNAJB1	2	GNLY_NK
484	1.20E-106	-0.34664332	0.997	0.995	2.62E-102	RPS8	2	GNLY_NK
485	1.46E-110	-0.34773955	0.997	0.997	3.17E-106	RPL13	2	GNLY_NK
486	5.46E-115	-0.34883816	0.997	0.997	1.19E-110	RPL39	2	GNLY_NK
487	8.64E-27	-0.34992287	0.569	0.716	1.88E-22	SRSF7	2	GNLY_NK
488	9.53E-85	-0.35024275	0.985	0.99	2.08E-80	RPS18	2	GNLY_NK

489	4.10E-21	-0.35027758	0.742	0.825	8.94E-17	KLF6	2	GNLY_NK
490	1.75E-18	-0.35116641	0.166	0.296	3.82E-14	AIM1	2	GNLY_NK
491	1.27E-06	-0.35820546	0.494	0.565	0.027589555	HMGN2	2	GNLY_NK
492	1.48E-22	-0.36608477	0.397	0.547	3.22E-18	PARP8	2	GNLY_NK
493	1.97E-25	-0.36907308	0.31	0.485	4.29E-21	CD96	2	GNLY_NK
494	4.93E-18	-0.37507194	0.368	0.504	1.07E-13	BTG2	2	GNLY_NK
495	1.01E-20	-0.38077717	0.234	0.384	2.20E-16	CLDND1	2	GNLY_NK
496	1.67E-26	-0.38264635	0.323	0.5	3.65E-22	CCNH	2	GNLY_NK
497	6.08E-29	-0.38459678	0.316	0.504	1.33E-24	LEPROTL1	2	GNLY_NK
498	4.51E-44	-0.38876404	0.82	0.907	9.83E-40	CXCR4	2	GNLY_NK
499	6.28E-29	-0.39018176	0.111	0.27	1.37E-24	PLP2	2	GNLY_NK
500	4.24E-49	-0.39559093	0.946	0.973	9.25E-45	ZFP36L2	2	GNLY_NK
501	6.66E-30	-0.41736744	0.273	0.459	1.45E-25	CD44	2	GNLY_NK
502	2.11E-22	-0.41846801	0.304	0.459	4.61E-18	NFKBIZ	2	GNLY_NK
503	4.41E-30	-0.43883298	0.114	0.277	9.62E-26	SATB1	2	GNLY_NK
504	2.74E-31	-0.43886304	0.658	0.803	5.98E-27	JUN	2	GNLY_NK
505	2.64E-19	-0.46530557	0.475	0.592	5.75E-15	TNFAIP3	2	GNLY_NK
506	6.37E-72	-0.46558435	0.75	0.903	1.39E-67	SARAF	2	GNLY_NK
507	3.40E-15	-0.4666535	0.207	0.321	7.41E-11	EGR1	2	GNLY_NK
508	1.07E-30	-0.47966421	0.711	0.804	2.33E-26	NFKBIA	2	GNLY_NK
509	5.48E-108	-0.48724565	0.939	0.97	1.20E-103	RPLP0	2	GNLY_NK
510	9.91E-33	-0.49947469	0.196	0.379	2.16E-28	ITM2A	2	GNLY_NK
511	1.66E-28	-0.5180027	0.126	0.287	3.62E-24	RGCC	2	GNLY_NK
512	6.21E-59	-0.52670824	0.807	0.907	1.35E-54	DUSP1	2	GNLY_NK
513	7.22E-14	-0.52986717	0.342	0.444	1.57E-09	TNF	2	GNLY_NK
514	1.30E-48	-0.53759552	0.245	0.481	2.83E-44	PDCD4	2	GNLY_NK
515	5.06E-37	-0.53948283	0.377	0.571	1.10E-32	FOSB	2	GNLY_NK
516	1.82E-47	-0.56084903	0.172	0.396	3.96E-43	EML4	2	GNLY_NK
517	1.63E-19	-0.57027809	0.266	0.4	3.55E-15	CCL3L3	2	GNLY_NK
518	3.83E-50	-0.58385799	0.135	0.364	8.35E-46	IFNGR1	2	GNLY_NK
519	1.94E-08	-0.60376026	0.309	0.391	0.000422268	HIST1H4C	2	GNLY_NK
520	1.13E-11	-0.63416691	0.268	0.359	2.47E-07	HMGB2	2	GNLY_NK
521	1.26E-69	-0.64792681	0.135	0.423	2.75E-65	SPOCK2	2	GNLY_NK
522	1.04E-45	-0.6566307	0.75	0.852	2.27E-41	FOS	2	GNLY_NK
523	1.05E-129	-0.83907109	0.78	0.931	2.29E-125	CD69	2	GNLY_NK
524	1.38E-33	-0.87507302	0.6	0.612	3.01E-29	KLRB1	2	GNLY_NK
525	5.21E-16	-0.9302289	0.236	0.351	1.14E-11	STMN1	2	GNLY_NK
526	1.41E-77	-1.11655427	0.135	0.417	3.07E-73	RGS1	2	GNLY_NK
527	5.07E-218	1.83168134	0.305	0.024	1.10E-213	CCL20	3	CCL20_CD8 MAIT
528	0	1.50005153	0.992	0.522	0	KLRB1	3	CCL20_CD8 MAIT
529	0	1.45740673	0.622	0.027	0	TRAV1-2	3	CCL20_CD8 MAIT
530	0	1.3246429	0.97	0.282	0	IL7R	3	CCL20_CD8 MAIT
531	0	1.26512982	0.566	0.029	0	SLC4A10	3	CCL20_CD8 MAIT
532	1.92E-266	1.208125	0.338	0.021	4.18E-262	TRBV6-1	3	CCL20_CD8 MAIT
533	2.77E-287	1.15307436	0.729	0.193	6.04E-283	LTB	3	CCL20_CD8 MAIT
534	0	1.13109694	0.784	0.223	0	KLRG1	3	CCL20_CD8 MAIT
535	2.47E-162	1.01970008	0.753	0.347	5.38E-158	TNF	3	CCL20_CD8 MAIT
536	3.88E-130	0.94437311	0.185	0.015	8.45E-126	TRAV13-2	3	CCL20_CD8 MAIT
537	2.48E-199	0.84159038	0.485	0.104	5.41E-195	AQP3	3	CCL20_CD8 MAIT
538	3.49E-157	0.82727006	0.947	0.749	7.62E-153	NFKBIA	3	CCL20_CD8 MAIT
539	1.52E-108	0.78388088	0.146	0.01	3.31E-104	TRBV6-4	3	CCL20_CD8 MAIT
540	7.97E-155	0.77502821	0.65	0.251	1.74E-150	NCR3	3	CCL20_CD8 MAIT
541	2.89E-133	0.76906133	0.731	0.358	6.29E-129	NFKBIZ	3	CCL20_CD8 MAIT
542	9.25E-95	0.74857744	0.361	0.109	2.02E-90	KLRC1	3	CCL20_CD8 MAIT
543	1.16E-206	0.74635434	0.35	0.042	2.54E-202	CCR6	3	CCL20_CD8 MAIT
544	1.47E-134	0.73175513	0.531	0.179	3.21E-130	SATB1	3	CCL20_CD8 MAIT
545	1.48E-67	0.72874491	0.596	0.322	3.23E-63	CCL3L3	3	CCL20_CD8 MAIT
546	4.12E-172	0.720089	0.385	0.069	8.99E-168	CD40LG	3	CCL20_CD8 MAIT
547	1.58E-155	0.71714267	0.989	0.881	3.45E-151	CD69	3	CCL20_CD8 MAIT
548	9.00E-44	0.71445463	0.454	0.263	1.96E-39	EGR1	3	CCL20_CD8 MAIT
549	2.34E-156	0.7133067	0.426	0.096	5.10E-152	CXCR6	3	CCL20_CD8 MAIT
550	1.15E-170	0.6978323	0.323	0.047	2.51E-166	COLQ	3	CCL20_CD8 MAIT
551	1.30E-176	0.69522544	0.32	0.043	2.83E-172	DPP4	3	CCL20_CD8 MAIT
552	3.04E-66	0.69228404	0.915	0.812	6.64E-62	FOS	3	CCL20_CD8 MAIT
553	4.50E-113	0.67758966	0.51	0.184	9.82E-109	AC092580.4	3	CCL20_CD8 MAIT
554	5.91E-120	0.67551728	0.951	0.873	1.29E-115	DUSP1	3	CCL20_CD8 MAIT
555	1.34E-122	0.66757091	0.671	0.295	2.92E-118	SPOCK2	3	CCL20_CD8 MAIT
556	6.48E-141	0.65810406	0.421	0.104	1.41E-136	TMIGD2	3	CCL20_CD8 MAIT
557	2.87E-109	0.65295724	0.6	0.254	6.26E-105	IFNGR1	3	CCL20_CD8 MAIT
558	2.37E-206	0.63749176	0.261	0.015	5.16E-202	RORC	3	CCL20_CD8 MAIT

559	1.18E-82	0.6328307	0.925	0.74	2.58E-78	JUN	3	CCL20_CD8 MAIT
560	5.03E-134	0.61574065	0.324	0.065	1.10E-129	RUNX2	3	CCL20_CD8 MAIT
561	3.57E-126	0.61445437	0.448	0.129	7.78E-122	CAMK4	3	CCL20_CD8 MAIT
562	6.42E-67	0.60983527	0.764	0.524	1.40E-62	TNFAIP3	3	CCL20_CD8 MAIT
563	1.35E-179	0.60922813	0.237	0.016	2.93E-175	IL23R	3	CCL20_CD8 MAIT
564	3.37E-71	0.60650271	0.697	0.427	7.35E-67	BTG2	3	CCL20_CD8 MAIT
565	1.95E-97	0.60576372	0.369	0.111	4.24E-93	CEBPD	3	CCL20_CD8 MAIT
566	8.33E-77	0.60560123	0.822	0.603	1.82E-72	PPP1R15A	3	CCL20_CD8 MAIT
567	1.08E-127	0.59727466	0.353	0.079	2.35E-123	JAML	3	CCL20_CD8 MAIT
568	8.83E-140	0.5887147	0.346	0.069	1.93E-135	SLAMF1	3	CCL20_CD8 MAIT
569	6.21E-111	0.56166956	0.31	0.07	1.35E-106	IKZF2	3	CCL20_CD8 MAIT
570	4.60E-122	0.55570439	0.242	0.036	1.00E-117	LST1	3	CCL20_CD8 MAIT
571	1.46E-68	0.55395528	0.612	0.343	3.19E-64	RORA	3	CCL20_CD8 MAIT
572	1.35E-97	0.53512203	0.346	0.096	2.95E-93	HPGD	3	CCL20_CD8 MAIT
573	1.88E-65	0.53325741	0.748	0.494	4.09E-61	GPR65	3	CCL20_CD8 MAIT
574	1.21E-75	0.51453097	0.381	0.137	2.63E-71	GPR171	3	CCL20_CD8 MAIT
575	6.90E-82	0.51296059	0.944	0.842	1.51E-77	DUSP2	3	CCL20_CD8 MAIT
576	8.01E-94	0.51086207	0.335	0.093	1.75E-89	ZBTB16	3	CCL20_CD8 MAIT
577	3.25E-63	0.50488226	0.2	0.05	7.10E-59	CH17-373J23.1	3	CCL20_CD8 MAIT
578	5.74E-53	0.50421427	0.252	0.086	1.25E-48	TRGV2	3	CCL20_CD8 MAIT
579	7.27E-143	0.49939094	0.881	0.367	1.59E-138	GZMK	3	CCL20_CD8 MAIT
580	5.18E-65	0.48297898	0.472	0.22	1.13E-60	GYG1	3	CCL20_CD8 MAIT
581	1.96E-65	0.48269612	0.735	0.467	4.27E-61	PARP8	3	CCL20_CD8 MAIT
582	5.41E-45	0.47857606	0.286	0.118	1.18E-40	CD83	3	CCL20_CD8 MAIT
583	9.46E-111	0.47751233	0.2	0.025	2.06E-106	PLEKHA5	3	CCL20_CD8 MAIT
584	3.03E-60	0.46959524	0.401	0.173	6.60E-56	ERN1	3	CCL20_CD8 MAIT
585	3.57E-35	0.46701154	0.494	0.298	7.79E-31	NR4A1	3	CCL20_CD8 MAIT
586	6.43E-83	0.46691526	0.388	0.131	1.40E-78	TRAT1	3	CCL20_CD8 MAIT
587	1.11E-63	0.46433137	0.336	0.124	2.43E-59	BCL2	3	CCL20_CD8 MAIT
588	3.01E-79	0.45718349	0.294	0.083	6.57E-75	SIRPG	3	CCL20_CD8 MAIT
589	8.41E-97	0.4566037	0.837	0.429	1.83E-92	CD3D	3	CCL20_CD8 MAIT
590	1.12E-29	0.45563199	0.186	0.075	2.43E-25	TRGV10	3	CCL20_CD8 MAIT
591	3.91E-28	0.44845489	0.397	0.234	8.54E-24	RGS2	3	CCL20_CD8 MAIT
592	8.11E-24	0.44763559	0.332	0.196	1.77E-19	BCL2A1	3	CCL20_CD8 MAIT
593	1.68E-60	0.44692938	0.658	0.383	3.66E-56	PDCD4	3	CCL20_CD8 MAIT
594	3.22E-50	0.44661344	0.317	0.13	7.01E-46	PBX4	3	CCL20_CD8 MAIT
595	7.65E-76	0.44172694	0.259	0.069	1.67E-71	CD28	3	CCL20_CD8 MAIT
596	5.61E-48	0.43860741	0.183	0.053	1.22E-43	MYC	3	CCL20_CD8 MAIT
597	1.22E-81	0.43570837	0.855	0.534	2.66E-77	CD52	3	CCL20_CD8 MAIT
598	1.42E-53	0.43534949	0.328	0.131	3.10E-49	SLC7A5	3	CCL20_CD8 MAIT
599	3.50E-39	0.43486976	0.479	0.271	7.64E-35	IER3	3	CCL20_CD8 MAIT
600	4.76E-55	0.43090369	0.668	0.4	1.04E-50	CD96	3	CCL20_CD8 MAIT
601	2.15E-61	0.43040331	0.349	0.135	4.69E-57	TTC39C	3	CCL20_CD8 MAIT
602	4.94E-88	0.4265246	0.734	0.352	1.08E-83	CD8A	3	CCL20_CD8 MAIT
603	6.71E-105	0.42022869	0.985	0.84	1.46E-100	S100A4	3	CCL20_CD8 MAIT
604	1.57E-34	0.41928063	0.701	0.493	3.42E-30	FOSB	3	CCL20_CD8 MAIT
605	8.90E-49	0.41742858	0.504	0.272	1.94E-44	TNFAIP8	3	CCL20_CD8 MAIT
606	1.43E-75	0.41464277	0.179	0.033	3.11E-71	CCR1	3	CCL20_CD8 MAIT
607	2.93E-67	0.41265506	0.259	0.075	6.40E-63	TLE1	3	CCL20_CD8 MAIT
608	5.94E-126	0.40357675	0.165	0.01	1.30E-121	ME1	3	CCL20_CD8 MAIT
609	1.28E-66	0.40178617	0.201	0.048	2.78E-62	NR1D1	3	CCL20_CD8 MAIT
610	8.11E-71	0.39934098	0.212	0.05	1.77E-66	TNFRSF25	3	CCL20_CD8 MAIT
611	5.05E-44	0.39759926	0.493	0.27	1.10E-39	G3BP2	3	CCL20_CD8 MAIT
612	9.04E-127	0.39663447	0.136	0.003	1.97E-122	P2RY14	3	CCL20_CD8 MAIT
613	4.02E-46	0.39633744	0.246	0.09	8.76E-42	PFKFB3	3	CCL20_CD8 MAIT
614	1.89E-48	0.39359317	0.307	0.126	4.11E-44	FLT3LG	3	CCL20_CD8 MAIT
615	2.22E-66	0.3907395	0.931	0.761	4.84E-62	S100A6	3	CCL20_CD8 MAIT
616	1.31E-45	0.38867524	0.403	0.195	2.86E-41	SYTL2	3	CCL20_CD8 MAIT
617	2.00E-54	0.38772306	0.179	0.045	4.35E-50	SPRY1	3	CCL20_CD8 MAIT
618	3.40E-52	0.38591033	0.326	0.13	7.42E-48	SIT1	3	CCL20_CD8 MAIT
619	3.64E-40	0.38367553	0.532	0.31	7.94E-36	EML4	3	CCL20_CD8 MAIT
620	2.39E-45	0.38342715	0.353	0.159	5.22E-41	PBXIP1	3	CCL20_CD8 MAIT
621	2.04E-45	0.38276663	0.342	0.153	4.44E-41	ODF2L	3	CCL20_CD8 MAIT
622	2.58E-36	0.3815316	0.359	0.185	5.62E-32	H1FX	3	CCL20_CD8 MAIT
623	3.68E-41	0.38096553	0.54	0.316	8.02E-37	GBP5	3	CCL20_CD8 MAIT
624	1.30E-54	0.38062437	0.289	0.104	2.83E-50	IL18RAP	3	CCL20_CD8 MAIT
625	1.89E-47	0.37915842	0.441	0.219	4.12E-43	CASP1	3	CCL20_CD8 MAIT
626	1.06E-37	0.37325947	0.24	0.098	2.31E-33	TRAF1	3	CCL20_CD8 MAIT
627	1.45E-29	0.3694492	0.246	0.117	3.15E-25	TMEM107	3	CCL20_CD8 MAIT
628	3.26E-22	0.36190578	0.495	0.339	7.11E-18	ZC3H12A	3	CCL20_CD8 MAIT

629	2.18E-34	0.36055883	0.635	0.425	4.76E-30	CCNH	3	CCL20_CD8 MAIT
630	4.66E-80	0.35809864	0.179	0.03	1.02E-75	CTSH	3	CCL20_CD8 MAIT
631	1.24E-40	0.3578639	0.315	0.14	2.71E-36	SESN1	3	CCL20_CD8 MAIT
632	1.51E-41	0.3578046	0.416	0.214	3.30E-37	RBMS1	3	CCL20_CD8 MAIT
633	2.13E-56	0.35742004	0.615	0.314	4.64E-52	CD3G	3	CCL20_CD8 MAIT
634	2.54E-39	0.35635696	0.521	0.306	5.53E-35	LPXN	3	CCL20_CD8 MAIT
635	2.07E-36	0.35498598	0.195	0.071	4.51E-32	FEZ1	3	CCL20_CD8 MAIT
636	1.17E-75	0.35467681	0.988	0.84	2.55E-71	IL32	3	CCL20_CD8 MAIT
637	5.52E-32	0.35209908	0.34	0.177	1.20E-27	PNP	3	CCL20_CD8 MAIT
638	8.12E-42	0.34991475	0.356	0.164	1.77E-37	CD160	3	CCL20_CD8 MAIT
639	2.01E-43	0.3482643	0.226	0.08	4.39E-39	IL18R1	3	CCL20_CD8 MAIT
640	3.36E-53	0.34452038	0.15	0.033	7.32E-49	CFH	3	CCL20_CD8 MAIT
641	5.06E-64	0.34328867	0.165	0.033	1.10E-59	DUSP16	3	CCL20_CD8 MAIT
642	8.02E-38	0.33746859	0.167	0.054	1.75E-33	SNX9	3	CCL20_CD8 MAIT
643	1.01E-37	0.3373719	0.157	0.048	2.21E-33	PTCH2	3	CCL20_CD8 MAIT
644	1.57E-93	0.33603203	0.108	0.004	3.43E-89	BLK	3	CCL20_CD8 MAIT
645	1.40E-73	0.33553375	0.114	0.011	3.06E-69	ADAM12	3	CCL20_CD8 MAIT
646	5.59E-38	0.33528265	0.613	0.379	1.22E-33	TC2N	3	CCL20_CD8 MAIT
647	9.09E-39	0.33191735	0.269	0.113	1.98E-34	PDE7A	3	CCL20_CD8 MAIT
648	1.84E-44	0.32976036	0.24	0.086	4.01E-40	TRGC2	3	CCL20_CD8 MAIT
649	7.38E-42	0.32795653	0.22	0.079	1.61E-37	ABCB1	3	CCL20_CD8 MAIT
650	4.93E-29	0.32748562	0.406	0.235	1.08E-24	GLRX	3	CCL20_CD8 MAIT
651	1.11E-43	0.32501955	0.178	0.054	2.41E-39	PHACTR2	3	CCL20_CD8 MAIT
652	7.47E-95	0.3238737	0.119	0.006	1.63E-90	LTK	3	CCL20_CD8 MAIT
653	1.95E-28	0.32311157	0.234	0.107	4.26E-24	RNU12	3	CCL20_CD8 MAIT
654	3.77E-32	0.3228003	0.51	0.312	8.22E-28	FKBP11	3	CCL20_CD8 MAIT
655	1.84E-30	0.31583004	0.663	0.46	4.01E-26	TERF2IP	3	CCL20_CD8 MAIT
656	1.74E-39	0.31355017	0.33	0.149	3.79E-35	THEMIS	3	CCL20_CD8 MAIT
657	2.20E-31	0.31332658	0.505	0.305	4.79E-27	CCDC107	3	CCL20_CD8 MAIT
658	1.18E-32	0.31262786	0.352	0.18	2.57E-28	PRNP	3	CCL20_CD8 MAIT
659	1.01E-32	0.31248942	0.369	0.192	2.20E-28	HELZ	3	CCL20_CD8 MAIT
660	1.60E-38	0.31225116	0.184	0.061	3.50E-34	RP11-18H21.1	3	CCL20_CD8 MAIT
661	1.41E-33	0.31070743	0.259	0.115	3.07E-29	OXNAD1	3	CCL20_CD8 MAIT
662	4.85E-37	0.30900595	0.239	0.096	1.06E-32	GALC	3	CCL20_CD8 MAIT
663	2.91E-18	0.30409111	0.191	0.099	6.35E-14	NFKB1	3	CCL20_CD8 MAIT
664	4.17E-37	0.30376489	0.953	0.875	9.10E-33	CXCR4	3	CCL20_CD8 MAIT
665	4.43E-33	0.30291706	0.217	0.089	9.65E-29	ATF7IP2	3	CCL20_CD8 MAIT
666	2.79E-36	0.3027738	0.284	0.124	6.09E-32	NCF1	3	CCL20_CD8 MAIT
667	3.09E-39	0.30183718	0.437	0.223	6.73E-35	CD6	3	CCL20_CD8 MAIT
668	1.99E-28	0.30150558	0.941	0.911	4.34E-24	IER2	3	CCL20_CD8 MAIT
669	6.52E-29	0.30015906	0.307	0.155	1.42E-24	MAF	3	CCL20_CD8 MAIT
670	3.27E-23	0.2995189	0.715	0.567	7.14E-19	MCL1	3	CCL20_CD8 MAIT
671	1.50E-39	0.29876284	0.279	0.116	3.28E-35	HNRNPLL	3	CCL20_CD8 MAIT
672	7.12E-39	0.29830909	0.199	0.069	1.55E-34	TNFSF14	3	CCL20_CD8 MAIT
673	2.91E-34	0.29701568	0.325	0.158	6.35E-30	PDE4B	3	CCL20_CD8 MAIT
674	2.17E-19	0.29509764	0.723	0.583	4.74E-15	CITED2	3	CCL20_CD8 MAIT
675	1.01E-81	0.29480622	0.118	0.009	2.19E-77	TSPAN15	3	CCL20_CD8 MAIT
676	7.73E-94	0.29004305	1	0.999	1.69E-89	MT-CO3	3	CCL20_CD8 MAIT
677	1.86E-31	0.28911058	0.301	0.145	4.06E-27	CDK6	3	CCL20_CD8 MAIT
678	2.51E-41	0.28823007	0.13	0.031	5.48E-37	PLXND1	3	CCL20_CD8 MAIT
679	5.93E-26	0.2874865	0.581	0.394	1.29E-21	CKLF	3	CCL20_CD8 MAIT
680	9.50E-29	0.28690712	0.255	0.12	2.07E-24	CTSA	3	CCL20_CD8 MAIT
681	4.63E-20	0.28655416	0.438	0.292	1.01E-15	ELOVL5	3	CCL20_CD8 MAIT
682	3.08E-24	0.28450605	0.483	0.307	6.71E-20	RBL2	3	CCL20_CD8 MAIT
683	8.86E-29	0.28431152	0.25	0.117	1.93E-24	TPD52	3	CCL20_CD8 MAIT
684	5.85E-68	0.28389698	0.106	0.01	1.28E-63	DKK3	3	CCL20_CD8 MAIT
685	3.33E-89	0.28237653	1	0.999	7.26E-85	MT-CO1	3	CCL20_CD8 MAIT
686	2.96E-60	0.2816251	0.995	0.979	6.46E-56	BTG1	3	CCL20_CD8 MAIT
687	1.79E-34	0.28138202	0.128	0.035	3.91E-30	TRGV4	3	CCL20_CD8 MAIT
688	2.14E-22	0.27968712	0.473	0.311	4.67E-18	MGAT4A	3	CCL20_CD8 MAIT
689	2.78E-30	0.27940817	0.205	0.085	6.06E-26	SUPT3H	3	CCL20_CD8 MAIT
690	1.10E-56	0.27717766	0.111	0.015	2.41E-52	CA2	3	CCL20_CD8 MAIT
691	1.32E-33	0.27453012	0.172	0.061	2.89E-29	CERK	3	CCL20_CD8 MAIT
692	2.96E-22	0.272075	0.506	0.337	6.46E-18	FNBP1	3	CCL20_CD8 MAIT
693	3.05E-17	0.27098301	0.27	0.157	6.66E-13	PHLDA1	3	CCL20_CD8 MAIT
694	1.64E-21	0.26922003	0.242	0.127	3.58E-17	OBFC1	3	CCL20_CD8 MAIT
695	2.75E-28	0.26907359	0.963	0.937	6.00E-24	ZFP36	3	CCL20_CD8 MAIT
696	6.65E-29	0.26818274	0.181	0.072	1.45E-24	PLCG1	3	CCL20_CD8 MAIT
697	2.07E-22	0.26569589	0.59	0.408	4.52E-18	ABRACL	3	CCL20_CD8 MAIT
698	2.71E-18	0.26500055	0.441	0.295	5.92E-14	MYADM	3	CCL20_CD8 MAIT

699	2.03E-34	0.26455831	0.941	0.842	4.42E-30	PABPC1	3	CCL20_CD8 MAIT
700	2.50E-25	0.26294286	0.404	0.236	5.45E-21	GPRIN3	3	CCL20_CD8 MAIT
701	2.13E-31	0.26111501	0.16	0.056	4.65E-27	TBC1D31	3	CCL20_CD8 MAIT
702	2.04E-20	0.2610811	0.272	0.151	4.45E-16	KDM6B	3	CCL20_CD8 MAIT
703	4.67E-41	0.2597288	0.11	0.023	1.02E-36	NRIP1	3	CCL20_CD8 MAIT
704	1.41E-23	0.2597025	0.272	0.143	3.06E-19	PAG1	3	CCL20_CD8 MAIT
705	5.52E-88	0.25909573	0.998	0.992	1.20E-83	TPT1	3	CCL20_CD8 MAIT
706	3.60E-33	0.25747692	0.169	0.059	7.84E-29	MPRIP	3	CCL20_CD8 MAIT
707	4.19E-18	0.25642209	0.386	0.244	9.14E-14	JUND	3	CCL20_CD8 MAIT
708	3.41E-36	0.25563986	0.164	0.053	7.44E-32	MKNK1	3	CCL20_CD8 MAIT
709	2.80E-32	0.25515433	0.159	0.055	6.11E-28	SPNS3	3	CCL20_CD8 MAIT
710	9.11E-54	0.25436806	0.992	0.958	1.99E-49	RPLP0	3	CCL20_CD8 MAIT
711	3.04E-57	0.25408081	0.101	0.012	6.62E-53	TMEM171	3	CCL20_CD8 MAIT
712	3.10E-27	0.25298363	0.324	0.167	6.76E-23	INPP4B	3	CCL20_CD8 MAIT
713	6.03E-20	0.25007381	0.185	0.089	1.31E-15	TIFA	3	CCL20_CD8 MAIT
714	3.84E-07	-0.25056403	0.153	0.208	0.008376662	MED15	3	CCL20_CD8 MAIT
715	8.24E-09	-0.25179487	0.139	0.205	0.00017979	STK38	3	CCL20_CD8 MAIT
716	6.43E-07	-0.25283785	0.858	0.853	0.014027521	HSP90AA1	3	CCL20_CD8 MAIT
717	1.34E-09	-0.25770125	0.259	0.331	2.92E-05	RTN4	3	CCL20_CD8 MAIT
718	2.99E-10	-0.25812078	0.11	0.183	6.51E-06	TSPAN14	3	CCL20_CD8 MAIT
719	8.39E-07	-0.25872765	0.252	0.302	0.018297236	TPST2	3	CCL20_CD8 MAIT
720	2.10E-08	-0.26012773	0.143	0.21	0.000456991	ATP1B3	3	CCL20_CD8 MAIT
721	1.21E-09	-0.26405534	0.245	0.32	2.65E-05	DSTN	3	CCL20_CD8 MAIT
722	1.01E-32	-0.26507154	0.989	0.977	2.19E-28	PFN1	3	CCL20_CD8 MAIT
723	1.11E-07	-0.26560379	0.334	0.376	0.002415393	RAD21	3	CCL20_CD8 MAIT
724	6.86E-12	-0.26764585	0.521	0.576	1.50E-07	FYB	3	CCL20_CD8 MAIT
725	6.96E-11	-0.26807597	0.104	0.181	1.52E-06	HSH2D	3	CCL20_CD8 MAIT
726	1.33E-11	-0.26834504	0.516	0.573	2.90E-07	DYNLL1	3	CCL20_CD8 MAIT
727	7.50E-08	-0.26969625	0.174	0.237	0.001636152	MTHFD2	3	CCL20_CD8 MAIT
728	1.76E-10	-0.27001932	0.209	0.293	3.84E-06	DCXR	3	CCL20_CD8 MAIT
729	1.11E-11	-0.27118798	0.851	0.816	2.43E-07	HMGB1	3	CCL20_CD8 MAIT
730	8.71E-57	-0.27173418	0.998	0.996	1.90E-52	RPS27	3	CCL20_CD8 MAIT
731	1.20E-08	-0.27179194	0.209	0.277	0.000260887	UPP1	3	CCL20_CD8 MAIT
732	3.82E-24	-0.27298234	0.979	0.889	8.34E-20	CCL5	3	CCL20_CD8 MAIT
733	8.99E-08	-0.27374827	0.151	0.209	0.00196027	TBX21	3	CCL20_CD8 MAIT
734	3.72E-11	-0.27469983	0.127	0.207	8.12E-07	SH3BP5	3	CCL20_CD8 MAIT
735	1.17E-20	-0.27650954	0.724	0.76	2.55E-16	SUB1	3	CCL20_CD8 MAIT
736	4.58E-07	-0.27711485	0.204	0.261	0.00997955	S1PR5	3	CCL20_CD8 MAIT
737	1.17E-06	-0.2776778	0.293	0.332	0.025497019	SELPLG	3	CCL20_CD8 MAIT
738	1.15E-09	-0.27809636	0.131	0.204	2.51E-05	SRRT	3	CCL20_CD8 MAIT
739	2.14E-11	-0.28197445	0.184	0.27	4.66E-07	IL10RA	3	CCL20_CD8 MAIT
740	1.12E-11	-0.28321001	0.381	0.444	2.44E-07	RAC1	3	CCL20_CD8 MAIT
741	1.50E-07	-0.28363625	0.297	0.352	0.0032714	HLA-DQB1	3	CCL20_CD8 MAIT
742	8.49E-56	-0.28508962	0.994	0.993	1.85E-51	TMSB10	3	CCL20_CD8 MAIT
743	6.58E-10	-0.28515079	0.404	0.448	1.43E-05	IFI16	3	CCL20_CD8 MAIT
744	1.24E-10	-0.2860246	0.408	0.457	2.69E-06	APMAP	3	CCL20_CD8 MAIT
745	2.65E-12	-0.2871987	0.262	0.349	5.78E-08	TECR	3	CCL20_CD8 MAIT
746	3.52E-13	-0.28952828	0.521	0.575	7.68E-09	TRBC2	3	CCL20_CD8 MAIT
747	1.04E-17	-0.2966906	0.635	0.672	2.27E-13	RAP1B	3	CCL20_CD8 MAIT
748	4.09E-10	-0.29837376	0.247	0.316	8.93E-06	HN1	3	CCL20_CD8 MAIT
749	1.63E-08	-0.29984939	0.177	0.244	0.000355895	TUBA1C	3	CCL20_CD8 MAIT
750	1.39E-07	-0.30532333	0.178	0.237	0.003035753	IFITM3	3	CCL20_CD8 MAIT
751	1.31E-07	-0.30745772	0.218	0.274	0.002866571	LYST	3	CCL20_CD8 MAIT
752	3.83E-27	-0.30923236	0.798	0.815	8.36E-23	ARPC2	3	CCL20_CD8 MAIT
753	4.70E-13	-0.30987298	0.109	0.195	1.03E-08	MIR4435-2HG	3	CCL20_CD8 MAIT
754	1.53E-11	-0.31507687	0.168	0.252	3.33E-07	GNPTAB	3	CCL20_CD8 MAIT
755	5.22E-11	-0.31587802	0.19	0.269	1.14E-06	STARD3NL	3	CCL20_CD8 MAIT
756	1.22E-11	-0.31619523	0.136	0.214	2.66E-07	CDC25B	3	CCL20_CD8 MAIT
757	4.13E-14	-0.32081491	0.11	0.2	9.00E-10	CDKN2D	3	CCL20_CD8 MAIT
758	1.04E-11	-0.32148254	0.331	0.401	2.27E-07	SERPINB1	3	CCL20_CD8 MAIT
759	1.13E-14	-0.32810704	0.129	0.227	2.46E-10	RASGRP2	3	CCL20_CD8 MAIT
760	6.74E-12	-0.3290263	0.739	0.767	1.47E-07	GADD45B	3	CCL20_CD8 MAIT
761	1.86E-15	-0.33733245	0.254	0.352	4.06E-11	ADD3	3	CCL20_CD8 MAIT
762	1.76E-12	-0.33774966	0.17	0.256	3.83E-08	EFHD2	3	CCL20_CD8 MAIT
763	2.95E-14	-0.3448467	0.261	0.356	6.42E-10	ITGA4	3	CCL20_CD8 MAIT
764	2.76E-13	-0.35396258	0.291	0.378	6.01E-09	FLNA	3	CCL20_CD8 MAIT
765	1.25E-26	-0.36012878	0.589	0.658	2.73E-22	LSP1	3	CCL20_CD8 MAIT
766	2.93E-17	-0.36093569	0.498	0.559	6.40E-13	CALR	3	CCL20_CD8 MAIT
767	6.83E-23	-0.36464682	0.903	0.86	1.49E-18	CST7	3	CCL20_CD8 MAIT
768	6.44E-13	-0.36470818	0.598	0.624	1.40E-08	H2AFZ	3	CCL20_CD8 MAIT

769	2.90E-18	-0.36580431	0.391	0.481	6.31E-14	CTSD	3	CCL20_CD8 MAIT
770	2.06E-12	-0.36699124	0.341	0.407	4.48E-08	PLEK	3	CCL20_CD8 MAIT
771	1.82E-16	-0.36875701	0.469	0.553	3.98E-12	TUBB4B	3	CCL20_CD8 MAIT
772	6.73E-17	-0.37426616	0.322	0.412	1.47E-12	CD63	3	CCL20_CD8 MAIT
773	1.94E-33	-0.38816826	0.768	0.804	4.24E-29	CYBA	3	CCL20_CD8 MAIT
774	2.07E-21	-0.39705829	0.106	0.228	4.52E-17	RASA3	3	CCL20_CD8 MAIT
775	7.83E-20	-0.40719366	0.326	0.44	1.71E-15	TMEM2	3	CCL20_CD8 MAIT
776	1.01E-27	-0.41041107	0.62	0.678	2.20E-23	ITGB2	3	CCL20_CD8 MAIT
777	7.65E-15	-0.41085393	0.359	0.478	1.67E-10	HSPA1A	3	CCL20_CD8 MAIT
778	7.94E-22	-0.4188325	0.144	0.269	1.73E-17	PXN	3	CCL20_CD8 MAIT
779	1.56E-21	-0.42144593	0.64	0.667	3.40E-17	CRIP1	3	CCL20_CD8 MAIT
780	1.56E-23	-0.43137818	0.292	0.415	3.40E-19	MBP	3	CCL20_CD8 MAIT
781	9.03E-66	-0.44676769	0.943	0.949	1.97E-61	IFITM1	3	CCL20_CD8 MAIT
782	9.58E-08	-0.45164539	0.548	0.551	0.002089677	HMGN2	3	CCL20_CD8 MAIT
783	6.81E-35	-0.45741308	0.51	0.626	1.48E-30	EMP3	3	CCL20_CD8 MAIT
784	1.51E-09	-0.47690862	0.395	0.472	3.29E-05	CCL3	3	CCL20_CD8 MAIT
785	3.64E-17	-0.47875951	0.174	0.281	7.94E-13	COTL1	3	CCL20_CD8 MAIT
786	6.00E-33	-0.48408954	0.185	0.36	1.31E-28	KLF2	3	CCL20_CD8 MAIT
787	1.34E-30	-0.49855955	0.333	0.468	2.93E-26	HLA-DPA1	3	CCL20_CD8 MAIT
788	3.41E-25	-0.49954818	0.113	0.246	7.44E-21	AOAH	3	CCL20_CD8 MAIT
789	2.49E-41	-0.50366807	0.502	0.646	5.43E-37	LITAF	3	CCL20_CD8 MAIT
790	3.58E-30	-0.50952277	0.191	0.345	7.80E-26	LINC00152	3	CCL20_CD8 MAIT
791	4.43E-32	-0.53524457	0.546	0.62	9.65E-28	CD247	3	CCL20_CD8 MAIT
792	1.07E-26	-0.53667673	0.232	0.374	2.34E-22	SAMSN1	3	CCL20_CD8 MAIT
793	3.41E-13	-0.55130882	0.423	0.473	7.43E-09	ARL6IP1	3	CCL20_CD8 MAIT
794	2.28E-38	-0.55462884	0.244	0.417	4.97E-34	DOK2	3	CCL20_CD8 MAIT
795	3.65E-19	-0.56635067	0.747	0.693	7.96E-15	PRF1	3	CCL20_CD8 MAIT
796	1.86E-68	-0.57014931	0.889	0.886	4.05E-64	IFITM2	3	CCL20_CD8 MAIT
797	2.33E-33	-0.5882027	0.648	0.673	5.07E-29	CTSW	3	CCL20_CD8 MAIT
798	8.97E-10	-0.60585303	0.452	0.483	1.96E-05	TUBB	3	CCL20_CD8 MAIT
799	4.57E-42	-0.64292797	0.125	0.313	9.96E-38	ITGB1	3	CCL20_CD8 MAIT
800	5.58E-41	-0.64402138	0.116	0.303	1.22E-36	HLA-DRB5	3	CCL20_CD8 MAIT
801	4.07E-52	-0.6510967	0.324	0.555	8.88E-48	TRBC1	3	CCL20_CD8 MAIT
802	3.08E-08	-0.66780444	0.307	0.348	0.000672576	HMGB2	3	CCL20_CD8 MAIT
803	4.13E-53	-0.67011345	0.173	0.399	9.00E-49	C12orf75	3	CCL20_CD8 MAIT
804	3.09E-70	-0.7122492	0.722	0.815	6.73E-66	CD74	3	CCL20_CD8 MAIT
805	3.01E-59	-0.71580049	0.266	0.488	6.57E-55	HLA-DPB1	3	CCL20_CD8 MAIT
806	2.41E-29	-0.75970432	0.516	0.601	5.26E-25	TUBA1B	3	CCL20_CD8 MAIT
807	1.21E-72	-0.79559628	0.344	0.576	2.64E-68	HLA-DRB1	3	CCL20_CD8 MAIT
808	2.90E-85	-0.83637564	0.334	0.587	6.33E-81	GSTP1	3	CCL20_CD8 MAIT
809	6.08E-50	-0.88340563	0.104	0.311	1.33E-45	CLIC3	3	CCL20_CD8 MAIT
810	1.80E-58	-0.89541267	0.289	0.51	3.92E-54	TYROBP	3	CCL20_CD8 MAIT
811	3.90E-100	-0.91830027	0.954	0.861	8.50E-96	NKG7	3	CCL20_CD8 MAIT
812	6.72E-80	-0.92859744	0.133	0.42	1.46E-75	ZEB2	3	CCL20_CD8 MAIT
813	2.12E-14	-0.95374962	0.258	0.344	4.63E-10	STMN1	3	CCL20_CD8 MAIT
814	2.92E-38	-0.95399276	0.242	0.432	6.36E-34	AREG	3	CCL20_CD8 MAIT
815	1.69E-55	-0.96935743	0.179	0.411	3.68E-51	LGALS1	3	CCL20_CD8 MAIT
816	4.40E-80	-1.00757532	0.388	0.626	9.59E-76	CMC1	3	CCL20_CD8 MAIT
817	5.20E-75	-1.01520235	0.157	0.429	1.13E-70	KLRF1	3	CCL20_CD8 MAIT
818	1.51E-71	-1.07955456	0.208	0.46	3.28E-67	SPON2	3	CCL20_CD8 MAIT
819	1.29E-143	-1.34180563	0.282	0.641	2.81E-139	KLRD1	3	CCL20_CD8 MAIT
820	1.05E-131	-1.36231115	0.244	0.59	2.28E-127	GZMH	3	CCL20_CD8 MAIT
821	1.41E-180	1.13547324	0.9	0.352	3.07E-176	IL7R	4	IL7R_CD4 T
822	1.18E-125	0.87462487	0.433	0.097	2.56E-121	GPR183	4	IL7R_CD4 T
823	1.10E-65	0.83389271	0.571	0.26	2.41E-61	LTB	4	IL7R_CD4 T
824	1.26E-59	0.83013083	0.502	0.225	2.74E-55	RGCC	4	IL7R_CD4 T
825	1.26E-168	0.78575369	0.233	0.013	2.75E-164	LEF1	4	IL7R_CD4 T
826	5.36E-76	0.74107841	0.355	0.101	1.17E-71	CD40LG	4	IL7R_CD4 T
827	7.78E-44	0.71533524	0.206	0.057	1.70E-39	SELL	4	IL7R_CD4 T
828	9.35E-208	0.63159542	0.236	0.007	2.04E-203	CD4	4	IL7R_CD4 T
829	1.14E-52	0.61968685	0.71	0.481	2.48E-48	LDHB	4	IL7R_CD4 T
830	5.49E-121	0.60383663	0.139	0.005	1.20E-116	CCR7	4	IL7R_CD4 T
831	2.31E-61	0.59162441	0.537	0.228	5.05E-57	COTL1	4	IL7R_CD4 T
832	5.14E-116	0.5741857	0.994	0.96	1.12E-111	RPLP0	4	IL7R_CD4 T
833	7.38E-50	0.57408723	0.811	0.567	1.61E-45	CD52	4	IL7R_CD4 T
834	1.37E-41	0.51158156	0.342	0.136	3.00E-37	IL6ST	4	IL7R_CD4 T
835	5.94E-39	0.50109447	0.441	0.214	1.30E-34	PLP2	4	IL7R_CD4 T
836	2.49E-40	0.49576509	0.463	0.229	5.42E-36	TMEM123	4	IL7R_CD4 T
837	2.98E-73	0.49576495	0.948	0.858	6.50E-69	EEF1B2	4	IL7R_CD4 T
838	2.77E-128	0.48979299	0.998	0.997	6.03E-124	RPS12	4	IL7R_CD4 T

839	1.36E-118	0.48530925	0.998	0.992	2.97E-114	TPT1	4	IL7R_CD4 T
840	4.98E-24	0.48525012	0.217	0.09	1.09E-19	LMNA	4	IL7R_CD4 T
841	1.74E-59	0.48198496	0.948	0.863	3.80E-55	SARAF	4	IL7R_CD4 T
842	2.25E-159	0.47945248	0.152	0.002	4.92E-155	MAL	4	IL7R_CD4 T
843	9.84E-84	0.47421404	0.986	0.927	2.15E-79	RPL9	4	IL7R_CD4 T
844	3.64E-36	0.47292047	0.119	0.023	7.93E-32	TNFRSF4	4	IL7R_CD4 T
845	1.27E-54	0.47274025	0.244	0.064	2.78E-50	RCAN3	4	IL7R_CD4 T
846	2.86E-130	0.47060815	1	0.997	6.24E-126	RPL34	4	IL7R_CD4 T
847	5.65E-46	0.46745321	0.252	0.076	1.23E-41	ICOS	4	IL7R_CD4 T
848	3.18E-124	0.46420058	0.997	0.995	6.94E-120	RPL32	4	IL7R_CD4 T
849	1.42E-83	0.46025941	0.976	0.947	3.10E-79	RPS5	4	IL7R_CD4 T
850	2.28E-112	0.45919104	1	0.995	4.97E-108	RPS8	4	IL7R_CD4 T
851	4.99E-134	0.4560245	1	0.997	1.09E-129	RPL13	4	IL7R_CD4 T
852	3.02E-122	0.45281247	1	0.999	6.58E-118	RPLP1	4	IL7R_CD4 T
853	1.86E-32	0.45228058	0.626	0.397	4.06E-28	CD44	4	IL7R_CD4 T
854	1.90E-46	0.45178448	0.857	0.684	4.15E-42	FXYD5	4	IL7R_CD4 T
855	3.30E-37	0.45061393	0.206	0.063	7.19E-33	TCF7	4	IL7R_CD4 T
856	6.35E-97	0.44998993	0.992	0.975	1.38E-92	RPL36	4	IL7R_CD4 T
857	1.25E-134	0.44903931	0.998	0.995	2.72E-130	RPL11	4	IL7R_CD4 T
858	5.58E-28	0.44782227	0.49	0.284	1.22E-23	NDFIP1	4	IL7R_CD4 T
859	1.32E-124	0.4425056	1	0.997	2.88E-120	RPL39	4	IL7R_CD4 T
860	3.71E-38	0.43975108	0.38	0.165	8.09E-34	CAMK4	4	IL7R_CD4 T
861	7.50E-98	0.43860719	0.998	0.988	1.64E-93	RPS13	4	IL7R_CD4 T
862	5.15E-102	0.43752184	0.992	0.988	1.12E-97	RPS18	4	IL7R_CD4 T
863	1.51E-113	0.43145238	0.994	0.991	3.30E-109	RPL18A	4	IL7R_CD4 T
864	1.72E-111	0.42936661	0.998	0.988	3.74E-107	RPL18	4	IL7R_CD4 T
865	1.27E-18	0.42928346	0.347	0.198	2.78E-14	TOB1	4	IL7R_CD4 T
866	4.31E-18	0.42880709	0.219	0.104	9.39E-14	SGK1	4	IL7R_CD4 T
867	7.28E-40	0.42475708	0.853	0.735	1.59E-35	EEF1G	4	IL7R_CD4 T
868	7.08E-28	0.42386832	0.984	0.96	1.54E-23	FTH1	4	IL7R_CD4 T
869	3.05E-38	0.42278818	0.788	0.595	6.65E-34	EIF3E	4	IL7R_CD4 T
870	6.57E-125	0.41937219	1	0.995	1.43E-120	RPS28	4	IL7R_CD4 T
871	5.89E-98	0.41655408	0.994	0.986	1.29E-93	RPL12	4	IL7R_CD4 T
872	1.91E-125	0.41628991	1	0.998	4.16E-121	RPL10	4	IL7R_CD4 T
873	1.15E-68	0.41507643	0.986	0.952	2.51E-64	RPS2	4	IL7R_CD4 T
874	2.09E-98	0.41345608	0.997	0.986	4.55E-94	RPS21	4	IL7R_CD4 T
875	1.27E-50	0.41130185	0.924	0.84	2.76E-46	RPL7	4	IL7R_CD4 T
876	5.55E-30	0.41070179	0.368	0.175	1.21E-25	INPP4B	4	IL7R_CD4 T
877	5.19E-89	0.40267987	0.997	0.984	1.13E-84	RPS25	4	IL7R_CD4 T
878	8.92E-42	0.3987634	0.895	0.776	1.94E-37	RPL36A	4	IL7R_CD4 T
879	1.53E-91	0.39772491	0.99	0.985	3.33E-87	RPS10	4	IL7R_CD4 T
880	2.83E-68	0.39514104	0.978	0.956	6.16E-64	RPL10A	4	IL7R_CD4 T
881	1.97E-75	0.39233505	0.998	0.982	4.30E-71	RPS6	4	IL7R_CD4 T
882	5.79E-116	0.39202368	1	0.996	1.26E-111	RPL30	4	IL7R_CD4 T
883	1.31E-22	0.39175432	0.238	0.106	2.87E-18	SLAMF1	4	IL7R_CD4 T
884	3.92E-98	0.39124295	1	0.99	8.54E-94	RPLP2	4	IL7R_CD4 T
885	1.11E-61	0.39104789	0.962	0.924	2.42E-57	RPS9	4	IL7R_CD4 T
886	3.88E-60	0.39058616	0.987	0.956	8.46E-56	RPL22	4	IL7R_CD4 T
887	1.09E-99	0.39025504	0.998	0.991	2.38E-95	RPL37	4	IL7R_CD4 T
888	1.92E-29	0.39007085	0.338	0.156	4.19E-25	AQP3	4	IL7R_CD4 T
889	7.74E-38	0.38717312	0.135	0.029	1.69E-33	FRMD4B	4	IL7R_CD4 T
890	1.13E-98	0.38716008	0.998	0.992	2.46E-94	RPS14	4	IL7R_CD4 T
891	3.04E-17	0.38537341	0.487	0.33	6.62E-13	SAMSN1	4	IL7R_CD4 T
892	1.94E-85	0.3815449	0.995	0.987	4.23E-81	RPL14	4	IL7R_CD4 T
893	1.01E-60	0.37620401	0.155	0.023	2.20E-56	TRABD2A	4	IL7R_CD4 T
894	1.09E-85	0.37189569	0.997	0.985	2.37E-81	RPL29	4	IL7R_CD4 T
895	1.27E-100	0.37161043	0.998	0.998	2.77E-96	AC090498.1	4	IL7R_CD4 T
896	4.05E-12	0.37091903	0.274	0.163	8.84E-08	SOD2	4	IL7R_CD4 T
897	3.12E-86	0.37072248	1	0.994	6.79E-82	RPS3A	4	IL7R_CD4 T
898	3.51E-68	0.37004607	0.994	0.969	7.64E-64	RPL8	4	IL7R_CD4 T
899	1.11E-28	0.36878921	0.33	0.152	2.43E-24	CCDC109B	4	IL7R_CD4 T
900	4.91E-23	0.36751984	0.303	0.15	1.07E-18	PAG1	4	IL7R_CD4 T
901	2.22E-25	0.36553459	0.417	0.227	4.84E-21	RNASET2	4	IL7R_CD4 T
902	4.35E-11	0.36192579	0.298	0.188	9.48E-07	BIRC3	4	IL7R_CD4 T
903	1.92E-93	0.36113118	0.997	0.992	4.19E-89	RPS23	4	IL7R_CD4 T
904	2.68E-11	0.36083881	0.734	0.642	5.84E-07	ANXA1	4	IL7R_CD4 T
905	2.37E-28	0.36038084	0.214	0.079	5.17E-24	THEM4	4	IL7R_CD4 T
906	2.65E-23	0.35986091	0.704	0.547	5.78E-19	GLTSCR2	4	IL7R_CD4 T
907	3.71E-23	0.35635685	0.228	0.097	8.10E-19	TIMP1	4	IL7R_CD4 T
908	1.61E-38	0.35619551	0.162	0.04	3.51E-34	SH3YL1	4	IL7R_CD4 T

909	2.03E-92	0.35427524	0.998	0.994	4.42E-88	RPL19	4	IL7R_CD4 T
910	1.85E-13	0.35186782	0.388	0.256	4.03E-09	AIM1	4	IL7R_CD4 T
911	1.09E-43	0.35163645	0.954	0.888	2.37E-39	RPL38	4	IL7R_CD4 T
912	1.78E-60	0.34548836	0.981	0.953	3.88E-56	RPL24	4	IL7R_CD4 T
913	9.38E-24	0.34439535	0.212	0.086	2.05E-19	CCR6	4	IL7R_CD4 T
914	8.65E-81	0.34400833	0.992	0.989	1.89E-76	RPL35A	4	IL7R_CD4 T
915	7.15E-10	0.34344129	0.498	0.369	1.56E-05	HSPD1	4	IL7R_CD4 T
916	8.73E-14	0.34225466	0.702	0.552	1.90E-09	TNFAIP3	4	IL7R_CD4 T
917	6.05E-49	0.34194825	0.989	0.961	1.32E-44	RPSA	4	IL7R_CD4 T
918	5.03E-67	0.34159556	0.99	0.987	1.10E-62	RPS4X	4	IL7R_CD4 T
919	4.54E-106	0.33815895	0.125	0.004	9.91E-102	IL6R	4	IL7R_CD4 T
920	1.33E-16	0.33702571	0.269	0.144	2.90E-12	SOCS3	4	IL7R_CD4 T
921	3.26E-100	0.33644626	1	1	7.11E-96	EEF1A1	4	IL7R_CD4 T
922	3.02E-21	0.33568761	0.537	0.345	6.59E-17	SPOCK2	4	IL7R_CD4 T
923	1.58E-34	0.33566568	0.933	0.851	3.44E-30	RPL4	4	IL7R_CD4 T
924	1.41E-50	0.3340016	0.984	0.971	3.07E-46	RPL5	4	IL7R_CD4 T
925	2.25E-11	0.331712	0.388	0.271	4.90E-07	UGP2	4	IL7R_CD4 T
926	4.90E-17	0.32818613	0.891	0.799	1.07E-12	KLF6	4	IL7R_CD4 T
927	1.03E-47	0.32750254	0.976	0.948	2.24E-43	GNB2L1	4	IL7R_CD4 T
928	4.66E-32	0.32646587	0.929	0.81	1.02E-27	VIM	4	IL7R_CD4 T
929	2.85E-20	0.32610437	0.628	0.447	6.22E-16	LEPROTL1	4	IL7R_CD4 T
930	1.12E-81	0.32428972	0.997	0.994	2.45E-77	RPS15	4	IL7R_CD4 T
931	1.32E-18	0.32380645	0.3	0.16	2.88E-14	TTC39C	4	IL7R_CD4 T
932	1.16E-87	0.32374953	0.997	0.998	2.54E-83	RPS27A	4	IL7R_CD4 T
933	3.04E-54	0.32274802	0.986	0.97	6.63E-50	RPS16	4	IL7R_CD4 T
934	3.91E-09	0.32226733	0.455	0.347	8.52E-05	CD55	4	IL7R_CD4 T
935	4.80E-12	0.31994191	0.537	0.398	1.05E-07	EZR	4	IL7R_CD4 T
936	3.49E-10	0.31962994	0.42	0.299	7.61E-06	HSPB1	4	IL7R_CD4 T
937	6.53E-42	0.31851786	0.128	0.024	1.42E-37	NCF4	4	IL7R_CD4 T
938	1.22E-21	0.31548702	0.32	0.162	2.65E-17	TRAT1	4	IL7R_CD4 T
939	2.00E-41	0.31379394	0.987	0.958	4.35E-37	RPS29	4	IL7R_CD4 T
940	3.13E-16	0.31324286	0.559	0.397	6.83E-12	SNHG8	4	IL7R_CD4 T
941	1.46E-17	0.31150298	0.317	0.174	3.19E-13	PDE4B	4	IL7R_CD4 T
942	9.51E-51	0.3105093	0.989	0.976	2.07E-46	RPL17	4	IL7R_CD4 T
943	4.39E-27	0.30976924	0.19	0.067	9.57E-23	TNFRSF25	4	IL7R_CD4 T
944	4.03E-71	0.30957239	0.998	0.997	8.78E-67	RPS27	4	IL7R_CD4 T
945	1.37E-23	0.30863109	0.903	0.856	2.98E-19	PABPC1	4	IL7R_CD4 T
946	1.93E-13	0.30820866	0.39	0.258	4.21E-09	RGS10	4	IL7R_CD4 T
947	1.25E-20	0.30761888	0.211	0.091	2.72E-16	CD28	4	IL7R_CD4 T
948	2.58E-57	0.30559843	0.992	0.987	5.64E-53	RPL6	4	IL7R_CD4 T
949	7.23E-51	0.3053723	1	0.997	1.58E-46	RPS19	4	IL7R_CD4 T
950	7.25E-22	0.30459613	0.17	0.064	1.58E-17	SNX9	4	IL7R_CD4 T
951	1.43E-06	0.30425513	0.675	0.601	0.031281881	CITED2	4	IL7R_CD4 T
952	4.04E-23	0.30136221	0.857	0.75	8.82E-19	HINT1	4	IL7R_CD4 T
953	4.60E-35	0.30105016	0.143	0.033	1.00E-30	AHR	4	IL7R_CD4 T
954	1.74E-17	0.29941759	0.406	0.246	3.79E-13	CD6	4	IL7R_CD4 T
955	2.14E-18	0.29911879	0.165	0.067	4.66E-14	MYC	4	IL7R_CD4 T
956	2.84E-84	0.29898903	0.997	0.998	6.19E-80	RPS15A	4	IL7R_CD4 T
957	6.38E-45	0.29740573	0.989	0.952	1.39E-40	RPL13A	4	IL7R_CD4 T
958	3.04E-81	0.29422519	1	0.998	6.63E-77	RPL28	4	IL7R_CD4 T
959	4.82E-07	0.29395109	0.401	0.312	0.010507058	BATF	4	IL7R_CD4 T
960	6.47E-07	0.29354242	0.334	0.257	0.014109719	NOSIP	4	IL7R_CD4 T
961	6.04E-73	0.29339885	0.998	0.994	1.32E-68	RPS3	4	IL7R_CD4 T
962	2.50E-13	0.29074954	0.409	0.267	5.44E-09	ITK	4	IL7R_CD4 T
963	5.31E-15	0.29016863	0.233	0.123	1.16E-10	SYPL1	4	IL7R_CD4 T
964	1.20E-36	0.28849634	0.124	0.025	2.61E-32	SERINC5	4	IL7R_CD4 T
965	1.61E-17	0.2862851	0.277	0.146	3.50E-13	FLT3LG	4	IL7R_CD4 T
966	3.63E-09	0.28574849	0.334	0.234	7.91E-05	FOXP1	4	IL7R_CD4 T
967	1.98E-15	0.28538037	0.246	0.129	4.32E-11	OXNAD1	4	IL7R_CD4 T
968	1.79E-17	0.2811509	0.204	0.094	3.91E-13	TNFSF8	4	IL7R_CD4 T
969	9.53E-60	0.28008414	0.992	0.99	2.08E-55	RPS7	4	IL7R_CD4 T
970	7.42E-15	0.27954827	0.227	0.117	1.62E-10	TRADD	4	IL7R_CD4 T
971	4.11E-15	0.27797186	0.417	0.266	8.97E-11	RSL1D1	4	IL7R_CD4 T
972	2.92E-19	0.27789583	0.255	0.122	6.36E-15	CD5	4	IL7R_CD4 T
973	1.02E-22	0.27507143	0.903	0.836	2.22E-18	EEF2	4	IL7R_CD4 T
974	8.24E-26	0.27274307	0.138	0.04	1.80E-21	KDSR	4	IL7R_CD4 T
975	2.54E-24	0.27226801	0.932	0.858	5.55E-20	RPL31	4	IL7R_CD4 T
976	8.42E-41	0.27080131	0.989	0.98	1.84E-36	RPL3	4	IL7R_CD4 T
977	3.90E-40	0.26925934	0.995	0.979	8.51E-36	RPL21	4	IL7R_CD4 T
978	1.40E-19	0.26902518	0.179	0.073	3.04E-15	RP11-18H21.1	4	IL7R_CD4 T

979	1.90E-25	0.26899554	0.937	0.889	4.13E-21	RPL27	4	IL7R_CD4 T
980	1.06E-16	0.26806561	0.157	0.065	2.31E-12	PDE3B	4	IL7R_CD4 T
981	2.28E-23	0.26720078	0.181	0.067	4.97E-19	TMEM14C	4	IL7R_CD4 T
982	1.82E-20	0.26698052	0.197	0.082	3.96E-16	CDC14A	4	IL7R_CD4 T
983	4.80E-12	0.26634434	0.274	0.163	1.05E-07	CORO1B	4	IL7R_CD4 T
984	1.22E-31	0.2629914	0.973	0.95	2.67E-27	RPL35	4	IL7R_CD4 T
985	2.72E-12	0.25957708	0.58	0.443	5.92E-08	GSTK1	4	IL7R_CD4 T
986	2.04E-31	0.2586483	0.976	0.955	4.45E-27	NACA	4	IL7R_CD4 T
987	1.20E-41	0.25803748	0.987	0.978	2.61E-37	RPL23A	4	IL7R_CD4 T
988	6.51E-12	0.25797439	0.249	0.143	1.42E-07	NCF1	4	IL7R_CD4 T
989	2.41E-17	0.25769446	0.846	0.765	5.27E-13	NPM1	4	IL7R_CD4 T
990	1.67E-56	0.25721179	0.997	0.993	3.64E-52	RPL26	4	IL7R_CD4 T
991	1.09E-16	0.2569175	0.219	0.105	2.37E-12	DGKA	4	IL7R_CD4 T
992	2.75E-28	0.25668282	0.973	0.916	5.99E-24	RPL41	4	IL7R_CD4 T
993	1.19E-25	0.25602499	0.13	0.037	2.59E-21	CD82	4	IL7R_CD4 T
994	2.82E-09	0.25570517	0.425	0.316	6.15E-05	ARHGAP15	4	IL7R_CD4 T
995	8.11E-23	0.25523456	0.921	0.862	1.77E-18	RPS11	4	IL7R_CD4 T
996	5.29E-11	0.25327866	0.586	0.466	1.15E-06	SMDT1	4	IL7R_CD4 T
997	2.10E-19	0.25258871	0.149	0.055	4.58E-15	TESPA1	4	IL7R_CD4 T
998	4.74E-11	0.2515504	0.979	0.966	1.03E-06	ZFP36L2	4	IL7R_CD4 T
999	2.24E-18	0.25041022	0.136	0.049	4.89E-14	TNFRSF18	4	IL7R_CD4 T
1000	4.12E-07	-0.25027256	0.155	0.238	0.008979151	HSPB11	4	IL7R_CD4 T
1001	2.51E-07	-0.25044325	0.187	0.28	0.00547891	NUCB2	4	IL7R_CD4 T
1002	2.30E-08	-0.25075627	0.113	0.203	0.000502113	UBE2E3	4	IL7R_CD4 T
1003	4.65E-07	-0.25128307	0.274	0.368	0.010135434	GBP5	4	IL7R_CD4 T
1004	6.07E-08	-0.25245351	0.17	0.265	0.001324088	TSEN54	4	IL7R_CD4 T
1005	1.54E-07	-0.25390435	0.127	0.213	0.003360361	NFKBIB	4	IL7R_CD4 T
1006	2.17E-09	-0.25526789	0.306	0.42	4.73E-05	POMP	4	IL7R_CD4 T
1007	1.12E-09	-0.25582689	0.532	0.601	2.45E-05	TMBIM6	4	IL7R_CD4 T
1008	6.49E-09	-0.25660904	0.255	0.363	0.000141581	RUNX3	4	IL7R_CD4 T
1009	1.28E-09	-0.25929241	0.138	0.245	2.80E-05	RAB8A	4	IL7R_CD4 T
1010	1.40E-07	-0.26005364	0.212	0.303	0.003055447	DCTN3	4	IL7R_CD4 T
1011	1.06E-08	-0.26083466	0.293	0.394	0.00023125	BTN3A2	4	IL7R_CD4 T
1012	3.07E-07	-0.26111853	0.113	0.192	0.006701529	CDKN2D	4	IL7R_CD4 T
1013	1.73E-09	-0.2617869	0.353	0.457	3.77E-05	ARPC5	4	IL7R_CD4 T
1014	4.56E-09	-0.26213278	0.353	0.453	9.95E-05	ANXA6	4	IL7R_CD4 T
1015	3.04E-09	-0.26247404	0.288	0.409	6.64E-05	STOM	4	IL7R_CD4 T
1016	1.00E-09	-0.26279654	0.284	0.402	2.18E-05	SH2D1A	4	IL7R_CD4 T
1017	2.08E-08	-0.26339233	0.261	0.365	0.000453731	AKNA	4	IL7R_CD4 T
1018	1.14E-08	-0.26509225	0.371	0.466	0.000247906	ARL4C	4	IL7R_CD4 T
1019	2.16E-06	-0.26518672	0.144	0.218	0.047136405	GPATCH8	4	IL7R_CD4 T
1020	4.24E-08	-0.26539717	0.117	0.205	0.000923972	ACAA2	4	IL7R_CD4 T
1021	8.07E-15	-0.26544266	0.746	0.816	1.76E-10	CORO1A	4	IL7R_CD4 T
1022	3.58E-07	-0.26552524	0.368	0.44	0.007810823	RAC1	4	IL7R_CD4 T
1023	1.42E-08	-0.26719394	0.311	0.408	0.000309207	TBC1D10C	4	IL7R_CD4 T
1024	5.16E-09	-0.26761606	0.128	0.228	0.000112636	MIEN1	4	IL7R_CD4 T
1025	2.08E-06	-0.26766875	0.149	0.235	0.045392139	CD8B	4	IL7R_CD4 T
1026	1.95E-11	-0.26768003	0.49	0.591	4.25E-07	ADGRE5	4	IL7R_CD4 T
1027	3.33E-08	-0.26799421	0.563	0.609	0.000726108	EMP3	4	IL7R_CD4 T
1028	1.91E-09	-0.26834231	0.263	0.373	4.16E-05	PTP4A2	4	IL7R_CD4 T
1029	7.21E-09	-0.26888077	0.468	0.555	0.000157166	HSP90B1	4	IL7R_CD4 T
1030	5.67E-21	-0.26946587	0.902	0.938	1.24E-16	PTPRC	4	IL7R_CD4 T
1031	1.79E-07	-0.26961723	0.204	0.292	0.003912795	C20orf24	4	IL7R_CD4 T
1032	6.87E-09	-0.27064497	0.222	0.329	0.000149788	ACTN4	4	IL7R_CD4 T
1033	4.51E-09	-0.27154966	0.391	0.484	9.84E-05	PSMB8	4	IL7R_CD4 T
1034	2.40E-14	-0.2737957	0.693	0.76	5.23E-10	SUB1	4	IL7R_CD4 T
1035	5.39E-08	-0.27402204	0.133	0.22	0.001175958	ADAM8	4	IL7R_CD4 T
1036	6.12E-10	-0.27433742	0.166	0.278	1.33E-05	RAB27A	4	IL7R_CD4 T
1037	7.27E-09	-0.2767663	0.185	0.289	0.000158541	TLN1	4	IL7R_CD4 T
1038	5.13E-08	-0.27713721	0.35	0.437	0.001118082	WDR1	4	IL7R_CD4 T
1039	3.94E-09	-0.27736985	0.198	0.301	8.59E-05	NDUFB7	4	IL7R_CD4 T
1040	1.45E-06	-0.27746702	0.127	0.205	0.031654281	FABP5	4	IL7R_CD4 T
1041	3.34E-20	-0.27852817	0.805	0.871	7.29E-16	H3F3A	4	IL7R_CD4 T
1042	4.80E-07	-0.27874465	0.257	0.336	0.010458876	SUN2	4	IL7R_CD4 T
1043	1.04E-07	-0.28050328	0.306	0.394	0.002257252	DOK2	4	IL7R_CD4 T
1044	1.56E-09	-0.28222693	0.304	0.417	3.41E-05	CFLAR	4	IL7R_CD4 T
1045	3.77E-10	-0.2841328	0.376	0.476	8.22E-06	CD164	4	IL7R_CD4 T
1046	2.19E-08	-0.28522453	0.122	0.211	0.000476951	PRKCB	4	IL7R_CD4 T
1047	7.25E-12	-0.28554533	0.455	0.561	1.58E-07	STK17A	4	IL7R_CD4 T
1048	7.90E-18	-0.28577862	0.756	0.827	1.72E-13	UBB	4	IL7R_CD4 T

1049	5.09E-11	-0.28623261	0.707	0.768	1.11E-06	GADD45B	4	IL7R_CD4 T
1050	1.37E-06	-0.28665503	0.158	0.234	0.029810831	SERPINB9	4	IL7R_CD4 T
1051	6.15E-12	-0.28819063	0.499	0.617	1.34E-07	RARRES3	4	IL7R_CD4 T
1052	3.55E-08	-0.28925994	0.342	0.448	0.000775129	BAX	4	IL7R_CD4 T
1053	4.11E-09	-0.28978858	0.285	0.382	8.97E-05	SCP2	4	IL7R_CD4 T
1054	3.05E-08	-0.29112794	0.139	0.23	0.000664154	ARHGAP25	4	IL7R_CD4 T
1055	1.03E-07	-0.29444316	0.124	0.205	0.002245419	ITGB7	4	IL7R_CD4 T
1056	2.32E-09	-0.29463768	0.108	0.205	5.06E-05	PTPRE	4	IL7R_CD4 T
1057	1.49E-10	-0.29617178	0.365	0.469	3.25E-06	TAPBP	4	IL7R_CD4 T
1058	5.46E-28	-0.29878695	0.767	0.87	1.19E-23	CD99	4	IL7R_CD4 T
1059	1.52E-09	-0.29933095	0.266	0.371	3.32E-05	BST2	4	IL7R_CD4 T
1060	3.35E-15	-0.29992722	0.642	0.716	7.30E-11	PSME1	4	IL7R_CD4 T
1061	3.64E-25	-0.29994847	0.81	0.885	7.93E-21	PTPRCAP	4	IL7R_CD4 T
1062	4.72E-12	-0.30076176	0.452	0.552	1.03E-07	COX8A	4	IL7R_CD4 T
1063	1.46E-07	-0.3011957	0.255	0.347	0.003192252	BHLHE40	4	IL7R_CD4 T
1064	3.22E-09	-0.30170302	0.261	0.361	7.02E-05	PRELID1	4	IL7R_CD4 T
1065	1.66E-07	-0.30184919	0.319	0.397	0.003618744	SERPINB1	4	IL7R_CD4 T
1066	2.45E-12	-0.30796412	0.347	0.467	5.34E-08	PSME2	4	IL7R_CD4 T
1067	2.06E-13	-0.31007789	0.393	0.521	4.50E-09	PPP1CA	4	IL7R_CD4 T
1068	5.09E-11	-0.31116515	0.365	0.473	1.11E-06	IQGAP1	4	IL7R_CD4 T
1069	1.62E-08	-0.31129616	0.158	0.254	0.000352448	PMAIP1	4	IL7R_CD4 T
1070	9.07E-09	-0.31247955	0.165	0.266	0.000197881	RHOB	4	IL7R_CD4 T
1071	1.29E-09	-0.31317162	0.209	0.311	2.80E-05	BANF1	4	IL7R_CD4 T
1072	1.99E-10	-0.31669396	0.238	0.355	4.34E-06	PSMB10	4	IL7R_CD4 T
1073	8.00E-10	-0.31723231	0.206	0.314	1.74E-05	UTRN	4	IL7R_CD4 T
1074	1.64E-09	-0.32039951	0.144	0.242	3.58E-05	DIP2A	4	IL7R_CD4 T
1075	1.81E-18	-0.3208113	0.635	0.743	3.95E-14	IL2RG	4	IL7R_CD4 T
1076	8.45E-12	-0.32146349	0.214	0.341	1.84E-07	ARHGAP9	4	IL7R_CD4 T
1077	6.28E-13	-0.32197136	0.209	0.343	1.37E-08	CARD16	4	IL7R_CD4 T
1078	4.69E-15	-0.32263756	0.396	0.539	1.02E-10	C9orf142	4	IL7R_CD4 T
1079	1.58E-11	-0.32278882	0.203	0.326	3.44E-07	MYO1G	4	IL7R_CD4 T
1080	8.68E-12	-0.32310352	0.176	0.305	1.89E-07	PTPN4	4	IL7R_CD4 T
1081	3.68E-14	-0.32539272	0.597	0.703	8.02E-10	NR4A2	4	IL7R_CD4 T
1082	3.85E-10	-0.32606	0.323	0.437	8.41E-06	MAPRE2	4	IL7R_CD4 T
1083	7.99E-37	-0.32835459	0.911	0.957	1.74E-32	HLA-E	4	IL7R_CD4 T
1084	1.74E-21	-0.32854052	0.886	0.921	3.80E-17	IER2	4	IL7R_CD4 T
1085	2.47E-12	-0.32962255	0.109	0.227	5.39E-08	RAB9A	4	IL7R_CD4 T
1086	5.86E-08	-0.33018367	0.225	0.312	0.001277661	CKS2	4	IL7R_CD4 T
1087	1.56E-23	-0.33025587	0.739	0.823	3.40E-19	RAC2	4	IL7R_CD4 T
1088	1.29E-10	-0.33169572	0.296	0.403	2.82E-06	MBP	4	IL7R_CD4 T
1089	2.74E-10	-0.33211559	0.163	0.274	5.97E-06	TES	4	IL7R_CD4 T
1090	1.30E-12	-0.3326538	0.374	0.487	2.83E-08	PRKCH	4	IL7R_CD4 T
1091	3.79E-17	-0.33275567	0.453	0.594	8.27E-13	CAP1	4	IL7R_CD4 T
1092	2.49E-14	-0.33363257	0.122	0.258	5.44E-10	LAG3	4	IL7R_CD4 T
1093	1.57E-12	-0.3341588	0.26	0.393	3.43E-08	UCP2	4	IL7R_CD4 T
1094	1.55E-13	-0.33852523	0.119	0.248	3.37E-09	AGTRAP	4	IL7R_CD4 T
1095	1.34E-11	-0.33888233	0.339	0.449	2.92E-07	ZAP70	4	IL7R_CD4 T
1096	7.20E-13	-0.3391677	0.257	0.382	1.57E-08	BSG	4	IL7R_CD4 T
1097	1.62E-08	-0.34041866	0.16	0.251	0.000352801	TXK	4	IL7R_CD4 T
1098	9.14E-13	-0.34291888	0.135	0.258	1.99E-08	MYO1F	4	IL7R_CD4 T
1099	2.65E-13	-0.34305641	0.151	0.285	5.78E-09	C5orf56	4	IL7R_CD4 T
1100	8.84E-17	-0.34383179	0.775	0.828	1.93E-12	HMGB1	4	IL7R_CD4 T
1101	3.73E-12	-0.34580748	0.384	0.511	8.14E-08	IRF1	4	IL7R_CD4 T
1102	7.09E-29	-0.34617802	0.914	0.952	1.55E-24	IFITM1	4	IL7R_CD4 T
1103	2.40E-10	-0.34926835	0.2	0.308	5.24E-06	SPN	4	IL7R_CD4 T
1104	7.70E-15	-0.34933143	0.361	0.504	1.68E-10	BZW1	4	IL7R_CD4 T
1105	1.96E-27	-0.34939222	0.694	0.814	4.28E-23	CLIC1	4	IL7R_CD4 T
1106	3.71E-16	-0.34966313	0.388	0.543	8.10E-12	SYTL3	4	IL7R_CD4 T
1107	3.70E-12	-0.34977953	0.228	0.356	8.07E-08	PRKACB	4	IL7R_CD4 T
1108	2.38E-09	-0.35379835	0.273	0.373	5.19E-05	FLNA	4	IL7R_CD4 T
1109	1.27E-20	-0.35433306	0.591	0.726	2.76E-16	LCP1	4	IL7R_CD4 T
1110	4.62E-12	-0.35891654	0.211	0.329	1.01E-07	PPP3CC	4	IL7R_CD4 T
1111	2.51E-18	-0.36701994	0.29	0.458	5.47E-14	DHRS7	4	IL7R_CD4 T
1112	1.46E-12	-0.36846149	0.141	0.266	3.17E-08	SYNE1	4	IL7R_CD4 T
1113	1.64E-13	-0.37336378	0.214	0.341	3.58E-09	SASH3	4	IL7R_CD4 T
1114	1.21E-17	-0.37505991	0.396	0.545	2.63E-13	CLEC2B	4	IL7R_CD4 T
1115	7.82E-41	-0.37520891	0.876	0.946	1.71E-36	CFL1	4	IL7R_CD4 T
1116	7.40E-12	-0.37714763	0.124	0.238	1.61E-07	IFITM3	4	IL7R_CD4 T
1117	6.70E-16	-0.38176655	0.162	0.314	1.46E-11	APOBEC3G	4	IL7R_CD4 T
1118	3.61E-13	-0.38327944	0.307	0.432	7.86E-09	TMEM2	4	IL7R_CD4 T

1119	5.48E-26	-0.38385007	0.624	0.799	1.20E-21	ID2	4	IL7R_CD4 T
1120	4.76E-13	-0.38420971	0.374	0.502	1.04E-08	PIK3R1	4	IL7R_CD4 T
1121	5.81E-17	-0.39283741	0.422	0.558	1.27E-12	PDIA3	4	IL7R_CD4 T
1122	8.25E-19	-0.39459876	0.344	0.512	1.80E-14	PPP1R18	4	IL7R_CD4 T
1123	1.71E-16	-0.3981757	0.177	0.332	3.73E-12	RASSF1	4	IL7R_CD4 T
1124	5.98E-17	-0.40897127	0.423	0.562	1.30E-12	CALR	4	IL7R_CD4 T
1125	4.42E-18	-0.40962306	0.244	0.406	9.63E-14	HMGN3	4	IL7R_CD4 T
1126	2.06E-83	-0.41007141	0.986	0.994	4.50E-79	HLA-A	4	IL7R_CD4 T
1127	2.32E-76	-0.41670061	0.971	0.992	5.05E-72	HLA-C	4	IL7R_CD4 T
1128	9.98E-92	-0.41719992	0.989	0.998	2.18E-87	HLA-B	4	IL7R_CD4 T
1129	6.03E-16	-0.41895769	0.166	0.314	1.32E-11	ITGAL	4	IL7R_CD4 T
1130	3.87E-35	-0.42095508	0.696	0.826	8.45E-31	ARPC2	4	IL7R_CD4 T
1131	2.26E-18	-0.42201991	0.328	0.484	4.93E-14	BIN2	4	IL7R_CD4 T
1132	1.66E-16	-0.42348173	0.136	0.281	3.63E-12	RAB29	4	IL7R_CD4 T
1133	7.79E-23	-0.4272066	0.326	0.507	1.70E-18	DBI	4	IL7R_CD4 T
1134	2.10E-25	-0.42752722	0.507	0.648	4.59E-21	7-Sep	4	IL7R_CD4 T
1135	4.36E-20	-0.42769981	0.309	0.483	9.51E-16	CTSD	4	IL7R_CD4 T
1136	3.47E-25	-0.4358696	0.544	0.68	7.57E-21	RAP1B	4	IL7R_CD4 T
1137	1.57E-30	-0.44170912	0.387	0.615	3.42E-26	FYN	4	IL7R_CD4 T
1138	5.76E-18	-0.44291153	0.19	0.352	1.26E-13	SAMD3	4	IL7R_CD4 T
1139	1.88E-11	-0.44533241	0.197	0.324	4.11E-07	IER3	4	IL7R_CD4 T
1140	5.04E-20	-0.45545679	0.179	0.354	1.10E-15	RHOC	4	IL7R_CD4 T
1141	1.72E-18	-0.45545759	0.141	0.297	3.75E-14	CHST12	4	IL7R_CD4 T
1142	3.06E-31	-0.45666857	0.455	0.643	6.68E-27	PSMB9	4	IL7R_CD4 T
1143	4.59E-78	-0.46616861	0.998	0.998	1.00E-73	ACTB	4	IL7R_CD4 T
1144	2.04E-70	-0.46985005	0.946	0.983	4.45E-66	PFN1	4	IL7R_CD4 T
1145	4.75E-25	-0.47166324	0.353	0.536	1.04E-20	SLC9A3R1	4	IL7R_CD4 T
1146	2.86E-33	-0.47201759	0.469	0.666	6.23E-29	LSP1	4	IL7R_CD4 T
1147	1.60E-24	-0.47683621	0.333	0.519	3.50E-20	FAM49B	4	IL7R_CD4 T
1148	1.57E-20	-0.48023867	0.254	0.429	3.42E-16	DENND2D	4	IL7R_CD4 T
1149	1.23E-21	-0.50479709	0.111	0.284	2.67E-17	MAPK1	4	IL7R_CD4 T
1150	3.36E-36	-0.5056666	0.837	0.893	7.33E-32	IFITM2	4	IL7R_CD4 T
1151	2.57E-28	-0.51208933	0.292	0.496	5.61E-24	ARPC5L	4	IL7R_CD4 T
1152	2.44E-24	-0.51564758	0.124	0.313	5.31E-20	TPST2	4	IL7R_CD4 T
1153	5.02E-25	-0.51636268	0.239	0.431	1.10E-20	PTPN6	4	IL7R_CD4 T
1154	1.18E-29	-0.5190676	0.469	0.641	2.58E-25	GNG2	4	IL7R_CD4 T
1155	2.51E-25	-0.51943877	0.216	0.417	5.47E-21	CD63	4	IL7R_CD4 T
1156	3.14E-10	-0.52381492	0.385	0.474	6.84E-06	ARL6IP1	4	IL7R_CD4 T
1157	6.95E-44	-0.54347618	0.655	0.815	1.52E-39	CYBA	4	IL7R_CD4 T
1158	2.94E-61	-0.54757441	0.735	0.895	6.42E-57	HCST	4	IL7R_CD4 T
1159	2.17E-20	-0.55179517	0.29	0.484	4.73E-16	GZMK	4	IL7R_CD4 T
1160	3.32E-27	-0.55539735	0.216	0.449	7.23E-23	CD8A	4	IL7R_CD4 T
1161	3.42E-22	-0.56206522	0.441	0.63	7.45E-18	KLRB1	4	IL7R_CD4 T
1162	1.80E-18	-0.57323451	0.19	0.345	3.93E-14	KLRG1	4	IL7R_CD4 T
1163	3.87E-32	-0.58823062	0.344	0.566	8.44E-28	GPR65	4	IL7R_CD4 T
1164	1.01E-31	-0.59564311	0.127	0.357	2.19E-27	MATK	4	IL7R_CD4 T
1165	1.04E-26	-0.59705959	0.155	0.364	2.27E-22	HLA-DQB1	4	IL7R_CD4 T
1166	1.15E-24	-0.60399008	0.187	0.377	2.51E-20	PLAC8	4	IL7R_CD4 T
1167	3.29E-26	-0.61412051	0.158	0.359	7.17E-22	IL2RB	4	IL7R_CD4 T
1168	4.24E-56	-0.63442956	0.732	0.876	9.25E-52	DUSP2	4	IL7R_CD4 T
1169	6.91E-49	-0.63710587	0.315	0.62	1.51E-44	CTSC	4	IL7R_CD4 T
1170	3.28E-38	-0.65126067	0.212	0.475	7.14E-34	HLA-DPB1	4	IL7R_CD4 T
1171	1.33E-37	-0.66539848	0.195	0.448	2.91E-33	PYHIN1	4	IL7R_CD4 T
1172	8.58E-51	-0.68320181	0.434	0.695	1.87E-46	ITGB2	4	IL7R_CD4 T
1173	3.71E-42	-0.68571361	0.19	0.474	8.08E-38	HLA-DPA1	4	IL7R_CD4 T
1174	2.06E-42	-0.68706562	0.266	0.55	4.50E-38	HOPX	4	IL7R_CD4 T
1175	6.62E-36	-0.6873963	0.136	0.384	1.44E-31	C12orf75	4	IL7R_CD4 T
1176	1.09E-48	-0.69582745	0.276	0.566	2.37E-44	GZMM	4	IL7R_CD4 T
1177	1.36E-45	-0.69968906	0.187	0.48	2.96E-41	APMAP	4	IL7R_CD4 T
1178	4.48E-38	-0.70366035	0.174	0.436	9.77E-34	MAP3K8	4	IL7R_CD4 T
1179	2.20E-38	-0.70562771	0.1	0.353	4.80E-34	NCR3	4	IL7R_CD4 T
1180	4.54E-38	-0.72588587	0.301	0.538	9.90E-34	TRBC1	4	IL7R_CD4 T
1181	8.43E-54	-0.73882248	0.376	0.649	1.84E-49	LITAF	4	IL7R_CD4 T
1182	1.73E-45	-0.77120019	0.315	0.567	3.77E-41	GSTP1	4	IL7R_CD4 T
1183	2.10E-70	-0.78710831	0.493	0.773	4.57E-66	CD7	4	IL7R_CD4 T
1184	4.49E-54	-0.80329305	0.372	0.635	9.79E-50	CD247	4	IL7R_CD4 T
1185	3.15E-45	-0.84967486	0.185	0.49	6.87E-41	IFNG	4	IL7R_CD4 T
1186	1.26E-54	-0.85246531	0.258	0.566	2.75E-50	HLA-DRB1	4	IL7R_CD4 T
1187	2.90E-86	-0.8869693	0.537	0.829	6.32E-82	CD74	4	IL7R_CD4 T
1188	1.01E-133	-0.99229916	0.372	0.883	2.21E-129	GZMA	4	IL7R_CD4 T

1189	3.26E-56	-1.17300871	0.132	0.447	7.12E-52	SPON2	4	IL7R_CD4 T
1190	9.79E-91	-1.32165651	0.133	0.573	2.13E-86	GZMH	4	IL7R_CD4 T
1191	6.85E-207	-1.34363067	0.483	0.957	1.49E-202	CCL5	4	IL7R_CD4 T
1192	3.03E-193	-1.3600958	0.452	0.918	6.61E-189	CST7	4	IL7R_CD4 T
1193	6.32E-137	-1.45376437	0.214	0.723	1.38E-132	CTSW	4	IL7R_CD4 T
1194	3.26E-153	-1.66792043	0.241	0.759	7.10E-149	PRF1	4	IL7R_CD4 T
1195	4.73E-204	-1.78226196	0.322	0.945	1.03E-199	CCL4	4	IL7R_CD4 T
1196	5.94E-121	-1.86302833	0.155	0.633	1.30E-116	CMC1	4	IL7R_CD4 T
1197	9.69E-133	-1.99465407	0.187	0.729	2.11E-128	CCL4L2	4	IL7R_CD4 T
1198	4.46E-303	-2.68488193	0.225	0.958	9.74E-299	NKG7	4	IL7R_CD4 T
1199	5.61E-204	2.38899168	0.387	0.034	1.22E-199	PTGDS	5	PTGDS_NK
1200	0	1.87795879	0.828	0.086	0	FCER1G	5	PTGDS_NK
1201	1.79E-271	1.37052446	0.414	0.025	3.91E-267	MYOM2	5	PTGDS_NK
1202	1.94E-96	1.30283153	0.769	0.366	4.22E-92	AREG	5	PTGDS_NK
1203	4.50E-152	1.25878262	0.88	0.374	9.81E-148	SPON2	5	PTGDS_NK
1204	1.97E-161	1.23721827	0.971	0.398	4.29E-157	GZMB	5	PTGDS_NK
1205	1.15E-73	1.21084936	0.767	0.432	2.50E-69	CCL3	5	PTGDS_NK
1206	1.08E-89	1.17189046	0.335	0.066	2.36E-85	LAIR2	5	PTGDS_NK
1207	2.64E-81	1.01338583	0.618	0.244	5.76E-77	CLIC3	5	PTGDS_NK
1208	3.21E-127	0.97119239	0.984	0.68	6.99E-123	PRF1	5	PTGDS_NK
1209	4.73E-116	0.95735628	0.826	0.302	1.03E-111	FCGR3A	5	PTGDS_NK
1210	8.40E-103	0.94698408	0.566	0.165	1.83E-98	SH2D1B	5	PTGDS_NK
1211	7.01E-112	0.89000433	0.973	0.541	1.53E-107	KLRD1	5	PTGDS_NK
1212	1.28E-103	0.86968598	0.364	0.067	2.80E-99	C1orf162	5	PTGDS_NK
1213	1.70E-92	0.86291347	0.776	0.295	3.71E-88	FGFBP2	5	PTGDS_NK
1214	6.18E-99	0.85625685	0.821	0.342	1.35E-94	KLRF1	5	PTGDS_NK
1215	7.64E-53	0.79084574	0.62	0.335	1.67E-48	PLAC8	5	PTGDS_NK
1216	1.95E-124	0.77380607	1	0.869	4.25E-120	NKG7	5	PTGDS_NK
1217	3.59E-79	0.77029369	0.887	0.583	7.82E-75	CD247	5	PTGDS_NK
1218	1.47E-46	0.76097293	0.652	0.388	3.22E-42	MAP3K8	5	PTGDS_NK
1219	3.34E-44	0.7588326	0.274	0.079	7.27E-40	KIR2DL3	5	PTGDS_NK
1220	1.22E-58	0.75537192	0.419	0.142	2.66E-54	TMIGD2	5	PTGDS_NK
1221	2.44E-51	0.75166413	0.274	0.071	5.32E-47	KIR3DL2	5	PTGDS_NK
1222	9.62E-45	0.7491397	0.464	0.206	2.10E-40	IFITM3	5	PTGDS_NK
1223	2.54E-48	0.74666474	0.98	0.87	5.55E-44	CCL4	5	PTGDS_NK
1224	1.10E-81	0.74547267	0.333	0.069	2.40E-77	AKR1C3	5	PTGDS_NK
1225	3.83E-92	0.74450469	0.937	0.43	8.35E-88	TYROBP	5	PTGDS_NK
1226	1.15E-40	0.73870224	0.864	0.753	2.52E-36	GADD45B	5	PTGDS_NK
1227	2.64E-68	0.73042521	0.796	0.348	5.76E-64	GNLY	5	PTGDS_NK
1228	6.27E-68	0.72139851	0.335	0.082	1.37E-63	HAVCR2	5	PTGDS_NK
1229	8.10E-55	0.69049482	0.357	0.108	1.77E-50	FCRL3	5	PTGDS_NK
1230	3.92E-52	0.66669576	0.41	0.139	8.54E-48	IGFBP7	5	PTGDS_NK
1231	1.23E-67	0.66662594	0.914	0.648	2.69E-63	CTSW	5	PTGDS_NK
1232	2.25E-74	0.66326475	0.937	0.727	4.91E-70	CD7	5	PTGDS_NK
1233	5.41E-38	0.65711263	0.339	0.127	1.18E-33	CX3CR1	5	PTGDS_NK
1234	2.65E-16	0.63983261	0.446	0.299	5.78E-12	IER3	5	PTGDS_NK
1235	4.04E-46	0.63104162	0.498	0.22	8.81E-42	TXK	5	PTGDS_NK
1236	7.51E-31	0.61192337	0.561	0.342	1.64E-26	CD55	5	PTGDS_NK
1237	1.10E-35	0.60494748	0.471	0.232	2.41E-31	S1PR5	5	PTGDS_NK
1238	5.79E-60	0.60343103	0.984	0.858	1.26E-55	CST7	5	PTGDS_NK
1239	3.39E-33	0.58967298	0.534	0.308	7.38E-29	SELPLG	5	PTGDS_NK
1240	1.62E-28	0.58767977	0.43	0.228	3.54E-24	ICAM2	5	PTGDS_NK
1241	1.48E-26	0.58460123	0.258	0.097	3.22E-22	KIR2DL1	5	PTGDS_NK
1242	7.94E-35	0.5835779	0.312	0.118	1.73E-30	GSAP	5	PTGDS_NK
1243	3.64E-38	0.58053421	0.296	0.1	7.94E-34	CD38	5	PTGDS_NK
1244	7.67E-26	0.56246901	0.527	0.321	1.67E-21	BHLHE40	5	PTGDS_NK
1245	4.92E-26	0.56063249	0.319	0.145	1.07E-21	MCTP2	5	PTGDS_NK
1246	8.77E-37	0.55186119	0.5	0.231	1.91E-32	ADGRG1	5	PTGDS_NK
1247	2.04E-37	0.54360085	0.247	0.073	4.46E-33	ITGAM	5	PTGDS_NK
1248	3.96E-42	0.54110466	0.833	0.653	8.64E-38	ITGB2	5	PTGDS_NK
1249	2.03E-27	0.53779653	0.428	0.225	4.42E-23	EFHD2	5	PTGDS_NK
1250	1.27E-37	0.53719438	0.287	0.096	2.76E-33	PTPN12	5	PTGDS_NK
1251	3.00E-63	0.5306072	0.197	0.031	6.54E-59	GSN	5	PTGDS_NK
1252	1.11E-36	0.53051182	0.147	0.029	2.43E-32	KIR2DL4	5	PTGDS_NK
1253	2.93E-29	0.53014795	0.584	0.348	6.39E-25	ZEB2	5	PTGDS_NK
1254	2.97E-29	0.51936981	0.6	0.378	6.47E-25	PLEK	5	PTGDS_NK
1255	2.72E-38	0.51333687	0.253	0.075	5.93E-34	NCR1	5	PTGDS_NK
1256	2.05E-27	0.51203946	0.794	0.67	4.48E-23	NEAT1	5	PTGDS_NK
1257	1.24E-28	0.50772668	0.344	0.152	2.71E-24	HSH2D	5	PTGDS_NK
1258	5.79E-25	0.50622987	0.312	0.137	1.26E-20	C1orf21	5	PTGDS_NK

1259	2.52E-17	0.50005638	0.364	0.21	5.49E-13	AOAH	5	PTGDS_NK
1260	1.73E-23	0.49944714	0.731	0.604	3.77E-19	SQSTM1	5	PTGDS_NK
1261	8.66E-25	0.4927355	0.633	0.45	1.89E-20	CTSD	5	PTGDS_NK
1262	7.88E-31	0.49003466	0.188	0.053	1.72E-26	RASSF4	5	PTGDS_NK
1263	4.03E-23	0.48977984	0.419	0.232	8.80E-19	PXN	5	PTGDS_NK
1264	1.02E-24	0.48916761	0.636	0.457	2.22E-20	LIMD2	5	PTGDS_NK
1265	8.13E-56	0.48909536	0.993	0.972	1.77E-51	SRGN	5	PTGDS_NK
1266	7.59E-33	0.48340097	0.208	0.06	1.66E-28	TBXAS1	5	PTGDS_NK
1267	1.44E-53	0.48107127	0.975	0.879	3.14E-49	IFITM2	5	PTGDS_NK
1268	6.34E-45	0.47931199	0.172	0.033	1.38E-40	CHST2	5	PTGDS_NK
1269	7.74E-62	0.47596764	0.995	0.944	1.69E-57	IFITM1	5	PTGDS_NK
1270	1.20E-19	0.4754403	0.317	0.158	2.61E-15	FGR	5	PTGDS_NK
1271	2.39E-31	0.4734715	0.9	0.776	5.20E-27	NFKBIA	5	PTGDS_NK
1272	5.53E-89	0.47187121	0.122	0.005	1.21E-84	RAMP1	5	PTGDS_NK
1273	1.10E-19	0.46934688	0.622	0.492	2.39E-15	LY6E	5	PTGDS_NK
1274	1.21E-25	0.46933929	0.301	0.127	2.64E-21	PRSS23	5	PTGDS_NK
1275	1.60E-22	0.46920163	0.353	0.175	3.48E-18	TTC38	5	PTGDS_NK
1276	6.35E-34	0.46756318	0.821	0.652	1.39E-29	RAP1B	5	PTGDS_NK
1277	5.11E-21	0.46644183	0.726	0.614	1.11E-16	GNG2	5	PTGDS_NK
1278	9.16E-20	0.46581171	0.269	0.126	2.00E-15	CEP78	5	PTGDS_NK
1279	3.44E-13	0.46451093	0.344	0.217	7.50E-09	GBP2	5	PTGDS_NK
1280	5.67E-15	0.46407794	0.624	0.518	1.24E-10	SYTL3	5	PTGDS_NK
1281	8.40E-17	0.46027981	0.33	0.185	1.83E-12	YES1	5	PTGDS_NK
1282	4.54E-16	0.45933448	0.251	0.125	9.89E-12	MAFF	5	PTGDS_NK
1283	2.56E-23	0.45354526	0.258	0.106	5.59E-19	UBE2F	5	PTGDS_NK
1284	3.55E-31	0.45038728	0.794	0.605	7.73E-27	LITAF	5	PTGDS_NK
1285	5.50E-20	0.44923557	0.6	0.436	1.20E-15	APMAP	5	PTGDS_NK
1286	3.88E-21	0.44332798	0.215	0.084	8.46E-17	FNDC3B	5	PTGDS_NK
1287	1.58E-43	0.44289744	0.14	0.022	3.45E-39	ADAMTS1	5	PTGDS_NK
1288	3.11E-14	0.44164387	0.247	0.13	6.77E-10	IL18RAP	5	PTGDS_NK
1289	1.07E-20	0.43875406	0.69	0.571	2.33E-16	JAK1	5	PTGDS_NK
1290	2.05E-16	0.43633826	0.518	0.378	4.46E-12	SERPINB1	5	PTGDS_NK
1291	1.20E-17	0.43398608	0.385	0.224	2.61E-13	GNPTAB	5	PTGDS_NK
1292	3.17E-23	0.43158571	0.686	0.522	6.91E-19	GZMM	5	PTGDS_NK
1293	2.26E-17	0.42981523	0.412	0.253	4.93E-13	MAPK1	5	PTGDS_NK
1294	6.96E-14	0.42766083	0.498	0.37	1.52E-09	TCF25	5	PTGDS_NK
1295	3.27E-29	0.42219575	0.869	0.792	7.12E-25	CYBA	5	PTGDS_NK
1296	1.76E-07	0.42039416	0.326	0.237	0.003845166	PMAIP1	5	PTGDS_NK
1297	1.25E-17	0.42034953	0.249	0.117	2.72E-13	GFOD1	5	PTGDS_NK
1298	1.55E-07	0.41931123	0.324	0.243	0.003390851	HIF1A	5	PTGDS_NK
1299	8.35E-17	0.41796402	0.26	0.128	1.82E-12	ZBTB16	5	PTGDS_NK
1300	2.93E-16	0.41793035	0.206	0.09	6.39E-12	IRF8	5	PTGDS_NK
1301	2.62E-14	0.41713682	0.357	0.221	5.72E-10	DIP2A	5	PTGDS_NK
1302	8.73E-22	0.41501796	0.188	0.065	1.90E-17	RP11-81H14.2	5	PTGDS_NK
1303	3.70E-13	0.41359614	0.308	0.183	8.08E-09	SH3BP5	5	PTGDS_NK
1304	1.29E-15	0.41323664	0.584	0.454	2.82E-11	CD47	5	PTGDS_NK
1305	1.57E-15	0.41257678	0.584	0.458	3.41E-11	BIN2	5	PTGDS_NK
1306	5.82E-45	0.4082978	0.12	0.016	1.27E-40	SIGLEC7	5	PTGDS_NK
1307	3.61E-14	0.40800108	0.441	0.302	7.88E-10	SAMHD1	5	PTGDS_NK
1308	4.25E-18	0.40483977	0.294	0.147	9.28E-14	LYN	5	PTGDS_NK
1309	1.22E-12	0.40437606	0.308	0.185	2.65E-08	PTPRE	5	PTGDS_NK
1310	1.46E-19	0.40341183	0.206	0.082	3.18E-15	SLC15A4	5	PTGDS_NK
1311	6.79E-16	0.40084477	0.482	0.326	1.48E-11	IL2RB	5	PTGDS_NK
1312	2.28E-16	0.3981822	0.251	0.123	4.97E-12	RIN3	5	PTGDS_NK
1313	2.18E-22	0.39436302	0.213	0.079	4.75E-18	OSBPL5	5	PTGDS_NK
1314	5.95E-12	0.39396015	0.477	0.359	1.30E-07	SLA	5	PTGDS_NK
1315	1.02E-16	0.39278951	0.283	0.144	2.22E-12	CD300A	5	PTGDS_NK
1316	1.15E-15	0.3914187	0.529	0.381	2.50E-11	MBP	5	PTGDS_NK
1317	2.27E-12	0.39102837	0.452	0.322	4.95E-08	MATK	5	PTGDS_NK
1318	3.61E-33	0.3906304	0.127	0.024	7.86E-29	GRASP	5	PTGDS_NK
1319	2.55E-11	0.38910639	0.391	0.271	5.56E-07	CHST12	5	PTGDS_NK
1320	5.41E-17	0.388446	0.204	0.086	1.18E-12	GK5	5	PTGDS_NK
1321	1.91E-16	0.38235465	0.133	0.045	4.17E-12	GSTM2	5	PTGDS_NK
1322	5.50E-19	0.38224825	0.502	0.295	1.20E-14	XCL2	5	PTGDS_NK
1323	5.34E-14	0.38162558	0.425	0.282	1.16E-09	TPST2	5	PTGDS_NK
1324	3.33E-21	0.38160782	0.923	0.815	7.26E-17	DDIT4	5	PTGDS_NK
1325	3.22E-27	0.38014267	0.154	0.04	7.01E-23	MLC1	5	PTGDS_NK
1326	3.20E-17	0.37848602	0.17	0.065	6.98E-13	THEMIS2	5	PTGDS_NK
1327	1.24E-14	0.37516248	0.29	0.158	2.71E-10	GLIPR2	5	PTGDS_NK
1328	7.17E-21	0.37471117	0.808	0.725	1.56E-16	IL2RG	5	PTGDS_NK

1329	1.56E-09	0.37063781	0.514	0.391	3.41E-05	CREM	5	PTGDS_NK
1330	2.49E-14	0.36915276	0.867	0.82	5.42E-10	TXNIP	5	PTGDS_NK
1331	9.01E-15	0.36823214	0.231	0.111	1.97E-10	MTSS1	5	PTGDS_NK
1332	5.81E-12	0.3672348	0.382	0.259	1.27E-07	SSH2	5	PTGDS_NK
1333	3.89E-58	0.36393758	0.118	0.011	8.49E-54	FES	5	PTGDS_NK
1334	3.69E-13	0.3638066	0.548	0.421	8.04E-09	AES	5	PTGDS_NK
1335	6.06E-12	0.36357717	0.321	0.196	1.32E-07	RASA3	5	PTGDS_NK
1336	1.04E-11	0.36082525	0.19	0.096	2.27E-07	HIPK2	5	PTGDS_NK
1337	8.64E-11	0.36038492	0.527	0.412	1.88E-06	PYHIN1	5	PTGDS_NK
1338	4.53E-09	0.35909976	0.269	0.173	9.87E-05	TMBIM1	5	PTGDS_NK
1339	3.42E-14	0.35789169	0.581	0.456	7.47E-10	CD164	5	PTGDS_NK
1340	2.55E-10	0.35660982	0.425	0.321	5.56E-06	CDC42SE1	5	PTGDS_NK
1341	2.99E-12	0.35529537	0.326	0.202	6.52E-08	ADAM8	5	PTGDS_NK
1342	6.90E-09	0.35458346	0.371	0.277	0.000150457	PTPN2	5	PTGDS_NK
1343	4.37E-07	0.35106867	0.26	0.177	0.009527824	CDKN2D	5	PTGDS_NK
1344	1.35E-12	0.34935232	0.631	0.51	2.95E-08	HOPX	5	PTGDS_NK
1345	2.23E-11	0.34926885	0.48	0.362	4.85E-07	PSAP	5	PTGDS_NK
1346	3.24E-07	0.34902128	0.362	0.281	0.007076631	SDF4	5	PTGDS_NK
1347	4.88E-17	0.34834414	0.118	0.036	1.06E-12	CXCR2	5	PTGDS_NK
1348	2.72E-20	0.34813303	0.955	0.914	5.92E-16	IER2	5	PTGDS_NK
1349	2.35E-07	0.34781941	0.414	0.336	0.005118525	PSMB10	5	PTGDS_NK
1350	1.58E-12	0.34727257	0.459	0.316	3.45E-08	KLF2	5	PTGDS_NK
1351	4.77E-13	0.34484216	0.455	0.318	1.04E-08	CARD16	5	PTGDS_NK
1352	2.49E-10	0.34181411	0.188	0.099	5.44E-06	B4GALT4	5	PTGDS_NK
1353	3.98E-14	0.33961751	0.133	0.049	8.67E-10	GPR141	5	PTGDS_NK
1354	1.39E-10	0.33866708	0.179	0.091	3.04E-06	SERPINB6	5	PTGDS_NK
1355	2.78E-10	0.33793885	0.251	0.147	6.06E-06	KLF3	5	PTGDS_NK
1356	3.23E-27	0.3376788	0.133	0.031	7.04E-23	IRS2	5	PTGDS_NK
1357	5.62E-12	0.33684321	0.267	0.151	1.22E-07	CEBPD	5	PTGDS_NK
1358	3.07E-09	0.33557955	0.432	0.327	6.70E-05	RHOC	5	PTGDS_NK
1359	1.01E-09	0.33480052	0.269	0.166	2.20E-05	SLA2	5	PTGDS_NK
1360	2.35E-08	0.33280037	0.572	0.491	0.000513358	IRF1	5	PTGDS_NK
1361	8.90E-11	0.33213807	0.579	0.486	1.94E-06	MSN	5	PTGDS_NK
1362	3.80E-19	0.32858706	0.109	0.029	8.29E-15	LINC00299	5	PTGDS_NK
1363	6.92E-08	0.32765258	0.545	0.457	0.001509745	PRDM1	5	PTGDS_NK
1364	9.26E-11	0.32722666	0.226	0.126	2.02E-06	TFDP2	5	PTGDS_NK
1365	4.98E-10	0.32400062	0.638	0.581	1.09E-05	EIF3G	5	PTGDS_NK
1366	4.86E-10	0.32345761	0.167	0.083	1.06E-05	CLASP1	5	PTGDS_NK
1367	2.34E-14	0.32344999	0.133	0.05	5.10E-10	CXXC5	5	PTGDS_NK
1368	1.21E-10	0.32275117	0.19	0.098	2.64E-06	PLEKHA1	5	PTGDS_NK
1369	6.43E-14	0.32206574	0.661	0.5	1.40E-09	TRBC1	5	PTGDS_NK
1370	1.50E-18	0.31978798	0.783	0.661	3.27E-14	CCND3	5	PTGDS_NK
1371	5.67E-09	0.31880918	0.29	0.191	0.000123659	TBX21	5	PTGDS_NK
1372	6.86E-15	0.31692497	0.238	0.114	1.50E-10	LAT2	5	PTGDS_NK
1373	5.02E-07	0.31456973	0.235	0.155	0.010943021	ADD1	5	PTGDS_NK
1374	9.02E-07	0.31209475	0.188	0.116	0.019667961	DHRS3	5	PTGDS_NK
1375	1.35E-06	0.31191826	0.238	0.158	0.029345345	ICAM1	5	PTGDS_NK
1376	1.75E-06	0.31189419	0.346	0.263	0.038261819	IQGAP2	5	PTGDS_NK
1377	2.78E-08	0.3102605	0.455	0.354	0.000607001	FLNA	5	PTGDS_NK
1378	2.29E-11	0.30984936	0.158	0.073	5.00E-07	FHL3	5	PTGDS_NK
1379	2.66E-08	0.3092562	0.407	0.308	0.00057913	PPP3CC	5	PTGDS_NK
1380	6.06E-10	0.30516111	0.624	0.537	1.32E-05	PDIA3	5	PTGDS_NK
1381	3.06E-08	0.30489669	0.181	0.103	0.000666986	SCLT1	5	PTGDS_NK
1382	5.30E-13	0.30320335	0.649	0.531	1.16E-08	GSTP1	5	PTGDS_NK
1383	1.30E-06	0.30248226	0.498	0.419	0.028320933	MAPRE2	5	PTGDS_NK
1384	2.54E-07	0.30174562	0.568	0.512	0.005548635	SRSF2	5	PTGDS_NK
1385	5.39E-35	0.30095599	0.998	0.989	1.17E-30	HLA-C	5	PTGDS_NK
1386	2.73E-16	0.29966337	0.751	0.653	5.94E-12	CD53	5	PTGDS_NK
1387	2.74E-08	0.29923093	0.301	0.202	0.000597855	GPATCH8	5	PTGDS_NK
1388	2.48E-11	0.29917824	0.115	0.045	5.40E-07	MIR181A1HG	5	PTGDS_NK
1389	1.41E-09	0.29897015	0.292	0.185	3.08E-05	STK38	5	PTGDS_NK
1390	7.41E-07	0.29696597	0.568	0.498	0.016155032	PTGER4	5	PTGDS_NK
1391	3.86E-07	0.29680393	0.477	0.405	0.00841463	DENND2D	5	PTGDS_NK
1392	6.98E-08	0.29601233	0.344	0.245	0.001521315	SYNE1	5	PTGDS_NK
1393	1.16E-13	0.29588019	0.871	0.815	2.52E-09	UBB	5	PTGDS_NK
1394	1.36E-12	0.29494529	0.138	0.056	2.97E-08	CTBP2	5	PTGDS_NK
1395	2.18E-09	0.29353271	0.19	0.103	4.74E-05	TLE1	5	PTGDS_NK
1396	8.62E-07	0.29323833	0.367	0.284	0.01879227	PRKAR1A	5	PTGDS_NK
1397	3.80E-07	0.29321433	0.414	0.327	0.008284795	ADD3	5	PTGDS_NK
1398	1.01E-18	0.29124812	0.111	0.03	2.21E-14	AK5	5	PTGDS_NK

1399	4.02E-09	0.29096803	0.654	0.589	8.76E-05	ISG20	5	PTGDS_NK
1400	6.89E-07	0.28940535	0.434	0.348	0.015031606	ATM	5	PTGDS_NK
1401	2.46E-09	0.28923022	0.158	0.08	5.37E-05	UNC119	5	PTGDS_NK
1402	1.35E-21	0.28881662	0.894	0.805	2.94E-17	ARPC2	5	PTGDS_NK
1403	5.45E-07	0.28837968	0.176	0.105	0.011885579	IGF2R	5	PTGDS_NK
1404	3.14E-07	0.28566754	0.299	0.206	0.006849543	PTGDR	5	PTGDS_NK
1405	1.80E-09	0.28542582	0.486	0.37	3.93E-05	UCP2	5	PTGDS_NK
1406	7.34E-07	0.28472388	0.215	0.137	0.016004607	MVD	5	PTGDS_NK
1407	6.70E-07	0.27975602	0.511	0.443	0.014620647	ARHGEF1	5	PTGDS_NK
1408	1.08E-07	0.27818425	0.133	0.068	0.002360146	SELL	5	PTGDS_NK
1409	5.23E-12	0.27703094	0.708	0.596	1.14E-07	EMP3	5	PTGDS_NK
1410	5.16E-07	0.27510942	0.269	0.185	0.0112553	SORL1	5	PTGDS_NK
1411	3.77E-08	0.27482073	0.566	0.494	0.000821926	FAM49B	5	PTGDS_NK
1412	1.04E-11	0.27272948	0.118	0.046	2.27E-07	TMED1	5	PTGDS_NK
1413	3.75E-07	0.27087838	0.509	0.434	0.008176194	DHRS7	5	PTGDS_NK
1414	1.51E-30	0.27081956	0.93	0.583	3.30E-26	KLRB1	5	PTGDS_NK
1415	2.79E-11	0.27075447	0.844	0.778	6.09E-07	PNRC1	5	PTGDS_NK
1416	2.61E-07	0.27040337	0.149	0.082	0.005701716	APLP2	5	PTGDS_NK
1417	5.28E-08	0.270025	0.271	0.177	0.001150989	G6PD	5	PTGDS_NK
1418	1.71E-06	0.26804137	0.774	0.735	0.037308186	ZFP36L1	5	PTGDS_NK
1419	4.51E-11	0.26691124	0.152	0.068	9.85E-07	PDGFD	5	PTGDS_NK
1420	8.39E-12	0.26680855	0.767	0.68	1.83E-07	CIRBP	5	PTGDS_NK
1421	6.69E-07	0.26485562	0.167	0.098	0.014584345	CD226	5	PTGDS_NK
1422	1.63E-06	0.26484036	0.319	0.236	0.035577589	SLC44A2	5	PTGDS_NK
1423	7.53E-07	0.2641982	0.466	0.378	0.016417089	FCMR	5	PTGDS_NK
1424	6.43E-29	0.26402371	0.998	0.997	1.40E-24	HLA-B	5	PTGDS_NK
1425	2.25E-07	0.26166634	0.71	0.639	0.004908656	PPP1R15A	5	PTGDS_NK
1426	1.51E-06	0.26160492	0.127	0.068	0.032931023	BPGM	5	PTGDS_NK
1427	4.60E-07	0.26036932	0.17	0.098	0.010037752	S1PR1	5	PTGDS_NK
1428	5.53E-08	0.25787128	0.147	0.076	0.001205048	PAM	5	PTGDS_NK
1429	7.70E-07	0.25766026	0.348	0.259	0.016787287	TNFRSF14	5	PTGDS_NK
1430	5.30E-41	0.25708959	1	1	1.16E-36	B2M	5	PTGDS_NK
1431	8.37E-15	0.25533137	0.982	0.968	1.83E-10	FTL	5	PTGDS_NK
1432	1.03E-07	0.25497694	0.149	0.08	0.002239495	AGK	5	PTGDS_NK
1433	2.72E-08	0.25076881	0.629	0.535	0.00059406	GPR65	5	PTGDS_NK
1434	4.04E-07	-0.25093441	0.154	0.269	0.008807357	CASP1	5	PTGDS_NK
1435	8.62E-11	-0.25151824	0.76	0.9	1.88E-06	CXCR4	5	PTGDS_NK
1436	5.45E-11	-0.25186791	0.661	0.796	1.19E-06	RPL27A	5	PTGDS_NK
1437	4.37E-15	-0.2529209	0.853	0.933	9.52E-11	ATP5E	5	PTGDS_NK
1438	3.79E-08	-0.2548726	0.527	0.654	0.000826439	C12orf57	5	PTGDS_NK
1439	1.72E-08	-0.25489817	0.192	0.334	0.000374327	ZBTB38	5	PTGDS_NK
1440	7.90E-08	-0.25520101	0.369	0.514	0.001722228	EIF3H	5	PTGDS_NK
1441	3.05E-07	-0.25520591	0.441	0.576	0.006642388	FYB	5	PTGDS_NK
1442	8.36E-26	-0.25752239	0.982	0.99	1.82E-21	RPL18	5	PTGDS_NK
1443	3.70E-07	-0.25856398	0.206	0.328	0.008074494	ELOVL5	5	PTGDS_NK
1444	1.51E-24	-0.25876172	0.95	0.981	3.30E-20	RPL23A	5	PTGDS_NK
1445	2.36E-22	-0.2630231	0.959	0.979	5.16E-18	RPL17	5	PTGDS_NK
1446	2.45E-17	-0.26352693	0.871	0.933	5.34E-13	RPS9	5	PTGDS_NK
1447	5.00E-16	-0.26558647	0.928	0.964	1.09E-11	RPS29	5	PTGDS_NK
1448	3.56E-20	-0.26644087	0.916	0.956	7.77E-16	RPL35	5	PTGDS_NK
1449	4.71E-08	-0.26902622	0.326	0.472	0.001027619	ERP29	5	PTGDS_NK
1450	3.95E-35	-0.2716524	0.986	0.994	8.62E-31	RPL26	5	PTGDS_NK
1451	7.47E-24	-0.27213108	0.973	0.988	1.63E-19	RPL12	5	PTGDS_NK
1452	1.22E-18	-0.27231686	0.925	0.952	2.66E-14	RPS5	5	PTGDS_NK
1453	1.35E-06	-0.27464557	0.17	0.278	0.02952816	NUCB2	5	PTGDS_NK
1454	1.15E-07	-0.27609262	0.38	0.528	0.002501232	PARP8	5	PTGDS_NK
1455	7.95E-21	-0.27648813	0.93	0.961	1.73E-16	RPL10A	5	PTGDS_NK
1456	2.23E-08	-0.2781582	0.554	0.698	0.000485813	SRSF7	5	PTGDS_NK
1457	3.39E-30	-0.28044357	0.982	0.986	7.39E-26	RPS10	5	PTGDS_NK
1458	1.27E-07	-0.28148923	0.862	0.889	0.002770538	DUSP1	5	PTGDS_NK
1459	2.36E-09	-0.28265075	0.229	0.387	5.15E-05	PRDX1	5	PTGDS_NK
1460	2.97E-10	-0.2843019	0.303	0.474	6.49E-06	MYH9	5	PTGDS_NK
1461	1.97E-07	-0.28619278	0.219	0.353	0.004290729	ITM2A	5	PTGDS_NK
1462	2.75E-33	-0.28767695	0.982	0.993	6.00E-29	RPS23	5	PTGDS_NK
1463	1.22E-10	-0.28847485	0.249	0.424	2.65E-06	PKM	5	PTGDS_NK
1464	3.04E-16	-0.28859157	0.783	0.915	6.63E-12	MT-ND2	5	PTGDS_NK
1465	2.76E-07	-0.28915332	0.45	0.571	0.006009901	HERPUD1	5	PTGDS_NK
1466	2.72E-09	-0.29233851	0.208	0.358	5.94E-05	AKIRIN2	5	PTGDS_NK
1467	4.56E-09	-0.29255044	0.29	0.44	9.94E-05	CKLF	5	PTGDS_NK
1468	1.14E-35	-0.29263095	0.991	0.991	2.50E-31	RPS7	5	PTGDS_NK

1469	1.14E-06	-0.29443287	0.167	0.279	0.024792463	AIM1	5	PTGDS_NK
1470	1.85E-09	-0.29746118	0.138	0.276	4.03E-05	MZT2A	5	PTGDS_NK
1471	9.91E-40	-0.30119484	0.991	0.995	2.16E-35	RPL19	5	PTGDS_NK
1472	8.37E-09	-0.3013194	0.308	0.462	0.00018254	CD96	5	PTGDS_NK
1473	5.26E-39	-0.30150077	0.984	0.995	1.15E-34	RPS15	5	PTGDS_NK
1474	5.53E-34	-0.30243154	0.989	0.991	1.21E-29	RPL18A	5	PTGDS_NK
1475	1.99E-29	-0.30333043	0.955	0.986	4.35E-25	RPS6	5	PTGDS_NK
1476	2.56E-09	-0.30475125	0.149	0.289	5.58E-05	ATP1A1	5	PTGDS_NK
1477	2.25E-48	-0.30495205	1	0.998	4.92E-44	RPS15A	5	PTGDS_NK
1478	4.21E-07	-0.30542515	0.122	0.229	0.009182809	BCL2A1	5	PTGDS_NK
1479	1.08E-37	-0.30562874	0.995	0.995	2.36E-33	RPS28	5	PTGDS_NK
1480	1.03E-08	-0.30794546	0.606	0.725	0.000224124	CALM2	5	PTGDS_NK
1481	1.34E-10	-0.30820444	0.147	0.302	2.93E-06	SNHG12	5	PTGDS_NK
1482	2.27E-11	-0.3083189	0.52	0.674	4.95E-07	CRIP1	5	PTGDS_NK
1483	3.20E-09	-0.31080558	0.188	0.331	6.97E-05	TXN	5	PTGDS_NK
1484	1.35E-22	-0.31154705	0.921	0.958	2.95E-18	RPS2	5	PTGDS_NK
1485	1.15E-10	-0.31201991	0.17	0.327	2.50E-06	TNFAIP8	5	PTGDS_NK
1486	8.59E-10	-0.31511789	0.233	0.384	1.87E-05	LAT	5	PTGDS_NK
1487	8.97E-38	-0.31853988	0.982	0.988	1.96E-33	RPS4X	5	PTGDS_NK
1488	1.11E-34	-0.31919568	0.991	0.989	2.41E-30	RPS13	5	PTGDS_NK
1489	1.12E-18	-0.31972331	0.792	0.916	2.44E-14	CCL5	5	PTGDS_NK
1490	2.45E-12	-0.3231493	0.572	0.714	5.35E-08	S100A10	5	PTGDS_NK
1491	2.74E-18	-0.32400188	0.769	0.876	5.99E-14	EEF1B2	5	PTGDS_NK
1492	1.49E-10	-0.32493329	0.215	0.378	3.24E-06	SPOCK2	5	PTGDS_NK
1493	1.42E-10	-0.32714803	0.102	0.237	3.10E-06	OCIAD2	5	PTGDS_NK
1494	3.72E-08	-0.32865785	0.17	0.297	0.000811757	FAM46C	5	PTGDS_NK
1495	1.60E-09	-0.33106567	0.274	0.426	3.49E-05	SNHG8	5	PTGDS_NK
1496	2.27E-33	-0.33119635	0.943	0.974	4.95E-29	RPS16	5	PTGDS_NK
1497	1.11E-41	-0.33262143	0.998	0.997	2.41E-37	RPL34	5	PTGDS_NK
1498	1.78E-43	-0.33393365	0.995	0.997	3.87E-39	RPL13	5	PTGDS_NK
1499	4.22E-41	-0.33483933	0.977	0.99	9.20E-37	RPL35A	5	PTGDS_NK
1500	4.67E-41	-0.33706688	0.998	0.997	1.02E-36	RPS27	5	PTGDS_NK
1501	5.78E-11	-0.33800808	0.335	0.511	1.26E-06	TERF2IP	5	PTGDS_NK
1502	5.48E-45	-0.33844842	1	0.999	1.20E-40	RPLP1	5	PTGDS_NK
1503	1.21E-42	-0.34055389	0.977	0.993	2.64E-38	RPLP2	5	PTGDS_NK
1504	8.65E-24	-0.34099143	0.887	0.953	1.89E-19	MT-ND1	5	PTGDS_NK
1505	1.14E-31	-0.34173392	0.928	0.975	2.49E-27	RPL8	5	PTGDS_NK
1506	7.25E-48	-0.34447831	0.989	0.996	1.58E-43	RPL11	5	PTGDS_NK
1507	2.32E-09	-0.34677918	0.1	0.224	5.05E-05	TOB1	5	PTGDS_NK
1508	9.70E-11	-0.34709897	0.167	0.324	2.12E-06	G3BP2	5	PTGDS_NK
1509	1.50E-55	-0.34965148	0.989	0.995	3.26E-51	RPS3	5	PTGDS_NK
1510	6.03E-08	-0.3538165	0.186	0.322	0.001314162	HSPB1	5	PTGDS_NK
1511	2.09E-33	-0.35510082	0.946	0.979	4.56E-29	RPL36	5	PTGDS_NK
1512	3.54E-07	-0.35893488	0.271	0.399	0.007712285	HBB	5	PTGDS_NK
1513	1.12E-07	-0.36029522	0.405	0.542	0.002440113	FOSB	5	PTGDS_NK
1514	2.44E-48	-0.36171746	0.986	0.993	5.31E-44	RPS14	5	PTGDS_NK
1515	5.15E-58	-0.37276685	0.991	0.996	1.12E-53	RPL30	5	PTGDS_NK
1516	7.93E-18	-0.37980671	0.568	0.75	1.73E-13	RPS20	5	PTGDS_NK
1517	1.90E-12	-0.38116364	0.269	0.448	4.14E-08	PDCD4	5	PTGDS_NK
1518	9.88E-20	-0.38321796	0.624	0.802	2.15E-15	RPL36A	5	PTGDS_NK
1519	3.81E-55	-0.38556904	0.991	0.996	8.30E-51	RPS8	5	PTGDS_NK
1520	1.18E-11	-0.38594315	0.19	0.354	2.57E-07	HLA-DQB1	5	PTGDS_NK
1521	4.15E-43	-0.38649553	0.977	0.99	9.05E-39	RPS18	5	PTGDS_NK
1522	1.49E-59	-0.39383798	1	0.997	3.25E-55	RPL39	5	PTGDS_NK
1523	4.32E-44	-0.39421775	0.971	0.987	9.43E-40	RPS25	5	PTGDS_NK
1524	2.85E-61	-0.39882471	0.989	0.995	6.21E-57	RPL32	5	PTGDS_NK
1525	2.29E-11	-0.40007917	0.197	0.359	4.99E-07	SAMSN1	5	PTGDS_NK
1526	1.01E-11	-0.40596579	0.294	0.459	2.21E-07	HLA-DPB1	5	PTGDS_NK
1527	3.75E-14	-0.40983862	0.265	0.458	8.18E-10	HLA-DPA1	5	PTGDS_NK
1528	7.35E-15	-0.41191654	0.324	0.521	1.60E-10	LDHB	5	PTGDS_NK
1529	3.82E-15	-0.41434066	0.17	0.366	8.33E-11	EML4	5	PTGDS_NK
1530	1.74E-51	-0.4222376	0.968	0.988	3.80E-47	RPS21	5	PTGDS_NK
1531	1.42E-81	-0.43007871	0.995	0.999	3.09E-77	RPL28	5	PTGDS_NK
1532	1.40E-16	-0.43138915	0.224	0.441	3.05E-12	TUBA4A	5	PTGDS_NK
1533	3.99E-39	-0.44228319	0.916	0.968	8.70E-35	RPLP0	5	PTGDS_NK
1534	2.11E-17	-0.4580862	0.12	0.317	4.61E-13	LIME1	5	PTGDS_NK
1535	1.03E-06	-0.47548528	0.154	0.261	0.02236473	HSPH1	5	PTGDS_NK
1536	1.55E-61	-0.47939235	0.993	0.998	3.38E-57	RPS19	5	PTGDS_NK
1537	1.12E-11	-0.4801905	0.183	0.34	2.45E-07	KLRG1	5	PTGDS_NK
1538	3.62E-08	-0.48926626	0.369	0.51	0.00079023	HSPE1	5	PTGDS_NK

1539	3.21E-15	-0.49941416	0.238	0.428	7.00E-11	EZR	5	PTGDS_NK
1540	2.94E-84	-0.50341521	0.998	0.997	6.41E-80	RPS12	5	PTGDS_NK
1541	1.52E-20	-0.51186171	0.197	0.44	3.31E-16	CD44	5	PTGDS_NK
1542	1.83E-33	-0.51278409	0.552	0.793	3.99E-29	PPDPF	5	PTGDS_NK
1543	3.79E-23	-0.54190329	0.208	0.466	8.27E-19	CNN2	5	PTGDS_NK
1544	1.23E-08	-0.55664811	0.249	0.37	0.000268576	RGS1	5	PTGDS_NK
1545	7.75E-34	-0.56056032	0.618	0.808	1.69E-29	S100A6	5	PTGDS_NK
1546	5.84E-08	-0.5773232	0.258	0.393	0.00127381	HSPD1	5	PTGDS_NK
1547	2.16E-24	-0.58036685	0.19	0.442	4.72E-20	TC2N	5	PTGDS_NK
1548	8.43E-23	-0.61230005	0.326	0.55	1.84E-18	HLA-DRB1	5	PTGDS_NK
1549	2.73E-11	-0.7524373	0.206	0.352	5.94E-07	HMGB2	5	PTGDS_NK
1550	2.26E-25	-0.87575775	0.188	0.443	4.93E-21	TNF	5	PTGDS_NK
1551	4.65E-63	-0.91737755	0.554	0.845	1.01E-58	VIM	5	PTGDS_NK
1552	8.95E-100	-1.02086773	0.385	0.908	1.95E-95	IL32	5	PTGDS_NK
1553	2.96E-74	-1.06041504	0.199	0.681	6.47E-70	CD2	5	PTGDS_NK
1554	3.00E-45	-1.21053044	0.109	0.449	6.55E-41	CD8A	5	PTGDS_NK
1555	1.28E-132	-1.25866084	0.283	0.895	2.79E-128	CD3E	5	PTGDS_NK
1556	5.44E-70	-1.26364357	0.181	0.628	1.19E-65	CD52	5	PTGDS_NK
1557	5.66E-43	-1.66125926	0.113	0.435	1.23E-38	IL7R	5	PTGDS_NK
1558	3.46E-286	1.70622307	0.438	0.022	7.54E-282	TRBV9	6	TRBV9_CD8 Teff
1559	0	1.51100965	0.393	0.009	0	TRAV38-2DV8	6	TRBV9_CD8 Teff
1560	2.08E-88	1.14922293	0.15	0.009	4.53E-84	TRBV13	6	TRBV9_CD8 Teff
1561	1.05E-67	0.99066125	0.26	0.044	2.28E-63	RP11-291B21.2	6	TRBV9_CD8 Teff
1562	4.09E-109	0.88557073	0.327	0.043	8.91E-105	TRGV5	6	TRBV9_CD8 Teff
1563	3.62E-89	0.88539756	0.925	0.499	7.90E-85	GZMH	6	TRBV9_CD8 Teff
1564	7.41E-81	0.86552855	0.853	0.395	1.62E-76	CD8A	6	TRBV9_CD8 Teff
1565	1.87E-58	0.83067601	0.845	0.577	4.09E-54	CD52	6	TRBV9_CD8 Teff
1566	1.26E-51	0.71580702	0.687	0.349	2.74E-47	CD3G	6	TRBV9_CD8 Teff
1567	1.08E-24	0.69171013	0.399	0.191	2.36E-20	HLA-DRA	6	TRBV9_CD8 Teff
1568	3.43E-48	0.68419283	0.817	0.485	7.49E-44	CD3D	6	TRBV9_CD8 Teff
1569	2.96E-36	0.64538523	0.324	0.103	6.46E-32	TRGV2	6	TRBV9_CD8 Teff
1570	4.90E-41	0.63786112	0.512	0.207	1.07E-36	CD8B	6	TRBV9_CD8 Teff
1571	6.56E-27	0.57574096	0.404	0.181	1.43E-22	HLA-DQA1	6	TRBV9_CD8 Teff
1572	9.07E-51	0.57143309	0.875	0.412	1.98E-46	GZMB	6	TRBV9_CD8 Teff
1573	4.94E-25	0.54317456	0.726	0.52	1.08E-20	HLA-DRB1	6	TRBV9_CD8 Teff
1574	7.56E-41	0.54184482	0.687	0.308	1.65E-36	FGFBP2	6	TRBV9_CD8 Teff
1575	1.56E-25	0.48798635	0.895	0.791	3.39E-21	CD74	6	TRBV9_CD8 Teff
1576	7.75E-45	0.48783356	0.992	0.871	1.69E-40	NKG7	6	TRBV9_CD8 Teff
1577	9.84E-16	0.48161428	0.438	0.267	2.15E-11	ITGB1	6	TRBV9_CD8 Teff
1578	2.94E-16	0.47938644	0.188	0.071	6.40E-12	TRGV3	6	TRBV9_CD8 Teff
1579	4.98E-19	0.46183818	0.637	0.434	1.09E-14	HLA-DPB1	6	TRBV9_CD8 Teff
1580	5.28E-77	0.44957424	0.122	0.006	1.15E-72	ZNF683	6	TRBV9_CD8 Teff
1581	5.91E-22	0.44206609	0.651	0.429	1.29E-17	HLA-DPA1	6	TRBV9_CD8 Teff
1582	1.54E-15	0.43870603	0.435	0.257	3.36E-11	HLA-DRB5	6	TRBV9_CD8 Teff
1583	2.44E-29	0.41030173	0.961	0.904	5.33E-25	SH3BGRL3	6	TRBV9_CD8 Teff
1584	6.68E-18	0.40214708	0.44	0.239	1.46E-13	ADGRG1	6	TRBV9_CD8 Teff
1585	2.03E-15	0.38926327	0.28	0.131	4.43E-11	PRSS23	6	TRBV9_CD8 Teff
1586	1.23E-21	0.38181307	0.967	0.86	2.68E-17	S100A4	6	TRBV9_CD8 Teff
1587	1.63E-09	0.37125903	0.296	0.175	3.57E-05	THEMIS	6	TRBV9_CD8 Teff
1588	2.39E-10	0.36673627	0.499	0.353	5.22E-06	FLNA	6	TRBV9_CD8 Teff
1589	1.58E-10	0.34788452	0.263	0.143	3.44E-06	C1orf21	6	TRBV9_CD8 Teff
1590	1.34E-18	0.34142174	0.579	0.353	2.92E-14	LGALS1	6	TRBV9_CD8 Teff
1591	1.67E-13	0.33340674	0.554	0.354	3.64E-09	ZEB2	6	TRBV9_CD8 Teff
1592	8.34E-12	0.32895613	0.158	0.064	1.82E-07	LILRB1	6	TRBV9_CD8 Teff
1593	8.79E-13	0.32587519	0.213	0.096	1.92E-08	S1PR1	6	TRBV9_CD8 Teff
1594	2.05E-16	0.32351731	0.186	0.067	4.47E-12	RP11-81H14.2	6	TRBV9_CD8 Teff
1595	8.81E-11	0.32050418	0.377	0.235	1.92E-06	LAG3	6	TRBV9_CD8 Teff
1596	1.12E-10	0.31800233	0.346	0.209	2.44E-06	RNF166	6	TRBV9_CD8 Teff
1597	3.14E-10	0.3138134	0.244	0.13	6.84E-06	CD5	6	TRBV9_CD8 Teff
1598	1.90E-09	0.31055794	0.31	0.188	4.14E-05	ITGB7	6	TRBV9_CD8 Teff
1599	6.19E-09	0.31036082	0.302	0.185	0.000135069	SH3BP5	6	TRBV9_CD8 Teff
1600	2.17E-20	0.30718564	0.222	0.078	4.73E-16	LAIR2	6	TRBV9_CD8 Teff
1601	1.36E-09	0.30500348	0.482	0.342	2.96E-05	LYAR	6	TRBV9_CD8 Teff
1602	1.84E-06	0.29197528	0.238	0.15	0.040036116	KLF3	6	TRBV9_CD8 Teff
1603	1.79E-08	0.28915386	0.546	0.416	0.000390151	NME2	6	TRBV9_CD8 Teff
1604	7.76E-07	0.27248145	0.366	0.254	0.016932488	COTL1	6	TRBV9_CD8 Teff
1605	7.99E-17	0.26894494	0.85	0.556	1.74E-12	KLRD1	6	TRBV9_CD8 Teff
1606	2.28E-06	0.26542573	0.496	0.389	0.049651686	STOM	6	TRBV9_CD8 Teff
1607	2.07E-16	0.26203187	0.889	0.691	4.51E-12	PRF1	6	TRBV9_CD8 Teff
1608	4.29E-07	0.26040824	0.363	0.257	0.009363057	CD6	6	TRBV9_CD8 Teff

1609	1.78E-07	0.25287534	0.205	0.115	0.003883298	UBL3	6	TRBV9_CD8 Teff
1610	1.68E-07	0.25127262	0.183	0.098	0.003661245	CD226	6	TRBV9_CD8 Teff
1611	1.84E-06	-0.26894165	0.324	0.441	0.040028307	PDCD4	6	TRBV9_CD8 Teff
1612	5.99E-07	-0.26937628	0.147	0.271	0.013052811	LYST	6	TRBV9_CD8 Teff
1613	8.16E-07	-0.27342983	0.294	0.438	0.017787454	CKLF	6	TRBV9_CD8 Teff
1614	2.31E-07	-0.28121214	0.108	0.227	0.005030163	TOX	6	TRBV9_CD8 Teff
1615	1.88E-06	-0.28472951	0.197	0.318	0.040940817	CSRNP1	6	TRBV9_CD8 Teff
1616	2.65E-07	-0.28794561	0.521	0.646	0.005770288	TAGAP	6	TRBV9_CD8 Teff
1617	7.62E-07	-0.30491894	0.219	0.343	0.016623361	RHOC	6	TRBV9_CD8 Teff
1618	6.07E-11	-0.31268141	0.914	0.944	1.32E-06	ZFP36	6	TRBV9_CD8 Teff
1619	1.18E-07	-0.31431753	0.133	0.26	0.002578801	RBMS1	6	TRBV9_CD8 Teff
1620	7.30E-08	-0.32235563	0.114	0.239	0.001591257	CCND2	6	TRBV9_CD8 Teff
1621	7.17E-11	-0.33617043	0.817	0.864	1.56E-06	DUSP2	6	TRBV9_CD8 Teff
1622	5.02E-08	-0.38196039	0.548	0.651	0.001094348	PPP1R15A	6	TRBV9_CD8 Teff
1623	4.40E-09	-0.3947181	0.673	0.781	9.59E-05	JUN	6	TRBV9_CD8 Teff
1624	3.64E-08	-0.41098804	0.194	0.327	0.000793062	IFNGR1	6	TRBV9_CD8 Teff
1625	8.86E-10	-0.44314068	0.335	0.486	1.93E-05	BTG2	6	TRBV9_CD8 Teff
1626	1.28E-08	-0.45174638	0.731	0.789	0.00027936	NFKBIA	6	TRBV9_CD8 Teff
1627	3.37E-19	-0.49512283	0.809	0.892	7.35E-15	DUSP1	6	TRBV9_CD8 Teff
1628	1.41E-12	-0.51228894	0.161	0.337	3.07E-08	NCR3	6	TRBV9_CD8 Teff
1629	8.10E-12	-0.52524665	0.263	0.438	1.77E-07	NFKBIZ	6	TRBV9_CD8 Teff
1630	3.08E-08	-0.52553437	0.186	0.318	0.000671699	IER3	6	TRBV9_CD8 Teff
1631	1.94E-07	-0.54896188	0.92	0.875	0.004240201	CCL4	6	TRBV9_CD8 Teff
1632	6.79E-24	-0.61758178	0.806	0.907	1.48E-19	CD69	6	TRBV9_CD8 Teff
1633	1.96E-14	-0.64266769	0.152	0.347	4.28E-10	NR4A1	6	TRBV9_CD8 Teff
1634	9.39E-19	-0.65783616	0.319	0.546	2.05E-14	FOSB	6	TRBV9_CD8 Teff
1635	4.70E-12	-0.69782841	0.202	0.371	1.02E-07	RGS1	6	TRBV9_CD8 Teff
1636	4.59E-13	-0.73097806	0.133	0.304	1.00E-08	LTB	6	TRBV9_CD8 Teff
1637	1.57E-26	-0.75805891	0.241	0.53	3.41E-22	TRBC1	6	TRBV9_CD8 Teff
1638	9.41E-26	-0.77145303	0.679	0.842	2.05E-21	FOS	6	TRBV9_CD8 Teff
1639	2.15E-09	-0.86026784	0.601	0.675	4.69E-05	CCL4L2	6	TRBV9_CD8 Teff
1640	5.99E-11	-0.87806877	0.296	0.418	1.31E-06	IL7R	6	TRBV9_CD8 Teff
1641	1.51E-28	-0.89741057	0.175	0.482	3.30E-24	GZMK	6	TRBV9_CD8 Teff
1642	1.03E-09	-0.91731484	0.324	0.466	2.24E-05	CCL3	6	TRBV9_CD8 Teff
1643	1.56E-25	-0.93740869	0.13	0.394	3.40E-21	KLRF1	6	TRBV9_CD8 Teff
1644	2.16E-27	-0.98786616	0.371	0.625	4.72E-23	KLRB1	6	TRBV9_CD8 Teff
1645	1.40E-20	-1.03182836	0.152	0.388	3.04E-16	CCL3L3	6	TRBV9_CD8 Teff
1646	2.10E-32	-1.10391503	0.177	0.488	4.57E-28	TYROBP	6	TRBV9_CD8 Teff
1647	5.39E-21	-1.23519961	0.183	0.411	1.18E-16	AREG	6	TRBV9_CD8 Teff
1648	5.08E-211	2.60016714	0.996	0.298	1.11E-206	STMN1	7	STMN1_NK-cycling
1649	2.42E-112	2.20179349	0.883	0.352	5.27E-108	HIST1H4C	7	STMN1_NK-cycling
1650	0	2.04312952	0.786	0.032	0	UBE2C	7	STMN1_NK-cycling
1651	1.27E-171	1.99210306	0.964	0.313	2.77E-167	HMGB2	7	STMN1_NK-cycling
1652	1.83E-154	1.99040868	0.992	0.454	3.98E-150	TUBB	7	STMN1_NK-cycling
1653	0	1.98909396	0.835	0.025	0	TYMS	7	STMN1_NK-cycling
1654	6.63E-140	1.98405413	0.984	0.568	1.45E-135	TUBA1B	7	STMN1_NK-cycling
1655	8.49E-146	1.8059396	0.992	0.531	1.85E-141	HMGN2	7	STMN1_NK-cycling
1656	0	1.77117043	0.806	0.04	0	NUSAP1	7	STMN1_NK-cycling
1657	0	1.75682718	0.843	0.038	0	MKI67	7	STMN1_NK-cycling
1658	0	1.73241916	0.762	0.029	0	RRM2	7	STMN1_NK-cycling
1659	1.21E-98	1.71299856	0.911	0.444	2.63E-94	ARL6IP1	7	STMN1_NK-cycling
1660	0	1.69337667	0.73	0.033	0	ASPM	7	STMN1_NK-cycling
1661	0	1.67773553	0.738	0.032	0	CENPF	7	STMN1_NK-cycling
1662	2.02E-304	1.63271553	0.839	0.086	4.40E-300	SMC4	7	STMN1_NK-cycling
1663	0	1.63032361	0.742	0.035	0	TOP2A	7	STMN1_NK-cycling
1664	1.27E-196	1.45784425	0.786	0.122	2.78E-192	PTTG1	7	STMN1_NK-cycling
1665	0	1.39981225	0.69	0.029	0	TPX2	7	STMN1_NK-cycling
1666	6.50E-112	1.35098704	0.984	0.603	1.42E-107	H2AFZ	7	STMN1_NK-cycling
1667	0	1.35043795	0.746	0.059	0	CKS1B	7	STMN1_NK-cycling
1668	0	1.29966487	0.669	0.025	0	CDK1	7	STMN1_NK-cycling
1669	0	1.29353047	0.665	0.027	0	BIRC5	7	STMN1_NK-cycling
1670	2.30E-270	1.25824637	0.524	0.029	5.02E-266	CDC20	7	STMN1_NK-cycling
1671	3.75E-117	1.22487462	1	0.814	8.18E-113	HMGB1	7	STMN1_NK-cycling
1672	4.02E-272	1.18452903	0.677	0.055	8.76E-268	MAD2L1	7	STMN1_NK-cycling
1673	0	1.16324747	0.625	0.024	0	CDCA8	7	STMN1_NK-cycling
1674	0	1.1617878	0.548	0.021	0	KIAA0101	7	STMN1_NK-cycling
1675	0	1.15191984	0.54	0.021	0	CCNB2	7	STMN1_NK-cycling
1676	3.52E-122	1.14990964	0.891	0.281	7.68E-118	H2AFV	7	STMN1_NK-cycling
1677	2.26E-34	1.12907759	0.726	0.352	4.92E-30	LGALS1	7	STMN1_NK-cycling
1678	0	1.12880146	0.585	0.019	0	DLGAP5	7	STMN1_NK-cycling

1679	0	1.12771975	0.609	0.025	0	CDKN3	7	STMN1_NK-cycling
1680	2.47E-96	1.1240323	0.931	0.395	5.38E-92	ANP32B	7	STMN1_NK-cycling
1681	1.76E-216	1.1112413	0.653	0.066	3.84E-212	KIF20B	7	STMN1_NK-cycling
1682	1.49E-90	1.11028988	0.984	0.816	3.24E-86	VIM	7	STMN1_NK-cycling
1683	0	1.10451334	0.613	0.021	0	GTSE1	7	STMN1_NK-cycling
1684	0	1.09554011	0.556	0.024	0	HMMR	7	STMN1_NK-cycling
1685	0	1.09072354	0.573	0.021	0	CCNA2	7	STMN1_NK-cycling
1686	1.33E-153	1.08902947	0.774	0.137	2.91E-149	TMPO	7	STMN1_NK-cycling
1687	0	1.08726363	0.488	0.017	0	PLK1	7	STMN1_NK-cycling
1688	2.17E-86	1.07433653	0.601	0.141	4.74E-82	KPNA2	7	STMN1_NK-cycling
1689	0	1.07079548	0.613	0.023	0	KIFC1	7	STMN1_NK-cycling
1690	0	1.06867319	0.585	0.021	0	CASC5	7	STMN1_NK-cycling
1691	7.27E-109	1.05850103	0.903	0.296	1.59E-104	NUCKS1	7	STMN1_NK-cycling
1692	5.00E-186	1.05680541	0.694	0.089	1.09E-181	KIF22	7	STMN1_NK-cycling
1693	8.59E-96	1.0383897	0.851	0.278	1.87E-91	CKS2	7	STMN1_NK-cycling
1694	0	1.03800132	0.613	0.031	0	RACGAP1	7	STMN1_NK-cycling
1695	2.97E-211	1.01672889	0.419	0.024	6.48E-207	CCNB1	7	STMN1_NK-cycling
1696	0	1.01583699	0.577	0.027	0	UBE2T	7	STMN1_NK-cycling
1697	2.26E-117	1.01008723	0.589	0.101	4.93E-113	PCNA	7	STMN1_NK-cycling
1698	2.90E-107	1.00081791	0.859	0.244	6.33E-103	NUCB2	7	STMN1_NK-cycling
1699	1.51E-80	0.98257386	0.875	0.346	3.29E-76	RAD21	7	STMN1_NK-cycling
1700	0	0.98188139	0.46	0.011	0	PKMYT1	7	STMN1_NK-cycling
1701	2.91E-307	0.97401351	0.56	0.028	6.36E-303	NUF2	7	STMN1_NK-cycling
1702	3.90E-74	0.96504363	0.972	0.648	8.50E-70	CRIP1	7	STMN1_NK-cycling
1703	5.68E-286	0.96095348	0.516	0.025	1.24E-281	TROAP	7	STMN1_NK-cycling
1704	3.18E-85	0.95797444	0.891	0.347	6.94E-81	DEK	7	STMN1_NK-cycling
1705	8.36E-301	0.95713788	0.573	0.03	1.82E-296	PRC1	7	STMN1_NK-cycling
1706	1.85E-175	0.95318997	0.516	0.049	4.04E-171	ATAD2	7	STMN1_NK-cycling
1707	2.12E-74	0.94433891	0.782	0.254	4.62E-70	DUT	7	STMN1_NK-cycling
1708	9.61E-259	0.93311646	0.56	0.036	2.10E-254	CENPM	7	STMN1_NK-cycling
1709	0	0.9319932	0.488	0.017	0	KIF23	7	STMN1_NK-cycling
1710	5.25E-81	0.92609574	0.883	0.354	1.15E-76	CALM3	7	STMN1_NK-cycling
1711	1.15E-239	0.92251649	0.536	0.036	2.51E-235	EZH2	7	STMN1_NK-cycling
1712	4.05E-121	0.91589437	0.5	0.07	8.84E-117	MCM7	7	STMN1_NK-cycling
1713	5.50E-60	0.91587274	0.96	0.705	1.20E-55	CALM2	7	STMN1_NK-cycling
1714	0	0.90977754	0.464	0.01	0	FAM64A	7	STMN1_NK-cycling
1715	6.78E-79	0.90804949	0.722	0.21	1.48E-74	TUBA1C	7	STMN1_NK-cycling
1716	1.58E-187	0.90331625	0.625	0.069	3.44E-183	CENPK	7	STMN1_NK-cycling
1717	0	0.88944959	0.512	0.018	0	HJURP	7	STMN1_NK-cycling
1718	4.29E-100	0.88933574	0.71	0.157	9.36E-96	TRDC	7	STMN1_NK-cycling
1719	6.41E-220	0.88777292	0.573	0.047	1.40E-215	SMC2	7	STMN1_NK-cycling
1720	2.20E-90	0.87799822	0.762	0.207	4.79E-86	ANP32E	7	STMN1_NK-cycling
1721	0	0.8767604	0.492	0.013	0	RAD51AP1	7	STMN1_NK-cycling
1722	0	0.86402184	0.5	0.014	0	CKAP2L	7	STMN1_NK-cycling
1723	0	0.8631052	0.444	0.013	0	CDT1	7	STMN1_NK-cycling
1724	9.16E-90	0.85663951	0.673	0.162	2.00E-85	CDKN2D	7	STMN1_NK-cycling
1725	0	0.8495909	0.476	0.016	0	AURKB	7	STMN1_NK-cycling
1726	1.47E-85	0.84705547	0.722	0.199	3.21E-81	DDX39A	7	STMN1_NK-cycling
1727	2.77E-112	0.84562256	0.694	0.139	6.03E-108	CARHSP1	7	STMN1_NK-cycling
1728	7.90E-75	0.84158825	0.964	0.523	1.72E-70	COX8A	7	STMN1_NK-cycling
1729	2.11E-122	0.8404069	0.617	0.101	4.61E-118	LMNB1	7	STMN1_NK-cycling
1730	8.45E-87	0.83646378	0.694	0.178	1.84E-82	CDC25B	7	STMN1_NK-cycling
1731	1.03E-156	0.83328315	0.508	0.053	2.25E-152	CDKN2C	7	STMN1_NK-cycling
1732	5.83E-264	0.83015199	0.448	0.019	1.27E-259	KIF14	7	STMN1_NK-cycling
1733	7.64E-255	0.82096278	0.448	0.02	1.67E-250	CENPE	7	STMN1_NK-cycling
1734	3.80E-83	0.81910362	0.617	0.139	8.30E-79	IGFBP7	7	STMN1_NK-cycling
1735	4.40E-77	0.81437414	0.827	0.277	9.59E-73	BANF1	7	STMN1_NK-cycling
1736	4.20E-62	0.81341614	0.766	0.283	9.16E-58	HN1	7	STMN1_NK-cycling
1737	1.35E-85	0.81249171	0.669	0.165	2.94E-81	H2AFY	7	STMN1_NK-cycling
1738	6.34E-61	0.81230368	0.407	0.085	1.38E-56	HIST1H1E	7	STMN1_NK-cycling
1739	2.04E-254	0.80774078	0.448	0.021	4.45E-250	NCAPH	7	STMN1_NK-cycling
1740	7.18E-105	0.8066835	1	0.991	1.57E-100	PTMA	7	STMN1_NK-cycling
1741	1.58E-109	0.80474385	0.661	0.127	3.45E-105	MZT1	7	STMN1_NK-cycling
1742	2.22E-285	0.80428397	0.407	0.013	4.85E-281	UHRF1	7	STMN1_NK-cycling
1743	1.44E-258	0.79868001	0.456	0.021	3.14E-254	ASF1B	7	STMN1_NK-cycling
1744	1.33E-276	0.79577297	0.452	0.018	2.90E-272	CDCA2	7	STMN1_NK-cycling
1745	2.43E-291	0.79354886	0.431	0.014	5.30E-287	ZWINT	7	STMN1_NK-cycling
1746	1.00E-225	0.79250237	0.468	0.028	2.19E-221	SGOL2	7	STMN1_NK-cycling
1747	1.46E-60	0.79229353	0.806	0.313	3.19E-56	HP1BP3	7	STMN1_NK-cycling
1748	7.98E-66	0.78862969	0.702	0.221	1.74E-61	RHEB	7	STMN1_NK-cycling

1749	7.23E-301	0.78542045	0.423	0.013	1.58E-296	CENPA	7	STMN1_NK-cycling
1750	4.49E-50	0.7844899	0.911	0.521	9.79E-46	TUBB4B	7	STMN1_NK-cycling
1751	1.00E-208	0.7841715	0.423	0.024	2.19E-204	DHFR	7	STMN1_NK-cycling
1752	8.18E-260	0.78138111	0.431	0.018	1.78E-255	NCAPG	7	STMN1_NK-cycling
1753	3.03E-123	0.77961869	0.585	0.091	6.61E-119	SKA2	7	STMN1_NK-cycling
1754	5.26E-269	0.77944582	0.383	0.012	1.15E-264	SPC25	7	STMN1_NK-cycling
1755	1.48E-246	0.77938918	0.444	0.021	3.24E-242	CDCA3	7	STMN1_NK-cycling
1756	5.01E-64	0.77893852	0.94	0.519	1.09E-59	RAN	7	STMN1_NK-cycling
1757	1.03E-64	0.76545915	0.681	0.209	2.26E-60	LSM5	7	STMN1_NK-cycling
1758	1.02E-159	0.76535575	0.528	0.057	2.22E-155	NCAPD2	7	STMN1_NK-cycling
1759	0	0.76214495	0.435	0.011	0	KIF15	7	STMN1_NK-cycling
1760	2.42E-268	0.7603133	0.355	0.009	5.28E-264	CRNDE	7	STMN1_NK-cycling
1761	1.44E-287	0.7588871	0.411	0.013	3.13E-283	CDCA5	7	STMN1_NK-cycling
1762	9.47E-263	0.75842113	0.391	0.013	2.07E-258	CLSPN	7	STMN1_NK-cycling
1763	7.11E-88	0.7577236	0.633	0.142	1.55E-83	USP1	7	STMN1_NK-cycling
1764	9.01E-141	0.75581422	0.54	0.067	1.96E-136	TACC3	7	STMN1_NK-cycling
1765	3.45E-68	0.75456914	0.879	0.334	7.53E-64	C12orf75	7	STMN1_NK-cycling
1766	6.53E-297	0.74488842	0.44	0.015	1.42E-292	KIF2C	7	STMN1_NK-cycling
1767	5.26E-120	0.7431345	0.492	0.066	1.15E-115	CKAP5	7	STMN1_NK-cycling
1768	2.35E-236	0.73639531	0.331	0.01	5.12E-232	TK1	7	STMN1_NK-cycling
1769	3.85E-63	0.73241881	0.778	0.271	8.40E-59	DCTN3	7	STMN1_NK-cycling
1770	9.19E-257	0.73023047	0.419	0.017	2.00E-252	CENPU	7	STMN1_NK-cycling
1771	6.21E-202	0.72910255	0.435	0.027	1.35E-197	SAC3D1	7	STMN1_NK-cycling
1772	3.16E-131	0.72876254	0.472	0.055	6.90E-127	CKAP2	7	STMN1_NK-cycling
1773	7.05E-99	0.72708272	0.484	0.076	1.54E-94	TMEM106C	7	STMN1_NK-cycling
1774	2.03E-190	0.72546847	0.496	0.04	4.44E-186	RPL39L	7	STMN1_NK-cycling
1775	6.79E-135	0.71918243	0.262	0.014	1.48E-130	HIST1H1B	7	STMN1_NK-cycling
1776	1.48E-100	0.714045	0.492	0.077	3.23E-96	DNAJC9	7	STMN1_NK-cycling
1777	5.48E-227	0.70783372	0.387	0.017	1.20E-222	CENPW	7	STMN1_NK-cycling
1778	6.10E-48	0.70736736	0.806	0.36	1.33E-43	H2AFX	7	STMN1_NK-cycling
1779	4.39E-235	0.70502003	0.395	0.017	9.57E-231	KIF11	7	STMN1_NK-cycling
1780	3.11E-54	0.70498585	0.919	0.468	6.77E-50	HMGN1	7	STMN1_NK-cycling
1781	1.53E-86	0.70279746	0.472	0.082	3.34E-82	RP11-620J15.3	7	STMN1_NK-cycling
1782	1.05E-144	0.70103253	0.476	0.05	2.30E-140	DTYMK	7	STMN1_NK-cycling
1783	1.02E-241	0.70056881	0.419	0.019	2.22E-237	DEPDC1B	7	STMN1_NK-cycling
1784	7.97E-70	0.69881473	0.593	0.148	1.74E-65	FAM111A	7	STMN1_NK-cycling
1785	3.97E-242	0.69661833	0.403	0.017	8.65E-238	BUB1	7	STMN1_NK-cycling
1786	3.19E-53	0.69457967	0.871	0.406	6.95E-49	GNG5	7	STMN1_NK-cycling
1787	1.49E-48	0.6930274	0.835	0.359	3.25E-44	UCP2	7	STMN1_NK-cycling
1788	6.73E-120	0.68948808	0.476	0.062	1.47E-115	CD59	7	STMN1_NK-cycling
1789	2.41E-293	0.68873047	0.363	0.008	5.25E-289	DEPDC1	7	STMN1_NK-cycling
1790	6.09E-217	0.68574062	0.415	0.022	1.33E-212	PARPBP	7	STMN1_NK-cycling
1791	1.39E-164	0.68509273	0.391	0.028	3.04E-160	AURKA	7	STMN1_NK-cycling
1792	1.06E-178	0.68429916	0.363	0.021	2.32E-174	MXD3	7	STMN1_NK-cycling
1793	1.37E-88	0.67746176	0.548	0.106	2.98E-84	SAE1	7	STMN1_NK-cycling
1794	6.92E-66	0.677313	0.585	0.148	1.51E-61	RPA3	7	STMN1_NK-cycling
1795	8.93E-52	0.67588153	0.911	0.453	1.95E-47	BUB3	7	STMN1_NK-cycling
1796	7.72E-114	0.67075136	0.452	0.058	1.68E-109	PHF19	7	STMN1_NK-cycling
1797	3.43E-223	0.669693	0.383	0.017	7.48E-219	SGOL1	7	STMN1_NK-cycling
1798	6.43E-203	0.66824598	0.411	0.023	1.40E-198	NDC80	7	STMN1_NK-cycling
1799	1.48E-49	0.65976883	0.734	0.275	3.23E-45	LBR	7	STMN1_NK-cycling
1800	7.64E-147	0.65521122	0.419	0.037	1.67E-142	NCAPD3	7	STMN1_NK-cycling
1801	1.06E-221	0.65122203	0.399	0.019	2.30E-217	GGH	7	STMN1_NK-cycling
1802	4.93E-273	0.64595512	0.367	0.01	1.08E-268	APOBEC3B	7	STMN1_NK-cycling
1803	2.92E-70	0.64574701	0.609	0.153	6.38E-66	SMS	7	STMN1_NK-cycling
1804	9.55E-60	0.64213915	0.73	0.232	2.08E-55	ILF2	7	STMN1_NK-cycling
1805	1.75E-56	0.64145375	0.573	0.161	3.81E-52	DNMT1	7	STMN1_NK-cycling
1806	2.46E-195	0.6397958	0.452	0.031	5.37E-191	CENPN	7	STMN1_NK-cycling
1807	2.38E-60	0.63594732	0.988	0.834	5.19E-56	HNRNPA2B1	7	STMN1_NK-cycling
1808	2.00E-92	0.63162238	0.448	0.069	4.37E-88	RRM1	7	STMN1_NK-cycling
1809	3.81E-69	0.6314177	1	0.997	8.31E-65	ACTB	7	STMN1_NK-cycling
1810	9.79E-188	0.63057757	0.359	0.019	2.13E-183	FANCI	7	STMN1_NK-cycling
1811	8.28E-110	0.62808964	0.395	0.046	1.81E-105	BARD1	7	STMN1_NK-cycling
1812	2.47E-69	0.62732911	0.577	0.137	5.38E-65	EBP	7	STMN1_NK-cycling
1813	1.25E-45	0.62690771	0.895	0.495	2.72E-41	SLC25A5	7	STMN1_NK-cycling
1814	7.91E-206	0.62644974	0.331	0.013	1.73E-201	NEK2	7	STMN1_NK-cycling
1815	9.69E-177	0.6242797	0.351	0.019	2.11E-172	TCF19	7	STMN1_NK-cycling
1816	4.06E-63	0.62363036	0.621	0.163	8.86E-59	KIR3DL1	7	STMN1_NK-cycling
1817	1.41E-46	0.61970886	0.347	0.077	3.07E-42	HIST1H1D	7	STMN1_NK-cycling
1818	7.35E-43	0.61543257	0.819	0.369	1.60E-38	HMGN3	7	STMN1_NK-cycling

1819	4.20E-45	0.61265457	0.597	0.199	9.15E-41	RANBP1	7	STMN1_NK-cycling
1820	1.28E-68	0.6093466	0.609	0.151	2.79E-64	LSM4	7	STMN1_NK-cycling
1821	2.12E-63	0.60742717	0.581	0.147	4.63E-59	UBE2S	7	STMN1_NK-cycling
1822	2.78E-140	0.60564044	0.323	0.022	6.07E-136	FBXO5	7	STMN1_NK-cycling
1823	1.54E-46	0.60468762	0.589	0.187	3.37E-42	HLA-DRA	7	STMN1_NK-cycling
1824	1.26E-61	0.60407013	0.698	0.205	2.76E-57	SNRPD1	7	STMN1_NK-cycling
1825	1.52E-58	0.60196844	0.992	0.858	3.31E-54	H3F3A	7	STMN1_NK-cycling
1826	2.41E-35	0.60140309	0.742	0.336	5.27E-31	ANXA2	7	STMN1_NK-cycling
1827	9.13E-112	0.59780744	0.411	0.048	1.99E-107	ITGB3BP	7	STMN1_NK-cycling
1828	1.53E-38	0.59712159	0.52	0.176	3.34E-34	PTMS	7	STMN1_NK-cycling
1829	2.00E-52	0.59605402	0.673	0.216	4.35E-48	PSIP1	7	STMN1_NK-cycling
1830	4.60E-52	0.59484162	0.415	0.098	1.00E-47	SPTBN1	7	STMN1_NK-cycling
1831	6.21E-218	0.59436291	0.339	0.012	1.36E-213	ANLN	7	STMN1_NK-cycling
1832	1.12E-37	0.59319647	0.875	0.464	2.44E-33	RPS27L	7	STMN1_NK-cycling
1833	3.71E-38	0.59107629	0.843	0.428	8.10E-34	ARPC5	7	STMN1_NK-cycling
1834	3.90E-223	0.58934062	0.306	0.009	8.52E-219	ESCO2	7	STMN1_NK-cycling
1835	3.26E-40	0.58790132	0.669	0.25	7.10E-36	HLA-DRB5	7	STMN1_NK-cycling
1836	3.22E-170	0.58464607	0.379	0.024	7.02E-166	ARHGAP11A	7	STMN1_NK-cycling
1837	1.01E-158	0.58435916	0.327	0.019	2.21E-154	BRCA2	7	STMN1_NK-cycling
1838	4.82E-45	0.58323263	0.601	0.197	1.05E-40	SIVA1	7	STMN1_NK-cycling
1839	1.14E-159	0.58216783	0.339	0.021	2.49E-155	ARHGAP11B	7	STMN1_NK-cycling
1840	4.42E-189	0.58056794	0.315	0.013	9.63E-185	CEP55	7	STMN1_NK-cycling
1841	1.63E-64	0.57815836	0.504	0.115	3.56E-60	MIS18BP1	7	STMN1_NK-cycling
1842	6.09E-52	0.57737291	0.706	0.234	1.33E-47	VDAC3	7	STMN1_NK-cycling
1843	1.72E-172	0.5772771	0.343	0.019	3.75E-168	NCAPG2	7	STMN1_NK-cycling
1844	9.23E-66	0.57407843	0.532	0.123	2.01E-61	MAD2L2	7	STMN1_NK-cycling
1845	1.51E-36	0.5712698	0.915	0.523	3.30E-32	GSTP1	7	STMN1_NK-cycling
1846	7.61E-184	0.56994979	0.347	0.018	1.66E-179	ECT2	7	STMN1_NK-cycling
1847	4.07E-204	0.56917834	0.298	0.01	8.87E-200	PBK	7	STMN1_NK-cycling
1848	1.68E-114	0.569093	0.351	0.034	3.65E-110	WHSC1	7	STMN1_NK-cycling
1849	7.64E-196	0.56877909	0.327	0.014	1.67E-191	TTK	7	STMN1_NK-cycling
1850	5.18E-43	0.56600316	0.899	0.359	1.13E-38	GNLY	7	STMN1_NK-cycling
1851	8.09E-93	0.56587553	0.258	0.022	1.77E-88	HIST1H2AJ	7	STMN1_NK-cycling
1852	2.22E-55	0.56499091	0.44	0.102	4.85E-51	SMC1A	7	STMN1_NK-cycling
1853	4.85E-45	0.56400661	0.98	0.721	1.06E-40	YBX1	7	STMN1_NK-cycling
1854	1.78E-60	0.56294071	0.387	0.075	3.88E-56	MCM3	7	STMN1_NK-cycling
1855	3.31E-58	0.56224169	0.468	0.109	7.22E-54	DDB2	7	STMN1_NK-cycling
1856	1.47E-191	0.56055412	0.306	0.012	3.20E-187	KIF4A	7	STMN1_NK-cycling
1857	7.03E-103	0.55951934	0.323	0.032	1.53E-98	LIG1	7	STMN1_NK-cycling
1858	2.40E-93	0.55777676	0.387	0.051	5.24E-89	REEP4	7	STMN1_NK-cycling
1859	5.51E-77	0.55703589	0.488	0.092	1.20E-72	PRPSAP1	7	STMN1_NK-cycling
1860	3.07E-55	0.55520782	1	0.939	6.70E-51	GAPDH	7	STMN1_NK-cycling
1861	1.11E-48	0.55486838	0.597	0.183	2.43E-44	LSM3	7	STMN1_NK-cycling
1862	4.29E-36	0.55249114	0.738	0.308	9.36E-32	TPM4	7	STMN1_NK-cycling
1863	1.69E-107	0.55071748	0.254	0.018	3.68E-103	HELLS	7	STMN1_NK-cycling
1864	1.82E-164	0.54952189	0.298	0.014	3.96E-160	SPAG5	7	STMN1_NK-cycling
1865	3.00E-44	0.54847568	0.633	0.217	6.55E-40	DCK	7	STMN1_NK-cycling
1866	1.60E-52	0.54806219	0.573	0.162	3.50E-48	MIR4435-2HG	7	STMN1_NK-cycling
1867	6.93E-47	0.54736071	1	0.958	1.51E-42	ACTG1	7	STMN1_NK-cycling
1868	8.41E-33	0.54557276	0.786	0.399	1.83E-28	CDK2AP2	7	STMN1_NK-cycling
1869	2.52E-45	0.54351166	0.629	0.209	5.49E-41	IDH2	7	STMN1_NK-cycling
1870	7.07E-49	0.54050169	0.605	0.187	1.54E-44	MZT2B	7	STMN1_NK-cycling
1871	7.84E-68	0.53951596	0.423	0.08	1.71E-63	NUDT1	7	STMN1_NK-cycling
1872	1.12E-210	0.5380432	0.319	0.011	2.43E-206	PSRC1	7	STMN1_NK-cycling
1873	5.28E-40	0.53692882	0.98	0.691	1.15E-35	S100A10	7	STMN1_NK-cycling
1874	4.21E-72	0.53513192	0.435	0.08	9.17E-68	CBX5	7	STMN1_NK-cycling
1875	6.23E-53	0.53463804	0.282	0.047	1.36E-48	MCM5	7	STMN1_NK-cycling
1876	6.59E-181	0.53342953	0.29	0.011	1.44E-176	NEIL3	7	STMN1_NK-cycling
1877	1.03E-35	0.53162831	0.875	0.507	2.25E-31	HNRNPA3	7	STMN1_NK-cycling
1878	1.21E-29	0.53117078	0.556	0.229	2.64E-25	NASP	7	STMN1_NK-cycling
1879	2.45E-108	0.52766772	0.274	0.021	5.35E-104	FEN1	7	STMN1_NK-cycling
1880	6.00E-94	0.5265092	0.371	0.046	1.31E-89	GMNN	7	STMN1_NK-cycling
1881	7.63E-37	0.5251098	0.774	0.357	1.66E-32	ERH	7	STMN1_NK-cycling
1882	1.66E-35	0.52193572	0.726	0.298	3.62E-31	CBX3	7	STMN1_NK-cycling
1883	4.04E-42	0.52150345	0.532	0.168	8.82E-38	SMC3	7	STMN1_NK-cycling
1884	3.02E-120	0.51996408	0.246	0.014	6.58E-116	HMGB3	7	STMN1_NK-cycling
1885	2.69E-51	0.51985069	0.476	0.121	5.88E-47	CNTRL	7	STMN1_NK-cycling
1886	1.24E-144	0.51802186	0.27	0.014	2.70E-140	KIF20A	7	STMN1_NK-cycling
1887	8.65E-76	0.51669825	0.399	0.065	1.89E-71	ACYP1	7	STMN1_NK-cycling
1888	2.36E-34	0.51403253	0.71	0.295	5.14E-30	HNRNPR	7	STMN1_NK-cycling

1889	7.74E-43	0.5136244	0.867	0.356	1.69E-38	KLRF1	7	STMN1_NK-cycling
1890	2.16E-201	0.51343608	0.294	0.009	4.70E-197	MELK	7	STMN1_NK-cycling
1891	1.88E-40	0.51334648	0.972	0.794	4.11E-36	CLIC1	7	STMN1_NK-cycling
1892	3.57E-96	0.50969038	0.323	0.034	7.79E-92	LMNB2	7	STMN1_NK-cycling
1893	3.27E-48	0.50660562	0.472	0.122	7.12E-44	STRA13	7	STMN1_NK-cycling
1894	2.39E-34	0.50615757	0.927	0.59	5.21E-30	EMP3	7	STMN1_NK-cycling
1895	5.07E-59	0.50596874	0.48	0.11	1.11E-54	DBF4	7	STMN1_NK-cycling
1896	7.22E-45	0.5053347	0.609	0.195	1.58E-40	DCAF7	7	STMN1_NK-cycling
1897	1.08E-43	0.50451819	0.556	0.172	2.36E-39	RBBP7	7	STMN1_NK-cycling
1898	1.74E-56	0.50415517	1	0.978	3.80E-52	PFN1	7	STMN1_NK-cycling
1899	2.03E-193	0.50171695	0.274	0.008	4.44E-189	SKA1	7	STMN1_NK-cycling
1900	3.85E-49	0.50121414	1	0.935	8.40E-45	CFL1	7	STMN1_NK-cycling
1901	2.08E-86	0.50074507	0.29	0.031	4.54E-82	NRM	7	STMN1_NK-cycling
1902	1.21E-52	0.50052186	1	0.941	2.65E-48	PPIA	7	STMN1_NK-cycling
1903	1.07E-58	0.49935432	0.456	0.1	2.33E-54	CD38	7	STMN1_NK-cycling
1904	1.56E-37	0.49815905	0.706	0.273	3.41E-33	NONO	7	STMN1_NK-cycling
1905	6.69E-134	0.49662841	0.242	0.011	1.46E-129	ORC6	7	STMN1_NK-cycling
1906	2.07E-34	0.4930761	0.952	0.704	4.52E-30	SUMO2	7	STMN1_NK-cycling
1907	2.04E-32	0.49247371	0.786	0.366	4.44E-28	RALY	7	STMN1_NK-cycling
1908	3.74E-212	0.49119682	0.29	0.008	8.16E-208	FOXM1	7	STMN1_NK-cycling
1909	7.71E-90	0.4841722	0.351	0.043	1.68E-85	HIRIP3	7	STMN1_NK-cycling
1910	1.39E-28	0.48392704	0.827	0.458	3.04E-24	LRRFIP1	7	STMN1_NK-cycling
1911	1.12E-38	0.48126422	0.694	0.259	2.45E-34	DCXR	7	STMN1_NK-cycling
1912	9.95E-27	0.48003024	0.488	0.191	2.17E-22	SLBP	7	STMN1_NK-cycling
1913	2.35E-29	0.47954921	0.609	0.253	5.12E-25	CDKN1A	7	STMN1_NK-cycling
1914	1.97E-33	0.47763858	0.593	0.223	4.29E-29	PLP2	7	STMN1_NK-cycling
1915	1.34E-149	0.47206321	0.242	0.009	2.91E-145	MND1	7	STMN1_NK-cycling
1916	7.71E-28	0.4717975	0.855	0.471	1.68E-23	DBI	7	STMN1_NK-cycling
1917	4.03E-41	0.4711066	0.427	0.115	8.80E-37	HPRT1	7	STMN1_NK-cycling
1918	2.02E-24	0.47021877	0.915	0.563	4.41E-20	TPI1	7	STMN1_NK-cycling
1919	2.31E-80	0.46996821	0.282	0.032	5.04E-76	CCDC34	7	STMN1_NK-cycling
1920	2.73E-49	0.46563171	0.274	0.047	5.95E-45	CDKN2A	7	STMN1_NK-cycling
1921	1.96E-89	0.46555264	0.351	0.043	4.27E-85	BCL2L12	7	STMN1_NK-cycling
1922	6.42E-37	0.46458884	0.613	0.216	1.40E-32	BLOC1S1	7	STMN1_NK-cycling
1923	1.80E-19	0.46337727	0.831	0.518	3.93E-15	HNRNPF	7	STMN1_NK-cycling
1924	3.39E-39	0.46253072	0.968	0.776	7.39E-35	RHOA	7	STMN1_NK-cycling
1925	2.48E-31	0.46225667	0.806	0.385	5.41E-27	SRP9	7	STMN1_NK-cycling
1926	1.99E-146	0.4613656	0.23	0.008	4.34E-142	HIST1H3B	7	STMN1_NK-cycling
1927	1.70E-42	0.46065709	0.56	0.174	3.70E-38	YWHAH	7	STMN1_NK-cycling
1928	2.28E-30	0.45634434	0.589	0.233	4.97E-26	MRPL51	7	STMN1_NK-cycling
1929	3.28E-160	0.45519031	0.238	0.008	7.16E-156	STIL	7	STMN1_NK-cycling
1930	1.18E-57	0.45380456	0.383	0.076	2.58E-53	RUVBL2	7	STMN1_NK-cycling
1931	2.19E-111	0.45328213	0.27	0.02	4.78E-107	SPDL1	7	STMN1_NK-cycling
1932	2.23E-81	0.45295005	0.254	0.025	4.85E-77	CENPH	7	STMN1_NK-cycling
1933	2.46E-31	0.45286629	0.351	0.1	5.38E-27	HIST1H1C	7	STMN1_NK-cycling
1934	6.65E-181	0.45074117	0.274	0.009	1.45E-176	DIAPH3	7	STMN1_NK-cycling
1935	1.00E-120	0.44207784	0.298	0.022	2.19E-116	FAM83D	7	STMN1_NK-cycling
1936	7.12E-91	0.44061228	0.266	0.024	1.55E-86	TTF2	7	STMN1_NK-cycling
1937	2.24E-30	0.44000456	0.633	0.246	4.89E-26	KHDRBS1	7	STMN1_NK-cycling
1938	1.27E-67	0.43900798	0.315	0.046	2.77E-63	RNASEH2A	7	STMN1_NK-cycling
1939	8.99E-34	0.43418499	0.524	0.179	1.96E-29	UBE2E3	7	STMN1_NK-cycling
1940	1.51E-28	0.43414928	0.992	0.804	3.30E-24	ARPC2	7	STMN1_NK-cycling
1941	2.43E-28	0.43381118	0.952	0.608	5.29E-24	PSMB9	7	STMN1_NK-cycling
1942	3.58E-61	0.43377792	0.282	0.041	7.82E-57	DLEU2	7	STMN1_NK-cycling
1943	9.54E-29	0.43221442	0.649	0.271	2.08E-24	NDUFA2	7	STMN1_NK-cycling
1944	1.15E-32	0.43192125	0.387	0.113	2.51E-28	HAT1	7	STMN1_NK-cycling
1945	7.94E-28	0.43188562	0.98	0.789	1.73E-23	CD74	7	STMN1_NK-cycling
1946	1.41E-28	0.43177911	0.657	0.281	3.07E-24	SPN	7	STMN1_NK-cycling
1947	2.23E-133	0.43147006	0.246	0.012	4.86E-129	BRCA1	7	STMN1_NK-cycling
1948	3.87E-55	0.43145008	0.315	0.055	8.45E-51	CCDC18	7	STMN1_NK-cycling
1949	1.95E-124	0.42958654	0.242	0.013	4.25E-120	KIF18A	7	STMN1_NK-cycling
1950	2.98E-133	0.42696919	0.242	0.012	6.51E-129	BUB1B	7	STMN1_NK-cycling
1951	8.17E-152	0.42467495	0.262	0.011	1.78E-147	SHCBP1	7	STMN1_NK-cycling
1952	6.58E-129	0.42425963	0.226	0.01	1.43E-124	POC1A	7	STMN1_NK-cycling
1953	1.06E-55	0.42409996	0.246	0.035	2.31E-51	RFC4	7	STMN1_NK-cycling
1954	8.92E-23	0.4240259	0.714	0.353	1.95E-18	NAP1L1	7	STMN1_NK-cycling
1955	5.63E-142	0.42366026	0.23	0.009	1.23E-137	C21orf58	7	STMN1_NK-cycling
1956	2.32E-28	0.42305382	0.552	0.215	5.05E-24	HSPB11	7	STMN1_NK-cycling
1957	6.51E-120	0.42225848	0.218	0.01	1.42E-115	WDR34	7	STMN1_NK-cycling
1958	9.25E-102	0.4221069	0.234	0.016	2.02E-97	ATAD5	7	STMN1_NK-cycling

1959	2.65E-28	0.42209083	0.972	0.807	5.77E-24	RAC2	7	STMN1_NK-cycling
1960	5.04E-114	0.42079018	0.254	0.016	1.10E-109	GPSM2	7	STMN1_NK-cycling
1961	6.71E-21	0.41939694	0.73	0.365	1.46E-16	PGAM1	7	STMN1_NK-cycling
1962	1.23E-173	0.41746702	0.218	0.005	2.68E-169	FAM111B	7	STMN1_NK-cycling
1963	1.53E-118	0.41705593	0.234	0.013	3.33E-114	CCNF	7	STMN1_NK-cycling
1964	7.00E-29	0.41702269	0.512	0.19	1.53E-24	CCT5	7	STMN1_NK-cycling
1965	1.20E-24	0.41586845	0.859	0.518	2.62E-20	HLA-DRB1	7	STMN1_NK-cycling
1966	1.78E-138	0.41573272	0.226	0.009	3.89E-134	CDC25C	7	STMN1_NK-cycling
1967	5.30E-25	0.41494475	0.899	0.548	1.16E-20	LDHA	7	STMN1_NK-cycling
1968	1.66E-141	0.41462817	0.258	0.012	3.63E-137	VANGL1	7	STMN1_NK-cycling
1969	2.97E-72	0.41448498	0.254	0.028	6.48E-68	ACOT7	7	STMN1_NK-cycling
1970	3.87E-25	0.41335298	0.726	0.343	8.43E-21	SNRPB	7	STMN1_NK-cycling
1971	1.88E-36	0.4130177	0.395	0.11	4.10E-32	HMGA1	7	STMN1_NK-cycling
1972	5.98E-163	0.41266086	0.246	0.008	1.30E-158	SKA3	7	STMN1_NK-cycling
1973	6.58E-37	0.41231518	0.508	0.16	1.43E-32	CEP57	7	STMN1_NK-cycling
1974	9.38E-19	0.41175031	0.681	0.361	2.05E-14	SYNE2	7	STMN1_NK-cycling
1975	8.98E-23	0.41093905	0.706	0.354	1.96E-18	BSG	7	STMN1_NK-cycling
1976	1.29E-39	0.40983278	0.379	0.096	2.80E-35	SCLT1	7	STMN1_NK-cycling
1977	2.42E-35	0.40911767	0.581	0.198	5.29E-31	TALDO1	7	STMN1_NK-cycling
1978	5.55E-143	0.4081494	0.198	0.006	1.21E-138	DTL	7	STMN1_NK-cycling
1979	1.05E-38	0.40805972	0.823	0.321	2.30E-34	FCGR3A	7	STMN1_NK-cycling
1980	9.24E-58	0.40699935	0.347	0.063	2.02E-53	HADH	7	STMN1_NK-cycling
1981	7.60E-61	0.40622595	0.399	0.076	1.66E-56	VRK1	7	STMN1_NK-cycling
1982	6.33E-48	0.4060431	0.431	0.105	1.38E-43	TPRKB	7	STMN1_NK-cycling
1983	4.33E-124	0.40580594	0.246	0.013	9.45E-120	CBR3	7	STMN1_NK-cycling
1984	3.95E-24	0.40523131	0.778	0.386	8.61E-20	SNRPG	7	STMN1_NK-cycling
1985	1.60E-23	0.40491402	0.597	0.257	3.48E-19	CLTA	7	STMN1_NK-cycling
1986	4.17E-115	0.40451123	0.25	0.016	9.10E-111	KNSTRN	7	STMN1_NK-cycling
1987	8.08E-117	0.4040373	0.254	0.016	1.76E-112	ARHGEF39	7	STMN1_NK-cycling
1988	9.30E-103	0.40385726	0.242	0.017	2.03E-98	KIAA1524	7	STMN1_NK-cycling
1989	5.19E-22	0.40264783	0.669	0.338	1.13E-17	SDCBP	7	STMN1_NK-cycling
1990	3.31E-21	0.40235853	0.895	0.555	7.23E-17	SRSF3	7	STMN1_NK-cycling
1991	6.42E-46	0.40105484	0.347	0.075	1.40E-41	YEATS4	7	STMN1_NK-cycling
1992	3.35E-32	0.39677159	0.633	0.237	7.31E-28	STARD3NL	7	STMN1_NK-cycling
1993	1.71E-23	0.39591834	0.778	0.394	3.73E-19	PTPN6	7	STMN1_NK-cycling
1994	2.06E-23	0.39382027	0.931	0.62	4.49E-19	7-Sep	7	STMN1_NK-cycling
1995	2.59E-21	0.39344449	0.879	0.581	5.65E-17	TMBIM6	7	STMN1_NK-cycling
1996	9.47E-31	0.39269715	0.464	0.154	2.06E-26	HNRNPAB	7	STMN1_NK-cycling
1997	2.14E-65	0.39151188	0.254	0.032	4.66E-61	LRR1	7	STMN1_NK-cycling
1998	1.32E-33	0.3911327	0.431	0.131	2.88E-29	PSMC3	7	STMN1_NK-cycling
1999	1.46E-23	0.39044557	0.968	0.812	3.18E-19	UBB	7	STMN1_NK-cycling
2000	2.17E-27	0.38985182	0.585	0.229	4.72E-23	PSMG2	7	STMN1_NK-cycling
2001	9.22E-107	0.38890396	0.21	0.011	2.01E-102	CHEK2	7	STMN1_NK-cycling
2002	1.99E-20	0.38878687	0.819	0.495	4.34E-16	HNRNPA0	7	STMN1_NK-cycling
2003	6.95E-87	0.3887483	0.222	0.017	1.52E-82	INCENP	7	STMN1_NK-cycling
2004	1.54E-38	0.38736602	0.391	0.102	3.37E-34	SUZ12	7	STMN1_NK-cycling
2005	6.89E-24	0.38660101	0.927	0.733	1.50E-19	MYL12B	7	STMN1_NK-cycling
2006	1.19E-27	0.38556578	0.593	0.238	2.60E-23	PSMA4	7	STMN1_NK-cycling
2007	7.48E-33	0.38554643	0.444	0.139	1.63E-28	AP1S2	7	STMN1_NK-cycling
2008	2.30E-21	0.38361086	0.847	0.457	5.01E-17	ATP5G3	7	STMN1_NK-cycling
2009	2.23E-42	0.38248886	0.355	0.082	4.87E-38	PDS5B	7	STMN1_NK-cycling
2010	2.54E-26	0.3819013	0.524	0.199	5.55E-22	NDUFAF3	7	STMN1_NK-cycling
2011	4.05E-20	0.38146217	0.427	0.172	8.83E-16	ATF3	7	STMN1_NK-cycling
2012	3.81E-49	0.37975621	0.343	0.069	8.31E-45	RANGAP1	7	STMN1_NK-cycling
2013	3.25E-39	0.37954187	0.476	0.136	7.10E-35	C1orf21	7	STMN1_NK-cycling
2014	1.13E-24	0.37867444	0.657	0.29	2.47E-20	SNRPD3	7	STMN1_NK-cycling
2015	7.79E-27	0.37832764	0.581	0.229	1.70E-22	MDH1	7	STMN1_NK-cycling
2016	6.63E-29	0.37803467	0.597	0.229	1.45E-24	NAA38	7	STMN1_NK-cycling
2017	2.11E-50	0.37796982	0.294	0.051	4.60E-46	CTBP2	7	STMN1_NK-cycling
2018	8.73E-36	0.37781784	0.452	0.135	1.90E-31	RTN3	7	STMN1_NK-cycling
2019	5.09E-83	0.37760789	0.181	0.011	1.11E-78	TIMELESS	7	STMN1_NK-cycling
2020	2.46E-22	0.37681382	0.742	0.363	5.37E-18	SRSF10	7	STMN1_NK-cycling
2021	2.05E-33	0.37560409	0.359	0.098	4.47E-29	KIR2DL1	7	STMN1_NK-cycling
2022	4.16E-22	0.37401998	0.698	0.335	9.08E-18	LYAR	7	STMN1_NK-cycling
2023	3.39E-25	0.37395262	0.44	0.159	7.40E-21	SDF2L1	7	STMN1_NK-cycling
2024	1.03E-42	0.37384051	0.367	0.086	2.25E-38	CBFB	7	STMN1_NK-cycling
2025	7.85E-81	0.3736509	0.29	0.033	1.71E-76	CCDC88A	7	STMN1_NK-cycling
2026	4.58E-48	0.37356403	0.315	0.061	9.99E-44	TOPBP1	7	STMN1_NK-cycling
2027	2.76E-59	0.37250362	0.194	0.02	6.03E-55	LMO7	7	STMN1_NK-cycling
2028	8.47E-21	0.3723857	0.79	0.425	1.85E-16	IFI16	7	STMN1_NK-cycling

2029	1.08E-40	0.37203441	0.161	0.021	2.35E-36	HIST1H2AI	7	STMN1_NK-cycling
2030	1.73E-24	0.37091696	0.569	0.236	3.76E-20	PNRC2	7	STMN1_NK-cycling
2031	4.39E-18	0.36972128	0.899	0.633	9.57E-14	C12orf57	7	STMN1_NK-cycling
2032	7.28E-29	0.36968823	0.367	0.113	1.59E-24	POP4	7	STMN1_NK-cycling
2033	2.80E-25	0.36957822	0.617	0.26	6.11E-21	CACYBP	7	STMN1_NK-cycling
2034	5.12E-37	0.36838486	0.419	0.116	1.12E-32	SSRP1	7	STMN1_NK-cycling
2035	1.94E-109	0.36822932	0.23	0.014	4.23E-105	PRR11	7	STMN1_NK-cycling
2036	1.38E-19	0.36710232	0.69	0.344	3.01E-15	SET	7	STMN1_NK-cycling
2037	2.30E-28	0.36647204	0.496	0.177	5.02E-24	FBXL5	7	STMN1_NK-cycling
2038	1.16E-27	0.36520466	0.452	0.157	2.52E-23	FDPS	7	STMN1_NK-cycling
2039	1.15E-24	0.36508476	0.508	0.199	2.51E-20	FDFT1	7	STMN1_NK-cycling
2040	1.01E-87	0.36411839	0.21	0.015	2.19E-83	ZNF367	7	STMN1_NK-cycling
2041	3.01E-39	0.36362095	0.387	0.099	6.57E-35	EXOSC8	7	STMN1_NK-cycling
2042	1.94E-120	0.36289606	0.198	0.008	4.23E-116	PLK4	7	STMN1_NK-cycling
2043	1.42E-36	0.3624987	0.327	0.078	3.11E-32	AKR1C3	7	STMN1_NK-cycling
2044	5.00E-25	0.36240284	0.48	0.181	1.09E-20	MED30	7	STMN1_NK-cycling
2045	2.16E-22	0.36197325	0.516	0.216	4.71E-18	PTBP1	7	STMN1_NK-cycling
2046	1.92E-21	0.36163455	0.403	0.15	4.19E-17	TMEM30A	7	STMN1_NK-cycling
2047	9.53E-20	0.35897368	0.621	0.295	2.08E-15	RBBP4	7	STMN1_NK-cycling
2048	2.38E-142	0.35838118	0.21	0.007	5.18E-138	KIF18B	7	STMN1_NK-cycling
2049	2.13E-21	0.3572993	0.629	0.292	4.65E-17	DSTN	7	STMN1_NK-cycling
2050	1.86E-29	0.35596057	0.403	0.126	4.06E-25	RRP7A	7	STMN1_NK-cycling
2051	4.86E-19	0.35585679	0.637	0.312	1.06E-14	RNF167	7	STMN1_NK-cycling
2052	4.72E-19	0.35474449	0.798	0.43	1.03E-14	YWHAQ	7	STMN1_NK-cycling
2053	7.30E-20	0.35334749	0.94	0.571	1.59E-15	PTGES3	7	STMN1_NK-cycling
2054	4.28E-18	0.35298181	0.831	0.493	9.33E-14	PPP1CA	7	STMN1_NK-cycling
2055	5.92E-22	0.35281803	0.504	0.206	1.29E-17	HNRNPD	7	STMN1_NK-cycling
2056	1.10E-09	0.35214461	0.605	0.387	2.41E-05	AREG	7	STMN1_NK-cycling
2057	3.82E-20	0.35144713	0.649	0.314	8.33E-16	SUN2	7	STMN1_NK-cycling
2058	7.87E-20	0.35048691	0.75	0.37	1.72E-15	ATP5F1	7	STMN1_NK-cycling
2059	7.89E-44	0.35039096	0.343	0.075	1.72E-39	CCDC14	7	STMN1_NK-cycling
2060	5.03E-40	0.34979738	0.423	0.11	1.10E-35	LAT2	7	STMN1_NK-cycling
2061	7.09E-156	0.3494167	0.202	0.005	1.55E-151	RAD51	7	STMN1_NK-cycling
2062	6.18E-28	0.3484619	0.484	0.17	1.35E-23	SUMO3	7	STMN1_NK-cycling
2063	1.08E-22	0.34842692	0.601	0.255	2.35E-18	ANAPC11	7	STMN1_NK-cycling
2064	1.00E-19	0.34750139	0.657	0.318	2.19E-15	PDIA6	7	STMN1_NK-cycling
2065	4.51E-19	0.34739051	0.75	0.396	9.85E-15	PKM	7	STMN1_NK-cycling
2066	9.30E-54	0.34622851	0.278	0.044	2.03E-49	EMC9	7	STMN1_NK-cycling
2067	3.65E-50	0.34565104	0.306	0.056	7.96E-46	CBX1	7	STMN1_NK-cycling
2068	1.38E-38	0.3454324	0.302	0.067	3.00E-34	HDGF	7	STMN1_NK-cycling
2069	6.91E-20	0.34421807	0.722	0.348	1.51E-15	COX5A	7	STMN1_NK-cycling
2070	2.05E-103	0.34295452	0.202	0.011	4.47E-99	RCCD1	7	STMN1_NK-cycling
2071	7.21E-33	0.34282995	0.411	0.122	1.57E-28	GPAA1	7	STMN1_NK-cycling
2072	2.90E-17	0.34264658	0.722	0.37	6.32E-13	P4HB	7	STMN1_NK-cycling
2073	4.23E-22	0.3417933	0.593	0.253	9.24E-18	PTBP3	7	STMN1_NK-cycling
2074	6.31E-16	0.34112447	0.899	0.634	1.38E-11	COX6C	7	STMN1_NK-cycling
2075	1.44E-25	0.34103922	0.633	0.262	3.14E-21	ITGB1	7	STMN1_NK-cycling
2076	4.16E-19	0.34091495	0.766	0.417	9.06E-15	RAC1	7	STMN1_NK-cycling
2077	4.78E-19	0.34076492	0.802	0.435	1.04E-14	ATP5J2	7	STMN1_NK-cycling
2078	2.09E-69	0.340648	0.218	0.021	4.57E-65	DSN1	7	STMN1_NK-cycling
2079	3.26E-137	0.34001909	0.169	0.004	7.11E-133	CDC45	7	STMN1_NK-cycling
2080	1.77E-17	0.33997903	0.665	0.336	3.85E-13	PA2G4	7	STMN1_NK-cycling
2081	9.25E-31	0.33896077	0.476	0.156	2.02E-26	AP1M1	7	STMN1_NK-cycling
2082	1.65E-43	0.33805578	0.323	0.068	3.59E-39	MICB	7	STMN1_NK-cycling
2083	8.20E-19	0.33705027	0.661	0.319	1.79E-14	PSMA6	7	STMN1_NK-cycling
2084	3.75E-41	0.33683459	0.375	0.091	8.17E-37	G2E3	7	STMN1_NK-cycling
2085	8.11E-34	0.33631114	0.375	0.104	1.77E-29	CHRAC1	7	STMN1_NK-cycling
2086	6.71E-25	0.33578249	0.508	0.194	1.46E-20	ITGB1BP1	7	STMN1_NK-cycling
2087	1.04E-126	0.33549777	0.181	0.006	2.27E-122	FBXO43	7	STMN1_NK-cycling
2088	7.40E-104	0.3349337	0.165	0.006	1.61E-99	WDR62	7	STMN1_NK-cycling
2089	3.47E-25	0.33479904	0.383	0.128	7.57E-21	NDE1	7	STMN1_NK-cycling
2090	2.48E-131	0.3347405	0.19	0.006	5.40E-127	SPC24	7	STMN1_NK-cycling
2091	1.22E-48	0.3338976	0.234	0.035	2.65E-44	PALLD	7	STMN1_NK-cycling
2092	5.59E-30	0.33385268	0.565	0.202	1.22E-25	UBE2A	7	STMN1_NK-cycling
2093	2.51E-21	0.333755	0.645	0.302	5.47E-17	LINC00152	7	STMN1_NK-cycling
2094	3.42E-34	0.33298116	0.206	0.038	7.47E-30	MCM6	7	STMN1_NK-cycling
2095	1.07E-33	0.33297255	0.238	0.05	2.33E-29	FGFR1OP	7	STMN1_NK-cycling
2096	2.02E-26	0.33260816	0.633	0.257	4.41E-22	KIF2A	7	STMN1_NK-cycling
2097	1.52E-31	0.33095281	0.306	0.08	3.31E-27	TMEM18	7	STMN1_NK-cycling
2098	2.45E-19	0.33028593	0.528	0.23	5.35E-15	TAF15	7	STMN1_NK-cycling

2099	4.52E-16	0.32957471	0.843	0.533	9.86E-12	HSP90B1	7	STMN1_NK-cycling
2100	4.67E-69	0.32889899	0.173	0.013	1.02E-64	MFGE8	7	STMN1_NK-cycling
2101	2.97E-28	0.32767569	0.375	0.115	6.49E-24	IMPDH2	7	STMN1_NK-cycling
2102	1.10E-47	0.32758432	0.21	0.029	2.41E-43	SLC1A4	7	STMN1_NK-cycling
2103	1.85E-26	0.32641461	0.524	0.194	4.02E-22	PRDX3	7	STMN1_NK-cycling
2104	4.54E-18	0.32633275	0.996	0.91	9.89E-14	MYL12A	7	STMN1_NK-cycling
2105	6.21E-88	0.3252152	0.19	0.012	1.35E-83	RFC3	7	STMN1_NK-cycling
2106	1.74E-33	0.32366741	0.339	0.089	3.80E-29	MPST	7	STMN1_NK-cycling
2107	1.14E-53	0.32330843	0.185	0.02	2.49E-49	DPYSL2	7	STMN1_NK-cycling
2108	2.23E-42	0.32212468	0.177	0.024	4.86E-38	HIST1H2AL	7	STMN1_NK-cycling
2109	9.87E-74	0.32205307	0.185	0.014	2.15E-69	RTKN2	7	STMN1_NK-cycling
2110	1.13E-20	0.32167416	0.637	0.289	2.46E-16	PSMD8	7	STMN1_NK-cycling
2111	4.10E-21	0.32152296	0.399	0.151	8.95E-17	TIPRL	7	STMN1_NK-cycling
2112	6.34E-98	0.32096334	0.185	0.009	1.38E-93	FAM72B	7	STMN1_NK-cycling
2113	5.89E-21	0.32085066	0.532	0.227	1.28E-16	EIF4E	7	STMN1_NK-cycling
2114	1.19E-25	0.32076067	0.472	0.168	2.60E-21	RECQL	7	STMN1_NK-cycling
2115	4.54E-16	0.3207452	0.762	0.413	9.91E-12	EIF5A	7	STMN1_NK-cycling
2116	3.61E-87	0.32064828	0.185	0.011	7.88E-83	CHEK1	7	STMN1_NK-cycling
2117	4.72E-17	0.32044045	0.98	0.821	1.03E-12	GZMA	7	STMN1_NK-cycling
2118	3.45E-39	0.32043065	0.315	0.07	7.51E-35	ANKRD36C	7	STMN1_NK-cycling
2119	9.29E-27	0.32020039	0.359	0.112	2.03E-22	HENMT1	7	STMN1_NK-cycling
2120	3.61E-22	0.32017016	0.593	0.248	7.87E-18	RAB11A	7	STMN1_NK-cycling
2121	6.22E-44	0.31981099	0.246	0.042	1.36E-39	CDCA4	7	STMN1_NK-cycling
2122	5.74E-31	0.31946782	0.319	0.085	1.25E-26	ARMC1	7	STMN1_NK-cycling
2123	2.15E-35	0.31847836	0.363	0.094	4.69E-31	CCDC82	7	STMN1_NK-cycling
2124	2.64E-21	0.31841813	0.69	0.312	5.76E-17	SNRPF	7	STMN1_NK-cycling
2125	1.97E-26	0.31827119	0.367	0.116	4.31E-22	ANXA4	7	STMN1_NK-cycling
2126	1.08E-42	0.31813852	0.206	0.031	2.36E-38	CDK2	7	STMN1_NK-cycling
2127	1.58E-17	0.31794428	0.851	0.495	3.45E-13	RBM8A	7	STMN1_NK-cycling
2128	6.03E-24	0.31693986	0.282	0.083	1.31E-19	NABP2	7	STMN1_NK-cycling
2129	1.18E-49	0.31597298	0.202	0.026	2.57E-45	C19orf48	7	STMN1_NK-cycling
2130	3.13E-64	0.31508513	0.218	0.023	6.84E-60	MIS18A	7	STMN1_NK-cycling
2131	1.27E-18	0.31483332	0.629	0.298	2.78E-14	MYO1G	7	STMN1_NK-cycling
2132	8.22E-69	0.3142841	0.149	0.009	1.79E-64	HIST1H2AH	7	STMN1_NK-cycling
2133	1.81E-30	0.31370427	0.335	0.092	3.94E-26	ATL3	7	STMN1_NK-cycling
2134	6.77E-86	0.31219712	0.198	0.013	1.48E-81	MTFR2	7	STMN1_NK-cycling
2135	2.40E-25	0.31208037	0.379	0.125	5.24E-21	KATNBL1	7	STMN1_NK-cycling
2136	1.66E-57	0.31203933	0.202	0.023	3.61E-53	BORA	7	STMN1_NK-cycling
2137	2.45E-21	0.31195734	0.653	0.286	5.34E-17	MAGOH	7	STMN1_NK-cycling
2138	2.09E-37	0.31123835	0.306	0.07	4.56E-33	ACTL6A	7	STMN1_NK-cycling
2139	4.80E-55	0.3101391	0.206	0.024	1.05E-50	MASTL	7	STMN1_NK-cycling
2140	2.47E-114	0.3099152	0.169	0.006	5.38E-110	TRIP13	7	STMN1_NK-cycling
2141	6.22E-14	0.3097598	0.883	0.596	1.36E-09	TAGLN2	7	STMN1_NK-cycling
2142	1.93E-67	0.30970312	0.181	0.015	4.21E-63	CENPJ	7	STMN1_NK-cycling
2143	2.06E-45	0.3090827	0.262	0.045	4.49E-41	KLRC4	7	STMN1_NK-cycling
2144	1.60E-37	0.30753564	0.21	0.036	3.49E-33	FDXR	7	STMN1_NK-cycling
2145	5.12E-23	0.30720738	0.387	0.136	1.12E-18	SUPT16H	7	STMN1_NK-cycling
2146	2.06E-26	0.30711597	0.435	0.148	4.49E-22	FAM195B	7	STMN1_NK-cycling
2147	3.32E-17	0.30656039	0.46	0.201	7.24E-13	ANXA5	7	STMN1_NK-cycling
2148	5.26E-15	0.30465685	0.754	0.442	1.15E-10	UXT	7	STMN1_NK-cycling
2149	3.10E-62	0.30465344	0.21	0.022	6.76E-58	RNF26	7	STMN1_NK-cycling
2150	2.03E-32	0.30446695	0.298	0.074	4.42E-28	IMMP1L	7	STMN1_NK-cycling
2151	8.17E-20	0.30404131	0.544	0.235	1.78E-15	GTF2A2	7	STMN1_NK-cycling
2152	8.17E-40	0.30389644	0.315	0.069	1.78E-35	ODF2	7	STMN1_NK-cycling
2153	8.02E-23	0.30372445	0.407	0.146	1.75E-18	BAZ1B	7	STMN1_NK-cycling
2154	1.11E-21	0.30274292	0.375	0.135	2.41E-17	NUDCD2	7	STMN1_NK-cycling
2155	7.02E-34	0.30273499	0.294	0.07	1.53E-29	RAD51C	7	STMN1_NK-cycling
2156	4.97E-21	0.30250661	0.411	0.154	1.08E-16	RNASEH2B	7	STMN1_NK-cycling
2157	1.26E-20	0.30249033	0.448	0.177	2.74E-16	NUDT21	7	STMN1_NK-cycling
2158	1.37E-25	0.30231637	0.452	0.158	3.00E-21	VBP1	7	STMN1_NK-cycling
2159	6.12E-16	0.30178956	0.69	0.348	1.33E-11	CSNK2B	7	STMN1_NK-cycling
2160	1.22E-15	0.30092788	0.569	0.275	2.66E-11	HSD17B11	7	STMN1_NK-cycling
2161	8.46E-23	0.30069513	0.492	0.189	1.85E-18	CMC2	7	STMN1_NK-cycling
2162	8.49E-30	0.30029322	0.302	0.08	1.85E-25	CCDC167	7	STMN1_NK-cycling
2163	1.92E-29	0.30020039	0.343	0.098	4.19E-25	COMMD4	7	STMN1_NK-cycling
2164	2.04E-15	0.29939021	0.915	0.6	4.44E-11	COX6A1	7	STMN1_NK-cycling
2165	2.67E-22	0.29765052	0.536	0.215	5.82E-18	AP2S1	7	STMN1_NK-cycling
2166	7.21E-25	0.29741495	0.367	0.12	1.57E-20	STIP1	7	STMN1_NK-cycling
2167	3.37E-24	0.29708377	0.427	0.152	7.34E-20	IFI6	7	STMN1_NK-cycling
2168	5.51E-23	0.29615029	0.573	0.232	1.20E-18	CCT2	7	STMN1_NK-cycling

2169	2.21E-46	0.29605511	0.214	0.031	4.81E-42	DHRS4L2	7	STMN1_NK-cycling
2170	5.22E-15	0.29601386	0.754	0.423	1.14E-10	BAX	7	STMN1_NK-cycling
2171	7.99E-31	0.29591746	0.371	0.107	1.74E-26	NELFE	7	STMN1_NK-cycling
2172	5.28E-52	0.29589104	0.198	0.024	1.15E-47	DRAXIN	7	STMN1_NK-cycling
2173	6.18E-81	0.29586719	0.161	0.009	1.35E-76	SMTN	7	STMN1_NK-cycling
2174	2.88E-14	0.295867	0.871	0.581	6.28E-10	COX7A2	7	STMN1_NK-cycling
2175	1.76E-40	0.29559295	0.27	0.053	3.84E-36	SLFN13	7	STMN1_NK-cycling
2176	6.74E-46	0.2953913	0.258	0.044	1.47E-41	ALDH16A1	7	STMN1_NK-cycling
2177	1.15E-36	0.29518751	0.323	0.076	2.50E-32	TESC	7	STMN1_NK-cycling
2178	1.34E-17	0.29496261	0.661	0.328	2.93E-13	RBX1	7	STMN1_NK-cycling
2179	1.01E-24	0.29482729	0.335	0.105	2.20E-20	IKBIP	7	STMN1_NK-cycling
2180	1.25E-57	0.29402535	0.202	0.022	2.73E-53	DDX11	7	STMN1_NK-cycling
2181	6.93E-57	0.29373146	0.198	0.022	1.51E-52	ARHGAP19	7	STMN1_NK-cycling
2182	1.74E-59	0.29336061	0.141	0.01	3.80E-55	MCM2	7	STMN1_NK-cycling
2183	9.39E-40	0.29320099	0.23	0.041	2.05E-35	RHNO1	7	STMN1_NK-cycling
2184	2.39E-24	0.29308342	0.274	0.078	5.20E-20	KIR3DL2	7	STMN1_NK-cycling
2185	9.02E-33	0.29307474	0.294	0.072	1.97E-28	METTL10	7	STMN1_NK-cycling
2186	7.36E-17	0.29291593	0.649	0.323	1.60E-12	KPNB1	7	STMN1_NK-cycling
2187	5.86E-47	0.29230086	0.19	0.024	1.28E-42	CCP110	7	STMN1_NK-cycling
2188	3.50E-14	0.29204585	0.77	0.454	7.63E-10	PSMA7	7	STMN1_NK-cycling
2189	4.09E-15	0.29189375	0.976	0.801	8.92E-11	CORO1A	7	STMN1_NK-cycling
2190	3.35E-111	0.29132979	0.153	0.004	7.31E-107	RAD54L	7	STMN1_NK-cycling
2191	7.57E-44	0.29094672	0.214	0.033	1.65E-39	NCAPH2	7	STMN1_NK-cycling
2192	8.65E-20	0.28998928	0.399	0.152	1.89E-15	ARL6IP6	7	STMN1_NK-cycling
2193	1.21E-19	0.28974538	0.435	0.173	2.65E-15	ELAVL1	7	STMN1_NK-cycling
2194	1.29E-100	0.28937503	0.19	0.009	2.81E-96	GEN1	7	STMN1_NK-cycling
2195	5.01E-55	0.28930905	0.19	0.021	1.09E-50	POLD1	7	STMN1_NK-cycling
2196	3.75E-85	0.28879483	0.141	0.006	8.17E-81	GINS2	7	STMN1_NK-cycling
2197	5.01E-66	0.28853062	0.202	0.019	1.09E-61	RFC5	7	STMN1_NK-cycling
2198	1.33E-69	0.28811582	0.169	0.012	2.91E-65	CENPO	7	STMN1_NK-cycling
2199	5.37E-26	0.28786555	0.335	0.102	1.17E-21	NCAM1	7	STMN1_NK-cycling
2200	2.66E-80	0.28748075	0.161	0.009	5.81E-76	CIT	7	STMN1_NK-cycling
2201	1.44E-17	0.28733964	0.665	0.326	3.15E-13	NDUFB4	7	STMN1_NK-cycling
2202	1.25E-18	0.28727934	0.528	0.228	2.72E-14	SSNA1	7	STMN1_NK-cycling
2203	4.24E-20	0.28533472	0.536	0.22	9.24E-16	RAB8A	7	STMN1_NK-cycling
2204	1.57E-21	0.2850232	0.524	0.212	3.43E-17	LUC7L2	7	STMN1_NK-cycling
2205	3.98E-31	0.28449949	0.315	0.082	8.69E-27	DESI2	7	STMN1_NK-cycling
2206	2.70E-28	0.28443252	0.298	0.08	5.89E-24	PDLIM1	7	STMN1_NK-cycling
2207	6.88E-62	0.28414343	0.161	0.013	1.50E-57	FANCD2	7	STMN1_NK-cycling
2208	3.52E-49	0.28295731	0.169	0.019	7.69E-45	FANCG	7	STMN1_NK-cycling
2209	2.44E-75	0.2825508	0.157	0.009	5.32E-71	C1orf112	7	STMN1_NK-cycling
2210	4.77E-48	0.28216893	0.181	0.022	1.04E-43	CENPQ	7	STMN1_NK-cycling
2211	4.46E-22	0.28190654	0.46	0.174	9.72E-18	UQCRC1	7	STMN1_NK-cycling
2212	1.33E-15	0.28187493	0.661	0.338	2.90E-11	SFPQ	7	STMN1_NK-cycling
2213	2.09E-32	0.28180621	0.202	0.038	4.56E-28	SLC43A3	7	STMN1_NK-cycling
2214	1.05E-14	0.28152199	0.94	0.775	2.28E-10	PCBP2	7	STMN1_NK-cycling
2215	3.63E-20	0.2815139	0.512	0.209	7.92E-16	SNRPC	7	STMN1_NK-cycling
2216	2.68E-16	0.28129542	0.685	0.342	5.84E-12	ATP5C1	7	STMN1_NK-cycling
2217	2.64E-74	0.28072542	0.194	0.015	5.75E-70	HAUS8	7	STMN1_NK-cycling
2218	4.70E-13	0.28028805	0.927	0.689	1.03E-08	PCBP1	7	STMN1_NK-cycling
2219	5.46E-23	0.28017621	0.448	0.163	1.19E-18	RNASEH2C	7	STMN1_NK-cycling
2220	2.05E-20	0.27990271	0.423	0.163	4.48E-16	TMEM71	7	STMN1_NK-cycling
2221	2.47E-65	0.27972019	0.149	0.01	5.38E-61	CENPP	7	STMN1_NK-cycling
2222	2.26E-65	0.27941327	0.157	0.011	4.93E-61	MCM4	7	STMN1_NK-cycling
2223	2.47E-28	0.27939502	0.254	0.062	5.38E-24	HAUS1	7	STMN1_NK-cycling
2224	2.97E-35	0.27933669	0.206	0.037	6.48E-31	APOLD1	7	STMN1_NK-cycling
2225	3.22E-43	0.27847305	0.165	0.02	7.02E-39	WDR76	7	STMN1_NK-cycling
2226	1.51E-17	0.27845189	0.633	0.301	3.29E-13	RASSF1	7	STMN1_NK-cycling
2227	1.65E-34	0.27775049	0.214	0.039	3.59E-30	TSPAN2	7	STMN1_NK-cycling
2228	7.63E-25	0.27765321	0.379	0.124	1.66E-20	PRPS1	7	STMN1_NK-cycling
2229	3.92E-15	0.27739334	0.613	0.305	8.54E-11	NDUFA12	7	STMN1_NK-cycling
2230	1.19E-23	0.27717327	0.31	0.096	2.59E-19	CDC27	7	STMN1_NK-cycling
2231	3.90E-18	0.27681201	0.577	0.265	8.51E-14	KLRC3	7	STMN1_NK-cycling
2232	3.31E-45	0.27659803	0.226	0.035	7.21E-41	MGME1	7	STMN1_NK-cycling
2233	8.95E-26	0.27652493	0.387	0.125	1.95E-21	CEP78	7	STMN1_NK-cycling
2234	2.03E-37	0.27621306	0.266	0.054	4.44E-33	ASCL2	7	STMN1_NK-cycling
2235	6.27E-76	0.27619836	0.181	0.013	1.37E-71	ATP8B3	7	STMN1_NK-cycling
2236	1.79E-52	0.276096	0.222	0.029	3.90E-48	RP11-545E17.3	7	STMN1_NK-cycling
2237	3.00E-51	0.27599212	0.177	0.02	6.53E-47	ZNF714	7	STMN1_NK-cycling
2238	4.94E-16	0.27596744	0.984	0.858	1.08E-11	HNRNPA1	7	STMN1_NK-cycling

2239	4.53E-13	0.27540293	0.964	0.883	9.87E-09	IFITM2	7	STMN1_NK-cycling
2240	9.19E-20	0.27378029	0.27	0.086	2.00E-15	KIR2DL3	7	STMN1_NK-cycling
2241	5.16E-29	0.27310557	0.246	0.058	1.12E-24	SLC25A40	7	STMN1_NK-cycling
2242	2.26E-12	0.27236686	0.746	0.441	4.92E-08	PSME2	7	STMN1_NK-cycling
2243	2.33E-36	0.27121805	0.222	0.041	5.08E-32	CRAT	7	STMN1_NK-cycling
2244	1.94E-13	0.27108013	0.738	0.429	4.22E-09	ANXA6	7	STMN1_NK-cycling
2245	1.30E-24	0.27062447	0.359	0.114	2.83E-20	RP11-25K19.1	7	STMN1_NK-cycling
2246	1.07E-22	0.27062167	0.383	0.132	2.32E-18	SLC1A5	7	STMN1_NK-cycling
2247	1.96E-16	0.27035719	0.544	0.257	4.28E-12	ZNF706	7	STMN1_NK-cycling
2248	3.24E-15	0.27030929	0.633	0.32	7.08E-11	ADD3	7	STMN1_NK-cycling
2249	5.24E-21	0.27029853	0.456	0.177	1.14E-16	ARPP19	7	STMN1_NK-cycling
2250	3.61E-19	0.2696628	0.46	0.189	7.87E-15	BTG3	7	STMN1_NK-cycling
2251	6.07E-44	0.26857535	0.198	0.028	1.32E-39	XRCC6BP1	7	STMN1_NK-cycling
2252	6.53E-19	0.2680285	0.629	0.29	1.42E-14	GLUL	7	STMN1_NK-cycling
2253	5.53E-41	0.26775415	0.185	0.027	1.21E-36	HYI	7	STMN1_NK-cycling
2254	4.31E-76	0.26754564	0.149	0.008	9.40E-72	DSCC1	7	STMN1_NK-cycling
2255	2.98E-17	0.26752915	0.552	0.25	6.49E-13	NAPA	7	STMN1_NK-cycling
2256	2.21E-49	0.26649464	0.194	0.024	4.83E-45	COQ2	7	STMN1_NK-cycling
2257	2.27E-33	0.26604182	0.234	0.048	4.94E-29	SCD5	7	STMN1_NK-cycling
2258	2.19E-23	0.26600255	0.464	0.17	4.77E-19	U2AF1	7	STMN1_NK-cycling
2259	2.14E-37	0.26593459	0.218	0.039	4.67E-33	TEX30	7	STMN1_NK-cycling
2260	4.02E-28	0.26586539	0.339	0.097	8.77E-24	TMEM14A	7	STMN1_NK-cycling
2261	1.54E-102	0.26526575	0.137	0.004	3.37E-98	MCM10	7	STMN1_NK-cycling
2262	3.66E-23	0.26524094	0.367	0.123	7.97E-19	TFDP2	7	STMN1_NK-cycling
2263	3.85E-13	0.2645646	0.681	0.388	8.40E-09	CAST	7	STMN1_NK-cycling
2264	2.53E-09	0.26450909	0.81	0.536	5.51E-05	CALR	7	STMN1_NK-cycling
2265	6.00E-18	0.26450111	0.516	0.224	1.31E-13	KRAS	7	STMN1_NK-cycling
2266	5.12E-21	0.26446667	0.456	0.175	1.12E-16	FAM96A	7	STMN1_NK-cycling
2267	7.65E-26	0.26435687	0.375	0.119	1.67E-21	MPC1	7	STMN1_NK-cycling
2268	1.33E-40	0.26433968	0.173	0.024	2.90E-36	CEP57L1	7	STMN1_NK-cycling
2269	5.80E-13	0.26431157	0.673	0.362	1.26E-08	UQCRQ	7	STMN1_NK-cycling
2270	4.87E-37	0.26400125	0.185	0.029	1.06E-32	RBBP8	7	STMN1_NK-cycling
2271	3.72E-12	0.26366481	0.891	0.649	8.11E-08	TPM3	7	STMN1_NK-cycling
2272	5.34E-23	0.26320825	0.411	0.144	1.17E-18	DCP2	7	STMN1_NK-cycling
2273	4.88E-22	0.26220621	0.302	0.095	1.06E-17	HPS3	7	STMN1_NK-cycling
2274	7.06E-31	0.26220379	0.274	0.066	1.54E-26	NNT	7	STMN1_NK-cycling
2275	1.71E-30	0.26212271	0.222	0.047	3.74E-26	TUBGCP3	7	STMN1_NK-cycling
2276	7.65E-14	0.26148768	0.641	0.343	1.67E-09	PLAC8	7	STMN1_NK-cycling
2277	6.52E-30	0.26141744	0.141	0.022	1.42E-25	RP11-386G11.1	7	STMN1_NK-cycling
2278	4.76E-36	0.26134958	0.19	0.031	1.04E-31	UBL7-AS1	7	STMN1_NK-cycling
2279	6.86E-22	0.26107757	0.363	0.125	1.50E-17	FAM200B	7	STMN1_NK-cycling
2280	8.06E-20	0.26105949	0.343	0.122	1.76E-15	PHTF2	7	STMN1_NK-cycling
2281	2.40E-22	0.26089053	0.355	0.119	5.23E-18	HCFC1R1	7	STMN1_NK-cycling
2282	4.43E-17	0.26075558	0.508	0.226	9.67E-13	TCP1	7	STMN1_NK-cycling
2283	9.24E-16	0.25983771	0.573	0.268	2.02E-11	POLR2K	7	STMN1_NK-cycling
2284	2.53E-20	0.25926909	0.444	0.17	5.52E-16	MAPRE1	7	STMN1_NK-cycling
2285	1.83E-79	0.25871933	0.149	0.008	3.99E-75	POLQ	7	STMN1_NK-cycling
2286	1.42E-19	0.25867727	0.597	0.257	3.11E-15	UQCRFS1	7	STMN1_NK-cycling
2287	5.42E-12	0.25853815	0.871	0.583	1.18E-07	SLC25A3	7	STMN1_NK-cycling
2288	9.16E-19	0.25845356	0.395	0.153	2.00E-14	CYB5B	7	STMN1_NK-cycling
2289	2.14E-18	0.25829838	0.484	0.202	4.67E-14	SNRPA	7	STMN1_NK-cycling
2290	2.15E-22	0.25822579	0.339	0.112	4.69E-18	DAZAP1	7	STMN1_NK-cycling
2291	8.23E-26	0.25814064	0.274	0.075	1.79E-21	HACD3	7	STMN1_NK-cycling
2292	8.71E-09	0.25810511	0.649	0.416	0.000189999	WSB1	7	STMN1_NK-cycling
2293	7.16E-34	0.25740238	0.161	0.024	1.56E-29	BLM	7	STMN1_NK-cycling
2294	1.11E-17	0.25703925	0.31	0.113	2.42E-13	HLA-DMA	7	STMN1_NK-cycling
2295	1.71E-16	0.25676484	0.46	0.199	3.73E-12	TMX1	7	STMN1_NK-cycling
2296	7.03E-28	0.25661593	0.52	0.18	1.53E-23	SH2D1B	7	STMN1_NK-cycling
2297	7.73E-34	0.25620389	0.262	0.057	1.68E-29	HMGXB4	7	STMN1_NK-cycling
2298	6.93E-75	0.25620293	0.125	0.005	1.51E-70	CDC6	7	STMN1_NK-cycling
2299	3.99E-23	0.25617409	0.25	0.07	8.71E-19	HLTF	7	STMN1_NK-cycling
2300	4.27E-14	0.25609079	0.738	0.401	9.31E-10	SPCS2	7	STMN1_NK-cycling
2301	1.46E-26	0.25560504	0.315	0.09	3.18E-22	PSMA2	7	STMN1_NK-cycling
2302	7.68E-70	0.25515536	0.19	0.016	1.67E-65	SUV39H1	7	STMN1_NK-cycling
2303	1.18E-11	0.25491851	0.718	0.435	2.58E-07	HLA-DPB1	7	STMN1_NK-cycling
2304	6.20E-72	0.25454984	0.145	0.008	1.35E-67	OIP5	7	STMN1_NK-cycling
2305	3.16E-48	0.25449235	0.161	0.017	6.89E-44	CEP85	7	STMN1_NK-cycling
2306	4.15E-24	0.25402837	0.278	0.079	9.04E-20	APLP2	7	STMN1_NK-cycling
2307	1.98E-10	0.25379134	0.911	0.672	4.32E-06	CHCHD2	7	STMN1_NK-cycling
2308	2.84E-15	0.25367	0.387	0.165	6.19E-11	PRKDC	7	STMN1_NK-cycling

2309	8.31E-28	0.25359874	0.202	0.043	1.81E-23	TFDP1	7	STMN1_NK-cycling
2310	3.41E-19	0.25275148	0.351	0.127	7.44E-15	GUSB	7	STMN1_NK-cycling
2311	7.59E-21	0.25242055	0.27	0.084	1.66E-16	TPGS2	7	STMN1_NK-cycling
2312	1.03E-19	0.25160526	0.327	0.114	2.24E-15	RANGRF	7	STMN1_NK-cycling
2313	2.67E-19	0.25114882	0.496	0.202	5.83E-15	NDUFB6	7	STMN1_NK-cycling
2314	2.66E-13	0.25095257	0.984	0.785	5.80E-09	ATP5G2	7	STMN1_NK-cycling
2315	1.61E-20	0.25074911	0.153	0.034	3.51E-16	HIST1H2AG	7	STMN1_NK-cycling
2316	8.31E-14	-0.25614522	0.988	0.981	1.81E-09	RPL21	7	STMN1_NK-cycling
2317	1.37E-16	-0.25827585	1	0.985	2.98E-12	RPL29	7	STMN1_NK-cycling
2318	3.51E-19	-0.26103528	0.996	0.989	7.65E-15	RPS24	7	STMN1_NK-cycling
2319	2.84E-15	-0.26122096	1	0.987	6.20E-11	RPL14	7	STMN1_NK-cycling
2320	1.42E-06	-0.26243424	0.71	0.762	0.030885668	HSP90AB1	7	STMN1_NK-cycling
2321	7.09E-21	-0.2730255	1	0.994	1.55E-16	RPS15	7	STMN1_NK-cycling
2322	1.81E-07	-0.27832986	0.931	0.858	0.003954204	PABPC1	7	STMN1_NK-cycling
2323	3.59E-10	-0.28061544	0.948	0.934	7.82E-06	PTPRC	7	STMN1_NK-cycling
2324	7.02E-08	-0.28530416	0.879	0.798	0.001531702	TOMM7	7	STMN1_NK-cycling
2325	1.26E-22	-0.28768059	1	0.992	2.74E-18	RPL37	7	STMN1_NK-cycling
2326	8.70E-08	-0.28805571	0.871	0.795	0.001896742	RPS4Y1	7	STMN1_NK-cycling
2327	1.27E-08	-0.29432514	0.907	0.841	0.000276601	EEF2	7	STMN1_NK-cycling
2328	2.47E-21	-0.2961694	0.996	0.995	5.40E-17	RPS8	7	STMN1_NK-cycling
2329	2.87E-07	-0.30583471	0.819	0.757	0.006268032	UQCRB	7	STMN1_NK-cycling
2330	7.35E-28	-0.30898434	0.996	0.995	1.60E-23	RPS3A	7	STMN1_NK-cycling
2331	8.34E-15	-0.30921191	0.984	0.955	1.82E-10	RPL13A	7	STMN1_NK-cycling
2332	1.94E-17	-0.31109271	0.988	0.956	4.23E-13	NACA	7	STMN1_NK-cycling
2333	5.82E-10	-0.31230883	0.923	0.865	1.27E-05	EEF1B2	7	STMN1_NK-cycling
2334	6.56E-11	-0.31260833	0.992	0.973	1.43E-06	SRGN	7	STMN1_NK-cycling
2335	7.92E-28	-0.31539754	0.996	0.992	1.73E-23	RPS23	7	STMN1_NK-cycling
2336	1.23E-07	-0.32028291	0.823	0.739	0.002689155	COMMD6	7	STMN1_NK-cycling
2337	4.37E-14	-0.3206417	0.976	0.947	9.52E-10	MT-ND1	7	STMN1_NK-cycling
2338	2.78E-23	-0.32074225	0.996	0.989	6.06E-19	RPL18	7	STMN1_NK-cycling
2339	3.12E-11	-0.32171121	0.94	0.865	6.80E-07	RPS11	7	STMN1_NK-cycling
2340	4.92E-28	-0.32821807	1	0.99	1.07E-23	RPS7	7	STMN1_NK-cycling
2341	3.74E-25	-0.33099995	0.996	0.987	8.15E-21	RPL6	7	STMN1_NK-cycling
2342	2.82E-12	-0.3327193	0.931	0.857	6.16E-08	RPL4	7	STMN1_NK-cycling
2343	4.11E-33	-0.33511616	1	0.998	8.95E-29	RPL28	7	STMN1_NK-cycling
2344	4.12E-20	-0.33701434	0.976	0.971	8.99E-16	RPL8	7	STMN1_NK-cycling
2345	1.63E-11	-0.33760979	0.903	0.837	3.56E-07	ITM2B	7	STMN1_NK-cycling
2346	4.76E-22	-0.33783327	0.988	0.977	1.04E-17	RPL17	7	STMN1_NK-cycling
2347	3.02E-17	-0.33786576	0.988	0.949	6.59E-13	RPS5	7	STMN1_NK-cycling
2348	1.32E-11	-0.34498996	0.847	0.848	2.88E-07	CD3E	7	STMN1_NK-cycling
2349	1.28E-28	-0.35042717	0.996	0.988	2.79E-24	RPL7A	7	STMN1_NK-cycling
2350	6.99E-29	-0.3571068	1	0.987	1.53E-24	RPS4X	7	STMN1_NK-cycling
2351	2.19E-33	-0.35722421	1	0.994	4.78E-29	RPL26	7	STMN1_NK-cycling
2352	7.23E-12	-0.35973233	0.927	0.905	1.58E-07	CCL5	7	STMN1_NK-cycling
2353	2.23E-07	-0.36107345	0.935	0.887	0.004852795	CXCR4	7	STMN1_NK-cycling
2354	1.80E-21	-0.36391055	0.996	0.99	3.93E-17	MT-ND3	7	STMN1_NK-cycling
2355	3.13E-15	-0.3688686	0.98	0.93	6.83E-11	UBC	7	STMN1_NK-cycling
2356	9.71E-12	-0.37294097	0.907	0.902	2.12E-07	TSC22D3	7	STMN1_NK-cycling
2357	3.21E-16	-0.37552841	0.911	0.864	7.00E-12	RPL31	7	STMN1_NK-cycling
2358	7.68E-42	-0.3775658	1	0.998	1.67E-37	RPS15A	7	STMN1_NK-cycling
2359	2.50E-36	-0.37850253	1	0.995	5.45E-32	RPL11	7	STMN1_NK-cycling
2360	5.76E-12	-0.38129044	0.984	0.967	1.26E-07	ZFP36L2	7	STMN1_NK-cycling
2361	2.11E-07	-0.38594135	0.69	0.647	0.004610542	CYTIP	7	STMN1_NK-cycling
2362	3.98E-07	-0.38976959	0.661	0.614	0.008674434	EIF3E	7	STMN1_NK-cycling
2363	2.17E-28	-0.39149999	0.996	0.985	4.74E-24	RPS25	7	STMN1_NK-cycling
2364	9.45E-30	-0.39461765	0.996	0.981	2.06E-25	RPL3	7	STMN1_NK-cycling
2365	9.76E-12	-0.39631323	0.75	0.739	2.13E-07	CD37	7	STMN1_NK-cycling
2366	1.57E-06	-0.39832207	0.641	0.602	0.034315564	RARRES3	7	STMN1_NK-cycling
2367	3.59E-23	-0.39862871	0.972	0.959	7.82E-19	RPL22	7	STMN1_NK-cycling
2368	7.12E-38	-0.40867798	0.992	0.991	1.55E-33	RPLP2	7	STMN1_NK-cycling
2369	9.12E-16	-0.41386007	0.895	0.88	1.99E-11	DDX5	7	STMN1_NK-cycling
2370	7.97E-20	-0.4185913	0.964	0.961	1.74E-15	RPS29	7	STMN1_NK-cycling
2371	9.50E-37	-0.41877294	0.984	0.991	2.07E-32	RPL18A	7	STMN1_NK-cycling
2372	1.92E-38	-0.42414564	0.992	0.992	4.19E-34	RPS14	7	STMN1_NK-cycling
2373	1.98E-51	-0.42752795	1	0.998	4.33E-47	RPL10	7	STMN1_NK-cycling
2374	3.43E-44	-0.4317629	0.992	0.995	7.47E-40	RPL32	7	STMN1_NK-cycling
2375	1.24E-28	-0.43306135	0.98	0.958	2.71E-24	RPL10A	7	STMN1_NK-cycling
2376	5.47E-40	-0.43532177	0.992	0.989	1.19E-35	RPL35A	7	STMN1_NK-cycling
2377	8.37E-31	-0.44046371	0.98	0.977	1.83E-26	RPL36	7	STMN1_NK-cycling
2378	1.80E-08	-0.44061589	0.859	0.806	0.000391885	KLF6	7	STMN1_NK-cycling

2379	2.19E-06	-0.4413904	0.133	0.25	0.047860763	SATB1	7	STMN1_NK-cycling
2380	4.11E-10	-0.44292553	0.883	0.821	8.96E-06	TXNIP	7	STMN1_NK-cycling
2381	1.77E-07	-0.44728939	0.77	0.736	0.003863621	ZFP36L1	7	STMN1_NK-cycling
2382	4.34E-08	-0.46432061	0.524	0.577	0.000945903	ALOX5AP	7	STMN1_NK-cycling
2383	6.62E-49	-0.46437537	1	0.995	1.44E-44	RPS28	7	STMN1_NK-cycling
2384	1.10E-43	-0.46457607	0.992	0.985	2.41E-39	RPS10	7	STMN1_NK-cycling
2385	3.65E-52	-0.47140275	1	0.994	7.97E-48	RPL19	7	STMN1_NK-cycling
2386	3.19E-42	-0.47200677	0.992	0.993	6.95E-38	TPT1	7	STMN1_NK-cycling
2387	6.78E-39	-0.47340314	0.996	0.989	1.48E-34	RPS18	7	STMN1_NK-cycling
2388	2.48E-12	-0.47746013	0.702	0.68	5.40E-08	CD48	7	STMN1_NK-cycling
2389	1.64E-37	-0.48221026	0.988	0.972	3.57E-33	RPL5	7	STMN1_NK-cycling
2390	1.38E-09	-0.48308852	0.536	0.593	3.01E-05	FYN	7	STMN1_NK-cycling
2391	6.44E-44	-0.48426126	0.996	0.989	1.40E-39	RPS13	7	STMN1_NK-cycling
2392	7.37E-36	-0.48849932	0.972	0.956	1.61E-31	RPL24	7	STMN1_NK-cycling
2393	1.76E-15	-0.49408132	0.903	0.917	3.84E-11	IER2	7	STMN1_NK-cycling
2394	2.63E-08	-0.49490663	0.601	0.594	0.000573843	MCL1	7	STMN1_NK-cycling
2395	2.29E-09	-0.50329448	0.371	0.465	4.99E-05	APRT	7	STMN1_NK-cycling
2396	4.53E-55	-0.50467228	0.992	0.997	9.88E-51	RPL39	7	STMN1_NK-cycling
2397	2.11E-06	-0.50596196	0.242	0.329	0.045973229	GIMAP1	7	STMN1_NK-cycling
2398	2.21E-73	-0.51164773	1	1	4.81E-69	EEF1A1	7	STMN1_NK-cycling
2399	5.06E-35	-0.52136444	0.972	0.955	1.10E-30	RPS2	7	STMN1_NK-cycling
2400	2.68E-07	-0.52163954	0.347	0.423	0.005848365	RHOH	7	STMN1_NK-cycling
2401	1.47E-10	-0.52498599	0.427	0.539	3.20E-06	GZMM	7	STMN1_NK-cycling
2402	1.59E-48	-0.52504085	0.992	0.987	3.47E-44	RPL12	7	STMN1_NK-cycling
2403	9.44E-08	-0.5287804	0.359	0.437	0.002059531	PDCD4	7	STMN1_NK-cycling
2404	1.45E-34	-0.53036676	0.972	0.982	3.17E-30	BTG1	7	STMN1_NK-cycling
2405	1.87E-22	-0.53107007	0.915	0.87	4.09E-18	SARAF	7	STMN1_NK-cycling
2406	3.23E-72	-0.53532201	1	0.998	7.05E-68	RPS27A	7	STMN1_NK-cycling
2407	2.33E-08	-0.54368463	0.343	0.427	0.00050705	TC2N	7	STMN1_NK-cycling
2408	6.81E-20	-0.54542298	0.77	0.783	1.48E-15	PNRC1	7	STMN1_NK-cycling
2409	4.42E-34	-0.54612114	0.944	0.933	9.63E-30	RPL9	7	STMN1_NK-cycling
2410	5.61E-26	-0.56162729	0.875	0.878	1.22E-21	HCST	7	STMN1_NK-cycling
2411	3.46E-34	-0.57478342	0.907	0.901	7.55E-30	PFDN5	7	STMN1_NK-cycling
2412	1.05E-32	-0.57515856	0.964	0.969	2.30E-28	FTL	7	STMN1_NK-cycling
2413	1.21E-16	-0.57820485	0.847	0.862	2.63E-12	DUSP2	7	STMN1_NK-cycling
2414	6.01E-11	-0.58616228	0.44	0.521	1.31E-06	PARP8	7	STMN1_NK-cycling
2415	8.50E-70	-0.59097356	0.996	0.997	1.85E-65	RPL13	7	STMN1_NK-cycling
2416	1.17E-26	-0.59231501	0.79	0.872	2.56E-22	IL32	7	STMN1_NK-cycling
2417	4.70E-11	-0.602082	0.274	0.421	1.03E-06	SNHG8	7	STMN1_NK-cycling
2418	1.07E-26	-0.61106201	0.927	0.943	2.33E-22	ZFP36	7	STMN1_NK-cycling
2419	3.42E-70	-0.6146413	0.996	0.997	7.47E-66	RPL34	7	STMN1_NK-cycling
2420	5.43E-41	-0.62847453	0.927	0.922	1.18E-36	RPL41	7	STMN1_NK-cycling
2421	1.20E-15	-0.6286894	0.556	0.648	2.62E-11	CD2	7	STMN1_NK-cycling
2422	1.83E-80	-0.64104724	1	0.996	3.99E-76	RPL30	7	STMN1_NK-cycling
2423	4.01E-79	-0.6623799	1	0.997	8.75E-75	RPS27	7	STMN1_NK-cycling
2424	8.35E-11	-0.66695198	0.649	0.676	1.82E-06	DNAJB1	7	STMN1_NK-cycling
2425	2.17E-12	-0.68446567	0.492	0.598	4.73E-08	CD52	7	STMN1_NK-cycling
2426	1.18E-80	-0.71583396	1	0.997	2.57E-76	RPS12	7	STMN1_NK-cycling
2427	1.05E-25	-0.75719715	0.879	0.888	2.28E-21	DUSP1	7	STMN1_NK-cycling
2428	1.72E-12	-0.80707731	0.149	0.336	3.75E-08	KLRG1	7	STMN1_NK-cycling
2429	7.10E-98	-0.84355868	0.996	0.998	1.55E-93	AC090498.1	7	STMN1_NK-cycling
2430	3.65E-47	-0.85241771	0.899	0.965	7.97E-43	JUNB	7	STMN1_NK-cycling
2431	6.66E-32	-0.93893814	0.883	0.902	1.45E-27	CD69	7	STMN1_NK-cycling
2432	4.84E-18	-0.99680765	0.19	0.434	1.06E-13	CD8A	7	STMN1_NK-cycling
2433	5.02E-31	-1.06542397	0.137	0.522	1.09E-26	CD3D	7	STMN1_NK-cycling
2434	2.37E-15	-1.18690654	0.552	0.612	5.18E-11	KLRB1	7	STMN1_NK-cycling
2435	5.22E-53	1.11037219	0.743	0.294	1.14E-48	XCL2	8	XCL2_NK
2436	1.79E-59	1.02855882	0.912	0.568	3.89E-55	CMC1	8	XCL2_NK
2437	2.52E-64	0.9643816	0.748	0.26	5.50E-60	KLRC3	8	XCL2_NK
2438	2.66E-42	0.8049556	0.774	0.362	5.80E-38	KLRF1	8	XCL2_NK
2439	2.12E-49	0.77980588	0.907	0.451	4.61E-45	TYROBP	8	XCL2_NK
2440	2.62E-27	0.7340273	0.535	0.226	5.71E-23	XCL1	8	XCL2_NK
2441	1.90E-24	0.73352807	0.425	0.172	4.14E-20	KIR3DL1	8	XCL2_NK
2442	8.38E-45	0.70209614	0.283	0.055	1.83E-40	GCSAM	8	XCL2_NK
2443	6.87E-25	0.6827679	0.429	0.171	1.50E-20	TRDC	8	XCL2_NK
2444	1.53E-19	0.60908844	0.69	0.48	3.33E-15	PIK3R1	8	XCL2_NK
2445	2.73E-25	0.59228249	0.327	0.11	5.95E-21	APBA2	8	XCL2_NK
2446	1.37E-26	0.57799028	0.81	0.529	2.98E-22	GSTP1	8	XCL2_NK
2447	1.42E-22	0.56429138	0.788	0.501	3.10E-18	TRBC1	8	XCL2_NK
2448	5.05E-19	0.54761917	0.394	0.172	1.10E-14	TIGIT	8	XCL2_NK

2449	6.72E-31	0.53931383	0.938	0.559	1.47E-26	KLRD1	8	XCL2_NK
2450	4.00E-12	0.49487374	0.69	0.448	8.73E-08	CCL3	8	XCL2_NK
2451	2.40E-13	0.49130155	0.367	0.183	5.24E-09	CRTAM	8	XCL2_NK
2452	1.52E-16	0.48542579	0.186	0.056	3.32E-12	AC069363.1	8	XCL2_NK
2453	1.05E-34	0.48317632	0.996	0.903	2.29E-30	CCL5	8	XCL2_NK
2454	2.78E-10	0.47905795	0.248	0.118	6.06E-06	LAT2	8	XCL2_NK
2455	8.30E-15	0.47700868	0.239	0.088	1.81E-10	KIR2DL3	8	XCL2_NK
2456	1.10E-23	0.47307085	0.929	0.735	2.41E-19	CD7	8	XCL2_NK
2457	6.37E-19	0.45722462	0.429	0.185	1.39E-14	HLA-DQA1	8	XCL2_NK
2458	1.06E-11	0.45174193	0.434	0.258	2.30E-07	MAPK1	8	XCL2_NK
2459	5.26E-35	0.45108799	0.146	0.019	1.15E-30	SPRY2	8	XCL2_NK
2460	1.22E-10	0.44887741	0.327	0.176	2.66E-06	TRG-AS1	8	XCL2_NK
2461	4.91E-13	0.43458455	0.376	0.188	1.07E-08	FCRL6	8	XCL2_NK
2462	1.95E-09	0.42949737	0.341	0.194	4.26E-05	CD160	8	XCL2_NK
2463	2.26E-11	0.42658053	0.668	0.545	4.94E-07	STK17A	8	XCL2_NK
2464	1.81E-15	0.42011781	0.212	0.072	3.95E-11	KLRC2	8	XCL2_NK
2465	1.96E-08	0.41004862	0.482	0.341	0.000426731	AKIRIN2	8	XCL2_NK
2466	7.79E-12	0.39213218	0.133	0.04	1.70E-07	SYK	8	XCL2_NK
2467	1.25E-17	0.38765013	0.124	0.026	2.72E-13	SPECC1	8	XCL2_NK
2468	8.95E-22	0.38557516	0.996	0.882	1.95E-17	IFITM2	8	XCL2_NK
2469	7.47E-10	0.37682646	0.376	0.215	1.63E-05	AOAH	8	XCL2_NK
2470	2.61E-07	0.36675112	0.212	0.109	0.005684346	PLCG2	8	XCL2_NK
2471	2.93E-08	0.36154395	0.204	0.096	0.000638615	A1BG	8	XCL2_NK
2472	1.70E-08	0.34883835	0.381	0.23	0.000371611	GNPTAB	8	XCL2_NK
2473	4.24E-10	0.34670136	0.115	0.035	9.25E-06	KIR2DL4	8	XCL2_NK
2474	1.38E-07	0.33940296	0.27	0.15	0.003016682	CD300A	8	XCL2_NK
2475	1.70E-07	0.33656094	0.681	0.571	0.003714284	ALOX5AP	8	XCL2_NK
2476	4.80E-08	0.33493916	0.571	0.443	0.001047711	APMAP	8	XCL2_NK
2477	6.27E-07	0.33271188	0.54	0.43	0.013677659	PDCD4	8	XCL2_NK
2478	2.55E-07	0.32797592	0.478	0.351	0.005571106	C19orf60	8	XCL2_NK
2479	5.72E-07	0.321213	0.46	0.333	0.012484375	IL2RB	8	XCL2_NK
2480	1.11E-07	0.30909	0.181	0.082	0.002424511	KIR3DL2	8	XCL2_NK
2481	1.63E-07	0.30560783	0.412	0.262	0.003563432	HLA-DRB5	8	XCL2_NK
2482	8.49E-07	0.30328297	0.199	0.101	0.018506064	ABCB1	8	XCL2_NK
2483	1.64E-06	0.30098102	0.195	0.101	0.035762656	RCBTB2	8	XCL2_NK
2484	6.10E-07	0.29775321	0.177	0.085	0.013311804	NCR1	8	XCL2_NK
2485	2.33E-07	0.27659644	0.588	0.437	0.005089521	HLA-DPA1	8	XCL2_NK
2486	1.65E-14	0.26295838	0.973	0.947	3.60E-10	IFITM1	8	XCL2_NK
2487	2.09E-07	0.25759844	0.695	0.526	0.004551742	HLA-DRB1	8	XCL2_NK
2488	5.75E-10	0.25007529	0.938	0.876	1.25E-05	HCST	8	XCL2_NK
2489	1.69E-06	-0.25446009	0.58	0.722	0.036830611	LAPTM5	8	XCL2_NK
2490	1.15E-09	-0.32808767	0.841	0.911	2.51E-05	SH3BGRL3	8	XCL2_NK
2491	1.09E-06	-0.37267781	0.288	0.462	0.023780779	ARL4C	8	XCL2_NK
2492	9.12E-08	-0.38288536	0.314	0.514	0.001987983	LDHB	8	XCL2_NK
2493	3.74E-10	-0.39320571	0.248	0.48	8.16E-06	LIMD2	8	XCL2_NK
2494	1.25E-08	-0.42192986	0.425	0.611	0.000272144	EMP3	8	XCL2_NK
2495	3.06E-08	-0.44138196	0.19	0.386	0.000666481	UCP2	8	XCL2_NK
2496	6.01E-11	-0.49814127	0.451	0.671	1.31E-06	CRIP1	8	XCL2_NK
2497	7.18E-11	-0.51050183	0.115	0.333	1.57E-06	SELPLG	8	XCL2_NK
2498	1.80E-11	-0.5254131	0.642	0.792	3.93E-07	NFKBIA	8	XCL2_NK
2499	1.14E-07	-0.52710408	0.111	0.267	0.002479563	COTL1	8	XCL2_NK
2500	8.11E-13	-0.57695081	0.265	0.511	1.77E-08	S100A11	8	XCL2_NK
2501	1.16E-20	-0.63344026	0.611	0.801	2.53E-16	S100A6	8	XCL2_NK
2502	1.63E-15	-0.70946021	0.115	0.376	3.56E-11	SPOCK2	8	XCL2_NK
2503	1.09E-13	-0.75102184	0.102	0.337	2.39E-09	KLRG1	8	XCL2_NK
2504	2.55E-09	-0.80648739	0.19	0.375	5.57E-05	LGALS1	8	XCL2_NK
2505	1.73E-22	-0.85130241	0.226	0.582	3.77E-18	TNFAIP3	8	XCL2_NK
2506	5.60E-08	-0.97298159	0.553	0.683	0.001220429	MT2A	8	XCL2_NK
2507	2.22E-19	-0.97513056	0.133	0.435	4.84E-15	CD8A	8	XCL2_NK
2508	2.90E-51	-1.09607554	0.575	0.879	6.32E-47	S100A4	8	XCL2_NK
2509	4.55E-31	-1.12077138	0.102	0.522	9.92E-27	CD3D	8	XCL2_NK
2510	1.84E-33	-1.13224097	0.177	0.611	4.01E-29	CD52	8	XCL2_NK
2511	2.47E-141	2.30271846	0.929	0.216	5.39E-137	XCL1	9	XCL1_NK
2512	2.01E-235	1.93473494	0.94	0.116	4.39E-231	FCER1G	9	XCL1_NK
2513	9.14E-82	1.6993531	0.88	0.293	1.99E-77	XCL2	9	XCL1_NK
2514	3.70E-72	1.55897592	0.923	0.38	8.07E-68	AREG	9	XCL1_NK
2515	7.86E-99	1.52038604	0.902	0.319	1.71E-94	IL2RB	9	XCL1_NK
2516	3.94E-53	1.37094734	0.896	0.443	8.60E-49	CCL3	9	XCL1_NK
2517	3.60E-72	1.14933642	0.628	0.141	7.85E-68	KLRC1	9	XCL1_NK
2518	5.22E-75	1.10306569	0.989	0.735	1.14E-70	CD7	9	XCL1_NK

2519	1.13E-91	1.0832884	0.53	0.085	2.46E-87	IRF8	9	XCL1_NK
2520	5.60E-61	1.04419131	0.65	0.185	1.22E-56	CD160	9	XCL1_NK
2521	5.89E-58	1.04256937	0.951	0.57	1.28E-53	CMC1	9	XCL1_NK
2522	1.16E-45	1.00838602	0.699	0.259	2.53E-41	CLIC3	9	XCL1_NK
2523	1.12E-53	0.99709191	0.913	0.527	2.45E-49	GSTP1	9	XCL1_NK
2524	4.65E-78	0.9824016	0.541	0.101	1.01E-73	CD38	9	XCL1_NK
2525	1.02E-63	0.96189587	0.918	0.361	2.23E-59	KLRF1	9	XCL1_NK
2526	3.03E-57	0.92998932	0.973	0.452	6.60E-53	TYROBP	9	XCL1_NK
2527	6.60E-56	0.88952335	0.574	0.15	1.44E-51	TMIGD2	9	XCL1_NK
2528	1.85E-33	0.88325566	0.475	0.154	4.04E-29	ICAM1	9	XCL1_NK
2529	5.05E-50	0.86464546	0.683	0.227	1.10E-45	TXK	9	XCL1_NK
2530	1.44E-32	0.83880629	0.705	0.343	3.14E-28	CLDND1	9	XCL1_NK
2531	2.76E-41	0.82876298	0.579	0.184	6.02E-37	YES1	9	XCL1_NK
2532	2.39E-47	0.80033605	0.541	0.146	5.21E-43	MCTP2	9	XCL1_NK
2533	1.73E-54	0.77604608	0.301	0.044	3.77E-50	GSTM2	9	XCL1_NK
2534	3.99E-56	0.7658277	0.503	0.111	8.71E-52	LAT2	9	XCL1_NK
2535	5.77E-27	0.75104441	0.781	0.479	1.26E-22	PIK3R1	9	XCL1_NK
2536	2.37E-43	0.74703997	0.989	0.597	5.18E-39	KLRB1	9	XCL1_NK
2537	1.09E-41	0.73316545	0.984	0.561	2.38E-37	KLRD1	9	XCL1_NK
2538	1.01E-44	0.68625744	0.437	0.102	2.21E-40	PLCG2	9	XCL1_NK
2539	3.58E-136	0.68373809	0.257	0.01	7.80E-132	LDB2	9	XCL1_NK
2540	7.48E-44	0.68171554	0.995	0.973	1.63E-39	SRGN	9	XCL1_NK
2541	2.71E-28	0.66906872	0.552	0.211	5.90E-24	AOAH	9	XCL1_NK
2542	3.75E-33	0.6685841	0.437	0.128	8.18E-29	RAB11FIP1	9	XCL1_NK
2543	3.15E-50	0.6610975	0.399	0.078	6.86E-46	NCR1	9	XCL1_NK
2544	8.52E-36	0.65608325	0.301	0.063	1.86E-31	SPRY1	9	XCL1_NK
2545	5.52E-45	0.65481299	1	0.946	1.20E-40	IFITM1	9	XCL1_NK
2546	9.44E-42	0.64510204	0.607	0.182	2.06E-37	SH2D1B	9	XCL1_NK
2547	1.02E-27	0.6349526	0.35	0.102	2.23E-23	TLE1	9	XCL1_NK
2548	1.58E-88	0.62355744	0.246	0.017	3.45E-84	TOX2	9	XCL1_NK
2549	8.93E-25	0.62017463	0.71	0.385	1.95E-20	CD63	9	XCL1_NK
2550	1.42E-07	0.60519426	0.546	0.368	0.00310006	CCL3L3	9	XCL1_NK
2551	3.79E-148	0.60444827	0.24	0.007	8.26E-144	IL18	9	XCL1_NK
2552	5.04E-23	0.60419861	0.656	0.317	1.10E-18	ARHGAP9	9	XCL1_NK
2553	3.42E-21	0.59812097	0.825	0.567	7.45E-17	ALOX5AP	9	XCL1_NK
2554	7.07E-30	0.59491938	0.574	0.214	1.54E-25	IFITM3	9	XCL1_NK
2555	4.09E-19	0.5841227	0.437	0.172	8.91E-15	TRDC	9	XCL1_NK
2556	1.57E-30	0.56973094	0.328	0.082	3.43E-26	CLASP1	9	XCL1_NK
2557	1.22E-20	0.55715702	0.623	0.323	2.66E-16	MATK	9	XCL1_NK
2558	1.74E-48	0.55658861	0.262	0.037	3.80E-44	SYK	9	XCL1_NK
2559	1.35E-215	0.55020765	0.202	0.001	2.94E-211	BCO2	9	XCL1_NK
2560	1.09E-17	0.54732524	0.956	0.827	2.37E-13	FOS	9	XCL1_NK
2561	1.02E-44	0.54720951	0.251	0.036	2.22E-40	GSN	9	XCL1_NK
2562	1.03E-14	0.54323937	0.923	0.781	2.24E-10	NFKBIA	9	XCL1_NK
2563	5.55E-184	0.54060518	0.219	0.003	1.21E-179	ADGRG3	9	XCL1_NK
2564	3.60E-20	0.53502194	0.798	0.542	7.86E-16	STK17A	9	XCL1_NK
2565	1.93E-14	0.53376197	0.355	0.153	4.21E-10	CEBPD	9	XCL1_NK
2566	7.41E-102	0.53308612	0.213	0.01	1.62E-97	SPTSSB	9	XCL1_NK
2567	3.73E-16	0.5294277	0.246	0.079	8.13E-12	MIR222HG	9	XCL1_NK
2568	7.43E-13	0.52863434	0.825	0.687	1.62E-08	NR4A2	9	XCL1_NK
2569	1.29E-92	0.52788054	0.219	0.012	2.82E-88	SERPINE2	9	XCL1_NK
2570	2.76E-13	0.52757444	0.634	0.403	6.02E-09	DENND2D	9	XCL1_NK
2571	1.48E-25	0.52533806	0.344	0.099	3.23E-21	RASA1	9	XCL1_NK
2572	1.03E-18	0.52255263	0.842	0.577	2.25E-14	EIF3G	9	XCL1_NK
2573	4.45E-24	0.52078739	0.344	0.104	9.70E-20	NCAM1	9	XCL1_NK
2574	3.06E-14	0.51728788	0.656	0.4	6.67E-10	MAP3K8	9	XCL1_NK
2575	1.01E-14	0.51503532	0.322	0.128	2.21E-10	MAFF	9	XCL1_NK
2576	1.96E-25	0.49662829	0.355	0.103	4.28E-21	PTPN12	9	XCL1_NK
2577	2.27E-30	0.49589582	0.262	0.055	4.94E-26	SLFN13	9	XCL1_NK
2578	2.06E-91	0.49470865	0.191	0.009	4.48E-87	ADAMTS17	9	XCL1_NK
2579	2.03E-14	0.4915414	0.454	0.218	4.43E-10	HELZ	9	XCL1_NK
2580	3.73E-16	0.49048678	0.29	0.103	8.14E-12	DUSP10	9	XCL1_NK
2581	1.42E-14	0.48963796	0.454	0.212	3.09E-10	TOX	9	XCL1_NK
2582	3.98E-17	0.4868375	0.743	0.455	8.68E-13	CTSD	9	XCL1_NK
2583	1.32E-12	0.48105315	0.486	0.258	2.88E-08	MAPK1	9	XCL1_NK
2584	1.91E-14	0.47714596	0.361	0.151	4.16E-10	CXCR6	9	XCL1_NK
2585	3.23E-07	0.47714045	0.579	0.423	0.007037616	NFKBIZ	9	XCL1_NK
2586	4.61E-15	0.47142586	0.973	0.875	1.01E-10	CCL4	9	XCL1_NK
2587	7.00E-22	0.46834415	0.355	0.114	1.53E-17	DHRS3	9	XCL1_NK
2588	3.75E-17	0.4655654	0.776	0.493	8.18E-13	LY6E	9	XCL1_NK

2589	8.90E-13	0.46082862	0.612	0.352	1.94E-08	BST2	9	XCL1_NK
2590	4.11E-57	0.4599787	0.23	0.023	8.95E-53	ATP8B4	9	XCL1_NK
2591	1.82E-17	0.45844622	0.404	0.159	3.96E-13	HSH2D	9	XCL1_NK
2592	1.75E-13	0.45631558	0.361	0.158	3.82E-09	TCIRG1	9	XCL1_NK
2593	9.74E-23	0.45602501	0.88	0.45	2.12E-18	GZMK	9	XCL1_NK
2594	1.10E-13	0.45546764	0.328	0.135	2.40E-09	KAT6B	9	XCL1_NK
2595	8.01E-25	0.44957799	0.984	0.884	1.75E-20	IFITM2	9	XCL1_NK
2596	1.67E-12	0.44856687	0.563	0.32	3.64E-08	SASH3	9	XCL1_NK
2597	4.73E-15	0.44569549	0.306	0.115	1.03E-10	DUSP6	9	XCL1_NK
2598	2.06E-14	0.44195368	0.355	0.151	4.49E-10	MPG	9	XCL1_NK
2599	2.16E-36	0.44084739	0.164	0.019	4.71E-32	SPRY2	9	XCL1_NK
2600	4.18E-09	0.44069386	0.585	0.39	9.11E-05	REL	9	XCL1_NK
2601	2.92E-20	0.43951382	0.301	0.092	6.38E-16	COLQ	9	XCL1_NK
2602	9.82E-17	0.43517217	0.202	0.056	2.14E-12	GRAMD3	9	XCL1_NK
2603	1.23E-08	0.43472397	0.475	0.282	0.000269225	FAM46C	9	XCL1_NK
2604	3.10E-13	0.43251662	0.699	0.44	6.76E-09	APMAP	9	XCL1_NK
2605	3.01E-15	0.42896237	0.492	0.234	6.56E-11	OFD1	9	XCL1_NK
2606	8.73E-15	0.42778969	0.393	0.163	1.90E-10	FGR	9	XCL1_NK
2607	1.87E-29	0.42606383	0.197	0.033	4.08E-25	KIR2DL4	9	XCL1_NK
2608	5.69E-11	0.42531862	0.639	0.412	1.24E-06	TMEM2	9	XCL1_NK
2609	7.90E-20	0.4211006	0.257	0.072	1.72E-15	SLC16A3	9	XCL1_NK
2610	5.54E-12	0.41906414	0.361	0.166	1.21E-07	SESN1	9	XCL1_NK
2611	1.63E-13	0.41667957	0.322	0.132	3.56E-09	ZBTB16	9	XCL1_NK
2612	2.10E-19	0.41538625	0.355	0.12	4.58E-15	GFOD1	9	XCL1_NK
2613	4.61E-36	0.41494927	0.186	0.025	1.01E-31	SPECC1	9	XCL1_NK
2614	8.87E-16	0.41393568	0.295	0.105	1.93E-11	FASLG	9	XCL1_NK
2615	4.35E-14	0.41304794	0.443	0.205	9.48E-10	PTGDR	9	XCL1_NK
2616	9.37E-12	0.4122382	0.459	0.232	2.04E-07	EOMES	9	XCL1_NK
2617	1.62E-12	0.40604786	0.339	0.146	3.54E-08	PCMTD1	9	XCL1_NK
2618	2.42E-12	0.40549513	0.694	0.443	5.28E-08	CD96	9	XCL1_NK
2619	4.12E-18	0.40487586	0.339	0.118	8.99E-14	SKAP2	9	XCL1_NK
2620	3.84E-14	0.40181539	0.809	0.583	8.37E-10	FYN	9	XCL1_NK
2621	6.07E-12	0.40119987	0.579	0.341	1.32E-07	FKBP11	9	XCL1_NK
2622	3.66E-11	0.40065215	0.454	0.243	7.97E-07	VPS37B	9	XCL1_NK
2623	2.08E-13	0.39742151	0.295	0.114	4.53E-09	BAZ2B	9	XCL1_NK
2624	1.12E-12	0.39617922	0.377	0.171	2.45E-08	CD244	9	XCL1_NK
2625	1.40E-34	0.39574242	0.175	0.023	3.05E-30	P2RY11	9	XCL1_NK
2626	9.13E-16	0.39570585	0.301	0.108	1.99E-11	RUNX2	9	XCL1_NK
2627	2.96E-08	0.39257316	0.383	0.211	0.000646207	GATA3	9	XCL1_NK
2628	4.34E-17	0.39227561	0.23	0.066	9.46E-13	CAPN12	9	XCL1_NK
2629	1.04E-09	0.39001915	0.372	0.187	2.26E-05	INPP5D	9	XCL1_NK
2630	5.16E-16	0.38777501	0.383	0.15	1.13E-11	YARS	9	XCL1_NK
2631	1.05E-21	0.38709654	0.191	0.041	2.29E-17	LZTFL1	9	XCL1_NK
2632	1.12E-11	0.384162	0.262	0.105	2.44E-07	TMEM141	9	XCL1_NK
2633	3.67E-23	0.38321558	0.18	0.035	8.00E-19	SLC44A1	9	XCL1_NK
2634	8.28E-15	0.38284027	0.235	0.075	1.81E-10	ERGIC1	9	XCL1_NK
2635	1.28E-12	0.37653069	0.311	0.126	2.79E-08	GSAP	9	XCL1_NK
2636	2.39E-13	0.37630022	0.175	0.051	5.21E-09	HDAC9	9	XCL1_NK
2637	5.88E-19	0.37487227	0.191	0.046	1.28E-14	MIR181A1HG	9	XCL1_NK
2638	4.02E-10	0.37249327	0.694	0.503	8.77E-06	ATP6V0C	9	XCL1_NK
2639	3.12E-09	0.37239534	0.623	0.406	6.80E-05	SLC38A1	9	XCL1_NK
2640	1.90E-14	0.37008042	0.88	0.597	4.13E-10	CD247	9	XCL1_NK
2641	2.01E-11	0.36550834	0.667	0.396	4.37E-07	CFLAR	9	XCL1_NK
2642	1.70E-10	0.36294486	0.475	0.256	3.70E-06	SYNRG	9	XCL1_NK
2643	4.65E-10	0.36212495	0.301	0.137	1.01E-05	PDE7A	9	XCL1_NK
2644	9.86E-12	0.36199528	0.585	0.328	2.15E-07	PRDX5	9	XCL1_NK
2645	1.92E-12	0.35854571	0.295	0.119	4.18E-08	CASP3	9	XCL1_NK
2646	4.63E-13	0.35817368	0.219	0.072	1.01E-08	CLASP2	9	XCL1_NK
2647	9.15E-10	0.35621112	0.404	0.205	2.00E-05	ADAM8	9	XCL1_NK
2648	4.70E-09	0.3556852	0.366	0.19	0.000102557	ACAA2	9	XCL1_NK
2649	7.19E-11	0.35473225	0.672	0.426	1.57E-06	PDCD4	9	XCL1_NK
2650	1.21E-27	0.35413685	0.137	0.018	2.64E-23	CHKA	9	XCL1_NK
2651	8.19E-12	0.35249516	0.29	0.119	1.79E-07	WDSUB1	9	XCL1_NK
2652	8.42E-11	0.35205196	0.508	0.273	1.84E-06	CHST12	9	XCL1_NK
2653	1.20E-09	0.35174065	0.23	0.095	2.61E-05	MPST	9	XCL1_NK
2654	7.28E-09	0.35046247	0.355	0.181	0.000158725	NR4A3	9	XCL1_NK
2655	1.60E-06	0.34913046	0.776	0.64	0.034895518	PPP1R15A	9	XCL1_NK
2656	1.38E-09	0.34645756	0.246	0.104	3.01E-05	LPAR6	9	XCL1_NK
2657	5.17E-09	0.34608462	0.219	0.09	0.00011269	FNDC3B	9	XCL1_NK
2658	1.57E-11	0.34554294	0.306	0.128	3.42E-07	DGKD	9	XCL1_NK

2659	2.61E-11	0.3446531	0.268	0.106	5.70E-07	MIB2	9	XCL1_NK
2660	1.15E-08	0.34463634	0.497	0.302	0.000250516	ECH1	9	XCL1_NK
2661	1.41E-08	0.34445151	0.743	0.519	0.000306928	SYTL3	9	XCL1_NK
2662	7.99E-08	0.34327889	0.443	0.264	0.001742965	PIM2	9	XCL1_NK
2663	2.22E-11	0.33871426	0.612	0.353	4.84E-07	RGS1	9	XCL1_NK
2664	5.29E-08	0.33647637	0.459	0.278	0.001153084	PPP2R1A	9	XCL1_NK
2665	1.39E-07	0.33584067	0.273	0.137	0.003020768	ITCH	9	XCL1_NK
2666	1.73E-09	0.3354331	0.421	0.22	3.77E-05	NDUFS8	9	XCL1_NK
2667	3.09E-14	0.3351457	0.219	0.068	6.74E-10	DZIP3	9	XCL1_NK
2668	1.62E-09	0.33482873	0.284	0.128	3.53E-05	TIPARP	9	XCL1_NK
2669	4.02E-09	0.33386362	0.454	0.259	8.76E-05	NAP1L4	9	XCL1_NK
2670	2.79E-07	0.33265759	0.475	0.299	0.006084182	PNN	9	XCL1_NK
2671	2.79E-12	0.33245602	0.284	0.111	6.08E-08	S100PBP	9	XCL1_NK
2672	3.82E-13	0.33050741	0.235	0.079	8.33E-09	RP11-1000B6.3	9	XCL1_NK
2673	2.86E-11	0.33000416	0.301	0.127	6.23E-07	RIN3	9	XCL1_NK
2674	3.11E-07	0.32980525	0.432	0.264	0.006788131	IL16	9	XCL1_NK
2675	2.05E-09	0.32901278	0.443	0.24	4.48E-05	RNF125	9	XCL1_NK
2676	1.50E-07	0.32844148	0.694	0.53	0.003272458	GZMM	9	XCL1_NK
2677	6.12E-11	0.32763635	0.208	0.075	1.34E-06	FHL3	9	XCL1_NK
2678	1.11E-18	0.32566317	0.388	0.138	2.42E-14	TRGV9	9	XCL1_NK
2679	2.45E-11	0.32472128	0.268	0.105	5.34E-07	ELL2	9	XCL1_NK
2680	1.24E-07	0.32257283	0.464	0.276	0.002703543	NDUFV1	9	XCL1_NK
2681	9.16E-09	0.31956746	0.557	0.331	0.000199725	ITGA4	9	XCL1_NK
2682	8.41E-10	0.3168953	0.191	0.07	1.83E-05	MMP25-AS1	9	XCL1_NK
2683	1.28E-14	0.31239936	0.186	0.052	2.79E-10	CXXC5	9	XCL1_NK
2684	3.77E-08	0.31216988	0.372	0.198	0.000822156	MYBL1	9	XCL1_NK
2685	9.48E-07	0.31097935	0.497	0.33	0.020677671	RHOC	9	XCL1_NK
2686	7.68E-07	0.31040744	0.601	0.413	0.016745766	ETS1	9	XCL1_NK
2687	5.57E-07	0.3097489	0.432	0.262	0.012139135	GYG1	9	XCL1_NK
2688	1.36E-06	0.3093565	0.328	0.185	0.02965535	PDE4B	9	XCL1_NK
2689	3.90E-08	0.30712371	0.76	0.575	0.000849549	JAK1	9	XCL1_NK
2690	1.19E-07	0.30633905	0.617	0.421	0.002599402	DDX17	9	XCL1_NK
2691	1.77E-06	0.30423772	0.41	0.249	0.038500018	STARD3NL	9	XCL1_NK
2692	4.91E-08	0.30294271	0.361	0.19	0.001070831	LPCAT1	9	XCL1_NK
2693	2.26E-07	0.30263544	0.459	0.273	0.004930482	KLRC3	9	XCL1_NK
2694	1.01E-11	0.30262861	0.235	0.084	2.21E-07	PDLIM1	9	XCL1_NK
2695	3.58E-24	0.30013492	1	0.999	7.81E-20	MT-CO3	9	XCL1_NK
2696	1.46E-06	0.30008345	0.35	0.202	0.031749383	SMARCA2	9	XCL1_NK
2697	4.29E-08	0.29977452	0.322	0.164	0.000935575	TNFRSF1A	9	XCL1_NK
2698	1.75E-14	0.29975087	0.158	0.04	3.81E-10	RHOBTB3	9	XCL1_NK
2699	2.80E-08	0.29915693	0.311	0.153	0.000611473	LYN	9	XCL1_NK
2700	2.24E-06	0.2989165	0.355	0.209	0.048770685	RAP1GDS1	9	XCL1_NK
2701	9.69E-12	0.29783062	0.208	0.071	2.11E-07	UBASH3B	9	XCL1_NK
2702	2.94E-07	0.29758353	0.404	0.229	0.006407292	AGTRAP	9	XCL1_NK
2703	1.20E-06	0.2960298	0.404	0.25	0.026240906	METTL9	9	XCL1_NK
2704	1.03E-07	0.29557842	0.984	0.941	0.002245164	ZFP36	9	XCL1_NK
2705	1.62E-24	0.29526826	0.137	0.02	3.54E-20	SIGLEC7	9	XCL1_NK
2706	1.13E-07	0.29501553	0.197	0.084	0.002459935	TRIB2	9	XCL1_NK
2707	1.82E-09	0.29426533	0.328	0.155	3.96E-05	PARVG	9	XCL1_NK
2708	1.12E-10	0.29300055	0.18	0.06	2.44E-06	ACP5	9	XCL1_NK
2709	3.82E-11	0.29274953	0.262	0.102	8.34E-07	CLPTM1	9	XCL1_NK
2710	2.85E-07	0.29243101	0.399	0.229	0.006224729	CPNE1	9	XCL1_NK
2711	2.66E-08	0.2895942	1	0.898	0.000581023	CD69	9	XCL1_NK
2712	1.86E-07	0.28948894	0.268	0.132	0.004047658	B3GAT3	9	XCL1_NK
2713	5.63E-11	0.2877052	0.131	0.036	1.23E-06	IRS2	9	XCL1_NK
2714	5.57E-08	0.28761166	0.689	0.492	0.001214301	WIPF1	9	XCL1_NK
2715	1.28E-14	0.28676565	0.142	0.033	2.78E-10	HIP1	9	XCL1_NK
2716	1.41E-08	0.28517105	0.23	0.099	0.000308321	HIPK2	9	XCL1_NK
2717	2.04E-11	0.28334754	0.224	0.08	4.44E-07	BEX4	9	XCL1_NK
2718	3.56E-08	0.2832035	0.464	0.264	0.0007764	NUCB2	9	XCL1_NK
2719	3.33E-32	0.2817519	0.104	0.009	7.26E-28	B3GNT7	9	XCL1_NK
2720	7.44E-08	0.28144147	0.164	0.063	0.001622902	ABHD2	9	XCL1_NK
2721	1.82E-07	0.28131004	0.317	0.163	0.003976174	LPIN1	9	XCL1_NK
2722	1.69E-09	0.2806396	0.186	0.068	3.68E-05	TBXAS1	9	XCL1_NK
2723	2.25E-13	0.28062896	0.973	0.857	4.91E-09	PABPC1	9	XCL1_NK
2724	4.64E-07	0.27836491	0.415	0.237	0.010119184	SLC44A2	9	XCL1_NK
2725	1.11E-06	0.27832263	0.208	0.097	0.024190561	DPP8	9	XCL1_NK
2726	1.07E-09	0.27810032	0.268	0.114	2.33E-05	ECI2	9	XCL1_NK
2727	3.85E-07	0.27718096	0.164	0.067	0.008388919	ABCG1	9	XCL1_NK
2728	3.37E-09	0.27671969	0.169	0.06	7.36E-05	COG2	9	XCL1_NK

2729	2.71E-83	0.27646992	0.109	0.002	5.92E-79	SMIM24	9	XCL1_NK
2730	3.75E-11	0.27563277	0.175	0.056	8.18E-07	STAM	9	XCL1_NK
2731	4.23E-09	0.2746918	0.301	0.139	9.23E-05	RCHY1	9	XCL1_NK
2732	1.38E-08	0.27411421	0.235	0.102	0.0003019	TWISTNB	9	XCL1_NK
2733	4.42E-10	0.2737239	0.175	0.06	9.65E-06	MMD	9	XCL1_NK
2734	1.33E-06	0.27314768	0.208	0.098	0.028919901	DNM2	9	XCL1_NK
2735	9.13E-11	0.27256934	0.23	0.085	1.99E-06	C1orf162	9	XCL1_NK
2736	1.29E-06	0.2720802	0.781	0.634	0.028138884	TAGAP	9	XCL1_NK
2737	2.98E-07	0.27154833	0.169	0.069	0.006500376	CTDP1	9	XCL1_NK
2738	1.18E-18	0.27112701	0.131	0.023	2.58E-14	GNAQ	9	XCL1_NK
2739	8.78E-07	0.27099318	0.18	0.08	0.019152294	SNX10	9	XCL1_NK
2740	7.40E-11	0.27046204	0.148	0.044	1.61E-06	LIMK1	9	XCL1_NK
2741	8.10E-07	0.26805793	0.284	0.149	0.017671867	SKIL	9	XCL1_NK
2742	5.15E-07	0.26781419	0.175	0.074	0.011220717	KLRC2	9	XCL1_NK
2743	4.09E-08	0.26732581	0.929	0.778	0.000890996	PNRC1	9	XCL1_NK
2744	1.51E-06	0.26429393	0.508	0.321	0.032821144	PPP1R12A	9	XCL1_NK
2745	7.44E-07	0.26423683	0.246	0.122	0.01621786	HDAC7	9	XCL1_NK
2746	1.13E-08	0.26095438	0.235	0.101	0.00024682	ABCB1	9	XCL1_NK
2747	6.61E-07	0.25985568	0.186	0.081	0.014405074	EI24	9	XCL1_NK
2748	4.89E-11	0.25902924	0.169	0.053	1.07E-06	OSBPL2	9	XCL1_NK
2749	1.05E-11	0.25735887	0.164	0.049	2.28E-07	ITGA6	9	XCL1_NK
2750	1.84E-09	0.25715087	0.142	0.045	4.02E-05	CCDC28B	9	XCL1_NK
2751	1.87E-19	0.25592809	0.126	0.021	4.09E-15	MGST2	9	XCL1_NK
2752	7.46E-07	0.253736	0.661	0.468	0.016268241	ARPC5L	9	XCL1_NK
2753	6.28E-28	0.25245707	0.126	0.015	1.37E-23	FBP1	9	XCL1_NK
2754	2.25E-07	0.25181532	0.191	0.082	0.004910215	AGK	9	XCL1_NK
2755	2.66E-07	0.25062008	0.12	0.042	0.005811051	GPD1L	9	XCL1_NK
2756	2.75E-10	0.25044368	0.197	0.069	5.99E-06	FAM49A	9	XCL1_NK
2757	3.00E-12	-0.25455876	0.995	0.995	6.54E-08	RPL11	9	XCL1_NK
2758	1.45E-06	-0.25947867	0.918	0.934	0.031644726	RPL9	9	XCL1_NK
2759	2.43E-12	-0.25979802	0.995	0.997	5.30E-08	RPL34	9	XCL1_NK
2760	4.26E-13	-0.26204548	0.995	0.995	9.29E-09	RPL32	9	XCL1_NK
2761	5.15E-10	-0.26367595	0.934	0.959	1.12E-05	UBA52	9	XCL1_NK
2762	2.50E-13	-0.26626843	0.995	0.998	5.45E-09	AC090498.1	9	XCL1_NK
2763	2.17E-11	-0.27525571	0.967	0.979	4.74E-07	RPL23A	9	XCL1_NK
2764	1.21E-10	-0.27731185	0.973	0.987	2.63E-06	RPS21	9	XCL1_NK
2765	4.78E-12	-0.28995663	0.978	0.987	1.04E-07	RPL12	9	XCL1_NK
2766	2.94E-19	-0.29126146	1	0.994	6.41E-15	RPS3	9	XCL1_NK
2767	4.54E-07	-0.29302435	0.956	0.963	0.009901722	FTH1	9	XCL1_NK
2768	4.87E-11	-0.29522311	0.989	0.998	1.06E-06	RPS19	9	XCL1_NK
2769	3.95E-17	-0.29644229	1	0.996	8.62E-13	RPL30	9	XCL1_NK
2770	6.79E-16	-0.29943064	0.989	0.993	1.48E-11	RPS14	9	XCL1_NK
2771	2.59E-08	-0.3012167	0.896	0.913	0.000564235	HSPA8	9	XCL1_NK
2772	4.21E-13	-0.32429737	0.956	0.978	9.19E-09	RPL36	9	XCL1_NK
2773	1.59E-16	-0.32437026	0.989	0.997	3.48E-12	RPS27	9	XCL1_NK
2774	1.68E-09	-0.32863524	0.973	0.969	3.66E-05	FTL	9	XCL1_NK
2775	5.42E-38	-0.3380903	1	1	1.18E-33	B2M	9	XCL1_NK
2776	4.96E-15	-0.34380486	0.989	0.993	1.08E-10	TMSB10	9	XCL1_NK
2777	5.83E-09	-0.36647237	0.568	0.707	0.0001272	FXYD5	9	XCL1_NK
2778	7.19E-20	-0.38200541	0.962	0.989	1.57E-15	RPL14	9	XCL1_NK
2779	1.44E-30	-0.39526711	0.995	0.998	3.15E-26	RPL28	9	XCL1_NK
2780	8.60E-20	-0.39780314	0.956	0.972	1.87E-15	RPS16	9	XCL1_NK
2781	6.67E-21	-0.39998799	0.978	0.986	1.46E-16	RPS25	9	XCL1_NK
2782	2.98E-09	-0.40381794	0.628	0.74	6.51E-05	RPS20	9	XCL1_NK
2783	3.48E-25	-0.4075367	0.989	0.997	7.59E-21	RPS12	9	XCL1_NK
2784	4.19E-07	-0.41152217	0.634	0.705	0.009134097	S100A10	9	XCL1_NK
2785	1.40E-15	-0.44481442	0.803	0.898	3.05E-11	RPL38	9	XCL1_NK
2786	1.28E-06	-0.44557624	0.104	0.261	0.027812303	FAM65B	9	XCL1_NK
2787	1.78E-13	-0.44752707	0.749	0.852	3.89E-09	CD3E	9	XCL1_NK
2788	2.87E-12	-0.44889274	0.661	0.778	6.26E-08	PPDPF	9	XCL1_NK
2789	2.88E-07	-0.45194034	0.344	0.506	0.006278044	S100A11	9	XCL1_NK
2790	1.10E-18	-0.4708049	0.907	0.963	2.40E-14	RPS29	9	XCL1_NK
2791	2.40E-07	-0.47601665	0.246	0.419	0.005224257	EZR	9	XCL1_NK
2792	1.83E-08	-0.476314	0.301	0.497	0.000398566	PPP2R5C	9	XCL1_NK
2793	1.18E-07	-0.48034667	0.246	0.406	0.002572312	CAST	9	XCL1_NK
2794	5.98E-08	-0.5040213	0.18	0.359	0.001304751	ANXA2	9	XCL1_NK
2795	5.97E-12	-0.5267034	0.393	0.653	1.30E-07	CD2	9	XCL1_NK
2796	6.96E-11	-0.60284329	0.246	0.462	1.52E-06	ARL4C	9	XCL1_NK
2797	9.96E-10	-0.60465063	0.257	0.449	2.17E-05	HLA-DPA1	9	XCL1_NK
2798	2.77E-10	-0.61499872	0.18	0.382	6.04E-06	PRDX1	9	XCL1_NK

2799	4.41E-10	-0.62957578	0.355	0.538	9.62E-06	HLA-DRB1	9	XCL1_NK
2800	6.86E-10	-0.63663744	0.164	0.363	1.50E-05	C12orf75	9	XCL1_NK
2801	2.65E-08	-0.63792511	0.142	0.328	0.00057765	BATF	9	XCL1_NK
2802	4.66E-16	-0.65365531	0.432	0.651	1.02E-11	C12orf57	9	XCL1_NK
2803	5.12E-11	-0.6594139	0.158	0.381	1.12E-06	SYNE2	9	XCL1_NK
2804	1.18E-10	-0.66254355	0.153	0.369	2.57E-06	FLNA	9	XCL1_NK
2805	5.89E-27	-0.67178679	0.426	0.882	1.28E-22	IL32	9	XCL1_NK
2806	3.28E-11	-0.81707958	0.115	0.335	7.15E-07	KLRG1	9	XCL1_NK
2807	5.23E-15	-0.82396825	0.191	0.455	1.14E-10	HLA-DPB1	9	XCL1_NK
2808	2.99E-09	-0.82929068	0.219	0.43	6.53E-05	TNF	9	XCL1_NK
2809	2.69E-39	-0.85450312	0.76	0.913	5.87E-35	SH3BGRL3	9	XCL1_NK
2810	1.16E-22	-0.89215426	0.279	0.615	2.53E-18	EMP3	9	XCL1_NK
2811	3.51E-12	-0.95440498	0.224	0.448	7.65E-08	GZMB	9	XCL1_NK
2812	3.45E-11	-0.99566733	0.137	0.349	7.53E-07	FCGR3A	9	XCL1_NK
2813	2.34E-25	-1.04079461	0.328	0.673	5.10E-21	CRIP1	9	XCL1_NK
2814	5.80E-24	-1.05238155	0.322	0.663	1.27E-19	ANXA1	9	XCL1_NK
2815	9.23E-37	-1.13513088	0.486	0.834	2.01E-32	VIM	9	XCL1_NK
2816	8.00E-19	-1.13996882	0.24	0.534	1.75E-14	GZMH	9	XCL1_NK
2817	1.99E-06	-1.2540128	0.175	0.316	0.043334318	MT1X	9	XCL1_NK
2818	7.58E-16	-1.44288	0.464	0.685	1.65E-11	MT2A	9	XCL1_NK
2819	5.39E-58	-1.52338244	0.235	0.811	1.18E-53	S100A6	9	XCL1_NK
2820	6.24E-62	-1.59096226	0.437	0.881	1.36E-57	S100A4	9	XCL1_NK
2821	3.79E-12	-1.84227394	0.175	0.389	8.27E-08	GNLY	9	XCL1_NK
2822	2.61E-121	2.02932585	0.988	0.309	5.69E-117	STMN1	10	STMN1_T-cycling
2823	0	1.98067443	0.876	0.041	0	UBE2C	10	STMN1_T-cycling
2824	1.18E-120	1.95676688	0.988	0.322	2.57E-116	HMGB2	10	STMN1_T-cycling
2825	0	1.95083471	0.888	0.039	0	ASPM	10	STMN1_T-cycling
2826	0	1.91727119	0.863	0.05	0	MKI67	10	STMN1_T-cycling
2827	0	1.87987245	0.832	0.04	0	CENPF	10	STMN1_T-cycling
2828	3.52E-98	1.82063631	0.994	0.462	7.67E-94	TUBB	10	STMN1_T-cycling
2829	0	1.81281885	0.839	0.043	0	TOP2A	10	STMN1_T-cycling
2830	6.10E-91	1.69250799	1	0.573	1.33E-86	TUBA1B	10	STMN1_T-cycling
2831	7.00E-59	1.6388547	0.857	0.361	1.53E-54	HIST1H4C	10	STMN1_T-cycling
2832	0	1.60455653	0.851	0.051	0	NUSAP1	10	STMN1_T-cycling
2833	8.73E-74	1.59860923	0.932	0.451	1.90E-69	ARL6IP1	10	STMN1_T-cycling
2834	4.44E-84	1.49968904	0.988	0.538	9.68E-80	HMGN2	10	STMN1_T-cycling
2835	0	1.46680602	0.789	0.037	0	TPX2	10	STMN1_T-cycling
2836	1.07E-197	1.41487246	0.863	0.097	2.34E-193	SMC4	10	STMN1_T-cycling
2837	1.97E-283	1.3511674	0.658	0.033	4.29E-279	CDC20	10	STMN1_T-cycling
2838	0	1.33510093	0.683	0.029	0	HMMR	10	STMN1_T-cycling
2839	4.20E-291	1.30791239	0.683	0.034	9.16E-287	CDK1	10	STMN1_T-cycling
2840	1.45E-79	1.30084732	0.994	0.818	3.16E-75	VIM	10	STMN1_T-cycling
2841	0	1.29419099	0.764	0.034	0	BIRC5	10	STMN1_T-cycling
2842	2.58E-97	1.26666753	0.938	0.289	5.62E-93	H2AFV	10	STMN1_T-cycling
2843	8.70E-164	1.25991662	0.565	0.046	1.90E-159	RRM2	10	STMN1_T-cycling
2844	1.71E-213	1.22764213	0.776	0.069	3.72E-209	CKS1B	10	STMN1_T-cycling
2845	2.65E-167	1.22587031	0.571	0.045	5.78E-163	TYMS	10	STMN1_T-cycling
2846	2.27E-135	1.22379378	0.826	0.131	4.95E-131	PTTG1	10	STMN1_T-cycling
2847	3.77E-91	1.21395119	0.857	0.214	8.22E-87	TUBA1C	10	STMN1_T-cycling
2848	0	1.19179137	0.652	0.027	0	CCNA2	10	STMN1_T-cycling
2849	1.16E-277	1.1889403	0.652	0.033	2.54E-273	CDKN3	10	STMN1_T-cycling
2850	7.15E-263	1.15025468	0.658	0.036	1.56E-258	PRC1	10	STMN1_T-cycling
2851	1.46E-298	1.14923804	0.646	0.029	3.19E-294	TROAP	10	STMN1_T-cycling
2852	2.32E-177	1.14194743	0.727	0.072	5.05E-173	KIF20B	10	STMN1_T-cycling
2853	0	1.13969552	0.64	0.026	0	CCNB2	10	STMN1_T-cycling
2854	3.06E-91	1.09431846	0.733	0.144	6.68E-87	KPNA2	10	STMN1_T-cycling
2855	8.60E-263	1.09052119	0.621	0.031	1.88E-258	KIFC1	10	STMN1_T-cycling
2856	3.34E-250	1.08683203	0.59	0.029	7.29E-246	CASC5	10	STMN1_T-cycling
2857	2.06E-56	1.08532253	0.969	0.609	4.50E-52	H2AFZ	10	STMN1_T-cycling
2858	2.05E-246	1.08018964	0.553	0.026	4.47E-242	CCNB1	10	STMN1_T-cycling
2859	9.73E-220	1.07963263	0.764	0.062	2.12E-215	MAD2L1	10	STMN1_T-cycling
2860	6.72E-65	1.06884477	0.87	0.307	1.46E-60	NUCKS1	10	STMN1_T-cycling
2861	1.98E-254	1.05286323	0.584	0.028	4.31E-250	DLGAP5	10	STMN1_T-cycling
2862	1.52E-266	1.04570325	0.64	0.033	3.32E-262	CDCA8	10	STMN1_T-cycling
2863	1.01E-150	1.02867694	0.708	0.081	2.20E-146	RP11-620J15.3	10	STMN1_T-cycling
2864	4.50E-91	1.02764446	0.752	0.147	9.80E-87	TMPO	10	STMN1_T-cycling
2865	9.01E-58	1.01984195	0.888	0.405	1.97E-53	ANP32B	10	STMN1_T-cycling
2866	3.34E-272	1.00389194	0.559	0.023	7.28E-268	KIF14	10	STMN1_T-cycling
2867	5.44E-239	0.99618298	0.534	0.025	1.19E-234	CDCA3	10	STMN1_T-cycling
2868	2.64E-39	0.99005593	0.863	0.411	5.76E-35	TNF	10	STMN1_T-cycling

2869	1.24E-59	0.989322	0.894	0.361	2.70E-55	CALM3	10	STMN1_T-cycling
2870	5.51E-174	0.97176408	0.646	0.056	1.20E-169	CKAP2	10	STMN1_T-cycling
2871	3.88E-71	0.97066497	0.938	0.316	8.47E-67	HP1BP3	10	STMN1_T-cycling
2872	1.59E-133	0.97008754	0.739	0.097	3.46E-129	KIF22	10	STMN1_T-cycling
2873	2.38E-239	0.96475039	0.615	0.034	5.20E-235	NUF2	10	STMN1_T-cycling
2874	6.20E-58	0.9618931	0.901	0.353	1.35E-53	RAD21	10	STMN1_T-cycling
2875	3.87E-228	0.9548554	0.509	0.023	8.43E-224	PLK1	10	STMN1_T-cycling
2876	2.43E-234	0.93763375	0.584	0.031	5.31E-230	GTSE1	10	STMN1_T-cycling
2877	5.02E-262	0.93569902	0.559	0.024	1.10E-257	HJURP	10	STMN1_T-cycling
2878	3.01E-51	0.9228408	0.646	0.17	6.55E-47	ATF3	10	STMN1_T-cycling
2879	3.66E-103	0.92133802	0.689	0.107	7.98E-99	LMNB1	10	STMN1_T-cycling
2880	3.37E-55	0.92051078	0.665	0.177	7.35E-51	PTMS	10	STMN1_T-cycling
2881	2.85E-246	0.91605521	0.54	0.024	6.21E-242	CENPE	10	STMN1_T-cycling
2882	2.67E-194	0.91438582	0.59	0.04	5.82E-190	RACGAP1	10	STMN1_T-cycling
2883	1.48E-44	0.91147068	0.925	0.71	3.23E-40	CALM2	10	STMN1_T-cycling
2884	1.97E-258	0.90977521	0.547	0.023	4.29E-254	KIF23	10	STMN1_T-cycling
2885	1.16E-100	0.89737276	0.646	0.096	2.53E-96	SPTBN1	10	STMN1_T-cycling
2886	3.11E-159	0.8949482	0.609	0.054	6.78E-155	SMC2	10	STMN1_T-cycling
2887	8.90E-247	0.89421059	0.528	0.023	1.94E-242	CDCA2	10	STMN1_T-cycling
2888	1.49E-62	0.86155978	0.801	0.222	3.25E-58	PLP2	10	STMN1_T-cycling
2889	6.50E-49	0.85556362	0.981	0.818	1.42E-44	HMGB1	10	STMN1_T-cycling
2890	1.89E-161	0.85531812	0.491	0.034	4.12E-157	SGOL2	10	STMN1_T-cycling
2891	1.26E-122	0.85326742	0.602	0.07	2.74E-118	CKAP5	10	STMN1_T-cycling
2892	4.05E-49	0.84436783	0.814	0.288	8.84E-45	CKS2	10	STMN1_T-cycling
2893	1.18E-48	0.84298095	0.82	0.288	2.58E-44	HN1	10	STMN1_T-cycling
2894	3.18E-74	0.83453191	0.665	0.135	6.94E-70	MZT1	10	STMN1_T-cycling
2895	7.23E-241	0.81875202	0.516	0.022	1.58E-236	DEPDC1B	10	STMN1_T-cycling
2896	3.55E-59	0.80943249	0.714	0.185	7.74E-55	CDC25B	10	STMN1_T-cycling
2897	2.69E-113	0.80520607	0.553	0.063	5.87E-109	NCAPD2	10	STMN1_T-cycling
2898	4.54E-263	0.80274056	0.441	0.013	9.89E-259	KIF20A	10	STMN1_T-cycling
2899	7.98E-177	0.79522691	0.491	0.03	1.74E-172	CCDC34	10	STMN1_T-cycling
2900	9.63E-43	0.79106597	0.77	0.279	2.10E-38	DCTN3	10	STMN1_T-cycling
2901	7.55E-130	0.79097653	0.429	0.032	1.65E-125	KIAA0101	10	STMN1_T-cycling
2902	1.21E-154	0.78779488	0.379	0.02	2.65E-150	CDCA5	10	STMN1_T-cycling
2903	2.49E-39	0.78303532	0.814	0.34	5.44E-35	ANXA2	10	STMN1_T-cycling
2904	2.61E-199	0.77746709	0.472	0.023	5.68E-195	NCAPG	10	STMN1_T-cycling
2905	2.53E-45	0.77379166	0.776	0.256	5.52E-41	NUCB2	10	STMN1_T-cycling
2906	3.93E-34	0.76755409	0.82	0.357	8.56E-30	DEK	10	STMN1_T-cycling
2907	2.62E-51	0.76326323	0.739	0.216	5.71E-47	ANP32E	10	STMN1_T-cycling
2908	6.64E-103	0.76203776	0.453	0.047	1.45E-98	CENPM	10	STMN1_T-cycling
2909	1.36E-186	0.75745746	0.441	0.022	2.96E-182	BUB1	10	STMN1_T-cycling
2910	5.35E-42	0.7568637	0.708	0.228	1.17E-37	RHEB	10	STMN1_T-cycling
2911	9.39E-59	0.74871648	0.702	0.176	2.05E-54	YWHAH	10	STMN1_T-cycling
2912	1.43E-197	0.74283033	0.472	0.024	3.13E-193	AURKB	10	STMN1_T-cycling
2913	7.82E-192	0.74144201	0.46	0.023	1.71E-187	CKAP2L	10	STMN1_T-cycling
2914	8.15E-130	0.73564825	0.348	0.02	1.78E-125	UHRF1	10	STMN1_T-cycling
2915	6.14E-36	0.73520797	0.901	0.526	1.34E-31	RAN	10	STMN1_T-cycling
2916	2.52E-58	0.72804088	0.391	0.06	5.50E-54	ATAD2	10	STMN1_T-cycling
2917	1.04E-143	0.72752666	0.491	0.038	2.27E-139	UBE2T	10	STMN1_T-cycling
2918	1.19E-167	0.72653484	0.46	0.028	2.61E-163	ARHGAP11A	10	STMN1_T-cycling
2919	4.32E-31	0.72600105	0.801	0.367	9.42E-27	H2AFX	10	STMN1_T-cycling
2920	1.97E-96	0.72108702	0.528	0.067	4.29E-92	CD59	10	STMN1_T-cycling
2921	7.03E-43	0.71860786	0.988	0.941	1.53E-38	GAPDH	10	STMN1_T-cycling
2922	1.46E-106	0.71404172	0.584	0.073	3.19E-102	TACC3	10	STMN1_T-cycling
2923	2.12E-179	0.71364129	0.429	0.021	4.63E-175	KIF2C	10	STMN1_T-cycling
2924	1.74E-24	0.71341693	0.292	0.071	3.81E-20	CCL20	10	STMN1_T-cycling
2925	2.59E-34	0.70934584	0.901	0.531	5.65E-30	COX8A	10	STMN1_T-cycling
2926	2.91E-131	0.70812951	0.41	0.029	6.35E-127	ASF1B	10	STMN1_T-cycling
2927	2.21E-34	0.70691514	0.932	0.526	4.82E-30	TUBB4B	10	STMN1_T-cycling
2928	1.09E-110	0.70054568	0.398	0.033	2.38E-106	AURKA	10	STMN1_T-cycling
2929	3.02E-56	0.69653868	0.64	0.149	6.58E-52	CARHSP1	10	STMN1_T-cycling
2930	1.76E-245	0.69074309	0.497	0.019	3.84E-241	SGOL1	10	STMN1_T-cycling
2931	9.81E-25	0.68771862	0.298	0.074	2.14E-20	TRBV6-1	10	STMN1_T-cycling
2932	9.30E-84	0.68421179	0.41	0.047	2.03E-79	EZH2	10	STMN1_T-cycling
2933	4.08E-46	0.68398813	0.714	0.215	8.89E-42	LSM5	10	STMN1_T-cycling
2934	8.70E-111	0.68152198	0.329	0.022	1.90E-106	PKMYT1	10	STMN1_T-cycling
2935	2.98E-182	0.67865353	0.435	0.021	6.49E-178	KIF11	10	STMN1_T-cycling
2936	1.36E-27	0.67182836	0.404	0.114	2.97E-23	PCNA	10	STMN1_T-cycling
2937	3.53E-189	0.67151078	0.441	0.021	7.71E-185	CENPW	10	STMN1_T-cycling
2938	1.35E-134	0.6629313	0.391	0.025	2.93E-130	MXD3	10	STMN1_T-cycling

2939	1.85E-156	0.6591154	0.441	0.027	4.04E-152	PARPBP	10	STMN1_T-cycling
2940	1.40E-50	0.65526838	0.634	0.157	3.05E-46	LSM4	10	STMN1_T-cycling
2941	4.56E-172	0.65401321	0.385	0.017	9.95E-168	SPC25	10	STMN1_T-cycling
2942	2.83E-180	0.65238605	0.385	0.016	6.17E-176	GPSM2	10	STMN1_T-cycling
2943	1.62E-100	0.64865895	0.497	0.056	3.53E-96	DTYMK	10	STMN1_T-cycling
2944	3.21E-194	0.64727934	0.398	0.016	6.99E-190	NEK2	10	STMN1_T-cycling
2945	7.59E-13	0.64663867	0.646	0.365	1.66E-08	CCL3L3	10	STMN1_T-cycling
2946	3.52E-133	0.6457662	0.379	0.024	7.67E-129	HYI	10	STMN1_T-cycling
2947	1.10E-167	0.64395592	0.41	0.021	2.39E-163	ECT2	10	STMN1_T-cycling
2948	1.36E-50	0.63398138	0.559	0.124	2.96E-46	CNTRL	10	STMN1_T-cycling
2949	3.53E-93	0.63367772	0.447	0.049	7.69E-89	BARD1	10	STMN1_T-cycling
2950	8.45E-44	0.63166758	0.677	0.192	1.84E-39	MZT2B	10	STMN1_T-cycling
2951	3.34E-61	0.63113635	0.596	0.119	7.29E-57	MIS18BP1	10	STMN1_T-cycling
2952	1.97E-82	0.63110984	0.478	0.063	4.30E-78	PHF19	10	STMN1_T-cycling
2953	4.11E-67	0.63028362	0.559	0.099	8.96E-63	SKA2	10	STMN1_T-cycling
2954	8.95E-173	0.63027052	0.404	0.02	1.95E-168	CENPA	10	STMN1_T-cycling
2955	1.39E-178	0.62947601	0.366	0.015	3.04E-174	KIF4A	10	STMN1_T-cycling
2956	6.16E-110	0.62352998	0.335	0.023	1.34E-105	CDT1	10	STMN1_T-cycling
2957	8.29E-183	0.6193235	0.385	0.016	1.81E-178	ANLN	10	STMN1_T-cycling
2958	7.01E-60	0.61649853	0.484	0.084	1.53E-55	CBX5	10	STMN1_T-cycling
2959	8.55E-46	0.61627304	0.602	0.153	1.86E-41	UBE2S	10	STMN1_T-cycling
2960	1.39E-119	0.61460056	0.36	0.024	3.03E-115	RAD51AP1	10	STMN1_T-cycling
2961	1.28E-136	0.60861441	0.354	0.02	2.80E-132	KIF15	10	STMN1_T-cycling
2962	1.15E-135	0.60813941	0.416	0.028	2.51E-131	NCAPH	10	STMN1_T-cycling
2963	1.05E-134	0.60291094	0.335	0.018	2.29E-130	SPAG5	10	STMN1_T-cycling
2964	1.26E-149	0.6019631	0.391	0.022	2.75E-145	ZWINT	10	STMN1_T-cycling
2965	1.05E-189	0.59805699	0.398	0.016	2.29E-185	TTK	10	STMN1_T-cycling
2966	3.90E-35	0.59723932	0.547	0.156	8.51E-31	FAM111A	10	STMN1_T-cycling
2967	1.56E-25	0.58552941	0.863	0.461	3.39E-21	BUB3	10	STMN1_T-cycling
2968	1.88E-19	0.58545702	0.901	0.655	4.11E-15	CRIP1	10	STMN1_T-cycling
2969	1.03E-72	0.58347285	0.447	0.062	2.24E-68	CDKN2C	10	STMN1_T-cycling
2970	9.10E-69	0.58314592	0.503	0.081	1.98E-64	CENPK	10	STMN1_T-cycling
2971	6.10E-30	0.58294983	0.839	0.355	1.33E-25	NAP1L1	10	STMN1_T-cycling
2972	2.00E-13	0.57862661	0.727	0.364	4.37E-09	SYNE2	10	STMN1_T-cycling
2973	3.56E-34	0.5775873	0.571	0.172	7.77E-30	CDKN2D	10	STMN1_T-cycling
2974	4.67E-166	0.57496772	0.366	0.016	1.02E-161	CEP55	10	STMN1_T-cycling
2975	2.38E-100	0.57458003	0.366	0.031	5.19E-96	NDC80	10	STMN1_T-cycling
2976	2.01E-81	0.57231804	0.354	0.036	4.38E-77	SAC3D1	10	STMN1_T-cycling
2977	1.11E-33	0.5717979	0.671	0.222	2.42E-29	DCK	10	STMN1_T-cycling
2978	2.98E-29	0.56981625	0.994	0.991	6.51E-25	PTMA	10	STMN1_T-cycling
2979	6.38E-109	0.56873432	0.348	0.025	1.39E-104	CENPU	10	STMN1_T-cycling
2980	3.43E-103	0.56510896	0.329	0.024	7.48E-99	BRCA2	10	STMN1_T-cycling
2981	3.79E-94	0.56448292	0.398	0.039	8.27E-90	CENPN	10	STMN1_T-cycling
2982	3.16E-28	0.56200636	0.857	0.403	6.90E-24	CDK2AP2	10	STMN1_T-cycling
2983	1.43E-39	0.56088014	0.329	0.06	3.11E-35	CD70	10	STMN1_T-cycling
2984	9.40E-24	0.56086072	0.863	0.477	2.05E-19	HMGN1	10	STMN1_T-cycling
2985	3.07E-30	0.55769606	0.981	0.837	6.70E-26	HNRNPA2B1	10	STMN1_T-cycling
2986	1.25E-61	0.55368477	0.323	0.039	2.73E-57	WHSC1	10	STMN1_T-cycling
2987	4.47E-130	0.55301095	0.304	0.015	9.74E-126	PRR11	10	STMN1_T-cycling
2988	3.43E-36	0.55216396	0.46	0.114	7.48E-32	DDB2	10	STMN1_T-cycling
2989	1.06E-117	0.54302969	0.335	0.021	2.31E-113	FAM64A	10	STMN1_T-cycling
2990	2.94E-89	0.54225012	0.317	0.026	6.42E-85	FAM83D	10	STMN1_T-cycling
2991	4.61E-28	0.54141194	0.497	0.153	1.00E-23	USP1	10	STMN1_T-cycling
2992	2.39E-117	0.53882496	0.354	0.024	5.22E-113	NCAPG2	10	STMN1_T-cycling
2993	3.86E-25	0.5367967	0.323	0.082	8.42E-21	MCM7	10	STMN1_T-cycling
2994	2.33E-150	0.53644547	0.311	0.012	5.07E-146	DIAPH3	10	STMN1_T-cycling
2995	1.06E-33	0.53498481	0.665	0.209	2.30E-29	DDX39A	10	STMN1_T-cycling
2996	5.15E-139	0.53427687	0.261	0.009	1.12E-134	IL26	10	STMN1_T-cycling
2997	5.80E-139	0.53040953	0.323	0.015	1.26E-134	PSRC1	10	STMN1_T-cycling
2998	5.00E-83	0.52963315	0.422	0.049	1.09E-78	RPL39L	10	STMN1_T-cycling
2999	9.90E-51	0.52752438	0.422	0.075	2.16E-46	RRM1	10	STMN1_T-cycling
3000	9.91E-25	0.52728297	0.696	0.283	2.16E-20	LBR	10	STMN1_T-cycling
3001	7.10E-124	0.52727138	0.317	0.017	1.55E-119	KNSTRN	10	STMN1_T-cycling
3002	3.67E-27	0.52526994	0.814	0.353	8.00E-23	SPOCK2	10	STMN1_T-cycling
3003	3.49E-154	0.52392085	0.329	0.014	7.61E-150	KIF18A	10	STMN1_T-cycling
3004	8.86E-40	0.52271955	0.528	0.132	1.93E-35	HPGD	10	STMN1_T-cycling
3005	9.45E-22	0.521353	0.174	0.031	2.06E-17	TRBV6-4	10	STMN1_T-cycling
3006	9.00E-46	0.51952065	0.23	0.027	1.96E-41	HIST1H2AJ	10	STMN1_T-cycling
3007	2.10E-23	0.51547732	0.851	0.463	4.58E-19	LRRFIP1	10	STMN1_T-cycling
3008	4.07E-138	0.51164822	0.317	0.015	8.87E-134	NEIL3	10	STMN1_T-cycling

3009	2.19E-15	0.51094439	0.578	0.268	4.78E-11	DUT	10	STMN1_T-cycling
3010	8.86E-99	0.51093008	0.335	0.026	1.93E-94	ARHGAP11B	10	STMN1_T-cycling
3011	9.06E-126	0.50756011	0.311	0.016	1.98E-121	HMGB3	10	STMN1_T-cycling
3012	4.99E-113	0.50672966	0.404	0.032	1.09E-108	NRM	10	STMN1_T-cycling
3013	2.10E-94	0.50660367	0.273	0.017	4.59E-90	HIST1H1B	10	STMN1_T-cycling
3014	1.43E-55	0.50578859	0.453	0.078	3.13E-51	RUVBL2	10	STMN1_T-cycling
3015	6.09E-45	0.50510694	0.429	0.085	1.33E-40	NUDT1	10	STMN1_T-cycling
3016	7.65E-66	0.50498126	0.273	0.026	1.67E-61	TCF19	10	STMN1_T-cycling
3017	2.50E-89	0.50257948	0.323	0.027	5.46E-85	GGH	10	STMN1_T-cycling
3018	2.48E-116	0.49827234	0.304	0.017	5.42E-112	APOBEC3B	10	STMN1_T-cycling
3019	2.58E-160	0.49720025	0.366	0.017	5.62E-156	KIAA1524	10	STMN1_T-cycling
3020	2.53E-134	0.49499397	0.304	0.014	5.51E-130	SHCBP1	10	STMN1_T-cycling
3021	1.02E-32	0.494664	0.559	0.163	2.22E-28	TMEM71	10	STMN1_T-cycling
3022	9.01E-56	0.488866	0.416	0.068	1.97E-51	HDGF	10	STMN1_T-cycling
3023	6.58E-130	0.48818689	0.28	0.012	1.43E-125	MND1	10	STMN1_T-cycling
3024	2.06E-163	0.48684423	0.329	0.013	4.50E-159	BUB1B	10	STMN1_T-cycling
3025	2.61E-103	0.48559577	0.304	0.02	5.69E-99	LMO7	10	STMN1_T-cycling
3026	4.81E-29	0.48490908	0.665	0.23	1.05E-24	CLEC2D	10	STMN1_T-cycling
3027	1.50E-140	0.4845746	0.311	0.014	3.26E-136	PBK	10	STMN1_T-cycling
3028	5.90E-106	0.48158952	0.298	0.018	1.29E-101	ARHGEF39	10	STMN1_T-cycling
3029	5.20E-50	0.48119793	0.273	0.035	1.13E-45	DHFR	10	STMN1_T-cycling
3030	2.48E-48	0.47707971	0.404	0.072	5.41E-44	RANGAP1	10	STMN1_T-cycling
3031	1.85E-31	0.47696207	0.634	0.2	4.03E-27	ANXA5	10	STMN1_T-cycling
3032	2.59E-19	0.47499659	0.733	0.341	5.66E-15	SFPQ	10	STMN1_T-cycling
3033	7.41E-27	0.47495592	0.658	0.242	1.62E-22	ILF2	10	STMN1_T-cycling
3034	2.24E-82	0.47477483	0.23	0.014	4.88E-78	ATP8B3	10	STMN1_T-cycling
3035	1.43E-23	0.47157931	0.366	0.103	3.11E-19	HIST1H1C	10	STMN1_T-cycling
3036	6.96E-35	0.46947681	0.36	0.076	1.52E-30	LINC00649	10	STMN1_T-cycling
3037	1.27E-125	0.46829541	0.292	0.014	2.77E-121	MELK	10	STMN1_T-cycling
3038	3.71E-90	0.46756267	0.317	0.025	8.10E-86	FANCI	10	STMN1_T-cycling
3039	1.08E-188	0.46604197	0.298	0.008	2.35E-184	CIT	10	STMN1_T-cycling
3040	1.43E-23	0.46491325	0.764	0.34	3.12E-19	SDCBP	10	STMN1_T-cycling
3041	5.31E-86	0.46397904	0.242	0.015	1.16E-81	ESCO2	10	STMN1_T-cycling
3042	1.18E-30	0.46288946	0.478	0.132	2.58E-26	TMEM107	10	STMN1_T-cycling
3043	8.23E-64	0.46147399	0.36	0.046	1.79E-59	EMC9	10	STMN1_T-cycling
3044	1.51E-64	0.4612712	0.404	0.056	3.29E-60	REEP4	10	STMN1_T-cycling
3045	2.07E-27	0.45732862	0.404	0.108	4.51E-23	SMC1A	10	STMN1_T-cycling
3046	8.89E-32	0.45448529	0.373	0.087	1.94E-27	DNAJC9	10	STMN1_T-cycling
3047	1.01E-45	0.45163262	0.298	0.044	2.21E-41	CAPG	10	STMN1_T-cycling
3048	5.38E-92	0.45156838	0.304	0.023	1.17E-87	SPDL1	10	STMN1_T-cycling
3049	7.11E-41	0.44893934	0.205	0.024	1.55E-36	CLSPN	10	STMN1_T-cycling
3050	1.74E-23	0.44892597	0.565	0.198	3.79E-19	PRDX3	10	STMN1_T-cycling
3051	3.16E-16	0.44001423	0.596	0.258	6.90E-12	PTBP3	10	STMN1_T-cycling
3052	2.42E-45	0.43996247	0.46	0.093	5.27E-41	G2E3	10	STMN1_T-cycling
3053	5.31E-58	0.43925974	0.335	0.044	1.16E-53	DLEU2	10	STMN1_T-cycling
3054	1.85E-43	0.43728857	0.342	0.058	4.03E-39	CCDC18	10	STMN1_T-cycling
3055	3.41E-114	0.43694663	0.217	0.008	7.43E-110	POLQ	10	STMN1_T-cycling
3056	6.65E-07	0.43471022	0.981	0.85	0.0145012	HSP90AA1	10	STMN1_T-cycling
3057	2.57E-16	0.43395866	0.77	0.396	5.61E-12	TUBA1A	10	STMN1_T-cycling
3058	2.33E-10	0.43359391	0.795	0.525	5.09E-06	FOSB	10	STMN1_T-cycling
3059	8.65E-30	0.42968844	0.547	0.162	1.89E-25	SMS	10	STMN1_T-cycling
3060	2.23E-58	0.42910935	0.267	0.028	4.87E-54	FBXO5	10	STMN1_T-cycling
3061	3.83E-33	0.42651443	0.267	0.047	8.35E-29	NCAPD3	10	STMN1_T-cycling
3062	2.07E-19	0.42633446	0.534	0.204	4.51E-15	NDUFAF3	10	STMN1_T-cycling
3063	4.12E-23	0.4262282	0.335	0.092	8.98E-19	HIST1H1E	10	STMN1_T-cycling
3064	2.65E-27	0.42615641	0.422	0.116	5.77E-23	SAE1	10	STMN1_T-cycling
3065	5.83E-17	0.42371223	0.82	0.435	1.27E-12	YWHAQ	10	STMN1_T-cycling
3066	2.53E-20	0.4236828	0.702	0.306	5.51E-16	CBX3	10	STMN1_T-cycling
3067	5.82E-43	0.4207245	0.292	0.044	1.27E-38	CDCA4	10	STMN1_T-cycling
3068	3.62E-21	0.42034787	0.677	0.276	7.89E-17	NDUFA2	10	STMN1_T-cycling
3069	6.09E-15	0.41697139	0.267	0.083	1.33E-10	HIST1H1D	10	STMN1_T-cycling
3070	5.05E-28	0.41465407	0.46	0.13	1.10E-23	NDE1	10	STMN1_T-cycling
3071	5.59E-19	0.4146287	0.646	0.277	1.22E-14	HSD17B11	10	STMN1_T-cycling
3072	8.96E-45	0.41401911	0.503	0.108	1.95E-40	TPRKB	10	STMN1_T-cycling
3073	3.32E-23	0.41394722	0.484	0.159	7.24E-19	SLC7A5	10	STMN1_T-cycling
3074	2.49E-15	0.41291614	0.832	0.503	5.44E-11	SLC25A5	10	STMN1_T-cycling
3075	7.13E-14	0.41262018	0.863	0.498	1.55E-09	HNRNPA0	10	STMN1_T-cycling
3076	2.23E-26	0.41147903	0.671	0.236	4.85E-22	MRPL51	10	STMN1_T-cycling
3077	2.54E-70	0.41059798	0.292	0.028	5.54E-66	CENPH	10	STMN1_T-cycling
3078	9.20E-09	0.40901199	0.534	0.292	0.000200665	EGR1	10	STMN1_T-cycling

3079	1.47E-123	0.40825229	0.255	0.01	3.20E-119	FAM72B	10	STMN1_T-cycling
3080	4.34E-33	0.40762263	0.373	0.082	9.46E-29	VRK1	10	STMN1_T-cycling
3081	2.97E-38	0.40703845	0.317	0.056	6.47E-34	ITGB3BP	10	STMN1_T-cycling
3082	3.91E-29	0.40415933	0.36	0.086	8.54E-25	TMEM106C	10	STMN1_T-cycling
3083	1.79E-31	0.40280135	0.41	0.1	3.91E-27	PRPSAP1	10	STMN1_T-cycling
3084	3.57E-123	0.40091358	0.273	0.012	7.79E-119	POC1A	10	STMN1_T-cycling
3085	4.89E-15	0.39644729	0.348	0.124	1.07E-10	RNU12	10	STMN1_T-cycling
3086	4.61E-42	0.39584455	0.199	0.022	1.01E-37	HIST1H2AI	10	STMN1_T-cycling
3087	1.21E-100	0.39524752	0.242	0.012	2.63E-96	SKA3	10	STMN1_T-cycling
3088	2.11E-25	0.39514697	0.503	0.157	4.61E-21	RPA3	10	STMN1_T-cycling
3089	1.50E-28	0.39399301	0.484	0.137	3.28E-24	ARL4A	10	STMN1_T-cycling
3090	2.86E-23	0.39271452	0.478	0.154	6.24E-19	IFI6	10	STMN1_T-cycling
3091	2.61E-17	0.39175163	0.602	0.258	5.69E-13	CDKN1A	10	STMN1_T-cycling
3092	1.06E-15	0.39002904	0.77	0.38	2.31E-11	RBMX	10	STMN1_T-cycling
3093	1.19E-20	0.38935709	0.596	0.231	2.60E-16	CCNG1	10	STMN1_T-cycling
3094	5.06E-45	0.38744019	0.317	0.049	1.10E-40	BCL2L12	10	STMN1_T-cycling
3095	5.65E-68	0.38726188	0.217	0.016	1.23E-63	ORC6	10	STMN1_T-cycling
3096	1.16E-22	0.38650079	0.441	0.138	2.53E-18	XPO1	10	STMN1_T-cycling
3097	2.13E-100	0.38626217	0.236	0.011	4.65E-96	STIL	10	STMN1_T-cycling
3098	1.18E-45	0.38625645	0.273	0.037	2.57E-41	CCDC88A	10	STMN1_T-cycling
3099	4.71E-63	0.38622391	0.236	0.02	1.03E-58	CEP152	10	STMN1_T-cycling
3100	6.21E-33	0.38477914	0.385	0.087	1.35E-28	DPP4	10	STMN1_T-cycling
3101	2.62E-78	0.3837872	0.242	0.017	5.70E-74	DEPDC1	10	STMN1_T-cycling
3102	2.97E-31	0.38181202	0.453	0.117	6.47E-27	IMPDH2	10	STMN1_T-cycling
3103	2.17E-17	0.3807539	0.522	0.206	4.73E-13	SIVA1	10	STMN1_T-cycling
3104	1.83E-14	0.3805294	0.745	0.38	3.98E-10	SH2D1A	10	STMN1_T-cycling
3105	1.09E-26	0.38031025	0.497	0.146	2.37E-22	DCP2	10	STMN1_T-cycling
3106	1.13E-21	0.38002069	0.422	0.132	2.47E-17	MAD2L2	10	STMN1_T-cycling
3107	1.16E-12	0.37843384	0.907	0.697	2.54E-08	S100A10	10	STMN1_T-cycling
3108	5.65E-16	0.37689859	0.776	0.392	1.23E-11	SRP9	10	STMN1_T-cycling
3109	7.80E-53	0.37605033	0.236	0.024	1.70E-48	CEP57L1	10	STMN1_T-cycling
3110	1.30E-15	0.37576716	0.665	0.317	2.84E-11	TPM4	10	STMN1_T-cycling
3111	1.32E-67	0.37554091	0.242	0.02	2.88E-63	INCENP	10	STMN1_T-cycling
3112	4.21E-59	0.37532135	0.161	0.01	9.19E-55	FAM111B	10	STMN1_T-cycling
3113	1.23E-17	0.37485907	0.783	0.375	2.68E-13	ATP5F1	10	STMN1_T-cycling
3114	1.03E-68	0.37364535	0.23	0.017	2.25E-64	CBR3	10	STMN1_T-cycling
3115	1.51E-26	0.37362673	0.565	0.18	3.30E-22	FBXL5	10	STMN1_T-cycling
3116	4.37E-20	0.37329809	0.988	0.86	9.52E-16	HNRNPA1	10	STMN1_T-cycling
3117	1.44E-92	0.37318879	0.211	0.01	3.15E-88	KIF18B	10	STMN1_T-cycling
3118	4.28E-58	0.37258308	0.242	0.023	9.32E-54	ARHGAP19	10	STMN1_T-cycling
3119	9.09E-26	0.37166804	0.354	0.09	1.98E-21	UBALD2	10	STMN1_T-cycling
3120	2.97E-24	0.3694421	0.28	0.064	6.47E-20	SPRY1	10	STMN1_T-cycling
3121	1.14E-14	0.36899098	0.994	0.959	2.49E-10	ACTG1	10	STMN1_T-cycling
3122	3.85E-16	0.36794288	0.696	0.318	8.40E-12	KLRG1	10	STMN1_T-cycling
3123	2.46E-52	0.36588281	0.211	0.02	5.36E-48	ATAD5	10	STMN1_T-cycling
3124	2.39E-16	0.36525267	0.329	0.108	5.20E-12	IKBIP	10	STMN1_T-cycling
3125	3.98E-25	0.36482811	0.161	0.024	8.68E-21	HELLS	10	STMN1_T-cycling
3126	5.31E-23	0.36447446	0.671	0.251	1.16E-18	KHDRBS1	10	STMN1_T-cycling
3127	9.08E-87	0.3640172	0.205	0.01	1.98E-82	SMTN	10	STMN1_T-cycling
3128	3.63E-14	0.36394594	0.913	0.585	7.91E-10	TMBIM6	10	STMN1_T-cycling
3129	3.20E-19	0.36353322	0.596	0.228	6.99E-15	KIF5B	10	STMN1_T-cycling
3130	2.27E-25	0.36314634	0.46	0.137	4.95E-21	ARHGEF6	10	STMN1_T-cycling
3131	1.52E-17	0.36238374	0.59	0.24	3.31E-13	PNRC2	10	STMN1_T-cycling
3132	6.54E-97	0.3619177	0.248	0.014	1.43E-92	FOXM1	10	STMN1_T-cycling
3133	6.66E-46	0.36166224	0.242	0.029	1.45E-41	CEP70	10	STMN1_T-cycling
3134	4.18E-19	0.36138645	0.273	0.073	9.11E-15	CH17-373J23.1	10	STMN1_T-cycling
3135	1.49E-12	0.36110789	0.211	0.065	3.25E-08	PTCH2	10	STMN1_T-cycling
3136	3.86E-35	0.35810423	0.354	0.072	8.42E-31	ACYP1	10	STMN1_T-cycling
3137	1.25E-26	0.35676467	0.472	0.135	2.72E-22	NUDCD2	10	STMN1_T-cycling
3138	1.22E-55	0.35610328	0.211	0.018	2.66E-51	CRNDE	10	STMN1_T-cycling
3139	9.69E-20	0.35571973	0.478	0.166	2.11E-15	CEP57	10	STMN1_T-cycling
3140	2.01E-12	0.35403966	0.913	0.726	4.39E-08	YBX1	10	STMN1_T-cycling
3141	1.77E-66	0.35298158	0.199	0.013	3.86E-62	C21orf58	10	STMN1_T-cycling
3142	2.43E-84	0.35213631	0.23	0.014	5.30E-80	SKA1	10	STMN1_T-cycling
3143	4.80E-95	0.34970538	0.255	0.015	1.05E-90	MTFR2	10	STMN1_T-cycling
3144	1.51E-28	0.34639408	0.304	0.065	3.29E-24	TOPBP1	10	STMN1_T-cycling
3145	6.50E-32	0.34621657	0.236	0.039	1.42E-27	LIG1	10	STMN1_T-cycling
3146	5.18E-12	0.34591746	0.758	0.417	1.13E-07	WSB1	10	STMN1_T-cycling
3147	2.74E-68	0.34539101	0.186	0.011	5.97E-64	PLK4	10	STMN1_T-cycling
3148	4.28E-19	0.34530598	0.497	0.174	9.33E-15	SMC3	10	STMN1_T-cycling

3149	7.09E-37	0.34522159	0.435	0.096	1.55E-32	CDC27	10	STMN1_T-cycling
3150	7.06E-29	0.34520105	0.441	0.117	1.54E-24	DBF4	10	STMN1_T-cycling
3151	2.33E-11	0.34431654	0.87	0.513	5.08E-07	HNRNPA3	10	STMN1_T-cycling
3152	4.21E-14	0.34194914	0.429	0.172	9.17E-10	DNMT1	10	STMN1_T-cycling
3153	1.50E-20	0.34176944	0.509	0.177	3.27E-16	H2AFY	10	STMN1_T-cycling
3154	5.03E-34	0.34102384	0.354	0.074	1.10E-29	METTL10	10	STMN1_T-cycling
3155	7.24E-17	0.34044961	1	0.998	1.58E-12	ACTB	10	STMN1_T-cycling
3156	6.54E-12	0.33977521	0.708	0.379	1.43E-07	HMGN3	10	STMN1_T-cycling
3157	5.20E-15	0.3394401	0.652	0.29	1.13E-10	BANF1	10	STMN1_T-cycling
3158	1.46E-10	0.33749052	0.839	0.561	3.18E-06	TNFAIP3	10	STMN1_T-cycling
3159	2.10E-10	0.33748644	0.925	0.79	4.58E-06	S100A6	10	STMN1_T-cycling
3160	8.01E-22	0.33728913	0.652	0.243	1.75E-17	TMEM123	10	STMN1_T-cycling
3161	6.49E-35	0.33688306	0.354	0.072	1.42E-30	ODF2	10	STMN1_T-cycling
3162	8.42E-73	0.33672574	0.23	0.016	1.84E-68	CCNF	10	STMN1_T-cycling
3163	4.37E-31	0.33593272	0.46	0.118	9.54E-27	TRIM69	10	STMN1_T-cycling
3164	1.83E-18	0.33518323	0.509	0.187	3.98E-14	FABP5	10	STMN1_T-cycling
3165	7.57E-20	0.33501083	0.503	0.175	1.65E-15	SUMO3	10	STMN1_T-cycling
3166	2.41E-21	0.33499841	0.609	0.222	5.25E-17	BLOC1S1	10	STMN1_T-cycling
3167	1.83E-25	0.33472356	0.373	0.097	4.00E-21	CD28	10	STMN1_T-cycling
3168	3.73E-55	0.33451175	0.261	0.028	8.14E-51	TTF2	10	STMN1_T-cycling
3169	7.28E-19	0.33434942	0.478	0.167	1.59E-14	FAS	10	STMN1_T-cycling
3170	1.03E-11	0.33430027	0.901	0.743	2.24E-07	CCNI	10	STMN1_T-cycling
3171	2.11E-29	0.33421767	0.292	0.06	4.60E-25	HMGXB4	10	STMN1_T-cycling
3172	4.19E-70	0.33413832	0.149	0.006	9.15E-66	E2F7	10	STMN1_T-cycling
3173	4.88E-22	0.33401434	0.323	0.086	1.06E-17	DESI2	10	STMN1_T-cycling
3174	3.22E-25	0.33329833	0.379	0.101	7.03E-21	SCLT1	10	STMN1_T-cycling
3175	8.40E-36	0.33323818	0.292	0.051	1.83E-31	FGFR1OP	10	STMN1_T-cycling
3176	8.70E-14	0.33299479	0.646	0.303	1.90E-09	HNRNPR	10	STMN1_T-cycling
3177	7.25E-12	0.3322323	0.255	0.089	1.58E-07	FEZ1	10	STMN1_T-cycling
3178	5.47E-65	0.33123903	0.199	0.014	1.19E-60	CHEK1	10	STMN1_T-cycling
3179	7.34E-12	0.33117534	0.503	0.228	1.60E-07	PSIP1	10	STMN1_T-cycling
3180	9.92E-74	0.33107326	0.23	0.016	2.16E-69	FGFR1	10	STMN1_T-cycling
3181	7.47E-30	0.33102482	0.429	0.108	1.63E-25	PMVK	10	STMN1_T-cycling
3182	2.28E-39	0.33097151	0.28	0.044	4.98E-35	ILVBL	10	STMN1_T-cycling
3183	1.97E-40	0.32975824	0.217	0.027	4.29E-36	MASTL	10	STMN1_T-cycling
3184	5.68E-35	0.32967404	0.317	0.059	1.24E-30	CBX1	10	STMN1_T-cycling
3185	1.93E-11	0.32957689	0.882	0.536	4.21E-07	HSP90B1	10	STMN1_T-cycling
3186	4.63E-60	0.32922632	0.193	0.014	1.01E-55	RCCD1	10	STMN1_T-cycling
3187	3.09E-74	0.32889919	0.174	0.008	6.75E-70	WDR62	10	STMN1_T-cycling
3188	1.21E-24	0.32815197	0.435	0.126	2.63E-20	SYPL1	10	STMN1_T-cycling
3189	1.91E-29	0.32814314	0.317	0.068	4.16E-25	BRD8	10	STMN1_T-cycling
3190	8.14E-24	0.32795593	0.311	0.076	1.78E-19	ID1	10	STMN1_T-cycling
3191	8.15E-17	0.32780533	0.422	0.152	1.78E-12	CEBPD	10	STMN1_T-cycling
3192	1.46E-16	0.32662121	0.453	0.167	3.18E-12	AQP3	10	STMN1_T-cycling
3193	3.72E-12	0.32649589	0.826	0.497	8.11E-08	RBM3	10	STMN1_T-cycling
3194	2.48E-35	0.32608783	0.255	0.041	5.41E-31	LMNB2	10	STMN1_T-cycling
3195	2.77E-17	0.32505164	0.59	0.233	6.05E-13	TAF15	10	STMN1_T-cycling
3196	6.79E-13	0.32426144	0.677	0.341	1.48E-08	LYAR	10	STMN1_T-cycling
3197	1.37E-14	0.32409342	0.696	0.308	3.00E-10	ELOVL5	10	STMN1_T-cycling
3198	8.50E-37	0.32384165	0.304	0.053	1.85E-32	GMNN	10	STMN1_T-cycling
3199	2.64E-69	0.32347666	0.199	0.012	5.76E-65	ARHGAP33	10	STMN1_T-cycling
3200	2.51E-10	0.32313716	0.348	0.151	5.47E-06	KLRC1	10	STMN1_T-cycling
3201	9.89E-17	0.3213696	0.975	0.964	2.16E-12	RPLP0	10	STMN1_T-cycling
3202	1.14E-25	0.3197969	0.323	0.077	2.48E-21	RSRC1	10	STMN1_T-cycling
3203	2.43E-22	0.3187258	0.565	0.192	5.30E-18	CMC2	10	STMN1_T-cycling
3204	1.26E-23	0.3171957	0.398	0.112	2.75E-19	PAF1	10	STMN1_T-cycling
3205	1.11E-28	0.31665843	0.261	0.05	2.42E-24	CDK5RAP2	10	STMN1_T-cycling
3206	3.76E-26	0.3142614	0.453	0.128	8.20E-22	CD5	10	STMN1_T-cycling
3207	1.90E-45	0.31419037	0.217	0.024	4.13E-41	ZWILCH	10	STMN1_T-cycling
3208	2.49E-19	0.31408644	0.273	0.072	5.42E-15	HLTF	10	STMN1_T-cycling
3209	3.19E-26	0.31388855	0.342	0.083	6.96E-22	ZSCAN16-AS1	10	STMN1_T-cycling
3210	1.26E-17	0.3127639	0.342	0.108	2.76E-13	SUZ12	10	STMN1_T-cycling
3211	7.76E-48	0.31155754	0.248	0.029	1.69E-43	XRCC6BP1	10	STMN1_T-cycling
3212	3.10E-13	0.31136178	0.615	0.282	6.77E-09	NONO	10	STMN1_T-cycling
3213	5.93E-19	0.31068542	0.46	0.156	1.29E-14	PBX4	10	STMN1_T-cycling
3214	2.82E-22	0.31060436	0.41	0.121	6.16E-18	SLC4A10	10	STMN1_T-cycling
3215	1.17E-21	0.31046105	0.373	0.107	2.56E-17	NUP50	10	STMN1_T-cycling
3216	3.15E-22	0.31042129	0.453	0.141	6.88E-18	MORF4L2	10	STMN1_T-cycling
3217	1.41E-24	0.3096912	0.41	0.114	3.08E-20	DAZAP1	10	STMN1_T-cycling
3218	2.60E-13	0.30834447	0.435	0.173	5.67E-09	CEP350	10	STMN1_T-cycling

3219	4.15E-09	0.30709616	0.919	0.736	9.04E-05	MYL12B	10	STMN1_T-cycling
3220	4.39E-26	0.30688443	0.329	0.078	9.57E-22	TIMM10	10	STMN1_T-cycling
3221	1.52E-11	0.30613714	0.627	0.326	3.32E-07	NR4A1	10	STMN1_T-cycling
3222	1.46E-21	0.30586432	0.348	0.097	3.18E-17	HPS3	10	STMN1_T-cycling
3223	8.59E-13	0.30541077	0.994	0.91	1.87E-08	HSPA8	10	STMN1_T-cycling
3224	2.64E-12	0.3044809	0.963	0.861	5.75E-08	H3F3A	10	STMN1_T-cycling
3225	3.75E-44	0.30425412	0.161	0.014	8.18E-40	HIST1H3B	10	STMN1_T-cycling
3226	4.44E-12	0.30349537	0.764	0.402	9.69E-08	FKBP5	10	STMN1_T-cycling
3227	4.27E-10	0.30339438	0.453	0.209	9.31E-06	RANBP1	10	STMN1_T-cycling
3228	2.17E-21	0.30283264	0.236	0.053	4.74E-17	PLEKHA5	10	STMN1_T-cycling
3229	2.41E-32	0.30273316	0.23	0.036	5.26E-28	LIN54	10	STMN1_T-cycling
3230	4.94E-26	0.30240603	0.317	0.074	1.08E-21	ANKRD36C	10	STMN1_T-cycling
3231	5.07E-16	0.30235944	0.311	0.098	1.11E-11	SPAG9	10	STMN1_T-cycling
3232	1.01E-18	0.30077607	0.516	0.184	2.20E-14	MED30	10	STMN1_T-cycling
3233	8.57E-11	0.30049182	0.944	0.777	1.87E-06	PCBP2	10	STMN1_T-cycling
3234	8.75E-14	0.30027747	0.559	0.242	1.91E-09	EIF4A3	10	STMN1_T-cycling
3235	2.33E-75	0.30019045	0.18	0.009	5.09E-71	SPC24	10	STMN1_T-cycling
3236	1.15E-23	0.29956513	0.224	0.045	2.50E-19	POLH	10	STMN1_T-cycling
3237	2.25E-09	0.29933424	0.77	0.454	4.90E-05	EIF3L	10	STMN1_T-cycling
3238	1.87E-110	0.29903049	0.161	0.004	4.07E-106	NMU	10	STMN1_T-cycling
3239	7.46E-18	0.2987919	0.54	0.203	1.63E-13	DCAF7	10	STMN1_T-cycling
3240	6.41E-29	0.2984453	0.28	0.056	1.40E-24	PLIN3	10	STMN1_T-cycling
3241	3.93E-46	0.29802882	0.267	0.035	8.57E-42	LRR1	10	STMN1_T-cycling
3242	7.43E-12	0.29793532	0.391	0.161	1.62E-07	NAMPT	10	STMN1_T-cycling
3243	1.09E-11	0.29592801	0.708	0.37	2.37E-07	UCP2	10	STMN1_T-cycling
3244	1.18E-15	0.29588949	0.59	0.244	2.57E-11	VDAC3	10	STMN1_T-cycling
3245	2.12E-11	0.2948352	0.814	0.447	4.63E-07	ATPIF1	10	STMN1_T-cycling
3246	3.43E-17	0.29477769	0.609	0.24	7.49E-13	MLF2	10	STMN1_T-cycling
3247	5.67E-32	0.29469946	0.379	0.084	1.24E-27	TPGS2	10	STMN1_T-cycling
3248	2.46E-12	0.29319566	0.696	0.35	5.37E-08	XRCC6	10	STMN1_T-cycling
3249	2.99E-09	0.2925314	0.944	0.828	6.52E-05	FOS	10	STMN1_T-cycling
3250	2.48E-16	0.29204634	0.292	0.088	5.41E-12	PDS5B	10	STMN1_T-cycling
3251	1.20E-13	0.29204181	0.429	0.168	2.63E-09	RNASEH2C	10	STMN1_T-cycling
3252	1.13E-07	0.29174547	0.851	0.639	0.002462753	C12orf57	10	STMN1_T-cycling
3253	5.88E-14	0.29103223	0.453	0.181	1.28E-09	RBBP7	10	STMN1_T-cycling
3254	2.30E-63	0.29081402	0.161	0.009	5.01E-59	FBXO43	10	STMN1_T-cycling
3255	5.90E-10	0.2899606	0.845	0.519	1.29E-05	ATP5B	10	STMN1_T-cycling
3256	2.49E-10	0.28969254	0.621	0.322	5.43E-06	NDUFV2	10	STMN1_T-cycling
3257	9.15E-69	0.28937458	0.149	0.007	2.00E-64	HIST1H3G	10	STMN1_T-cycling
3258	5.95E-14	0.28856087	0.317	0.109	1.30E-09	TRGC2	10	STMN1_T-cycling
3259	2.99E-37	0.28841287	0.224	0.03	6.52E-33	CD9	10	STMN1_T-cycling
3260	9.28E-20	0.28825021	0.547	0.193	2.02E-15	FXR1	10	STMN1_T-cycling
3261	3.40E-26	0.28821005	0.304	0.069	7.41E-22	SNX9	10	STMN1_T-cycling
3262	5.76E-14	0.28789227	0.46	0.184	1.26E-09	ANKRD28	10	STMN1_T-cycling
3263	8.90E-38	0.28761555	0.199	0.024	1.94E-33	TMEM171	10	STMN1_T-cycling
3264	3.94E-20	0.28710328	0.472	0.156	8.58E-16	COX20	10	STMN1_T-cycling
3265	3.46E-15	0.2863623	0.615	0.256	7.54E-11	MZT2A	10	STMN1_T-cycling
3266	4.60E-21	0.28618099	0.286	0.071	1.00E-16	GOT1	10	STMN1_T-cycling
3267	9.54E-32	0.2860754	0.354	0.076	2.08E-27	IMMP1L	10	STMN1_T-cycling
3268	2.64E-11	0.28596779	0.627	0.306	5.76E-07	TNFAIP8	10	STMN1_T-cycling
3269	8.60E-41	0.28412201	0.211	0.025	1.87E-36	RNF26	10	STMN1_T-cycling
3270	4.58E-08	0.28409328	0.211	0.085	0.00099806	MCM3	10	STMN1_T-cycling
3271	5.48E-15	0.28335446	0.609	0.259	1.19E-10	ZNF706	10	STMN1_T-cycling
3272	1.55E-13	0.28301014	0.571	0.244	3.39E-09	PSMA4	10	STMN1_T-cycling
3273	7.73E-16	0.28273957	0.385	0.133	1.69E-11	CEP85L	10	STMN1_T-cycling
3274	1.87E-12	0.28259582	0.696	0.35	4.08E-08	HNRNPUL1	10	STMN1_T-cycling
3275	6.27E-18	0.28235992	0.447	0.154	1.37E-13	FUBP1	10	STMN1_T-cycling
3276	3.17E-09	0.28224064	0.907	0.596	6.92E-05	EMP3	10	STMN1_T-cycling
3277	1.07E-31	0.28214732	0.161	0.019	2.33E-27	TK1	10	STMN1_T-cycling
3278	4.22E-16	0.28049996	0.491	0.187	9.20E-12	MAZ	10	STMN1_T-cycling
3279	2.80E-09	0.28041939	0.87	0.561	6.12E-05	SRSF3	10	STMN1_T-cycling
3280	9.46E-09	0.27975999	0.925	0.709	0.000206328	SUMO2	10	STMN1_T-cycling
3281	5.68E-19	0.27973128	0.398	0.128	1.24E-14	FAM200B	10	STMN1_T-cycling
3282	4.42E-12	0.27918745	0.609	0.296	9.64E-08	GLUL	10	STMN1_T-cycling
3283	1.77E-48	0.27766039	0.211	0.021	3.87E-44	ZNF714	10	STMN1_T-cycling
3284	1.84E-24	0.2772178	0.373	0.098	4.00E-20	CCDC82	10	STMN1_T-cycling
3285	1.58E-28	0.27720257	0.342	0.077	3.45E-24	ANAPC15	10	STMN1_T-cycling
3286	1.88E-10	0.27702632	0.938	0.78	4.10E-06	RHOA	10	STMN1_T-cycling
3287	3.85E-49	0.27688019	0.236	0.026	8.39E-45	MIS18A	10	STMN1_T-cycling
3288	1.23E-08	0.27597772	0.801	0.493	0.000267709	S100A11	10	STMN1_T-cycling

3289	6.21E-19	0.27569325	0.894	0.397	1.35E-14	IL7R	10	STMN1_T-cycling
3290	3.04E-43	0.27552382	0.18	0.017	6.62E-39	BRCA1	10	STMN1_T-cycling
3291	2.13E-14	0.2751873	0.559	0.236	4.64E-10	SATB1	10	STMN1_T-cycling
3292	2.32E-14	0.2747153	0.54	0.225	5.07E-10	SSR3	10	STMN1_T-cycling
3293	8.57E-53	0.27471292	0.161	0.011	1.87E-48	NUDT6	10	STMN1_T-cycling
3294	1.22E-24	0.27465341	0.242	0.049	2.66E-20	KATNAL1	10	STMN1_T-cycling
3295	4.92E-37	0.27458914	0.23	0.032	1.07E-32	RP11-545E17.3	10	STMN1_T-cycling
3296	5.52E-90	0.2742553	0.199	0.009	1.20E-85	OIP5	10	STMN1_T-cycling
3297	8.49E-23	0.27423072	0.323	0.083	1.85E-18	CTCF	10	STMN1_T-cycling
3298	1.68E-50	0.27420842	0.137	0.008	3.66E-46	EXPH5	10	STMN1_T-cycling
3299	2.13E-09	0.27410859	0.745	0.393	4.65E-05	SNRPG	10	STMN1_T-cycling
3300	6.63E-34	0.27340553	0.311	0.058	1.45E-29	PERP	10	STMN1_T-cycling
3301	6.63E-09	0.27299679	0.739	0.447	0.000144617	UXT	10	STMN1_T-cycling
3302	7.61E-15	0.27242892	0.23	0.065	1.66E-10	ANKRD36	10	STMN1_T-cycling
3303	1.72E-16	0.27206195	0.211	0.053	3.76E-12	IL23R	10	STMN1_T-cycling
3304	7.43E-09	0.27121196	0.863	0.554	0.000162075	LDHA	10	STMN1_T-cycling
3305	2.19E-40	0.26958156	0.205	0.024	4.77E-36	FZR1	10	STMN1_T-cycling
3306	6.64E-17	0.26952438	0.447	0.16	1.45E-12	TXNDC12	10	STMN1_T-cycling
3307	5.45E-16	0.26948032	0.199	0.049	1.19E-11	SRGAP2	10	STMN1_T-cycling
3308	1.12E-18	0.26857282	0.36	0.111	2.44E-14	PSMD14	10	STMN1_T-cycling
3309	1.36E-24	0.26781832	0.217	0.041	2.96E-20	ZMYM1	10	STMN1_T-cycling
3310	1.71E-10	0.26774061	0.963	0.943	3.73E-06	PPIA	10	STMN1_T-cycling
3311	5.93E-26	0.2676409	0.311	0.071	1.29E-21	PHACTR2	10	STMN1_T-cycling
3312	1.78E-08	0.26679223	1	0.999	0.000388328	MT-CO3	10	STMN1_T-cycling
3313	4.46E-10	0.26642139	0.764	0.433	9.72E-06	ABRACL	10	STMN1_T-cycling
3314	4.32E-17	0.26587683	0.516	0.192	9.43E-13	LSM3	10	STMN1_T-cycling
3315	3.72E-16	0.26574689	0.335	0.108	8.11E-12	IKZF2	10	STMN1_T-cycling
3316	2.00E-26	0.26572644	0.311	0.07	4.37E-22	ZDHHC12	10	STMN1_T-cycling
3317	7.00E-24	0.26545458	0.323	0.08	1.53E-19	YEATS4	10	STMN1_T-cycling
3318	1.25E-14	0.26512129	0.522	0.21	2.72E-10	GNAI3	10	STMN1_T-cycling
3319	5.11E-30	0.26420689	0.323	0.068	1.11E-25	HADH	10	STMN1_T-cycling
3320	3.91E-10	0.26355316	0.646	0.325	8.52E-06	ADD3	10	STMN1_T-cycling
3321	2.10E-19	0.26333774	0.248	0.061	4.57E-15	SLC25A40	10	STMN1_T-cycling
3322	4.12E-11	0.26319555	0.745	0.384	8.99E-07	ACTR2	10	STMN1_T-cycling
3323	3.31E-24	0.26318163	0.447	0.13	7.22E-20	TRAV1-2	10	STMN1_T-cycling
3324	6.14E-24	0.26314519	0.304	0.073	1.34E-19	TMEM14C	10	STMN1_T-cycling
3325	2.22E-07	0.2628535	0.106	0.031	0.004842201	HIST1H2BH	10	STMN1_T-cycling
3326	3.96E-16	0.26225866	0.379	0.13	8.64E-12	HINT2	10	STMN1_T-cycling
3327	1.85E-08	0.26223721	0.851	0.588	0.000402529	SLC25A3	10	STMN1_T-cycling
3328	3.01E-16	0.26152406	0.658	0.273	6.56E-12	UGP2	10	STMN1_T-cycling
3329	1.74E-19	0.26082145	0.391	0.12	3.81E-15	FOPNL	10	STMN1_T-cycling
3330	8.48E-29	0.26065993	0.186	0.027	1.85E-24	FEN1	10	STMN1_T-cycling
3331	3.17E-34	0.26044338	0.124	0.011	6.91E-30	DTL	10	STMN1_T-cycling
3332	1.26E-39	0.26006883	0.211	0.026	2.75E-35	SRGAP2C	10	STMN1_T-cycling
3333	1.11E-07	0.25982683	0.304	0.141	0.002428189	RELB	10	STMN1_T-cycling
3334	1.40E-20	0.2594138	0.304	0.081	3.05E-16	SEC22C	10	STMN1_T-cycling
3335	9.24E-12	0.25912121	0.671	0.318	2.01E-07	SUN2	10	STMN1_T-cycling
3336	1.54E-10	0.258859	0.553	0.26	3.37E-06	ATF7IP	10	STMN1_T-cycling
3337	7.94E-22	0.25878338	0.366	0.102	1.73E-17	TMEM261	10	STMN1_T-cycling
3338	1.02E-16	0.25873389	0.441	0.156	2.23E-12	LETMD1	10	STMN1_T-cycling
3339	5.15E-51	0.25836223	0.13	0.007	1.12E-46	RAD54L	10	STMN1_T-cycling
3340	2.60E-11	0.25755312	0.509	0.228	5.67E-07	KRAS	10	STMN1_T-cycling
3341	5.38E-13	0.25730356	0.342	0.126	1.17E-08	PHTF2	10	STMN1_T-cycling
3342	1.62E-27	0.25699778	0.168	0.024	3.54E-23	RFWD3	10	STMN1_T-cycling
3343	6.71E-11	0.2569466	0.317	0.124	1.46E-06	CRY1	10	STMN1_T-cycling
3344	1.20E-21	0.25638435	0.292	0.073	2.62E-17	MICB	10	STMN1_T-cycling
3345	1.36E-19	0.2562754	0.36	0.107	2.96E-15	RUNX2	10	STMN1_T-cycling
3346	9.35E-17	0.25627026	0.373	0.124	2.04E-12	MDFIC	10	STMN1_T-cycling
3347	7.18E-20	0.2559937	0.503	0.17	1.57E-15	TRAT1	10	STMN1_T-cycling
3348	7.33E-20	0.25580749	0.13	0.02	1.60E-15	NEMP1	10	STMN1_T-cycling
3349	2.72E-12	0.25516485	0.267	0.09	5.94E-08	OXCT1	10	STMN1_T-cycling
3350	1.58E-31	0.25409991	0.161	0.019	3.44E-27	CEP85	10	STMN1_T-cycling
3351	1.20E-08	0.25358343	0.95	0.705	0.000262261	LCP1	10	STMN1_T-cycling
3352	1.87E-16	0.25349318	0.547	0.209	4.08E-12	PSMB8-AS1	10	STMN1_T-cycling
3353	1.78E-15	0.25347436	0.161	0.036	3.87E-11	HIST1H2AG	10	STMN1_T-cycling
3354	1.82E-14	0.2532235	0.441	0.171	3.97E-10	PHLDA1	10	STMN1_T-cycling
3355	1.39E-13	0.25317639	0.553	0.231	3.04E-09	EIF4E	10	STMN1_T-cycling
3356	2.84E-10	0.25307026	0.298	0.116	6.20E-06	PBRM1	10	STMN1_T-cycling
3357	3.61E-08	0.2522709	0.913	0.637	0.000787836	PPP1R15A	10	STMN1_T-cycling
3358	2.79E-14	0.25206279	0.609	0.266	6.08E-10	CACYBP	10	STMN1_T-cycling

3359	1.73E-41	0.25170587	0.205	0.023	3.76E-37	KMT5A	10	STMN1_T-cycling
3360	1.33E-07	0.2512164	1	0.997	0.002909489	MT-CYB	10	STMN1_T-cycling
3361	1.05E-10	0.25115305	0.342	0.137	2.29E-06	TBC1D5	10	STMN1_T-cycling
3362	2.05E-15	0.25080108	0.484	0.182	4.47E-11	LMAN1	10	STMN1_T-cycling
3363	3.36E-16	0.25062363	0.261	0.074	7.33E-12	HCFC1	10	STMN1_T-cycling
3364	3.04E-11	0.2503798	0.317	0.123	6.62E-07	HPRT1	10	STMN1_T-cycling
3365	3.68E-25	0.25020479	0.18	0.029	8.04E-21	WEE1	10	STMN1_T-cycling
3366	4.26E-18	0.2500287	0.335	0.101	9.30E-14	TIFA	10	STMN1_T-cycling
3367	1.01E-60	0.2500176	0.149	0.008	2.21E-56	DDIAS	10	STMN1_T-cycling
3368	2.96E-07	-0.25287527	0.957	0.972	0.006444453	RPL8	10	STMN1_T-cycling
3369	7.92E-09	-0.2644346	0.957	0.985	0.000172812	RPS6	10	STMN1_T-cycling
3370	3.11E-13	-0.28199786	0.975	0.995	6.79E-09	RPS3	10	STMN1_T-cycling
3371	3.75E-07	-0.28722993	0.876	0.894	0.008175818	RPL27	10	STMN1_T-cycling
3372	8.92E-10	-0.28896972	0.913	0.983	1.94E-05	RPL21	10	STMN1_T-cycling
3373	2.63E-12	-0.29099959	0.963	0.996	5.73E-08	RPL11	10	STMN1_T-cycling
3374	3.41E-14	-0.29699814	0.975	0.995	7.44E-10	RPS15	10	STMN1_T-cycling
3375	8.35E-09	-0.29932485	0.95	0.962	0.000182019	RPS29	10	STMN1_T-cycling
3376	6.20E-16	-0.30230051	0.981	0.999	1.35E-11	RPS15A	10	STMN1_T-cycling
3377	1.06E-09	-0.31385	0.932	0.956	2.31E-05	RPS2	10	STMN1_T-cycling
3378	4.68E-12	-0.3191777	0.944	0.979	1.02E-07	RPL17	10	STMN1_T-cycling
3379	1.17E-10	-0.32291141	0.932	0.978	2.54E-06	RPL36	10	STMN1_T-cycling
3380	2.76E-14	-0.32513411	0.975	0.995	6.03E-10	RPL32	10	STMN1_T-cycling
3381	2.19E-12	-0.32514419	0.944	0.987	4.77E-08	RPS25	10	STMN1_T-cycling
3382	5.40E-15	-0.32664832	0.969	0.992	1.18E-10	RPL18A	10	STMN1_T-cycling
3383	1.84E-14	-0.33135827	0.969	0.99	4.00E-10	RPS13	10	STMN1_T-cycling
3384	5.28E-08	-0.33162621	0.882	0.867	0.001150387	RPS11	10	STMN1_T-cycling
3385	1.61E-14	-0.33632196	0.925	0.987	3.51E-10	RPS10	10	STMN1_T-cycling
3386	1.09E-08	-0.34075062	0.925	0.875	0.000238646	PTPRCAP	10	STMN1_T-cycling
3387	1.43E-12	-0.34238613	0.938	0.957	3.12E-08	RPL24	10	STMN1_T-cycling
3388	1.12E-09	-0.35007495	0.882	0.895	2.44E-05	RPL38	10	STMN1_T-cycling
3389	1.95E-15	-0.35624497	0.95	0.982	4.26E-11	RPL3	10	STMN1_T-cycling
3390	1.54E-17	-0.36472195	0.975	0.998	3.36E-13	RPL39	10	STMN1_T-cycling
3391	2.03E-16	-0.37734726	0.925	0.989	4.43E-12	RPL12	10	STMN1_T-cycling
3392	1.96E-16	-0.37895071	0.969	0.989	4.28E-12	RPS18	10	STMN1_T-cycling
3393	4.44E-20	-0.38452386	0.969	0.996	9.68E-16	RPS28	10	STMN1_T-cycling
3394	1.70E-19	-0.38516644	0.932	0.994	3.71E-15	RPS14	10	STMN1_T-cycling
3395	4.55E-23	-0.38702927	0.975	0.994	9.92E-19	RPL26	10	STMN1_T-cycling
3396	2.60E-14	-0.38960939	0.944	0.96	5.66E-10	RPL22	10	STMN1_T-cycling
3397	1.31E-19	-0.39137407	0.969	0.998	2.85E-15	RPL34	10	STMN1_T-cycling
3398	2.29E-23	-0.39509345	0.969	0.995	5.00E-19	RPL19	10	STMN1_T-cycling
3399	2.05E-16	-0.40005432	0.919	0.974	4.48E-12	RPL5	10	STMN1_T-cycling
3400	3.31E-21	-0.40041458	0.95	0.99	7.22E-17	RPL35A	10	STMN1_T-cycling
3401	1.54E-26	-0.40228512	0.994	0.998	3.36E-22	RPL10	10	STMN1_T-cycling
3402	3.69E-21	-0.41872278	0.981	0.998	8.05E-17	RPS12	10	STMN1_T-cycling
3403	1.21E-17	-0.42433544	0.919	0.96	2.65E-13	RPL10A	10	STMN1_T-cycling
3404	6.13E-19	-0.42734142	0.932	0.958	1.34E-14	NACA	10	STMN1_T-cycling
3405	1.80E-07	-0.43370544	0.671	0.682	0.00391954	CD48	10	STMN1_T-cycling
3406	3.41E-09	-0.44301896	0.783	0.738	7.43E-05	CD37	10	STMN1_T-cycling
3407	2.44E-26	-0.44335793	0.994	0.997	5.31E-22	RPL13	10	STMN1_T-cycling
3408	1.13E-06	-0.45917338	0.789	0.741	0.024563731	CD7	10	STMN1_T-cycling
3409	8.43E-09	-0.46838144	0.627	0.661	0.000183914	ALDOA	10	STMN1_T-cycling
3410	4.55E-36	-0.47429765	0.994	0.998	9.92E-32	RPS27A	10	STMN1_T-cycling
3411	3.76E-31	-0.47634589	0.95	0.99	8.21E-27	RPS24	10	STMN1_T-cycling
3412	1.17E-18	-0.47993864	0.925	0.933	2.55E-14	RPL9	10	STMN1_T-cycling
3413	4.00E-11	-0.50201894	0.758	0.742	8.72E-07	COMMD6	10	STMN1_T-cycling
3414	3.33E-13	-0.50349246	0.95	0.963	7.26E-09	JUNB	10	STMN1_T-cycling
3415	2.93E-39	-0.54192572	0.969	0.997	6.40E-35	RPL30	10	STMN1_T-cycling
3416	2.13E-21	-0.54261981	0.888	0.923	4.64E-17	RPL41	10	STMN1_T-cycling
3417	2.06E-20	-0.54515501	0.876	0.902	4.48E-16	PFDN5	10	STMN1_T-cycling
3418	3.66E-11	-0.55714854	0.789	0.825	7.98E-07	TXNIP	10	STMN1_T-cycling
3419	4.26E-11	-0.57875926	0.621	0.672	9.30E-07	CCND3	10	STMN1_T-cycling
3420	2.81E-09	-0.58027422	0.528	0.606	6.13E-05	RARRES3	10	STMN1_T-cycling
3421	9.21E-08	-0.60962269	0.627	0.619	0.002009547	LITAF	10	STMN1_T-cycling
3422	1.17E-08	-0.62235919	0.335	0.465	0.000255481	APRT	10	STMN1_T-cycling
3423	1.17E-06	-0.63228084	0.205	0.336	0.025484292	MATK	10	STMN1_T-cycling
3424	1.58E-21	-0.64739273	0.919	0.906	3.45E-17	CCL5	10	STMN1_T-cycling
3425	1.15E-07	-0.65260546	0.621	0.606	0.002502748	CD247	10	STMN1_T-cycling
3426	6.97E-10	-0.67540117	0.429	0.538	1.52E-05	GZMM	10	STMN1_T-cycling
3427	1.72E-09	-0.69636124	0.311	0.475	3.75E-05	LIMD2	10	STMN1_T-cycling
3428	4.49E-28	-0.70044224	0.919	0.948	9.79E-24	IFITM1	10	STMN1_T-cycling

3429	2.97E-08	-0.70051458	0.236	0.389	0.000646839	DOK2	10	STMN1_T-cycling
3430	1.68E-19	-0.71275563	0.764	0.799	3.66E-15	CYBA	10	STMN1_T-cycling
3431	1.55E-53	-0.71332665	0.975	0.997	3.37E-49	RPS27	10	STMN1_T-cycling
3432	3.08E-56	-0.74901992	0.981	0.999	6.72E-52	AC090498.1	10	STMN1_T-cycling
3433	2.97E-22	-0.8044854	0.907	0.886	6.48E-18	IFITM2	10	STMN1_T-cycling
3434	4.06E-38	-0.81075474	0.944	0.969	8.85E-34	FTL	10	STMN1_T-cycling
3435	6.79E-11	-0.86609248	0.348	0.517	1.48E-06	TRBC1	10	STMN1_T-cycling
3436	1.45E-11	-0.89147784	0.72	0.703	3.16E-07	PRF1	10	STMN1_T-cycling
3437	5.07E-19	-0.89190138	0.547	0.67	1.11E-14	ITGB2	10	STMN1_T-cycling
3438	3.19E-30	-0.94367283	0.795	0.87	6.95E-26	CST7	10	STMN1_T-cycling
3439	1.45E-42	-1.0528641	0.714	0.883	3.16E-38	HCST	10	STMN1_T-cycling
3440	6.02E-13	-1.08823381	0.13	0.385	1.31E-08	KLRF1	10	STMN1_T-cycling
3441	2.00E-09	-1.09413458	0.267	0.417	4.36E-05	SPON2	10	STMN1_T-cycling
3442	5.44E-26	-1.24406496	0.435	0.675	1.19E-21	CTSW	10	STMN1_T-cycling
3443	3.16E-20	-1.30787391	0.286	0.582	6.90E-16	KLRD1	10	STMN1_T-cycling
3444	8.17E-15	-1.38354241	0.236	0.475	1.78E-10	TYROBP	10	STMN1_T-cycling
3445	1.86E-34	-1.38557854	0.888	0.878	4.06E-30	NKG7	10	STMN1_T-cycling
3446	1.41E-19	-1.54563059	0.354	0.588	3.09E-15	CMC1	10	STMN1_T-cycling
3447	2.99E-25	-1.5771068	0.143	0.536	6.53E-21	GZMH	10	STMN1_T-cycling
3448	2.65E-09	-1.62073701	0.118	0.316	5.78E-05	XCL2	10	STMN1_T-cycling
3449	1.65E-19	-1.67671924	0.106	0.45	3.61E-15	GZMB	10	STMN1_T-cycling
3450	0	2.26597471	0.805	0.009	0	TRDV2	11	TRDV2_γδ T
3451	2.39E-95	1.62790185	0.748	0.133	5.21E-91	TRGV9	11	TRDV2_γδ T
3452	2.41E-35	1.10868009	0.545	0.148	5.27E-31	KLRC1	11	TRDV2_γδ T
3453	2.82E-18	0.88151271	0.463	0.175	6.15E-14	TRDC	11	TRDV2_γδ T
3454	9.58E-25	0.84151636	0.577	0.217	2.09E-20	TRAC	11	TRDV2_γδ T
3455	2.14E-15	0.56476097	0.463	0.179	4.66E-11	CD27	11	TRDV2_γδ T
3456	2.32E-15	0.5395634	0.683	0.321	5.05E-11	KLRG1	11	TRDV2_γδ T
3457	1.64E-13	0.48508881	0.837	0.499	3.57E-09	CD3D	11	TRDV2_γδ T
3458	1.66E-08	0.45688545	0.805	0.589	0.000361914	CD52	11	TRDV2_γδ T
3459	1.21E-09	0.44321625	0.22	0.074	2.65E-05	ITM2C	11	TRDV2_γδ T
3460	3.04E-10	0.41191362	0.642	0.364	6.63E-06	CD3G	11	TRDV2_γδ T
3461	1.55E-08	0.38384005	0.74	0.457	0.00033757	GZMK	11	TRDV2_γδ T
3462	5.96E-09	0.3555534	0.154	0.044	0.00012993	SPG20	11	TRDV2_γδ T
3463	3.73E-11	0.28575373	0.984	0.955	8.14E-07	RPS2	11	TRDV2_γδ T
3464	1.41E-08	0.28071908	0.179	0.056	0.000306663	CXCR3	11	TRDV2_γδ T
3465	7.77E-09	0.28067186	0.13	0.033	0.000169457	BTLA	11	TRDV2_γδ T
3466	1.88E-09	0.26728549	0.171	0.049	4.10E-05	IL12RB2	11	TRDV2_γδ T
3467	2.96E-08	0.25662061	1	0.963	0.00064588	RPSA	11	TRDV2_γδ T
3468	4.74E-15	0.25279277	0.992	0.997	1.03E-10	RPS12	11	TRDV2_γδ T
3469	4.01E-09	-0.77433413	0.163	0.429	8.74E-05	CD8A	11	TRDV2_γδ T
3470	4.85E-09	-0.81825335	0.22	0.474	0.000105663	TYROBP	11	TRDV2_γδ T
3471	1.85E-08	-1.0766866	0.122	0.347	0.000403323	FCGR3A	11	TRDV2_γδ T
3472	3.87E-12	-1.16690647	0.341	0.587	8.45E-08	CMC1	11	TRDV2_γδ T

Table 15: List of DEGs of NK/T cell clusters in reperfusion stage (PR versus EP)

List of differentially expressed genes of CD8B_CD8 Tem in reperfusion stage (PR versus EP)					
gene	p_val	avg_logFC	pct.1	pct.2	p_val_adj
HSPD1	8.05E-51	2.222933777	0.711	0.251	1.76E-46
ACTB	1.63E-45	-0.589868612	0.963	1	3.55E-41
CREM	5.04E-43	1.236624637	0.678	0.199	1.10E-38
HSPH1	2.34E-38	1.74646851	0.579	0.157	5.09E-34
PFN1	1.28E-36	-0.601476178	0.868	0.982	2.79E-32
SRGN	3.52E-36	0.646933929	0.985	0.944	7.68E-32
HSP90AA1	7.47E-36	1.540630962	0.923	0.819	1.63E-31
HSPE1	4.73E-32	1.623968582	0.718	0.4	1.03E-27
MYL12A	2.70E-31	-0.738624215	0.608	0.884	5.88E-27
HSPB1	2.95E-30	1.391570374	0.597	0.23	6.43E-26
FAM177A1	6.94E-30	1.131688519	0.564	0.188	1.51E-25
CORO1A	1.26E-29	-0.851627765	0.458	0.785	2.74E-25
LDHA	7.63E-27	0.84027034	0.711	0.378	1.66E-22
LITAF	2.68E-26	0.827102215	0.733	0.389	5.84E-22
HLA-B	2.90E-26	0.380769826	1	0.996	6.32E-22
HSP90AB1	1.35E-25	0.984542631	0.868	0.676	2.95E-21
SARAF	2.58E-25	0.577287326	0.945	0.83	5.62E-21
GZMA	8.46E-24	-0.630942791	0.615	0.859	1.85E-19
CCND3	1.16E-23	-0.795979519	0.278	0.647	2.53E-19
HBB	1.26E-22	-1.152208716	0.088	0.432	2.76E-18
DEFA3	3.29E-22	-1.089053543	0.051	0.378	7.18E-18
RPS29	4.33E-22	0.406228788	0.985	0.962	9.44E-18
BATF	1.58E-21	0.93330058	0.52	0.213	3.45E-17
HCST	3.74E-21	-0.535016737	0.681	0.902	8.17E-17
TNFAIP3	1.35E-20	0.734567212	0.637	0.3	2.94E-16
ATP1B3	1.51E-20	0.950103416	0.392	0.107	3.28E-16
PPP1R18	1.94E-20	-0.887145114	0.147	0.474	4.23E-16
SH2D2A	1.33E-19	0.823427049	0.469	0.177	2.89E-15
CD7	3.49E-19	0.820749222	0.696	0.443	7.61E-15
H3F3B	1.19E-18	0.46540319	0.993	0.931	2.59E-14
IGLV2-14	1.22E-18	-0.886812772	0.04	0.313	2.66E-14
FTH1	1.54E-18	0.7771178	0.952	0.928	3.35E-14
RELB	7.51E-18	0.74837701	0.245	0.036	1.64E-13
NOP58	8.44E-18	0.799171606	0.469	0.188	1.84E-13
PTPRCAP	4.76E-17	-0.513656173	0.685	0.837	1.04E-12
GZMB	8.39E-17	1.1878217	0.333	0.098	1.83E-12
AIM1	1.24E-16	0.796896877	0.462	0.186	2.71E-12
SYAP1	5.41E-16	0.825821332	0.355	0.112	1.18E-11
PRF1	7.39E-16	0.872442775	0.553	0.32	1.61E-11
HMGB2	3.22E-15	0.868052224	0.48	0.235	7.03E-11
LMNA	7.16E-15	0.81495689	0.216	0.036	1.56E-10
HSPA1A	7.52E-15	1.497939655	0.656	0.432	1.64E-10
CLIC1	1.20E-14	-0.516054917	0.491	0.711	2.62E-10
EML4	3.84E-14	0.64558613	0.509	0.257	8.38E-10
GPSM3	4.08E-14	-0.646995883	0.282	0.541	8.91E-10
TNFRSF9	4.62E-14	0.739828182	0.264	0.065	1.01E-09
LEPROTL1	7.21E-14	0.684974729	0.597	0.367	1.57E-09
EZR	1.16E-13	0.778797977	0.549	0.32	2.53E-09
DENND2D	1.24E-13	-0.819726694	0.125	0.369	2.71E-09
PIK3R1	1.79E-13	0.577709406	0.696	0.452	3.89E-09
CKLF	3.35E-13	-0.669145027	0.176	0.432	7.30E-09
FKBP4	3.38E-13	0.82855746	0.238	0.058	7.38E-09
DNAJB6	4.50E-13	0.747762081	0.509	0.28	9.82E-09
PTMA	6.14E-13	0.31719806	1	0.971	1.34E-08
SOCS3	6.92E-13	0.661181578	0.278	0.083	1.51E-08
PDLIM2	6.99E-13	-0.680935072	0.062	0.277	1.52E-08
SLC7A5	7.06E-13	0.737894538	0.267	0.078	1.54E-08
SOD1	7.11E-13	0.699227052	0.656	0.445	1.55E-08
IFITM1	7.81E-13	0.473897394	0.901	0.861	1.70E-08
GPR171	1.24E-12	0.702940472	0.3	0.098	2.71E-08
PFKFB3	3.12E-12	0.611270615	0.201	0.04	6.80E-08
S100A4	3.15E-12	-0.524164258	0.535	0.736	6.86E-08
IGKV4-1	4.70E-12	-0.728815577	0.084	0.302	1.02E-07
IL10	8.65E-12	1.064871893	0.154	0.02	1.89E-07
TBC1D10C	9.25E-12	-0.624894925	0.147	0.383	2.02E-07
CD3E	1.05E-11	-0.335953335	0.868	0.931	2.30E-07
SLC9A3R1	1.28E-11	-0.54748326	0.22	0.463	2.78E-07

DDX24	1.51E-11	0.5450586	0.582	0.349	3.30E-07
MTHFD2	1.69E-11	0.712756052	0.311	0.121	3.69E-07
SH3BGRL3	1.78E-11	-0.351588899	0.725	0.89	3.89E-07
CNOT6L	2.11E-11	0.61865655	0.469	0.255	4.61E-07
IFITM2	2.34E-11	0.590850772	0.795	0.689	5.10E-07
S100A8	5.49E-11	-0.731778063	0.168	0.418	1.20E-06
BCL3	6.44E-11	0.486286361	0.147	0.02	1.40E-06
SH3BP1	7.81E-11	-0.625599727	0.04	0.215	1.70E-06
SELK	8.98E-11	0.596133987	0.491	0.293	1.96E-06
ACAP1	9.90E-11	-0.559701269	0.275	0.506	2.16E-06
PPP1CB	1.03E-10	0.568022692	0.333	0.136	2.25E-06
STAT3	1.09E-10	0.568849332	0.476	0.264	2.37E-06
ISG20	1.28E-10	0.546420566	0.659	0.499	2.80E-06
ARID5A	1.37E-10	0.616183705	0.363	0.161	2.98E-06
PTPN6	1.68E-10	-0.557245876	0.081	0.28	3.66E-06
TRAF3IP3	1.69E-10	-0.60243535	0.114	0.324	3.68E-06
GZMK	1.90E-10	-0.40602238	0.667	0.828	4.15E-06
LCK	2.01E-10	-0.486736568	0.326	0.55	4.39E-06
LAT	2.30E-10	-0.531661218	0.147	0.365	5.01E-06
USP36	2.54E-10	0.549043863	0.26	0.087	5.53E-06
TSPYL2	3.24E-10	0.647050401	0.513	0.302	7.06E-06
IGKV3-15	3.86E-10	-0.632172913	0.007	0.148	8.42E-06
IRF1	3.86E-10	0.58388313	0.513	0.306	8.43E-06
ODC1	4.29E-10	0.578678969	0.355	0.163	9.35E-06
ODF3B	4.78E-10	0.435509727	0.143	0.022	1.04E-05
SRRT	5.20E-10	0.586735227	0.436	0.235	1.13E-05
CACYBP	5.77E-10	0.811280094	0.407	0.219	1.26E-05
C19orf60	6.37E-10	-0.554847421	0.165	0.376	1.39E-05
MAP3K8	7.12E-10	0.646209639	0.396	0.206	1.55E-05
RHBDD2	8.02E-10	0.472842986	0.267	0.092	1.75E-05
ITGB2	8.04E-10	-0.48646711	0.37	0.593	1.75E-05
LIME1	9.91E-10	-0.551216404	0.15	0.358	2.16E-05
EIF1	1.19E-09	0.259879053	0.989	0.982	2.59E-05
UBB	1.38E-09	0.421878434	0.872	0.718	3.01E-05
ANXA1	1.65E-09	0.758998004	0.542	0.331	3.59E-05
TAGLN2	1.73E-09	0.575039782	0.575	0.383	3.76E-05
ARID5B	1.97E-09	0.561897064	0.286	0.11	4.29E-05
CD27	2.15E-09	-0.570932364	0.183	0.391	4.69E-05
RAC2	2.81E-09	-0.379392757	0.509	0.711	6.12E-05
GABARAPL1	3.24E-09	0.552469159	0.26	0.098	7.07E-05
SMAP2	3.33E-09	0.459766817	0.392	0.195	7.25E-05
RBL2	3.35E-09	-0.620287771	0.117	0.298	7.30E-05
PLEK	3.72E-09	-0.540442519	0.114	0.304	8.10E-05
JUN	4.59E-09	-0.412219455	0.736	0.848	0.00010007
TXNIP	4.83E-09	-0.327638798	0.612	0.823	0.000105425
LCP2	4.90E-09	-0.495666582	0.231	0.447	0.000106765
TMA7	5.84E-09	-0.335040986	0.729	0.866	0.000127302
MT-ATP8	6.60E-09	0.306540718	0.96	0.922	0.000143993
TIFA	6.76E-09	0.473783372	0.158	0.036	0.000147362
COX7A2L	7.25E-09	-0.508572525	0.154	0.349	0.000158129
SERPINB9	7.67E-09	0.489588675	0.264	0.105	0.000167161
RNF19A	8.23E-09	0.530479478	0.377	0.192	0.000179494
PGK1	1.04E-08	0.615636411	0.527	0.374	0.000226847
CD99	1.63E-08	-0.301915263	0.681	0.832	0.000356027
GSPT1	1.91E-08	0.495785499	0.209	0.069	0.00041683
APOBEC3G	2.47E-08	-0.532903382	0.099	0.268	0.000539227
ELL2	2.81E-08	0.443176483	0.194	0.06	0.000613886
LYST	2.82E-08	0.459981636	0.436	0.257	0.000615884
AC092580.4	2.84E-08	0.586357186	0.249	0.101	0.000619227
MCL1	2.87E-08	0.479588436	0.608	0.445	0.000625429
ATP6V0C	3.13E-08	0.383204112	0.593	0.38	0.000682517
NFKBIA	3.34E-08	0.432196948	0.773	0.613	0.000728693
LSP1	3.46E-08	-0.443889986	0.399	0.564	0.000754967
EMD	4.44E-08	0.480293206	0.388	0.213	0.000968058
SKAP1	5.12E-08	-0.465963048	0.238	0.434	0.001115663
NUDC	5.21E-08	0.587310914	0.322	0.154	0.001135398
PRDM1	5.40E-08	0.590692053	0.469	0.298	0.001176898
GIMAP1	5.90E-08	-0.504461395	0.168	0.353	0.001286233
IGLV2-23	6.83E-08	-0.5950803	0.062	0.21	0.001490246
HSPA5	7.38E-08	0.468966806	0.56	0.391	0.001610331

PPP1R16B	8.55E-08	0.502735353	0.165	0.049	0.00186396
GBP2	8.56E-08	0.472894711	0.264	0.116	0.001866991
RAP1GDS1	8.58E-08	-0.436933054	0.055	0.201	0.001870518
DDIT4	9.15E-08	0.374784969	0.766	0.64	0.001994359
MCOLN2	1.04E-07	0.44883009	0.212	0.076	0.002271519
CFL1	1.11E-07	-0.250730961	0.81	0.89	0.002426915
FAM173A	1.45E-07	-0.440163413	0.022	0.141	0.003154342
TRAC	1.55E-07	-0.508758533	0.128	0.293	0.003384714
BIRC3	1.79E-07	0.510729252	0.267	0.121	0.003894042
PDE4D	1.91E-07	0.388314675	0.168	0.051	0.004164837
EVL	2.16E-07	-0.41712894	0.366	0.539	0.00471675
SASH3	2.29E-07	-0.476403439	0.117	0.28	0.004991678
DHCR7	2.47E-07	0.357862011	0.128	0.029	0.005383793
SBNO2	2.49E-07	0.385509046	0.114	0.022	0.005434075
IGKV2-30	3.09E-07	-0.525873718	0.088	0.235	0.006732602
BAZ1A	3.17E-07	0.451241708	0.363	0.197	0.006919927
SPOCK2	3.30E-07	0.47470438	0.33	0.177	0.007194046
DDX21	3.67E-07	0.500850275	0.366	0.213	0.008003832
SDCBP	4.11E-07	0.462549049	0.377	0.219	0.008972121
TUBB4B	4.38E-07	0.371098309	0.637	0.459	0.00954722
NFATC1	4.41E-07	0.437886991	0.139	0.038	0.009622219
RCSD1	4.43E-07	-0.442956477	0.143	0.304	0.009666
SIT1	4.99E-07	-0.498580134	0.092	0.237	0.010879848
DCK	5.28E-07	-0.44849156	0.092	0.242	0.011517805
ROMO1	5.46E-07	0.450841187	0.436	0.275	0.01190682
PDCL3	5.96E-07	0.448988245	0.212	0.085	0.013005659
CSK	6.24E-07	-0.423076447	0.128	0.289	0.01360364
ALB	7.01E-07	0.31691141	0.179	0.06	0.015297503
CD3D	7.32E-07	-0.292590285	0.597	0.749	0.015969612
SC5D	7.33E-07	0.446523837	0.176	0.06	0.015988497
AREG	8.72E-07	0.652270193	0.425	0.264	0.019021409
NAMPT	8.80E-07	0.453832901	0.249	0.114	0.019196602
FAM49B	9.43E-07	-0.396824488	0.22	0.405	0.020566501
1-Sep	9.76E-07	-0.405511339	0.3	0.481	0.021286509
HMGB1	1.06E-06	-0.30702836	0.656	0.79	0.023081957
CISH	1.11E-06	-0.375879526	0.015	0.114	0.024259642
WAS	1.19E-06	-0.458641508	0.201	0.36	0.025998862
DDX17	1.24E-06	-0.393056249	0.238	0.412	0.02712774
MYADM	1.36E-06	0.55283504	0.388	0.251	0.029615685
RGS14	1.45E-06	-0.410005282	0.077	0.215	0.031687554
TNFRSF1A	1.52E-06	-0.378760395	0.018	0.121	0.03317113
UPF3A	1.58E-06	-0.395955582	0.037	0.15	0.034483436
DUSP1	1.59E-06	-0.288605299	0.908	0.917	0.034579743
GPBP1	1.63E-06	0.508077262	0.451	0.306	0.035564928
BAG3	1.70E-06	0.423357035	0.117	0.029	0.036968557
IFITM3	1.73E-06	0.399158494	0.136	0.04	0.037769967
OAZ1	1.74E-06	0.30166557	0.839	0.792	0.037946705
RANGAP1	1.80E-06	0.406745815	0.132	0.038	0.039317421
PTGDR	1.84E-06	-0.422081006	0.04	0.154	0.040159684
PCBP1	1.94E-06	-0.317159927	0.454	0.6	0.042389465
KIAA1551	1.96E-06	-0.439747044	0.278	0.441	0.042718883
SIRPG	1.98E-06	-0.451741155	0.066	0.192	0.043144531
REL	2.15E-06	0.421278374	0.388	0.233	0.04684517

List of differentially expressed genes of GNLY_NK in reperfusion stage (PR versus EP)

gene	p_val	avg_logFC	pct.1	pct.2	p_val_adj
LAIR2	2.28E-25	1.216642086	0.456	0.105	4.97E-21
GBP2	1.37E-23	0.911236414	0.569	0.19	2.98E-19
SDCBP	1.20E-21	0.938274798	0.638	0.281	2.63E-17
CORO1A	1.16E-20	-0.768494009	0.575	0.842	2.54E-16
HLA-B	2.12E-20	0.308211162	1	1	4.62E-16
NFKBIA	3.40E-19	0.751977156	0.912	0.654	7.41E-15
SRGN	3.55E-18	0.487186714	0.988	0.978	7.75E-14
RELB	4.92E-18	0.827579867	0.356	0.087	1.07E-13
DEFA3	7.63E-18	-1.122473163	0.031	0.393	1.66E-13
HBB	1.07E-17	-1.350903235	0.081	0.451	2.34E-13
CST7	2.12E-17	0.472482768	1	0.982	4.63E-13

RPS29	3.02E-17	0.453179536	0.975	0.923	6.58E-13
CCL4	6.44E-17	0.880235251	0.944	0.907	1.40E-12
PTPRCAP	7.19E-16	-0.546631697	0.744	0.915	1.57E-11
FCER1G	1.26E-15	1.029947177	0.312	0.073	2.74E-11
ACTB	1.76E-15	-0.361531353	1	1	3.84E-11
NFKB2	2.35E-15	0.629945235	0.262	0.049	5.13E-11
CD3E	3.62E-15	-0.561809148	0.481	0.834	7.89E-11
GZMB	8.02E-15	0.527474122	0.975	0.937	1.75E-10
MT-ATP6	1.06E-14	-0.309686905	0.988	0.99	2.31E-10
SLC9A3R1	3.25E-14	-0.677910947	0.35	0.66	7.08E-10
HIF1A	3.57E-13	0.764317701	0.419	0.16	7.79E-09
TNFAIP3	5.97E-13	0.657958335	0.712	0.445	1.30E-08
ZBTB16	9.67E-13	0.566612976	0.181	0.026	2.11E-08
HSPB1	9.97E-13	1.072768514	0.481	0.223	2.17E-08
GZMA	1.69E-12	-0.433396423	0.775	0.947	3.69E-08
LCK	6.61E-12	-0.609976038	0.344	0.638	1.44E-07
BATF	7.92E-12	0.752323798	0.531	0.294	1.73E-07
HSPD1	8.89E-12	1.495735643	0.525	0.296	1.94E-07
GNLY	1.15E-11	-0.309055698	0.919	1	2.50E-07
SYTL3	1.20E-11	0.53601358	0.738	0.518	2.61E-07
GZMH	2.29E-11	-0.423485316	0.75	0.925	4.99E-07
DUSP1	2.48E-11	-0.562917627	0.744	0.885	5.41E-07
PRDM1	2.99E-11	0.593417237	0.688	0.451	6.52E-07
PTGDS	3.32E-11	1.091454054	0.244	0.061	7.25E-07
PFN1	4.25E-11	-0.299837749	0.988	0.996	9.26E-07
BCL3	4.49E-11	0.482723033	0.156	0.022	9.79E-07
H3F3B	5.32E-11	0.30785705	0.981	0.97	1.16E-06
ISG20	5.86E-11	0.504133254	0.794	0.593	1.28E-06
RGS19	8.09E-11	-0.786967768	0.05	0.294	1.76E-06
SH3BGRL3	2.53E-10	-0.34780161	0.906	0.974	5.52E-06
CADM1	6.95E-10	0.551813449	0.2	0.047	1.52E-05
MAP3K8	7.01E-10	0.672169179	0.6	0.393	1.53E-05
GSTP1	7.16E-10	-0.484474395	0.519	0.757	1.56E-05
ACTG1	8.20E-10	-0.351265211	0.962	0.974	1.79E-05
TBC1D10C	8.45E-10	-0.61510488	0.175	0.449	1.84E-05
S100A4	9.11E-10	-0.369258322	0.881	0.974	1.99E-05
IRF8	1.15E-09	0.52650287	0.206	0.051	2.52E-05
EIF1	2.29E-09	0.265043381	0.988	0.984	4.99E-05
HPGD	3.13E-09	0.577022673	0.188	0.045	6.84E-05
LDHA	4.07E-09	0.534125318	0.656	0.457	8.88E-05
STOM	4.63E-09	0.57469389	0.538	0.334	0.000100953
SH3BP1	4.85E-09	-0.627827544	0.088	0.324	0.000105757
SH2D2A	4.91E-09	0.636881947	0.531	0.33	0.000107076
LAT	5.88E-09	-0.601871502	0.169	0.423	0.000128135
IGLV2-14	6.99E-09	-0.679230387	0.1	0.338	0.000152504
TRBC1	8.47E-09	-0.408642063	0.65	0.86	0.000184675
KIR2DL3	8.52E-09	0.547722771	0.394	0.178	0.000185726
SQSTM1	1.25E-08	0.4793744	0.762	0.597	0.000273348
CCND3	2.08E-08	-0.412493324	0.488	0.743	0.000453176
GADD45B	2.21E-08	0.485312103	0.856	0.757	0.000481314
PNRC1	3.26E-08	0.421505556	0.831	0.686	0.000710734
TRAF3IP3	4.98E-08	-0.513972305	0.212	0.453	0.001086604
SLFN5	5.12E-08	-0.617882803	0.056	0.257	0.00111729
CNN2	5.57E-08	-0.573756108	0.131	0.354	0.001214115
CCL3	6.27E-08	0.683328265	0.712	0.508	0.001366416
EGR2	6.96E-08	0.457342875	0.119	0.02	0.001518636
ICAM1	8.11E-08	0.552684788	0.262	0.101	0.001767707
BIN2	8.61E-08	-0.484278021	0.362	0.593	0.001877656
CD63	8.76E-08	-0.493066948	0.369	0.595	0.001911134
RASSF1	9.73E-08	-0.536063078	0.188	0.421	0.00212089
SMAP2	1.11E-07	0.500550656	0.362	0.174	0.002417465
CSK	1.31E-07	-0.591163073	0.15	0.364	0.002855067
RAC2	1.75E-07	-0.345808816	0.744	0.866	0.003818483
ACAP1	1.95E-07	-0.590124587	0.194	0.409	0.004243444
MYL12A	2.00E-07	-0.310670381	0.869	0.941	0.004356239
TSC22D4	2.04E-07	-0.539610157	0.119	0.328	0.004440964
FOSB	2.24E-07	-0.647186457	0.269	0.484	0.004876772
CCL4L2	2.62E-07	0.966338275	0.775	0.694	0.005705627
ACTR3	2.66E-07	-0.465073301	0.456	0.662	0.005799922
IGLV2-23	2.85E-07	-0.63578709	0.031	0.206	0.006224854

LSP1	3.00E-07	-0.379319482	0.562	0.771	0.006550335
CKLF	3.40E-07	-0.549060837	0.15	0.352	0.007405077
S100A8	3.92E-07	-0.590664203	0.225	0.447	0.008544464
KIR3DL2	5.74E-07	0.615341782	0.294	0.134	0.012514215
SNAP23	5.97E-07	0.44132595	0.3	0.13	0.013017106
SBNO2	7.44E-07	0.358490839	0.112	0.022	0.016227326
SLA2	8.72E-07	0.47958875	0.369	0.192	0.019025294
TTC38	8.78E-07	-0.508451272	0.231	0.443	0.019148813
1-Sep	9.26E-07	-0.467143841	0.194	0.403	0.020197055
ANKRD28	1.02E-06	0.4884527	0.362	0.184	0.022313333
BIN1	1.04E-06	-0.480202656	0.156	0.354	0.022658652
FKBP4	1.14E-06	0.737022474	0.206	0.077	0.024923923
AGTRAP	1.27E-06	-0.546227945	0.131	0.318	0.027643746
CSNK1D	1.32E-06	0.464733012	0.356	0.186	0.028841675
PIM1	1.34E-06	0.496634991	0.5	0.314	0.029229165
GBP4	1.47E-06	0.422481033	0.288	0.126	0.032108168
HCST	1.48E-06	-0.327835084	0.844	0.903	0.032190838
KIR2DL4	1.51E-06	0.415650131	0.131	0.032	0.032902659
C9orf142	1.52E-06	-0.465922358	0.45	0.625	0.03311928
NDUFB7	1.69E-06	-0.452264018	0.169	0.389	0.036883362
HSPH1	1.69E-06	1.283876553	0.381	0.235	0.036901903
TBXAS1	1.93E-06	0.462444486	0.138	0.036	0.042125471
NFKB1	1.97E-06	0.38178623	0.188	0.061	0.042925267

List of differentially expressed genes of CCL20_CD8 MAIT in reperfusion stage (PR versus EP)

gene	p_val	avg_logFC	pct.1	pct.2	p_val_adj
RELB	3.54E-45	1.352257968	0.683	0.114	7.73E-41
NFKB1	8.62E-42	1.315078632	0.658	0.112	1.88E-37
BATF	1.57E-40	1.54123061	0.792	0.261	3.41E-36
NFKB2	1.04E-35	1.157677184	0.617	0.118	2.26E-31
TNFRSF18	1.29E-34	0.881495513	0.383	0.021	2.81E-30
CD7	1.38E-33	0.899609782	0.983	0.819	3.01E-29
CREM	6.00E-32	1.166597527	0.817	0.337	1.31E-27
HSPD1	1.48E-30	1.804722976	0.767	0.309	3.22E-26
HLA-B	2.57E-29	0.510095423	1	0.996	5.60E-25
SLC7A5	6.48E-29	0.995233998	0.758	0.28	1.41E-24
HSP90AA1	8.75E-29	1.542809929	0.95	0.853	1.91E-24
NINJ1	1.34E-28	1.095716948	0.567	0.128	2.92E-24
SERPINB9	1.36E-28	1.08911042	0.658	0.204	2.97E-24
HLA-A	5.09E-28	0.539012923	1	0.992	1.11E-23
SRGN	1.34E-27	0.795841642	0.975	0.96	2.93E-23
TXNIP	3.47E-27	-1.083605532	0.408	0.882	7.56E-23
MYL12A	2.39E-25	-0.7446495	0.833	0.958	5.22E-21
FEZ1	3.36E-25	1.129316497	0.567	0.156	7.33E-21
FAM177A1	3.65E-24	1.21891897	0.7	0.312	7.95E-20
CORO1A	1.29E-23	-0.841944148	0.633	0.907	2.82E-19
SYAP1	5.99E-23	1.04109338	0.592	0.179	1.31E-18
FURIN	6.60E-23	0.802690696	0.333	0.038	1.44E-18
PBX4	3.30E-22	1.035846314	0.642	0.253	7.19E-18
SYTL3	2.88E-20	0.838504044	0.842	0.497	6.29E-16
GNG2	3.81E-20	0.821553517	0.875	0.6	8.31E-16
TNFRSF4	6.78E-20	0.665278206	0.2	0.006	1.48E-15
BACH2	8.14E-20	0.557959357	0.25	0.019	1.78E-15
CCND3	8.19E-20	-0.973636759	0.242	0.697	1.79E-15
HSPE1	9.86E-20	1.409826444	0.775	0.501	2.15E-15
S100A4	2.56E-19	-0.508035538	0.933	0.994	5.57E-15
FTH1	2.80E-19	1.098986069	0.958	0.973	6.10E-15
BIRC3	8.37E-19	0.913736253	0.583	0.202	1.82E-14
LMNA	1.08E-18	0.930577651	0.333	0.057	2.35E-14
HSP90AB1	3.50E-18	0.836527447	0.942	0.8	7.64E-14
PFKFB3	5.86E-18	0.805031109	0.533	0.179	1.28E-13
TNFRSF1B	1.28E-17	0.803327491	0.608	0.236	2.80E-13
MIR155HG	3.67E-17	0.886049418	0.283	0.04	8.00E-13
DUSP1	4.35E-17	-0.511488575	0.933	0.981	9.48E-13
ACTB	7.19E-17	-0.419903782	1	1	1.57E-12
TRAF3IP3	2.41E-16	-0.931514133	0.1	0.52	5.26E-12
TNFRSF9	3.20E-16	0.581202124	0.192	0.013	6.99E-12

SNX9	3.59E-16	0.809629105	0.442	0.131	7.84E-12
EVL	4.78E-16	-0.72908588	0.342	0.72	1.04E-11
CCL20	5.10E-16	1.414884803	0.567	0.215	1.11E-11
PIM2	7.97E-16	0.884610699	0.608	0.28	1.74E-11
DENND2D	8.33E-16	-0.946562747	0.092	0.501	1.82E-11
STAT4	9.00E-16	0.743231593	0.733	0.427	1.96E-11
PDE4A	1.04E-15	0.592589147	0.217	0.021	2.27E-11
EML4	1.44E-15	0.709137597	0.775	0.48	3.13E-11
KLRG1	1.63E-15	-0.724824907	0.55	0.794	3.56E-11
IRF8	2.58E-15	0.672838801	0.35	0.08	5.62E-11
FOXO1	3.18E-15	0.700267721	0.342	0.078	6.94E-11
LCK	3.65E-15	-0.772820484	0.325	0.699	7.95E-11
ELOVL5	4.12E-15	0.774249249	0.692	0.387	8.98E-11
PTPN6	9.01E-15	-0.859119267	0.083	0.491	1.96E-10
PTPRCAP	1.06E-14	-0.541796533	0.808	0.931	2.32E-10
DENND4A	2.57E-14	0.701287912	0.383	0.103	5.60E-10
DEFA3	2.78E-14	-0.983500795	0.042	0.413	6.05E-10
SLC9A3R1	3.70E-14	-0.79572804	0.15	0.543	8.06E-10
GPR171	4.21E-14	0.838586708	0.658	0.352	9.18E-10
REL	5.54E-14	0.897758006	0.742	0.448	1.21E-09
SH3BGRL3	6.85E-14	-0.528049942	0.85	0.958	1.49E-09
KDM6B	8.21E-14	0.669564374	0.508	0.192	1.79E-09
TRAF4	8.89E-14	0.44022101	0.192	0.019	1.94E-09
SEC14L1	1.25E-13	0.766467847	0.417	0.135	2.73E-09
GZMA	2.05E-13	-0.537823481	0.783	0.916	4.47E-09
IFNG	2.36E-13	1.179495813	0.633	0.305	5.15E-09
LITAF	2.58E-13	0.717528256	0.75	0.459	5.63E-09
SOCS3	3.09E-13	0.689868353	0.508	0.196	6.75E-09
RPS29	3.32E-13	0.402622065	0.975	0.975	7.25E-09
SH2D2A	3.90E-13	0.774217344	0.675	0.387	8.51E-09
OTULIN	5.24E-13	0.51013363	0.258	0.048	1.14E-08
LYST	7.38E-13	0.666387178	0.467	0.175	1.61E-08
GPBP1	8.19E-13	0.637958651	0.775	0.472	1.79E-08
H3F3B	1.14E-12	0.453790782	1	0.987	2.48E-08
HIF1A	1.47E-12	0.834060549	0.525	0.236	3.20E-08
BCL3	1.75E-12	0.568348613	0.275	0.059	3.82E-08
CKLF	1.79E-12	-0.65119725	0.242	0.611	3.91E-08
JUN	2.04E-12	-0.527876296	0.767	0.968	4.46E-08
STAT3	2.34E-12	0.6997497	0.567	0.282	5.10E-08
TRABD2A	2.69E-12	0.562911552	0.192	0.025	5.88E-08
PIM3	3.51E-12	0.646216088	0.35	0.103	7.66E-08
TBC1D10C	3.96E-12	-0.744184539	0.142	0.499	8.64E-08
TANK	4.25E-12	0.727724289	0.55	0.259	9.27E-08
GZMB	4.66E-12	0.680360193	0.217	0.036	1.02E-07
BHLHE40	5.72E-12	0.604518259	0.617	0.299	1.25E-07
HOPX	6.23E-12	-0.675294644	0.258	0.589	1.36E-07
MAP3K8	6.31E-12	0.636520593	0.683	0.364	1.38E-07
HSP90B1	6.73E-12	0.713437731	0.775	0.537	1.47E-07
GIMAP4	9.03E-12	-0.701238936	0.275	0.634	1.97E-07
HSPA5	1.10E-11	0.584716842	0.758	0.493	2.39E-07
STARD7	1.64E-11	0.710110634	0.467	0.2	3.58E-07
MT2A	1.96E-11	0.96997981	0.95	0.796	4.26E-07
HSPH1	2.22E-11	0.980126647	0.45	0.194	4.84E-07
TRAF1	3.00E-11	0.687286741	0.467	0.189	6.53E-07
ODC1	3.63E-11	0.609630987	0.367	0.118	7.92E-07
PPP1R18	3.89E-11	-0.684723308	0.2	0.549	8.48E-07
RORA	4.02E-11	0.598757048	0.8	0.556	8.76E-07
CD44	4.41E-11	0.621069326	0.717	0.478	9.62E-07
SARAF	4.51E-11	0.40119833	0.95	0.928	9.84E-07
TSC22D4	4.53E-11	-0.693908175	0.067	0.389	9.87E-07
TRAF3	5.58E-11	0.437196098	0.167	0.021	1.22E-06
HBB	1.30E-10	-0.893636452	0.092	0.406	2.84E-06
BCL2A1	1.38E-10	0.780146024	0.542	0.269	3.01E-06
ATP1B3	1.45E-10	0.644022602	0.325	0.103	3.16E-06
ACAP1	1.67E-10	-0.641643162	0.325	0.642	3.63E-06
BCL2	1.80E-10	-0.779796766	0.058	0.352	3.93E-06
GIMAP1	1.88E-10	-0.726886498	0.142	0.453	4.10E-06
EZR	2.16E-10	0.649910293	0.675	0.408	4.71E-06
GIMAP7	3.04E-10	-0.681975079	0.183	0.512	6.62E-06
CD3E	3.37E-10	-0.352401776	0.908	0.973	7.36E-06

PIM1	3.66E-10	0.626229412	0.7	0.474	7.97E-06
ZHX2	3.86E-10	0.56290588	0.275	0.074	8.43E-06
AC006129.2	3.96E-10	-0.68955794	0.133	0.448	8.64E-06
CD52	4.29E-10	-0.52561808	0.708	0.863	9.35E-06
GPR183	4.29E-10	0.634236961	0.358	0.126	9.36E-06
HSPA1A	4.41E-10	1.559239908	0.567	0.318	9.62E-06
THBS1	4.58E-10	0.371661006	0.133	0.013	9.98E-06
SMAP2	4.83E-10	0.605083361	0.567	0.312	1.05E-05
TNFAIP3	4.91E-10	0.515193877	0.883	0.737	1.07E-05
ARHGAP31	4.96E-10	0.348134471	0.158	0.021	1.08E-05
PPP1CB	5.98E-10	0.593609691	0.508	0.244	1.30E-05
GTF3C1	6.21E-10	0.625696566	0.433	0.185	1.35E-05
SATB1	8.81E-10	0.666998032	0.725	0.516	1.92E-05
ATF3	8.94E-10	0.758789427	0.45	0.187	1.95E-05
VAV1	1.10E-09	0.494723063	0.325	0.105	2.41E-05
MTHFD2	1.20E-09	0.562429979	0.392	0.154	2.63E-05
RBM17	1.30E-09	0.544326558	0.458	0.206	2.84E-05
PGK1	1.39E-09	0.663237662	0.667	0.448	3.03E-05
RBBP8	1.62E-09	0.352229311	0.133	0.015	3.54E-05
RUNX3	1.66E-09	0.561600165	0.567	0.301	3.62E-05
SDCBP	1.82E-09	0.567434603	0.583	0.309	3.97E-05
TIFA	2.19E-09	0.70125796	0.35	0.133	4.78E-05
SH3BP1	2.39E-09	-0.625178466	0.058	0.331	5.22E-05
GPCPD1	2.43E-09	0.596342882	0.375	0.149	5.30E-05
TNIP1	3.20E-09	0.535010885	0.492	0.244	6.97E-05
METRNL	3.60E-09	0.322553978	0.117	0.011	7.85E-05
PHLDB2	4.11E-09	0.309287398	0.108	0.008	8.97E-05
HCST	6.31E-09	-0.378488256	0.858	0.947	0.00013767
IL2RA	6.99E-09	0.317423482	0.133	0.017	0.000152341
STARD10	7.13E-09	0.336136536	0.133	0.017	0.000155537
PFN1	7.30E-09	-0.309596988	0.975	0.992	0.000159306
CPD	7.32E-09	0.51578242	0.325	0.116	0.000159675
DDX24	7.81E-09	0.587477516	0.683	0.465	0.000170353
HIVEP2	8.16E-09	0.548217098	0.283	0.093	0.000178034
DENND3	8.81E-09	0.442313727	0.175	0.034	0.000192135
STAM	1.01E-08	0.38785295	0.183	0.038	0.000219906
SPPL2A	1.04E-08	0.49529852	0.375	0.145	0.000227545
EIF5A	1.20E-08	0.533562532	0.642	0.408	0.00026151
C19orf60	1.22E-08	-0.634124626	0.133	0.411	0.000265986
PRMT2	1.22E-08	-0.539461556	0.217	0.516	0.000267042
NAMPT	1.27E-08	0.743808511	0.417	0.189	0.000276741
HLA-DQB1	1.48E-08	0.590235691	0.483	0.244	0.000323554
IL7R	1.53E-08	0.408366659	1	0.979	0.000333853
IL4R	1.90E-08	0.47377994	0.208	0.051	0.000413292
IL21R	2.13E-08	0.456531613	0.25	0.074	0.000465329
CAST	2.56E-08	0.594696674	0.617	0.385	0.00055742
PSTPIP2	2.65E-08	0.447007082	0.225	0.061	0.000578393
RNF115	2.68E-08	0.596277431	0.458	0.229	0.000583633
ELL2	2.81E-08	0.444103569	0.292	0.099	0.000613473
ISG20	2.83E-08	0.522290456	0.767	0.594	0.000618216
PDE4D	2.88E-08	0.57511942	0.425	0.198	0.000627471
BAZ1A	3.12E-08	0.521052013	0.533	0.297	0.000681418
SURF4	3.38E-08	0.507810504	0.517	0.265	0.0007363
PHLDA1	3.59E-08	0.616441416	0.417	0.181	0.000782549
CHD9	3.94E-08	-0.631087427	0.042	0.282	0.000859517
AC092580.4	4.14E-08	0.617316842	0.667	0.503	0.000903809
ANKRD28	4.21E-08	0.589542127	0.408	0.187	0.000918021
PYHIN1	4.48E-08	-0.59286637	0.15	0.419	0.000977426
AREG	4.61E-08	0.761832373	0.475	0.244	0.001006119
KIAA1551	5.45E-08	-0.615415027	0.2	0.461	0.001188974
IL16	6.20E-08	-0.534664878	0.125	0.387	0.001353015
POLR3E	6.58E-08	0.417801943	0.192	0.046	0.001435208
SAMSN1	7.22E-08	0.512962506	0.4	0.177	0.001574991
SCML4	9.44E-08	-0.533117851	0.25	0.509	0.002058955
LCP2	9.94E-08	-0.517700911	0.317	0.589	0.002168402
CD244	1.01E-07	-0.520432501	0.017	0.229	0.002198965
BIN2	1.07E-07	-0.559742086	0.267	0.514	0.002337653
PPP1R16B	1.08E-07	0.456784496	0.258	0.086	0.00235523
SYTL2	1.38E-07	0.542864967	0.583	0.354	0.0030107
TAGAP	1.41E-07	-0.540060056	0.575	0.752	0.003083212

TMC8	1.48E-07	-0.578038079	0.15	0.389	0.003227268
TNIP2	1.54E-07	0.423024368	0.217	0.063	0.003364739
ZBTB38	1.76E-07	-0.597602317	0.183	0.427	0.003830103
JAML	1.92E-07	-0.529809693	0.108	0.362	0.004189846
HSH2D	2.30E-07	0.47299675	0.233	0.074	0.005015926
CYB5A	2.52E-07	0.509546202	0.3	0.118	0.005490907
SYNGR2	2.75E-07	0.475507182	0.383	0.179	0.006000108
STX11	2.77E-07	0.423677312	0.242	0.078	0.006041806
EMD	2.82E-07	0.469019762	0.5	0.282	0.00614267
SH2D3A	2.84E-07	0.376606339	0.175	0.042	0.006186822
FLT3LG	2.91E-07	-0.562482799	0.1	0.343	0.006349644
MEAF6	3.33E-07	-0.538920881	0.142	0.383	0.007270278
PABPC1	3.56E-07	0.36758852	0.925	0.956	0.007763923
DDX21	3.71E-07	0.589641897	0.55	0.343	0.008096906
POR	3.80E-07	0.37431531	0.192	0.053	0.008285203
CDC42SE2	3.83E-07	0.484002949	0.692	0.459	0.008362595
IGLV2-14	3.87E-07	-0.569043992	0.05	0.267	0.008446142
ITGB2	4.06E-07	-0.508685717	0.433	0.621	0.008843453
THEMIS	4.18E-07	-0.539963075	0.133	0.385	0.009109102
ZC3H12A	4.19E-07	0.394422812	0.658	0.404	0.009132563
FYN	4.29E-07	0.398739566	0.783	0.688	0.009350543
CD74	4.72E-07	0.500074295	0.808	0.672	0.010292283
CAMK4	5.10E-07	0.461060005	0.642	0.413	0.01112585
HDAC7	5.84E-07	-0.494385296	0.008	0.194	0.012746182
TMIGD2	6.18E-07	-0.540550787	0.183	0.436	0.013483624
SYNJ2	7.02E-07	0.446152239	0.175	0.046	0.015303564
PDLIM2	7.24E-07	-0.566477593	0.1	0.32	0.015784296
JUND	7.60E-07	0.478500492	0.558	0.339	0.016567566
RANGAP1	7.82E-07	0.430359105	0.175	0.046	0.017063388
ARRB2	8.19E-07	-0.523907169	0.125	0.352	0.017852652
LGALS1	8.68E-07	0.661179906	0.35	0.16	0.01893153
ECE1	8.71E-07	0.384709287	0.158	0.038	0.019002243
DUSP2	8.77E-07	-0.342368019	0.933	0.962	0.019123758
CNOT6L	9.55E-07	0.490297327	0.5	0.291	0.020819116
CDKN1B	1.06E-06	-0.542250131	0.183	0.415	0.023148175
DHRS3	1.08E-06	0.395833726	0.308	0.126	0.02352358
SYNRG	1.09E-06	-0.532612029	0.142	0.375	0.023719336
IL32	1.10E-06	0.339294721	0.992	0.989	0.024049836
ODF3B	1.11E-06	0.386654423	0.133	0.027	0.024288205
CST7	1.12E-06	0.381195832	0.942	0.905	0.024324106
TNFRSF1A	1.15E-06	-0.502657418	0.067	0.28	0.025147518
ADGRE5	1.24E-06	0.463936198	0.758	0.648	0.027108442
CASP1	1.25E-06	-0.492817673	0.225	0.463	0.027202073
SAMHD1	1.26E-06	-0.507074416	0.092	0.309	0.027566302
CENPM	1.31E-06	0.261809056	0.125	0.023	0.028637667
IQCG	1.50E-06	0.525577144	0.175	0.048	0.032780869
CFL1	1.57E-06	-0.292514365	0.908	0.973	0.034245142
NDUFV2	1.67E-06	0.45153868	0.533	0.337	0.036479386
FLI1	1.88E-06	-0.46765258	0.142	0.371	0.040955012
LDHA	1.95E-06	0.540697981	0.658	0.474	0.04261063
ILF3-AS1	2.13E-06	-0.529014394	0.083	0.288	0.046394154
SIT1	2.13E-06	-0.477318747	0.133	0.352	0.046411914

List of differentially expressed genes of IL7R_CD4 T in reperfusion stage (PR versus EP)

gene	p_val	avg_logFC	pct.1	pct.2	p_val_adj
RPS29	1.61E-21	0.49204538	0.994	0.996	3.51E-17
HSPD1	6.08E-21	1.777465719	0.737	0.413	1.33E-16
SRGN	3.15E-18	0.694745316	0.981	0.934	6.87E-14
FTH1	2.56E-17	0.814293406	1	0.971	5.59E-13
HSPE1	2.99E-16	1.471268859	0.75	0.533	6.52E-12
AIM1	5.07E-16	0.974283717	0.628	0.285	1.11E-11
MYL12A	1.65E-15	-0.654717163	0.756	0.926	3.60E-11
FAM177A1	1.79E-15	1.054493779	0.596	0.248	3.91E-11
HSPB1	2.08E-15	1.283389274	0.647	0.322	4.53E-11
MTHFD2	3.29E-15	0.899589329	0.5	0.153	7.18E-11
BATF	1.20E-14	0.867199005	0.679	0.314	2.62E-10
ACTB	1.59E-13	-0.488200033	0.994	1	3.46E-09

HSP90AB1	1.87E-13	0.983716063	0.853	0.744	4.07E-09
HSPH1	9.73E-13	1.463750537	0.551	0.273	2.12E-08
DEFA3	2.65E-12	-0.588041463	0.051	0.36	5.77E-08
HLA-B	5.25E-12	0.400684844	0.994	0.988	1.15E-07
PFN1	5.27E-12	-0.537538817	0.904	0.979	1.15E-07
TRAF3IP3	5.97E-12	-0.814730662	0.103	0.413	1.30E-07
HSP90AA1	7.52E-12	1.438483771	0.859	0.855	1.64E-07
CREM	5.54E-11	0.783513272	0.622	0.355	1.21E-06
CORO1A	6.41E-11	-0.576354968	0.545	0.822	1.40E-06
S100A4	1.52E-10	-0.621214774	0.731	0.897	3.31E-06
CCND3	1.88E-10	-0.579867385	0.34	0.649	4.10E-06
BCL3	2.00E-10	0.550839856	0.231	0.029	4.36E-06
CD52	2.42E-10	-0.549906761	0.66	0.843	5.27E-06
LMNA	1.64E-09	0.898078798	0.397	0.14	3.58E-05
RELB	2.66E-09	0.648337492	0.301	0.079	5.80E-05
LCK	2.67E-09	-0.646070309	0.282	0.574	5.82E-05
CSK	2.72E-09	-0.590466202	0.064	0.322	5.94E-05
LDHA	4.77E-09	0.49983531	0.782	0.595	0.000104065
ARID5A	6.40E-09	0.699013181	0.397	0.161	0.000139533
CNOT6L	7.40E-09	0.590895814	0.481	0.227	0.000161283
HBB	8.28E-09	-0.663499846	0.096	0.36	0.00018058
S100A8	8.55E-09	-0.671657426	0.173	0.467	0.000186548
BTG1	1.29E-08	-0.361849633	0.974	1	0.000281373
JUN	1.86E-08	-0.53728445	0.692	0.897	0.000405972
LAT	2.41E-08	-0.574735157	0.154	0.421	0.000525702
9-Sep	2.76E-08	-0.518320024	0.179	0.467	0.000601081
IGLV2-14	3.50E-08	-0.665699694	0.058	0.281	0.000764051
TBC1D10C	4.14E-08	-0.483602699	0.128	0.384	0.00090207
EZR	4.39E-08	0.758509495	0.641	0.463	0.000957293
CITED2	7.67E-08	-0.59409763	0.494	0.781	0.001671688
SLC7A5	1.05E-07	0.636933767	0.314	0.107	0.002297461
SOD1	1.16E-07	0.62139276	0.686	0.545	0.002520388
BIRC3	1.36E-07	0.796454402	0.474	0.252	0.002962047
DRAP1	1.45E-07	-0.519646474	0.122	0.36	0.003164827
ITGA4	1.78E-07	-0.631389355	0.115	0.343	0.003879232
FKBP4	1.85E-07	0.757559728	0.288	0.107	0.004032038
SARAF	2.20E-07	0.3296558	0.942	0.942	0.004804569
PELI1	3.44E-07	0.564534553	0.25	0.07	0.007493161
HLA-A	3.45E-07	0.280375373	1	0.971	0.007527773
SNX9	3.49E-07	0.55120467	0.301	0.107	0.007610331
FERMT3	3.59E-07	-0.472386523	0.071	0.273	0.007825521
AC006129.2	3.69E-07	-0.580576529	0.135	0.368	0.008036584
RCSD1	3.86E-07	-0.493257147	0.167	0.409	0.008418237
RNF19A	6.51E-07	0.641097301	0.385	0.186	0.01420292
HLA-C	6.58E-07	0.31308804	0.981	0.979	0.014352504
H3F3B	7.47E-07	0.356514994	0.994	0.967	0.016285247
PRDM1	7.97E-07	0.600450543	0.551	0.355	0.017377514
DUSP1	8.05E-07	-0.442773997	0.872	0.946	0.017559829
DDX21	8.64E-07	0.532084884	0.481	0.281	0.018842856
SH2D2A	9.65E-07	0.71905929	0.385	0.194	0.021044148
DNAJA4	1.29E-06	0.568486397	0.128	0.012	0.028137869
IGKV2-30	1.59E-06	-0.509630487	0.038	0.215	0.034760143
SYAP1	1.88E-06	0.601962226	0.346	0.161	0.040962015

List of differentially expressed genes of PTGDS_NK in reperfusion stage (PR versus EP)

gene	p_val	avg_logFC	pct.1	pct.2	p_val_adj
HLA-B	1.16E-24	0.523820774	1	0.993	2.54E-20
B2M	5.87E-24	0.430143591	1	1	1.28E-19
DEFA3	1.16E-20	-1.140667673	0.034	0.489	2.52E-16
CORO1A	8.38E-19	-0.773128438	0.536	0.922	1.83E-14
MYOM2	1.19E-18	-1.186449053	0.112	0.574	2.59E-14
CD3E	1.22E-18	-1.359147821	0.028	0.433	2.66E-14
CST7	4.50E-18	0.647125065	1	0.986	9.82E-14
LAIR2	2.71E-17	1.411457104	0.603	0.17	5.91E-13
LCK	2.72E-17	-0.995104196	0.151	0.617	5.93E-13
CCL4	1.48E-16	0.8723943	0.978	0.965	3.22E-12
GNLY	3.10E-16	-0.690146854	0.536	0.993	6.76E-12

RPS29	4.43E-16	0.611026725	0.983	0.872	9.67E-12
NFKBIA	7.53E-16	0.66460417	0.978	0.837	1.64E-11
GZMB	8.27E-16	0.684492252	0.972	0.965	1.80E-11
SRGN	2.41E-14	0.522031466	0.989	0.993	5.26E-10
HIF1A	9.76E-14	1.087514318	0.531	0.149	2.13E-09
MT-CO3	4.82E-13	-0.358122726	1	1	1.05E-08
PRDM1	2.74E-12	0.847573272	0.715	0.426	5.98E-08
HLA-C	2.86E-12	0.374863197	0.994	1	6.23E-08
PFN1	3.34E-12	-0.39027806	0.972	1	7.28E-08
CCL4L2	5.47E-12	1.013717444	0.838	0.532	1.19E-07
GBP2	9.66E-12	0.940848359	0.514	0.17	2.11E-07
HBB	1.74E-11	-0.811391436	0.061	0.369	3.79E-07
ACTB	1.93E-11	-0.451787225	1	1	4.21E-07
KIR3DL2	1.10E-10	1.005929795	0.447	0.135	2.39E-06
TC2N	3.77E-10	-0.652772917	0.045	0.312	8.23E-06
EIF3G	7.38E-10	-0.645808285	0.464	0.773	1.61E-05
PNRC1	7.95E-10	0.52983949	0.905	0.752	1.73E-05
ISG20	8.43E-10	0.629389972	0.777	0.546	1.84E-05
TNFAIP3	1.03E-09	0.758715255	0.777	0.518	2.25E-05
MT-ATP6	1.97E-09	-0.330253549	0.989	0.993	4.30E-05
CALM1	3.76E-09	-0.413715308	0.788	0.965	8.19E-05
CD69	7.04E-09	0.753723455	0.939	0.865	0.000153515
H3F3B	7.48E-09	0.322557117	0.994	0.993	0.00016321
FTH1	7.68E-09	0.558819377	0.955	0.936	0.000167571
LAT	8.24E-09	-0.642136229	0.067	0.319	0.000179769
S100A4	9.01E-09	-0.531226087	0.743	0.95	0.000196549
GADD45B	9.56E-09	0.630992657	0.888	0.787	0.000208546
SAMD3	9.61E-09	-0.63971307	0.19	0.504	0.000209565
IFNG	9.65E-09	0.718545833	0.67	0.34	0.000210445
IGKV3-15	1.19E-08	-0.630644265	0.006	0.184	0.000260044
SLC9A3R1	1.26E-08	-0.621508902	0.307	0.617	0.000274878
SYTL3	1.64E-08	0.679492444	0.715	0.56	0.000357804
HCST	1.81E-08	-0.452301358	0.788	0.929	0.000394944
C1orf162	2.02E-08	-0.650123087	0.19	0.489	0.00044088
RASGRP2	2.33E-08	-0.696513891	0.123	0.39	0.000507652
PTPRCAP	2.75E-08	-0.436670108	0.749	0.936	0.000598705
BATF	4.20E-08	0.863764357	0.436	0.191	0.000915468
DDIT4	1.22E-07	0.480706874	0.933	0.894	0.002664547
TBC1D10C	1.27E-07	-0.506373449	0.218	0.532	0.002772304
ACTG1	1.37E-07	-0.365815069	0.933	0.993	0.002996102
CCL3L3	1.39E-07	1.084885801	0.553	0.319	0.00303224
NACA	1.55E-07	-0.32452496	0.866	0.979	0.003382851
ANXA6	1.71E-07	-0.543025209	0.179	0.461	0.00373705
CNN2	1.88E-07	-0.66004961	0.084	0.312	0.004089397
CIRBP	2.05E-07	0.46679209	0.838	0.723	0.004480789
MT-ND3	2.37E-07	0.355880354	0.983	1	0.005173235
RGS2	2.81E-07	-0.899428571	0.056	0.255	0.006134261
S100A8	3.20E-07	-0.614888487	0.162	0.433	0.006979776
SH2D2A	3.41E-07	0.636181478	0.525	0.284	0.007439357
RGS19	3.96E-07	-0.603511756	0.084	0.312	0.008632638
BIN1	4.10E-07	-0.595864402	0.101	0.333	0.008950193
SLA	4.33E-07	0.696684377	0.559	0.362	0.009448392
IL2RB	4.51E-07	-0.540118699	0.285	0.596	0.009846236
HSPA8	4.65E-07	-0.30384536	0.804	0.929	0.010149746
CD99	4.98E-07	-0.384100585	0.709	0.901	0.010856872
P2RY8	7.85E-07	-0.488602234	0.028	0.199	0.017108606
ACAP1	9.79E-07	-0.544340701	0.207	0.475	0.021348173
RELB	1.02E-06	0.773317796	0.302	0.092	0.022287664
HLA-DRB1	1.13E-06	-0.36907006	0.156	0.418	0.024620018
NFKB2	1.70E-06	0.641097603	0.251	0.057	0.037072198
STAT1	1.74E-06	0.646418984	0.279	0.078	0.037898685
HRASLS2	1.82E-06	-0.519954903	0.05	0.234	0.039716834
IGKV1-12	1.91E-06	-0.437865358	0	0.121	0.041647193
NFKBIZ	2.16E-06	0.794140726	0.598	0.39	0.046999865

List of differentially expressed genes of TRBV9_CD8 Teff in reperfusion stage (PR versus EP)

gene	p_val	avg_logFC	pct.1	pct.2	p_val_adj

RPS29	8.13E-20	0.695713535	0.983	0.981	1.77E-15
TRAV38-2DV8	9.95E-16	-1.487751452	0.075	0.571	2.17E-11
HLA-B	1.51E-15	0.464962927	1	1	3.29E-11
TRBV13	7.95E-15	2.180406841	0.45	0	1.73E-10
TRBV9	3.39E-14	-1.520573927	0.142	0.6	7.39E-10
CD52	3.97E-14	-0.831924803	0.658	0.952	8.65E-10
RPS27	5.18E-12	0.344246775	1	1	1.13E-07
DUSP1	2.99E-11	-0.783189837	0.708	0.914	6.51E-07
DEFA3	5.06E-11	-0.873806286	0.033	0.39	1.10E-06
S100A4	7.53E-11	-0.640796909	0.925	0.99	1.64E-06
SRGN	1.70E-10	0.508031703	0.992	0.981	3.71E-06
SYTL3	1.78E-10	0.857191564	0.75	0.467	3.88E-06
GZMB	9.56E-10	0.78548654	0.967	0.819	2.09E-05
VIM	1.42E-08	-0.546297954	0.658	0.914	0.000309022
MT-ND3	1.50E-08	0.50204372	1	0.99	0.000326888
TRAV17	2.76E-08	1.151057253	0.275	0.01	0.000601959
S100A6	4.13E-08	-0.490873875	0.742	0.943	0.000901545
MT1E	5.17E-08	-0.436760086	0.125	0.467	0.001127607
CD160	6.91E-08	-0.661674783	0.008	0.238	0.001506603
RPL37A	8.86E-08	0.372322002	0.992	0.952	0.001932124
GADD45B	1.27E-07	0.708182415	0.833	0.629	0.002762975
CORO1A	3.28E-07	-0.518500012	0.675	0.886	0.007159248
SH2D2A	8.94E-07	0.641304034	0.508	0.21	0.01950466
JUN	9.96E-07	-0.558149441	0.567	0.819	0.021728654
KLRB1	1.27E-06	0.767580614	0.592	0.267	0.02759171
CNN2	1.37E-06	-0.760394258	0.217	0.514	0.029788249
CD3E	1.40E-06	-0.405438376	0.833	0.952	0.030629827
PRDM1	1.89E-06	0.764345399	0.592	0.333	0.041246082

List of differentially expressed genes of STMN1_NK-cycling in reperfusion stage (PR versus EP)

gene	p_val	avg_logFC	pct.1	pct.2	p_val_adj
RELB	2.06E-13	1.300348985	0.692	0.07	4.48E-09
NFKB2	1.78E-11	0.95560249	0.692	0.1	3.89E-07
HSPD1	5.47E-10	1.757245543	0.923	0.45	1.19E-05
BIRC3	4.75E-09	1.098563487	0.538	0.07	0.000103536
HSP90AB1	5.81E-09	1.321491792	1	0.72	0.0001266
SRGN	8.40E-09	0.792676492	1	0.99	0.000183287
BCL3	3.06E-08	0.728436717	0.462	0.05	0.000667698
MYL12A	3.65E-08	-0.754616523	0.962	1	0.000796247
LYST	4.53E-08	0.83424971	0.808	0.26	0.0009878
PTPRCAP	6.83E-08	-0.82242316	0.885	0.97	0.001488466
HSP90AA1	1.07E-07	0.989428236	1	0.99	0.002338342
KIR2DL4	1.99E-07	0.771439625	0.423	0.05	0.004342851
NOP58	2.52E-07	0.861538206	0.731	0.29	0.005499465
IRF8	2.65E-07	0.873490481	0.462	0.07	0.005788861
CD72	4.06E-07	0.565618837	0.385	0.04	0.008858909
HSPE1	5.53E-07	1.426215876	0.885	0.75	0.012058158
NINJ1	6.30E-07	0.754362223	0.462	0.07	0.013734395
FKBP4	6.56E-07	0.900272243	0.538	0.12	0.014300038
HLA-B	7.56E-07	0.354989676	1	1	0.016494783
SQSTM1	9.24E-07	0.88345055	0.962	0.71	0.020151215
SPAG4	9.94E-07	0.355071033	0.231	0	0.021674731
SYTL3	1.74E-06	0.790148529	0.885	0.55	0.037866668
SYAP1	1.76E-06	0.82118838	0.692	0.25	0.038343784
CORO1A	2.09E-06	-0.705967413	0.885	0.99	0.045601022

List of differentially expressed genes of XCL2_NK in reperfusion stage (PR versus EP)

gene	p_val	avg_logFC	pct.1	pct.2	p_val_adj
CREM	7.66E-13	1.69361501	0.828	0.138	1.67E-08
PFN1	2.25E-08	-0.64073968	0.966	1	0.000490558
HSP90AA1	5.35E-08	1.555807717	0.966	0.805	0.001167247
HSPD1	6.18E-08	1.938812949	0.759	0.345	0.00134795
ACTB	6.65E-08	-0.519394517	1	1	0.001450415

AREG	3.06E-07	1.058548593	0.897	0.402	0.006672001
STAT3	3.96E-07	1.1401577	0.655	0.218	0.008639454
HMGB2	7.30E-07	1.19220818	0.586	0.149	0.015921425
SRGN	1.58E-06	0.626265785	1	0.989	0.034424996
SH2D2A	2.19E-06	0.980060295	0.724	0.276	0.047731639

List of differentially expressed genes of XCL1_NK in reperfusion stage (PR versus EP)

gene	p_val	avg_logFC	pct.1	pct.2	p_val_adj
FAM177A1	3.10E-10	1.41156954	0.88	0.282	6.77E-06
CD74	2.51E-08	1.097577558	1	0.704	0.000547475
HIF1A	1.08E-07	1.118392394	0.68	0.155	0.002347343
HLA-B	2.71E-07	0.527725127	1	1	0.005907822
NFKB2	4.39E-07	0.989340469	0.52	0.07	0.009567839
RELB	7.20E-07	1.128162496	0.52	0.085	0.015699868
DENND2D	1.93E-06	-1.254265885	0.2	0.775	0.042109517
HSPD1	1.93E-06	1.809060491	0.76	0.324	0.042135007

List of differentially expressed genes of STMN1_T-cycling in reperfusion stage (PR versus EP)

gene	p_val	avg_logFC	pct.1	pct.2	p_val_adj
RELB	3.77E-13	1.424862839	0.778	0.103	8.22E-09
NFKB2	2.04E-11	1.241949758	0.694	0.088	4.45E-07
HSP90AB1	2.65E-11	1.517584822	1	0.809	5.78E-07
HSPD1	4.25E-11	2.272745016	0.861	0.441	9.27E-07
SRGN	6.06E-11	0.878952956	1	0.971	1.32E-06
HSP90AA1	1.37E-10	1.74356844	1	0.971	2.99E-06
FEZ1	2.66E-09	1.251860004	0.639	0.132	5.80E-05
SLC7A5	3.42E-09	0.97274761	0.861	0.353	7.46E-05
CALM2	7.88E-09	-0.789790379	0.833	0.985	0.000171802
UBE2C	8.23E-09	-1.070431634	0.694	0.926	0.00017958
HLA-A	1.01E-08	0.490489011	1	1	0.000220066
HSPH1	1.09E-08	1.859602344	0.722	0.25	0.00023711
HLA-B	2.52E-08	0.496952915	1	1	0.000549068
SYTL3	4.83E-08	0.89006737	0.861	0.456	0.001052965
HSPE1	5.00E-08	1.733749418	0.806	0.588	0.001089934
HSPA1A	5.12E-08	2.431293032	0.833	0.559	0.001117292
HMGB2	5.36E-08	-0.638576872	0.972	0.985	0.001169679
HSPB1	6.85E-08	1.046071955	0.833	0.515	0.001494365
CST7	9.23E-08	0.820185777	0.972	0.706	0.002013428
CD7	9.84E-08	0.925277582	0.917	0.779	0.002146457
SMC4	1.08E-07	-0.812503214	0.667	0.912	0.002358369
MYL12A	1.69E-07	-0.705484993	0.889	0.971	0.003678819
LMNB1	1.84E-07	-0.864105845	0.306	0.853	0.004019056
SYAP1	2.97E-07	1.073480395	0.694	0.309	0.006473323
DLGAP5	3.18E-07	-0.9243318	0.167	0.735	0.006939434
PLK1	3.50E-07	-0.910432383	0.139	0.676	0.007634794
CDCA3	4.15E-07	-0.815834769	0.139	0.691	0.009053963
CKAP2	5.23E-07	-0.856670537	0.278	0.779	0.011415794
RHEB	5.97E-07	-0.821110569	0.444	0.882	0.013009624
HMMR	6.25E-07	-0.832387115	0.389	0.824	0.013626733
FAM177A1	8.41E-07	0.909478085	0.75	0.353	0.018344764
PDE4A	9.84E-07	0.587328669	0.361	0.015	0.021449442
CDC25B	9.96E-07	-0.797922048	0.444	0.838	0.0217196
TNFRSF1B	1.06E-06	0.860663566	0.694	0.279	0.023139858
CDC20	1.35E-06	-0.927281265	0.389	0.794	0.029353376
DEPDC1B	1.68E-06	-0.752711266	0.167	0.676	0.036743624
ODF3B	1.75E-06	0.556217507	0.306	0	0.038078797
USP36	1.77E-06	0.730731725	0.583	0.176	0.038502897
TUBA1B	1.88E-06	-0.589117912	1	1	0.041025088
CREM	1.93E-06	1.073032482	0.694	0.338	0.042185853
CDKN2D	2.01E-06	-0.827968388	0.306	0.735	0.043870021
CORO1A	2.15E-06	-0.619338167	0.861	0.971	0.046800674
MXD3	2.23E-06	-0.796294179	0.056	0.529	0.048574267

List of differentially expressed genes of TRDV2_γδ T in reperfusion stage (PR versus EP)

gene	p_val	avg_logFC	pct.1	pct.2	p_val_adj
NA					

Table 16: List of DEGs of NK/T cell clusters in overall stage (PR versus PP)

List of differentially expressed genes of CD8B_CD8 Tem in overall stage (PR versus PP)					
gene	p_val	avg_logFC	pct.1	pct.2	p_val_adj
HSP90AA1	5.84E-52	1.864173465	0.923	0.753	1.27E-47
HSPD1	2.06E-50	2.187888599	0.711	0.286	4.50E-46
ACTB	2.62E-39	-0.551272671	0.963	0.998	5.72E-35
DEFA3	3.69E-38	-1.311068333	0.051	0.532	8.05E-34
PFN1	1.22E-37	-0.62228755	0.868	0.982	2.67E-33
HSPH1	5.33E-37	1.680984764	0.579	0.18	1.16E-32
MYL12A	3.82E-33	-0.739800013	0.608	0.908	8.33E-29
RPS29	3.85E-33	0.518891318	0.985	0.965	8.40E-29
HSPE1	8.01E-33	1.680278076	0.718	0.442	1.75E-28
CORO1A	1.59E-32	-0.849035626	0.458	0.814	3.48E-28
HSPB1	1.94E-32	1.444236801	0.597	0.235	4.24E-28
CREM	2.85E-32	0.965896948	0.678	0.268	6.21E-28
HSP90AB1	1.10E-28	1.052010184	0.868	0.703	2.40E-24
FAM177A1	1.59E-27	1.081384768	0.564	0.225	3.47E-23
HBB	2.03E-27	-1.123161167	0.088	0.481	4.42E-23
GZMA	6.51E-26	-0.679694614	0.615	0.865	1.42E-21
CCND3	1.60E-25	-0.817193139	0.278	0.663	3.50E-21
HSPA1A	7.34E-25	1.615767382	0.656	0.346	1.60E-20
BATF	1.85E-23	0.951329101	0.52	0.209	4.04E-19
RPS27	5.66E-23	0.277321558	0.993	0.996	1.23E-18
LITAF	9.09E-22	0.725277029	0.733	0.454	1.98E-17
HCST	1.44E-20	-0.527467436	0.681	0.894	3.15E-16
PTPRCAP	4.11E-20	-0.510143672	0.685	0.883	8.96E-16
SH2D2A	4.53E-20	0.826913847	0.469	0.194	9.87E-16
S100A4	2.10E-19	-0.675100708	0.535	0.793	4.58E-15
LDHA	2.74E-19	0.668545944	0.711	0.442	5.97E-15
CLIC1	2.86E-19	-0.577877213	0.491	0.763	6.24E-15
SOD1	4.35E-19	0.866477086	0.656	0.427	9.48E-15
TNFAIP3	8.45E-19	0.670307638	0.637	0.335	1.84E-14
UBB	6.08E-18	0.596387988	0.872	0.73	1.33E-13
RELB	2.15E-17	0.727553872	0.245	0.043	4.68E-13
SRGN	8.38E-17	0.394618722	0.985	0.969	1.83E-12
CD3E	1.11E-16	-0.357461551	0.868	0.943	2.41E-12
PTMA	2.54E-16	0.358246532	1	0.973	5.53E-12
RPS21	4.28E-16	0.272844132	0.996	0.99	9.34E-12
PPP1R18	7.80E-16	-0.739678699	0.147	0.429	1.70E-11
NOP58	1.14E-15	0.730857877	0.469	0.219	2.49E-11
SARAF	1.16E-15	0.410412016	0.945	0.885	2.53E-11
LCK	2.04E-15	-0.566633735	0.326	0.611	4.44E-11
ISG20	3.25E-15	0.658299954	0.659	0.464	7.10E-11
GZMK	3.43E-15	-0.533968975	0.667	0.834	7.47E-11
LMNA	6.03E-15	0.794955339	0.216	0.039	1.31E-10
STAT3	6.37E-15	0.677930401	0.476	0.239	1.39E-10
SYAP1	6.96E-15	0.807381417	0.355	0.131	1.52E-10
FOS	6.96E-15	0.468351802	0.886	0.671	1.52E-10
LAT	7.93E-15	-0.604634085	0.147	0.431	1.73E-10
CNOT6L	8.57E-15	0.68958627	0.469	0.229	1.87E-10
CACYBP	2.27E-14	0.950599116	0.407	0.186	4.96E-10
ODC1	4.18E-14	0.709762515	0.355	0.137	9.11E-10
FTH1	6.49E-14	0.638106141	0.952	0.933	1.42E-09
H3F3B	7.12E-14	0.374508177	0.993	0.961	1.55E-09
HMGB2	1.04E-13	0.802594886	0.48	0.266	2.28E-09
SH3BGRL3	1.50E-13	-0.367534829	0.725	0.89	3.27E-09
GZMB	2.60E-13	1.138936569	0.333	0.133	5.68E-09
CKLF	4.15E-13	-0.595923801	0.176	0.44	9.06E-09
ROMO1	4.88E-13	0.639914763	0.436	0.213	1.06E-08
NFKBIA	7.52E-13	0.49782323	0.773	0.581	1.64E-08
PPP1CB	1.35E-12	0.630374313	0.333	0.129	2.94E-08
IL10	1.70E-12	1.070152046	0.154	0.02	3.70E-08
PIK3R1	2.40E-12	0.528454585	0.696	0.491	5.23E-08
DENND2D	2.44E-12	-0.640776403	0.125	0.366	5.33E-08
SLC9A3R1	2.52E-12	-0.526527591	0.22	0.483	5.50E-08
USP36	3.48E-12	0.565566935	0.26	0.078	7.59E-08
TNFRSF9	4.04E-12	0.720911474	0.264	0.088	8.82E-08
TSPYL2	4.49E-12	0.625337364	0.513	0.288	9.79E-08
CD99	4.84E-12	-0.362544894	0.681	0.861	1.06E-07
APOBEC3G	5.27E-12	-0.60104459	0.099	0.323	1.15E-07

CD7	1.07E-11	0.630706163	0.696	0.579	2.33E-07
RAC2	1.15E-11	-0.415288908	0.509	0.759	2.51E-07
RHBDD2	1.23E-11	0.547261606	0.267	0.088	2.68E-07
MTHFD2	2.23E-11	0.691524603	0.311	0.129	4.86E-07
EIF1	2.40E-11	0.285116151	0.989	0.975	5.24E-07
EZR	2.53E-11	0.685321829	0.549	0.374	5.52E-07
SH3BP1	3.03E-11	-0.573015922	0.04	0.221	6.62E-07
ATP1B3	3.15E-11	0.715551642	0.392	0.194	6.88E-07
DNAJB6	3.26E-11	0.687691074	0.509	0.315	7.10E-07
TRAF3IP3	4.71E-11	-0.607463216	0.114	0.331	1.03E-06
ITGB2	4.75E-11	-0.509365547	0.37	0.603	1.04E-06
KIAA1551	6.13E-11	-0.556638276	0.278	0.511	1.34E-06
S100A8	8.20E-11	-0.718001041	0.168	0.415	1.79E-06
ODF3B	8.63E-11	0.447723342	0.143	0.022	1.88E-06
TBC1D10C	1.57E-10	-0.569861344	0.147	0.368	3.42E-06
LCP2	1.59E-10	-0.502587614	0.231	0.472	3.46E-06
ALB	1.67E-10	0.489638256	0.179	0.041	3.63E-06
IFITM2	2.17E-10	0.529366155	0.795	0.683	4.74E-06
PTPN6	2.21E-10	-0.487045311	0.081	0.28	4.83E-06
AIM1	3.16E-10	0.603607755	0.462	0.266	6.88E-06
EVL	3.21E-10	-0.455123983	0.366	0.603	7.01E-06
IRF1	3.39E-10	0.585911228	0.513	0.319	7.39E-06
THEMIS	3.61E-10	-0.496798457	0.125	0.342	7.86E-06
PDLIM2	4.35E-10	-0.539372463	0.062	0.241	9.49E-06
FAM49B	4.43E-10	-0.472880449	0.22	0.47	9.67E-06
SAT1	4.46E-10	0.604773823	0.751	0.628	9.73E-06
SLC7A5	4.50E-10	0.621772539	0.267	0.104	9.81E-06
SOCS3	5.61E-10	0.597241086	0.278	0.112	1.22E-05
SMAP2	7.14E-10	0.486072958	0.392	0.198	1.56E-05
PGK1	7.27E-10	0.629874582	0.527	0.364	1.59E-05
BCL3	9.14E-10	0.45988018	0.147	0.029	1.99E-05
GPR171	1.06E-09	0.61045027	0.3	0.129	2.30E-05
PPP1CA	1.43E-09	-0.43773421	0.22	0.458	3.12E-05
METTL12	1.64E-09	0.527978638	0.308	0.133	3.57E-05
PRF1	1.86E-09	0.653649788	0.553	0.421	4.05E-05
SIT1	2.82E-09	-0.511104548	0.092	0.274	6.14E-05
MAP1LC3A	3.06E-09	0.484423026	0.136	0.027	6.68E-05
OAZ1	3.50E-09	0.364228862	0.839	0.767	7.63E-05
FAM173A	3.59E-09	-0.511790164	0.022	0.164	7.83E-05
CD48	4.03E-09	-0.353921111	0.498	0.706	8.79E-05
SRRT	4.80E-09	0.56997526	0.436	0.27	0.000104767
GPSM3	5.11E-09	-0.455419717	0.282	0.493	0.000111489
ACAP1	5.47E-09	-0.478432936	0.275	0.497	0.000119315
IGKV2-24	7.08E-09	-0.573277349	0.037	0.184	0.000154346
PLEK	7.10E-09	-0.488318628	0.114	0.301	0.000154728
SDCBP	7.73E-09	0.589185076	0.377	0.217	0.000168667
FKBP4	8.66E-09	0.719433917	0.238	0.096	0.000188898
EMD	8.84E-09	0.505883901	0.388	0.217	0.00019286
RAP1GDS1	8.90E-09	-0.449724282	0.055	0.217	0.000194099
CD27	8.95E-09	-0.486916446	0.183	0.397	0.000195204
SLC2A3	1.10E-08	0.405301599	0.663	0.499	0.000239969
SQSTM1	1.40E-08	0.443589432	0.604	0.45	0.000305946
TIFA	1.45E-08	0.466782132	0.158	0.041	0.000315884
SBNO2	1.56E-08	0.391336309	0.114	0.018	0.000340387
RGS19	1.68E-08	-0.44525006	0.04	0.188	0.000367279
ARID5A	1.94E-08	0.5317173	0.363	0.194	0.000422157
DDX24	2.06E-08	0.445367694	0.582	0.391	0.000449619
CCL4L2	2.10E-08	-0.489659009	0.623	0.8	0.000458115
HBA2	2.56E-08	-0.444846697	0.026	0.157	0.000559288
DAD1	2.58E-08	-0.449595163	0.253	0.466	0.000561658
C19orf60	2.84E-08	-0.46883135	0.165	0.356	0.000619868
STARD7	3.47E-08	0.561404218	0.282	0.137	0.000755947
LEPROTL1	3.97E-08	0.508423755	0.597	0.472	0.0008651
TSC22D4	4.37E-08	-0.459206503	0.121	0.297	0.000952595
CD3D	5.77E-08	-0.300313296	0.597	0.757	0.001258935
NEAT1	6.70E-08	0.405586088	0.63	0.468	0.001461264
GADD45B	7.39E-08	0.484545953	0.777	0.712	0.001612459
RBL2	7.70E-08	-0.521405946	0.117	0.286	0.001678276
DDX21	7.92E-08	0.508927344	0.366	0.213	0.001727394
RPL36A	8.99E-08	0.341961154	0.828	0.763	0.001960525

HLA-DQB1	9.49E-08	0.501993097	0.538	0.37	0.00206892
LSP1	1.08E-07	-0.446335954	0.399	0.575	0.002346138
CXCR4	1.16E-07	-0.328181733	0.864	0.939	0.002532696
ACTR3	1.21E-07	-0.399011137	0.293	0.507	0.002641223
CD3G	1.28E-07	-0.405664552	0.366	0.566	0.002782066
PRDM1	1.33E-07	0.543362166	0.469	0.321	0.002889793
NFKB2	1.37E-07	0.405504406	0.147	0.041	0.002988096
ELL2	1.72E-07	0.428885363	0.194	0.072	0.003742629
LIME1	1.85E-07	-0.461256429	0.15	0.327	0.00402587
CSK	1.92E-07	-0.391563467	0.128	0.305	0.004190786
NAMPT	2.02E-07	0.508454353	0.249	0.115	0.004406096
RHOF	2.08E-07	-0.399488976	0.114	0.278	0.004530608
CDC25B	2.10E-07	-0.459432422	0.04	0.168	0.004589871
RAB37	2.27E-07	-0.419228732	0.018	0.131	0.004956934
PHYH	2.36E-07	0.365561305	0.128	0.031	0.005146893
STX10	3.15E-07	-0.386483621	0.044	0.176	0.006877091
TMA7	3.17E-07	-0.284692316	0.729	0.863	0.006923749
TRAC	3.29E-07	-0.557806057	0.128	0.288	0.007168271
DUSP2	3.31E-07	-0.30073126	0.857	0.9	0.00720999
HRASLS2	3.95E-07	-0.37657552	0.048	0.182	0.008609494
IFITM1	4.03E-07	0.339361031	0.901	0.906	0.008796732
GSPT1	4.22E-07	0.468484679	0.209	0.086	0.009204503
MT2A	4.33E-07	2.455383383	0.56	0.444	0.009437091
TNFRSF1A	4.46E-07	-0.34718085	0.018	0.129	0.009727634
MACF1	4.70E-07	-0.435094378	0.132	0.297	0.010244495
EML4	4.78E-07	0.428756638	0.509	0.366	0.010428469
SKAP1	5.10E-07	-0.419976321	0.238	0.423	0.01112987
SELK	5.70E-07	0.441193665	0.491	0.354	0.012437368
SERPINB9	5.73E-07	0.430439233	0.264	0.129	0.012487483
MCL1	5.83E-07	0.406454478	0.608	0.495	0.01270782
WAS	6.32E-07	-0.375741759	0.201	0.38	0.013782106
SC5D	7.51E-07	0.454677519	0.176	0.065	0.016381298
SLC16A7	7.99E-07	-0.380694701	0.037	0.157	0.017432521
CD52	8.08E-07	-0.439079678	0.37	0.544	0.017628296
MRPL1	8.25E-07	0.404819906	0.179	0.067	0.017995635
EMP3	8.43E-07	-0.376881807	0.311	0.501	0.01838904
AC092580.4	8.88E-07	0.554063008	0.249	0.125	0.019357063
NUDC	9.38E-07	0.565352516	0.322	0.19	0.020460272
ATP6V0E2	9.49E-07	-0.417696925	0.062	0.19	0.020685923
CD8B	1.00E-06	-0.40889142	0.392	0.564	0.02182854
APBB1IP	1.00E-06	-0.39412883	0.242	0.421	0.021836642
FAM129A	1.02E-06	0.432569148	0.227	0.102	0.022292134
SP100	1.05E-06	-0.413388334	0.234	0.407	0.022882274
PDE4D	1.09E-06	0.357122572	0.168	0.059	0.023793243
COX7A2L	1.10E-06	-0.40954613	0.154	0.317	0.023963277
LMNB1	1.12E-06	0.373436808	0.143	0.045	0.024411325
SYTL3	1.25E-06	0.484874462	0.586	0.476	0.027236339
S100A6	1.29E-06	-0.338756728	0.546	0.72	0.028035533
LY6E	1.31E-06	-0.40388595	0.289	0.464	0.028572646
HMGB1	1.35E-06	-0.268800179	0.656	0.793	0.029442918
LYST	1.37E-06	0.426252115	0.436	0.297	0.029961901
HSPA8	1.50E-06	0.421179666	0.919	0.863	0.032718623
PELI1	1.56E-06	0.342492114	0.117	0.031	0.033980018
CISH	1.71E-06	-0.400379177	0.015	0.11	0.037201231
PFKFB3	1.75E-06	0.491655377	0.201	0.086	0.038172627
C9orf142	1.76E-06	-0.333766946	0.293	0.497	0.038427303
TOX	1.77E-06	-0.412744227	0.114	0.264	0.038708404
TMC8	1.79E-06	-0.414138962	0.125	0.276	0.039139299
BCL2L1	1.98E-06	-0.360848833	0.051	0.174	0.043217778
TNIP2	1.99E-06	0.377106408	0.172	0.065	0.043368425
HMGN3	2.00E-06	-0.350100795	0.092	0.235	0.043618229
PIM3	2.24E-06	0.425639586	0.22	0.102	0.048784794

List of differentially expressed genes of GNLY_NK in overall stage (PR versus PP)

gene	p_val	avg_logFC	pct.1	pct.2	p_val_adj
DEFA3	9.41E-26	-1.282942518	0.031	0.514	2.05E-21
HBB	9.23E-23	-1.344750544	0.081	0.534	2.01E-18

GBP2	1.27E-21	0.889762791	0.569	0.222	2.78E-17
LAIR2	3.04E-21	1.190346135	0.456	0.136	6.64E-17
ACTB	1.07E-20	-0.440374215	1	0.998	2.34E-16
SDCBP	2.66E-20	0.906488881	0.638	0.306	5.81E-16
RELB	5.25E-20	0.875146632	0.356	0.076	1.15E-15
CD3E	2.88E-19	-0.65128516	0.481	0.888	6.28E-15
TMSB4X	6.46E-19	-0.289212644	1	1	1.41E-14
CORO1A	8.58E-18	-0.674656225	0.575	0.862	1.87E-13
NFKBIA	1.58E-17	0.701279738	0.912	0.704	3.44E-13
HSP90AA1	1.72E-17	1.52680208	0.881	0.832	3.75E-13
RPS29	4.04E-17	0.442404471	0.975	0.958	8.81E-13
SYTL3	7.84E-17	0.690419915	0.738	0.482	1.71E-12
PFN1	1.58E-16	-0.362942801	0.988	1	3.45E-12
SLC9A3R1	2.55E-16	-0.716455066	0.35	0.706	5.57E-12
S100A4	8.08E-16	-0.460025426	0.881	0.98	1.76E-11
FCER1G	1.65E-15	0.978868252	0.312	0.072	3.60E-11
TNFAIP3	1.68E-15	0.738789019	0.712	0.43	3.66E-11
SH3BGRL3	8.10E-15	-0.395727698	0.906	0.984	1.77E-10
PTPRCAP	3.12E-14	-0.455988909	0.744	0.92	6.81E-10
SQSTM1	5.19E-14	0.687206263	0.762	0.592	1.13E-09
BATF	1.06E-13	0.810696644	0.531	0.29	2.30E-09
GZMA	1.36E-13	-0.446368063	0.775	0.952	2.96E-09
HSPD1	1.37E-13	1.634316557	0.525	0.302	2.98E-09
ACTG1	1.42E-13	-0.409812564	0.962	0.982	3.10E-09
HSPB1	1.58E-13	1.199138824	0.481	0.228	3.44E-09
CST7	2.40E-13	0.407252597	1	0.99	5.24E-09
PRDM1	2.50E-13	0.652921524	0.688	0.454	5.46E-09
GSTP1	5.71E-13	-0.541319316	0.519	0.8	1.24E-08
GZMH	5.74E-13	-0.444961169	0.75	0.946	1.25E-08
ALB	3.73E-12	0.605082972	0.181	0.028	8.13E-08
SRGN	4.52E-12	0.378753343	0.988	0.988	9.87E-08
LCK	5.13E-12	-0.566064518	0.344	0.676	1.12E-07
GNLY	8.20E-12	-0.318371891	0.919	0.998	1.79E-07
H3F3B	8.26E-12	0.345261086	0.981	0.96	1.80E-07
ISG20	8.70E-12	0.507470284	0.794	0.588	1.90E-07
NFKB2	1.70E-11	0.574226701	0.262	0.072	3.71E-07
RGS19	3.63E-11	-0.709386773	0.05	0.306	7.92E-07
RAC2	5.12E-11	-0.409879981	0.744	0.914	1.12E-06
NEAT1	7.71E-11	0.532452404	0.812	0.654	1.68E-06
LSP1	8.32E-11	-0.445536326	0.562	0.836	1.82E-06
HSPA1A	1.02E-10	1.864345099	0.588	0.37	2.22E-06
TBC1D10C	1.37E-10	-0.625490149	0.175	0.48	2.98E-06
BCL3	1.44E-10	0.471761822	0.156	0.024	3.14E-06
MYL12A	1.45E-10	-0.3306826	0.869	0.98	3.17E-06
GZMB	1.52E-10	0.423466741	0.975	0.976	3.31E-06
HSPH1	1.93E-10	1.524293395	0.381	0.182	4.21E-06
SLA2	2.48E-10	0.637221987	0.369	0.156	5.41E-06
TC2N	5.94E-10	-0.581202403	0.112	0.388	1.30E-05
CTD-3252C9.4	6.89E-10	0.556617064	0.25	0.076	1.50E-05
TRBC1	9.43E-10	-0.418387485	0.65	0.9	2.06E-05
ACAP1	9.57E-10	-0.589703308	0.194	0.478	2.09E-05
HIF1A	1.13E-09	0.656339387	0.419	0.206	2.47E-05
ZBTB16	1.41E-09	0.519526273	0.181	0.04	3.08E-05
CCL4	1.50E-09	0.569389049	0.944	0.958	3.26E-05
RP11-284N8.3	3.12E-09	0.35100764	0.119	0.014	6.80E-05
CSK	3.56E-09	-0.623180102	0.15	0.408	7.77E-05
NFKB1	4.06E-09	0.491125378	0.188	0.046	8.86E-05
BIN1	4.21E-09	-0.530420761	0.156	0.418	9.18E-05
RPS19	4.46E-09	-0.270076944	0.988	0.998	9.72E-05
SH3BP1	4.86E-09	-0.574740055	0.088	0.336	0.000106026
KIR2DL3	5.39E-09	0.563151812	0.394	0.184	0.000117448
ANKRD28	5.90E-09	0.573485149	0.362	0.158	0.000128736
TRAF3IP3	6.14E-09	-0.503272259	0.212	0.492	0.000133854
HCST	6.78E-09	-0.349616653	0.844	0.944	0.000147836
NDUFB7	7.04E-09	-0.533081783	0.169	0.448	0.000153523
IL32	7.17E-09	-0.305061732	0.744	0.94	0.000156283
CCND3	9.17E-09	-0.382755443	0.488	0.788	0.00020004
SH2D2A	1.24E-08	0.618955754	0.531	0.352	0.000270714
HPGD	1.38E-08	0.579841912	0.188	0.05	0.000300025
CD63	1.79E-08	-0.469347658	0.369	0.64	0.000389984

CADM1	1.89E-08	0.523493028	0.2	0.056	0.000411272
STOM	4.65E-08	0.541705018	0.538	0.372	0.001014552
BIN2	5.75E-08	-0.520531271	0.362	0.614	0.001254534
CSTB	6.81E-08	-0.537949709	0.138	0.372	0.001485888
CNN2	9.40E-08	-0.560025049	0.131	0.354	0.002051015
GBP4	1.04E-07	0.494088678	0.288	0.12	0.00226722
MT2A	1.09E-07	1.816632988	0.75	0.7	0.002381339
PPP1CA	1.26E-07	-0.368914419	0.394	0.69	0.00274587
TSC22D4	1.49E-07	-0.492727782	0.119	0.342	0.003241561
VASP	1.71E-07	-0.492027874	0.188	0.428	0.003739594
PTGDS	1.84E-07	0.95440511	0.244	0.09	0.004007614
S100A6	2.07E-07	-0.270629515	0.788	0.926	0.004511499
RASSF1	2.27E-07	-0.479814842	0.188	0.428	0.004947169
TTC38	2.31E-07	-0.465638845	0.231	0.474	0.005037891
SLFN5	3.10E-07	-0.494526921	0.056	0.244	0.006766227
FOS	3.85E-07	0.386713212	0.756	0.64	0.008397931
SMAP2	4.08E-07	0.512239921	0.362	0.192	0.008898815
MIR155HG	4.92E-07	0.506637065	0.15	0.038	0.010724396
CD99	5.09E-07	-0.277984089	0.85	0.95	0.011098097
HSPE1	5.15E-07	1.552200847	0.519	0.416	0.011222823
REL	5.35E-07	0.499978953	0.488	0.318	0.011670572
CIRBP	5.54E-07	0.393680502	0.762	0.658	0.012086838
HOPX	6.05E-07	-0.338070524	0.644	0.844	0.01318776
CXCR4	6.20E-07	-0.381238656	0.681	0.878	0.013527443
UBB	6.82E-07	0.420729841	0.856	0.83	0.014879418
CCL3	6.93E-07	0.580212304	0.712	0.544	0.015107507
CKLF	6.93E-07	-0.434381493	0.15	0.364	0.01511323
LAT	7.15E-07	-0.42898973	0.169	0.41	0.015598111
SPN	7.73E-07	-0.46052858	0.25	0.478	0.016847893
ADGRG1	9.05E-07	-0.348324555	0.444	0.726	0.019742397
1-Sep	9.20E-07	-0.434922799	0.194	0.414	0.020066053
DENND2D	9.55E-07	-0.38234574	0.281	0.532	0.020832717
CALM1	9.61E-07	-0.251362491	0.9	0.958	0.020961069
GADD45B	9.74E-07	0.475177663	0.856	0.832	0.021241573
ACTR3	1.22E-06	-0.403638689	0.456	0.658	0.026552556
ANXA6	1.27E-06	-0.420270411	0.388	0.594	0.027699627
GPSM3	1.34E-06	-0.417102213	0.388	0.616	0.029117539
ARPC1B	1.57E-06	-0.357788984	0.456	0.7	0.034278342
KIR2DL4	1.57E-06	0.424598901	0.131	0.032	0.034288269
KLRC3	1.71E-06	-0.355328995	0.425	0.672	0.037399566
GMFG	1.72E-06	-0.361383651	0.394	0.664	0.037487707
TRG-AS1	1.74E-06	-0.526936905	0.156	0.358	0.03784573
AC241585.2	1.75E-06	0.391846626	0.169	0.052	0.038261456
PLEK	1.81E-06	-0.383901274	0.456	0.658	0.039403451
HNRNPK	2.05E-06	-0.35562536	0.512	0.748	0.044723549
APBB1IP	2.15E-06	-0.353571156	0.275	0.526	0.046782313
SNAP23	2.24E-06	0.470128075	0.3	0.146	0.048804382
IRF1	2.26E-06	0.484224819	0.656	0.544	0.049302064

List of differentially expressed genes of CCL20_CD8 MAIT in overall stage (PR versus PP)

gene	p_val	avg_logFC	pct.1	pct.2	p_val_adj
TNFRSF18	1.20E-41	0.903740807	0.383	0.008	2.62E-37
BATF	1.13E-40	1.537429107	0.792	0.271	2.47E-36
MT2A	6.55E-40	2.383184436	0.95	0.653	1.43E-35
NFKB2	9.35E-39	1.19711472	0.617	0.114	2.04E-34
RELB	7.42E-38	1.252317467	0.683	0.164	1.62E-33
NFKB1	6.10E-36	1.245655728	0.658	0.154	1.33E-31
HSP90AA1	1.28E-34	1.768541618	0.95	0.842	2.79E-30
CD7	3.35E-32	0.862137875	0.983	0.83	7.31E-28
SLC7A5	1.15E-31	1.048187252	0.758	0.271	2.52E-27
FEZ1	1.46E-28	1.196286331	0.567	0.142	3.18E-24
CREM	6.38E-28	1.064556889	0.817	0.379	1.39E-23
HSPD1	4.81E-27	1.737570699	0.767	0.393	1.05E-22
FURIN	1.08E-26	0.833281343	0.333	0.028	2.36E-22
MYL12A	2.71E-26	-0.727655488	0.833	0.966	5.92E-22
SYTL3	1.19E-24	0.950964227	0.842	0.475	2.60E-20
CORO1A	4.81E-24	-0.803664853	0.633	0.918	1.05E-19

TXNIP	2.06E-23	-0.973456572	0.408	0.856	4.48E-19
SYAP1	4.30E-23	1.035702392	0.592	0.188	9.38E-19
FAM177A1	2.47E-22	1.15938103	0.7	0.343	5.40E-18
SRGN	6.59E-22	0.66284383	0.975	0.968	1.44E-17
DEFA3	1.18E-21	-1.199488235	0.042	0.545	2.57E-17
HSPE1	4.04E-21	1.455270736	0.775	0.483	8.81E-17
S100A4	4.50E-21	-0.500483859	0.933	0.99	9.81E-17
HLA-B	7.30E-21	0.389163328	1	1	1.59E-16
NINJ1	9.06E-21	0.93745367	0.567	0.194	1.98E-16
CCND3	3.10E-20	-0.905837819	0.242	0.717	6.77E-16
TNFRSF4	9.71E-20	0.65721733	0.2	0.008	2.12E-15
SERPINB9	2.68E-19	0.866823557	0.658	0.297	5.84E-15
HSP90AB1	3.66E-19	0.866842475	0.942	0.816	7.98E-15
PBX4	7.15E-19	0.942974927	0.642	0.301	1.56E-14
RPS29	1.48E-18	0.495935303	0.975	0.974	3.23E-14
GZMA	1.62E-18	-0.61141366	0.783	0.938	3.54E-14
BACH2	1.71E-18	0.54166161	0.25	0.024	3.74E-14
TNFRSF1B	3.67E-18	0.790350682	0.608	0.242	7.99E-14
FTH1	4.63E-18	1.007619994	0.958	0.978	1.01E-13
TRAF3IP3	2.85E-17	-0.918384952	0.1	0.545	6.21E-13
GNG2	4.07E-17	0.731332686	0.875	0.681	8.87E-13
LMNA	4.91E-17	0.897417075	0.333	0.066	1.07E-12
PTPRCAP	6.65E-17	-0.562915027	0.808	0.934	1.45E-12
GPR171	1.38E-16	0.913745171	0.658	0.343	3.01E-12
HLA-A	1.40E-16	0.384584235	1	0.988	3.05E-12
SNX9	3.82E-16	0.808172343	0.442	0.136	8.33E-12
EML4	4.12E-16	0.735598072	0.775	0.523	8.98E-12
PPP1CB	5.44E-16	0.764442763	0.508	0.184	1.19E-11
KLRG1	6.05E-16	-0.742954538	0.55	0.832	1.32E-11
HBB	7.69E-16	-1.053581408	0.092	0.509	1.68E-11
SH2D2A	1.54E-14	0.820663667	0.675	0.405	3.37E-10
PDE4A	1.54E-14	0.603126028	0.217	0.028	3.37E-10
DENND2D	1.85E-14	-0.851354855	0.092	0.487	4.03E-10
EVL	5.23E-14	-0.66773758	0.342	0.703	1.14E-09
FOXO1	6.24E-14	0.687213608	0.342	0.09	1.36E-09
SOCS3	6.72E-14	0.707607932	0.508	0.2	1.47E-09
LITAF	7.17E-14	0.714816919	0.75	0.483	1.56E-09
ELOVL5	1.17E-13	0.736090384	0.692	0.425	2.56E-09
SEC14L1	1.24E-13	0.77739107	0.417	0.142	2.70E-09
CKLF	1.49E-13	-0.676969997	0.242	0.635	3.24E-09
PFKFB3	1.72E-13	0.718325993	0.533	0.24	3.75E-09
SLC9A3R1	2.81E-13	-0.769756819	0.15	0.533	6.12E-09
HSPH1	4.29E-13	1.052879728	0.45	0.182	9.36E-09
GZMK	9.46E-13	-0.516259325	0.792	0.914	2.06E-08
SATB1	1.14E-12	0.766994282	0.725	0.499	2.48E-08
H3F3B	1.36E-12	0.446057558	1	0.976	2.98E-08
CD52	1.63E-12	-0.601281467	0.708	0.882	3.55E-08
IL7R	1.81E-12	0.512007118	1	0.954	3.95E-08
MT1E	2.31E-12	1.81719514	0.375	0.124	5.03E-08
BIRC3	2.94E-12	0.73905148	0.583	0.291	6.42E-08
PTPN6	3.05E-12	-0.746631119	0.083	0.445	6.64E-08
LCK	3.23E-12	-0.684228177	0.325	0.669	7.03E-08
CPD	3.79E-12	0.595471315	0.325	0.092	8.27E-08
AREG	3.82E-12	0.772017453	0.475	0.184	8.33E-08
HCST	4.57E-12	-0.440607161	0.858	0.956	9.96E-08
SH3BGRL3	4.82E-12	-0.475623182	0.85	0.926	1.05E-07
BCL2	4.91E-12	-0.790378004	0.058	0.389	1.07E-07
ACTB	7.57E-12	-0.324973174	1	0.998	1.65E-07
BCL3	8.98E-12	0.557016628	0.275	0.066	1.96E-07
OTULIN	9.10E-12	0.487644012	0.258	0.058	1.98E-07
CD3E	9.27E-12	-0.381802532	0.908	0.972	2.02E-07
MTHFD2	1.12E-11	0.62637811	0.392	0.14	2.43E-07
REL	1.39E-11	0.800753566	0.742	0.513	3.02E-07
HIVEP2	1.44E-11	0.607004451	0.283	0.074	3.15E-07
LYST	1.82E-11	0.634935819	0.467	0.198	3.97E-07
IL21R	2.03E-11	0.519162849	0.25	0.056	4.42E-07
PDE4D	2.30E-11	0.691702226	0.425	0.17	5.01E-07
DDX24	2.44E-11	0.668904002	0.683	0.435	5.32E-07
IRF8	2.48E-11	0.608549905	0.35	0.112	5.41E-07
ODC1	2.66E-11	0.628059397	0.367	0.122	5.81E-07

TSC22D4	3.01E-11	-0.672748284	0.067	0.397	6.56E-07
TNIP1	3.10E-11	0.613869007	0.492	0.238	6.76E-07
ATF3	3.59E-11	0.808610597	0.45	0.178	7.82E-07
HIF1A	4.63E-11	0.767042505	0.525	0.261	1.01E-06
PIM2	5.12E-11	0.738448108	0.608	0.363	1.12E-06
AC092580.4	6.69E-11	0.747582603	0.667	0.479	1.46E-06
HSP90B1	7.59E-11	0.678625503	0.775	0.583	1.65E-06
SMAP2	7.98E-11	0.638094568	0.567	0.305	1.74E-06
RUNX3	8.95E-11	0.61971066	0.567	0.309	1.95E-06
NAMPT	9.03E-11	0.838414899	0.417	0.174	1.97E-06
TNFRSF9	9.07E-11	0.535300282	0.192	0.034	1.98E-06
PPP1R18	9.38E-11	-0.604070093	0.2	0.555	2.05E-06
ZHX2	9.53E-11	0.6028798	0.275	0.074	2.08E-06
STARD7	1.14E-10	0.69527827	0.467	0.224	2.48E-06
TBC1D10C	1.16E-10	-0.64486516	0.142	0.481	2.54E-06
PIM3	1.20E-10	0.598875394	0.35	0.118	2.61E-06
STAT4	1.57E-10	0.554402768	0.733	0.523	3.43E-06
PSTPIP2	1.92E-10	0.510190659	0.225	0.05	4.18E-06
GIMAP7	2.08E-10	-0.752285559	0.183	0.507	4.55E-06
DENND3	2.09E-10	0.478055576	0.175	0.028	4.55E-06
STAT3	2.09E-10	0.641181309	0.567	0.323	4.56E-06
PIM1	2.60E-10	0.621237694	0.7	0.497	5.67E-06
CD44	3.03E-10	0.577823158	0.717	0.499	6.62E-06
IL2RA	4.33E-10	0.329773359	0.133	0.014	9.45E-06
HOPX	4.89E-10	-0.612796668	0.258	0.563	1.07E-05
ISG20	5.23E-10	0.572132737	0.767	0.565	1.14E-05
ELL2	5.87E-10	0.50103021	0.292	0.09	1.28E-05
BAZ1A	6.97E-10	0.598833524	0.533	0.291	1.52E-05
RNF115	1.04E-09	0.646153732	0.458	0.218	2.27E-05
GZMB	1.09E-09	0.53368796	0.217	0.048	2.37E-05
GIMAP1	1.54E-09	-0.66764596	0.142	0.445	3.35E-05
PFN1	1.55E-09	-0.313287697	0.975	0.99	3.37E-05
TRAF1	2.10E-09	0.681265539	0.467	0.234	4.59E-05
MIR155HG	2.15E-09	0.692084518	0.283	0.086	4.69E-05
VAV1	2.35E-09	0.492558086	0.325	0.114	5.13E-05
IFNG	2.64E-09	0.831916661	0.633	0.365	5.75E-05
PYHIN1	2.79E-09	-0.635486227	0.15	0.449	6.08E-05
GPBP1	3.47E-09	0.49464916	0.775	0.563	7.56E-05
CCDC6	4.13E-09	0.481842477	0.25	0.072	9.00E-05
IL4R	4.21E-09	0.496351548	0.208	0.05	9.18E-05
CCL4L2	5.23E-09	-0.903386481	0.725	0.81	0.000114067
TANK	5.61E-09	0.622171723	0.55	0.319	0.000122267
SARAF	6.09E-09	0.347290875	0.95	0.932	0.000132788
GADD45G	6.18E-09	0.449566822	0.208	0.05	0.000134669
KIAA1551	6.26E-09	-0.636722311	0.2	0.489	0.000136426
PGK1	6.95E-09	0.617779886	0.667	0.497	0.000151638
JAML	7.34E-09	-0.585867544	0.108	0.403	0.000160073
AC006129.2	7.76E-09	-0.652325905	0.133	0.423	0.000169176
HSPA1A	8.92E-09	1.197331756	0.567	0.349	0.000194481
CNOT6L	9.48E-09	0.565274133	0.5	0.261	0.000206695
TAGAP	1.09E-08	-0.48718604	0.575	0.8	0.000238039
GIMAP4	1.10E-08	-0.560264033	0.275	0.591	0.000240268
CHD9	1.22E-08	-0.527834257	0.042	0.301	0.000266595
PDLIM2	1.33E-08	-0.60817372	0.1	0.371	0.000290252
FYN	1.33E-08	0.455788297	0.783	0.697	0.000290975
MAP3K8	1.37E-08	0.529698562	0.683	0.459	0.000299076
TRABD2A	1.38E-08	0.509308475	0.192	0.044	0.000300693
GPCPD1	1.70E-08	0.541611064	0.375	0.162	0.00037103
SDCBP	1.86E-08	0.534314341	0.583	0.343	0.000405658
ATP1B3	2.49E-08	0.623095277	0.325	0.136	0.000543345
KDM6B	2.65E-08	0.533051231	0.508	0.293	0.000577406
CAST	2.71E-08	0.573693019	0.617	0.405	0.00059018
ARHGAP31	2.83E-08	0.340747998	0.158	0.03	0.000617414
LGALS1	3.18E-08	0.76814888	0.35	0.156	0.000694027
ZC3H7A	3.32E-08	0.484170928	0.275	0.094	0.000723095
C19orf60	3.56E-08	-0.593271349	0.133	0.411	0.000775974
SH3BP1	4.01E-08	-0.519420792	0.058	0.303	0.000874388
GTF3C1	4.42E-08	0.575848335	0.433	0.22	0.000963672
SBNO2	6.41E-08	0.337926726	0.133	0.022	0.00139862
METRNL	6.80E-08	0.316966945	0.117	0.016	0.001482539

TRAF4	6.81E-08	0.37900798	0.192	0.048	0.001486019
CCL4	6.99E-08	-0.58948312	0.95	0.976	0.001524256
PPP1CA	7.44E-08	-0.507710711	0.308	0.571	0.001622066
ITGB2	8.52E-08	-0.550119133	0.433	0.663	0.001858584
RORA	8.81E-08	0.478023726	0.8	0.621	0.001921342
LY9	9.24E-08	-0.477342349	0.075	0.325	0.002015671
RANGAP1	9.69E-08	0.463432893	0.175	0.042	0.002112526
OBFC1	1.14E-07	-0.542159515	0.058	0.299	0.002489152
EZR	1.26E-07	0.528750588	0.675	0.479	0.002758562
PHLDB2	1.41E-07	0.303201309	0.108	0.014	0.003065033
SYNRG	1.41E-07	-0.528826606	0.142	0.407	0.003081174
ANKRD28	1.49E-07	0.551322962	0.408	0.196	0.003257117
LCP2	1.56E-07	-0.512245391	0.317	0.593	0.003394547
TMIGD2	1.68E-07	-0.521588731	0.183	0.465	0.00366613
THBS1	1.83E-07	0.35958617	0.133	0.024	0.00398907
DDX21	2.12E-07	0.617916063	0.55	0.367	0.00462603
ARRB2	2.22E-07	-0.531701287	0.125	0.373	0.004850703
ACAP1	2.24E-07	-0.528653216	0.325	0.601	0.004875057
SPPL2A	2.44E-07	0.46130434	0.375	0.168	0.0053189
SURF4	2.50E-07	0.489316259	0.517	0.301	0.005452937
TSC22D3	2.61E-07	0.363007118	0.942	0.9	0.005687609
BHLHE40	2.75E-07	0.438346154	0.617	0.391	0.005991192
CLIC1	2.75E-07	-0.358585725	0.733	0.88	0.006000499
CAMK4	2.87E-07	0.476593247	0.642	0.435	0.006252098
SAMHD1	2.91E-07	-0.542015247	0.092	0.331	0.006355981
NFATC2	2.96E-07	0.503920924	0.433	0.23	0.006457733
TMC8	2.97E-07	-0.577388748	0.15	0.391	0.006477451
PRMT2	3.17E-07	-0.515458081	0.217	0.491	0.006910204
PPP1R16B	3.17E-07	0.437541559	0.258	0.092	0.006914821
CD244	3.19E-07	-0.555906351	0.017	0.216	0.006946897
ATRX	3.27E-07	-0.521051321	0.192	0.449	0.007135604
EIF5A	3.30E-07	0.477220694	0.642	0.461	0.00720674
EMD	3.56E-07	0.475248765	0.5	0.297	0.007763872
EPAS1	4.24E-07	0.335492575	0.133	0.026	0.009240939
CD3G	4.49E-07	-0.493499574	0.417	0.655	0.009786183
CDKN1B	4.52E-07	-0.594837738	0.183	0.431	0.00984777
ODF3B	4.63E-07	0.386643407	0.133	0.026	0.01010515
CAPZB	4.65E-07	-0.419187462	0.367	0.651	0.010136027
IER2	4.74E-07	-0.470252442	0.892	0.938	0.010330723
TRAF3	4.89E-07	0.388846856	0.167	0.042	0.010659284
DHRS3	4.90E-07	0.435153254	0.308	0.132	0.01068515
ACAA2	5.13E-07	-0.426668192	0.067	0.305	0.011197513
RCSD1	5.22E-07	-0.520590302	0.117	0.361	0.011394172
STARD10	5.58E-07	0.318278852	0.133	0.026	0.012171095
SQSTM1	6.18E-07	0.457544742	0.842	0.689	0.013484317
ADNP2	6.20E-07	0.326125751	0.117	0.02	0.013529394
TNFAIP3	6.51E-07	0.399118531	0.883	0.762	0.014192336
RNF19A	6.52E-07	0.521856445	0.5	0.309	0.0142149
DENND4A	6.73E-07	0.520644472	0.383	0.194	0.014671586
BTN2A2	6.95E-07	0.290394395	0.125	0.024	0.015163815
ALB	7.45E-07	0.399692624	0.192	0.056	0.01624428
JUNB	8.35E-07	-0.35189677	0.933	0.972	0.018210315
HSPA8	8.59E-07	0.667469513	0.925	0.904	0.018737369
DUSP2	8.78E-07	-0.376523877	0.933	0.93	0.019146103
RBBP8	8.98E-07	0.330932766	0.133	0.028	0.019573403
BIN2	9.25E-07	-0.506001902	0.267	0.509	0.020182879
JOSD1	1.04E-06	0.400577703	0.217	0.072	0.022644001
PABPC1	1.09E-06	0.343965685	0.925	0.932	0.023817823
PELI1	1.23E-06	0.360768007	0.158	0.04	0.026854403
JUN	1.32E-06	-0.482399394	0.767	0.922	0.028869574
CYB5A	1.44E-06	0.509803614	0.3	0.136	0.031458579
IL16	1.47E-06	-0.447688236	0.125	0.361	0.032075825
POLR3E	1.47E-06	0.425252058	0.192	0.06	0.032095357
NR3C1	1.47E-06	0.465736435	0.508	0.311	0.032101597
CTD-3252C9.4	1.52E-06	0.548056757	0.367	0.182	0.033224319
GRAP2	1.62E-06	-0.491997639	0.033	0.222	0.035414161
BTG2	1.69E-06	-0.552210464	0.533	0.733	0.036945896
CCDC85B	1.86E-06	-0.417673438	0.225	0.479	0.0405769
CISH	2.04E-06	-0.464114007	0.033	0.224	0.04456259
SLAMF6	2.14E-06	-0.455105576	0.042	0.236	0.046669341

LPIN1	2.15E-06	0.529575565	0.383	0.2	0.046876348
CDK6	2.15E-06	-0.472559599	0.108	0.345	0.046974819
CENPM	2.25E-06	0.275502983	0.125	0.026	0.049058206
NCR3	2.26E-06	-0.451205167	0.475	0.695	0.049388079
RGS19	2.28E-06	-0.429347942	0.075	0.291	0.049714563

List of differentially expressed genes of IL7R_CD4 T in overall stage (PR versus PP)

gene	p_val	avg_logFC	pct.1	pct.2	p_val_adj
RPS29	1.52E-24	0.556979753	0.994	0.974	3.30E-20
DEFA3	4.36E-23	-1.001018821	0.051	0.554	9.50E-19
MYL12A	7.77E-22	-0.741343487	0.756	0.961	1.70E-17
FAM177A1	1.33E-21	1.307093474	0.596	0.167	2.91E-17
HSPE1	7.88E-21	1.624640777	0.75	0.412	1.72E-16
HSPD1	1.35E-20	1.8221006	0.737	0.425	2.94E-16
S100A4	2.40E-19	-0.743160228	0.731	0.97	5.23E-15
ACTB	2.31E-17	-0.515506892	0.994	1	5.04E-13
HBB	2.51E-17	-1.351868715	0.096	0.528	5.47E-13
HSP90AA1	5.00E-17	1.697238944	0.859	0.785	1.09E-12
BATF	7.11E-16	0.847446294	0.679	0.305	1.55E-11
HSPH1	1.07E-15	1.577276677	0.551	0.215	2.33E-11
SRGN	1.15E-15	0.638340744	0.981	0.957	2.52E-11
RPS27	3.24E-14	0.281796867	1	1	7.07E-10
CCND3	5.00E-14	-0.613219986	0.34	0.768	1.09E-09
TRAF3IP3	5.37E-14	-0.832860777	0.103	0.455	1.17E-09
AIM1	7.05E-14	0.911845994	0.628	0.335	1.54E-09
HSPB1	7.79E-14	1.262886726	0.647	0.369	1.70E-09
PFN1	1.68E-13	-0.5148979	0.904	0.94	3.67E-09
MTHFD2	5.79E-13	0.838128558	0.5	0.185	1.26E-08
HSP90AB1	7.65E-13	0.999257277	0.853	0.815	1.67E-08
FTH1	9.20E-13	0.698154656	1	0.987	2.01E-08
SOD2	4.74E-12	1.043264846	0.462	0.167	1.03E-07
CD52	6.57E-12	-0.558459913	0.66	0.88	1.43E-07
CREM	1.24E-11	0.805921847	0.622	0.33	2.70E-07
LDHA	3.55E-11	0.63736976	0.782	0.588	7.74E-07
CORO1A	4.86E-11	-0.549362314	0.545	0.803	1.06E-06
LCK	7.16E-11	-0.616837629	0.282	0.631	1.56E-06
CNOT6L	8.79E-11	0.74568909	0.481	0.21	1.92E-06
RPL36A	4.43E-10	0.435524457	0.897	0.854	9.65E-06
NOP58	6.68E-10	0.698013768	0.513	0.245	1.46E-05
SOD1	2.55E-09	0.702413187	0.686	0.536	5.56E-05
DRAP1	3.00E-09	-0.637856801	0.122	0.395	6.54E-05
TSC22D4	3.05E-09	-0.583622685	0.077	0.343	6.65E-05
STAT3	4.39E-09	0.630391092	0.532	0.27	9.57E-05
PTPN6	5.11E-09	-0.597037808	0.077	0.335	0.000111495
MT-ND3	5.16E-09	0.430965199	0.987	1	0.000112517
MT2A	8.74E-09	2.536929877	0.628	0.425	0.000190504
SLC9A3R1	1.24E-08	-0.534246668	0.167	0.455	0.000270945
HSPA1A	1.33E-08	1.144135147	0.66	0.429	0.000290898
BIRC3	1.77E-08	0.835732366	0.474	0.227	0.000386467
FERMT3	2.03E-08	-0.44550467	0.071	0.305	0.000442249
PPP1R16B	3.57E-08	0.505298997	0.186	0.026	0.000778559
RELB	5.32E-08	0.646818247	0.301	0.094	0.001160128
SKAP1	6.05E-08	-0.52829682	0.218	0.494	0.001318613
SLC7A5	7.57E-08	0.67589527	0.314	0.107	0.001649919
PPP1R18	7.69E-08	-0.542822097	0.186	0.451	0.001677928
SELL	1.14E-07	1.01919581	0.314	0.112	0.002492119
RPS19BP1	1.31E-07	-0.460223103	0.109	0.352	0.002850894
HLA-B	1.60E-07	0.282726781	0.994	0.987	0.003488812
CFL1	1.64E-07	-0.311353787	0.808	0.944	0.003579695
AQP3	1.78E-07	-0.513086317	0.167	0.425	0.003889408
ARID5A	1.97E-07	0.629422954	0.397	0.185	0.004299492
LMNA	2.66E-07	0.79119022	0.397	0.176	0.005792176
BAZ1A	3.36E-07	0.662388582	0.442	0.236	0.007337442
TBC1D10C	3.43E-07	-0.532880153	0.128	0.356	0.007471988
MT-ND5	3.84E-07	-0.323721705	0.917	0.983	0.008376805
HBA2	5.01E-07	-0.634928076	0.006	0.163	0.01092333
KIAA1551	5.51E-07	-0.581266831	0.244	0.506	0.012022523

NFKB2	6.26E-07	0.475831554	0.231	0.06	0.013649773
RASSF7	6.76E-07	-0.473891246	0.032	0.206	0.014732614
CD74	6.94E-07	0.687799864	0.667	0.481	0.015137282
LBH	7.29E-07	-0.45403096	0.321	0.601	0.015898803
FKBP4	7.68E-07	0.7086907	0.288	0.112	0.016745845
PELI1	8.14E-07	0.631854878	0.25	0.082	0.017743291
CLIC1	8.24E-07	-0.42175961	0.558	0.79	0.017976907
ACAP1	8.52E-07	-0.455624146	0.327	0.588	0.01858159
ITK	9.43E-07	0.604964962	0.551	0.348	0.020561554
BCL3	1.05E-06	0.455642434	0.231	0.064	0.022840282
LY9	1.21E-06	-0.42668225	0.045	0.227	0.026453379
CFH	1.28E-06	-0.470878365	0.038	0.215	0.027877562
RNF19A	1.29E-06	0.670436872	0.385	0.193	0.028145769
CSK	1.31E-06	-0.399290231	0.064	0.266	0.028672905
PA2G4	1.44E-06	-0.433253828	0.173	0.416	0.031434314
PRDM1	1.45E-06	0.614978413	0.551	0.365	0.031620146
RAC2	1.58E-06	-0.401358619	0.615	0.807	0.034518018
ITGA4	1.68E-06	-0.536484557	0.115	0.326	0.036566382
REEP5	1.88E-06	-0.477254912	0.154	0.386	0.04102491
SOCS3	1.97E-06	0.609848661	0.397	0.206	0.043030079
PPIA	2.06E-06	-0.276070613	0.891	0.974	0.044899266

List of differentially expressed genes of PTGDS_NK in overall stage (PR versus PP)

gene	p_val	avg_logFC	pct.1	pct.2	p_val_adj
MYOM2	4.79E-24	-1.355786341	0.112	0.672	1.04E-19
DEFA3	4.24E-23	-1.384386246	0.034	0.541	9.24E-19
CD3E	4.26E-21	-1.509850098	0.028	0.484	9.28E-17
ACTB	1.02E-18	-0.569476746	1	1	2.22E-14
RPS29	5.65E-18	0.688678538	0.983	0.91	1.23E-13
NFKBIA	2.08E-17	0.75902464	0.978	0.861	4.53E-13
LAIR2	3.67E-17	1.458203139	0.603	0.131	8.00E-13
B2M	3.75E-17	0.361496712	1	1	8.17E-13
GNLY	1.05E-16	-0.844731346	0.536	0.951	2.30E-12
HLA-B	3.95E-16	0.386701843	1	1	8.62E-12
LCK	5.20E-16	-0.909483581	0.151	0.607	1.13E-11
CST7	6.37E-16	0.641902426	1	0.959	1.39E-11
HBB	1.04E-15	-0.98332495	0.061	0.467	2.27E-11
GZMB	1.24E-12	0.63156238	0.972	0.975	2.69E-08
PRDM1	2.06E-12	0.882080672	0.715	0.434	4.50E-08
CORO1A	2.51E-12	-0.621554786	0.536	0.885	5.46E-08
PFN1	2.52E-12	-0.389708812	0.972	0.992	5.50E-08
SRGN	5.24E-12	0.477464808	0.989	1	1.14E-07
S100A4	6.68E-12	-0.661859838	0.743	0.959	1.46E-07
ANXA6	1.71E-11	-0.69604325	0.179	0.582	3.73E-07
HCST	2.09E-11	-0.54876317	0.788	0.951	4.56E-07
MT-ND3	2.46E-11	0.452744208	0.983	0.992	5.36E-07
RPS19	2.76E-11	-0.489657988	0.994	1	6.02E-07
LAT	8.97E-11	-0.682080026	0.067	0.377	1.96E-06
HIF1A	1.06E-10	1.003043222	0.531	0.221	2.31E-06
TNFAIP8	1.11E-10	-0.636761295	0.039	0.32	2.41E-06
H3F3B	4.81E-10	0.407361994	0.994	0.992	1.05E-05
CCL4	1.83E-09	0.605482301	0.978	1	3.99E-05
BIN1	2.15E-09	-0.647287769	0.101	0.41	4.69E-05
NEAT1	2.30E-09	0.602814993	0.849	0.689	5.02E-05
HLA-DQB1	6.91E-09	-0.501861064	0.067	0.336	0.000150677
CALM1	7.08E-09	-0.420293951	0.788	0.992	0.000154374
RPS8	1.01E-08	-0.310153672	0.983	1	0.000219704
RGS19	1.96E-08	-0.584638235	0.084	0.361	0.000428155
SLC9A3R1	2.11E-08	-0.600921691	0.307	0.664	0.00046047
IGKV2-24	3.13E-08	-0.745530624	0.022	0.221	0.000683294
NACA	4.69E-08	-0.377430398	0.866	0.967	0.001023491
SH3BP1	4.86E-08	-0.602162487	0.106	0.385	0.001059167
HRASLS2	5.85E-08	-0.606876417	0.05	0.279	0.001275419
GBP2	6.19E-08	0.786426727	0.514	0.295	0.001350658
CD69	7.14E-08	0.716002354	0.939	0.893	0.00155777
SYTL3	8.21E-08	0.715130772	0.715	0.566	0.001791403
TNFAIP3	8.53E-08	0.695807644	0.777	0.615	0.001861236

SRSF2	8.66E-08	0.634619194	0.67	0.467	0.00188916
MALAT1	1.07E-07	0.330549152	1	0.992	0.00233874
KIR3DL2	1.10E-07	0.878467805	0.447	0.18	0.00238829
TC2N	1.11E-07	-0.54612214	0.045	0.262	0.002416523
CD99	1.16E-07	-0.387359271	0.709	0.943	0.002531018
HLA-DRB1	1.50E-07	-0.444190224	0.156	0.467	0.003267071
ISG20	1.64E-07	0.552724953	0.777	0.598	0.003583229
FCER1G	2.25E-07	-0.43977654	0.771	0.885	0.004907017
CXCR4	2.30E-07	-0.514938579	0.62	0.861	0.005025484
RGS2	2.70E-07	-0.625662036	0.056	0.27	0.005898091
ZYX	2.86E-07	-0.553314451	0.168	0.467	0.006233135
ZMYM6NB	3.01E-07	-0.526545482	0.05	0.262	0.006555774
SH2D1A	3.73E-07	-0.536151092	0.151	0.434	0.008144837
RPL24	3.84E-07	-0.327863816	0.866	0.967	0.008378823
RPL7	4.63E-07	-0.374932019	0.676	0.893	0.010100193
IL2RB	4.78E-07	-0.499599734	0.285	0.639	0.010418564
LSP1	5.50E-07	-0.387260404	0.503	0.869	0.011993761
EIF3G	5.96E-07	-0.571389776	0.464	0.738	0.01299867
RCBTB2	6.30E-07	-0.427085364	0.017	0.18	0.01374639
C12orf75	6.58E-07	-0.468036104	0.212	0.549	0.01434571
FAM65B	6.75E-07	-0.552824815	0.156	0.426	0.014726923
HLA-E	7.15E-07	0.314275024	0.961	0.992	0.015590948
CAP1	1.06E-06	-0.443663454	0.346	0.68	0.023137424
RNF115	1.17E-06	0.771820069	0.397	0.18	0.02552955
PPP2R2B	1.17E-06	-0.488965538	0.045	0.238	0.02557866
MRPL51	1.19E-06	-0.448493788	0.095	0.336	0.025883629
GADD45B	1.34E-06	0.569403949	0.888	0.918	0.029257776
TBC1D10C	1.41E-06	-0.353190588	0.218	0.549	0.030704055
CCDC85B	1.41E-06	-0.439424589	0.229	0.549	0.030777359
RPL7A	1.44E-06	-0.260033291	0.961	0.992	0.031464072
BATF	1.50E-06	0.812736459	0.436	0.221	0.032764251
C1orf162	1.88E-06	-0.507513196	0.19	0.475	0.041079037
NCR3	1.92E-06	-0.537674766	0.223	0.516	0.041762439
C11orf73	1.94E-06	-0.44198709	0.056	0.254	0.042227743

List of differentially expressed genes of TRBV9_CD8 Teff in overall stage (PR versus PP)

gene	p_val	avg_logFC	pct.1	pct.2	p_val_adj
RPS29	4.79E-22	0.68428191	0.983	0.949	1.05E-17
DEFA3	2.28E-19	-1.270826667	0.033	0.574	4.97E-15
TRBV13	3.12E-18	2.180406841	0.45	0	6.80E-14
MT-ND3	2.82E-16	0.728094786	1	0.985	6.15E-12
TRAV38-2DV8	2.30E-15	-1.501787488	0.075	0.537	5.03E-11
S100A4	3.34E-15	-0.647077464	0.925	0.985	7.28E-11
HBB	1.59E-14	-1.09829183	0.108	0.574	3.46E-10
TRBV9	5.81E-14	-1.452391531	0.142	0.574	1.27E-09
HLA-B	5.13E-13	0.363688041	1	1	1.12E-08
CD52	6.84E-13	-0.669349715	0.658	0.926	1.49E-08
SYTL3	7.33E-13	0.909959061	0.75	0.471	1.60E-08
CD3E	2.57E-12	-0.537129068	0.833	0.963	5.62E-08
RPS27	4.36E-12	0.290621713	1	0.993	9.52E-08
TRAV17	7.15E-11	1.173506145	0.275	0	1.56E-06
CNN2	1.80E-10	-0.734637862	0.217	0.647	3.93E-06
NEAT1	1.97E-10	0.820395605	0.783	0.544	4.30E-06
GZMB	4.15E-10	0.762089835	0.967	0.838	9.06E-06
VIM	1.00E-09	-0.592702826	0.658	0.904	2.19E-05
ISG20	2.08E-09	0.67247969	0.775	0.566	4.53E-05
TMSB4X	2.24E-09	-0.258905126	1	1	4.89E-05
DUSP2	7.54E-09	-0.654234574	0.725	0.919	0.000164501
SH2D2A	8.69E-09	0.782668465	0.508	0.199	0.000189512
PRDM1	1.28E-08	0.918105594	0.592	0.324	0.000278805
NFKBIA	1.31E-08	0.590678413	0.883	0.596	0.000286485
HOPX	1.79E-08	-0.652276116	0.35	0.662	0.000389521
CD160	1.96E-08	-0.644262136	0.008	0.25	0.000428248
KLRB1	7.20E-08	0.841438165	0.592	0.257	0.001570834
CTSW	1.77E-07	0.539477651	0.867	0.743	0.003861132
OSTF1	2.00E-07	-0.524380117	0.267	0.603	0.004353187
S100A6	2.94E-07	-0.447286732	0.742	0.963	0.00641311

CORO1A	3.38E-07	-0.472183146	0.675	0.868	0.007370483
TOB1	1.55E-06	-0.612445708	0.092	0.346	0.033877723
PTPRCAP	2.07E-06	-0.361027348	0.842	0.971	0.04518204

List of differentially expressed genes of STMN1_NK-cycling in overall stage (PR versus PP)

gene	p_val	avg_logFC	pct.1	pct.2	p_val_adj
NFKB2	6.54E-11	0.900508962	0.692	0.139	1.43E-06
RELB	1.11E-10	1.165243379	0.692	0.156	2.42E-06
HSPD1	1.85E-10	1.77147971	0.923	0.475	4.03E-06
HSP90AB1	4.31E-10	1.43383896	1	0.639	9.39E-06
HSP90AA1	5.12E-10	1.232263446	1	0.984	1.12E-05
PTPRCAP	4.84E-09	-0.860364569	0.885	0.984	0.00010546
HSPE1	8.64E-09	1.654427594	0.885	0.615	0.000188356
FKBP4	3.87E-08	0.898351381	0.538	0.098	0.000843723
LMNA	8.17E-08	0.772231303	0.462	0.066	0.001780662
SRGN	8.38E-08	0.651059025	1	0.992	0.001826843
SYTL3	8.53E-08	0.892174446	0.885	0.5	0.001859221
BCL3	1.57E-07	0.668454517	0.462	0.074	0.003419253
SQSTM1	1.62E-07	0.931549761	0.962	0.713	0.003542064
BATF	2.39E-07	0.925114834	0.808	0.369	0.00521488
HSPH1	2.80E-07	2.207696395	0.692	0.311	0.006116694
CORO1A	5.85E-07	-0.650329923	0.885	0.984	0.012757564
MYL12A	9.57E-07	-0.65924265	0.962	1	0.020870756
CD72	9.89E-07	0.526777336	0.385	0.049	0.021566206
KIR2DL4	1.18E-06	0.706082029	0.423	0.074	0.025742752
CBLB	1.19E-06	0.717224172	0.769	0.336	0.025967567
TNFRSF4	1.28E-06	0.503912824	0.231	0.008	0.027934818

List of differentially expressed genes of XCL2_NK in overall stage (PR versus PP)

gene	p_val	avg_logFC	pct.1	pct.2	p_val_adj
HSP90AA1	3.31E-11	1.939212301	0.966	0.8	7.23E-07
SH2D2A	2.83E-10	1.312662137	0.724	0.2	6.17E-06
HSPD1	4.95E-10	2.202340767	0.759	0.291	1.08E-05
HSPH1	8.40E-09	1.972242036	0.586	0.136	0.000183272
PFN1	1.02E-08	-0.722329294	0.966	0.973	0.000221687
CREM	1.41E-08	1.205776613	0.828	0.318	0.000306942
HSPE1	2.13E-08	1.829115419	0.759	0.355	0.000463717
TNFAIP3	4.45E-08	1.627858318	0.586	0.136	0.0009706
TNFRSF9	9.98E-08	1.269306043	0.379	0.036	0.002175432
MYL12A	1.55E-07	-0.777353872	0.69	0.945	0.003380626
NFKBIA	7.23E-07	1.085989628	0.897	0.564	0.015773513
HSPA1A	7.52E-07	1.911872521	0.724	0.4	0.01639998
STAT3	1.78E-06	1.006871949	0.655	0.282	0.038719168

List of differentially expressed genes of XCL1_NK in overall stage (PR versus PP)

gene	p_val	avg_logFC	pct.1	pct.2	p_val_adj
NFKB2	9.33E-10	1.069013128	0.52	0.034	2.03E-05
FAM177A1	1.00E-08	1.163500715	0.88	0.402	0.000218674

List of differentially expressed genes of STMN1_T-cycling in overall stage (PR versus PP)

gene	p_val	avg_logFC	pct.1	pct.2	p_val_adj
HSP90AA1	1.19E-10	1.868282445	1	0.982	2.60E-06
RELB	2.53E-09	1.230377656	0.778	0.246	5.51E-05
SRGN	4.72E-09	0.77154237	1	0.93	0.000102987
UBE2C	2.35E-08	-1.064196191	0.694	0.93	0.000512069
NFKB2	2.50E-08	1.098389648	0.694	0.175	0.000544447
HSPD1	3.49E-08	1.803146412	0.861	0.579	0.000761983

FEZ1	6.18E-08	1.2011413	0.639	0.158	0.001348625
CD7	2.17E-07	0.934683896	0.917	0.719	0.004724883
CST7	2.67E-07	0.778646579	0.972	0.789	0.005812949
MT2A	3.53E-07	1.446208757	0.944	0.895	0.007687614
HLA-A	4.19E-07	0.484245555	1	0.947	0.009148375
HSP90AB1	6.49E-07	1.013755736	1	0.825	0.014147395
IL7R	9.27E-07	0.964529292	0.972	0.772	0.020223979
STAT3	9.89E-07	0.750314052	0.75	0.263	0.021569618
SLC7A5	1.10E-06	0.783049428	0.861	0.404	0.024080662
DEFA3	1.39E-06	-1.005818638	0.056	0.579	0.030402621

List of differentially expressed genes of TRDV2_γδ T in overall stage (PR versus PP)

gene	p_val	avg_logFC	pct.1	pct.2	p_val_adj
NA					

Table 17: List of DEGs of NK/T cell clusters in cold preservation stage (EP versus PP)

List of differentially expressed genes of CD8B_CD8 Tem in cold preservation stage (EP versus PP)

gene	p_val	avg_logFC	pct.1	pct.2	p_val_adj
MT2A	1.79E-26	1.519939992	0.696	0.444	3.90E-22
IGLV2-23	2.13E-23	0.81934981	0.21	0.01	4.64E-19
IGLV2-14	4.71E-16	0.625417176	0.313	0.104	1.03E-11
IGKV2-30	4.93E-15	0.638919979	0.235	0.059	1.08E-10
JUN	4.58E-14	0.417789208	0.848	0.718	1.00E-09
MT1X	7.30E-13	0.806331113	0.443	0.26	1.59E-08
IGKV2-24	2.83E-11	-0.491907125	0.043	0.184	6.16E-07
MT1E	5.15E-11	1.046989459	0.23	0.086	1.12E-06
CXCR4	5.37E-10	-0.345994827	0.884	0.939	1.17E-05
SRGN	2.24E-08	-0.252315208	0.944	0.969	0.000489083
HSP90AA1	7.61E-08	0.323542503	0.819	0.753	0.001659745
CCL4	1.69E-07	-0.47808937	0.94	0.957	0.003688326
IGKV4-1	3.59E-07	0.450329332	0.302	0.172	0.007830559
CCL4L2	3.86E-07	-0.611460126	0.732	0.8	0.008412857
FUS	2.12E-06	-0.305655656	0.57	0.714	0.046180334

List of differentially expressed genes of GNLY_NK in cold preservation stage (EP versus PP)

gene	p_val	avg_logFC	pct.1	pct.2	p_val_adj
MT2A	1.79E-26	1.519939992	0.696	0.444	3.90E-22
IGLV2-23	2.13E-23	0.81934981	0.21	0.01	4.64E-19
IGLV2-14	4.71E-16	0.625417176	0.313	0.104	1.03E-11
IGKV2-30	4.93E-15	0.638919979	0.235	0.059	1.08E-10
JUN	4.58E-14	0.417789208	0.848	0.718	1.00E-09
MT1X	7.30E-13	0.806331113	0.443	0.26	1.59E-08
IGKV2-24	2.83E-11	-0.491907125	0.043	0.184	6.16E-07
MT1E	5.15E-11	1.046989459	0.23	0.086	1.12E-06
CXCR4	5.37E-10	-0.345994827	0.884	0.939	1.17E-05
SRGN	2.24E-08	-0.252315208	0.944	0.969	0.000489083
HSP90AA1	7.61E-08	0.323542503	0.819	0.753	0.001659745
CCL4	1.69E-07	-0.47808937	0.94	0.957	0.003688326
IGKV4-1	3.59E-07	0.450329332	0.302	0.172	0.007830559
CCL4L2	3.86E-07	-0.611460126	0.732	0.8	0.008412857
FUS	2.12E-06	-0.305655656	0.57	0.714	0.046180334

List of differentially expressed genes of CCL20_CD8 MAIT in cold preservation stage (EP versus PP)

gene	p_val	avg_logFC	pct.1	pct.2	p_val_adj
MT2A	4.73E-25	1.413204626	0.796	0.653	1.03E-20
IGLV2-23	5.04E-21	0.581831071	0.206	0.018	1.10E-16
MT1X	1.68E-18	0.906673469	0.516	0.291	3.66E-14
CCL4	1.18E-15	-0.617762444	0.958	0.976	2.57E-11
MT1E	3.07E-15	0.96455502	0.316	0.124	6.70E-11
CCL4L2	3.57E-15	-0.807311008	0.707	0.81	7.79E-11
IGKV2-24	9.17E-14	-0.456071671	0.048	0.21	2.00E-09
MT-ATP8	9.35E-13	-0.305787504	0.975	0.99	2.04E-08
IGKV3-15	2.84E-12	0.446128648	0.168	0.036	6.19E-08
IGLV2-11	4.50E-11	0.427504044	0.175	0.044	9.82E-07
IGLV2-14	1.27E-10	0.431086156	0.267	0.116	2.78E-06
PTGER4	9.81E-09	-0.35669941	0.499	0.655	0.000213944
CTD-3252C9.4	3.75E-07	0.389192823	0.307	0.182	0.008167649
PHLDA1	8.45E-07	-0.370494247	0.181	0.319	0.018430102

List of differentially expressed genes of IL7R_CD4 T in cold preservation stage (EP versus PP)

gene	p_val	avg_logFC	pct.1	pct.2	p_val_adj
IGLV2-23	2.46E-14	0.776266991	0.244	0.009	5.36E-10
MT2A	1.01E-09	1.690040158	0.599	0.425	2.21E-05
MT1X	1.14E-09	1.067418789	0.393	0.163	2.48E-05
MT1E	5.60E-09	1.134897518	0.244	0.056	0.000122104

IGLV2-11	1.51E-07	0.625309158	0.207	0.047	0.00328824
IGLV2-14	2.28E-07	0.541589087	0.281	0.099	0.004964001
FOSB	8.25E-07	0.255242271	0.748	0.536	0.018000527

List of differentially expressed genes of PTGDS_NK in cold preservation stage (EP versus PP)

gene	p_val	avg_logFC	pct.1	pct.2	p_val_adj
MT-ATP6	4.72E-10	0.360779687	0.993	1	1.03E-05
DUSP1	9.58E-07	0.490723835	0.936	0.861	0.020885859
MT2A	1.91E-06	1.055518805	0.794	0.721	0.041727368

List of differentially expressed genes of TRBV9_CD8 Teff in cold preservation stage (EP versus PP)

gene	p_val	avg_logFC	pct.1	pct.2	p_val_adj
MT2A	7.94E-12	1.614555982	0.876	0.654	1.73E-07
MT-ATP6	5.65E-11	0.457782371	1	0.978	1.23E-06
FOS	9.16E-09	0.855600894	0.819	0.588	0.000199737
IGLV2-23	1.56E-08	0.762055374	0.248	0.015	0.000340647
MT1E	7.52E-08	1.369606616	0.467	0.176	0.001639611

List of differentially expressed genes of STMN1_NK-cycling in cold preservation stage (EP versus PP)

gene	p_val	avg_logFC	pct.1	pct.2	p_val_adj
IGLV2-14	1.37E-07	0.582528702	0.33	0.057	0.002996482
IGLV2-23	1.43E-06	0.375927728	0.2	0.008	0.031148678

List of differentially expressed genes of XCL2_NK in cold preservation stage (EP versus PP)

gene	p_val	avg_logFC	pct.1	pct.2	p_val_adj
IGLV2-14	3.92E-07	0.917703789	0.356	0.073	0.008554032

List of differentially expressed genes of XCL1_NK in cold preservation stage (EP versus PP)

gene	p_val	avg_logFC	pct.1	pct.2	p_val_adj
IGLV2-23	1.60E-06	0.673911049	0.268	0.011	0.034893375

List of differentially expressed genes of STMN1_T-cycling in cold preservation stage (EP versus PP)

gene	p_val	avg_logFC	pct.1	pct.2	p_val_adj
NA					

List of differentially expressed genes of TRDV2_γδ T in cold preservation stage (EP versus PP)

gene	p_val	avg_logFC	pct.1	pct.2	p_val_adj
NA					

Table 18. List of markers information for B/Plasma cell clusters, related to Figure 6.

	p_val	avg_logFC	pct.1	pct.2	p_val_adj	gene	cluster	cell type
1	2.02E-178	1.903253941	0.592	0.014	4.41E-174	TCL1A	1	TCL1A_B cell
2	4.12E-142	1.436694916	0.916	0.475	8.98E-138	TXNIP	1	TCL1A_B cell
3	2.29E-165	1.377151425	0.995	0.856	5.00E-161	BTG1	1	TCL1A_B cell
4	3.13E-155	1.306186162	0.983	0.675	6.82E-151	CD37	1	TCL1A_B cell
5	3.08E-75	1.24399586	0.457	0.11	6.72E-71	FCER2	1	TCL1A_B cell
6	9.32E-138	1.239902014	0.96	0.766	2.03E-133	CXCR4	1	TCL1A_B cell
7	1.04E-88	1.233283308	0.358	0.022	2.26E-84	IL4R	1	TCL1A_B cell
8	1.97E-112	1.20093406	0.86	0.342	4.29E-108	CD69	1	TCL1A_B cell
9	1.55E-94	1.190490537	0.587	0.147	3.38E-90	LINC00926	1	TCL1A_B cell
10	9.72E-80	1.161304204	0.432	0.076	2.12E-75	BACH2	1	TCL1A_B cell
11	1.15E-106	1.139984245	0.916	0.736	2.50E-102	DUSP1	1	TCL1A_B cell
12	2.83E-53	1.118714893	0.4	0.118	6.18E-49	SESN1	1	TCL1A_B cell
13	3.67E-87	1.115070711	0.782	0.318	8.01E-83	CD83	1	TCL1A_B cell
14	7.08E-58	1.086702747	0.732	0.613	1.54E-53	CD55	1	TCL1A_B cell
15	4.55E-58	1.085517356	0.363	0.071	9.92E-54	STAG3	1	TCL1A_B cell
16	8.57E-121	1.066952153	0.998	0.676	1.87E-116	HLA-DRA	1	TCL1A_B cell
17	4.42E-20	1.03802117	0.314	0.17	9.64E-16	CLEC2B	1	TCL1A_B cell
18	2.09E-28	1.032883369	0.39	0.215	4.56E-24	YBX3	1	TCL1A_B cell
19	6.97E-66	1.023377505	0.658	0.329	1.52E-61	FCMR	1	TCL1A_B cell
20	1.49E-110	0.984742983	0.943	0.776	3.25E-106	LAPTM5	1	TCL1A_B cell
21	2.22E-97	0.972400564	0.96	0.941	4.85E-93	JUNB	1	TCL1A_B cell
22	5.11E-97	0.96654971	0.941	0.809	1.11E-92	HLA-DRB1	1	TCL1A_B cell
23	9.18E-58	0.964750798	0.836	0.736	2.00E-53	FOS	1	TCL1A_B cell
24	2.31E-57	0.958977596	0.398	0.096	5.03E-53	FAM129C	1	TCL1A_B cell
25	3.57E-65	0.951831601	0.445	0.109	7.79E-61	AFF3	1	TCL1A_B cell
26	7.10E-139	0.944758769	1	0.976	1.55E-134	RPS27	1	TCL1A_B cell
27	2.79E-157	0.944393469	1	0.993	6.09E-153	RPS19	1	TCL1A_B cell
28	3.39E-55	0.938827879	0.462	0.155	7.40E-51	HVCN1	1	TCL1A_B cell
29	3.93E-38	0.933296022	0.486	0.256	8.57E-34	FOXP1	1	TCL1A_B cell
30	2.79E-48	0.931242212	0.666	0.426	6.09E-44	SLC2A3	1	TCL1A_B cell
31	4.49E-45	0.926711455	0.562	0.297	9.80E-41	IRF8	1	TCL1A_B cell
32	2.07E-15	0.920255282	0.455	0.421	4.51E-11	TMEM123	1	TCL1A_B cell
33	7.45E-184	0.905581964	0.998	0.983	1.62E-179	RPL18A	1	TCL1A_B cell
34	3.22E-37	0.897403113	0.545	0.352	7.02E-33	CCND3	1	TCL1A_B cell
35	9.23E-45	0.894171868	0.464	0.181	2.01E-40	TUBA1A	1	TCL1A_B cell
36	3.47E-75	0.891292857	0.683	0.232	7.57E-71	LTB	1	TCL1A_B cell
37	2.28E-84	0.888365532	0.921	0.831	4.97E-80	TSC22D3	1	TCL1A_B cell
38	2.39E-55	0.883974459	0.295	0.041	5.20E-51	IGHD	1	TCL1A_B cell
39	2.35E-111	0.866249858	1	0.99	5.14E-107	CD74	1	TCL1A_B cell
40	1.18E-71	0.865430185	0.901	0.86	2.58E-67	ZFP36L2	1	TCL1A_B cell
41	4.17E-44	0.830770973	0.728	0.699	9.09E-40	GLTSCR2	1	TCL1A_B cell
42	1.43E-19	0.829731626	0.636	0.672	3.13E-15	GADD45B	1	TCL1A_B cell
43	6.40E-42	0.82961239	0.556	0.275	1.40E-37	REL	1	TCL1A_B cell
44	1.98E-78	0.829093129	0.892	0.646	4.32E-74	HLA-DPB1	1	TCL1A_B cell
45	4.87E-78	0.828094446	0.894	0.676	1.06E-73	CD52	1	TCL1A_B cell
46	3.65E-38	0.823728577	0.307	0.086	7.95E-34	FCRL1	1	TCL1A_B cell
47	1.19E-25	0.822016443	0.563	0.465	2.59E-21	BIRC3	1	TCL1A_B cell
48	2.86E-14	0.818773171	0.487	0.48	6.24E-10	RNASE6	1	TCL1A_B cell
49	1.74E-143	0.81842033	0.993	0.967	3.80E-139	RPL21	1	TCL1A_B cell
50	1.56E-127	0.816576626	0.998	0.98	3.39E-123	RPL39	1	TCL1A_B cell
51	2.48E-19	0.810617238	0.504	0.437	5.42E-15	SNX2	1	TCL1A_B cell
52	6.10E-140	0.80814405	1	0.979	1.33E-135	RPL32	1	TCL1A_B cell
53	2.54E-133	0.806746957	0.998	0.981	5.54E-129	RPL30	1	TCL1A_B cell
54	1.03E-80	0.805512547	0.921	0.799	2.25E-76	HLA-DQB1	1	TCL1A_B cell
55	1.02E-55	0.803352225	0.757	0.5	2.22E-51	ZFP36L1	1	TCL1A_B cell
56	2.94E-15	0.797468064	0.528	0.535	6.42E-11	RHOH	1	TCL1A_B cell
57	4.30E-43	0.793029585	0.41	0.141	9.37E-39	TRIM22	1	TCL1A_B cell
58	2.17E-77	0.790043661	0.985	0.97	4.72E-73	FTH1	1	TCL1A_B cell
59	1.51E-157	0.788740235	1	0.992	3.29E-153	RPL13	1	TCL1A_B cell
60	4.78E-78	0.78746167	0.909	0.778	1.04E-73	HLA-DPA1	1	TCL1A_B cell
61	2.35E-12	0.786880639	0.42	0.357	5.13E-08	STK17A	1	TCL1A_B cell
62	9.85E-35	0.782732603	0.418	0.185	2.15E-30	LBH	1	TCL1A_B cell
63	2.87E-32	0.77662535	0.27	0.079	6.26E-28	FAM26F	1	TCL1A_B cell
64	1.61E-23	0.775854706	0.309	0.14	3.52E-19	[illegible]P11-693J15.	1	TCL1A_B cell
65	5.50E-43	0.77250797	0.744	0.77	1.20E-38	RPS4Y1	1	TCL1A_B cell
66	1.48E-19	0.768132389	0.53	0.467	3.23E-15	SP100	1	TCL1A_B cell
67	1.00E-10	0.767008596	0.305	0.224	2.19E-06	BTLA	1	TCL1A_B cell
68	3.83E-13	0.765474594	0.428	0.358	8.34E-09	SELL	1	TCL1A_B cell

69	1.03E-23	0.763416103	0.278	0.116	2.24E-19	CAMK2D	1	TCL1A_B cell
70	1.73E-124	0.760519944	0.998	0.963	3.77E-120	RPL12	1	TCL1A_B cell
71	7.91E-125	0.758614669	0.998	0.984	1.73E-120	RPL34	1	TCL1A_B cell
72	3.14E-132	0.754155297	0.995	0.972	6.84E-128	RPL26	1	TCL1A_B cell
73	7.34E-77	0.754023849	0.798	0.295	1.60E-72	MS4A1	1	TCL1A_B cell
74	1.72E-38	0.753202401	0.332	0.096	3.76E-34	CD72	1	TCL1A_B cell
75	5.15E-134	0.752358485	1	0.983	1.12E-129	RPS23	1	TCL1A_B cell
76	2.47E-69	0.749989668	0.877	0.599	5.39E-65	HLA-DQA1	1	TCL1A_B cell
77	1.71E-127	0.749058744	1	0.983	3.73E-123	RPS27A	1	TCL1A_B cell
78	1.77E-137	0.744867002	0.998	0.975	3.85E-133	RPS3A	1	TCL1A_B cell
79	1.20E-44	0.743512181	0.75	0.658	2.63E-40	ZFAS1	1	TCL1A_B cell
80	2.13E-44	0.739411329	0.779	0.716	4.65E-40	HLA-DRB5	1	TCL1A_B cell
81	9.97E-44	0.736447297	0.749	0.705	2.17E-39	COMMD6	1	TCL1A_B cell
82	1.10E-36	0.73530169	0.196	0.025	2.40E-32	PCDH9	1	TCL1A_B cell
83	3.17E-20	0.733029127	0.411	0.251	6.90E-16	ID3	1	TCL1A_B cell
84	1.86E-108	0.724140528	0.985	0.946	4.06E-104	RPSA	1	TCL1A_B cell
85	1.52E-13	0.723606223	0.481	0.446	3.31E-09	RCSD1	1	TCL1A_B cell
86	3.26E-114	0.720423676	0.993	0.961	7.11E-110	RPL22	1	TCL1A_B cell
87	1.06E-34	0.719472796	0.727	0.776	2.31E-30	PNRC1	1	TCL1A_B cell
88	3.69E-88	0.713885204	0.943	0.914	8.04E-84	PFDN5	1	TCL1A_B cell
89	4.75E-131	0.708149539	0.992	0.974	1.04E-126	RPS5	1	TCL1A_B cell
90	1.13E-124	0.706091535	0.997	0.978	2.47E-120	RPL37	1	TCL1A_B cell
91	7.71E-108	0.70481687	0.985	0.955	1.68E-103	RPL10A	1	TCL1A_B cell
92	1.01E-14	0.70470532	0.344	0.204	2.21E-10	AREG	1	TCL1A_B cell
93	4.72E-15	0.703162542	0.474	0.41	1.03E-10	TNFAIP8	1	TCL1A_B cell
94	1.04E-09	0.702697878	0.47	0.469	2.27E-05	DNAJB1	1	TCL1A_B cell
95	7.11E-10	0.700527887	0.538	0.649	1.55E-05	CD79B	1	TCL1A_B cell
96	1.62E-15	0.69885842	0.573	0.608	3.52E-11	LIMD2	1	TCL1A_B cell
97	6.68E-20	0.698533893	0.644	0.65	1.46E-15	DDIT4	1	TCL1A_B cell
98	3.48E-14	0.695910366	0.568	0.61	7.58E-10	TAGAP	1	TCL1A_B cell
99	5.29E-138	0.688459574	1	0.988	1.15E-133	RPL18	1	TCL1A_B cell
100	1.69E-34	0.685130245	0.396	0.154	3.69E-30	1-Mar	1	TCL1A_B cell
101	1.06E-33	0.683547272	0.185	0.026	2.31E-29	CD200	1	TCL1A_B cell
102	2.10E-121	0.673909632	0.998	0.967	4.59E-117	RPL35A	1	TCL1A_B cell
103	4.49E-20	0.669211465	0.32	0.158	9.80E-16	FAM65B	1	TCL1A_B cell
104	4.84E-124	0.668465748	0.998	0.975	1.06E-119	RPS10	1	TCL1A_B cell
105	3.27E-10	0.664024728	0.255	0.162	7.14E-06	MYADM	1	TCL1A_B cell
106	2.81E-25	0.663789758	0.698	0.791	6.12E-21	CIRBP	1	TCL1A_B cell
107	3.67E-27	0.659696109	0.285	0.098	8.01E-23	MTSS1	1	TCL1A_B cell
108	3.42E-88	0.65876637	0.987	0.973	7.45E-84	EIF1	1	TCL1A_B cell
109	1.04E-45	0.658170588	0.784	0.849	2.27E-41	SARAF	1	TCL1A_B cell
110	3.93E-07	0.657954497	0.408	0.416	0.008565061	SNHG7	1	TCL1A_B cell
111	5.36E-121	0.655797484	0.998	0.974	1.17E-116	RPL29	1	TCL1A_B cell
112	1.33E-101	0.64682966	0.997	0.963	2.89E-97	RPS25	1	TCL1A_B cell
113	1.20E-09	0.643538161	0.535	0.649	2.62E-05	NCF1	1	TCL1A_B cell
114	4.42E-26	0.641808749	0.31	0.122	9.64E-22	HLA-DMB	1	TCL1A_B cell
115	4.28E-22	0.639108184	0.691	0.803	9.34E-18	BRD2	1	TCL1A_B cell
116	3.17E-93	0.635640107	0.981	0.957	6.91E-89	RPL13A	1	TCL1A_B cell
117	5.30E-08	0.628130535	0.388	0.35	0.001155979	ANKRD44	1	TCL1A_B cell
118	5.45E-64	0.627713727	0.916	0.918	1.19E-59	RPL38	1	TCL1A_B cell
119	1.61E-106	0.626301191	1	0.983	3.51E-102	RPS21	1	TCL1A_B cell
120	7.84E-28	0.626225826	0.288	0.095	1.71E-23	CD22	1	TCL1A_B cell
121	1.74E-112	0.624211263	0.997	0.987	3.79E-108	FAU	1	TCL1A_B cell
122	4.50E-116	0.624125265	0.998	0.973	9.81E-112	RPS13	1	TCL1A_B cell
123	1.05E-28	0.620311027	0.31	0.107	2.29E-24	CXCR5	1	TCL1A_B cell
124	4.05E-38	0.616748126	0.777	0.796	8.84E-34	RPS20	1	TCL1A_B cell
125	2.44E-08	0.614896806	0.27	0.19	0.00053169	UVRAG	1	TCL1A_B cell
126	3.07E-23	0.614853999	0.189	0.048	6.70E-19	ARRDC2	1	TCL1A_B cell
127	1.04E-12	0.614533724	0.555	0.57	2.27E-08	HLA-DMA	1	TCL1A_B cell
128	5.92E-09	0.612416361	0.334	0.244	0.000129206	ZNF331	1	TCL1A_B cell
129	1.67E-07	0.608710123	0.459	0.487	0.003651846	LYN	1	TCL1A_B cell
130	3.36E-80	0.60505303	0.973	0.94	7.32E-76	RPL9	1	TCL1A_B cell
131	2.94E-72	0.603121783	0.987	0.928	6.40E-68	TMSB4X	1	TCL1A_B cell
132	3.29E-23	0.601386927	0.511	0.321	7.18E-19	BANK1	1	TCL1A_B cell
133	3.26E-09	0.598492807	0.275	0.189	7.11E-05	DCK	1	TCL1A_B cell
134	4.03E-35	0.597732084	0.843	0.921	8.79E-31	IER2	1	TCL1A_B cell
135	1.24E-87	0.593601633	0.988	0.965	2.71E-83	GNB2L1	1	TCL1A_B cell
136	6.90E-14	0.592638031	0.322	0.204	1.51E-09	EVL	1	TCL1A_B cell
137	2.45E-84	0.589065504	0.981	0.967	5.35E-80	RPS9	1	TCL1A_B cell
138	3.03E-89	0.588350658	0.997	0.958	6.60E-85	RPL17	1	TCL1A_B cell
139	3.29E-77	0.587410036	0.965	0.961	7.17E-73	RPS11	1	TCL1A_B cell

140	2.65E-17	0.586642119	0.519	0.41	5.77E-13	IFITM2	1	TCL1A_B cell
141	5.66E-109	0.58594472	1	0.985	1.23E-104	RPL19	1	TCL1A_B cell
142	5.00E-07	0.583341898	0.322	0.269	0.010911229	MAPRE2	1	TCL1A_B cell
143	1.76E-88	0.582288545	1	0.983	3.84E-84	RPS12	1	TCL1A_B cell
144	1.26E-98	0.581764759	1	0.993	2.75E-94	RPL11	1	TCL1A_B cell
145	3.72E-22	0.580820094	0.305	0.131	8.10E-18	LY86	1	TCL1A_B cell
146	7.29E-95	0.578041496	1	0.996	1.59E-90	EEF1A1	1	TCL1A_B cell
147	3.90E-88	0.574680412	1	0.968	8.51E-84	RPS6	1	TCL1A_B cell
148	2.70E-78	0.573799954	0.992	0.974	5.89E-74	TMSB10	1	TCL1A_B cell
149	7.92E-11	0.573195416	0.283	0.186	1.73E-06	RASGRP2	1	TCL1A_B cell
150	7.53E-114	0.572791522	1	0.997	1.64E-109	RPL10	1	TCL1A_B cell
151	1.65E-16	0.572655851	0.221	0.095	3.59E-12	ITPR1	1	TCL1A_B cell
152	1.45E-104	0.572135393	1	0.997	3.17E-100	AC090498.1	1	TCL1A_B cell
153	2.61E-71	0.571875509	0.958	0.958	5.69E-67	UBA52	1	TCL1A_B cell
154	2.32E-36	0.569476601	0.16	0.011	5.07E-32	SATB1	1	TCL1A_B cell
155	2.96E-105	0.56776675	1	0.988	6.45E-101	RPS15	1	TCL1A_B cell
156	3.00E-10	0.566850148	0.229	0.135	6.55E-06	CTB-133G6.1	1	TCL1A_B cell
157	2.03E-61	0.565029852	0.926	0.957	4.43E-57	EEF1D	1	TCL1A_B cell
158	3.61E-104	0.564461271	1	0.991	7.87E-100	RPL28	1	TCL1A_B cell
159	5.02E-94	0.56410226	0.997	0.987	1.10E-89	RPLP2	1	TCL1A_B cell
160	6.63E-69	0.559822445	0.973	0.941	1.45E-64	RPS29	1	TCL1A_B cell
161	6.79E-14	0.553401343	0.221	0.103	1.48E-09	RP11-796E2.4	1	TCL1A_B cell
162	1.94E-39	0.552293385	0.804	0.84	4.24E-35	TOMM7	1	TCL1A_B cell
163	1.90E-51	0.546600705	0.889	0.921	4.14E-47	PABPC1	1	TCL1A_B cell
164	9.59E-20	0.546486169	0.256	0.101	2.09E-15	CCR7	1	TCL1A_B cell
165	1.93E-06	0.544913161	0.196	0.132	0.042120137	SNX29	1	TCL1A_B cell
166	2.59E-09	0.544608643	0.572	0.704	5.65E-05	SF1	1	TCL1A_B cell
167	1.36E-09	0.543945052	0.26	0.167	2.96E-05	UTRN	1	TCL1A_B cell
168	1.28E-17	0.541742731	0.659	0.805	2.79E-13	EIF3E	1	TCL1A_B cell
169	7.84E-15	0.537234126	0.197	0.082	1.71E-10	SSBP2	1	TCL1A_B cell
170	4.50E-16	0.536107262	0.175	0.062	9.81E-12	AC245100.1	1	TCL1A_B cell
171	8.15E-07	0.535919055	0.524	0.678	0.017775215	ANAPC16	1	TCL1A_B cell
172	8.06E-17	0.535355906	0.717	0.774	1.76E-12	NFKBIA	1	TCL1A_B cell
173	8.09E-20	0.532821799	0.648	0.625	1.76E-15	PTPRC	1	TCL1A_B cell
174	8.10E-104	0.53038478	1	0.992	1.77E-99	RPS8	1	TCL1A_B cell
175	4.66E-13	0.526722295	0.302	0.184	1.02E-08	TRBC2	1	TCL1A_B cell
176	3.05E-79	0.525945229	0.992	0.974	6.66E-75	RPL15	1	TCL1A_B cell
177	1.13E-06	0.525519998	0.545	0.701	0.024699493	ATP6V1G1	1	TCL1A_B cell
178	1.74E-93	0.525287047	0.997	0.985	3.79E-89	RPS28	1	TCL1A_B cell
179	4.01E-96	0.52485969	1	0.99	8.75E-92	RPL8	1	TCL1A_B cell
180	4.15E-10	0.523573676	0.391	0.312	9.05E-06	SYPL1	1	TCL1A_B cell
181	6.49E-46	0.519320206	0.892	0.927	1.42E-41	CD79A	1	TCL1A_B cell
182	1.99E-12	0.515784643	0.226	0.115	4.35E-08	PKIG	1	TCL1A_B cell
183	1.48E-76	0.515255699	0.988	0.963	3.23E-72	RPL6	1	TCL1A_B cell
184	1.01E-16	0.514872543	0.196	0.072	2.20E-12	PIK3IP1	1	TCL1A_B cell
185	9.43E-70	0.511628888	0.985	0.973	2.06E-65	RPL37A	1	TCL1A_B cell
186	9.58E-14	0.506781324	0.234	0.111	2.09E-09	VPREB3	1	TCL1A_B cell
187	1.10E-11	0.506374814	0.423	0.325	2.40E-07	RNASET2	1	TCL1A_B cell
188	2.04E-30	0.505457233	0.762	0.855	4.46E-26	PCBP2	1	TCL1A_B cell
189	6.48E-72	0.504518126	0.985	0.967	1.41E-67	RPL5	1	TCL1A_B cell
190	5.24E-87	0.50296992	0.998	0.974	1.14E-82	RPS7	1	TCL1A_B cell
191	8.85E-68	0.502767507	0.975	0.964	1.93E-63	NACA	1	TCL1A_B cell
192	1.97E-72	0.501724151	0.995	0.988	4.29E-68	RPS18	1	TCL1A_B cell
193	1.24E-38	0.501076446	0.865	0.901	2.70E-34	RPL31	1	TCL1A_B cell
194	3.26E-14	0.500342349	0.676	0.823	7.12E-10	UBB	1	TCL1A_B cell
195	2.53E-09	0.499650995	0.351	0.261	5.52E-05	ADAM28	1	TCL1A_B cell
196	7.38E-71	0.497049411	0.992	0.963	1.61E-66	RPL23A	1	TCL1A_B cell
197	7.75E-08	0.496580817	0.58	0.707	0.001689929	FUS	1	TCL1A_B cell
198	2.28E-30	0.49586723	0.874	0.935	4.97E-26	ZFP36	1	TCL1A_B cell
199	7.15E-08	0.495057465	0.184	0.108	0.001559016	PLEKHA2	1	TCL1A_B cell
200	7.82E-73	0.494267926	0.992	0.968	1.71E-68	RPL36	1	TCL1A_B cell
201	1.18E-27	0.493579998	0.147	0.017	2.57E-23	ZBTB16	1	TCL1A_B cell
202	5.63E-81	0.490051489	0.997	0.988	1.23E-76	RPS15A	1	TCL1A_B cell
203	1.30E-06	0.488247342	0.513	0.587	0.0284492	CD53	1	TCL1A_B cell
204	1.03E-28	0.479845909	0.791	0.893	2.24E-24	YWHAZ	1	TCL1A_B cell
205	2.21E-27	0.476812069	0.793	0.917	4.83E-23	DDX5	1	TCL1A_B cell
206	2.10E-07	0.47355938	0.218	0.141	0.004571764	BCL11A	1	TCL1A_B cell
207	6.00E-56	0.473032669	0.965	0.964	1.31E-51	RPL24	1	TCL1A_B cell
208	7.23E-13	0.472267306	0.256	0.133	1.58E-08	PARP15	1	TCL1A_B cell
209	1.50E-23	0.468042012	0.143	0.023	3.28E-19	P11-231C14	1	TCL1A_B cell
210	4.13E-12	0.467991832	0.627	0.712	9.01E-08	ARHGDIB	1	TCL1A_B cell

211	3.71E-08	0.464502739	0.582	0.591	0.000809435	NR4A2	1	TCL1A_B cell
212	2.15E-44	0.46395731	0.914	0.92	4.68E-40	RPL27	1	TCL1A_B cell
213	4.38E-69	0.460994366	0.997	0.973	9.56E-65	RPS14	1	TCL1A_B cell
214	1.01E-12	0.455743137	0.14	0.049	2.20E-08	LAIR1	1	TCL1A_B cell
215	1.48E-20	0.453717978	0.73	0.829	3.22E-16	PTPRCAP	1	TCL1A_B cell
216	7.10E-14	0.451740132	0.241	0.115	1.55E-09	KIAA0226L	1	TCL1A_B cell
217	4.09E-10	0.449910912	0.214	0.113	8.91E-06	TMEM2	1	TCL1A_B cell
218	1.37E-48	0.44469091	0.998	0.989	2.98E-44	PTMA	1	TCL1A_B cell
219	2.63E-55	0.443657913	0.99	0.97	5.75E-51	RPL14	1	TCL1A_B cell
220	2.77E-11	0.440027565	0.272	0.155	6.04E-07	BLK	1	TCL1A_B cell
221	1.97E-63	0.436637226	0.997	0.987	4.29E-59	RPS3	1	TCL1A_B cell
222	4.73E-61	0.434219954	0.998	0.996	1.03E-56	MT-ND3	1	TCL1A_B cell
223	1.20E-59	0.433614772	0.99	0.977	2.62E-55	RPS24	1	TCL1A_B cell
224	8.83E-07	0.431512677	0.17	0.102	0.019249869	GSAP	1	TCL1A_B cell
225	8.35E-23	0.43125597	0.11	0.01	1.82E-18	HS3ST1	1	TCL1A_B cell
226	3.30E-13	0.426949423	0.683	0.837	7.19E-09	CCNI	1	TCL1A_B cell
227	2.60E-48	0.426026019	0.978	0.966	5.66E-44	RPL35	1	TCL1A_B cell
228	3.31E-10	0.423340573	0.142	0.06	7.21E-06	ZNF318	1	TCL1A_B cell
229	3.95E-58	0.42313682	0.995	0.976	8.62E-54	RPS16	1	TCL1A_B cell
230	4.07E-18	0.422899614	0.718	0.833	8.88E-14	RPL23	1	TCL1A_B cell
231	9.29E-59	0.422224939	0.988	0.983	2.03E-54	RPL3	1	TCL1A_B cell
232	5.61E-63	0.420077048	0.993	0.984	1.22E-58	RPS4X	1	TCL1A_B cell
233	3.85E-33	0.417766342	0.906	0.973	8.40E-29	MT-ND2	1	TCL1A_B cell
234	2.07E-13	0.415203521	0.125	0.037	4.52E-09	COL19A1	1	TCL1A_B cell
235	3.27E-10	0.413591228	0.137	0.055	7.14E-06	PTGER4	1	TCL1A_B cell
236	4.55E-07	0.41080932	0.599	0.749	0.009913298	MYL12A	1	TCL1A_B cell
237	8.80E-16	0.409124546	0.118	0.027	1.92E-11	C1orf162	1	TCL1A_B cell
238	3.22E-31	0.406564873	0.912	0.924	7.03E-27	RPL7	1	TCL1A_B cell
239	1.99E-09	0.405795502	0.614	0.786	4.35E-05	HNRNPK	1	TCL1A_B cell
240	5.94E-12	0.403182625	0.13	0.044	1.29E-07	AC241585.2	1	TCL1A_B cell
241	3.94E-07	0.399620293	0.607	0.791	0.008594075	FXYD5	1	TCL1A_B cell
242	2.16E-07	0.399293331	0.6	0.741	0.004708124	CALM2	1	TCL1A_B cell
243	2.79E-10	0.397491265	0.145	0.062	6.09E-06	MGAT5	1	TCL1A_B cell
244	7.39E-11	0.395398603	0.16	0.068	1.61E-06	GAPT	1	TCL1A_B cell
245	2.58E-10	0.391027735	0.72	0.78	5.62E-06	JUN	1	TCL1A_B cell
246	3.08E-08	0.387903291	0.201	0.112	0.000671683	TGIF1	1	TCL1A_B cell
247	6.52E-21	0.385591098	0.899	0.972	1.42E-16	H3F3B	1	TCL1A_B cell
248	1.10E-12	0.384344935	0.268	0.139	2.39E-08	CD24	1	TCL1A_B cell
249	2.66E-12	0.38277617	0.793	0.907	5.81E-08	HSP90AB1	1	TCL1A_B cell
250	3.40E-07	0.381873111	0.159	0.087	0.007410291	ARHGAP17	1	TCL1A_B cell
251	3.41E-07	0.380983217	0.175	0.101	0.007436225	PAX5	1	TCL1A_B cell
252	4.85E-31	0.379256836	0.931	0.961	1.06E-26	RPL41	1	TCL1A_B cell
253	1.17E-09	0.377857777	0.196	0.1	2.54E-05	SPIB	1	TCL1A_B cell
254	1.11E-26	0.371166366	0.852	0.914	2.42E-22	COX4I1	1	TCL1A_B cell
255	7.17E-09	0.36995985	0.123	0.051	0.000156409	ARAP2	1	TCL1A_B cell
256	4.22E-07	0.369288714	0.111	0.051	0.009202666	SESTD1	1	TCL1A_B cell
257	6.13E-33	0.369069874	0.943	0.989	1.34E-28	MT-ND4	1	TCL1A_B cell
258	2.86E-21	0.362325736	0.798	0.874	6.23E-17	BTF3	1	TCL1A_B cell
259	1.78E-10	0.360374198	0.142	0.057	3.88E-06	HHEX	1	TCL1A_B cell
260	1.98E-15	0.3588783	0.771	0.88	4.31E-11	HNRNPA1	1	TCL1A_B cell
261	9.78E-08	0.3567027	0.634	0.796	0.002131826	SH3BGRL3	1	TCL1A_B cell
262	1.52E-16	0.356068425	0.759	0.905	3.32E-12	HLA-E	1	TCL1A_B cell
263	3.16E-11	0.355565704	0.108	0.032	6.89E-07	NT5E	1	TCL1A_B cell
264	6.81E-07	0.355218333	0.728	0.879	0.014850896	HSP90AA1	1	TCL1A_B cell
265	9.75E-11	0.354664238	0.138	0.053	2.13E-06	ABLIM1	1	TCL1A_B cell
266	1.21E-07	0.354503107	0.135	0.066	0.002640474	HLA-DOA	1	TCL1A_B cell
267	4.18E-10	0.351460908	0.126	0.048	9.12E-06	P11-624C23	1	TCL1A_B cell
268	8.05E-11	0.343245306	0.681	0.836	1.76E-06	ARPC3	1	TCL1A_B cell
269	7.32E-10	0.342286498	0.693	0.812	1.60E-05	RPS17	1	TCL1A_B cell
270	1.62E-07	0.342213742	0.15	0.076	0.00352641	TRAF5	1	TCL1A_B cell
271	2.42E-07	0.341601019	0.128	0.062	0.005278048	JAZF1	1	TCL1A_B cell
272	2.10E-13	0.341516725	0.126	0.037	4.57E-09	IL13RA1	1	TCL1A_B cell
273	3.41E-34	0.339371569	0.966	0.966	7.43E-30	EEF1B2	1	TCL1A_B cell
274	4.03E-09	0.334241759	0.668	0.857	8.79E-05	HNRNPDL	1	TCL1A_B cell
275	2.55E-23	0.334193901	0.904	0.951	5.55E-19	RPL4	1	TCL1A_B cell
276	1.77E-21	0.333433459	0.87	0.915	3.86E-17	EEF1G	1	TCL1A_B cell
277	3.61E-15	0.331034131	0.799	0.895	7.86E-11	RPL36A	1	TCL1A_B cell
278	6.22E-08	0.325232781	0.103	0.041	0.001355925	C12orf42	1	TCL1A_B cell
279	2.34E-09	0.325158398	0.126	0.051	5.11E-05	LGALS9	1	TCL1A_B cell
280	1.75E-07	0.321515955	0.18	0.099	0.003814677	ZEB2	1	TCL1A_B cell
281	1.08E-44	0.320784229	1	0.997	2.36E-40	RPLP1	1	TCL1A_B cell

282	4.17E-09	0.314348442	0.115	0.043	9.10E-05	GPR18	1	TCL1A_B cell
283	8.00E-09	0.308183377	0.132	0.057	0.000174475	LYSMD2	1	TCL1A_B cell
284	7.49E-13	0.304015495	0.781	0.892	1.63E-08	RPL27A	1	TCL1A_B cell
285	1.35E-12	0.303307602	0.774	0.882	2.95E-08	HINT1	1	TCL1A_B cell
286	3.59E-07	0.30290112	0.174	0.348	0.007838441	RNF13	1	TCL1A_B cell
287	6.04E-15	0.301626112	0.848	0.928	1.32E-10	OAZ1	1	TCL1A_B cell
288	2.71E-43	0.297203759	1	1	5.91E-39	MALAT1	1	TCL1A_B cell
289	9.39E-07	0.285841496	0.175	0.344	0.020486462	CD46	1	TCL1A_B cell
290	4.50E-24	0.278048412	0.953	0.991	9.82E-20	MT-ND1	1	TCL1A_B cell
291	1.30E-06	0.277683435	0.243	0.459	0.028378536	AMD1	1	TCL1A_B cell
292	8.81E-09	0.273838451	0.206	0.427	0.000192201	CNOT7	1	TCL1A_B cell
293	1.41E-09	0.271862	0.757	0.881	3.07E-05	COX7C	1	TCL1A_B cell
294	4.03E-27	0.271851421	0.995	0.988	8.78E-23	RPL7A	1	TCL1A_B cell
295	2.27E-07	0.269806314	0.15	0.308	0.004958925	AP3B1	1	TCL1A_B cell
296	1.72E-06	0.268171193	0.182	0.352	0.037511611	RNASEH2B	1	TCL1A_B cell
297	9.54E-07	0.263861881	0.179	0.349	0.02080277	CASP4	1	TCL1A_B cell
298	2.01E-07	0.263395514	0.209	0.412	0.004386714	HSPA4	1	TCL1A_B cell
299	1.38E-09	0.256408568	0.199	0.426	3.00E-05	U2SURP	1	TCL1A_B cell
300	1.15E-06	0.255973561	0.209	0.4	0.025038891	RAB11A	1	TCL1A_B cell
301	7.83E-07	0.254579775	0.125	0.258	0.01706678	NSL1	1	TCL1A_B cell
302	6.81E-07	0.253102627	0.167	0.329	0.014845458	CGGBP1	1	TCL1A_B cell
303	5.18E-08	0.252664564	0.201	0.408	0.001129778	BTK	1	TCL1A_B cell
304	4.51E-08	0.252094394	0.143	0.304	0.000984021	TMEM134	1	TCL1A_B cell
305	2.01E-19	0.251760962	0.976	0.973	4.39E-15	RPS2	1	TCL1A_B cell
306	1.18E-06	0.251716974	0.216	0.404	0.025795888	DNAJB6	1	TCL1A_B cell
307	1.19E-07	0.250267804	0.16	0.327	0.002585892	NIPBL	1	TCL1A_B cell
308	3.52E-20	-0.250257254	0.388	0.772	7.68E-16	COX6C	1	TCL1A_B cell
309	6.84E-36	-0.250932722	0.118	0.47	1.49E-31	S100A10	1	TCL1A_B cell
310	6.69E-30	-0.252711775	0.272	0.707	1.46E-25	ZNF706	1	TCL1A_B cell
311	8.66E-23	-0.2527966	0.346	0.768	1.89E-18	NDUFB11	1	TCL1A_B cell
312	4.07E-46	-0.253189041	0.147	0.596	8.87E-42	TOP1	1	TCL1A_B cell
313	1.31E-45	-0.253198973	0.118	0.536	2.85E-41	CYC1	1	TCL1A_B cell
314	5.06E-33	-0.25433657	0.256	0.702	1.10E-28	FAM46C	1	TCL1A_B cell
315	8.92E-55	-0.254464986	0.125	0.601	1.95E-50	TMED5	1	TCL1A_B cell
316	2.44E-26	-0.255960806	0.305	0.74	5.33E-22	TCEB2	1	TCL1A_B cell
317	7.89E-30	-0.258404481	0.253	0.676	1.72E-25	ATP5J2	1	TCL1A_B cell
318	1.50E-40	-0.259726086	0.174	0.614	3.27E-36	PSMD8	1	TCL1A_B cell
319	4.70E-25	-0.263852587	0.346	0.766	1.03E-20	LSP1	1	TCL1A_B cell
320	3.92E-49	-0.264046299	0.121	0.564	8.55E-45	MRPL51	1	TCL1A_B cell
321	4.94E-36	-0.266949232	0.998	0.998	1.08E-31	B2M	1	TCL1A_B cell
322	2.98E-31	-0.270060514	0.258	0.691	6.49E-27	USMG5	1	TCL1A_B cell
323	2.49E-48	-0.270555606	0.11	0.53	5.44E-44	PSMA3	1	TCL1A_B cell
324	1.63E-53	-0.273182096	0.132	0.608	3.55E-49	RPS19BP1	1	TCL1A_B cell
325	2.09E-44	-0.27466596	0.169	0.628	4.55E-40	ATP5C1	1	TCL1A_B cell
326	9.43E-42	-0.27882241	0.177	0.619	2.06E-37	CDK2AP2	1	TCL1A_B cell
327	1.29E-52	-0.279985672	0.101	0.54	2.82E-48	PSMA5	1	TCL1A_B cell
328	1.12E-53	-0.280321225	0.106	0.557	2.44E-49	NDUFAF3	1	TCL1A_B cell
329	6.01E-54	-0.28277526	0.13	0.607	1.31E-49	MRPS34	1	TCL1A_B cell
330	1.25E-33	-0.286600384	0.219	0.628	2.72E-29	ENO1	1	TCL1A_B cell
331	5.28E-24	-0.290033302	0.384	0.813	1.15E-19	MT-ND6	1	TCL1A_B cell
332	2.11E-47	-0.294570917	0.14	0.589	4.60E-43	SRSF9	1	TCL1A_B cell
333	5.42E-40	-0.295077405	0.216	0.692	1.18E-35	GUK1	1	TCL1A_B cell
334	4.64E-35	-0.295540293	0.224	0.651	1.01E-30	ERH	1	TCL1A_B cell
335	1.31E-35	-0.298094205	0.258	0.715	2.85E-31	POLR2L	1	TCL1A_B cell
336	4.60E-28	-0.29818925	0.312	0.727	1.00E-23	TPI1	1	TCL1A_B cell
337	7.49E-38	-0.30032745	0.207	0.647	1.63E-33	NDUFV2	1	TCL1A_B cell
338	5.27E-34	-0.301259527	0.258	0.71	1.15E-29	CHCHD10	1	TCL1A_B cell
339	1.01E-17	-0.305952852	0.895	0.973	2.20E-13	HLA-A	1	TCL1A_B cell
340	1.29E-33	-0.310641967	0.266	0.727	2.81E-29	TMEM59	1	TCL1A_B cell
341	3.25E-59	-0.311271732	0.116	0.607	7.09E-55	NDUFA11	1	TCL1A_B cell
342	1.05E-39	-0.316029321	0.212	0.675	2.30E-35	PRDX5	1	TCL1A_B cell
343	3.30E-60	-0.319555527	0.108	0.594	7.21E-56	SDF4	1	TCL1A_B cell
344	1.23E-45	-0.322311579	0.185	0.662	2.68E-41	NDUFB4	1	TCL1A_B cell
345	2.88E-39	-0.326297431	0.224	0.684	6.28E-35	GNG5	1	TCL1A_B cell
346	4.80E-58	-0.327936077	0.116	0.598	1.05E-53	BSG	1	TCL1A_B cell
347	1.46E-58	-0.3291519	0.103	0.575	3.18E-54	NDUFAB1	1	TCL1A_B cell
348	2.45E-47	-0.329530874	0.165	0.628	5.34E-43	PSMB3	1	TCL1A_B cell
349	1.23E-23	-0.330345165	0.371	0.749	2.69E-19	RAN	1	TCL1A_B cell
350	6.61E-28	-0.334699089	0.425	0.817	1.44E-23	SSR2	1	TCL1A_B cell
351	1.03E-21	-0.335055282	0.511	0.829	2.25E-17	UQCRH	1	TCL1A_B cell
352	2.72E-12	-0.338979733	0.617	0.894	5.92E-08	SAT1	1	TCL1A_B cell

353	5.46E-53	-0.340835405	0.143	0.621	1.19E-48	TM9SF2	1	TCL1A_B cell
354	1.72E-37	-0.345022748	0.266	0.746	3.74E-33	NDUFA1	1	TCL1A_B cell
355	1.97E-33	-0.345197736	0.322	0.775	4.30E-29	PRDX1	1	TCL1A_B cell
356	1.27E-54	-0.345474414	0.157	0.66	2.77E-50	SELT	1	TCL1A_B cell
357	6.46E-27	-0.351068616	0.452	0.82	1.41E-22	OST4	1	TCL1A_B cell
358	4.72E-57	-0.353560799	0.106	0.572	1.03E-52	MRPS24	1	TCL1A_B cell
359	3.65E-34	-0.356752961	0.285	0.732	7.96E-30	PDIA3	1	TCL1A_B cell
360	2.10E-58	-0.36499705	0.108	0.579	4.58E-54	NDUFS6	1	TCL1A_B cell
361	7.90E-60	-0.370710651	0.111	0.592	1.72E-55	ARPC5L	1	TCL1A_B cell
362	6.57E-46	-0.374441565	0.204	0.674	1.43E-41	COX7B	1	TCL1A_B cell
363	4.20E-56	-0.375465952	0.116	0.579	9.16E-52	PPA1	1	TCL1A_B cell
364	1.14E-42	-0.375497657	0.28	0.78	2.49E-38	MCL1	1	TCL1A_B cell
365	9.74E-54	-0.376849547	0.126	0.589	2.13E-49	NDUFB6	1	TCL1A_B cell
366	2.22E-10	-0.382392935	0.708	0.908	4.85E-06	ACTG1	1	TCL1A_B cell
367	4.10E-31	-0.383241499	0.4	0.792	8.95E-27	COX6B1	1	TCL1A_B cell
368	1.04E-50	-0.384794537	0.174	0.66	2.27E-46	NDUFS5	1	TCL1A_B cell
369	1.75E-47	-0.388578152	0.197	0.677	3.81E-43	PEBP1	1	TCL1A_B cell
370	4.45E-47	-0.388990015	0.199	0.682	9.71E-43	ROMO1	1	TCL1A_B cell
371	2.95E-36	-0.392379385	0.214	0.625	6.43E-32	GSTP1	1	TCL1A_B cell
372	7.35E-33	-0.394372398	0.464	0.836	1.60E-28	RPL36AL	1	TCL1A_B cell
373	2.63E-63	-0.399111126	0.105	0.597	5.73E-59	SND1	1	TCL1A_B cell
374	3.02E-42	-0.400396559	0.245	0.731	6.58E-38	CD99	1	TCL1A_B cell
375	8.35E-56	-0.401278766	0.115	0.579	1.82E-51	ANAPC11	1	TCL1A_B cell
376	2.48E-33	-0.402294711	0.366	0.779	5.40E-29	COX7A2	1	TCL1A_B cell
377	9.78E-63	-0.402434738	0.106	0.598	2.13E-58	AURKAIP1	1	TCL1A_B cell
378	3.49E-39	-0.407131217	0.273	0.743	7.61E-35	KRTCAP2	1	TCL1A_B cell
379	2.90E-55	-0.412536142	0.143	0.629	6.32E-51	PSMB6	1	TCL1A_B cell
380	2.57E-52	-0.417106637	0.148	0.618	5.60E-48	ATP5G1	1	TCL1A_B cell
381	2.46E-38	-0.417263908	0.297	0.747	5.37E-34	NDUFA4	1	TCL1A_B cell
382	1.00E-32	-0.417947739	0.417	0.821	2.19E-28	COX6A1	1	TCL1A_B cell
383	1.90E-40	-0.418579737	0.243	0.692	4.15E-36	ATP5A1	1	TCL1A_B cell
384	3.27E-55	-0.420776486	0.113	0.564	7.13E-51	H2AFV	1	TCL1A_B cell
385	7.98E-25	-0.420864695	0.659	0.884	1.74E-20	RPS26	1	TCL1A_B cell
386	5.88E-55	-0.42750285	0.17	0.678	1.28E-50	ERGIC3	1	TCL1A_B cell
387	1.01E-50	-0.439541657	0.838	0.979	2.20E-46	HLA-C	1	TCL1A_B cell
388	2.37E-39	-0.439633685	0.263	0.699	5.16E-35	PIM2	1	TCL1A_B cell
389	3.42E-34	-0.44466512	0.309	0.727	7.47E-30	LDHA	1	TCL1A_B cell
390	2.65E-44	-0.446979484	0.248	0.718	5.77E-40	LDHB	1	TCL1A_B cell
391	5.55E-51	-0.463282536	0.207	0.684	1.21E-46	COX8A	1	TCL1A_B cell
392	9.93E-65	-0.463730887	0.103	0.598	2.17E-60	SEC13	1	TCL1A_B cell
393	1.27E-51	-0.468973179	0.212	0.717	2.78E-47	ARF4	1	TCL1A_B cell
394	3.51E-46	-0.473800575	0.245	0.724	7.65E-42	SELK	1	TCL1A_B cell
395	4.88E-42	-0.477880892	0.376	0.807	1.06E-37	TMBIM6	1	TCL1A_B cell
396	1.54E-50	-0.47962129	0.216	0.694	3.36E-46	ATP5J	1	TCL1A_B cell
397	2.10E-63	-0.511355124	0.132	0.638	4.58E-59	DERL1	1	TCL1A_B cell
398	5.96E-70	-0.519662521	0.126	0.658	1.30E-65	SRPRA	1	TCL1A_B cell
399	7.97E-62	-0.522473026	0.206	0.757	1.74E-57	PSAP	1	TCL1A_B cell
400	2.49E-72	-0.524185496	0.101	0.626	5.43E-68	ELL2	1	TCL1A_B cell
401	2.92E-65	-0.525217455	0.123	0.632	6.36E-61	PRDX2	1	TCL1A_B cell
402	1.34E-66	-0.534643334	0.138	0.659	2.92E-62	PRELID1	1	TCL1A_B cell
403	5.38E-62	-0.536983449	0.167	0.675	1.17E-57	PKM	1	TCL1A_B cell
404	2.45E-50	-0.542177263	0.265	0.747	5.33E-46	POU2AF1	1	TCL1A_B cell
405	9.59E-55	-0.546117029	0.239	0.739	2.09E-50	DAD1	1	TCL1A_B cell
406	2.23E-59	-0.550487463	0.172	0.667	4.85E-55	COX5A	1	TCL1A_B cell
407	4.08E-37	-0.554341553	0.486	0.825	8.89E-33	CHCHD2	1	TCL1A_B cell
408	1.39E-59	-0.557951741	0.164	0.663	3.04E-55	CANX	1	TCL1A_B cell
409	7.97E-49	-0.566977703	0.373	0.803	1.74E-44	NME2	1	TCL1A_B cell
410	1.56E-58	-0.576094499	0.187	0.688	3.40E-54	TMED2	1	TCL1A_B cell
411	6.05E-71	-0.583125566	0.125	0.648	1.32E-66	PGAM1	1	TCL1A_B cell
412	1.72E-67	-0.585916045	0.143	0.666	3.76E-63	SSR1	1	TCL1A_B cell
413	2.28E-56	-0.596275719	0.214	0.702	4.97E-52	TRAM1	1	TCL1A_B cell
414	2.87E-54	-0.600612354	0.285	0.752	6.27E-50	RABAC1	1	TCL1A_B cell
415	1.00E-84	-0.605663448	0.103	0.68	2.19E-80	VOPP1	1	TCL1A_B cell
416	5.51E-46	-0.614805157	0.504	0.855	1.20E-41	MIF	1	TCL1A_B cell
417	1.19E-59	-0.64809184	0.233	0.725	2.59E-55	ATP5G3	1	TCL1A_B cell
418	3.66E-55	-0.653662852	0.297	0.777	7.99E-51	MTDH	1	TCL1A_B cell
419	2.59E-66	-0.655576053	0.202	0.728	5.65E-62	COPE	1	TCL1A_B cell
420	1.55E-67	-0.661188854	0.167	0.686	3.37E-63	UQCRQ	1	TCL1A_B cell
421	1.07E-69	-0.675390751	0.172	0.707	2.34E-65	TMED10	1	TCL1A_B cell
422	2.11E-75	-0.680014491	0.135	0.679	4.60E-71	KDELR1	1	TCL1A_B cell
423	8.18E-66	-0.694549657	0.162	0.66	1.78E-61	PSME2	1	TCL1A_B cell

424	1.56E-64	-0.698592856	0.283	0.776	3.41E-60	TMEM258	1	TCL1A_B cell
425	4.70E-77	-0.701099078	0.327	0.869	1.02E-72	SRGN	1	TCL1A_B cell
426	3.39E-68	-0.727050681	0.241	0.764	7.40E-64	SPCS1	1	TCL1A_B cell
427	2.97E-79	-0.729804062	0.126	0.679	6.48E-75	TMED9	1	TCL1A_B cell
428	6.61E-55	-0.732189817	0.543	0.895	1.44E-50	VIM	1	TCL1A_B cell
429	2.07E-78	-0.75973197	0.133	0.674	4.51E-74	DDOST	1	TCL1A_B cell
430	1.49E-72	-0.78365523	0.302	0.778	3.24E-68	SPCS2	1	TCL1A_B cell
431	4.11E-88	-0.792296736	0.108	0.68	8.96E-84	LMAN2	1	TCL1A_B cell
432	2.64E-76	-0.792483577	0.152	0.679	5.75E-72	OSTC	1	TCL1A_B cell
433	2.65E-32	-0.818984758	0.212	0.559	5.78E-28	HIST1H4C	1	TCL1A_B cell
434	2.81E-66	-0.84624481	0.344	0.808	6.14E-62	CALR	1	TCL1A_B cell
435	9.45E-35	-0.850379475	0.194	0.539	2.06E-30	CRIP1	1	TCL1A_B cell
436	3.53E-89	-0.909624853	0.126	0.683	7.70E-85	RPN1	1	TCL1A_B cell
437	5.83E-76	-0.963742561	0.283	0.87	1.27E-71	NEAT1	1	TCL1A_B cell
438	2.24E-80	-1.014667261	0.27	0.741	4.88E-76	SEC61G	1	TCL1A_B cell
439	1.18E-88	-1.023274993	0.196	0.736	2.58E-84	UBE2J1	1	TCL1A_B cell
440	8.37E-93	-1.029429778	0.116	0.672	1.83E-88	KDELR2	1	TCL1A_B cell
441	2.30E-95	-1.040217689	0.121	0.684	5.01E-91	RPN2	1	TCL1A_B cell
442	4.48E-81	-1.132019534	0.46	0.85	9.77E-77	SUB1	1	TCL1A_B cell
443	3.05E-67	-1.136309282	0.13	0.588	6.64E-63	IGLL5	1	TCL1A_B cell
444	4.07E-101	-1.162616253	0.153	0.724	8.88E-97	PDIA6	1	TCL1A_B cell
445	2.29E-103	-1.175906209	0.256	0.786	5.00E-99	SEC61B	1	TCL1A_B cell
446	6.80E-107	-1.235148425	0.118	0.703	1.48E-102	P4HB	1	TCL1A_B cell
447	9.35E-109	-1.277682327	0.128	0.706	2.04E-104	ITM2C	1	TCL1A_B cell
448	2.11E-93	-1.283020254	0.282	0.78	4.60E-89	HSPA5	1	TCL1A_B cell
449	1.70E-99	-1.409247395	0.57	0.933	3.70E-95	GAPDH	1	TCL1A_B cell
450	4.89E-116	-1.438036598	0.11	0.698	1.07E-111	SSR3	1	TCL1A_B cell
451	9.64E-117	-1.579085134	0.346	0.813	2.10E-112	SSR4	1	TCL1A_B cell
452	4.11E-128	-2.1082817	0.307	0.82	8.96E-124	PPIB	1	TCL1A_B cell
453	2.35E-131	-2.244340179	0.331	0.835	5.12E-127	HSP90B1	1	TCL1A_B cell
454	8.60E-132	-2.39742786	0.125	0.715	1.88E-127	MZB1	1	TCL1A_B cell
455	6.11E-76	-3.141316134	0.324	0.699	1.33E-71	IGKC	1	TCL1A_B cell
456	3.43E-10	-3.444368705	0.248	0.414	7.48E-06	IGHV3-23	1	TCL1A_B cell
457	3.36E-129	-3.603727389	0.297	0.796	7.33E-125	JCHAIN	1	TCL1A_B cell
458	7.50E-07	-3.619191278	0.159	0.279	0.016356591	IGKV2-30	1	TCL1A_B cell
459	3.56E-15	-3.754724779	0.263	0.511	7.77E-11	IGKV4-1	1	TCL1A_B cell
460	2.28E-09	-3.902188725	0.103	0.229	4.97E-05	IGKV2-24	1	TCL1A_B cell
461	4.91E-15	-4.38886905	0.133	0.316	1.07E-10	IGHV3-74	1	TCL1A_B cell
462	1.46E-11	-4.422941182	0.155	0.329	3.17E-07	IGKV1D-16	1	TCL1A_B cell
463	2.30E-20	3.032105972	0.438	0.211	5.02E-16	IGKV1D-16	2	IGKV1D-16_plasma cell
464	1.05E-08	3.016093115	0.121	0.046	0.000229236	IGKV3D-20	2	IGKV1D-16_plasma cell
465	2.37E-11	2.852721186	0.208	0.093	5.17E-07	IGKV1-16	2	IGKV1D-16_plasma cell
466	7.03E-10	2.272105431	0.106	0.033	1.53E-05	IGHV3-72	2	IGKV1D-16_plasma cell
467	1.47E-06	2.126379	0.152	0.078	0.032164325	IGKV2D-28	2	IGKV1D-16_plasma cell
468	6.68E-08	2.12120103	0.248	0.134	0.001456238	IGLV2-23	2	IGKV1D-16_plasma cell
469	2.23E-06	2.11467285	0.525	0.472	0.048705575	IGHM	2	IGKV1D-16_plasma cell
470	2.27E-23	2.086959569	0.425	0.194	4.95E-19	IGHV3-74	2	IGKV1D-16_plasma cell
471	2.03E-20	2.055296114	0.527	0.298	4.42E-16	IGHV3-23	2	IGKV1D-16_plasma cell
472	1.86E-12	1.941416715	0.448	0.249	4.06E-08	IGKV1-5	2	IGKV1D-16_plasma cell
473	2.19E-08	1.90728478	0.275	0.15	0.000476815	IGHV3-7	2	IGKV1D-16_plasma cell
474	4.40E-34	1.776444211	0.504	0.204	9.60E-30	IGKV1-12	2	IGKV1D-16_plasma cell
475	3.97E-170	1.768261265	0.992	0.5	8.66E-166	JCHAIN	2	IGKV1D-16_plasma cell
476	1.47E-07	1.681521479	0.329	0.207	0.003206016	IGKV2-30	2	IGKV1D-16_plasma cell
477	6.39E-35	1.656739128	0.452	0.192	1.39E-30	IGHG2	2	IGKV1D-16_plasma cell
478	2.16E-11	1.575845656	0.146	0.052	4.70E-07	IGKV2D-30	2	IGKV1D-16_plasma cell
479	7.58E-13	1.542495593	0.298	0.147	1.65E-08	IGKV2-24	2	IGKV1D-16_plasma cell
480	7.53E-78	1.505113791	0.806	0.491	1.64E-73	IGKC	2	IGKV1D-16_plasma cell
481	1.06E-22	1.487931347	0.508	0.251	2.31E-18	IGHA1	2	IGKV1D-16_plasma cell
482	9.86E-16	1.48487467	0.608	0.364	2.15E-11	IGKV4-1	2	IGKV1D-16_plasma cell
483	2.92E-175	1.443693835	0.996	0.347	6.38E-171	MZB1	2	IGKV1D-16_plasma cell
484	2.53E-14	1.357954831	0.2	0.073	5.51E-10	IGHA2	2	IGKV1D-16_plasma cell
485	5.61E-09	1.292585612	0.158	0.069	0.000122417	IGKV2-28	2	IGKV1D-16_plasma cell
486	7.36E-127	1.203173108	0.992	0.55	1.61E-122	HSP90B1	2	IGKV1D-16_plasma cell
487	1.39E-41	1.193414508	0.612	0.279	3.02E-37	IGHG1	2	IGKV1D-16_plasma cell
488	2.06E-151	1.14566975	0.977	0.543	4.49E-147	SSR4	2	IGKV1D-16_plasma cell
489	1.31E-06	1.145009227	0.14	0.065	0.028461757	IGKV2-29	2	IGKV1D-16_plasma cell
490	5.34E-145	1.142869772	0.954	0.285	1.17E-140	FKBP11	2	IGKV1D-16_plasma cell
491	1.49E-06	1.138965101	0.21	0.118	0.032537569	IGHV6-1	2	IGKV1D-16_plasma cell
492	2.41E-11	1.10785252	0.138	0.046	5.26E-07	IGKV1-6	2	IGKV1D-16_plasma cell
493	2.45E-09	1.09638899	0.177	0.077	5.34E-05	IGKV1-27	2	IGKV1D-16_plasma cell
494	5.23E-119	1.093582551	0.992	0.526	1.14E-114	PPIB	2	IGKV1D-16_plasma cell

495	3.38E-130	1.084643795	0.975	0.33	7.38E-126	XBP1	2	IGKV1D-16_plasma cell
496	1.55E-120	1.019138796	0.954	0.305	3.38E-116	MYDGF	2	IGKV1D-16_plasma cell
497	1.25E-136	1.006136044	0.952	0.286	2.72E-132	DERL3	2	IGKV1D-16_plasma cell
498	4.26E-09	0.990768325	0.225	0.113	9.30E-05	IGHG3	2	IGKV1D-16_plasma cell
499	5.49E-125	0.970410262	0.946	0.286	1.20E-120	PDIA4	2	IGKV1D-16_plasma cell
500	3.24E-95	0.967162176	0.971	0.486	7.06E-91	HSPA5	2	IGKV1D-16_plasma cell
501	4.43E-09	0.960862198	0.244	0.125	9.65E-05	IGLV6-57	2	IGKV1D-16_plasma cell
502	2.54E-127	0.957590813	0.956	0.324	5.55E-123	LMAN1	2	IGKV1D-16_plasma cell
503	1.86E-121	0.952283264	0.917	0.283	4.05E-117	PRDX4	2	IGKV1D-16_plasma cell
504	1.77E-110	0.94179126	0.931	0.363	3.85E-106	ITM2C	2	IGKV1D-16_plasma cell
505	1.06E-102	0.917324011	0.946	0.361	2.31E-98	SEC11C	2	IGKV1D-16_plasma cell
506	2.63E-118	0.886668683	0.908	0.289	5.73E-114	CD38	2	IGKV1D-16_plasma cell
507	1.41E-45	0.884300665	0.958	0.574	3.07E-41	NEAT1	2	IGKV1D-16_plasma cell
508	3.56E-116	0.863597759	0.929	0.321	7.76E-112	HM13	2	IGKV1D-16_plasma cell
509	3.30E-24	0.856883435	0.304	0.109	7.19E-20	IGHG4	2	IGKV1D-16_plasma cell
510	5.89E-102	0.85572413	0.825	0.255	1.28E-97	RRBP1	2	IGKV1D-16_plasma cell
511	2.31E-98	0.840920232	0.856	0.257	5.04E-94	TXNDC5	2	IGKV1D-16_plasma cell
512	3.42E-102	0.835328893	0.946	0.343	7.46E-98	SSR3	2	IGKV1D-16_plasma cell
513	3.84E-96	0.797484316	0.917	0.324	8.38E-92	SPCS3	2	IGKV1D-16_plasma cell
514	3.86E-97	0.782263772	0.854	0.313	8.41E-93	CD27	2	IGKV1D-16_plasma cell
515	1.63E-86	0.781670657	0.894	0.354	3.55E-82	RPN2	2	IGKV1D-16_plasma cell
516	8.86E-87	0.768237371	0.848	0.286	1.93E-82	IGLL5	2	IGKV1D-16_plasma cell
517	5.41E-92	0.757885502	0.817	0.253	1.18E-87	TNFRSF17	2	IGKV1D-16_plasma cell
518	3.02E-95	0.726094592	0.879	0.287	6.58E-91	HDLBP	2	IGKV1D-16_plasma cell
519	2.79E-93	0.718881829	0.879	0.311	6.08E-89	ERLEC1	2	IGKV1D-16_plasma cell
520	4.22E-96	0.715164477	0.902	0.317	9.21E-92	FKBP2	2	IGKV1D-16_plasma cell
521	2.11E-80	0.698349895	0.935	0.355	4.61E-76	P4HB	2	IGKV1D-16_plasma cell
522	3.67E-98	0.690356503	0.9	0.295	8.00E-94	VIMP	2	IGKV1D-16_plasma cell
523	1.51E-70	0.688304966	0.933	0.42	3.29E-66	UBE2J1	2	IGKV1D-16_plasma cell
524	1.35E-95	0.685205747	0.715	0.2	2.94E-91	ANKRD28	2	IGKV1D-16_plasma cell
525	6.99E-116	0.684690404	0.744	0.197	1.52E-111	FNDC3B	2	IGKV1D-16_plasma cell
526	4.53E-89	0.672531168	0.906	0.285	9.88E-85	SDF2L1	2	IGKV1D-16_plasma cell
527	2.14E-75	0.659068562	0.9	0.353	4.66E-71	RPN1	2	IGKV1D-16_plasma cell
528	4.86E-65	0.651240584	0.912	0.398	1.06E-60	PDIA6	2	IGKV1D-16_plasma cell
529	1.93E-72	0.631046102	0.881	0.355	4.22E-68	DDOST	2	IGKV1D-16_plasma cell
530	1.30E-92	0.624041614	0.756	0.238	2.84E-88	NUCB2	2	IGKV1D-16_plasma cell
531	6.42E-97	0.619989703	0.854	0.272	1.40E-92	TXNDC11	2	IGKV1D-16_plasma cell
532	6.83E-77	0.612537238	0.904	0.34	1.49E-72	LMAN2	2	IGKV1D-16_plasma cell
533	1.83E-90	0.598036385	0.733	0.221	3.99E-86	PRDM1	2	IGKV1D-16_plasma cell
534	1.64E-67	0.590335845	0.89	0.342	3.57E-63	KDELR2	2	IGKV1D-16_plasma cell
535	1.33E-72	0.590209884	0.698	0.239	2.89E-68	LINC00152	2	IGKV1D-16_plasma cell
536	3.76E-58	0.571277081	0.938	0.474	8.21E-54	RABAC1	2	IGKV1D-16_plasma cell
537	5.27E-54	0.552859996	0.94	0.492	1.15E-49	SEC61B	2	IGKV1D-16_plasma cell
538	6.18E-78	0.537671384	0.84	0.28	1.35E-73	SEC61A1	2	IGKV1D-16_plasma cell
539	9.82E-51	0.532827109	0.558	0.201	2.14E-46	SLC7A5	2	IGKV1D-16_plasma cell
540	7.72E-60	0.52700367	0.894	0.352	1.68E-55	TMED9	2	IGKV1D-16_plasma cell
541	3.02E-51	0.524104762	0.95	0.501	6.58E-47	SPCS2	2	IGKV1D-16_plasma cell
542	1.95E-74	0.515732455	0.652	0.205	4.25E-70	ZBP1	2	IGKV1D-16_plasma cell
543	2.31E-78	0.515008271	0.64	0.182	5.04E-74	TRIB1	2	IGKV1D-16_plasma cell
544	8.68E-27	0.510032958	0.419	0.173	1.89E-22	LINC01480	2	IGKV1D-16_plasma cell
545	6.50E-50	0.508210075	0.919	0.464	1.42E-45	SEC61G	2	IGKV1D-16_plasma cell
546	4.14E-79	0.505457348	0.64	0.185	9.03E-75	CHPF	2	IGKV1D-16_plasma cell
547	4.44E-54	0.503367792	0.879	0.369	9.68E-50	OSTC	2	IGKV1D-16_plasma cell
548	2.23E-48	0.492018887	0.888	0.4	4.86E-44	TMED10	2	IGKV1D-16_plasma cell
549	1.53E-61	0.489408684	0.821	0.318	3.34E-57	ELL2	2	IGKV1D-16_plasma cell
550	2.64E-48	0.482356684	0.89	0.414	5.76E-44	TRAM1	2	IGKV1D-16_plasma cell
551	6.01E-62	0.4793188	0.862	0.306	1.31E-57	MANF	2	IGKV1D-16_plasma cell
552	2.91E-44	0.471198867	0.921	0.571	6.35E-40	TMBIM6	2	IGKV1D-16_plasma cell
553	4.17E-78	0.469468634	0.771	0.251	9.09E-74	SRPRB	2	IGKV1D-16_plasma cell
554	3.40E-70	0.467422443	0.729	0.244	7.41E-66	CKAP4	2	IGKV1D-16_plasma cell
555	2.78E-53	0.466563234	0.91	0.426	6.07E-49	FAM46C	2	IGKV1D-16_plasma cell
556	8.52E-55	0.464804293	0.923	0.448	1.86E-50	PSAP	2	IGKV1D-16_plasma cell
557	8.30E-63	0.463933244	0.648	0.218	1.81E-58	HYOU1	2	IGKV1D-16_plasma cell
558	1.56E-36	0.459725994	0.852	0.447	3.39E-32	PIM2	2	IGKV1D-16_plasma cell
559	1.85E-77	0.455667719	0.783	0.231	4.03E-73	AQP3	2	IGKV1D-16_plasma cell
560	1.10E-47	0.454348052	0.865	0.365	2.40E-43	CANX	2	IGKV1D-16_plasma cell
561	8.58E-40	0.452573969	0.898	0.479	1.87E-35	SPCS1	2	IGKV1D-16_plasma cell
562	1.17E-64	0.45036555	0.7	0.232	2.55E-60	STT3A	2	IGKV1D-16_plasma cell
563	5.94E-78	0.444538819	0.683	0.207	1.30E-73	SLAMF7	2	IGKV1D-16_plasma cell
564	7.06E-54	0.442221093	0.856	0.384	1.54E-49	ERGIC3	2	IGKV1D-16_plasma cell
565	3.03E-37	0.441987694	0.76	0.37	6.61E-33	LGALS1	2	IGKV1D-16_plasma cell

566	5.02E-62	0.441498691	0.517	0.146	1.09E-57	ABCB9	2	IGKV1D-16_plasma cell
567	2.51E-35	0.437821113	0.944	0.655	5.47E-31	ISG20	2	IGKV1D-16_plasma cell
568	1.29E-37	0.436563322	0.952	0.546	2.82E-33	CALR	2	IGKV1D-16_plasma cell
569	4.17E-74	0.434301583	0.61	0.183	9.10E-70	SIL1	2	IGKV1D-16_plasma cell
570	4.10E-54	0.431017981	0.84	0.337	8.93E-50	DERL1	2	IGKV1D-16_plasma cell
571	1.96E-57	0.427975575	0.46	0.128	4.28E-53	IGF1	2	IGKV1D-16_plasma cell
572	9.05E-61	0.425175421	0.696	0.241	1.97E-56	SRM	2	IGKV1D-16_plasma cell
573	4.08E-60	0.425030422	0.694	0.245	8.89E-56	IDH2	2	IGKV1D-16_plasma cell
574	1.69E-47	0.424183356	0.865	0.366	3.68E-43	KDELR1	2	IGKV1D-16_plasma cell
575	2.58E-52	0.417749085	0.504	0.159	5.62E-48	CCDC88A	2	IGKV1D-16_plasma cell
576	1.86E-45	0.416782825	0.819	0.36	4.05E-41	SRPRA	2	IGKV1D-16_plasma cell
577	6.67E-44	0.407815974	0.852	0.362	1.46E-39	SSR1	2	IGKV1D-16_plasma cell
578	1.98E-31	0.406815123	0.398	0.147	4.31E-27	ATF5	2	IGKV1D-16_plasma cell
579	2.16E-63	0.406745745	0.758	0.269	4.70E-59	SAR1B	2	IGKV1D-16_plasma cell
580	5.99E-61	0.403792794	0.719	0.248	1.31E-56	LIME1	2	IGKV1D-16_plasma cell
581	9.99E-42	0.401011248	0.95	0.638	2.18E-37	SUB1	2	IGKV1D-16_plasma cell
582	1.01E-59	0.400791708	0.517	0.152	2.20E-55	RP11-16E12.:	2	IGKV1D-16_plasma cell
583	2.82E-58	0.400784989	0.658	0.242	6.16E-54	SEL1L	2	IGKV1D-16_plasma cell
584	1.04E-59	0.398285977	0.775	0.292	2.27E-55	LRPAP1	2	IGKV1D-16_plasma cell
585	3.96E-50	0.393652533	0.458	0.145	8.64E-46	ERN1	2	IGKV1D-16_plasma cell
586	2.37E-35	0.392208134	0.89	0.459	5.17E-31	DAD1	2	IGKV1D-16_plasma cell
587	1.39E-51	0.390178803	0.794	0.302	3.02E-47	DNAJB11	2	IGKV1D-16_plasma cell
588	6.94E-27	0.389478881	0.148	0.019	1.51E-22	DNAAF1	2	IGKV1D-16_plasma cell
589	1.64E-62	0.388581848	0.681	0.244	3.58E-58	CD59	2	IGKV1D-16_plasma cell
590	9.73E-54	0.386746654	0.746	0.295	2.12E-49	SEC14L1	2	IGKV1D-16_plasma cell
591	5.39E-58	0.384643238	0.521	0.155	1.18E-53	CPEB4	2	IGKV1D-16_plasma cell
592	1.61E-61	0.383305695	0.723	0.262	3.51E-57	DNAJC1	2	IGKV1D-16_plasma cell
593	2.12E-65	0.382422194	0.669	0.24	4.63E-61	FNDC3A	2	IGKV1D-16_plasma cell
594	3.69E-57	0.370666214	0.573	0.187	8.04E-53	GAS6	2	IGKV1D-16_plasma cell
595	2.76E-35	0.370611302	0.854	0.402	6.01E-31	TMED2	2	IGKV1D-16_plasma cell
596	2.52E-48	0.370248056	0.56	0.206	5.50E-44	EDEM1	2	IGKV1D-16_plasma cell
597	4.25E-59	0.368654602	0.746	0.278	9.27E-55	TMEM208	2	IGKV1D-16_plasma cell
598	9.29E-54	0.368638458	0.71	0.271	2.03E-49	DNAJC3	2	IGKV1D-16_plasma cell
599	4.49E-52	0.364930452	0.577	0.201	9.80E-48	SLC38A5	2	IGKV1D-16_plasma cell
600	1.18E-53	0.363512281	0.623	0.237	2.58E-49	SPATS2	2	IGKV1D-16_plasma cell
601	4.82E-58	0.361262903	0.621	0.22	1.05E-53	SLC44A1	2	IGKV1D-16_plasma cell
602	6.05E-53	0.359307355	0.794	0.356	1.32E-48	SEL1L3	2	IGKV1D-16_plasma cell
603	2.05E-53	0.356800995	0.756	0.277	4.46E-49	CRELD2	2	IGKV1D-16_plasma cell
604	2.45E-58	0.35466859	0.717	0.27	5.34E-54	ALG5	2	IGKV1D-16_plasma cell
605	1.49E-34	0.352634307	0.91	0.425	3.24E-30	COPE	2	IGKV1D-16_plasma cell
606	5.99E-55	0.350916162	0.673	0.26	1.31E-50	ACADVL	2	IGKV1D-16_plasma cell
607	2.81E-44	0.35086388	0.565	0.214	6.13E-40	SEC24D	2	IGKV1D-16_plasma cell
608	1.75E-49	0.343662624	0.565	0.206	3.82E-45	MAN1A1	2	IGKV1D-16_plasma cell
609	2.05E-29	0.340094448	0.885	0.475	4.47E-25	PDIA3	2	IGKV1D-16_plasma cell
610	1.95E-52	0.337045974	0.66	0.246	4.25E-48	CALU	2	IGKV1D-16_plasma cell
611	5.30E-46	0.330584638	0.783	0.307	1.16E-41	SEC13	2	IGKV1D-16_plasma cell
612	2.76E-27	0.328993555	0.892	0.477	6.01E-23	POU2AF1	2	IGKV1D-16_plasma cell
613	6.84E-43	0.327222764	0.504	0.185	1.49E-38	MIR4435-2H(	2	IGKV1D-16_plasma cell
614	5.81E-53	0.326753602	0.64	0.235	1.27E-48	HSPA13	2	IGKV1D-16_plasma cell
615	1.17E-45	0.326681327	0.738	0.315	2.56E-41	OS9	2	IGKV1D-16_plasma cell
616	1.70E-47	0.326531022	0.479	0.153	3.70E-43	RHBDD1	2	IGKV1D-16_plasma cell
617	3.27E-32	0.326423717	0.317	0.098	7.12E-28	CADM1	2	IGKV1D-16_plasma cell
618	6.12E-39	0.326102453	0.538	0.205	1.33E-34	P11-1070N1(	2	IGKV1D-16_plasma cell
619	1.49E-30	0.325927624	0.875	0.432	3.25E-26	ARF4	2	IGKV1D-16_plasma cell
620	5.74E-52	0.317928946	0.65	0.248	1.25E-47	SLC38A10	2	IGKV1D-16_plasma cell
621	1.04E-45	0.315203738	0.373	0.099	2.26E-41	CHST2	2	IGKV1D-16_plasma cell
622	7.95E-18	0.311412426	0.76	0.451	1.73E-13	CITED2	2	IGKV1D-16_plasma cell
623	8.87E-48	0.309422846	0.606	0.234	1.93E-43	SLC39A7	2	IGKV1D-16_plasma cell
624	9.72E-53	0.308165323	0.529	0.171	2.12E-48	PDK1	2	IGKV1D-16_plasma cell
625	3.48E-28	0.305640289	0.538	0.246	7.58E-24	RNF213	2	IGKV1D-16_plasma cell
626	9.31E-23	0.305300375	0.2	0.052	2.03E-18	IGHJ4	2	IGKV1D-16_plasma cell
627	8.47E-25	0.303706964	0.352	0.144	1.85E-20	CCR2	2	IGKV1D-16_plasma cell
628	6.56E-58	0.303346489	0.633	0.222	1.43E-53	EDEM2	2	IGKV1D-16_plasma cell
629	7.54E-34	0.303139304	0.569	0.246	1.65E-29	ITGB7	2	IGKV1D-16_plasma cell
630	1.75E-53	0.300479919	0.671	0.264	3.81E-49	CTSD	2	IGKV1D-16_plasma cell
631	1.43E-44	0.297431687	0.521	0.187	3.12E-40	SEC24A	2	IGKV1D-16_plasma cell
632	4.04E-32	0.297124685	0.629	0.289	8.81E-28	TNFRSF13B	2	IGKV1D-16_plasma cell
633	3.51E-45	0.296840735	0.71	0.298	7.66E-41	MGAT1	2	IGKV1D-16_plasma cell
634	1.54E-35	0.295523835	0.423	0.153	3.35E-31	BIK	2	IGKV1D-16_plasma cell
635	7.94E-39	0.295307966	0.604	0.237	1.73E-34	UAP1	2	IGKV1D-16_plasma cell
636	3.76E-44	0.290309034	0.435	0.141	8.20E-40	KCNK6	2	IGKV1D-16_plasma cell

637	2.29E-48	0.288661145	0.567	0.202	4.99E-44	BCL2L11	2	IGKV1D-16_plasma cell
638	1.12E-36	0.288464525	0.65	0.28	2.45E-32	GLRX	2	IGKV1D-16_plasma cell
639	6.81E-23	0.286484311	0.904	0.507	1.49E-18	TMEM258	2	IGKV1D-16_plasma cell
640	4.60E-26	0.283149062	0.875	0.453	1.00E-21	SELK	2	IGKV1D-16_plasma cell
641	5.88E-46	0.282186776	0.696	0.258	1.28E-41	MLEC	2	IGKV1D-16_plasma cell
642	3.26E-46	0.280348957	0.594	0.223	7.11E-42	NCLN	2	IGKV1D-16_plasma cell
643	1.30E-46	0.279192124	0.565	0.214	2.83E-42	KLF13	2	IGKV1D-16_plasma cell
644	2.53E-27	0.275045385	0.831	0.399	5.51E-23	UQCRQ	2	IGKV1D-16_plasma cell
645	1.17E-46	0.274600778	0.577	0.217	2.54E-42	CPNE5	2	IGKV1D-16_plasma cell
646	2.13E-41	0.273930491	0.631	0.242	4.66E-37	GMPPB	2	IGKV1D-16_plasma cell
647	3.00E-45	0.270456093	0.679	0.268	6.54E-41	TXNDC15	2	IGKV1D-16_plasma cell
648	1.16E-40	0.270013993	0.683	0.298	2.52E-36	RAB30	2	IGKV1D-16_plasma cell
649	7.85E-38	0.269396292	0.49	0.187	1.71E-33	PECAM1	2	IGKV1D-16_plasma cell
650	1.91E-48	0.26859324	0.608	0.22	4.16E-44	B4GALT3	2	IGKV1D-16_plasma cell
651	9.44E-52	0.267809013	0.721	0.287	2.06E-47	FCRL5	2	IGKV1D-16_plasma cell
652	4.33E-51	0.265948812	0.521	0.169	9.44E-47	CECR1	2	IGKV1D-16_plasma cell
653	1.83E-32	0.26500449	0.598	0.267	3.99E-28	HCST	2	IGKV1D-16_plasma cell
654	1.28E-42	0.264585897	0.667	0.259	2.78E-38	PREB	2	IGKV1D-16_plasma cell
655	5.28E-14	0.26399595	0.167	0.057	1.15E-09	GPR15	2	IGKV1D-16_plasma cell
656	7.36E-44	0.262918744	0.635	0.251	1.60E-39	ARFGAP3	2	IGKV1D-16_plasma cell
657	1.88E-37	0.262454865	0.838	0.362	4.10E-33	VOPP1	2	IGKV1D-16_plasma cell
658	5.65E-48	0.261081621	0.696	0.278	1.23E-43	DERL2	2	IGKV1D-16_plasma cell
659	1.12E-40	0.260858802	0.66	0.278	2.44E-36	NUCB1	2	IGKV1D-16_plasma cell
660	1.79E-43	0.259905259	0.55	0.21	3.90E-39	ST6GALNAC	2	IGKV1D-16_plasma cell
661	1.01E-40	0.253923084	0.592	0.223	2.21E-36	SLC35B1	2	IGKV1D-16_plasma cell
662	5.18E-31	0.252977965	0.59	0.256	1.13E-26	PIM1	2	IGKV1D-16_plasma cell
663	2.46E-19	0.251340221	0.275	0.106	5.37E-15	ADM	2	IGKV1D-16_plasma cell
664	2.67E-41	0.251200312	0.415	0.138	5.82E-37	NOMO2	2	IGKV1D-16_plasma cell
665	2.71E-39	0.250994563	0.485	0.182	5.92E-35	MEI1	2	IGKV1D-16_plasma cell
666	1.71E-06	-0.257573697	0.848	0.702	0.037232046	SRP14	2	IGKV1D-16_plasma cell
667	2.84E-07	-0.296586036	0.827	0.679	0.006185919	EIF3K	2	IGKV1D-16_plasma cell
668	2.80E-07	-0.321613805	0.827	0.693	0.006100135	GABARAP	2	IGKV1D-16_plasma cell
669	1.59E-06	-0.335601066	0.829	0.65	0.034754177	ARPC2	2	IGKV1D-16_plasma cell
670	7.92E-10	-0.347841951	0.769	0.757	1.73E-05	JUN	2	IGKV1D-16_plasma cell
671	2.35E-09	-0.353417135	0.848	0.71	5.13E-05	UQCRB	2	IGKV1D-16_plasma cell
672	3.21E-14	-0.35356176	0.85	0.782	7.00E-10	SLC25A6	2	IGKV1D-16_plasma cell
673	4.63E-37	-0.35498578	1	1	1.01E-32	MALAT1	2	IGKV1D-16_plasma cell
674	5.43E-35	-0.359022443	0.994	0.973	1.18E-30	MT-ND1	2	IGKV1D-16_plasma cell
675	2.37E-37	-0.363456825	0.985	0.993	5.16E-33	RPLP0	2	IGKV1D-16_plasma cell
676	3.54E-28	-0.367953371	0.952	0.945	7.72E-24	EEF2	2	IGKV1D-16_plasma cell
677	8.81E-41	-0.382948274	0.979	0.99	1.92E-36	FTL	2	IGKV1D-16_plasma cell
678	1.90E-11	-0.392137769	0.673	0.685	4.13E-07	KLF6	2	IGKV1D-16_plasma cell
679	2.41E-10	-0.393882818	0.785	0.664	5.26E-06	PCBP1	2	IGKV1D-16_plasma cell
680	9.88E-19	-0.416884532	0.877	0.829	2.16E-14	ACTG1	2	IGKV1D-16_plasma cell
681	6.17E-51	-0.441319032	0.994	0.998	1.35E-46	MT-ND3	2	IGKV1D-16_plasma cell
682	2.89E-07	-0.449654088	0.725	0.597	0.006297249	EIF4G2	2	IGKV1D-16_plasma cell
683	7.17E-16	-0.451300799	0.84	0.723	1.56E-11	CALM1	2	IGKV1D-16_plasma cell
684	1.56E-06	-0.455016889	0.285	0.351	0.034028007	IMPDH2	2	IGKV1D-16_plasma cell
685	6.49E-49	-0.455455424	0.956	0.998	1.42E-44	TPT1	2	IGKV1D-16_plasma cell
686	9.24E-07	-0.467544038	0.735	0.621	0.020160866	EIF4B	2	IGKV1D-16_plasma cell
687	5.16E-08	-0.46814013	0.767	0.612	0.001125932	SLC25A5	2	IGKV1D-16_plasma cell
688	1.37E-06	-0.468703996	0.156	0.243	0.029948651	CYFIP2	2	IGKV1D-16_plasma cell
689	1.54E-06	-0.47308553	0.106	0.188	0.033521138	CAMK1D	2	IGKV1D-16_plasma cell
690	4.97E-11	-0.473347773	0.81	0.663	1.08E-06	RAC2	2	IGKV1D-16_plasma cell
691	1.60E-21	-0.474666574	0.842	0.721	3.48E-17	RHOA	2	IGKV1D-16_plasma cell
692	4.46E-11	-0.477828283	0.738	0.624	9.72E-07	HMGN1	2	IGKV1D-16_plasma cell
693	3.74E-40	-0.47940508	0.99	0.968	8.16E-36	MT-ND4	2	IGKV1D-16_plasma cell
694	3.31E-08	-0.483141443	0.138	0.245	0.000722182	RP9	2	IGKV1D-16_plasma cell
695	1.35E-08	-0.483370296	0.108	0.213	0.000294493	CYB561A3	2	IGKV1D-16_plasma cell
696	6.31E-07	-0.494933852	0.706	0.596	0.013762766	UXT	2	IGKV1D-16_plasma cell
697	1.23E-08	-0.497155226	0.108	0.214	0.000268623	C6orf48	2	IGKV1D-16_plasma cell
698	7.55E-07	-0.501964653	0.627	0.545	0.01645667	ACTR3	2	IGKV1D-16_plasma cell
699	1.15E-06	-0.504506293	0.16	0.252	0.024970932	FKBP5	2	IGKV1D-16_plasma cell
700	1.64E-06	-0.505395335	0.144	0.228	0.035837483	RP5-1171I10.	2	IGKV1D-16_plasma cell
701	1.06E-21	-0.505618779	0.865	0.769	2.31E-17	HNRNPDL	2	IGKV1D-16_plasma cell
702	7.18E-23	-0.511786411	0.81	0.727	1.57E-18	TMA7	2	IGKV1D-16_plasma cell
703	7.79E-24	-0.513421763	0.858	0.793	1.70E-19	YBX1	2	IGKV1D-16_plasma cell
704	8.32E-68	-0.516031236	0.979	0.995	1.81E-63	RPL7A	2	IGKV1D-16_plasma cell
705	1.25E-16	-0.520298392	0.76	0.686	2.73E-12	EIF3F	2	IGKV1D-16_plasma cell
706	1.12E-42	-0.521218447	0.912	0.897	2.44E-38	OAZ1	2	IGKV1D-16_plasma cell
707	2.06E-06	-0.521219518	0.131	0.215	0.044832758	MYADM	2	IGKV1D-16_plasma cell

708	1.10E-09	-0.529098322	0.688	0.627	2.39E-05	C12orf57	2	IGKV1D-16_plasma cell
709	2.21E-07	-0.530661914	0.167	0.264	0.004825399	TSPYL2	2	IGKV1D-16_plasma cell
710	1.40E-06	-0.531814117	0.262	0.345	0.030600031	ELOVL5	2	IGKV1D-16_plasma cell
711	1.07E-41	-0.533148233	0.906	0.848	2.34E-37	HNRNPA2B1	2	IGKV1D-16_plasma cell
712	5.04E-08	-0.534872222	0.148	0.248	0.001098317	IFT57	2	IGKV1D-16_plasma cell
713	1.26E-06	-0.536922383	0.231	0.315	0.027483205	NR3C1	2	IGKV1D-16_plasma cell
714	7.68E-13	-0.538529856	0.731	0.634	1.67E-08	SKP1	2	IGKV1D-16_plasma cell
715	5.30E-10	-0.540489536	0.771	0.691	1.16E-05	PPP1R15A	2	IGKV1D-16_plasma cell
716	3.92E-40	-0.547365011	0.923	0.922	8.54E-36	PPIA	2	IGKV1D-16_plasma cell
717	6.57E-13	-0.549631045	0.644	0.616	1.43E-08	EIF3H	2	IGKV1D-16_plasma cell
718	8.80E-38	-0.551018019	0.908	0.895	1.92E-33	H3F3A	2	IGKV1D-16_plasma cell
719	2.06E-37	-0.552520919	0.825	0.809	4.49E-33	ATP5G2	2	IGKV1D-16_plasma cell
720	8.92E-28	-0.552703059	0.915	0.916	1.94E-23	CD79A	2	IGKV1D-16_plasma cell
721	7.14E-41	-0.559674783	0.85	0.837	1.56E-36	COX7C	2	IGKV1D-16_plasma cell
722	1.17E-28	-0.560585115	0.881	0.828	2.55E-24	HSPA8	2	IGKV1D-16_plasma cell
723	1.79E-07	-0.562025936	0.158	0.252	0.00391251	MARCKSL1	2	IGKV1D-16_plasma cell
724	2.69E-19	-0.567986136	0.729	0.667	5.86E-15	SNRPD2	2	IGKV1D-16_plasma cell
725	5.13E-09	-0.570677193	0.6	0.547	0.000111906	TPM3	2	IGKV1D-16_plasma cell
726	2.16E-13	-0.574037009	0.7	0.635	4.72E-09	EIF3L	2	IGKV1D-16_plasma cell
727	1.99E-08	-0.576490553	0.59	0.535	0.000433043	RBM8A	2	IGKV1D-16_plasma cell
728	9.49E-11	-0.57786615	0.806	0.741	2.07E-06	EZR	2	IGKV1D-16_plasma cell
729	2.27E-36	-0.581729549	0.879	0.84	4.95E-32	NPM1	2	IGKV1D-16_plasma cell
730	3.06E-10	-0.582597803	0.694	0.593	6.67E-06	LSM7	2	IGKV1D-16_plasma cell
731	3.38E-11	-0.583465927	0.106	0.231	7.38E-07	UTRN	2	IGKV1D-16_plasma cell
732	8.97E-09	-0.583596112	0.64	0.552	0.000195575	GDI2	2	IGKV1D-16_plasma cell
733	8.47E-74	-0.587809343	0.962	0.988	1.85E-69	RPS24	2	IGKV1D-16_plasma cell
734	7.74E-08	-0.588040699	0.648	0.603	0.001687865	STK4	2	IGKV1D-16_plasma cell
735	3.01E-07	-0.588865779	0.431	0.46	0.006573649	NSA2	2	IGKV1D-16_plasma cell
736	2.58E-07	-0.589070068	0.56	0.546	0.00563108	MBNL1	2	IGKV1D-16_plasma cell
737	3.16E-14	-0.589744961	0.102	0.253	6.90E-10	LAT2	2	IGKV1D-16_plasma cell
738	3.03E-09	-0.591812839	0.258	0.36	6.60E-05	MTPN	2	IGKV1D-16_plasma cell
739	3.36E-53	-0.594199257	0.877	0.909	7.33E-49	EEF1G	2	IGKV1D-16_plasma cell
740	6.47E-32	-0.594351275	0.788	0.767	1.41E-27	RPS17	2	IGKV1D-16_plasma cell
741	1.55E-09	-0.594653566	0.404	0.467	3.37E-05	IFI16	2	IGKV1D-16_plasma cell
742	8.68E-12	-0.597089116	0.652	0.6	1.89E-07	C19orf43	2	IGKV1D-16_plasma cell
743	1.46E-25	-0.59926733	0.885	0.793	3.18E-21	HMGB1	2	IGKV1D-16_plasma cell
744	1.11E-18	-0.600382976	0.702	0.669	2.41E-14	SMDT1	2	IGKV1D-16_plasma cell
745	1.04E-07	-0.601437779	0.623	0.587	0.002278324	SNHG8	2	IGKV1D-16_plasma cell
746	3.05E-24	-0.601679761	0.752	0.723	6.65E-20	FXYD5	2	IGKV1D-16_plasma cell
747	1.69E-49	-0.607692943	0.969	0.944	3.68E-45	MT-ND2	2	IGKV1D-16_plasma cell
748	1.55E-07	-0.609124747	0.481	0.495	0.003377615	SH3BGRL	2	IGKV1D-16_plasma cell
749	1.67E-08	-0.60919941	0.45	0.5	0.000363271	SMAP2	2	IGKV1D-16_plasma cell
750	3.09E-09	-0.610166915	0.238	0.342	6.75E-05	CD19	2	IGKV1D-16_plasma cell
751	7.18E-07	-0.611301023	0.756	0.647	0.01566644	MEF2C	2	IGKV1D-16_plasma cell
752	6.25E-83	-0.613416638	0.969	0.994	1.36E-78	RPS4X	2	IGKV1D-16_plasma cell
753	2.92E-09	-0.61451813	0.488	0.501	6.37E-05	MYL12B	2	IGKV1D-16_plasma cell
754	2.37E-11	-0.616684269	0.573	0.564	5.18E-07	HNRNPA0	2	IGKV1D-16_plasma cell
755	2.86E-07	-0.617987161	0.592	0.567	0.006229749	SRSF5	2	IGKV1D-16_plasma cell
756	1.15E-06	-0.618423806	0.331	0.391	0.025041128	SH3BP5	2	IGKV1D-16_plasma cell
757	7.62E-09	-0.619031596	0.56	0.551	0.000166151	7-Sep	2	IGKV1D-16_plasma cell
758	1.58E-08	-0.619445673	0.269	0.362	0.000344276	STX7	2	IGKV1D-16_plasma cell
759	5.55E-24	-0.620310435	0.79	0.707	1.21E-19	HNRNPK	2	IGKV1D-16_plasma cell
760	3.84E-10	-0.620973541	0.612	0.538	8.38E-06	ANP32B	2	IGKV1D-16_plasma cell
761	7.33E-07	-0.623342344	0.492	0.498	0.015976308	SLC38A1	2	IGKV1D-16_plasma cell
762	2.16E-12	-0.628449958	0.148	0.289	4.70E-08	BTLA	2	IGKV1D-16_plasma cell
763	1.71E-07	-0.629349956	0.227	0.316	0.003738871	ATF7IP	2	IGKV1D-16_plasma cell
764	1.44E-09	-0.629703599	0.438	0.485	3.14E-05	PRR13	2	IGKV1D-16_plasma cell
765	2.08E-06	-0.633057839	0.504	0.488	0.045268306	ARL6IP1	2	IGKV1D-16_plasma cell
766	5.58E-10	-0.633470292	0.525	0.528	1.22E-05	CD47	2	IGKV1D-16_plasma cell
767	6.98E-10	-0.636809003	0.617	0.606	1.52E-05	EIF4A2	2	IGKV1D-16_plasma cell
768	7.15E-58	-0.644289738	0.9	0.891	1.56E-53	COX4I1	2	IGKV1D-16_plasma cell
769	1.70E-12	-0.651223609	0.11	0.028	3.71E-08	IGKV1D-12	2	IGKV1D-16_plasma cell
770	2.72E-24	-0.652127146	0.769	0.675	5.93E-20	SUMO2	2	IGKV1D-16_plasma cell
771	1.29E-15	-0.652690215	0.631	0.626	2.81E-11	ANAPC16	2	IGKV1D-16_plasma cell
772	2.41E-13	-0.656694667	0.598	0.581	5.25E-09	GPX4	2	IGKV1D-16_plasma cell
773	4.93E-07	-0.656828115	0.208	0.296	0.01076171	YBX3	2	IGKV1D-16_plasma cell
774	2.56E-09	-0.657183311	0.258	0.364	5.59E-05	SSH2	2	IGKV1D-16_plasma cell
775	5.77E-49	-0.658625549	0.846	0.847	1.26E-44	HINT1	2	IGKV1D-16_plasma cell
776	3.40E-39	-0.65870924	0.817	0.774	7.42E-35	ARPC3	2	IGKV1D-16_plasma cell
777	1.27E-17	-0.659547412	0.658	0.643	2.77E-13	SRSF7	2	IGKV1D-16_plasma cell
778	1.88E-08	-0.659944219	0.481	0.507	0.000409501	PTPN6	2	IGKV1D-16_plasma cell

779	3.83E-07	-0.668506281	0.371	0.426	0.00836074	TSPAN3	2	IGKV1D-16_plasma cell
780	4.84E-11	-0.669319859	0.25	0.37	1.05E-06	SERTAD1	2	IGKV1D-16_plasma cell
781	3.06E-08	-0.669783226	0.215	0.31	0.000667731	DDIT3	2	IGKV1D-16_plasma cell
782	5.14E-32	-0.674985771	0.808	0.762	1.12E-27	UBB	2	IGKV1D-16_plasma cell
783	8.16E-104	-0.675667507	0.994	0.999	1.78E-99	RPLP1	2	IGKV1D-16_plasma cell
784	5.97E-27	-0.675758905	0.775	0.73	1.30E-22	SH3BGRL3	2	IGKV1D-16_plasma cell
785	6.54E-28	-0.677336055	0.919	0.887	1.43E-23	IER2	2	IGKV1D-16_plasma cell
786	1.70E-45	-0.681427947	0.873	0.821	3.71E-41	CFL1	2	IGKV1D-16_plasma cell
787	4.26E-70	-0.682906212	0.925	0.939	9.29E-66	RPL4	2	IGKV1D-16_plasma cell
788	6.77E-19	-0.685766457	0.619	0.635	1.48E-14	HMGN2	2	IGKV1D-16_plasma cell
789	7.56E-77	-0.686583726	0.96	0.979	1.65E-72	RPS2	2	IGKV1D-16_plasma cell
790	2.67E-14	-0.687279581	0.106	0.256	5.83E-10	UVRAG	2	IGKV1D-16_plasma cell
791	2.21E-12	-0.688212092	0.454	0.511	4.81E-08	CNN2	2	IGKV1D-16_plasma cell
792	9.80E-81	-0.690296841	0.938	0.976	2.14E-76	EEF1B2	2	IGKV1D-16_plasma cell
793	2.09E-08	-0.690499758	0.662	0.594	0.000455934	CD79B	2	IGKV1D-16_plasma cell
794	4.51E-81	-0.693065576	0.933	0.976	9.83E-77	RPL24	2	IGKV1D-16_plasma cell
795	8.54E-13	-0.695344792	0.121	0.255	1.86E-08	SMIM14	2	IGKV1D-16_plasma cell
796	1.22E-10	-0.69644402	0.4	0.473	2.65E-06	EIF1B	2	IGKV1D-16_plasma cell
797	2.45E-50	-0.705748247	0.873	0.834	5.35E-46	HNRNPA1	2	IGKV1D-16_plasma cell
798	8.32E-15	-0.707807309	0.45	0.507	1.81E-10	NAP1L1	2	IGKV1D-16_plasma cell
799	4.51E-11	-0.707928253	0.6	0.595	9.84E-07	TAGAP	2	IGKV1D-16_plasma cell
800	2.71E-16	-0.708777136	0.15	0.316	5.91E-12	SP110	2	IGKV1D-16_plasma cell
801	8.01E-13	-0.709453056	0.46	0.51	1.75E-08	ST13	2	IGKV1D-16_plasma cell
802	7.39E-19	-0.711976844	0.529	0.608	1.61E-14	CD48	2	IGKV1D-16_plasma cell
803	9.78E-37	-0.712595099	0.79	0.785	2.13E-32	CCNI	2	IGKV1D-16_plasma cell
804	1.40E-19	-0.713484831	0.644	0.651	3.05E-15	ATP6V1G1	2	IGKV1D-16_plasma cell
805	3.36E-15	-0.713492225	0.171	0.329	7.33E-11	MAPRE2	2	IGKV1D-16_plasma cell
806	5.96E-54	-0.713615844	0.85	0.857	1.30E-49	RPL27A	2	IGKV1D-16_plasma cell
807	6.38E-19	-0.714115487	0.646	0.672	1.39E-14	FUS	2	IGKV1D-16_plasma cell
808	2.40E-57	-0.715199891	0.815	0.861	5.23E-53	BTF3	2	IGKV1D-16_plasma cell
809	1.25E-40	-0.715851358	0.8	0.793	2.72E-36	RPL23	2	IGKV1D-16_plasma cell
810	1.98E-11	-0.717906667	0.346	0.438	4.31E-07	SNHG7	2	IGKV1D-16_plasma cell
811	9.15E-43	-0.719882641	0.898	0.868	1.99E-38	DDX5	2	IGKV1D-16_plasma cell
812	4.77E-16	-0.72265132	0.135	0.299	1.04E-11	IFITM1	2	IGKV1D-16_plasma cell
813	7.26E-22	-0.730196021	0.81	0.75	1.58E-17	BRD2	2	IGKV1D-16_plasma cell
814	7.20E-15	-0.731079663	0.15	0.3	1.57E-10	CD40	2	IGKV1D-16_plasma cell
815	1.81E-09	-0.731892035	0.296	0.388	3.95E-05	MAP3K8	2	IGKV1D-16_plasma cell
816	7.70E-09	-0.73447126	0.321	0.403	0.000167966	NR4A1	2	IGKV1D-16_plasma cell
817	6.81E-98	-0.735446414	0.969	0.99	1.49E-93	RPL3	2	IGKV1D-16_plasma cell
818	8.75E-07	-0.736460874	0.41	0.438	0.019092105	POLD4	2	IGKV1D-16_plasma cell
819	3.35E-38	-0.737222694	0.833	0.825	7.30E-34	SARAF	2	IGKV1D-16_plasma cell
820	3.79E-33	-0.739573288	0.765	0.754	8.26E-29	EIF3E	2	IGKV1D-16_plasma cell
821	2.15E-44	-0.740870935	0.881	0.848	4.70E-40	HLA-E	2	IGKV1D-16_plasma cell
822	1.18E-19	-0.741618379	0.577	0.617	2.57E-15	DAZAP2	2	IGKV1D-16_plasma cell
823	2.26E-17	-0.74188544	0.369	0.498	4.92E-13	UBXN1	2	IGKV1D-16_plasma cell
824	3.84E-28	-0.74433882	0.827	0.785	8.36E-24	PTPRCAP	2	IGKV1D-16_plasma cell
825	1.73E-10	-0.74907179	0.385	0.455	3.78E-06	CMTM6	2	IGKV1D-16_plasma cell
826	2.20E-97	-0.750587565	0.94	0.981	4.80E-93	RPL35	2	IGKV1D-16_plasma cell
827	5.00E-19	-0.755847071	0.604	0.615	1.09E-14	SNX3	2	IGKV1D-16_plasma cell
828	1.56E-14	-0.756567422	0.344	0.437	3.40E-10	STMN1	2	IGKV1D-16_plasma cell
829	3.15E-96	-0.769929708	0.954	0.985	6.87E-92	RPL37A	2	IGKV1D-16_plasma cell
830	2.79E-122	-0.773535234	0.983	0.997	6.09E-118	RPL8	2	IGKV1D-16_plasma cell
831	2.85E-13	-0.773795313	0.269	0.397	6.22E-09	ANKRD44	2	IGKV1D-16_plasma cell
832	3.78E-14	-0.776806683	0.267	0.4	8.24E-10	DRAM2	2	IGKV1D-16_plasma cell
833	1.08E-59	-0.77818172	0.821	0.879	2.35E-55	RPL36A	2	IGKV1D-16_plasma cell
834	1.09E-12	-0.780974534	0.421	0.498	2.39E-08	LYN	2	IGKV1D-16_plasma cell
835	7.40E-20	-0.782118615	0.271	0.448	1.61E-15	CELF2	2	IGKV1D-16_plasma cell
836	8.30E-09	-0.787941892	0.496	0.527	0.000181017	DUSP2	2	IGKV1D-16_plasma cell
837	1.39E-16	-0.788694113	0.206	0.369	3.03E-12	SWAP70	2	IGKV1D-16_plasma cell
838	1.15E-94	-0.789672742	0.933	0.98	2.51E-90	NACA	2	IGKV1D-16_plasma cell
839	2.18E-15	-0.793162518	0.308	0.441	4.76E-11	EVI2B	2	IGKV1D-16_plasma cell
840	3.15E-10	-0.7958684	0.317	0.404	6.88E-06	LY9	2	IGKV1D-16_plasma cell
841	1.24E-71	-0.796312772	0.956	0.945	2.71E-67	H3F3B	2	IGKV1D-16_plasma cell
842	6.29E-68	-0.799812312	0.888	0.873	1.37E-63	PFN1	2	IGKV1D-16_plasma cell
843	4.08E-89	-0.800495244	0.942	0.948	8.90E-85	EEF1D	2	IGKV1D-16_plasma cell
844	2.30E-25	-0.801524516	0.635	0.669	5.03E-21	SF1	2	IGKV1D-16_plasma cell
845	4.55E-113	-0.804067221	0.944	0.993	9.91E-109	RPL15	2	IGKV1D-16_plasma cell
846	2.62E-18	-0.804473477	0.4	0.533	5.71E-14	BIRC3	2	IGKV1D-16_plasma cell
847	9.76E-17	-0.804607747	0.279	0.435	2.13E-12	LRRFIP1	2	IGKV1D-16_plasma cell
848	1.70E-87	-0.807800977	0.956	0.989	3.70E-83	TMSB10	2	IGKV1D-16_plasma cell
849	2.49E-98	-0.8087312	0.933	0.957	5.43E-94	RPL41	2	IGKV1D-16_plasma cell

850	2.93E-61	-0.809395679	0.796	0.835	6.40E-57	PCBP2	2	IGKV1D-16_plasma cell
851	4.69E-121	-0.810248918	0.946	0.995	1.02E-116	RPS7	2	IGKV1D-16_plasma cell
852	8.52E-87	-0.811896175	0.865	0.938	1.86E-82	RPL27	2	IGKV1D-16_plasma cell
853	2.15E-20	-0.814513686	0.64	0.651	4.68E-16	SYNGR2	2	IGKV1D-16_plasma cell
854	1.61E-14	-0.815467213	0.356	0.463	3.52E-10	BAX	2	IGKV1D-16_plasma cell
855	1.18E-90	-0.81557312	0.915	0.979	2.56E-86	RPS11	2	IGKV1D-16_plasma cell
856	6.78E-15	-0.816991354	0.575	0.549	1.48E-10	TUBB	2	IGKV1D-16_plasma cell
857	3.72E-119	-0.819927915	0.95	0.994	8.12E-115	RPS16	2	IGKV1D-16_plasma cell
858	4.85E-18	-0.821927993	0.571	0.606	1.06E-13	LIMD2	2	IGKV1D-16_plasma cell
859	1.05E-86	-0.824697278	0.856	0.944	2.30E-82	RPL7	2	IGKV1D-16_plasma cell
860	2.46E-10	-0.824745114	0.502	0.527	5.36E-06	FOSB	2	IGKV1D-16_plasma cell
861	9.43E-20	-0.828920066	0.392	0.523	2.06E-15	GPSM3	2	IGKV1D-16_plasma cell
862	2.66E-123	-0.83453155	0.994	0.999	5.81E-119	RPL10	2	IGKV1D-16_plasma cell
863	3.43E-14	-0.838211136	0.483	0.551	7.47E-10	RHOH	2	IGKV1D-16_plasma cell
864	9.28E-31	-0.839539751	0.644	0.669	2.02E-26	CORO1A	2	IGKV1D-16_plasma cell
865	2.62E-21	-0.841233011	0.34	0.487	5.71E-17	COTL1	2	IGKV1D-16_plasma cell
866	2.34E-29	-0.848226835	0.75	0.764	5.09E-25	PNRC1	2	IGKV1D-16_plasma cell
867	1.82E-48	-0.850283042	0.881	0.865	3.98E-44	HSP90AB1	2	IGKV1D-16_plasma cell
868	7.42E-110	-0.859515747	0.938	0.986	1.62E-105	GNB2L1	2	IGKV1D-16_plasma cell
869	1.40E-113	-0.866820008	0.942	0.984	3.06E-109	RPL5	2	IGKV1D-16_plasma cell
870	2.88E-80	-0.871296318	0.902	0.913	6.27E-76	PABPC1	2	IGKV1D-16_plasma cell
871	3.43E-26	-0.871721958	0.671	0.677	7.47E-22	TAGLN2	2	IGKV1D-16_plasma cell
872	1.70E-136	-0.875154803	0.983	0.998	3.70E-132	RPS8	2	IGKV1D-16_plasma cell
873	4.83E-123	-0.87726371	0.929	0.987	1.05E-118	RPL6	2	IGKV1D-16_plasma cell
874	1.51E-35	-0.880501796	0.671	0.71	3.29E-31	MYL12A	2	IGKV1D-16_plasma cell
875	1.41E-125	-0.885122226	0.977	0.996	3.07E-121	RPS15A	2	IGKV1D-16_plasma cell
876	2.90E-40	-0.88692705	0.729	0.772	6.32E-36	CIRBP	2	IGKV1D-16_plasma cell
877	1.78E-29	-0.892672673	0.5	0.619	3.87E-25	PPDPF	2	IGKV1D-16_plasma cell
878	4.42E-83	-0.905428836	0.84	0.907	9.63E-79	RPL31	2	IGKV1D-16_plasma cell
879	5.41E-18	-0.909586283	0.258	0.421	1.18E-13	STK17A	2	IGKV1D-16_plasma cell
880	2.47E-123	-0.912278389	0.952	0.99	5.38E-119	RPS5	2	IGKV1D-16_plasma cell
881	2.44E-28	-0.914221054	0.448	0.608	5.31E-24	HLA-DMA	2	IGKV1D-16_plasma cell
882	9.42E-138	-0.918650537	0.979	0.997	2.06E-133	RPS15	2	IGKV1D-16_plasma cell
883	4.50E-24	-0.920992074	0.292	0.482	9.82E-20	TNFAIP8	2	IGKV1D-16_plasma cell
884	3.63E-33	-0.926551111	0.14	0.41	7.92E-29	SYPL1	2	IGKV1D-16_plasma cell
885	2.49E-112	-0.93066487	0.923	0.971	5.43E-108	UBA52	2	IGKV1D-16_plasma cell
886	3.25E-46	-0.936578582	0.923	0.912	7.09E-42	ZFP36	2	IGKV1D-16_plasma cell
887	7.34E-132	-0.937019896	0.938	0.991	1.60E-127	RPL14	2	IGKV1D-16_plasma cell
888	2.93E-20	-0.940408871	0.352	0.495	6.38E-16	RCSD1	2	IGKV1D-16_plasma cell
889	5.28E-31	-0.941040206	0.444	0.606	1.15E-26	CD53	2	IGKV1D-16_plasma cell
890	2.29E-18	-0.944511664	0.648	0.665	5.00E-14	GADD45B	2	IGKV1D-16_plasma cell
891	6.13E-122	-0.948032011	0.94	0.983	1.34E-117	RPS9	2	IGKV1D-16_plasma cell
892	1.48E-139	-0.956209679	0.971	0.997	3.22E-135	RPS3	2	IGKV1D-16_plasma cell
893	1.99E-127	-0.957641601	0.927	0.989	4.34E-123	RPL23A	2	IGKV1D-16_plasma cell
894	2.73E-46	-0.961276992	0.606	0.713	5.94E-42	ARHGDIB	2	IGKV1D-16_plasma cell
895	1.65E-14	-0.962231243	0.165	0.313	3.59E-10	ZNF331	2	IGKV1D-16_plasma cell
896	1.63E-130	-0.964310783	0.975	0.996	3.57E-126	RPS18	2	IGKV1D-16_plasma cell
897	7.04E-31	-0.965705283	0.694	0.796	1.54E-26	FOS	2	IGKV1D-16_plasma cell
898	9.38E-26	-0.966800177	0.125	0.351	2.05E-21	ADAM28	2	IGKV1D-16_plasma cell
899	3.76E-143	-0.969671575	0.967	0.997	8.21E-139	RPS28	2	IGKV1D-16_plasma cell
900	1.36E-138	-0.970880011	0.971	0.997	2.96E-134	FAU	2	IGKV1D-16_plasma cell
901	5.18E-29	-0.973715146	0.306	0.515	1.13E-24	SNX2	2	IGKV1D-16_plasma cell
902	4.24E-142	-0.975744551	0.973	0.999	9.24E-138	RPL18	2	IGKV1D-16_plasma cell
903	1.96E-25	-0.975766875	0.227	0.438	4.28E-21	SELL	2	IGKV1D-16_plasma cell
904	2.67E-72	-0.97802409	0.842	0.866	5.82E-68	YWHAZ	2	IGKV1D-16_plasma cell
905	2.14E-15	-0.978434221	0.39	0.498	4.67E-11	DNAJB1	2	IGKV1D-16_plasma cell
906	3.51E-140	-0.979121514	0.94	0.997	7.66E-136	RPS13	2	IGKV1D-16_plasma cell
907	8.39E-36	-0.980370592	0.715	0.77	1.83E-31	NFKBIA	2	IGKV1D-16_plasma cell
908	3.66E-39	-0.98097328	0.838	0.826	7.99E-35	HSP90AA1	2	IGKV1D-16_plasma cell
909	1.53E-134	-0.985686513	0.944	0.994	3.33E-130	RPS14	2	IGKV1D-16_plasma cell
910	7.90E-116	-0.986396183	0.946	0.989	1.72E-111	EIF1	2	IGKV1D-16_plasma cell
911	5.51E-139	-0.987229498	0.944	0.996	1.20E-134	RPL29	2	IGKV1D-16_plasma cell
912	3.86E-51	-0.988265766	0.615	0.724	8.42E-47	CALM2	2	IGKV1D-16_plasma cell
913	1.02E-135	-0.998160284	0.929	0.992	2.23E-131	RPL36	2	IGKV1D-16_plasma cell
914	7.18E-32	-1.003183143	0.173	0.425	1.57E-27	RNASET2	2	IGKV1D-16_plasma cell
915	1.36E-148	-1.006474321	0.971	0.997	2.97E-144	RPL19	2	IGKV1D-16_plasma cell
916	4.09E-149	-1.009634578	0.979	0.999	8.92E-145	RPL28	2	IGKV1D-16_plasma cell
917	1.23E-145	-1.015479424	0.973	0.996	2.69E-141	RPLP2	2	IGKV1D-16_plasma cell
918	7.05E-29	-1.016914795	0.154	0.397	1.54E-24	FOXP1	2	IGKV1D-16_plasma cell
919	1.12E-32	-1.01955189	0.327	0.546	2.45E-28	SP100	2	IGKV1D-16_plasma cell
920	9.53E-134	-1.034196842	0.923	0.989	2.08E-129	RPL17	2	IGKV1D-16_plasma cell

921	2.72E-34	-1.044729798	0.544	0.686	5.93E-30	DDIT4	2	IGKV1D-16_plasma cell
922	8.93E-117	-1.046746519	0.979	0.993	1.95E-112	ACTB	2	IGKV1D-16_plasma cell
923	2.56E-126	-1.057118758	0.917	0.983	5.58E-122	RPL10A	2	IGKV1D-16_plasma cell
924	2.72E-20	-1.062609167	0.11	0.302	5.93E-16	AREG	2	IGKV1D-16_plasma cell
925	1.85E-142	-1.069256774	0.931	0.994	4.03E-138	RPL35A	2	IGKV1D-16_plasma cell
926	6.98E-140	-1.070201998	0.977	0.998	1.52E-135	PTMA	2	IGKV1D-16_plasma cell
927	2.01E-143	-1.080861398	0.931	0.995	4.38E-139	RPS6	2	IGKV1D-16_plasma cell
928	2.70E-148	-1.081245092	0.956	0.992	5.89E-144	RPS10	2	IGKV1D-16_plasma cell
929	1.84E-125	-1.095225824	0.912	0.984	4.01E-121	RPL13A	2	IGKV1D-16_plasma cell
930	2.70E-134	-1.097150718	0.921	0.99	5.89E-130	RPL22	2	IGKV1D-16_plasma cell
931	9.54E-21	-1.104495928	0.515	0.615	2.08E-16	NR4A2	2	IGKV1D-16_plasma cell
932	2.87E-26	-1.11084431	0.135	0.366	6.26E-22	ID3	2	IGKV1D-16_plasma cell
933	6.12E-128	-1.113229371	0.877	0.978	1.33E-123	RPL9	2	IGKV1D-16_plasma cell
934	5.88E-40	-1.115150356	0.646	0.683	1.28E-35	TUBA1B	2	IGKV1D-16_plasma cell
935	6.15E-52	-1.117993087	0.148	0.513	1.34E-47	CCND3	2	IGKV1D-16_plasma cell
936	6.99E-153	-1.119797036	0.988	0.998	1.52E-148	RPL11	2	IGKV1D-16_plasma cell
937	2.57E-169	-1.121528685	0.994	0.999	5.60E-165	AC090498.1	2	IGKV1D-16_plasma cell
938	7.41E-143	-1.127407468	0.95	0.996	1.62E-138	RPL37	2	IGKV1D-16_plasma cell
939	3.29E-83	-1.129856675	0.733	0.862	7.16E-79	TOMM7	2	IGKV1D-16_plasma cell
940	1.40E-112	-1.132679936	0.831	0.948	3.06E-108	RPL38	2	IGKV1D-16_plasma cell
941	3.34E-60	-1.132809385	0.696	0.785	7.28E-56	RPS4Y1	2	IGKV1D-16_plasma cell
942	3.68E-148	-1.133718595	0.992	0.999	8.02E-144	EEF1A1	2	IGKV1D-16_plasma cell
943	5.11E-153	-1.137830523	0.962	0.998	1.11E-148	RPS21	2	IGKV1D-16_plasma cell
944	2.90E-141	-1.138508211	0.969	0.995	6.31E-137	RPL18A	2	IGKV1D-16_plasma cell
945	6.01E-56	-1.147092213	0.806	0.897	1.31E-51	ZFP36L2	2	IGKV1D-16_plasma cell
946	9.40E-41	-1.14892547	0.242	0.521	2.05E-36	IFITM2	2	IGKV1D-16_plasma cell
947	4.01E-46	-1.148951827	0.485	0.686	8.74E-42	PTPRC	2	IGKV1D-16_plasma cell
948	2.61E-20	-1.149082532	0.11	0.303	5.70E-16	HSPB1	2	IGKV1D-16_plasma cell
949	2.29E-53	-1.157660325	0.585	0.754	5.00E-49	GLTSCR2	2	IGKV1D-16_plasma cell
950	1.27E-116	-1.167000033	0.875	0.979	2.76E-112	RPS29	2	IGKV1D-16_plasma cell
951	8.46E-142	-1.18255422	0.938	0.989	1.85E-137	RPL21	2	IGKV1D-16_plasma cell
952	1.70E-136	-1.188270992	0.902	0.979	3.71E-132	RPSA	2	IGKV1D-16_plasma cell
953	5.54E-153	-1.197012105	0.985	0.998	1.21E-148	RPL13	2	IGKV1D-16_plasma cell
954	6.55E-126	-1.211054621	0.942	0.987	1.43E-121	FTH1	2	IGKV1D-16_plasma cell
955	2.31E-29	-1.216715032	0.177	0.413	5.04E-25	GPR183	2	IGKV1D-16_plasma cell
956	2.70E-65	-1.216827545	0.571	0.774	5.89E-61	COMMD6	2	IGKV1D-16_plasma cell
957	1.89E-82	-1.218080153	0.673	0.833	4.12E-78	RPS20	2	IGKV1D-16_plasma cell
958	1.00E-153	-1.222589818	0.944	0.997	2.19E-149	RPS3A	2	IGKV1D-16_plasma cell
959	8.43E-152	-1.23285682	0.919	0.995	1.84E-147	RPS25	2	IGKV1D-16_plasma cell
960	1.59E-153	-1.254781914	0.944	0.992	3.47E-149	RPL26	2	IGKV1D-16_plasma cell
961	1.34E-44	-1.275881807	0.152	0.469	2.93E-40	IRF8	2	IGKV1D-16_plasma cell
962	3.48E-115	-1.276084991	0.842	0.953	7.59E-111	PFDN5	2	IGKV1D-16_plasma cell
963	6.63E-43	-1.276308724	0.525	0.698	1.45E-38	CD55	2	IGKV1D-16_plasma cell
964	4.27E-156	-1.277504144	0.958	0.999	9.32E-152	RPS12	2	IGKV1D-16_plasma cell
965	1.32E-138	-1.291913081	0.967	0.997	2.87E-134	RPL34	2	IGKV1D-16_plasma cell
966	1.67E-73	-1.294784927	0.779	0.891	3.63E-69	TSC22D3	2	IGKV1D-16_plasma cell
967	1.70E-156	-1.30301307	0.917	0.995	3.70E-152	RPL12	2	IGKV1D-16_plasma cell
968	9.59E-162	-1.331264256	0.965	0.997	2.09E-157	RPS23	2	IGKV1D-16_plasma cell
969	1.29E-159	-1.345626496	0.969	0.995	2.82E-155	RPS27A	2	IGKV1D-16_plasma cell
970	2.38E-72	-1.366502004	0.485	0.762	5.18E-68	ZFAS1	2	IGKV1D-16_plasma cell
971	3.29E-150	-1.38530604	0.988	0.998	7.18E-146	RPS19	2	IGKV1D-16_plasma cell
972	3.63E-159	-1.413758909	0.95	0.999	7.91E-155	RPL32	2	IGKV1D-16_plasma cell
973	8.33E-64	-1.426398417	0.135	0.548	1.82E-59	FCMR	2	IGKV1D-16_plasma cell
974	1.78E-160	-1.481814515	0.958	0.997	3.89E-156	RPL30	2	IGKV1D-16_plasma cell
975	2.74E-149	-1.494249863	0.952	0.998	5.97E-145	RPL39	2	IGKV1D-16_plasma cell
976	1.60E-57	-1.516994841	0.25	0.598	3.48E-53	SLC2A3	2	IGKV1D-16_plasma cell
977	9.17E-71	-1.527780862	0.594	0.789	2.00E-66	HLA-DRB5	2	IGKV1D-16_plasma cell
978	7.04E-85	-1.529850619	0.925	0.955	1.53E-80	JUNB	2	IGKV1D-16_plasma cell
979	1.35E-140	-1.536722069	0.862	0.978	2.95E-136	TMSB4X	2	IGKV1D-16_plasma cell
980	3.05E-77	-1.652858414	0.296	0.691	6.65E-73	ZFP36L1	2	IGKV1D-16_plasma cell
981	1.53E-84	-1.670133239	0.625	0.858	3.33E-80	DUSP1	2	IGKV1D-16_plasma cell
982	1.03E-108	-1.719151105	0.654	0.882	2.25E-104	HLA-DPA1	2	IGKV1D-16_plasma cell
983	7.53E-116	-1.726147508	0.638	0.901	1.64E-111	LAPTM5	2	IGKV1D-16_plasma cell
984	3.37E-105	-1.751952413	0.669	0.901	7.35E-101	HLA-DQB1	2	IGKV1D-16_plasma cell
985	2.28E-151	-1.823278021	0.948	0.997	4.98E-147	RPS27	2	IGKV1D-16_plasma cell
986	2.79E-112	-1.890608918	0.467	0.85	6.08E-108	CD52	2	IGKV1D-16_plasma cell
987	7.67E-123	-1.90619621	0.977	0.999	1.67E-118	CD74	2	IGKV1D-16_plasma cell
988	1.02E-103	-1.925634665	0.638	0.9	2.22E-99	CXCR4	2	IGKV1D-16_plasma cell
989	1.05E-24	-1.983073742	0.317	0.11	2.30E-20	IGLV3-1	2	IGKV1D-16_plasma cell
990	5.07E-120	-2.052778591	0.338	0.819	1.11E-115	HLA-DQA1	2	IGKV1D-16_plasma cell
991	1.45E-108	-2.084269409	0.454	0.827	3.17E-104	HLA-DPB1	2	IGKV1D-16_plasma cell

992	1.86E-113	-2.111987245	0.708	0.905	4.07E-109	HLA-DRB1	2	IGKV1D-16_plasma cell
993	8.10E-105	-2.274135979	0.258	0.753	1.77E-100	TXNIP	2	IGKV1D-16_plasma cell
994	7.85E-129	-2.466759998	0.746	0.959	1.71E-124	BTG1	2	IGKV1D-16_plasma cell
995	1.47E-143	-2.555972913	0.44	0.9	3.21E-139	CD37	2	IGKV1D-16_plasma cell
996	2.14E-153	-3.252208497	0.421	0.914	4.67E-149	HLA-DRA	2	IGKV1D-16_plasma cell
997	7.08E-63	1.095257237	0.692	0.27	1.54E-58	GPR183	3	GPR183_B cell
998	8.84E-54	1.067968213	0.499	0.129	1.93E-49	TNF	3	GPR183_B cell
999	2.38E-49	0.943706912	0.692	0.39	5.19E-45	COTL1	3	GPR183_B cell
1000	1.03E-60	0.894579272	0.449	0.087	2.25E-56	AC079767.4	3	GPR183_B cell
1001	1.61E-40	0.887004139	0.786	0.542	3.50E-36	NR4A2	3	GPR183_B cell
1002	1.45E-33	0.87401537	0.299	0.08	3.17E-29	CD70	3	GPR183_B cell
1003	1.81E-50	0.8577743	0.44	0.107	3.94E-46	BCL2A1	3	GPR183_B cell
1004	8.09E-54	0.845377722	0.757	0.293	1.76E-49	LTB	3	GPR183_B cell
1005	2.56E-57	0.794944598	0.384	0.069	5.59E-53	CD82	3	GPR183_B cell
1006	2.30E-45	0.790738651	0.865	0.639	5.02E-41	KLF6	3	GPR183_B cell
1007	7.00E-20	0.788160339	0.437	0.208	1.53E-15	HSPB1	3	GPR183_B cell
1008	5.89E-52	0.777756771	0.537	0.153	1.28E-47	ARHGAP24	3	GPR183_B cell
1009	9.58E-57	0.773687966	0.877	0.364	2.09E-52	MS4A1	3	GPR183_B cell
1010	1.43E-51	0.741327396	0.918	0.637	3.13E-47	HLA-DQA1	3	GPR183_B cell
1011	3.50E-46	0.735830589	0.853	0.522	7.63E-42	ZFP36L1	3	GPR183_B cell
1012	4.96E-22	0.727240473	0.654	0.487	1.08E-17	DUSP2	3	GPR183_B cell
1013	1.31E-42	0.723371123	0.446	0.12	2.85E-38	CD24	3	GPR183_B cell
1014	1.33E-17	0.721631572	0.499	0.36	2.90E-13	CD44	3	GPR183_B cell
1015	1.43E-51	0.709735622	1	0.731	3.11E-47	HLA-DRA	3	GPR183_B cell
1016	5.27E-46	0.709444214	0.868	0.43	1.15E-41	CD69	3	GPR183_B cell
1017	8.33E-35	0.697246412	0.346	0.106	1.82E-30	CLECL1	3	GPR183_B cell
1018	4.61E-43	0.69379592	0.698	0.31	1.00E-38	BANK1	3	GPR183_B cell
1019	1.01E-48	0.693428736	0.845	0.384	2.19E-44	CD83	3	GPR183_B cell
1020	4.25E-22	0.692100326	0.393	0.179	9.27E-18	SMIM14	3	GPR183_B cell
1021	3.58E-17	0.678604271	0.525	0.362	7.81E-13	LRRFIP1	3	GPR183_B cell
1022	9.26E-19	0.675004069	0.411	0.229	2.02E-14	RILPL2	3	GPR183_B cell
1023	8.00E-25	0.662335707	0.795	0.75	1.75E-20	EZR	3	GPR183_B cell
1024	4.60E-07	0.662196556	0.331	0.235	0.010038952	EGR1	3	GPR183_B cell
1025	1.80E-63	0.659599308	0.988	0.943	3.92E-59	RPS29	3	GPR183_B cell
1026	2.44E-46	0.657437954	0.941	0.677	5.32E-42	HLA-DPB1	3	GPR183_B cell
1027	3.28E-51	0.656993525	0.965	0.81	7.15E-47	HLA-DQB1	3	GPR183_B cell
1028	1.01E-19	0.651639741	0.246	0.085	2.20E-15	RP5-887A10.	3	GPR183_B cell
1029	6.55E-20	0.647800432	0.686	0.639	1.43E-15	SYNGR2	3	GPR183_B cell
1030	3.13E-26	0.639112779	0.757	0.603	6.84E-22	PTPRC	3	GPR183_B cell
1031	4.12E-52	0.636717415	1	0.992	8.99E-48	CD74	3	GPR183_B cell
1032	2.56E-14	0.634366585	0.607	0.5	5.57E-10	FOSB	3	GPR183_B cell
1033	1.22E-77	0.633544469	1	0.986	2.65E-73	RPS12	3	GPR183_B cell
1034	1.22E-34	0.623176112	0.836	0.654	2.67E-30	ZFAS1	3	GPR183_B cell
1035	4.93E-13	0.621067166	0.399	0.254	1.07E-08	PMAIP1	3	GPR183_B cell
1036	1.66E-26	0.615347297	0.575	0.307	3.62E-22	RNASET2	3	GPR183_B cell
1037	6.73E-29	0.614058466	0.674	0.392	1.47E-24	IFITM2	3	GPR183_B cell
1038	8.87E-19	0.613244198	0.279	0.118	1.93E-14	AIM2	3	GPR183_B cell
1039	3.44E-46	0.612574832	0.971	0.825	7.50E-42	HLA-DRB1	3	GPR183_B cell
1040	5.69E-43	0.611475407	0.891	0.766	1.24E-38	RPS20	3	GPR183_B cell
1041	2.27E-41	0.609625284	0.883	0.815	4.95E-37	TOMM7	3	GPR183_B cell
1042	8.83E-49	0.608025216	0.997	0.981	1.93E-44	RPS27	3	GPR183_B cell
1043	5.84E-33	0.607296966	0.947	0.907	1.27E-28	ZFP36	3	GPR183_B cell
1044	3.00E-20	0.607072569	0.478	0.247	6.54E-16	ADAM28	3	GPR183_B cell
1045	1.54E-43	0.606110116	0.941	0.793	3.36E-39	HLA-DPA1	3	GPR183_B cell
1046	9.13E-14	0.600979887	0.519	0.427	1.99E-09	CDC42SE2	3	GPR183_B cell
1047	2.09E-15	0.60057961	0.519	0.348	4.56E-11	NR4A1	3	GPR183_B cell
1048	3.35E-76	0.595433657	1	0.997	7.30E-72	EEF1A1	3	GPR183_B cell
1049	1.59E-32	0.590019155	0.196	0.029	3.47E-28	CD1C	3	GPR183_B cell
1050	3.58E-32	0.586986038	0.639	0.304	7.81E-28	REL	3	GPR183_B cell
1051	7.85E-32	0.586579817	0.425	0.14	1.71E-27	BLK	3	GPR183_B cell
1052	4.72E-78	0.586105693	0.997	0.977	1.03E-73	RPS14	3	GPR183_B cell
1053	4.24E-20	0.579166131	0.672	0.537	9.24E-16	CD53	3	GPR183_B cell
1054	2.44E-50	0.577601167	0.962	0.914	5.32E-46	PFDN5	3	GPR183_B cell
1055	3.75E-12	0.575561079	0.446	0.28	8.17E-08	HSPA1A	3	GPR183_B cell
1056	7.06E-36	0.575245836	0.9	0.712	1.54E-31	CD52	3	GPR183_B cell
1057	1.70E-45	0.573159524	0.991	0.937	3.70E-41	TMSB4X	3	GPR183_B cell
1058	5.16E-17	0.571553948	0.252	0.093	1.12E-12	NR4A3	3	GPR183_B cell
1059	4.30E-18	0.5651494	0.293	0.12	9.38E-14	PIKFYVE	3	GPR183_B cell
1060	7.96E-19	0.564847535	0.422	0.216	1.74E-14	IFITM1	3	GPR183_B cell
1061	3.64E-13	0.562938405	0.352	0.198	7.93E-09	MARCKSL1	3	GPR183_B cell
1062	1.46E-13	0.562616768	0.839	0.827	3.19E-09	HSP90AA1	3	GPR183_B cell

1063	5.65E-21	0.561507698	0.757	0.702	1.23E-16	PPP1R15A	3	GPR183_B cell
1064	3.73E-15	0.55843918	0.519	0.348	8.13E-11	LY9	3	GPR183_B cell
1065	5.52E-49	0.555535851	1	0.983	1.20E-44	RPL39	3	GPR183_B cell
1066	5.46E-15	0.554096099	0.217	0.085	1.19E-10	TNFSF9	3	GPR183_B cell
1067	1.40E-67	0.550022616	0.997	0.995	3.05E-63	RPL11	3	GPR183_B cell
1068	1.55E-53	0.546448432	0.997	0.987	3.38E-49	RPL34	3	GPR183_B cell
1069	2.66E-33	0.545524267	0.862	0.707	5.79E-29	HLA-DRB5	3	GPR183_B cell
1070	8.33E-57	0.545045717	0.994	0.97	1.82E-52	RPS25	3	GPR183_B cell
1071	2.94E-08	0.538439119	0.273	0.177	0.000640463	MARCKS	3	GPR183_B cell
1072	1.17E-19	0.537235447	0.522	0.295	2.55E-15	SYPL1	3	GPR183_B cell
1073	8.06E-28	0.536567429	0.742	0.449	1.76E-23	SLC2A3	3	GPR183_B cell
1074	6.51E-43	0.534645792	1	0.987	1.42E-38	ACTB	3	GPR183_B cell
1075	2.67E-48	0.532985634	0.997	0.984	5.82E-44	RPL30	3	GPR183_B cell
1076	8.60E-37	0.531119832	0.909	0.847	1.88E-32	YWHAZ	3	GPR183_B cell
1077	1.01E-14	0.5286895	0.431	0.27	2.20E-10	EML4	3	GPR183_B cell
1078	4.90E-25	0.528376843	0.795	0.702	1.07E-20	COMMD6	3	GPR183_B cell
1079	5.14E-08	0.525049575	0.484	0.425	0.001120585	CMTM6	3	GPR183_B cell
1080	1.51E-31	0.521577546	0.372	0.106	3.30E-27	KIAA0226L	3	GPR183_B cell
1081	2.18E-06	0.521558613	0.399	0.328	0.047649457	HSPH1	3	GPR183_B cell
1082	5.37E-21	0.521036744	0.745	0.689	1.17E-16	MYL12A	3	GPR183_B cell
1083	2.33E-26	0.519937113	0.326	0.098	5.09E-22	TGIF1	3	GPR183_B cell
1084	2.23E-52	0.518805379	0.997	0.986	4.86E-48	RPS27A	3	GPR183_B cell
1085	2.71E-58	0.518500508	0.997	0.986	5.92E-54	RPS21	3	GPR183_B cell
1086	5.06E-17	0.517806778	0.264	0.103	1.10E-12	NFATC1	3	GPR183_B cell
1087	1.72E-69	0.512924044	0.994	0.99	3.75E-65	RPS15A	3	GPR183_B cell
1088	1.90E-11	0.512388335	0.545	0.486	4.13E-07	MYL12B	3	GPR183_B cell
1089	2.77E-46	0.511161131	0.979	0.902	6.03E-42	RPL38	3	GPR183_B cell
1090	4.68E-34	0.505803466	0.956	0.945	1.02E-29	JUNB	3	GPR183_B cell
1091	1.96E-25	0.504142769	0.317	0.097	4.28E-21	DEK	3	GPR183_B cell
1092	3.87E-28	0.499898229	0.249	0.055	8.44E-24	SCIMP	3	GPR183_B cell
1093	1.24E-49	0.499865003	0.997	0.986	2.71E-45	RPS23	3	GPR183_B cell
1094	6.15E-45	0.499812966	1	0.983	1.34E-40	RPL32	3	GPR183_B cell
1095	1.82E-06	0.499474376	0.455	0.427	0.039611329	ARL6IP5	3	GPR183_B cell
1096	1.73E-64	0.498086802	0.997	0.988	3.77E-60	RPS3	3	GPR183_B cell
1097	5.88E-27	0.494909958	0.9	0.862	1.28E-22	HSP90AB1	3	GPR183_B cell
1098	5.74E-14	0.494795861	0.525	0.378	1.25E-09	EVI2B	3	GPR183_B cell
1099	3.54E-44	0.494498435	0.982	0.944	7.73E-40	RPL9	3	GPR183_B cell
1100	1.30E-15	0.491457179	0.707	0.656	2.84E-11	FUS	3	GPR183_B cell
1101	9.09E-63	0.491219188	0.997	0.987	1.98E-58	RPS28	3	GPR183_B cell
1102	4.38E-46	0.49039448	0.988	0.959	9.54E-42	RPL13A	3	GPR183_B cell
1103	7.32E-12	0.487255401	0.452	0.269	1.60E-07	ID3	3	GPR183_B cell
1104	9.45E-56	0.480027804	1	0.988	2.06E-51	RPS18	3	GPR183_B cell
1105	3.27E-24	0.479830968	0.927	0.86	7.13E-20	ZFP36L2	3	GPR183_B cell
1106	5.09E-12	0.47728793	0.229	0.104	1.11E-07	PEA15	3	GPR183_B cell
1107	1.52E-58	0.476090164	0.997	0.998	3.32E-54	AC090498.1	3	GPR183_B cell
1108	2.12E-52	0.475899756	0.991	0.972	4.62E-48	RPL36	3	GPR183_B cell
1109	9.73E-14	0.475421303	0.232	0.097	2.12E-09	PVT1	3	GPR183_B cell
1110	3.13E-07	0.474243431	0.361	0.274	0.006834378	FNBP1	3	GPR183_B cell
1111	3.26E-10	0.47353315	0.66	0.68	7.11E-06	MEF2C	3	GPR183_B cell
1112	1.34E-32	0.472627257	0.921	0.85	2.93E-28	RPL36A	3	GPR183_B cell
1113	1.69E-06	0.472019252	0.27	0.185	0.036796112	RIC3	3	GPR183_B cell
1114	1.42E-29	0.471993322	0.886	0.85	3.10E-25	HLA-E	3	GPR183_B cell
1115	6.84E-23	0.471544451	0.328	0.11	1.49E-18	VPREB3	3	GPR183_B cell
1116	4.35E-57	0.467873789	0.997	0.972	9.48E-53	RPL14	3	GPR183_B cell
1117	6.07E-09	0.466661128	0.261	0.151	0.000132444	DDAH2	3	GPR183_B cell
1118	6.48E-25	0.466392272	0.308	0.09	1.41E-20	SPIB	3	GPR183_B cell
1119	1.17E-50	0.464933365	0.994	0.975	2.56E-46	RPS6	3	GPR183_B cell
1120	1.43E-69	0.46377884	1	0.997	3.11E-65	RPLP1	3	GPR183_B cell
1121	3.16E-55	0.463456189	0.994	0.989	6.89E-51	RPLP2	3	GPR183_B cell
1122	7.06E-13	0.462616313	0.457	0.295	1.54E-08	SWAP70	3	GPR183_B cell
1123	3.39E-15	0.462230839	0.328	0.153	7.40E-11	CYBB	3	GPR183_B cell
1124	2.63E-32	0.460039313	0.968	0.732	5.72E-28	CD37	3	GPR183_B cell
1125	1.14E-10	0.459966248	0.557	0.471	2.49E-06	GPSM3	3	GPR183_B cell
1126	3.16E-07	0.459295914	0.305	0.203	0.006894611	NFKBID	3	GPR183_B cell
1127	4.84E-09	0.458631177	0.68	0.674	0.000105567	TAGLN2	3	GPR183_B cell
1128	5.16E-11	0.454860218	0.748	0.757	1.13E-06	NFKBIA	3	GPR183_B cell
1129	8.63E-47	0.453592248	0.991	0.968	1.88E-42	RPL23A	3	GPR183_B cell
1130	5.04E-30	0.452245572	0.944	0.803	1.10E-25	CXCR4	3	GPR183_B cell
1131	2.78E-28	0.451661792	0.947	0.841	6.07E-24	TSC22D3	3	GPR183_B cell
1132	1.91E-09	0.449461369	0.528	0.448	4.17E-05	UBXN1	3	GPR183_B cell
1133	3.99E-15	0.448421287	0.413	0.224	8.70E-11	CD40	3	GPR183_B cell

1134	7.27E-15	0.446658677	0.437	0.242	1.58E-10	FCRLA	3	GPR183_B cell
1135	2.23E-09	0.445762591	0.616	0.607	4.87E-05	EIF4A2	3	GPR183_B cell
1136	4.35E-11	0.445727114	0.625	0.578	9.49E-07	PPDPF	3	GPR183_B cell
1137	4.33E-53	0.445600273	1	0.992	9.44E-49	RPL28	3	GPR183_B cell
1138	1.93E-19	0.445585854	0.352	0.133	4.21E-15	CXCR5	3	GPR183_B cell
1139	1.20E-07	0.444782386	0.44	0.345	0.002620524	MAP3K8	3	GPR183_B cell
1140	5.60E-20	0.442359595	0.323	0.113	1.22E-15	CCR7	3	GPR183_B cell
1141	3.45E-09	0.442255258	0.545	0.489	7.52E-05	PTPN6	3	GPR183_B cell
1142	1.60E-07	0.442224228	0.37	0.251	0.003496096	ZNF331	3	GPR183_B cell
1143	2.03E-27	0.441708213	0.906	0.869	4.43E-23	DDX5	3	GPR183_B cell
1144	1.88E-08	0.441646861	0.56	0.528	0.000409473	WSB1	3	GPR183_B cell
1145	3.63E-15	0.440160001	0.314	0.142	7.91E-11	PARP14	3	GPR183_B cell
1146	3.03E-10	0.439294886	0.584	0.556	6.61E-06	TPM3	3	GPR183_B cell
1147	1.58E-17	0.439024666	0.748	0.682	3.44E-13	CALM2	3	GPR183_B cell
1148	9.34E-47	0.434057912	0.997	0.981	2.04E-42	RPL3	3	GPR183_B cell
1149	5.72E-19	0.429656053	0.346	0.133	1.25E-14	PARP15	3	GPR183_B cell
1150	1.62E-08	0.428962178	0.255	0.146	0.000354376	RAB11FIP1	3	GPR183_B cell
1151	2.40E-44	0.428091719	0.988	0.975	5.22E-40	RPL35A	3	GPR183_B cell
1152	3.74E-26	0.42718356	0.88	0.849	8.16E-22	RPL27A	3	GPR183_B cell
1153	1.06E-43	0.426443486	1	1	2.31E-39	MALAT1	3	GPR183_B cell
1154	6.79E-22	0.425460814	0.613	0.331	1.48E-17	IRF8	3	GPR183_B cell
1155	4.17E-11	0.425075294	0.63	0.601	9.10E-07	DAZAP2	3	GPR183_B cell
1156	1.21E-38	0.423896849	1	0.979	2.64E-34	RPS3A	3	GPR183_B cell
1157	2.82E-21	0.423194165	0.452	0.183	6.15E-17	1-Mar	3	GPR183_B cell
1158	2.09E-39	0.422909444	0.994	0.976	4.57E-35	RPL26	3	GPR183_B cell
1159	2.15E-08	0.422728369	0.326	0.213	0.000469527	PRDM2	3	GPR183_B cell
1160	1.28E-07	0.421598489	0.519	0.469	0.002795003	LCP1	3	GPR183_B cell
1161	3.25E-39	0.421236545	0.988	0.967	7.08E-35	RPL17	3	GPR183_B cell
1162	3.90E-52	0.421166544	0.997	0.994	8.51E-48	RPS8	3	GPR183_B cell
1163	2.18E-15	0.420549776	0.255	0.1	4.75E-11	MBP	3	GPR183_B cell
1164	1.47E-15	0.417016074	0.777	0.801	3.20E-11	PTPRCAP	3	GPR183_B cell
1165	1.57E-15	0.41577403	0.751	0.624	3.43E-11	DDIT4	3	GPR183_B cell
1166	1.73E-11	0.414687566	0.628	0.551	3.77E-07	HLA-DMA	3	GPR183_B cell
1167	4.60E-40	0.413091851	0.997	0.981	1.00E-35	RPL37	3	GPR183_B cell
1168	1.16E-08	0.412872183	0.457	0.343	0.000253161	DRAM2	3	GPR183_B cell
1169	4.62E-26	0.411780168	0.974	0.942	1.01E-21	H3F3B	3	GPR183_B cell
1170	2.55E-45	0.41098485	1	0.988	5.56E-41	FAU	3	GPR183_B cell
1171	7.61E-10	0.408935969	0.531	0.408	1.66E-05	TNFAIP8	3	GPR183_B cell
1172	1.12E-49	0.408897878	0.997	0.984	2.44E-45	TPT1	3	GPR183_B cell
1173	1.12E-12	0.408368316	0.551	0.396	2.44E-08	CRIP1	3	GPR183_B cell
1174	1.02E-45	0.407872222	0.994	0.989	2.22E-41	RPL19	3	GPR183_B cell
1175	5.68E-14	0.407157382	0.66	0.57	1.24E-09	CD48	3	GPR183_B cell
1176	2.10E-32	0.405936438	0.982	0.962	4.58E-28	EEF1B2	3	GPR183_B cell
1177	8.20E-17	0.403121506	0.466	0.23	1.79E-12	TUBA1A	3	GPR183_B cell
1178	3.25E-19	0.39854699	0.786	0.691	7.09E-15	GLTSCR2	3	GPR183_B cell
1179	3.91E-40	0.398521456	0.994	0.98	8.52E-36	RPS10	3	GPR183_B cell
1180	1.17E-21	0.398083725	0.906	0.87	2.54E-17	PFN1	3	GPR183_B cell
1181	3.06E-24	0.396996467	0.232	0.056	6.66E-20	BCAS4	3	GPR183_B cell
1182	4.25E-34	0.395849622	0.997	0.994	9.27E-30	RPL13	3	GPR183_B cell
1183	1.66E-07	0.395792552	0.135	0.059	0.003625321	KLK1	3	GPR183_B cell
1184	2.74E-11	0.395011199	0.141	0.045	5.97E-07	LDLRAD4	3	GPR183_B cell
1185	1.16E-38	0.394672062	0.994	0.966	2.54E-34	RPL22	3	GPR183_B cell
1186	1.31E-31	0.391675125	0.968	0.928	2.87E-27	RPL4	3	GPR183_B cell
1187	7.03E-36	0.391583816	0.985	0.964	1.53E-31	NACA	3	GPR183_B cell
1188	1.10E-20	0.390355936	0.812	0.749	2.39E-16	RPS4Y1	3	GPR183_B cell
1189	1.02E-32	0.385455657	0.988	0.952	2.23E-28	RPSA	3	GPR183_B cell
1190	1.81E-09	0.383590872	0.566	0.469	3.95E-05	SP100	3	GPR183_B cell
1191	1.65E-43	0.383012626	1	0.997	3.61E-39	RPL10	3	GPR183_B cell
1192	2.10E-11	0.382439581	0.677	0.656	4.58E-07	SF1	3	GPR183_B cell
1193	9.51E-24	0.381499332	0.962	0.948	2.07E-19	MT-ND2	3	GPR183_B cell
1194	1.58E-17	0.380971611	0.361	0.148	3.45E-13	LY86	3	GPR183_B cell
1195	3.99E-17	0.380620136	0.387	0.17	8.71E-13	FAM65B	3	GPR183_B cell
1196	7.47E-22	0.379412759	0.903	0.77	1.63E-17	DUSP1	3	GPR183_B cell
1197	1.61E-33	0.37830649	0.152	0.012	3.51E-29	MCOLN2	3	GPR183_B cell
1198	1.08E-09	0.377294346	0.66	0.682	2.35E-05	SMDT1	3	GPR183_B cell
1199	1.31E-28	0.377134308	0.991	0.881	2.85E-24	BTG1	3	GPR183_B cell
1200	1.36E-06	0.376973069	0.519	0.491	0.029767178	ST13	3	GPR183_B cell
1201	9.85E-28	0.376428284	0.956	0.801	2.15E-23	LAPTM5	3	GPR183_B cell
1202	4.77E-18	0.376054227	0.232	0.07	1.04E-13	TRAF5	3	GPR183_B cell
1203	3.09E-18	0.375551597	0.229	0.069	6.75E-14	SESN3	3	GPR183_B cell
1204	9.81E-11	0.374512195	0.173	0.067	2.14E-06	BBC3	3	GPR183_B cell

1205	1.31E-07	0.373907493	0.672	0.71	0.002863507	RAC2	3	GPR183_B cell
1206	9.43E-28	0.372977073	0.927	0.88	2.06E-23	RPL31	3	GPR183_B cell
1207	6.34E-10	0.371805416	0.387	0.245	1.38E-05	SP110	3	GPR183_B cell
1208	6.44E-39	0.371247252	1	0.977	1.41E-34	RPS13	3	GPR183_B cell
1209	4.47E-15	0.369621678	0.774	0.757	9.75E-11	PNRC1	3	GPR183_B cell
1210	1.28E-11	0.369424066	0.721	0.676	2.79E-07	ARHGDIB	3	GPR183_B cell
1211	7.09E-08	0.368273589	0.534	0.482	0.001545677	NAP1L1	3	GPR183_B cell
1212	2.40E-23	0.367272813	0.915	0.909	5.24E-19	PABPC1	3	GPR183_B cell
1213	9.67E-14	0.366973585	0.155	0.047	2.11E-09	RAB31	3	GPR183_B cell
1214	7.22E-36	0.365523119	0.991	0.98	1.57E-31	RPS16	3	GPR183_B cell
1215	8.24E-10	0.365261677	0.22	0.104	1.80E-05	CAPG	3	GPR183_B cell
1216	1.59E-16	0.364880584	0.229	0.077	3.47E-12	ALOX5	3	GPR183_B cell
1217	2.78E-38	0.363473257	0.994	0.992	6.06E-34	RPS15	3	GPR183_B cell
1218	5.57E-07	0.358885618	0.572	0.588	0.012144128	GPX4	3	GPR183_B cell
1219	1.17E-08	0.3584059	0.478	0.359	0.000255348	SELL	3	GPR183_B cell
1220	1.92E-31	0.35713524	0.988	0.969	4.19E-27	RPL5	3	GPR183_B cell
1221	5.53E-31	0.357120212	0.991	0.972	1.21E-26	RPL21	3	GPR183_B cell
1222	3.76E-31	0.355433218	0.994	0.955	8.21E-27	RPS11	3	GPR183_B cell
1223	2.11E-07	0.355143503	0.63	0.665	0.004601807	RSRP1	3	GPR183_B cell
1224	2.78E-12	0.353837209	0.323	0.158	6.06E-08	SNX9	3	GPR183_B cell
1225	8.37E-07	0.353416024	0.44	0.344	0.018244072	ANKRD44	3	GPR183_B cell
1226	1.14E-07	0.35312038	0.238	0.132	0.002483539	PTPN22	3	GPR183_B cell
1227	1.01E-29	0.352817215	0.994	0.974	2.20E-25	EIF1	3	GPR183_B cell
1228	1.54E-29	0.352361752	0.997	0.969	3.35E-25	RPL12	3	GPR183_B cell
1229	2.47E-09	0.352343624	0.686	0.656	5.39E-05	CORO1A	3	GPR183_B cell
1230	7.30E-17	0.351725438	0.155	0.038	1.59E-12	BHLHE40	3	GPR183_B cell
1231	6.64E-17	0.351678521	0.167	0.043	1.45E-12	MNDA	3	GPR183_B cell
1232	7.90E-15	0.350863461	0.903	0.893	1.72E-10	IER2	3	GPR183_B cell
1233	3.12E-15	0.349526344	0.76	0.761	6.80E-11	CIRBP	3	GPR183_B cell
1234	3.64E-32	0.349097765	0.988	0.968	7.94E-28	RPS9	3	GPR183_B cell
1235	1.95E-21	0.347333001	0.144	0.023	4.26E-17	SIGLEC10	3	GPR183_B cell
1236	3.59E-25	0.347306493	0.982	0.972	7.83E-21	MT-ND4	3	GPR183_B cell
1237	5.32E-20	0.343585402	0.889	0.839	1.16E-15	BTF3	3	GPR183_B cell
1238	2.53E-14	0.343345616	0.903	0.827	5.52E-10	ACTG1	3	GPR183_B cell
1239	1.31E-29	0.342848969	0.988	0.959	2.85E-25	RPL10A	3	GPR183_B cell
1240	1.61E-07	0.341599329	0.622	0.624	0.003501817	HNRNPC	3	GPR183_B cell
1241	3.13E-14	0.341483824	0.865	0.837	6.82E-10	HSPA8	3	GPR183_B cell
1242	6.32E-18	0.34004633	0.821	0.778	1.38E-13	CCNI	3	GPR183_B cell
1243	1.52E-13	0.33962217	0.264	0.109	3.31E-09	BIN1	3	GPR183_B cell
1244	8.46E-32	0.338082382	0.988	0.968	1.84E-27	RPL6	3	GPR183_B cell
1245	7.05E-18	0.335337628	0.85	0.819	1.54E-13	PCBP2	3	GPR183_B cell
1246	1.78E-24	0.335240639	0.95	0.911	3.88E-20	RPL27	3	GPR183_B cell
1247	4.70E-22	0.333667078	0.95	0.946	1.02E-17	EEF2	3	GPR183_B cell
1248	5.05E-08	0.333011585	0.361	0.225	0.001101304	AREG	3	GPR183_B cell
1249	5.05E-31	0.33113754	0.994	0.979	1.10E-26	RPS7	3	GPR183_B cell
1250	2.70E-27	0.3285211	0.991	0.975	5.88E-23	MT-ND1	3	GPR183_B cell
1251	4.63E-09	0.327618265	0.666	0.644	0.000100955	PTGES3	3	GPR183_B cell
1252	1.37E-13	0.32428513	0.93	0.916	2.98E-09	UBC	3	GPR183_B cell
1253	1.24E-30	0.323212919	0.997	0.979	2.71E-26	RPL29	3	GPR183_B cell
1254	2.22E-07	0.322226592	0.238	0.133	0.004851112	INPP5D	3	GPR183_B cell
1255	2.08E-09	0.322191823	0.173	0.073	4.53E-05	TLR10	3	GPR183_B cell
1256	1.01E-11	0.320363477	0.783	0.798	2.20E-07	RPL23	3	GPR183_B cell
1257	1.89E-07	0.318160292	0.61	0.626	0.00411139	EIF3H	3	GPR183_B cell
1258	2.44E-11	0.317754805	0.246	0.111	5.32E-07	BCL2	3	GPR183_B cell
1259	2.88E-26	0.313375027	0.994	0.979	6.28E-22	HLA-B	3	GPR183_B cell
1260	4.05E-27	0.311499413	0.997	0.995	8.83E-23	RPS19	3	GPR183_B cell
1261	5.17E-11	0.310255376	0.284	0.138	1.13E-06	ALOX5AP	3	GPR183_B cell
1262	1.11E-12	0.308205105	0.862	0.843	2.42E-08	HINT1	3	GPR183_B cell
1263	6.36E-10	0.308130217	0.739	0.749	1.39E-05	UQCRB	3	GPR183_B cell
1264	6.73E-27	0.305238324	1	0.996	1.47E-22	MT-ND3	3	GPR183_B cell
1265	4.73E-30	0.304986714	1	0.99	1.03E-25	RPL18	3	GPR183_B cell
1266	7.00E-23	0.304036468	0.988	0.97	1.53E-18	RPS2	3	GPR183_B cell
1267	6.12E-10	0.303633535	0.701	0.707	1.33E-05	EIF3F	3	GPR183_B cell
1268	2.22E-25	0.301387731	0.979	0.953	4.84E-21	UBA52	3	GPR183_B cell
1269	6.97E-30	0.300046229	0.997	0.985	1.52E-25	RPS4X	3	GPR183_B cell
1270	5.38E-13	0.299389928	0.771	0.753	1.17E-08	EIF3E	3	GPR183_B cell
1271	2.46E-09	0.298087451	0.346	0.194	5.37E-05	TRBC2	3	GPR183_B cell
1272	2.61E-19	0.29727449	0.956	0.912	5.70E-15	RPL7	3	GPR183_B cell
1273	2.86E-17	0.295355476	0.138	0.028	6.24E-13	LRRK2	3	GPR183_B cell
1274	2.84E-21	0.295234595	0.974	0.946	6.20E-17	RPL41	3	GPR183_B cell
1275	3.63E-09	0.294589243	0.37	0.213	7.91E-05	EVL	3	GPR183_B cell

1276	4.23E-08	0.294223985	0.727	0.755	0.000923255	TMA7	3	GPR183_B cell
1277	4.05E-12	0.292996266	0.39	0.192	8.82E-08	TRIM22	3	GPR183_B cell
1278	4.27E-26	0.29113243	0.123	0.011	9.31E-22	TFEC	3	GPR183_B cell
1279	3.50E-10	0.288302842	0.698	0.641	7.62E-06	CD55	3	GPR183_B cell
1280	2.59E-18	0.287468018	0.83	0.572	5.66E-14	TXNIP	3	GPR183_B cell
1281	8.97E-28	0.285616085	0.982	0.973	1.96E-23	FTH1	3	GPR183_B cell
1282	4.24E-10	0.285150913	0.199	0.084	9.25E-06	STAT6	3	GPR183_B cell
1283	9.09E-12	0.283587304	0.818	0.812	1.98E-07	ATP5G2	3	GPR183_B cell
1284	2.06E-06	0.283567634	0.683	0.701	0.04487605	ARPC2	3	GPR183_B cell
1285	5.17E-07	0.283448552	0.132	0.057	0.011270288	HAGHL	3	GPR183_B cell
1286	1.02E-11	0.282824935	0.774	0.788	2.23E-07	ARPC3	3	GPR183_B cell
1287	6.87E-08	0.282804134	0.196	0.096	0.001498192	PDE7A	3	GPR183_B cell
1288	1.04E-07	0.282556646	0.636	0.655	0.002267961	EIF4B	3	GPR183_B cell
1289	7.71E-11	0.281298975	0.416	0.225	1.68E-06	LBH	3	GPR183_B cell
1290	6.54E-22	0.279007251	0.95	0.946	1.43E-17	EEF1D	3	GPR183_B cell
1291	2.54E-16	0.278006501	0.152	0.036	5.53E-12	KYNU	3	GPR183_B cell
1292	1.04E-09	0.276424183	0.229	0.102	2.27E-05	ZEB2	3	GPR183_B cell
1293	3.17E-13	0.274499617	0.883	0.843	6.92E-09	NPM1	3	GPR183_B cell
1294	3.13E-11	0.274454356	0.853	0.831	6.84E-07	CFL1	3	GPR183_B cell
1295	2.08E-10	0.273972441	0.232	0.101	4.54E-06	PAX5	3	GPR183_B cell
1296	3.31E-13	0.272549703	0.877	0.898	7.22E-09	COX4I1	3	GPR183_B cell
1297	4.76E-16	0.271533008	0.941	0.891	1.04E-11	EEF1G	3	GPR183_B cell
1298	2.30E-21	0.270757934	0.982	0.975	5.01E-17	RPL37A	3	GPR183_B cell
1299	2.43E-07	0.26818957	0.545	0.412	0.005294508	FCMR	3	GPR183_B cell
1300	5.18E-07	0.267504229	0.683	0.704	0.011300132	SUMO2	3	GPR183_B cell
1301	1.69E-22	0.267012855	0.985	0.97	3.70E-18	GNB2L1	3	GPR183_B cell
1302	1.33E-11	0.264600665	0.842	0.84	2.89E-07	COX7C	3	GPR183_B cell
1303	6.96E-25	0.262747638	0.994	0.993	1.52E-20	RPL8	3	GPR183_B cell
1304	1.86E-09	0.262065599	0.305	0.156	4.05E-05	HLA-DMB	3	GPR183_B cell
1305	3.77E-08	0.261358442	0.323	0.191	0.000821174	UVRAG	3	GPR183_B cell
1306	2.47E-08	0.259372344	0.161	0.068	0.000538062	AIM1	3	GPR183_B cell
1307	6.76E-10	0.25870495	0.83	0.793	1.47E-05	SLC25A6	3	GPR183_B cell
1308	6.40E-09	0.255337518	0.109	0.034	0.000139529	ADRBK2	3	GPR183_B cell
1309	9.97E-20	0.252633692	0.979	0.961	2.17E-15	RPL24	3	GPR183_B cell
1310	5.06E-09	0.252478613	0.258	0.126	0.000110297	PKIG	3	GPR183_B cell
1311	2.73E-22	-0.25092441	0.114	0.438	5.95E-18	COPB2	3	GPR183_B cell
1312	1.90E-19	-0.252299111	0.126	0.428	4.15E-15	NDUFC1	3	GPR183_B cell
1313	3.68E-13	-0.253542326	0.24	0.515	8.02E-09	ATP5G1	3	GPR183_B cell
1314	2.09E-15	-0.265401156	0.276	0.58	4.55E-11	PRDX5	3	GPR183_B cell
1315	4.25E-13	-0.267017018	0.284	0.567	9.26E-09	PSMB1	3	GPR183_B cell
1316	2.48E-22	-0.269299008	0.141	0.487	5.41E-18	LRPAP1	3	GPR183_B cell
1317	3.78E-17	-0.269834665	0.191	0.5	8.25E-13	SRSF9	3	GPR183_B cell
1318	3.20E-10	-0.274714383	0.499	0.676	6.97E-06	COX7A2	3	GPR183_B cell
1319	1.02E-25	-0.275381983	0.106	0.456	2.23E-21	ALG5	3	GPR183_B cell
1320	1.66E-16	-0.276156612	0.22	0.533	3.62E-12	CDK2AP2	3	GPR183_B cell
1321	1.98E-17	-0.279384293	0.179	0.484	4.32E-13	PPA1	3	GPR183_B cell
1322	3.28E-24	-0.28192465	0.126	0.474	7.16E-20	MGAT1	3	GPR183_B cell
1323	2.95E-21	-0.282404544	0.123	0.439	6.42E-17	GLRX	3	GPR183_B cell
1324	7.61E-17	-0.287506093	0.188	0.481	1.66E-12	ANAPC11	3	GPR183_B cell
1325	1.91E-23	-0.288112188	0.135	0.481	4.16E-19	SEC14L1	3	GPR183_B cell
1326	7.95E-13	-0.290562685	0.326	0.586	1.73E-08	ATP5J	3	GPR183_B cell
1327	5.00E-22	-0.291517843	0.135	0.467	1.09E-17	CISD2	3	GPR183_B cell
1328	5.09E-19	-0.292001939	0.214	0.559	1.11E-14	REEP5	3	GPR183_B cell
1329	1.90E-20	-0.293182399	0.158	0.484	4.15E-16	NDUFB7	3	GPR183_B cell
1330	3.01E-17	-0.301533293	0.22	0.544	6.57E-13	SRPRA	3	GPR183_B cell
1331	1.83E-06	-0.302175111	0.17	0.303	0.039881858	HMGB2	3	GPR183_B cell
1332	6.33E-19	-0.306021464	0.182	0.496	1.38E-14	NDUFB6	3	GPR183_B cell
1333	5.65E-22	-0.306631191	0.173	0.527	1.23E-17	DNAJB9	3	GPR183_B cell
1334	5.64E-21	-0.310229694	0.141	0.458	1.23E-16	CYC1	3	GPR183_B cell
1335	5.93E-08	-0.31353701	0.619	0.735	0.001293538	CHCHD2	3	GPR183_B cell
1336	3.36E-15	-0.315159624	0.326	0.622	7.32E-11	SELK	3	GPR183_B cell
1337	1.83E-11	-0.320263992	0.534	0.724	3.99E-07	SSR2	3	GPR183_B cell
1338	2.09E-23	-0.328122281	0.141	0.483	4.57E-19	MRPS24	3	GPR183_B cell
1339	9.60E-13	-0.328860888	0.513	0.7	2.09E-08	TMBIM6	3	GPR183_B cell
1340	3.28E-14	-0.32951468	0.361	0.637	7.15E-10	PDIA3	3	GPR183_B cell
1341	8.76E-15	-0.330027999	0.32	0.604	1.91E-10	ARF4	3	GPR183_B cell
1342	1.96E-09	-0.333632125	0.754	0.823	4.27E-05	RPS26	3	GPR183_B cell
1343	9.93E-15	-0.334505931	0.375	0.638	2.16E-10	KRTCAP2	3	GPR183_B cell
1344	5.81E-10	-0.335573989	0.54	0.689	1.27E-05	NME2	3	GPR183_B cell
1345	4.05E-16	-0.338162633	0.317	0.622	8.83E-12	POLR2L	3	GPR183_B cell
1346	2.26E-22	-0.338178043	0.208	0.573	4.94E-18	UFM1	3	GPR183_B cell

1347	8.38E-13	-0.342028565	0.39	0.634	1.83E-08	POU2AF1	3	GPR183_B cell
1348	5.43E-12	-0.367730042	0.355	0.602	1.18E-07	PIM2	3	GPR183_B cell
1349	1.30E-09	-0.375778808	0.677	0.753	2.83E-05	MIF	3	GPR183_B cell
1350	1.43E-11	-0.393195963	0.469	0.654	3.11E-07	MTDH	3	GPR183_B cell
1351	5.87E-17	-0.394072534	0.317	0.597	1.28E-12	ATP5A1	3	GPR183_B cell
1352	2.50E-20	-0.409066949	0.252	0.586	5.44E-16	ROMO1	3	GPR183_B cell
1353	5.55E-22	-0.414148776	0.235	0.575	1.21E-17	ERGIC3	3	GPR183_B cell
1354	8.25E-20	-0.420705806	0.246	0.564	1.80E-15	COX5A	3	GPR183_B cell
1355	7.12E-20	-0.429418922	0.188	0.497	1.55E-15	TXN	3	GPR183_B cell
1356	5.56E-26	-0.431409691	0.144	0.501	1.21E-21	DNAJB11	3	GPR183_B cell
1357	1.20E-28	-0.439188705	0.106	0.468	2.62E-24	LRRC59	3	GPR183_B cell
1358	3.45E-16	-0.441616207	0.425	0.63	7.53E-12	SPCS1	3	GPR183_B cell
1359	2.18E-18	-0.443543621	0.109	0.376	4.75E-14	CCND2	3	GPR183_B cell
1360	3.36E-14	-0.444678663	0.372	0.607	7.33E-10	ATP5G3	3	GPR183_B cell
1361	8.63E-21	-0.459554025	0.343	0.628	1.88E-16	DAD1	3	GPR183_B cell
1362	7.80E-26	-0.466122298	0.24	0.629	1.70E-21	FAM46C	3	GPR183_B cell
1363	9.67E-08	-0.469835927	0.109	0.243	0.00210917	CLEC2B	3	GPR183_B cell
1364	1.31E-17	-0.48092137	0.267	0.549	2.85E-13	PSME2	3	GPR183_B cell
1365	1.89E-28	-0.48208941	0.167	0.542	4.11E-24	DERL1	3	GPR183_B cell
1366	4.47E-27	-0.495884675	0.199	0.568	9.74E-23	CANX	3	GPR183_B cell
1367	1.27E-34	-0.497411062	0.1	0.504	2.76E-30	TXNDC11	3	GPR183_B cell
1368	2.67E-23	-0.499607068	0.267	0.583	5.81E-19	TMED2	3	GPR183_B cell
1369	2.20E-25	-0.50462574	0.208	0.56	4.80E-21	SSR1	3	GPR183_B cell
1370	1.60E-30	-0.509134121	0.132	0.528	3.48E-26	ELL2	3	GPR183_B cell
1371	7.81E-24	-0.521412711	0.252	0.576	1.70E-19	UQCRQ	3	GPR183_B cell
1372	5.54E-25	-0.5246997	0.211	0.555	1.21E-20	GSTP1	3	GPR183_B cell
1373	6.10E-28	-0.539774023	0.252	0.609	1.33E-23	TRAM1	3	GPR183_B cell
1374	1.82E-27	-0.549921354	0.214	0.566	3.97E-23	KDELR1	3	GPR183_B cell
1375	8.73E-26	-0.55865588	0.29	0.616	1.90E-21	COPE	3	GPR183_B cell
1376	5.30E-29	-0.561164986	0.519	0.782	1.16E-24	ISG20	3	GPR183_B cell
1377	1.46E-19	-0.587138967	0.493	0.693	3.18E-15	CALR	3	GPR183_B cell
1378	1.25E-28	-0.611422622	0.243	0.597	2.73E-24	TMED10	3	GPR183_B cell
1379	1.43E-26	-0.615206918	0.399	0.663	3.12E-22	TMEM258	3	GPR183_B cell
1380	1.94E-32	-0.621063153	0.179	0.571	4.23E-28	TMED9	3	GPR183_B cell
1381	1.84E-32	-0.64949675	0.191	0.567	4.02E-28	DDOST	3	GPR183_B cell
1382	1.37E-42	-0.66037494	0.123	0.587	2.99E-38	PLPP5	3	GPR183_B cell
1383	1.92E-36	-0.675086255	0.129	0.541	4.20E-32	ERLEC1	3	GPR183_B cell
1384	3.17E-40	-0.694712718	0.117	0.557	6.91E-36	FKBP2	3	GPR183_B cell
1385	2.58E-36	-0.694722921	0.173	0.566	5.63E-32	LMAN2	3	GPR183_B cell
1386	9.18E-35	-0.702918245	0.378	0.678	2.00E-30	SPCS2	3	GPR183_B cell
1387	9.50E-40	-0.708937934	0.114	0.537	2.07E-35	VIMP	3	GPR183_B cell
1388	4.15E-37	-0.709468215	0.305	0.667	9.05E-33	RABAC1	3	GPR183_B cell
1389	2.16E-35	-0.756062171	0.202	0.576	4.71E-31	OSTC	3	GPR183_B cell
1390	1.72E-40	-0.757280563	0.109	0.536	3.74E-36	MANF	3	GPR183_B cell
1391	2.05E-29	-0.784852813	0.408	0.659	4.47E-25	SEC61B	3	GPR183_B cell
1392	2.31E-31	-0.786106061	0.293	0.619	5.04E-27	UBE2J1	3	GPR183_B cell
1393	1.07E-38	-0.796979302	0.176	0.575	2.33E-34	RPN1	3	GPR183_B cell
1394	2.09E-34	-0.804617384	0.211	0.566	4.56E-30	RPN2	3	GPR183_B cell
1395	1.20E-28	-0.873719496	0.592	0.751	2.62E-24	SUB1	3	GPR183_B cell
1396	1.11E-44	-0.887836442	0.135	0.565	2.43E-40	HM13	3	GPR183_B cell
1397	6.98E-40	-0.906701235	0.173	0.562	1.52E-35	KDELR2	3	GPR183_B cell
1398	2.63E-37	-0.920281427	0.326	0.646	5.73E-33	SEC61G	3	GPR183_B cell
1399	1.09E-34	-0.930962391	0.27	0.598	2.37E-30	PDIA6	3	GPR183_B cell
1400	2.56E-38	-0.972976825	0.226	0.583	5.58E-34	ITM2C	3	GPR183_B cell
1401	1.55E-47	-1.049496971	0.126	0.566	3.37E-43	SPCS3	3	GPR183_B cell
1402	9.98E-50	-1.075885795	0.135	0.577	2.18E-45	LMAN1	3	GPR183_B cell
1403	3.07E-50	-1.174318723	0.167	0.59	6.68E-46	P4HB	3	GPR183_B cell
1404	2.42E-41	-1.185053829	0.381	0.67	5.28E-37	HSPA5	3	GPR183_B cell
1405	1.46E-40	-1.204617387	0.24	0.582	3.19E-36	SEC11C	3	GPR183_B cell
1406	1.41E-52	-1.307564424	0.152	0.586	3.08E-48	SSR3	3	GPR183_B cell
1407	3.48E-53	-1.453909303	0.431	0.712	7.58E-49	SSR4	3	GPR183_B cell
1408	4.21E-53	-1.497816923	0.114	0.564	9.18E-49	MYDGF	3	GPR183_B cell
1409	1.91E-50	-1.85162724	0.428	0.702	4.17E-46	PPIB	3	GPR183_B cell
1410	3.66E-58	-1.857637446	0.132	0.589	7.97E-54	XBP1	3	GPR183_B cell
1411	1.62E-46	-1.944662925	0.501	0.707	3.54E-42	HSP90B1	3	GPR183_B cell
1412	3.13E-62	-2.331669996	0.147	0.608	6.83E-58	MZB1	3	GPR183_B cell
1413	1.28E-19	-2.644616696	0.487	0.596	2.80E-15	IGKC	3	GPR183_B cell
1414	1.23E-13	-2.767347682	0.109	0.325	2.69E-09	IGKV1-12	3	GPR183_B cell
1415	6.62E-39	-2.91448055	0.457	0.672	1.44E-34	JCHAIN	3	GPR183_B cell
1416	2.80E-16	-3.136788905	0.188	0.41	6.10E-12	IGHG1	3	GPR183_B cell
1417	4.93E-12	-3.141923462	0.126	0.293	1.07E-07	IGHG2	3	GPR183_B cell

1418	2.52E-09	-3.170180796	0.155	0.337	5.49E-05	IGKV1-5	3	GPR183_B cell
1419	2.54E-07	-3.32719388	0.284	0.463	0.00555005	IGKV4-1	3	GPR183_B cell
1420	2.99E-08	-3.565872179	0.229	0.39	0.000651549	IGHV3-23	3	GPR183_B cell
1421	1.33E-07	-4.335588742	0.141	0.282	0.0028983	IGHV3-74	3	GPR183_B cell
1422	1.50E-107	1.950135993	0.923	0.364	3.27E-103	HIST1H4C	4	HIST1H4C_plasma cell
1423	4.90E-265	1.794701543	0.888	0.039	1.07E-260	RRM2	4	HIST1H4C_plasma cell
1424	1.66E-124	1.632991625	1	0.782	3.63E-120	GAPDH	4	HIST1H4C_plasma cell
1425	1.13E-171	1.385835262	0.95	0.165	2.47E-167	HMGB2	4	HIST1H4C_plasma cell
1426	2.32E-31	1.380900946	0.375	0.103	5.06E-27	IGHV6-1	4	HIST1H4C_plasma cell
1427	2.11E-119	1.299158913	1	0.674	4.60E-115	SUB1	4	HIST1H4C_plasma cell
1428	4.66E-99	1.19969223	1	0.591	1.02E-94	PPIB	4	HIST1H4C_plasma cell
1429	1.32E-118	1.197520318	0.954	0.282	2.88E-114	AQP3	4	HIST1H4C_plasma cell
1430	3.98E-113	1.186455758	0.896	0.229	8.68E-109	CCND2	4	HIST1H4C_plasma cell
1431	5.74E-08	1.174682976	0.317	0.165	0.001250784	IGKV2-24	4	HIST1H4C_plasma cell
1432	8.75E-86	1.133780256	0.985	0.621	1.91E-81	TUBA1B	4	HIST1H4C_plasma cell
1433	1.30E-243	1.131963959	0.861	0.045	2.83E-239	TYMS	4	HIST1H4C_plasma cell
1434	3.92E-103	1.117189706	0.992	0.62	8.55E-99	H2AFZ	4	HIST1H4C_plasma cell
1435	9.15E-115	1.109476456	0.988	0.36	2.00E-110	SDF2L1	4	HIST1H4C_plasma cell
1436	2.37E-85	1.102243149	1	0.612	5.17E-81	HSP90B1	4	HIST1H4C_plasma cell
1437	3.80E-110	1.098936233	0.988	0.517	8.29E-106	SEC61G	4	HIST1H4C_plasma cell
1438	4.20E-113	1.081128875	0.996	0.428	9.17E-109	P4HB	4	HIST1H4C_plasma cell
1439	3.60E-102	1.077031959	0.985	0.423	7.86E-98	SSR3	4	HIST1H4C_plasma cell
1440	1.17E-106	1.069526349	0.981	0.414	2.56E-102	PSME2	4	HIST1H4C_plasma cell
1441	2.21E-89	1.067871275	0.988	0.571	4.82E-85	HMGN2	4	HIST1H4C_plasma cell
1442	8.62E-102	1.06547995	0.961	0.325	1.88E-97	TXNDC5	4	HIST1H4C_plasma cell
1443	2.31E-108	1.058756424	0.985	0.491	5.05E-104	ATP5G3	4	HIST1H4C_plasma cell
1444	2.09E-108	1.05542212	0.988	0.548	4.56E-104	SEC61B	4	HIST1H4C_plasma cell
1445	2.22E-95	1.053382889	0.977	0.394	4.84E-91	MYDGF	4	HIST1H4C_plasma cell
1446	5.76E-82	1.047449834	0.988	0.42	1.26E-77	XBP1	4	HIST1H4C_plasma cell
1447	2.94E-103	1.030468143	1	0.787	6.41E-99	HMGB1	4	HIST1H4C_plasma cell
1448	1.56E-106	1.022954044	0.965	0.349	3.40E-102	TXN	4	HIST1H4C_plasma cell
1449	1.13E-103	1.020854249	0.973	0.407	2.47E-99	KDELR2	4	HIST1H4C_plasma cell
1450	3.58E-91	1.01765322	0.981	0.522	7.80E-87	LDHA	4	HIST1H4C_plasma cell
1451	1.45E-96	1.007872528	0.985	0.46	3.17E-92	PDIA6	4	HIST1H4C_plasma cell
1452	1.12E-187	1.002464616	0.923	0.115	2.43E-183	PTTG1	4	HIST1H4C_plasma cell
1453	2.75E-92	0.99571157	0.985	0.563	6.00E-88	RAN	4	HIST1H4C_plasma cell
1454	4.99E-89	0.981384264	0.992	0.437	1.09E-84	SEC11C	4	HIST1H4C_plasma cell
1455	7.47E-77	0.972949151	0.965	0.487	1.63E-72	TUBB	4	HIST1H4C_plasma cell
1456	2.25E-84	0.966213601	0.985	0.599	4.90E-80	CALR	4	HIST1H4C_plasma cell
1457	7.00E-93	0.961663727	0.992	0.666	1.53E-88	CHCHD2	4	HIST1H4C_plasma cell
1458	1.11E-92	0.953837419	0.988	0.373	2.43E-88	PDIA4	4	HIST1H4C_plasma cell
1459	8.67E-100	0.951562187	0.969	0.392	1.89E-95	PGAM1	4	HIST1H4C_plasma cell
1460	3.22E-118	0.947898579	0.969	0.321	7.03E-114	H2AFV	4	HIST1H4C_plasma cell
1461	9.24E-90	0.935673958	1	0.695	2.02E-85	MIF	4	HIST1H4C_plasma cell
1462	1.99E-100	0.934289899	0.973	0.318	4.33E-96	STMN1	4	HIST1H4C_plasma cell
1463	7.83E-91	0.928789341	0.985	0.398	1.71E-86	SPCS3	4	HIST1H4C_plasma cell
1464	4.99E-222	0.916142809	0.741	0.026	1.09E-217	CDC20	4	HIST1H4C_plasma cell
1465	2.68E-94	0.913029576	0.973	0.426	5.85E-90	OSTC	4	HIST1H4C_plasma cell
1466	1.69E-91	0.908958697	0.988	0.78	3.69E-87	RPS26	4	HIST1H4C_plasma cell
1467	3.53E-80	0.908127998	0.988	0.556	7.71E-76	MTDH	4	HIST1H4C_plasma cell
1468	5.50E-96	0.905941902	0.977	0.367	1.20E-91	MANF	4	HIST1H4C_plasma cell
1469	3.93E-98	0.902740917	0.996	0.433	8.58E-94	UQCRQ	4	HIST1H4C_plasma cell
1470	7.20E-98	0.901910433	0.973	0.424	1.57E-93	COX5A	4	HIST1H4C_plasma cell
1471	1.47E-85	0.898477912	0.996	0.483	3.21E-81	UBE2J1	4	HIST1H4C_plasma cell
1472	2.76E-78	0.889046694	0.977	0.525	6.02E-74	TPI1	4	HIST1H4C_plasma cell
1473	2.93E-90	0.879559826	0.992	0.549	6.40E-86	TMEM258	4	HIST1H4C_plasma cell
1474	1.81E-90	0.877004304	0.961	0.453	3.94E-86	COX8A	4	HIST1H4C_plasma cell
1475	9.18E-225	0.868336373	0.73	0.022	2.00E-220	UBE2C	4	HIST1H4C_plasma cell
1476	8.84E-93	0.856287678	0.988	0.481	1.93E-88	COPE	4	HIST1H4C_plasma cell
1477	6.10E-167	0.853231841	0.892	0.12	1.33E-162	SMC4	4	HIST1H4C_plasma cell
1478	4.17E-81	0.84838334	0.969	0.429	9.10E-77	PKM	4	HIST1H4C_plasma cell
1479	1.99E-123	0.839701371	0.954	0.245	4.34E-119	HN1	4	HIST1H4C_plasma cell
1480	1.37E-105	0.837568624	0.946	0.307	2.98E-101	LRRC59	4	HIST1H4C_plasma cell
1481	4.14E-88	0.834083642	0.946	0.312	9.03E-84	TNFRSF17	4	HIST1H4C_plasma cell
1482	6.30E-83	0.834036019	0.977	0.446	1.37E-78	SNRPG	4	HIST1H4C_plasma cell
1483	2.16E-86	0.818992974	0.988	0.46	4.72E-82	ATP5J	4	HIST1H4C_plasma cell
1484	4.36E-239	0.809667666	0.807	0.033	9.50E-235	KIAA0101	4	HIST1H4C_plasma cell
1485	1.06E-81	0.803630362	0.985	0.418	2.31E-77	RPN1	4	HIST1H4C_plasma cell
1486	1.07E-74	0.800600192	0.996	0.604	2.34E-70	NME2	4	HIST1H4C_plasma cell
1487	2.60E-241	0.798516642	0.869	0.043	5.66E-237	MKI67	4	HIST1H4C_plasma cell
1488	6.52E-207	0.79637913	0.846	0.067	1.42E-202	MYBL2	4	HIST1H4C_plasma cell

1489	3.06E-102	0.795111132	0.911	0.24	6.67E-98	FABP5	4	HIST1H4C_plasma cell
1490	7.93E-79	0.792758402	0.973	0.412	1.73E-74	ENO1	4	HIST1H4C_plasma cell
1491	5.69E-58	0.792011844	0.992	0.44	1.24E-53	MZB1	4	HIST1H4C_plasma cell
1492	5.99E-75	0.791459525	0.985	0.411	1.31E-70	LMAN1	4	HIST1H4C_plasma cell
1493	2.58E-69	0.789925165	0.981	0.554	5.63E-65	HSPA5	4	HIST1H4C_plasma cell
1494	1.88E-83	0.789568239	0.965	0.407	4.09E-79	PRELID1	4	HIST1H4C_plasma cell
1495	2.28E-91	0.787167105	0.873	0.229	4.98E-87	DUT	4	HIST1H4C_plasma cell
1496	1.17E-70	0.784060035	0.969	0.494	2.56E-66	LDHB	4	HIST1H4C_plasma cell
1497	4.00E-87	0.78346123	0.992	0.636	8.72E-83	COX6A1	4	HIST1H4C_plasma cell
1498	2.21E-151	0.783151464	0.923	0.157	4.82E-147	GGH	4	HIST1H4C_plasma cell
1499	3.90E-79	0.781233869	0.992	0.607	8.49E-75	COX6B1	4	HIST1H4C_plasma cell
1500	4.05E-87	0.776514663	0.88	0.257	8.84E-83	HMGA1	4	HIST1H4C_plasma cell
1501	1.39E-81	0.764768019	0.954	0.38	3.03E-77	ATP5G1	4	HIST1H4C_plasma cell
1502	3.09E-79	0.763746001	0.985	0.56	6.73E-75	SPCS2	4	HIST1H4C_plasma cell
1503	1.05E-11	0.754538478	0.672	0.388	2.30E-07	IGKV4-1	4	HIST1H4C_plasma cell
1504	4.58E-23	0.753665097	0.749	0.371	9.99E-19	CRIP1	4	HIST1H4C_plasma cell
1505	1.69E-94	0.752569544	0.954	0.336	3.69E-90	ANAPC11	4	HIST1H4C_plasma cell
1506	3.56E-70	0.750663244	0.969	0.419	7.76E-66	RPN2	4	HIST1H4C_plasma cell
1507	2.36E-205	0.748667803	0.822	0.056	5.14E-201	NUSAP1	4	HIST1H4C_plasma cell
1508	2.24E-77	0.748354091	0.977	0.442	4.89E-73	COX7B	4	HIST1H4C_plasma cell
1509	4.24E-88	0.746355656	0.954	0.337	9.26E-84	PPA1	4	HIST1H4C_plasma cell
1510	2.19E-63	0.745615909	0.973	0.438	4.78E-59	ITM2C	4	HIST1H4C_plasma cell
1511	7.88E-126	0.741749262	0.876	0.168	1.72E-121	C12orf75	4	HIST1H4C_plasma cell
1512	1.71E-93	0.74093223	0.923	0.275	3.73E-89	RANBP1	4	HIST1H4C_plasma cell
1513	3.68E-73	0.737391281	0.977	0.471	8.03E-69	ATP5A1	4	HIST1H4C_plasma cell
1514	9.28E-112	0.735758597	0.95	0.254	2.02E-107	CALM3	4	HIST1H4C_plasma cell
1515	2.37E-68	0.734631373	0.992	0.679	5.17E-64	UQCRH	4	HIST1H4C_plasma cell
1516	5.03E-68	0.727515344	0.996	0.91	1.10E-63	PPIA	4	HIST1H4C_plasma cell
1517	1.21E-79	0.725202418	0.969	0.42	2.65E-75	NDUFS5	4	HIST1H4C_plasma cell
1518	4.07E-62	0.719662479	0.985	0.376	8.88E-58	FKBP11	4	HIST1H4C_plasma cell
1519	1.28E-74	0.719566354	0.981	0.409	2.80E-70	LMAN2	4	HIST1H4C_plasma cell
1520	5.14E-63	0.716127749	0.985	0.598	1.12E-58	SLC25A5	4	HIST1H4C_plasma cell
1521	1.87E-77	0.714132483	0.977	0.412	4.08E-73	SSR1	4	HIST1H4C_plasma cell
1522	6.96E-81	0.713627981	0.969	0.392	1.52E-76	PSMB3	4	HIST1H4C_plasma cell
1523	3.32E-76	0.71228127	0.973	0.422	7.25E-72	NDUFV2	4	HIST1H4C_plasma cell
1524	2.38E-94	0.709336903	0.931	0.3	5.19E-90	PRDX3	4	HIST1H4C_plasma cell
1525	8.33E-87	0.708090922	0.961	0.34	1.82E-82	SEC61A1	4	HIST1H4C_plasma cell
1526	3.15E-38	0.708019324	0.992	0.817	6.86E-34	ACTG1	4	HIST1H4C_plasma cell
1527	2.00E-70	0.70669571	0.985	0.365	4.36E-66	CD38	4	HIST1H4C_plasma cell
1528	9.91E-68	0.706395858	0.961	0.422	2.16E-63	KDELR1	4	HIST1H4C_plasma cell
1529	4.44E-71	0.70397335	0.973	0.416	9.69E-67	TMED9	4	HIST1H4C_plasma cell
1530	7.86E-79	0.703704487	0.977	0.377	1.71E-74	PRDX2	4	HIST1H4C_plasma cell
1531	1.33E-71	0.693005689	0.969	0.433	2.90E-67	ERH	4	HIST1H4C_plasma cell
1532	6.15E-68	0.692736342	0.911	0.357	1.34E-63	IGLL5	4	HIST1H4C_plasma cell
1533	6.43E-99	0.69179138	0.95	0.29	1.40E-94	PSMB2	4	HIST1H4C_plasma cell
1534	6.38E-99	0.691588407	0.938	0.265	1.39E-94	SRM	4	HIST1H4C_plasma cell
1535	1.24E-81	0.690809243	0.958	0.345	2.70E-77	DNAJB11	4	HIST1H4C_plasma cell
1536	1.40E-204	0.6890414	0.749	0.038	3.06E-200	DHFR	4	HIST1H4C_plasma cell
1537	4.37E-97	0.686272213	0.95	0.305	9.52E-93	CYC1	4	HIST1H4C_plasma cell
1538	7.23E-66	0.684284589	0.961	0.449	1.58E-61	TMED2	4	HIST1H4C_plasma cell
1539	2.29E-67	0.679873582	0.973	0.415	5.00E-63	DDOST	4	HIST1H4C_plasma cell
1540	1.56E-56	0.676114469	0.981	0.614	3.41E-52	ALDOA	4	HIST1H4C_plasma cell
1541	6.58E-119	0.67530349	0.9	0.192	1.43E-114	P11-1070N1(	4	HIST1H4C_plasma cell
1542	1.45E-63	0.672725958	0.969	0.552	3.15E-59	ATP5B	4	HIST1H4C_plasma cell
1543	4.41E-100	0.672347081	0.934	0.281	9.61E-96	MLEC	4	HIST1H4C_plasma cell
1544	5.89E-246	0.671198816	0.83	0.031	1.28E-241	BIRC5	4	HIST1H4C_plasma cell
1545	2.03E-66	0.670629892	0.969	0.37	4.43E-62	VIMP	4	HIST1H4C_plasma cell
1546	3.29E-61	0.670076282	0.965	0.402	7.18E-57	HM13	4	HIST1H4C_plasma cell
1547	2.99E-91	0.669943918	0.934	0.284	6.51E-87	ANXA2	4	HIST1H4C_plasma cell
1548	3.73E-221	0.669811347	0.788	0.036	8.13E-217	TK1	4	HIST1H4C_plasma cell
1549	2.70E-56	0.669437445	0.981	0.526	5.89E-52	SPCS1	4	HIST1H4C_plasma cell
1550	2.66E-233	0.667461157	0.803	0.034	5.80E-229	SHCBP1	4	HIST1H4C_plasma cell
1551	1.23E-80	0.667206932	0.954	0.349	2.69E-76	NDUFB6	4	HIST1H4C_plasma cell
1552	2.06E-66	0.666456347	0.965	0.464	4.50E-62	ATP5J2	4	HIST1H4C_plasma cell
1553	5.13E-80	0.662415701	0.946	0.348	1.12E-75	SEC13	4	HIST1H4C_plasma cell
1554	2.00E-84	0.657653565	0.942	0.35	4.37E-80	AURKAIP1	4	HIST1H4C_plasma cell
1555	1.61E-72	0.65315353	0.958	0.386	3.52E-68	PSMB6	4	HIST1H4C_plasma cell
1556	8.36E-71	0.652000144	0.923	0.349	1.82E-66	C1QBP	4	HIST1H4C_plasma cell
1557	6.28E-50	0.64949294	0.981	0.781	1.37E-45	YBX1	4	HIST1H4C_plasma cell
1558	5.50E-64	0.649491986	0.985	0.534	1.20E-59	NDUFA4	4	HIST1H4C_plasma cell
1559	4.63E-110	0.649201259	0.861	0.205	1.01E-105	NME1	4	HIST1H4C_plasma cell

1560	2.84E-68	0.646845109	0.992	0.666	6.20E-64	RPL36AL	4	HIST1H4C_plasma cell
1561	4.78E-89	0.646290591	0.954	0.329	1.04E-84	NDUFAB1	4	HIST1H4C_plasma cell
1562	2.86E-53	0.645136263	0.973	0.523	6.24E-49	POU2AF1	4	HIST1H4C_plasma cell
1563	9.24E-67	0.64504719	0.985	0.585	2.01E-62	COX7A2	4	HIST1H4C_plasma cell
1564	1.07E-86	0.644079026	0.915	0.273	2.34E-82	MTHFD2	4	HIST1H4C_plasma cell
1565	4.42E-243	0.644073318	0.873	0.042	9.64E-239	MAD2L1	4	HIST1H4C_plasma cell
1566	7.21E-213	0.643493893	0.761	0.034	1.57E-208	CDKN3	4	HIST1H4C_plasma cell
1567	1.23E-88	0.641952852	0.876	0.244	2.68E-84	UAP1	4	HIST1H4C_plasma cell
1568	6.36E-60	0.641677069	0.969	0.456	1.39E-55	TMED10	4	HIST1H4C_plasma cell
1569	3.12E-107	0.641192104	0.927	0.249	6.81E-103	GMPPB	4	HIST1H4C_plasma cell
1570	2.32E-198	0.638731701	0.826	0.062	5.06E-194	FEN1	4	HIST1H4C_plasma cell
1571	8.88E-89	0.63839525	0.969	0.331	1.94E-84	NDUFS6	4	HIST1H4C_plasma cell
1572	2.33E-62	0.637830832	0.992	0.504	5.08E-58	DAD1	4	HIST1H4C_plasma cell
1573	3.02E-72	0.637230589	0.977	0.356	6.59E-68	HDLBP	4	HIST1H4C_plasma cell
1574	9.63E-91	0.636733043	0.938	0.303	2.10E-86	NDUFA6	4	HIST1H4C_plasma cell
1575	2.08E-63	0.636124956	0.985	0.493	4.54E-59	POLR2L	4	HIST1H4C_plasma cell
1576	1.38E-64	0.634958285	0.961	0.42	3.02E-60	CANX	4	HIST1H4C_plasma cell
1577	4.35E-57	0.627212129	0.969	0.365	9.49E-53	PRDX4	4	HIST1H4C_plasma cell
1578	5.85E-68	0.626906634	0.992	0.443	1.28E-63	ROMO1	4	HIST1H4C_plasma cell
1579	1.79E-97	0.626572887	0.954	0.291	3.90E-93	LSM3	4	HIST1H4C_plasma cell
1580	5.08E-08	0.625355554	0.162	0.063	0.001108196	IGKV2D-30	4	HIST1H4C_plasma cell
1581	3.77E-78	0.625335714	0.927	0.318	8.21E-74	CRELD2	4	HIST1H4C_plasma cell
1582	2.57E-93	0.624146353	0.934	0.28	5.60E-89	LIME1	4	HIST1H4C_plasma cell
1583	5.36E-59	0.620713237	0.977	0.589	1.17E-54	COX6C	4	HIST1H4C_plasma cell
1584	2.31E-126	0.619289818	0.776	0.103	5.05E-122	MCM7	4	HIST1H4C_plasma cell
1585	3.26E-87	0.619087253	0.954	0.312	7.11E-83	CISD2	4	HIST1H4C_plasma cell
1586	1.74E-87	0.617352289	0.934	0.3	3.79E-83	PSMA3	4	HIST1H4C_plasma cell
1587	5.36E-88	0.612534169	0.934	0.304	1.17E-83	PSMA5	4	HIST1H4C_plasma cell
1588	1.10E-86	0.609684656	0.954	0.328	2.40E-82	MRPL51	4	HIST1H4C_plasma cell
1589	8.76E-71	0.609027785	0.931	0.344	1.91E-66	SNRPB	4	HIST1H4C_plasma cell
1590	8.81E-69	0.606871948	0.923	0.364	1.92E-64	MDH2	4	HIST1H4C_plasma cell
1591	1.02E-48	0.606027239	0.923	0.416	2.23E-44	GSTP1	4	HIST1H4C_plasma cell
1592	5.44E-59	0.605742192	0.973	0.494	1.19E-54	ZNF706	4	HIST1H4C_plasma cell
1593	4.39E-65	0.604636869	0.961	0.388	9.57E-61	DERL1	4	HIST1H4C_plasma cell
1594	2.61E-101	0.603479702	0.826	0.178	5.70E-97	KPNA2	4	HIST1H4C_plasma cell
1595	1.43E-72	0.602797019	0.653	0.143	3.11E-68	HIST1H1E	4	HIST1H4C_plasma cell
1596	2.68E-65	0.601147345	0.961	0.391	5.84E-61	CDK2AP2	4	HIST1H4C_plasma cell
1597	3.43E-82	0.600259848	0.934	0.312	7.47E-78	PSMA6	4	HIST1H4C_plasma cell
1598	5.55E-87	0.597026061	0.911	0.284	1.21E-82	UQCRC1	4	HIST1H4C_plasma cell
1599	6.70E-49	0.596654717	0.977	0.566	1.46E-44	PRDX1	4	HIST1H4C_plasma cell
1600	9.47E-54	0.59250517	0.977	0.468	2.07E-49	TRAM1	4	HIST1H4C_plasma cell
1601	1.08E-87	0.5921674	0.931	0.305	2.37E-83	NDUFB3	4	HIST1H4C_plasma cell
1602	5.04E-240	0.591205344	0.753	0.018	1.10E-235	CCNA2	4	HIST1H4C_plasma cell
1603	7.73E-212	0.590766528	0.691	0.02	1.68E-207	CDK1	4	HIST1H4C_plasma cell
1604	8.37E-71	0.590070922	0.942	0.357	1.83E-66	SRSF9	4	HIST1H4C_plasma cell
1605	6.09E-237	0.589641231	0.819	0.034	1.33E-232	CENPW	4	HIST1H4C_plasma cell
1606	3.98E-111	0.589469808	0.911	0.229	8.69E-107	TXNDC17	4	HIST1H4C_plasma cell
1607	2.89E-61	0.588154586	0.965	0.459	6.30E-57	GNG5	4	HIST1H4C_plasma cell
1608	1.42E-67	0.587778525	0.938	0.39	3.09E-63	PSMD8	4	HIST1H4C_plasma cell
1609	1.18E-134	0.584106627	0.61	0.048	2.57E-130	HIST1H2AJ	4	HIST1H4C_plasma cell
1610	9.23E-52	0.583821099	0.977	0.518	2.01E-47	EIF5A	4	HIST1H4C_plasma cell
1611	2.32E-190	0.579222827	0.741	0.046	5.05E-186	BUB1	4	HIST1H4C_plasma cell
1612	3.37E-82	0.579192498	0.946	0.31	7.35E-78	TALDO1	4	HIST1H4C_plasma cell
1613	1.46E-75	0.579092464	0.95	0.344	3.19E-71	ANXA5	4	HIST1H4C_plasma cell
1614	1.58E-113	0.577919177	0.869	0.183	3.45E-109	CD320	4	HIST1H4C_plasma cell
1615	3.68E-39	0.575211131	0.981	0.745	8.02E-35	VIM	4	HIST1H4C_plasma cell
1616	1.23E-62	0.575048079	0.969	0.437	2.68E-58	PSMB1	4	HIST1H4C_plasma cell
1617	1.81E-116	0.57487252	0.88	0.189	3.94E-112	DCPS	4	HIST1H4C_plasma cell
1618	4.71E-64	0.573621196	0.95	0.409	1.03E-59	ATP5F1	4	HIST1H4C_plasma cell
1619	3.33E-86	0.572003463	0.907	0.28	7.27E-82	NANS	4	HIST1H4C_plasma cell
1620	6.59E-65	0.571696184	0.958	0.395	1.44E-60	ATP5C1	4	HIST1H4C_plasma cell
1621	1.96E-54	0.570992384	0.973	0.496	4.27E-50	C14orf2	4	HIST1H4C_plasma cell
1622	8.42E-55	0.570356166	0.996	0.882	1.84E-50	H3F3A	4	HIST1H4C_plasma cell
1623	4.17E-80	0.570203471	0.915	0.282	9.09E-76	CKAP4	4	HIST1H4C_plasma cell
1624	8.07E-84	0.568931657	0.961	0.325	1.76E-79	LSM5	4	HIST1H4C_plasma cell
1625	6.74E-21	0.56848871	0.506	0.221	1.47E-16	IGHG2	4	HIST1H4C_plasma cell
1626	1.05E-73	0.568475113	0.876	0.278	2.29E-69	IDH2	4	HIST1H4C_plasma cell
1627	4.05E-146	0.568015491	0.846	0.122	8.82E-142	TMEM106C	4	HIST1H4C_plasma cell
1628	9.94E-78	0.566953239	0.954	0.332	2.17E-73	NDUFB7	4	HIST1H4C_plasma cell
1629	3.53E-76	0.563752248	0.958	0.315	7.69E-72	RRBP1	4	HIST1H4C_plasma cell
1630	6.56E-172	0.563317199	0.745	0.058	1.43E-167	PHF19	4	HIST1H4C_plasma cell

1631	2.05E-58	0.561565901	0.958	0.442	4.47E-54	PARK7	4	HIST1H4C_plasma cell
1632	9.07E-46	0.561517064	0.95	0.382	1.98E-41	DERL3	4	HIST1H4C_plasma cell
1633	2.84E-85	0.560241176	0.892	0.278	6.19E-81	PDCD5	4	HIST1H4C_plasma cell
1634	1.68E-76	0.558588365	0.938	0.325	3.66E-72	UQCRFS1	4	HIST1H4C_plasma cell
1635	8.62E-72	0.558123773	0.892	0.304	1.88E-67	TKT	4	HIST1H4C_plasma cell
1636	1.66E-119	0.554820864	0.71	0.096	3.62E-115	PCNA	4	HIST1H4C_plasma cell
1637	1.10E-92	0.554542548	0.911	0.245	2.39E-88	HNRNPAB	4	HIST1H4C_plasma cell
1638	4.74E-42	0.553405604	0.927	0.398	1.03E-37	LGALS1	4	HIST1H4C_plasma cell
1639	6.94E-242	0.552638796	0.753	0.018	1.51E-237	ZWINT	4	HIST1H4C_plasma cell
1640	8.58E-77	0.549124659	0.641	0.131	1.87E-72	HIST1H2BJ	4	HIST1H4C_plasma cell
1641	2.66E-54	0.549030569	0.965	0.378	5.79E-50	ERLEC1	4	HIST1H4C_plasma cell
1642	1.55E-52	0.548355225	0.958	0.445	3.38E-48	PEBP1	4	HIST1H4C_plasma cell
1643	2.50E-78	0.547786624	0.927	0.299	5.44E-74	SRPRB	4	HIST1H4C_plasma cell
1644	1.57E-61	0.547731571	0.965	0.427	3.41E-57	TUFM	4	HIST1H4C_plasma cell
1645	3.89E-177	0.544130227	0.753	0.058	8.49E-173	APOBEC3B	4	HIST1H4C_plasma cell
1646	1.32E-78	0.541396255	0.942	0.308	2.89E-74	SAR1B	4	HIST1H4C_plasma cell
1647	9.01E-70	0.540387029	0.934	0.331	1.96E-65	MRPS24	4	HIST1H4C_plasma cell
1648	3.43E-51	0.538485466	0.946	0.46	7.47E-47	UQCR10	4	HIST1H4C_plasma cell
1649	2.90E-150	0.537600448	0.772	0.087	6.33E-146	HMGB3	4	HIST1H4C_plasma cell
1650	4.73E-81	0.537228125	0.907	0.28	1.03E-76	NDUFC1	4	HIST1H4C_plasma cell
1651	5.61E-52	0.536445826	0.988	0.53	1.22E-47	TCEB2	4	HIST1H4C_plasma cell
1652	3.44E-154	0.535846804	0.483	0.008	7.50E-150	PLK1	4	HIST1H4C_plasma cell
1653	2.10E-46	0.534290923	0.969	0.622	4.58E-42	SLC25A3	4	HIST1H4C_plasma cell
1654	9.36E-82	0.533418136	0.931	0.285	2.04E-77	SNRPD1	4	HIST1H4C_plasma cell
1655	5.60E-81	0.530412888	0.9	0.278	1.22E-76	PREB	4	HIST1H4C_plasma cell
1656	2.60E-96	0.530293702	0.88	0.228	5.68E-92	SLC35B1	4	HIST1H4C_plasma cell
1657	3.37E-99	0.530046721	0.815	0.172	7.35E-95	GYG1	4	HIST1H4C_plasma cell
1658	8.05E-70	0.527949836	0.965	0.362	1.75E-65	MRPS34	4	HIST1H4C_plasma cell
1659	3.63E-87	0.52766739	0.907	0.264	7.91E-83	STT3A	4	HIST1H4C_plasma cell
1660	1.47E-64	0.526418731	0.934	0.359	3.20E-60	ILF2	4	HIST1H4C_plasma cell
1661	5.81E-79	0.526271352	0.931	0.305	1.27E-74	IER3IP1	4	HIST1H4C_plasma cell
1662	4.16E-56	0.526257004	0.961	0.41	9.08E-52	VOPP1	4	HIST1H4C_plasma cell
1663	9.48E-177	0.526220763	0.672	0.033	2.07E-172	ASPM	4	HIST1H4C_plasma cell
1664	1.01E-48	0.525674203	0.969	0.477	2.20E-44	USMG5	4	HIST1H4C_plasma cell
1665	3.96E-46	0.524052912	0.965	0.48	8.64E-42	ARF4	4	HIST1H4C_plasma cell
1666	1.95E-93	0.522430337	0.876	0.237	4.25E-89	STOML2	4	HIST1H4C_plasma cell
1667	2.70E-87	0.521407258	0.907	0.261	5.89E-83	GARS	4	HIST1H4C_plasma cell
1668	7.56E-47	0.52028235	0.961	0.527	1.65E-42	ARF1	4	HIST1H4C_plasma cell
1669	2.37E-67	0.519010152	0.927	0.333	5.16E-63	VDAC1	4	HIST1H4C_plasma cell
1670	1.36E-59	0.517104915	0.942	0.378	2.96E-55	CCT8	4	HIST1H4C_plasma cell
1671	3.50E-46	0.516999789	0.973	0.522	7.63E-42	RBM3	4	HIST1H4C_plasma cell
1672	3.97E-172	0.516360336	0.629	0.026	8.66E-168	TOP2A	4	HIST1H4C_plasma cell
1673	7.21E-47	0.515462614	0.988	0.637	1.57E-42	SSR2	4	HIST1H4C_plasma cell
1674	1.67E-41	0.515368778	0.985	0.533	3.64E-37	RABAC1	4	HIST1H4C_plasma cell
1675	3.20E-61	0.515156001	0.969	0.39	6.98E-57	GHITM	4	HIST1H4C_plasma cell
1676	8.43E-64	0.514837535	0.919	0.33	1.84E-59	PA2G4	4	HIST1H4C_plasma cell
1677	9.97E-100	0.513693541	0.876	0.213	2.17E-95	TIMM8B	4	HIST1H4C_plasma cell
1678	4.29E-54	0.513506114	0.938	0.407	9.35E-50	FKBP1A	4	HIST1H4C_plasma cell
1679	5.50E-67	0.513053792	0.923	0.339	1.20E-62	BUB3	4	HIST1H4C_plasma cell
1680	1.90E-46	0.512985677	0.977	0.53	4.14E-42	COX5B	4	HIST1H4C_plasma cell
1681	3.35E-48	0.508223724	0.973	0.463	7.30E-44	SET	4	HIST1H4C_plasma cell
1682	6.79E-97	0.508217951	0.931	0.243	1.48E-92	HNRNPD	4	HIST1H4C_plasma cell
1683	6.10E-84	0.506181231	0.934	0.278	1.33E-79	SNRPC	4	HIST1H4C_plasma cell
1684	1.49E-76	0.505713065	0.919	0.297	3.25E-72	SRP72	4	HIST1H4C_plasma cell
1685	1.42E-204	0.505621171	0.668	0.019	3.09E-200	CCNB2	4	HIST1H4C_plasma cell
1686	3.69E-86	0.505113749	0.9	0.262	8.05E-82	MRPL57	4	HIST1H4C_plasma cell
1687	3.69E-48	0.503713141	0.981	0.487	8.04E-44	ANP32B	4	HIST1H4C_plasma cell
1688	2.63E-111	0.501716612	0.861	0.183	5.73E-107	PSMA2	4	HIST1H4C_plasma cell
1689	5.86E-80	0.501262259	0.892	0.26	1.28E-75	EPRS	4	HIST1H4C_plasma cell
1690	7.80E-90	0.499893872	0.799	0.17	1.70E-85	MCM5	4	HIST1H4C_plasma cell
1691	3.54E-94	0.4992962	0.919	0.257	7.72E-90	FDPS	4	HIST1H4C_plasma cell
1692	4.41E-60	0.4985604	0.954	0.392	9.62E-56	ATPIF1	4	HIST1H4C_plasma cell
1693	5.40E-197	0.497430638	0.637	0.017	1.18E-192	TPX2	4	HIST1H4C_plasma cell
1694	8.29E-58	0.496915026	0.977	0.425	1.81E-53	NDUFB9	4	HIST1H4C_plasma cell
1695	7.35E-52	0.495936099	0.961	0.441	1.60E-47	PSMA7	4	HIST1H4C_plasma cell
1696	2.60E-54	0.495392089	1	0.858	5.67E-50	SERF2	4	HIST1H4C_plasma cell
1697	3.72E-172	0.495222567	0.784	0.066	8.11E-168	SMC2	4	HIST1H4C_plasma cell
1698	3.27E-81	0.495181335	0.911	0.284	7.13E-77	C20orf24	4	HIST1H4C_plasma cell
1699	2.45E-46	0.494362427	0.992	0.52	5.34E-42	NDUFA1	4	HIST1H4C_plasma cell
1700	5.33E-139	0.494316743	0.537	0.027	1.16E-134	CENPF	4	HIST1H4C_plasma cell
1701	3.92E-53	0.493776942	0.969	0.426	8.55E-49	NDUFB4	4	HIST1H4C_plasma cell

1702	1.75E-196	0.493337545	0.745	0.042	3.81E-192	UBE2T	4	HIST1H4C_plasma cell
1703	9.80E-153	0.491790628	0.552	0.022	2.14E-148	HIST1H1B	4	HIST1H4C_plasma cell
1704	6.39E-108	0.491385188	0.811	0.161	1.39E-103	CSRP1	4	HIST1H4C_plasma cell
1705	5.44E-197	0.4907881	0.749	0.042	1.19E-192	CDC6	4	HIST1H4C_plasma cell
1706	2.02E-94	0.489024212	0.896	0.231	4.40E-90	SURF4	4	HIST1H4C_plasma cell
1707	1.19E-49	0.488888049	0.965	0.448	2.59E-45	PRDX5	4	HIST1H4C_plasma cell
1708	4.98E-84	0.487859325	0.919	0.275	1.09E-79	SIVA1	4	HIST1H4C_plasma cell
1709	5.88E-163	0.487685944	0.595	0.027	1.28E-158	GINS2	4	HIST1H4C_plasma cell
1710	2.81E-62	0.485676026	0.95	0.363	6.14E-58	TOP1	4	HIST1H4C_plasma cell
1711	3.47E-44	0.485079988	0.954	0.49	7.56E-40	PGK1	4	HIST1H4C_plasma cell
1712	4.94E-64	0.482579561	0.934	0.356	1.08E-59	SF3B5	4	HIST1H4C_plasma cell
1713	1.85E-55	0.481892307	0.977	0.399	4.04E-51	SRPRA	4	HIST1H4C_plasma cell
1714	4.31E-103	0.481513289	0.853	0.194	9.41E-99	UFD1L	4	HIST1H4C_plasma cell
1715	6.96E-71	0.481159085	0.911	0.303	1.52E-66	MINOS1	4	HIST1H4C_plasma cell
1716	4.58E-63	0.478832412	0.946	0.348	9.98E-59	SND1	4	HIST1H4C_plasma cell
1717	1.04E-68	0.478729865	0.942	0.339	2.26E-64	EIF2S2	4	HIST1H4C_plasma cell
1718	2.00E-76	0.477319566	0.888	0.267	4.35E-72	CALU	4	HIST1H4C_plasma cell
1719	9.92E-62	0.473917492	0.946	0.354	2.16E-57	XRCC5	4	HIST1H4C_plasma cell
1720	7.84E-37	0.473808559	0.961	0.571	1.71E-32	LSP1	4	HIST1H4C_plasma cell
1721	5.61E-70	0.473783517	0.919	0.322	1.22E-65	NDUFAF3	4	HIST1H4C_plasma cell
1722	7.03E-46	0.473430861	0.973	0.478	1.53E-41	UQCR11	4	HIST1H4C_plasma cell
1723	2.30E-69	0.473232266	0.965	0.348	5.01E-65	RBX1	4	HIST1H4C_plasma cell
1724	8.10E-108	0.472158172	0.88	0.191	1.77E-103	CCDC167	4	HIST1H4C_plasma cell
1725	2.05E-80	0.470404347	0.958	0.28	4.46E-76	DDX39A	4	HIST1H4C_plasma cell
1726	2.05E-53	0.46897237	0.969	0.424	4.47E-49	NDUFB10	4	HIST1H4C_plasma cell
1727	2.54E-112	0.468570444	0.819	0.152	5.54E-108	SSRP1	4	HIST1H4C_plasma cell
1728	4.79E-43	0.468261262	0.973	0.593	1.04E-38	PSME1	4	HIST1H4C_plasma cell
1729	4.41E-57	0.466336784	0.958	0.392	9.61E-53	TECR	4	HIST1H4C_plasma cell
1730	4.80E-78	0.466014083	0.869	0.26	1.05E-73	SLIRP	4	HIST1H4C_plasma cell
1731	3.90E-43	0.465697833	0.996	0.57	8.51E-39	JCHAIN	4	HIST1H4C_plasma cell
1732	4.33E-181	0.464887806	0.807	0.067	9.45E-177	DTYMK	4	HIST1H4C_plasma cell
1733	7.99E-47	0.464852146	0.938	0.459	1.74E-42	PPP1CA	4	HIST1H4C_plasma cell
1734	4.78E-83	0.463629911	0.927	0.26	1.04E-78	ANP32E	4	HIST1H4C_plasma cell
1735	1.08E-88	0.4617341	0.876	0.228	2.35E-84	VDAC3	4	HIST1H4C_plasma cell
1736	1.35E-62	0.46092939	0.954	0.359	2.95E-58	NDUFA11	4	HIST1H4C_plasma cell
1737	5.91E-81	0.460928507	0.923	0.276	1.29E-76	CBWD1	4	HIST1H4C_plasma cell
1738	9.88E-84	0.460105243	0.842	0.222	2.16E-79	PHB	4	HIST1H4C_plasma cell
1739	4.91E-34	0.45824378	0.958	0.5	1.07E-29	SELK	4	HIST1H4C_plasma cell
1740	3.56E-84	0.458139125	0.946	0.278	7.75E-80	PSMA4	4	HIST1H4C_plasma cell
1741	1.02E-75	0.458100678	0.942	0.314	2.22E-71	NDUFS8	4	HIST1H4C_plasma cell
1742	3.77E-103	0.45720874	0.757	0.131	8.23E-99	UBE2S	4	HIST1H4C_plasma cell
1743	5.06E-50	0.456502905	0.958	0.416	1.10E-45	SELT	4	HIST1H4C_plasma cell
1744	9.39E-124	0.455592164	0.672	0.074	2.05E-119	MCM3	4	HIST1H4C_plasma cell
1745	1.77E-46	0.455168291	0.961	0.474	3.87E-42	CYCS	4	HIST1H4C_plasma cell
1746	1.21E-35	0.454449838	0.988	0.666	2.63E-31	CLIC1	4	HIST1H4C_plasma cell
1747	1.42E-126	0.453908728	0.819	0.132	3.11E-122	EBP	4	HIST1H4C_plasma cell
1748	4.27E-135	0.453711264	0.792	0.1	9.30E-131	CENPM	4	HIST1H4C_plasma cell
1749	1.96E-84	0.453034512	0.834	0.212	4.27E-80	SLC38A5	4	HIST1H4C_plasma cell
1750	5.55E-65	0.451869871	0.915	0.317	1.21E-60	TMEM208	4	HIST1H4C_plasma cell
1751	8.61E-69	0.451475902	0.907	0.304	1.88E-64	CCT7	4	HIST1H4C_plasma cell
1752	1.89E-60	0.451428235	0.954	0.373	4.11E-56	YWHAE	4	HIST1H4C_plasma cell
1753	6.04E-137	0.449729481	0.784	0.106	1.32E-132	BOLA3	4	HIST1H4C_plasma cell
1754	6.32E-37	0.449652846	0.981	0.651	1.38E-32	OST4	4	HIST1H4C_plasma cell
1755	9.67E-40	0.448426016	0.985	0.548	2.11E-35	ATP5D	4	HIST1H4C_plasma cell
1756	8.70E-81	0.448051695	0.919	0.277	1.90E-76	RER1	4	HIST1H4C_plasma cell
1757	1.38E-71	0.447825536	0.915	0.305	3.01E-67	MESDC2	4	HIST1H4C_plasma cell
1758	1.45E-130	0.447226617	0.51	0.025	3.17E-126	HIST1H2AI	4	HIST1H4C_plasma cell
1759	1.70E-190	0.447086143	0.637	0.019	3.70E-186	DLGAP5	4	HIST1H4C_plasma cell
1760	9.47E-154	0.446395452	0.784	0.083	2.07E-149	KIF22	4	HIST1H4C_plasma cell
1761	1.12E-102	0.446103434	0.903	0.204	2.44E-98	LSM4	4	HIST1H4C_plasma cell
1762	1.18E-36	0.446070439	0.981	0.522	2.57E-32	KRTCAP2	4	HIST1H4C_plasma cell
1763	9.36E-89	0.445707969	0.873	0.216	2.04E-84	SLC9A3R1	4	HIST1H4C_plasma cell
1764	4.32E-33	0.445350354	0.981	0.656	9.43E-29	RPL22L1	4	HIST1H4C_plasma cell
1765	5.40E-54	0.445178606	0.923	0.344	1.18E-49	TXNDC11	4	HIST1H4C_plasma cell
1766	8.65E-183	0.444879073	0.606	0.017	1.89E-178	PKMYT1	4	HIST1H4C_plasma cell
1767	2.64E-140	0.443708506	0.56	0.031	5.75E-136	CCNB1	4	HIST1H4C_plasma cell
1768	1.33E-201	0.442076312	0.734	0.034	2.90E-197	CENPU	4	HIST1H4C_plasma cell
1769	5.11E-95	0.441794656	0.641	0.097	1.12E-90	LILRB4	4	HIST1H4C_plasma cell
1770	2.41E-97	0.4417038	0.842	0.194	5.26E-93	MTCH2	4	HIST1H4C_plasma cell
1771	1.20E-94	0.441678	0.629	0.096	2.61E-90	PSAT1	4	HIST1H4C_plasma cell
1772	1.28E-154	0.44158621	0.764	0.077	2.79E-150	BCL2L12	4	HIST1H4C_plasma cell

1773	9.50E-78	0.440856726	0.896	0.258	2.07E-73	CCT6A	4	HIST1H4C_plasma cell
1774	1.22E-67	0.439665216	0.927	0.298	2.66E-63	DNAJC3	4	HIST1H4C_plasma cell
1775	5.84E-75	0.439503986	0.934	0.297	1.27E-70	PSMD7	4	HIST1H4C_plasma cell
1776	9.11E-60	0.439390889	0.876	0.295	1.99E-55	GLRX	4	HIST1H4C_plasma cell
1777	5.69E-32	0.437481155	0.969	0.485	1.24E-27	PIM2	4	HIST1H4C_plasma cell
1778	1.64E-65	0.437374994	0.938	0.334	3.57E-61	NOL7	4	HIST1H4C_plasma cell
1779	3.67E-96	0.434867474	0.826	0.187	8.01E-92	ISOC2	4	HIST1H4C_plasma cell
1780	5.95E-46	0.433934409	0.965	0.448	1.30E-41	NDUFA13	4	HIST1H4C_plasma cell
1781	8.49E-76	0.433584541	0.838	0.223	1.85E-71	HIST1H1C	4	HIST1H4C_plasma cell
1782	4.04E-192	0.433535109	0.633	0.017	8.80E-188	GTSE1	4	HIST1H4C_plasma cell
1783	2.06E-77	0.433482294	0.892	0.267	4.49E-73	PPM1G	4	HIST1H4C_plasma cell
1784	1.82E-49	0.433407422	0.958	0.412	3.97E-45	GTF3A	4	HIST1H4C_plasma cell
1785	6.08E-96	0.433267453	0.892	0.224	1.33E-91	AP2S1	4	HIST1H4C_plasma cell
1786	4.19E-105	0.432406784	0.741	0.13	9.13E-101	EBNA1BP2	4	HIST1H4C_plasma cell
1787	5.84E-85	0.432266929	0.888	0.239	1.27E-80	HYOU1	4	HIST1H4C_plasma cell
1788	3.42E-73	0.431690067	0.892	0.273	7.46E-69	CCNC	4	HIST1H4C_plasma cell
1789	3.20E-46	0.431244469	0.961	0.452	6.98E-42	POMP	4	HIST1H4C_plasma cell
1790	2.58E-72	0.430091288	0.931	0.31	5.63E-68	TCEB1	4	HIST1H4C_plasma cell
1791	1.97E-77	0.430030994	0.892	0.247	4.31E-73	RPA3	4	HIST1H4C_plasma cell
1792	8.37E-36	0.429997568	0.988	0.568	1.83E-31	NDUFB11	4	HIST1H4C_plasma cell
1793	2.90E-82	0.429919933	0.896	0.253	6.31E-78	C19orf24	4	HIST1H4C_plasma cell
1794	2.47E-106	0.429784141	0.764	0.134	5.38E-102	PAICS	4	HIST1H4C_plasma cell
1795	7.87E-153	0.429559045	0.753	0.067	1.72E-148	KIF20B	4	HIST1H4C_plasma cell
1796	2.15E-182	0.429108781	0.656	0.029	4.68E-178	HMMR	4	HIST1H4C_plasma cell
1797	2.28E-71	0.428669606	0.865	0.258	4.98E-67	PIM1	4	HIST1H4C_plasma cell
1798	1.30E-42	0.426171537	0.969	0.39	2.83E-38	FKBP2	4	HIST1H4C_plasma cell
1799	3.73E-95	0.426025441	0.838	0.188	8.13E-91	AHCY	4	HIST1H4C_plasma cell
1800	4.45E-142	0.425990136	0.676	0.064	9.71E-138	TUBG1	4	HIST1H4C_plasma cell
1801	1.95E-146	0.425890489	0.737	0.076	4.24E-142	GMNN	4	HIST1H4C_plasma cell
1802	3.23E-90	0.425749835	0.876	0.229	7.05E-86	PSMC3	4	HIST1H4C_plasma cell
1803	1.10E-37	0.425642899	0.977	0.502	2.41E-33	CD99	4	HIST1H4C_plasma cell
1804	2.91E-89	0.423001071	0.822	0.203	6.34E-85	NUDT5	4	HIST1H4C_plasma cell
1805	5.47E-105	0.420941449	0.876	0.193	1.19E-100	MRPS16	4	HIST1H4C_plasma cell
1806	4.99E-54	0.41812775	0.942	0.377	1.09E-49	TOMM5	4	HIST1H4C_plasma cell
1807	5.32E-78	0.417639776	0.892	0.264	1.16E-73	TMEM167A	4	HIST1H4C_plasma cell
1808	9.70E-111	0.417270068	0.834	0.159	2.12E-106	STRA13	4	HIST1H4C_plasma cell
1809	7.90E-88	0.4169167	0.865	0.223	1.72E-83	MRPS15	4	HIST1H4C_plasma cell
1810	3.39E-140	0.416673121	0.768	0.088	7.38E-136	LMNB1	4	HIST1H4C_plasma cell
1811	2.28E-69	0.416076543	0.9	0.288	4.96E-65	COPB2	4	HIST1H4C_plasma cell
1812	4.38E-67	0.415471504	0.927	0.32	9.55E-63	NAA38	4	HIST1H4C_plasma cell
1813	1.37E-81	0.413682633	0.826	0.224	2.98E-77	YIF1B	4	HIST1H4C_plasma cell
1814	7.01E-69	0.412896133	0.865	0.259	1.53E-64	SNRPE	4	HIST1H4C_plasma cell
1815	4.15E-113	0.411976335	0.853	0.163	9.04E-109	MRPL37	4	HIST1H4C_plasma cell
1816	1.30E-64	0.410014061	0.919	0.306	2.82E-60	CBX3	4	HIST1H4C_plasma cell
1817	4.81E-122	0.40898661	0.691	0.083	1.05E-117	DNMT1	4	HIST1H4C_plasma cell
1818	1.01E-60	0.408883289	0.919	0.334	2.20E-56	NDUFA2	4	HIST1H4C_plasma cell
1819	2.67E-69	0.408495892	0.849	0.256	5.82E-65	GPI	4	HIST1H4C_plasma cell
1820	1.67E-89	0.408217733	0.552	0.074	3.64E-85	PHGDH	4	HIST1H4C_plasma cell
1821	1.85E-150	0.407939865	0.768	0.081	4.04E-146	SKA2	4	HIST1H4C_plasma cell
1822	1.22E-64	0.406730394	0.934	0.328	2.67E-60	CCT2	4	HIST1H4C_plasma cell
1823	5.33E-49	0.405538132	0.934	0.396	1.16E-44	ARHGDIA	4	HIST1H4C_plasma cell
1824	8.33E-71	0.403577968	0.888	0.28	1.82E-66	EMC7	4	HIST1H4C_plasma cell
1825	8.88E-62	0.402592389	0.942	0.353	1.94E-57	NDUFS7	4	HIST1H4C_plasma cell
1826	1.35E-75	0.402483302	0.857	0.256	2.94E-71	TIMM17A	4	HIST1H4C_plasma cell
1827	2.57E-119	0.401116893	0.78	0.124	5.61E-115	POP7	4	HIST1H4C_plasma cell
1828	7.34E-85	0.400969014	0.834	0.207	1.60E-80	UBA5	4	HIST1H4C_plasma cell
1829	2.16E-65	0.400610055	0.884	0.295	4.70E-61	MYEOV2	4	HIST1H4C_plasma cell
1830	2.63E-27	0.40054116	0.985	0.655	5.73E-23	NCL	4	HIST1H4C_plasma cell
1831	5.81E-118	0.399967764	0.784	0.129	1.27E-113	FH	4	HIST1H4C_plasma cell
1832	3.72E-67	0.399729004	0.892	0.298	8.11E-63	MZT2B	4	HIST1H4C_plasma cell
1833	2.01E-112	0.397861283	0.834	0.156	4.39E-108	MRPL27	4	HIST1H4C_plasma cell
1834	1.88E-51	0.397606761	0.927	0.375	4.10E-47	HAX1	4	HIST1H4C_plasma cell
1835	7.09E-90	0.396654399	0.819	0.195	1.55E-85	UQCC2	4	HIST1H4C_plasma cell
1836	6.11E-40	0.396367556	0.969	0.491	1.33E-35	HNRNPA3	4	HIST1H4C_plasma cell
1837	2.69E-62	0.395964414	0.915	0.333	5.86E-58	ANAPC5	4	HIST1H4C_plasma cell
1838	1.73E-104	0.393956826	0.807	0.16	3.78E-100	MYCBP	4	HIST1H4C_plasma cell
1839	1.16E-46	0.393300787	0.969	0.425	2.52E-42	MRPL52	4	HIST1H4C_plasma cell
1840	9.41E-88	0.39316002	0.842	0.206	2.05E-83	GTF3C6	4	HIST1H4C_plasma cell
1841	1.43E-61	0.39305688	0.857	0.284	3.11E-57	NHP2	4	HIST1H4C_plasma cell
1842	3.85E-55	0.392639593	0.915	0.331	8.39E-51	SEC14L1	4	HIST1H4C_plasma cell
1843	4.88E-53	0.392529101	0.911	0.353	1.06E-48	ARPC5L	4	HIST1H4C_plasma cell

1844	8.11E-97	0.39200137	0.819	0.174	1.77E-92	GMPPA	4	HIST1H4C_plasma cell
1845	2.08E-80	0.391964899	0.9	0.25	4.54E-76	PSMC5	4	HIST1H4C_plasma cell
1846	1.23E-77	0.391129896	0.849	0.233	2.68E-73	MRPS7	4	HIST1H4C_plasma cell
1847	1.35E-53	0.390984496	0.938	0.369	2.94E-49	RPS19BP1	4	HIST1H4C_plasma cell
1848	7.84E-74	0.390490024	0.896	0.266	1.71E-69	MDH1	4	HIST1H4C_plasma cell
1849	1.06E-27	0.389937313	0.958	0.522	2.31E-23	PDIA3	4	HIST1H4C_plasma cell
1850	2.45E-79	0.389862953	0.834	0.223	5.34E-75	MPC1	4	HIST1H4C_plasma cell
1851	7.98E-22	0.389202098	0.811	0.535	1.74E-17	IGKC	4	HIST1H4C_plasma cell
1852	1.82E-58	0.388020898	0.911	0.325	3.98E-54	MMADHC	4	HIST1H4C_plasma cell
1853	1.93E-45	0.387910034	0.958	0.424	4.20E-41	NDUFB2	4	HIST1H4C_plasma cell
1854	2.63E-58	0.387851995	0.896	0.311	5.74E-54	BANF1	4	HIST1H4C_plasma cell
1855	1.70E-48	0.387696757	0.942	0.37	3.70E-44	ELL2	4	HIST1H4C_plasma cell
1856	8.23E-45	0.387674799	0.961	0.441	1.79E-40	PSMB8	4	HIST1H4C_plasma cell
1857	9.35E-61	0.387504386	0.9	0.303	2.04E-56	ALG5	4	HIST1H4C_plasma cell
1858	1.72E-73	0.387173009	0.819	0.226	3.74E-69	REXO2	4	HIST1H4C_plasma cell
1859	1.17E-120	0.385765969	0.691	0.088	2.55E-116	CHAC2	4	HIST1H4C_plasma cell
1860	3.31E-163	0.385242264	0.722	0.058	7.23E-159	LRR1	4	HIST1H4C_plasma cell
1861	5.67E-200	0.384978495	0.653	0.017	1.24E-195	KIFC1	4	HIST1H4C_plasma cell
1862	7.88E-72	0.384397715	0.792	0.2	1.72E-67	SLBP	4	HIST1H4C_plasma cell
1863	9.37E-35	0.384280843	0.988	0.75	2.04E-30	ATP5L	4	HIST1H4C_plasma cell
1864	4.88E-60	0.383122666	0.869	0.308	1.06E-55	ADRM1	4	HIST1H4C_plasma cell
1865	2.41E-73	0.38229423	0.788	0.198	5.25E-69	TUBA1C	4	HIST1H4C_plasma cell
1866	4.21E-90	0.381412954	0.726	0.143	9.18E-86	GCSH	4	HIST1H4C_plasma cell
1867	3.94E-34	0.381162728	0.958	0.519	8.60E-30	SRSF2	4	HIST1H4C_plasma cell
1868	3.85E-56	0.380957801	0.919	0.337	8.39E-52	LRPAP1	4	HIST1H4C_plasma cell
1869	3.94E-69	0.38083037	0.884	0.278	8.59E-65	NDUFA12	4	HIST1H4C_plasma cell
1870	1.98E-84	0.380798965	0.884	0.237	4.31E-80	MRPL22	4	HIST1H4C_plasma cell
1871	1.53E-147	0.380100846	0.683	0.057	3.35E-143	RNASEH2A	4	HIST1H4C_plasma cell
1872	1.87E-39	0.37989162	0.931	0.378	4.08E-35	CD27	4	HIST1H4C_plasma cell
1873	2.84E-83	0.379737985	0.83	0.219	6.20E-79	CLPP	4	HIST1H4C_plasma cell
1874	1.57E-64	0.379487795	0.834	0.262	3.43E-60	EIF4G1	4	HIST1H4C_plasma cell
1875	8.46E-68	0.379327191	0.884	0.276	1.84E-63	FDX1	4	HIST1H4C_plasma cell
1876	3.02E-83	0.379030067	0.822	0.211	6.59E-79	MRPL14	4	HIST1H4C_plasma cell
1877	3.77E-88	0.378749774	0.849	0.209	8.22E-84	CKLF	4	HIST1H4C_plasma cell
1878	2.52E-83	0.378076242	0.795	0.182	5.50E-79	/IIR4435-2H(	4	HIST1H4C_plasma cell
1879	4.06E-117	0.377822824	0.753	0.106	8.86E-113	CKS1B	4	HIST1H4C_plasma cell
1880	5.70E-218	0.377602045	0.668	0.012	1.24E-213	TRIP13	4	HIST1H4C_plasma cell
1881	3.13E-82	0.377173089	0.772	0.182	6.82E-78	SLC25A4	4	HIST1H4C_plasma cell
1882	2.87E-69	0.376858085	0.861	0.262	6.25E-65	MRPL41	4	HIST1H4C_plasma cell
1883	6.77E-80	0.3765419	0.853	0.222	1.48E-75	TIMM13	4	HIST1H4C_plasma cell
1884	6.33E-60	0.37618212	0.888	0.313	1.38E-55	IFNAR2	4	HIST1H4C_plasma cell
1885	2.85E-61	0.375468144	0.911	0.317	6.23E-57	SMARCB1	4	HIST1H4C_plasma cell
1886	6.15E-91	0.375168107	0.691	0.128	1.34E-86	SEMA4A	4	HIST1H4C_plasma cell
1887	1.55E-53	0.374981191	0.888	0.322	3.39E-49	SHMT2	4	HIST1H4C_plasma cell
1888	7.93E-103	0.373675077	0.745	0.133	1.73E-98	RPL26L1	4	HIST1H4C_plasma cell
1889	5.43E-19	0.373606733	0.985	0.828	1.18E-14	NPM1	4	HIST1H4C_plasma cell
1890	2.66E-131	0.372658567	0.803	0.106	5.80E-127	NUDT1	4	HIST1H4C_plasma cell
1891	1.47E-89	0.371898878	0.857	0.209	3.20E-85	MCUR1	4	HIST1H4C_plasma cell
1892	2.31E-60	0.371836484	0.911	0.308	5.03E-56	SNRPF	4	HIST1H4C_plasma cell
1893	4.47E-52	0.371088844	0.923	0.36	9.75E-48	SNRPD3	4	HIST1H4C_plasma cell
1894	2.08E-82	0.370289029	0.838	0.203	4.53E-78	SLC1A5	4	HIST1H4C_plasma cell
1895	5.44E-61	0.369044843	0.903	0.311	1.19E-56	3ADD45GIP'	4	HIST1H4C_plasma cell
1896	8.79E-187	0.368656435	0.618	0.017	1.92E-182	NCAPG	4	HIST1H4C_plasma cell
1897	8.64E-94	0.368562422	0.776	0.161	1.88E-89	MRPS12	4	HIST1H4C_plasma cell
1898	1.41E-181	0.367741302	0.606	0.017	3.08E-177	ASF1B	4	HIST1H4C_plasma cell
1899	1.95E-24	0.366600924	0.996	0.857	4.24E-20	PFN1	4	HIST1H4C_plasma cell
1900	2.24E-32	0.366546764	0.961	0.528	4.88E-28	TRMT112	4	HIST1H4C_plasma cell
1901	4.62E-66	0.366470332	0.896	0.295	1.01E-61	UBE2L3	4	HIST1H4C_plasma cell
1902	1.40E-76	0.365632391	0.834	0.23	3.06E-72	HSD17B10	4	HIST1H4C_plasma cell
1903	6.43E-43	0.365255489	0.934	0.414	1.40E-38	NOP10	4	HIST1H4C_plasma cell
1904	3.53E-60	0.364682545	0.9	0.312	7.70E-56	COPZ1	4	HIST1H4C_plasma cell
1905	3.24E-78	0.364453012	0.9	0.246	7.06E-74	SPATS2	4	HIST1H4C_plasma cell
1906	4.04E-57	0.363550837	0.892	0.3	8.82E-53	DNAJC1	4	HIST1H4C_plasma cell
1907	2.74E-42	0.363367758	0.958	0.45	5.97E-38	SF3B2	4	HIST1H4C_plasma cell
1908	2.38E-124	0.36246685	0.718	0.09	5.18E-120	RAD51C	4	HIST1H4C_plasma cell
1909	1.21E-93	0.362051801	0.768	0.157	2.63E-89	PDXK	4	HIST1H4C_plasma cell
1910	1.04E-73	0.361374021	0.803	0.219	2.27E-69	PSMD2	4	HIST1H4C_plasma cell
1911	6.69E-78	0.360786543	0.857	0.232	1.46E-73	NCLN	4	HIST1H4C_plasma cell
1912	2.51E-26	0.36066608	0.988	0.735	5.48E-22	SERP1	4	HIST1H4C_plasma cell
1913	9.15E-193	0.36030415	0.606	0.012	2.00E-188	NCAPH	4	HIST1H4C_plasma cell
1914	9.67E-105	0.359959359	0.768	0.133	2.11E-100	SNRNP25	4	HIST1H4C_plasma cell

1915	1.01E-130	0.359805248	0.618	0.054	2.20E-126	EZH2	4	HIST1H4C_plasma cell
1916	7.21E-81	0.358801993	0.764	0.187	1.57E-76	TXNL4A	4	HIST1H4C_plasma cell
1917	1.74E-187	0.358592699	0.614	0.016	3.79E-183	NUF2	4	HIST1H4C_plasma cell
1918	1.37E-73	0.358591169	0.931	0.271	2.99E-69	ILF3	4	HIST1H4C_plasma cell
1919	2.00E-63	0.358536683	0.865	0.271	4.35E-59	EAF2	4	HIST1H4C_plasma cell
1920	1.70E-46	0.358408813	0.938	0.386	3.71E-42	PRDX6	4	HIST1H4C_plasma cell
1921	4.47E-54	0.358127896	0.946	0.347	9.76E-50	SDF4	4	HIST1H4C_plasma cell
1922	3.09E-76	0.35788908	0.857	0.239	6.75E-72	ETFA	4	HIST1H4C_plasma cell
1923	2.60E-85	0.357447068	0.846	0.211	5.66E-81	SSSCA1	4	HIST1H4C_plasma cell
1924	5.35E-48	0.356780855	0.907	0.36	1.17E-43	BSG	4	HIST1H4C_plasma cell
1925	2.16E-59	0.356725851	0.903	0.318	4.72E-55	DYNLRB1	4	HIST1H4C_plasma cell
1926	3.32E-63	0.356692894	0.934	0.316	7.24E-59	SRP54	4	HIST1H4C_plasma cell
1927	5.47E-30	0.356609744	0.954	0.494	1.19E-25	CHCHD10	4	HIST1H4C_plasma cell
1928	3.94E-81	0.356585029	0.815	0.198	8.58E-77	MARS	4	HIST1H4C_plasma cell
1929	9.34E-34	0.356522182	0.958	0.48	2.04E-29	SERBP1	4	HIST1H4C_plasma cell
1930	2.43E-58	0.356481932	0.869	0.295	5.29E-54	TXNDC15	4	HIST1H4C_plasma cell
1931	1.09E-92	0.356452151	0.714	0.135	2.37E-88	IFI27L1	4	HIST1H4C_plasma cell
1932	1.67E-32	0.354510768	0.954	0.481	3.64E-28	ATP5I	4	HIST1H4C_plasma cell
1933	9.54E-48	0.353227833	0.865	0.294	2.08E-43	NUCB2	4	HIST1H4C_plasma cell
1934	1.92E-186	0.352937339	0.583	0.01	4.19E-182	AURKB	4	HIST1H4C_plasma cell
1935	5.51E-59	0.352448066	0.795	0.245	1.20E-54	B4GALT3	4	HIST1H4C_plasma cell
1936	1.63E-42	0.35241707	0.915	0.391	3.55E-38	RBBP4	4	HIST1H4C_plasma cell
1937	1.65E-78	0.352394297	0.846	0.23	3.60E-74	MRPL40	4	HIST1H4C_plasma cell
1938	1.83E-78	0.352247928	0.849	0.221	3.98E-74	SNRPA	4	HIST1H4C_plasma cell
1939	2.53E-122	0.351696223	0.668	0.075	5.52E-118	CDKN2A	4	HIST1H4C_plasma cell
1940	8.54E-92	0.351306556	0.73	0.141	1.86E-87	EEF1E1	4	HIST1H4C_plasma cell
1941	3.89E-29	0.350985265	0.977	0.634	8.49E-25	ERP29	4	HIST1H4C_plasma cell
1942	2.09E-100	0.350380562	0.83	0.174	4.55E-96	PSMC2	4	HIST1H4C_plasma cell
1943	5.29E-61	0.350264397	0.888	0.284	1.15E-56	CBWD5	4	HIST1H4C_plasma cell
1944	8.26E-29	0.349962171	0.965	0.535	1.80E-24	ATP6V0B	4	HIST1H4C_plasma cell
1945	1.25E-54	0.349519337	0.907	0.33	2.73E-50	HNRNPR	4	HIST1H4C_plasma cell
1946	1.46E-97	0.349516696	0.788	0.158	3.18E-93	C11orf98	4	HIST1H4C_plasma cell
1947	1.32E-70	0.349358395	0.811	0.226	2.88E-66	ZNF593	4	HIST1H4C_plasma cell
1948	2.62E-130	0.349331985	0.653	0.062	5.71E-126	RRM1	4	HIST1H4C_plasma cell
1949	5.56E-112	0.349186832	0.788	0.128	1.21E-107	CARHSP1	4	HIST1H4C_plasma cell
1950	4.50E-83	0.349069629	0.842	0.193	9.82E-79	TMPO	4	HIST1H4C_plasma cell
1951	1.77E-79	0.349029204	0.521	0.074	3.86E-75	HIST1H2AE	4	HIST1H4C_plasma cell
1952	1.28E-84	0.348791723	0.811	0.196	2.79E-80	EMC6	4	HIST1H4C_plasma cell
1953	1.08E-68	0.348734395	0.884	0.283	2.34E-64	RNASEH2C	4	HIST1H4C_plasma cell
1954	3.54E-146	0.348428874	0.78	0.084	7.71E-142	VRK1	4	HIST1H4C_plasma cell
1955	1.30E-76	0.348022883	0.869	0.245	2.83E-72	NDUFS3	4	HIST1H4C_plasma cell
1956	4.14E-91	0.347960372	0.795	0.174	9.03E-87	MRPS14	4	HIST1H4C_plasma cell
1957	1.22E-112	0.347588817	0.618	0.068	2.66E-108	HELLS	4	HIST1H4C_plasma cell
1958	1.86E-73	0.347230089	0.896	0.258	4.07E-69	TRABD	4	HIST1H4C_plasma cell
1959	4.54E-31	0.346856248	0.992	0.787	9.90E-27	MYL6	4	HIST1H4C_plasma cell
1960	7.84E-62	0.346774357	0.641	0.155	1.71E-57	BIK	4	HIST1H4C_plasma cell
1961	1.06E-155	0.345952306	0.552	0.02	2.31E-151	CDT1	4	HIST1H4C_plasma cell
1962	7.80E-82	0.345772976	0.795	0.193	1.70E-77	HINT2	4	HIST1H4C_plasma cell
1963	2.28E-23	0.345274025	0.992	0.939	4.97E-19	HLA-A	4	HIST1H4C_plasma cell
1964	3.48E-74	0.344979866	0.795	0.209	7.60E-70	COPG1	4	HIST1H4C_plasma cell
1965	3.30E-102	0.344736314	0.699	0.109	7.20E-98	CDC25B	4	HIST1H4C_plasma cell
1966	1.24E-44	0.344728721	0.958	0.414	2.71E-40	C14orf166	4	HIST1H4C_plasma cell
1967	2.82E-93	0.344567251	0.857	0.195	6.16E-89	UCHL5	4	HIST1H4C_plasma cell
1968	4.83E-86	0.344027505	0.849	0.203	1.05E-81	STT3B	4	HIST1H4C_plasma cell
1969	1.19E-47	0.343100347	0.931	0.385	2.59E-43	XRCC6	4	HIST1H4C_plasma cell
1970	5.56E-187	0.342987361	0.587	0.011	1.21E-182	CDCA5	4	HIST1H4C_plasma cell
1971	1.16E-83	0.342214198	0.83	0.206	2.54E-79	PSMB5	4	HIST1H4C_plasma cell
1972	8.31E-78	0.341776165	0.78	0.19	1.81E-73	GSPT1	4	HIST1H4C_plasma cell
1973	3.57E-113	0.341215654	0.448	0.023	7.79E-109	HIST1H3B	4	HIST1H4C_plasma cell
1974	9.03E-56	0.340492109	0.861	0.299	1.97E-51	GORASP2	4	HIST1H4C_plasma cell
1975	4.46E-97	0.340306609	0.703	0.118	9.72E-93	TRAP1	4	HIST1H4C_plasma cell
1976	7.12E-74	0.340145232	0.861	0.249	1.55E-69	GPAA1	4	HIST1H4C_plasma cell
1977	3.65E-61	0.339422479	0.83	0.249	7.95E-57	ITGB7	4	HIST1H4C_plasma cell
1978	2.45E-72	0.339090507	0.834	0.234	5.35E-68	ATOX1	4	HIST1H4C_plasma cell
1979	4.77E-97	0.338030632	0.757	0.143	1.04E-92	YARS	4	HIST1H4C_plasma cell
1980	1.59E-22	0.337603347	0.977	0.811	3.47E-18	CFL1	4	HIST1H4C_plasma cell
1981	3.21E-65	0.337000847	0.853	0.262	6.99E-61	POLR2E	4	HIST1H4C_plasma cell
1982	2.79E-52	0.336860892	0.911	0.357	6.09E-48	SF3B6	4	HIST1H4C_plasma cell
1983	4.68E-109	0.336847287	0.707	0.1	1.02E-104	DNAJC9	4	HIST1H4C_plasma cell
1984	1.57E-73	0.336619921	0.861	0.247	3.42E-69	PDAP1	4	HIST1H4C_plasma cell
1985	1.36E-135	0.336586888	0.672	0.062	2.96E-131	RFC4	4	HIST1H4C_plasma cell

1986	1.07E-79	0.336502637	0.834	0.209	2.34E-75	SYNCRIP	4	HIST1H4C_plasma cell
1987	4.55E-47	0.336201555	0.942	0.386	9.93E-43	YWHAQ	4	HIST1H4C_plasma cell
1988	2.35E-69	0.336186767	0.822	0.239	5.12E-65	ACO2	4	HIST1H4C_plasma cell
1989	1.02E-114	0.3358131	0.544	0.045	2.22E-110	MCM6	4	HIST1H4C_plasma cell
1990	9.25E-86	0.335780906	0.788	0.181	2.02E-81	FTSJ1	4	HIST1H4C_plasma cell
1991	1.02E-64	0.33467782	0.834	0.241	2.22E-60	SLC44A1	4	HIST1H4C_plasma cell
1992	1.03E-134	0.334499934	0.579	0.04	2.24E-130	PRC1	4	HIST1H4C_plasma cell
1993	8.41E-84	0.334251202	0.78	0.178	1.83E-79	SDHB	4	HIST1H4C_plasma cell
1994	4.38E-56	0.334026179	0.737	0.215	9.54E-52	GAS6	4	HIST1H4C_plasma cell
1995	1.31E-52	0.333404744	0.857	0.29	2.85E-48	NASP	4	HIST1H4C_plasma cell
1996	1.40E-11	0.333189302	0.996	0.977	3.06E-07	TMSB10	4	HIST1H4C_plasma cell
1997	6.46E-57	0.333115904	0.873	0.295	1.41E-52	GNL3	4	HIST1H4C_plasma cell
1998	2.64E-46	0.332763499	0.946	0.418	5.77E-42	CSNK2B	4	HIST1H4C_plasma cell
1999	1.79E-77	0.33195926	0.502	0.069	3.91E-73	HIST1H2BF	4	HIST1H4C_plasma cell
2000	1.47E-81	0.331230501	0.826	0.2	3.20E-77	CASP3	4	HIST1H4C_plasma cell
2001	2.17E-28	0.330520428	0.942	0.438	4.73E-24	ERGIC3	4	HIST1H4C_plasma cell
2002	1.10E-74	0.330487346	0.826	0.221	2.39E-70	TMED3	4	HIST1H4C_plasma cell
2003	5.38E-102	0.32913093	0.784	0.141	1.17E-97	MRPL13	4	HIST1H4C_plasma cell
2004	7.91E-60	0.328338312	0.919	0.319	1.72E-55	SSBP1	4	HIST1H4C_plasma cell
2005	6.32E-52	0.328166983	0.907	0.345	1.38E-47	FAM96B	4	HIST1H4C_plasma cell
2006	1.04E-69	0.328044452	0.869	0.254	2.26E-65	FUNDC2	4	HIST1H4C_plasma cell
2007	5.71E-80	0.327886443	0.834	0.214	1.25E-75	TMEM160	4	HIST1H4C_plasma cell
2008	5.64E-87	0.327397171	0.707	0.137	1.23E-82	TOMM40	4	HIST1H4C_plasma cell
2009	1.55E-62	0.326983808	0.884	0.273	3.37E-58	CD59	4	HIST1H4C_plasma cell
2010	3.58E-67	0.32665307	0.768	0.201	7.82E-63	WARS	4	HIST1H4C_plasma cell
2011	3.66E-111	0.325977758	0.707	0.102	7.99E-107	GOT2	4	HIST1H4C_plasma cell
2012	7.09E-68	0.325247801	0.857	0.247	1.55E-63	CBWD2	4	HIST1H4C_plasma cell
2013	7.01E-42	0.324352869	0.691	0.224	1.53E-37	H1FX	4	HIST1H4C_plasma cell
2014	2.92E-108	0.323965822	0.776	0.127	6.36E-104	CBX5	4	HIST1H4C_plasma cell
2015	6.51E-59	0.32396194	0.888	0.297	1.42E-54	WBSCR22	4	HIST1H4C_plasma cell
2016	3.05E-74	0.323221035	0.853	0.237	6.64E-70	FAM136A	4	HIST1H4C_plasma cell
2017	1.56E-97	0.322755692	0.707	0.117	3.40E-93	MTFP1	4	HIST1H4C_plasma cell
2018	1.41E-124	0.322708158	0.73	0.09	3.07E-120	SAE1	4	HIST1H4C_plasma cell
2019	1.80E-19	0.322603271	0.981	0.612	3.93E-15	TMBIM6	4	HIST1H4C_plasma cell
2020	2.05E-33	0.322599713	0.969	0.518	4.47E-29	SRSF3	4	HIST1H4C_plasma cell
2021	9.29E-79	0.322520129	0.857	0.226	2.03E-74	DECR1	4	HIST1H4C_plasma cell
2022	5.75E-26	0.321164856	0.985	0.596	1.25E-21	EDF1	4	HIST1H4C_plasma cell
2023	8.34E-84	0.319831752	0.741	0.159	1.82E-79	AARS	4	HIST1H4C_plasma cell
2024	3.30E-94	0.318871664	0.722	0.131	7.19E-90	ACAT1	4	HIST1H4C_plasma cell
2025	5.91E-41	0.318317017	0.915	0.398	1.29E-36	VDAC2	4	HIST1H4C_plasma cell
2026	1.11E-30	0.317656467	0.973	0.533	2.42E-26	SNU13	4	HIST1H4C_plasma cell
2027	1.54E-79	0.317484308	0.857	0.224	3.35E-75	NUDT21	4	HIST1H4C_plasma cell
2028	1.13E-80	0.316892764	0.842	0.215	2.47E-76	COPS3	4	HIST1H4C_plasma cell
2029	2.62E-48	0.316850545	0.892	0.335	5.72E-44	TOR3A	4	HIST1H4C_plasma cell
2030	4.11E-44	0.316777326	0.919	0.39	8.97E-40	NEDD8	4	HIST1H4C_plasma cell
2031	7.18E-49	0.316721675	0.942	0.372	1.57E-44	COX17	4	HIST1H4C_plasma cell
2032	1.13E-92	0.316636319	0.73	0.133	2.47E-88	SMC1A	4	HIST1H4C_plasma cell
2033	1.30E-168	0.316149926	0.66	0.034	2.83E-164	CENPN	4	HIST1H4C_plasma cell
2034	1.14E-66	0.315542246	0.869	0.257	2.48E-62	CDV3	4	HIST1H4C_plasma cell
2035	1.37E-63	0.315498261	0.869	0.273	2.98E-59	YIF1A	4	HIST1H4C_plasma cell
2036	2.56E-63	0.315248107	0.792	0.226	5.59E-59	SEC24D	4	HIST1H4C_plasma cell
2037	8.67E-164	0.314887201	0.595	0.023	1.89E-159	TROAP	4	HIST1H4C_plasma cell
2038	3.02E-41	0.314866941	0.969	0.428	6.59E-37	PARP1	4	HIST1H4C_plasma cell
2039	1.91E-56	0.314618473	0.88	0.31	4.18E-52	SEC63	4	HIST1H4C_plasma cell
2040	2.23E-37	0.314514524	0.942	0.418	4.87E-33	REEP5	4	HIST1H4C_plasma cell
2041	1.34E-102	0.314082556	0.78	0.143	2.92E-98	RUVBL2	4	HIST1H4C_plasma cell
2042	1.37E-48	0.313888296	0.834	0.312	2.99E-44	MRPL20	4	HIST1H4C_plasma cell
2043	4.57E-66	0.313738669	0.88	0.278	9.97E-62	EMC4	4	HIST1H4C_plasma cell
2044	1.46E-25	0.313658334	0.969	0.56	3.18E-21	RPS27L	4	HIST1H4C_plasma cell
2045	6.31E-53	0.313465122	0.931	0.342	1.38E-48	NDUFB1	4	HIST1H4C_plasma cell
2046	3.61E-69	0.313384552	0.884	0.275	7.87E-65	MRPL43	4	HIST1H4C_plasma cell
2047	1.17E-53	0.312860525	0.903	0.315	2.55E-49	ABRACL	4	HIST1H4C_plasma cell
2048	1.28E-57	0.312345796	0.849	0.273	2.78E-53	SLC38A10	4	HIST1H4C_plasma cell
2049	2.18E-95	0.311997368	0.668	0.109	4.76E-91	MRPL17	4	HIST1H4C_plasma cell
2050	6.64E-55	0.311541577	0.896	0.314	1.45E-50	TOMM22	4	HIST1H4C_plasma cell
2051	1.31E-34	0.311531933	0.942	0.445	2.86E-30	JTB	4	HIST1H4C_plasma cell
2052	2.42E-71	0.310966335	0.865	0.254	5.28E-67	DCTN3	4	HIST1H4C_plasma cell
2053	4.74E-80	0.31088422	0.753	0.172	1.03E-75	LAP3	4	HIST1H4C_plasma cell
2054	2.42E-89	0.310856521	0.822	0.18	5.27E-85	USP14	4	HIST1H4C_plasma cell
2055	4.62E-81	0.310801018	0.741	0.163	1.01E-76	CLPTM1L	4	HIST1H4C_plasma cell
2056	3.12E-56	0.310276723	0.857	0.291	6.80E-52	RNH1	4	HIST1H4C_plasma cell

2057	1.29E-90	0.310121112	0.741	0.143	2.82E-86	TARS	4	HIST1H4C_plasma cell
2058	8.80E-91	0.309979652	0.741	0.144	1.92E-86	POLR2H	4	HIST1H4C_plasma cell
2059	5.09E-78	0.309903171	0.784	0.19	1.11E-73	ALG3	4	HIST1H4C_plasma cell
2060	8.28E-53	0.309552237	0.892	0.336	1.81E-48	ATP5H	4	HIST1H4C_plasma cell
2061	1.58E-39	0.309265569	0.907	0.384	3.44E-35	RALY	4	HIST1H4C_plasma cell
2062	2.25E-154	0.309237048	0.541	0.018	4.91E-150	CDCA3	4	HIST1H4C_plasma cell
2063	2.05E-62	0.309014632	0.861	0.275	4.48E-58	MCTS1	4	HIST1H4C_plasma cell
2064	5.61E-103	0.308844874	0.583	0.069	1.22E-98	CTNNAL1	4	HIST1H4C_plasma cell
2065	3.94E-66	0.308498432	0.876	0.264	8.60E-62	LSM2	4	HIST1H4C_plasma cell
2066	8.74E-82	0.307980489	0.734	0.159	1.91E-77	MRPL36	4	HIST1H4C_plasma cell
2067	3.51E-18	0.307966286	0.981	0.69	7.65E-14	ISG20	4	HIST1H4C_plasma cell
2068	5.61E-37	0.307579607	0.973	0.457	1.22E-32	C11orf31	4	HIST1H4C_plasma cell
2069	9.44E-74	0.306718856	0.772	0.195	2.06E-69	MRPL34	4	HIST1H4C_plasma cell
2070	4.08E-55	0.306672442	0.931	0.336	8.90E-51	ENY2	4	HIST1H4C_plasma cell
2071	2.26E-80	0.305843312	0.768	0.179	4.92E-76	DPM2	4	HIST1H4C_plasma cell
2072	5.30E-67	0.305761949	0.822	0.232	1.16E-62	PSMB7	4	HIST1H4C_plasma cell
2073	5.67E-53	0.305447453	0.9	0.326	1.24E-48	RBM17	4	HIST1H4C_plasma cell
2074	2.72E-74	0.305377782	0.795	0.206	5.93E-70	SUCLG1	4	HIST1H4C_plasma cell
2075	1.12E-155	0.304661171	0.533	0.016	2.45E-151	DIAPH3	4	HIST1H4C_plasma cell
2076	1.90E-132	0.304106193	0.571	0.037	4.14E-128	ATAD2	4	HIST1H4C_plasma cell
2077	2.41E-99	0.302668334	0.807	0.155	5.26E-95	ADSL	4	HIST1H4C_plasma cell
2078	1.13E-129	0.302608234	0.556	0.036	2.47E-125	FBXO5	4	HIST1H4C_plasma cell
2079	8.80E-92	0.302598019	0.772	0.161	1.92E-87	MRPS18C	4	HIST1H4C_plasma cell
2080	4.70E-60	0.302552403	0.822	0.251	1.02E-55	SLC39A7	4	HIST1H4C_plasma cell
2081	1.49E-30	0.301090177	0.911	0.458	3.25E-26	TUBB4B	4	HIST1H4C_plasma cell
2082	8.55E-150	0.300535683	0.506	0.014	1.87E-145	CLSPN	4	HIST1H4C_plasma cell
2083	6.31E-42	0.300333351	0.95	0.405	1.38E-37	DBI	4	HIST1H4C_plasma cell
2084	6.34E-84	0.299588312	0.83	0.2	1.38E-79	MRPL38	4	HIST1H4C_plasma cell
2085	6.51E-43	0.299348801	0.927	0.388	1.42E-38	SHFM1	4	HIST1H4C_plasma cell
2086	6.61E-73	0.299265852	0.541	0.087	1.44E-68	HIST1H2AM	4	HIST1H4C_plasma cell
2087	1.36E-75	0.298622859	0.849	0.219	2.97E-71	CCT5	4	HIST1H4C_plasma cell
2088	7.73E-26	0.298497799	0.965	0.522	1.68E-21	VCP	4	HIST1H4C_plasma cell
2089	5.34E-35	0.298322264	0.911	0.388	1.16E-30	TM9SF2	4	HIST1H4C_plasma cell
2090	1.40E-53	0.298060766	0.896	0.31	3.06E-49	TAP1	4	HIST1H4C_plasma cell
2091	9.15E-72	0.297910197	0.865	0.245	2.00E-67	RTN3	4	HIST1H4C_plasma cell
2092	2.29E-178	0.297752122	0.51	0.003	5.00E-174	CENPA	4	HIST1H4C_plasma cell
2093	1.69E-27	0.29754082	0.981	0.556	3.69E-23	YWHAB	4	HIST1H4C_plasma cell
2094	1.88E-48	0.297389665	0.915	0.344	4.11E-44	AKR1A1	4	HIST1H4C_plasma cell
2095	1.43E-74	0.297382262	0.822	0.211	3.13E-70	MRPL4	4	HIST1H4C_plasma cell
2096	5.94E-105	0.296980114	0.695	0.103	1.29E-100	HADH	4	HIST1H4C_plasma cell
2097	2.88E-50	0.296827719	0.865	0.306	6.29E-46	UBE2G1	4	HIST1H4C_plasma cell
2098	1.60E-66	0.296640915	0.88	0.264	3.50E-62	GTF2A2	4	HIST1H4C_plasma cell
2099	1.80E-87	0.296563241	0.672	0.12	3.93E-83	ALG14	4	HIST1H4C_plasma cell
2100	1.83E-81	0.295388609	0.807	0.189	3.99E-77	NDUFC2	4	HIST1H4C_plasma cell
2101	1.94E-68	0.295199902	0.861	0.254	4.23E-64	SSNA1	4	HIST1H4C_plasma cell
2102	2.49E-47	0.295183639	0.931	0.362	5.43E-43	TMEM256	4	HIST1H4C_plasma cell
2103	4.63E-74	0.294459141	0.803	0.201	1.01E-69	CHST12	4	HIST1H4C_plasma cell
2104	1.33E-79	0.294180096	0.73	0.154	2.90E-75	SLC1A4	4	HIST1H4C_plasma cell
2105	8.94E-91	0.294051715	0.641	0.101	1.95E-86	C19orf48	4	HIST1H4C_plasma cell
2106	3.27E-52	0.293932855	0.649	0.177	7.13E-48	ABCB9	4	HIST1H4C_plasma cell
2107	5.24E-28	0.29379541	0.946	0.466	1.14E-23	GUK1	4	HIST1H4C_plasma cell
2108	1.01E-84	0.293573271	0.378	0.026	2.21E-80	HIST1H3C	4	HIST1H4C_plasma cell
2109	3.60E-75	0.293466204	0.533	0.081	7.86E-71	HIST1H2AG	4	HIST1H4C_plasma cell
2110	1.06E-68	0.292554494	0.83	0.233	2.31E-64	SRA1	4	HIST1H4C_plasma cell
2111	9.20E-123	0.292382726	0.672	0.074	2.01E-118	TFDP1	4	HIST1H4C_plasma cell
2112	2.89E-141	0.292315113	0.49	0.016	6.29E-137	CDCA8	4	HIST1H4C_plasma cell
2113	4.66E-46	0.291752749	0.923	0.368	1.02E-41	NOP58	4	HIST1H4C_plasma cell
2114	4.83E-48	0.291477292	0.799	0.278	1.05E-43	ARFGAP3	4	HIST1H4C_plasma cell
2115	2.08E-88	0.290853508	0.695	0.126	4.54E-84	IL6R	4	HIST1H4C_plasma cell
2116	3.17E-78	0.290821231	0.792	0.188	6.91E-74	EIF2S1	4	HIST1H4C_plasma cell
2117	8.28E-110	0.289845816	0.49	0.035	1.81E-105	SLC43A3	4	HIST1H4C_plasma cell
2118	8.43E-71	0.289820983	0.826	0.232	1.84E-66	PSMD11	4	HIST1H4C_plasma cell
2119	1.45E-81	0.289062572	0.822	0.195	3.16E-77	MPC2	4	HIST1H4C_plasma cell
2120	2.19E-23	0.288517774	0.969	0.655	4.77E-19	SUMO2	4	HIST1H4C_plasma cell
2121	2.23E-150	0.288323093	0.568	0.025	4.86E-146	CASC5	4	HIST1H4C_plasma cell
2122	6.41E-24	0.288216077	0.973	0.601	1.40E-19	HMGN1	4	HIST1H4C_plasma cell
2123	5.61E-22	0.288162517	1	0.844	1.22E-17	ATP5E	4	HIST1H4C_plasma cell
2124	7.21E-60	0.287936384	0.869	0.291	1.57E-55	NAA10	4	HIST1H4C_plasma cell
2125	1.71E-153	0.287839446	0.517	0.014	3.72E-149	DEPDC1B	4	HIST1H4C_plasma cell
2126	3.20E-60	0.287600469	0.846	0.258	6.97E-56	SCAMP2	4	HIST1H4C_plasma cell
2127	1.78E-83	0.287486533	0.815	0.186	3.89E-79	PSMD14	4	HIST1H4C_plasma cell

2128	1.14E-21	0.287272712	0.969	0.636	2.48E-17	SNRPD2	4	HIST1H4C_plasma cell
2129	6.31E-159	0.287216317	0.525	0.013	1.38E-154	CDCA2	4	HIST1H4C_plasma cell
2130	1.80E-51	0.287188865	0.884	0.306	3.92E-47	DERL2	4	HIST1H4C_plasma cell
2131	1.20E-74	0.286676903	0.83	0.217	2.62E-70	ATG3	4	HIST1H4C_plasma cell
2132	3.55E-78	0.286604286	0.656	0.123	7.75E-74	CCR2	4	HIST1H4C_plasma cell
2133	7.16E-72	0.286452873	0.734	0.176	1.56E-67	TMEM214	4	HIST1H4C_plasma cell
2134	1.29E-87	0.286131158	0.819	0.183	2.82E-83	PPP1R7	4	HIST1H4C_plasma cell
2135	2.42E-55	0.285883671	0.776	0.235	5.28E-51	CPNE5	4	HIST1H4C_plasma cell
2136	3.08E-74	0.285527163	0.834	0.224	6.71E-70	RPS6KB2	4	HIST1H4C_plasma cell
2137	6.68E-45	0.285026095	0.784	0.277	1.46E-40	ITGB1	4	HIST1H4C_plasma cell
2138	3.82E-84	0.284589409	0.822	0.191	8.34E-80	COMMD1	4	HIST1H4C_plasma cell
2139	6.11E-82	0.284559784	0.722	0.149	1.33E-77	TAF10	4	HIST1H4C_plasma cell
2140	9.11E-65	0.284522228	0.707	0.183	1.99E-60	NOLC1	4	HIST1H4C_plasma cell
2141	2.90E-179	0.284174957	0.56	0.01	6.32E-175	SPAG5	4	HIST1H4C_plasma cell
2142	3.11E-34	0.284001177	0.942	0.447	6.77E-30	GMFG	4	HIST1H4C_plasma cell
2143	3.33E-22	0.283871505	0.973	0.593	7.27E-18	PTGES3	4	HIST1H4C_plasma cell
2144	4.70E-48	0.283140311	0.873	0.318	1.02E-43	ERGIC2	4	HIST1H4C_plasma cell
2145	1.30E-82	0.283105487	0.807	0.185	2.83E-78	MRPL23	4	HIST1H4C_plasma cell
2146	8.42E-82	0.282944282	0.795	0.186	1.84E-77	CCDC124	4	HIST1H4C_plasma cell
2147	2.03E-85	0.282257732	0.737	0.152	4.43E-81	PDHA1	4	HIST1H4C_plasma cell
2148	1.40E-54	0.282048679	0.873	0.297	3.05E-50	NUCB1	4	HIST1H4C_plasma cell
2149	9.38E-82	0.281437315	0.795	0.189	2.05E-77	PIN1	4	HIST1H4C_plasma cell
2150	1.36E-37	0.281296871	0.367	0.081	2.97E-33	PTP4A3	4	HIST1H4C_plasma cell
2151	1.00E-38	0.281153432	0.78	0.282	2.19E-34	S100A10	4	HIST1H4C_plasma cell
2152	3.02E-96	0.280571182	0.51	0.052	6.58E-92	SPN	4	HIST1H4C_plasma cell
2153	2.56E-42	0.280364673	0.714	0.238	5.59E-38	CHPF	4	HIST1H4C_plasma cell
2154	5.93E-66	0.280309325	0.869	0.26	1.29E-61	ELAVL1	4	HIST1H4C_plasma cell
2155	8.17E-78	0.280037518	0.741	0.165	1.78E-73	THOC7	4	HIST1H4C_plasma cell
2156	3.69E-80	0.279802646	0.788	0.188	8.04E-76	CMC2	4	HIST1H4C_plasma cell
2157	1.00E-64	0.279291885	0.9	0.271	2.19E-60	LINC00152	4	HIST1H4C_plasma cell
2158	1.18E-72	0.27886413	0.699	0.157	2.58E-68	BAK1	4	HIST1H4C_plasma cell
2159	5.30E-64	0.278765074	0.722	0.186	1.16E-59	GRK6	4	HIST1H4C_plasma cell
2160	5.62E-30	0.278384558	0.942	0.478	1.23E-25	PSMB9	4	HIST1H4C_plasma cell
2161	2.92E-73	0.278318279	0.71	0.161	6.36E-69	HIGD1A	4	HIST1H4C_plasma cell
2162	4.55E-65	0.278096681	0.884	0.273	9.93E-61	PCMT1	4	HIST1H4C_plasma cell
2163	5.68E-78	0.277964577	0.834	0.214	1.24E-73	TRAPPC2L	4	HIST1H4C_plasma cell
2164	1.33E-23	0.277704226	0.958	0.548	2.90E-19	UBL5	4	HIST1H4C_plasma cell
2165	1.71E-32	0.277146542	0.977	0.483	3.73E-28	WDR83OS	4	HIST1H4C_plasma cell
2166	2.26E-90	0.276946565	0.73	0.136	4.93E-86	PSMD1	4	HIST1H4C_plasma cell
2167	1.31E-57	0.276903938	0.857	0.277	2.85E-53	MORF4L2	4	HIST1H4C_plasma cell
2168	2.48E-110	0.276742973	0.405	0.016	5.41E-106	CENPE	4	HIST1H4C_plasma cell
2169	5.69E-87	0.276196588	0.66	0.116	1.24E-82	SNF8	4	HIST1H4C_plasma cell
2170	3.24E-68	0.276099331	0.792	0.207	7.07E-64	CTSC	4	HIST1H4C_plasma cell
2171	2.02E-10	0.27601838	1	0.988	4.40E-06	ACTB	4	HIST1H4C_plasma cell
2172	5.60E-69	0.275999439	0.811	0.217	1.22E-64	SAR1A	4	HIST1H4C_plasma cell
2173	1.84E-39	0.275897779	0.931	0.397	4.01E-35	C19orf53	4	HIST1H4C_plasma cell
2174	2.30E-60	0.275262513	0.869	0.278	5.02E-56	RAB8A	4	HIST1H4C_plasma cell
2175	1.64E-46	0.274738369	0.764	0.262	3.58E-42	SLAMF7	4	HIST1H4C_plasma cell
2176	2.33E-143	0.274545319	0.571	0.031	5.08E-139	PARPBP	4	HIST1H4C_plasma cell
2177	2.59E-25	0.274035455	0.988	0.64	5.66E-21	SRGN	4	HIST1H4C_plasma cell
2178	9.88E-135	0.273405949	0.606	0.043	2.15E-130	NCAPD3	4	HIST1H4C_plasma cell
2179	2.58E-99	0.273301492	0.722	0.122	5.62E-95	ANAPC15	4	HIST1H4C_plasma cell
2180	2.79E-55	0.272937661	0.788	0.246	6.08E-51	ADI1	4	HIST1H4C_plasma cell
2181	6.56E-73	0.272906055	0.714	0.17	1.43E-68	SRP68	4	HIST1H4C_plasma cell
2182	4.02E-58	0.272862728	0.78	0.22	8.77E-54	TPST2	4	HIST1H4C_plasma cell
2183	2.11E-16	0.272708686	1	0.991	4.60E-12	PTMA	4	HIST1H4C_plasma cell
2184	2.31E-87	0.272521952	0.795	0.165	5.03E-83	H2AFY	4	HIST1H4C_plasma cell
2185	1.57E-103	0.2724053	0.61	0.076	3.43E-99	UCK2	4	HIST1H4C_plasma cell
2186	9.46E-31	0.271891734	0.51	0.164	2.06E-26	ATF5	4	HIST1H4C_plasma cell
2187	5.55E-40	0.271858304	0.919	0.379	1.21E-35	EWSR1	4	HIST1H4C_plasma cell
2188	4.80E-80	0.271718662	0.653	0.12	1.05E-75	DENND6B	4	HIST1H4C_plasma cell
2189	3.31E-83	0.271689471	0.78	0.167	7.22E-79	POLE3	4	HIST1H4C_plasma cell
2190	1.67E-153	0.271196309	0.475	0.007	3.65E-149	HJURP	4	HIST1H4C_plasma cell
2191	3.96E-100	0.270861297	0.737	0.124	8.64E-96	MRPL15	4	HIST1H4C_plasma cell
2192	1.62E-99	0.270698886	0.645	0.091	3.54E-95	BRI3BP	4	HIST1H4C_plasma cell
2193	4.88E-75	0.270633997	0.761	0.179	1.06E-70	PMVK	4	HIST1H4C_plasma cell
2194	2.39E-46	0.269947835	0.896	0.336	5.21E-42	ATRAID	4	HIST1H4C_plasma cell
2195	5.87E-41	0.269871471	0.923	0.382	1.28E-36	SEC11A	4	HIST1H4C_plasma cell
2196	9.95E-69	0.269856403	0.803	0.217	2.17E-64	HSBP1	4	HIST1H4C_plasma cell
2197	2.97E-96	0.269736855	0.622	0.085	6.48E-92	POLR3K	4	HIST1H4C_plasma cell
2198	9.62E-152	0.269464926	0.51	0.014	2.10E-147	NDC80	4	HIST1H4C_plasma cell

2199	3.40E-48	0.26911879	0.792	0.267	7.41E-44	HSPA13	4	HIST1H4C_plasma cell
2200	8.11E-32	0.268945836	0.938	0.431	1.77E-27	UFM1	4	HIST1H4C_plasma cell
2201	1.25E-109	0.268823077	0.598	0.063	2.73E-105	LIG1	4	HIST1H4C_plasma cell
2202	8.68E-48	0.26841125	0.903	0.345	1.89E-43	CCT3	4	HIST1H4C_plasma cell
2203	3.64E-65	0.268239338	0.807	0.223	7.94E-61	NUTF2	4	HIST1H4C_plasma cell
2204	2.92E-100	0.267905688	0.726	0.121	6.37E-96	DAZAP1	4	HIST1H4C_plasma cell
2205	2.58E-100	0.267840893	0.44	0.029	5.63E-96	MCM4	4	HIST1H4C_plasma cell
2206	2.42E-58	0.267823925	0.506	0.097	5.28E-54	HIST1H1D	4	HIST1H4C_plasma cell
2207	1.32E-80	0.267683002	0.78	0.178	2.88E-76	VBP1	4	HIST1H4C_plasma cell
2208	1.09E-79	0.2667355	0.749	0.161	2.37E-75	MRPL28	4	HIST1H4C_plasma cell
2209	2.06E-42	0.266354832	0.903	0.357	4.50E-38	PHPT1	4	HIST1H4C_plasma cell
2210	3.35E-132	0.266261654	0.49	0.021	7.31E-128	CHEK1	4	HIST1H4C_plasma cell
2211	7.10E-65	0.26612617	0.857	0.264	1.55E-60	POLR2K	4	HIST1H4C_plasma cell
2212	1.94E-82	0.266027276	0.486	0.057	4.24E-78	HIST1H2AL	4	HIST1H4C_plasma cell
2213	2.62E-54	0.265851103	0.834	0.28	5.71E-50	TMEM147	4	HIST1H4C_plasma cell
2214	2.81E-38	0.265270795	0.923	0.396	6.13E-34	EIF3A	4	HIST1H4C_plasma cell
2215	1.66E-18	0.265128847	0.992	0.736	3.62E-14	RPS17	4	HIST1H4C_plasma cell
2216	1.76E-38	0.2647937	0.514	0.142	3.84E-34	RNU12	4	HIST1H4C_plasma cell
2217	4.38E-67	0.264122421	0.745	0.185	9.56E-63	ENTPD1	4	HIST1H4C_plasma cell
2218	3.10E-50	0.264079306	0.51	0.114	6.76E-46	HIST1H2BC	4	HIST1H4C_plasma cell
2219	9.62E-68	0.263905799	0.741	0.18	2.10E-63	CAPN2	4	HIST1H4C_plasma cell
2220	3.75E-91	0.263321997	0.776	0.154	8.18E-87	TIMM10	4	HIST1H4C_plasma cell
2221	2.98E-77	0.262660349	0.803	0.189	6.51E-73	HSPB11	4	HIST1H4C_plasma cell
2222	3.71E-54	0.262563088	0.873	0.305	8.09E-50	AP2M1	4	HIST1H4C_plasma cell
2223	3.09E-31	0.262276517	0.938	0.455	6.74E-27	ARL6IP4	4	HIST1H4C_plasma cell
2224	6.14E-44	0.261993142	0.876	0.337	1.34E-39	ERP44	4	HIST1H4C_plasma cell
2225	7.91E-101	0.261568588	0.714	0.112	1.72E-96	ICT1	4	HIST1H4C_plasma cell
2226	4.68E-182	0.261523681	0.533	0.005	1.02E-177	SPC25	4	HIST1H4C_plasma cell
2227	7.91E-80	0.261395868	0.726	0.155	1.72E-75	CHCHD1	4	HIST1H4C_plasma cell
2228	2.14E-78	0.261265619	0.753	0.17	4.67E-74	DEF8	4	HIST1H4C_plasma cell
2229	4.26E-82	0.26037427	0.722	0.147	9.28E-78	FKBP3	4	HIST1H4C_plasma cell
2230	1.43E-104	0.26036956	0.695	0.102	3.12E-100	PAFAH1B3	4	HIST1H4C_plasma cell
2231	4.52E-70	0.260175357	0.83	0.227	9.85E-66	MRPL3	4	HIST1H4C_plasma cell
2232	1.87E-98	0.260069984	0.61	0.081	4.08E-94	GLRX5	4	HIST1H4C_plasma cell
2233	2.72E-56	0.259886475	0.884	0.293	5.92E-52	VPS29	4	HIST1H4C_plasma cell
2234	1.93E-39	0.259866881	0.88	0.351	4.22E-35	SLC3A2	4	HIST1H4C_plasma cell
2235	3.02E-63	0.259577386	0.799	0.228	6.58E-59	PIGT	4	HIST1H4C_plasma cell
2236	5.36E-64	0.259139941	0.784	0.217	1.17E-59	PSMD3	4	HIST1H4C_plasma cell
2237	1.36E-73	0.258987332	0.83	0.211	2.98E-69	MRPL16	4	HIST1H4C_plasma cell
2238	2.06E-77	0.258458981	0.714	0.15	4.50E-73	DPAGT1	4	HIST1H4C_plasma cell
2239	1.79E-80	0.258399958	0.637	0.113	3.91E-76	POLD2	4	HIST1H4C_plasma cell
2240	9.31E-75	0.25783243	0.664	0.136	2.03E-70	PRPF19	4	HIST1H4C_plasma cell
2241	3.63E-71	0.257067834	0.718	0.167	7.92E-67	IARS	4	HIST1H4C_plasma cell
2242	1.09E-61	0.256664046	0.737	0.198	2.37E-57	SEC24A	4	HIST1H4C_plasma cell
2243	5.62E-60	0.2558636	0.834	0.26	1.23E-55	UQCRC2	4	HIST1H4C_plasma cell
2244	5.00E-83	0.255788442	0.676	0.124	1.09E-78	FXN	4	HIST1H4C_plasma cell
2245	5.15E-68	0.255598733	0.788	0.213	1.12E-63	MRPL55	4	HIST1H4C_plasma cell
2246	6.75E-161	0.255582243	0.533	0.014	1.47E-156	OIP5	4	HIST1H4C_plasma cell
2247	1.30E-72	0.254544854	0.676	0.146	2.84E-68	COMTD1	4	HIST1H4C_plasma cell
2248	3.42E-123	0.254492231	0.444	0.016	7.45E-119	CDC45	4	HIST1H4C_plasma cell
2249	1.77E-56	0.254192238	0.876	0.285	3.86E-52	CLTA	4	HIST1H4C_plasma cell
2250	9.19E-51	0.253933326	0.896	0.321	2.00E-46	EIF1AX	4	HIST1H4C_plasma cell
2251	1.34E-38	0.253925102	0.919	0.409	2.91E-34	RAC1	4	HIST1H4C_plasma cell
2252	1.12E-51	0.253281767	0.88	0.313	2.43E-47	ACP1	4	HIST1H4C_plasma cell
2253	5.42E-111	0.253053574	0.382	0.011	1.18E-106	UHRF1	4	HIST1H4C_plasma cell
2254	2.97E-93	0.252961384	0.788	0.15	6.48E-89	HAT1	4	HIST1H4C_plasma cell
2255	9.56E-49	0.252752531	0.938	0.349	2.09E-44	TMEM50A	4	HIST1H4C_plasma cell
2256	2.67E-46	0.252410133	0.931	0.351	5.82E-42	ARPC5	4	HIST1H4C_plasma cell
2257	8.28E-107	0.252234279	0.664	0.086	1.81E-102	ACAT2	4	HIST1H4C_plasma cell
2258	2.37E-32	0.251934133	0.521	0.17	5.16E-28	SDC1	4	HIST1H4C_plasma cell
2259	3.55E-39	0.251885852	0.9	0.37	7.75E-35	PRMT1	4	HIST1H4C_plasma cell
2260	6.13E-85	0.251807161	0.707	0.135	1.34E-80	SFXN1	4	HIST1H4C_plasma cell
2261	8.78E-74	0.250863655	0.749	0.175	1.91E-69	DCUN1D5	4	HIST1H4C_plasma cell
2262	1.19E-68	0.250376014	0.826	0.227	2.60E-64	PSMD9	4	HIST1H4C_plasma cell
2263	1.49E-10	-0.250949088	0.486	0.215	3.24E-06	HMHA1	4	HIST1H4C_plasma cell
2264	7.60E-09	-0.251278385	0.425	0.198	0.00016566	INTS6	4	HIST1H4C_plasma cell
2265	1.18E-09	-0.251670844	0.683	0.332	2.58E-05	LITAF	4	HIST1H4C_plasma cell
2266	2.26E-12	-0.251992897	0.996	0.985	4.93E-08	TPT1	4	HIST1H4C_plasma cell
2267	1.14E-11	-0.252493135	0.815	0.392	2.49E-07	NSA2	4	HIST1H4C_plasma cell
2268	5.23E-08	-0.25257348	0.556	0.278	0.001139913	CAST	4	HIST1H4C_plasma cell
2269	3.71E-08	-0.254182909	0.344	0.156	0.000809802	SMC6	4	HIST1H4C_plasma cell

2270	2.80E-10	-0.254671514	0.517	0.244	6.12E-06	TNFAIP3	4	HIST1H4C_plasma cell
2271	4.43E-12	-0.256829401	0.815	0.401	9.65E-08	TCF4	4	HIST1H4C_plasma cell
2272	1.33E-06	-0.258072548	0.799	0.439	0.029112429	LAPTM4A	4	HIST1H4C_plasma cell
2273	2.15E-06	-0.258641037	0.375	0.187	0.046959137	SPI1	4	HIST1H4C_plasma cell
2274	1.57E-10	-0.259031347	0.595	0.28	3.43E-06	MEAF6	4	HIST1H4C_plasma cell
2275	5.22E-09	-0.260375522	0.645	0.325	0.000113769	PTP4A1	4	HIST1H4C_plasma cell
2276	1.59E-08	-0.260516747	0.741	0.381	0.00034581	SP140	4	HIST1H4C_plasma cell
2277	7.89E-10	-0.262031774	0.68	0.339	1.72E-05	MYCBP2	4	HIST1H4C_plasma cell
2278	3.15E-12	-0.262161435	0.753	0.345	6.88E-08	PAIP2	4	HIST1H4C_plasma cell
2279	2.95E-08	-0.262521154	0.741	0.374	0.000644041	WHSC1L1	4	HIST1H4C_plasma cell
2280	1.63E-07	-0.265169255	0.822	0.442	0.003554346	MYL12B	4	HIST1H4C_plasma cell
2281	1.94E-07	-0.26582966	0.378	0.182	0.004227663	TMEM154	4	HIST1H4C_plasma cell
2282	7.18E-07	-0.266851475	0.598	0.314	0.01564812	ABI1	4	HIST1H4C_plasma cell
2283	1.57E-06	-0.26875768	0.799	0.436	0.034270711	CCNL1	4	HIST1H4C_plasma cell
2284	1.68E-06	-0.269014093	0.764	0.398	0.036649658	ICAM3	4	HIST1H4C_plasma cell
2285	1.35E-06	-0.269537467	0.792	0.436	0.029488136	ARGLU1	4	HIST1H4C_plasma cell
2286	1.57E-14	-0.270511223	0.614	0.258	3.43E-10	CSK	4	HIST1H4C_plasma cell
2287	9.88E-08	-0.272955895	0.436	0.21	0.002155372	RGS19	4	HIST1H4C_plasma cell
2288	8.66E-11	-0.275771173	0.564	0.256	1.89E-06	ARHGEF1	4	HIST1H4C_plasma cell
2289	4.28E-12	-0.276869492	0.479	0.202	9.33E-08	PTBP3	4	HIST1H4C_plasma cell
2290	7.00E-07	-0.277647851	0.521	0.269	0.015259635	TBRG1	4	HIST1H4C_plasma cell
2291	4.76E-08	-0.278913743	0.49	0.238	0.001038229	CSNK1D	4	HIST1H4C_plasma cell
2292	1.10E-08	-0.281590358	0.822	0.422	0.000240507	PTPN1	4	HIST1H4C_plasma cell
2293	1.96E-09	-0.283010583	0.483	0.221	4.28E-05	RSF1	4	HIST1H4C_plasma cell
2294	4.24E-08	-0.283210833	0.533	0.264	0.00092435	PPP1R18	4	HIST1H4C_plasma cell
2295	6.22E-10	-0.283487557	0.541	0.252	1.36E-05	IDS	4	HIST1H4C_plasma cell
2296	1.44E-06	-0.283829432	0.687	0.368	0.031477123	ATRX	4	HIST1H4C_plasma cell
2297	2.83E-08	-0.284286625	0.359	0.163	0.000617955	VCPKMT	4	HIST1H4C_plasma cell
2298	1.05E-07	-0.284324	0.792	0.418	0.002292808	PRR13	4	HIST1H4C_plasma cell
2299	6.23E-09	-0.284663105	0.409	0.192	0.000135831	METTL12	4	HIST1H4C_plasma cell
2300	1.14E-10	-0.285274112	0.541	0.245	2.50E-06	ZCCHC11	4	HIST1H4C_plasma cell
2301	1.08E-06	-0.285336611	1	0.967	0.023630752	RPL23A	4	HIST1H4C_plasma cell
2302	2.72E-08	-0.285944987	1	0.971	0.000592098	RPL36	4	HIST1H4C_plasma cell
2303	1.39E-07	-0.288687279	1	0.975	0.003030519	RPS6	4	HIST1H4C_plasma cell
2304	7.79E-09	-0.291103395	0.749	0.379	0.000169779	ARL6IP5	4	HIST1H4C_plasma cell
2305	3.95E-12	-0.291233876	0.564	0.246	8.61E-08	PIAS1	4	HIST1H4C_plasma cell
2306	1.01E-09	-0.29159658	0.444	0.2	2.21E-05	SCPEP1	4	HIST1H4C_plasma cell
2307	2.33E-09	-0.292404834	0.992	0.983	5.08E-05	RPL3	4	HIST1H4C_plasma cell
2308	5.68E-07	-0.292707787	0.533	0.27	0.012387316	CNPPD1	4	HIST1H4C_plasma cell
2309	2.95E-12	-0.295369196	0.533	0.236	6.44E-08	LTA4H	4	HIST1H4C_plasma cell
2310	5.94E-07	-0.296621938	0.459	0.234	0.012964088	MGEA5	4	HIST1H4C_plasma cell
2311	2.38E-09	-0.297137146	0.598	0.292	5.18E-05	ACAP2	4	HIST1H4C_plasma cell
2312	4.94E-14	-0.297627969	0.653	0.279	1.08E-09	MTPN	4	HIST1H4C_plasma cell
2313	1.07E-24	-0.299187351	0.398	0.121	2.34E-20	IGHG4	4	HIST1H4C_plasma cell
2314	1.63E-10	-0.29922039	0.614	0.288	3.56E-06	MAT2A	4	HIST1H4C_plasma cell
2315	3.05E-09	-0.300446553	0.718	0.357	6.66E-05	GGNBP2	4	HIST1H4C_plasma cell
2316	2.80E-10	-0.300694814	0.544	0.245	6.11E-06	CDC42SE1	4	HIST1H4C_plasma cell
2317	2.05E-09	-0.302239202	0.417	0.187	4.48E-05	CYFIP2	4	HIST1H4C_plasma cell
2318	2.84E-10	-0.303958766	0.533	0.242	6.19E-06	CHD1	4	HIST1H4C_plasma cell
2319	2.56E-07	-0.304927774	0.664	0.345	0.005574555	ZFAND5	4	HIST1H4C_plasma cell
2320	9.71E-13	-0.306505575	1	0.991	2.12E-08	RPS15	4	HIST1H4C_plasma cell
2321	7.74E-09	-0.311400037	0.494	0.234	0.000168881	ADD3	4	HIST1H4C_plasma cell
2322	1.07E-06	-0.311408568	0.579	0.299	0.023247915	IKZF1	4	HIST1H4C_plasma cell
2323	4.11E-12	-0.31567032	0.51	0.246	8.96E-08	IGKV1-12	4	HIST1H4C_plasma cell
2324	1.13E-14	-0.31620328	0.618	0.262	2.46E-10	ADK	4	HIST1H4C_plasma cell
2325	3.13E-08	-0.317002354	0.514	0.249	0.000681619	TRAF3IP3	4	HIST1H4C_plasma cell
2326	7.25E-11	-0.319438721	0.429	0.181	1.58E-06	RP9	4	HIST1H4C_plasma cell
2327	1.90E-06	-0.320548361	0.614	0.319	0.041541285	PELI1	4	HIST1H4C_plasma cell
2328	1.06E-08	-0.320992603	0.745	0.379	0.000230292	UCP2	4	HIST1H4C_plasma cell
2329	6.75E-10	-0.32103298	0.506	0.228	1.47E-05	PRKCB	4	HIST1H4C_plasma cell
2330	1.32E-08	-0.323016502	0.664	0.323	0.000288153	SNHG15	4	HIST1H4C_plasma cell
2331	2.52E-10	-0.325935885	1	0.989	5.49E-06	RPS18	4	HIST1H4C_plasma cell
2332	5.05E-08	-0.327260122	0.413	0.196	0.001101059	FKBP5	4	HIST1H4C_plasma cell
2333	9.03E-08	-0.327779798	0.506	0.248	0.001970056	TANK	4	HIST1H4C_plasma cell
2334	5.26E-09	-0.328033608	0.68	0.333	0.000114655	ATP2B1	4	HIST1H4C_plasma cell
2335	1.14E-17	-0.329141153	1	0.993	2.48E-13	RPS8	4	HIST1H4C_plasma cell
2336	1.67E-09	-0.329265298	0.618	0.295	3.64E-05	DNAJB6	4	HIST1H4C_plasma cell
2337	7.37E-08	-0.329935197	0.483	0.236	0.001606488	OSER1	4	HIST1H4C_plasma cell
2338	6.53E-09	-0.330121024	0.463	0.214	0.000142486	GGA2	4	HIST1H4C_plasma cell
2339	1.13E-06	-0.331709654	0.517	0.274	0.024548396	SGK1	4	HIST1H4C_plasma cell
2340	5.34E-07	-0.332111242	0.51	0.262	0.011636003	CCDC107	4	HIST1H4C_plasma cell

2341	5.25E-08	-0.333043061	0.988	0.969	0.001145947	RPS9	4	HIST1H4C_plasma cell
2342	3.35E-07	-0.339928934	0.521	0.26	0.007296469	MAX	4	HIST1H4C_plasma cell
2343	1.64E-09	-0.340594729	0.996	0.963	3.57E-05	NACA	4	HIST1H4C_plasma cell
2344	3.98E-15	-0.346829087	1	0.988	8.68E-11	RPL19	4	HIST1H4C_plasma cell
2345	1.70E-08	-0.34881412	0.992	0.967	0.000370728	RPL17	4	HIST1H4C_plasma cell
2346	2.47E-14	-0.348899986	1	0.979	5.39E-10	RPS13	4	HIST1H4C_plasma cell
2347	5.03E-07	-0.351017623	0.985	0.954	0.010977897	RPSA	4	HIST1H4C_plasma cell
2348	8.66E-07	-0.351967906	0.625	0.322	0.018880003	SCAF11	4	HIST1H4C_plasma cell
2349	6.35E-09	-0.352865993	0.556	0.262	0.000138455	SNHG12	4	HIST1H4C_plasma cell
2350	7.60E-12	-0.352988203	1	0.977	1.66E-07	RPS5	4	HIST1H4C_plasma cell
2351	1.00E-23	-0.354476938	0.622	0.269	2.19E-19	IGHA1	4	HIST1H4C_plasma cell
2352	5.51E-07	-0.358515613	0.664	0.34	0.012020246	ANKRD10	4	HIST1H4C_plasma cell
2353	1.22E-08	-0.360331254	0.66	0.319	0.000266481	CCNH	4	HIST1H4C_plasma cell
2354	4.65E-10	-0.361906521	0.653	0.314	1.01E-05	CIB1	4	HIST1H4C_plasma cell
2355	6.34E-09	-0.364026183	0.985	0.908	0.000138324	UBC	4	HIST1H4C_plasma cell
2356	4.38E-08	-0.365184961	0.456	0.217	0.000954632	BTLA	4	HIST1H4C_plasma cell
2357	3.89E-18	-0.376080451	1	0.993	8.48E-14	RPL28	4	HIST1H4C_plasma cell
2358	6.22E-14	-0.385317188	1	0.988	1.36E-09	RPLP2	4	HIST1H4C_plasma cell
2359	2.12E-07	-0.3863061	0.637	0.312	0.004627266	ITSN2	4	HIST1H4C_plasma cell
2360	4.32E-09	-0.39119127	0.992	0.96	9.42E-05	RPL10A	4	HIST1H4C_plasma cell
2361	3.25E-15	-0.39172987	1	0.979	7.09E-11	RPL29	4	HIST1H4C_plasma cell
2362	6.13E-12	-0.394376768	0.988	0.977	1.34E-07	MT-ND1	4	HIST1H4C_plasma cell
2363	2.33E-09	-0.397902927	0.764	0.379	5.07E-05	BAX	4	HIST1H4C_plasma cell
2364	2.99E-18	-0.407246876	1	0.997	6.51E-14	AC090498.1	4	HIST1H4C_plasma cell
2365	2.16E-07	-0.419721378	0.402	0.186	0.004699658	DCK	4	HIST1H4C_plasma cell
2366	6.96E-09	-0.431807417	0.988	0.945	0.000151864	RPL9	4	HIST1H4C_plasma cell
2367	7.68E-08	-0.44458079	0.992	0.847	0.001674145	HERPUD1	4	HIST1H4C_plasma cell
2368	1.93E-22	-0.444832476	1	0.987	4.20E-18	RPS28	4	HIST1H4C_plasma cell
2369	3.57E-20	-0.446376951	1	0.991	7.79E-16	RPL18	4	HIST1H4C_plasma cell
2370	4.75E-19	-0.455778972	0.996	0.98	1.04E-14	RPS10	4	HIST1H4C_plasma cell
2371	1.83E-24	-0.467000929	1	0.99	3.98E-20	RPS15A	4	HIST1H4C_plasma cell
2372	7.40E-29	-0.475329706	1	0.997	1.61E-24	RPL10	4	HIST1H4C_plasma cell
2373	7.18E-15	-0.477100374	0.996	0.956	1.57E-10	RPS11	4	HIST1H4C_plasma cell
2374	1.78E-13	-0.479495403	0.996	0.971	3.88E-09	RPL12	4	HIST1H4C_plasma cell
2375	2.01E-16	-0.481601467	1	0.986	4.38E-12	RPS21	4	HIST1H4C_plasma cell
2376	5.32E-22	-0.49549452	0.996	0.978	1.16E-17	RPS14	4	HIST1H4C_plasma cell
2377	1.83E-19	-0.49659299	1	0.994	3.99E-15	RPL11	4	HIST1H4C_plasma cell
2378	1.17E-18	-0.523981496	1	0.98	2.56E-14	RPS3A	4	HIST1H4C_plasma cell
2379	1.46E-19	-0.535950059	1	0.986	3.18E-15	RPS12	4	HIST1H4C_plasma cell
2380	1.80E-13	-0.548644552	0.992	0.943	3.93E-09	MT-ND2	4	HIST1H4C_plasma cell
2381	2.65E-11	-0.551846214	0.973	0.837	5.77E-07	HLA-E	4	HIST1H4C_plasma cell
2382	9.13E-17	-0.555514469	0.992	0.96	1.99E-12	RPL13A	4	HIST1H4C_plasma cell
2383	2.29E-10	-0.558698099	0.992	0.904	4.99E-06	RPL38	4	HIST1H4C_plasma cell
2384	1.18E-06	-0.559114002	1	0.971	0.025775539	FTH1	4	HIST1H4C_plasma cell
2385	3.92E-24	-0.559799998	0.996	0.974	8.55E-20	RPL35A	4	HIST1H4C_plasma cell
2386	8.41E-19	-0.568903614	0.992	0.968	1.83E-14	RPL22	4	HIST1H4C_plasma cell
2387	6.58E-27	-0.578603664	0.996	0.989	1.44E-22	FAU	4	HIST1H4C_plasma cell
2388	4.88E-21	-0.58322594	0.996	0.977	1.06E-16	RPL26	4	HIST1H4C_plasma cell
2389	1.49E-20	-0.592390847	0.996	0.97	3.25E-16	MT-ND4	4	HIST1H4C_plasma cell
2390	6.30E-08	-0.601093881	0.969	0.767	0.001374109	PTPRCAP	4	HIST1H4C_plasma cell
2391	1.29E-13	-0.603238572	0.973	0.859	2.82E-09	DDX5	4	HIST1H4C_plasma cell
2392	1.53E-19	-0.604919899	0.992	0.973	3.33E-15	RPL21	4	HIST1H4C_plasma cell
2393	5.89E-27	-0.60644398	0.996	0.997	1.29E-22	EEF1A1	4	HIST1H4C_plasma cell
2394	5.91E-25	-0.60799729	1	0.981	1.29E-20	RPL37	4	HIST1H4C_plasma cell
2395	6.23E-21	-0.608051914	1	0.986	1.36E-16	RPS23	4	HIST1H4C_plasma cell
2396	6.89E-14	-0.6177303	0.942	0.808	1.50E-09	SARAF	4	HIST1H4C_plasma cell
2397	1.72E-22	-0.626977856	0.992	0.975	3.76E-18	EIF1	4	HIST1H4C_plasma cell
2398	1.96E-06	-0.634970061	0.923	0.768	0.042761603	RPS20	4	HIST1H4C_plasma cell
2399	1.95E-20	-0.642246952	1	0.97	4.25E-16	RPS25	4	HIST1H4C_plasma cell
2400	1.95E-25	-0.644358992	0.996	0.994	4.25E-21	RPL13	4	HIST1H4C_plasma cell
2401	1.12E-21	-0.646075707	0.992	0.988	2.44E-17	RPS27A	4	HIST1H4C_plasma cell
2402	4.80E-07	-0.646753689	0.12	0.236	0.010469929	SMIM14	4	HIST1H4C_plasma cell
2403	4.82E-27	-0.659668201	0.996	0.986	1.05E-22	RPL18A	4	HIST1H4C_plasma cell
2404	1.87E-06	-0.671381162	0.174	0.275	0.040866532	CD40	4	HIST1H4C_plasma cell
2405	1.12E-06	-0.672498708	0.1	0.211	0.024419681	:P11-693J15.	4	HIST1H4C_plasma cell
2406	6.71E-37	-0.678110813	0.996	0.997	1.46E-32	MT-ND3	4	HIST1H4C_plasma cell
2407	1.07E-18	-0.683450831	0.996	0.944	2.32E-14	RPS29	4	HIST1H4C_plasma cell
2408	1.52E-10	-0.693413141	0.961	0.805	3.31E-06	TOMM7	4	HIST1H4C_plasma cell
2409	2.69E-17	-0.705173619	0.981	0.941	5.86E-13	TMSB4X	4	HIST1H4C_plasma cell
2410	1.79E-08	-0.712572301	0.931	0.732	0.000389622	CIRBP	4	HIST1H4C_plasma cell
2411	3.79E-08	-0.761378776	0.1	0.23	0.000827565	FAM65B	4	HIST1H4C_plasma cell

2412	6.63E-08	-0.773215408	0.938	0.728	0.001446848	EZR	4	HIST1H4C_plasma cell
2413	1.02E-25	-0.773285253	1	0.984	2.23E-21	RPL32	4	HIST1H4C_plasma cell
2414	6.07E-31	-0.785052104	0.973	0.906	1.32E-26	CD79A	4	HIST1H4C_plasma cell
2415	9.52E-22	-0.794746589	0.965	0.884	2.08E-17	IER2	4	HIST1H4C_plasma cell
2416	1.30E-23	-0.800429349	1	0.995	2.84E-19	RPS19	4	HIST1H4C_plasma cell
2417	1.06E-13	-0.814399414	0.803	0.753	2.31E-09	JUN	4	HIST1H4C_plasma cell
2418	8.73E-26	-0.83091459	1	0.984	1.90E-21	RPL30	4	HIST1H4C_plasma cell
2419	5.90E-08	-0.848257976	0.116	0.243	0.001286043	FCER2	4	HIST1H4C_plasma cell
2420	3.13E-11	-0.875105797	0.73	0.674	6.83E-07	KLF6	4	HIST1H4C_plasma cell
2421	1.45E-07	-0.902987164	0.201	0.306	0.003169058	ADAM28	4	HIST1H4C_plasma cell
2422	1.36E-06	-0.909323886	0.514	0.494	0.02965577	BIRC3	4	HIST1H4C_plasma cell
2423	1.87E-10	-0.91111538	0.842	0.699	4.08E-06	COMMD6	4	HIST1H4C_plasma cell
2424	1.99E-06	-0.929404985	0.772	0.609	0.043434415	PTPRC	4	HIST1H4C_plasma cell
2425	3.66E-11	-0.934413102	0.834	0.688	7.99E-07	GLTSCR2	4	HIST1H4C_plasma cell
2426	4.79E-09	-0.945876811	0.297	0.395	0.000104523	LY9	4	HIST1H4C_plasma cell
2427	1.71E-09	-0.951808508	0.483	0.527	3.73E-05	FOSB	4	HIST1H4C_plasma cell
2428	2.26E-39	-0.956074233	0.996	0.988	4.93E-35	RPL34	4	HIST1H4C_plasma cell
2429	4.17E-20	-0.963132928	0.838	0.747	9.10E-16	PNRC1	4	HIST1H4C_plasma cell
2430	7.84E-09	-0.977787487	0.158	0.293	0.000170979	ZNF331	4	HIST1H4C_plasma cell
2431	1.14E-78	-0.981296874	1	1	2.49E-74	MALAT1	4	HIST1H4C_plasma cell
2432	6.80E-18	-0.991831866	0.162	0.39	1.48E-13	RNASET2	4	HIST1H4C_plasma cell
2433	1.88E-33	-0.994538831	0.938	0.911	4.09E-29	ZFP36	4	HIST1H4C_plasma cell
2434	4.84E-33	-0.995828946	0.996	0.984	1.06E-28	RPL39	4	HIST1H4C_plasma cell
2435	1.63E-12	-1.002186294	0.676	0.643	3.56E-08	DDIT4	4	HIST1H4C_plasma cell
2436	2.55E-36	-1.013663471	0.973	0.915	5.56E-32	PFDN5	4	HIST1H4C_plasma cell
2437	1.13E-10	-1.016843152	0.266	0.4	2.47E-06	NR4A1	4	HIST1H4C_plasma cell
2438	6.06E-09	-1.056355255	0.741	0.647	0.000132119	GADD45B	4	HIST1H4C_plasma cell
2439	2.04E-13	-1.070370249	0.772	0.674	4.45E-09	ZFAS1	4	HIST1H4C_plasma cell
2440	3.35E-08	-1.080384967	0.205	0.321	0.00072948	ID3	4	HIST1H4C_plasma cell
2441	4.07E-20	-1.113765619	0.259	0.478	8.88E-16	IFITM2	4	HIST1H4C_plasma cell
2442	1.84E-12	-1.117397155	0.266	0.403	4.01E-08	BANK1	4	HIST1H4C_plasma cell
2443	2.79E-11	-1.147070152	0.228	0.371	6.09E-07	GPR183	4	HIST1H4C_plasma cell
2444	6.69E-09	-1.179617807	0.405	0.443	0.000145801	FCMR	4	HIST1H4C_plasma cell
2445	2.42E-24	-1.207518955	0.757	0.771	5.29E-20	FOS	4	HIST1H4C_plasma cell
2446	2.93E-18	-1.210541685	0.197	0.416	6.39E-14	IRF8	4	HIST1H4C_plasma cell
2447	7.08E-14	-1.234273905	0.873	0.714	1.54E-09	HLA-DRB5	4	HIST1H4C_plasma cell
2448	2.21E-36	-1.258973937	0.873	0.873	4.82E-32	ZFP36L2	4	HIST1H4C_plasma cell
2449	6.60E-20	-1.279733638	0.158	0.403	1.44E-15	REL	4	HIST1H4C_plasma cell
2450	2.84E-16	-1.30258963	0.691	0.645	6.19E-12	CD55	4	HIST1H4C_plasma cell
2451	3.67E-24	-1.341300032	0.919	0.805	8.00E-20	HLA-DPA1	4	HIST1H4C_plasma cell
2452	1.50E-16	-1.354535406	0.876	0.726	3.28E-12	CD52	4	HIST1H4C_plasma cell
2453	1.13E-34	-1.360818408	0.996	0.982	2.47E-30	RPS27	4	HIST1H4C_plasma cell
2454	3.04E-34	-1.440187335	0.83	0.831	6.63E-30	LAPTM5	4	HIST1H4C_plasma cell
2455	1.22E-24	-1.49114101	0.313	0.537	2.66E-20	SLC2A3	4	HIST1H4C_plasma cell
2456	2.10E-36	-1.567778615	0.888	0.831	4.58E-32	HLA-DQB1	4	HIST1H4C_plasma cell
2457	9.89E-25	-1.571774026	0.452	0.611	2.16E-20	NR4A2	4	HIST1H4C_plasma cell
2458	1.78E-31	-1.584314656	0.386	0.619	3.88E-27	ZFP36L1	4	HIST1H4C_plasma cell
2459	3.21E-21	-1.616336543	0.737	0.682	7.00E-17	HLA-DQA1	4	HIST1H4C_plasma cell
2460	1.54E-56	-1.621439606	0.958	0.945	3.35E-52	JUNB	4	HIST1H4C_plasma cell
2461	1.34E-61	-1.62823512	0.718	0.885	2.92E-57	TSC22D3	4	HIST1H4C_plasma cell
2462	7.15E-34	-1.8056698	0.903	0.844	1.56E-29	HLA-DRB1	4	HIST1H4C_plasma cell
2463	2.10E-06	-1.815158725	0.266	0.139	0.045804598	IGLV6-57	4	HIST1H4C_plasma cell
2464	1.51E-31	-1.833875055	0.699	0.732	3.29E-27	HLA-DPB1	4	HIST1H4C_plasma cell
2465	5.68E-65	-1.863571704	0.996	0.993	1.24E-60	CD74	4	HIST1H4C_plasma cell
2466	4.84E-53	-1.886320855	0.653	0.82	1.06E-48	DUSP1	4	HIST1H4C_plasma cell
2467	7.35E-50	-1.983124615	0.772	0.84	1.60E-45	CXCR4	4	HIST1H4C_plasma cell
2468	1.08E-36	-2.180284333	0.753	0.781	2.35E-32	CD37	4	HIST1H4C_plasma cell
2469	2.19E-06	-2.20183458	0.286	0.145	0.047714359	IGLV3-1	4	HIST1H4C_plasma cell
2470	4.68E-44	-2.228789989	0.355	0.666	1.02E-39	TXNIP	4	HIST1H4C_plasma cell
2471	1.19E-41	-2.309046918	0.1	0.534	2.58E-37	CD83	4	HIST1H4C_plasma cell
2472	8.15E-58	-2.451089939	0.884	0.905	1.78E-53	BTG1	4	HIST1H4C_plasma cell
2473	4.38E-47	-2.481343046	0.116	0.58	9.56E-43	CD69	4	HIST1H4C_plasma cell
2474	4.59E-34	-2.683929388	0.826	0.775	1.00E-29	HLA-DRA	4	HIST1H4C_plasma cell
2475	6.29E-21	4.738029825	0.539	0.149	1.37E-16	IGLV3-1	5	IGLV3-1_plasma cell
2476	4.43E-10	3.413961729	0.224	0.053	9.65E-06	IGKV1D-39	5	IGLV3-1_plasma cell
2477	2.30E-11	3.222210486	0.211	0.043	5.02E-07	IGKV1D-12	5	IGLV3-1_plasma cell
2478	1.77E-06	3.193111637	0.158	0.041	0.038531594	IGLV3-21	5	IGLV3-1_plasma cell
2479	3.98E-08	3.133366579	0.145	0.03	0.000866986	IGKV1D-8	5	IGLV3-1_plasma cell
2480	1.82E-15	2.774235159	0.487	0.142	3.97E-11	IGLV6-57	5	IGLV3-1_plasma cell
2481	3.72E-15	2.326135586	0.711	0.353	8.11E-11	IGHG1	5	IGLV3-1_plasma cell
2482	8.55E-08	2.06248366	0.237	0.07	0.001863984	IGHV3-33	5	IGLV3-1_plasma cell

2483	2.83E-08	1.907296838	0.224	0.058	0.000618012	IGHV1-46	5	IGLV3-1_plasma cell
2484	2.76E-07	1.886359494	0.171	0.043	0.006024647	IGKV1-17	5	IGLV3-1_plasma cell
2485	2.33E-15	1.708231318	0.447	0.148	5.09E-11	IGHG4	5	IGLV3-1_plasma cell
2486	4.62E-08	1.597380787	0.289	0.095	0.001008145	IGKV1-27	5	IGLV3-1_plasma cell
2487	1.10E-07	1.572963549	0.263	0.078	0.002395476	IGLV1-40	5	IGLV3-1_plasma cell
2488	1.55E-22	1.392333259	0.724	0.231	3.37E-18	HSPB1	5	IGLV3-1_plasma cell
2489	4.58E-07	1.296747322	0.711	0.569	0.009998034	IGKC	5	IGLV3-1_plasma cell
2490	2.58E-39	1.295242336	1	0.644	5.63E-35	SSR4	5	IGLV3-1_plasma cell
2491	6.29E-13	1.259456975	0.276	0.061	1.37E-08	IGKV1-6	5	IGLV3-1_plasma cell
2492	1.14E-45	1.157295332	0.579	0.085	2.49E-41	JSRP1	5	IGLV3-1_plasma cell
2493	1.78E-81	1.021514079	0.632	0.049	3.87E-77	TIMP1	5	IGLV3-1_plasma cell
2494	9.69E-10	1.007886899	0.184	0.037	2.11E-05	IGKV1D-33	5	IGLV3-1_plasma cell
2495	5.68E-15	0.981177615	0.645	0.244	1.24E-10	RGCC	5	IGLV3-1_plasma cell
2496	3.60E-10	0.953212506	0.632	0.309	7.85E-06	HSPA1B	5	IGLV3-1_plasma cell
2497	3.37E-55	0.870930527	0.868	0.178	7.34E-51	SPAG4	5	IGLV3-1_plasma cell
2498	8.22E-13	0.863252782	0.671	0.295	1.79E-08	HSPA1A	5	IGLV3-1_plasma cell
2499	6.71E-17	0.841520606	0.303	0.059	1.46E-12	BAG3	5	IGLV3-1_plasma cell
2500	7.89E-24	0.837400834	0.921	0.431	1.72E-19	PRDX4	5	IGLV3-1_plasma cell
2501	7.88E-18	0.832219244	0.908	0.444	1.72E-13	DERL3	5	IGLV3-1_plasma cell
2502	1.78E-58	0.820130307	0.803	0.135	3.88E-54	SELM	5	IGLV3-1_plasma cell
2503	3.47E-12	0.78413587	0.711	0.346	7.56E-08	LINC00152	5	IGLV3-1_plasma cell
2504	9.08E-45	0.77531641	0.684	0.115	1.98E-40	LMNA	5	IGLV3-1_plasma cell
2505	7.16E-10	0.730502095	0.395	0.132	1.56E-05	IGHG3	5	IGLV3-1_plasma cell
2506	5.79E-44	0.724263313	0.829	0.194	1.26E-39	SDC1	5	IGLV3-1_plasma cell
2507	2.76E-22	0.700841178	0.434	0.093	6.01E-18	DUSP5	5	IGLV3-1_plasma cell
2508	1.28E-62	0.698361146	0.539	0.047	2.80E-58	CST3	5	IGLV3-1_plasma cell
2509	1.42E-08	0.688219306	0.132	0.023	0.000309526	IGHJ6	5	IGLV3-1_plasma cell
2510	4.13E-21	0.673875448	0.289	0.043	9.01E-17	DNAAF1	5	IGLV3-1_plasma cell
2511	1.04E-18	0.644122891	0.947	0.452	2.28E-14	FKBP2	5	IGLV3-1_plasma cell
2512	8.61E-21	0.603535417	0.961	0.532	1.88E-16	NPC2	5	IGLV3-1_plasma cell
2513	7.73E-26	0.603154228	0.868	0.341	1.69E-21	CD63	5	IGLV3-1_plasma cell
2514	4.76E-15	0.591758332	0.5	0.155	1.04E-10	ZFAND2A	5	IGLV3-1_plasma cell
2515	1.72E-16	0.59169057	0.961	0.558	3.74E-12	TMEM59	5	IGLV3-1_plasma cell
2516	1.19E-11	0.58873412	0.224	0.044	2.59E-07	MT1F	5	IGLV3-1_plasma cell
2517	1.43E-95	0.579072709	0.658	0.042	3.11E-91	CD9	5	IGLV3-1_plasma cell
2518	1.18E-33	0.575011431	0.711	0.167	2.58E-29	LY96	5	IGLV3-1_plasma cell
2519	3.47E-13	0.563313999	0.947	0.442	7.57E-09	FKBP11	5	IGLV3-1_plasma cell
2520	2.72E-26	0.538959911	0.763	0.25	5.92E-22	CREB3L2	5	IGLV3-1_plasma cell
2521	6.05E-09	0.538279263	0.408	0.157	0.000131962	MIR155HG	5	IGLV3-1_plasma cell
2522	4.59E-15	0.53086153	0.868	0.442	1.00E-10	DNAJB9	5	IGLV3-1_plasma cell
2523	3.04E-46	0.529344927	0.474	0.053	6.64E-42	DPEP1	5	IGLV3-1_plasma cell
2524	7.95E-19	0.526119071	0.697	0.272	1.73E-14	GAS6	5	IGLV3-1_plasma cell
2525	5.95E-10	0.525957047	0.908	0.471	1.30E-05	GSTP1	5	IGLV3-1_plasma cell
2526	2.98E-23	0.515345946	0.145	0.008	6.51E-19	IGKV1D-17	5	IGLV3-1_plasma cell
2527	8.36E-11	0.514915271	0.671	0.348	1.82E-06	PELI1	5	IGLV3-1_plasma cell
2528	4.11E-17	0.50979618	0.711	0.286	8.96E-13	TRIB1	5	IGLV3-1_plasma cell
2529	7.33E-08	0.508745224	0.895	0.673	0.001598028	KLF6	5	IGLV3-1_plasma cell
2530	5.52E-09	0.499499549	0.987	0.863	0.000120484	HERPUD1	5	IGLV3-1_plasma cell
2531	1.65E-18	0.493297765	0.368	0.077	3.60E-14	RASD1	5	IGLV3-1_plasma cell
2532	7.08E-10	0.486271867	0.118	0.016	1.54E-05	GNLY	5	IGLV3-1_plasma cell
2533	2.45E-16	0.476297686	0.829	0.356	5.34E-12	NUCB2	5	IGLV3-1_plasma cell
2534	1.46E-07	0.475180291	0.592	0.326	0.003194255	ANKRD28	5	IGLV3-1_plasma cell
2535	6.21E-16	0.474461205	0.816	0.366	1.35E-11	DNAJC1	5	IGLV3-1_plasma cell
2536	2.41E-114	0.474170997	0.408	0.006	5.26E-110	CTHRC1	5	IGLV3-1_plasma cell
2537	1.34E-12	0.464900281	0.908	0.425	2.91E-08	HDLBP	5	IGLV3-1_plasma cell
2538	6.37E-47	0.464368239	0.711	0.115	1.39E-42	A1BG	5	IGLV3-1_plasma cell
2539	8.65E-31	0.420883494	0.158	0.006	1.89E-26	RP11-598F7.[illegible]	5	IGLV3-1_plasma cell
2540	1.66E-09	0.420695079	0.947	0.572	3.63E-05	KRTCAP2	5	IGLV3-1_plasma cell
2541	3.20E-22	0.41844643	0.553	0.142	6.97E-18	P4HA1	5	IGLV3-1_plasma cell
2542	8.03E-16	0.417086008	0.711	0.289	1.75E-11	CHPF	5	IGLV3-1_plasma cell
2543	3.88E-20	0.414749617	0.684	0.22	8.46E-16	S100A11	5	IGLV3-1_plasma cell
2544	9.67E-20	0.413287136	0.684	0.245	2.11E-15	MEI1	5	IGLV3-1_plasma cell
2545	4.36E-07	0.411162091	0.645	0.341	0.009500072	S100A10	5	IGLV3-1_plasma cell
2546	9.33E-13	0.387326494	0.447	0.147	2.03E-08	KLHL6	5	IGLV3-1_plasma cell
2547	1.93E-09	0.373180475	0.868	0.434	4.20E-05	ELL2	5	IGLV3-1_plasma cell
2548	3.84E-43	0.372023275	0.382	0.035	8.38E-39	FAM92B	5	IGLV3-1_plasma cell
2549	1.46E-13	0.371908405	0.671	0.275	3.19E-09	H1FX	5	IGLV3-1_plasma cell
2550	2.39E-17	0.368840345	0.684	0.249	5.20E-13	PECAM1	5	IGLV3-1_plasma cell
2551	2.31E-13	0.365686195	0.711	0.313	5.04E-09	ZBTB38	5	IGLV3-1_plasma cell
2552	3.17E-13	0.362634469	0.803	0.395	6.90E-09	CFLAR	5	IGLV3-1_plasma cell
2553	2.60E-54	0.362111354	0.263	0.009	5.67E-50	CTSW	5	IGLV3-1_plasma cell

2554	7.35E-21	0.360027258	0.342	0.063	1.60E-16	QPCT	5	IGLV3-1_plasma cell
2555	4.70E-15	0.344321756	0.697	0.266	1.03E-10	ANKRD37	5	IGLV3-1_plasma cell
2556	2.29E-18	0.342737156	0.75	0.277	4.99E-14	SIL1	5	IGLV3-1_plasma cell
2557	5.52E-17	0.342403146	0.632	0.227	1.20E-12	EIF2AK4	5	IGLV3-1_plasma cell
2558	5.19E-07	0.334154767	0.921	0.55	0.011322174	SELK	5	IGLV3-1_plasma cell
2559	4.79E-14	0.327084365	0.75	0.32	1.04E-09	DSTN	5	IGLV3-1_plasma cell
2560	4.73E-09	0.326879139	0.658	0.32	0.000103118	SLAMF7	5	IGLV3-1_plasma cell
2561	1.94E-07	0.322252105	0.868	0.445	0.004231658	ERLEC1	5	IGLV3-1_plasma cell
2562	1.26E-06	0.314738276	0.816	0.459	0.027385463	LGALS1	5	IGLV3-1_plasma cell
2563	9.88E-101	0.314622426	0.329	0.003	2.15E-96	TNFRSF4	5	IGLV3-1_plasma cell
2564	1.63E-14	0.312940881	0.5	0.159	3.56E-10	PYCR1	5	IGLV3-1_plasma cell
2565	9.28E-49	0.3129351	0.276	0.013	2.02E-44	ASS1	5	IGLV3-1_plasma cell
2566	2.43E-27	0.31278619	0.224	0.019	5.29E-23	LGALSL	5	IGLV3-1_plasma cell
2567	1.03E-10	0.311039209	0.829	0.387	2.25E-06	CRELD2	5	IGLV3-1_plasma cell
2568	2.18E-08	0.307496709	0.987	0.497	0.000474586	SEC11C	5	IGLV3-1_plasma cell
2569	7.48E-22	0.306194336	0.526	0.133	1.63E-17	LGALS3	5	IGLV3-1_plasma cell
2570	3.21E-61	0.291562804	0.355	0.017	7.01E-57	TNFRSF18	5	IGLV3-1_plasma cell
2571	3.99E-15	0.287959457	0.421	0.117	8.71E-11	ITGA6	5	IGLV3-1_plasma cell
2572	1.88E-18	0.283504173	0.421	0.092	4.10E-14	ATF3	5	IGLV3-1_plasma cell
2573	4.55E-11	0.283089589	0.526	0.209	9.91E-07	COMTD1	5	IGLV3-1_plasma cell
2574	4.70E-08	0.282579186	0.658	0.345	0.001024302	PRDM1	5	IGLV3-1_plasma cell
2575	1.47E-21	0.281814637	0.145	0.009	3.21E-17	KCNMA1	5	IGLV3-1_plasma cell
2576	1.13E-07	0.281695637	0.921	0.56	0.002465628	PSAP	5	IGLV3-1_plasma cell
2577	3.20E-35	0.276997884	0.526	0.081	6.97E-31	FCGRT	5	IGLV3-1_plasma cell
2578	2.46E-12	0.276543903	0.803	0.385	5.36E-08	FCRL5	5	IGLV3-1_plasma cell
2579	1.62E-09	0.274068489	0.566	0.251	3.53E-05	HMCES	5	IGLV3-1_plasma cell
2580	2.03E-16	0.27366357	0.303	0.059	4.43E-12	IQCG	5	IGLV3-1_plasma cell
2581	3.68E-14	0.271603636	0.605	0.236	8.02E-10	ARSA	5	IGLV3-1_plasma cell
2582	2.20E-88	0.269789348	0.408	0.013	4.80E-84	KDELR3	5	IGLV3-1_plasma cell
2583	5.99E-118	0.265380461	0.355	0.002	1.31E-113	SLC22A17	5	IGLV3-1_plasma cell
2584	2.75E-15	0.26151652	0.605	0.215	5.99E-11	MAGED1	5	IGLV3-1_plasma cell
2585	3.05E-08	0.259162811	0.329	0.113	0.000664719	PTP4A3	5	IGLV3-1_plasma cell
2586	6.39E-10	0.255929313	0.75	0.362	1.39E-05	TXNDC15	5	IGLV3-1_plasma cell
2587	4.41E-18	0.252131137	0.789	0.28	9.62E-14	JUND	5	IGLV3-1_plasma cell
2588	3.92E-08	-0.451715847	0.947	0.899	0.000855827	OAZ1	5	IGLV3-1_plasma cell
2589	1.17E-10	-0.513975807	1	0.968	2.56E-06	MT-ND5	5	IGLV3-1_plasma cell
2590	5.57E-11	-0.543875584	0.934	0.863	1.21E-06	ATP5E	5	IGLV3-1_plasma cell
2591	3.50E-09	-0.549332233	0.829	0.952	7.63E-05	TMSB4X	5	IGLV3-1_plasma cell
2592	1.80E-06	-0.555445889	0.829	0.713	0.039232852	EIF3K	5	IGLV3-1_plasma cell
2593	1.14E-08	-0.583668999	0.921	0.808	0.000248349	ATP5G2	5	IGLV3-1_plasma cell
2594	1.53E-12	-0.585992758	0.987	0.945	3.35E-08	EEF2	5	IGLV3-1_plasma cell
2595	2.15E-06	-0.629632213	0.724	0.702	0.04693573	RAC2	5	IGLV3-1_plasma cell
2596	4.01E-19	-0.635958607	1	0.986	8.75E-15	TPT1	5	IGLV3-1_plasma cell
2597	4.35E-10	-0.637810146	0.974	0.949	9.50E-06	MT-ND2	5	IGLV3-1_plasma cell
2598	2.04E-06	-0.639015024	0.895	0.895	0.044527417	IER2	5	IGLV3-1_plasma cell
2599	1.02E-17	-0.642804914	0.987	0.982	2.22E-13	HLA-B	5	IGLV3-1_plasma cell
2600	5.90E-11	-0.645449338	0.921	0.837	1.29E-06	COX7C	5	IGLV3-1_plasma cell
2601	1.36E-07	-0.646275203	0.855	0.742	0.002967788	UQCRB	5	IGLV3-1_plasma cell
2602	3.16E-09	-0.676520136	0.882	0.822	6.90E-05	PCBP2	5	IGLV3-1_plasma cell
2603	2.15E-20	-0.683572843	0.987	0.991	4.68E-16	RPLP0	5	IGLV3-1_plasma cell
2604	4.85E-07	-0.70857391	0.829	0.842	0.010566934	ACTG1	5	IGLV3-1_plasma cell
2605	5.76E-09	-0.708799504	0.855	0.792	0.000125508	HNRNPDL	5	IGLV3-1_plasma cell
2606	6.22E-16	-0.7235371	0.961	0.951	1.36E-11	RPL41	5	IGLV3-1_plasma cell
2607	7.36E-07	-0.724777269	0.645	0.702	0.016043902	MYL12A	5	IGLV3-1_plasma cell
2608	1.69E-08	-0.725820784	0.684	0.707	0.000367928	EIF3F	5	IGLV3-1_plasma cell
2609	4.02E-24	-0.742243915	1	0.999	8.76E-20	MT-ND4L	5	IGLV3-1_plasma cell
2610	1.03E-11	-0.742607316	0.895	0.844	2.25E-07	HINT1	5	IGLV3-1_plasma cell
2611	1.58E-08	-0.754157139	0.763	0.728	0.000343667	HNRNPK	5	IGLV3-1_plasma cell
2612	3.64E-15	-0.75590661	0.987	0.973	7.94E-11	MT-ND4	5	IGLV3-1_plasma cell
2613	4.74E-11	-0.759142045	0.947	0.83	1.03E-06	CFL1	5	IGLV3-1_plasma cell
2614	1.35E-09	-0.760861558	0.789	0.786	2.94E-05	CCNI	5	IGLV3-1_plasma cell
2615	7.04E-08	-0.762903593	0.75	0.742	0.001534303	SH3BGRL3	5	IGLV3-1_plasma cell
2616	4.54E-12	-0.764547615	0.934	0.86	9.90E-08	HNRNPA2B1	5	IGLV3-1_plasma cell
2617	9.68E-11	-0.770028413	0.908	0.874	2.11E-06	DDX5	5	IGLV3-1_plasma cell
2618	9.80E-08	-0.787414873	0.211	0.49	0.002136385	LCP1	5	IGLV3-1_plasma cell
2619	1.54E-24	-0.789297949	1	0.999	3.37E-20	MT-CYB	5	IGLV3-1_plasma cell
2620	7.60E-17	-0.793285845	0.961	0.946	1.66E-12	EEF1D	5	IGLV3-1_plasma cell
2621	3.49E-17	-0.793722358	0.987	0.997	7.61E-13	MT-ND3	5	IGLV3-1_plasma cell
2622	2.18E-06	-0.80527118	0.487	0.618	0.04759566	CD79B	5	IGLV3-1_plasma cell
2623	2.96E-26	-0.812549417	0.987	0.998	6.46E-22	RPLP1	5	IGLV3-1_plasma cell
2624	1.55E-08	-0.816676504	0.724	0.701	0.00033788	NCL	5	IGLV3-1_plasma cell

2625	8.37E-20	-0.818946346	0.961	0.966	1.82E-15	EEF1B2	5	IGLV3-1_plasma cell
2626	5.73E-14	-0.819784996	0.895	0.876	1.25E-09	PFN1	5	IGLV3-1_plasma cell
2627	2.86E-11	-0.82781767	0.789	0.772	6.23E-07	RPS17	5	IGLV3-1_plasma cell
2628	1.11E-06	-0.828463607	0.711	0.694	0.024100873	CALM2	5	IGLV3-1_plasma cell
2629	1.07E-14	-0.82944068	0.842	0.849	2.33E-10	BTF3	5	IGLV3-1_plasma cell
2630	2.56E-21	-0.83263327	0.987	0.998	5.59E-17	RPL10	5	IGLV3-1_plasma cell
2631	2.87E-26	-0.849000514	0.987	0.991	6.27E-22	RPL7A	5	IGLV3-1_plasma cell
2632	4.70E-10	-0.85752002	0.526	0.69	1.03E-05	SMCHD1	5	IGLV3-1_plasma cell
2633	3.25E-09	-0.857622208	0.5	0.633	7.09E-05	LSP1	5	IGLV3-1_plasma cell
2634	1.59E-21	-0.857670144	1	0.963	3.46E-17	RPL24	5	IGLV3-1_plasma cell
2635	3.55E-14	-0.858454559	0.895	0.849	7.73E-10	NPM1	5	IGLV3-1_plasma cell
2636	1.63E-20	-0.858832696	0.987	0.978	3.55E-16	MT-ND1	5	IGLV3-1_plasma cell
2637	9.13E-07	-0.860625214	0.579	0.68	0.019921326	TAGLN2	5	IGLV3-1_plasma cell
2638	2.20E-06	-0.875697851	0.592	0.669	0.04789548	FUS	5	IGLV3-1_plasma cell
2639	1.33E-10	-0.876766498	0.697	0.759	2.90E-06	EIF3E	5	IGLV3-1_plasma cell
2640	1.00E-07	-0.880181158	0.224	0.489	0.002187812	LYN	5	IGLV3-1_plasma cell
2641	1.38E-29	-0.890207352	1	1	3.00E-25	MT-CO2	5	IGLV3-1_plasma cell
2642	2.68E-15	-0.896778731	0.908	0.853	5.85E-11	RPL27A	5	IGLV3-1_plasma cell
2643	7.10E-20	-0.899088229	0.908	0.893	1.55E-15	COX4I1	5	IGLV3-1_plasma cell
2644	9.89E-23	-0.900847536	1	0.99	2.16E-18	FAU	5	IGLV3-1_plasma cell
2645	7.09E-22	-0.906556686	0.961	0.968	1.55E-17	NACA	5	IGLV3-1_plasma cell
2646	3.48E-13	-0.906866073	0.882	0.807	7.59E-09	YBX1	5	IGLV3-1_plasma cell
2647	6.57E-14	-0.919091497	0.855	0.859	1.43E-09	YWHAZ	5	IGLV3-1_plasma cell
2648	5.15E-08	-0.920385918	0.184	0.469	0.001122379	RCSD1	5	IGLV3-1_plasma cell
2649	3.00E-23	-0.936798331	0.961	0.977	6.53E-19	RPL14	5	IGLV3-1_plasma cell
2650	7.20E-22	-0.936891046	0.947	0.947	1.57E-17	HLA-A	5	IGLV3-1_plasma cell
2651	3.08E-25	-0.944509622	0.961	0.985	6.71E-21	RPL3	5	IGLV3-1_plasma cell
2652	4.08E-27	-0.946172403	0.987	0.994	8.91E-23	RPL8	5	IGLV3-1_plasma cell
2653	6.91E-08	-0.950367743	0.237	0.493	0.001507914	RNASE6	5	IGLV3-1_plasma cell
2654	1.60E-10	-0.952570043	0.882	0.826	3.49E-06	TOMM7	5	IGLV3-1_plasma cell
2655	9.99E-15	-0.955279443	0.763	0.786	2.18E-10	ARPC3	5	IGLV3-1_plasma cell
2656	3.63E-19	-0.955912771	0.921	0.898	7.91E-15	H3F3A	5	IGLV3-1_plasma cell
2657	2.10E-16	-0.961528549	0.829	0.845	4.58E-12	HNRNPA1	5	IGLV3-1_plasma cell
2658	9.04E-22	-0.963956976	0.842	0.903	1.97E-17	EEF1G	5	IGLV3-1_plasma cell
2659	3.29E-12	-0.968889417	0.921	0.923	7.18E-08	PFDN5	5	IGLV3-1_plasma cell
2660	4.97E-24	-0.976282237	0.961	0.981	1.08E-19	RPS14	5	IGLV3-1_plasma cell
2661	1.53E-25	-0.979431281	0.961	0.97	3.35E-21	RPL35	5	IGLV3-1_plasma cell
2662	4.49E-25	-0.986356711	0.947	0.973	9.80E-21	RPL6	5	IGLV3-1_plasma cell
2663	1.49E-22	-0.997792344	0.934	0.92	3.26E-18	RPL7	5	IGLV3-1_plasma cell
2664	1.04E-23	-1.000466763	0.974	0.985	2.28E-19	MT-ATP8	5	IGLV3-1_plasma cell
2665	6.18E-24	-1.002243015	1	0.998	1.35E-19	AC090498.1	5	IGLV3-1_plasma cell
2666	1.67E-29	-1.006227666	0.974	0.988	3.65E-25	RPS4X	5	IGLV3-1_plasma cell
2667	1.66E-19	-1.00886254	0.855	0.913	3.63E-15	PABPC1	5	IGLV3-1_plasma cell
2668	3.38E-10	-1.010085857	0.118	0.462	7.36E-06	COTL1	5	IGLV3-1_plasma cell
2669	7.01E-09	-1.011693022	0.566	0.715	0.00015277	GLTSCR2	5	IGLV3-1_plasma cell
2670	2.02E-14	-1.013282792	0.75	0.797	4.42E-10	RPL23	5	IGLV3-1_plasma cell
2671	6.80E-26	-1.018403845	0.987	0.989	1.48E-21	RPS28	5	IGLV3-1_plasma cell
2672	1.46E-08	-1.022328694	0.342	0.575	0.000318696	HLA-DMA	5	IGLV3-1_plasma cell
2673	7.29E-24	-1.036081595	0.987	0.977	1.59E-19	RPL35A	5	IGLV3-1_plasma cell
2674	2.86E-19	-1.036347885	0.974	0.975	6.25E-15	FTH1	5	IGLV3-1_plasma cell
2675	3.62E-27	-1.051062254	1	0.999	7.89E-23	MT-ATP6	5	IGLV3-1_plasma cell
2676	9.72E-25	-1.065443386	0.961	0.972	2.12E-20	RPS9	5	IGLV3-1_plasma cell
2677	5.21E-27	-1.066136984	1	0.992	1.14E-22	RPL18	5	IGLV3-1_plasma cell
2678	4.37E-30	-1.068516865	1	1	9.53E-26	MT-CO1	5	IGLV3-1_plasma cell
2679	3.78E-26	-1.074758447	0.974	0.983	8.23E-22	RPL29	5	IGLV3-1_plasma cell
2680	1.70E-26	-1.076374312	0.947	0.974	3.71E-22	GNB2L1	5	IGLV3-1_plasma cell
2681	1.57E-27	-1.076611424	0.987	0.992	3.41E-23	RPS15	5	IGLV3-1_plasma cell
2682	1.04E-18	-1.079389251	0.868	0.863	2.28E-14	RPL36A	5	IGLV3-1_plasma cell
2683	5.99E-15	-1.083425412	0.803	0.819	1.31E-10	HMGB1	5	IGLV3-1_plasma cell
2684	1.11E-06	-1.089092054	0.105	0.361	0.024281193	GPR183	5	IGLV3-1_plasma cell
2685	2.29E-27	-1.113288314	0.961	0.962	5.00E-23	RPS11	5	IGLV3-1_plasma cell
2686	1.37E-26	-1.11369097	0.934	0.935	2.98E-22	RPL4	5	IGLV3-1_plasma cell
2687	2.11E-26	-1.114464638	0.974	0.973	4.60E-22	RPL5	5	IGLV3-1_plasma cell
2688	2.88E-20	-1.115085399	0.803	0.92	6.29E-16	CD79A	5	IGLV3-1_plasma cell
2689	1.65E-09	-1.115970174	0.632	0.723	3.59E-05	COMMD6	5	IGLV3-1_plasma cell
2690	6.92E-25	-1.120316273	0.895	0.919	1.51E-20	RPL27	5	IGLV3-1_plasma cell
2691	8.42E-15	-1.126121801	0.553	0.77	1.84E-10	RPS4Y1	5	IGLV3-1_plasma cell
2692	9.80E-15	-1.127235652	0.316	0.677	2.14E-10	CORO1A	5	IGLV3-1_plasma cell
2693	1.21E-27	-1.127774199	0.961	0.981	2.64E-23	RPS5	5	IGLV3-1_plasma cell
2694	2.11E-19	-1.128669078	0.921	0.917	4.61E-15	RPL38	5	IGLV3-1_plasma cell
2695	4.93E-18	-1.129036175	0.566	0.806	1.08E-13	PTPRCAP	5	IGLV3-1_plasma cell

2696	2.32E-21	-1.140586489	0.855	0.891	5.05E-17	RPL31	5	IGLV3-1_plasma cell
2697	2.42E-27	-1.147757462	0.987	0.975	5.28E-23	RPL36	5	IGLV3-1_plasma cell
2698	1.66E-25	-1.152284735	0.908	0.923	3.63E-21	PPIA	5	IGLV3-1_plasma cell
2699	1.56E-25	-1.156804599	1	0.997	3.40E-21	EEF1A1	5	IGLV3-1_plasma cell
2700	1.97E-24	-1.162077286	0.961	0.951	4.30E-20	RPL9	5	IGLV3-1_plasma cell
2701	1.11E-30	-1.163544561	0.974	0.983	2.42E-26	RPS16	5	IGLV3-1_plasma cell
2702	1.02E-21	-1.16554505	0.961	0.981	2.22E-17	TMSB10	5	IGLV3-1_plasma cell
2703	1.48E-30	-1.169637821	1	0.979	3.24E-26	RPL15	5	IGLV3-1_plasma cell
2704	4.42E-14	-1.185658152	0.224	0.613	9.64E-10	LIMD2	5	IGLV3-1_plasma cell
2705	4.07E-14	-1.187181667	0.434	0.695	8.88E-10	ARHGDIB	5	IGLV3-1_plasma cell
2706	8.27E-29	-1.188478295	0.934	0.959	1.80E-24	UBA52	5	IGLV3-1_plasma cell
2707	2.99E-27	-1.189468007	0.908	0.974	6.53E-23	RPL17	5	IGLV3-1_plasma cell
2708	4.39E-30	-1.192951686	0.947	0.978	9.56E-26	RPL37A	5	IGLV3-1_plasma cell
2709	3.73E-30	-1.194894517	0.987	0.991	8.14E-26	RPS15A	5	IGLV3-1_plasma cell
2710	7.09E-32	-1.199585435	0.987	0.982	1.55E-27	RPS7	5	IGLV3-1_plasma cell
2711	7.12E-29	-1.202377057	0.974	0.991	1.55E-24	RPS18	5	IGLV3-1_plasma cell
2712	5.69E-15	-1.204785257	0.697	0.794	1.24E-10	RPS20	5	IGLV3-1_plasma cell
2713	1.09E-27	-1.210868253	1	0.992	2.39E-23	PTMA	5	IGLV3-1_plasma cell
2714	4.19E-30	-1.218094648	0.987	0.99	9.15E-26	RPLP2	5	IGLV3-1_plasma cell
2715	3.65E-26	-1.22577348	0.974	0.974	7.95E-22	RPS25	5	IGLV3-1_plasma cell
2716	3.30E-24	-1.229446992	0.947	0.991	7.19E-20	ACTB	5	IGLV3-1_plasma cell
2717	1.60E-24	-1.240047088	0.987	0.989	3.49E-20	RPL34	5	IGLV3-1_plasma cell
2718	1.25E-24	-1.260902637	0.974	0.974	2.73E-20	RPL12	5	IGLV3-1_plasma cell
2719	3.34E-28	-1.269443371	0.947	0.973	7.29E-24	RPL22	5	IGLV3-1_plasma cell
2720	1.28E-14	-1.276872689	0.237	0.65	2.78E-10	PTPRC	5	IGLV3-1_plasma cell
2721	1.54E-30	-1.278200633	0.987	0.994	3.35E-26	RPL28	5	IGLV3-1_plasma cell
2722	2.03E-31	-1.288829417	0.934	0.974	4.44E-27	RPL23A	5	IGLV3-1_plasma cell
2723	1.30E-30	-1.291776696	0.974	0.982	2.83E-26	RPS13	5	IGLV3-1_plasma cell
2724	2.98E-29	-1.296283003	0.934	0.985	6.49E-25	RPS10	5	IGLV3-1_plasma cell
2725	3.85E-33	-1.297458036	0.934	0.983	8.39E-29	RPS24	5	IGLV3-1_plasma cell
2726	4.28E-33	-1.300104501	0.987	0.995	9.32E-29	RPS8	5	IGLV3-1_plasma cell
2727	6.33E-25	-1.303501057	0.947	0.952	1.38E-20	RPS29	5	IGLV3-1_plasma cell
2728	6.63E-13	-1.312190444	0.434	0.699	1.45E-08	ZFAS1	5	IGLV3-1_plasma cell
2729	2.23E-07	-1.322565317	0.434	0.592	0.004868472	ZFP36L1	5	IGLV3-1_plasma cell
2730	2.56E-31	-1.324488963	0.974	0.996	5.58E-27	RPL11	5	IGLV3-1_plasma cell
2731	7.02E-32	-1.326863371	0.974	0.991	1.53E-27	RPL19	5	IGLV3-1_plasma cell
2732	3.39E-23	-1.331047408	1	0.985	7.39E-19	RPL39	5	IGLV3-1_plasma cell
2733	7.44E-08	-1.343830389	0.276	0.515	0.001622684	SLC2A3	5	IGLV3-1_plasma cell
2734	4.83E-32	-1.343941382	0.987	0.99	1.05E-27	RPS3	5	IGLV3-1_plasma cell
2735	8.87E-30	-1.346675605	0.974	0.984	1.93E-25	RPL37	5	IGLV3-1_plasma cell
2736	8.87E-29	-1.354463514	0.961	0.976	1.93E-24	RPL21	5	IGLV3-1_plasma cell
2737	4.26E-29	-1.364498602	0.947	0.966	9.29E-25	RPL13A	5	IGLV3-1_plasma cell
2738	2.88E-31	-1.370628364	0.974	0.989	6.27E-27	RPS21	5	IGLV3-1_plasma cell
2739	1.09E-30	-1.377794598	1	0.994	2.38E-26	RPL13	5	IGLV3-1_plasma cell
2740	3.72E-29	-1.381595591	0.974	0.983	8.10E-25	RPS3A	5	IGLV3-1_plasma cell
2741	1.35E-28	-1.400234366	0.961	0.98	2.94E-24	RPL26	5	IGLV3-1_plasma cell
2742	2.54E-31	-1.407414011	0.974	0.988	5.54E-27	RPL18A	5	IGLV3-1_plasma cell
2743	3.94E-10	-1.417138382	0.763	0.833	8.58E-06	CXCR4	5	IGLV3-1_plasma cell
2744	4.77E-27	-1.418755544	1	0.985	1.04E-22	RPL32	5	IGLV3-1_plasma cell
2745	1.16E-26	-1.438044771	0.987	0.987	2.54E-22	RPL30	5	IGLV3-1_plasma cell
2746	1.66E-33	-1.446577535	0.895	0.977	3.62E-29	RPS2	5	IGLV3-1_plasma cell
2747	7.19E-17	-1.464174881	1	0.993	1.57E-12	CD74	5	IGLV3-1_plasma cell
2748	1.06E-32	-1.476140501	0.961	0.979	2.30E-28	RPS6	5	IGLV3-1_plasma cell
2749	3.53E-32	-1.477138269	0.934	0.966	7.69E-28	RPL10A	5	IGLV3-1_plasma cell
2750	5.03E-29	-1.49137484	0.987	0.988	1.10E-24	RPS12	5	IGLV3-1_plasma cell
2751	3.87E-30	-1.498838905	0.974	0.989	8.45E-26	RPS27A	5	IGLV3-1_plasma cell
2752	7.13E-31	-1.536396845	0.974	0.989	1.55E-26	RPS23	5	IGLV3-1_plasma cell
2753	7.66E-42	-1.611030705	1	1	1.67E-37	MT-CO3	5	IGLV3-1_plasma cell
2754	1.58E-19	-1.652375487	0.513	0.845	3.44E-15	LAPTM5	5	IGLV3-1_plasma cell
2755	1.85E-31	-1.66115252	1	0.995	4.04E-27	RPS19	5	IGLV3-1_plasma cell
2756	4.25E-25	-1.686339497	0.987	0.984	9.28E-21	RPS27	5	IGLV3-1_plasma cell
2757	9.28E-34	-1.735997548	0.868	0.963	2.02E-29	RPSA	5	IGLV3-1_plasma cell
2758	1.59E-24	-1.8357033	0.461	0.856	3.48E-20	HLA-DQB1	5	IGLV3-1_plasma cell
2759	1.14E-24	-1.843209755	0.158	0.762	2.49E-20	HLA-DRB5	5	IGLV3-1_plasma cell
2760	2.35E-13	-2.095824993	0.789	0.907	5.11E-09	BTG1	5	IGLV3-1_plasma cell
2761	4.15E-31	-2.103401764	0.211	0.848	9.05E-27	HLA-DPA1	5	IGLV3-1_plasma cell
2762	3.43E-21	-2.131233188	0.382	0.794	7.47E-17	CD37	5	IGLV3-1_plasma cell
2763	3.88E-25	-2.13532123	0.132	0.753	8.47E-21	HLA-DPB1	5	IGLV3-1_plasma cell
2764	6.24E-10	-2.146385687	0.237	0.526	1.36E-05	CD69	5	IGLV3-1_plasma cell
2765	3.46E-27	-2.264654458	0.132	0.775	7.55E-23	CD52	5	IGLV3-1_plasma cell
2766	2.32E-32	-2.470572299	0.276	0.878	5.07E-28	HLA-DRB1	5	IGLV3-1_plasma cell

2767	4.57E-30	-3.313857281	0.118	0.812	9.96E-26	HLA-DRA	5	IGLV3-1_plasma cell
2768	3.19E-10	1.533527211	0.553	0.21	6.95E-06	MT2A	6	MT2A_B
2769	5.05E-169	1.486172213	0.83	0.02	1.10E-164	FGR	6	MT2A_B
2770	8.61E-62	1.466909004	0.766	0.074	1.88E-57	FCRL3	6	MT2A_B
2771	1.80E-11	1.356271972	0.681	0.396	3.94E-07	FCRL5	6	MT2A_B
2772	4.02E-10	1.330323194	0.638	0.356	8.76E-06	CIB1	6	MT2A_B
2773	6.18E-12	1.275424122	0.596	0.239	1.35E-07	ADGRE5	6	MT2A_B
2774	1.22E-22	1.219083185	1	0.72	2.66E-18	HLA-DPB1	6	MT2A_B
2775	2.48E-20	1.212372142	0.957	0.448	5.40E-16	MS4A1	6	MT2A_B
2776	5.26E-36	1.212038215	0.617	0.09	1.15E-31	TNFRSF1B	6	MT2A_B
2777	4.60E-17	1.137557482	0.957	0.742	1.00E-12	CD52	6	MT2A_B
2778	3.25E-20	1.134376566	1	0.682	7.09E-16	HLA-DQA1	6	MT2A_B
2779	1.35E-15	1.132749945	0.936	0.731	2.95E-11	HLA-DRB5	6	MT2A_B
2780	1.41E-23	1.130507931	0.702	0.16	3.08E-19	CD72	6	MT2A_B
2781	4.22E-10	1.101603278	0.766	0.416	9.21E-06	CRIP1	6	MT2A_B
2782	7.27E-19	1.10148574	0.298	0.037	1.59E-14	CST7	6	MT2A_B
2783	8.50E-24	1.051731042	0.596	0.113	1.85E-19	ZEB2	6	MT2A_B
2784	2.07E-20	1.038093537	1	0.816	4.52E-16	HLA-DPA1	6	MT2A_B
2785	1.49E-19	1.011491231	1	0.826	3.25E-15	LAPTM5	6	MT2A_B
2786	8.74E-14	1.010723161	0.894	0.509	1.91E-09	DUSP2	6	MT2A_B
2787	2.14E-11	0.994441861	0.745	0.402	4.66E-07	TSPAN3	6	MT2A_B
2788	5.65E-24	0.994199301	0.362	0.042	1.23E-19	NKG7	6	MT2A_B
2789	7.98E-12	0.992557582	0.745	0.353	1.74E-07	MAP3K8	6	MT2A_B
2790	2.66E-10	0.979813931	0.617	0.288	5.79E-06	LY6E	6	MT2A_B
2791	2.61E-23	0.972398538	1	0.993	5.70E-19	CD74	6	MT2A_B
2792	2.16E-105	0.95341768	0.404	0.005	4.71E-101	SYT1	6	MT2A_B
2793	5.44E-14	0.953281644	0.574	0.188	1.19E-09	ITGB2	6	MT2A_B
2794	1.75E-07	0.935323693	0.66	0.449	0.003817398	RHOB	6	MT2A_B
2795	5.74E-09	0.933710673	0.511	0.218	0.000125111	PLEK	6	MT2A_B
2796	1.60E-15	0.929143333	0.979	0.835	3.48E-11	HLA-DQB1	6	MT2A_B
2797	2.13E-16	0.927667943	1	0.913	4.65E-12	CD79A	6	MT2A_B
2798	1.20E-12	0.923632741	0.723	0.373	2.62E-08	LITAF	6	MT2A_B
2799	1.53E-12	0.918331971	0.681	0.268	3.33E-08	FCRLA	6	MT2A_B
2800	3.97E-23	0.913243644	0.426	0.063	8.65E-19	MPP6	6	MT2A_B
2801	1.84E-17	0.90816974	0.617	0.146	4.02E-13	CD22	6	MT2A_B
2802	2.42E-20	0.903247508	0.617	0.134	5.27E-16	TMEM2	6	MT2A_B
2803	9.13E-17	0.889874903	1	0.776	1.99E-12	HLA-DRA	6	MT2A_B
2804	8.85E-15	0.878094163	1	0.848	1.93E-10	HLA-DRB1	6	MT2A_B
2805	3.71E-11	0.874266195	0.468	0.143	8.09E-07	CLECL1	6	MT2A_B
2806	2.90E-08	0.872713663	0.596	0.302	0.000631618	CSK	6	MT2A_B
2807	9.79E-75	0.852731225	0.404	0.011	2.13E-70	GPR137B	6	MT2A_B
2808	1.56E-11	0.849585582	0.681	0.304	3.40E-07	CD19	6	MT2A_B
2809	5.35E-11	0.846203531	0.787	0.439	1.17E-06	COTL1	6	MT2A_B
2810	3.95E-09	0.832852847	0.468	0.158	8.61E-05	ALOX5AP	6	MT2A_B
2811	5.21E-25	0.830094705	0.532	0.08	1.14E-20	SCIMP	6	MT2A_B
2812	1.04E-16	0.827637079	1	0.946	2.26E-12	TMSB4X	6	MT2A_B
2813	2.46E-07	0.82749368	0.489	0.225	0.005372566	SERPINB1	6	MT2A_B
2814	8.29E-12	0.826042707	0.915	0.625	1.81E-07	PTPRC	6	MT2A_B
2815	1.95E-07	0.822820211	0.489	0.228	0.004244286	LRMP	6	MT2A_B
2816	8.89E-13	0.798200142	0.553	0.16	1.94E-08	BCL2A1	6	MT2A_B
2817	1.72E-08	0.794038406	0.766	0.582	0.000374845	CD48	6	MT2A_B
2818	6.24E-10	0.770485813	0.383	0.101	1.36E-05	KMO	6	MT2A_B
2819	7.51E-07	0.769050672	0.723	0.572	0.01636871	GDI2	6	MT2A_B
2820	4.53E-16	0.767541034	0.596	0.148	9.89E-12	MTSS1	6	MT2A_B
2821	4.76E-31	0.766367646	0.468	0.051	1.04E-26	RIN3	6	MT2A_B
2822	2.51E-10	0.761338525	0.872	0.794	5.47E-06	PTPRCAP	6	MT2A_B
2823	3.34E-08	0.759052291	0.702	0.439	0.000728726	POU2F2	6	MT2A_B
2824	1.73E-07	0.754499039	0.489	0.233	0.003765072	S100A11	6	MT2A_B
2825	1.07E-07	0.749902249	0.723	0.494	0.002343709	PTPN6	6	MT2A_B
2826	4.53E-12	0.742069679	0.362	0.078	9.87E-08	ARL4D	6	MT2A_B
2827	9.92E-11	0.731426303	0.809	0.497	2.16E-06	ATP6V0E1	6	MT2A_B
2828	1.14E-40	0.730396567	0.34	0.019	2.48E-36	MS4A7	6	MT2A_B
2829	8.89E-36	0.728956479	0.298	0.017	1.94E-31	HCK	6	MT2A_B
2830	8.70E-09	0.722401599	0.426	0.148	0.00018975	ACP5	6	MT2A_B
2831	4.82E-08	0.71861221	0.766	0.548	0.001050458	7-Sep	6	MT2A_B
2832	7.26E-13	0.715541817	0.234	0.034	1.58E-08	SPON2	6	MT2A_B
2833	3.24E-12	0.701334202	0.191	0.023	7.07E-08	PPP1R14A	6	MT2A_B
2834	1.22E-11	0.698072354	0.936	0.855	2.67E-07	HLA-E	6	MT2A_B
2835	7.90E-19	0.694857697	0.362	0.05	1.72E-14	NCR3	6	MT2A_B
2836	1.67E-09	0.689248393	0.766	0.373	3.64E-05	BANK1	6	MT2A_B
2837	1.97E-10	0.665648158	0.298	0.061	4.29E-06	GPR18	6	MT2A_B

2838	1.53E-10	0.659938841	0.34	0.083	3.34E-06	CLN8	6	MT2A_B
2839	5.00E-09	0.659724904	0.851	0.694	0.000109061	ARPC2	6	MT2A_B
2840	1.50E-12	0.646976528	0.447	0.107	3.27E-08	EBF1	6	MT2A_B
2841	2.86E-38	0.63523808	0.298	0.015	6.23E-34	ITGB2-AS1	6	MT2A_B
2842	8.09E-31	0.634915972	0.277	0.017	1.76E-26	SPATA13	6	MT2A_B
2843	1.53E-06	0.633880094	0.553	0.266	0.033293916	ZNF331	6	MT2A_B
2844	1.96E-08	0.621423766	0.277	0.068	0.00042687	KLK1	6	MT2A_B
2845	1.29E-38	0.614260051	0.213	0.006	2.81E-34	LINC01013	6	MT2A_B
2846	1.20E-07	0.609148854	0.362	0.123	0.002625266	CYTH4	6	MT2A_B
2847	1.01E-06	0.605259826	0.553	0.29	0.022067955	MEF2A	6	MT2A_B
2848	1.16E-06	0.600607262	0.66	0.409	0.025191904	CNPY3	6	MT2A_B
2849	8.58E-30	0.59917293	0.213	0.01	1.87E-25	₹P11-444D3.	6	MT2A_B
2850	2.41E-11	0.59719556	0.362	0.081	5.25E-07	CBLB	6	MT2A_B
2851	2.17E-06	0.588801961	0.426	0.167	0.047305981	PARP15	6	MT2A_B
2852	3.95E-26	0.587988114	0.277	0.021	8.61E-22	CD84	6	MT2A_B
2853	3.55E-10	0.579286078	0.255	0.05	7.74E-06	IFNLR1	6	MT2A_B
2854	5.30E-10	0.574777656	0.681	0.25	1.16E-05	LBH	6	MT2A_B
2855	1.95E-08	0.567414209	0.894	0.75	0.000425547	RHOA	6	MT2A_B
2856	1.68E-07	0.563764152	0.957	0.753	0.00366111	EZR	6	MT2A_B
2857	1.43E-16	0.560900994	0.234	0.026	3.12E-12	FGD4	6	MT2A_B
2858	1.05E-10	0.559546892	1	0.973	2.29E-06	MT-ND4	6	MT2A_B
2859	2.02E-07	0.557839013	0.532	0.214	0.004410993	TRBC2	6	MT2A_B
2860	1.84E-06	0.554485264	0.872	0.739	0.040134967	SH3BGRL3	6	MT2A_B
2861	9.03E-08	0.551072227	0.574	0.225	0.001969096	1-Mar	6	MT2A_B
2862	4.97E-59	0.543104169	0.213	0.002	1.08E-54	ITGAX	6	MT2A_B
2863	1.18E-10	0.53806691	0.319	0.071	2.57E-06	SH3BP2	6	MT2A_B
2864	1.35E-06	0.514207158	0.809	0.591	0.029438292	LIMD2	6	MT2A_B
2865	8.12E-12	0.507026626	1	1	1.77E-07	MALAT1	6	MT2A_B
2866	6.53E-09	0.505261252	0.213	0.039	0.000142475	TTN	6	MT2A_B
2867	1.29E-06	0.500559206	0.979	0.874	0.028174943	PFN1	6	MT2A_B
2868	4.97E-15	0.497210295	0.149	0.011	1.08E-10	GALNTL6	6	MT2A_B
2869	5.95E-10	0.490542486	0.213	0.035	1.30E-05	MEF2C-AS1	6	MT2A_B
2870	1.01E-09	0.474962935	0.277	0.058	2.20E-05	PPP1R16B	6	MT2A_B
2871	2.37E-09	0.470076404	0.234	0.044	5.17E-05	LRRK2	6	MT2A_B
2872	4.03E-08	0.468586995	0.17	0.029	0.000878372	TFEC	6	MT2A_B
2873	1.96E-06	0.46810161	0.532	0.217	0.042691198	ARHGAP24	6	MT2A_B
2874	1.69E-07	0.46746824	0.298	0.078	0.003682255	JAZF1	6	MT2A_B
2875	2.96E-09	0.462772263	0.234	0.045	6.46E-05	INPP5F	6	MT2A_B
2876	5.75E-07	0.462105636	0.979	0.86	0.012529501	RPL36A	6	MT2A_B
2877	3.61E-08	0.455991352	0.979	0.958	0.000787486	UBA52	6	MT2A_B
2878	1.14E-08	0.454939545	0.149	0.021	0.000247941	HOPX	6	MT2A_B
2879	4.37E-25	0.454822949	0.191	0.01	9.52E-21	SLC11A1	6	MT2A_B
2880	1.74E-11	0.449568504	1	1	3.80E-07	MT-CO3	6	MT2A_B
2881	9.03E-11	0.447001645	1	0.974	1.97E-06	FTH1	6	MT2A_B
2882	6.24E-09	0.445609729	0.255	0.057	0.00013609	UBASH3B	6	MT2A_B
2883	4.54E-09	0.441277384	1	0.999	9.91E-05	MT-ATP6	6	MT2A_B
2884	1.24E-15	0.438744668	0.234	0.026	2.71E-11	LIMS2	6	MT2A_B
2885	1.71E-07	0.43854235	1	0.982	0.003734743	HLA-B	6	MT2A_B
2886	7.86E-08	0.430643372	0.979	0.974	0.001713663	RPL12	6	MT2A_B
2887	1.02E-15	0.419540725	0.149	0.01	2.23E-11	TUBB6	6	MT2A_B
2888	4.20E-07	0.41556177	0.979	0.95	0.009152242	MT-ND2	6	MT2A_B
2889	8.92E-25	0.414926762	0.17	0.007	1.94E-20	MCTP1	6	MT2A_B
2890	1.33E-07	0.413217801	0.255	0.061	0.002907223	MNDA	6	MT2A_B
2891	2.25E-13	0.411769579	1	1	4.92E-09	MT-CO1	6	MT2A_B
2892	4.41E-14	0.410465419	0.234	0.029	9.62E-10	SGK223	6	MT2A_B
2893	7.42E-07	0.409894738	0.213	0.051	0.016178523	SACS	6	MT2A_B
2894	1.31E-15	0.409582244	0.213	0.022	2.85E-11	LCP2	6	MT2A_B
2895	2.05E-06	0.40422042	0.957	0.9	0.044682346	OAZ1	6	MT2A_B
2896	1.33E-06	0.398906282	0.957	0.892	0.02910382	COX4I1	6	MT2A_B
2897	5.71E-07	0.397198773	1	0.989	0.012460567	ACTB	6	MT2A_B
2898	1.74E-07	0.390212007	1	0.997	0.00380181	MT-ND3	6	MT2A_B
2899	9.39E-08	0.378217293	0.17	0.03	0.00204785	VNN2	6	MT2A_B
2900	4.89E-07	0.37695912	1	0.983	0.010663866	RPL37	6	MT2A_B
2901	2.65E-07	0.375950786	0.17	0.031	0.005770541	MX2	6	MT2A_B
2902	1.55E-08	0.368115774	0.553	0.174	0.000339063	HLA-DMB	6	MT2A_B
2903	1.65E-12	0.355377498	0.17	0.018	3.61E-08	TBX21	6	MT2A_B
2904	2.28E-07	0.352844781	1	0.998	0.004964431	AC090498.1	6	MT2A_B
2905	9.10E-29	0.350297029	0.17	0.006	1.98E-24	LILRB2	6	MT2A_B
2906	6.15E-07	0.350001206	0.149	0.026	0.013405231	NKRF	6	MT2A_B
2907	8.81E-08	0.348283559	0.213	0.042	0.001921488	FAM46A	6	MT2A_B
2908	1.49E-10	0.34714444	1	0.998	3.24E-06	B2M	6	MT2A_B

2909	2.65E-16	0.340857371	0.128	0.007	5.79E-12	GAS7	6	MT2A_B
2910	6.03E-08	0.338071202	0.213	0.042	0.001315103	SCML4	6	MT2A_B
2911	9.31E-07	0.337575067	0.298	0.083	0.020308557	HLA-DOA	6	MT2A_B
2912	4.52E-10	0.33682786	0.149	0.018	9.87E-06	DPYSL2	6	MT2A_B
2913	3.37E-07	0.332216479	0.574	0.243	0.007346823	HSPB1	6	MT2A_B
2914	1.91E-42	0.329425441	0.106	0	4.16E-38	RBPMS2	6	MT2A_B
2915	1.15E-09	0.325688526	1	0.999	2.52E-05	MT-CYB	6	MT2A_B
2916	6.85E-09	0.319526582	0.128	0.015	0.000149429	C11orf21	6	MT2A_B
2917	2.09E-13	0.316212055	0.191	0.021	4.56E-09	TP53I11	6	MT2A_B
2918	3.75E-07	0.314169927	1	0.985	0.008174311	MT-ATP8	6	MT2A_B
2919	5.20E-07	0.306887896	0.149	0.026	0.01134939	₹P11-25K19.	6	MT2A_B
2920	1.88E-26	0.301628494	0.106	0.002	4.10E-22	SIGLEC6	6	MT2A_B
2921	3.05E-30	0.299038433	0.106	0.001	6.65E-26	MFAP5	6	MT2A_B
2922	5.32E-07	0.295340904	0.106	0.014	0.011596607	CAB39L	6	MT2A_B
2923	4.56E-08	0.289573054	0.106	0.011	0.000993928	LINC01504	6	MT2A_B
2924	1.89E-23	0.283748806	0.106	0.002	4.11E-19	DUSP15	6	MT2A_B
2925	1.69E-11	0.270453268	0.106	0.007	3.69E-07	ZFP30	6	MT2A_B
2926	1.23E-12	0.267975809	0.128	0.01	2.68E-08	ANK3	6	MT2A_B
2927	5.13E-16	0.26571727	0.106	0.005	1.12E-11	CREB5	6	MT2A_B
2928	1.09E-12	0.258556421	0.149	0.013	2.39E-08	GRAP2	6	MT2A_B
2929	8.94E-07	0.255844464	1	0.999	0.019503185	MT-ND4L	6	MT2A_B
2930	2.26E-17	0.250115408	0.106	0.004	4.93E-13	SYT11	6	MT2A_B
2931	7.28E-07	-0.753436066	0.106	0.483	0.015885208	FKBP2	6	MT2A_B
2932	7.89E-07	-0.823597694	0.149	0.515	0.017203606	OSTC	6	MT2A_B
2933	1.16E-06	-0.971655405	0.17	0.502	0.025300815	LMAN1	6	MT2A_B
2934	7.94E-07	-1.000009443	0.106	0.46	0.017324547	SDF2L1	6	MT2A_B
2935	2.04E-07	-1.005568893	0.128	0.498	0.004446389	KDELR2	6	MT2A_B
2936	1.31E-06	-1.048132504	0.213	0.523	0.028666955	ITM2C	6	MT2A_B
2937	8.14E-08	-1.110688131	0.106	0.493	0.001775328	SPCS3	6	MT2A_B
2938	5.62E-07	-1.165575915	0.34	0.623	0.012245526	HSPA5	6	MT2A_B
2939	5.03E-07	-1.206271263	0.191	0.512	0.010980326	SSR3	6	MT2A_B
2940	4.70E-07	-1.615018911	0.191	0.511	0.010253139	XBP1	6	MT2A_B
2941	4.54E-07	-1.872995562	0.489	0.673	0.009908128	HSP90B1	6	MT2A_B
2942	3.21E-10	-3.660951514	0.255	0.642	7.00E-06	JCHAIN	6	MT2A_B

Table 19: List of DEGs of B/plasma cell clusters in reperfusion stage (PR versus EP)

List of differentially expressed genes of TCL1A_B in reperfusion stage (PR versus EP)

gene	p_val	avg_logFC	pct.1	pct.2	p_val_adj
HLA-DRB1	1.69E-22	0.744614242	0.993	0.909	3.69E-18
RPS29	1.24E-19	0.477193332	0.98	0.963	2.70E-15
EZR	9.19E-18	0.883420533	0.859	0.477	2.01E-13
DEFA3	3.26E-16	-1.317341296	0.02	0.391	7.11E-12
HBB	8.59E-15	-1.184467256	0.06	0.428	1.87E-10
HSPD1	3.31E-14	1.437538703	0.597	0.288	7.21E-10
CD52	7.88E-13	-0.540645341	0.765	0.918	1.72E-08
TMSB4X	9.13E-12	-0.405825787	0.953	0.996	1.99E-07
HSPH1	1.63E-11	1.210657831	0.463	0.169	3.56E-07
FTH1	7.47E-11	0.601408743	0.987	0.984	1.63E-06
IRF1	1.44E-10	0.836845924	0.523	0.239	3.15E-06
HSPB1	3.40E-10	1.174585413	0.416	0.156	7.41E-06
NCF1	4.24E-10	-0.689886089	0.315	0.617	9.25E-06
DUSP1	6.21E-10	-0.460712268	0.906	0.934	1.35E-05
CREM	1.16E-08	0.90679892	0.349	0.119	0.000252916
RPS2	5.76E-08	0.289889048	0.98	0.963	0.001256644
GADD45B	6.11E-08	0.547805398	0.826	0.584	0.001332831
IGKV4-1	6.45E-08	-0.702885271	0.128	0.391	0.001406742
POLD4	1.17E-07	-0.610918625	0.188	0.453	0.002557463
HSP90AB1	1.58E-07	0.653883322	0.913	0.765	0.003436944
FAM96A	2.06E-07	-0.691994128	0.114	0.337	0.004485478
IGLV2-14	2.93E-07	-0.35775404	0.107	0.358	0.00637909
IGHM	3.30E-07	-0.58993278	0.369	0.605	0.007193809
PELI1	3.32E-07	0.694590819	0.295	0.099	0.007236103
FCMR	3.35E-07	-0.517085654	0.49	0.691	0.007297734
TNFAIP8	4.70E-07	-0.567740629	0.295	0.514	0.010253585
SNX9	4.83E-07	0.660104964	0.403	0.181	0.010522997
IGKV2-30	6.51E-07	-0.705280316	0.067	0.267	0.014192662
IFITM1	9.28E-07	0.700974876	0.369	0.16	0.020235656
ZC3H12A	1.04E-06	0.597831773	0.349	0.144	0.022757075
HVCN1	1.40E-06	-0.590860347	0.262	0.477	0.030507031
TMEM123	1.91E-06	0.607656966	0.591	0.387	0.041547935
RNASE6	2.07E-06	-0.591719273	0.282	0.506	0.045189025

List of differentially expressed genes of IGKV1D-16_plasma in reperfusion stage (PR versus EP)

gene	p_val	avg_logFC	pct.1	pct.2	p_val_adj
EZR	2.31E-18	0.894453505	0.956	0.716	5.04E-14
TYMP	4.23E-13	0.55543685	0.689	0.273	9.23E-09
VIM	1.14E-12	0.760901904	0.933	0.903	2.49E-08
DNAAF1	1.64E-12	1.091029916	0.456	0.091	3.58E-08
IGKV1-17	2.24E-12	3.552342257	0.289	0.011	4.88E-08
EIF1	3.45E-12	0.461241033	0.978	0.926	7.51E-08
TPM4	6.51E-12	0.580554235	0.9	0.682	1.42E-07
RPS29	6.89E-12	0.569386007	0.922	0.858	1.50E-07
IGHV3-72	1.24E-11	1.065752085	0.311	0.023	2.71E-07
SAT1	8.99E-11	0.833640151	0.978	0.881	1.96E-06
HSPB1	1.47E-10	0.651749758	0.356	0.057	3.22E-06
PDIA4	4.31E-10	-0.527938442	0.9	0.955	9.39E-06
MALAT1	5.91E-10	0.542628387	1	1	1.29E-05
IGLV1-44	9.80E-10	1.021771034	0.278	0.028	2.14E-05
CHST2	1.57E-09	0.363326833	0.633	0.244	3.43E-05
MT-ATP6	3.32E-09	-0.616311639	1	1	7.24E-05
SPCS2	3.95E-09	-0.36397345	0.922	0.949	8.62E-05
HIST1H2BN	6.41E-09	-0.385041272	0.033	0.369	0.000139737
SLC7A5	1.00E-08	0.502688651	0.8	0.466	0.000218552
IGHV1-18	1.04E-08	0.629723709	0.233	0.017	0.000226084
HSP90AA1	1.45E-08	0.757754762	0.933	0.824	0.000316228
PSMB8	2.27E-08	-0.412529377	0.422	0.716	0.000496128
DEFA3	2.81E-08	0.325578255	0.078	0.42	0.000612222
IGHV3-30	4.98E-08	0.501741817	0.456	0.125	0.001085132
ZFP36	5.38E-08	0.517847435	0.956	0.892	0.001173022
IGHV3-48	5.78E-08	1.064220758	0.289	0.045	0.001260804
JUN	6.69E-08	-0.97789997	0.633	0.835	0.001459396

FAM49A	9.04E-08	0.294967126	0.578	0.261	0.001971712
YWHAH	2.16E-07	0.366489462	0.544	0.25	0.004700104
PELI1	2.46E-07	0.443671132	0.689	0.409	0.005362781
IGKV1-9	2.88E-07	2.562857523	0.233	0.034	0.006271763
MT-CO3	3.03E-07	-0.425817768	1	1	0.006608162
IGLV2-8	3.50E-07	1.833160499	0.378	0.108	0.007640772
IGHV3-21	4.75E-07	0.64646531	0.244	0.04	0.010367655
IGHV3-11	4.83E-07	3.926195176	0.244	0.04	0.010537522
IGKV3-11	5.70E-07	0.779063639	0.389	0.125	0.01243697
ZBTB32	6.09E-07	0.362080942	0.422	0.159	0.013281535
MIR4435-2HG	7.19E-07	0.527270486	0.667	0.438	0.015671281
PIM3	9.23E-07	0.375674177	0.589	0.341	0.020137205
RPN1	1.08E-06	-0.354951984	0.833	0.92	0.023577566
LINC01480	1.11E-06	0.561602245	0.611	0.341	0.024309764
CALR	1.16E-06	-0.455492422	0.944	0.932	0.025241228
IGKV3D-20	1.22E-06	4.764920456	0.289	0.074	0.026532796
EIF5	1.31E-06	0.451474677	0.867	0.625	0.028533917
SAR1B	1.72E-06	-0.364105632	0.544	0.773	0.037545909
FAM69A	1.93E-06	-0.269786718	0.111	0.381	0.041987322
BST2	2.11E-06	-0.416545858	0.389	0.625	0.045912288

List of differentially expressed genes of GPR183_B in reperfusion stage (PR versus EP)

gene	p_val	avg_logFC	pct.1	pct.2	p_val_adj
EZR	3.53E-17	0.981303692	0.943	0.683	7.70E-13
CD52	1.25E-16	-0.708733064	0.764	0.976	2.73E-12
RPS29	9.16E-15	0.451423781	0.984	0.984	2.00E-10
HBB	5.50E-14	-1.042700008	0.033	0.431	1.20E-09
HLA-DRB1	1.07E-13	0.636032379	0.984	0.967	2.33E-09
HSPD1	1.10E-13	1.564488757	0.74	0.415	2.40E-09
DEFA3	8.69E-12	-1.011090756	0.024	0.374	1.89E-07
HSPB1	2.49E-11	1.656011428	0.675	0.301	5.44E-07
EIF1	9.43E-10	0.408901316	1	0.992	2.06E-05
PIM3	4.21E-09	0.828393449	0.415	0.098	9.18E-05
HSP90AA1	4.27E-09	1.149603516	0.894	0.846	9.31E-05
HSP90AB1	5.97E-09	0.588339669	0.959	0.846	0.000130092
HSPH1	1.22E-08	1.092432173	0.602	0.301	0.000266162
SRGN	1.46E-08	0.76904541	0.789	0.577	0.000318901
CREM	2.42E-08	0.993261999	0.512	0.187	0.000528333
FCMR	4.19E-08	-0.554871295	0.358	0.748	0.000914314
YWHAH	1.46E-07	0.638445169	0.423	0.13	0.003177082
PTMA	1.75E-07	0.319571753	1	0.992	0.003825404
AC006129.2	3.67E-07	-0.644790223	0.073	0.333	0.008003677
IRF1	4.60E-07	0.714053982	0.52	0.236	0.010038415
CD79B	4.78E-07	-0.554268705	0.317	0.61	0.010429248
METTL7A	6.40E-07	-0.560605015	0.024	0.236	0.013964794
NCF1	6.46E-07	-0.549065659	0.293	0.61	0.014081769
LRRFIP1	8.17E-07	0.803430111	0.659	0.48	0.017810221
KLF10	8.21E-07	0.757095019	0.423	0.154	0.017911542
CD37	8.23E-07	-0.310596662	0.919	1	0.017937145
TMSB4X	1.12E-06	-0.331347773	0.976	1	0.024367418
ZFP36	1.16E-06	0.470283247	0.976	0.919	0.025222046
TMSB10	1.20E-06	-0.417932923	0.967	0.992	0.026196283
NUDC	1.20E-06	0.652971897	0.455	0.195	0.026238314
RNASE6	1.27E-06	-0.688913629	0.179	0.455	0.027753799
TXNIP	1.50E-06	-0.442354791	0.659	0.894	0.032802561

List of differentially expressed genes of HIST1H4C_plasma in reperfusion stage (PR versus EP)

gene	p_val	avg_logFC	pct.1	pct.2	p_val_adj
SMC4	1.96E-08	-1.083428432	0.071	0.938	0.000427668
TMCO1	6.76E-08	-0.691651724	0.071	0.927	0.001474304
PREB	7.10E-08	-0.874947523	0.143	0.938	0.001549154
MT1G	1.18E-07	1.125531564	0.286	0	0.002583345
CD48	2.93E-07	-0.776327859	0.071	0.865	0.006381248

EIF4G2	4.69E-07	-0.850663102	0.357	0.979	0.010236239
UAP1	4.81E-07	-0.866269685	0.214	0.885	0.010495265
TNFRSF17	5.02E-07	-1.17089351	0.357	0.969	0.010938138
CPNE5	5.42E-07	-0.631979484	0.071	0.865	0.01182234
H2AFV	6.57E-07	-0.812014123	0.571	0.99	0.014318852
TYMS	6.83E-07	-0.987795847	0.143	0.896	0.014897426
POLR2E	7.05E-07	-0.652442546	0.071	0.896	0.015377085
STMN1	7.54E-07	-1.043484265	0.5	1	0.016447416
HNRNPA2B1	1.04E-06	-0.80645167	0.714	1	0.022720358
RRM2	1.06E-06	-1.322436268	0.214	0.927	0.023055982
MRPL27	1.43E-06	-0.624920618	0.071	0.833	0.03128374
RAC1	1.66E-06	-0.735377044	0.214	0.958	0.036217394
CALR	1.87E-06	-0.949091794	0.786	1	0.040797438
PPP1CA	2.13E-06	-0.842162784	0.357	0.948	0.046529994
SPCS2	2.23E-06	-0.75550275	0.714	1	0.048699656

List of differentially expressed genes of IGLV3-1_plasma in reperfusion stage (PR versus EP)

gene	p_val	avg_logFC	pct.1	pct.2	p_val_adj
NA					

List of differentially expressed genes of MT2A_B in reperfusion stage (PR versus EP)

gene	p_val	avg_logFC	pct.1	pct.2	p_val_adj
NA					

Table 20: List of DEGs of B/plasma cell clusters in overall stage (PR versus PP)

List of differentially expressed genes of TCL1A_B in overall stage (PR versus PP)

gene	p_val	avg_logFC	pct.1	pct.2	p_val_adj
DEFA3	2.55E-23	-1.396640005	0.02	0.532	5.56E-19
RPS29	4.25E-17	0.431807446	0.98	0.98	9.26E-13
HBB	1.41E-16	-1.244928267	0.06	0.478	3.07E-12
GADD45B	2.26E-16	1.031445797	0.826	0.557	4.92E-12
HLA-DRB1	3.36E-16	0.611811516	0.993	0.94	7.32E-12
TMSB4X	3.12E-15	-0.478509329	0.953	1	6.80E-11
CD52	9.07E-15	-0.612106694	0.765	0.96	1.98E-10
EZR	1.40E-14	0.826528643	0.859	0.567	3.05E-10
HSP90AA1	2.91E-14	1.386344513	0.839	0.662	6.34E-10
IRF1	7.95E-13	1.017047393	0.523	0.209	1.73E-08
HSP90AB1	1.03E-12	0.859360976	0.913	0.736	2.26E-08
HSPD1	2.34E-12	1.412823656	0.597	0.318	5.10E-08
FTH1	6.97E-12	0.626937347	0.987	0.985	1.52E-07
TCL1A	2.10E-11	-0.771042031	0.376	0.726	4.59E-07
HSPH1	2.26E-11	1.366064472	0.463	0.169	4.93E-07
CD37	2.70E-10	-0.325343613	0.953	1	5.89E-06
RNASE6	4.69E-10	-0.699004245	0.282	0.617	1.02E-05
ZNF331	6.47E-10	0.875698523	0.51	0.209	1.41E-05
HVCN1	1.10E-09	-0.659334317	0.262	0.592	2.40E-05
RPS19	2.11E-09	-0.258397444	1	1	4.60E-05
MT-ND3	4.17E-09	0.405106245	1	0.995	9.09E-05
FCMR	1.23E-08	-0.579154687	0.49	0.741	0.000267781
TNFAIP8	4.31E-08	-0.608547369	0.295	0.557	0.000939548
NFKBIA	8.39E-08	0.651343256	0.785	0.672	0.001829553
SNX9	9.81E-08	0.736005737	0.403	0.164	0.002139173
POLD4	1.34E-07	-0.5788944	0.188	0.473	0.002918239
SAT1	1.39E-07	0.6775107	0.725	0.517	0.003036815
MT2A	2.37E-07	2.920102573	0.228	0.045	0.005167088
REL	3.15E-07	0.710776719	0.664	0.483	0.006860835
TMSB10	6.12E-07	-0.27010603	0.973	1	0.01334352
TCP1	6.39E-07	0.685481159	0.309	0.104	0.013933483
NCF1	8.74E-07	-0.524608384	0.315	0.597	0.019052751
FAM96A	1.22E-06	-0.542573815	0.114	0.343	0.026679377

List of differentially expressed genes of IGKV1D-16_plasma in overall stage (PR versus PP)

gene	p_val	avg_logFC	pct.1	pct.2	p_val_adj
DNAAF1	1.82E-16	1.169179665	0.456	0.065	3.96E-12
MALAT1	4.45E-15	0.662227178	1	1	9.70E-11
HSP90AA1	6.91E-14	1.021153317	0.933	0.808	1.51E-09
EZR	1.22E-13	0.72055693	0.956	0.818	2.66E-09
TPM4	1.51E-13	0.622662579	0.9	0.678	3.30E-09
SPCS2	1.22E-12	-0.440041126	0.922	0.963	2.66E-08
HSPB1	2.65E-12	0.651127115	0.356	0.051	5.79E-08
RPS29	7.06E-12	0.504307661	0.922	0.869	1.54E-07
EAF2	2.08E-11	-0.365351431	0.3	0.687	4.53E-07
MT2A	2.44E-11	2.736085184	0.378	0.07	5.33E-07
SLC7A5	2.68E-11	0.673436723	0.8	0.533	5.85E-07
VIM	3.65E-11	0.699034332	0.933	0.921	7.97E-07
EHMT1	5.62E-11	0.63060262	0.7	0.369	1.23E-06
PDIA4	5.66E-11	-0.4948748	0.9	0.958	1.23E-06
TYMP	6.43E-11	0.518303615	0.689	0.388	1.40E-06
PSMB8	9.68E-11	-0.495572382	0.422	0.757	2.11E-06
CALR	1.34E-10	-0.511761998	0.944	0.972	2.92E-06
IGHV3-30	2.07E-10	0.675198868	0.456	0.112	4.51E-06
FAM49A	6.14E-10	0.332148762	0.578	0.252	1.34E-05
NDUFB3	2.79E-09	-0.418820105	0.433	0.762	6.09E-05
SAR1B	5.54E-09	-0.40715594	0.544	0.836	0.000120911
IGKV1-9	6.51E-09	2.878618613	0.233	0.028	0.000141904
IGKV1-17	7.68E-09	3.510662815	0.289	0.056	0.000167562
HBB	8.66E-09	-0.519874973	0.133	0.495	0.000188919
RELB	9.06E-09	0.313828563	0.578	0.271	0.000197572
SAT1	1.15E-08	0.640205368	0.978	0.907	0.000250145
COPB2	1.78E-08	-0.383392289	0.422	0.724	0.0003881

PPIB	2.74E-08	-0.384803443	0.989	0.986	0.000598571
TNFRSF17	2.83E-08	-0.559257877	0.7	0.86	0.000616887
NEAT1	7.63E-08	0.27853941	0.967	0.953	0.001663885
IGLV2-23	7.84E-08	0.791557322	0.378	0.112	0.001709261
RP11-16E12.2	9.98E-08	-0.362405969	0.289	0.626	0.002176998
MDH1	1.00E-07	-0.324589404	0.244	0.551	0.002187418
AKAP2	1.11E-07	-0.282632456	0.133	0.444	0.002415864
PSMB9	1.18E-07	-0.368044008	0.444	0.738	0.002571383
YWHAH	1.72E-07	0.391762187	0.544	0.308	0.003759647
IGHV3-21	1.80E-07	3.250999658	0.244	0.042	0.003929853
ATP5J	2.73E-07	-0.315672603	0.789	0.841	0.005955853
SSR1	3.40E-07	-0.410390655	0.744	0.888	0.00741683
LMAN2	4.02E-07	-0.334911086	0.822	0.939	0.008756779
AREG	4.16E-07	0.404307321	0.267	0.065	0.009081932
SEC14L1	4.19E-07	-0.361274106	0.6	0.808	0.009136576
PSMA3	4.38E-07	-0.296831927	0.389	0.696	0.009554031
HMGB1	4.92E-07	-0.540461855	0.811	0.911	0.010726092
DCPS	5.27E-07	-0.325310507	0.289	0.593	0.011499709
UBE2J1	6.10E-07	-0.403373692	0.9	0.93	0.013309453
MIR4435-2HG	6.93E-07	0.524883023	0.667	0.491	0.015112444
SMARCB1	7.26E-07	-0.281534268	0.333	0.654	0.015840281
IGLV7-43	7.53E-07	3.912447638	0.111	0	0.016427911
CHST2	8.08E-07	0.269963533	0.633	0.369	0.017620586
VDAC3	1.04E-06	-0.260169402	0.2	0.519	0.022577124
BST2	1.59E-06	-0.359680338	0.389	0.668	0.03475817
IGHV3-11	1.67E-06	3.939931439	0.244	0.056	0.036509713
CREM	1.76E-06	0.281933049	0.389	0.159	0.038489127
PSMA5	1.99E-06	-0.307138998	0.467	0.734	0.043317487
ATPIF1	2.08E-06	-0.367012885	0.511	0.734	0.045455296

List of differentially expressed genes of GPR183_B in overall stage (PR versus PP)

gene	p_val	avg_logFC	pct.1	pct.2	p_val_adj
DEFA3	1.97E-18	-1.221424172	0.024	0.558	4.29E-14
HBB	7.35E-18	-1.368766083	0.033	0.537	1.60E-13
RPS29	4.04E-17	0.564090706	0.984	1	8.80E-13
CD52	4.12E-12	-0.666089992	0.764	0.979	8.98E-08
HSPD1	7.88E-12	1.564709623	0.74	0.432	1.72E-07
HSP90AA1	1.98E-11	1.421947849	0.894	0.758	4.32E-07
EZR	1.19E-10	0.766933236	0.943	0.747	2.59E-06
HSPB1	2.68E-10	1.635780701	0.675	0.305	5.85E-06
TMSB4X	2.95E-10	-0.417065343	0.976	1	6.43E-06
HSPH1	2.89E-09	1.253519792	0.602	0.263	6.31E-05
LRRFIP1	2.67E-08	0.990151726	0.659	0.411	0.000581966
HSPA1A	3.66E-08	1.579430451	0.618	0.274	0.000798175
POLD4	4.14E-08	-0.76150346	0.252	0.579	0.000903709
PTPN6	8.80E-08	-0.557080425	0.333	0.716	0.001918839
IRF1	8.82E-08	0.861092718	0.52	0.179	0.001922679
CD24	1.39E-07	-0.751332498	0.285	0.589	0.003027671
GADD45B	1.49E-07	0.780652529	0.756	0.453	0.003258048
HSPE1	1.64E-07	1.280226969	0.659	0.421	0.003578111
TMSB10	1.76E-07	-0.45068214	0.967	1	0.003836033
HLA-DRB1	3.36E-07	0.470093017	0.984	0.958	0.007332278
HSP90AB1	4.63E-07	0.54363969	0.959	0.895	0.010100312
NCF1	4.75E-07	-0.654224898	0.293	0.642	0.010359038
TXNIP	8.56E-07	-0.469103035	0.659	0.968	0.018665676
NUDC	1.11E-06	0.707009041	0.455	0.147	0.024229985
NEAT1	1.30E-06	0.73028563	0.748	0.516	0.028245001
FCGR2B	1.63E-06	-0.634884241	0.171	0.484	0.035634884
SAT1	1.99E-06	0.713499832	0.87	0.747	0.043290176

List of differentially expressed genes of HIST1H4C_plasma in overall stage (PR versus PP)

gene	p_val	avg_logFC	pct.1	pct.2	p_val_adj
SMC4	7.94E-09	-1.118661875	0.071	0.94	0.00017312

MT1G	8.43E-09	1.118679354	0.286	0.007	0.000183825
PREB	2.64E-08	-0.866924694	0.143	0.946	0.000576702
HNRNPA2B1	3.95E-08	-0.969171444	0.714	1	0.000860606
PSMD11	4.32E-08	-0.656648191	0.143	0.926	0.000942367
PPP1CA	4.64E-08	-0.970999204	0.357	0.987	0.00101281
STMN1	4.82E-08	-1.208135099	0.5	1	0.001050844
UAP1	5.16E-08	-1.002419793	0.214	0.933	0.001125273
CD48	5.59E-08	-0.78051479	0.071	0.893	0.001218622
TNFRSF17	7.15E-08	-1.159190103	0.357	0.987	0.001558764
MRPL27	9.02E-08	-0.632297117	0.071	0.906	0.001967077
TYMS	9.09E-08	-1.199757967	0.143	0.906	0.001981302
H2AFV	9.32E-08	-0.905970941	0.571	0.993	0.002033468
TMCO1	9.40E-08	-0.654286173	0.071	0.899	0.002050184
RAC1	1.23E-07	-0.862775372	0.214	0.96	0.002683555
SAA1	1.28E-07	0.617421598	0.357	0.027	0.002787673
BCL2L12	1.51E-07	-0.579899881	0	0.846	0.003289261
POLR2E	1.69E-07	-0.803801734	0.071	0.899	0.003675567
NUDT5	1.74E-07	-0.7235822	0.143	0.906	0.00378892
RRM2	1.75E-07	-1.65361618	0.214	0.926	0.003814149
PSMD4	1.75E-07	-0.579221309	0.071	0.893	0.003825993
TK1	1.85E-07	-0.762373407	0.071	0.872	0.004033548
PGK1	2.22E-07	-0.946004005	0.429	0.987	0.004841596
NDUFC1	2.66E-07	-0.812855193	0.286	0.96	0.00579714
TIMM10	2.83E-07	-0.549587026	0.071	0.859	0.006168378
ACADM	3.17E-07	-0.602620704	0.071	0.872	0.006922733
ZWINT	3.48E-07	-0.631742546	0	0.819	0.007580672
CALR	3.74E-07	-0.977959084	0.786	0.993	0.008161369
PSMB2	3.79E-07	-0.774662667	0.429	0.987	0.008265828
BOLA3	4.21E-07	-0.600547001	0.071	0.872	0.009177678
UBE2T	4.26E-07	-0.576050267	0	0.812	0.009293467
EIF4G2	4.34E-07	-0.838509616	0.357	0.966	0.00945699
DHFR	4.45E-07	-0.835408637	0.071	0.832	0.009711128
SNRNP40	5.37E-07	-0.559387663	0.071	0.832	0.011702646
MRPL14	5.53E-07	-0.644290505	0.214	0.893	0.012051563
NDUFA9	5.80E-07	-0.531597539	0.071	0.859	0.012653323
HNRNPAB	5.98E-07	-0.819084715	0.286	0.966	0.013050939
TMPO	6.20E-07	-0.675101416	0.214	0.919	0.013517292
COPS3	7.02E-07	-0.62273729	0.143	0.893	0.015302402
POLR2J	7.24E-07	-0.635040268	0.071	0.886	0.015782569
ATP5G3	7.26E-07	-0.833009073	0.714	1	0.015832347
MRPS33	7.29E-07	-0.573233289	0.143	0.872	0.015893709
CFL1	7.71E-07	-0.990256174	0.571	1	0.01681817
RBM3	8.19E-07	-0.881615212	0.571	1	0.017870837
PSMD8	8.28E-07	-0.805620839	0.357	0.98	0.018064358
COPS8	8.51E-07	-0.45353841	0.071	0.832	0.018555037
SHMT2	8.83E-07	-0.776767848	0.143	0.96	0.019254424
APH1A	9.30E-07	-0.567653349	0.214	0.913	0.020291414
SDHB	9.64E-07	-0.59427377	0.071	0.872	0.021032002
PPIA	1.14E-06	-0.797020627	0.929	1	0.024909513
TIMM50	1.15E-06	-0.445324396	0	0.779	0.024982651
SRP9	1.16E-06	-0.5347155	0.286	0.953	0.025311866
CCT8	1.17E-06	-0.750536798	0.5	0.987	0.025614004
ATP5G1	1.17E-06	-0.935116991	0.357	1	0.025614004
SAR1B	1.23E-06	-0.372095427	0.357	0.993	0.026767766
UFD1L	1.26E-06	-0.563703397	0.143	0.946	0.027447415
OST4	1.33E-06	-0.721212295	0.643	1	0.028911074
ATPIF1	1.36E-06	-0.787522736	0.357	0.987	0.029684484
CDK1	1.39E-06	-0.671021483	0	0.772	0.030268773
COX7A2L	1.52E-06	-0.664669795	0.357	0.96	0.033238158
HSPA6	1.54E-06	1.291989472	0.214	0.007	0.033514236
POLR2K	1.58E-06	-0.5858739	0.214	0.899	0.03441014
RAB5C	1.65E-06	-0.461545468	0.071	0.872	0.036055498
PSMD2	1.73E-06	-0.655298265	0.071	0.886	0.037737167
SF3A3	1.76E-06	-0.510782718	0.071	0.799	0.038387785
UBL7	1.87E-06	-0.461157758	0.071	0.799	0.040736577
THOC3	1.89E-06	-0.527240757	0.143	0.846	0.041255208
HADH	1.92E-06	-0.523261036	0.071	0.792	0.041922479
C1QBP	2.00E-06	-0.857079623	0.5	0.973	0.043577028
SLIRP	2.05E-06	-0.682704405	0.286	0.906	0.044668348
VAPA	2.06E-06	-0.643405072	0.214	0.899	0.044868272

MORF4L2	2.06E-06	-0.598906758	0.214	0.919	0.044869128
AP2M1	2.17E-06	-0.613694662	0.214	0.94	0.047415411
FAM96B	2.23E-06	-0.656377607	0.429	0.946	0.048622098

List of differentially expressed genes of IGLV3-1_plasma in overall stage (PR versus PP)

gene	p_val	avg_logFC	pct.1	pct.2	p_val_adj
HSP90AA1	9.80E-09	2.381811041	1	0.909	0.000213612
HSPB1	7.42E-08	2.416749035	1	0.5	0.001619118
MIR155HG	2.22E-06	1.410327444	0.793	0.182	0.048513589

List of differentially expressed genes of MT2A_B in overall stage (PR versus PP)

gene	p_val	avg_logFC	pct.1	pct.2	p_val_adj
NA					

Table 21: List of DEGs of B/plasma cell clusters in cold preservation stage (EP versus PP)

List of differentially expressed genes of TCL1A_B in cold preservation stage (EP versus PP)

gene	p_val	avg_logFC	pct.1	pct.2	p_val_adj
FOS	6.89E-10	0.625809403	0.909	0.771	1.50E-05
IGLV2-14	1.01E-09	0.492652885	0.358	0.109	2.20E-05
FOSB	1.15E-08	0.701482895	0.576	0.363	0.000250557
DUSP1	6.22E-07	0.332743337	0.934	0.9	0.01357407

List of differentially expressed genes of IGKV1D-16_plasma in cold preservation stage (EP versus PP)

gene	p_val	avg_logFC	pct.1	pct.2	p_val_adj
IGLV2-23	1.72E-08	1.128521546	0.347	0.112	0.00037444
PPP1R15A	2.25E-06	-0.34259683	0.665	0.818	0.048997878

List of differentially expressed genes of GPR183_B in cold preservation stage (EP versus PP)

gene	p_val	avg_logFC	pct.1	pct.2	p_val_adj
FOS	8.96E-07	0.70874471	0.87	0.611	0.019543812

List of differentially expressed genes of HIST1H4C_plasma in cold preservation stage (EP versus PP)

gene	p_val	avg_logFC	pct.1	pct.2	p_val_adj
PFN1	4.56E-09	-0.375343135	1	1	9.95E-05
YBX1	3.52E-08	-0.324359341	0.99	1	0.000768019
PPIA	5.93E-08	-0.282625043	1	1	0.001292756
RPL9	9.90E-08	-0.251662311	1	0.993	0.002158052
RPS2	3.06E-07	-0.325932951	1	1	0.006664824
NME2	4.05E-07	-0.270956904	1	1	0.008841276
SLC25A5	7.28E-07	-0.297217841	1	0.993	0.015875543
CORO1A	1.04E-06	-0.389784373	0.958	0.98	0.022712883
RPL7	1.12E-06	-0.252704043	1	1	0.024485358
C1QBP	1.94E-06	-0.309047258	0.906	0.973	0.042369624
GPI	2.08E-06	-0.266286668	0.812	0.919	0.045306505

List of differentially expressed genes of IGLV3-1_plasma in cold preservation stage (EP versus PP)

gene	p_val	avg_logFC	pct.1	pct.2	p_val_adj
NA					

List of differentially expressed genes of MT2A_B in cold preservation stage (EP versus PP)

gene	p_val	avg_logFC	pct.1	pct.2	p_val_adj
NA					
